# Molecular evolution of the members of the Snq2/Pdr18 subfamily of Pdr transporters in the Hemiascomycete yeasts

**DOI:** 10.1093/femsyr/foaf026

**Published:** 2025-05-27

**Authors:** Paulo Jorge Dias

**Affiliations:** iBB—Institute for Bioengineering and Biosciences, Instituto Superior Técnico, University of Lisbon, 1049-001 Lisboa, Portugal; Associate Laboratory i4HB—Institute for Health and Bioeconomy at Instituto Superior Técnico, University of Lisbon, 1049-001 Lisboa, Portugal

**Keywords:** Snq2/Pdr18 subfamily, ATP-Binding Cassette-Pleiotropic Drug Resistance, comparative genomics, molecular evolution, selective forces, episodic positive selection

## Abstract

The transporters of the ATP-Binding Cassette (ABC) Superfamily involved in the Multidrug Resistance (MDR) phenomena are also known as ABC-Pleiotropic Drug Resistance (PDR) proteins. The homologs of the *Saccharomyces cerevisiae SNQ2* and *PDR18* genes were identified in 171 yeast genomes, representing 68 different hemiascomycetous species. All early-divergent yeast species analyzed in this work lack Snq2/Pdr18 homologs, suggesting that the origin of these ABC-PDR genes in hemiascomycete yeasts resulted from a horizontal transfer event. The evolutionary pathway of the Snq2/Pdr18 protein subfamily in pathogenic *Candida* species was also reconstructed, revealing a main gene lineage leading to the *Candida albicans SNQ2* gene. The results indicate that, after the gene duplication event at the origin of the *SNQ2/PDR18* paralogs, the *PDR18* ortholog has been under strong diversifying selection and suggest that a small portion of the sequence of the *SNQ2* ancestral ortholog might have been under mild positive selection. The results also showed that strong positive selection was exerted over one of the two paralogs generated by the Whole Genome Duplication (WGD) event, corresponding to the duplicate at the origin of a “short-lived” WGD sublineage.

## Teaser

In this study, the transporters of the ATP-Binding Cassette (ABC) Superfamily involved in the multidrug resistance phenomena, also known as ABC- Pleiotropic Drug Resistance proteins, are analyzed from an evolutionary perspective.

## Introduction

The transporters of the ATP-Binding Cassette (ABC) Superfamily involved in the Multidrug Resistance/Multixenobiotic Resistance (MDR/MXR) phenomena (Bosmann 1971, Higgins 2007) are also known as ABC-Pleiotropic Drug Resistance (PDR) proteins (Prasad and Panwar 2004, Jungwirth and Kuchler 2006, Monk and Goffeau 2008, Lamping et al. 2010, Prasad and Goffeau 2012, Piecuch and Obłak 2014). The structural organization of the ABC-PDR transporters consists of two distinct types of domains: the nucleotide-binding domain (NBD), which is required for ATP recognition and hydrolysis and provides the energy for the transport of chemical compounds, and the transmembrane domain (TMD), which ensures the insertion and stability of these proteins in cellular membranes (Decottignies and Goffeau 1997, Paulsen et al. 1998, Saier 2003, Gaur et al. 2005, Jungwirth and Kuchler 2006, Lamping et al. 2010, Prasad and Goffeau 2012, Prasad et al. 2016). Full-structure ABC-PDR transporters comprise two cytoplasmic NBDs, N-NBD and C-NBD, and two TMDs, N-TMD and C-TMD, containing a total of 12 transmembrane-spanning segments (TMSs) (Jungwirth and Kuchler 2006, Lamping et al. 2010, Prasad and Goffeau 2012). The NBDs of ABC proteins typically contain seven conserved motifs, with the H-, P-, Q-, and D-loops interacting with the ABC signature motif from the opposite NBD to form two composite nucleotide-binding pockets (CNBP1 and CNBP2) (Taglicht and Michaelis 1998, Jones and George 2004). The ABC-PDR proteins encoded in organisms classified by higher eukaryotic clades exhibit a [TMD-NBD]2 domain topology, whereas fungal organisms encode PDR transporters with a reversed domain topology ([NBD-TMD]2 configuration) (Decottignies and Goffeau 1997, Paulsen et al. 1998, Gaur et al. 2005, Jungwirth and Kuchler 2006, Lamping et al. 2010, Prasad and Goffeau 2012, Prasad et al. 2016).

For more than three decades, *Saccharomyces cerevisiae* has been served as an experimental platform in the functional characterization of the ABC-PDR efflux pumps in the hemiascomycete yeasts (Decottignies and Goffeau 1997, Paulsen et al. 1998, Taglicht and Michaelis 1998, Prasad et al. 2016). This model organism encodes nine full-structure ABC-PDR genes, *AUS1, PDR5, PDR10, PDR11, PDR12, PDR15, SNQ2, PDR18*, and YOL075C, and one additional ABC-PDR gene, *ADP1*, encoding an N-terminal truncated protein containing only one NBD and eight TM segments. Phylogenetic analysis of the ABC-PDR efflux pumps in 55 different fungal species revealed that these proteins divide into 10 distinct clusters (Lamping et al. 2010). One of these clusters comprises the homologs of the *S. cerevisiae SNQ2* and *PDR18* genes (Gbelska et al. 2006, Seret et al. 2009, Lamping et al. 2010, Godinho et al. 2018a). The yeast *SNQ2* gene was one of the first ABC-PDR efflux pumps described as involved in the MDR/MXR phenomenon, reported to confer yeast resistance to two chemical mutagens, 4-nitroquinoline 1-oxide and triaziquone (Servos et al. 1993). Subsequent studies linked the increased expression of this gene to higher tolerance of yeast cells to a wide number of drugs and chemical compounds (Hirata et al. 1994, Mahé et al. 1996a,b, Miyahara et al. 1996, Kolaczkowski et al. 1998, Ververidis et al. 2001, Piper et al. 2003, Wehrschütz-Sigl et al. 2004, van Leeuwen et al. 2012, Ling et al. 2013, Nishida et al. 2013, Snider et al. 2013, Tsujimoto et al. 2015). The *PDR18* gene has been functionally associated with the regulation of the ergosterol content present in the yeast plasma membrane (Cabrito et al. 2011, Teixeira et al. 2012, Godinho et al. 2018b, Ribeiro et al. 2022). Increased expression of this gene was first associated with enhanced yeast resistance to herbicides 2,4-dichlorophenoxyacetic acid (2,4-D), 2-methyl-4-chlorophenoxyacetic acid, barban, the agricultural fungicide mancozeb and the metal cations Zn^2+^, Mn^2+^, Cu^2+^, and Cd^2+^ (Cabrito et al. 2011), as well as to ethanol (Teixeira et al. 2012) and acetic acid (Godinho et al. 2018b, Ribeiro et al. 2022). The maintenance of yeast plasma membrane ergosterol content, regulated by *PDR18*, under 2,4-D or acetic acid stress, was linked to resistance to multiple stresses (Cabrito et al. 2011, Teixeira et al. 2012, Godinho et al. 2018a). Transcriptional activation of *PDR18* and several ergosterol biosynthetic genes was also observed in response to acetic acid stress, with ergosterol homeostasis playing a crucial role in counteracting acetic acid-induced decreases in plasma membrane lipid order, increasing membrane nonspecific permeability, and decreasing transmembrane electrochemical potential (Godinho et al. 2018b, Ribeiro et al. 2022). In contrast, no role in lipid homeostasis has been assigned to the Snq2 transporter, despite the modulation of ergosterol content associated with Pdr18.

A few years ago, my research group extensively characterized the *S. cerevisiae SNQ2/PDR18* paralog genes and their *Nakaseomyces glabratus* homolog, the *CgSNQ2* gene, from an evolutionary and chemical profiling comparative perspective (Godinho et al. 2018a) (the past name of *N. glabratus* species was *Candida glabrata*; in the remaining of this work, we decided to use the medically-relevant name of this species due to the strong association of the ABC-PDR transporters with yeast MDR). This study showed that the *SNQ2* and *PDR18* genes determine genetic resistance to 9 and 29 different chemical compounds, respectively (from a total of 35 chemical compounds tested), though these sets show little overlap (Godinho et al. 2018a). This study also expanded the evolutionary history of the Snq2/Pdr18 subfamily in yeast species classified within the Saccharomycetaceae taxonomic family (Godinho et al. 2018a). Compared with a previous study by Seret et al. (2009), which focused on 2 post-Whole Genome Duplication (WGD) species, the inclusion of 14 additional post-WGD yeast species, representing 6 different post-WGD taxonomic genera, dismissed the evolutionary scenario proposing the origin of the two paralog genes during the WGD event. Gene neighborhood analysis indicated that the duplication event leading to the two paralog genes originated from a more recent mutation event (Godinho et al. 2018a). The study also confirmed the presence of two gene sublineages formed during the WGD event, one “long-lived” sublineage originating the *S. cerevisiae SNQ2/PDR18* genes and a “short-lived” sublineage comprising genes from the *Tetrapisispora* and *Vanderwaltozyma* yeast species (Godinho et al. 2018a). Although Godinho et al. (2018a) extensively characterized the chemical resistance profiles of the *SNQ2* and *PDR18* genes and their *C. glabrata* ortholog, the *CgSNQ2* gene, it is plausible that the true functions of these *S. cerevisiae* paralogs have yet to be fully discovered. Furthermore, some evolutionary questions remain unsolved. For instance, which selective forces, positive or neutral selection, dominated the evolution of the two *S. cerevisiae* paralogs after the gene duplication event? Which amino acid residues contributed to the functional divergence of the Snq2 and Pdr18 efflux pumps? Are there still unknown protein motifs that characterize the Snq2/Pdr18 homologs? Have positive and neutral selection played roles in the functional divergence of other Snq2/Pdr18 gene subfamily members? If both these two selective forces were involved in the evolution of the Snq2/Pdr18 homologs, did they act on different gene lineages or at different points in time? Taking advantage of the extensive chemical resistance profiles of the two *S. cerevisiae* paralog genes and the *CgSNQ2* ortholog, this study aims to analyze these unresolved evolutionary questions.

In the first stage, the complete set of Snq2/Pdr18 homologs encoded in 161 yeast genomes was identified. The selected strains encompass 68 yeast species, spanning a broad taxonomic range across > 8 families of the Saccharomycotina subphylum. The species selected include some of the most important yeast species used in industry, biotechnology, and clinical settings. Classic phylogenetic approaches combined with gene neighborhood analysis will be employed to reconstruct the evolutionary history of the Snq2/Pdr18 subfamily members encoded in species from the CTG clade species and other hemiascomycete families. An entropy-based approach, complemented with moving average (MA) functions, will be used to identify novel protein motifs in the amino acid sequence of Snq2/Pdr18 homologs. Models of molecular evolution, supported by in silico methods for inferring ancestral gene sequences, will provide new insights into the functional differentiation of selected Snq2/Pdr18 homologs. Finally, the Three-Dimensional (3D) protein structure models of *S. cerevisiae* Snq2 and Pdr18 proteins, predicted by the AlphaFold 2 database (DB), will be used to integrate the identified motifs and the amino acid residues at sites detected to have been under the action of positive selection, from a structural perspective.

## Materials and methods

### DB construction, genome annotation, and yeast strains and species selected for analysis

Two local DBs were developed in-house using the MySQL Relational Database Management System. The first DB, henceforth referred to as GenomeDB, compiles the biological information from a total of 1 110 525 Open Reading Frames (ORFs) encoded in 171 hemiascomycete genomes with publicly available sequences, corresponding to 68 different yeast species. The genome sequences of these yeast strains were downloaded from the following reference DBs: Genbank (Genbank n.d.), DOE Joint Genome Institute (DOE Joint Genome Institute n.d.), Saccharomyces Genome Database (SGD) (Saccharomyces Genome Database n.d.), Candida Genome Database (Candida Genome Database n.d.), and Yeast Gene Order Browser (Yeast Gene Order Browser n.d.). When high-quality genome annotations were available, the corresponding gene transfer format/general feature format 3 annotation file was obtained directly from the corresponding genome DB. When the genome annotation was of poor quality—such as when sequencing coverage was low or the genome assembly produced a large number of contig sequences—the available genome annotation was not used. Instead, the yeast genome annotation pipeline (Proux-Wéra et al. 2012) was employed to generate consistent and homogeneous genome annotations for these strains. The biological information comprised in the GenomeDB includes ORF name, species, strain, genome DB where the data was originally retrieved, chromosome/contig location, order in the corresponding chromosome, strand, coding region limits, ORF start position, ORF end position, ORF length, translated ORF length, alternative names, description and corresponding DNA, and amino acid sequences.

The second DB, henceforth named BlastDB, compiles the amino acid sequence similarity for all possible pairwise combinations between the translated ORFs comprised in the GenomeDB (~328 million blastp entries). This DB contains the output of each pairwise sequence comparison (Altschul et al. 1990), including the length of the alignment, e-value, percentage of identity and similarity, and alignment score. The parameters used for blastp to calculate amino acid pairwise sequence similarity were as follows: open gap (-1), extend gap (-1), threshold for extending hits (11), and word size (3).

Table A1_Supplementary Data summarizes the available information on these yeast species and strains. The Saccharomycetaceae family comprises the largest number of yeast species selected for analysis in this study, including 118 yeast strains representing 30 hemiascomycete species. The Saccharomycetaceae are divided into 12 distinct genera (Kurtzman 2011). The hemiascomycete yeasts classified in the CTG phylogenetic clade are divided into two taxonomic families: Debaryomycetaceae and Metschnikowiaceae (Massey et al. 2003, Butler et al. 2009, Santos et al. 2011). The CTG clade species selected for analysis include 15 species from Debaryomycetaceae, 3 from Metschnikowiaceae, and one ancestral yeast species, *Babjeviella inositovora* (Riley et al. 2016, Krassowski et al. 2018). This study also includes *Pachysolen tannophilus*, a yeast species that translates the CTG codon into alanine instead of leucine (classified in the Pachysolenaceae family) (Liu et al. 2012, Riley et al. 2016, Krassowski et al. 2018), and *Ascoidea rubescens*, which translates the CTG codon into serine instead of leucine, although it does not belong to the CTG clade (classified in the Ascoideaceae family) (Krassowski et al. 2018).

Although the GenomeDB did not initially include strains from the *Saccharomyces eubayanus*, strains FM1318 and CBS12357 (Baker et al. 2015, Okuno et al. 2016) were added to the dataset to corroborate results regarding the phylogenetic origin of the Snq2/Pdr18 homologs within the *Saccharomyces* genus. Several strains belonging to the same yeast species, corresponding to a total of 13 different yeast species, were included in this study: *C. albicans* (11 strains), *C. glabrata* (2 strains), *Clavispora lusitaniae* (2 strains), *Cyberlindnera jadinii* (2 strains), *Dekkera bruxellensis* (2 strains), *Debaryomyces hansenii* (2 strains), *Komagataella pastoris* (3 strains), *Naumovozyma castellii* (2 strains), *Saccharomyces bayanus* (2 strains), *Saccharomyces cerevisiae* (60 strains), *Saccharomyces eubayanus* (2 strains), *Saccharomyces paradoxus* (25 strains), and *Zygosaccharomyces bailii* (2 strains). The four-letter species abbreviation used in Table A1_Supplementary Data was also adopted for yeast gene names. The number following the first four letters abbreviates the yeast strain under analysis. To standardize the annotation, ORF names are presented in lowercase letters.

### Identification of the members of the Snq2/Pdr18 subfamily

The identification of ORFs encoding ABC-PDR proteins was based on the amino acid sequence similarity information compiled in BlastDB (Dias and Sá-Correia 2013, Dias and Sá-Correia 2014, Palma et al. 2017, Godinho et al. 2018a). Pairwise blastp hits between translated ORFs in this DB were represented as a network, where edges indicated amino acid sequence similarity between pairs of translated ORFs (Dias and Sá-Correia 2013, Dias and Sá-Correia 2014, Palma et al. 2017, Godinho et al. 2018a). This network was subsequently constrained using a range of different e-value thresholds. The resulting networks were traversed using known *S. cerevisiae* ABC-PDR proteins as starting nodes (Dias and Sá-Correia 2013, Dias and Sá-Correia 2014, Palma et al. 2017, Godinho et al. 2018a). This approach allowed for the classification of each translated ORF into one, and only one, cluster of amino acid sequence similarity. Two software suites were employed to construct a preliminary phylogenetic tree using the amino acid sequences from the gathered set of ABC-PDR proteins (Supplementary Data A2): (i) the Muscle software (Edgar 2004, Muscle 3.8 n.d.) was used to perform a multiple sequence alignment of the protein set and (ii) the PHYLIP software (Felsenstein 1989, PHYLIP 3.6.98 n.d.) provided two algorithms, Protdist and Neighbor, whose successive application allowed the generation of a distance-based tree.

In addition to confirming the phylogenetic distribution of the ABC-PDR proteins, the construction of this preliminary phylogenetic tree served two additional purposes (Fig. A3_Supplementary Data): (i) to detect fragments or sequence frameshifts present in the gathered set of ABC-PDR proteins and (ii) to identify potential Snq2/Pdr18 homologs that did not cluster with the majority of the gene subfamily members due to the DNA sequencing or gene annotation errors (see the next subsection for further details).

### Two-dimensional protein structure prediction and detection and correction of errors in the sequences of the members of the Snq2/Pdr18 subfamily

Five software suites were used to obtain an initial two-dimensional (2D) protein structure prediction for the potential Snq2/Pdr18 homolog: (i) TMHMM (Sonnhammer et al. 1998, TMHMM 2.0 n.d.), (ii) HMMTOP (Tusnády and Simon 2001, HMMTOP 2.0 n.d. ), (iii) MEMSAT2 (Jones and George 2004), (iv) MEMSAT3 (Jones 2007, MEMSAT3 n.d.), and (v) TOPPRED (von Heijne 1992, Claros and Heijne 1994, MESSA n.d.). The underlying models and algorithms of these five software suites include Hidden Markov Models (HMMs) (TMHMM and HMMTOP), Neural Network Models (NNMs) (MEMSAT2 and MEMSAT3), and a hydrophobicity-based sliding window algorithm (TOPPRED). This diverse approach ensured robust 2D protein structure predictions for each member of the Snq2/Pdr18 subfamily. Additionally, the ABC-PDR proteins were aligned, and the corresponding homology information was processed using the Polyphobius software suite (Käll et al. 2005, PolyPhobius 1.0.1 n.d.) to refine individual 2D protein structure predictions for each Snq2/Pdr18 homolog. Analysis of the results revealed that the majority of predictions from the five software suites and Polyphobius only partially coincided. Consequently, only regions identified by Polyphobius that resided within the transmembrane (TM) regions of all aligned amino acid sequences were considered reliable. This approach facilitated the generation of a consensus core for each TM.

Four different approaches were used to test for potential sequence errors in the gathered protein set: (i) To identify false members of the Snq2/Pdr18 subfamily, the amino acid sequences of translated ORFs located on long branches of the preliminary phylogenetic tree were subject to blastp searches against the Genbank DB; (ii) To detect potential protein fragments or concatenations, the size of each translated ORF was compared with the known lengths of established Snq2/Pdr18 subfamily members; (iii) To identify potential protein truncations, 2D protein structure prediction tools were employed to determine whether translated ORFs encoded full-size ABC-PDR proteins or truncated variations; and (iv) To detect errors such as false stop codons and sequence frameshifts, the alignment software suite Jalview (Clamp et al. 2004, Waterhouse et al. 2009, Jalview 2.11.3 n.d.) was used for visual inspection of each translated ORF, comparing them with the multiple alignments of all potential Snq2/Pdr18 homologs under analysis.

The detected errors were corrected using a trial-and-error strategy involving: (i) The successive application of the EMBOSS Needle and Transeq algorithms (Rice et al. 2000, EMBOSS 6.6.0 n.d.) and (ii) The comparison of the alignment output with a highly homologous reference protein sequence. In the case of potential frameshift errors, the Needle algorithm was first used to construct a pairwise sequence alignment between the target and reference proteins to determine the point where the two proteins start misaligning. Small corrections in the nucleotide sequence—such as the deletion of unknown nucleotides (N)—were proposed to resolve the frameshift error. The modified sequence was then translated using the Transeq algorithm, and the alignment was rechecked using Needle. This iterative approach successfully recovered full-size ABC-PDR structures for most Snq2/Pdr18 homologs with frameshift errors. In cases of potential protein fragments, missing sequence portions were manually retrieved, and a new sequence was constructed by merging the translated ORF's DNA sequence with upstream and/or downstream regions. Subsequently, the iterative sequence correction approach described above was applied until a full-size ABC-PDR protein structure was recovered. If numerous errors were detected in a particular Snq2/Pdr18 homolog, indicating low-quality genome sequencing, the encoded protein was deemed non-eligible and excluded from the dataset.

To identify potential misclassified Snq2/Pdr18 homologs, the amino acid sequences of 1648 gathered ABC-PDR proteins were used to build a local *blastp* DB. The *Blastp* algorithm was then employed to identify the highest-scoring protein sequence for each potential fragment or sequence frameshift. This pairwise sequence alignment uncovered a few Snq2/Pdr18 homologs that clustered with other ABC-PDR proteins due to “long branch attraction.” Additionally, this analysis also revealed that most subfamily members found in the genome sequences of *S. bayanus* strains MCYC 623 and 623–6C, and the *Saccharomyces uvarum* strain CBS 7001, encoded protein fragments. Since these two yeast species diverged near the root of the *Saccharomyces* genus, they are crucial for reconstructing the evolutionary pathway of the Snq2/Pdr18 homologs during the transition from the *Kazachstania* and *Naumovozyma* genera to the *Saccharomyces* genus.

Although *Saccharomyces eubayanus* is a species not included in the GenomeDB, its divergence also occurred near the root of the *Saccharomyces* genus, making it valuable for compensating for the lack of data from *S. uvarum* and the two *S. bayanus* hybrid strains. A second local *blastp* DB was therefore constructed using the complete protein sets encoded in two *S. eubayanus* strains, FM1318 and CBS12357 (Baker et al. 2015, Okuno et al. 2016). Then, the amino acid sequences of the *S. cerevisiae* Snq2 and Pdr18 transporters were used to identify corresponding homologs, revealing two Snq2/Pdr18 homologs in each *S. eubayanus* strain.

The decision to include more than one strain for 13 yeast species enabled the detection of intraspecies gene number variations within the Snq2/Pdr18 subfamily. Variations were found in three species: *S. cerevisiae, S. paradoxus*, and *N. castellii*. For instance, the genome of *S. cerevisiae* strain CEN.PK113-7D lacks an *SNQ2* ortholog, while the *S. cerevisiae* strains RM11-1a, Sigma1278b, VL3, and W303 lack a *PDR18* ortholog. The *S. paradoxus* strain UFRJ50791 also lacks an *SNQ2* ortholog. Among the *N. castellii* strains analyzed, CBS 4309 encoded three full-size ABC-PDR proteins, whereas NRRL Y-12630 encodes only one. However, the genome of strain NRRL Y-12630 also contains two Snq2/Pdr18 protein fragments, likely resulting from contig-end truncations, suggesting that both *N. castellii* strains may encode three full-size Snq2/Pdr18 homologs.

### Phylogenetic analysis

The MUSCLE software (Edgar 2004, Muscle 3.8 n.d.) was used to build a multiple alignments of the amino acid sequences of the validated members of the Snq2/Pdr18 subfamily. Functions provided by the R libraries seqinr (Charif and Lobry 2007, Seqinr 4.2-36 n.d.) and ape (Paradis et al. 2004, Ape 5.7-1 n.d.) were used to convert the resulting fasta file into a nexus file, the input format required by MrBayes (Huelsenbeck and Ronquist 2001, Ronquist and Huelsenbeck 2003, MrBayes 3.2.7a n.d.). The Message Passing Interface (MPI) version of MrBayes (Altekar et al. 2004) was used to accelerate the computation of the Bayesian phylogenetic trees. wian_1_1_a02920 was selected as the root of the phylogenetic tree, as this ORF encoded the most divergent amino acid sequence in the protein set. The Markov Chain Monte Carlo (MCMC) sampling approach implemented by this phylogenetic software suite was based on one cold chain and nine heated chains. A fixed-rate amino acid prior model was set, allowing the MCMC sampler to explore all nine available models by regularly proposing new ones (upon parameter convergence, each model contributes to the results in proportion to its posterior probability). Rate variation across sites was assumed to follow a gamma distribution, and the general time reversible model was set to represent the likelihood function. The default values were used for all remaining MrBayes MPI parameters. The MCMC simulations ran two independent runs, each initiated from two random trees. After running the two MCMC simulations for 107 000 generations, the standard deviation of split frequencies fell below 0.05, and the potential scale reduction factor approached 1.0 for all parameters, indicating their convergence. The two parameters of the gamma distribution, assumed equally by MrBayes, converged to a value of 0.696 (95% Highest Posterior Density (HPD) interval = 0.668–0.721). The total tree length converged to 28.718 (95% HPD interval = 27.183–29.987) (Supplementary Data A4). This confirmed the convergence of the model parameters, ensuring the generation of highly similar phylogenetic trees.

PhyML (Guindon et al. 2010, PhyML 3.0 n.d.), a software suite based on a Maximum Likelihood (ML) algorithm, was used to confirm the phylogenetic results obtained using MrBayes (Fig. A5_Supplementary Data). The default values were used for most parameters, except for the optimal phylogenetic tree search strategy, where the subtree pruning and regrafting algorithm was used instead of nearest neighbor interchanges.

Graphical environments from Dendroscope (Huson et al. 2007, Huson and Scornavacca 2012, Dendroscope 3.5.7 n.d.), FigTree (FigTree 1.4.3 n.d.), and the PhyloTree app (PhyloTree 0.1 n.d.) in the Cytoscape 2.8.3 software suite (Shannon et al. 2003, Cytoscape 2.8.3 n.d.) were used to analyze the constructed phylogenetic trees. The credibility of the clusters in both trees was assessed by inspecting MrBayes bipartition probabilities and PhyML bootstrapping values for each internal node, analyzed using FigTree and the PhyloTree app. This analysis confirmed that the two phylogenetic methods, based on distinct statistical frameworks, yielded similar results. Therefore, the phylogenetic analysis presented in the remainder of this study is based solely on the Bayesian tree.

To verify the robustness of the MUSCLE-derived multiple alignments and assess whether transmembrane (TM) segment predictions by the TMHMM software suite could improve the alignment, the PRALINE server (Simossis and Heringa 2005, Pirovano et al. 2008) (https://www.ibi.vu.nl/programs/pralinewww) was used to generate an alternative alignment (“Transmembrane structure prediction” option enabled) (Fig. A6_Supplementary Data). Subsequently, the Protdist and Neighbor algorithms provided by the PHYLIP software suite (Felsenstein 1989, PHYLIP 3.6.98 n.d.) were used to construct a phylogenetic tree from this alternative alignment (Fig. A7_Supplementary Data). Comparison of these trees revealed that the phylogenetic clusters identified using MrBayes and PhyML were also recovered in the tree generated from the PRALINE-TM alignment (Fig. A7_Supplementary Data). Thus, it was concluded that the impact of TM information on improving the quality of the multiple sequence alignment was negligible. Therefore, the MUSCLE software suite was selected as the default methodology for aligning members of the Snq2/Pdr18 subfamily.

### Gene neighborhood analysis

Gene neighborhood analysis was used to determine the existence (or absence) of synteny between members of the Snq2/Pdr18 subfamily (Dias et al. 2010, Dias and Sá-Correia 2013, Dias and Sá-Correia 2014, Palma et al. 2017, Godinho et al. 2018a). First, R scripting was employed to retrieve 15 neighboring genes on each side of the query genes, along with the corresponding sequence clustering classification from GenomeDB. The rationale behind synteny analysis relies on the assumption that two genes from different yeast species, whose translation products belong to the same sequence cluster (homologs by similarity), will be members of the same gene lineage if they share at least one pair of neighboring genes that are also homologous by similarity. This process is repeated for all possible heterospecific pairwise comparisons of homologs deduced from the sequence clusters. The number of shared neighbors between members of the Snq2/Pdr18 subfamily allows for determining the degree of synteny among them. The sequence clustering classification of the 30 neighboring genes around each query gene was performed using a conservative BLASTp e-value threshold of E-50 to limit the inclusion of false-positive sequences alongside true cluster members. When additional evidence was needed to corroborate dubious synteny connections between genes, sequence clustering was performed at a less restrictive e-value threshold (E-40).

Second, R scripting was used to convert the tabular data representing the chromosomal neighborhood of each Snq2/Pdr18 subfamily member into the input format required by the Cytoscape 3.9 software suite (Shannon et al. 2003, Su et al. 2014, Cytoscape 3.10.1 n.d.), enabling the construction of the corresponding network. In this network, nodes represent query genes, and edges represent pairs of neighboring genes classified within the same sequence cluster. Relevant biological information, detailed below, was also imported into the Cytoscape network as edge attributes. The existence of synteny between query genes was verified by analyzing the network topology (i.e. the number of shared neighbor pairs) and the biological information associated with the corresponding edges (Dias and Sá-Correia 2013– (Dias et al. 2010, Palma et al. 2017, Godinho et al. 2018a). The advantage of this framework is that it facilitates the exploration of synteny relationships within a straightforward mathematical context of network topology exploration. Three sources of biological information were used to assess the strength of each neighbor pair connection: i) Proximity of the neighboring genes to the query genes (the closer the neighbor, the stronger the synteny evidence); ii) Sequence similarity between neighboring genes (the greater the similarity, the stronger the synteny evidence); iii) Size of the sequence cluster to which the homologous neighbors belong (a smaller cluster size suggests a lower probability that two homologous neighbors are near two query genes by chance).

### Reconstruction of the DNA sequences of ancestral genes

MrBayes was used to reconstruct the sequences of selected ancestral Snq2/Pdr18 homologs (Huelsenbeck and Ronquist 2001, Ronquist and Huelsenbeck 2003). This reconstruction involved developing scripts in the R programming language to obtain a guided alignment of DNA sequences for the Snq2/Pdr18 homologs. The alignment was based on the corresponding amino acid sequence alignment, which was constructed using the MUSCLE software suite. Since protein-coding sequences exhibit a three-position offset in their autocorrelation due to their codon-based architecture (Yang 1995), a partitioning scheme was used to constrain the evolution of the three nucleotides comprising each codon. Each nucleotide was allowed to evolve at its own rate throughout the entire DNA sequence alignment, with the corresponding parameters unlinked across the three partitions. Two parameters were free to vary, while their average rate was constrained to a value of 1.0. The remaining MrBayes parameter scheme used to reconstruct the DNA sequences of ancestral genes is described in a previous “Materials and Methods” section. When published evidence was available regarding the evolution of a specific member of the Snq2/Pdr18 subfamily, this information was integrated as an a priori tree topology constraint in the input file used to configure MrBayes parameters (command “prset topologypr”).

### Analysis of the conservation of the amino acid sequences and identification of protein motifs

The LogOddsLogo software suite (Yu et al. 2015, LogLogoOdds 1.1.1 n.d. ) was used to analyze the amino acid sequence conservation of members of the Snq2/Pdr18 subfamily. LogOddsLogo employs a Bayesian algorithm that determines the information content present in each column of the multiple alignments of amino acid sequences, a variable henceforth referred to as the entropy score. The values of the LogOddsLogo parameters used to analyze the Snq2/Pdr18 proteins were set to their default settings.

Four MA functions, available through the R library technical trading rules (TTR 0.24-4 n.d.), were used to measure the regional conservation of the amino acid sequences of the Snq2/Pdr18 homologs. The four MA functions used to analyze the multiple alignments of the Snq2/Pdr18 proteins were (i) the Simple Moving Average (SMA), (ii) the Exponential Moving Average (EMA), (iii) the Double EMA (DEMA), and (iv) the Arnaud Legoux MA (ALMA). A preliminary analysis of different MA fixed windows indicated that a seven-amino-acid-residue window was adequate for representing the regional entropy score. The values of the parameters used by each of these MA functions were the default values.

The four regional entropy scores were used to calculate an aggregated value, a variable henceforth referred to as the sequence conservation classification score. This score quantifies the number of MA functions showing a regional entropy score greater than a predefined threshold value (set equal to 2.0 in this study). Because four MA functions were used to calculate this aggregated value, the sequence conservation classification score could take five possible values (0, 1, 2, 3, and 4). The sequence conservation classification score was used to classify the amino acid sequences of the members of the Snq2/Pdr18 subfamily into three different types of regions based on protein conservation: (i) protein motif, (ii) reasonably conserved sequence, and (iii) poorly conserved sequence. When successive sites in the multiple alignments exhibited a classification score of 2, 3, or 4, the corresponding amino acid region was considered to comprise a protein motif. If a single site in the multiple alignments showed a classification score equal to 1 or 2, the corresponding amino acid region was considered reasonably conserved. Amino acid regions showing a classification score equal to 0 were considered poorly conserved. The use of a fixed, predetermined number of amino acid residues in the MA window required inspecting the upstream sequence of each detected protein motif for the presence of additional conserved amino acid residues that may have gone unnoticed. If adjacent amino acid residues to the detected protein motif showed reasonable conservation, the motif was extended to include these residues.

### Detection of the past action of selective forces

Three models of molecular evolution available in the HYpothesis testing using PHYlogenies (HyPhy) software suite (Pond et al. 2005, HyPhy 2.5.60 n.d.) were used to detect the past action of selective forces on the members of the Snq2/Pdr18 subfamily: (i) the “adaptive Branch-Site Random Effects Likelihood” (aBSREL) model (Smith et al. 2015), (ii) the “Fast, Unconstrained Bayesian AppRoximation” (FUBAR) model (Murrell et al. 2013), and (iii) the “Mixed Effects Model of Evolution” (MEME) (Murrell et al. 2012).

The aBSREL algorithm detects branches (lineages) in the phylogenetic tree that have experienced episodic diversifying selection (Smith et al. 2015). First, the algorithm estimates the number of rate categories needed to capture the complexity of the evolutionary process on a specific branch (Smith et al. 2015). Then, it tests the statistical significance of the estimated rates (Smith et al. 2015). The default parameters were used as input for the aBSREL model, with the alpha parameter allowed to vary from branch to branch and the omega parameter allowed to vary among branch-site combinations. Two mathematical limitations associated with the aBSREL model, derived from the assumptions introduced during the development of the corresponding algorithm, might explain why the model failed to assign statistical significance to the past action of positive selection over the first *SNQ2* ancestral gene. The first mathematical limitation arises from the fact that the complexity of the aBSREL model is inferred from the data itself (Smith et al. 2015). When the analytical expression of the probability distribution is not known a priori, standard theoretical results on the asymptotic distribution of likelihood ratio (LR) tests cannot be applied. As a result, the developers of the aBSREL model resorted to determining an empirical distribution in the less favorable case scenario, assuming that the null hypothesis (dN/dS = 1) is true. However, this strategy decreases the power of the LR test, thereby reducing the sensitivity of the aBSREL model. The second limitation stems from the fact that researchers often test multiple or all branches of the phylogenetic tree for signs of positive selection, which introduces a family-wise error rate (Goeman and Solari 2014). To control this error and generate corrected p-values, the developers of the aBSREL model adopted the Holm-Bonferroni sequential rejection test. Testing multiple or all phylogenetic branches has both advantages and disadvantages. On one hand, testing all branches of the Snq2/Pdr18 phylogenetic tree prevents preconceived notions from biasing the analysis. On the other hand, due to the conservative nature of the Holm-Bonferroni procedure (Goeman and Solari 2014), testing multiple branches significantly reduces the power of the LR test. We chose to use the aBSREL model to test all phylogenetic branches, aiming to identify a restricted number of branches for subsequent testing using the MEME/MrBayes methodology. This decision further reduced the statistical power of the aBSREL LR test.

The FUBAR algorithm identifies the sites in the multiple alignments that have undergone negative, neutral, and pervasive positive selection (Murrell et al. 2013). The model assumes a constant dN/dS parameter per site (Murrell et al. 2013). Most of the input parameters for this model were set to their default values, except for: (i) the number of grid points per dimension (50), (ii) the number of MCMC chains (10), (iii) the length of each chain (10 000 000), (iv) the discarded burn-in samples (1 000 000), (v) the number of samples drawn from each chain (100 000), and (vi) the concentration parameter of the Dirichlet prior (0.5). The FUBAR model is simpler than the MEME model because the value of the dN/dS parameter is assumed constant per site. However, in the majority of the cases, this assumption is biologically unrealistic (Murrell et al. 2013). For instance, when the predominant force shaping the evolution of a site is positive selection, methodologies assuming a constant dN/dS (per site) may predict that the majority of lineages in the phylogenetic tree will be under diversifying selection (pervasive positive selection), even if this is not always accurate. On the other hand, when the predominant force shaping the evolution of a codon site is negative selection on most lineages, methodologies assuming a constant dN/dS (per site) might be unable to identify cases involving episodic positive selection. The detection of purifying selection on most lineages can obscure the signal of positive selection on the few lineages undergoing functional differentiation. For these reasons, in specific cases where the aBSREL model successfully identified past episodes of positive selection or other evolutionarily significant cases, it was decided to complement the FUBAR results by using a more complex model (i.e. MEME) that does not impose biologically unrealistic assumptions.

For the analyses based on the aBSREL and FUBAR models, the Snq2/Pdr18 homologs were divided into three gene groups, each encoded in yeast species representing different taxonomic ranges within the Saccharomycotina subphylum. The first group consists of the genes comprised in the WGD sublineage at the origin of the *S. cerevisiae* Snq2 and Pdr18 genes (the “long-lived” WGD sublineage) (“Identification of Phylogenetic Branches under the Past Action of Selective Forces” and “Identification of Sites under the Past Action of Selective Forces” sections). The second group consists of the Snq2/Pdr18 homologs encoded in Saccharomycetaceae species (“Identification of Phylogenetic Branches under the Past Action of Selective Forces” and “Identification of Sites under the Past Action of Selective Forces” sections). The third group is formed by all members of this gene subfamily encoded in the hemiascomycete yeasts analyzed in this study (“Identification of Phylogenetic Branches under the Past Action of Selective Forces” and “Identification of Sites under the Past Action of Selective Forces” sections). Two different reasons underlie the decision to divide the Snq2/Pdr18 homologs into different groups. First, the removal of less related sequences, in comparison to the ones within the taxonomic range of interest, improves the quality of the multiple alignment. As a result, it increases the discriminative power of the aBSREL model. Second, the molecular evolution models used in this study are unable to handle DNA sequences that require the use of more than one genetic code for translation. The division of the members of this ABC-PDR subfamily into different groups allowed us to overcome this problem, except when attempting to analyze the whole set of 197 non-repeated sequences (“Identification of Phylogenetic Branches under the Past Action of Selective Forces” and “Identification of Sites under the Past Action of Selective Forces” sections). Since resolving this limitation would require redesigning these models at the code level, we decided to use the genetic code shared by the majority of yeast species under analysis (the standard code), noting that the results obtained can only be considered an approximation in this particular case.

Since the FUBAR algorithm cannot detect episodic positive selection, the MEME model was used to identify the past action of this selective force on individual sites in specific branches of the Snq2/Pdr18 phylogenetic tree (Murrell et al. 2012). Unlike the FUBAR model, the MEME model allows the dN/dS parameter to vary both from codon site to codon site and from phylogenetic branch to phylogenetic branch (Murrell et al. 2012). This model can identify the molecular footprints of episodic positive selection, whereas methodologies assuming a constant dN/dS (per site) would only detect signs of purifying selection (Murrell et al. 2012). The default parameters were used for the MEME model, with the dN/dS estimated from data without branch corrections. Although the MEME model allows the value of the dN/dS parameter to vary from site to site and from branch to branch, it has also been reported that this model can overestimate the number of sites involved in past episodic positive selection (Murrell et al. 2012). Therefore, to control this type of error, it was decided to use an independent methodology to corroborate the likelihood of the detected sites. The ability of MrBayes to infer the nucleotide and amino acid sequences of the ancestral genes and, subsequently, checking for the presence of a nonsynonymous substitution at the sites detected by the MEME model was selected as the corroboration methodology. Henceforth, this more stringent approach for the detection of positive selection in branch-site combinations will be referred to as the MEME/MrBayes approach. This new approach was used to corroborate the sites detected by the MEME model based on the following criteria: in cases where MEME and MrBayes predictions agree, the detection of the past action of episodic positive selection at a particular site was considered reliable and was accepted; otherwise, the action of this selective force was considered dubious and was rejected.

The total number of possible branch-site combinations involved in analyzing a specific multiple alignments and its corresponding phylogenetic tree is determined by multiplying the number of branches in the tree by the number of sites in the alignment. Due to the large number of possible cases at stake, the analysis of branch-site combinations by the MEME model was restricted to a handful of cases involving Snq2/Pdr18 ancestral genes that had a key role in the evolution of this ABC-PDR subfamily or whose phylogenetic position is at the root of taxonomic families of interest. The first set of branch-site combinations chosen for analysis consisted of the initial ancestral genes that emerged at the origin of the two sublineages. One sublineage led to the *S. cerevisiae SNQ2* gene, while the other sublineage led to the *S. cerevisiae PDR18* gene. The analysis also included the corresponding pre-duplication parental ancestral gene. The second set of branch-site combinations under analysis involved the two ancestral ohnolog genes formed at the WGD event and the corresponding pre-duplication, protoploid parental gene. The third and fourth sets of branch-site combinations under analysis involved the ancestral genes associated with the origin of the members of the Snq2/Pdr18 subfamily encoded in the Saccharomycetaceae yeasts and with the origin of the members of the Snq2/Pdr18 subfamily encoded in the Debaryomycetaceae yeasts.

Finally, two facts mitigated the incapacity of these models to handle more than one genetic code when analyzing the entire set of members of the Snq2/Pdr18 subfamily. First, the aBSREL and MEME models assume independence of phylogenetic branches for parameter estimation. Consequently, fitting an inadequate genetic code to a particular branch does not affect the estimated value of dN/dS for any other branch. Second, because there are only 1–38 CUG codons per gene in each CTG clade species (Santos et al. 2011), and the P. tannophilus genome sequence comprises only a few hundred CUG codons in its entire protein set (Riley et al. 2016), the impact of mistranslation of a few codons should be negligible during the estimation of the dN/dS parameter for each phylogenetic branch (in the case of the aBSREL model) or for each site (in the case of the FUBAR model). These two facts suggest that the gathered results are close to those obtained if the HyPhy software suite were able to handle the three different genetic codes simultaneously.

### Integration of bioinformatics results into 2D protein structure models

The bioinformatics results obtained in this study using the models provided by the HyPhy software suite were graphically integrated with the remaining in silico results using the following pipeline. The RCytoscape library (Rcytoscape 1.10.0 n.d.) and scripting in the R programming language were used to construct the alignment of amino acid sequences from selected members of the Snq2/Pdr18 subfamily within the Cytoscape 2.8.3 graphical environment (Shannon et al. 2003, Cytoscape 2.8.3 n.d.). Subsequently, the dot-app package (Fitts et al. 2017, Dot app 0.9.6 n.d.) was used to export the aligned sequences. Finally, this dot file was imported into OmniGraffle (Omnigraffle 7.22.5 n.d.), and the graphical environment provided by this software was used to integrate the aligned amino acid sequences of Snq2/Pdr18 subfamily members with the following information: (i) the reconstruction of the amino acid sequences of selected ancestral Snq2/Pdr18 proteins (determined by the MrBayes software suite); (ii) the EBF values and the number of synonymous substitution (dS) and nonsynonymous substitution (dN) simulated for the statistically significant sites (determined by the MEME model); (iii) the sites detected using the more stringent MEME/MrBayes approach; (iv) the LogOddsLogo entropy score (in bits) representing the amino acid sequence conservation of the members of the Snq2/Pdr18 subfamily; (v) the dN/dS values estimated for each site (determined using the FUBAR model); (vi) the location of each protein motif and the moderately conserved amino acid regions identified in this study; and (vii) the “consensus core” obtained for each transmembrane segment based on the 2D protein structure predictions made by five software suites and PolyPhobius.

### Integration of motif prediction and MEME/MrBayes results into 3D protein structure models

The Saccharomyces Genome Database (Huson et al. 2007) was used to interface with the AlphaFold DB (Jumper et al. 2021, Varadi et al. 2022) to gather the predicted 3D protein structure models for the yeast proteins Snq2 (https://alphafold.ebi.ac.uk/entry/P32568) and Pdr18 (https://alphafold.ebi.ac.uk/entry/P53756). To confirm that these two models closely represent the real protein structures of Snq2 and Pdr18, the PROCHECK software (Laskowski et al. 1993) (https://saves.mbi.ucla.edu) was used to construct the corresponding Ramachandran plots (Ramachandran et al. 1963). The Ramachandran plot constructed for the Snq2 3D protein structure model showed the following results: 1212 (90.0%) residues in the most favored regions, 112 (8.3%) residues in additional allowed regions, 9 (0.7%) residues in generously allowed regions, and 13 (1.0%) residues in disallowed regions. The Ramachandran plot constructed for the Pdr18 3D protein structure model showed: 1125 (94.5%) residues in the most favored regions, 60 (5.0%) residues in additional allowed regions, 4 (0.3%) residues in generously allowed regions, and 1 (0.1%) residue in disallowed regions.

After the quality assessment step, the two gathered AlphaFold DB PDB format files were imported into the PyMOL (The PyMOL Molecular Graphics System n.d.) graphical environment, and the “show” button of the Object Control Menu was used to color the entire protein structure white. For the graphical integration of motif prediction results into the AlphaFold DB 3D protein structure models, the command “select Motif_X, resi Y_1_ + Y_2_ + Y_3_+···+Y_N_” was used to select the amino acid residues within each motif, with “X” representing each motif 1,2,…,16, and “Y_1_ + Y_2_ + Y_3_+···+Y_N”_ representing the sequence of successive amino acid residues present in each motif X. Then, each of the 16 predicted motifs was assigned a specific color, and the “show” button was used to represent the selected residues in cartoons and sticks. For integrating the results of the MEME/MrBayes analysis into the 3D protein models, the command “sele resi Y_1_ + Y_2_ + Y_3_+···+Y_N_” was used to select the amino acid residues predicted to have been under the past action of episodic positive selection, with “Y_1_ + Y_2_ + Y_3_+···+Y_N_” representing the sequence of the MEME/MrBayes amino acid residues. Then, the “show” button was used to represent the selected residues as spheres and color them red.

Subsequently, the following PyMOL (The PyMOL Molecular Graphics System n.d.) menu options were used to rotate the two protein models around the corresponding axes: Movie → Program → Camera Loop → X-roll → 32 s and Movie → Program → Camera Loop → Y-roll → 32 s. The resulting sequences of PNG files were exported using the Export Movie As menu option. Finally, the Open Image Sequence menu option in QuickTime Player was used to import the two ordered sequences of PNG files and construct two videos in MOV format with the following parameters: frame rate of 24 frames per second, resolution set to “Actual Size,” and encoder set to “Greater Compatibility (H.264).”

## Results

### Identification of the members of the Snq2/Pdr18 subfamily in hemiascomycete yeasts

A previous phylogenetic study conducted by Godinho et al. (2018a) analyzed 171 hemiascomycetous strains, corresponding to 68 yeast species (Table A1_Supplementary Data), identifying a total of 1648 translated ORFs showing protein similarity with members of the ABC-PDR family. Starting from this initial dataset, we used the MUSCLE software suite to align the corresponding amino acid sequences (Supplementary Data A2). The resulting alignment was input into the PHYLIP Protdist and Neighbor algorithms (Felsenstein 1989, PHYLIP 3.6.98 n.d.) to construct a preliminary, distance-based phylogenetic tree representing the yeast ABC-PDR proteins (Supplementary Data A2 and Fig. A3_Supplementary Data). This tree revealed that the distribution of the *S. cerevisiae* ABC-PDR proteins in phylogenetic branches was consistent with previously published studies focusing on ABC-PDR proteins (Gbelska et al. 2006, Seret et al. 2009, Godinho et al. 2018a). This tree also showed that the majority of the homologs of the *S. cerevisiae* Snq2/Pdr18 proteins resided in a single well-defined phylogenetic branch. The existence of potentially more remote members of this gene family in this dataset was also scrutinized in this work. However, the Blastp network traversal approach used by Godinho *et al*. (Godinho et al. 2018a) gathered members of another gene family (hexose transporters) that required manual removal. Therefore, we concluded that it was unnecessary to employ more sensitive algorithms than Blastp (such as Jackhmmer of PSI-BLAST) to identify potentially more remote ABC-PDR homologs. The fact that false positive sequences were retrieved when less stringent Blastp e-values were used, necessitating their subsequent manual removal, clearly indicates that the full set of members of the ABC-PDR gene family present in the GenomeDB has already been identified.

The preliminary analysis of the amino acid sequences gathered from the GenomeDB showed that a total of 277 translated ORFs were *bona fide* members of the Snq2/Pdr18 subfamily. However, the 2D protein structure analysis and the visual inspection of the multiple alignments of the amino acid sequences confirmed the existence of a number of fragments and sequence frameshifts in this protein set. The DNA sequences of 58 Snq2/Pdr18 homologs showed some type of error, although the correction of these sequences involved only minor interventions (the majority involving the removal of a single unknown “N” nucleotide). After correcting the detected sequence errors, 263 Snq2/Pdr18 homologs were considered to comprise full-size structure ABC-PDR proteins. The use of more than one strain to represent a given yeast species resulted in a number of repeated amino acid sequences in the retained protein set. Therefore, only one amino acid sequence per group of identical sequences was retained, resulting in a smaller, simplified protein set comprising a total of 197 non-repeated full-size structure ABC-PDR proteins.

### Phylogenetic analysis of the members of the Snq2/Pdr18 subfamily

**Figure 1.**
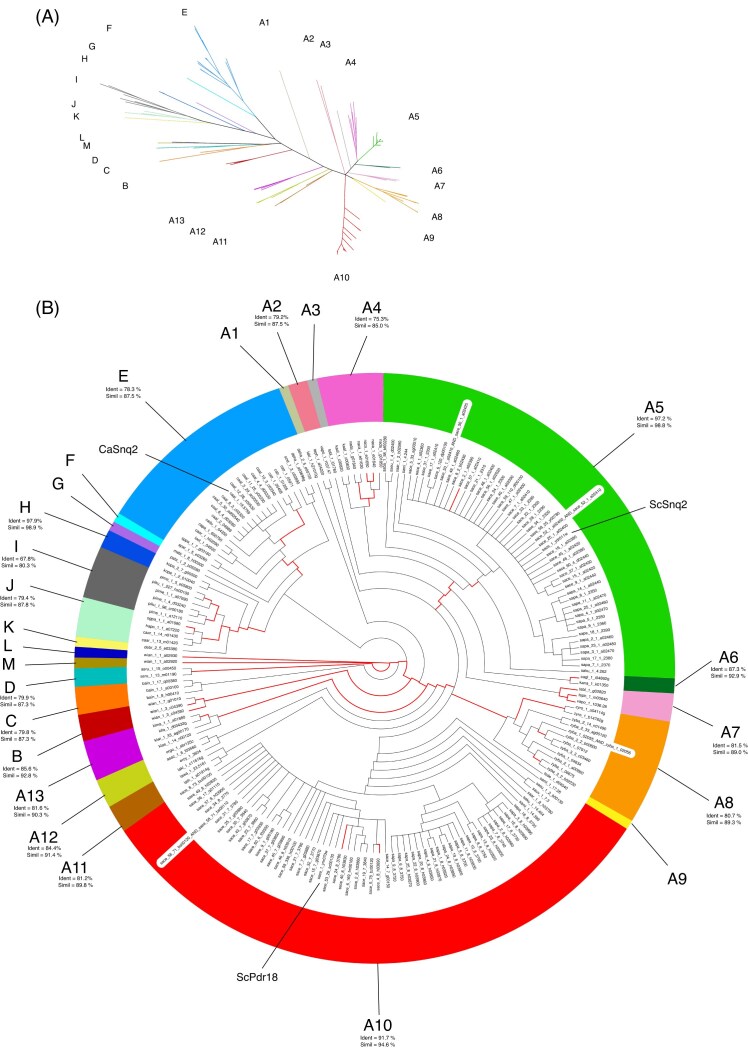
Phylogenetic tree representing the members of the Snq2/Pdr18 subfamily, comprising 197 non-repeated amino acid sequences, divided into 25 clusters, constructed using the MrBayes software suite (Huelsenbeck and Ronquist 2001, Ronquist and Huelsenbeck 2003, MrBayes 3.2.7a n.d.). (A) Radial phylogram showing the amino acid sequence similarity distances between the Snq2/Pdr18 homologs. (B) Circular cladogram showing the tree topology, with phylogenetic branches detected by the aBSREL model as statistically significant for the past action of positive selection highlighted in red. Each cluster shows the average percentage of amino acid sequence identity and similarity shared by the corresponding Snq2/Pdr18 homologs. In the case of phylogenetic clusters comprising only one homolog of the Snq2/Pdr18 proteins, the percentage of identity and similarity is not shown. The colors of the tree branches identify each phylogenetic cluster. The names of the *S. cerevisiae* (ScSnq2 and ScPdr18) and *C. albicans* (CaSnq2) members are indicated. The gene and species annotations adopted in this study use the four-letter code described in Table A1_Supplementary Data. The corresponding multiple alignments and nexus phylogenetic tree are provided in Supplementary Data A4.

The 197 amino acid sequences of the Snq2/Pdr18 homologs were aligned using the Muscle software suite (Edgar 2004, Muscle 3.8 n.d.). This multiple sequence alignment was used to reconstruct a phylogenetic tree representing the diversity of the members within the Snq2/Pdr18 subfamily (Fig. 1 and Supplementary Data A4). A Bayesian-based approach provided by the MrBayes software was used to infer the phylogenetic relationships and distances between the Snq2/Pdr18 homologs (Huelsenbeck and Ronquist 2001, Ronquist and Huelsenbeck 2003, MrBayes 3.2.7a n.d.). The phylogenetic tree representing the members of the Snq2/Pdr18 subfamily was divided into 25 clusters based on the topology, amino acid sequence distance, and MrBayes bipartition probabilities (Fig. 1). The Needleman–Wunsch algorithm, provided by the EMBOSS software suite (Rice et al. 2000, EMBOSS 6.6.0 n.d.), was used to construct all possible pairwise alignments between the 197 non-repeated Snq2/Pdr18 proteins, enabling the determination of global amino acid sequence similarity within the ABC-PDR protein family. On average, the amino acid sequence identity and biochemical similarity shared among the subfamily members were 63.5% and 76.7%, respectively. The percentage of sequence identity and biochemical similarity of Snq2/Pdr18 homologs residing in the same phylogenetic cluster was also determined and is presented in Fig. 1.

This phylogenetic tree allowed us to analyze the taxonomic distribution of these ABC-PDR genes among the different taxonomic families within the Saccharomycotina subphylum. Members of the Snq2/Pdr18 subfamily were found encoded in yeast species belonging to seven taxonomic families: Saccharomycetaceae, Debaryomycetaceae, Metschnikowiaceae, Phaffomycetaceae, Pichiaceae, Wickerhamomycetaceae, and Ascoideaceae (Fig. 1, Table A1_Supplementary Data, and Supplementary Data A4). In the Saccharomycetaceae, the Snq2/Pdr18 homologs showed a bipartition probability of 100% of residing in the same phylogenetic cluster, and all genome sequences of yeast species classified in this taxonomic family encoded at least one member of this subfamily. These findings suggest that the Snq2/Pdr18 homologs in the Saccharomycetaceae have descended from one common ancestral gene. However, the only yeast species considered in this study that is classified in the sister clade of the Saccharomycetaceae family, *Hanseniaspora valbyensis* (Saccharomycodaceae family), does not encode a member of the Snq2/Pdr18 subfamily.

In the Debaryomycetaceae, 10 out of 14 yeast species classified in this CTG clade group encode at least one Snq2/Pdr18 homolog. Although the genome sequences of the late-divergent Debaryomycetaceae species show an abundance of Snq2/Pdr18 homologs, one late-divergent Debaryomycetaceae species, *C. maltosa*, does not encode any Snq2/Pdr18 homolog, possibly due to gene loss. The four ancestral CTG clade species belonging to this taxonomic family, *M. guilliermondii* (*Meyerozyma* genus), *Pichia sorbitophila* (*Millerozyma* genus), *C. tenuis* (*Yamadazyma* genus), and *C. tanzawaensis* (Kurtzman 2011), lack Snq2/Pdr18 homologs. In the sister clade of the Debaryomycetaceae, corresponding to the Metschnikowiaceae taxonomic family (Groenewald et al. 2023), the genome sequences of *Hyphopichia burtonii* and *Clavispora lusitaniae* species lack Snq2/Pdr18 homologs, while *Metschnikowia bicuspidata* encodes one Snq2/Pdr18 homolog. This study also included a more ancestral CTG clade yeast species, *Babjeviella inositovora* (Groenewald et al. 2023). The phylogenetic tree showed that this yeast species encodes three Snq2/Pdr18 homologs, which exhibit strong amino acid similarity with proteins encoded in species classified in the Wickerhamomycetaceae and Ascoideaceae families.

In the remaining taxonomic families and phylogenetic groups belonging to the Saccharomycotina subphylum, we observed a heterogeneous pattern regarding the taxonomic dispersal of the Snq2/Pdr18 homologs (Fig. 1, Table A1_Supplementary Data, and Supplementary Data A4). All six yeast species classified in the Pichiaceae family encode Snq2/Pdr18 homologs. In the Wickerhamomycetaceae family, the *Wickerhamomyces anomalus* strain NRRL Y-366 encodes five Snq2/Pdr18 homologs. In the Phaffomycetaceae family, all the *Komagataella pastoris* strains analyzed in this work encode one Snq2/Pdr18 homolog, while the two *Cyberlindnera jadinii* strains analyzed in this study do not encode any. *Pachysolen tannophilus*, a yeast species that translates the CTG codon into alanine and is classified in the Pachysolenaceae family, was found to encode a single Snq2/Pdr18 homolog. Interestingly, the genome sequences of all early-divergent hemiascomycete species included in the GenomeDB (*Nadsonia fulvescens* var. *elongata, C. caseinolytica, Lipomyces starkeyi*, and *Yarrowia lipolytica*) lack members of the Snq2/Pdr18 subfamily. This observation was confirmed by two blastp searches at Genbank performed against all the proteins encoded in the yeast species classified in the Trigonopsidaceae (taxid:1 540 145), Dipodascaceae (taxid:34 353), Trichomonascaceae (taxid:410 830), and Lipomycetaceae (taxid:29 827) taxonomic families using the *S. cerevisiae* Snq2 and Pdr18 proteins as query amino acid sequences.

### Gene neighborhood analysis of the Snq2/Pdr18 homologs

In a previous study by our research group, gene neighborhood analysis was used to reconstruct the evolutionary history of the members of the Snq2/Pdr18 subfamily encoded in Saccharomycetaceae (Godinho et al. 2018a). Therefore, in this section, only yeast species belonging to the CTG clade or classified in taxonomic families other than Saccharomycetaceae were targeted for detailed evolutionary analysis using this comparative genomics approach.

**Figure 2.**
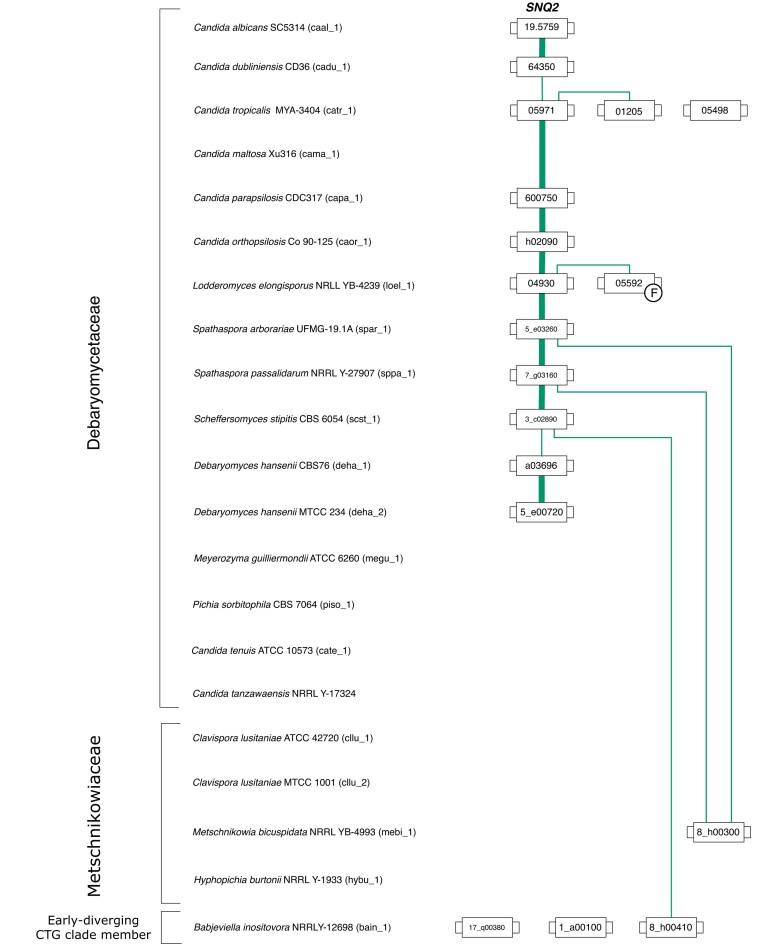
Gene lineage comprising the members of the Snq2/Pdr18 subfamily encoded in the CTG clade species analyzed in this study. The genes represented in this figure are encoded in yeast species classified within the Debaryomycetaceae or Metschnikowiaceae taxonomic families, or in the early-diverging CTG clade yeast, *Babjeviella inositovora*. Each box represents a gene. Lines connect genes that share common neighbors. *F* indicates that the corresponding gene was classified as a fragment. Line thickness represents the strength of synteny between genes. The 30 neighborhood and the truncated 5 neighborhood chromosome environments where these genes reside are provided in Supplementary Data A8 and Fig. A9_Supplementary Data, respectively.

The gene neighborhood analysis showed that the chromosomal environments where the majority of the Snq2/Pdr18 homologs reside have been strongly conserved in the CTG clade species after the divergence of *D. hansenii* (Supplementary Data A8 and Fig. A9_Supplementary Data). The Snq2/Pdr18 homologs encoded in the *D. hansenii* genome showed poor synteny with those found in the late-divergent Debaryomycetaceae yeasts (Supplementary Data A8 and Fig. A9_Supplementary Data). In fact, these *D. hansenii* ABC-PDR genes share only three common neighbors with the Snq2/Pdr18 homologs encoded in the genome sequence of *S. stipitis* (members of the clusters of amino acid sequence similarity 225, 2671, and 13 543). In the late-divergent CTG clade species, the members of the clusters of amino acid sequence similarity 225, 13 543, and 13 836 were the most frequently found in the vicinity of the Snq2/Pdr18 homologs. The *C. albicans SNQ2* gene (caal 1 19.5759) (Karababa et al. 2004) was also confirmed as the sole member of the Snq2/Pdr18 subfamily encoded in this CTG clade species (Gaur et al. 2005) (Fig. 2). This analysis also led to the conclusion that the majority of yeast species classified in the Debaryomycetaceae family encode only a single Snq2/Pdr18 homolog in their genome sequences. An exception to this rule is observed in *C. tropicalis* and *Lodderomyces elongisporus*, where a number of chromosomal rearrangements have resulted in several gene expansion events, leading to the formation of three Snq2/Pdr18 homologs in the genome sequence of *C. tropicalis* and two Snq2/Pdr18 homologs in the genome sequence of *L. elongisporus* (Fig. 2, Supplementary Data A8, and Fig. A9_Supplementary Data). These chromosomal rearrangements have resulted in a significant loss of synteny between the three *C. tropicalis* genes and the Snq2/Pdr18 homologs encoded in the other CTG clade species (Fig. 2, Supplementary Data A8, and Fig. A9_Supplementary Data). Overall, these findings support the vertical origin of these ABC-PDR genes during the speciation events that led to the formation of the various species included in the Debaryomycetaceae yeasts (Fig. 2).

In the Metschnikowiaceae family, the gene neighborhood analysis showed that ORF mebi_1_8_h00300 shares weak synteny (only two common neighbors) with the Snq2/Pdr18 homologs encoded in the two *Spathaspora* genomes analyzed in this study (Supplementary Data A8 and Fig. A9_Supplementary Data). In addition, the analysis of the phylogenetic tree demonstrated that the amino acid sequence of ORF mebi_1_8_h00300 diverges just before the phylogenetic branch encompassing all the Snq2/Pdr18 homologs encoded in the Debaryomycetaceae yeasts, suggesting a close evolutionary relationship between this *M. bicuspidata* ORF and the Debaryomycetaceae Snq2/Pdr18 proteins. Conversely, the genome sequences of three ancestral Debaryomycetaceae yeast species, *C. tanzawaensis, C. tenuis, M. guilliermondii*, and *P. sorbitophila*, and two out of the three Metschnikowiaceae species analyzed in this study lack Snq2/Pdr18 homologs, suggesting that the origin of Snq2/Pdr18 homologs in the Debaryomycetaceae and Metschnikowiaceae families is independent. Because *B. inositovora* is an ancestral CTG clade species that does not belong to the aforementioned taxonomic families (Groenewald et al. 2023), the Snq2/Pdr18 homologs encoded in its genome sequence were analyzed in an attempt to gain new insights into the origin of this gene subfamily in the CTG clade species. This yeast species encodes three Snq2/Pdr18 homologs sharing strong amino acid sequence similarity among them (79.9% sequence identity and 87.3% amino acid biochemical similarity, respectively) but also strong sequence similarity with the Snq2/Pdr18 homologs encoded in the Wickerhamomycetaceae and Ascoideaceae genomes (Fig. 1). These results suggest that this ancestral CTG clade species might have acquired these ABC-PDR genes by lateral transference from a donor species belonging to one of these two taxonomic families. In addition, considering these amino acid sequence similarity results, it is likely that the weak synteny observed between ORF bain_1_8_h00410 and *S. stipitis* ORF scst_1_3_c02890 (only two common neighbors) is spurious, and the product of random genome rearrangements (Supplementary Data A8 and Fig. A9_Supplementary Data). These results show that the existing phylogenetic resolution is not sufficient to discern the true evolutionary trajectories followed by these genes and that reaching a consensual scenario for the origin of the members of the Snq2/Pdr18 subfamily included in the CTG clade will require sampling additional yeast species belonging to the taxonomic families and phylogenetic groups mentioned above.

Gene neighborhood analysis was used to uncover new insights into the evolution of the members of the Snq2/Pdr18 subfamily in twelve yeast strains that do not belong to the Saccharomycetaceae family or the CTG clade. These strains are classified into five taxonomic families (Groenewald et al. 2023): (i) Wickerhamomycetaceae (one *W. anomalus* strain), (ii) Pichiaceae (one *P. kudriavzevii* strain, one *P. membranifaciens* strain, one *D. bruxellensis* strain, one *O. parapolymorpha* strain, one *H. polymorpha* strain, and one *C. arabinofermentans* strain), (iii) Phaffomycetaceae (three *K. pastoris* strains), (iv) Ascoideaceae (one *A. rubescens* strain), and (v) Pachysolenaceae (one *P. tannophilus* strain). Strong synteny was observed (i) among the Snq2/Pdr18 homologs encoded in the *D. bruxellensis, O. parapolymorpha, H. polymorpha*, and *C. arabinofermentans* species (Pichiaceae family) and (ii) among the three Snq2/Pdr18 homologs encoded in the *K. pastoris* yeast strains (Phaffomycetaceae family) (Supplementary Data A8, Fig. A10_Supplementary Data, and Fig. A11_Supplementary Data). On the other hand, the synteny evidence linking the members of the Snq2/Pdr18 subfamily encoded in the remaining strains mentioned above is weak, consisting of only a few common neighbors (Supplementary Data A8, Fig. A10_Supplementary Data, and Fig. A11_Supplementary Data).

### Analysis of the conservation of the amino acid sequences, identification of protein motifs, and integration of predicted motifs into protein structure models

The LogOddsLogo (Yu et al. 2015, LogLogoOdds 1.1.1 n.d. ) entropy analysis of the members of the Snq2/Pdr18 subfamily using a sliding-window approach enabled the detection of 16 highly conserved regions in the amino acid sequences of these proteins (Table 1, Table A12_Supplementary Data, and Supplementary Data A13). Because these regions exhibited a high sequence conservation classification score and spanned more than seven amino acid residues in length, they were designated as protein motifs.

**Table 1.** List of the 16 predicted protein motifs along with the following complementary information: (i) Protein logos; (ii) Whether a specific motif is known in the published literature and, if so; (iii) The corresponding name; (iv) The protein region where each of these motifs resides; and (v) The color used to identify each motif in Video 1.

Motif	Amino acid sequence logo	Is a known protein motif?	Protein region	Name	Color in Video 1
1	L[V/A]LG[R/K]PG[A/S]G[C/S][S/T][S/T]	Yes	N-NBD	Walker A motif	Red
2	EX[D/E]XH[F/Y/L]P[H/Y/F][L/I][T/N][V/L]	Yes	N-NBD	Q loop	Green
3	[R/S]G[V/I]SGG[E/Q]RKRVS[I/L]AEA	Yes	N-NBD	ABC signature	Blue
4	[G/A]XX[Y/F/T]X[W/F/Y]D[N/S][A/S][T/S]R[G/E]LD[A/S/X]S[T/S]	Yes	N-NBD	Walker B motif	Yellow
5	[V/I/C]XXYQ[A/P/Y][S/G/A]EXI[Y/F]	Yes	N-NBD	H loop	Magenta
6	[Y/F]XXX[H/N/Y]XWRNX[G/A][I/F]X	No	Half of this motif resides in the EL3, the other half of this motif resides in the TMS6	–	Cyan
7	GX[L/M/I][T/V]AL[M/I]G[E/A]SGAGKTTLLN[T/V]	Yes	C-NBD	Walter A motif	Orange
8	[F/S]X[R/K/S][R/S/K][T/V]GYVQQQDXHX	Yes	C-NBD	Q loop	Wheat
9	GL[N/S]V[E/G]Q[R/K]KK[L/V][S/T][I/V]	Yes	C-NBD	ABC signature	Brown
10	[K/R]PX[L/I]LLF[L/V]DEP[T/S]SGLD[S/F]Q[S/A/E][A/S][X/L]	Yes	C-NBD	Walker B motif	Green Forest
11	[L/I][A/S/T]X[A/S]GQ[S/A/C][I/V][L/I]CT[I/V]HQPS[A/S][T/V]L[F/I]E	Yes	C-NBD	H loop	Blue Density
12	GGX[T/V][V/I/T]Y[F/C/W][G/N]	No	Inter-region between C-NBD and C-TMD	–	Yellow Sand
13	[E/D]NPAEY[I/V/M]LXXIGAG	No	Inter-region between C-NBD and C-TMD	–	Deep Purple
14	SXX[F/Y][H/R]WX	No	ICL3	–	Light Teal
15	XX[P/V]X[F/W]W-[T/K/H][F/W]MX	No	ECL6	–	Deep Olive
16	XXXWRNX	No	ECL6	–	Blue White

The LogOddsLogo Entropy Score/Moving Average Functions results and the frequentist profiles of the 16 predicted motifs are provided in Table A12_Supplementary Data and Supplementary Data A13, respectively.

On the other hand, the predicted 3D protein structure models for the *S. cerevisiae* proteins Snq2 (https://AlphaFold.ebi.ac.uk/entry/P32568) and Pdr18 (https://AlphaFold.ebi.ac.uk/entry/P53756) were obtained from the AlphaFold DB (Jumper et al. 2021, Varadi et al. 2022) via the interface provided by the SGD DB (Saccharomyces Genome Database n.d.) (Video 1, Supplementary Data A14, and Supplementary Data A15). The analysis of the data points representing the combination of Phi and Psi angles occurring in individual amino acid residues in the Ramachandran plots (Ramachandran et al. 1963), constructed for these two models using the PROCHECK software (Laskowski et al. 1993), confirmed that both predicted 3D protein structures exhibited high stereochemical quality (Fig. A16_Supplementary Data and Fig. A17_Supplementary Data).

Subsequently, the 16 predicted protein motifs (Table 1, Table A12_Supplementary Data, and Supplementary Data A13) were mapped onto the 2D protein structure models (Fig. A30_Supplementary Data) and the 3D protein structure models developed in this study (Video 1, Supplementary Data A14, and Supplementary Data A15). In addition to the motifs known to be present in the cytoplasmic NBDs of the ABC-PDR proteins, six additional motifs, not previously described in the published literature, were detected in the amino acid sequence of the members of the Snq2/Pdr18 subfamily (Video 1, Table A12_Supplementary Data, and Supplementary Data A13): motif 6, located at the beginning of the TMS 6; motifs 12 and 13, both residing between the C-NBD H loop and TMS 7; motif 14, located within Intracellular loop (ICL) 3; and motif 15 and motif 16, both residing in Extracellular loop (ECL) 6. Interestingly, the ICL 3 and ECL 6 regions have been reported to comprise conserved structural features playing an important role in the transport mechanism (Lamping et al. 2010). In addition, due to its proximity to TMS 11, motif 15 is likely to correspond to the conserved hydrophobic motif predicted to form a beta-sheet structure in the ABC proteins (Lamping et al. 2010).

The entropy analysis of the members of the Snq2/Pdr18 subfamily also identified 17 reasonably conserved regions in the amino acid sequence of these ABC-PDR proteins (Table A12_Supplementary Data and Supplementary Data A13). However, these regions were not assigned the status of protein motifs. This decision was based on two factors: (i) a lower sequence conservation classification score (equal to 1 or 2, contrasting with the higher scores associated with the motifs) and (ii) a total length limited to only the seven amino acid residues included in the fixed MA window, whereas all motifs allowed the extension of the MA window to include adjacent columns in the multiple alignment. Nevertheless, the sequence conservation observed in these regions is considered genuine and noteworthy.

The amino acid sequences of the new protein motifs and the reasonably conserved regions newly identified in the *S. cerevisiae* Snq2 and Pdr18 proteins were used to perform a search against two reference DBs focused on the characterization of biological domains and sequence motifs: (i) the PROSITE collection of known protein motifs and (ii) the protein signatures compiled by the Interpro DB (which integrates contributions from 13 reference DBs involved in the classification of proteins using a variety of different methodologies). The sequence searches confirmed the absence of significant hits against the entire set of known protein domains and motifs, confirming the novelty of the motifs identified in this work.

### Evolution of the various truncated isoforms of the Pdr18 protein in species of the *Saccharomyces* genus

**Figure 3.**
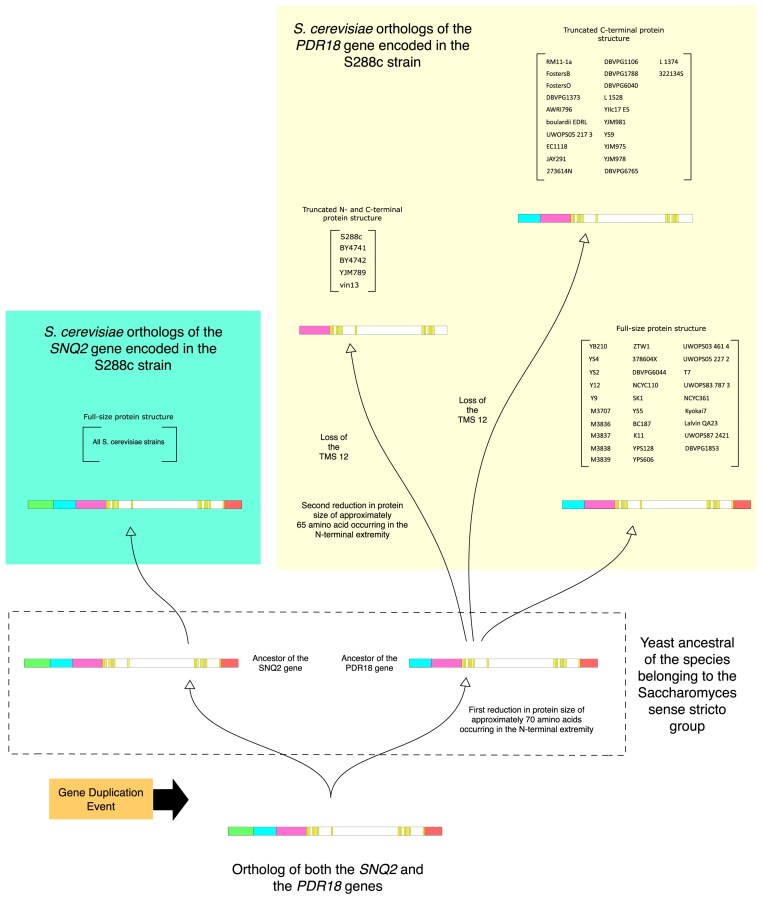
Evolution of the truncated isoforms of the members of the Snq2/Pdr18 subfamily in the *Saccharomyces* genus. The *S. cerevisiae* strains encoding each type of *PDR18* isoform are shown in brackets. The local gene duplication event at the origin of the *SNQ2* and the *PDR18* sublineages is indicated.

The comparison of the members of the Snq2/Pdr18 subfamily encoded in the *Saccharomyces* species with those encoded in the non-*Saccharomyces* post-WGD species revealed that, with the exception of ORF sami_1_17.26, all Pdr18 orthologs exhibit a shorter N-terminal extremity (Video 1 and Fig. 3). Since the Snq2 homologs encoded in the *Saccharomyces* species and the Snq2/Pdr18 homologs encoded in non-*Saccharomyces* post-WGD species have ~75–180 additional amino acid residues, the size difference between the Snq2 and Pdr18 homologs is considerable. Video 1 clearly highlights the difference in length of the N-terminal extremities of these two ABC-PDR proteins. The simplest explanation for the shorter N-terminal extremity of Pdr18 proteins is the occurrence of a deletion in the DNA sequence of the ancestral gene that gave rise to all Pdr18 orthologs after the gene duplication event (Fig. 3, protein portion identified by the green block).

In addition to this ancestral sequence deletion, the analysis of the aligned sequences of the members of the Snq2/Pdr18 subfamily led to the conclusion that the Pdr18 orthologs can be classified into three distinct categories, based on the presence or absence of additional deletions (Fig. 3). The first category comprises Pdr18 orthologs that do not show deletions in their amino acid sequences, a pattern observed in 19 of the 60 *S. cerevisiae* strains analyzed in this study. The second category comprises Pdr18 orthologs that exhibit a deletion in the C-terminal extremity, a pattern observed in 22 of the 60 *S. cerevisiae* strains analyzed in this study. The occurrence of this deletion has the consequence of imposing the loss of the twelfth TMS and a change in the compartmental localization of the C-terminal extremity in this type of Pdr18 ortholog, shifting from the cytosolic compartment to the cell exterior. The third category comprises Pdr18 orthologs showing deletions in both N-terminal and C-terminal extremities. This pattern is observed in 5 of the 60 *S. cerevisiae* strains analyzed: the reference strain S288c (the predicted 3D structure of this protein is detailed in Video 1), the S288c-derived auxotrophic strains BY4741 and BY4742, the clinical strain YJM789, and the wine strain Vin13. The N-terminal sequence deletion in these strains results in a shortening of the Pdr18 orthologs by more than 80 amino acid residues. It is likely that the truncation of the C-terminal extremity and the modification of the 2D topology of the Pdr18 orthologs classified in categories II and III have a significant impact on protein function and stability (Mason et al. 2014, Lin et al. 2017).

### Models of molecular evolution

Three models provided by the HyPhy phylogenetic suite—aBSREL (Smith et al. 2015), FUBAR (Murrell et al. 2013), and MEME (Murrell et al. 2012)—were used to detect the past action of selective forces on the members of the Snq2/Pdr18 subfamily.

#### Identification of phylogenetic branches under the past action of selective forces

The aBSREL model (Smith et al. 2015) was used to identify branches that have undergone positive selection in three different phylogenetic trees representing the members of the Snq2/Pdr18 subfamily. First, the aBSREL model was employed to detect instances of positive selection in the 123 non-repeated amino acid sequences of the members belonging to the WGD sublineage of the ABC-PDR subfamily at the origin of the *S. cerevisiae* Snq2/Pdr18 genes (the “long-lived” WGD sublineage). Previous research by my group established that the duplication event responsible for these two *S. cerevisiae* genes occurred near the root of the *Saccharomyces* clade. To ensure accurate analysis, specific topology constraints were imposed when constructing the MrBayes phylogenetic tree: (i) All orthologs of the *SNQ2* gene were grouped within one phylogenetic branch, (ii) all orthologs of the *PDR18* gene were grouped within another phylogenetic branch, and (iii) the orthologs of the *SNQ2* and *PDR18* genes coalesced into a single phylogenetic branch, excluding any other subfamily members. Branches with an aBSREL-corrected *P*-value below .05 were considered statistically significant (Supplementary Data A18 and Fig. A19_Supplementary Data). The aBSREL algorithm assigned two rate categories to model the evolution of the ancestral gene at the origin of the *PDR18* gene sublineage: 88.4% of the amino acid sequence of this ancestral gene was inferred to have been under purifying selection, while the remaining 11.6% was inferred to have been under strong positive selection [estimated dN/dS = 10.3, Likelihood Ratio Test (LRT) = 27.3, uncorrected *P*-value = 3.8 × 10 − 7, corrected *P*-value = 6.7 × 10 − 5] (Supplementary Data A18 and Fig. A19_Supplementary Data). This result indicates that the ancestral PDR18 ortholog experienced strong positive selection along the branch leading to its divergence, consistent with potential adaptive changes following gene duplication. Although two rate categories were also assigned to model the evolution of the ancestral gene at the origin of the *SNQ2* sublineage, the aBSREL model did not consider this result statistically significant: 90.7% of the amino acid sequence of this ancestral gene was inferred to have been under negative selection, while the remaining 9.3% was inferred to have been under mild positive selection (estimated dN/dS = 2.95, LRT = 8.2) (Supplementary Data A18 and Fig. A19_Supplementary Data). The aBSREL model predicted that 2.3% of the amino acid sequence encoded by the ancestral gene that gave rise to most Snq2/Pdr18 subfamily members in *Kazachstania* and *Naumovozyma* species was under strong positive selection (LRT = 16.5, uncorrected p-value = 8.7 × 10 − 5, corrected *P*-value = .015) (Supplementary Data A18 and Fig. A19_Supplementary Data). The sequence of ORF cagl_1_i04862, encoded in the sole *Nakaseomyces* species analyzed in this study, also showed a strong signal of positive selection (LRT = 16.5, uncorrected *P*-value = 4.5 × 10 − 5, corrected *P*-value = .0079) (Supplementary Data A18 and Fig. A19_Supplementary Data). On the other hand, kana_1_k01350, the ORF that clusters with cagl_1_i04862 in the phylogenetic tree, did not show evidence of past positive selection. The *N. castellii* and *N. dairensis* Snq2/Pdr18 homologs were also identified as being under strong diversifying selection [Node117 (LRT = 17.8, *P* = .0098); Node118 (LRT = 20.1, *P* = .003); Node121 (LRT = 29.2, *P* = .00003)] (Supplementary Data A18 and Fig. A19_Supplementary Data). This result is consistent with the functional differentiation of these Snq2/Pdr18 homologs during their expansion through successive local gene duplication events. Two extant genes encoded in *S. cerevisiae* strains AWRI796 and ForstersB were also identified as being under strong positive diversifying selection [sace_2_1_a02390 (LRT = 40.2, *P* = 1.3E-7); sace_4_8_h03690 (LRT = 110.4, *P* = .0)] (Supplementary Data A18 and Fig. A19_Supplementary Data).

Second, a total of 146 non-repeated Snq2/Pdr18 amino acid sequences were identified in the genomes of the Saccharomycetaceae species analyzed in this study. The abundance of Snq2/Pdr18 homologs and the diversity of yeast species within the Saccharomycetaceae family provided an opportunity to use the aBSREL model to investigate the relationship between the functional differentiation of these genes and the origin of each genus within the taxonomic family. Previous research from my group demonstrated that the WGD event resulted in the formation of two distinct sublineages. One sublineage gave rise to the *S. cerevisiae SNQ2* and *PDR18* genes, while the other encompassed three ABC-PDR genes encoded in *Tetrapisispora blattae, T. phaffii*, and *Vanderwaltozyma polyspora*. This phylogenetic information was incorporated into the MrBayes input parameter file using three topology constraints. The results obtained with the aBSREL model indicated that the first ancestral homolog of the *SNQ2/PDR18* genes in post-WGD species did not show evidence of past positive selection (LRT = 0.4504, *P* = 1.0). In contrast, the phylogenetic branch leading to the ancestral gene that gave rise to the three ABC-PDR genes in *T. blattae, T. phaffii*, and *V. polyspora* exhibited a strong signal of positive selection (LRT = 16.4, *P* = .0192) (Supplementary Data A20). In fact, all the phylogenetic branches leading to these three genes were identified as having undergone strong positive selection (Supplementary Data A20). The phylogenetic branches leading to the following yeast species—(i) the *Torulaspora/Zygosaccharomyces* genus (LRT = 22.07, *P* = 1.17E-3), (ii) the *Zygosaccharomyces* genus (LRT = 16.28, *P* = .02), and (iii) the two Snq2/Pdr18 homologs encoded in *Z. rouxii* (LRT = 21.82, *P* = 1.3E-3)—also showed signs of the past action of positive selection (Supplementary Data A20).

Third, the aBSREL model was used to detect the past positive selection on the 197 non-repeated sequences of the Snq2/Pdr18 homologs. The aim was to gain insight into the evolutionary process of these ABC-PDR genes in deep phylogenetic branches or within clades representing stable taxonomic groups. However, it should be noted that the aBSREL model cannot handle DNA sequences requiring multiple genetic codes for translation. Given that yeast species employing distinct genetic codes were included in this analysis, and that the universal genetic code was used for translating all 197 non-repeated sequences of the Snq2/Pdr18 homologs, caution should be exercised when interpreting the results obtained for genes encoded in the genomes of CTG clade species and *P. tannophilus*. These findings should be considered only as an approximation. The gathered (approximate) results indicate that the phylogenetic branches leading to most of the genes at the origin of all Snq2/Pdr18 homologs encoded in ancestral yeasts at the taxonomic family level were statistically significant (Fig. 1 and Supplementary Data A21): Saccharomycetaceae (LRT = 49.4, *P* = 1.8E-09), Debaryomycetaceae (LRT = 59.4, *P* = 1.2E-11), CTG clade (branch comprising both the Debaryomycetaceae and the Metschnikowiaceae families) (LRT = 67.5, *P* = 2.3E-13), Wickerhamomycetaceae [wian_1_1_a02930 (LRT = 51.4, *P* = 6.7E-10); wian_1_1_a02920 (LRT = 37.9, *P* = 5.7E-07); Node305 (LRT = 65.1, *P* = 7.1E-13)], and Ascoideaceae (LRT = 35.4, *P* = 2.0E-06) (Fig. 1 and Supplementary Data A21). The phylogenetic branch encompassing the Snq2/Pdr18 homologs encoded in yeast species classified in the Pichiaceae family was also found to be statistically significant (LRT = 106.7, *P* = 0.0) (Fig. 1 and Supplementary Data A21).

#### Identification of sites under the past action of selective forces

The FUBAR model (Murrell et al. 2013) was utilized to detect the past action of selective forces on individual sites within the multiple alignments of Snq2/Pdr18 subfamily members. Overall, the results obtained using the FUBAR model showed that negative selection was the dominant selective force shaping the evolution of Snq2/Pdr18 homologs in hemiascomycete yeasts (Supplementary Data_A22, Supplementary Data_A23, Supplementary Data_A24, Fig. A30_Supplementary Data, Fig. A31_Supplementary Data, and Fig. A32_Supplementary Data). This finding is consistent with the strong conservation of amino acid sequences observed in these proteins throughout the evolution of hemiascomycete yeasts (Table A12_Supplementary Data).

At the taxonomic level of the Saccharomycetaceae family, with the exception of a few cases of pervasive positive selection outside the NBD and TMD regions—corresponding to 3.66% of the multiple alignments of Snq2/Pdr18 homologs—the FUBAR model estimated that 89.96% of the aligned sites were under strong purifying selection (0 ≤ w < 0.5), while 6.38% were under weak purifying selection (0.5 ≤ w < 1.0) (Supplementary Data A23 and Fig. A31_Supplementary Data).

At the taxonomic level of the Saccharomycotina subphylum, the FUBAR model estimated that only 4.55% of the aligned sites in the multiple alignments of Snq2/Pdr18 homologs were under pervasive positive selection, consisting of amino acid residues located outside the NBD and TMD regions. Additionally, this model estimated that 78.55% of the sites were under strong purifying selection (0 ≤ w < 0.5) and 16.9% were under weak purifying selection (0.5 ≤ w < 1.0) (Supplementary Data A24 and Fig. A32_Supplementary Data).

The results reported above do not exclude the possibility that episodes of functional diversification of sites occurred at specific points in time during the evolution of Snq2/Pdr18 subfamily members. The next subsection analyzes four combinations of branches and sites selected for their biological interest.

#### Identification of branch-site combinations under the past action of selective forces

The MEME model (Murrell et al. 2012) was used to capture episodes of diversifying evolution affecting individual sites in specific branches of three different Snq2/Pdr18 phylogenetic trees.

First, the MEME model was used to analyze the functional differentiation of the *SNQ2* and *PDR18* genes after the gene duplication event that occurred in the genome of the most recent common ancestor of all species classified in the *Saccharomyces* genus. After setting a cutoff value of 2.0 for the Empirical Bayes Factor (EBF) score, a total of 51 sites were identified as being under episodic positive selection in the first *SNQ2* ancestral gene, while 82 sites were identified in the first *PDR18* ancestral gene (Supplementary Data A25, Supplementary Data A26, Supplementary Data A27, and Fig. A30_Supplementary Data).

It has been reported that the MEME model may overestimate the number of sites predicted to have undergone episodic positive selection (Murrell et al. 2012). To address this potential bias, the MrBayes software suite was utilized to infer the DNA and amino acid sequences of the three ancestral Snq2/Pdr18 homologs involved in the duplication event. These homologs include the pre-duplication gene and the two resulting duplicates before the divergence of *Saccharomyces* species. To ensure greater reliability, a consistency check was imposed between the results obtained from the MEME model and the MrBayes software suite, which led to a reduction in the number of identified sites predicted to have undergone episodic positive selection. The adoption of this consistency check is henceforth referred to as the MEME/MrBayes approach.

**Figure 4.**
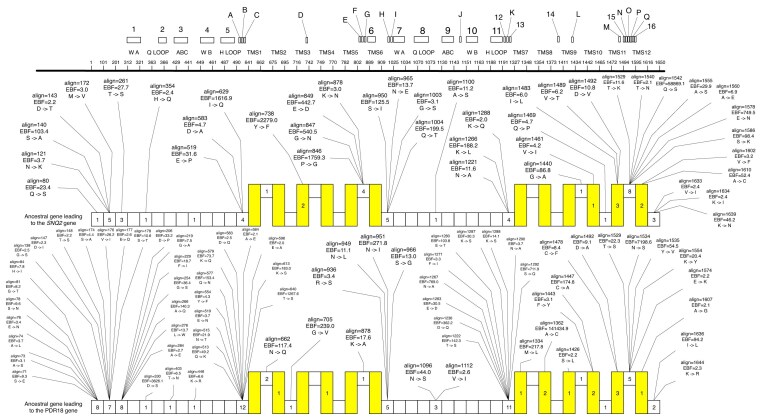
Representation of the sites identified under episodic positive selection by the MEME/MrBayes approach in the three ancestral genes at the origin of the *S. cerevisiae SNQ2* and *PDR18* genes in the *Saccharomyces* genus (the pre-duplication gene and the two resulting duplicates) within the corresponding 2D protein structure models. The location of each detected site (“align=”), the corresponding Empirical Bayes Factor (“EBF=”) score calculated by the MEME model, and the ancestral states of the amino acid residues inferred using the MrBayes software suite (Huelsenbeck and Ronquist 2001, Ronquist and Huelsenbeck 2003, MrBayes 3.2.7a n.d.) are shown. The asymmetric division of the different protein regions is based on known motifs present in the nucleotide-binding domains (NBDs) and on the inferred limits of the transmembrane regions. The locations of each of the 16 protein motifs and the 17 moderately conserved amino acid regions identified in this study are also shown. Fig. A30_Supplementary Data details the locations of the sites detected by the MEME/MrBayes approach at the amino acid sequence level.

Regarding the sublineage leading to the *S. cerevisiae SNQ2* gene, the adoption of the MEME/MrBayes approach resulted in a reduction in the number of sites showing signs of past episodic positive selection, decreasing from 51 to 41 (Fig. 4, Video 2, Supplementary Data A26, and Fig. A30_Supplementary Data). These sites were located in specific regions of the protein: N-region A (6), N-NBD (1), N-region B (3), TMS3 (1), ECL3 (4), middle region A (4), C-NBD (1), middle region B (3), ECL6 (1), TMS10 (1), ICL4 (1), TMS11 (3), ECL6 (7), TMS12 (2), and C-region (3). The amino acid residues corresponding to the detected sites are shown in red in the 3D protein structure model inferred for Snq2 (Video 2). Interestingly, the analysis of the Snq2 model revealed a significant accumulation of amino acid residues (14) detected by the MEME/MrBayes approach in protein regions exposed to the cellular exterior.

Regarding the sublineage leading to the *S. cerevisiae PDR18* gene, the adoption of the MEME/MrBayes approach resulted in a decrease in the number of codon sites indicating past episodic positive selection, reducing from 82 to 69 (Fig. 4, Video 2, Supplementary Data A27, and Fig. A30_Supplementary Data). These sites were located in specific regions of the protein: N-region A (21), N-NBD (3), N-region B (11), ECL1 (1), ICL1 (1), ECL3 (1), middle region A (4), C-NBD (2), middle region B (10), TMS7 (1), TMS8 (1), TMS9 (1), TMS10 (2), TMS11 (2), ECL6 (5), TMS12 (1), and C-region (2). The amino acid residues corresponding to the detected sites are shown in red in the 3D protein structure model inferred for Pdr18 (Video 2). By comparing the amino acid sequences of the Snq2 and Pdr18 proteins with those inferred for the corresponding ancestral genes, it was observed that only 31 and 30 new amino acid residues resulting from dNs detected by the MEME/MrBayes approach were preserved in the two *S. cerevisiae* genes, respectively. In other words, until the emergence of the *S. cerevisiae* species, at least 10 and 39 of the detected sites, where these amino acid substitutions occurred in the Snq2 and Pdr18 orthologs, respectively, were free to evolve or were advantageous for the corresponding *Saccharomyces* populations during the corresponding evolutionary time window.

This study also analyzed the overlap between the sites identified as being under episodic positive selection and the sixteen protein motifs and seventeen moderately conserved regions identified. For the first *SNQ2* ancestral gene, the MEME/MrBayes approach detected one site in region D (TMS 3), one site in region M (TMS 11), and two sites in region P (ECL 6) (Videos 1 and 2, Fig. 4, Supplementary Data A26, and Fig. A30_Supplementary Data). In contrast, for the first *PDR18* ancestral gene, the MEME/MrBayes approach did not identify any amino acid residues under positive selection within these protein motifs and moderately conserved regions (Videos 1 and 2, Fig. 4, Supplementary Data A27, and Fig. A30_Supplementary Data). The MEME/MrBayes results were also used to investigate whether transmembrane regions exhibited a higher occurrence of sites detected as having undergone positive selection in the past (Videos 1 and 2, Fig. 4, Supplementary Data A26, Supplementary Data A27, and Fig. A30_Supplementary Data. The results revealed that out of the 41 detected sites, 7 (17.07%) were located within the transmembrane segments of the Snq2 protein. For the Pdr18 protein, 8 out of the 69 detected sites (11.59%) were found within the transmembrane regions.

Second, the MEME model was used to analyze the functional differentiation of the members of the Snq2/Pdr18 subfamily at the WGD event. After setting a cutoff value of 2.0 for the Empirical Bayes Factor (EBF) score, a total of seven sites were identified as having been under positive selection in the ancestral gene orthologous to the *SNQ2/PDR18* genes (the “long-lived” WGS sublineage) (Supplementary Data A28 and Fig. A31_Supplementary Data). On the other hand, 50 sites were identified in the ancestral genes paralogous to the *SNQ2/PDR18* genes (the “short-lived” WGD sublineage) (Supplementary Data A28 and Fig. A31_Supplementary Data). The MrBayes software suite was employed to infer the amino acid sequences of the pre-WGD ancestral gene and the two post-WGD duplicates (before the divergence of the *Vanderwaltozyma polyspora* species). Subsequently, the MEME/MrBayes approach was applied to obtain a more reliable and restricted set of sites inferred to have been under the past action of positive selection and involved in the differentiation of the Snq2/Pdr18 ohnologs formed at the WGD event.

**Figure 5.**
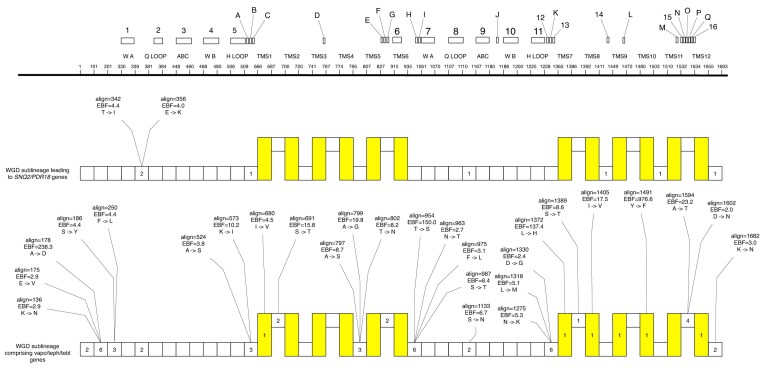
Representation of the sites identified under episodic positive selection in the ancestral genes at the origin of the two sublineages formed during the Whole-Genome Duplication (WGD) event (the pre-WGD gene and the two post-WGD duplicates, also known as ohnologs) in a 2D protein structure model (conventions as in Fig. 4). Fig. A31_Supplementary Data details the locations of the sites detected by the MEME/MrBayes approach at the amino acid sequence level.

Regarding the ancestral gene that gave rise to the WGD sublineage leading to the *S. cerevisiae SNQ2/PDR18* genes, the adoption of the MEME/MrBayes approach resulted in a decrease in the number of codon sites with signs of episodic positive selection from seven to two (Fig. 5 and Fig. A31_Supplementary Data). These two sites reside between the Walker A motif and the Q loop of the N-terminal domain (site A: alignment position 342; site B: alignment position 356). The results obtained using the MEME/MrBayes approach are consistent with those gathered using the aBSREL model, suggesting that positive selection was not a predominant force in the evolution of this ancestral ohnolog gene immediately after the WGD event.

Regarding the ancestral ohnolog gene that gave rise to the “short-lived” WGD sublineage comprising one *Vanderwaltozyma* and two *Tetrapisispora* genes, the adoption of the MEME/MrBayes approach resulted in a decrease in the number of sites with signs of past episodic positive selection from 50 to 27 (Fig. 5 and Fig. A31_Supplementary Data). The 27 detected sites reside in the following regions: N-region A (5), N-region B (2), TMS1 (1), ECL1 (1), ICL2 (3), middle region A (4), C-NBD (1), middle region B (3), TMS7 (1), ECL4 (1), TMS8 (1), TMS10 (1), ECL6 (2), and C-region (1). With the exception of a single site located between the Q loop and the ABC signature motif of the C-NBD, the MEME/MrBayes approach predicted that neither NBD comprised sites under the past action of positive selection, a result consistent with the ATP-binding and hydrolysis functions assigned to these conserved regions. The overlap between sites detected as having undergone past positive selection and the sixteen protein motifs and seventeen moderately conserved regions was also analyzed (Fig. 5 and Fig. A31_Supplementary Data). The results showed no signs of past positive selection in these motifs and moderately conserved regions, dismissing their involvement in the functional differentiation of the Snq2/Pdr18 ancestral ohnolog genes that originated at the WGD event.

Third, the detection of past selective forces acting on branch-site combinations involving the ancestral genes associated with the origin of the Snq2/Pdr18 subfamily members encoded in Saccharomycetaceae yeasts and those encoded in Debaryomycetaceae yeasts was also attempted in this study. However, we concluded that the results obtained for these two specific cases at the family taxonomic level (Supplementary Data A29 and Fig. A32_Supplementary Data) were not trustworthy due to the absence of corroborating evidence. In fact, not only does the deep position of the Saccharomycotina yeasts encoding these two ancestral genes in the phylogenetic tree raise severe concerns regarding the applicability of the methodologies used in this study to detect past selective forces at this taxonomic level, but the lack of Snq2/Pdr18 homologs in the sister taxonomic families of these two yeast groups also impaired the use of the MEME/MrBayes approach in the corresponding analysis.

## Discussion

Previous phylogenetic studies focusing on Snq2/Pdr18 efflux pumps have analyzed a limited number of yeast genomes (Gaur et al. 2005, Gbelska et al. 2006, Seret et al. 2009) or only yeast species belonging to the Saccharomycetaceae family (Godinho et al. 2018a), thus limiting the scope and applicability of these studies. In comparison, the present work expands upon previous evolutionary studies by including yeast species from six additional taxonomic families (Debaryomycetaceae, Metschnikowiaceae, Phaffomycetaceae, Pichiaceae, Wickerhamomycetaceae, and Ascoideaceae) and four early-divergent hemiascomycete yeasts (*N. fulvescens* var. *elongata, C. caseinolytica, L. starkeyi*, and *Y. lipolytica*) (Kurtzman 2011, Groenewald et al. 2023) (Table A1_Supplementary Data). The inclusion of a representative set of members from the Snq2/Pdr18 subfamily allowed the analysis of the evolution of these genes across multiple taxonomic families. The analysis of the taxonomic distribution of these efflux pumps among different families revealed a consistent phylogenetic pattern: their absence in the genomes of early-divergent hemiascomycete species. The origin of these ABC-PDR genes in hemiascomycete yeasts resulted from a horizontal transfer event to the ancestral yeast species, which gave rise to these genes in the late-divergent Saccharomycotina taxonomic families. Overall, the Snq2/Pdr18 homologs showed great heterogeneity in their taxonomic distribution among the late-divergent families in the Saccharomycotina subphylum, with these genes potentially being present, partially absent, or completely absent in these taxonomic groups (Table A1_Supplementary Data).

The evolutionary history of Snq2/Pdr18 subfamily members in CTG clade species was also reconstructed in this work. Gene neighborhood analysis revealed the existence of a single main lineage comprising this type of ABC-PDR protein in the Debaryomycetaceae yeasts. All pathogenic yeast species classified in this taxonomic family (*C. albicans, C. tropicalis, C. parapsilosis, D. hansenii, C. dubliniensis*, and *C. orthopsilosis*) encoded at least one Snq2/Pdr18 subfamily member. The genome sequences of *C. glabrata*, classified in the Saccharomycetaceae family, and *P. kudriavzevii* (*C. krusei*), classified in the Pichiaceae family, also showed one and two Snq2/Pdr18 homologs, respectively. Only the pathogenic yeast *Clavispora lusitaniae* (*C. lusitaniae*) did not encode Snq2/Pdr18 homologs. With this exception, there appears to be an association between the presence of this type of ABC-PDR protein in yeast genomes and pathogenicity, a finding consistent with the role assigned to *S. cerevisiae* Snq2 and Pdr18 and *C. glabrata* Snq2 proteins in yeast drug resistance.

The detection of past actions by selective forces on existing and ancestral genes allows us to gain evolutionary insights that complement traditional tree-building methodologies and comparative genomics approaches. This type of study helps characterize the functional divergence of genes and the selective forces responsible for the fixation of duplicate genes in a population's gene pool. Such studies are rare for two main reasons. First, understanding complex phenomena such as the evolution and functional divergence of two paralogous genes requires detailed biochemical characterization, which is a slow and resource-intensive process. Second, obtaining experimental data to differentiate between various gene duplication models necessitates not only characterizing the functions of the two existing paralogs but also at least one ortholog encoded in a species that diverged before the gene duplication event. By leveraging the well-characterized chemical resistance profiles associated with the two *S. cerevisiae SNQ2/PDR18* genes and the pre-duplication homolog encoded in *C. glabrata* (*CgSNQ2*), the analysis of branch-site combinations performed in this study provided new insights into the process of functional divergence of the two paralogous genes and allowed the detection of amino acid residues with potential evolutionary importance for the physiological function that this type of ABC-PDR proteins plays in yeast cells.

Overall, the models of molecular evolution used in this study showed that strong positive selection was exerted on the first *PDR18* ancestral gene. The aBSREL model estimated a dN/dS value of 10.3, corresponding to 11.6% of the aligned amino acid sequences, while the MEME/MrBayes approach detected a total of 69 sites with signs of past positive selection (Fig. 4 and Video 2). In addition, the analysis of the multiple alignments of Snq2/Pdr18 homologs encoded in *Saccharomyces* species showed that *PDR18* orthologs have accumulated several deletions in their sequence since the duplication event (Fig. 3). For instance, compared to the *SNQ2* amino acid sequence, all *PDR18* orthologs exhibit a shorter N-terminal extremity. This suggests that an ancient deletion occurred in the first *PDR18* ortholog, which was subsequently inherited by all *Saccharomyces* species. Besides this ancestral deletion, 24 of the 60 *S. cerevisiae* strains analyzed in this study encoded *PDR18* amino acid sequences with additional deletions in their N-terminal and/or C-terminal extremities. Among these truncated proteins is Pdr18 itself (encoded in the S288c reference strain). In fact, the C-terminal deletion in Pdr18 is so large that it encompasses the entire TM12 region, resulting in a shift in the compartmental localization of the C-terminal extremity of this protein from the cytosol to the cell exterior. Deletions in the coding sequence of proteins can result in severe functional abnormalities (Mason et al. 2014, Lin et al. 2017). Therefore, it is possible that the truncated isoforms of the Pdr18 protein deviate functionally from the “normal” function associated with the full-size protein. In future work, it would be interesting to confirm whether *S. cerevisiae* strains encoding Pdr18 orthologs, carrying different combinations of these deletions, exhibit the same set of drug susceptibilities as those reported for the BY4741-derived *pdr18∆* mutant strain. Additionally, in future studies, the 3D protein structure of the *S. cerevisiae* Pdr18 protein predicted in this study could help elucidate the impact of the indels occurring in the N-terminal and C-terminal extremities of the Pdr18 gene on the normal positioning of the motifs involved in ATP recognition and hydrolysis of this ABC-PDR protein. This could aid in understanding how these reactions occur mechanistically in the truncated Pdr18 protein, both from a structural and functional perspective.

The models of molecular evolution used in this study to analyze the evolution of the first *SNQ2* ancestral gene uncovered seemingly contradictory results. The MEME/MrBayes approach detected 41 sites with signs of episodic positive selection in this ancestral gene (corresponding to 2.7% of the aligned amino acid sequence) (Fig. 4 and Video 2). On the other hand, the aBSREL model was unable to assign statistical significance to the phylogenetic branch at the origin of the first *SNQ2* ancestral gene, despite a high aBSREL LRT score of 8.17 (Supplementary Data A18 and Fig. A19_Supplementary Data). Nevertheless, two rate categories were used to model the selective forces acting on the first *SNQ2* ancestral gene, with one rate category corresponding to positive selection (estimated dN/dS value of 2.95), encompassing 9.3% of the aligned amino acid sequence. Considering that two mathematical limitations of the aBSREL model reduce the statistical power of the corresponding LR test (details are provided in the “Materials and Methods” section), it seems reasonable to conclude that a small portion of the sequence of the first *SNQ2* ancestral gene truly accumulated a number of mutations immediately after the gene duplication. This phenomenon might be the result of the acquisition of random mutations during the time period when both duplicates were free to evolve due to the existence of two identical copies of the same gene encoded in this ancestral yeast. This explains why the MEME/MrBayes approach detected a limited number of sites with signs of positive selection. Interestingly, the existence of 14 amino acid residues detected by the MEME/MrBayes approach in the cellular exterior (Fig. 4 and Video 2) indicates that the extracellular regions of the Snq2 protein might have been the preferred target for these mutations.

Which evolutionary scenario—neofunctionalization, subfunctionalization, or gene dosage effect—best explains the functional differentiation of the *SNQ2* and *PDR18* genes? The gene dosage effect scenario is highly unlikely due to the strong evidence uncovered in this study, which supports the role of positive selection in driving the evolution of at least one of the gene copies after the duplication event. Although the subfunctionalization scenario cannot be completely ruled out—since both *S. cerevisiae* genes confer resistance to chemical compounds not observed in the *CgSNQ2* gene, and the MEME/MrBayes approach detected signs of positive selection in the sequence of the first *SNQ2* ancestral gene—the evidence of positive selection in this ancestral gene is much weaker compared to the signal detected in the sequence of the first *PDR18* ancestral gene. Furthermore, the mutations occurring in the first *SNQ2* ancestral gene might be the result of random mutations acquired during the period when both gene copies were free to evolve. Hence, the subfunctionalization scenario is not the most plausible explanation. The evolutionary scenario that best fits the gathered in silico results and the available chemical profiling data is the neofunctionalization of the *PDR18* gene. Two findings support this conclusion. First, both the MEME/MrBayes approach and the aBSREL model detected strong positive selection in the sequence of the first *PDR18* ancestral gene. Second, the amino acid sequence encoded by the *S. cerevisiae PDR18* gene has accumulated numerous point mutations and a variable number of deletions since the gene duplication event (with the number of deletions depending on the *S. cerevisiae* strain analyzed), resulting in the acquisition of yeast resistance to a wider range of chemical compounds compared to those associated with the *SNQ2* and *CgSNQ2* genes. In summary, the neofunctionalization model known as “Gene Duplication With a Modified Function” (consult (Innan and Kondrashov 2010) for further details) aligns with the in silico results and chemical profiling data, indicating that a new function emerged in the first *PDR18* ancestral gene while the original function was preserved in the first *SNQ2* ancestral gene. Under this scenario, the mutations observed in the extracellular regions of the Snq2 protein might have contributed to the optimization of the original function of the *SNQ2* gene.

New evolutionary insights were also obtained regarding the two Snq2/Pdr18 ohnolog genes. The ohnolog at the origin of the *S. cerevisiae SNQ2* and *PDR18* genes (“long-lived” WGD sublineage) did not show any signs of diversifying selection after the WGD event (Fig. 5). In contrast, the ohnolog at the origin of the three genes encoded in the *T. blattae, T. phaffii*, and *V. polyspora* genomes (“short-lived” WGD sublineage) exhibited a strong signal of past positive selection (Fig. 5). These results suggest that the ancestral function of the Snq2/Pdr18 homologs encoded in the genomes of the protoploid Saccharomycetaceae species might have been preserved in the sublineage at the origin of the *S. cerevisiae SNQ2* and *PDR18* genes, while the other duplicate acquired mutations that led to the development of a new adaptive function, which was retained in the genome sequences of these three yeast species. Although the strong amino acid sequence divergence of the genes within the “short-lived” WGD sublineage, along with the results obtained from the MEME/MrBayes approach and the aBSREL model, support the evolutionary scenario of neofunctionalization, there is currently no available functional data for any of these ohnolog genes. As a result, this scenario remains hypothetical. Therefore, in a future study, it would be interesting to conduct a comprehensive biochemical and physiological characterization of the genes within the “short-lived” WGD sublineage, as well as a few additional Snq2/Pdr18 homologs encoded in the protoploid Saccharomycetaceae species, to confirm the hypothesized functional divergence of these genes.

In this study, we conducted an extensive analysis of the Snq2/Pdr18 gene subfamily, employing various in silico approaches, including conventional phylogenetic methods, Comparative Genomics, Sequence Conservation analysis, and Models of Molecular Evolution. Overall, this work has provided valuable new insights into the evolutionary history and functional characteristics of these genes, particularly from a MDR perspective. Future work should focus on determining the chemical resistance profiles of additional pre- and post-WGD species. This will help confirm the association between drug resistance to specific chemical compounds and evolutionary events involving members of the ABC-PDR subfamily, such as gene duplications and losses. Future studies should also confirm the relationship between residue mutations and the development of drug resistance against specific chemical compounds. Plasmid-based mutagenesis experiments focusing on the Snq2 and Pdr18 genes in the genetic background of the corresponding single-deletion strains would allow the study of changes in amino acid residues to match those observed in paralog genes or those inferred for ancestral genes. This approach could potentially provide new valuable insights into the function of these genes. Furthermore, because the true physiological functions of each paralog gene in yeast cells may still be undiscovered, future work should also be planned to characterize yeast mutant strains carrying knock-outs for these genes, as well as engineered strains for their overexpression. Global omics approaches such as transcriptomics, proteomics, metabolomics, and fluxomics could provide valuable insights into their function.

## Supplementary Material

foaf026_Supplemental_Files
