# Supplementary material for: Molecular evolution of the members of the Snq2/Pdr18 subfamily of Pdr transporters in the Hemiascomycete yeasts
Source: FEMS Yeast Res. 2025 May 27;25:foaf026. doi: 10.1093/femsyr/foaf026 (PMC12202755; doi:10.1093/femsyr/foaf026)
Supplement: foaf026_Supplemental_Files [file foaf026_supplemental_files.zip › Figure A10_Supplementary Data.pdf]

Candida arabinofermentans NRRL YB-2248	13343	8461	21773	13344	134	caar_1_13_m01420	12612	12613	2390	21772	21771
	12855	13164	3674	13404	134	caar_1_14_n01430	21814	0	0	0	0
Dekkera bruxellensis CBS 2499	44768	3674	13404	13404	134	debr_2_5_e03380	44721	3084	518	12887	12793
Hansenula polymorpha NCYC 495	11038	14101	12855	13164	13404	hapo_1_1_a07220	1628	174	7267	3525	13263
Ogataea parapolymorpha DL-1	11038	14101	12855	13164	13404	ogpa_1_1_a01680	1628	174	7267	3525	13263
Wickerhamomyces anomalus NRRL Y-366	100365	12298	7184	14551	11038	wian_1_1_a02920	134	17246	100366	100367	1464
	12298	7184	14551	11038	134	wian_1_1_a02930	17246	100366	100367	1464	7843
	100903	100904	15275	43191	43191	wian_1_3_c04380	134	13973	7241	285	43678
	100904	15275	43191	43191	134	wian_1_3_c04390	13973	7241	285	43678	13975
	7184	12877	1581	134	134	wian_1_7_g01010	9735	16803	4116	135558	13821
Ascoidea rubescens NRRL Y17699	15481	15	11530	6222	15482	asru_1_13_m01190	153	285	1615	13404	15478
	14299	13927	15580	15581	14297	asru_1_15_o00450	14295	14296	153	12773	15582
Pichia kudriavzevii M12	0	0	0	0	0	piku_1_227_hs00100	12155	21794	13983	0	0
	485	89481	89482	11966	89483	piku_1_96_cr00180	11681	14186	14410	12155	89484
Pichia membranifaciens NRRL Y-2026	3978	15094	8204	10962	9462	pime_1_1_a12110	14955	285	12610	13807	13808
	12858	12857	3093	3093	88683	pime_1_4_d03240	14230	14231	13673	12773	153
	90253	13654	90252	12712	21590	pime_1_5_e05800	12155	21794	23024	14074	763
	6856	89640	14498	88826	22423	pime_1_1_a07690	7790	14619	89076	13795	89639
Komagataella pastoris CBS 7435	14830	14083	69749	6706	69750	kopa_1_2_b10040	8151	15268	20072	69751	69752
Komagataella pastoris DSMZ 70382	14830	14083	69749	6706	69750	kopa_2_7_g00500	8151	15268	20072	69751	69752
Komagataella pastoris GS115	14830	14083	69749	6706	69750	kopa_3_2_b02790	8151	15268	20072	69751	69752
Pachysolen tannophilus NRRL Y-2460	87378	87377	12895	13396	87376	pata_1_2_b05590	14827	8144	87375	13291	5470
