# Supplementary material for: Molecular evolution of the members of the Snq2/Pdr18 subfamily of Pdr transporters in the Hemiascomycete yeasts
Source: FEMS Yeast Res. 2025 May 27;25:foaf026. doi: 10.1093/femsyr/foaf026 (PMC12202755; doi:10.1093/femsyr/foaf026)
Supplement: foaf026_Supplemental_Files [file foaf026_supplemental_files.zip › Figure A3_Supplementary Data.pdf]

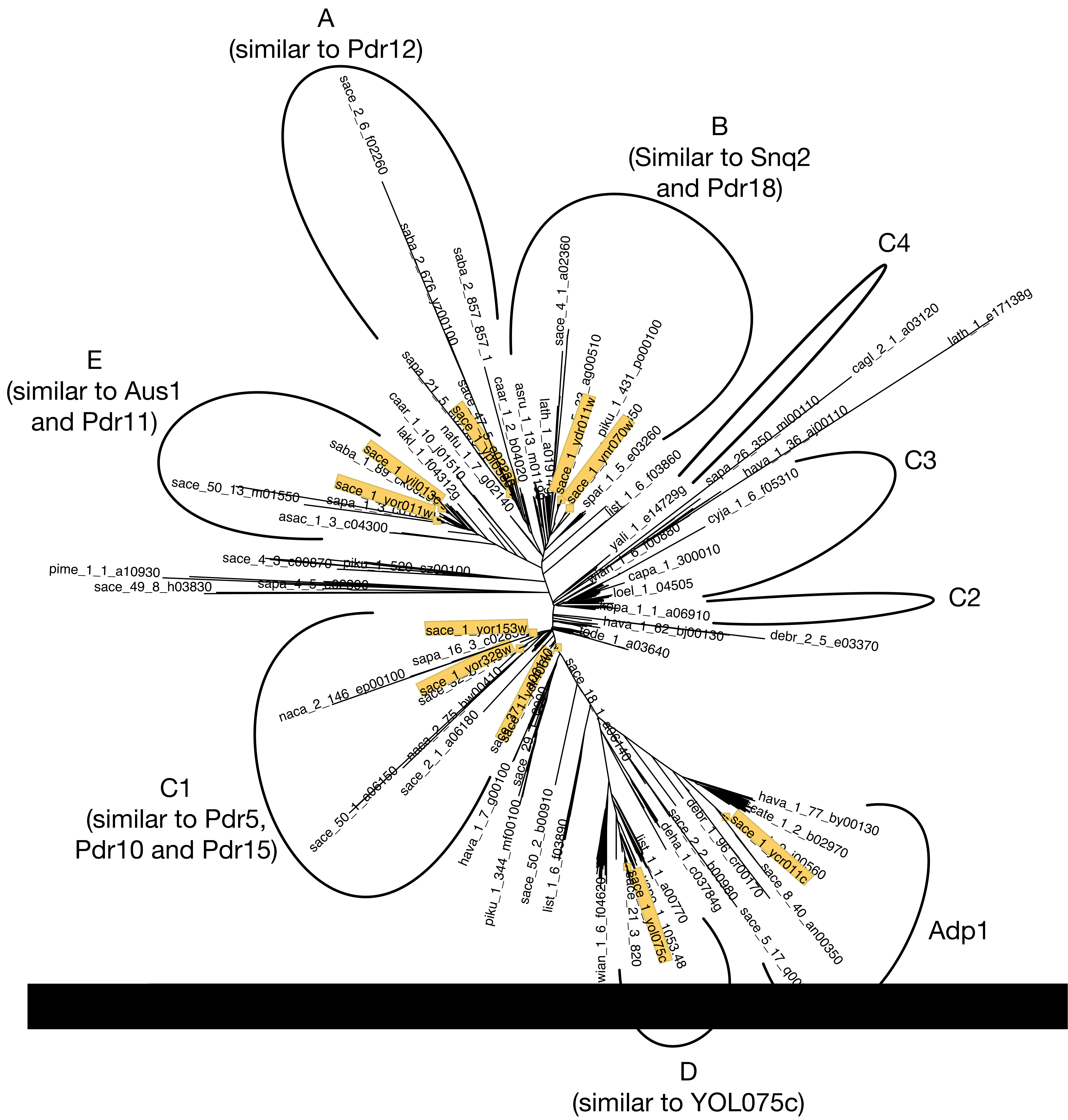
A
(similar to Pdr12)
B
(Similar to Snq2
and Pdr18)
C4
C3
C2
E
(similar to Aus1
and Pdr11)
C1
(similar to Pdr5,
Pdr10 and Pdr15)
Adp1
D
(similar to YOL075c)
sace_2_6_f02260
saba_2_676_yz00100
saba_2_857_857_1
caar_1_2_b04020
sace_4_1_a02360
piku_1_431_po00100
spar_1_5_e03260
list_1_6_f03860
sace_1_ydr011w
sace_1_ynr070w
sace_1_yil013c
sace_1_yor011w
sace_50_13_m01550
asac_1_3_c04300
laki_1_f04312g
caar_1_10_j01510
nafu_1_7_g02140
pime_1_1_a10930
sace_49_8_h03830
sace_4_3_c00870
cagl_2_1_a03120
lath_1_e17138g
sapa_26_350_ml00110
hava_1_36_aj00110
cyja_1_6_f05310
yali_1_e14729g
capa_1_300010
loel_1_04505
kopa_1_1_a06910
hava_1_62_bj00130
debr_2_5_e03370
tode_1_a03640
sace_1_yor153w
sace_1_yor328w
naca_2_146_ep00100
sace_2_1_a06180
sace_50_1_a06150
hava_1_7_g00100
piku_1_344_mf00100
sace_50_2_b00910
list_1_6_f03890
sace_18_1_a06140
hava_1_77_by00130
sace_1_ycr011c
sace_8_40_an00350
debr_1_96_cr00170
deha_1_c03784g
sace_2_2_b00980
sace_1_yol075c
sace_21_3_820
list_1_1_a00770
wian_1_6_f04620
[redacted]
