# Supplementary material for: Molecular evolution of the members of the Snq2/Pdr18 subfamily of Pdr transporters in the Hemiascomycete yeasts
Source: FEMS Yeast Res. 2025 May 27;25:foaf026. doi: 10.1093/femsyr/foaf026 (PMC12202755; doi:10.1093/femsyr/foaf026)
Supplement: foaf026_Supplemental_Files [file foaf026_supplemental_files.zip › Figure A6_Supplementary Data.pdf]

Results colour-coded for transmembrane structure

The current colourscheme of the alignment is for **transmembrane structure type**.

The transmembrane structure for each sequence is represented by a colour. If a sequence in the alignment has no colours assigned, this means that no prediction was possible for that sequence (if this was requested).
The colour assignment is:

TM SEGMENT (T) You have selected to perform transmembrane structure prediction using **TMHMM v2.0** (Krogh et. al, 2001).

```
                       .........10 .........20 .........30 .........40 .........50
(PRED) asac_1_6_f03560 ---------- ---------- -----MVEAT GLQREAEAEE HDSRT-----
(PRED) ergo_1_abr125c  ---------- MLSHHPSTCP DPQRYMIEAT GPQ-WAKTEG HDSRT-----
(PRED) ercy_1_3604     ---------- ---------- ---------- -----MGLEM RGSVS-----
(PRED) cagl_1_i04862g  ---------- ---------- ---------- ---------- ----------
(PRED) kaaf_1_c00830   ---------- ---------- ---------- ---------- ----------
(PRED) kana_1_k01350   ---------- ---------- ---------- ---------- ----------
(PRED) saar_1_2_b02590 ---------- ---------- ---------- ---------- ----------
(PRED) sace_1_ydr011w  ---------- ---------- ---------- ---------- ----------
(PRED) sace_16_1_a0238 ---------- ---------- ---------- ---------- ----------
(PRED) sace_45_1_a0242 ---------- ---------- ---------- ---------- ----------
(PRED) sace_48_1_a0238 ---------- ---------- ---------- ---------- ----------
(PRED) sace_60_4_d0244 ---------- ---------- ---------- ---------- ----------
(PRED) sace_52_1_a0240 ---------- ---------- ---------- ---------- ----------
(PRED) sace_46_1_a0240 ---------- ---------- ---------- ---------- ----------
(PRED) sace_25_1_a0240 ---------- ---------- ---------- ---------- ----------
(PRED) sace_24_1_2300  ---------- ---------- ---------- ---------- ----------
(PRED) sace_47_1_a0240 ---------- ---------- ---------- ---------- ----------
(PRED) sace_7_1_a02410 ---------- ---------- ---------- ---------- ----------
(PRED) sace_59_110_df0 ---------- ---------- ---------- ---------- ----------
(PRED) sace_56_1_a0202 ---------- ---------- ---------- ---------- ----------
(PRED) sace_40_1_a0239 ---------- ---------- ---------- ---------- ----------
(PRED) sace_15_1_a0242 ---------- ---------- ---------- ---------- ----------
(PRED) sace_37_1_a0243 ---------- ---------- ---------- ---------- ----------
(PRED) sace_9_1_a02440 ---------- ---------- ---------- ---------- ----------
(PRED) sace_22_1_2300  ---------- ---------- ---------- ---------- ----------
(PRED) sace_29_1_2290  ---------- ---------- ---------- ---------- ----------
(PRED) sace_34_1_2320  ---------- ---------- ---------- ---------- ----------
(PRED) sace_58_25_y007 ---------- ---------- ---------- ---------- ----------
(PRED) sace_23_1_2290  ---------- ---------- ---------- ---------- ----------
(PRED) sace_6_120_dp00 ---------- ---------- ---------- ---------- ----------
(PRED) sace_57_1_a0241 ---------- ---------- ---------- ---------- ----------
(PRED) sace_17_1_a0241 ---------- ---------- ---------- ---------- ----------
(PRED) sace_21_1_2310  ---------- ---------- ---------- ---------- ----------
(PRED) sace_49_1_a0246 ---------- ---------- ---------- ---------- ----------
(PRED) sace_8_2_b02430 ---------- ---------- ---------- ---------- ----------
(PRED) sace_31_1_2300  ---------- ---------- ---------- ---------- ----------
(PRED) sace_50_1_a0241 ---------- ---------- ---------- ---------- ----------
(PRED) sace_4_1_a02360 ---------- ---------- ---------- ---------- ----------
(PRED) sace_2_1_a02390 ---------- ---------- ---------- ---------- ----------
(PRED) sace_5_33_ag005 ---------- ---------- ---------- ---------- ----------
(PRED) sapa_11_1_a0247 ---------- ---------- ---------- ---------- ----------
(PRED) sapa_25_1_a0246 ---------- ---------- ---------- ---------- ----------
(PRED) sapa_4_1_a02470 ---------- ---------- ---------- ---------- ----------
(PRED) sapa_5_1_2350   ---------- ---------- ---------- ---------- ----------
(PRED) sapa_9_1_2360   ---------- ---------- ---------- ---------- ----------
(PRED) sapa_14_1_a0244 ---------- ---------- ---------- ---------- ----------
(PRED) sapa_8_1_2350   ---------- ---------- ---------- ---------- ----------
(PRED) sapa_17_1_2380  ---------- ---------- ---------- ---------- ----------
(PRED) sapa_7_1_2370   ---------- ---------- ---------- ---------- ----------
(PRED) sapa_2_1_a02460 ---------- ---------- ---------- ---------- ----------
(PRED) sapa_23_1_a0248 ---------- ---------- ---------- ---------- ----------
(PRED) sapa_3_1_a02470 ---------- ---------- ---------- ---------- ----------
(PRED) sapa_18_1_2390  ---------- ---------- ---------- ---------- ----------
(PRED) sami_1_4_244    ---------- ---------- ---------- ---------- ----------
(PRED) saku_1_4_262    ---------- ---------- ---------- ---------- ----------
(PRED) saba_1_58_bf002 ---------- ---------- ---------- ---------- ----------
(PRED) saeu_1_4_d02400 ---------- ---------- ---------- ---------- ----------
(PRED) naca_1_e01640   ---------- ---------- ---------- ---------- ----------
(PRED) nada_1_g01850   ---------- ---------- ---------- ---------- ----------
(PRED) naca_1_e01630   MTKV----AN VFCGCFCAPR RERI-PVDIR NLREEVITKH IKLPSYITLK
(PRED) nada_1_g01840   ---------- ---------- ---------- ---------- ----------
(PRED) kaaf_1_c00820   ---------- ---------- ---------- -MESE---SE ISSTPLNETQ
(PRED) teph_1_m00640   ---------- ---------- ---------- ---------- ----------
```

```
(PRED) vapo_1_1036_28  ---------- ---------- ---------- ---------- ----------
(PRED) tebl_1_i01760   MDPIIGNNSS IRGSESNIDL DEKSGPAKHT NINTTRQSTQ LSSENLVALH
(PRED) tode_1_d04040   ---------- ---------- ---------- ---------- ----------
(PRED) naca_1_e01650   ---------- ---------- ------MDES NLQS------ FSGDSNPASK
(PRED) tebl_1_g02820   ---------- ---------- ------MSWS KSRSTSDSSQ LELESYNGTG
(PRED) lakl_1_c11616g  ---------- ---------- ---------- ---------- ----------
(PRED) saar_1_8_h03780 ---------- ---------- ---------- ---------- ----------
(PRED) sace_14_7_g0015 ---------- ---------- ---------- ---------- ----------
(PRED) sace_15_7_g0387 ---------- ---------- ---------- ---------- ----------
(PRED) sace_24_8_3780  ---------- ---------- ---------- ---------- ----------
(PRED) sace_40_8_h0383 ---------- ---------- ---------- ---------- ----------
(PRED) sace_6_169_fm00 ---------- ---------- ---------- ---------- ----------
(PRED) sace_19_7_3840  ---------- ---------- ---------- ---------- ----------
(PRED) sace_32_7_3770  ---------- ---------- ---------- ---------- ----------
(PRED) sace_56_17_q011 ---------- ---------- ---------- ---------- ----------
(PRED) sace_5_78_bz001 ---------- ---------- ---------- ---------- ----------
(PRED) sace_2_8_h03860 ---------- ---------- ---------- ---------- ----------
(PRED) sace_53_29_ac00 ---------- ---------- ---------- ---------- ----------
(PRED) sace_17_7_g0393 ---------- ---------- ---------- ---------- ----------
(PRED) sace_25_7_g0388 ---------- ---------- ---------- ---------- ----------
(PRED) sace_37_7_g0385 ---------- ---------- ---------- ---------- ----------
(PRED) sace_9_7_g00180 ---------- ---------- ---------- ---------- ----------
(PRED) sace_60_6_f0335 ---------- ---------- ---------- ---------- ----------
(PRED) sace_59_336_lx0 ---------- ---------- ---------- ---------- ----------
(PRED) sace_31_7_3780  ---------- ---------- ---------- ---------- ----------
(PRED) sace_34_8_3770  ---------- ---------- ---------- ---------- ----------
(PRED) sace_58_71_bs00 ---------- ---------- ---------- ---------- ----------
(PRED) sace_7_7_g03880 ---------- ---------- ---------- ---------- ----------
(PRED) sace_35_7_3840  ---------- ---------- ---------- ---------- ----------
(PRED) sace_43_7_g0387 ---------- ---------- ---------- ---------- ----------
(PRED) sace_57_8_h0390 ---------- ---------- ---------- ---------- ----------
(PRED) sace_45_7_g0389 ---------- ---------- ---------- ---------- ----------
(PRED) sace_46_8_h0391 ---------- ---------- ---------- ---------- ----------
(PRED) sace_23_7_3860  ---------- ---------- ---------- ---------- ----------
(PRED) sace_21_7_3790  ---------- ---------- ---------- ---------- ----------
(PRED) sace_8_73_bu001 ---------- ---------- ---------- ---------- ----------
(PRED) sapa_1_8_h03820 ---------- ---------- ---------- ---------- ----------
(PRED) sapa_21_8_h0387 ---------- ---------- ---------- ---------- ----------
(PRED) sapa_20_8_h0386 ---------- ---------- ---------- ---------- ----------
(PRED) sapa_22_8_h0390 ---------- ---------- ---------- ---------- ----------
(PRED) sapa_25_8_h0387 ---------- ---------- ---------- ---------- ----------
(PRED) sapa_6_8_3750   ---------- ---------- ---------- ---------- ----------
(PRED) sapa_9_8_3720   ---------- ---------- ---------- ---------- ----------
(PRED) sapa_19_8_h0390 ---------- ---------- ---------- ---------- ----------
(PRED) sapa_24_8_h0385 ---------- ---------- ---------- ---------- ----------
(PRED) sapa_4_8_h03850 ---------- ---------- ---------- ---------- ----------
(PRED) sapa_10_8_3760  ---------- ---------- ---------- ---------- ----------
(PRED) sapa_13_8_h0382 ---------- ---------- ---------- ---------- ----------
(PRED) sapa_8_8_3750   ---------- ---------- ---------- ---------- ----------
(PRED) sapa_11_8_h0383 ---------- ---------- ---------- ---------- ----------
(PRED) sapa_5_8_3700   ---------- ---------- ---------- ---------- ----------
(PRED) sapa_16_8_h0389 ---------- ---------- ---------- ---------- ----------
(PRED) sapa_17_8_3730  ---------- ---------- ---------- ---------- ----------
(PRED) sapa_2_8_h03860 ---------- ---------- ---------- ---------- ----------
(PRED) sapa_7_8_3740   ---------- ---------- ---------- ---------- ----------
(PRED) sapa_23_8_h0385 ---------- ---------- ---------- ---------- ----------
(PRED) sapa_3_8_h03890 ---------- ---------- ---------- ---------- ----------
(PRED) sapa_18_8_3730  ---------- ---------- ---------- ---------- ----------
(PRED) sami_1_14_399   ---------- ---------- ---------- ---------- ----------
(PRED) sace_4_8_h03690 ---------- ---------- ---------- ---------- ----------
(PRED) saku_1_14_404   ---------- ---------- ---------- ---------- ----------
(PRED) sace_1_ynr070w  ---------- ---------- ---------- ---------- ----------
(PRED) sace_49_8_h0383 ---------- ---------- ---------- ---------- ----------
(PRED) saeu_1_2_b00130 ---------- ---------- ---------- ---------- ----------
(PRED) sauv_1_7_3      ---------- ---------- ---------- ---------- ----------
(PRED) sami_1_17_26    ---------- ---------- ---------- ---------- ----------
(PRED) zyba_1_02055_AN ---------- ---------M DPADNSMEER HSAESTGTTP AVSTPP----
(PRED) zyba_1_07912    ---------- ---------- ------MEER RSAESTETIP AVSTPP----
(PRED) zyba_2_2_b00600 ---------- ---------M DPVDNFMEER RSAESTETTP AVSTPP----
(PRED) zyba_3_3_c03460 ---------- ---------M DPVDNLMEER RSAESTETIP AVSTPP----
(PRED) zyba_1_04634    ---------- ---------M NSLHNQSIEK NSLDEAD--- --STPS----
(PRED) zyba_1_06675    ---------- ---------M NSLHNQPVEK NSLGEAD--- --STPS----
(PRED) zyba_3_2_b02230 ---------- ---------M NSLHNQPVEK NSLGEAD--- --STPS----
(PRED) zyba_2_1_a00860 ---------- ---------M NSLHNQPVEK NSLGEAD--- --STPS----
(PRED) zyro_1_a04114g  ---------- ---------M KK--GNVLET GSSSPIDS-- --NSPI----
```

```
(PRED) zyro_1_b14762g ---------- ---------M EK--GM---- -ASDEADS-- --ATPI----
(PRED) zyba_2_14_n0149 ---------- ---------- ------MLQK S-VTGA---- ----PS----
(PRED) zyba_2_33_ag001 ---------- ---------- ------MLQE AGATEN---- ----TS----
(PRED) lath_1_a01914g ---------- ---------- ---------- ---------- ----------
(PRED) lawa_1_23_5161 ---------- ---------- ---------- ---------- ----------
(PRED) klae_1_14_n0012 ---------- ---------- ---------- ---------- ----------
(PRED) klla_1_d03432g ---------- ---------- ---------- ---------- ----------
(PRED) klma_1_1_a01880 ---------- ---------- ---------- ---------- ----------
(PRED) klwi_1_33_ag001 ---------- ---------- ---------- ---------- ----------
(PRED) teph_1_a04220 ---------- ---------- ---------- ---------- ----------
(PRED) vapo_1_1037_47 ---------- ---------- ---------- ---------- ----------
(PRED) pata_1_2_b05590 ------MSAP MEYNQGSGET SESNGKSSDT YSEKDVDSFR GPDTLGGGGN
(PRED) wian_1_3_c04380 ---------- ---------M SVIRNRS--- ------SSFE RQSS------
(PRED) wian_1_3_c04390 ---------- ---------- --MSGASIT- --ADQVEGFH PTAS------
(PRED) wian_1_7_g01010 ---------- ---------M SIINNNSTD- --EQTVQDFH PP--------
(PRED) bain_1_1_a00100 ---------- ---------- ---------- ---------- ----------
(PRED) bain_1_17_q0038 ---------- ---------- ---------- ---------- ----------
(PRED) bain_1_8_h00410 ---------- ---------- ---------- ---------- ----------
(PRED) caal_1_19_5759 ---------- ---------- ---------- ---------- ----------
(PRED) caal_11_25_y002 ---------- ---------- ---------- ---------- ----------
(PRED) caal_4_4_d03320 ---------- ---------- ---------- ---------- ----------
(PRED) caal_12_26_z005 ---------- ---------- ---------- ---------- ----------
(PRED) caal_5_30_ad005 ---------- ---------- ---------- ---------- ----------
(PRED) caal_8_3_c03320 ---------- ---------- ---------- ---------- ----------
(PRED) caal_6_4_d03280 ---------- ---------- ---------- ---------- ----------
(PRED) caal_10_3_c0334 ---------- ---------- ---------- ---------- ----------
(PRED) caal_3_29_ac005 ---------- ---------- ---------- ---------- ----------
(PRED) caal_2_04989 ---------- ---------- ---------- ---------- ----------
(PRED) cadu_1_64350 ---------- ---------- ---------- ---------- ----------
(PRED) caor_1_h02090 ---------- ---------- ---------- ---------- ----------
(PRED) capa_1_600750 ---------- ---------- ---------- ---------- ----------
(PRED) loel_1_04930 ---------- ---------- ---------- ---------- ----------
(PRED) spar_1_5_e03260 ---------- ---------- ---------- ---------- ----------
(PRED) sppa_1_7_g03160 ---------- ---------- ---------- ---------- ----------
(PRED) catr_1_01205 ---------- ---------- ---------- ---------- ----------
(PRED) catr_1_05498 ---------- ---------- ---------- ---------- ----------
(PRED) catr_1_05971 ---------- ---------- ---------- ---------- ----------
(PRED) deha_1_a03696g ---------- ---------- ---------- ---------- ----------
(PRED) deha_2_5_e00720 ---------- ---------- ---------- ---------- ----------
(PRED) scst_1_3_c02890 ---------- ---------- ---------- ---------- ----------
(PRED) mebi_1_8_h00300 ---------- ---------- ---------- ---------- ----------
(PRED) lakl_1_h21010g ---------- ---------- ---------- ---------- ----------
(PRED) caar_1_13_m0142 ---------- ---------- ---------- ---------- ----------
(PRED) caar_1_14_n0143 ---------- ---------- ---------- ---------- ----------
(PRED) hapo_1_1_a07220 ---------- ---------- ---------- ---------- ----MS----
(PRED) ogpa_1_1_a01680 ---------- ---------- ---------- ---------- ----MS----
(PRED) piku_1_96_cr001 ---------- ---------M STSS--AS-- ---SNGK--- --YETF----
(PRED) pime_1_4_d03240 ---------- ---------M SDSSNVASPA EKESNSDRSS LVYEVD----
(PRED) pime_1_1_a12110 ---------- ---------M SSFSGAES-- --VPLGK--- ---EKD----
(PRED) piku_1_227_hs00 ---------- ---------- ---------- ---------- ----------
(PRED) pime_1_5_e05800 ---------- ---------- ---------- ---------- -MSNPV----
(PRED) pime_1_1_a07690 ---------- ---------- ---------- ---------- -MTDSI----
(PRED) debr_2_5_e03380 ---------- ---------M SSSSQSNEKP HRASTSS--- ---ESI----
(PRED) kopa_1_2_b10040 ---------- ---------M ERDTHEADPA ---------- ---QPV----
(PRED) kopa_2_7_g00500 ---------- ---------M ERDTHEADRA ---------- ---EHV----
(PRED) asru_1_13_m0119 ---------- ---------- ---------- ---------- ----------
(PRED) asru_1_15_o0045 ---------- ---------- ---------- ---------- ----------
(PRED) wian_1_1_a02920 ---------- ---------- ---------- ---------- ----------
(PRED) wian_1_1_a02930 ---------- ---------- ---------- ---------- ----------

                .........60 .........70 .........80 .........90 .........100
(PRED) asac_1_6_f03560 ---------- ---------- ---------- ---------- ---S-----L
(PRED) ergo_1_abr125c ---------- ---------- ---------- ---------- ---S-----L
(PRED) ercy_1_3604 ---------- ---------- ---------- ---------- ---S-----M
(PRED) cagl_1_i04862g ---------- ---------- --------M- ---------- -SSS-----S
(PRED) kaaf_1_c00830 -----MSSSI ---------- -SE--DKKA- ---------- -PYE-----S
(PRED) kana_1_k01350 ------MNSI ----Y----- DTN--STAS- ---------- -SAS-----S
(PRED) saar_1_2_b02590 ------MNSV ----ENM-Q- ASL--SDAI- ---------- -DRS-----S
(PRED) sace_1_ydr011w ------MSNI ----KST-Q- DSS--HNAV- ---------- -ARS-----S
(PRED) sace_16_1_a0238 ------MSNI ----KST-Q- DSS--HNAV- ---------- -ARS-----S
(PRED) sace_45_1_a0242 ------MSNI ----KST-Q- DSS--HNAV- ---------- -ARS-----S
(PRED) sace_48_1_a0238 ------MSNI ----KST-Q- DSS--HNAV- ---------- -ARS-----S
(PRED) sace_60_4_d0244 ------MSNI ----KST-Q- DSS--HNAV- ---------- -ARS-----S
(PRED) sace_52_1_a0240 ------MSNI ----KST-Q- DSS--HNAV- ---------- -ARS-----S
(PRED) sace_46_1_a0240 ------MSNI ----KST-Q- DSS--HNAV- ---------- -ARS-----S
```

(PRED)	sace_25_1_a0240	------MSNI	----KST-Q-	DSS--HNAV-	----------	-ARS-----S
(PRED)	sace_24_1_2300	------MSNI	----KST-Q-	DSS--HNAV-	----------	-ARS-----S
(PRED)	sace_47_1_a0240	------MSNI	----KST-Q-	DSS--HNAV-	----------	-ARS-----S
(PRED)	sace_7_1_a02410	------MSNI	----KST-Q-	DSS--HNAV-	----------	-ARS-----S
(PRED)	sace_59_110_df0	------MSNI	----KST-Q-	DSS--HNAV-	----------	-ARS-----S
(PRED)	sace_56_1_a0202	------MSNI	----KST-Q-	DSS--HNAV-	----------	-ARS-----S
(PRED)	sace_40_1_a0239	------MSNI	----KST-Q-	DSS--HNAV-	----------	-ARS-----S
(PRED)	sace_15_1_a0242	------MSNI	----KST-Q-	DSS--HNAV-	----------	-ARS-----S
(PRED)	sace_37_1_a0243	------MSNI	----KST-Q-	DSS--HNAV-	----------	-ARS-----S
(PRED)	sace_9_1_a02440	------MSNI	----KST-Q-	DSS--HNAV-	----------	-ARS-----S
(PRED)	sace_22_1_2300	------MSNI	----KST-Q-	DSS--HNAV-	----------	-ARS-----S
(PRED)	sace_29_1_2290	------MSNI	----KST-Q-	DSS--HNAV-	----------	-ARS-----S
(PRED)	sace_34_1_2320	------MSNI	----KST-Q-	DSS--HNAV-	----------	-ARS-----S
(PRED)	sace_58_25_y007	------MSNI	----KST-Q-	DSS--HNAV-	----------	-ARS-----S
(PRED)	sace_23_1_2290	------MSNI	----KST-Q-	DSS--HNAV-	----------	-ARS-----S
(PRED)	sace_6_120_dp00	------MSNI	----KST-Q-	DSS--HNAV-	----------	-ARS-----S
(PRED)	sace_57_1_a0241	------MSNI	----KST-Q-	DSS--HNAV-	----------	-ARS-----S
(PRED)	sace_17_1_a0241	------MSNI	----KST-Q-	DSS--HNAV-	----------	-ARS-----S
(PRED)	sace_21_1_2310	------MSNI	----KST-Q-	DSS--HNAV-	----------	-ARS-----S
(PRED)	sace_49_1_a0246	------MSNI	----KST-Q-	DSS--HNAV-	----------	-ARS-----S
(PRED)	sace_8_2_b02430	------MSNI	----KST-Q-	DSS--HNAV-	----------	-ARS-----S
(PRED)	sace_31_1_2300	------MSNI	----KST-Q-	DSS--HNAV-	----------	-ARS-----S
(PRED)	sace_50_1_a0241	------MSNI	----KST-Q-	DSS--HXAV-	----------	-ARS-----S
(PRED)	sace_4_1_a02360	------MSNI	----KST-Q-	DSS--HNAV-	----------	-ARS-----S
(PRED)	sace_2_1_a02390	------MSNI	----KST-Q-	DSS--HNAV-	----------	-ARS-----S
(PRED)	sace_5_33_ag005	------MSNI	----KST-Q-	DSS--HNAV-	----------	-ARS-----S
(PRED)	sapa_11_1_a0247	------MSNI	----TNT-Q-	DSS--NNTV-	----------	-VRS-----S
(PRED)	sapa_25_1_a0246	------MSNI	----TNT-Q-	DSS--NNTV-	----------	-VRS-----S
(PRED)	sapa_4_1_a02470	------MSNI	----TNT-Q-	DSS--NNTV-	----------	-VRS-----S
(PRED)	sapa_5_1_2350	------MSNI	----TNT-Q-	DSS--NNTV-	----------	-VRS-----S
(PRED)	sapa_9_1_2360	------MSNI	----TNT-Q-	DSS--NNTV-	----------	-VRS-----S
(PRED)	sapa_14_1_a0244	------MSNI	----TNT-Q-	DSS--NNTV-	----------	-VKS-----S
(PRED)	sapa_8_1_2350	------MSNI	----TNT-Q-	DSS--NNTV-	----------	-VKS-----S
(PRED)	sapa_17_1_2380	------MSNI	----TNT-Q-	DSS--NNTV-	----------	-VRS-----S
(PRED)	sapa_7_1_2370	------MSNI	----TNT-Q-	DSS--NNTV-	----------	-VRS-----S
(PRED)	sapa_2_1_a02460	------MSNI	----TNT-Q-	DSS--NNTV-	----------	-VRS-----S
(PRED)	sapa_23_1_a0248	------MSNI	----TNT-Q-	DSS--NNTV-	----------	-VRS-----S
(PRED)	sapa_3_1_a02470	------MSNI	----TNT-Q-	DSS--NNTV-	----------	-VRS-----S
(PRED)	sapa_18_1_2390	------MSNI	----TNT-Q-	DSS--NNTV-	----------	-VRS-----S
(PRED)	sami_1_4_244	------MSSI	----KNT-Q-	NSS--GNIV-	----------	-ARS-----S
(PRED)	saku_1_4_262	------MSSI	----KNT-R-	DGS--NNVA-	----------	-TRS-----S
(PRED)	saba_1_58_bf002	------MSSI	----KNA-HG	GSK--DSPV-	----------	-TRS-----S
(PRED)	saeu_1_4_d02400	------MSNT	----KNA-HG	GIS--ASPV-	----------	-TRS-----S
(PRED)	naca_1_e01640	------MSN-	----KST-H-	RLT--VASD-	----------	-TQS-----L
(PRED)	nada_1_g01850	------MSG-	----KNN-DT	TVG--AAHQ-	----------	-VDS-----S
(PRED)	naca_1_e01630	-----YTHFL	----TQTFQK	LQL--NERI-	----------	-LMD-----R
(PRED)	nada_1_g01840	----------	----------	----------	----------	--MS-----S
(PRED)	kaaf_1_c00820	-----CMSEI	----YQ----	HKD--SNVE-	----------	-GKP-----D
(PRED)	teph_1_m00640	------MSSF	----VS----	--------S-	----------	-VHS-----N
(PRED)	vapo_1_1036_28	------MSQD	----TD----	-------FS-	----------	-VSS-----D
(PRED)	tebl_1_i01760	EGEFFSMDNI	REQPKDTHSS	SSTDDENSIK	EFSPSHREPP	RERS-----M
(PRED)	tode_1_d04040	----------	----------	----MSSAV-	----------	--RD-----E
(PRED)	naca_1_e01650	----------	----------	----------	----------	-GDD-----G
(PRED)	tebl_1_g02820	-------SN-	----------	-----SESV-	----------	---N-----L
(PRED)	lakl_1_c11616g	----------	----------	----------	----------	---M-----S
(PRED)	saar_1_8_h03780	----------	----------	----------	----------	----------
(PRED)	sace_14_7_g0015	----------	----------	----------	----------	----------
(PRED)	sace_15_7_g0387	----------	----------	----------	----------	----------
(PRED)	sace_24_8_3780	----------	----------	----------	----------	----------
(PRED)	sace_40_8_h0383	----------	----------	----------	----------	----------
(PRED)	sace_6_169_fm00	----------	----------	----------	----------	----------
(PRED)	sace_19_7_3840	----------	----------	----------	----------	----------
(PRED)	sace_32_7_3770	----------	----------	----------	----------	----------
(PRED)	sace_56_17_q011	----------	----------	----------	----------	----------
(PRED)	sace_5_78_bz001	----------	----------	----------	----------	----------
(PRED)	sace_2_8_h03860	----------	----------	----------	----------	----------
(PRED)	sace_53_29_ac00	----------	----------	----------	----------	----------
(PRED)	sace_17_7_g0393	----------	----------	----------	----------	----------
(PRED)	sace_25_7_g0388	----------	----------	----------	----------	----------
(PRED)	sace_37_7_g0385	----------	----------	----------	----------	----------
(PRED)	sace_9_7_g00180	----------	----------	----------	----------	----------
(PRED)	sace_60_6_f0335	----------	----------	----------	----------	----------
(PRED)	sace_59_336_lx0	----------	----------	----------	----------	----------
(PRED)	sace_31_7_3780	----------	----------	----------	----------	----------
(PRED)	sace_34_8_3770	----------	----------	----------	----------	----------

```
(PRED) sace_58_71_bs00 ----------  ----------  ----------  ----------  ----------
(PRED) sace_7_7_g03880 ----------  ----------  ----------  ----------  ----------
(PRED) sace_35_7_3840  ----------  ----------  ----------  ----------  ----------
(PRED) sace_43_7_g0387 ----------  ----------  ----------  ----------  ----------
(PRED) sace_57_8_h0390 ----------  ----------  ----------  ----------  ----------
(PRED) sace_45_7_g0389 ----------  ----------  ----------  ----------  ----------
(PRED) sace_46_8_h0391 ----------  ----------  ----------  ----------  ----------
(PRED) sace_23_7_3860  ----------  ----------  ----------  ----------  ----------
(PRED) sace_21_7_3790  ----------  ----------  ----------  ----------  ----------
(PRED) sace_8_73_bu001 ----------  ----------  ----------  ----------  ----------
(PRED) sapa_1_8_h03820 ----------  ----------  ----------  ----------  ----------
(PRED) sapa_21_8_h0387 ----------  ----------  ----------  ----------  ----------
(PRED) sapa_20_8_h0386 ----------  ----------  ----------  ----------  ----------
(PRED) sapa_22_8_h0390 ----------  ----------  ----------  ----------  ----------
(PRED) sapa_25_8_h0387 ----------  ----------  ----------  ----------  ----------
(PRED) sapa_6_8_3750   ----------  ----------  ----------  ----------  ----------
(PRED) sapa_9_8_3720   ----------  ----------  ----------  ----------  ----------
(PRED) sapa_19_8_h0390 ----------  ----------  ----------  ----------  ----------
(PRED) sapa_24_8_h0385 ----------  ----------  ----------  ----------  ----------
(PRED) sapa_4_8_h03850 ----------  ----------  ----------  ----------  ----------
(PRED) sapa_10_8_3760  ----------  ----------  ----------  ----------  ----------
(PRED) sapa_13_8_h0382 ----------  ----------  ----------  ----------  ----------
(PRED) sapa_8_8_3750   ----------  ----------  ----------  ----------  ----------
(PRED) sapa_11_8_h0383 ----------  ----------  ----------  ----------  ----------
(PRED) sapa_5_8_3700   ----------  ----------  ----------  ----------  ----------
(PRED) sapa_16_8_h0389 ----------  ----------  ----------  ----------  ----------
(PRED) sapa_17_8_3730  ----------  ----------  ----------  ----------  ----------
(PRED) sapa_2_8_h03860 ----------  ----------  ----------  ----------  ----------
(PRED) sapa_7_8_3740   ----------  ----------  ----------  ----------  ----------
(PRED) sapa_23_8_h0385 ----------  ----------  ----------  ----------  ----------
(PRED) sapa_3_8_h03890 ----------  ----------  ----------  ----------  ----------
(PRED) sapa_18_8_3730  ----------  ----------  ----------  ----------  ----------
(PRED) sami_1_14_399   ----------  ----------  ----------  ----------  ----------
(PRED) sace_4_8_h03690 ----------  ----------  ----------  ----------  ----------
(PRED) saku_1_14_404   ----------  ----------  ----------  ----------  ----------
(PRED) sace_1_ynr070w  ----------  ----------  ----------  ----------  ----------
(PRED) sace_49_8_h0383 ----------  ----------  ----------  ----------  ----------
(PRED) saeu_1_2_b00130 ----------  ----------  ----------  ----------  ----------
(PRED) sauv_1_7_3      ----------  ----------  ----------  ----------  ----------
(PRED) sami_1_17_26    ----------  ---------M  PSSTGSGRI-  ----------  --SS-----A
(PRED) zyba_1_02055_AN ----------  ----------  ----------  ----------  -VY------G
(PRED) zyba_1_07912    ----------  ----------  ----------  ----------  -VY------G
(PRED) zyba_2_2_b00600 ----------  ----------  ----------  ----------  -VY------G
(PRED) zyba_3_3_c03460 ----------  ----------  ----------  ----------  -VY------G
(PRED) zyba_1_04634    ----------  ----------  ----------  ----------  -FYN-----G
(PRED) zyba_1_06675    ----------  ----------  ----------  ----------  -FYD-----G
(PRED) zyba_3_2_b02230 ----------  ----------  ----------  ----------  -FYD-----G
(PRED) zyba_2_1_a00860 ----------  ----------  ----------  ----------  -FYD-----G
(PRED) zyro_1_a04114g  ----------  ----------  ----------  ----------  -SYD-----G
(PRED) zyro_1_b14762g  ----------  ----------  ----------  ----------  -SYE-----D
(PRED) zyba_2_14_n0149 ----------  ----------  ----------  ----------  -VHS-----G
(PRED) zyba_2_33_ag001 ----------  ----------  ----------  ----------  -VHS-----R
(PRED) lath_1_a01914g  ----------  ---MSADVQA  KSGAEPRS--  ----------  -GAA-----S
(PRED) lawa_1_23_5161  ----------  ---MSGEAQL  NS-----S--  ----------  -RRS-----F
(PRED) klae_1_14_n0012 ----------  ----------  ----------  ----------  ----------
(PRED) klla_1_d03432g  ----------  ----------  ----------  ----------  -MEG-----S
(PRED) klma_1_1_a01880 ----------  ----------  ----------  ----------  ----------
(PRED) klwi_1_33_ag001 ----------  ----------  ----------  ----------  ----------
(PRED) teph_1_a04220   ----------  ----------  ----------  ----------  -MKE-----D
(PRED) vapo_1_1037_47  ----------  ----------  ----------  ----------  -MAD-----N
(PRED) pata_1_2_b05590 GGGG------  ----------  ----------  ----------  -HGG-----F
(PRED) wian_1_3_c04380 ----------  ----------  ----------  ----------  -DSS-----D
(PRED) wian_1_3_c04390 ----------  ----------  ----------  ----------  -QQN-----L
(PRED) wian_1_7_g01010 ----------  ----------  ----------  ----------  ----------
(PRED) bain_1_1_a00100 ----------  ----------  ----------  ----------  -MSSL----N
(PRED) bain_1_17_q0038 ----------  ----------  ----------  ----------  -MSSP----N
(PRED) bain_1_8_h00410 ----------  ----------  ----------  ----------  -MNSD----S
(PRED) caal_1_19_5759  ----------  ----------  ----------  ----------  -MSSE----D
(PRED) caal_11_25_y002 ----------  ----------  ----------  ----------  -MSSE----D
(PRED) caal_4_4_d03320 ----------  ----------  ----------  ----------  -MSSE----D
(PRED) caal_12_26_z005 ----------  ----------  ----------  ----------  -MSSE----D
(PRED) caal_5_30_ad005 ----------  ----------  ----------  ----------  -MSSE----D
(PRED) caal_8_3_c03320 ----------  ----------  ----------  ----------  -MSSE----D
(PRED) caal_6_4_d03280 ----------  ----------  ----------  ----------  -MSSE----D
(PRED) caal_10_3_c0334 ----------  ----------  ----------  ----------  -MSSE----D
```

(PRED)	caal_3_29_ac005	----------	----------	----------	----------	-MSSE----D
(PRED)	caal_2_04989	----------	----------	----------	----------	-MSSE----D
(PRED)	cadu_1_64350	----------	----------	----------	----------	-MSSE----D
(PRED)	caor_1_h02090	----------	----------	----------	----------	-MDSN----S
(PRED)	capa_1_600750	----------	----------	----------	----------	-MSSN----S
(PRED)	loel_1_04930	----------	----------	----------	----------	-MIVD----E
(PRED)	spar_1_5_e03260	----------	----------	----------	----------	-MSSSSSRLS
(PRED)	sppa_1_7_g03160	----------	----------	----------	----------	-MSSSSSRLS
(PRED)	catr_1_01205	----------	----------	----------	----------	--------MX
(PRED)	catr_1_05498	----------	----------	----------	----------	-MVQDDQS--
(PRED)	catr_1_05971	----------	----------	----------	----------	-MPREEDSGE
(PRED)	deha_1_a03696g	----------	----------	----------	----------	-MGVD----S
(PRED)	deha_2_5_e00720	----------	----------	----------	----------	-MGIN----S
(PRED)	scst_1_3_c02890	----------	----------	----------	----------	-MSSKEEA-S
(PRED)	mebi_1_8_h00300	----------	----------	----------	----------	----------
(PRED)	lakl_1_h21010g	----------	----------	----------	----------	----------
(PRED)	caar_1_13_m0142	----------	----------	----------	----------	----------
(PRED)	caar_1_14_n0143	----------	----------	----------	----------	----------
(PRED)	hapo_1_1_a07220	----------	----------	----------	----------	---------V
(PRED)	ogpa_1_1_a01680	----------	----------	----------	----------	---------A
(PRED)	piku_1_96_cr001	----------	----------	----------	----------	---------S
(PRED)	pime_1_4_d03240	----------	----------	----------	----------	---------S
(PRED)	pime_1_1_a12110	----------	----------	----------	----------	---------N
(PRED)	piku_1_227_hs00	----------	----------	----------	----------	----------
(PRED)	pime_1_5_e05800	----------	----------	----------	----------	---------V
(PRED)	pime_1_1_a07690	----------	----------	----------	----------	---------E
(PRED)	debr_2_5_e03380	----------	----------	----------	----------	---------P
(PRED)	kopa_1_2_b10040	----------	----------	----------	----------	---------L
(PRED)	kopa_2_7_g00500	----------	----------	----------	----------	---------L
(PRED)	asru_1_13_m0119	----------	----------	----------	----------	----------
(PRED)	asru_1_15_o0045	----------	----------	----------	----------	---------M
(PRED)	wian_1_1_a02920	----------	----------	----------	----------	----------
(PRED)	wian_1_1_a02930	----------	----------	----------	----------	----------

		110	120	130	140	150
(PRED)	asac_1_6_f03560	SGSFAPEMVA	YG--------	------SDAG	VEERERLSRV	MTLQS-----
(PRED)	ergo_1_abr125c	SGSSAPELVA	YG--------	------SEAG	AEERERLSRV	MTLQS-----
(PRED)	ercy_1_3604	SSKEMMDRVE	SE--------	------PSRK	RIEREDLARV	LTRGS-----
(PRED)	cagl_1_i04862g	EISVAGSDVS	FEGRLTQHGR	--------HE	ETPADQLTKI	LSGRSHEDAD
(PRED)	kaaf_1_c00830	VASSASSEES	FR--GQYDDQ	N-----KRNS	EFNAETLSRT	LSRLSQ----
(PRED)	kana_1_k01350	EVSFHGGSEH	LKHREKKHRE	------ISED	DTPADRLTKM	LTSTS-----
(PRED)	saar_1_2_b02590	SASFAASEKS	FP--GAAHNK	------DGES	DTPADRLTKM	LTGPKV----
(PRED)	sace_1_ydr011w	SASFAASEES	FT--GITHDK	------DEQS	DTPADKLTKM	LTGPAR----
(PRED)	sace_16_1_a0238	SASFAASEES	FT--GITHDK	------DEQS	DTPADKLTKM	LTGPAR----
(PRED)	sace_45_1_a0242	SASFAASEES	FT--GITHDK	------DEQS	DTPADKLTKM	LTGPAR----
(PRED)	sace_48_1_a0238	SASFAASEES	FT--GITHDK	------DEQS	DTPADKLTKM	LTGPAR----
(PRED)	sace_60_4_d0244	SASFAASEES	FT--GITHDK	------DEQS	DTPADKLTKM	LTGPAR----
(PRED)	sace_52_1_a0240	SASFAASEES	FT--GITHDK	------DEQS	DTPADKLTKM	LTGPAR----
(PRED)	sace_46_1_a0240	SASFAASEES	FT--GITHDK	------DEQS	DTPADKLTKM	LTGPAR----
(PRED)	sace_25_1_a0240	SASFAASEES	FT--GITHDK	------DEQS	DTPADKLTKM	LTGPAR----
(PRED)	sace_24_1_2300	SASFAASEES	FT--GITHDK	------DEQS	DTPADKLTKM	LTGPAR----
(PRED)	sace_47_1_a0240	SASFAASEES	FT--GITHDK	------DEQS	DTPADKLTKM	LTGPAR----
(PRED)	sace_7_1_a02410	SASFAASEES	FT--GITHDK	------DEQS	DTPADKLTKM	LTGPAR----
(PRED)	sace_59_110_df0	SASFAASEES	FT--GITHDK	------DEQS	DTPADKLTKM	LTGPAR----
(PRED)	sace_56_1_a0202	SASFAASEES	FT--GITHDK	------DEQS	DTPADKLTKM	LTGPAR----
(PRED)	sace_40_1_a0239	SASFAASEES	FT--GITHDK	------DEQS	DTPADKLTKM	LTGPAR----
(PRED)	sace_15_1_a0242	SASFAASEES	FT--GITHDK	------DEQS	DTPADKLTKM	LTGPAR----
(PRED)	sace_37_1_a0243	SASFAASEES	FT--GITHDK	------DEQS	DTPADKLTKM	LTGPAR----
(PRED)	sace_9_1_a02440	SASFAASEES	FT--GITHDK	------DEQS	DTPADKLTKM	LTGPAR----
(PRED)	sace_22_1_2300	SASFAASEES	FT--GITHDK	------DEQS	DTPADKLTKM	LTGPAR----
(PRED)	sace_29_1_2290	SASFAASEES	FT--GITHDK	------DEQS	DTPADKLTKM	LTGPAR----
(PRED)	sace_34_1_2320	SASFAASEES	FT--GITHDK	------DEQS	DTPADKLTKM	LTGPAR----
(PRED)	sace_58_25_y007	SASFAASEES	FT--GITHDK	------DEQS	DTPADKLTKM	LTGPAR----
(PRED)	sace_23_1_2290	SASFAASEES	FT--GITHDK	------DEQS	DTPADKLTKM	LTGPAR----
(PRED)	sace_6_120_dp00	SASFAASEES	FT--GITHDK	------DEQS	DTPADKLTKM	LTGPAR----
(PRED)	sace_57_1_a0241	SASFAASEES	FT--GITHDK	------DEQS	DTPADKLTKM	LTGPAR----
(PRED)	sace_17_1_a0241	SASFAASEES	FT--GITHDK	------DEQS	DTPADKLTKM	LTGPAR----
(PRED)	sace_21_1_2310	SASFAASEES	FT--GITHDK	------DEQS	DTPADKLTKM	LTGRAR----
(PRED)	sace_49_1_a0246	SASFAASEES	FT--GITHDK	------DEQS	DTPADKLTKM	LTGPAR----
(PRED)	sace_8_2_b02430	SASFAASEES	FT--GITHDK	------DEQS	DTPADKLTKM	LTGPAR----
(PRED)	sace_31_1_2300	SASFAASEES	FT--GITHDK	------DEQS	DTPVDKLTKM	LTGPAR----
(PRED)	sace_50_1_a0241	SASFAASEES	FT--GITHDK	------DEQS	DTPADKLTKM	LTGPAR----
(PRED)	sace_4_1_a02360	SASFAASEES	FT--GITHDK	------DEQS	NTPADKLTKM	LTEPAR----
(PRED)	sace_2_1_a02390	SASFAASEES	FT--GITHDK	------DEQS	DTPADKLTKM	LTGPAR----
(PRED)	sace_5_33_ag005	SASFAASEES	FT--GITHDK	------DEQS	XTPADKLTKM	LTXPAR----

(PRED)	sapa_11_1_a0247	SASFAASEES	FT--GISHEK	------DKHR	DTPADRLTKM	LTGPAK----
(PRED)	sapa_25_1_a0246	SASFAASEES	FT--GISHEK	------DKHR	DTPADRLTKM	LTGPAK----
(PRED)	sapa_4_1_a02470	SASFAASEES	FT--GISHEK	------DKHR	DTPADRLTKM	LTGPAK----
(PRED)	sapa_5_1_2350	SASFAASEES	FT--GISHEK	------DKHR	DTPADRLTKM	LTGPAK----
(PRED)	sapa_9_1_2360	SASFAASEES	FT--GISHEK	------DKHR	DTPADRLTKM	LTGPAK----
(PRED)	sapa_14_1_a0244	SASFAASEES	FT--GISHEK	------DKHR	DTPADRLTKM	LTGPAK----
(PRED)	sapa_8_1_2350	SASFAASEES	FT--GISHEK	------DKHR	DTPADRLTKM	LTGPAK----
(PRED)	sapa_17_1_2380	SASFAASEES	FT--GISHDK	------DKHR	DTPADRLTKM	LTGPAK----
(PRED)	sapa_7_1_2370	SASFAASEES	FT--GISHDK	------DKHR	DTPADRLTKM	LTGPAK----
(PRED)	sapa_2_1_a02460	SASFAASEES	FT--GISHDK	------DKHR	DTPADRLTKM	LTGPAK----
(PRED)	sapa_23_1_a0248	SASFAASEES	FT--GISHDK	------DKHR	DTPADRLTKM	LTGPAK----
(PRED)	sapa_3_1_a02470	SASFAASEES	FT--GISHDK	------DKHR	DTPADRLTKM	LTGPAK----
(PRED)	sapa_18_1_2390	SASFAASEES	FT--GISHDK	------DKHR	DTPADRLTKM	LTGPAK----
(PRED)	sami_1_4_244	SASIAASDES	FS--GVAHDK	------DKQN	DTPADRLTKM	LTGPVR----
(PRED)	saku_1_4_262	SASYAASEES	FS--GAAHDK	------EKQN	DTPADKLTRM	LTGPAR----
(PRED)	saba_1_58_bf002	SASYAASEVS	FS--GTAHGK	------DEQN	DTLAHRHSKT	LTGQAM----
(PRED)	saeu_1_4_d02400	SASYAASEVS	FS--GSTHNN	------NEQN	-----KDSKT	LSQHAR----
(PRED)	naca_1_e01640	SDSVANSELS	FK--GEPQHM	KNGGTHNDET	GTNGDKLTRM	LTQNSI----
(PRED)	nada_1_g01850	SASIANTELS	FQ--GIRHHS	ASHDNFTEGQ	ESAGDKLSRI	FTQGS-----
(PRED)	naca_1_e01630	MDSSIQSAST	SGKIVP--ES	TPPEEAVSDA	CSTASSVGEI	AVGTTT----
(PRED)	nada_1_g01840	AGSSSSSPNV	GKEYHSDNQN	TYNMDTLAET	HTNNTKVDQP	PIKTTT----
(PRED)	kaaf_1_c00820	GLRPDTSEDK	FT--GML---	------TSSS	DERV-KENKH	LT--------
(PRED)	teph_1_m00640	SASVADAEES	FK--GNNSDD	------VLAA	RTSGSFIRRT	LS--------
(PRED)	vapo_1_1036_28	SQSIASKDQS	FS--GADIAN	------PHNR	NGDNNSSLHI	ST--------
(PRED)	tebl_1_i01760	SRDFTSIPNN	STTSFHSNKE	RAKRLNLASN	DTPSDRLTKL	LTSSST----
(PRED)	tode_1_d04040	RESLAGSDVS	FRD-ALGQEE	------LFKG	SRPDLELRHT	TTFDAQ----
(PRED)	naca_1_e01650	SNSIALSESS	FV--------	---------G	RNPEKQTINN	KTGEV-----
(PRED)	tebl_1_g02820	QEKCALRVQN	SN--GSSHVE	D-----DHAK	STFEETISRV	MSNHSR----
(PRED)	lakl_1_c11616g	QPSTSSSSEA	DDDVSFNRSK	AIEYRSGVVS	ETPIDHVTRI	MSRRS-----
(PRED)	saar_1_8_h03780	----------	----------	----------	----------	----------
(PRED)	sace_14_7_g0015	----------	----------	----------	----------	----------
(PRED)	sace_15_7_g0387	----------	----------	----------	----------	----------
(PRED)	sace_24_8_3780	----------	----------	----------	----------	----------
(PRED)	sace_40_8_h0383	----------	----------	----------	----------	----------
(PRED)	sace_6_169_fm00	----------	----------	----------	----------	----------
(PRED)	sace_19_7_3840	----------	----------	----------	----------	----------
(PRED)	sace_32_7_3770	----------	----------	----------	----------	----------
(PRED)	sace_56_17_q011	----------	----------	----------	----------	----------
(PRED)	sace_5_78_bz001	----------	----------	----------	----------	----------
(PRED)	sace_2_8_h03860	----------	----------	----------	----------	----------
(PRED)	sace_53_29_ac00	----------	----------	----------	----------	----------
(PRED)	sace_17_7_g0393	----------	----------	----------	----------	----------
(PRED)	sace_25_7_g0388	----------	----------	----------	----------	----------
(PRED)	sace_37_7_g0385	----------	----------	----------	----------	----------
(PRED)	sace_9_7_g00180	----------	----------	----------	----------	----------
(PRED)	sace_60_6_f0335	----------	----------	----------	----------	----------
(PRED)	sace_59_336_lx0	----------	----------	----------	----------	----------
(PRED)	sace_31_7_3780	----------	----------	----------	----------	----------
(PRED)	sace_34_8_3770	----------	----------	----------	----------	----------
(PRED)	sace_58_71_bs00	----------	----------	----------	----------	----------
(PRED)	sace_7_7_g03880	----------	----------	----------	----------	----------
(PRED)	sace_35_7_3840	----------	----------	----------	----------	----------
(PRED)	sace_43_7_g0387	----------	----------	----------	----------	----------
(PRED)	sace_57_8_h0390	----------	----------	----------	----------	----------
(PRED)	sace_45_7_g0389	----------	----------	----------	----------	----------
(PRED)	sace_46_8_h0391	----------	----------	----------	----------	----------
(PRED)	sace_23_7_3860	----------	----------	----------	----------	----------
(PRED)	sace_21_7_3790	----------	----------	----------	----------	----------
(PRED)	sace_8_73_bu001	----------	----------	----------	----------	----------
(PRED)	sapa_1_8_h03820	----------	----------	----------	----------	----------
(PRED)	sapa_21_8_h0387	----------	----------	----------	----------	----------
(PRED)	sapa_20_8_h0386	----------	----------	----------	----------	----------
(PRED)	sapa_22_8_h0390	----------	----------	----------	----------	----------
(PRED)	sapa_25_8_h0387	----------	----------	----------	----------	----------
(PRED)	sapa_6_8_3750	----------	----------	----------	----------	----------
(PRED)	sapa_9_8_3720	----------	----------	----------	----------	----------
(PRED)	sapa_19_8_h0390	----------	----------	----------	----------	----------
(PRED)	sapa_24_8_h0385	----------	----------	----------	----------	----------
(PRED)	sapa_4_8_h03850	----------	----------	----------	----------	----------
(PRED)	sapa_10_8_3760	----------	----------	----------	----------	----------
(PRED)	sapa_13_8_h0382	----------	----------	----------	----------	----------
(PRED)	sapa_8_8_3750	----------	----------	----------	----------	----------
(PRED)	sapa_11_8_h0383	----------	----------	----------	----------	----------
(PRED)	sapa_5_8_3700	----------	----------	----------	----------	----------
(PRED)	sapa_16_8_h0389	----------	----------	----------	----------	----------

(PRED)	sapa_17_8_3730	----------	----------	----------	----------	----------
(PRED)	sapa_2_8_h03860	----------	----------	----------	----------	----------
(PRED)	sapa_7_8_3740	----------	----------	----------	----------	----------
(PRED)	sapa_23_8_h0385	----------	----------	----------	----------	----------
(PRED)	sapa_3_8_h03890	----------	----------	----------	----------	----------
(PRED)	sapa_18_8_3730	----------	----------	----------	----------	----------
(PRED)	sami_1_14_399	----------	----------	----------	----------	----------
(PRED)	sace_4_8_h03690	----------	----------	----------	----------	----------
(PRED)	saku_1_14_404	----------	----------	----------	----------	----------
(PRED)	sace_1_ynr070w	----------	----------	----------	----------	----------
(PRED)	sace_49_8_h0383	----------	----------	----------	----------	----------
(PRED)	saeu_1_2_b00130	----------	----------	----------	----------	----------
(PRED)	sauv_1_7_3	----------	----------	----------	----------	----------
(PRED)	sami_1_17_26	ESEVSLSNEN	FQTKPII---	---------D	DTQGDKIVKM	LT--------
(PRED)	zyba_1_02055_AN	AEQAQKNPHS	HG---NSEQD	KKYSKDEDED	EDNGAALHEM	LTRT------
(PRED)	zyba_1_07912	AEQAQ-NPHS	HG---NSEQD	KQNSKDQDED	EDNGAALHEM	LTRT------
(PRED)	zyba_2_2_b00600	AEQAQ-NPHS	HG---NSEQD	KQNSKDQDED	EDNGAALHEM	LTRT------
(PRED)	zyba_3_3_c03460	AEQAQ-NPHS	HG---NSEQD	KQNSKDQDED	EDNGAALHEM	LTRT------
(PRED)	zyba_1_04634	GER-----RE	FH---NNE--	----------	-----GLQEM	LTQS------
(PRED)	zyba_1_06675	GER-----HE	FH---NNE--	----------	-----GLQEM	PTQS------
(PRED)	zyba_3_2_b02230	GER-----HE	FH---NNE--	----------	-----GLQEM	PTQS------
(PRED)	zyba_2_1_a00860	GER-----HE	FH---NNE--	----------	-----GLQEM	PTQS------
(PRED)	zyro_1_a04114g	GSLVGVGHTE	GNTGANTSGF	SRGDIERGAN	--DDVELHEM	LAKY------
(PRED)	zyro_1_b14762g	NSVVGITANA	DGTGVETDRV	ERAPQQNALN	NEDGGKLHEM	LTRN------
(PRED)	zyba_2_14_n0149	VFS-------	-----DDE--	----------	-----EFHEM	ITHG------
(PRED)	zyba_2_33_ag001	VFS-------	-----EEE--	----------	-----VFHEM	ITHG------
(PRED)	lath_1_a01914g	SRSEAEHEAA	YT--GPGTKE	EFQDARQSDG	QAARDHIVRL	MARRT-----
(PRED)	lawa_1_23_5161	TDEEEEAEKR	YF--GPEGEP	EFKDAPGGDG	QSAQAHITRL	TARRT-----
(PRED)	klae_1_14_n0012	---MDFERQS	ED--DGVSTA	------SSVH	DLGDVRDKER	SSEDK-----
(PRED)	klla_1_d03432g	NNGLSSRPEK	LD--GDPRST	------SDTS	SSGSCTSLKE	FSGNN-----
(PRED)	klma_1_1_a01880	----------	----------	------MEVS	STGSGIS-RA	VSVEE-----
(PRED)	klwi_1_33_ag001	----------	ME--GDQGSK	------ASTA	S-----SLKA	FDNER-----
(PRED)	teph_1_a04220	HTNIQESEIK	YNNNR--NQG	------PMDD	EASSSKLSYS	HV----SDIG
(PRED)	vapo_1_1037_47	ISEINEKLGD	LNNSRANNNG	------ATND	E-SEETLEPE	HILENFDNTG
(PRED)	pata_1_2_b05590	RGTFRGRVSH	GSGGVEGTIG	DGEYMEDDDE	GAEADADADA	EA--------
(PRED)	wian_1_3_c04380	RGSTR---EY	GNG-------	------DEVT	GNDYEEIARI	VT--------
(PRED)	wian_1_3_c04390	QQVER---SS	TQF-------	------QD-A	ASDYEEIARI	VT--------
(PRED)	wian_1_7_g01010	DAVSR---PQ	VAG-------	------DD--	ASEYEEIARI	VT--------
(PRED)	bain_1_1_a00100	NTSSS--MDE	AE--KYNDSA	------KKAS	VSP-------	----LSGSDS
(PRED)	bain_1_17_q0038	NTSSD--MEE	AE--KYNGSA	------KKPS	ESP-------	----LSDSDS
(PRED)	bain_1_8_h00410	TSSQELILDF	GS--KAPGHS	------VTQE	EEY-------	----FEASNR
(PRED)	caal_1_19_5759	IGSSSSSLQE	YV--GQQQHN	---------K	IQP-------	----STDDDY
(PRED)	caal_11_25_y002	IGSSSSSLQE	YV--GQQQHN	---------K	IQP-------	----STDDDY
(PRED)	caal_4_4_d03320	IGSSSSSLQE	YV--GQQQHN	---------K	IQP-------	----STDDDY
(PRED)	caal_12_26_z005	IGSSSSSLQE	YV--GQQQHN	---------K	IQP-------	----STDDDY
(PRED)	caal_5_30_ad005	IGSSSSSLQE	YV--GQQQHN	---------K	IQP-------	----STDDDY
(PRED)	caal_8_3_c03320	IGSSSSSLQE	YV--GQQQHN	---------K	IQP-------	----STDDDY
(PRED)	caal_6_4_d03280	IGSSSSSLQE	YV--GQQQHN	---------K	IQP-------	----STDDDY
(PRED)	caal_10_3_c0334	IGSSSSSLQE	YV--GQQQHN	---------K	IQP-------	----STDDDY
(PRED)	caal_3_29_ac005	IGSSSSSLQE	YV--GQQQHN	---------K	IQP-------	----STDDDY
(PRED)	caal_2_04989	IGSSSSSLQE	YV--GQQQHN	---------K	IQP-------	----STDDDY
(PRED)	cadu_1_64350	IESSSSSLQE	YV--G-QQHH	---------K	TQP-------	----STDEDY
(PRED)	caor_1_h02090	TTASTSSIED	FT--GQHPHH	HSYNHNESEK	VNE-NGLRRT	DSVLGSDEDF
(PRED)	capa_1_600750	STASTSSIED	FT--GQRNHH	---DHIGNEK	IDDNNSLRRT	DSVLGSEDDY
(PRED)	loel_1_04930	SSSTNSSIAE	YT--G---HH	H---QDQKEK	L-EAEKLSRY	ESHSQFQADY
(PRED)	spar_1_5_e03260	ITSEIQSLEE	FR--PVKS--	---------V	FEP-------	------EDDY
(PRED)	sppa_1_7_g03160	ITSDIQSLEE	FR--PVKS--	---------V	FEP-------	------EDDY
(PRED)	catr_1_01205	Y-STETSIVD	YD--TNYYR-	--------QQ	LQL-------	----EQVEGE
(PRED)	catr_1_05498	--SNSYSIEE	YG--ADYF--	-------GQQ	GSS-------	----GLHDES
(PRED)	catr_1_05971	Y-STSPSIGE	YD--ADYHRN	---QNQQQQQ	QHG-------	----DLQDDY
(PRED)	deha_1_a03696g	ISNANDGATI	SS--SI---N	---------E	YYG-------	-----ADQ--
(PRED)	deha_2_5_e00720	ISNSHEGATS	TS--SI---N	---------E	YYG-------	-----ADQ--
(PRED)	scst_1_3_c02890	RQSSESGSDD	VV--DIFHGQ	---------H	YEG-------	---QKLEE--
(PRED)	mebi_1_8_h00300	MANTVSEIKS	VT--NATDVA	----------	----------	----ASDSTG
(PRED)	lakl_1_h21010g	----------	----------	----------	----------	----------
(PRED)	caar_1_13_m0142	----------	-M--------	------MLFP	ITKLIE-MKS	L-TFI-----
(PRED)	caar_1_14_n0143	---------M	LK--------	------IFVR	FMLQMS-NRN	L-ENF-----
(PRED)	hapo_1_1_a07220	EMERGPSGET	VD--------	------SVRP	FEIDDE-ARD	I-AEL-----
(PRED)	ogpa_1_1_a01680	EMERGQSNET	VD--------	------SVRP	FEVDDE-GRE	I-AEL-----
(PRED)	piku_1_96_cr001	MAETGKENKT	EF--------	--------EN	NADEDELSEN	I-SNI-----
(PRED)	pime_1_4_d03240	FHPGASHEKQ	QHGVPAPQAG	------SQEH	HQIRDDFSDE	V-DYI-----
(PRED)	pime_1_1_a12110	DTSQSGDNEI	VY--------	-------YQP	SSNEDEVAEE	L-QYL-----
(PRED)	piku_1_227_hs00	-MDPNTDTEA	L---------	---------P	G--TEAYY--	----------
(PRED)	pime_1_5_e05800	SADAEEKADS	IC--------	------GPQP	G--TTEYYDS	VNASI-----
(PRED)	pime_1_1_a07690	SKRLPLDGSS	VT--------	------EDDG	DLKSEQRFQE	LNDTI-----

```
(PRED) debr_2_5_e03380 ESYHGYSDQE E--------- --------EE FRESQDIGE- ----L-----
(PRED) kopa_1_2_b10040 SHNNSSGDEV L--------- ---------S YRAEDEQAQ- ----------
(PRED) kopa_2_7_g00500 SHDNSSGDEV L--------- ---------S YRAEDEQAQ- ----------
(PRED) asru_1_13_m0119 -MNPSHSDTD STVESFYG-- -DPLKDNANN LDRIITNASY QIASTE----
(PRED) asru_1_15_o0045 LVANKISDDS STINSFHGKV SDLILDNRN- -DQI--SSDQ DLVDDE----
(PRED) wian_1_1_a02920 --MNT-SPER ALE------- --L------- -------EKS QTFDEY----
(PRED) wian_1_1_a02930 -MSNS-SSDS RYEEKPYGGE NDI------- -------ADK ESRDEF----

                       .........160 .........170 .........180 .........190 .........200
(PRED) asac_1_6_f03560 -------TGE ARA----A-- --LDPGMAQK VE-------- SLARTLSQHT
(PRED) ergo_1_abr125c  -------MGE ARA----A-- --LDPGMAQK VE-------- SLARTLSQHT
(PRED) ercy_1_3604     -------VGG GAA----GVA GELDEEVIGK IK-------- SLARTLSRHT
(PRED) cagl_1_i04862g  GDDAHSDNRS ILSRSRRSST AELSPEMVGR VQ-------- SLADVLSRHT
(PRED) kaaf_1_c00830   ------DVEN ---------- -GRPSEIVSQ VE-------- TLAEILSKHT
(PRED) kana_1_k01350   ------DVAS HTSHASHATV GMFSPDVASK VE-------- SLARELSRKT
(PRED) saar_1_2_b02590 ------ETLD Q--------N DDMSPNVASK VE-------- SFAEALSRHT
(PRED) sace_1_ydr011w  ------DTAS QIS----ATV SEMAPDVVSK VE-------- SFADALSRHT
(PRED) sace_16_1_a0238 ------DTAS QIS----ATV SEMAPDVVSK VE-------- SFADALSRHT
(PRED) sace_45_1_a0242 ------DTAS QIS----ATV SEMAPDVVSK VE-------- SFADALSRHT
(PRED) sace_48_1_a0238 ------DTAS QIS----ATV SEMAPDVVSK VE-------- SFADALSRHT
(PRED) sace_60_4_d0244 ------DTAS QIS----ATV SEMAPDVVSK VE-------- SFADALSRHT
(PRED) sace_52_1_a0240 ------DTAS QIS----ATV SEMAPDVVSK VE-------- SFADALSRHT
(PRED) sace_46_1_a0240 ------DTAS QIS----ATV SEMAPDVVSK VE-------- SFADALSRHT
(PRED) sace_25_1_a0240 ------DTAS QIS----ATV SEMAPDVVSK VE-------- SFADALSRHT
(PRED) sace_24_1_2300  ------DTAS QIS----ATV SEMAPDVVSK VE-------- SFADALSRHT
(PRED) sace_47_1_a0240 ------DTAS QIS----ATV SEMAPDVVSK VE-------- SFADALSRHT
(PRED) sace_7_1_a02410 ------DTAS QIS----ATV SEMAPDVVSK VE-------- SFADALSRHT
(PRED) sace_59_110_df0 ------DTAS QIS----ATV SEMAPDVVSK VE-------- SFADALSRHT
(PRED) sace_56_1_a0202 ------DTAS QIS----ATV SEMAPDVVSK VE-------- SFADALSRHT
(PRED) sace_40_1_a0239 ------DTAS QIS----ATV SEMAPDVVSK VE-------- SFADALSRHT
(PRED) sace_15_1_a0242 ------DTAS QIS----ATV SEMAPDVVSK VE-------- SFADALSRHT
(PRED) sace_37_1_a0243 ------DTAS QIN----ATL SEMAPDVVSK VE-------- SFADALSRHT
(PRED) sace_9_1_a02440 ------DTAS QIS----ATV SEMAPDVVSK VE-------- SFADALSRHT
(PRED) sace_22_1_2300  ------DTAS QIS----ATV SEMAPDVVSK VE-------- SFADALSRHT
(PRED) sace_29_1_2290  ------DTAS QIS----ATV SEMAPDVVSK VE-------- SFADALSRHT
(PRED) sace_34_1_2320  ------DTAS QIS----ATV SEMAPDVVSK VE-------- SFADALSRHT
(PRED) sace_58_25_y007 ------DTAS QIS----ATV SEMAPDVVSK VE-------- SFADALSRHT
(PRED) sace_23_1_2290  ------DTAS QIS----ATV SEMAPDVVSK VE-------- SFADALSRHT
(PRED) sace_6_120_dp00 ------DTAS QIS----ATV SEMAPDVVSK VE-------- SFADALSRHT
(PRED) sace_57_1_a0241 ------DTAS QIS----ATL SEIAPDVVSK VE-------- SFADALSRHT
(PRED) sace_17_1_a0241 ------DTAS QIS----ATV SEMAPDVVSK VE-------- SFADALSRHT
(PRED) sace_21_1_2310  ------DTAS QIS----ATV SEMAPDVVSK VE-------- SFADALSRHT
(PRED) sace_49_1_a0246 ------DTAS QIS----ATV SEMAPDVVSK VE-------- SFADALSRHT
(PRED) sace_8_2_b02430 ------DTAS QIS----ATV SEMAPDVVSK VE-------- SFADALSRHT
(PRED) sace_31_1_2300  ------GTAS QIS----ATV SEMAPDVVSK VE-------- SFADALSRHT
(PRED) sace_50_1_a0241 ------DTAS QIS----ATV SEMAPDVVSK VE-------- SFADALSRHT
(PRED) sace_4_1_a02360 ------DTAS QIS----ATV SEMAPDVVSK VE-------- SFADALSRHT
(PRED) sace_2_1_a02390 ------DTAS QIS----ATV SEMAPDVVSK VE-------- SFADALSRHT
(PRED) sace_5_33_ag005 ------DTAS QIS----ATV SEMAPDVVSK VE-------- SFADALSRHT
(PRED) sapa_11_1_a0247 ------DSAS QIS----AAM SDMSPDVVSK VE-------- SFADALSRHT
(PRED) sapa_25_1_a0246 ------DSAS QIS----AAM SDMSPDVVSK VE-------- SFADALSRHT
(PRED) sapa_4_1_a02470 ------DSAS QIS----AAM SDMSPDVVSK VE-------- SFADALSRHT
(PRED) sapa_5_1_2350   ------DSAS QIS----AAM SDMSPDVVSK VE-------- SFADALSRHT
(PRED) sapa_9_1_2360   ------DSAS QIS----AAM SDMSPDVVSK VE-------- SFADALSRHT
(PRED) sapa_14_1_a0244 ------DSAS QIS----AAM SDMSPDVVSK VE-------- SFADALSRHT
(PRED) sapa_8_1_2350   ------DSAS QIS----AAM SDMSPDVVSK VE-------- SFADALSRHT
(PRED) sapa_17_1_2380  ------DSAS QIS----AAM SEMSPDVVSK VE-------- SFADALSRHT
(PRED) sapa_7_1_2370   ------DSAS QIS----AAM SEMSPDVVSK VE-------- SFADALSRHT
(PRED) sapa_2_1_a02460 ------DSAS QIS----AAM SEMSPDVVSK VE-------- SFADALSRHT
(PRED) sapa_23_1_a0248 ------DSAS QIS----AAM SEMSPDVVSK VE-------- SFADALSRHT
(PRED) sapa_3_1_a02470 ------DSAS QIS----AAM SEMSPDVVSK VE-------- SFADALSRHT
(PRED) sapa_18_1_2390  ------DSAS QIS----AAI SGMSPDVVSK VE-------- SFADALSRHT
(PRED) sami_1_4_244    ------DSAS QIS----ATV SDMSPDVVSK VE-------- SFADALSRHT
(PRED) saku_1_4_262    ------DTVS HVS----ATV SDMSPDVVSK VE-------- SFADALSRHT
(PRED) saba_1_58_bf002 ------DSMS QLS----ASV SNMPPDVVSK VE-------- SLADALSRHT
(PRED) saeu_1_4_d02400 ------DSMS QIS----IAA SNMTPDVISK VE-------- SMADALSRHT
(PRED) naca_1_e01640   ------IDNE SIT------- --FSPEIISK VE-------- TFADALSHYT
(PRED) nada_1_g01850   ------GSYQ ASQ------- --FSADVVSN VE-------- SLAEVLSHHT
(PRED) naca_1_e01630   ------EPLK NRAKLNYASS GELTADMVSR VE-------- SFADALSHHT
(PRED) nada_1_g01840   ------NNKK NYSNGNEESF TYLSDNMITR VE-------- SFADALSRHT
(PRED) kaaf_1_c00820   ------HNRN E--------- SSLSDDMTSK VE-------- QFAEALARHT
(PRED) teph_1_m00640   ------HRSS VV-------- --MSDDAMSQ VQ-------- DLARTLSHRT
(PRED) vapo_1_1036_28  ------RRAS MV-------- --MSDDVKSQ VE-------- CLAKTLSRHT
(PRED) tebl_1_i01760   -PSHHKDSMT EPHRRDRRLS TELSPDLISQ IE-------- SLAKTLSFHT
(PRED) tode_1_d04040   ---------- ---------- ------TIEQ IH-------- TLARTLSKRT
```

```
(PRED) naca_1_e01650   ------STDS QDS------- DELNKDYIHR VE-------- TVADVLSRHT
(PRED) tebl_1_g02820   -------NEK FI-------- --VTDDMAKS IN-------- EYTAKLKKHT
(PRED) lakl_1_c11616g  -------TID EQQ-----NP DQTDPEVISK VE-------- TLARTLSKHR
(PRED) saar_1_8_h03780 ---------- ---------- ---------- ---------- -----MAHRN
(PRED) sace_14_7_g0015 ---------- ---------- ---------- ---------- -----MDFHT
(PRED) sace_15_7_g0387 ---------- ---------T LIRSSDGRPQ IN-------- --LNIMDFHT
(PRED) sace_24_8_3780  ---------- ---------T LIRSSDGRPQ IN-------- --LNIMDFHT
(PRED) sace_40_8_h0383 ---------- ---------T LIRSSDGRPQ IN-------- --LNIMDFHT
(PRED) sace_6_169_fm00 ---------- ---------T LIRSSDGRPQ IN-------- --LNIMDFHT
(PRED) sace_19_7_3840  ---------- ---------T LIRSSDGRPQ IN-------- --LNIMDFHT
(PRED) sace_32_7_3770  ---------- ---------T LIRSSDGRPQ IN-------- --LNIMDFHT
(PRED) sace_56_17_q011 ---------- ---------T LIRSSDGRPQ IN-------- --LNIMDFHT
(PRED) sace_5_78_bz001 ---------- ---------T LIRSSDGRPQ IN-------- --LNIMDFHT
(PRED) sace_2_8_h03860 ---------- ---------- ---------- ---------- -----MDFHT
(PRED) sace_53_29_ac00 ---------- ---------- ---------- ---------- -----MDFHT
(PRED) sace_17_7_g0393 ---------- ---------T LIRSSDGRPH IN-------- --LNIMDFHT
(PRED) sace_25_7_g0388 ---------- ---------T LIRSSDGRPH IN-------- --LNIMDFHT
(PRED) sace_37_7_g0385 ---------- ---------T LIRSSDGRPH IN-------- --LNIMDFHT
(PRED) sace_9_7_g00180 ---------- ---------T LIRSSDGRPH IN-------- --LNIMDFHT
(PRED) sace_60_6_f0335 ---------- ---------T LIRSSDGRPH IN-------- --LNIMDFHT
(PRED) sace_59_336_lx0 ---------- ---------T LIRSSDGRPH IN-------- --LNIMDFHT
(PRED) sace_31_7_3780  ---------- ---------T LIRSSDGRPQ IN-------- --LNIMDFHT
(PRED) sace_34_8_3770  ---------- ---------T LIRSSDGRPQ IN-------- --LNIMDFHT
(PRED) sace_58_71_bs00 ---------- ---------T LIRSSDGRPQ IN-------- --LNIMDFHT
(PRED) sace_7_7_g03880 ---------- ---------T LIRSSDGRPQ IN-------- --LNIMDFHT
(PRED) sace_35_7_3840  ---------- ---------T LIRSSDGRPQ IN-------- --LNIMDFHT
(PRED) sace_43_7_g0387 ---------- ---------T LIRSSDGRPQ IN-------- --LNIMDFHT
(PRED) sace_57_8_h0390 ---------- ---------T LIRSSDGRPQ IN-------- --LNIMDFHT
(PRED) sace_45_7_g0389 ---------- ---------T LIRSSDGRPH IN-------- --LNIMDFHT
(PRED) sace_46_8_h0391 ---------- ---------T LIRSSDGRPH IN-------- --LNIMDFHT
(PRED) sace_23_7_3860  ---------- ---------T LIRSSDGRPQ IN-------- --LNIMDFHT
(PRED) sace_21_7_3790  ---------- ---------T LIRSSDGRPQ IN-------- --LNIMDFHT
(PRED) sace_8_73_bu001 ---------- ---------- ---------- ---------- -----MDFHT
(PRED) sapa_1_8_h03820 ---------- ---------- ---------- ---------- -----MALHT
(PRED) sapa_21_8_h0387 ---------- ---------- ---------- ---------- -----MALHT
(PRED) sapa_20_8_h0386 ---------- ---------- ---------- ---------- -----MALHT
(PRED) sapa_22_8_h0390 ---------- ---------- ---------- ---------- -----MALHT
(PRED) sapa_25_8_h0387 ---------- ---------- ---------- ---------- -----MALHT
(PRED) sapa_6_8_3750   ---------- ---------- ---------- ---------- -----MALHT
(PRED) sapa_9_8_3720   ---------- ---------- ---------- ---------- -----MALHT
(PRED) sapa_19_8_h0390 ---------- ---------- ---------- ---------- -----MALHT
(PRED) sapa_24_8_h0385 ---------- ---------- ---------- ---------- -----MALHT
(PRED) sapa_4_8_h03850 ---------- ---------- ---------- ---------- -----MALHT
(PRED) sapa_10_8_3760  ---------- ---------- ---------- ---------- -----MALHT
(PRED) sapa_13_8_h0382 ---------- ---------- ---------- ---------- -----MALHT
(PRED) sapa_8_8_3750   ---------- ---------- ---------- ---------- -----MALHT
(PRED) sapa_11_8_h0383 ---------- ---------N PIGDSDGRSQ NN-------- --LNIMALHT
(PRED) sapa_5_8_3700   ---------- ---------N PIGDSDGRPQ NN-------- --LNIMALHT
(PRED) sapa_16_8_h0389 ---------- ---------N PIRDSDGRSQ VN-------- --LNIMALHT
(PRED) sapa_17_8_3730  ---------- ---------N PIRDSDGRSQ VN-------- --LNIMALHT
(PRED) sapa_2_8_h03860 ---------- ---------N PIRDSDGRSQ VN-------- --LNIMALHT
(PRED) sapa_7_8_3740   ---------- ---------N PIRDSDGRSQ VN-------- --LNIMALHT
(PRED) sapa_23_8_h0385 ---------- ---------N PIRDSDGRSQ VN-------- --LNIMALHT
(PRED) sapa_3_8_h03890 ---------- ---------- ---------- ---------- -----MALHT
(PRED) sapa_18_8_3730  ---------- ---------N PIGDSDGRSQ VN-------- --LNIMALHT
(PRED) sami_1_14_399   ---------- ---------- ---------- ---------- -----MTTQT
(PRED) sace_4_8_h03690 ---------- ---------- ---------- ---------- -----MDFXT
(PRED) saku_1_14_404   ---------- ---------- ---------- ---------- -----MAFRT
(PRED) sace_1_ynr070w  ---------- ---------- ---------- ---------- ----------
(PRED) sace_49_8_h0383 ---------- ---------- ---------- ---------- ----------
(PRED) saeu_1_2_b00130 ---------- ---------- ---------- ---------- -----MARYT
(PRED) sauv_1_7_3      ---------- ---------- ---------- ---------- -----MALHT
(PRED) sami_1_17_26    -------SHS HISSFNDCRN DEVNDDGGPG IDEDTVARIQ TIARTLSHHT
(PRED) zyba_1_02055_AN ------GSGG QSS------- -QLCPEVASR VR-------- SLARTLSTRR
(PRED) zyba_1_07912    ------RSGG QSS------- -QLCPEVASR VR-------- SLARTLSTRR
(PRED) zyba_2_2_b00600 ------RSGG QSS------- -QLCPEVASR VR-------- SLARTLSTRR
(PRED) zyba_3_3_c03460 ------RSGG QSS------- -QLCPEVASR VR-------- SLARTLSTRR
(PRED) zyba_1_04634    ------KTSS QSS------- -PLSPEMASK VR-------- TLARTLSSRR
(PRED) zyba_1_06675    ------ESGS QSS------- -PLSPEMAFK VR-------- TLARTLSSKQ
(PRED) zyba_3_2_b02230 ------ESGS QSS------- -PLSPEMAFK VR-------- TLARTLSSKQ
(PRED) zyba_2_1_a00860 ------ESGS QSS------- -PLSPEMAFK VR-------- TLARTLSSKQ
(PRED) zyro_1_a04114g  ------KSNG QGS------- -TFTPELASK VE-------- SLARTLSKRK
(PRED) zyro_1_b14762g  ------RSNG QGS------- -QFSPEVTSK VQ-------- SLARTLSKHR
(PRED) zyba_2_14_n0149 ------RMGG QNS------- -QLSPDVASK VR-------- SIARTLSHRR
(PRED) zyba_2_33_ag001 ------KTGG QNT------- -QLSPDVASK VR-------- SLARTLSHRR
```

```
(PRED) lath_1_a01914g ------SSVG  EEE-------  -DLDPEVLSR  VE--------  TLARTLSSHR
(PRED) lawa_1_23_5161 ------NS-G  -LQ-------  -DLDAESISR  IH--------  TLARTLSNYQ
(PRED) klae_1_14_n0012------IDI-  ----------  --MAPEYAQR  VE--------  SMVRTLSRLT
(PRED) klla_1_d03432g ------HELH  QDTDQQVVKD  AAEDPAFLKK  VE--------  TLSRSLSRRT
(PRED) klma_1_1_a01880------FDVA  VDKG-EVIKR  ASEDPEFLKK  VE--------  TLSKTLSRRS
(PRED) klwi_1_33_ag001------FE--  ---------Q  ARADPEFLHK  VE--------  TLARTLSHRT
(PRED) teph_1_a04220  ------SHQT  TTSE----EN  LDNISRYIRS  IS--------  SLGS-----E
(PRED) vapo_1_1037_47 RSFAKDSRPS  SISEHESTES  LTN-SRLVNR  IT--------  SLARTFSHVE
(PRED) pata_1_2_b05590------TSLG  H---------  --YRGDALTR  LS--------  TLSKTLSHMT
(PRED) wian_1_3_c04380------DSHS  Q---------  --EEGGVLHK  LE--------  TLSKQLSRRT
(PRED) wian_1_3_c04390------NSQN  E---------  --HDGGVLHK  LE--------  TLSKTLSRRT
(PRED) wian_1_7_g01010------NSQN  Q---------  --ENGGVLQK  LE--------  TLSKHLSKRT
(PRED) bain_1_1_a00100S---EHDFVA  DPHARDEATR  IMTNPAIVER  LT--------  SYTERLSRH-
(PRED) bain_1_17_q0038L---EHDFVA  DPHVRHEATR  IMTNLAAVER  LA--------  SYTKQLSHH-
(PRED) bain_1_8_h00410E---AITRLI  SNIDPDDPNN  VLNR---LES  LT--------  NEYGMANAE-
(PRED) caal_1_19_5759 N---EDDYES  RRLHLVRTVS  SINHHNFDEK  FD--------  TISREISRQV
(PRED) caal_11_25_y002N---EDDYES  RRLHLVRTVS  SINHHNFDEK  FD--------  TISREISRQV
(PRED) caal_4_4_d03320N---EDDYES  RRLHLVRTVS  SINHHNFDEK  FD--------  TISREISRQV
(PRED) caal_12_26_z005N---EDDYES  RRLHLVRTVS  SINHHNFDEK  FD--------  SISREISRQV
(PRED) caal_5_30_ad005N---EDDYES  RRLHLVRTVS  SINHHNFDEK  FD--------  SISREISRQV
(PRED) caal_8_3_c03320N---EDDYES  RRLHLVRTVS  SINHHNFDEK  FD--------  SISREISRQV
(PRED) caal_6_4_d03280N---EDDYES  RRLHLVRTVS  SINHHNFDEK  FD--------  SISREISRQV
(PRED) caal_10_3_c0334N---EDDYES  RRLHLVRTVS  SINHHNFDEK  FD--------  SISREISRQV
(PRED) caal_3_29_ac005N---EDDYES  RRLHLVRTVS  SINHHNFDEK  FD--------  TISREISRQV
(PRED) caal_2_04989   N---EDDYES  RRLHLVRTVS  SINHHNFDEK  FD--------  TISREISRQV
(PRED) cadu_1_64350   NDYDENNDES  RRLHLVRTVS  SINRHNFDEK  FD--------  SISREISRQV
(PRED) caor_1_h02090  D---DNNDES  RRLHLVRTIT  ALSNKNSSDQ  LD--------  SLSRHISRQI
(PRED) capa_1_600750  D---DNNEES  RRLHLVRTIT  ALSNKASTDQ  LD--------  SLSRHISRQI
(PRED) loel_1_04930   N---DNDEES  RRLHLVRTIT  ALSQKEGAGA  LD--------  SISREISRQV
(PRED) spar_1_5_e03260D---ENDEAS  RHASLIRTIS  SMHQIDYDEK  LN--------  SLSREISREI
(PRED) sppa_1_7_g03160D---EHDDES  RRMSLIRTIT  SMHQKDYDDK  LN--------  TLSREISRQI
(PRED) catr_1_01205   E---EEGEES  RRLNLVRTIS  AIENHEIDKK  LD--------  SISREISREI
(PRED) catr_1_05498   T---NIDEET  RRASLVRTIT  AIEANEYQQK  FD--------  SISREISRQI
(PRED) catr_1_05971   D---ENDEES  RRLHLVRTIS  AIEAHDFEQK  FD--------  SISREISRQV
(PRED) deha_1_a03696g ----EAVEQN  DRLELVRSIT  SGNSRDVLDR  LE--------  TLSREMSRR-
(PRED) deha_2_5_e00720----ELIEQN  NRLELVRSIT  SGNSGDVLDR  LE--------  TLSREMSRR-
(PRED) scst_1_3_c02890----EPTDEQ  RRLELVRTVT  ELNSQSVLDR  LE--------  TLSRQLSQH-
(PRED) mebi_1_8_h00300T---HDSQMT  PAGSVADYVV  SPNSANFLSQ  VE--------  TLSRRMSRKS
(PRED) lakl_1_h21010g ----MSFLLP  RLQQGSQDDY  NHADEANGEK  IH--------  --ASESSSDT
(PRED) caar_1_13_m0142------KLSL  --R----SVS  NNQDDNVLTR  LS--------  TLSKTLSKMT
(PRED) caar_1_14_n0143------XKTL  S-R----SVT  NNAEDNVLTR  LS--------  TLSKTLTRMT
(PRED) hapo_1_1_a07220------HRSI  THQ----SSG  QGQQGDVLTR  LS--------  TLSRTMSRMN
(PRED) ogpa_1_1_a01680------HRSI  THQ----SSG  QGQQGDVLTR  LS--------  TLSRTMSKMN
(PRED) piku_1_96_cr001------YRAI  THETEFNASN  GKDSDPILKR  LS--------  TLSKTLSTMN
(PRED) pime_1_4_d03240------HRAL  TRG---QSEE  HPETD-VLRR  LS--------  TLSQTLSHMN
(PRED) pime_1_1_a12110------QRSV  TNQ-------  ----D-LMSK  LS--------  ELSHQMSHMT
(PRED) piku_1_227_hs00-------RDL  NNT----ISS  DSD---VMER  LS--------  KLSRRLSHMT
(PRED) pime_1_5_e05800------HRSL  SGS----LTG  NDEHMNVMTR  LS--------  TLSKQMSKLT
(PRED) pime_1_1_a07690------HRSV  TNN----PND  N-----VMTR  LS--------  TLSKTLSTKN
(PRED) debr_2_5_e03380------HRVV  TQ-----SRQ  SSVGPDVLTR  LS--------  TLSQTLSHMS
(PRED) kopa_1_2_b10040------LEGV  N---------  -------LDR  LQ--------  SLTKQMSHVT
(PRED) kopa_2_7_g00500------LDGV  N---------  -------LDR  LQ--------  SLTKQMSHVT
(PRED) asru_1_13_m0119------DY-G  DLVRIVTSQN  AHQQGGVLDR  LE--------  SLARTLSTRT
(PRED) asru_1_15_o0045------DQRG  EIMRILTS-N  VHQEGGVFDQ  IQ--------  SLSKTLSQRT
(PRED) wian_1_1_a02920--------EE  DLIRSVTNDI  NT---GVLNT  FE--------  NLVKTMSGKT
(PRED) wian_1_1_a02930------EQEQ  DLIRVVSQDG  IN---KVLTR  FE--------  SLARTISKKN

                      .........210 .........220 .........230 .........240 .........250
(PRED) asac_1_6_f03560ARDGRL----  ----------  VVDP-----D  D----FDMKA  ILSGLVSASR
(PRED) ergo_1_abr125c VRDGRL----  ----------  VVDP-----A  D----FDMKA  ILSGLVSASQ
(PRED) ercy_1_3604    TRDGPL----  ----------  DVGG-----D  D----FDMKA  IFSALVQDSQ
(PRED) cagl_1_i04862g SRSGG--NI-  -DL-------  SQLSE----S  DR---FDAER  IIGSFVRDAD
(PRED) kaaf_1_c00830  TRDGE---L-  -QL-------  RN-------D  DS---FDAEA  IFAAFARDSE
(PRED) kana_1_k01350  TKDGS-----  -QL-------  NCE-P----D  EG---FDAAA  IIGSFVRDAD
(PRED) saar_1_2_b02590TRSGA---F-  -NM-------  APS-D----D  SG---FDAHA  IFKNFVRDAD
(PRED) sace_1_ydr011w TRSGA---F-  -NM-------  DSD-S----D  DG---FDAHA  IFESFVRDAD
(PRED) sace_16_1_a0238TRSGA---F-  -NM-------  DSD-S----D  DG---FDAHA  IFESFVRDAD
(PRED) sace_45_1_a0242TRSGA---F-  -NM-------  DSD-S----D  DG---FDAHA  IFESFVRDAD
(PRED) sace_48_1_a0238TRSGA---F-  -NM-------  DSD-S----D  DG---FDAHA  IFESFVRDAD
(PRED) sace_60_4_d0244TRSGA---F-  -NM-------  DSD-S----D  DG---FDAHA  IFESFVRDAD
(PRED) sace_52_1_a0240TRSGA---F-  -NM-------  DSD-S----D  DG---FDAHA  IFESFVRDAD
(PRED) sace_46_1_a0240TRSGA---F-  -NM-------  DSD-S----D  DG---FDAHA  IFESFVRDAD
(PRED) sace_25_1_a0240TRSGA---F-  -NM-------  DSD-S----D  DG---FDAHA  IFESFVRDAD
(PRED) sace_24_1_2300 TRSGA---F-  -NM-------  DSD-S----D  DG---FDAHA  IFESFVRDAD
(PRED) sace_47_1_a0240TRSGA---F-  -NM-------  DSD-S----D  DG---FDAHA  IFESFVRDAD
```

(PRED)	sace_7_1_a02410	TRSGA---F-	-NM-------	DSD-S----D	DG---FDAHA	IFESFVRDAD
(PRED)	sace_59_110_df0	TRSGA---F-	-NM-------	DSD-N----D	DG---FDAHA	IFESFVRDAD
(PRED)	sace_56_1_a0202	TRSGA---F-	-NM-------	DSD-S----D	DG---FDAHA	IFESFVRDAD
(PRED)	sace_40_1_a0239	TRSGA---F-	-NM-------	DSD-S----G	DG---FDAHA	IFESFVRDAD
(PRED)	sace_15_1_a0242	TRSGA---F-	-NM-------	DSD-S----D	DG---FDAHA	IFESFVRDAD
(PRED)	sace_37_1_a0243	TRSGA---F-	-NM-------	DSD-S----D	DG---FDAHA	IFESFVRDAD
(PRED)	sace_9_1_a02440	TRSGA---F-	-NM-------	DSD-S----D	DG---FDAHA	IFESFVRDAD
(PRED)	sace_22_1_2300	TRSGA---F-	-NM-------	DSD-S----D	DG---FDAHA	IFESFVRDAD
(PRED)	sace_29_1_2290	TRSGA---F-	-NM-------	DSD-S----D	DG---FDAHA	IFESFVRDAD
(PRED)	sace_34_1_2320	TRSGA---F-	-NM-------	DSD-S----D	DG---FDAHA	IFESFVRDAD
(PRED)	sace_58_25_y007	TRSGA---F-	-NM-------	DSD-S----D	DG---FDAHA	IFESFVRDAD
(PRED)	sace_23_1_2290	TRSGA---F-	-NM-------	DSD-S----D	DG---FDAHA	IFESFVRDAD
(PRED)	sace_6_120_dp00	TRSGA---F-	-NM-------	DSD-S----D	DG---FDAHA	IFESFVRDAD
(PRED)	sace_57_1_a0241	TRSGA---F-	-NM-------	DSD-S----D	DG---FDAHA	IFESFVRDAD
(PRED)	sace_17_1_a0241	TRSGA---F-	-NM-------	DSD-S----D	DG---FDAHA	IFESFVRDAD
(PRED)	sace_21_1_2310	TRSGA---F-	-NM-------	DSD-S----D	DG---FDAHA	IFESFVRDAD
(PRED)	sace_49_1_a0246	TRSGA---F-	-NM-------	DSD-S----D	DG---FDAHA	IFESFVRDAD
(PRED)	sace_8_2_b02430	TRSGA---F-	-NM-------	DSD-S----D	DG---FDAHA	IFESFVRDAD
(PRED)	sace_31_1_2300	TRSGA---F-	-NM-------	DSD-S----D	DG---FDAHA	IFESFVRDAD
(PRED)	sace_50_1_a0241	TRSGA---F-	-NM-------	DSD-S----D	DG---FDAHA	IFESFVRDAD
(PRED)	sace_4_1_a02360	TRSGA---F-	-NM-------	DSD-S----D	DG---FDAHA	IFESFVRDAD
(PRED)	sace_2_1_a02390	TRSGA---F-	-NM-------	DSD-S----D	DG---FDAHA	IFESFVRDAD
(PRED)	sace_5_33_ag005	TRSGA---F-	-NM-------	DSD-S----D	DG---FDAHA	IFESFVRDAD
(PRED)	sapa_11_1_a0247	TRSGA---F-	-NM-------	DSG-D----D	DA---FDAHA	IFESFVRDAD
(PRED)	sapa_25_1_a0246	TRSGA---F-	-NM-------	DSG-D----D	DA---FDAHA	IFESFVRDAD
(PRED)	sapa_4_1_a02470	TRSGA---F-	-NM-------	DSG-D----D	DA---FDAHA	IFESFVRDAD
(PRED)	sapa_5_1_2350	TRSGA---F-	-NM-------	DSG-D----D	DA---FDAHA	IFESFVRDAD
(PRED)	sapa_9_1_2360	TRSGA---F-	-NM-------	DSG-D----D	DA---FDAHA	IFESFVRDAD
(PRED)	sapa_14_1_a0244	TRSGA---F-	-NM-------	DSG-A----D	DA---FDAHA	IFESFVRDAD
(PRED)	sapa_8_1_2350	TRSGA---F-	-NM-------	DSG-A----D	DA---FDAHA	IFESFVRDAD
(PRED)	sapa_17_1_2380	TRSGA---F-	-NM-------	DSG-D----D	DA---FDAHA	IFESFVRDAD
(PRED)	sapa_7_1_2370	TRSGA---F-	-NM-------	DSG-D----D	DA---FDAHA	IFESFVRDAD
(PRED)	sapa_2_1_a02460	TRSGA---F-	-NM-------	DSG-D----D	DA---FDAHA	IFESFVRDAD
(PRED)	sapa_23_1_a0248	TRSGA---F-	-NM-------	DSG-D----D	DA---FDAHA	IFESFVRDAD
(PRED)	sapa_3_1_a02470	TRSGA---F-	-NM-------	DSG-D----D	DA---FDAHA	IFESFVRDAD
(PRED)	sapa_18_1_2390	TRSGA---F-	-NM-------	DSG-D----D	DA---FDAHA	IFESFVRDAD
(PRED)	sami_1_4_244	TRSGA---F-	-NM-------	DSG-D----D	DG---FDAHA	IFESFVRDAD
(PRED)	saku_1_4_262	TRSGA---F-	-NM-------	DAG-G----D	DG---FDAHA	IFESFVRDAD
(PRED)	saba_1_58_bf002	TRSGA---F-	-NM-------	DS-------G	DG---FDAQA	IFESFVRHAD
(PRED)	saeu_1_4_d02400	TRSGA---F-	-NM-------	DS-------D	DG---FDAHA	IFESFVRHAD
(PRED)	naca_1_e01640	TRSGP---I-	-DL-------	DSQAG----D	DG---FDARA	IFANFVRDAE
(PRED)	nada_1_g01850	TRDGP---V-	-PT-------	E---G----N	D----FDAAA	IFSKFVRDAN
(PRED)	naca_1_e01630	TRSGS---F-	-DM-------	KNV-S----D	DG---FDAQE	IFERFVRDAD
(PRED)	nada_1_g01840	TRSGS---F-	-NT-------	D---S----D	DG---FNIEA	IFSRFVRDAD
(PRED)	kaaf_1_c00820	SRSGEFKLF-	-SD-------	NGSSS----Q	DSEREFDANA	IFDSFVKDSE
(PRED)	teph_1_m00640	TRDGPIEDVL	HEI-------	ESMAN----D	AE--EFDARK	IFSDMVRGAN
(PRED)	vapo_1_1036_28	TRDGPVKDIF	ENI-------	EA-SL----D	SD--AFDAKA	IFTSFVNGAN
(PRED)	tebl_1_i01760	TRDGVLNFMT	TSTPTSSSSN	DSEHDTLSFE	ESDEDFNAKM	IISEFVRNAN
(PRED)	tode_1_d04040	NKSSGDDDD-	----------	NDDEGGFARA	SELHGFDAQK	ILAGMVSEAN
(PRED)	naca_1_e01650	TRSGRAIE--	----------	DPELQAANSD	S----FDARA	IFASFVRDAE
(PRED)	tebl_1_g02820	TRDGD---FD	DSD-------	EIFNTVTTNE	DDETGFNAQR	IIRDMVNNAN
(PRED)	lakl_1_c11616g	ARDGPL----	-QI-------	DP-------N	D----FDAAA	ILSAFVRDSE
(PRED)	saar_1_8_h03780	IQNSPM----	-GFN------	--SPD----S	ST---FDASA	IIRSFVKEAS
(PRED)	sace_14_7_g0015	VKDGDT----	-ELR------	--CPI----P	DT---FDASA	IIKSYVKEAS
(PRED)	sace_15_7_g0387	VKDGDT----	-ELR------	--CPI----P	DT---FDASA	IIKSYVKEAS
(PRED)	sace_24_8_3780	VKDGDT----	-ELR------	--CPI----P	DT---FDASA	IIKSYVKEAS
(PRED)	sace_40_8_h0383	VKDGDT----	-ELR------	--CPI----P	DT---FDASA	IIKSYVKEAS
(PRED)	sace_6_169_fm00	VKDGDT----	-ELR------	--CPI----P	DT---FDASA	IIKSYVKEAS
(PRED)	sace_19_7_3840	VKDGDT----	-ELR------	--CPI----P	DT---FDASA	IIKSYVKEAS
(PRED)	sace_32_7_3770	VKDGDT----	-ELR------	--CPI----P	DT---FDASA	IIKSYVKEAS
(PRED)	sace_56_17_q011	VKDGDT----	-ELR------	--CPI----P	DT---FDASA	IIKSYVKEAS
(PRED)	sace_5_78_bz001	VKDGDT----	-ELR------	--CPI----P	DT---FDASA	IIKSYVKEAS
(PRED)	sace_2_8_h03860	VKDGDT----	-ELR------	--CPI----P	DT---FDASA	IIKSYVKEAS
(PRED)	sace_53_29_ac00	VKDGDT----	-ELR------	--CPI----P	DT---FDASA	IIKSYVKEAS
(PRED)	sace_17_7_g0393	VKDGDT----	-ELR------	--CPI----P	DT---FDASA	IIKSYVKEAS
(PRED)	sace_25_7_g0388	VKDGDT----	-ELR------	--CPI----P	DT---FDASA	IIKSYVKEAS
(PRED)	sace_37_7_g0385	VKDGDT----	-ELR------	--CPI----P	DT---FDASA	IIKSYVKEAS
(PRED)	sace_9_7_g00180	VKDGDT----	-ELR------	--CPI----P	DT---FDASA	IIKSYVKEAS
(PRED)	sace_60_6_f0335	VKDGDT----	-ELR------	--CPI----P	DT---FDASA	IIKSYVKEAS
(PRED)	sace_59_336_lx0	VKDGDT----	-ELR------	--CPI----P	DT---FDASA	IIKSYVKEAS
(PRED)	sace_31_7_3780	VKDGDT----	-ELR------	--CPI----P	DT---FDASA	IIKSYVKEAS
(PRED)	sace_34_8_3770	VKDGDT----	-ELR------	--CPI----P	DT---FDASA	IIKSYVKEAS
(PRED)	sace_58_71_bs00	VKDGDT----	-ELR------	--CPI----P	DT---FDASA	IIKSYVKEAS
(PRED)	sace_7_7_g03880	VKDGDT----	-ELR------	--CPI----P	DT---FDASA	IIKSYVKEAS
(PRED)	sace_35_7_3840	VKDGDT----	-ELR------	--CPI----P	DT---FDASA	IIKSYVKEAS

(PRED)	sace_43_7_g0387	VKDGDT----	-ELR------	--CPI----P	DT---FDASA	IIKSYVKEAS
(PRED)	sace_57_8_h0390	VKDGDT----	-ELR------	--CPI----P	DT---FDASA	IIKSYVKEAS
(PRED)	sace_45_7_g0389	VKDGDT----	-ELR------	--CPI----P	DT---FDASA	IIKSYVKEAS
(PRED)	sace_46_8_h0391	VKDGDT----	-ELR------	--CPI----P	DT---FDASA	IIKSYVKEAS
(PRED)	sace_23_7_3860	VKDGDT----	-ELR------	--CPI----P	DT---FDASA	IIKSYVKEAS
(PRED)	sace_21_7_3790	VKDGDT----	-ELR------	--CPI----P	DT---FDASA	IIKSYVKEAS
(PRED)	sace_8_73_bu001	VKDGDT----	-ELR------	--CPI----P	DT---FDASA	IIKSYVKEAS
(PRED)	sapa_1_8_h03820	IREDDT----	-ELG------	--CPN----P	DT---FDASA	IIKSYVKEAS
(PRED)	sapa_21_8_h0387	IREDDT----	-ELG------	--CPN----P	DT---FDASA	IIKSYVKEAS
(PRED)	sapa_20_8_h0386	IREDDT----	-ELG------	--CPN----P	DT---FDASA	IIKSYVKEAS
(PRED)	sapa_22_8_h0390	IREDDT----	-ELG------	--CPN----P	DT---FDASA	IIKSYVKEAS
(PRED)	sapa_25_8_h0387	IREDDT----	-ELG------	--CPN----P	DT---FDASA	IIKSYVKEAS
(PRED)	sapa_6_8_3750	IREDDT----	-ELG------	--CPN----P	DT---FDASA	IIKSYVKEAS
(PRED)	sapa_9_8_3720	IREDDT----	-ELG------	--CPN----P	DT---FDASA	IIKSYVKEAS
(PRED)	sapa_19_8_h0390	IREDDT----	-ELG------	--CPN----P	DT---FDASA	IIKSYVKEAS
(PRED)	sapa_24_8_h0385	IREDDT----	-ELG------	--CPN----P	DT---FDASA	IIKSYVKEAS
(PRED)	sapa_4_8_h03850	IREDDT----	-ELG------	--CPN----P	DT---FDASA	IIKSYVKEAS
(PRED)	sapa_10_8_3760	IREDDT----	-ELG------	--CPN----P	DT---FDASA	IIKSYVKEAS
(PRED)	sapa_13_8_h0382	IRDDDT----	-ELG------	--CPN----P	DT---FDASA	IIKSYVKEAS
(PRED)	sapa_8_8_3750	IRDDDT----	-ELG------	--CPN----P	DT---FDASA	IIKSYVKEAS
(PRED)	sapa_11_8_h0383	IREDDT----	-ELG------	--CPN----P	DT---FDASA	IIKSYVKEAS
(PRED)	sapa_5_8_3700	IREDDT----	-ELG------	--CPN----P	DT---FDASA	IIKSYVKEAS
(PRED)	sapa_16_8_h0389	IRDNDT----	-ELG------	--CPN----P	DT---FDASA	IIKSYVKEAS
(PRED)	sapa_17_8_3730	IRDNDT----	-ELG------	--CPN----P	DT---FDASA	IIKSYVKEAS
(PRED)	sapa_2_8_h03860	IRDNDT----	-ELG------	--CPN----P	DT---FDASA	IIKSYVKEAS
(PRED)	sapa_7_8_3740	IRDNDT----	-ELG------	--CPN----P	DT---FDASA	IIKSYVKEAS
(PRED)	sapa_23_8_h0385	IRDNDT----	-ELG------	--CPN----P	DA---FDASA	IIKSYVKEAS
(PRED)	sapa_3_8_h03890	IRDNDT----	-ELG------	--CPN----P	DT---FDASA	IIKSYVKEAS
(PRED)	sapa_18_8_3730	IRDDDT----	-ELG------	--CLN----P	DT---FDASA	ITKSYVKEAS
(PRED)	sami_1_14_399	MKDDPT----	-ELD------	--SPN----S	NT---YDASA	VIKSFVKEAS
(PRED)	sace_4_8_h03690	VKDGDT----	-ELR------	--CPI----P	DT---FDASA	IIKSYVKEAS
(PRED)	saku_1_14_404	MENDPV----	-DVE------	--SPD----S	DT---FDASA	TIRSFVKEAS
(PRED)	sace_1_ynr070w	----------	----------	----------	----------	----------
(PRED)	sace_49_8_h0383	----------	----------	----------	----------	----------
(PRED)	saeu_1_2_b00130	TRDDSM----	-KLE------	--SPD----S	PT---FDASA	TIGTFVKEAS
(PRED)	sauv_1_7_3	MKNDPM----	-DLESSDPMD	LKSPG----P	PT---FDASA	IIGTYVKEAS
(PRED)	sami_1_17_26	LRDGPI----	-DWE------	--KPC----S	EA---FDASA	IIGSFVRDAS
(PRED)	zyba_1_02055_AN	LSDGPL----	----------	--EAD--TDE	DG---FNAER	ILKSFVREAH
(PRED)	zyba_1_07912	LSDGPL----	----------	--EAD--TDE	DG---FNAER	ILKSFVHEAH
(PRED)	zyba_2_2_b00600	LSDGPL----	----------	--EAD--TDE	DG---FNAER	ILKSFVHEAH
(PRED)	zyba_3_3_c03460	LSDGPL----	----------	--EAD--TDE	DG---FNAER	ILKSFVHEAH
(PRED)	zyba_1_04634	ISDELH----	----------	--ELES-SDE	TG---FNAKQ	IIHDFARDAK
(PRED)	zyba_1_06675	ISDDLR----	----------	--ELES-SDE	NG---FNAKQ	IIHDFVRDAK
(PRED)	zyba_3_2_b02230	ISDDLR----	----------	--ELES-SDE	NG---FNAKQ	IIHDFVRDAK
(PRED)	zyba_2_1_a00860	ISDDLR----	----------	--ELES-SDE	NG---FNAKQ	IIHDFVRDAK
(PRED)	zyro_1_a04114g	SADGKF----	----------	--DLSSVDDE	DG---FDAEK	ILRHFVKNAS
(PRED)	zyro_1_b14762g	LSDGSL----	----------	--DLPSGDDE	DG---FNAER	ILRHFVKDAN
(PRED)	zyba_2_14_n0149	LSNDSL----	----------	--QDG--SDE	DG---FNAEK	ILKRFVREAK
(PRED)	zyba_2_33_ag001	LSDGSL----	----------	--HGE--SDE	DG---FNAEK	ILIRFVREAK
(PRED)	lath_1_a01914g	MRNGVL----	-QVD------	---------P	DD---FDAEA	IIRAHVRGSE
(PRED)	lawa_1_23_5161	IPGNAL----	-EVD------	---------P	DN---FDAEA	IIRAHVRGSE
(PRED)	klae_1_14_n0012	TRQSGW----	----------	--------DE	EED--FNMYK	EIQGILKSSE
(PRED)	klla_1_d03432g	TTLSEW----	----------	--------DE	VDEN-FDMYR	EIQGILKASN
(PRED)	klma_1_1_a01880	TTLSEW----	----------	--------DE	VDGD-FDMYR	EIRGILQASR
(PRED)	klwi_1_33_ag001	SRNSNW----	----------	--------EE	EDEETFNMYK	VIQGIFDASK
(PRED)	teph_1_a04220	ESKGHL----	----------	----------	ELSIHKEAKT	IIKNFVKDAK
(PRED)	vapo_1_1037_47	TSSSEA----	----------	----------	DFETIKDPRK	IIEKFVRDAF
(PRED)	pata_1_2_b05590	AKDMN-----	----------	---NFVIDKN	D----FDLQE	LLALLAGKAE
(PRED)	wian_1_3_c04380	AKD-G-----	----------	---PFEIDPN	D----FDLKR	VLGAFFKSAD
(PRED)	wian_1_3_c04390	ARQ-G-----	----------	---PYEIDPN	D----FDFQR	ILQTFLKSAD
(PRED)	wian_1_7_g01010	AE--------	----------	---PFKVDPD	D----FDFKT	ILGHFLKSAD
(PRED)	bain_1_1_a00100	TTRDQP--IV	IDP-------	---------E	D----FDLQL	ILSLFVKRAS
(PRED)	bain_1_17_q0038	TTRDQP--IV	INP-------	---------E	D----FSLQF	ILSLFVKRAY
(PRED)	bain_1_8_h00410	AFKNKPGLNL	KDA-------	---------S	D----FNLDR	ILKAFINKAN
(PRED)	caal_1_19_5759	TNK--EGEFQ	LRL-------	---------D	E----FNLAK	ILANFVYFAK
(PRED)	caal_11_25_y002	TNK--EGEFQ	LRL-------	---------D	E----FNLAK	ILANFVYFAK
(PRED)	caal_4_4_d03320	TNK--EGEFQ	LRL-------	---------D	E----FNLAK	ILANFVYFAK
(PRED)	caal_12_26_z005	TNK--EGEFQ	LRL-------	---------D	E----FNLAK	ILANFVYFAK
(PRED)	caal_5_30_ad005	TNK--EGEFQ	LRL-------	---------D	E----FNLAK	ILANFVYFAK
(PRED)	caal_8_3_c03320	TNK--EGEFQ	LRL-------	---------D	E----FNLAK	ILANFVYFAK
(PRED)	caal_6_4_d03280	TNK--EGEFQ	LRL-------	---------D	E----FNLAK	ILANFVYFAK
(PRED)	caal_10_3_c0334	TNK--EGEFQ	LRL-------	---------D	E----FNLAK	ILANFVYFAK
(PRED)	caal_3_29_ac005	TNK--EGEFQ	LRL-------	---------D	E----FNLAK	ILANFVYFAK
(PRED)	caal_2_04989	TNK--EGEFQ	LRL-------	---------D	E----FNLAK	ILANFVYFAK
(PRED)	cadu_1_64350	TNK--EGEFQ	LRL-------	---------D	E----FNLAK	ILANFVYFAK

(PRED)	caor_1_h02090	TNK--EGGFT	MKM-------	---------E	E----FSLLR	ILSNFVYFAK
(PRED)	capa_1_600750	SRK--DGEFT	MKM-------	---------E	E----FSLLR	ILSNFVYFAK
(PRED)	loel_1_04930	SNK--DGGFE	LKL-------	---------D	E----FNLMR	ILSNFVYFAK
(PRED)	spar_1_5_e03260	THK--EGEFQ	LQL-------	---------D	E----FNLGK	ILANFAYFSK
(PRED)	sppa_1_7_g03160	SHK--DGEFS	LQL-------	---------D	E----FNLGK	ILANFSYFAK
(PRED)	catr_1_01205	TNK--EGNFK	LKS-------	---------D	E----FNLTK	ILSNFVYFAR
(PRED)	catr_1_05498	TNK--EGEFQ	LKL-------	---------E	E----FNLVK	ILANFVYFAK
(PRED)	catr_1_05971	TNK--EGEFQ	LNL-------	---------D	E----FNLGK	ILANFVYFAR
(PRED)	deha_1_a03696g	TTR--DGRIE	IDP-------	---------N	N----FDLHK	ILSSFIETSK
(PRED)	deha_2_5_e00720	TTR--DGRIE	IDP-------	---------N	N----FDLHK	ILSSFIETSK
(PRED)	scst_1_3_c02890	TTR--DGKIV	IDP-------	---------H	D----FDLHK	ILANFVYLAS
(PRED)	mebi_1_8_h00300	TRSVYDGDLE	VNT-------	---------D	D----FELSK	LYKTFLYHAE
(PRED)	lakl_1_h21010g	SRSEK-----	----------	---------Q	DQNLYSEYAS	ILGKRLQDAR
(PRED)	caar_1_13_m0142	AKEMSDLKMN	PD--------	----------	D----FDLGK	ILKFLAKKNE
(PRED)	caar_1_14_n0143	AKEMKEFRID	PN--------	----------	D----FDLKT	ILDFMSRKNK
(PRED)	hapo_1_1_a07220	AKQMEKLEID	PN--------	----------	D----FDLKR	ILEYMKGRSN
(PRED)	ogpa_1_1_a01680	AKQMEKLEID	PN--------	----------	D----FDLKR	ILEYMKGRSN
(PRED)	piku_1_96_cr001	AESMKSFKIN	EN--------	----------	D----FDLKA	ILEFMARGNL
(PRED)	pime_1_4_d03240	AEDMKTFKID	PN--------	----------	D----FDLQA	ILQFMAHRNE
(PRED)	pime_1_1_a12110	AKEMEDFHID	QN--------	----------	N----FDLQK	ILDFLINRNQ
(PRED)	piku_1_227_hs00	ANDMETFQMD	PA--------	----------	D----FNLDK	ILKHIVNFAQ
(PRED)	pime_1_5_e05800	ANEMETFQLD	PQ--------	----------	D----FDLQK	ILQYIVNFNA
(PRED)	pime_1_1_a07690	ANEMELFQMD	AD--------	----------	D----FDLAK	ILKYLVNFNE
(PRED)	debr_2_5_e03380	HADMQAFHID	PN--------	----------	D----FDLKS	VLKFISSRHD
(PRED)	kopa_1_2_b10040	ASEMATM-VD	LN--------	----------	D----FDLTR	ILAVFAEKAE
(PRED)	kopa_2_7_g00500	ASEMATM-VD	LN--------	----------	D----FDLTR	ILAVFAEKAE
(PRED)	asru_1_13_m0119	-VRDGPLTID	PD--------	----------	D----FDLQT	ILRTVVRISD
(PRED)	asru_1_15_o0045	NLTTNTFNID	IN--------	----------	D----FDLQK	ILRKFVRMSD
(PRED)	wian_1_1_a02920	-FKEFPMAID	PN--------	----------	D----YDLQT	ILGAFVNDTT
(PRED)	wian_1_1_a02930	-FKEFPMAID	PN--------	----------	D----FDLQT	ILGAFVNDTT

		260	270	280	290	300
(PRED)	asac_1_6_f03560	KQDIPMRQTG	VLVEGVTVQG	VDSSCVEGHD	YGDVLKLPLT	VVQSV-----
(PRED)	ergo_1_abr125c	RQDIPMRQTG	VLVEGVTVQG	VDSSCVEGHD	YGDVLALPLT	AVRAV-----
(PRED)	ercy_1_3604	RQNMEFRQAG	VLLEDVVVKG	TDSSYVEGND	YGDVLKLPLT	IAKAM-----
(PRED)	cagl_1_i04862g	EQGIHLRKAG	VTLEHVSARG	ADSTAMEGAT	FGNVLCLPYT	IYKAI-----
(PRED)	kaaf_1_c00830	EQGIHIRKAG	VTLENVSAEG	FDASALEGAT	FGNILCLPLT	IYKGI-----
(PRED)	kana_1_k01350	EQGIHIRKAG	VTLENVGCSG	FDASALEGAT	FGNILCLPYT	IYKGI-----
(PRED)	saar_1_2_b02590	EQGIHIRKAG	VTIENMGARG	VDESALEGAT	FGNILCLPLT	IIKGI-----
(PRED)	sace_1_ydr011w	EQGIHIRKAG	VTIEDVSAKG	VDASALEGAT	FGNILCLPLT	IFKGI-----
(PRED)	sace_16_1_a0238	EQGIHIRKAG	VTIEDVSAKG	VDASALEGAT	FGNILCLPLT	IFKGI-----
(PRED)	sace_45_1_a0242	EQGIHIRKAG	VTIEDVSAKG	VDASALEGAT	FGNILCLPLT	IFKGI-----
(PRED)	sace_48_1_a0238	EQGIHIRKAG	VTIEDVSAKG	VDASALEGAT	FGNILCLPLT	IFKGI-----
(PRED)	sace_60_4_d0244	EQGIHIRKAG	VTIEDVSAKG	VDASALEGAT	FGNILCLPLT	IFKGI-----
(PRED)	sace_52_1_a0240	EQGIHIRKAG	VTIEDVSAKG	VDASALEGAT	FGNILCLPLT	IFKGI-----
(PRED)	sace_46_1_a0240	EQGIHIRKAG	VTIEDVSAKG	VDASALEGAT	FGNILCLPLT	IFKGI-----
(PRED)	sace_25_1_a0240	EQGIHIRKAG	VTIEDVSAKG	VDASALEGAT	FGNILCLPLT	IFKGI-----
(PRED)	sace_24_1_2300	EQGIHIRKAG	VTIEDVSAKG	VDASALEGAT	FGNILCLPLT	IFKGI-----
(PRED)	sace_47_1_a0240	EQGIHIRKAG	VTIEDVSAKG	VDASALEGAT	FGNILCLPLT	IFKGI-----
(PRED)	sace_7_1_a02410	EQGIHIRKAG	VTIEDVSAKG	VDASALEGAT	FGNILCLPLT	IFKGI-----
(PRED)	sace_59_110_df0	EQGIHIRKAG	VTIEDVSAKG	VDASALEGAT	FGNILCLPLT	IFKGI-----
(PRED)	sace_56_1_a0202	EQGIHIRKAG	VTIEDVSAKG	VDASALEGAT	FGNILCLPLT	IFKGI-----
(PRED)	sace_40_1_a0239	EQGIHIRKAG	VTIEDVSAKG	VDASALEGAT	FGNILCLPLT	IFKGI-----
(PRED)	sace_15_1_a0242	EQGIHIRKAG	VTIEDVSAKG	VDASALEGAT	FGNILCLPLT	IFKGI-----
(PRED)	sace_37_1_a0243	EQGIHIRKAG	VTIEDVSAKG	VDASALEGAT	FGNILCLPLT	IFKGI-----
(PRED)	sace_9_1_a02440	EQGIHIRKAG	VTIEDVSAKG	VDASALEGAT	FGNILCLPLT	IFKGI-----
(PRED)	sace_22_1_2300	EQGIHIRKAG	VTIEDVSAKG	VDASALEGAT	FGNILCLPLT	IFKGI-----
(PRED)	sace_29_1_2290	EQGIHIRKAG	VTIEDVSAKG	VDASALEGAT	FGNILCLPLT	IFKGI-----
(PRED)	sace_34_1_2320	EQGIHIRKAG	VTIEDVSAKG	VDASALEGAT	FGNILCLPLT	IFKGI-----
(PRED)	sace_58_25_y007	EQGIHIRKAG	VTIEDVSAKG	VDASALEGAT	FGNILCLPLT	IFKGI-----
(PRED)	sace_23_1_2290	EQGIHIRKAG	VTIEDVSAKG	VDASALEGAT	FGNILCLPLT	IFKGI-----
(PRED)	sace_6_120_dp00	EQGIHIRKAG	VTIEDVSAKG	VDASALEGAT	FGNILCLPLT	IFKGI-----
(PRED)	sace_57_1_a0241	EQSIHIRKAG	VTIEDVSAKG	VDASALEGAT	FGNILCLPLT	IFKGI-----
(PRED)	sace_17_1_a0241	EQGIHIRKAG	VTIEDVSAKG	VDASALEGAT	FGNILCLPLT	IFKGI-----
(PRED)	sace_21_1_2310	EQGIHIRKAG	VTIEDVSAKG	VDASALEGAT	FGNILCLPLT	IFKGI-----
(PRED)	sace_49_1_a0246	EQGIHIRKAG	VTIEDVSAKG	VDASALEGAT	FGNILCLPLT	IFKGI-----
(PRED)	sace_8_2_b02430	EQGIHIRKAG	VTIEDVSAKG	VDASALEGAT	FGNILCLPLT	IFKGI-----
(PRED)	sace_31_1_2300	EQGIHIRKAG	VTIEDVSAKG	VDASALEGAT	FGNILCLPLT	IFKGI-----
(PRED)	sace_50_1_a0241	EQGIHIRKAG	VTIEDVSAKG	VDASALEGAT	FGNILCLPLT	IFKGI-----
(PRED)	sace_4_1_a02360	EQSIHIRKAG	VTIEDVSAKG	VDASALEGAT	FGNILCLPLT	IFKGI-----
(PRED)	sace_2_1_a02390	EQGIHIRKAG	VTIEDVSAKG	VDASALEGAT	FGNILCLPLT	IFKGI-----
(PRED)	sace_5_33_ag005	EXXIHIRKAG	VTIEDVSAKG	VDASALXGAT	FGNILCLPLT	IFKGI-----
(PRED)	sapa_11_1_a0247	EQGIHIRKAG	VTIENVSAKG	VDASALEGAT	FGNILCLPLT	IFKGI-----
(PRED)	sapa_25_1_a0246	EQGIHIRKAG	VTIENVSAKG	VDASALEGAT	FGNILCLPLT	IFKGI-----
(PRED)	sapa_4_1_a02470	EQGIHIRKAG	VTIENVSAKG	VDASALEGAT	FGNILCLPLT	IFKGI-----

(PRED)	sapa_5_1_2350	EQGIHIRKAG	VTIENVSAKG	VDASALEGAT	FGNILCLPLT	IFKGI-----
(PRED)	sapa_9_1_2360	EQGIHIRKAG	VTIENVSAKG	VDASALEGAT	FGNILCLPLT	IFKGI-----
(PRED)	sapa_14_1_a0244	EQGIHIRKAG	VTIENVSAKG	VDASALEGAT	FGNILCLPLT	IFKGI-----
(PRED)	sapa_8_1_2350	EQGIHIRKAG	VTIENVSAKG	VDASALEGAT	FGNILCLPLT	IFKGI-----
(PRED)	sapa_17_1_2380	EQGIHIRKAG	VTIENVSAKG	VDASALEGAT	FGNILCLPLT	IFKGI-----
(PRED)	sapa_7_1_2370	EQGIHIRKAG	VTIENVSAKG	VDASALEGAT	FGNILCLPLT	IFKGI-----
(PRED)	sapa_2_1_a02460	EQGIHIRKAG	VTIENVSAKG	VDASALEGAT	FGNILCLPLT	IFKGI-----
(PRED)	sapa_23_1_a0248	EQGIHIRKAG	VTIENVSAKG	VDASALEGAT	FGNILCLPLT	IFKGI-----
(PRED)	sapa_3_1_a02470	EQGIHIRKAG	VTIENVSAKG	VDASALEGAT	FGNILCLPLT	IFKGI-----
(PRED)	sapa_18_1_2390	EQGIHIRKAG	VTIENVSAKG	VDASALEGAT	FGNILCLPLT	IFKGI-----
(PRED)	sami_1_4_244	EQGIHIRKAG	VTIENVSAKG	VDASALEGAT	FGNILCLPLT	IFKGI-----
(PRED)	saku_1_4_262	EQGIHIRKAG	VTIENISAKG	VDASALEGAT	FGNILCLPLS	IFRGI-----
(PRED)	saba_1_58_bf002	EQGIHIRKAG	VTIENVGAQG	SDESALEGAT	FGNILCLPLT	IIKGI-----
(PRED)	saeu_1_4_d02400	EQGIHIRKAG	VTIEDVGAQG	FDESALEGAT	FGNILCLPLT	IVKGI-----
(PRED)	naca_1_e01640	EQGIHIRKAG	VTLEDVGAEG	TDASALEGAT	FGNILCLPYT	IYKGI-----
(PRED)	nada_1_g01850	EQGIHIRKSG	VTLEDVSAEG	LDSSALEGAT	FGNILCLPLT	IYKGI-----
(PRED)	naca_1_e01630	EQGIHIRKAG	VTMEDVTAEG	VDASALEGAT	FGNVLCLPYT	IYKGI-----
(PRED)	nada_1_g01840	EQGIHLRKSG	VTLEGVGAQG	LDTSALEGST	FGSFLMLPYT	IYKKF-----
(PRED)	kaaf_1_c00820	EQGIHIRRAT	VTIENLSVQG	FDKSSMEGKT	FGNFLLLPLT	IYRAI-----
(PRED)	teph_1_m00640	EQGIHIRKAG	VIMEKVSAEG	VDASTVEGTT	FGDILCLPLT	IYKGI-----
(PRED)	vapo_1_1036_28	EQGIHLRKAG	VILEDISAEG	TDASALEGQT	FGDILSLPYT	IYKAI-----
(PRED)	tebl_1_i01760	EQGIHLRKAG	VILEDVGAKG	IDSSAANATT	VMDIILLPYT	IFNFV-----
(PRED)	tode_1_d04040	DQGIHSRETG	VIMEEVGAEG	VDESALEGAT	FGNILCLPVT	IYKGI-----
(PRED)	naca_1_e01650	EQGIHVRKAG	VTLEDVSAEG	INASMSEGRT	VGDLLAFPYT	IHKYI-----
(PRED)	tebl_1_g02820	EQGIHLRKSG	VTLEEVCADG	IDASALEGQT	VGDILTLPYT	IYKGI-----
(PRED)	lakl_1_c11616g	EQGIHIRKSG	VIAEDVSVTG	VDARFLEGQT	FGDILMLPRT	VFRGI-----
(PRED)	saar_1_8_h03780	EQGIHLRKSG	VTMEYVSVEG	LDSTFLEGRT	FGDILCLPWT	IIKRI-----
(PRED)	sace_14_7_g0015	EQGIHLRKAG	VAMECVSVEG	LDSSFLEGQT	FGDILCLPWT	IIKGI-----
(PRED)	sace_15_7_g0387	EQGIHLRKAG	VAMECVSVEG	LDSSFLEGQT	FGDILCLPWT	IIKGI-----
(PRED)	sace_24_8_3780	EQGIHLRKAG	VAMECVSVEG	LDSSFLEGQT	FGDILCLPWT	IIKGI-----
(PRED)	sace_40_8_h0383	EQGIHLRKAG	VAMECVSVEG	LDSSFLEGQT	FGDILCLPWT	IIKGI-----
(PRED)	sace_6_169_fm00	EQGIHLRKAG	VAMECVSVEG	LDSSFLEGQT	FGDILCLPWT	IIKGI-----
(PRED)	sace_19_7_3840	EQGIHLRKAG	VAMECVSVEG	LDSSFLEGQT	FGDILCLPWT	IIKGI-----
(PRED)	sace_32_7_3770	EQGIHLRKAG	VAMECVSVEG	LDSSFLEGQT	FGDILCLPWT	IIKGI-----
(PRED)	sace_56_17_q011	EQGIHLRKAG	VAMECVSVEG	LDSSFLEGQT	FGDILCLPWT	IIKGI-----
(PRED)	sace_5_78_bz001	EQGIHLRKAG	VAMECVSVEG	LDSSFLEGQT	FGDILCLPWT	IIKGI-----
(PRED)	sace_2_8_h03860	EQGIHLRKAG	VAMECVSVEG	LDSSFLEGQT	FGDILCLPWT	IIKGI-----
(PRED)	sace_53_29_ac00	EQGIHLRKAG	VAMECVSVEG	LDSSFLEGQT	FGDILCLPWT	IIKGI-----
(PRED)	sace_17_7_g0393	EQGIHLRKAG	VAMECVSVEG	LDSSFLEGQT	FGDILCLPWT	IIKGI-----
(PRED)	sace_25_7_g0388	EQGIHLRKAG	VAMECVSVEG	LDSSFLEGQT	FGDILCLPWT	IIKGI-----
(PRED)	sace_37_7_g0385	EQGIHLRKAG	VAMECVSVEG	LDSSFLEGQT	FGDILCLPWT	IIKGI-----
(PRED)	sace_9_7_g00180	EQGIHLRKAG	VAMECVSVEG	LDSSFLEGQT	FGDILCLPWT	IIKGI-----
(PRED)	sace_60_6_f0335	EQGIHLRKAG	VAMECVSVEG	LDSSFLEGQT	FGDILCLPWT	IIKGI-----
(PRED)	sace_59_336_lx0	EQGIHLRKAG	VAMECVSVEG	LDSSFLEGQT	FGDILCLPWT	IIKGI-----
(PRED)	sace_31_7_3780	EQGIHLRKAG	VAMECVSVEG	LDSSFLEGQT	FGDILCLPWT	IIKGI-----
(PRED)	sace_34_8_3770	EQGIHLRKAG	VAMECVSVEG	LDSSFLEGQT	FGDILCLPWT	IIKGI-----
(PRED)	sace_58_71_bs00	EQGIHLRKAG	VAMECVSVEG	LDSSFLEGQT	FGDILCLPWT	IIKGI-----
(PRED)	sace_7_7_g03880	EQGIHLRKAG	VAMECVSVEG	LDSSFLEGQT	FGDILCLPWT	IIKGI-----
(PRED)	sace_35_7_3840	EQGIHLRKAG	VAMECVSVEG	LDSSFLEGQT	FGDILCLPWT	IIKGI-----
(PRED)	sace_43_7_g0387	EQGIHLRKAG	VAMECVSVEG	LDSSFLEGQT	FGDILCLPWT	IIKGI-----
(PRED)	sace_57_8_h0390	EQGIHLRKAG	VAMECVSVEG	LDSSFLEGQT	FGDILCLPWT	IIKGI-----
(PRED)	sace_45_7_g0389	EQGIHLRKAG	VAMECVSVEG	LDSSFLEGQT	YGDILCLPWT	IIKGI-----
(PRED)	sace_46_8_h0391	EQGIHLRKAG	VAMECVSVEG	LDSSFLEGQT	FGDILCLPWT	IIKGI-----
(PRED)	sace_23_7_3860	EQGIHLRKAG	VAMECVSVEG	LDSSFLEGQT	FGDILCLPWT	IIKGI-----
(PRED)	sace_21_7_3790	EQGIHLRKAG	VAMECVSVEG	LDSSFLEGQT	FGDILCLPWT	IIKGI-----
(PRED)	sace_8_73_bu001	EQGIHLRKAG	VAMECVSVEG	LDSSFLEGQT	FGDILCLPWT	IIKGI-----
(PRED)	sapa_1_8_h03820	EQGIHLRKAG	VAMECVSVEG	LDSSFLEGQT	FGDILCLPWT	IIKGI-----
(PRED)	sapa_21_8_h0387	EQGIHLRKAG	VAMECVSVEG	LDSSFLEGQT	FGDILCLPWT	IIKGI-----
(PRED)	sapa_20_8_h0386	EQGIHLRKAG	VAMECVSVEG	LDSSFLEGQT	FGDILCLPWT	IIKGI-----
(PRED)	sapa_22_8_h0390	EQGIHLRKAG	VAMECVSVEG	LDSSFLEGQT	FGDILCLPWT	IIKGI-----
(PRED)	sapa_25_8_h0387	EQGIHLRKAG	VAMECVSVEG	LDSSFLEGQT	FGDILCLPWT	IIKGI-----
(PRED)	sapa_6_8_3750	EQGIHLRKAG	VAMECVSVEG	LDSSFLEGQT	FGDILCLPWT	IIKGI-----
(PRED)	sapa_9_8_3720	EQGIHLRKAG	VAMECVSVEG	LDSSFLEGQT	FGDILCLPWT	IIKGI-----
(PRED)	sapa_19_8_h0390	EQGIHLRKAG	VAMECVSVEG	LDSSFLEGQT	FGDILCLPWT	IIKGI-----
(PRED)	sapa_24_8_h0385	EQGIHLRKAG	VAMECVSVEG	LDSSFLEGQT	FGDILCLPWT	IIKGI-----
(PRED)	sapa_4_8_h03850	EQGIHLRKAG	VAMECVSVEG	LDSSFLEGQT	FGDILCLPWT	IIKGI-----
(PRED)	sapa_10_8_3760	EQGIHLRKAG	VAMECVSVEG	LDSSFLEGQT	FGDILCLPWT	IIKGI-----
(PRED)	sapa_13_8_h0382	EQGIHLRKAG	VAMECVSVEG	LDSSFLEGQT	FGDILCLPWT	IIKGI-----
(PRED)	sapa_8_8_3750	EQGIHLRKAG	VAMECVSVEG	LDSSFLEGQT	FGDILCLPWT	IIKGI-----
(PRED)	sapa_11_8_h0383	EQGIHLRKAG	VAMECVSVEG	LDSSFLEGQT	FGDILCLPWT	IIKGI-----
(PRED)	sapa_5_8_3700	EQGIHLRKAG	VAMECVSVEG	LDSSFLEGQT	FGDILCLPWT	IIKGI-----
(PRED)	sapa_16_8_h0389	EQGIYLRKAG	VAMECVSVEG	LDSSFLEGQT	FGDILCLPWT	IIKGI-----
(PRED)	sapa_17_8_3730	EQGIYLRKAG	VAMECVSVEG	LDSSFLEGQT	FGDILCLPWT	IIKGI-----
(PRED)	sapa_2_8_h03860	EQGIYLRKAG	VAMECVSVEG	LDSSFLEGQT	FGDILCLPWT	IIKGI-----
(PRED)	sapa_7_8_3740	EQGIYLRKAG	VAMECVSVEG	LDSSFLEGQT	FGDILCLPWT	IIKGI-----

(PRED)	sapa_23_8_h0385	EQGIYLRKAG	VAMECVSVEG	LDSSFLEGQT	FGDILCLPWT	IIKGI-----
(PRED)	sapa_3_8_h03890	EQGIYLRKAG	VAMECVSVEG	LDSSFLEGQT	FGDILCLPWT	IIKGI-----
(PRED)	sapa_18_8_3730	EQGIYLCKAG	VAMECVSVEG	LDSSFLEGQT	FGDILCLPWT	IIKGI-----
(PRED)	sami_1_14_399	EQGIHLRKAG	VTMECVSVEG	LDSRFLEGQT	FGDILCLPWT	IIKGV-----
(PRED)	sace_4_8_h03690	EQGIHLRKAG	VAMECVSVEG	LDSSFFGGPN	LWRYFVFTRT	IIKGI-----
(PRED)	saku_1_14_404	EQGIHLRKAG	VSMECVSVEG	IDSSLLEGQT	FGDILCLPWT	IVKGI-----
(PRED)	sace_1_ynr070w	----------	--MECVSVEG	LDSSFLEGQT	FGDILCLPWT	IIKGI-----
(PRED)	sace_49_8_h0383	----------	--MECVSVEG	LDSSFLEGQT	FGDILCLPWT	IIKGI-----
(PRED)	saeu_1_2_b00130	EQGIHLRKAG	VTMENISAEG	LDSTFLEGKT	FGDILCLPWT	IIKGI-----
(PRED)	sauv_1_7_3	EQGIHLRKAG	VTMECVSAEG	LDSTFLEGKT	FGDILCLPWT	IIKGI-----
(PRED)	sami_1_17_26	EQGIHLRKAG	VTMESVSAEG	FDSTFLEGQT	FGDILCLPWT	IIKGI-----
(PRED)	zyba_1_02055_AN	QQGIDFRKTG	VVMEDVTASG	TDSSALEGHT	FGDLLCLPLT	VYRAV-----
(PRED)	zyba_1_07912	QQGIDFRKTG	VVMEDVTASG	TDSSALEGHT	FGDLLCLPLT	VYRAV-----
(PRED)	zyba_2_2_b00600	QQGIDFRKTG	VVMEDVTASG	TDSSALEGHT	FGDLLCLPLT	IYRAV-----
(PRED)	zyba_3_3_c03460	QQGIDFRKTG	VVMEDVTASG	TDSSALEGHT	FGDLLCLPLT	VYRAV-----
(PRED)	zyba_1_04634	QQGIEFRKTG	VVMEDVTASG	TDRGALEGST	FGDILCLPLT	IYRAV-----
(PRED)	zyba_1_06675	QQGIEFRKTG	VVMEDVTASG	TDRGALEGTT	FGDILCLPLT	IYRAI-----
(PRED)	zyba_3_2_b02230	QQGIEFRKTG	VVMEDVTASG	TDRGALEGTT	FGDILCLPLT	IYRAI-----
(PRED)	zyba_2_1_a00860	QQGIEFRKTG	VVMEDVTASG	TDRGALEGTT	FGDILCLPLT	IYRAI-----
(PRED)	zyro_1_a04114g	SQGIDFRKTG	ITMEDVSATG	LDNSFLEGST	FADIALLPYT	IFKFL-----
(PRED)	zyro_1_b14762g	SQNIDFRKTG	VTMEDVSATG	IDTTSLEGST	FADLVLLPYT	IVRAI-----
(PRED)	zyba_2_14_n0149	EQGIDVRKTG	VLLENVSATG	VDSSEVEGFS	FTDILFLPLT	IYNGI-----
(PRED)	zyba_2_33_ag001	EQGIDVRKTG	VLLENVSATG	VDSSEVEGFS	FTDIVLLPLT	IYNGI-----
(PRED)	lath_1_a01914g	EQGIHLRRAG	VIGENFTVVG	KDCSSVEAQT	FEDILLLPRT	IFRGI-----
(PRED)	lawa_1_23_5161	SQGIHLRKAG	VIAENFTVVG	KDCSSAEAQT	FEDILLLPRT	IFRGV-----
(PRED)	klae_1_14_n0012	EEGIHLRQSG	LTARNVVVKG	IDAQFLEGTD	YSNLLLLPKT	IFEAI-----
(PRED)	klla_1_d03432g	DGGIHLRKAG	ITARNVAVKG	VDAQFLEGAT	YGDMLMLPAT	IFKGI-----
(PRED)	klma_1_1_a01880	DEGIHLRKSG	ITARGVKVKG	VDAQFLEGST	YGDMMMLPVT	IYKGI-----
(PRED)	klwi_1_33_ag001	DQGIHLRQSG	LSARNVAVKG	VDAQFLEGSN	YGDILMMPVT	IVKGI-----
(PRED)	teph_1_a04220	EQGMHIREAG	VIIKDLSMEV	ADRSTKEGKT	FADVLLFPLT	LFREL-----
(PRED)	vapo_1_1037_47	EQGIHIRNTS	IIMEDITVQV	PDTSRQEAST	FGDFILLPIT	IYKMI-----
(PRED)	pata_1_2_b05590	EQGIQFKEIG	VEMSDVSVLG	VDDSTSVVVT	AGDIAMGPFL	GLQKL-----
(PRED)	wian_1_3_c04380	EQGIHLRSTG	VVLKDVTTIG	VDAASSFAPT	VGDLALFPIT	MFQKL-----
(PRED)	wian_1_3_c04390	EQGIHLRSTG	VVFKDVTTTG	IDATASYAPT	VGDLAMGPVS	IFRQI-----
(PRED)	wian_1_7_g01010	EQGIHLRSTG	VVMKDVTTTG	IDASSSFAPT	VKDLALGPLT	LLRQF-----
(PRED)	bain_1_1_a00100	AQGIQLRSSS	VAFTDVAAYG	IDESVSFAPT	VGNILNLP-G	TIIEQ-----
(PRED)	bain_1_17_q0038	SQGIQLRSSS	VAFTDVAAYG	IDESVSFAST	VGNILNLP-S	TIIEQ-----
(PRED)	bain_1_8_h00410	DQGILLRSSG	VTIEGLSTLG	VDGSATFALT	LGDCINFP-R	TLGEL-----
(PRED)	caal_1_19_5759	KQGIVLRKSG	ITFQDLCVYG	VDESFAIAPT	VTDLLKGPVG	AVQAI-----
(PRED)	caal_11_25_y002	KQGIVLRKSG	ITFQDLCVYG	VDESFAIAPT	VTDLLKGPVG	AVQAI-----
(PRED)	caal_4_4_d03320	KQGIVLRKSG	ITFQDLCVYG	VDESFAIAPT	VTDLLKGPVG	AVQAI-----
(PRED)	caal_12_26_z005	KQGIVLRKSG	ITFQDLCVYG	VDESFAIAPT	VTDLLKGPVG	AVQAI-----
(PRED)	caal_5_30_ad005	KQGIVLRKSG	ITFQDLCVYG	VDESFAIAPT	VTDLLKGPVG	AVQAI-----
(PRED)	caal_8_3_c03320	KQGIVLRKSG	ITFQDLCVYG	VDESFAIAPT	VTDLLKGPVG	AVQAI-----
(PRED)	caal_6_4_d03280	KQGIVLRKSG	ITFQDLCVYG	VDESFAIAPT	VTDLLKGPVG	AVQAI-----
(PRED)	caal_10_3_c0334	KQGIVLRKSG	ITFQDLCVYG	VDESFAIAPT	VTDLLKGPVG	AVQAI-----
(PRED)	caal_3_29_ac005	KQGIVLRKSG	ITFQDLCVYG	VDESFAIAPT	VTDLLKGPVG	AVQAI-----
(PRED)	caal_2_04989	KQGIVLRKSG	ITFQDLCVYG	VDESFAIAPT	VTDLLKGPVG	AVQAI-----
(PRED)	cadu_1_64350	KQGIVLRKSG	ITFQDLCVYG	VDESFAIAPT	VTDLLKGPIG	GIQAI-----
(PRED)	caor_1_h02090	KQGLAMRCSG	VSFQDLCVYG	NDESFAIVPT	VLDLLKGPIG	GIQQA-----
(PRED)	capa_1_600750	KQGLAMRSSG	ISFQDLCVYG	NDESFAIVPT	VLDLLKGPIG	GVQQA-----
(PRED)	loel_1_04930	KQGIVLRSSG	ITFKDLCTYG	TDESFAVVPT	CLDLLKGPIG	GIQQI-----
(PRED)	spar_1_5_e03260	KQGIKMRSSG	ITFRDLNVYG	IDESFAVVPT	ALDILKGPVG	AVQGI-----
(PRED)	sppa_1_7_g03160	KQGIKMRSSG	ITIRDLNVYG	IDESFAVVPT	ALDILKGPVG	AVQGI-----
(PRED)	catr_1_01205	KQGIVLRKSG	ITFQDLSVYG	VDDSVAIVPT	VMDLIKGPIN	GIIQT-----
(PRED)	catr_1_05498	KQGINLRKSG	VTFKDLSVFG	VDDSVAVVPT	VLDVLKGPVY	GIQEL-----
(PRED)	catr_1_05971	KQGIVLRKSG	ITFKDLCVYG	VDDSVAIVPT	VMDILKGPVA	GISAA-----
(PRED)	deha_1_a03696g	SQGIKLRNSG	VSFKDLTVYG	VDESFSVAVT	VYDLMKGPIG	GIQRA-----
(PRED)	deha_2_5_e00720	SQGIKLRKSG	VSFKDLTVYG	VDESFSVAVT	VYDLMKGPIG	GIQRA-----
(PRED)	scst_1_3_c02890	NQGIRLRQSG	ISFKDLSVFG	VDESFAVVPT	VSELAKGPVG	AIQAA-----
(PRED)	mebi_1_8_h00300	QEGLILRELG	VSFENLSVYG	KDQTYTYLQT	MSDVLKGPYG	AIRSA-----
(PRED)	lakl_1_h21010g	ANGVKFRNCG	VTGENVTIKG	FDSSFQFQST	VQDIVFFPLT	IYRAV-----
(PRED)	caar_1_13_m0142	EQGIPARSTD	VIFENFTVIG	NNMAASIVPT	VADIIFAPYF	LIKN------
(PRED)	caar_1_14_n0143	EQGMVSISTD	VVFENLEIIG	KNMSASIVPT	VGDVFFHPII	KLID------
(PRED)	hapo_1_1_a07220	EQGIGGRTTD	LIFEDLEVVG	KNTTVSIVPT	AGDIFFGPIL	KLAD------
(PRED)	ogpa_1_1_a01680	EQGIGGRTTD	LIFEDLEVVG	KNTTVSIVPT	AGDVFFGPIL	KLVD------
(PRED)	piku_1_96_cr001	RNGIQKKNCE	VVFKDLTITG	KNTSASVVKD	VSDVFFPFYS	LIRN------
(PRED)	pime_1_4_d03240	EQGIKVKTAE	IIFKNMSVTG	KNTSASIVKD	VGDIFFPVYS	YFRD------
(PRED)	pime_1_1_a12110	ENGIKTKKAE	VVFRNLTVTG	KNTSASVLKD	VSDIFVPFYS	IIKA------
(PRED)	piku_1_227_hs00	ENNTSKPQLE	VVFKDLTVLG	RNTSASVLLD	VSDVFFKPIQ	VLYD------
(PRED)	pime_1_5_e05800	KSNVPKPQLE	VVFRDLTVVG	KNASASVLKD	VS-IFFFPIV	YLVD------
(PRED)	pime_1_1_a07690	NNGYENNEVD	VVFKDLTVIG	KNNAASVLRD	VSDVFFKPFV	LLYN------
(PRED)	debr_2_5_e03380	ELGVPEKKTW	VVYDELSVMG	KDSSTATVQD	VGSLLFFWVD	WVRRLGGVGR
(PRED)	kopa_1_2_b10040	QRGLPIKSTA	VELKDVSVLG	VNDSASLLPT	VSDLLYLPST	IARK------
(PRED)	kopa_2_7_g00500	QRGLPIKSTA	VELKDVNVLG	VNDSASLLPT	VQDLLYLPST	IARK------

```
(PRED) asru_1_13_m0119 KDGIHLRQAG VRFKNVTTTG IDALTSSAET ALDLLTAPFT IHK-------
(PRED) asru_1_15_o0045 VEDIHIKKAG IIFKNVNVYG QNEASSFSPD ILSFLNIYQN I-K-------
(PRED) wian_1_1_a02920 ERGLKLRTAG ITFNNVSTIG KDNSVAFAPT LADLASAPLN LVK-------
(PRED) wian_1_1_a02930 ERGLKLRTAG ITFNNVSTIG KDNSVAFAPT LADLASAPLD LFK-------

                       .........310 .........320 .........330 .........340 .........350
(PRED) asac_1_6_f03560 ---------- ------RGAM -RR-RY---- ---------- ----------
(PRED) ergo_1_abr125c  ---------- ------RQAM -RR-RY---- ---------- ----------
(PRED) ercy_1_3604     ---------- ------RARK -NGVRM---- ---------- ----------
(PRED) cagl_1_i04862g  ---------- ------RDKS -GS-KM---- ---------- ----------
(PRED) kaaf_1_c00830   ---------- ------KSKK -GN-KM---- ---------- ----------
(PRED) kana_1_k01350   ---------- ------KAKR -QA-KM---- ---------- ----------
(PRED) saar_1_2_b02590 ---------- ------KAKR -RQ-KM---- ---------- ----------
(PRED) sace_1_ydr011w  ---------- ------KAKR -HQ-KM---- ---------- ----------
(PRED) sace_16_1_a0238 ---------- ------KAKR -HQ-KM---- ---------- ----------
(PRED) sace_45_1_a0242 ---------- ------KAKR -HQ-KM---- ---------- ----------
(PRED) sace_48_1_a0238 ---------- ------KAKR -HQ-KM---- ---------- ----------
(PRED) sace_60_4_d0244 ---------- ------KAKR -HQ-KM---- ---------- ----------
(PRED) sace_52_1_a0240 ---------- ------KAKR -HQ-KM---- ---------- ----------
(PRED) sace_46_1_a0240 ---------- ------KAKR -HQ-KM---- ---------- ----------
(PRED) sace_25_1_a0240 ---------- ------KAKR -HQ-KM---- ---------- ----------
(PRED) sace_24_1_2300  ---------- ------KAKR -HQ-KM---- ---------- ----------
(PRED) sace_47_1_a0240 ---------- ------KAKR -HQ-KM---- ---------- ----------
(PRED) sace_7_1_a02410 ---------- ------KAKR -HQ-KM---- ---------- ----------
(PRED) sace_59_110_df0 ---------- ------KAKR -HQ-KM---- ---------- ----------
(PRED) sace_56_1_a0202 ---------- ------KAKR -HQ-KM---- ---------- ----------
(PRED) sace_40_1_a0239 ---------- ------KAKR -HQ-KM---- ---------- ----------
(PRED) sace_15_1_a0242 ---------- ------KAKR -HQ-KM---- ---------- ----------
(PRED) sace_37_1_a0243 ---------- ------KAKR -HQ-KM---- ---------- ----------
(PRED) sace_9_1_a02440 ---------- ------KAKR -HQ-KM---- ---------- ----------
(PRED) sace_22_1_2300  ---------- ------KAKR -HQ-KM---- ---------- ----------
(PRED) sace_29_1_2290  ---------- ------KAKR -HQ-KM---- ---------- ----------
(PRED) sace_34_1_2320  ---------- ------KAKR -HQ-KM---- ---------- ----------
(PRED) sace_58_25_y007 ---------- ------KAKR -HQ-KM---- ---------- ----------
(PRED) sace_23_1_2290  ---------- ------KAKR -HQ-KM---- ---------- ----------
(PRED) sace_6_120_dp00 ---------- ------KAKR -HQ-KM---- ---------- ----------
(PRED) sace_57_1_a0241 ---------- ------KAKR -HQ-KM---- ---------- ----------
(PRED) sace_17_1_a0241 ---------- ------KAKR -HQ-KM---- ---------- ----------
(PRED) sace_21_1_2310  ---------- ------KAKR -HQ-KM---- ---------- ----------
(PRED) sace_49_1_a0246 ---------- ------KAKR -HQ-KM---- ---------- ----------
(PRED) sace_8_2_b02430 ---------- ------KAKR -HQ-KM---- ---------- ----------
(PRED) sace_31_1_2300  ---------- ------KAKR -HQ-KM---- ---------- ----------
(PRED) sace_50_1_a0241 ---------- ------KAKR -HQ-KM---- ---------- ----------
(PRED) sace_4_1_a02360 ---------- ------KAKR -HQ-KM---- ---------- ----------
(PRED) sace_2_1_a02390 ---------- ------KAKR -HQ-KM---- ---------- ----------
(PRED) sace_5_33_ag005 ---------- ------KAKR -HQ-KM---- ---------- ----------
(PRED) sapa_11_1_a0247 ---------- ------KAKR -HQ-KM---- ---------- ----------
(PRED) sapa_25_1_a0246 ---------- ------KAKR -HQ-KM---- ---------- ----------
(PRED) sapa_4_1_a02470 ---------- ------KAKR -HQ-KM---- ---------- ----------
(PRED) sapa_5_1_2350   ---------- ------KAKR -HQ-KM---- ---------- ----------
(PRED) sapa_9_1_2360   ---------- ------KAKR -HQ-KM---- ---------- ----------
(PRED) sapa_14_1_a0244 ---------- ------KAKR -HQ-KM---- ---------- ----------
(PRED) sapa_8_1_2350   ---------- ------KAKR -HQ-KM---- ---------- ----------
(PRED) sapa_17_1_2380  ---------- ------KAKR -HQ-KM---- ---------- ----------
(PRED) sapa_7_1_2370   ---------- ------KAKR -HQ-KM---- ---------- ----------
(PRED) sapa_2_1_a02460 ---------- ------KAKR -HQ-KM---- ---------- ----------
(PRED) sapa_23_1_a0248 ---------- ------KAKR -HQ-KM---- ---------- ----------
(PRED) sapa_3_1_a02470 ---------- ------KAKR -HQ-KM---- ---------- ----------
(PRED) sapa_18_1_2390  ---------- ------KAKR -HQ-KM---- ---------- ----------
(PRED) sami_1_4_244    ---------- ------KAKR -HQ-KM---- ---------- ----------
(PRED) saku_1_4_262    ---------- ------KAKK -HQ-KM---- ---------- ----------
(PRED) saba_1_58_bf002 ---------- ------KAKK -QK-KM---- ---------- ----------
(PRED) saeu_1_4_d02400 ---------- ------KAKR -HK-KM---- ---------- ----------
(PRED) naca_1_e01640   ---------- ------KARK -SM-KM---- ---------- ----------
(PRED) nada_1_g01850   ---------- ------KARK -NM-KM---- ---------- ----------
(PRED) naca_1_e01630   ---------- ------RARR -NR-KW---- ---------- ----------
(PRED) nada_1_g01840   ---------- ------KAKK -RM-KM---- ---------- ----------
(PRED) kaaf_1_c00820   ---------- ------KNKR -RN-RM---- ---------- ----------
(PRED) teph_1_m00640   ---------- ------RAAK -QR-KM---- ---------- ----------
(PRED) vapo_1_1036_28  ---------- ------KTKR -QS-KM---- ---------- ----------
(PRED) tebl_1_i01760   ---------- ------KNLR -NR-RM---- ---------- ----------
(PRED) tode_1_d04040   ---------- ------KAKK -NS-KM---- ---------- ----------
(PRED) naca_1_e01650   ---------- ------RSMS -NT-KM---- ---------- ----------
(PRED) tebl_1_g02820   ---------- ------RNSK -NK-KI---- ---------- ----------
(PRED) lakl_1_c11616g  ---------- ------RASR -HK-KM---- ---------- ----------
```

```
(PRED) saar_1_8_h03780 ---------- ------RESK -NRKKM---- ---------- ----------
(PRED) sace_14_7_g0015 ---------- ------RERK -NRNKM---- ---------- ----------
(PRED) sace_15_7_g0387 ---------- ------RERK -NRNKM---- ---------- ----------
(PRED) sace_24_8_3780  ---------- ------RERK -NRNKM---- ---------- ----------
(PRED) sace_40_8_h0383 ---------- ------RERK -NRNKM---- ---------- ----------
(PRED) sace_6_169_fm00 ---------- ------RERK -NRNKM---- ---------- ----------
(PRED) sace_19_7_3840  ---------- ------RERK -NRNKM---- ---------- ----------
(PRED) sace_32_7_3770  ---------- ------RERK -NRNKM---- ---------- ----------
(PRED) sace_56_17_q011 ---------- ------RERK -NRNKM---- ---------- ----------
(PRED) sace_5_78_bz001 ---------- ------RERK -NRNKM---- ---------- ----------
(PRED) sace_2_8_h03860 ---------- ------RERK -NRNKM---- ---------- ----------
(PRED) sace_53_29_ac00 ---------- ------RERK -NRNKM---- ---------- ----------
(PRED) sace_17_7_g0393 ---------- ------RERK -NRNKM---- ---------- ----------
(PRED) sace_25_7_g0388 ---------- ------RERK -NRNKM---- ---------- ----------
(PRED) sace_37_7_g0385 ---------- ------RERK -NRNKM---- ---------- ----------
(PRED) sace_9_7_g00180 ---------- ------RERK -NRNKM---- ---------- ----------
(PRED) sace_60_6_f0335 ---------- ------RERK -NRNKM---- ---------- ----------
(PRED) sace_59_336_lx0 ---------- ------RERK -NRNKM---- ---------- ----------
(PRED) sace_31_7_3780  ---------- ------RERK -NRNKM---- ---------- ----------
(PRED) sace_34_8_3770  ---------- ------RERK -NRNKM---- ---------- ----------
(PRED) sace_58_71_bs00 ---------- ------RERK -NRNKM---- ---------- ----------
(PRED) sace_7_7_g03880 ---------- ------RERK -NRNKM---- ---------- ----------
(PRED) sace_35_7_3840  ---------- ------RERK -NRNKM---- ---------- ----------
(PRED) sace_43_7_g0387 ---------- ------RERK -NRNKM---- ---------- ----------
(PRED) sace_57_8_h0390 ---------- ------RERK -NRNKM---- ---------- ----------
(PRED) sace_45_7_g0389 ---------- ------RERK -NRNKM---- ---------- ----------
(PRED) sace_46_8_h0391 ---------- ------RERK -NRNKM---- ---------- ----------
(PRED) sace_23_7_3860  ---------- ------RERK -NRNKM---- ---------- ----------
(PRED) sace_21_7_3790  ---------- ------RERK -NRNKM---- ---------- ----------
(PRED) sace_8_73_bu001 ---------- ------RERK -NRNKM---- ---------- ----------
(PRED) sapa_1_8_h03820 ---------- ------RERK -NRNKM---- ---------- ----------
(PRED) sapa_21_8_h0387 ---------- ------RERK -NRNKM---- ---------- ----------
(PRED) sapa_20_8_h0386 ---------- ------RERK -NRNKM---- ---------- ----------
(PRED) sapa_22_8_h0390 ---------- ------RERK -NRNKM---- ---------- ----------
(PRED) sapa_25_8_h0387 ---------- ------RERK -NRNKM---- ---------- ----------
(PRED) sapa_6_8_3750   ---------- ------RERK -NRNKM---- ---------- ----------
(PRED) sapa_9_8_3720   ---------- ------RERK -NRNKM---- ---------- ----------
(PRED) sapa_19_8_h0390 ---------- ------RERK -NRNKM---- ---------- ----------
(PRED) sapa_24_8_h0385 ---------- ------RERK -NRNKM---- ---------- ----------
(PRED) sapa_4_8_h03850 ---------- ------RERK -NRNKM---- ---------- ----------
(PRED) sapa_10_8_3760  ---------- ------RERK -NRNKM---- ---------- ----------
(PRED) sapa_13_8_h0382 ---------- ------RERK -NRNKM---- ---------- ----------
(PRED) sapa_8_8_3750   ---------- ------RERK -NRNKM---- ---------- ----------
(PRED) sapa_11_8_h0383 ---------- ------RERK -NRNKM---- ---------- ----------
(PRED) sapa_5_8_3700   ---------- ------RERK -NRNKM---- ---------- ----------
(PRED) sapa_16_8_h0389 ---------- ------RERK -NRNKM---- ---------- ----------
(PRED) sapa_17_8_3730  ---------- ------RERK -NRNKM---- ---------- ----------
(PRED) sapa_2_8_h03860 ---------- ------RERK -NRNKM---- ---------- ----------
(PRED) sapa_7_8_3740   ---------- ------RERK -NRNKM---- ---------- ----------
(PRED) sapa_23_8_h0385 ---------- ------RERK -NRNKM---- ---------- ----------
(PRED) sapa_3_8_h03890 ---------- ------RERK -NRNKM---- ---------- ----------
(PRED) sapa_18_8_3730  ---------- ------RERK -NRNKM---- ---------- ----------
(PRED) sami_1_14_399   ---------- ------RERR -NRKKM---- ---------- ----------
(PRED) sace_4_8_h03690 ---------- ------RERK -NRNKM---- ---------- ----------
(PRED) saku_1_14_404   ---------- ------RERK -NRSKM---- ---------- ----------
(PRED) sace_1_ynr070w  ---------- ------RERK -NRNKM---- ---------- ----------
(PRED) sace_49_8_h0383 ---------- ------RERK -NRNKM---- ---------- ----------
(PRED) saeu_1_2_b00130 ---------- ------RESR -KHHKM---- ---------- ----------
(PRED) sauv_1_7_3      ---------- ------RHSK -MHNKM---- ---------- ----------
(PRED) sami_1_17_26    ---------- ------REIK -NRKRM---- ---------- ----------
(PRED) zyba_1_02055_AN ---------- ------KAMR -HR-KM---- ---------- ----------
(PRED) zyba_1_07912    ---------- ------KAMR -HR-KM---- ---------- ----------
(PRED) zyba_2_2_b00600 ---------- ------KAMR -HR-KM---- ---------- ----------
(PRED) zyba_3_3_c03460 ---------- ------KAMR -HR-KM---- ---------- ----------
(PRED) zyba_1_04634    ---------- ------KAAR -HR-KL---- ---------- ----------
(PRED) zyba_1_06675    ---------- ------RAAR -HR-KL---- ---------- ----------
(PRED) zyba_3_2_b02230 ---------- ------RAAR -HR-KL---- ---------- ----------
(PRED) zyba_2_1_a00860 ---------- ------RAAR -HR-KL---- ---------- ----------
(PRED) zyro_1_a04114g  ---------- ------KGVR -HR-RM---- ---------- ----------
(PRED) zyro_1_b14762g  ---------- ------YKKR -HQ-RM---- ---------- ----------
(PRED) zyba_2_14_n0149 ---------- ------KNMK -HR-KM---- ---------- ----------
(PRED) zyba_2_33_ag001 ---------- ------KNMK -HR-KM---- ---------- ----------
(PRED) lath_1_a01914g  ---------- ------REAK -NR-KT---- ---------- ----------
(PRED) lawa_1_23_5161  ---------- ------REVK -NR-GN---- ---------- ----------
(PRED) klae_1_14_n0012 ---------- ------KQAR --QSSL---- ---------- ----------
```

```
(PRED) klla_1_d03432g ---------- ------KKAR --QTTL---- ---------- ----------
(PRED) klma_1_1_a01880 ---------- ------RKAR --QTTL---- ---------- ----------
(PRED) klwi_1_33_ag001 ---------- ------NMMR --QTSL---- ---------- ----------
(PRED) teph_1_a04220 ---------- ------QRL- -KHTPK---- ---------- ----------
(PRED) vapo_1_1037_47 ---------- ------KKFT -QHKKM---- ---------- ----------
(PRED) pata_1_2_b05590 -------FSR GKNDLKKEKK TGDDKY---- ---------- ----------
(PRED) wian_1_3_c04380 ---------- ------KALR --HRKT---- ---------- ----------
(PRED) wian_1_3_c04390 ---------- ------KEKK --NAKT---- ---------- ----------
(PRED) wian_1_7_g01010 ---------- ------KESR --NVKH---- ---------- ----------
(PRED) bain_1_1_a00100 ---------- --------IK NRRTPE---- ---------- ----------
(PRED) bain_1_17_q0038 ---------- --------IK NRRTPE---- ---------- ----------
(PRED) bain_1_8_h00410 ---------- --------YK TSRLPH---- ---------- ----------
(PRED) caal_1_19_5759 ---------- --------LS QMKTPP---- ---------- ----------
(PRED) caal_11_25_y002 ---------- --------LS QMKTPP---- ---------- ----------
(PRED) caal_4_4_d03320 ---------- --------LS QMKTPP---- ---------- ----------
(PRED) caal_12_26_z005 ---------- --------LS QMKTPP---- ---------- ----------
(PRED) caal_5_30_ad005 ---------- --------LS QMKTPP---- ---------- ----------
(PRED) caal_8_3_c03320 ---------- --------LS QMKTPP---- ---------- ----------
(PRED) caal_6_4_d03280 ---------- --------LS QMKTPP---- ---------- ----------
(PRED) caal_10_3_c0334 ---------- --------LS QMKTPP---- ---------- ----------
(PRED) caal_3_29_ac005 ---------- --------LS QMKTPP---- ---------- ----------
(PRED) caal_2_04989 ---------- --------LS QMKTPP---- ---------- ----------
(PRED) cadu_1_64350 ---------- --------LS QMKTPP---- ---------- ----------
(PRED) caor_1_h02090 ---------- --------VS KMRTPK---- ---------- ----------
(PRED) capa_1_600750 ---------- --------IS KMRTPK---- ---------- ----------
(PRED) loel_1_04930 ---------- --------VS KARTPN---- ---------- ----------
(PRED) spar_1_5_e03260 ---------- --------MA KMKTPD---- ---------- ----------
(PRED) sppa_1_7_g03160 ---------- --------MA KIRTPN---- ---------- ----------
(PRED) catr_1_01205 ---------- --------IK ERNTPN---- ---------- ----------
(PRED) catr_1_05498 ---------- --------IR KIKTPK---- ---------- ----------
(PRED) catr_1_05971 ---------- --------IK KAKTPN---- ---------- ----------
(PRED) deha_1_a03696g ---------- --------MA KRKIQD---- ---------- ----------
(PRED) deha_2_5_e00720 ---------- --------MA KRKIQD---- ---------- ----------
(PRED) scst_1_3_c02890 ---------- --------MA KRKVPD---- ---------- ----------
(PRED) mebi_1_8_h00300 ---------- --------KE AKKISD---- ---------- ----------
(PRED) lakl_1_h21010g ---------- ------QAKK HHH--K---- ---------- ----------
(PRED) caar_1_13_m0142 ---------- K-LSKKTQAA TDLSKLPKT- ---------- ----------
(PRED) caar_1_14_n0143 ---------- R-LSNK--DT VDLSKMEKT- ---------- ----------
(PRED) hapo_1_1_a07220 ---------- K-FSSKKQQQ ADFNKLEKT- ---------- ----------
(PRED) ogpa_1_1_a01680 ---------- K-LSSKKQQQ ADFNKLEKT- ---------- ----------
(PRED) piku_1_96_cr001 ---------- R-RERKKN-S FDFKKLPKS- ---------- ----------
(PRED) pime_1_4_d03240 ---------- R-KEKQSS-S FDFGKLPKT- ---------- ----------
(PRED) pime_1_1_a12110 ---------- K-MNKKKNGG FDFKKLPKT- ---------- ----------
(PRED) piku_1_227_hs00 ---------- RCFNRHRKET FDFSKLPKK- ---------- ----------
(PRED) pime_1_5_e05800 ---------- K-FRKDKKDT FDFSKLAKK- ---------- ----------
(PRED) pime_1_1_a07690 ---------- R-LKRNKEQT FDFKSLPKT- ---------- ----------
(PRED) debr_2_5_e03380 GGGERGDGNV EGKNRERGNA EDISKESDTN AISQQGNAGT YQQEDIADTQ
(PRED) kopa_1_2_b10040 ---------- ---IRNRKPA L--------- ---------- ----------
(PRED) kopa_2_7_g00500 ---------- ---IRNRKPA L--------- ---------- ----------
(PRED) asru_1_13_m0119 ---------- ------KIKS VRHPSI---- ---------- ----------
(PRED) asru_1_15_o0045 ---------- ------TLKN AKN-NQ---- ---------- ----------
(PRED) wian_1_1_a02920 ---------- ------KSPT ---------- ---------- ----------
(PRED) wian_1_1_a02930 ---------- ------KSQN ---------- ---------- ----------

                .........360 .........370 .........380 .........390 .........400
(PRED) asac_1_6_f03560 ---------- ---------- ---------- ---------- ----------
(PRED) ergo_1_abr125c ---------- ---------- ---------- ---------- ----------
(PRED) ercy_1_3604 ---------- ---------- ---------- ---------- ----------
(PRED) cagl_1_i04862g ---------- ---------- ---------- ---------- ----------
(PRED) kaaf_1_c00830 ---------- ---------- ---------- ---------- ----------
(PRED) kana_1_k01350 ---------- ---------- ---------- ---------- ----------
(PRED) saar_1_2_b02590 ---------- ---------- ---------- ---------- ----------
(PRED) sace_1_ydr011w ---------- ---------- ---------- ---------- ----------
(PRED) sace_16_1_a0238 ---------- ---------- ---------- ---------- ----------
(PRED) sace_45_1_a0242 ---------- ---------- ---------- ---------- ----------
(PRED) sace_48_1_a0238 ---------- ---------- ---------- ---------- ----------
(PRED) sace_60_4_d0244 ---------- ---------- ---------- ---------- ----------
(PRED) sace_52_1_a0240 ---------- ---------- ---------- ---------- ----------
(PRED) sace_46_1_a0240 ---------- ---------- ---------- ---------- ----------
(PRED) sace_25_1_a0240 ---------- ---------- ---------- ---------- ----------
(PRED) sace_24_1_2300 ---------- ---------- ---------- ---------- ----------
(PRED) sace_47_1_a0240 ---------- ---------- ---------- ---------- ----------
(PRED) sace_7_1_a02410 ---------- ---------- ---------- ---------- ----------
(PRED) sace_59_110_df0 ---------- ---------- ---------- ---------- ----------
(PRED) sace_56_1_a0202 ---------- ---------- ---------- ---------- ----------
```

```
(PRED) sace_40_1_a0239 ---------- ---------- ---------- ---------- ----------
(PRED) sace_15_1_a0242 ---------- ---------- ---------- ---------- ----------
(PRED) sace_37_1_a0243 ---------- ---------- ---------- ---------- ----------
(PRED) sace_9_1_a02440 ---------- ---------- ---------- ---------- ----------
(PRED) sace_22_1_2300  ---------- ---------- ---------- ---------- ----------
(PRED) sace_29_1_2290  ---------- ---------- ---------- ---------- ----------
(PRED) sace_34_1_2320  ---------- ---------- ---------- ---------- ----------
(PRED) sace_58_25_y007 ---------- ---------- ---------- ---------- ----------
(PRED) sace_23_1_2290  ---------- ---------- ---------- ---------- ----------
(PRED) sace_6_120_dp00 ---------- ---------- ---------- ---------- ----------
(PRED) sace_57_1_a0241 ---------- ---------- ---------- ---------- ----------
(PRED) sace_17_1_a0241 ---------- ---------- ---------- ---------- ----------
(PRED) sace_21_1_2310  ---------- ---------- ---------- ---------- ----------
(PRED) sace_49_1_a0246 ---------- ---------- ---------- ---------- ----------
(PRED) sace_8_2_b02430 ---------- ---------- ---------- ---------- ----------
(PRED) sace_31_1_2300  ---------- ---------- ---------- ---------- ----------
(PRED) sace_50_1_a0241 ---------- ---------- ---------- ---------- ----------
(PRED) sace_4_1_a02360 ---------- ---------- ---------- ---------- ----------
(PRED) sace_2_1_a02390 ---------- ---------- ---------- ---------- ----------
(PRED) sace_5_33_ag005 ---------- ---------- ---------- ---------- ----------
(PRED) sapa_11_1_a0247 ---------- ---------- ---------- ---------- ----------
(PRED) sapa_25_1_a0246 ---------- ---------- ---------- ---------- ----------
(PRED) sapa_4_1_a02470 ---------- ---------- ---------- ---------- ----------
(PRED) sapa_5_1_2350   ---------- ---------- ---------- ---------- ----------
(PRED) sapa_9_1_2360   ---------- ---------- ---------- ---------- ----------
(PRED) sapa_14_1_a0244 ---------- ---------- ---------- ---------- ----------
(PRED) sapa_8_1_2350   ---------- ---------- ---------- ---------- ----------
(PRED) sapa_17_1_2380  ---------- ---------- ---------- ---------- ----------
(PRED) sapa_7_1_2370   ---------- ---------- ---------- ---------- ----------
(PRED) sapa_2_1_a02460 ---------- ---------- ---------- ---------- ----------
(PRED) sapa_23_1_a0248 ---------- ---------- ---------- ---------- ----------
(PRED) sapa_3_1_a02470 ---------- ---------- ---------- ---------- ----------
(PRED) sapa_18_1_2390  ---------- ---------- ---------- ---------- ----------
(PRED) sami_1_4_244    ---------- ---------- ---------- ---------- ----------
(PRED) saku_1_4_262    ---------- ---------- ---------- ---------- ----------
(PRED) saba_1_58_bf002 ---------- ---------- ---------- ---------- ----------
(PRED) saeu_1_4_d02400 ---------- ---------- ---------- ---------- ----------
(PRED) naca_1_e01640   ---------- ---------- ---------- ---------- ----------
(PRED) nada_1_g01850   ---------- ---------- ---------- ---------- ----------
(PRED) naca_1_e01630   ---------- ---------- ---------- ---------- ----------
(PRED) nada_1_g01840   ---------- ---------- ---------- ---------- ----------
(PRED) kaaf_1_c00820   ---------- ---------- ---------- ---------- ----------
(PRED) teph_1_m00640   ---------- ---------- ---------- ---------- ----------
(PRED) vapo_1_1036_28  ---------- ---------- ---------- ---------- ----------
(PRED) tebl_1_i01760   ---------- ---------- ---------- ---------- ----------
(PRED) tode_1_d04040   ---------- ---------- ---------- ---------- ----------
(PRED) naca_1_e01650   ---------- ---------- ---------- ---------- ----------
(PRED) tebl_1_g02820   ---------- ---------- ---------- ---------- ----------
(PRED) lakl_1_c11616g  ---------- ---------- ---------- ---------- ----------
(PRED) saar_1_8_h03780 ---------- ---------- ---------- ---------- ----------
(PRED) sace_14_7_g0015 ---------- ---------- ---------- ---------- ----------
(PRED) sace_15_7_g0387 ---------- ---------- ---------- ---------- ----------
(PRED) sace_24_8_3780  ---------- ---------- ---------- ---------- ----------
(PRED) sace_40_8_h0383 ---------- ---------- ---------- ---------- ----------
(PRED) sace_6_169_fm00 ---------- ---------- ---------- ---------- ----------
(PRED) sace_19_7_3840  ---------- ---------- ---------- ---------- ----------
(PRED) sace_32_7_3770  ---------- ---------- ---------- ---------- ----------
(PRED) sace_56_17_q011 ---------- ---------- ---------- ---------- ----------
(PRED) sace_5_78_bz001 ---------- ---------- ---------- ---------- ----------
(PRED) sace_2_8_h03860 ---------- ---------- ---------- ---------- ----------
(PRED) sace_53_29_ac00 ---------- ---------- ---------- ---------- ----------
(PRED) sace_17_7_g0393 ---------- ---------- ---------- ---------- ----------
(PRED) sace_25_7_g0388 ---------- ---------- ---------- ---------- ----------
(PRED) sace_37_7_g0385 ---------- ---------- ---------- ---------- ----------
(PRED) sace_9_7_g00180 ---------- ---------- ---------- ---------- ----------
(PRED) sace_60_6_f0335 ---------- ---------- ---------- ---------- ----------
(PRED) sace_59_336_lx0 ---------- ---------- ---------- ---------- ----------
(PRED) sace_31_7_3780  ---------- ---------- ---------- ---------- ----------
(PRED) sace_34_8_3770  ---------- ---------- ---------- ---------- ----------
(PRED) sace_58_71_bs00 ---------- ---------- ---------- ---------- ----------
(PRED) sace_7_7_g03880 ---------- ---------- ---------- ---------- ----------
(PRED) sace_35_7_3840  ---------- ---------- ---------- ---------- ----------
(PRED) sace_43_7_g0387 ---------- ---------- ---------- ---------- ----------
(PRED) sace_57_8_h0390 ---------- ---------- ---------- ---------- ----------
(PRED) sace_45_7_g0389 ---------- ---------- ---------- ---------- ----------
```

```
(PRED) sace_46_8_h0391 ----------  ----------  ----------  ----------  ----------
(PRED) sace_23_7_3860  ----------  ----------  ----------  ----------  ----------
(PRED) sace_21_7_3790  ----------  ----------  ----------  ----------  ----------
(PRED) sace_8_73_bu001 ----------  ----------  ----------  ----------  ----------
(PRED) sapa_1_8_h03820 ----------  ----------  ----------  ----------  ----------
(PRED) sapa_21_8_h0387 ----------  ----------  ----------  ----------  ----------
(PRED) sapa_20_8_h0386 ----------  ----------  ----------  ----------  ----------
(PRED) sapa_22_8_h0390 ----------  ----------  ----------  ----------  ----------
(PRED) sapa_25_8_h0387 ----------  ----------  ----------  ----------  ----------
(PRED) sapa_6_8_3750   ----------  ----------  ----------  ----------  ----------
(PRED) sapa_9_8_3720   ----------  ----------  ----------  ----------  ----------
(PRED) sapa_19_8_h0390 ----------  ----------  ----------  ----------  ----------
(PRED) sapa_24_8_h0385 ----------  ----------  ----------  ----------  ----------
(PRED) sapa_4_8_h03850 ----------  ----------  ----------  ----------  ----------
(PRED) sapa_10_8_3760  ----------  ----------  ----------  ----------  ----------
(PRED) sapa_13_8_h0382 ----------  ----------  ----------  ----------  ----------
(PRED) sapa_8_8_3750   ----------  ----------  ----------  ----------  ----------
(PRED) sapa_11_8_h0383 ----------  ----------  ----------  ----------  ----------
(PRED) sapa_5_8_3700   ----------  ----------  ----------  ----------  ----------
(PRED) sapa_16_8_h0389 ----------  ----------  ----------  ----------  ----------
(PRED) sapa_17_8_3730  ----------  ----------  ----------  ----------  ----------
(PRED) sapa_2_8_h03860 ----------  ----------  ----------  ----------  ----------
(PRED) sapa_7_8_3740   ----------  ----------  ----------  ----------  ----------
(PRED) sapa_23_8_h0385 ----------  ----------  ----------  ----------  ----------
(PRED) sapa_3_8_h03890 ----------  ----------  ----------  ----------  ----------
(PRED) sapa_18_8_3730  ----------  ----------  ----------  ----------  ----------
(PRED) sami_1_14_399   ----------  ----------  ----------  ----------  ----------
(PRED) sace_4_8_h03690 ----------  ----------  ----------  ----------  ----------
(PRED) saku_1_14_404   ----------  ----------  ----------  ----------  ----------
(PRED) sace_1_ynr070w  ----------  ----------  ----------  ----------  ----------
(PRED) sace_49_8_h0383 ----------  ----------  ----------  ----------  ----------
(PRED) saeu_1_2_b00130 ----------  ----------  ----------  ----------  ----------
(PRED) sauv_1_7_3      ----------  ----------  ----------  ----------  ----------
(PRED) sami_1_17_26    ----------  ----------  ----------  ----------  ----------
(PRED) zyba_1_02055_AN ----------  ----------  ----------  ----------  ----------
(PRED) zyba_1_07912    ----------  ----------  ----------  ----------  ----------
(PRED) zyba_2_2_b00600 ----------  ----------  ----------  ----------  ----------
(PRED) zyba_3_3_c03460 ----------  ----------  ----------  ----------  ----------
(PRED) zyba_1_04634    ----------  ----------  ----------  ----------  ----------
(PRED) zyba_1_06675    ----------  ----------  ----------  ----------  ----------
(PRED) zyba_3_2_b02230 ----------  ----------  ----------  ----------  ----------
(PRED) zyba_2_1_a00860 ----------  ----------  ----------  ----------  ----------
(PRED) zyro_1_a04114g  ----------  ----------  ----------  ----------  ----------
(PRED) zyro_1_b14762g  ----------  ----------  ----------  ----------  ----------
(PRED) zyba_2_14_n0149 ----------  ----------  ----------  ----------  ----------
(PRED) zyba_2_33_ag001 ----------  ----------  ----------  ----------  ----------
(PRED) lath_1_a01914g  ----------  ----------  ----------  ----------  ----------
(PRED) lawa_1_23_5161  ----------  ----------  ----------  ----------  ----------
(PRED) klae_1_14_n0012 ----------  ----------  ----------  ----------  ----------
(PRED) klla_1_d03432g  ----------  ----------  ----------  ----------  ----------
(PRED) klma_1_1_a01880 ----------  ----------  ----------  ----------  ----------
(PRED) klwi_1_33_ag001 ----------  ----------  ----------  ----------  ----------
(PRED) teph_1_a04220   ----------  ----------  ----------  ----------  ----------
(PRED) vapo_1_1037_47  ----------  ----------  ----------  ----------  ----------
(PRED) pata_1_2_b05590 ----------  ----------  ----------  ----------  ----------
(PRED) wian_1_3_c04380 ----------  ----------  ----------  ----------  ----------
(PRED) wian_1_3_c04390 ----------  ----------  ----------  ----------  ----------
(PRED) wian_1_7_g01010 ----------  ----------  ----------  ----------  ----------
(PRED) bain_1_1_a00100 ----------  ----------  ----------  ----------  ----------
(PRED) bain_1_17_q0038 ----------  ----------  ----------  ----------  ----------
(PRED) bain_1_8_h00410 ----------  ----------  ----------  ----------  ----------
(PRED) caal_1_19_5759  ----------  ----------  ----------  ----------  ----------
(PRED) caal_11_25_y002 ----------  ----------  ----------  ----------  ----------
(PRED) caal_4_4_d03320 ----------  ----------  ----------  ----------  ----------
(PRED) caal_12_26_z005 ----------  ----------  ----------  ----------  ----------
(PRED) caal_5_30_ad005 ----------  ----------  ----------  ----------  ----------
(PRED) caal_8_3_c03320 ----------  ----------  ----------  ----------  ----------
(PRED) caal_6_4_d03280 ----------  ----------  ----------  ----------  ----------
(PRED) caal_10_3_c0334 ----------  ----------  ----------  ----------  ----------
(PRED) caal_3_29_ac005 ----------  ----------  ----------  ----------  ----------
(PRED) caal_2_04989    ----------  ----------  ----------  ----------  ----------
(PRED) cadu_1_64350    ----------  ----------  ----------  ----------  ----------
(PRED) caor_1_h02090   ----------  ----------  ----------  ----------  ----------
(PRED) capa_1_600750   ----------  ----------  ----------  ----------  ----------
(PRED) loel_1_04930    ----------  ----------  ----------  ----------  ----------
```

```
(PRED) spar_1_5_e03260 ----------  ----------  ----------  ----------  ----------
(PRED) sppa_1_7_g03160 ----------  ----------  ----------  ----------  ----------
(PRED) catr_1_01205    ----------  ----------  ----------  ----------  ----------
(PRED) catr_1_05498    ----------  ----------  ----------  ----------  ----------
(PRED) catr_1_05971    ----------  ----------  ----------  ----------  ----------
(PRED) deha_1_a03696g  ----------  ----------  ----------  ----------  ----------
(PRED) deha_2_5_e00720 ----------  ----------  ----------  ----------  ----------
(PRED) scst_1_3_c02890 ----------  ----------  ----------  ----------  ----------
(PRED) mebi_1_8_h00300 ----------  ----------  ----------  ----------  ----------
(PRED) lakl_1_h21010g  ----------  ----------  ----------  ----------  ----------
(PRED) caar_1_13_m0142 ----------  ----------  ----------  ----------  ----------
(PRED) caar_1_14_n0143 ----------  ----------  ----------  ----------  ----------
(PRED) hapo_1_1_a07220 ----------  ----------  ----------  ----------  ----------
(PRED) ogpa_1_1_a01680 ----------  ----------  ----------  ----------  ----------
(PRED) piku_1_96_cr001 ----------  ----------  ----------  ----------  ----------
(PRED) pime_1_4_d03240 ----------  ----------  ----------  ----------  ----------
(PRED) pime_1_1_a12110 ----------  ----------  ----------  ----------  ----------
(PRED) piku_1_227_hs00 ----------  ----------  ----------  ----------  ----------
(PRED) pime_1_5_e05800 ----------  ----------  ----------  ----------  ----------
(PRED) pime_1_1_a07690 ----------  ----------  ----------  ----------  ----------
(PRED) debr_2_5_e03380 ESNTFQQCNT  NSTSQKSNTF  QQNNTNSTSQ  KSNTFQQSNT  NSTSQKSTPN
(PRED) kopa_1_2_b10040 ----------  ----------  ----------  ----------  ----------
(PRED) kopa_2_7_g00500 ----------  ----------  ----------  ----------  ----------
(PRED) asru_1_13_m0119 ----------  ----------  ----------  ----------  ----------
(PRED) asru_1_15_o0045 ----------  ----------  ----------  ----------  ----------
(PRED) wian_1_1_a02920 ----------  ----------  ----------  ----------  ----------
(PRED) wian_1_1_a02930 ----------  ----------  ----------  ----------  ----------

                       .........410 .........420 .........430 .........440 .........450
(PRED) asac_1_6_f03560 ---RDILKSI  TLLARPGEMV  LVLGRPGAGC  TSLLKTIAGE  TDQFHS--VE
(PRED) ergo_1_abr125c  ---RNILESV  TLLARPGEMV  LVLGRPGAGC  TSLLKTVAGE  TDQFHS--VE
(PRED) ercy_1_3604     ---RNILEKV  DFIARPGEMV  LVLGRPGAGC  SSLLKTVAGD  TDQFKS--VS
(PRED) cagl_1_i04862g  ---RTILNDV  SGLARAGEMV  LVLGRPGAGC  SSMLKVTAGE  IDQFAGGV-E
(PRED) kaaf_1_c00830   ---KSILQNV  NALARPGEMV  LVLGRPGAGC  SSFLKVTAGE  IDQFAGGV-T
(PRED) kana_1_k01350   ---KQILQNV  NCLAKAGEMV  LVLGRPGAGC  SSFLKVTAGE  IDQFAGGV-L
(PRED) saar_1_2_b02590 ---RQIISNV  NALAEAGEMI  LVLGRPGAGC  SSFLKVAAGE  IDQFAGGV-S
(PRED) sace_1_ydr011w  ---RQIISNV  NALAEAGEMI  LVLGRPGAGC  SSFLKVTAGE  IDQFAGGV-S
(PRED) sace_16_1_a0238 ---RQIISNV  NALAEAGEMI  LVLGRPGAGC  SSFLKVTAGE  IDQFAGGV-S
(PRED) sace_45_1_a0242 ---RQIISNV  NALAEAGEMI  LVLGRPGAGC  SSFLKVTAGE  IDQFAGGV-S
(PRED) sace_48_1_a0238 ---RQIISNV  NALAEAGEMI  LVLGRPGAGC  SSFLKVTAGE  IDQFAGGV-S
(PRED) sace_60_4_d0244 ---RQIISNV  NALAEAGEMI  LVLGRPGAGC  SSFLKVTAGE  IDQFAGGV-S
(PRED) sace_52_1_a0240 ---RQIISNV  NALAEAGEMI  LVLGRPGAGC  SSFLKVTAGE  IDQFAGGV-S
(PRED) sace_46_1_a0240 ---RQIISNV  NALAEAGEMI  LVLGRPGAGC  SSFLKVTAGE  IDQFAGGV-S
(PRED) sace_25_1_a0240 ---RQIISNV  NALAEAGEMI  LVLGRPGAGC  SSFLKVTAGE  IDQFAGGV-S
(PRED) sace_24_1_2300  ---RQIISNV  NALAEAGEMI  LVLGRPGAGC  SSFLKVTAGE  IDQFAGGV-S
(PRED) sace_47_1_a0240 ---RQIISNV  NALAEAGEMI  LVLGRPGAGC  SSFLKVTAGE  IDQFAGGV-S
(PRED) sace_7_1_a02410 ---RQIISNV  NALAEAGEMI  LVLGRPGAGC  SSFLKVTAGE  IDQFAGGV-S
(PRED) sace_59_110_df0 ---RQIISNV  NALAEAGEMI  LVLGRPGAGC  SSFLKVTAGE  IDQFAGGV-S
(PRED) sace_56_1_a0202 ---RQIISNV  NALAEAGEMI  LVLGRPGAGC  SSFLKVTAGE  IDQFAGGV-S
(PRED) sace_40_1_a0239 ---RQIISNV  NALAEAGEMI  LVLGRPGAGC  SSFLKVTAGE  IDQFAGGV-S
(PRED) sace_15_1_a0242 ---RQIISNV  NALAEAGEMI  LVLGRPGAGC  SSFLKVTAGE  IDQFAGGV-S
(PRED) sace_37_1_a0243 ---RQIISNV  NALAEAGEMI  LVLGRPGAGC  SSFLKVTAGE  IDQFAGGV-S
(PRED) sace_9_1_a02440 ---RQIISNV  NALAEAGEMI  LVLGRPGAGC  SSFLKVTAGE  IDQFAGGV-S
(PRED) sace_22_1_2300  ---RQIISNV  NALAEAGEMI  LVLGRPGAGC  SSFLKVTAGE  IDQFAGGV-S
(PRED) sace_29_1_2290  ---RQIISNV  NALAEAGEMI  LVLGRPGAGC  SSFLKVTAGE  IDQFAGGV-S
(PRED) sace_34_1_2320  ---RQIISNV  NALAEAGEMI  LVLGRPGAGC  SSFLKVTAGE  IDQFAGGV-S
(PRED) sace_58_25_y007 ---RQIISNV  NALAEAGEMI  LVLGRPGAGC  SSFLKVTAGE  IDQFAGGV-S
(PRED) sace_23_1_2290  ---RQIISNV  NALAEAGEMI  LVLGRPGAGC  SSFLKVTAGE  IDQFAGGV-S
(PRED) sace_6_120_dp00 ---RQIISNV  NALAEAGEMI  LVLGRPGAGC  SSFLKVTAGE  IDQFAGGV-S
(PRED) sace_57_1_a0241 ---RQIISNV  NALAEAGEMI  LVLGRPGAGC  SSFLKVTAGE  IDQFAGGV-S
(PRED) sace_17_1_a0241 ---RQIISNV  NALAEAGEMI  LVLGRPGAGC  SSFLKVTAGE  IDQFAGGV-S
(PRED) sace_21_1_2310  ---RQIISNV  NALAEAGEMI  LVLGRPGAGC  SSFLKVTAGE  IDQFAGGV-S
(PRED) sace_49_1_a0246 ---RQIISNV  NALAEAGEMI  LVLGRPGAGC  SSFLKVTAGE  IDQFAGGV-S
(PRED) sace_8_2_b02430 ---RQIISNV  NALAEAGEMI  LVLGRPGAGC  SSFLKVTAGE  IDQFAGGV-S
(PRED) sace_31_1_2300  ---RQIISNV  NALAEAGEMI  LVLGRPGAGC  SSFLKVTAGE  IDQFAGGV-S
(PRED) sace_50_1_a0241 ---RQIISNV  NALAEAGEMI  LVLGRPGAGC  SSFLKVTAGE  IDQFAGGV-S
(PRED) sace_4_1_a02360 ---RQIISNV  NALAEAGEMI  LVLGRPGAGC  SSFLKVTAGE  IDQFAGGV-S
(PRED) sace_2_1_a02390 ---RQIISNV  NALAEAGEMI  LVLGRPGAGC  SSFLKVTAGE  IDQFAGGV-S
(PRED) sace_5_33_ag005 ---RQIISNV  NALAEAGEMI  LVLGRPGAGC  SSFLKVTAGE  IDQFAGGV-S
(PRED) sapa_11_1_a0247 ---RQIISNV  NALAEAGEMI  LVLGRPGAGC  SSFLKVTAGE  IDQFAGGV-S
(PRED) sapa_25_1_a0246 ---RQIISNV  NALAEAGEMI  LVLGRPGAGC  SSFLKVTAGE  IDQFAGGV-S
(PRED) sapa_4_1_a02470 ---RQIISNV  NALAEAGEMI  LVLGRPGAGC  SSFLKVTAGE  IDQFAGGV-S
(PRED) sapa_5_1_2350   ---RQIISNV  NALAEAGEMI  LVLGRPGAGC  SSFLKVTAGE  IDQFAGGV-S
(PRED) sapa_9_1_2360   ---RQIISNV  NALAEAGEMI  LVLGRPGAGC  SSFLKVTAGE  IDQFAGGV-S
(PRED) sapa_14_1_a0244 ---RQIISNV  NALAEAGEMI  LVLGRPGAGC  SSFLKVTAGE  IDQFAGGV-S
```

(PRED)	sapa_8_1_2350	---RQIISNV	NALAEAGEMI	LVLGRPGAGC	SSFLKVTAGE	IDQFAGGV-S
(PRED)	sapa_17_1_2380	---RQIITNV	NALAEAGEMI	LVLGRPGAGC	SSFLKVTAGE	IDQFAGGV-S
(PRED)	sapa_7_1_2370	---RQIITNV	NAIAEAGEMV	LVLGRPGAGC	SSFLKVTAGE	IDQFAGGV-S
(PRED)	sapa_2_1_a02460	---RQIITNV	NALAEAGEMI	LVLGRPGAGC	SSFLKVTAGE	IDQFAGGV-S
(PRED)	sapa_23_1_a0248	---RQIITNV	NALAEAGEMI	LVLGRPGAGC	SSFLKVTAGE	IDQFAGGV-S
(PRED)	sapa_3_1_a02470	---RQIITNV	NALAEAGEMI	LVLGRPGAGC	SSFLKVTAGE	IDQFAGGV-S
(PRED)	sapa_18_1_2390	---RQIITNV	NALAEAGEMI	LVLGRPGAGC	SSFLKVTAGE	IDQFAGGV-S
(PRED)	sami_1_4_244	---RQIISNI	NALAEAGEMI	LVLGRPGAGC	SSFLKVTAGE	IDQFAGGV-S
(PRED)	saku_1_4_262	---REIICNV	NALAEAGEMI	LVLGRPGAGC	SSFLKVTAGE	IDQFAGGV-S
(PRED)	saba_1_58_bf002	---RDIIQNV	NALAEAGEMV	LVLGRPGAGC	SSFLKVTAGE	IDQFAGGV-S
(PRED)	saeu_1_4_d02400	---RDIIRNV	NALAEAGEMV	LVLGRPGAGC	SSFLKVTAGE	IDQFAGGV-S
(PRED)	naca_1_e01640	---RKILQNV	NALAEAGEMV	LVLGRPGAGC	SSFLKVTAGE	TDQFAGGV-T
(PRED)	nada_1_g01850	---RRILNNV	NVLAKAGEMV	LVLGRPGAGC	SSFLKVTAGE	IDQFAGGI-K
(PRED)	naca_1_e01630	---RKIIQNS	YALAKPGEMI	LVLGRPGAGC	SSFLKVIAGE	IDQFPGGV-K
(PRED)	nada_1_g01840	---RQIIRNC	YALAEPGEMI	LVLGRPGAGC	SSLLKTIAGE	IDQFAGGV-E
(PRED)	kaaf_1_c00820	---TNILQNI	NGLANAGEMV	LVLGRPGAGC	SSFLKATAGE	ISQFAGGV-T
(PRED)	teph_1_m00640	---RSILNNV	NLLAEPGEMV	LVLGRPGAGC	SSFLKTAAGE	IDQFAGGV-V
(PRED)	vapo_1_1036_28	---RKILQNI	NCLAEPGEMV	LVLGRPGAGC	SSFLKVTAGE	IDQFAGGV-K
(PRED)	tebl_1_i01760	---MHILRNV	YAFAEPGEMV	LVLGRPGAGC	STFLKVTAGQ	IDQLAGGV-T
(PRED)	tode_1_d04040	---RKILRGV	NLLARPGEMV	LVLGRPGAGC	SSMLKTAAGV	TDQFAGGV-S
(PRED)	naca_1_e01650	---RKILQNC	NALAKPGEMI	LVLGRPGAGC	SSFLKVTAGE	IDQFAGGV-K
(PRED)	tebl_1_g02820	---RKIIQNI	NVLAKPGEMV	LVLGRPGAGC	SSFLKTTAGE	IDQFAGGV-E
(PRED)	lakl_1_c11616g	---RKIIQGV	NLLALPGEMI	LVLGRPGAGC	SSFLKTISGE	VDQFP-EV-T
(PRED)	saar_1_8_h03780	---RNILANV	SLLAKPGEMI	LVLGRPGAGC	SSFLKSAAGE	TSQFAGGV-T
(PRED)	sace_14_7_g0015	---KIXLKNV	SLLAKSGEMV	LVLGRPGAGC	TSFLKSAAGE	TSQFAGGVTT
(PRED)	sace_15_7_g0387	---KIILKNV	SLLAKSGEMV	LVLGRPGAGC	TSFLKSAAGE	TSQFAGGVTT
(PRED)	sace_24_8_3780	---KIILKNV	SLLAKSGEMV	LVLGRPGAGC	TSFLKSAAGE	TSQFAGGVTT
(PRED)	sace_40_8_h0383	---KIILKNV	SLLAKSGEMV	LVLGRPGAGC	TSFLKSAAGE	TSQFAGGVTT
(PRED)	sace_6_169_fm00	---KIILKNV	SLLAKSGEMV	LVLGRPGAGC	TSFLKSAAGE	TSQFAGGVTT
(PRED)	sace_19_7_3840	---KIILKNV	SLLAKSGEMV	LVLGRPGAGC	TSFLKSAAGE	TSQFAGGVTT
(PRED)	sace_32_7_3770	---KIILKNV	SLLAKPGEMV	LVLGRPGAGC	TSFLKSAAGE	TSQFAGGVTT
(PRED)	sace_56_17_q011	---KIILKNV	SLLAKPGEMV	LVLGRPGAGC	TSFLKSAAGE	TSQFAGGVTT
(PRED)	sace_5_78_bz001	---KIILKNV	SLLAKXGEMV	LVLGRPGAGC	TSFLKSAAGE	TSQFAGGVTT
(PRED)	sace_2_8_h03860	---KIILKNV	SLLAKSGEMV	LVLGRPGAGC	TSFLKSAAGE	TSQFAGGVTT
(PRED)	sace_53_29_ac00	---KIILKNV	SLLAKSGEMV	LVLGRPGAGC	TSFLKSAAGE	TSQFAGGVTT
(PRED)	sace_17_7_g0393	---KIILKNV	SLLAKPGEMV	LVLGRPGAGC	TSFLKSAAGE	TSQFAGGVTT
(PRED)	sace_25_7_g0388	---KIILKNV	SLLAKPGEMV	LVLGRPGAGC	TSFLKSAAGE	TSQFAGGVTT
(PRED)	sace_37_7_g0385	---KIILKNV	SLLAKPGEMV	LVLGRPGAGC	TSFLKSAAGE	TSQFAGGVTT
(PRED)	sace_9_7_g00180	---KIILKNV	SLLAKPGEMV	LVLGRPGAGC	TSFLKSAAGE	TSQFAGGVTT
(PRED)	sace_60_6_f0335	---KIILKNV	SLLAKPGEMV	LVLGRPGAGC	TSFLKSAAGE	TSQFAGGVTT
(PRED)	sace_59_336_lx0	---KIILKNV	SLLAKPGEMV	LVLGRPGAGC	TSFLKSAAGE	TSQFAGGVTT
(PRED)	sace_31_7_3780	---KIILKNV	SLLAKPGEMV	LVLGRPGAGC	TSFLKSAAGE	TSQFAGGVTT
(PRED)	sace_34_8_3770	---KIILKNV	SLLAKPGEMV	LVLGRPGAGC	TSFLKSAAGE	TSQFAGGVTT
(PRED)	sace_58_71_bs00	---KIILKNV	SLLAKPGEMV	LVLGRPGAGC	TSFLKSAAGE	TSQFAGGVTT
(PRED)	sace_7_7_g03880	---KIILKNV	SLLAKPGEMV	LVLGRPGAGC	TSFLKSAAGE	TSQFAGGVTT
(PRED)	sace_35_7_3840	---KIILKNV	SLLAKPGEMV	LVLGRPGAGC	TSFLKSAAGE	TSQFAGGVTT
(PRED)	sace_43_7_g0387	---KIILKNV	SLLAKPGEMV	LVLGRPGAGC	TSFLKSAAGE	TSQFAGGVTT
(PRED)	sace_57_8_h0390	---KIILKNV	SLLAKPGEMV	LVLGRPGAGC	TSFLKSAAGE	TSQFAGGVTT
(PRED)	sace_45_7_g0389	---KIILKNV	SLLAKPGEMV	LVLGRPGAGC	TSFLKSAAGE	TSQFAGGVTT
(PRED)	sace_46_8_h0391	---KIILKNV	SLLAKPGEMV	LVLGRPGAGC	TSFLKSAAGE	TSQFAGGVTT
(PRED)	sace_23_7_3860	---KIILKNV	SLLAKPGEMV	LVLGRPGAGC	TSFLKSAAGE	TSQFAGGVTT
(PRED)	sace_21_7_3790	---KIILKNV	SLLAKPGEMV	LVLGRPGAGC	TSFLKSAAGE	TSQFAGGVTT
(PRED)	sace_8_73_bu001	---KIILKNV	SLLAKXGEMV	LVLGRPGAGC	TSFLKSAAGE	TSQFAGGVTT
(PRED)	sapa_1_8_h03820	---KIILKDV	SLLAKQGEMV	LVLGRPGAGC	SSFLKSAAGE	TSQFAGGITT
(PRED)	sapa_21_8_h0387	---KIILKDV	SLLAKQGEMV	LVLGRPGAGC	SSFLKSAAGE	TSQFAGGITT
(PRED)	sapa_20_8_h0386	---KIILKDV	SLLAKQGEMV	LVLGRPGAGC	SSFLKSAAGE	TSQFAGGITT
(PRED)	sapa_22_8_h0390	---KIILKDV	SLLAKQGEMV	LVLGRPGAGC	SSFLKSAAGE	TSQFAGGITT
(PRED)	sapa_25_8_h0387	---KIILKDV	SLLAKQGEMV	LVLGRPGAGC	SSFLKSAAGE	TSQFAGGITT
(PRED)	sapa_6_8_3750	---KIILKDV	SLLAKQGEMV	LVLGRPGAGC	SSFLKSAAGE	TSQFAGGITT
(PRED)	sapa_9_8_3720	---KIILKDV	SLLAKQGEMV	LVLGRPGAGC	SSFLKSAAGE	TSQFAGGITT
(PRED)	sapa_19_8_h0390	---KIILKDV	SLLAKQGEMV	LVLGRPGAGC	SSFLKSAAGE	TSQFAGGITT
(PRED)	sapa_24_8_h0385	---KIILKDV	SLLAKQGEMV	LVLGRPGAGC	SSFLKSAAGE	TSQFAGGITT
(PRED)	sapa_4_8_h03850	---KIILKDV	SLLAKQGEMV	LVLGRPGAGC	SSFLKSAAGE	TSQFAGGITT
(PRED)	sapa_10_8_3760	---KIILKDV	SLLAKQGEMV	LVLGRPGAGC	SSFLKSAAGE	TSQFAGGITT
(PRED)	sapa_13_8_h0382	---KIILKDV	SLLAKQGEMV	LVLGRPGAGC	SSFLKSAAGE	TSQFAGGITT
(PRED)	sapa_8_8_3750	---KIILKDV	SLLAKQGEMV	LVLGRPGAGC	SSFLKSAAGE	TSQFAGGITT
(PRED)	sapa_11_8_h0383	---KIILKDV	SLLAKQGEMV	LVLGRPGAGC	SSFLKSAAGE	TSQFAGGITT
(PRED)	sapa_5_8_3700	---KIILKDV	SLLAKQGEMV	LVLGRPGAGC	SSFLKSAAGE	TSQFAGGITT
(PRED)	sapa_16_8_h0389	---KIILKDV	SLLAKQGEMV	LVLGRPGAGC	SSFLKTAAGE	TSQFAGGITT
(PRED)	sapa_17_8_3730	---KIILKDV	SLLAKQGEMV	LVLGRPGAGC	SSFLKTAAGE	TSQFAGGITT
(PRED)	sapa_2_8_h03860	---KIILKDV	SLLAKQGEMV	LVLGRPGAGC	SSFLKTAAGE	TSQFAGGITT
(PRED)	sapa_7_8_3740	---KIILKDV	SLLAKQGEMV	LVLGRPGAGC	SSFLKTAAGE	TSQFAGGITT
(PRED)	sapa_23_8_h0385	---KIILKDV	SLLAKQGEMV	LVLGRPGAGC	SSFLKTAAGE	TSQFAGGITT
(PRED)	sapa_3_8_h03890	---KIILKDV	SLLAKQGEMV	LVLGRPGAGC	SSFLKTAAGE	TSQFAGGITT
(PRED)	sapa_18_8_3730	---KIILKDV	SLLAKQGEMV	LVLGRPGAGC	SSFLKSAAGE	TSQFAGGITT

(PRED)	sami_1_14_399	---IMILKSV	SLLAKPGEMV	LVLGRPGAGC	SSFLKSAAGE	TSQFAGGVAT
(PRED)	sace_4_8_h03690	---KIILKNV	SLLAKPGEMV	LVLGRPGAGC	TSFLKSAAGE	TSQFAGGVTT
(PRED)	saku_1_14_404	---RKILKDV	SLLAKPGEMV	LVLGRPGAGC	SSLLKCAAGE	TNQFAGDL-T
(PRED)	sace_1_ynr070w	---KIILKNV	SLLAKSGEMV	LVLGRPGAGC	TSFLKSAAGE	TSQFAGGVTT
(PRED)	sace_49_8_h0383	---KIILKNV	SLLAKXGEMV	LVLGRPGAGC	TSFLKSAAGE	TSQFAGGVTT
(PRED)	saeu_1_2_b00130	---RTILTNV	SLLAKPGEMV	LVLGRPGAGC	SSLLKCAAGE	TSQFAGGV-T
(PRED)	sauv_1_7_3	---MTILTNV	SLLAKPGEMV	LVLGRPGAGC	SSLLKCAAGE	TSQFAGGV-T
(PRED)	sami_1_17_26	---RTILKSV	NLVAKSGEMV	LVLGRPGAGC	SSFLKIAAGE	TSQFAGGV-T
(PRED)	zyba_1_02055_AN	---RKIIRDV	NALAKPGEMV	LVLGRPGSGC	SSFLRTAAGV	TDQFAGGV-S
(PRED)	zyba_1_07912	---RKIIRDV	NALAKPGEMV	LVLGRPGSGC	SSFLRTAAGV	TDQFAGGV-A
(PRED)	zyba_2_2_b00600	---RKIIRDV	NALAKPGEMV	LVLGRPGSGC	SSFLRTAAGV	TDQFAGGV-A
(PRED)	zyba_3_3_c03460	---RKIIRDV	NALAKPGEMV	LVLGRPGSGC	SSFLRTTAGV	TDQFAGGV-A
(PRED)	zyba_1_04634	---RKIIRGV	NALAKPGEMV	LVLGRPGSGC	SSFLKTAAGI	TDEFAGGV-E
(PRED)	zyba_1_06675	---RKIIRGV	NVLAKPGEMV	LVLGRPGSGC	SSFLKTAAGI	TDEFAGGV-E
(PRED)	zyba_3_2_b02230	---RKIIRGV	NVLAKPGEMV	LVLGRPGSGC	SSFLKTAAGI	TDEFAGGV-E
(PRED)	zyba_2_1_a00860	---RKIIRGV	NVLAKPGEMV	LVLGRPGSGC	SSFLKTAAGI	TDEFAGGV-E
(PRED)	zyro_1_a04114g	---RKIIRNV	NAFALPGEMV	LVLGRPGSGC	SSFLKTAAGV	TDQFAGGV-E
(PRED)	zyro_1_b14762g	---RKILQNI	NVLAKPGEMI	LVLGRPGSGC	SSFLKTAAGV	TDQFAGGV-D
(PRED)	zyba_2_14_n0149	---RKILNDM	DSLARPGEMV	LVLGRPGSGC	SSFLKVAAGV	INQFAGGV-E
(PRED)	zyba_2_33_ag001	---RKVLNDM	NALARPGEMV	LVLGRPGSGC	SSFLKVAAGV	IRQFAGGV-E
(PRED)	lath_1_a01914g	---RDIVRNA	NILARPGEMI	LVLGRPGAGC	SSFLKTVSGE	VDNFEE--VR
(PRED)	lawa_1_23_5161	---RDIVRNA	SLLARPGDMV	LVLGRPGAGC	SSFLKTIAGE	TRNFEE--VR
(PRED)	klae_1_14_n0012	---RDIISGF	NVMLRPGEML	LVLGRPGSGC	STFLKTMAGE	LAHFKG--VE
(PRED)	klla_1_d03432g	---RDIISNV	NLLVRPGEML	LVLGRPGSGC	STFLKTMAGE	LSHFKG--VS
(PRED)	klma_1_1_a01880	---RDIISNI	NILVKPGEML	LVLGRPGSGC	STLLKTMAGE	LTHFKG--VE
(PRED)	klwi_1_33_ag001	---RDIISGV	NLFLRPGEML	LVLGRPGSGC	STFLKTMAGE	LSHFKG--VE
(PRED)	teph_1_a04220	---KKILKSI	NGVAEPGKIV	LVLGKPGSGS	TTLLKIIAGE	GSQCHGKQ-A
(PRED)	vapo_1_1037_47	---KSILSEV	NFLVKPGNMV	LVLGRPGSGC	STLLKTAVGE	TSSYKGNV-S
(PRED)	pata_1_2_b05590	---RKILRNI	NCLVNPGEMC	LVLGRPGAGC	SSFLKVVAGE	NEQFVK--VN
(PRED)	wian_1_3_c04380	---RKLIRNV	NGVVEPGEMC	LVLGRPGAGC	STLLRTIAGE	HDQFIG--VD
(PRED)	wian_1_3_c04390	---RSIVRNI	TGVVKPGEMC	LVLGRPGAGC	STLLRTIAGE	HDQFVS--VN
(PRED)	wian_1_7_g01010	---RKLIKNV	SGVVKPGEMC	LVLGRPGAGC	STLLKTIAAE	HDQFVS--VD
(PRED)	bain_1_1_a00100	---RKILHEM	AGLAHSGEMV	LVLGRPGAGC	SSFLKTIGG-	ETAQFTHT-S
(PRED)	bain_1_17_q0038	---RKILHEM	AGLAHSGEMV	LVLGRPGAGC	SSFLKTIGG-	EISSFTHT-S
(PRED)	bain_1_8_h00410	---IPIIRNI	NGMAREGEMV	LVLGRPGAGC	STLLKTIAG-	ETDNYQGT-S
(PRED)	caal_1_19_5759	---RKILKNL	NGFAKPGESV	LVLGRPGAGC	TTFLKALSGT	DFDLYKGV-T
(PRED)	caal_11_25_y002	---RKILKNL	NGFAKPGESV	LVLGRPGAGC	TTFLKALSGT	DFDLYKGV-T
(PRED)	caal_4_4_d03320	---RKILKNL	NGFAKPGESV	LVLGRPGAGC	TTFLKALSGT	DFDLYKGV-T
(PRED)	caal_12_26_z005	---RKILKNL	NGFAKPGESV	LVLGRPGAGC	TTFLKALSGT	DFDLYKGV-T
(PRED)	caal_5_30_ad005	---RKILKNL	NGFAKPGESV	LVLGRPGAGC	TTFLKALSGT	DFDLYKGV-T
(PRED)	caal_8_3_c03320	---RKILKNL	NGFAKPGESV	LVLGRPGAGC	TTFLKALSGT	DFDLYKGV-T
(PRED)	caal_6_4_d03280	---RKILKNL	NGFAKPGESV	LVLGRPGAGC	TTFLKALSGT	DFDLYKGV-T
(PRED)	caal_10_3_c0334	---RKILKNL	NGFAKPGESV	LVLGRPGAGC	TTFLKALSGT	DFDLYKGV-T
(PRED)	caal_3_29_ac005	---RKILKNL	NGFAKPGESV	LVLGRPGAGC	TTFLKALSGT	DFDLYKGV-T
(PRED)	caal_2_04989	---RKILKNL	NGFAKPGESV	LVLGRPGAGC	TTFLKALSGT	DFDLYKGV-T
(PRED)	cadu_1_64350	---RKILKNL	NGFAKPGESV	LVLGRPGAGC	TTFLKALSGT	DFDLYKGV-T
(PRED)	caor_1_h02090	---KTILNNL	NGFAKPGEMV	LVLGRPGAGC	TTFLKSLTGT	DFDLYKGV-E
(PRED)	capa_1_600750	---KTILNNL	NGLAKPGEMV	LVLGRPGAGC	TTFLKSLTGT	DFDLYRGV-E
(PRED)	loel_1_04930	---KKILHKL	NGCAKPGEMV	LVLGRPGAGC	TTFLKSISGT	DHDLYKGV-E
(PRED)	spar_1_5_e03260	---KNILHNI	NGFAKPGEMV	LVLGRPGAGC	TTFLKAISGT	DFDLYKGI-E
(PRED)	sppa_1_7_g03160	---RNLLHNL	NGFARPGEMV	LVLGRPGAGC	TTFLKAISGT	DFDLYKGV-E
(PRED)	catr_1_01205	---RQILKNF	NGFAKPGDMV	LVLGRPGAGC	TTFLKSLSGT	DFDLYKGI-D
(PRED)	catr_1_05498	---REILKSF	NGLAKPGDMV	LVLGRPGAGC	TTFLKALSGT	DFDLYKGI-E
(PRED)	catr_1_05971	---RMILKHL	NGFAKPGDMV	LVLGRPGAGC	TTFLKALSGT	DFDLYKGI-E
(PRED)	deha_1_a03696g	---RKIVSNV	NGYARSGEMV	LVLGRPGAGC	SSLLKAIGGT	DLDLFTGV-D
(PRED)	deha_2_5_e00720	---RKIIRDV	SGYARSGEMV	LVLGRPGAGC	SSFLKAIGGT	DLDLFTGV-E
(PRED)	scst_1_3_c02890	---RTILNKL	NGLARPGEMI	LVLGRPGAGC	SSFLKALSGT	DFDLFKGV-E
(PRED)	mebi_1_8_h00300	---RAILDSF	DGLVKSGEML	LVLGRPGSGC	STFLKTISNT	DTEMYTGL-E
(PRED)	lakl_1_h21010g	---RKILSQI	NFLIKPGEMC	LVLGRPGAGC	SSFLRTVAGE	LGNFTD--VD
(PRED)	caar_1_13_m0142	---REIIRNV	TGFAEAGGMT	LVLGRPGAGC	STLLKAVAGQ	TQTYRS--TE
(PRED)	caar_1_14_n0143	---RKIIRNV	TGYCEAGTLT	LVLGRPGAGC	STMLKALTGQ	TQTYVG--QG
(PRED)	hapo_1_1_a07220	---RKILQKF	NGICEAGTMT	LALGRPGSGC	SSLLKALAGE	TQTYVG--TS
(PRED)	ogpa_1_1_a01680	---RKILQNF	NGICEAGTMT	LALGRPGSGC	SSLLKVLAGE	TQTYVG--TS
(PRED)	piku_1_96_cr001	---RNIVKGA	SGYLEAGSMT	LVLGRPGAGC	SSLLKILSGE	TKTYTG--YE
(PRED)	pime_1_4_d03240	---RKIVKDV	TGFASPGTMT	LVLGRPGAGC	SSFLKVLCGE	TKTYLG--YE
(PRED)	pime_1_1_a12110	---RKIVKDA	SGFAEPGTMT	LVLGRPGAGC	STLLKVLCGQ	TKTYLG--YE
(PRED)	piku_1_227_hs00	---RKVIKDV	SGYCSPGTMT	LVLGRPGAGC	STLLKSLAGE	AKTYIG--IK
(PRED)	pime_1_5_e05800	---RKVIKDI	SGYALPGTMT	LVLGRPGAGC	STLLKTIAGE	RKTYIR--TD
(PRED)	pime_1_1_a07690	---RKIIRGV	TGFASPGTIT	LVLGRPGAGC	STFLKALTGE	VQTYKG--VE
(PRED)	debr_2_5_e03380	PAYRHLIHRV	NGIAEPGTLT	LVLGRPGAGC	STLLKALAGQ	TQAYEE--VQ
(PRED)	kopa_1_2_b10040	---RHILKGV	DFHTVPGEMC	LVLGRPGAGC	SSLLKTIAGE	TSHFVR--VE
(PRED)	kopa_2_7_g00500	---RHILKGV	DFHTVPGEMC	LVLGRPGAGC	SSLLKTIAGE	TTHFVK--VE
(PRED)	asru_1_13_m0119	---RDIISNV	NGLVKAGEMC	LVLGRPGAGC	STFLKSIAGE	TNQYVS--ST
(PRED)	asru_1_15_o0045	---KQIIKNI	NGIVKPGEMC	LVLGKPGSGC	STLLKTVAGE	ASQYSA--ID
(PRED)	wian_1_1_a02920	---RKLIKNI	NGFVNSGEML	LVLGRPGAGC	STFLKTISGE	THNYVG--TE

```
(PRED) wian_1_1_a02930 ---RKLIKNI  NGFVNSGEML  LVLGRPGAGC  STFLRTISGE  THHYVG--TE

                       .........460 .........470 .........480 .........490 .........500
(PRED) asac_1_6_f03560 GEISYDGISQ  REMMRRYRAE  VVYNSEIDVH  FPHLTVKQTL  DFALACKTP-
(PRED) ergo_1_abr125c  GAISYDGIPQ  REMMRRYRAE  VVYNSEIDVH  FPHLTVKQTL  DFALACKTP-
(PRED) ercy_1_3604     GSISYDGIPQ  DEMMSNFKAE  VIYNGEEDVH  FPHLTLRQTL  DFAIACKTP-
(PRED) cagl_1_i04862g  GEIMYDGIPQ  KEMMKRYKPD  VIYNGEQDVH  FPHLTVQQTL  DFAIACKTP-
(PRED) kaaf_1_c00830   GDVAYDGISQ  DEMMKNYRAD  VIYNGELDVH  FPYLTVKQTL  DFAIACKMP-
(PRED) kana_1_k01350   GDIAYDGIPQ  KEMMKHYKAD  VIYNGELDVH  FPYLTVQQTL  DFAIACKTP-
(PRED) saar_1_2_b02590 GDVAYDGIPQ  DEMMKHYKAD  VIYNGELDVH  FPYLTVKQTL  DFAIACKTP-
(PRED) sace_1_ydr011w  GEVAYDGIPQ  EEMMKRYKAD  VIYNGELDVH  FPYLTVKQTL  DFAIACKTP-
(PRED) sace_16_1_a0238 GEVAYDGIPQ  EEMMKRYKAD  VIYNGELDVH  FPYLTVKQTL  DFAIACKTP-
(PRED) sace_45_1_a0242 GEVAYDGIPQ  EEMMKRYKAD  VIYNGELDVH  FPYLTVKQTL  DFAIACKTP-
(PRED) sace_48_1_a0238 GEVAYDGIPQ  EEMMKRYKAD  VIYNGELDVH  FPYLTVKQTL  DFAIACKTP-
(PRED) sace_60_4_d0244 GEVAYDGIPQ  EEMMKRYKAD  VIYNGELDVH  FPYLTVKQTL  DFAIACKTP-
(PRED) sace_52_1_a0240 GEVAYDGIPQ  EEMMKRYKAD  VIYNGELDVH  FPYLTVKQTL  DFAIACKTP-
(PRED) sace_46_1_a0240 GEVAYDGIPQ  EEMMKRYKAD  VIYNGELDVH  FPYLTVKQTL  DFAIACKTP-
(PRED) sace_25_1_a0240 GEVAYDGIPQ  EEMMKRYKAD  VIYNGELDVH  FPYLTVKQTL  DFAIACKTP-
(PRED) sace_24_1_2300  GEVAYDGIPQ  EEMMKRYKAD  VIYNGELDVH  FPYLTVKQTL  DFAIACKTP-
(PRED) sace_47_1_a0240 GEVAYDGIPQ  EEMMKRYKAD  VIYNGELDVH  FPYLTVKQTL  DFAIACKTP-
(PRED) sace_7_1_a02410 GEVAYDGIPQ  EEMMKRYKAD  VIYNGELDVH  FPYLTVKQTL  DFAIACKTP-
(PRED) sace_59_110_df0 GEVAYDGIPQ  EEMMKRYKAD  VIYNGELDVH  FPYLTVKQTL  DFAIACKTP-
(PRED) sace_56_1_a0202 GEVAYDGIPQ  EEMMKRYKAD  VIYNGELDVH  FPYLTVKQTL  DFAIACKTP-
(PRED) sace_40_1_a0239 GEVAYDGIPQ  EEMMKRYKAD  VIYNGELDVH  FPYLTVKQTL  DFAIACKTP-
(PRED) sace_15_1_a0242 GEVAYDGIPQ  EEMMKRYKAD  VIYNGELDVH  FPYLTVKQTL  DFAIACKTP-
(PRED) sace_37_1_a0243 GEVAYDGIPQ  EEMMKRYKAD  VIYNGELDVH  FPYLTVKQTL  DFAIACKTP-
(PRED) sace_9_1_a02440 GEVAYDGIPQ  EEMMKRYKAD  VIYNGELDVH  FPYLTVKQTL  DFAIACKTP-
(PRED) sace_22_1_2300  GEVAYDGIPQ  EEMMKRYKAD  VIYNGELDVH  FPYLTVKQTL  DFAIACKTP-
(PRED) sace_29_1_2290  GEVAYDGIPQ  EEMMKRYKAD  VIYNGELDVH  FPYLTVKQTL  DFAIACKTP-
(PRED) sace_34_1_2320  GEVAYDGIPQ  EEMMKRYKAD  VIYNGELDVH  FPYLTVKQTL  DFAIACKTP-
(PRED) sace_58_25_y007 GEVAYDGIPQ  EEMMKRYKAD  VIYNGELDVH  FPYLTVKQTL  DFAIACKTP-
(PRED) sace_23_1_2290  GEVAYDGIPQ  EEMMKRYKAD  VIYNGELDVH  FPYLTVKQTL  DFAIACKTP-
(PRED) sace_6_120_dp00 GEVAYDGIPQ  EEMMKRYKAD  VIYNGELDVH  FPYLTVKQTL  DFAIACKTP-
(PRED) sace_57_1_a0241 GEVAYDGIPQ  EEMMKRYKAD  VIYNGELDVH  FPYLTVKQTL  DFAIACKTP-
(PRED) sace_17_1_a0241 GEVAYDGIPQ  EEMMKRYKAD  VIYNGELDVH  FPYLTVKQTL  DFAIACKTP-
(PRED) sace_21_1_2310  GEVAYDGIPQ  EEMMKRYKAD  VIYNGELDVH  FPYLTVKQTL  DFAIACKTP-
(PRED) sace_49_1_a0246 GEVAYDGIPQ  EEMMKRYKAD  VIYNGELDVH  FPYLTVKQTL  DFAIACKTP-
(PRED) sace_8_2_b02430 GEVAYDGIPQ  EEMMKRYKAD  VIYNGELDVH  FPYLTVKQTL  DFAIACKTP-
(PRED) sace_31_1_2300  GEVAYDGIPQ  EEMMKRYKAD  VIYNGELDVH  FPYLTVKQTL  DFAIACKTP-
(PRED) sace_50_1_a0241 GEVXYDGIPQ  EEMMKRYKAD  VIYNGELDVH  FPYLTVKQTL  DFAIACKTP-
(PRED) sace_4_1_a02360 GEVAYDGIPQ  EEMMKRYKAD  VIYNGELDVH  FPYLTVKQTL  DFAIACKTP-
(PRED) sace_2_1_a02390 GEVAYDGIPQ  EEMMKRYKAD  VIYNGELDVH  FPYLTVKQTL  DFAIACKTP-
(PRED) sace_5_33_ag005 GEVAYDGIPQ  EEMMKRYKAD  VIYNGELDVH  FPYLTVKQTL  DFAIACKTP-
(PRED) sapa_11_1_a0247 GDVAYDGIPQ  EEMMKRYKAD  VIYNGELDVH  FPYLTVKQTL  DFAIACKTP-
(PRED) sapa_25_1_a0246 GDVAYDGIPQ  EEMMKRYKAD  VIYNGELDVH  FPYLTVKQTL  DFAIACKTP-
(PRED) sapa_4_1_a02470 GDVAYDGIPQ  EEMMKRYKAD  VIYNGELDVH  FPYLTVKQTL  DFAIACKTP-
(PRED) sapa_5_1_2350   GDVAYDGIPQ  EEMMKRYKAD  VIYNGELDVH  FPYLTVKQTL  DFAIACKTP-
(PRED) sapa_9_1_2360   GDVAYDGIPQ  EEMMKRYKAD  VIYNGELDVH  FPYLTVKQTL  DFAIACKTP-
(PRED) sapa_14_1_a0244 GDVAYDGIPQ  EEMMKRYKAD  VIYNGELDVH  FPYLTVKQTL  DFAIACKTP-
(PRED) sapa_8_1_2350   GDVAYDGIPQ  EEMMKRYKAD  VIYNGELDVH  FPYLTVKQTL  DFAIACKTP-
(PRED) sapa_17_1_2380  GDVAYDGIPQ  DEMMKRYKAD  VIYNGELDVH  FPYLTVKQTL  DFAIACKTP-
(PRED) sapa_7_1_2370   GDVAYDGIPQ  DEMMKRYKAD  VIYNGELDVH  FPYLTVKQTL  DFAIACKTP-
(PRED) sapa_2_1_a02460 GDVAYDGIPQ  DEMMKRYKAD  VIYNGELDVH  FPYLTVKQTL  DFAIACKTP-
(PRED) sapa_23_1_a0248 GDVAYDGIPQ  DEMMKRYKAD  VIYNGELDVH  FPYLTVKQTL  DFAIACKTP-
(PRED) sapa_3_1_a02470 GDVAYDGIPQ  DEMMKRYKAD  VIYNGELDVH  FPYLTVKQTL  DFAIACKTP-
(PRED) sapa_18_1_2390  GDVAYDGIPQ  EEMMKRYKAD  IIYNGELDVH  FPYLTVKQTL  DFAIACKTP-
(PRED) sami_1_4_244    GDITYDGIPQ  KEMMKRYKAD  VIYNGELDVH  FPYLTVKQTL  DFAIACKTP-
(PRED) saku_1_4_262    GDIAYDGIPQ  KEMMKRYKAD  VIYNGELDVH  FPYLTVKQTL  DFAIACKTP-
(PRED) saba_1_58_bf002 GDVAYDGIPQ  EEMMKRYKAD  VIYNGELDVH  FPYLTVKQTL  DFAIACKTP-
(PRED) saeu_1_4_d02400 GEVSYDGIPQ  KEMMKKYKAD  VIYNGELDVH  FPYLTVKQTL  DFAIACKTP-
(PRED) naca_1_e01640   GEIAYDGIPQ  DEMMKKYRAD  VIYNGELDVH  FPYLTVQQTL  DFAIACKTP-
(PRED) nada_1_g01850   GDITYDGIPQ  KEMMKHYKAD  VIYNGELDVH  FPFLTVQQTL  DFAIACKTP-
(PRED) naca_1_e01630   GEITYDGIPQ  KEMMKHYKSD  VIYNGELDVH  FPHLTVQQTL  DFALACKTP-
(PRED) nada_1_g01840   GEMTYDGISQ  KEMMQKFKGD  VIYNGEQDVH  FPHITVQQTL  DFAIACKTP-
(PRED) kaaf_1_c00820   GDVSYDGIPQ  KEMMKKYKSD  VIYNGEVDVH  FPYLTVQQTL  DFAIACKTP-
(PRED) teph_1_m00640   GDISYDGIPQ  EEMMKKFKSD  VIYNGELDVH  FPYLTVKQTL  DFAIACKIP-
(PRED) vapo_1_1036_28  GDVSYDGIYQ  DEMMKNYKSD  VIYNGELDVH  FPYLTVKQTL  DFAIACKTP-
(PRED) tebl_1_i01760   GDISYDGIDQ  KEMMKHFRSD  VIYNGELDVH  FPYLTVQQTL  DFAIACRTP-
(PRED) tode_1_d04040   GDISYNGISQ  DEIMKDFRSD  VIYNGELDVH  FPYLTVKQTL  DFAIACKTP-
(PRED) naca_1_e01650   GDIAYDGIPQ  DEMMKKYKGD  VIYNGELDVH  FPFLTVQQTL  DFAIACKTP-
(PRED) tebl_1_g02820   GEIYYDGIPQ  SEMMKNYKAD  VIYNGELDVH  FPYLTVQQTL  DFAIACKTP-
(PRED) lakl_1_c11616g  GDISYDGIPQ  KDMMKKYKPD  VIYNGEMDVH  FPHLTVKQTL  DFALACKVP-
(PRED) saar_1_8_h03780 GDISYDGIPQ  KEMMQHYKSD  VIYNGEQDVH  FPYLTVKETL  DFAIACKMP-
(PRED) sace_14_7_g0015 GHISYDGIPQ  KEMMQHYKPD  VIYNGEQDVH  FPHLTVKQTL  DFAISCKMP-
(PRED) sace_15_7_g0387 GHISYDGIPQ  KEMMQHYKPD  VIYNGEQDVH  FPHLTVKQTL  DFAISCKMP-
```

(PRED)	sace_24_8_3780	GHISYDGIPQ	KEMMQHYKPD	VIYNGEQDVH	FPHLTVKQTL	DFAISCKMP-
(PRED)	sace_40_8_h0383	GHISYDGIPQ	KEMMQHYKPD	VIYNGEQDVH	FPHLTVKQTL	DFAISCKMP-
(PRED)	sace_6_169_fm00	GHISYDGIPQ	KEMMQHYKPD	VIYNGEQDVH	FPHLTVKQTL	DFAISCKMP-
(PRED)	sace_19_7_3840	GHISYDGIPQ	KEMMQHYKPD	VIYNGEQDVH	FPHLTVKQTL	DFAISCKMP-
(PRED)	sace_32_7_3770	GHISYDGIPQ	KEMMQHYKPD	VIYNGEQDVH	FPHLTVKQTL	DFAISCKMP-
(PRED)	sace_56_17_q011	GHISYDGIPQ	KEMMQHYKPD	VIYNGEQDVH	FPHLTVKQTL	DFAISCKMP-
(PRED)	sace_5_78_bz001	GHISYDGIPQ	KEMMQHYKPD	VXYNGEQDVH	FPHLTVKQTL	DFAFXCKMP-
(PRED)	sace_2_8_h03860	GHISYDGIPQ	KEMMQHYKPD	VIYNGEQDVH	FPHLTVKQTL	DFAISCKMP-
(PRED)	sace_53_29_ac00	GHISYDGIPQ	KEMMQHYKPD	VIYNGEQDVH	FPHLTVKQTL	DFAISCKMP-
(PRED)	sace_17_7_g0393	GHISYDGIPQ	KEMMQHYKPD	VIYNGEQDVH	FPHLTVKQTL	DFAISCKMP-
(PRED)	sace_25_7_g0388	GHISYDGIPQ	KEMMQHYKPD	VIYNGEQDVH	FPHLTVKQTL	DFAISCKMP-
(PRED)	sace_37_7_g0385	GHISYDGIPQ	KEMMQHYKPD	VIYNGEQDVH	FPHLTVKQTL	DFAISCKMP-
(PRED)	sace_9_7_g00180	GHISYDGIPQ	KEMMQHYKPD	VIYNGEQDVH	FPHLTVKQTL	DFAISCKMP-
(PRED)	sace_60_6_f0335	GHISYDGIPQ	KEMMQHYKPD	VIYNGEQDVH	FPHLTVKQTL	DFAISCKMP-
(PRED)	sace_59_336_lx0	GHISYDGIPQ	KEMMQHYKPD	VIYNGEQDVH	FPHLTVKQTL	DFAISCKMP-
(PRED)	sace_31_7_3780	GHISYDGIPQ	KEMMQHYKPD	VIYNGEQDVH	FPHLTVKQTL	DFAISCKMP-
(PRED)	sace_34_8_3770	GHISYDGIPQ	KEMMQHYKPD	VIYNGEQDVH	FPHLTVKQTL	DFAISCKMP-
(PRED)	sace_58_71_bs00	GHISYDGIPQ	KEMMQHYKPD	VIYNGEQDVH	FPHLTVKQTL	DFAISCKMP-
(PRED)	sace_7_7_g03880	GHISYDGIPQ	KEMMQHYKPD	VIYNGEQDVH	FPHLTVKQTL	DFAISCKMP-
(PRED)	sace_35_7_3840	GHISYDGIPQ	KEMMQHYKPD	VIYNGEQDVH	FPHLTVKQTL	DFAISCKMP-
(PRED)	sace_43_7_g0387	GHISYDGIPQ	KEMMQHYKPD	VIYNGEQDVH	FPHLTVKQTL	DFAISCKMP-
(PRED)	sace_57_8_h0390	GHISYDGIPQ	KEMMQHYKPD	VIYNGEQDVH	FPHLTVKQTL	DFAISCKMP-
(PRED)	sace_45_7_g0389	GHISYDGIPQ	KEMMQHYKPD	VIYNGEQDVH	FPHLTVKQTL	DFAISCKMP-
(PRED)	sace_46_8_h0391	GHISYDGIPQ	KEMMQHYKPD	VIYNGEQDVH	FPHLTVKQTL	DFAISCKMP-
(PRED)	sace_23_7_3860	GHISYDGIPQ	KEMMQHYKPD	VIYNGEQDVH	FPHLTVKQTL	DFAISCKMP-
(PRED)	sace_21_7_3790	GHISYDGIPQ	KEMMQHYKPD	VIYNGEQDVH	FPHLTVKQTL	DFAFYCKMP-
(PRED)	sace_8_73_bu001	GHISYDGIPQ	KEMMQHYKPD	VIYNGEQDVH	FPHLTVKQTL	DFAISCKMP-
(PRED)	sapa_1_8_h03820	GHISYDGIPQ	KEMMQHYKSD	VIYNGEQDVH	FPHLTVKQTL	DFAISCKMP-
(PRED)	sapa_21_8_h0387	GHISYDGIPQ	KEMMQHYKSD	VIYNGEQDVH	FPHLTVKQTL	DFAISCKMP-
(PRED)	sapa_20_8_h0386	GHISYDGIPQ	KEMMQHYKSD	VIYNGEQDVH	FPHLTVKQTL	DFAISCKMP-
(PRED)	sapa_22_8_h0390	GHISYDGIPQ	KEMMQHYKSD	VIYNGEQDVH	FPHLTVKQTL	DFAISCKMP-
(PRED)	sapa_25_8_h0387	GHISYDGIPQ	KEMMQHYKSD	VIYNGEQDVH	FPHLTVKQTL	DFAISCKMP-
(PRED)	sapa_6_8_3750	GHISYDGIPQ	KEMMQHYKSD	VIYNGEQDVH	FPHLTVKQTL	DFAISCKMP-
(PRED)	sapa_9_8_3720	GHISYDGIPQ	KEMMQHYKSD	VIYNGEQDVH	FPHLTVKQTL	DFAISCKMP-
(PRED)	sapa_19_8_h0390	GHISYDGIPQ	KEMMQHYKSD	VIYNGEQDVH	FPHLTVKQTL	DFAISCKMP-
(PRED)	sapa_24_8_h0385	GHISYDGIPQ	KEMMQHYKSD	VIYNGEQDVH	FPHLTVKQTL	DFAISCKMP-
(PRED)	sapa_4_8_h03850	GDISYDGIPQ	KEMMQHYKSD	VIYNGEQDVH	FPHLTVKQTL	DFAISCKMP-
(PRED)	sapa_10_8_3760	GHISYDGIPQ	KEMMQHYKSD	VIYNGEQDVH	FPHLTVKQTL	DFAISCKMP-
(PRED)	sapa_13_8_h0382	GHISYDGIPQ	KEMMQHYKSD	VIYNGEQDVH	FPHLTVKQTL	DFAISCKMP-
(PRED)	sapa_8_8_3750	GHISYDGIPQ	KEMMQHYKSD	VIYNGEQDVH	FPHLTVKQTL	DFAISCKMP-
(PRED)	sapa_11_8_h0383	GHISYDGIPQ	KEMMQHYKSD	VIYNGEQDVH	FPHLTVKQTL	DFAISCKMP-
(PRED)	sapa_5_8_3700	GHISYDGIPQ	KEMMQHYKSD	VIYNGEQDVH	FPHLTVKQTL	DFAISCKMP-
(PRED)	sapa_16_8_h0389	GHISYDGIPQ	KEMMQHYKSD	VIYNGEQDVH	FPHLTVKQTL	DFAISCKMP-
(PRED)	sapa_17_8_3730	GHISYDGIPQ	KEMMQHYKSD	VIYNGEQDVH	FPHLTVKQTL	DFAISCKMP-
(PRED)	sapa_2_8_h03860	GHISYDGIPQ	KEMMQHYKSD	VIYNGEQDVH	FPHLTVKQTL	DFAISCKMP-
(PRED)	sapa_7_8_3740	GHISYDGIPQ	KEMMQHYKSD	VIYNGEQDVH	FPHLTVKQTL	DFAISCKMP-
(PRED)	sapa_23_8_h0385	GHISYDGIPQ	KEMMQHYKSD	VIYNGEQDVH	FPHLTVKQTL	DFAISCKMP-
(PRED)	sapa_3_8_h03890	GHISYDGIPQ	KEMMQHYKSD	VIYNGEQDVH	FPHLTVKQTL	DFAISCKMP-
(PRED)	sapa_18_8_3730	GHISYDGIPQ	KEMMQHYKSD	VIYNGEQDVH	FPHLTVKQTL	DFAISCKMP-
(PRED)	sami_1_14_399	GAISYDGIPQ	KEMMRHYKSD	VIYNGEQDVH	FPHLTVKQTL	DFAIGCKMP-
(PRED)	sace_4_8_h03690	GHISYDGIPQ	KEMMQHYKPD	VXYNGEQDVH	FPHLTVKQTL	DFAFSVRCP-
(PRED)	saku_1_14_404	GDISYDGISQ	KEMMQHYKAD	VIYNGEQDVH	FPYLTVKQTL	DFAIACKMP-
(PRED)	sace_1_ynr070w	GHISYDGIPQ	KEMMQHYKPD	VIYNGEQDVH	FPHLTVKQTL	DFAISCKMP-
(PRED)	sace_49_8_h0383	GHISYDGIPQ	KEMMQHYKPD	VIYNGEQDVH	FPHLTVKQTL	DFAISCKMP-
(PRED)	saeu_1_2_b00130	GDISYDGIPQ	EEMMKCYKSD	VIYNGELDVH	FPYLTVKQTL	DFAIACKMP-
(PRED)	sauv_1_7_3	GDISYDGIPQ	EEMMKHYKSD	VIYNGELDVH	FPYLTVKQTL	DFAIACKMP-
(PRED)	sami_1_17_26	GDISYDGIPQ	DEMMRRYKSD	VIYNGELDVH	FPYLTVKQTL	DFAIACKTP-
(PRED)	zyba_1_02055_AN	GEISYDGIPQ	HTMMRHYRSD	VIYNGELDVH	FPYLTVRQTL	DFAIACKTP-
(PRED)	zyba_1_07912	GEISYDGIPQ	HTMMRHYRSD	VIYNGELDVH	FPYLTVRQTL	DFAIACKTP-
(PRED)	zyba_2_2_b00600	GEISYDGIPQ	HTMMRHYRSD	VIYNGELDVH	FPYLTVRQTL	DFAIACKTP-
(PRED)	zyba_3_3_c03460	GEISYDGIPQ	HTMMRHYRSD	VIYNGELDVH	FPYLTVRQTL	DFAIACKTP-
(PRED)	zyba_1_04634	GDISYDGVPQ	EIMMKSYKSS	VIYNGEMDVH	FPYLTVKQTL	DFAIACKTP-
(PRED)	zyba_1_06675	GDISYDGVPQ	ETMMKKYKSN	VIYNGEMDVH	FPYLTVKQTL	DFAIACKTP-
(PRED)	zyba_3_2_b02230	GDISYDGVPQ	ETMMKKYKSN	VIYNGEMDVH	FPYLTVKQTL	DFAIACKTP-
(PRED)	zyba_2_1_a00860	GDISYDGVPQ	ETMMKKYKSN	VIYNGEMDVH	FPYLTVKQTL	DFAIACKTP-
(PRED)	zyro_1_a04114g	GDIMYDGVPQ	DTMMKHYRSD	VIYNGELDVH	FPYLTVKQTL	DFAIACKTP-
(PRED)	zyro_1_b14762g	GEILYDGIPQ	NVMMKNYKSD	VIYNPELDVH	FPFLTVKQTL	DFAIACKTP-
(PRED)	zyba_2_14_n0149	GHISYDGVPQ	ATMLKQYRSD	LIYNGELDVH	FPYLTVKQTL	DFAIACKTP-
(PRED)	zyba_2_33_ag001	GHISYDGVPQ	ARMLKQYRSD	LIYNGELDVH	FPYLTVKQTL	DFAIACKTP-
(PRED)	lath_1_a01914g	GEISYDGISQ	KEMMAKFKTD	VIYNGETDVH	FPHLTVQQTL	DFALSCTTP-
(PRED)	lawa_1_23_5161	GSISYDGIPQ	EEMMKKYKTD	VIYNGEMDVH	FPHLTVQQTL	DFALSCTTP-
(PRED)	klae_1_14_n0012	GDIRYDGVDQ	KEMLKHFKSD	VIYNGEMDVH	FPHLTVQQTI	DFAIACKTP-
(PRED)	klla_1_d03432g	GDISYDGVSQ	KDMLKYFKSD	VIYNGEMDVH	FPHLTVQQTL	DFAVACKTP-
(PRED)	klma_1_1_a01880	GSISYDGVPL	KDMLKYFKAD	VIYNGEMDVH	FPHLTVQQTL	DFAVACKTP-
(PRED)	klwi_1_33_ag001	GDILYDGVPQ	KEMLKHFKSD	VIYNGEMDVH	FPHLTVKQTL	DFALACKTP-

```
(PRED) teph_1_a04220  GTVLYEGISQ  EEMIKKYKSD  LIYNGEDDVH  FPHLTVQQTL  DFAISCKIP-
(PRED) vapo_1_1037_47 GSVTYDGIPQ  KEMVKNYKSD  LIYCAETDIH  FPHLTVKQTL  DFALACKVQ-
(PRED) pata_1_2_b05590 GDITYNNISQ  PEMMKNFKKD  VIYNPELDVH  FPFLTVNQTL  RFAIGCRTP-
(PRED) wian_1_3_c04380 GEVHYDHISQ  HEMMKKYKSD  VIYNGELDIH  YPHLTVDETF  RFAIACKTP-
(PRED) wian_1_3_c04390 GDIHYDHIPQ  KEMMKKYKSD  VVYNGELDIH  FPHLTVDKTL  RFAIACKTP-
(PRED) wian_1_7_g01010 GDIHYDHIPQ  HEMAKKYKSD  IIYNGELDIH  FPHLTVDKTL  KFAIACKTP-
(PRED) bain_1_1_a00100 GNVLYDGIPQ  EDMLRDFKSD  LIYNPELDNH  FPHLTVDQTL  RFAIACKTP-
(PRED) bain_1_17_q0038 GNVLYDGIPQ  ADMLRDFKSD  LIYNPELENH  FPHLTVDQTL  RFAIACKTP-
(PRED) bain_1_8_h00410 GDISYNGLPQ  SEMMKYFRSD  VLYNPEYDVH  FPHLNVDQTL  RFAIGCKTP-
(PRED) caal_1_19_5759 GDIRYDGLPQ  KEMLKLFKND  LVYNPELDVH  FPHLTVDQTL  TFAIACKTP-
(PRED) caal_11_25_y002 GDIRYDGLPQ  KEMLKLFKND  LVYNPELDVH  FPHLTVDQTL  TFAIACKTP-
(PRED) caal_4_4_d03320 GDIRYDGLPQ  KEMLKLFKND  LVYNPELDVH  FPHLTVDQTL  TFAIACKTP-
(PRED) caal_12_26_z005 GDIRYDGLPQ  KEMLKLFKND  LVYNPELDVH  FPHLTVDQTL  TFAIACKTP-
(PRED) caal_5_30_ad005 GDIRYDGLPQ  KEMLKLFKND  LVYNPELDVH  FPHLTVDQTL  TFAIACKTP-
(PRED) caal_8_3_c03320 GDIRYDGLPQ  KEMLKLFKND  LVYNPELDVH  FPHLTVDQTL  TFAIACKTP-
(PRED) caal_6_4_d03280 GDIRYDGLPQ  KEMLKLFKND  LVYNPELDVH  FPHLTVDQTL  TFAIACKTP-
(PRED) caal_10_3_c0334 GDIRYDGLPQ  KEMLKLFKND  LVYNPELDVH  FPHLTVDQTL  TFAIACKTP-
(PRED) caal_3_29_ac005 GDIRYDGLPQ  KEMLKLFKND  LVYNPELDVH  FPHLTVDQTL  TFAIACKTP-
(PRED) caal_2_04989   GDIRYDGLPQ  KEMLKLFKND  LVYNPELDVH  FPHLTVDQTL  TFAIACKTP-
(PRED) cadu_1_64350   GDIRYDGLPQ  SEMLKLFKND  LVYNPELDVH  FPHLTVDQTL  TFAIACKTP-
(PRED) caor_1_h02090  GDIRYDGLTQ  HEMLNNYKND  LVYNPELDVH  FPHLTVDQTL  SFAIGCKTP-
(PRED) capa_1_600750  GDVRYDGLTQ  HEMLNNYKND  LVYNPELDVH  FPHLTVDQTL  SFAIGCKTP-
(PRED) loel_1_04930   GDIRYDGLSQ  KEMIKHFKND  LVYNPELDVH  FPHLTVDQTL  SFAIGCKTP-
(PRED) spar_1_5_e03260 GEILYDGISQ  SEMLKSFRND  LIYNPELDCH  FPHLTVDQTL  TFALSCKTP-
(PRED) sppa_1_7_g03160 GEVLYDGIHQ  SEMLKSFKND  LIYNPELDCH  FPHLTVDQTL  TFALSCKTP-
(PRED) catr_1_01205   GDIRYDGLPQ  KEMIKMFKND  LIYNPELDTH  FPHLTVDETL  TFAIGCKTP-
(PRED) catr_1_05498   GDIRYDGLPQ  NEMIKMFRND  LIYNPELDIH  FPHLTVDQTL  SFAIACKTP-
(PRED) catr_1_05971   GDVRYDGLPQ  KEMIKMFKND  LIYNPELDVH  FPHLTVDQTL  SFAIACKTP-
(PRED) deha_1_a03696g GDIRYDGITQ  KEMLKNFKND  LVYVPELDVH  FPHLTVEQTL  RFAIACKTP-
(PRED) deha_2_5_e00720 GDIRYDGITQ  KEMLKNFKND  LVYVPELDVH  FPHLTVEQTL  KFAIACKTP-
(PRED) scst_1_3_c02890 GDIRYDGIDQ  KTMLKNFKSE  LIYNPELDIH  FPHLTVEQTL  KFAIACKTP-
(PRED) mebi_1_8_h00300 GEIVYDGIER  DEMWKHFSSD  LIYNPELDVH  FPHLTVKQTL  DFALACKTP-
(PRED) lakl_1_h21010g GEISYDGIPQ  KEMIKHFKGD  IIYNGEMDVH  FPHLTVSQTL  EMAIACKEPH
(PRED) caar_1_13_m0142 GSVDFSGIPL  ETMMKRYKTQ  IIYNPELDVH  FPYLTVEQTI  KFAIGCKTP-
(PRED) caar_1_14_n0143 GSIVYNGIEQ  KEMLKRFENQ  LIYNPELDVH  YPYLTVEQTI  KFAIGCKTP-
(PRED) hapo_1_1_a07220 GEVIYNGISQ  KDMMKSFKNQ  VIYNPELDVH  YPYLTVEQTM  NFAIGCKTP-
(PRED) ogpa_1_1_a01680 GEVIYNGISQ  KDMMKSFKNQ  VIYNPELDVH  YPYLTVEQTM  NFAIGCKTP-
(PRED) piku_1_96_cr001 GEIFYGGIPS  EEMFKKHKDQ  LIYNPELDVH  FPFLTVEQTL  NFAIGCKTP-
(PRED) pime_1_4_d03240 GDVHYGGIES  KDMFKEHKNQ  LIYNPELDVH  FPYLTVEQTM  QFAIGCKTP-
(PRED) pime_1_1_a12110 GELLYGGIDS  KQMFKEFQNQ  LIYNPELDVH  LPYLTVEQTL  NFAIGCKTP-
(PRED) piku_1_227_hs00 GELAFNGIPS  NQLFKFFKNL  CVYNPELDVH  LPHLTVGETL  AFAISCKTP-
(PRED) pime_1_5_e05800 GQLSFSGIDQ  KEMFRFFKNL  LIYNPELDVH  FPYLTVGQTL  KFAIGCKTP-
(PRED) pime_1_1_a07690 GTLTFNGIDC  QKIFDNLSNT  LIYNPELDVH  FPFLTVEQTI  RFAVACKTP-
(PRED) debr_2_5_e03380 GVVSYGGIPQ  KDLVQQFASQ  LVYVPELDEH  FPYLTVEQTL  EFAIACKTP-
(PRED) kopa_1_2_b10040 GDIAYNNIPQ  AEMVKRFKNE  LIYNPELDLH  FPHLTVEETL  SFALACKTP-
(PRED) kopa_2_7_g00500 GEIAYNNIPQ  AEMVKRFKNE  LIYNPELDLH  FPHLTVEETL  SFALACKTP-
(PRED) asru_1_13_m0119 GDISFDGIPH  DIMMKDFKAD  VVYNPELDVH  FPHLTVDQTL  KFAISCKIP-
(PRED) asru_1_15_o0045 ADIKYDTIPQ  DQMMKYFKAD  VIYNPENDAH  FPHLTVEQTL  RFAITCKVP-
(PRED) wian_1_1_a02920 GEILYNGIPQ  DEMVKNFKSD  LIYNPEHDEH  FPHLTVEQTL  KFAVACRTP-
(PRED) wian_1_1_a02930 GEIFYNGIPQ  DEMVKNFKSD  LIYNPEHDEH  FPHLTVEQTL  KFAVACRTP-

                      .........510 .........520 .........530 .........540 .........550
(PRED) asac_1_6_f03560 HVRVNNVSRA  EYITLMRELY  ATVFGLRHTY  NTKVGDDYIR  GVSGGERKRV
(PRED) ergo_1_abr125c HMRVNNVSRA  EYITLMRELY  ATVFGLRHTY  DTKVGNDYIR  GVSGGERKRV
(PRED) ercy_1_3604    HVRVNSLTRA  DYISMMRELY  ATIFGLKHAY  DTPVGNEFVR  GVSGGERKRV
(PRED) cagl_1_i04862g SKRVNDVSRE  EYIASTRDLH  ATIFGLRHTY  HTKVGNDFVR  GVSGGERKRV
(PRED) kaaf_1_c00830  AKRINNISKS  EYIESTRDLY  ATIFGLRHTY  QTKVGNDFVR  GVSGGERKRV
(PRED) kana_1_k01350  AKRVNNVSRS  EYIASTRELY  ATIFGLRHTY  HTKVGNDFVR  GVSGGERKRV
(PRED) saar_1_2_b02590 AIRVNNVPKN  QYIASMRDLY  ATIFGLRHTY  NTKVGNDLVR  GVSGGERKRV
(PRED) sace_1_ydr011w ALRVNNVSKK  EYIASRRDLY  ATIFGLRHTY  NTKVGNDFVR  GVSGGERKRV
(PRED) sace_16_1_a0238 ALRVNNVSKK  EYIASRRDLY  ATIFGLRHTY  NTKVGNDFVR  GVSGGERKRV
(PRED) sace_45_1_a0242 ALRVNNVSKK  EYIASRRDLY  ATIFGLRHTY  NTKVGNDFVR  GVSGGERKRV
(PRED) sace_48_1_a0238 ALRVNNVSKK  EYIASRRDLY  ATIFGLRHTY  NTKVGNDFVR  GVSGGERKRV
(PRED) sace_60_4_d0244 ALRVNNVSKK  EYIASRRDLY  ATIFGLRHTY  NTKVGNDFVR  GVSGGERKRV
(PRED) sace_52_1_a0240 ALRVNNVSKK  EYIASRRDLY  ATIFGLRHTY  NTKVGNDFVR  GVSGGERKRV
(PRED) sace_46_1_a0240 ALRVNNVSKK  EYIASRRDLY  ATIFGLRHTY  NTKVGNDFVR  GVSGGERKRV
(PRED) sace_25_1_a0240 ALRVNNVSKK  EYIASRRDLY  ATIFGLRHTY  NTKVGNDFVR  GVSGGERKRV
(PRED) sace_24_1_2300 ALRVNNVSKK  EYIASRRDLY  ATIFGLRHTY  NTKVGNDFVR  GVSGGERKRV
(PRED) sace_47_1_a0240 ALRVNNVSKK  EYIASRRDLY  ATIFGLRHTY  NTKVGNDFVR  GVSGGERKRV
(PRED) sace_7_1_a02410 ALRVNNVSKK  EYIASRRDLY  ATIFGLRHTY  NTKVGNDFVR  GVSGGERKRV
(PRED) sace_59_110_df0 ALRVNNVSKK  EYIASRRDLY  ATIFGLRHTY  NTKVGNDFVR  GVSGGERKRV
(PRED) sace_56_1_a0202 ALRVNNVSKK  EYIASRRDLY  ATIFGLRHTY  NTKVGNDFVR  GVSGGERKRV
(PRED) sace_40_1_a0239 ALRVNNVSKK  EYIASRRDLY  ATIFGLRHTY  NTKVGNDFVR  GVSGGERKRV
(PRED) sace_15_1_a0242 ALRVNNVSKK  EYIASRRDLY  ATIFGLRHTY  NTKVGNDFVR  GVSGGERKRV
(PRED) sace_37_1_a0243 ALRVNNVSKK  EYIASRRDLY  ATIFGLRHTY  NTKVGNDFVR  GVSGGERKRV
```

(PRED)	sace_9_1_a02440	ALRVNNVSKK	EYIASRRDLY	ATIFGLRHTY	NTKVGNDFVR	GVSGGERKRV
(PRED)	sace_22_1_2300	ALRVNNVSKK	EYIASRRDLY	ATIFGLRHTY	NTKVGNDFVR	GVSGGERKRV
(PRED)	sace_29_1_2290	ALRVNNVSKK	EYIASRRDLY	ATIFGLRHTY	NTKVGNDFVR	GVSGGERKRV
(PRED)	sace_34_1_2320	ALRVNNVSKK	EYIASRRDLY	ATIFGLRHTY	NTKVGNDFVR	GVSGGERKRV
(PRED)	sace_58_25_y007	ALRVNNVSKK	EYIASRRDLY	ATIFGLRHTY	NTKVGNDFVR	GVSGGERKRV
(PRED)	sace_23_1_2290	ALRVNNVSKK	EYIASRRDLY	ATIFGLRHTY	NTKVGNDFVR	GVSGGERKRV
(PRED)	sace_6_120_dp00	ALRVNNVSKK	EYIASRRDLY	ATIFGLRHTY	NTKVGNDFVR	GVSGGERKRV
(PRED)	sace_57_1_a0241	ALRVNNVSKK	EYIASRRDLY	ATIFGLRHTY	NTKVGNDFVR	GVSGGERKRV
(PRED)	sace_17_1_a0241	ALRVNNVSKK	EYIASRRDLY	ATIFGLRHTY	NTKVGNDFVR	GVSGGERKRV
(PRED)	sace_21_1_2310	ALRVNNVSKK	EYIASRRDLY	ATIFGLRHTY	NTKVGNDFVR	GVSGGERKRV
(PRED)	sace_49_1_a0246	ALRVNNVSKK	EYIASRRDLY	ATIFGLRHTY	NTKVGNDFVR	GVSGGERKRV
(PRED)	sace_8_2_b02430	ALRVNNVSKK	EYIASRRDLY	ATIFGLRHTY	NTKVGNDFVR	GVSGGERKRV
(PRED)	sace_31_1_2300	ALRVNNVSKK	EYIASRRDLY	ATIFGLRHTY	NTKVGNDFVR	GVSGGERKRV
(PRED)	sace_50_1_a0241	ALRVNNVSKK	EYIASRRDLY	ATIFGLRHTY	NTKVGNDFVR	GVSGGERKRV
(PRED)	sace_4_1_a02360	ALRVNNVSKK	EYIASRRDLY	ATIFGLRHTY	NTKVGNDFVR	GVSGGERKRV
(PRED)	sace_2_1_a02390	ALRVNNVSKK	EYIASRRDLY	ATIFGLRHTY	NTKVGNDFVR	GVSGGERKRV
(PRED)	sace_5_33_ag005	ALRVNXVSKK	EYIASRRDLY	ATIFGLRHTY	NTKVGNDFVR	GVSGGERKRV
(PRED)	sapa_11_1_a0247	ALRVNNVSKK	EYIASRRDLY	ATIFGLRHTY	NTKVGNDFVR	GVSGGERKRV
(PRED)	sapa_25_1_a0246	ALRVNNVSKK	EYIASRRDLY	ATIFGLRHTY	NTKVGNDFVR	GVSGGERKRV
(PRED)	sapa_4_1_a02470	ALRVNNVSKK	EYIASRRDLY	ATIFGLRHTY	NTKVGNDFVR	GVSGGERKRV
(PRED)	sapa_5_1_2350	ALRVNNVSKK	EYIASRRDLY	ATIFGLRHTY	NTKVGNDFVR	GVSGGERKRV
(PRED)	sapa_9_1_2360	ALRVNNVSKK	EYIASRRDLY	ATIFGLRHTY	NTKVGNDFVR	GVSGGERKRV
(PRED)	sapa_14_1_a0244	ALRVNNVSKK	EYIASRRDLY	ATIFGLRHTY	NTKVGNDFVR	GVSGGERKRV
(PRED)	sapa_8_1_2350	ALRVNNVSKK	EYIASRRDLY	ATIFGLRHTY	NTKVGNDFVR	GVSGGERKRV
(PRED)	sapa_17_1_2380	ALRVNNVSKK	EYIASRRDLY	ATIFGLRHTY	NTKVGNDFVR	GVSGGERKRV
(PRED)	sapa_7_1_2370	ALRVNNVSKK	EYIASRRDLY	ATIFGLRHTY	NTKVGNDFVR	GVSGGERKRV
(PRED)	sapa_2_1_a02460	ALRVNNVSKK	EYIASRRDLY	ATIFGLRHTY	NTKVGNDFVR	GVSGGERKRV
(PRED)	sapa_23_1_a0248	ALRVNNVSKK	EYIASRRDLY	ATIFGLRHTY	NTKVGNDFVR	GVSGGERKRV
(PRED)	sapa_3_1_a02470	ALRVNNVSKK	EYIASRRDLY	ATIFGLRHTY	NTKVGNDFVR	GVSGGERKRV
(PRED)	sapa_18_1_2390	ALRVNNVSKK	EYIASRRDLY	ATIFGLRHTY	NTKVGNDFVR	GVSGGERKRV
(PRED)	sami_1_4_244	ALRVNNVTKK	EYISSRRDLY	ATIFGLRHTY	NTKVGNDFVR	GVSGGERKRV
(PRED)	saku_1_4_262	ALRVNNVSKE	EYIASRRHLY	ATIFGLRHTY	NTKVGNDFVR	GVSGGERKRV
(PRED)	saba_1_58_bf002	AVRVNNISRQ	EYIENRRDLF	ATIFGLRHTY	NTKVGNDFVR	GVSGGERKRV
(PRED)	saeu_1_4_d02400	AVRVNNISKK	EYIESRRDLY	ATIFGLRHTY	GTKVGNDFVR	GVSGGERKRV
(PRED)	naca_1_e01640	AKRVNDVSKE	EYIKSTRELY	ATIFGLRHTY	NTKVGNDFVR	GVSGGERKRV
(PRED)	nada_1_g01850	ANRVNGVSKA	EYIQSTRELY	ATIFGLRHTY	QTKVGNDFVR	GVSGGERKRV
(PRED)	naca_1_e01630	AKRVNNISRD	EYIKSSRELY	ATIFGLRHTY	NTKVGNDFVR	GVSGGERKRV
(PRED)	nada_1_g01840	ARRVNNVSRE	EYIVSTRDLY	ATIFGLRHTY	NTKVGNEYVR	GISGGERKRV
(PRED)	kaaf_1_c00820	STRVNNVSRK	EYIESRRDLF	ATIFGLTHTY	HTKVGNDFVR	GVSGGERKRV
(PRED)	teph_1_m00640	AKRVDNVPKE	DYIAAVRDLY	ATIFGLRHTY	QTFVGNDFVR	GVSGGERKRV
(PRED)	vapo_1_1036_28	AKRVDDVSRA	EYIAATRDLF	ATIFGLRHTY	NTKVGNDFVR	GVSGGERKRV
(PRED)	tebl_1_i01760	AVRVNNVSRK	EYIAAIRDLY	CTIFGLRHTY	NTKVGNDFVR	GVSGGERKRV
(PRED)	tode_1_d04040	AKRVNNMSEQ	EYIDFTRDFY	ATIFGLTHTY	DTKVGNDFVR	GVSGGERKRV
(PRED)	naca_1_e01650	AKRVNNISKA	EYVKTTRELY	ATIFGLRHTY	HTKVGNDFVR	GVSGGERKRV
(PRED)	tebl_1_g02820	AIRVDNLSRS	DYIAFIRDLY	ATIFGLRHTY	NTLVGNDFVR	GVSGGERKRV
(PRED)	lakl_1_c11616g	SVRVNNASRK	QYIASMRELY	ATIFGLRHTY	NTKVGNDFVR	GVSGGERKRV
(PRED)	saar_1_8_h03780	EKRVNDVTRA	EYIAANREFY	AKVFGLTHTY	DTRVGNDFVS	GVSGGERKRV
(PRED)	sace_14_7_g0015	AKRVNNVTKE	EYITANREFY	AKIFGLTHTF	DTKVGNDFIS	GVSGGERKRV
(PRED)	sace_15_7_g0387	AKRVNNVTKE	EYITANREFY	AKIFGLTHTF	DTKVGNDFIS	GVSGGERKRV
(PRED)	sace_24_8_3780	AKRVNNVTKE	EYITANREFY	AKIFGLTHTF	DTKVGNDFIS	GVSGGERKRV
(PRED)	sace_40_8_h0383	AKRVNNVTKE	EYITANREFY	AKIFGLTHTF	DTKVGNDFIS	GVSGGERKRV
(PRED)	sace_6_169_fm00	AKRVNNVTKE	EYITANREFY	AKIFGLTHTF	DTKVGNDFIS	GVSGGERKRV
(PRED)	sace_19_7_3840	AKRVNNVTKE	EYITANREFY	AKIFGLTHTF	DTKVGNDFIS	GVSGGERKRV
(PRED)	sace_32_7_3770	AKRVNNVTKE	EYITANREFY	AKIFGLTHTF	DTKVGNDFIS	GVSGGERKRV
(PRED)	sace_56_17_q011	AKRVNNVTKE	EYITANREFY	AKIFGLTHTF	DTKVGNDFIS	GVSGGERKRV
(PRED)	sace_5_78_bz001	AKRVNNVTKE	EYIXANREFY	AKIFGLTHTF	DTKVGNDFIS	GVSGGERKRV
(PRED)	sace_2_8_h03860	AKRVNNVTKE	EYITANREFY	AKIFGLTHTF	DTKVGNDFIS	GVSGGERKRV
(PRED)	sace_53_29_ac00	AKRVNNVTKE	EYITANREFY	AKIFGLTHTF	DTKVGNDFTS	GVSGGERKRV
(PRED)	sace_17_7_g0393	AKRVNNVTKE	EYITANREFY	AKIFGLTHTF	DTKVGNDFIS	GVSGGERKRV
(PRED)	sace_25_7_g0388	AKRVNNVTKE	EYITANREFY	AKIFGLTHTF	DTKVGNDFIS	GVSGGERKRV
(PRED)	sace_37_7_g0385	AKRVNNVTKE	EYITANREFY	AKIFGLTHTF	DTKVGNDFIS	GVSGGERKRV
(PRED)	sace_9_7_g00180	AKRVNNVTKE	EYITANREFY	AKIFGLTHTF	DTKVGNDFIS	GVSGGERKRV
(PRED)	sace_60_6_f0335	AKRVNNVTKE	EYITANREFY	AKIFGLTHTF	DTKVGNDFIS	GVSGGERKRV
(PRED)	sace_59_336_lx0	AKRVNNVTKE	EYITANREFY	AKIFGLTHTF	DTKVGNDFIS	GVSGGERKRV
(PRED)	sace_31_7_3780	AKRVNNVTKE	EYITANREFY	AKIFGLTHTF	DTKVGNDFIS	GVSGGERKRV
(PRED)	sace_34_8_3770	AKRVNNVTKE	EYITANREFY	AKIFGLTHTF	DTKVGNDFIS	GVSGGERKRV
(PRED)	sace_58_71_bs00	AKRVNNVTKE	EYITANREFY	AKIFGLTHTF	DTKVGNDFIS	GVSGGERKRV
(PRED)	sace_7_7_g03880	AKRVNNVTKE	EYITANREFY	AKIFGLTHTF	DTKVGNDFIS	GVSGGERKRV
(PRED)	sace_35_7_3840	AKRVNNVTKE	EYITANREFY	AKIFGLTHTF	DTKVGNDFIS	GVSGGERKRV
(PRED)	sace_43_7_g0387	AKRVNNVTKE	EYITANREFY	AKIFGLTHTF	DTKVGNDFIS	GVSGGERKRV
(PRED)	sace_57_8_h0390	AKRVNNVTKE	EYITANREFY	AKIFGLTHTF	DTKVGNDFIS	GVSGGERKRV
(PRED)	sace_45_7_g0389	AKRVNNVTKE	EYITANREFY	AKIFGLTHTF	DTKVGNDFIS	GVSGGERKRV
(PRED)	sace_46_8_h0391	AKRVNNVTKE	EYITANREFY	AKIFGLTHTF	DTKVGNDFIS	GVSGGERKRV
(PRED)	sace_23_7_3860	AKRVNNVTKE	EYITANREFY	AKIFGLTHTF	DTKVGNDFIS	GVSGGERKRV
(PRED)	sace_21_7_3790	AKRVNNVTKE	EYIAANREFY	AKIFGLTHTF	DTKVGNDFIS	GVSGGERKRV

(PRED)	sace_8_73_bu001	AKRVNNVTKE	EYITANREFY	AKIFGLTHTF	DTKVGNDFIS	GVSGGERKRV
(PRED)	sapa_1_8_h03820	SKRVNDVTKE	EYVVANREFY	AKIFGLTHTF	DTKVGNDFIS	GVSGGERKRV
(PRED)	sapa_21_8_h0387	SKRVNDVTKE	EYVVANREFY	AKIFGLTHTF	DTKVGNDFIS	GVSGGERKRV
(PRED)	sapa_20_8_h0386	SKRVNDVTKE	EYVVANREFY	AKIFGLTHTF	DTKVGNDFIS	GVSGGERKRV
(PRED)	sapa_22_8_h0390	SKRVNDVTKE	EYVVANREFY	AKIFGLTHTF	DTKVGNDFIS	GVSGGERKRV
(PRED)	sapa_25_8_h0387	SKRVNDVTKE	EYVVANREFY	AKIFGLTHTF	DTKVGNDFIS	GVSGGERKRV
(PRED)	sapa_6_8_3750	SKRVNDVTKE	EYVVANREFY	AKIFGLTHTF	DTKVGNDFIS	GVSGGERKRV
(PRED)	sapa_9_8_3720	SKRVNDVTKE	EYVVANREFY	AKIFGLTHTF	DTKVGNDFIS	GVSGGERKRV
(PRED)	sapa_19_8_h0390	SKRVNDVTKE	EYVVANREFY	AKIFGLTHTF	DTKVGNDFIS	GVSGGERKRV
(PRED)	sapa_24_8_h0385	SKRVNDVTKE	EYVVANREFY	AKIFGLTHTF	DTKVGNDFIS	GVSGGERKRV
(PRED)	sapa_4_8_h03850	SKRVNDVTKE	EYVVANREFY	AKIFGLTHTF	DTKVGNDFIS	GVSGGERKRV
(PRED)	sapa_10_8_3760	SKRVNDVTKE	EYVVANREFY	AKIFGLTHTF	DTKVGNDFIS	GVSGGERKRV
(PRED)	sapa_13_8_h0382	SKRVNDVTKE	EYVVANREFY	AKIFGLTHTF	DTKVGNDFIS	GVSGGERKRV
(PRED)	sapa_8_8_3750	SKRVNDVTKE	EYVVANREFY	AKIFGLTHTF	DTKVGNDFIS	GVSGGERKRV
(PRED)	sapa_11_8_h0383	SKRVNDVTKE	EYVVANREFY	AKIFGLTHTF	DTKVGNDFIS	GVSGGERKRV
(PRED)	sapa_5_8_3700	SKRVNDVTKE	EYVVANREFY	AKIFGLTHTF	DTKVGNDFIS	GVSGGERKRV
(PRED)	sapa_16_8_h0389	SKRVNDVTKE	EYVVANREFY	AKIFGLTHTF	DTKIGNDFIS	GVSGGERKRV
(PRED)	sapa_17_8_3730	SKRVNDVTKE	EYVVANREFY	AKIFGLTHTF	DTKIGNDFIS	GVSGGERKRV
(PRED)	sapa_2_8_h03860	SKRVNDVTKE	EYVVANREFY	AKIFGLTHTF	DTKIGNDFIS	GVSGGERKRV
(PRED)	sapa_7_8_3740	SKRVNDVTKE	EYVVANREFY	AKIFGLTHTF	DTKIGNDFIS	GVSGGERKRV
(PRED)	sapa_23_8_h0385	SKRVNDVTKE	EYVVANREFY	AKIFGLTHTF	DTKIGNDFIS	GVSGGERKRV
(PRED)	sapa_3_8_h03890	SKRVNDVTKE	EYVVANREFY	AKIFGLTHTF	DTKIGNDFIS	GVSGGERKRV
(PRED)	sapa_18_8_3730	SKRVNDVTKE	EYVVANREFY	AKIFGLTHTF	DTKIGNDFIS	GVSGGERKRV
(PRED)	sami_1_14_399	AKRVNDVTRE	EYVATTREFY	AKIFGLTHTY	DTKVGNDFIS	GVSGGERKRV
(PRED)	sace_4_8_h03690	QKRVNNVTKE	EYIXANREFY	AKIFGLTHTF	DTKVGNDFIS	GVSGGERKRV
(PRED)	saku_1_14_404	AKRVNNVTGA	EYIAANRDFY	AQIFGLAHTY	DTRVGNDFVS	GVSGGERKRV
(PRED)	sace_1_ynr070w	AKRVNNVTKE	EYITANREFY	AKIFGLTHTF	DTKVGNDFIS	GVSGGERKRV
(PRED)	sace_49_8_h0383	AKRVNNVTKE	EYITANREFY	AKIFGLTHTF	DTKVGNDFIS	GVSGGERKRV
(PRED)	saeu_1_2_b00130	SKRVNDVTKA	EYIATNRDFY	ATMFGLTHTY	DTRVGNDFVS	GVSGGERKRV
(PRED)	sauv_1_7_3	TKRVNNVTKA	EYIASNRDFY	ATMFGLTHTY	DTRVGNDFVS	GVSGGERKRV
(PRED)	sami_1_17_26	AKRVNNVTRA	EYIASQRDLY	ATIFGLTHTY	NTKVGNDFVR	GVSGGERKRV
(PRED)	zyba_1_02055_AN	AVRVNGVSRK	EYISATRDLY	ATIFGLTQIY	DTRVGDEFVR	GVSGGQRKRV
(PRED)	zyba_1_07912	AVRVNGVSRK	EYISATRDLY	ATIFGLTQIY	DTNVGDEFVR	GVSGGQRKRV
(PRED)	zyba_2_2_b00600	AVRVNGVSRK	EYISATRDLY	ATIFGLTQIY	DTNVGDEFVR	GVSGGQRKRV
(PRED)	zyba_3_3_c03460	AVRVNGVSRK	EYISATRDLY	ATIFGLTQIY	DTNVGDEFVR	GVSGGQRKRV
(PRED)	zyba_1_04634	AVRVNGVSRS	EYISATRDLY	ATIFGLTEVY	DTPVGNEFVR	GVSGGQRKRV
(PRED)	zyba_1_06675	AVRVNGVSRS	EYINTTRDLY	ATIFGLTEVY	DTPVGNEFVR	GISGGQRKRV
(PRED)	zyba_3_2_b02230	AVRVNGVSRS	EYINTTRDLY	ATIFGLTEVY	DTPVGNEFVR	GISGGQRKRV
(PRED)	zyba_2_1_a00860	AVRVNGVSRS	EYINTTRDLY	ATIFGLTEVY	DTPVGNEFVR	GISGGQRKRV
(PRED)	zyro_1_a04114g	AIRVNNVSRS	EYIRETRDLY	ATIFGLTHTY	DTKVGNDFVR	GVSGGERKRV
(PRED)	zyro_1_b14762g	AVRVNNVSRK	EHIRATRDLY	ATIFGLTHTY	DTKVGNDFVR	GVSGGERKRV
(PRED)	zyba_2_14_n0149	SVRVNGVSRR	EYVSAVRDLY	ATIFGLTEIY	NTKVGNDFVR	GVSGGQRKRV
(PRED)	zyba_2_33_ag001	SVRVNGVSRR	EYISAVRDLY	ATIFGLTEIY	NTKVGNDFVR	GVSGGQRKRV
(PRED)	lath_1_a01914g	QKRLDNASRS	EYITAMRELY	GTIFGLRHTY	NTKVGNDFVR	GVSGGERKRV
(PRED)	lawa_1_23_5161	RVRLDGASRS	EYITAMRELY	GTIFGLRHTY	NTKVGNDFVR	GVSGGERKRV
(PRED)	klae_1_14_n0012	NKRIYNMSKK	EYVQFMRDLY	ATIFGLRHTY	NTKVGNDFVR	GVSGGERKRV
(PRED)	klla_1_d03432g	SKRINDFTRQ	QYIEFIRDLY	ATIFGLKHTY	NTKVGDDFVR	GVSGGERKRV
(PRED)	klma_1_1_a01880	SKRIKNFTRK	QYVEFIRDLY	ATIFGLKHTY	NSKVGNDFVR	GVSGGERKRV
(PRED)	klwi_1_33_ag001	NKRINNFSRK	EYVEYMRDLY	ATIFGLKHTY	NTKVGDDFVR	GVSGGERKRV
(PRED)	teph_1_a04220	EKRVNNISKK	EYLIKTRELL	ATIFGLENTY	NTKVGNEYVR	GISGGERKRV
(PRED)	vapo_1_1037_47	NVRINNMSAK	KQIGIARDLL	ATIFGLRHTY	STKVGNDFVR	GVSGGERKRV
(PRED)	pata_1_2_b05590	SIRVDNISRE	KYITTMRDLW	CTVFGLTHTL	NTRVGNDFVR	GVSGGERKRV
(PRED)	wian_1_3_c04380	HTRINDASRE	QYVTAVRDLL	ATVFGLRHTY	NTKVGNEFVR	GVSGGERKRV
(PRED)	wian_1_3_c04390	HTRVNNASRE	QYITANRDLL	ATIFGLRHTY	NTKVGNDFVR	GVSGGERKRV
(PRED)	wian_1_7_g01010	HTRINNESRQ	QYITATRDIL	ATIFGLRHTY	NTKVGNDFVR	GVSGGERKRV
(PRED)	bain_1_1_a00100	ELRVNNVSRE	KFITAMRDLL	ATVFGLRHTY	NTKVGNDLVR	GVSGGERKRV
(PRED)	bain_1_17_q0038	ELRVNNVSRE	KFITAMRDLL	ATVFGLRHTY	NTKVGNDLVR	GVSGGERKRV
(PRED)	bain_1_8_h00410	NLRVNNVSRE	EYITAMRDIL	ATVFGLRHTF	HTKVGNDMVR	GVSGGERKRV
(PRED)	caal_1_19_5759	EMRINGVTRD	EFINAKKEIL	ATVFGLRHTY	HTKVGNDFVR	GVSGGERKRV
(PRED)	caal_11_25_y002	EMRINGVTRD	EFINAKKEIL	ATVFGLRHTY	HTKVGNDFVR	GVSGGERKRV
(PRED)	caal_4_4_d03320	EMRINGVTRD	EFINAKKEIL	ATVFGLRHTY	HTKVGNDFVR	GVSGGERKRV
(PRED)	caal_12_26_z005	EMRINGVTRD	EFINAKKEIL	ATVFGLRHTY	HTKVGNDFVR	GVSGGERKRV
(PRED)	caal_5_30_ad005	EMRINGVTRD	EFINAKKEIL	ATVFGLRHTY	HTKVGNDFVR	GVSGGERKRV
(PRED)	caal_8_3_c03320	EMRINGVTRD	EFINAKKEIL	ATVFGLRHTY	HTKVGNDFVR	GVSGGERKRV
(PRED)	caal_6_4_d03280	EMRINGVTRD	EFINAKKEIL	ATVFGLRHTY	HTKVGNDFVR	GVSGGERKRV
(PRED)	caal_10_3_c0334	EMRINGVTRD	EFINAKKEIL	ATVFGLRHTY	HTKVGNDFVR	GVSGGERKRV
(PRED)	caal_3_29_ac005	EMRINGVTRD	EFINAKKEIL	ATVFGLRHTY	HTKVGNDFVR	GVSGGERKRV
(PRED)	caal_2_04989	EMRINGVTRD	EFINAKKEIL	ATVFGLRHTY	HTKVGNDFVR	GVSGGERKRV
(PRED)	cadu_1_64350	EMRINGVTRD	EFINAKKEIL	ATVFGLRHTY	NTKVGNDFVR	GVSGGERKRV
(PRED)	caor_1_h02090	KMRLNGVTRE	QFVNAKKELL	ATVFGLRHTY	HTKVGNDFVR	GVSGGERKRV
(PRED)	capa_1_600750	KMRLNGVTRE	QFVNAKKELL	ATVFGLRHTY	HTKVGNDFVR	GVSGGERKRV
(PRED)	loel_1_04930	NVRIDGVSRE	QFVQAKKEIL	ATVFGLRHTY	HTKVGNDFVR	GVSGGERKRV
(PRED)	spar_1_5_e03260	NLRINGVSKS	EFIEAQKIIL	ATVFGLKHTF	HTKVGNDFVR	GVSGGERKRV
(PRED)	sppa_1_7_g03160	NLRINGVSRS	QFIEAQKIIL	ATVFGLKHTF	HTKVGNDFVR	GVSGGERKRV
(PRED)	catr_1_01205	NIRINGISRD	QFIKAKKEIL	ATVFGLRHTY	NTKVGNDYVR	GVSGGERKRV

(PRED)	catr_1_05498	NIRINGVTRE	QFINAKKEVL	ATVFGLRHTY	HTKVGNDYVR	GVSGGERKRV
(PRED)	catr_1_05971	NIRINGVTRE	QFINAKKEVL	ATVFGLRHTY	HTKVGNDYVR	GVSGGERKRV
(PRED)	deha_1_a03696g	ELRVNDVSRE	KFIDALKEIL	ATVFGLRHTY	HTKVGNDFVR	GVSGGERKRV
(PRED)	deha_2_5_e00720	ELRVNDVSRE	KFINALKEIL	ATVFGLRHTY	HTKVGNDFVR	GVSGGERKRV
(PRED)	scst_1_3_c02890	NMRVNGVSRG	QFINAMKEIL	ATVFGLRHTY	HTKVGNDFIR	GVSGGERKRV
(PRED)	mebi_1_8_h00300	NKRVNDKSVQ	EHIDYNRDLL	ATVFGLRHTY	STKVGNDYVR	GVSGGERKRV
(PRED)	lakl_1_h21010g	SLSDEKIPKK	QHIHGNRDLY	AGIFHLTHTF	NTKVGNDFVR	GVSGGERKRV
(PRED)	caar_1_13_m0142	SVRIDNISRN	EYIQVINDLY	LTLYGLKHVE	KTLVGDDFVR	GISGGQRKRV
(PRED)	caar_1_14_n0143	NVRINNVSRK	DYIQAINDLY	LTLYGLKHVE	KTLVGNDFVR	GISGGQRKRV
(PRED)	hapo_1_1_a07220	KVRIDNLSRS	EYIRTIKDLY	LTLYGLKHVE	KTLVGNDFVR	GISGGQRKRV
(PRED)	ogpa_1_1_a01680	KVRIDNLSRS	EYIRTIKDLY	LTLYGLKHVE	KTLVGNDFVR	GISGGQRKRV
(PRED)	piku_1_96_cr001	VVRLNNISRK	EYINVIKDLY	LTVFGLKHVE	KTLVGNDFVR	GISGGQRKRV
(PRED)	pime_1_4_d03240	SIRINDVSRN	EYINTIKDLY	LTLFGLKHVE	KTLVGNDFVR	GVSGGQRKRV
(PRED)	pime_1_1_a12110	DIRVNDMSRK	EYIAAIKDLY	LILFGLKHVE	KTLVGNDFVR	GISGGQRKRV
(PRED)	piku_1_227_hs00	NVRVDNETRG	EYISNMKNIY	EIIFGLKHVE	KTKVGNDFIR	GVSGGERKRV
(PRED)	pime_1_5_e05800	NIRVDNESRK	TYIENMMNLY	EILFGLKHVE	KTIVGNDFVR	GISGGERKRV
(PRED)	pime_1_1_a07690	TIRFDNKTRE	EYIENMKDLY	EILFGLKFVE	KTLVGSDFIR	GVSGGQRKRV
(PRED)	debr_2_5_e03380	AVRVQGVTRR	QYIRTIKELY	AAVFGLHHVQ	KTLVGGDFVR	GISGGQRKRV
(PRED)	kopa_1_2_b10040	RIRIDDISRK	KHVDNWLKIL	LTVYGLGHTR	NTIVGNDFVR	GVSGGERKRV
(PRED)	kopa_2_7_g00500	RIRIDDISRK	KHVDNWLKIL	LTVYGLSHTR	HTIVGNDFVR	GVSGGERKRV
(PRED)	asru_1_13_m0119	EIRPNNSTRD	QYITALRDIL	TTVFGLNHVK	NTKVGNDYVR	GVSGGQRKRV
(PRED)	asru_1_15_o0045	KLRIKGYTRD	QYINALMDIL	TTVFGLNHVR	NTKVGDDFVR	GVSGGERKRV
(PRED)	wian_1_1_a02920	NARPNSSTRE	QYISIMVNLL	ATVFGLRHIF	QTKVGNDVFR	GVSGGERKRV
(PRED)	wian_1_1_a02930	NTRPNASTRE	QYISIMVDLL	ATVFGLRHTY	QTKVGNDVFR	GVSGGERKRV

		 560	 570	 580	 590	 600
(PRED)	asac_1_6_f03560	SIAEALAANA	SVYCWDNATR	GLDASTALEY	AQAMRIMTNL	LQSTSLVTLY
(PRED)	ergo_1_abr125c	SIAEALAANA	SVYCWDNATR	GLDASTALEY	AQAMRIMTNL	LQSTSLVTLY
(PRED)	ercy_1_3604	SIAEALAANG	SVYCWDNATR	GLDASTALEY	AQAIRIMTNI	LKSTSLVTIY
(PRED)	cagl_1_i04862g	SIAEALVTKG	SIYCWDNATR	GLDASTALEY	AKAIRITTNL	LGSTAFVTIY
(PRED)	kaaf_1_c00830	SIAEALAARG	TVYCWDNATR	GLDASTALEY	AAAIRIMTNL	LKSTAFVTIY
(PRED)	kana_1_k01350	SIAEALAAKG	SIYCWDNATR	GLDASTALEY	ARAIRIMTNL	LGSTAFVTIY
(PRED)	saar_1_2_b02590	SIAEALAAKG	SIYCWDNATR	GLDASTALEY	AKAIRIMTNL	LKSTAFVTIY
(PRED)	sace_1_ydr011w	SIAEALAAKG	SIYCWDNATR	GLDASTALEY	AKAIRIMTNL	LKSTAFVTIY
(PRED)	sace_16_1_a0238	SIAEALAAKG	SIYCWDNATR	GLDASTALEY	AKAIRIMTNL	LKSTAFVTIY
(PRED)	sace_45_1_a0242	SIAEALAAKG	SIYCWDNATR	GLDASTALEY	AKAIRIMTNL	LKSTAFVTIY
(PRED)	sace_48_1_a0238	SIAEALAAKG	SIYCWDNATR	GLDASTALEY	AKAIRIMTNL	LKSTAFVTIY
(PRED)	sace_60_4_d0244	SIAEALAAKG	SIYCWDNATR	GLDASTALEY	AKAIRIMTNL	LKSTAFVTIY
(PRED)	sace_52_1_a0240	SIAEALAAKG	SIYCWDNATR	GLDASTALEY	AKAIRIMTNL	LKSTAFVTIY
(PRED)	sace_46_1_a0240	SIAEALAAKG	SIYCWDNATR	GLDASTALEY	AKAIRIMTNL	LKSTAFVTIY
(PRED)	sace_25_1_a0240	SIAEALAAKG	SIYCWDNATR	GLDASTALEY	AKAIRIMTNL	LKSTAFVTIY
(PRED)	sace_24_1_2300	SIAEALAAKG	SIYCWDNATR	GLDASTALEY	AKAIRIMTNL	LKSTAFVTIY
(PRED)	sace_47_1_a0240	SIAEALAAKG	SIYCWDNATR	GLDASTALEY	AKAIRIMTNL	LKSTAFVTIY
(PRED)	sace_7_1_a02410	SIAEALAAKG	SIYCWDNATR	GLDASTALEY	AKAIRIMTNL	LKSTAFVTIY
(PRED)	sace_59_110_df0	SIAEALAAKG	SIYCWDNATR	GLDASTALEY	AKAIRIMTNL	LKSTAFVTIY
(PRED)	sace_56_1_a0202	SIAEALAAKG	SIYCWDNATR	GLDASTALEY	AKAIRIMTNL	LKSTAFVTIY
(PRED)	sace_40_1_a0239	SIAEALAAKG	SIYCWDNATR	GLDASTALEY	AKAIRIMTNL	LKSTAFVTIY
(PRED)	sace_15_1_a0242	SIAEALAAKG	SIYCWDNATR	GLDASTALEY	TKAIRIMTNL	LKSTAFVTIY
(PRED)	sace_37_1_a0243	SIAEALAAKG	SIYCWDNATR	GLDASTALEY	TKAIRIMTNL	LKSTAFVTIY
(PRED)	sace_9_1_a02440	SIAEALAAKG	SIYCWDNATR	GLDASTALEY	TKAIRIMTNL	LKSTAFVTIY
(PRED)	sace_22_1_2300	SIAEALAAKG	SIYCWDNATR	GLDASTALEY	AKAIRIMTNL	LKSTAFVTIY
(PRED)	sace_29_1_2290	SIAEALAAKG	SIYCWDNATR	GLDASTALEY	AKAIRIMTNL	LKSTAFVTIY
(PRED)	sace_34_1_2320	SIAEALAAKG	SIYCWDNATR	GLDASTALEY	AKAIRIMTNL	LKSTAFVTIY
(PRED)	sace_58_25_y007	SIAEALAAKG	SIYCWDNATR	GLDASTALEY	AKAIRIMTNL	LKSTAFVTIY
(PRED)	sace_23_1_2290	SIAEALAAKG	SIYCWDNATR	GLDASTALEY	AKAIRIMTNL	LKSTAFVTIY
(PRED)	sace_6_120_dp00	SIAEALAAKG	SIYCWDNATR	GLDASTALEY	AKAIRIMTNL	LKSTAFVTIY
(PRED)	sace_57_1_a0241	SIAEALAAKG	SIYCWDNATR	GLDASTALEY	AKAIRIMTNL	LKSTAFVTIY
(PRED)	sace_17_1_a0241	SIAEALAAKG	SIYCWDNATR	GLDASTALEY	AKAIRVMTNL	LKSTAFVTIY
(PRED)	sace_21_1_2310	SIAEALAAKG	SIYCWDNATR	GLDASTALEY	AKAIRIMTNL	LKSTAFVTIY
(PRED)	sace_49_1_a0246	SIAEALAAKG	SIYCWDNATR	GLDASTALEY	AKAIRIMTNL	LKSTAFVTIY
(PRED)	sace_8_2_b02430	SIAEALAAKG	SIYCWDNATR	GLDASTALEY	AKAIRIMTNL	LKSTAFVTIY
(PRED)	sace_31_1_2300	SIAEALAAKG	SIYCWDNATR	GLDASTALEY	AKSIRIMTNL	LKSTAFVTIY
(PRED)	sace_50_1_a0241	SIAEALAAKG	SIYCWDNATR	GLDASTALEY	AKAIRIMTNL	LKSTAFVTIY
(PRED)	sace_4_1_a02360	SIAEALAAKG	SIYCWDNATR	GLDASTALEY	AKAIRIMTNL	LKSTAFVTIY
(PRED)	sace_2_1_a02390	SIAEALAAKG	SIYCWDNATR	GLDASTALEY	AKAIRIMTNL	LKSTAFVTIY
(PRED)	sace_5_33_ag005	SIAEALAAKG	SIYCWDNATR	GLDASTALEY	AKAIRIMTNL	LKSTAFVTIY
(PRED)	sapa_11_1_a0247	SIAEALAAKG	SIYCWDNATR	GLDASTALEY	AKAIRIMTNL	LKSTAFVTIY
(PRED)	sapa_25_1_a0246	SIAEALAAKG	SIYCWDNATR	GLDASTALEY	AKAIRIMTNL	LKSTAFVTIY
(PRED)	sapa_4_1_a02470	SIAEALAAKG	SIYCWDNATR	GLDASTALEY	AKAIRIMTNL	LKSTAFVTIY
(PRED)	sapa_5_1_2350	SIAEALAAKG	SIYCWDNATR	GLDASTALEY	AKAIRIMTNL	LKSTAFVTIY
(PRED)	sapa_9_1_2360	SIAEALAAKG	SIYCWDNATR	GLDASTALEY	AKAIRIMTNL	LKSTAFVTIY
(PRED)	sapa_14_1_a0244	SIAEALAAKG	SIYCWDNATR	GLDASTALEY	AKAIRIMTNL	LKSTAFVTIY
(PRED)	sapa_8_1_2350	SIAEALAAKG	SIYCWDNATR	GLDASTALEY	AKAIRIMTNL	LKSTAFVTIY
(PRED)	sapa_17_1_2380	SIAEALAAKG	SIYCWDNATR	GLDASTALEY	AKAIRIMTNL	LKSTAFVTIY
(PRED)	sapa_7_1_2370	SIAEALAAKG	SIYCWDNATR	GLDASTALEY	AKAIRIMTNL	LKSTAFVTIY

(PRED)	sapa_2_1_a02460	SIAEALAAKG	SIYCWDNATR	GLDASTALEY	AKAIRIMTNL	LKSTAFVTIY
(PRED)	sapa_23_1_a0248	SIAEALAAKG	SIYCWDNATR	GLDASTALEY	AKAIRIMTNL	LKSTAFVTIY
(PRED)	sapa_3_1_a02470	SIAEALAAKG	SIYCWDNATR	GLDASTALEY	AKAIRIMTNL	LKSTAFVTIY
(PRED)	sapa_18_1_2390	SIAEALAAKG	SIYCWDNATR	GLDASTALEY	AKAIRIMTNL	LKSTAFVTIY
(PRED)	sami_1_4_244	SIAEALAAKG	SIYCWDNATR	GLDASTALEY	AKAIRIMTNL	LKSTAFVTIY
(PRED)	saku_1_4_262	SIAEALAAKG	SIYCWDNATR	GLDSSTALEY	AKAIRIMTNL	LKSTAFVTIY
(PRED)	saba_1_58_bf002	SIAEALAAKG	SIYCWDNATR	GLDASTALEY	AKAIRIMTNL	LKSTAFVTIY
(PRED)	saeu_1_4_d02400	SIAEALAAKG	SIYCWDNATR	GLDASTALEY	AKAIRIMTNL	LKSTAFVTIY
(PRED)	naca_1_e01640	SIAEALAANG	TIYCWDNATR	GLDASTALEY	AKAIRIMTNL	LKSTAFVTIY
(PRED)	nada_1_g01850	SIAEALAANG	SIYCWDNATR	GLDASTALEY	TEAIRLMTNL	LGSTAFVTIY
(PRED)	naca_1_e01630	SIAEALAANG	SIYCWDNATR	GLDASTALEY	AKAIRIMTNL	LGSTAFVTIY
(PRED)	nada_1_g01840	SIAEALAANG	SIYCWDNATR	GLDASTALEY	AKAIRIMTNL	LHSTAFVTIY
(PRED)	kaaf_1_c00820	SIAEALAANG	SVYLWDNATR	GLDASTALEY	AKAIRIMTNL	LGSTAFVTIY
(PRED)	teph_1_m00640	SIAEALAARG	TVYCWDNATR	GLDASTALEY	AQAIRIMTNL	LGSTALVTIY
(PRED)	vapo_1_1036_28	SIAEALAARG	TIYCWDNATR	GLDASTALEY	AQAIRIMTNL	LESTAFVTIY
(PRED)	tebl_1_i01760	SIAEALAARG	SIYCWDNATR	GLDASTALEY	AQAIRVMTNL	LGSTAFVTLY
(PRED)	tode_1_d04040	SIAEAVVARG	SVYCWDNATR	GLDASTALEY	AKAIRIMTNL	MHSTALVTIY
(PRED)	naca_1_e01650	SIAEALVANG	SVYCWDNATR	GLDASTALEY	AKAIRIMTNL	LESTAFVTIY
(PRED)	tebl_1_g02820	SIAEALAARG	TIYCWDNATR	GLDASTALEY	AEAIRIMTNI	QQSTAFVTIY
(PRED)	lakl_1_c11616g	SIAEALAARG	SIYCWDNATR	GLDASTALEY	AQAIRIMTNL	LKSTALVTIY
(PRED)	saar_1_8_h03780	SIAEALAARG	SIYCWDNATR	GLDASTALEF	AQAIRIITSL	LGSTALVTIY
(PRED)	sace_14_7_g0015	SIAEALAAKG	SIYCWDNATR	GLDSSTALEF	ARAIRTMTNL	LGTTALVTVY
(PRED)	sace_15_7_g0387	SIAEALAAKG	SIYCWDNATR	GLDSSTALEF	ARAIRTMTNL	LGTTALVTVY
(PRED)	sace_24_8_3780	SIAEALAAKG	SIYCWDNATR	GLDSSTALEF	ARAIRTMTNL	LGTTALVTVY
(PRED)	sace_40_8_h0383	SIAEALAAKG	SIYCWDNATR	GLDSSTALEF	ARAIRTMTNL	LGTTALVTVY
(PRED)	sace_6_169_fm00	SIAEALAAKG	SIYCWDNATR	GLDSSTALEF	ARAIRTMTNL	LGTTALVTVY
(PRED)	sace_19_7_3840	SIAEALAAKG	SIYCWDNATR	GLDSSTALEF	ARAIRTMTNL	LGTTALVTVY
(PRED)	sace_32_7_3770	SIAEALAAKG	SIYCWDNATR	GLDSSTALEF	ARAIRTMTNL	LGTTALVTVY
(PRED)	sace_56_17_q011	SIAEALAAKG	SIYCWDNATR	GLDSSTALEF	ARAIRTMTNL	LGTTALVTVY
(PRED)	sace_5_78_bz001	SIAEALAAKG	SIYCWDNATR	GLDSSTALEF	ARAIRTMTNL	LGTTALVTVY
(PRED)	sace_2_8_h03860	SIAEALAAKG	SIYCWDNATR	GLDSSTALEF	ARAIRTMTNL	LGTTALVTVY
(PRED)	sace_53_29_ac00	SIAEALAAKG	SIYCWDNATR	GLDSSTALEF	ARAIRTMTNL	LGTTALVTVY
(PRED)	sace_17_7_g0393	SIAEALAAKG	SIYCWDNATR	GLDSSTALEF	ARAIRTMTNL	LGTTALVTVY
(PRED)	sace_25_7_g0388	SIAEALAAKG	SIYCWDNATR	GLDASTALEF	ARAIRTMTNL	LGTTALVTVY
(PRED)	sace_37_7_g0385	SIAEALAAKG	SIYCWDNATR	GLDSSTALEF	ARAIRTMTNL	LGTTALVTVY
(PRED)	sace_9_7_g00180	SIAEALAAKG	SIYCWDNATR	GLDSSTALEF	ARAIRTMTNL	LGTTALVTVY
(PRED)	sace_60_6_f0335	SIAEALAAKG	SIYCWDNATR	GLDSSTALEF	ARAIRTMTNL	LGTTALVTVY
(PRED)	sace_59_336_lx0	SIAEALAAKG	SIYCWDNATR	GLDSSTALEF	ARAIRTMTNL	LGTTALVTVY
(PRED)	sace_31_7_3780	SIAEALAAKG	SIYCWDNATR	GLDASTALEF	ARAIRTMTNL	LGTTALVTVY
(PRED)	sace_34_8_3770	SIAEALAAKG	SIYCWDNATR	GLDASTALEF	ARAIRTMTNL	LGTTALVTVY
(PRED)	sace_58_71_bs00	SIAEALAAKG	SIYCWDNATR	GLDASTALEF	ARAIRTMTNL	LGTTALVTVY
(PRED)	sace_7_7_g03880	SIAEALAAKG	SIYCWDNATR	GLDSSTALEF	ARAIRTMTNL	LGTTALVTVY
(PRED)	sace_35_7_3840	SIAEALAAKG	SIYCWDNATR	GLDASTALEF	ARAIRTMTNL	LGTTALVTVY
(PRED)	sace_43_7_g0387	SIAEALAAKG	SIYCWDNATR	GLDASTALEF	ARAIRTMTNL	LGTTALVTVY
(PRED)	sace_57_8_h0390	SIAEALAAKG	SIYCWDNATR	GLDSSTALEF	ARAIRTMTNL	LGTTALVTVY
(PRED)	sace_45_7_g0389	SIAEALAAKG	SIYCWDNATR	GLDASTALEF	ARAIRTMTNL	LGTTVLVTVY
(PRED)	sace_46_8_h0391	SIAEALAAKG	SIYCWDNATR	GLDASTALEF	ARAIRTMTNL	LGTTVLVTVY
(PRED)	sace_23_7_3860	SIAEALAAKG	SIYCWDNATR	GLDASTALEF	ARAIRTMTNL	LGTTALVTVY
(PRED)	sace_21_7_3790	SIAEALAAKG	PIYCWDNATR	GLDSSTALEF	ARAIRTMTNL	LGTTALVTVY
(PRED)	sace_8_73_bu001	SIAEALAAKG	SIYCWDNATR	GLDXSTALEF	ARAIRTMTNL	LGTTALVTVY
(PRED)	sapa_1_8_h03820	SIAEALAARG	SIYCWDNATR	GLDASTALEF	ARAIRVMTKL	LGTTALITIY
(PRED)	sapa_21_8_h0387	SIAEALAARG	SIYCWDNATR	GLDASTALEF	ARAIRVMTKL	LGTTALITIY
(PRED)	sapa_20_8_h0386	SIAEALAARG	SIYCWDNATR	GLDASTALEF	ARAIRVMTKL	LGTTALITIY
(PRED)	sapa_22_8_h0390	SIAEALAARG	SIYCWDNATR	GLDASTALEF	ARAIRVMTKL	LGTTALITIY
(PRED)	sapa_25_8_h0387	SIAEALAARG	SIYCWDNATR	GLDASTALEF	ARAIRVMTKL	LGTTALITIY
(PRED)	sapa_6_8_3750	SIAEALAARG	SIYCWDNATR	GLDASTALEF	ARAIRVMTKL	LGTTALITIY
(PRED)	sapa_9_8_3720	SIAEALAARG	SIYCWDNATR	GLDASTALEF	ARAIRVMTKL	LGTTALITIY
(PRED)	sapa_19_8_h0390	SIAEALAARG	SIYCWDNATR	GLDASTALEF	ARAIRVMTKL	LGTTALITIY
(PRED)	sapa_24_8_h0385	SIAEALAARG	SIYCWDNATR	GLDASTALEF	ARAIRVMTKL	LGTTALITIY
(PRED)	sapa_4_8_h03850	SIAEALAARG	SIYCWDNATR	GLDASTALEF	ARAIRVMTKL	LGTTALITIY
(PRED)	sapa_10_8_3760	SIAEALAARG	SIYCWDNATR	GLDASTALEF	ARAIRVMTKL	LGTTALITIY
(PRED)	sapa_13_8_h0382	SIAEALAARG	SIYCWDNATR	GLDASTALEF	ARAIRVMTKL	LGTTALITIY
(PRED)	sapa_8_8_3750	SIAEALAARG	SIYCWDNATR	GLDASTALEF	ARAIRVMTKL	LGTTALITIY
(PRED)	sapa_11_8_h0383	SIAEALAARG	SIYCWDNATR	GLDASTALEF	ARAIRVMTKL	LGTTALITIY
(PRED)	sapa_5_8_3700	SIAEALAARG	SIYCWDNATR	GLDASTALEF	ARAIRVMTKL	LGTTALITIY
(PRED)	sapa_16_8_h0389	SIAEALAARG	SIYCWDNATR	GLDASTALEF	ARAIRVMTKL	LGTTALITIY
(PRED)	sapa_17_8_3730	SIAEALAARG	SIYCWDNATR	GLDASTALEF	ARAIRVMTKL	LGTTALITIY
(PRED)	sapa_2_8_h03860	SIAEALAARG	SIYCWDNATR	GLDASTALEF	ARAIRVMTKL	LGTTALITIY
(PRED)	sapa_7_8_3740	SIAEALAARG	SIYCWDNATR	GLDASTALEF	ARAIRVMTKL	LGTTALITIY
(PRED)	sapa_23_8_h0385	SIAEALAARG	SIYCWDNATR	GLDASTALEF	ARAIRVMTKL	LGTTALITIY
(PRED)	sapa_3_8_h03890	SIAEALAARG	SIYCWDNATR	GLDASTALEF	ARAIRVMTKL	LGTTALITIY
(PRED)	sapa_18_8_3730	SIAEALAARG	SIYCWDNATR	GLDASTALDF	ARAIRVMTKL	LGTTALITIY
(PRED)	sami_1_14_399	SIAEALAARG	SIYCWDNATR	GLDASTALEF	AQAIRTMGKL	LGLTTLVTIY
(PRED)	sace_4_8_h03690	SIAEALAAKG	SIYCWDNATR	GLDSSTALEF	ARAIRTMTNL	LGTTALVTVY
(PRED)	saku_1_14_404	SIAEALAARG	SIYCWDNATR	GLDASTALEF	AQAIRTMTEL	LGSTALITIY

(PRED)	sace_1_ynr070w	SIAEALAAKG	SIYCWDNATR	GLDSSTALEF	ARAIRTMTNL	LGTTALVTVY
(PRED)	sace_49_8_h0383	SIAEALAAKG	SIYCWDNATR	GLDXSTALEF	ARAIRTMTNL	LGTTALVTVY
(PRED)	saeu_1_2_b00130	SIAEALAARG	SIYCWDNATR	GLDASTALEY	AQAIRVSTDL	LRSTALITLY
(PRED)	sauv_1_7_3	SIAEALAARG	SIYCWDNATR	GLDASTALEY	AQAIRVSTNL	LKSTALITLY
(PRED)	sami_1_17_26	SIAEALAARG	SIYCWDNATR	GLDASTALEY	AQAIRIMTNL	LGSTALVTVY
(PRED)	zyba_1_02055_AN	SIAEALAARG	TVYCWDNATR	GLDASTALEY	AQAIRIMTNL	LHSTAFVTIY
(PRED)	zyba_1_07912	SIAEALAARG	TVYCWDNATR	GLDASTALEY	AQAIRIMTNL	LHSTAFVTIY
(PRED)	zyba_2_2_b00600	SIAEALAARG	TVYCWDNATR	GLDASTALEY	AQAIRIMTNL	LHSTAFVTIY
(PRED)	zyba_3_3_c03460	SIAEALAARG	TVYCWDNATR	GLDASTALEY	AQAIRIMTNL	LHSTAFVTIY
(PRED)	zyba_1_04634	SIAEALAARG	TVYCWDNATR	GLDASTALEY	AHAIRILTDL	LRSTVFVTIY
(PRED)	zyba_1_06675	SIAEALAAKG	TVYCWDNATR	GLDASTALEY	AHAIRILTDL	LRSTVFVTIY
(PRED)	zyba_3_2_b02230	SIAEALAAKG	TVYCWDNATR	GLDASTALEY	AHAIRILTDL	LRSTVFVTIY
(PRED)	zyba_2_1_a00860	SIAEALAAKG	TVYCWDNATR	GLDASTALEY	AHAIRILTDL	LRSTVFVTIY
(PRED)	zyro_1_a04114g	SIAEALAARG	TFYCWDNATR	GLDASTALEY	AMAIRIMTNL	LNSTAFVTIY
(PRED)	zyro_1_b14762g	SIAEALAARG	TVYSWDNATR	GLDASTALEY	AQAIRIMTNL	LRSTAFVTIY
(PRED)	zyba_2_14_n0149	SIAEALAARG	TVYCWDNATR	GLDASTALEY	AQAIRLMTNL	LKSTAFVTIY
(PRED)	zyba_2_33_ag001	SIAEALAARG	TVYCWDNATR	GLDASTALEY	AQAIRLMTNL	LKSTAFVTIY
(PRED)	lath_1_a01914g	SIAEALASRG	SIYCWDNATR	GLDASTALEF	AQAIRIMTNL	QKSVAFVTIY
(PRED)	lawa_1_23_5161	SIAEALAARG	SIYCWDNATR	GLDASTALEF	AQAIRTMTNL	QKSIALVTIY
(PRED)	klae_1_14_n0012	SIAEALAANG	SIYCWDNATR	GLDASTALEY	TEAIRCMTNL	LKSTALITLY
(PRED)	klla_1_d03432g	SIAEALAARG	SIYCWDNATR	GLDASTALEY	TEAIRCMTNL	LKSTALITVY
(PRED)	klma_1_1_a01880	SIAEALAARG	SIYCWDNATR	GLDASTALEY	TEAIRCMTSL	LQSTALITIY
(PRED)	klwi_1_33_ag001	SIAEALAARG	SIYCWDNATR	GLDASTALEY	TEAIRCMTNL	LKSTALITLY
(PRED)	teph_1_a04220	SLAEAMCLRG	TVYCWDNATR	GLDASTALEY	AQAVRIITNL	LNSTAFISLY
(PRED)	vapo_1_1037_47	SIAEALAARG	TIYAWDNATR	GLDASTALEF	AQALRTFTEL	LKSTSFCCLY
(PRED)	pata_1_2_b05590	SIAEAMATQG	KIFCWDNATR	GLDASTALEY	SQAIRTSTNL	SKISSFVTIY
(PRED)	wian_1_3_c04380	SIAEALATKA	TVYCWDNATR	GLDASTALEY	AQAIRTSTNL	SKSVAFITIY
(PRED)	wian_1_3_c04390	SIAEALATKA	TVYCWDNATR	GLDASTALEY	AQAIRTSTNL	SKSVAFITIY
(PRED)	wian_1_7_g01010	SIAEALASKA	TVYCWDSATR	GLDASTALEY	AQAIRTSTNL	SKSVAFITIY
(PRED)	bain_1_1_a00100	SIAEALAARG	SVYCWDNATR	GLDASTALEF	AQAIRTSTNL	LKSVAFVTAY
(PRED)	bain_1_17_q0038	SIAEALAARG	SVYCWDNATR	GLDASTALEF	AQAIRTSTNL	LKSVAFVTAY
(PRED)	bain_1_8_h00410	SIAEALAARA	SIYCWDNATR	GLDASTALEF	TQAIRSTTNL	LKSVAFITIY
(PRED)	caal_1_19_5759	SIAEALACNG	SIYCWDNATR	GLDASTALEF	AQAIRTSTKL	LKTTAFVTIY
(PRED)	caal_11_25_y002	SIAEALACNG	SIYCWDNATR	GLDASTALEF	AQAIRTSTKL	LKTTAFVTIY
(PRED)	caal_4_4_d03320	SIAEALACNG	SIYCWDNATR	GLDASTALEF	AQAIRTSTKL	LKTTAFVTIY
(PRED)	caal_12_26_z005	SIAEALACNG	SIYCWDNATR	GLDASTALEF	AQAIRTSTKL	LKTTAFVTIY
(PRED)	caal_5_30_ad005	SIAEALACNG	SIYCWDNATR	GLDASTALEF	AQAIRTSTKL	LKTTAFVTIY
(PRED)	caal_8_3_c03320	SIAEALACNG	SIYCWDNATR	GLDASTALEF	AQAIRTSTKL	LKTTAFVTIY
(PRED)	caal_6_4_d03280	SIAEALACNG	SIYCWDNATR	GLDASTALEF	AQAIRTSTKL	LKTTAFVTIY
(PRED)	caal_10_3_c0334	SIAEALACNG	SIYCWDNATR	GLDASTALEF	AQAIRTSTKL	LKTTAFVTIY
(PRED)	caal_3_29_ac005	SIAEALACNG	SIYCWDNATR	GLDASTALEF	AQAIRTSTKL	LKTTAFVTIY
(PRED)	caal_2_04989	SIAEALACNG	SIYCWDNATR	GLDASTALEF	AQAIRTSTKL	LKTIAFVTIY
(PRED)	cadu_1_64350	SIAEALACNG	SIYCWDNATR	GLDASTALEF	AQAIRTSTKL	LKTTAFVTIY
(PRED)	caor_1_h02090	SIAEALACNG	SIYCWDNATR	GLDASTALEF	ARAIRTSTDI	LKTTAFVSIY
(PRED)	capa_1_600750	SIAEALACNG	SIYCWDNATR	GLDASTALEF	ARAIRTSTDI	LRSTAFVSIY
(PRED)	loel_1_04930	SIAEALACNG	TIYCWDNATR	GLDASTALEF	AQAIKTSTKI	LKTTSFVSIY
(PRED)	spar_1_5_e03260	SIAEALACNG	SLYFWDNATR	GLDASTALEF	TQAIRISTRL	LRTTAFITIY
(PRED)	sppa_1_7_g03160	SIAEALACSG	SLYCWDNATR	GLDASTALEF	TQAIRTSTKL	LRTTAFITIY
(PRED)	catr_1_01205	SIAEALACQA	SIYCWDNATR	GLDASTALEF	AQAIRTSTKL	LGTTAFITIY
(PRED)	catr_1_05498	SIAEALACHG	SIYCWDNATR	GLDSSTALEF	AQAIRTSTKL	LGTTAFVTIY
(PRED)	catr_1_05971	SIAEALACQG	SIYCWDNATR	GLDASTALEF	AQAIRTSTTL	MKTTAFVTIY
(PRED)	deha_1_a03696g	SIAEALACRG	SIYCWDNATR	GLDASTALEY	AHAIRTSTNL	LKNTAFVAIY
(PRED)	deha_2_5_e00720	SIAEALACRG	SIYCWDNATR	GLDASTALEY	AQAIRTSTNL	LKNTAFVAIY
(PRED)	scst_1_3_c02890	SIAEALACRG	SIYCWDNATR	GLDASTALEY	ARAIRTSTNL	LKTTAFVTIY
(PRED)	mebi_1_8_h00300	SIAEALACAG	KIYCWDNATR	GLDASTALEY	AQAIRVSTNL	LKKTAFVTIY
(PRED)	lakl_1_h21010g	SIAEAMAARG	SVYCWDNSSR	GLDSSSAYDY	IKAIRTVSDL	IKSVAFVAIY
(PRED)	caar_1_13_m0142	SIAEAMVTRA	SVFCFDNATR	GLDASTALEF	VESLRTSTNV	ANSATLVTIY
(PRED)	caar_1_14_n0143	SIAEAMVTRA	SVYCFDNATR	GLDASTALEF	VESLRTSTNI	TQSTSIVSIY
(PRED)	hapo_1_1_a07220	SIAEAMATRA	SVYCFDNATR	GLDASTALEF	VESLRTMTNI	TQSTSIVTIY
(PRED)	ogpa_1_1_a01680	SIAEAMATRA	SVYCFDNATR	GLDASTALEF	VESLRTMTNI	THSTSIVTIY
(PRED)	piku_1_96_cr001	SIAEAMVTRG	TVFCYDNATR	GLDASTALEF	VEALRTSTNI	TENTSVVTIY
(PRED)	pime_1_4_d03240	SIAEAMVTKG	TVYCFDNATR	GLDASTALEF	TEALRTSTNI	TKTTSLVTIY
(PRED)	pime_1_1_a12110	SIAEAMVTNG	TVYAFDNATR	ELDASTALEF	AEALRTSTNM	TKITSLVTIY
(PRED)	piku_1_227_hs00	SIAEAMVADG	MLTCYDNATR	GLDASTALEF	IENLRTVTNI	TKTTSVVTIY
(PRED)	pime_1_5_e05800	SIAEAMVSEG	SVYCFDNATR	GLDASTALEF	TQTLRTFTNV	TKATSVVTIY
(PRED)	pime_1_1_a07690	SIAEAMVTNG	NVYCFDNATR	GLDSSTALEF	VQALRTSTNI	YKTSSVVTIY
(PRED)	debr_2_5_e03380	SIAEAMATRG	TVYCYDNATR	GLDASTALEF	VRALRTSTNF	AGTACVVTAY
(PRED)	kopa_1_2_b10040	SIAEAMAANG	TVYCWDNATR	GLDASTALEF	TESVRATTNL	EQTTSFVTLY
(PRED)	kopa_2_7_g00500	SIAEAMAANG	TVYCWDNATR	GLDASTALEF	TESVRATTNL	EQTTSFVTLY
(PRED)	asru_1_13_m0119	SIAEALASRA	CIYCWDNATR	GLDSSTALEY	AQAIRVSTNL	LKSAALVTIY
(PRED)	asru_1_15_o0045	SIAEALASRA	CIYCWDNATR	GLDSSTALEY	AQAIRIATNF	LKHSSLVTLY
(PRED)	wian_1_1_a02920	SIAEALSARA	AVYCWDNATR	GLDASTALEY	AQAIRTTTNL	MKSASFVTIY
(PRED)	wian_1_1_a02930	SIAEALSARA	AVYCWDNATR	GLDASTALEY	AQAIRTTTNL	MKSASFVTIY

.........610 ....620 ....630 ....640 650

(PRED)	asac_1_6_f03560	QASENIYETF	DKVLVLFEGR	QIYFGDVMNA	KAYFECMGYI	CPARQSTAEF
(PRED)	ergo_1_abr125c	QASENIYETF	DKVLVLFEGR	QIYFGDVMSA	KAYFEEMGYT	CPPRQSTTEF
(PRED)	ercy_1_3604	QASEKIYEVF	DKVTVLYDGK	QIYFGDVMSA	REYFYEMGYM	CPPRQSTPEF
(PRED)	cagl_1_i04862g	QASENIYETF	DKVTVLYTGR	QIYFGPIDEA	KDYFYRMGYE	CPPRQVTAEF
(PRED)	kaaf_1_c00830	QASENIYEKF	DKVTVLYAGR	QIYYGPIHEA	KEYFAEMGYL	CPPRQATAEF
(PRED)	kana_1_k01350	QASENIYETF	DKVVVLYEGR	QIYYGEIDDA	KDYFAKMGYL	CPPRQVTAEF
(PRED)	saar_1_2_b02590	QASENIYETF	DKVTVLYSGK	QIYFGLINEA	KPYFAKMGYL	CPPRQATAEF
(PRED)	sace_1_ydr011w	QASENIYETF	DKVTVLYSGK	QIYFGLIHEA	KPYFAKMGYL	CPPRQATAEF
(PRED)	sace_16_1_a0238	QASENIYETF	DKVTVLYSGK	QIYFGLIHEA	KPYFAKMGYL	CPPRQATAEF
(PRED)	sace_45_1_a0242	QASENIYETF	DKVTVLYSGK	QIYFGLIHEA	KPYFAKMGYL	CPPRQATAEF
(PRED)	sace_48_1_a0238	QASENIYETF	DKVTVLYSGK	QIYFGLIHEA	KPYFAKMGYL	CPPRQATAEF
(PRED)	sace_60_4_d0244	QASENIYETF	DKVTVLYSGK	QIYFGLIHEA	KPYFAKMGYL	CPPRQATAEF
(PRED)	sace_52_1_a0240	QASENIYETF	DKVTVLYSGK	QIYFGLIHEA	KPYFAKMGYL	CPPRQATAEF
(PRED)	sace_46_1_a0240	QASENIYETF	DKVTVLYSGK	QIYFGLIHEA	KPYFAKMGYL	CPPRQATAEF
(PRED)	sace_25_1_a0240	QASENIYETF	DKVTVLYSGK	QIYFGLIHEA	KPYFAKMGYL	CPPRQATAEF
(PRED)	sace_24_1_2300	QASENIYETF	DKVTVLYSGK	QIYFGLIHEA	KPYFAKMGYL	CPPRQATAEF
(PRED)	sace_47_1_a0240	QASENIYETF	DKVTVLYSGK	QIYFGLIHEA	KPYFAKMGYL	CPPRQATAEF
(PRED)	sace_7_1_a02410	QASENIYETF	DKVTVLYSGK	QIYFGLIHEA	KPYFAKMGYL	CPPRQATAEF
(PRED)	sace_59_110_df0	QASENIYETF	DKVTVLYSGK	QIYFGLIHEA	KPYFAKMGYL	CPPRQATAEF
(PRED)	sace_56_1_a0202	QASENIYETF	DKVTVLYSGK	QIYFGLIHEA	KPYFAKMGYL	CPPRQATAEF
(PRED)	sace_40_1_a0239	QASENIYETF	DKVTVLYSGK	QIYFGLIHEA	KPYFAKMGYL	CPPRQATAEF
(PRED)	sace_15_1_a0242	QASENIYETF	DKVTVLYSGK	QIYFGLIHEA	KPYFAKMGYL	CPPRQATAEF
(PRED)	sace_37_1_a0243	QASENIYETF	DKVTVLYSGK	QIYFGLIHEA	KPYFAKMGYL	CPPRQATAEF
(PRED)	sace_9_1_a02440	QASENIYETF	DKVTVLYSGK	QIYFGLIHEA	KPYFAKMGYL	CPPRQATAEF
(PRED)	sace_22_1_2300	QASENIYETF	DKVTVLYSGK	QIYFGLIHEA	KPYFAKMGYL	CPPRQATAEF
(PRED)	sace_29_1_2290	QASENIYETF	DKVTVLYSGK	QIYFGLIHEA	KPYFAKMGYL	CPPRQATAEF
(PRED)	sace_34_1_2320	QASENIYETF	DKVTVLYSGK	QIYFGLIHEA	KPYFAKMGYL	CPPRQATAEF
(PRED)	sace_58_25_y007	QASENIYETF	DKVTVLYSGK	QIYFGLIHEA	KPYFAKMGYL	CPPRQATAEF
(PRED)	sace_23_1_2290	QASENIYETF	DKVTVLYSGK	QIYFGLIHEA	KPYFAKMGYL	CPPRQATAEF
(PRED)	sace_6_120_dp00	QASENIYETF	DKVTVLYSGK	QIYFGLIHEA	KPYFAKMGYL	CPPRQATAEF
(PRED)	sace_57_1_a0241	QASENIYETF	DKVTVLYSGK	QIYFGLIHEA	KPYFAKMGYL	CPPRQATAEF
(PRED)	sace_17_1_a0241	QASENIYETF	DKVTVLYSGK	QIYFGLIHEA	KPYFAKMGYL	CPPRQATAEF
(PRED)	sace_21_1_2310	QASENIYETF	DKVTVLYSGK	QIYFGLIHEA	KPYFAKMGYL	CPPRQATAEF
(PRED)	sace_49_1_a0246	QASENIYETF	DKVTVLYSGK	QIYFGLIHEA	KPYFAKMGYL	CPPRQATAEF
(PRED)	sace_8_2_b02430	QASENIYETF	DKVTVLYSGK	QIYFGLIHEA	KPYFAKMGYL	CPPRQATAEF
(PRED)	sace_31_1_2300	QASENIYETF	DKVTVLYSGK	QIYFGLIHEA	KPYFAKMGYL	CPPRQATAEF
(PRED)	sace_50_1_a0241	QASENIYETF	DXVTVLYSGK	QIYFGLIHEA	KPYFAKMGYL	CPPRQATAEF
(PRED)	sace_4_1_a02360	QASENIYETF	DKVTVLYSGK	QIYFGLIHEA	KPYFAKMGYL	CPPRQATAEF
(PRED)	sace_2_1_a02390	QASENIYETF	DKVTVLYSGK	QIYFGLIHEA	KPYFAKMGYL	CPPRQATAEF
(PRED)	sace_5_33_ag005	QASENIYETF	DKVTVLYSGK	QIYFGLIHEA	KPYFAKMGYL	CPPRQATAEF
(PRED)	sapa_11_1_a0247	QASENIYETF	DKVTVLYSGK	QIYFGLIHEA	KPYFAKMGYL	CPPRQATAEF
(PRED)	sapa_25_1_a0246	QASENIYETF	DKVTVLYSGK	QIYFGLIHEA	KPYFAKMGYL	CPPRQATAEF
(PRED)	sapa_4_1_a02470	QASENIYETF	DKVTVLYSGK	QIYFGLIHEA	KPYFAKMGYL	CPPRQATAEF
(PRED)	sapa_5_1_2350	QASENIYETF	DKVTVLYSGK	QIYFGLIHEA	KPYFAKMGYL	CPPRQATAEF
(PRED)	sapa_9_1_2360	QASENIYETF	DKVTVLYSGK	QIYFGLIHEA	KPYFAKMGYL	CPPRQATAEF
(PRED)	sapa_14_1_a0244	QASENIYETF	DKVTVLYSGK	QIYFGLIHEA	KPYFAKMGYL	CPPRQATAEF
(PRED)	sapa_8_1_2350	QASENIYETF	DKVTVLYSGK	QIYFGLIHEA	KPYFAKMGYL	CPPRQATAEF
(PRED)	sapa_17_1_2380	QASENIYETF	DKVTVLYSGK	QIYFGLIHEA	KPYFAKMGYL	CPPRQATAEF
(PRED)	sapa_7_1_2370	QASENIYETF	DKVTVLYSGK	QIYFGLIHEA	KPYFAKMGYL	CPPRQATAEF
(PRED)	sapa_2_1_a02460	QASENIYETF	DKVTVLYSGK	QIYFGLIHEA	KPYFAKMGYL	CPPRQATAEF
(PRED)	sapa_23_1_a0248	QASENIYETF	DKVTVLYSGK	QIYFGLIHEA	KPYFAKMGYL	CPPRQATAEF
(PRED)	sapa_3_1_a02470	QASENIYETF	DKVTVLYSGK	QIYFGLIHEA	KPYFAKMGYL	CPPRQATAEF
(PRED)	sapa_18_1_2390	QASENIYETF	DKVTVLYSGK	QIYFGLIHEA	KPYFAKMGYL	CPPRQATAEF
(PRED)	sami_1_4_244	QASENIYETF	DKVTVLYSGK	QIYFGLINEA	KPYFAKMGYL	CPPRQATAEF
(PRED)	saku_1_4_262	QASENIYETF	DKVTVLYSGK	QIYFGLINEA	KPYFAKMGYL	CPPRQVTAEF
(PRED)	saba_1_58_bf002	QASENIYETF	DKVTVLYSGR	QIYYGLIHEA	KPYFAKMGYL	CPPRQATAEF
(PRED)	saeu_1_4_d02400	QASENIYETF	DKVTVLYSGR	QIYYGPIHEA	KPYFAKMGYI	CPPRQVTAEF
(PRED)	naca_1_e01640	QASENIYETF	DKVTVLYSGR	QIYYGPIHEA	KDYFAQMGYL	CPPRQVTAEF
(PRED)	nada_1_g01850	QASENIYSLF	DKVSLLYNGR	QIYYGPVTEA	KEYFDRMGYE	CPPRQVTAEF
(PRED)	naca_1_e01630	QASENIYETF	DKVTVLAKGR	QIYFGKIEDA	KAYFENMGYI	CPPRQVMAEF
(PRED)	nada_1_g01840	QASENIYETF	DKVLVLCQGK	EIYYGHIENA	KAYFEKMGYD	CPPRQATAEF
(PRED)	kaaf_1_c00820	QASENIYETF	DKVTVLHSGR	QIYFGKIEDA	KKYFTDMGYI	CPPRQVTAEF
(PRED)	teph_1_m00640	QASENIYQTF	DKVTVLYLGR	QIYFGKIEDA	KVYFKKMGYE	CPPRQATAEF
(PRED)	vapo_1_1036_28	QASENIYECF	DKVVVLFAGR	QIYYGKINDA	KDYFARMGYI	CPSRQSTAEF
(PRED)	tebl_1_i01760	QASENIYNTF	DKVTVLYLGR	QIYYGKIEDA	IPYFEKMGYE	KPSRQPTAEF
(PRED)	tode_1_d04040	QASENIYETF	DKVTVLYSGR	QIYYGHTSKA	KNFFWKMGYS	CPPRQATAEF
(PRED)	naca_1_e01650	QASENIYETF	DKVTVLYDGR	QIYYGGIHEA	TEYFTEMGYE	RPSRQATAEY
(PRED)	tebl_1_g02820	QASENIYNCF	DKVTVLYLGK	QIYFGKIRDA	KDYFYRMGYE	CPSRQVTAEF
(PRED)	lakl_1_c11616g	QASENIYETF	DKVTVLYDGR	QIYFGTVTSA	KDYFERMGFI	CPPRQATAEF
(PRED)	saar_1_8_h03780	QASENIYETF	DKVTVLYAGR	QVFHGKTTHA	KKFFENMGYL	CPPRQSTAEY
(PRED)	sace_14_7_g0015	QASENIYETF	DKVTVLYAGR	QIFCGKTTEA	KDYFENMGYL	CPPRQSTAEY
(PRED)	sace_15_7_g0387	QASENIYETF	DKVTVLYAGR	QIFCGKTTEA	KDYFENMGYL	CPPRQSTAEY
(PRED)	sace_24_8_3780	QASENIYETF	DKVTVLYAGR	QIFCGKTTEA	KDYFENMGYL	CPPRQSTAEY
(PRED)	sace_40_8_h0383	QASENIYETF	DKVTVLYAGR	QIFCGKTTEA	KDYFENMGYL	CPPRQSTAEY
(PRED)	sace_6_169_fm00	QASENIYETF	DKVIVLYAGR	QIFCGKTTEA	KDYFENMGYL	CPPRQSTAEY

(PRED)	sace_19_7_3840	QASENIYETF	DKVIVLYAGR	QIFCGKTTEA	KDYFENMGYL	CPPRQSTAEY
(PRED)	sace_32_7_3770	QASENIYETF	DKVTVLYAGR	QIFCGKTTEA	KDYFENMGYL	CPPRQSTAEY
(PRED)	sace_56_17_q011	QASENIYETF	DKVTVLYAGR	QIFCGKTTEA	KDYFENMGYL	CPPRQSTAEY
(PRED)	sace_5_78_bz001	QASENIYETF	DKVTVXYAGR	QIFCGKTTEX	KDYFENMGYL	CXPRQSTAEX
(PRED)	sace_2_8_h03860	QASENIYETF	DKVIVLYAGR	QIFCGKTTEA	KDYFENMGYL	CPPRQSTAEY
(PRED)	sace_53_29_ac00	QASENIYETF	DKVTVLYAGR	QIFCGKTTEA	KDYFENMGYL	CPPRQSTAEY
(PRED)	sace_17_7_g0393	QASENIYETF	DKVTVLYAGR	QIFCGKTTEA	KDYFENMGYL	CPPRQSTAEY
(PRED)	sace_25_7_g0388	QASENIYETF	DKVTVLYAGR	QIFCGKTTEA	KDYFENMGYL	CPPRQSTAEY
(PRED)	sace_37_7_g0385	QASENIYETF	DKVTVLYAGR	QIFCGKTTEA	KDYFENMGYL	CPPRQSTAEY
(PRED)	sace_9_7_g00180	QASENIYETF	DKVTVLYAGR	QIFCGKTTEA	KDYFENMGYL	CPPRQSTAEY
(PRED)	sace_60_6_f0335	QASENIYETF	DKVTVLYAGR	QIFCGKTTEA	KDYFENMGYL	CPPRQSTAEY
(PRED)	sace_59_336_lx0	QASENIYETF	DKVTVLYAGR	QIFCGKTTEA	KDYFENMGYL	CPPRQSTAEY
(PRED)	sace_31_7_3780	QASENIYETF	DKVTVLYAGR	QIFCGKTTEA	KDYFENMGYL	CPPRQSTAEY
(PRED)	sace_34_8_3770	QASENIYETF	DKVTVLYAGR	QIFCGKTTEA	KDYFENMGYL	CPPRQSTAEY
(PRED)	sace_58_71_bs00	QASENIYETF	DKVTVLYAGR	QIFCGNTTEA	KDYFENMGYL	CPPRQSTAEY
(PRED)	sace_7_7_g03880	QASENIYETF	DKVTVLYAGR	QIFCGKTTEA	KDYFENMGYL	CPPRQSTAEY
(PRED)	sace_35_7_3840	QASENIYETF	DKVTVLYAGR	QIFCGKTTEA	KDYFENMGYL	CPPRQSTAEY
(PRED)	sace_43_7_g0387	QASENIYETF	DKVTVLYAGR	QIFCGKTTEA	KDYFENMGYL	CPPRQSTAEY
(PRED)	sace_57_8_h0390	QASENIYETF	DKVTVLYAGR	QIFCGKTTEA	KDYFENMGYL	CPPRQSTAEY
(PRED)	sace_45_7_g0389	QASENIYETF	DKVTVLYAGR	QIFCGKTTEA	KDYFENMGYL	CPPRQSTAEY
(PRED)	sace_46_8_h0391	QASENIYETF	DKVTVLYAGR	QIFCGKTTEA	KDYFENMGYL	CPPRQSTAEY
(PRED)	sace_23_7_3860	QASENIYETF	DKVTVLYAGR	QIFCGKTTEA	KDYFENMGYL	CPPRQSTAEY
(PRED)	sace_21_7_3790	QASENIYETF	DKVTVLYAGR	QIFCGKTTES	KDYFENMGYL	CPPRQSTAEY
(PRED)	sace_8_73_bu001	QASENIYETF	DKVTVLYAGR	QIFCGKTTEA	KDYFENMGYL	CPPRQSTAEY
(PRED)	sapa_1_8_h03820	QASENIYETF	DKVTVLYAGR	QIFYGKTAEA	KNYFENMGYL	CPPRQSTAEY
(PRED)	sapa_21_8_h0387	QASENIYETF	DKVTVLYAGR	QIFYGKTAEA	KNYFENMGYL	CPPRQSTAEY
(PRED)	sapa_20_8_h0386	QASENIYETF	DKVTVLYAGR	QIFYGKTAEA	KNYFENMGYL	CPPRQSTAEY
(PRED)	sapa_22_8_h0390	QASENIYETF	DKVTVLYAGR	QIFYGKTAEA	KNYFENMGYL	CPPRQSTAEY
(PRED)	sapa_25_8_h0387	QASENIYETF	DKVTVLYAGR	QIFYGKTAEA	KNYFENMGYL	CPPRQSTAEY
(PRED)	sapa_6_8_3750	QASENIYETF	DKVTVLYAGR	QIFYGKTAEA	KNYFENMGYL	CPPRQSTAEY
(PRED)	sapa_9_8_3720	QASENIYETF	DKVTVLYAGR	QIFYGKTAEA	KNYFENMGYL	CPPRQSTAEY
(PRED)	sapa_19_8_h0390	QASENIYETF	DKVTVLYAGR	QIFYGKTAEA	KNYFENMGYL	CPPRQSTAEY
(PRED)	sapa_24_8_h0385	QASENIYETF	DKVTVLYAGR	QIFYGKTAEA	KNYFENMGYL	CPPRQSTAEY
(PRED)	sapa_4_8_h03850	QASENIYETF	DKVTVLYAGR	QIFYGKTAEA	KNYFENMGYL	CPPRQSTAEY
(PRED)	sapa_10_8_3760	QASENIYETF	DKVTVLYAGR	QIFYGKTAEA	KNYFENMGYL	CPPRQSTAEY
(PRED)	sapa_13_8_h0382	QASENIYETF	DKVTVLYAGR	QIFYGKTAEA	KNYFENMGYL	CPPRQSTAEY
(PRED)	sapa_8_8_3750	QASENIYETF	DKVTVLYAGR	QIFYGKTAEA	KNYFENMGYL	CPPRQSTAEY
(PRED)	sapa_11_8_h0383	QASENIYETF	DKVTVLYAGR	QIFYGKTAEA	KNYFENMGYL	CPPRQSTAEY
(PRED)	sapa_5_8_3700	QASENIYETF	DKVTVLYAGR	QIFYGKTAEA	KNYFENMGYL	CPPRQSTAEY
(PRED)	sapa_16_8_h0389	QASENIYETF	DKVTVLYAGR	QIFYGKTTEA	KNYFENMGYL	CPPRQSTAEY
(PRED)	sapa_17_8_3730	QASENIYETF	DKVTVLYAGR	QIFYGKTTEA	KNYFENMGYL	CPPRQSTAEY
(PRED)	sapa_2_8_h03860	QASENIYETF	DKVTVLYAGR	QIFYGKTTEA	KNYFENMGYL	CPPRQSTAEY
(PRED)	sapa_7_8_3740	QASENIYETF	DKVTVLYAGR	QIFYGKTTEA	KNYFENMGYL	CPPRQSTAEY
(PRED)	sapa_23_8_h0385	QASENIYETF	DKVTVLYAGR	QIFYGKTTEA	KNYFENMGYL	CPPRQSTAEY
(PRED)	sapa_3_8_h03890	QASENIYETF	DKVTVLYAGR	QIFYGKTTEA	KNYFENMGYL	CPPRQSTAEY
(PRED)	sapa_18_8_3730	QASENIYETF	DNVTVLYAGR	QIFYGKTTEA	KNYFENMGYL	CPPRQSTAEY
(PRED)	sami_1_14_399	QASENIYETF	DKVTVLYAGR	QVFYGKATEA	KNYFESMGYL	CPPRQSTAEY
(PRED)	sace_4_8_h03690	QASENIYETF	DKVTVXYAGR	QIFCGKTTEX	KDYFENMGYL	CXPRQSTAEY
(PRED)	saku_1_14_404	QASENIYETF	DKVTVLYAGR	QVFYGKATEA	KDYFENMGYL	CPPRQSTAEY
(PRED)	sace_1_ynr070w	QASENIYETF	DKVTVLYAGR	QIFCGKTTEA	KDYFENMGYL	CPPRQSTAEY
(PRED)	sace_49_8_h0383	QASENIYETF	DKVTVLYAGR	QIFCGKTTEA	KDYFENMGYL	CPPRQSTAEY
(PRED)	saeu_1_2_b00130	QASENIYETF	DKVTVLYAGR	QVFYGKVFEA	KNYFETMGYL	CPPRQSTAEY
(PRED)	sauv_1_7_3	QASENIYETF	DKVTVLYAGR	QVFYGKVTEA	KNYFETMGYL	CPPRQATAEY
(PRED)	sami_1_17_26	QASEKIYETF	DKVTVLYAGR	QIFYGEVTEA	KNYFQAMGYL	CPARQSTAEY
(PRED)	zyba_1_02055_AN	QASENIYETF	DKVTVLHTGR	QIYYGSASAA	KAYFERMGYE	CPPRQATAEF
(PRED)	zyba_1_07912	QASENIYETF	DKVTVLHTGR	QIYYGSASAA	KAYFERMGYE	CPPRQAPAEF
(PRED)	zyba_2_2_b00600	QASENIYETF	DKVTVLHTGR	QIYYGSASAA	KAYFERMGYE	CPPRQATAEF
(PRED)	zyba_3_3_c03460	QASENIYETF	DKVTVLHTGR	QIYYGSASAA	KAYFERMGYE	CPPRQATAEF
(PRED)	zyba_1_04634	QASENIYDTF	DKVTVLHSGR	QIYYGPAEEA	KAYFENMGFE	CPARQATAEF
(PRED)	zyba_1_06675	QASENIYDTF	DKVTVFHSGR	QIYYGPAKEA	KAYFERMGFE	CPARQATAEF
(PRED)	zyba_3_2_b02230	QASENIYDTF	DKVTVFHSGR	QIYYGPAKEA	KAYFERMGFE	CPARQATAEF
(PRED)	zyba_2_1_a00860	QASENIYDTF	DKVTVFHSGR	QIYYGPAKEA	KAYFERMGFE	CPARQATAEF
(PRED)	zyro_1_a04114g	QASENIYETF	DKVTVLHSGR	QIYYGSARDA	KAYFEKMGYL	CPPRQATAEF
(PRED)	zyro_1_b14762g	QASENIYETF	DKVTVLHSGR	QIYYGSTKDA	KDYFARMGYL	CPPRQATAEF
(PRED)	zyba_2_14_n0149	QASENIYEEF	DKVCLLHSGR	QIYFGPAEDA	KSYFERLGYE	CPSRQATAEF
(PRED)	zyba_2_33_ag001	QASENIYQEF	DKVCLLHSGR	QIYFGSAGEA	KSYFERLGYE	CPPRQATAEF
(PRED)	lath_1_a01914g	QASENIYECF	DKVTVLFDGR	QIYYGHVEDA	KAYFKKLGFL	CPARQASAEF
(PRED)	lawa_1_23_5161	QASENIYECF	DKVTVLYDGR	QIFFGAIEQA	KAYFEKLGYR	CPARQASAEF
(PRED)	klae_1_14_n0012	QASENIYETF	DKVTVLYDGK	QIYYGTIHDA	KVFFENMGYV	CPDRQATAEF
(PRED)	klla_1_d03432g	QASENIYETF	DKVTILYEGK	QIYFGRIEEA	KKYFENLGFI	CPARQATAEF
(PRED)	klma_1_1_a01880	QASENIYETF	DKVTVLYDGK	QIYFGRIEDA	KAYFEKIGFV	CPARQATAEF
(PRED)	klwi_1_33_ag001	QASENIYENF	DKVTVLYDGK	QIYFGAVQNA	KKFFENLGFV	CPERQATAEF
(PRED)	teph_1_a04220	QPSEKIYNSF	DDVTVLYQGR	QVYFGPTTDA	KNYFEKMGYE	CPPRQSTAEF
(PRED)	vapo_1_1037_47	QVSENIFHTF	DMVTVLYSGK	QIYYGPVAKA	KDFFERMGYK	CPPRQDTAEF
(PRED)	pata_1_2_b05590	QAGENIYEKF	DKVTVLYQGR	QVYFGPATDA	KSYFENMGFE	CPARQSTAEF

(PRED)	wian_1_3_c04380	QAGENIYETF	DKVTILYDGR	QVYFGPADQA	KDYFERMGFQ	CPPRQATAEF
(PRED)	wian_1_3_c04390	QAGENIYETF	DKVTILYDGR	QVYFGPVEDA	KDYFERMGFH	CPPRQATAEF
(PRED)	wian_1_7_g01010	QAGENIYETF	DKVTILYDGR	QIYFGPTEEA	KGYFERMGFQ	CPPRQATAEF
(PRED)	bain_1_1_a00100	QAGEQIYELF	DKTMVLYAGR	QVYYGPVTEA	KKYFEDMGFQ	CPPRQSTAEF
(PRED)	bain_1_17_q0038	QASESIYELF	DKAMVLYAGR	QVYYGPVTEA	KKYFEDMGFQ	CPPRQSTAEF
(PRED)	bain_1_8_h00410	QAGEQIYELF	DKVTVLYLGR	QVYFGPIHEA	KPYFEAMGYQ	CPRRQSTAEF
(PRED)	caal_1_19_5759	QAGEGIYETF	DRVTVLYDGH	QVYYGPANKA	KKYFEDMGWE	CPPRQSTAEF
(PRED)	caal_11_25_y002	QAGEGIYETF	DRVTVLYDGH	QVYYGPANKA	KKYFEDMGWE	CPPRQSTAEF
(PRED)	caal_4_4_d03320	QAGEGIYETF	DRVTVLYDGH	QVYYGPANKA	KKYFEDMGWE	CPPRQSTAEF
(PRED)	caal_12_26_z005	QAGEGIYETF	DRVTVLYDGH	QVYYGPANKA	KKYFEDMGWE	CPPRQSTAEF
(PRED)	caal_5_30_ad005	QAGEGIYETF	DRVTVLYDGH	QVYYGPANKA	KKYFEDMGWE	CPPRQSTAEF
(PRED)	caal_8_3_c03320	QAGEGIYETF	DRVTVLYDGH	QVYYGPANKA	KKYFEDMGWE	CPPRQSTAEF
(PRED)	caal_6_4_d03280	QAGEGIYETF	DRVTVLYDGH	QVYYGPANKA	KKYFEDMGWE	CPPRQSTAEF
(PRED)	caal_10_3_c0334	QAGEGIYETF	DRVTVLYDGH	QVYYGPANKA	KKYFEDMGWE	CPPRQSTAEF
(PRED)	caal_3_29_ac005	QAGEGIYETF	DRVTVLYDGH	QVYYGPANKA	KKYFEDMGWE	CPPRQSTAEF
(PRED)	caal_2_04989	QAGEGIYEKF	DRVTVLYDGH	QVYYGPANKA	KKYFEDMGWE	CPPRQSTAEF
(PRED)	cadu_1_64350	QAGEGIYETF	DRVTVLYDGH	QIYYGPANKA	KKYFEDMGWE	CPPRQSTAEF
(PRED)	caor_1_h02090	QAGENIYECF	DKVTVLYHGR	QIYFGSAKRA	KKYFEDMGWE	CPARQTTAEF
(PRED)	capa_1_600750	QAGENIYECF	DKVTVLYHGR	QIYFGPAKTA	KKYFEDMGWQ	CPPRQTTAEF
(PRED)	loel_1_04930	QAGENIYECF	DKVTVLYHGR	QIYFGPANKA	KKFFEKMGWQ	CPPRQTTAEF
(PRED)	spar_1_5_e03260	QAGENIYEKF	DKVTVLYHGK	QVYFGPRDQA	KQYFENMGWE	CPLRQTTAEF
(PRED)	sppa_1_7_g03160	QAGENIYEKF	DKVTVLYHGK	QIYFGPRDKA	KRYFENMGWE	CPQRQTTAEF
(PRED)	catr_1_01205	QAGENIYEKF	DKVTVLYEGH	QIYYGPANRA	KKYFENMGWE	CPPRQSTAEF
(PRED)	catr_1_05498	QAGENIYEKF	DKVTILYDGH	QIYYGPANKA	KKYFENMGWE	CPPRQSTAEF
(PRED)	catr_1_05971	QAGENIYEKF	DKVTVLYDGH	QIYYGPANKA	KKYFEDMGWE	CPPRQSTAEF
(PRED)	deha_1_a03696g	QASENIYETF	DKVTVLYKGR	QVYFGPVMEA	KKYFEDMGYE	CPARQSTAEF
(PRED)	deha_2_5_e00720	QASENIYETF	DKVTVLYKGR	QVYFGPVMEA	KKYFEDMGYE	CPPRQSTAEF
(PRED)	scst_1_3_c02890	QAGEQIYETF	DKVTVLYKGR	QVYFGPILEA	KAYFENMGWQ	CPARQSTAEF
(PRED)	mebi_1_8_h00300	QAGEKIYETF	DKVTVLYNGK	QVYFGPVTEA	KAYFERMGFE	CPPRQTTAEF
(PRED)	lakl_1_h21010g	QAGENIYRKF	DKVTVLYDGR	QIYYGTINKA	KPYFERMGFQ	SSPRQTTPEF
(PRED)	caar_1_13_m0142	QAAENIYRLF	DHVTVLYLGR	QIYFGPIDEA	VDYFTRMGFK	KFPRETSPEF
(PRED)	caar_1_14_n0143	QASENIYKLF	DYVTVLYLGR	QIYFGPIDKA	VAYFNKMGYK	KASRETSAEF
(PRED)	hapo_1_1_a07220	QASENIYQLF	DNVTVLYYGR	QIYFGPIKEA	VDYFQRLGFV	KGARETSAEY
(PRED)	ogpa_1_1_a01680	QASENIYQLF	DNVTVLYYGR	QIYFGPIQEA	VDYFQRLGFV	KGARETSAEY
(PRED)	piku_1_96_cr001	QASENIYQLF	DYVTVLYLGR	QIYFGPIGEA	VGYFNRMGYH	KPERQTSSEF
(PRED)	pime_1_4_d03240	QASENIYQLF	DYVTVLYLGR	QIYFGKIQNA	TDFFYRMGYQ	KPTRQTTPEF
(PRED)	pime_1_1_a12110	QASENIYQLF	DYVTVLYLGR	QVYFGPISEA	SDYFERMGYA	RSPRETSSEF
(PRED)	piku_1_227_hs00	QASEKIYRLF	DYVTVLYLSR	QIYFGPIDKA	VEFFTKMGFV	KEDRQTSSEF
(PRED)	pime_1_5_e05800	QASERIYELF	DYVTILYLGR	QVYFGPIHKA	VMYFEKMGFI	KESRQTSCEF
(PRED)	pime_1_1_a07690	QASENIYQLF	DFVTVLYLGR	QIYFGPIHDA	VNYFENLGFA	KHPRETSSEF
(PRED)	debr_2_5_e03380	QASESIYQAF	DHVCVLYAGR	QVYFGPADRA	VAYFERLGFA	RPARQTSSEF
(PRED)	kopa_1_2_b10040	QPSERIYELF	DKVLVLYEGR	QIYFGPADAA	KQFFVDMGYD	CPPRQTTGEF
(PRED)	kopa_2_7_g00500	QPSERIYELF	DKVLVLYEGR	QIYFGPANAA	KQFFVDMGYD	CPPRQTTGEF
(PRED)	asru_1_13_m0119	QAGENIYETF	DKVTVLYEGR	QIYFGPVQNA	KHYFEKMGFQ	CPNRQSTPEF
(PRED)	asru_1_15_o0045	QAGERIYETF	DKVTILYDGY	QIYFGPIEQA	KRFFENMGYA	SLPRQSTTEF
(PRED)	wian_1_1_a02920	QASENIYNLF	DKVTVLYEGR	QIYFGPVERA	RQYFIDMGYG	PLNRQSTTEF
(PRED)	wian_1_1_a02930	QASENIYNLF	DKVTVLYEGR	QIYFGPVHKA	KQYFIDMGYE	PLNRQSTTEF
		660	670	680	690	700
(PRED)	asac_1_6_f03560	LTALT-----	DINGYHIVKP	GYESS--VPR	LAEEFEQYWL	NSKEYRTLVS
(PRED)	ergo_1_abr125c	LTALT-----	DTNGYHVVKP	GHEAT--VPR	LPEEFEQYWL	NSKEYRSLLS
(PRED)	ercy_1_3604	LTALT-----	DTNGYHKIRP	GFENR--VPR	TAEEFEAYWR	QSKEYRELKE
(PRED)	cagl_1_i04862g	LTALT-----	DVNGYHKIRP	GYENK--VPR	TAEEFERYWQ	ESPEYRQLLI
(PRED)	kaaf_1_c00830	LTALT-----	DPKGFHLIKP	GYEHK--VPR	SAEEFEAYWL	NSKEYAQLKN
(PRED)	kana_1_k01350	LTALT-----	DPNGLHQVKP	GYEDK--VPR	SAEEFETLWL	NSPEYQQLLA
(PRED)	saar_1_2_b02590	LTALT-----	DPSGFHLIKP	GYENK--VPR	TAGEFETYWL	NSPEFVQLKN
(PRED)	sace_1_ydr011w	LTALT-----	DPNGFHLIKP	GYENK--VPR	TAEEFETYWL	NSPEFAQMKK
(PRED)	sace_16_1_a0238	LTALT-----	DPNGFHLIKP	GYENK--VPR	TAEEFETYWL	NSPEFAQMKK
(PRED)	sace_45_1_a0242	LTALT-----	DPNGFHLIKP	GYENK--VPR	TAEEFETYWL	NSPEFAQMKK
(PRED)	sace_48_1_a0238	LTALT-----	DPNGFHLIKP	GYENK--VPR	TAEEFETYWL	NSPEFAQMKK
(PRED)	sace_60_4_d0244	LTALT-----	DPNGFHLIKP	GYENK--VPR	TAEEFETYWL	NSPEFAQMKK
(PRED)	sace_52_1_a0240	LTALT-----	DPNGFHLIKP	GYENK--VPR	TAEEFETYWL	NSPEFAQMKK
(PRED)	sace_46_1_a0240	LTALT-----	DPNGFHLIKP	GYENK--VPR	TAEEFETYWL	NSPEFAQMKK
(PRED)	sace_25_1_a0240	LTALT-----	DPNGFHLIKP	GYENK--VPR	TAEEFETYWL	NSPEFAQMKK
(PRED)	sace_24_1_2300	LTALT-----	DPNGFHLIKP	GYENK--VPR	TAEEFETYWL	NSPEFAQMKK
(PRED)	sace_47_1_a0240	LTALT-----	DPNGFHLIKP	GYENK--VPR	TAEEFETYWL	NSPEFAQMKK
(PRED)	sace_7_1_a02410	LTALT-----	DPNGFHLIKP	GYENK--VPR	TAEEFETYWL	NSPEFAQMKK
(PRED)	sace_59_110_df0	LTALT-----	DPNGFHLIKP	GYENK--VPR	TAEEFETYWL	NSPEFAQMKK
(PRED)	sace_56_1_a0202	LTALT-----	DPNGFHLIKP	GYENK--VPR	TAEEFETYWL	NSPEFAQMKK
(PRED)	sace_40_1_a0239	LTALT-----	DPNGFHLIKP	GYENK--VPR	TAEEFETYWL	NSPEFAQMKK
(PRED)	sace_15_1_a0242	LTALT-----	DPNGFHLIKP	GYENK--VPR	TAEEFETYWL	NSPEFAQMKK
(PRED)	sace_37_1_a0243	LTALT-----	DPNGFHLIKP	GYENK--VPR	TAEEFETYWL	NSPEFAQMKK
(PRED)	sace_9_1_a02440	LTALT-----	DPNGFHLIKP	GYENK--VPR	TAEEFETYWL	NSPEFAQMKK
(PRED)	sace_22_1_2300	LTALT-----	DPNGFHLIKP	GYENK--VPR	TAEEFETYWL	NSPEFAQMKK
(PRED)	sace_29_1_2290	LTALT-----	DPNGFHLIKP	GYENK--VPR	TAEEFETYWL	NSPEFAQMKK

(PRED)	sace_34_1_2320	LTALT-----	DPNGFHLIKP	GYENK--VPR	TAEEFETYWL	NSPEFAQMKK
(PRED)	sace_58_25_y007	LTALT-----	DPNGFHLIKP	GYENK--VPR	TAEEFETYWL	NSPEFAQMKK
(PRED)	sace_23_1_2290	LTALT-----	DPNGFHLIKP	GYENK--VPR	TAEEFETYWL	NSPEFAQMKK
(PRED)	sace_6_120_dp00	LTALT-----	DPNGFHLIKP	GYENK--VPR	TAEEFETYWL	NSPEFAQMKK
(PRED)	sace_57_1_a0241	LTALT-----	DPNGFHLIKP	GYENK--VPR	TAEEFETYWL	NSPEFAQMKK
(PRED)	sace_17_1_a0241	LTALT-----	DPNGFHLIKP	GYENK--VPR	TAEEFETYWL	NSPEFAQMKK
(PRED)	sace_21_1_2310	LTALT-----	DPNGFHLIKP	GYENK--VPR	TAEEFETYWL	NSPEFAQMKK
(PRED)	sace_49_1_a0246	LTALT-----	DPNGFHLIKP	GYENK--VPR	TAEEFETYWL	NSPEFAQMKK
(PRED)	sace_8_2_b02430	LTALT-----	DPNGFHLIKP	GYENK--VPR	TAEEFETYWL	NSPEFAQMKK
(PRED)	sace_31_1_2300	LTALT-----	DPNGFHLIKP	GYENK--VPR	TAEEFETYWL	NSPEFAQMKK
(PRED)	sace_50_1_a0241	LTALT-----	DPNGFHLIKP	GYENK--VPR	TAEEFETYWL	NSPEFAQMKK
(PRED)	sace_4_1_a02360	LTALT-----	DPNGFHLIKP	GYENK--VPR	TAEEFETYWL	NSPEFAQMKK
(PRED)	sace_2_1_a02390	LTALT-----	DPNGFHLIKP	GYENK--VPR	TAEEFETYWL	NSPEFAQMKK
(PRED)	sace_5_33_ag005	LTALT-----	DPNGFHLIKP	GYENK--VPR	TAEEFETYWL	NSPEFAQMKK
(PRED)	sapa_11_1_a0247	LTALT-----	DPNGYHLIKP	GYENK--VPR	TAEEFETYWL	NSPEFAQMKK
(PRED)	sapa_25_1_a0246	LTALT-----	DPNGYHLIKP	GYENK--VPR	TAEEFETYWL	NSPEFAQMKK
(PRED)	sapa_4_1_a02470	LTALT-----	DPNGYHLIKP	GYENK--VPR	TAEEFETYWL	NSPEFAQMKK
(PRED)	sapa_5_1_2350	LTALT-----	DPNGYHLIKP	GYENK--VPR	TAEEFETYWL	NSPEFAQMKK
(PRED)	sapa_9_1_2360	LTALT-----	DPNGYHLIKP	GYENK--VPR	TAEEFETYWL	NSPEFAQMKK
(PRED)	sapa_14_1_a0244	LTALT-----	DPNGYHLIKP	GYENK--VPR	TAEEFETYWL	NSPEFAQMKK
(PRED)	sapa_8_1_2350	LTALT-----	DPNGYHLIKP	GYENK--VPR	TAEEFETYWL	NSPEFAQMKK
(PRED)	sapa_17_1_2380	LTALT-----	DPNGYHLIKP	GYENK--VPR	TAEEFETYWL	NSPEFAQMKK
(PRED)	sapa_7_1_2370	LTALT-----	DPNGYHLIKP	GYENK--VPR	TAEEFETYWL	NSPEFAQMKK
(PRED)	sapa_2_1_a02460	LTALT-----	DPNGYHLIKP	GYENK--VPR	TAEEFETYWL	NSPEFAQMKK
(PRED)	sapa_23_1_a0248	LTALT-----	DPNGYHLIKP	GYENK--VPR	TAEEFETYWL	NSPEFAQMKK
(PRED)	sapa_3_1_a02470	LTALT-----	DPNGYHLIKP	GYENK--VPR	TAEEFETYWL	NSPEFAQMKK
(PRED)	sapa_18_1_2390	LTALT-----	DPNGYHLIKP	GYENK--VPR	TAEEFETYWL	NSPEFAQMKK
(PRED)	sami_1_4_244	LTALT-----	DPNGFHLIKP	GYEHK--VPR	TAEEFETYWL	NSPEFAQMKN
(PRED)	saku_1_4_262	LTALT-----	DPNGFHLIKP	GYENK--VPR	TAEEFETYWL	NSPEFAQLRN
(PRED)	saba_1_58_bf002	LTALT-----	DPNGFHKIKP	GYENR--VPR	TAKEFETYWL	NSPEFVQLRN
(PRED)	saeu_1_4_d02400	LTALT-----	DPNGFHKIKP	GYENK--VPR	TAKEFETYWT	NSPEFLQLKN
(PRED)	naca_1_e01640	LTALT-----	DPNGYHEIKP	GYEHK--VPR	TAEEFEKYWL	ESDEYAKLRS
(PRED)	nada_1_g01850	LTALT-----	DPNGLHQIKP	GYERK--IPR	SAKEFEDYWL	ASPEYAQLRK
(PRED)	naca_1_e01630	LTAIT-----	DPNGYHKIKS	GFENK--VPV	TPVELEEYWH	NSPEFAKLKE
(PRED)	nada_1_g01840	LTAIT-----	DPNGYHKIKP	GMESK--VPR	NSEELEAYWM	NSVEFVKLKE
(PRED)	kaaf_1_c00820	LTALT-----	DPHGFHKVKP	GYEDK--VPR	TAEEFENYWR	NSPELLQLKR
(PRED)	teph_1_m00640	LTALT-----	DPNGFHIITP	GYENK--VPR	TPEEFETYWE	NSDEFRQLQK
(PRED)	vapo_1_1036_28	LTALT-----	DVNGLHIIKE	GYEHK--VPR	TAEEFEHQWL	NSPEFQQLQQ
(PRED)	tebl_1_i01760	LTSMT-----	DLNGLHIIKP	GYEDK--VPR	TAKQFEEYWH	NSEEFNQLKH
(PRED)	tode_1_d04040	LTALT-----	DPNGFHEIRE	GFEHK--VPR	TAEEFENYWR	NSPEYSDLLT
(PRED)	naca_1_e01650	LTALT-----	DRNGYHQIRP	GFEDK--VPR	SVEEFERYWL	ESPEFAQLRA
(PRED)	tebl_1_g02820	LTALT-----	DPKGYHVIRP	GFESS--VPR	TSAEFEEYWR	NSPEFAQLKQ
(PRED)	lakl_1_c11616g	LTALT-----	DADGLHDIKP	GYENK--VPR	TAAEFEAYWR	ASPEFSQLKN
(PRED)	saar_1_8_h03780	LTALT-----	DPNGLHKIKP	GFEYQ--VPR	SAEEFEKYWL	DSPEFFNLQG
(PRED)	sace_14_7_g0015	LTAIT-----	DPNGLHEIKP	GFEYQ--VPH	TADEFEKYWL	DSPEYARLKG
(PRED)	sace_15_7_g0387	LTAIT-----	DPNGLHEIKP	GFEYQ--VPH	TADEFEKYWL	DSPEYARLKG
(PRED)	sace_24_8_3780	LTAIT-----	DPNGLHEIKP	GFEYQ--VPH	TTDEFEKYWL	DSPEYARLKG
(PRED)	sace_40_8_h0383	LTAIT-----	DPNGLHEIKP	GFEYQ--VPH	TTDEFEKYWL	DSPEYARLKG
(PRED)	sace_6_169_fm00	LTAIT-----	DPNGLHEIKP	GFEYQ--VPH	TTDEFEKYWL	DSPEYARLKG
(PRED)	sace_19_7_3840	LTAIT-----	DPNGLHEIKP	GFEYQ--VPH	TADEFEKYWL	DSPEYARLKG
(PRED)	sace_32_7_3770	LTAIT-----	DPNGLHEIKP	GFEYQ--VPH	TADEFEKYWL	DSPEYARLKG
(PRED)	sace_56_17_q011	LTAIT-----	DPNGLHEIKP	GFEYQ--VPH	TADEFEKYWL	DSPEYARLKG
(PRED)	sace_5_78_bz001	LTAIT-----	DPNGLHEIKP	GXEYQ--VPH	TADEFEKYWL	DSPEYARLKG
(PRED)	sace_2_8_h03860	LTAIT-----	DPNGLHEIKP	GFEYQ--VPH	TTDEFEKYWL	DSPEYARLKG
(PRED)	sace_53_29_ac00	LTAIT-----	DPNGLHEIKP	GFEYQ--VPH	TADEFEKYWL	DSPEYARLKG
(PRED)	sace_17_7_g0393	LTAIT-----	DPNGLHEIKP	GFEYQ--VPH	TADEFEKYWL	DSPEYARLKG
(PRED)	sace_25_7_g0388	LTAIT-----	DPNGLHEIKP	GFEYQ--VPH	TADEFEKYWL	DSPEYARLKG
(PRED)	sace_37_7_g0385	LTAIT-----	DPNGLHEIKP	GFEYQ--VPH	TADEFEKYWL	DSPEYARLKG
(PRED)	sace_9_7_g00180	LTAIT-----	DPNGLHEIKP	GFEYQ--VPH	TADEFEKYWL	DSPEYARLKG
(PRED)	sace_60_6_f0335	LTAIT-----	DPNGLHEIKP	GFEYQ--VPH	TADEFEKYWL	DSPEYARLKG
(PRED)	sace_59_336_lx0	LTAIT-----	DPNGLHEIKP	GFEYQ--VPH	TADEFEKYWL	DSPEYARLKG
(PRED)	sace_31_7_3780	LTAIT-----	DPNGLHEIKP	GFEYQ--VPH	TADEFEKYWL	DSPEYARLKG
(PRED)	sace_34_8_3770	LTAIT-----	DPNGLHEIKP	GFEYQ--VPH	TADEFEKYWL	DSPEYARLKG
(PRED)	sace_58_71_bs00	LTAIT-----	DPNGLHEIKP	GFEYQ--VPH	TADEFEKYWL	DSPEYARLKG
(PRED)	sace_7_7_g03880	LTAIT-----	DPNGLHEIKP	GFEYQ--VPH	TADEFEKYWL	DSPEYARLKG
(PRED)	sace_35_7_3840	LTAIT-----	DPNGLHEIKP	GFEYQ--VPH	TADEFEKYWL	DSPEYARLKG
(PRED)	sace_43_7_g0387	LTAIT-----	DPNGLHEIKP	GFEYQ--VPH	TADEFEKYWL	DSPEYARLKG
(PRED)	sace_57_8_h0390	LTAIT-----	DPNGLHEIKP	GFEYQ--VPH	TADEFEKYWL	DSPEYARLKG
(PRED)	sace_45_7_g0389	LTAIT-----	DPNGLHEIKP	GFEYQ--VPH	TADEFEKYWL	DSPEYARLKG
(PRED)	sace_46_8_h0391	LTAIT-----	DPNGLHEIKP	GFEYQ--VPH	TADEFEKYWL	DSPEYARLKG
(PRED)	sace_23_7_3860	LTAIT-----	DPNGLHEIKP	GFEYQ--VPH	TADEFEKYWL	DSPEYARLKG
(PRED)	sace_21_7_3790	LTAIT-----	DPNGLHEIKP	GFEYQ--VPH	TADEFEKYWL	DSPEYARLKG
(PRED)	sace_8_73_bu001	LTAIT-----	DPNGLHEIKP	GFEYQ--VPH	TADEFEKYWL	DSPEYARLKG
(PRED)	sapa_1_8_h03820	LTAIT-----	DPNGLHQIKP	GFEYE--VPH	TADEFEQYWL	DSPEYTCLQD
(PRED)	sapa_21_8_h0387	LTAIT-----	DPNGLHQIKP	GFEYE--VPH	TADEFEQYWL	DSPEYTCLQD

(PRED)	sapa_20_8_h0386	LTAIT-----	DPNGLHQIKP	GFEYE--VPH	TADEFEQYWL	DSPEYTCLQD
(PRED)	sapa_22_8_h0390	LTAIT-----	DPNGLHQIKP	GFEYE--VPH	TADEFEQYWL	DSPEYTCLQD
(PRED)	sapa_25_8_h0387	LTAIT-----	DPNGLHQIKP	GFEYE--VPH	TADEFEQYWL	DSPEYTCLQD
(PRED)	sapa_6_8_3750	LTAIT-----	DPNGLHQIKP	GFEYE--VPH	TADEFEQYWL	DSPEYTCLQD
(PRED)	sapa_9_8_3720	LTAIT-----	DPNGLHQIKP	GFEYE--VPH	TADEFEQYWL	DSPEYTCLQD
(PRED)	sapa_19_8_h0390	LTAIT-----	DPNGLHQIKP	GFEYE--VPH	TADEFEQYWL	DSPEYTCLQD
(PRED)	sapa_24_8_h0385	LTAIT-----	DPNGLHQIKP	GFEYE--VPH	TADEFEQYWL	DSPEYTCLQD
(PRED)	sapa_4_8_h03850	LTAIT-----	DPNGLHQIKP	GFEYE--VPH	TADEFEQYWL	DSPEYTCLQD
(PRED)	sapa_10_8_3760	LTAIT-----	DPNGLHQIKP	GFEYE--VPH	TADEFEQYWL	DSPEYTCLQD
(PRED)	sapa_13_8_h0382	LTAIT-----	DPNGLHQIKP	GIEYE--VPH	TADEFEQYWL	DSPEYTCLQD
(PRED)	sapa_8_8_3750	LTAIT-----	DPNGLHQIKP	GFEYE--VPH	TADEFEQYWL	DSPEYTCLQD
(PRED)	sapa_11_8_h0383	LTAIT-----	DPNGLHQIKP	GFEYE--VPH	TADEFEQYWL	DSPEYTCLQD
(PRED)	sapa_5_8_3700	LTAIT-----	DPNGLHQIKP	GFEYE--VPH	TADEFEQYWL	DSPEYTCLQD
(PRED)	sapa_16_8_h0389	LTAIT-----	DPNGLHQIKP	GFEYE--VPH	TADEFEQYWL	DSPEYACLQD
(PRED)	sapa_17_8_3730	LTAIT-----	DPNGLHQIKP	GFEYE--VPH	TADEFEQYWL	DSPEYACLQD
(PRED)	sapa_2_8_h03860	LTAIT-----	DPNGLHQIKP	GFEYE--VPH	TADEFEQYWL	DSPEYACLQD
(PRED)	sapa_7_8_3740	LTAIT-----	DPNGLHQIKP	GFEYE--VPH	TADEFEQYWL	DSPEYACLQD
(PRED)	sapa_23_8_h0385	LTAIT-----	DPNGLHQIKP	GFEYE--VPH	TADEFEQYWL	DSPEYACLQD
(PRED)	sapa_3_8_h03890	LTAIT-----	DPNGLHQIKP	GFEYE--VPH	TADEFEQYWL	DSPEYACLQD
(PRED)	sapa_18_8_3730	LTAIT-----	DPNGLHQIKP	GFEYE--VPH	TADEFEQYWL	DSPEYACLQD
(PRED)	sami_1_14_399	LTAIT-----	DPNGLHEIKP	GLRDQ--VPH	TAEEFEKYWL	NSPEYACLQR
(PRED)	sace_4_8_h03690	LTAIT-----	DPNGLHEIKP	GFEYQ--VPH	TADEFEKYWL	DSPEYARLKG
(PRED)	saku_1_14_404	LTAIT-----	DRNGLHKIKP	GFEFH--VPR	TADEFEKYWL	NSPNFSNLQR
(PRED)	sace_1_ynr070w	LTAIT-----	DPNGLHEIKP	GFEYQ--VPH	TADEFEKYWL	DSPEYARLKG
(PRED)	sace_49_8_h0383	LTAIT-----	DPNGLHEIKP	GFEYQ--VPH	TADEFEKYWL	DSPEYARLKG
(PRED)	saeu_1_2_b00130	LTALT-----	DPNGLHNIRP	GYEDQ--IPR	TADEFEKYWL	ASSECVSLQR
(PRED)	sauv_1_7_3	LTALT-----	DPNGLHKIRP	GYEDQ--IPR	TAEEFEKYWL	ASSECVSLQR
(PRED)	sami_1_17_26	LTALT-----	DPDGFHEVRP	GFENT--VPR	TAEEFERYWL	DSPEFDNLQR
(PRED)	zyba_1_02055_AN	LTALT-----	DPHGLHRVRS	GYEGK--VPR	TADELEAYWR	ASPEFAALKN
(PRED)	zyba_1_07912	LTALT-----	DPHGLHRVRS	GYEGK--VPR	TADELEAYWR	ASPEFAALKN
(PRED)	zyba_2_2_b00600	LTALT-----	DPHGLHRVRN	GYEGK--VPR	TADELEAYWR	ASPEFAALKN
(PRED)	zyba_3_3_c03460	LTALT-----	DPHGLHRVRS	GYEGK--VPR	TADELEAYWR	ASPEFAALKN
(PRED)	zyba_1_04634	LTAMT-----	DPDGLHQVRS	GFEGK--VPQ	TADDLEKYWQ	NSPEFAVLKR
(PRED)	zyba_1_06675	LTAMT-----	DPEGLHEVRS	GFEGK--VPQ	TADDLEKYWQ	NSPEFALLKR
(PRED)	zyba_3_2_b02230	LTAMT-----	DPEGLHEVRS	GFEGK--VPQ	TADDLEKYWQ	NSPEFALLKR
(PRED)	zyba_2_1_a00860	LTAMT-----	DPEGLHEVRS	GFEGK--VPQ	TADDLEKYWQ	NSPEFALLKR
(PRED)	zyro_1_a04114g	LTSLT-----	DPNGFHVVRE	GYENK--VPR	TAEDLENYWL	NSEECANLKK
(PRED)	zyro_1_b14762g	LTALT-----	DPNGYHKIRS	GFENK--VPR	TAEEFEDYWL	QSPEFTELRK
(PRED)	zyba_2_14_n0149	LTAIT-----	DPNGLHKILP	GFEDK--VPR	TAEELEHCWK	SSPEYEALKQ
(PRED)	zyba_2_33_ag001	LTAIT-----	DPNGLHKTIP	GFEDR--VPH	TAEDLERCWK	SSPEYEALKQ
(PRED)	lath_1_a01914g	LTALT-----	DPNGLHEYVP	GFENK--VPR	TADEFEKLWL	ESQEYQDLLK
(PRED)	lawa_1_23_5161	LTALT-----	DPQGLHEYVP	GFDDR--VPR	TADEFEKCWL	ESAEYQRLLA
(PRED)	klae_1_14_n0012	LTALT-----	DPSGLHDIKP	GFEGK--VPR	TRDEFVKYWE	ESEEYKNLID
(PRED)	klla_1_d03432g	LTSLT-----	DSKGLRRVRP	GFENK--VPR	TRDDFVRVWE	ESKEYHDLIQ
(PRED)	klma_1_1_a01880	LTSLT-----	DPNGFHHVKP	GYEGK--VPR	TREDFVRVWE	ESEEYQQLLQ
(PRED)	klwi_1_33_ag001	LTSLT-----	DPNGGFGVKP	GYENR--VPR	TRDDFVRCWE	QSQEFADLLS
(PRED)	teph_1_a04220	LTSVT-----	DLNGYHKFKD	GFEKN--VPK	TAIDFENYWV	NSPEYQELLR
(PRED)	vapo_1_1037_47	LTALT-----	DPNGLHLIKE	GFEGT--VPR	SADEFESYWK	SSPEFKQLLS
(PRED)	pata_1_2_b05590	LTAVT-----	DPIGRFP-KP	GFENK--VPK	TAAEFEEYWL	NSNEYHQLMR
(PRED)	wian_1_3_c04380	LTAIT-----	DPLGRYP-KP	GFEKK--VPE	SADDFERYWH	NSPEYKKMIN
(PRED)	wian_1_3_c04390	LTAIT-----	DPLGRFP-KD	GFEHK--VPQ	TADEFEAYWH	KSPEYEKLLN
(PRED)	wian_1_7_g01010	LTAIT-----	DPLGRFP-KS	GMEDK--VPR	TADDFEAYWH	NSDEYKLMVQ
(PRED)	bain_1_1_a00100	LTAIT-----	DPIGRFA-QP	GYEKR--VPH	TAEEFEKYWH	ESREYAKLTG
(PRED)	bain_1_17_q0038	LTAIT-----	DPIGRFA-KP	GYEKR--VPH	TAEEFERYWQ	ESRNYAKLTG
(PRED)	bain_1_8_h00410	LTAVT-----	DPIGRYT-KP	GWENR--VPQ	TAEEFEKYWH	RSPQYKALLE
(PRED)	caal_1_19_5759	LTAIT-----	DPIGRFP-RA	GWENK--VPR	TAQDFEHYWL	NSPQYQELMQ
(PRED)	caal_11_25_y002	LTAIT-----	DPIGRFP-RA	GWENK--VPR	TAQDFEHYWL	NSPQYQELMQ
(PRED)	caal_4_4_d03320	LTAIT-----	DPIGRFP-RA	GWENK--VPR	TAQDFEHYWL	NSPQYQELMQ
(PRED)	caal_12_26_z005	LTAIT-----	DPIGRFP-RA	GWENK--VPR	TAQDFEHYWL	NSPQYQELMQ
(PRED)	caal_5_30_ad005	LTAIT-----	DPIGRFP-RA	GWENK--VPR	TAQDFEHYWL	NSPQYQELMQ
(PRED)	caal_8_3_c03320	LTAIT-----	DPIGRFP-RA	GWENK--VPR	TAQDFEHYWL	NSPQYQELMQ
(PRED)	caal_6_4_d03280	LTAIT-----	DPIGRFP-RA	GWENK--VPR	TAQDFEHYWL	NSPQYQELMQ
(PRED)	caal_10_3_c0334	LTAIT-----	DPIGRFP-RA	GWENK--VPR	TAQDFEHYWS	NSPQYQELMQ
(PRED)	caal_3_29_ac005	LTAIT-----	DPIGRFP-RA	GWENK--VPR	TAQDFEHYWL	NSPQYQELMQ
(PRED)	caal_2_04989	LTAIT-----	DPIGRFP-RA	GWENK--VPR	TAQDFEHYWL	NSPQYQELMQ
(PRED)	cadu_1_64350	LTAIT-----	DPIGRFP-RA	GWENK--VPR	TAQDFEHYWL	NSPQYQELMQ
(PRED)	caor_1_h02090	LTALT-----	DPIGRFA-KE	GWENK--VPQ	TAEEFEAYWL	RSNEYKELLQ
(PRED)	capa_1_600750	LTALT-----	DPIGRFT-KK	GWENK--VPQ	TAEEFEARWL	ASKEYKLLLQ
(PRED)	loel_1_04930	LTALT-----	DPIGRFA-KP	GWENK--VPQ	TAEEFESYWL	RSEEYKLLLD
(PRED)	spar_1_5_e03260	LTAIT-----	DPIGRYP-RK	GFEDK--IPQ	TAEEFEEYWL	RSPEYKQLLQ
(PRED)	sppa_1_7_g03160	LTAVT-----	DPIGRYP-RQ	GYENK--VPQ	TAEEFEAYWL	KSPEYKQLIN
(PRED)	catr_1_01205	LTALT-----	DPIGRFP-KK	GWEYK--VPR	TAEEFEDRWL	NSNEYKELIQ
(PRED)	catr_1_05498	LTAVT-----	DPIGRFP-KK	GWEDK--VPR	TAEDFESRWL	NSPQYNELLN
(PRED)	catr_1_05971	LTALT-----	DPIGRFP-KK	GWENK--VPR	TAEDFESRWL	NSVQYKELLN
(PRED)	deha_1_a03696g	LTAVT-----	DPIGRYA-KP	GMGNK--VPS	TAEEFEDYWL	KSEQYRILQQ

```
(PRED) deha_2_5_e00720 LTAVT-----  DPIGRYP-KP  GMENK--VPN  TAEEFEDYWL  KSEQYRILQQ
(PRED) scst_1_3_c02890 LTAIT-----  DPLGRTA-KP  GYEDK--VPS  TAEDFERYWL  NSPEYKKMID
(PRED) mebi_1_8_h00300 LTAVT-----  DPAGRFP-FK  GMEDK--VPQ  TPEEFGNYWK  NSPEYSRLQQ
(PRED) lakl_1_h21010g  LTAITQRIDS  EDSTAVKIRE  EFKNT--IPR  TAEDFERVWR  GSPEYQELIQ
(PRED) caar_1_13_m0142 LTVVT-----  DPLA-RVPLP  GFE-NK-VPS  TPDDFEAYWL  QSPEYQQLLE
(PRED) caar_1_14_n0143 LTAVT-----  DPLA-RVIIE  GFE-NK-VPK  SADEFESYWL  NSPDYQEVLQ
(PRED) hapo_1_1_a07220 LTSVT-----  DPLA-RKVAP  GFE-HK-APR  NAEEFEARWL  SSPEFEALMK
(PRED) ogpa_1_1_a01680 LTSVT-----  DPLA-RKVAS  GFE-HK-VPR  NAEEFEARWR  SSPEFDALMK
(PRED) piku_1_96_cr001 LTAVT-----  DPLA-RHIKP  GFDISR-VPQ  TADEFEEYWK  NSPEYAKLLK
(PRED) pime_1_4_d03240 LTAIT-----  DPLA-RTLRP  GFHKSS-VPS  NADEFEEYWR  NSPEFAELEK
(PRED) pime_1_1_a12110 LTSVT-----  DPLA-RNLKP  G--VTD-APN  TADEFEQYWK  NSPEFANLCE
(PRED) piku_1_227_hs00 LTSIT-----  DANA-RKCRP  GLEEK--LPQ  TPEEFETYWR  NSLEYKELMN
(PRED) pime_1_5_e05800 LTSVT-----  DPLA-RKSKP  GL-EN--LPS  SADEFESYWK  NSPEYALLQQ
(PRED) pime_1_1_a07690 LTSIT-----  DPLA-RKIDP  SFKDKSLIPQ  TADQFEAVWR  NSEEFKSLQN
(PRED) debr_2_5_e03380 LTCVT-----  DPDA-RTARP  GFEGR--VPH  TAGQFESLWR  GSPEFAAL-K
(PRED) kopa_1_2_b10040 LTAVT-----  DPLQ-RYPRP  GFENR--VPI  NADEFQEYWR  ASSTYSDLQN
(PRED) kopa_2_7_g00500 LTAVT-----  DPLQ-RYPRP  GFENK--VPI  NADEFHEYWK  ASSTYSELQN
(PRED) asru_1_13_m0119 LTAIT-----  DPIGRIP-QP  GMEHK--VPR  NALEFEDYWL  KSDEYQQLMA
(PRED) asru_1_15_o0045 LTGVT-----  DSEERIA-KP  GFENK--VPR  TAQEFEDYWL  KSQDYQNLIT
(PRED) wian_1_1_a02920 LTSVT-----  DPLGRFA-RE  GYEHK--VPH  SPAEFEEYWL  KSQEYKVLKT
(PRED) wian_1_1_a02930 LTSVT-----  DPLGRFA-RE  GYEHK--VPH  TPAEFEEYWL  NSPEYKVLQT

                       .........710 .........720 .........730 .........740 .........750
(PRED) asac_1_6_f03560 EIEDYR-KEV  DA---RQTRD  QLEQFKLKGK  SKFTHKRSPF  MISFFEQVKL
(PRED) ergo_1_abr125c  EIEDYR-REV  DA---HETRD  QLEKFKLKGK  SKYTHKRSPF  MISFFEQVKL
(PRED) ercy_1_3604     EIEDYK-NQV  DG---DITKE  QLQKYKLQRK  SKYSRKRSPY  VLSFAEQVKL
(PRED) cagl_1_i04862g  DIDQYK-KEI  DT---EKTKE  IYDQSMQQEK  SKHARKKSYY  TVSFWEQIRL
(PRED) kaaf_1_c00830   EIQTYK-EEV  DS---EKTKE  LYDMSMADEK  SKGARKKSYY  TTSYLEQVRL
(PRED) kana_1_k01350   DIAAYK-TEV  DG---ARTKE  IYNQSMADEK  SKGTRKKSYY  TLTYWEQVRL
(PRED) saar_1_2_b02590 DITTYN-ERV  NT---EKTKE  VYDVSMAQEK  SKYTRKKSYY  TISYWQQVRL
(PRED) sace_1_ydr011w  DIAAYK-EKV  NT---EKTKE  VYDESMAQEK  SKYTRKKSYY  TVSYWEQVKL
(PRED) sace_16_1_a0238 DIAAYK-EKV  NT---EKTKE  VYDESMAQEK  SKYTRKKSYY  TVSYWEQVKL
(PRED) sace_45_1_a0242 DIAAYK-EKV  NT---EKTKE  VYDESMAQEK  SKYTRKKSYY  TVSYWEQVKL
(PRED) sace_48_1_a0238 DIAAYK-EKV  NT---EKTKE  VYDESMAQEK  SKYTRKKSYY  TVSYWEQVKL
(PRED) sace_60_4_d0244 DIAAYK-EKV  NT---EKTKE  VYDESMAQEK  SKYTRKKSYY  TVSYWEQVKL
(PRED) sace_52_1_a0240 DIAAYK-EKV  NT---EKTKE  VYDESMAQEK  SKYTRKKSYY  TVSYWEQVKL
(PRED) sace_46_1_a0240 DIAAYK-EKV  NT---EKTKE  VYDESMAQEK  SKYTRKKSYY  TVSYWEQVKL
(PRED) sace_25_1_a0240 DIAAYK-EKV  NT---EKTKE  VYDESMAQEK  SKYTRKKSYY  TVSYWEQVKL
(PRED) sace_24_1_2300  DIVAYK-EKV  NT---EKTKE  VYDESMAQEK  SKYTRKKSYY  TVSYWEQVKL
(PRED) sace_47_1_a0240 DIVAYK-EKV  NT---EKTKE  VYDESMAQEK  SKYTRKKSYY  TVSYWEQVKL
(PRED) sace_7_1_a02410 DIVAYK-EKV  NT---EKTKE  VYDESMAQEK  SKYTRKKSYY  TVSYWEQVKL
(PRED) sace_59_110_df0 DIVAYK-EKV  NT---EKTKE  VYDESMAQEK  SKYTRKKSYY  TVSYWEQVKL
(PRED) sace_56_1_a0202 DIVAYK-EKV  NT---EKTKE  VYDESMAQEK  SKYTRKKSYY  TVSYWEQVKL
(PRED) sace_40_1_a0239 DIVAYK-EKV  NT---EKTKE  VYDESMAQEK  SKYTRKKSYY  TVSYWEQVKL
(PRED) sace_15_1_a0242 DIAAYK-EKV  NT---EKTKE  VYDESMAQEK  SKYTRKRSYY  TVSYWEQVKL
(PRED) sace_37_1_a0243 DIAAYK-EKV  NT---EKTKE  VYDESMAQEK  SKYTRKRSYY  TVSYWEQVKL
(PRED) sace_9_1_a02440 DIAAYK-EKV  NT---EKTKE  VYDESMAQEK  SKYTRKRSYY  TVSYWEQVKL
(PRED) sace_22_1_2300  DIAAYK-EKV  NT---EKTKE  VYDESMAQEK  SKYTRKKSYY  TVSYWEQVKL
(PRED) sace_29_1_2290  DIAAYK-EKV  NT---EKTKE  VYDESMAQEK  SKYTRKKSYY  TVSYWEQVKL
(PRED) sace_34_1_2320  DIAAYK-EKV  NT---EKTKE  VYDESMAQEK  SKYTRKKSYY  TVSYWEQVKL
(PRED) sace_58_25_y007 DIAAYK-EKV  NT---EKTKE  VYDESMAQEK  SKYTRKKSYY  TVSYWEQVKL
(PRED) sace_23_1_2290  DIAAYK-EKV  NT---EKTKE  VYDESMAQEK  SKYTRKKSYY  TVSYWEQVKL
(PRED) sace_6_120_dp00 DIAAYK-EKV  NT---EKTKE  VYDESMAQEK  SKYTRKKSYY  TVSYWEQVKL
(PRED) sace_57_1_a0241 DIAAYK-EKV  NT---EKTKE  VYDESMAQEK  SKYTRKKSYY  TVSYWEQVKL
(PRED) sace_17_1_a0241 DTVAYK-EKV  NT---EKTKE  VYDESMAQEK  SKYTRKKSYY  TVSYWEQVKL
(PRED) sace_21_1_2310  DIAAYK-EKV  NT---EKTKE  VYDESMAQEK  SKYTRKKSYY  TVSYWEQVKL
(PRED) sace_49_1_a0246 DIVAYK-EKV  NT---EKTKE  VYDESMAQEK  SKYTRKKSYY  TVSYWEQVKL
(PRED) sace_8_2_b02430 DIVAYK-EKV  NT---EKTKE  VYDESMAQEK  SKYTRKKSYY  TVSYWEQVKL
(PRED) sace_31_1_2300  DIAAYK-EKV  NT---EKTKE  VYDESMAQEK  SKYTRKKSYY  TVSYWEQVKL
(PRED) sace_50_1_a0241 DIVAYK-EKV  NT---EKTKE  VYDESMAQEK  SKYTRKKSYY  TVSYWEQVKL
(PRED) sace_4_1_a02360 DIXAYK-EKV  NT---EKTKE  VYDESMAQEK  SKYTRKKSYY  TVSYWEQVKL
(PRED) sace_2_1_a02390 DIVAYK-EKV  NT---EKTKE  VYDESMAQEK  SKYTRKKSYY  TVSYWEQVKL
(PRED) sace_5_33_ag005 DIXAYK-EKV  NT---EKTKE  VYDESMAQEK  SXYTRKKSYY  TVSYWEQVKL
(PRED) sapa_11_1_a0247 DIATYK-EKV  NT---EKTKE  IYDESMAQEK  SKYTRKKSYY  TVSYWEQVKL
(PRED) sapa_25_1_a0246 DIATYK-EKV  NT---EKTKE  IYDESMAQEK  SKYTRKKSYY  TVSYWEQVKL
(PRED) sapa_4_1_a02470 DIATYK-EKV  NT---EKTKE  IYDESMAQEK  SKYTRKKSYY  TVSYWEQVKL
(PRED) sapa_5_1_2350   DIATYK-EKV  NT---EKTKE  IYDESMAQEK  SKYTRKKSYY  TVSYWEQVKL
(PRED) sapa_9_1_2360   DIATYK-EKV  NT---EKTKE  IYDESMAQEK  SKYTRKKSYY  TVSYWEQVKL
(PRED) sapa_14_1_a0244 DIATYK-EKV  NT---EKTKE  IYDESMAQEK  SKYTRKKSYY  TVSYWEQVKL
(PRED) sapa_8_1_2350   DIATYK-EKV  NT---EKTKE  IYDESMAQEK  SKYTRKKSYY  TVSYWEQVKL
(PRED) sapa_17_1_2380  DIAAYK-EKV  NT---EKTKE  IYDESMAQEK  SKYTRKKSYY  TVSYWEQVKL
(PRED) sapa_7_1_2370   DIAAYK-EKV  NT---EKTKE  IYDESMAQEK  SKYTRKKSYY  TVSYWEQVKL
(PRED) sapa_2_1_a02460 DIAAYK-EKV  NT---EKTKE  IYDESMAQEK  SKYTRKKSYY  TVSYWEQVKL
(PRED) sapa_23_1_a0248 DIAAYK-EKV  NT---EKTKE  IYDESMAQEK  SKYTRKKSYY  TVSYWEQVKL
(PRED) sapa_3_1_a02470 DIAAYK-EKV  NT---EKTKE  IYDESMAQEK  SKYTRKKSYY  TVSYWEQVKL
```

(PRED)	sapa_18_1_2390	DIAGYK-EKV	NT---EKTKE	IYDESMAQEK	SKYTRRKSYY	TVSYWEQVKL
(PRED)	sami_1_4_244	DITAYK-ERV	NT---EKTKE	VYDESMAQEK	SKYTRKKSYY	TISYWEQVRL
(PRED)	saku_1_4_262	DIAAYN-EKV	NT---EKTKE	VYNESMAQEK	SKFTRKKSYY	TVSYWQQVNL
(PRED)	saba_1_58_bf002	DIAAYK-ARV	NT---EKTKE	IYDVSMAQEK	SKYTRNKSYY	TISYWEQIRL
(PRED)	saeu_1_4_d02400	DIAAYK-ARV	NT---EKTKE	VYDASMAQEK	SKYTRSKSYY	TISYWQQIRL
(PRED)	naca_1_e01640	DINTYK-STV	DT---EKTRE	LYKESMADEK	AKGTRKKSYF	TISYMEQVKL
(PRED)	nada_1_g01850	DIAEYK-NSI	DT---DKTRE	IYNKSMAEEK	SKGTRKKSYY	TISYWDQILL
(PRED)	naca_1_e01630	DITTYK-TNV	DT---ENTRE	MYKKSMKEEK	SKHSSKKSFY	TVSFWEQVRL
(PRED)	nada_1_g01840	DISRYK-EDV	DT---ENTRN	LYKDSMADEK	SRFTRRKSRY	NTSYWEQVRL
(PRED)	kaaf_1_c00820	EIEDFK-SRV	DT---TKTKK	IYNESLSQEK	SKYTRKQSYY	TVSYFEQVRL
(PRED)	teph_1_m00640	DIADYK-SKI	DI---EKTKA	LYHESMDQEK	SKYASKKSYF	TISYPQQVQL
(PRED)	vapo_1_1036_28	DIQDYK-DRV	DA---EKTRE	LYQQSMNQEK	SKYSRKKSYF	TVSYPEQVKL
(PRED)	tebl_1_i01760	NIEIYK-KNI	DV---EKTRD	LYKQSIQQEK	SKSSRKQSRY	IISYFEQVRL
(PRED)	tode_1_d04040	DIEKYK-KEM	DT---EGTRE	SYRNSMIQEK	SKHARKSSYY	TVSYWRQLRL
(PRED)	naca_1_e01650	DITSYK-AEI	DS---QKTRE	LYNQSLAEEK	AKGTRTKSYY	TISYLEQIKL
(PRED)	tebl_1_g02820	DIVDYN-QSV	NT---DEIRQ	YYKSSMSQEK	AKGARKKSYY	TLSFPEQVKL
(PRED)	lakl_1_c11616g	EIEAYK-NQV	EA---EKTKE	LYDLSLSQEK	SKYTRSKSYF	TISYFQQVKL
(PRED)	saar_1_8_h03780	EIQEYK-QKV	NA---NNTRR	IYDESMSQEK	SKYARKKSYY	TISYWDQVKL
(PRED)	sace_14_7_g0015	EIQKYK-HEV	NT---EWTKK	TYNESMAQEK	SKGTRKKSYY	TVSYWEQIRL
(PRED)	sace_15_7_g0387	EIQKYK-HEV	NT---EWTKK	TYNESMAQEK	SKGTRKKSYY	TVSYWEQIRL
(PRED)	sace_24_8_3780	EIQKYK-HEV	NT---EWTKK	TYNESMAQEK	SKGTRKKSYY	TVSYWEQIRL
(PRED)	sace_40_8_h0383	EIQKYK-HEV	NT---EWTKK	TYNESMAQEK	SKGTRKKSYY	TVSYWEQIRL
(PRED)	sace_6_169_fm00	EIQKYK-HEV	NT---EWTKK	TYNESMAQEK	SKGTRKKSYY	TVSYWEQIRL
(PRED)	sace_19_7_3840	EIQKYK-HEV	NT---EWTKK	TYNESMAQEK	SKGTRKKSYY	TVSYWEQIRL
(PRED)	sace_32_7_3770	EIQKYK-HEV	NT---EWTKK	TYNESMAQEK	SKGTRKKSYY	TVSYWEQIRL
(PRED)	sace_56_17_q011	EIQKYK-HEV	NT---EWTKK	TYNESMAQEK	SKGTRKKSYY	TVSYWEQIRL
(PRED)	sace_5_78_bz001	EIQKYK-HEV	NT---EWTKK	TYNESMAQEK	SKGTRKKSYY	TVSYWEQIRL
(PRED)	sace_2_8_h03860	EIQKYK-HEV	NT---EWTKK	TYNESMAQEK	SKGTRKKSYY	TVSYWEQIRL
(PRED)	sace_53_29_ac00	EIQKYK-HEV	NT---EWTKK	TYNESMAQEK	SKGTRKKSYY	TVSYWEQIRL
(PRED)	sace_17_7_g0393	EIQKYK-HEV	NT---EWTKK	TYNESMAQEK	SKGTRKKSYY	TVSYWEQIRL
(PRED)	sace_25_7_g0388	EIQKYK-HEV	NT---EWTKK	TYNESMAQEK	SKGTRKKSYY	TVSYWEQIRL
(PRED)	sace_37_7_g0385	EIQKYK-HEV	NT---EWTKK	TYNESMAQEK	SKGTRKKSYY	TVSYWEQIRL
(PRED)	sace_9_7_g00180	EIQKYK-HEV	NT---EWTKK	TYNESMAQEK	SKGTRKKSYY	TVSYWEQIRL
(PRED)	sace_60_6_f0335	EIQKYK-HEV	NT---EWTKK	TYNESMAQEK	SKGTRKKSYY	TVSYWEQIRL
(PRED)	sace_59_336_lx0	EIQKYK-HEV	NT---EWTKK	TYNESMAQEK	SKGTRKKSYY	TVSYWEQIRL
(PRED)	sace_31_7_3780	EIQKYK-HEV	NT---EWTKK	TYNESMAQEK	SKGTRKKSYY	TVSYWEQIRL
(PRED)	sace_34_8_3770	EIQKYK-HEV	NT---EWTKK	TYNESMAQEK	SKGTRKKSYY	TVSYWEQIRL
(PRED)	sace_58_71_bs00	EIQKYK-HEV	NT---EWTKK	TYNESMAQEK	SKGTRKKSYY	TVSYWEQIRL
(PRED)	sace_7_7_g03880	EIQKYK-HEV	NT---EWTKK	TYNESMAQEK	SKGTRKKSYY	TVSYWEQIRL
(PRED)	sace_35_7_3840	EIQKYK-HEV	NT---EWTKK	TYNESMAQEK	SKGTRKKSYY	TVSYWEQIRL
(PRED)	sace_43_7_g0387	EIQKYK-HEV	NT---EWTKK	TYNESMAQEK	SKGTRKKSYY	TVSYWEQIRL
(PRED)	sace_57_8_h0390	EIQKYK-HEV	NT---EWTKK	TYNESMAQEK	SKGTRKKSYY	TVSYWEQIRL
(PRED)	sace_45_7_g0389	EIQKYK-HEV	NT---EWTKK	TYNESMAQEK	SKGTRKKSYY	TVSYWEQIRL
(PRED)	sace_46_8_h0391	EIQKYK-HEV	NT---EWTKK	TYNESMAQEK	SKGTRKKSYY	TVSYWEQIRL
(PRED)	sace_23_7_3860	EIQKYK-HEV	NT---EWTKK	TYNESMAQEK	SKGTRKKSYY	TVSYWEQIRL
(PRED)	sace_21_7_3790	EIQKYK-HEV	NT---EWTKK	TYNESMAQEK	SKGTRKKSYY	TVSYWEQIRL
(PRED)	sace_8_73_bu001	EIQKYK-HEV	NT---EWTKK	TYNESMAQEK	SKGTRKKSYY	TVSYWEQIRL
(PRED)	sapa_1_8_h03820	EIQNYK-HDV	NP---ERTKK	TYNESMAQEK	SKGARKHSYY	TISYWEQIRL
(PRED)	sapa_21_8_h0387	EIQNYK-HDV	NP---ERTKK	TYNESMAQEK	SKGARKHSYY	TISYWEQIRL
(PRED)	sapa_20_8_h0386	EIQNYK-HDV	NP---ERTKK	TYNESMAQEK	SKGARKHSYY	TISYWEQIRL
(PRED)	sapa_22_8_h0390	EIQNYK-HDV	NP---ERTKK	TYNESMAQEK	SKGARKHSYY	TISYWEQIRL
(PRED)	sapa_25_8_h0387	EIQNYK-HDV	NP---ERTKK	TYNESMAQEK	SKGARKHSYY	TISYWEQIRL
(PRED)	sapa_6_8_3750	EIQNYK-HDV	NP---ERTKK	TYNESMAQEK	SKGARKHSYY	TISYWEQIRL
(PRED)	sapa_9_8_3720	EIQNYK-HDV	NP---ERTKK	TYNESMAQEK	SKGARKHSYY	TISYWEQIRL
(PRED)	sapa_19_8_h0390	EIQNYK-HDV	NP---ERTKK	TYNESMAQEK	SKGARKHSYY	TISYWEQIRL
(PRED)	sapa_24_8_h0385	EIQNYK-HDV	NP---ERTKK	TYNESMAQEK	SKGARKHSYY	TISYWEQIRL
(PRED)	sapa_4_8_h03850	EIQNYK-HDV	NP---ERTKK	TYNESMAQEK	SKGARKHSYY	TISYWEQIRL
(PRED)	sapa_10_8_3760	EIQNYK-HDV	NP---ERTKK	TYNESMAQEK	SKGARKHSYY	TISYWEQIRL
(PRED)	sapa_13_8_h0382	EIQNYK-HDV	YP---ERTKK	TYNESMAQEK	SKGARKHSYY	TISYWEQIRL
(PRED)	sapa_8_8_3750	EIQNYK-HDV	YP---ERTKK	TYNESMAQEK	SKGARKHSYY	TISYWEQIRL
(PRED)	sapa_11_8_h0383	EIQNYK-HDV	NP---ERTKK	TYNESMAQEK	SKGARKHSYY	TISYWEQIRL
(PRED)	sapa_5_8_3700	EIQNYK-HDV	NP---ERTKK	TYNESMAQEK	SKGARKHSYY	TISYWEQIRL
(PRED)	sapa_16_8_h0389	EIQKYK-HDV	NP---ERTKK	TYNESMAQEK	SKGARKHSYY	TISYWEQIRL
(PRED)	sapa_17_8_3730	EIQKYK-HDV	NP---ERTKK	TYNESMAQEK	SKGARKHSYY	TISYWEQIRL
(PRED)	sapa_2_8_h03860	EIQKYK-HDV	NP---ERTKK	TYNESMAQEK	SKGARKHSYY	TISYWEQIRL
(PRED)	sapa_7_8_3740	EIQKYK-HDV	NP---ERTKK	TYNESMAQEK	SKGARKHSYY	TISYWEQIRL
(PRED)	sapa_23_8_h0385	EIQKYK-HDV	NP---ERTKK	TYNESMAQEK	SKGARKHSYY	TISYWEQIRL
(PRED)	sapa_3_8_h03890	EIQKYK-HDV	NP---ERTKK	TYNESMAQEK	SKGARKHSYY	TISYWEQIRL
(PRED)	sapa_18_8_3730	EIQNYK-HDV	NP---ERTKK	TYNESMAQEK	SKGARKHSYY	TISYWEQIRL
(PRED)	sami_1_14_399	EIQKSK-HEV	NA---EKTKK	AYNESMAQEK	SKGARKYSYY	TISYWEQVRL
(PRED)	sace_4_8_h03690	EIQKYK-HEV	NT---EWTKK	TYNESMAQEK	SKGTRKKSYY	TVSYWEQIRL
(PRED)	saku_1_14_404	EIQEYK-EEV	DT---QRTKR	TYNESMAQEK	SKGARKSSYY	TISYWKQVRL
(PRED)	sace_1_ynr070w	EIQKYK-HEV	NT---EWTKK	TYNESMAQEK	SKGTRKKSYY	TVSYWEQIRL
(PRED)	sace_49_8_h0383	EIQKYK-HEV	NT---EWTKK	TYNESMAQEK	SKGTRKKSYY	TVSYWEQIRL
(PRED)	saeu_1_2_b00130	EIQEYK-QEV	DT---EKTKA	IYNVSMEQEK	SRGARKSSYY	TISYWEQVKL

```
(PRED) sauv_1_7_3       EIQEYK-QEI DT---EKTRT IYNVSMEQEK SKGARKSSYY TISYWEQVKL
(PRED) sami_1_17_26     EIQELK-QKV DT---EKVKA IYDKSMAQEK SKGTRKSSYF TISYWEQVKL
(PRED) zyba_1_02055_AN  DIEEYK-SAV NG---EATEE FFSETMRAEK SKYARNKSYY TVSFPEQVRL
(PRED) zyba_1_07912     DIEEYK-SAV NG---EATEQ FFSESMRAEK SKYARNKSYY TVSFPEQVRL
(PRED) zyba_2_2_b00600  DIEEYK-SAV NG---EATEQ FFSESMRAEK SKYARNKSYY TVSFPEQVRL
(PRED) zyba_3_3_c03460  DIEEYK-SAV NG---EATEQ FFSESMRAEK SKYARNKSYY TVSFPEQVRL
(PRED) zyba_1_04634     DIMAYK-EAV DG---KNTEN FFAETMHAEK CKYVRKKSYY TVSFFEQVRL
(PRED) zyba_1_06675     DISAYK-EAV NG---EATEK FFDETMHAEK SKYVRKRSYY TVSFFEQVRL
(PRED) zyba_3_2_b02230  DISAYK-EAV NG---EATEK FFDETMHAEK SKYVRKRSYY TVSFFEQVRL
(PRED) zyba_2_1_a00860  DISAYK-EAV NG---EATEK FFDETMHAEK SKYVRKRSYY TVSFFEQVRL
(PRED) zyro_1_a04114g   AIQEYK-ASV DP---EATKQ FFAETMKAEK SKYARDKSYY TVSFPEQIRL
(PRED) zyro_1_b14762g   EVQEYN-AKV NS---DATQQ FFSATMKAEK SKFSRNKSYY TISFPEQVRL
(PRED) zyba_2_14_n0149  EITEYL-SNS DA---SKTEQ FFFKSLQAEK SKYARKKSYY TISLPEQIRL
(PRED) zyba_2_33_ag001  EIAEYL-SNA DA---SKTEQ FFSKSLQAEK SKYARKKSYY TISLPEQIRL
(PRED) lath_1_a01914g   DIDNYK-RET EA---EKTKE LFSASLAQEK SKLNRKKSKY TVSFAEQVKL
(PRED) lawa_1_23_5161   DIEDYK-QET EA---EKTKA MYDKSLAQEK SKLNSKRSKY TLSFVEQVRL
(PRED) klae_1_14_n0012  EIEHYESNEV DA---HKTKE IFKTSMAQEK PPIARKNSKY TVPYFAQVYL
(PRED) klla_1_d03432g   SIEHYETKEV DG---AKTIQ FFKESMVEEK DKASRKKSKF TISYWAQIRL
(PRED) klma_1_1_a01880  SIEHYETNEV DG---AQTLQ LYKESMAAEK DKASRKRSKY TISYWAQIVL
(PRED) klwi_1_33_ag001  EIENYLNKEV DS---EQTKQ LFKTSMSQEK DKGSRTGSRY TISYWAQVSL
(PRED) teph_1_a04220    RIQNYE-GHV NP---EKAEQ IYDTSIMEEK PKYSQLSSHY MITYFEQVRI
(PRED) vapo_1_1037_47   DIETYK-TEV DP---EKTRE IYTMSLKQEK TKWTRKSSYY TVSFPEQVKL
(PRED) pata_1_2_b05590  DIQDKR-AKV SP---DETKE AFVKSKQQEK MKYQRSTSRY TINFFMQLKL
(PRED) wian_1_3_c04380  QINDYE-QKT DA---AKTRE IYDKSLSQEK ---PRAHYRY TINYFQQLRL
(PRED) wian_1_3_c04390  EINEYE-SQT DA---SQTRE TYDKSLAQEK ---PKSHYRY TISYLTQLKL
(PRED) wian_1_7_g01010  EIDQYE-SIT DA---GKTRE TYDKSLSQEK ---PKVHYRY TITYPSQLKL
(PRED) bain_1_1_a00100  DIAAHN-AKV NV---DETKR ALHAAQKQEK MKHNRAKSQF TLTYLAQLRL
(PRED) bain_1_17_q0038  DIAVHN-ANI NA---DEVKR VLHAAQKQEK MKHNRAKSQF TLTYLAQLRL
(PRED) bain_1_8_h00410  EIARSS-GKA DAGPSDKTLQ LYHDSFKQER SHIKRRKSRY SLTYLGQLRL
(PRED) caal_1_19_5759   EIKDYN-DEI DE---DETRS KYYQSIQQEK MKGSRTKSPF TISYLEQLKL
(PRED) caal_11_25_y002  EIKDYN-DEI DE---DETRS KYYQSIQQEK MKGSRTKSPF TISYLEQLKL
(PRED) caal_4_4_d03320  EIKDYN-DEI DE---DETRS KYYQSIQQEK MKGSRTKSPF TISYLEQLKL
(PRED) caal_12_26_z005  EIKDYN-DEI DE---DETRS KYYQSIQQEK MKGSRTKSPF TISYLEQLKL
(PRED) caal_5_30_ad005  EIKDYN-DEI DE---DETRS KYYQSIQQEK MKGSRTKSPF TISYLEQLKL
(PRED) caal_8_3_c03320  EIKDYN-DEI DE---DETRS KYYQSIQQEK MKGSRTKSPF TISYLEQLKL
(PRED) caal_6_4_d03280  EIKDYN-DEI DE---DETRS KYYQSIQQEK MKGSRTKSPF TISYLEQLKL
(PRED) caal_10_3_c0334  EIKDYN-DEI DE---DETRS KYYQSIQQEK MKGSRTKSPF TISYLEQLKL
(PRED) caal_3_29_ac005  EIKDYN-DEI DE---DETRS KYYQSIQQEK MKGSRTKSPF TISYLEQLKL
(PRED) caal_2_04989     EIKDYN-DEI DE---DETRS KYYQSIQQEK MKGSRTKSPF TISYLEQLKL
(PRED) cadu_1_64350     EIKDYN-DEI DE---DETRG KYYESIQQEK MKGARTKSPF TISYLEQLKL
(PRED) caor_1_h02090    EIQDYN-NSI DA---DETRQ MYYKSITQEK MKGARKKSPY TISYLHQLKL
(PRED) capa_1_600750    EINDYN-DSI DA---DETRQ MYYKSISQEK MKGARKKSPY TISYLQQLKL
(PRED) loel_1_04930     EIDEFN-NSI DV---DEVRK EYYHSVQQEK MKGARQSSPF TISYLQQLKL
(PRED) spar_1_5_e03260  NIAEYN-SET NE---DETRK NYYESLKQEK MKGSRLNSLY TVSYLQQLKL
(PRED) sppa_1_7_g03160  DIDEYN-AET NE---DETRK NYYESLKQEK SKGARLNSIY TVSFFEQLKL
(PRED) catr_1_01205     EIDIYN-NQL NH---DEIRN QYYESVKQEK MKGARNSSPF TVSYLQQLKL
(PRED) catr_1_05498     EIDEYN-SQI DE---DQVRR DYYDSVIQEK MKGARKKSPF TVSYMQQLKL
(PRED) catr_1_05971     EIDEYN-SQI DE---DQVRR DYYDSVKQEK MKGARKSSRF TISYLEQLKL
(PRED) deha_1_a03696g   EIQEYN-DSI NE---DETRK GYYHSLKQEK MKYSRTNSKF TINYLQQLKL
(PRED) deha_2_5_e00720  EIQEYN-DSI DE---DETRR GYYQSLKQEK MKYSRNNSKF TVNYFQQLKL
(PRED) scst_1_3_c02890  EIEDYN-NEV NS---DETQT KYYESINQEK MKYARPQSKF TISFVEQLRL
(PRED) mebi_1_8_h00300  DIKKYK-QQF ST---EETKA ALLESIKQEK QKYQRVGSKY TANIFQQLKY
(PRED) lakl_1_h21010g   EIHAYN-NRV DS---EGTRA LYRDSITQQK SKRSRGTSRY TISYLEQIKL
(PRED) caar_1_13_m0142  TIKQK-RAQY QP---SVALE TFRDAHRQEK QNFSRKTSIY TTNFFVQLKL
(PRED) caar_1_14_n0143  TIAEK-KAKY EP---EKTAE TFDIVHEQEK QKYTFPSSHY TVNYFEQLKL
(PRED) hapo_1_1_a07220  KITEK-KATY NP---AATYD NFHAVHALEK QKLTSSKSKY VVNYFEQLKL
(PRED) ogpa_1_1_a01680  KIAEK-KATY NP---AATYD NFRSVHTLEK QRLTGAKSKY VVNYFEQLKL
(PRED) piku_1_96_cr001  TVDEK-IKIA DS---KETEM VFNSVRETEK QRWTSSTSPY TINFPQQLKL
(PRED) pime_1_4_d03240  IIDEK-LKAS NP---EKTEE TFKEVLNVEK QKWTSKKSPY TVNFLQQLKL
(PRED) pime_1_1_a12110  AIEKK-IQNS NH---EETGG TFNNVLQVEK QKHTSKRSLY TVNYFEQLKL
(PRED) piku_1_227_hs00  EIGEK-SAQY SA---DESFQ AIENSNHILK EKGTSKHSFY TVNYLTQLKL
(PRED) pime_1_5_e05800  EIDQK-LEAH SS---KQTSD DIQSVRKLLK EKGTSKHSLY TLNYLSQLKI
(PRED) pime_1_1_a07690  QIDERLSEKN DG---DTEFH NLKQNHELIK QKYTSKKSQY TINYLSQLKE
(PRED) debr_2_5_e03380  ARQAALQARQ HP---AATTQ AFREAHAADR QKYARKRSLY LVNYPQQLAL
(PRED) kopa_1_2_b10040  QFQETLKAGL S----ETTKE TFLKAAANEK MKGVSDNSKY TVNYFEQLRL
(PRED) kopa_2_7_g00500  QFQETMKAGL S----ETTKE TFLKATANEK MKGVSNNSKY TVNYLEQLRL
(PRED) asru_1_13_m0119  EIAEYD-AEH NE---EATKQ LFELSTEQSK KKSKRKNSKY TITILEQFKL
(PRED) asru_1_15_o0045  EINEYE-QEC AL---SPTQQ LFDESTKQAK MKFQRHKSKY TINLLEQFKL
(PRED) wian_1_1_a02920  TITQYN-STV DA---DETKK SFKKSLSKEK SLFTRANSRY TIDFASQLKL
(PRED) wian_1_1_a02930  SIAQYK-STA SA---DETKR LFKESLAHEK AKYSKKNSHY TISYPAQLKL

                        .........760 .........770 .........780 .........790 .........800
(PRED) asac_1_6_f03560  CTIRGFQRIY GDKSFTVINV VSAVVQALIT GSLYFASPSS TSGAFSRGGV
(PRED) ergo_1_abr125c   CTIRGFQRIY GDKSFTVINV VSAVVQALIT GSLYFSSPSN TSGAFSRGGI
(PRED) ercy_1_3604      CTIRGFQRIY GDKSFTLINT AAAIIQALIT GSLYFQSPEG TSGAFSRGGV
```

(PRED)	cagl_1_i04862g	CTKRGFQRIY	GDKAYTVITI	CSAIIQSLVS	GSLYYNTPSS	TSGAFSRGGV
(PRED)	kaaf_1_c00830	CTIRGFQRIY	GNKSYTVINV	ASGIIQAFIS	GSLFYKTPSS	TDGAFSRGGV
(PRED)	kana_1_k01350	CTRRGFQRIY	GNRDYTVINI	VSAVIQSFVS	GSMYYNSPSS	TNGAFTRGGV
(PRED)	saar_1_2_b02590	CTQRAFQRIY	GNKSYTIINV	CSAIIQSFIT	GSLFYNSPSS	TSGAFSRGGV
(PRED)	sace_1_ydr011w	CTQRGFQRIY	GNKSYTVINV	CSAIIQSFIT	GSLFYNTPSS	TSGAFSRGGV
(PRED)	sace_16_1_a0238	CTQRGFQRIY	GNKSYTVINV	CSAIIQSFIT	GSLFYNTPSS	TSGAFSRGGV
(PRED)	sace_45_1_a0242	CTQRGFQRIY	GNKSYTVINV	CSAIIQSFIT	GSLFYNTPSS	TSGAFSRGGV
(PRED)	sace_48_1_a0238	CTQRGFQRIY	GNKSYTVINV	CSAIIQSFIT	GSLFYNTPSS	TSGAFSRGGV
(PRED)	sace_60_4_d0244	CTQRGFQRIY	GNKSYTVINV	CSAIIQSFIT	GSLFYNTPSS	TSGAFSRGGV
(PRED)	sace_52_1_a0240	CTQRGFQRIY	GNKSYTVINV	CSAIIQSFIT	GSLFYNTPSS	TSGAFSRGGV
(PRED)	sace_46_1_a0240	CTQRGFQRIY	GNKSYTVINV	CSAIIQSFIT	GSLFYNTPSS	TSGAFSRGGV
(PRED)	sace_25_1_a0240	CTQRGFQRIY	GNKSYTVINV	CSAIIQSFIT	GSLFYNTPSS	TSGAFSRGGV
(PRED)	sace_24_1_2300	CTQRGFQRIY	GNKSYTVINV	CSAIIQSFIT	GSLFYNTPSS	TSGAFSRGGV
(PRED)	sace_47_1_a0240	CTQRGFQRIY	GNKSYTVINV	CSAIIQSFIT	GSLFYNTPSS	TSGAFSRGGV
(PRED)	sace_7_1_a02410	CTQRGFQRIY	GNKSYTVINV	CSAIIQSFIT	GSLFYNTPSS	TSGAFSRGGV
(PRED)	sace_59_110_df0	CTQRGFQRIY	GNKSYTVINV	CSAIIQSFIT	GSLFYNTPSS	TSGAFSRGGV
(PRED)	sace_56_1_a0202	CTQRGFQRIY	GNKSYTVINV	CSAIIQSFIT	GSLFYNTPSS	TSGAFSRGGV
(PRED)	sace_40_1_a0239	CTQRGFQRIY	GNKSYTVINV	CSAIIQSFIT	GSLFYNTPSS	TSGAFSRGGV
(PRED)	sace_15_1_a0242	CTQRGFQRIY	GNKSYTVINV	CSAIIQSFIT	GSLFYNTPSS	TSGAFSRGGV
(PRED)	sace_37_1_a0243	CTQRGFQRIY	GNKSYTVINV	CSAIIQSFIT	GSLFYNTPSS	TSGAFSRGGV
(PRED)	sace_9_1_a02440	CTQRGFQRIY	GNKSYTVINV	CSAIIQSFIT	GSLFYNTPSS	TSGAFSRGGV
(PRED)	sace_22_1_2300	CTQRGFQRIY	GNKSYTVINV	CSAIIQSFIT	GSLFYNTPSS	TSGAFSRGGV
(PRED)	sace_29_1_2290	CTQRGFQRIY	GNKSYTVINV	CSAIIQSFIT	GSLFYNTPSS	TSGAFSRGGV
(PRED)	sace_34_1_2320	CTQRGFQRIY	GNKSYTVINV	CSAIIQSFIT	GSLFYNTPSS	TSGAFSRGGV
(PRED)	sace_58_25_y007	CTQRGFQRIY	GNKSYTVINV	CSAIIQSFIT	GSLFYNTPSS	TSGAFSRGGV
(PRED)	sace_23_1_2290	CTQRGFQRIY	GNKSYTVINV	CSAIIQSFIT	GSLFYNTPSS	TSGAFSRGGV
(PRED)	sace_6_120_dp00	CTQRGFQRIY	GNKSYTVINV	CSAIIQSFIT	GSLFYNTPSS	TSGAFSRGGV
(PRED)	sace_57_1_a0241	CTQRGFQRIY	GNKSYTVINV	CSAIIQSFIT	GSLFYNTPSS	TSGAFSRGGV
(PRED)	sace_17_1_a0241	CTQRGFQRIY	GNKSYTVINV	CSAIIQSFIT	GSLFYNTPSS	TSGAFSRGGV
(PRED)	sace_21_1_2310	CTQRGFQRIY	GNKSYTVINV	CSAIIQSFIT	GSLFYNTPSS	TSGAFSRGGV
(PRED)	sace_49_1_a0246	CTQRGFQRIY	GNKSYTVINV	CSAIIQSFIX	GSLFYNTPSS	TXGAFSRGGV
(PRED)	sace_8_2_b02430	CTQRGFQRIY	GNKSYTVINV	CSAIIQSFIX	GSLFYNTPSS	TXGAFSRGGV
(PRED)	sace_31_1_2300	CTQRGFQRIY	GNKSYTVINV	CSAIIQSFIS	GSLFYNTPSS	TSGAFSRGGV
(PRED)	sace_50_1_a0241	CTQRGFQRIY	GNKSYTVINV	CSAIIQSFIT	GSLFYNTPSS	TSGAFSRGGV
(PRED)	sace_4_1_a02360	CTQRGFQRIY	GNKSYTVINV	CSAIIQSFIT	GSLFYNTPSS	TSGAFSRGGV
(PRED)	sace_2_1_a02390	CTQRGFQRIY	GNKSYTVINV	CSAIIQSFIT	GSLFYNTPSS	TSGAFSRGGV
(PRED)	sace_5_33_ag005	CTQRGFQRIY	GNKSYTVINV	CSAIIQSFIT	GSLFYNTPSS	TSGAFSRGGV
(PRED)	sapa_11_1_a0247	CTQRGIQRIY	GNKSYTVINV	CSAIIQSFIT	GSLFYNTPSS	TSGAFSRGGV
(PRED)	sapa_25_1_a0246	CTQRGIQRIY	GNKSYTVINV	CSAIIQSFIT	GSLFYNTPSS	TSGAFSRGGV
(PRED)	sapa_4_1_a02470	CTQRGIQRIY	GNKSYTVINV	CSAIIQSFIT	GSLFYNTPSS	TSGAFSRGGV
(PRED)	sapa_5_1_2350	CTQRGIQRIY	GNKSYTVINV	CSAIIQSFIT	GSLFYNTPSS	TSGAFSRGGV
(PRED)	sapa_9_1_2360	CTQRGIQRIY	GNKSYTVINV	CSAIIQSFIT	GSLFYNTPSS	TSGAFSRGGV
(PRED)	sapa_14_1_a0244	CTQRGIQRIY	GNKSYTVINV	CSAIIQSFIT	GSLFYNTPSS	TSGAFSRGGV
(PRED)	sapa_8_1_2350	CTQRGIQRIY	GNKSYTVINV	CSAIIQSFIT	GSLFYNTPSS	TSGAFSRGGV
(PRED)	sapa_17_1_2380	CTQRGVQRIY	GNKSYTVINV	CSAIIQSFIT	GSLFYNTPSS	TSGAFSRGGV
(PRED)	sapa_7_1_2370	CTQRGVQRIY	GNKSYTVINV	CSAIIQSFIT	GSLFYNTPSS	TSGAFSRGGV
(PRED)	sapa_2_1_a02460	CTLRGVQRIY	GNKSYTVINV	CSAIIQSFIT	GSLFYNTPSS	TSGAFSRGGV
(PRED)	sapa_23_1_a0248	CTLRGVQRIY	GNKSYTVINV	CSAIIQSFIT	GSLFYNTPSS	TSGAFSRGGV
(PRED)	sapa_3_1_a02470	CTLRGVQRIY	GNKSYTVINV	CSAIIQSFIT	GSLFYNTPSS	TSGAFSRGGV
(PRED)	sapa_18_1_2390	CTQRGVQRIY	GNKSYTVINV	CSAIIQSFIT	GSLFYNTPSS	TSGAFSRGGV
(PRED)	sami_1_4_244	CTQRGFQRIY	GNKSYTVINV	CSAIIQSFIT	GSLFYNTPSS	TSGAFSRGGV
(PRED)	saku_1_4_262	CTQRGFQRIY	GNKSYTVINV	CAAIIQSFIT	GSLFYNTPSS	TSGAFSRGGV
(PRED)	saba_1_58_bf002	CTQRGFQRIY	GNKSYTVINV	CSAIIQSFIT	GSLFYNTPSS	TSGAFSRGGV
(PRED)	saeu_1_4_d02400	CTQRGFQRIY	GNKSYTIINV	CSAIIQSFIT	GSLYYNTPSS	TSGAFSRGGV
(PRED)	naca_1_e01640	CTIRGFQRIY	GNKAYTVINV	SSSVIQAFIV	GSNFYASPSS	TNGAFSRGGV
(PRED)	nada_1_g01850	CTKRGFQRIY	GNKSYTVINT	CAAIIQSFIV	GSLFYNTPST	TNGAFSRGGV
(PRED)	naca_1_e01630	CTIRGTQRIY	GNKTYTVINI	CSAIIQAFIT	GSLFYNIPSD	TNGAFSRGGV
(PRED)	nada_1_g01840	CTIRGFQRVY	GNKSYTVINI	CAAVIQAFII	GSLFYNSPST	AQGAFSRGGI
(PRED)	kaaf_1_c00820	CTIRGLQRIY	GNKSYTVINI	CSAIIQAFIS	GSLFYNSPST	TNGAFSRGGV
(PRED)	teph_1_m00640	CVKRGFQRIY	GDKTSTVINT	VAAIVQAFVT	GSLFYKTPSA	TNGAFSRGGV
(PRED)	vapo_1_1036_28	CVQRGFQRIY	GDKTSTIINT	VAAIVQAFVT	GSLYYNSPST	TNGAFSRGGV
(PRED)	tebl_1_i01760	CTIRGFQRIY	GNKSFTVTNI	TAAIIQSLVS	GSLFYKAPSG	TNGAFTRGGV
(PRED)	tode_1_d04040	CSQRGFQRIY	GNKSYTIINV	IAAIVQSFIV	GSLCYNAPSS	TSGAFTRGGV
(PRED)	naca_1_e01650	CTKRGFQRIY	GNKAYTVINV	SSSVIQAFIA	GSLFYKSPSD	TSGSFSRSGV
(PRED)	tebl_1_g02820	CTIRGFQRIY	GNKSYTVTNC	VAALIQSFVT	GSLYYNSPST	TNGAFSRSGV
(PRED)	lakl_1_c11616g	CTIRGFQRIY	GDKAFTITNT	VAAIVQSLVT	GSLFYSTPSS	TSGAFSRSGV
(PRED)	saar_1_8_h03780	CTVRGFQRIY	GDKSYTVINT	CAAIAQAFIT	GSLFYQTPSS	TLGAFSRSGV
(PRED)	sace_14_7_g0015	CTIRGFLRIY	GDKSYTVINT	CAAIAQAFIT	GSLFYQAPSS	TLGAFSRSGV
(PRED)	sace_15_7_g0387	CTIRGFLRIY	GDKSYTVINT	CAAIAQAFIT	GSLFYQAPSS	TLGAFSRSGV
(PRED)	sace_24_8_3780	CTIRGFLRIY	GDKSYTVINT	CAAIAQAFIT	GSLFYQAPSS	TLGAFSRSGV
(PRED)	sace_40_8_h0383	CTIRGFLRIY	GDKSYTVINT	CAVIAQAFIT	GSLFYQAPSS	TLGAFSRSGV
(PRED)	sace_6_169_fm00	CTIRGFLRIY	GDKSYTVINT	CAAIAQAFIT	GSLFYQAPSS	TLGAFSRSGV
(PRED)	sace_19_7_3840	CTIRGFLRIY	GDKSYTVINT	CAAIAQAFIT	GSLFYQAPSS	TLGAFSRSGV
(PRED)	sace_32_7_3770	CTIRGFLRIY	GDKSYTVINT	CAAIAQAFIT	GSLFYQAPSS	TLGAFSRSGV
(PRED)	sace_56_17_q011	CTIRGFLRIY	GDKSYTVINT	CAAIAHAFIT	GSLFYQAPSS	TLGAFSRSGV

(PRED)	sace_5_78_bz001	CTIRGFLRIY	GDKSYTVINT	CAAIAQAFIT	GSLFYQAPSS	TLGAFSRSGV
(PRED)	sace_2_8_h03860	CTIRGFLRIY	GDKSYTVINT	CAAIAQAFIT	GSLFYQAPSS	TLGAFSRSGV
(PRED)	sace_53_29_ac00	CTIRGFLRIY	GDKSYTVINT	CAAIAQAFIT	GSLFYQAPSS	TLGAFSRSGV
(PRED)	sace_17_7_g0393	CTIRGFLRIY	GDKSYTVINT	CAAIAQAFIT	GSLFYQAPSS	TLGAFSRSGV
(PRED)	sace_25_7_g0388	CTIRGFLRIY	GDKSYTVINT	CAAIAQAFIT	GSLFYQAPSS	TLGAFSRSGV
(PRED)	sace_37_7_g0385	CTIRGFLRIY	GDKSYTVINT	CAAIAQAFIT	GSLFYQAPSS	TLGAFSRSGV
(PRED)	sace_9_7_g00180	CTIRGFLRIY	GDKSYTVINT	CAAIAQAFIT	GSLFYQAPSS	TLGAFSRSGV
(PRED)	sace_60_6_f0335	CTIRGFLRIY	GDKSYTVINT	CAAIAQAFIT	GSLFYQAPSS	TLGAFSRSGV
(PRED)	sace_59_336_lx0	CTIRGFLRIY	GDKSYTVINT	CAAIAQAFIT	GSLFYQAPSS	TLGAFSRSGV
(PRED)	sace_31_7_3780	CTIRGFLRIY	GDKSYTVINT	CAAIAQAFIT	GSLFYQAPSS	TLGAFSRSGV
(PRED)	sace_34_8_3770	CTIRGFLRIY	GDKSYTVINT	CAAIAQAFIT	GSLFYQAPSS	TLGAFSRSGV
(PRED)	sace_58_71_bs00	CTIRGFLRIY	GDKSYTVINT	CAAIAQAFIT	GSLFYQAPSS	TLGAFSRSGV
(PRED)	sace_7_7_g03880	CTIRGFLRIY	GDKSYTVINT	CAAIAQAFIT	GSLFYQAPSS	TLGAFSRSGV
(PRED)	sace_35_7_3840	CTIRGFLRIY	GDKSYTVINT	CAAIAQAFIT	GSLFYQAPSS	TLGAFSRSGV
(PRED)	sace_43_7_g0387	CTIRGFLRIY	GDKSYTVINT	CAAIAQAFIT	GSLFYQAPSS	TLGAFSRSGV
(PRED)	sace_57_8_h0390	CTIRGFLRIY	GDKSYTVINT	CAAIAQAFIT	GSLFYQAPSS	TLGAFSRSGV
(PRED)	sace_45_7_g0389	CTIRGFLRIY	GDKSYTVINT	CAAIAQAFIT	GSLFYQAPSS	TLGAFSRSGV
(PRED)	sace_46_8_h0391	CTIRGFLRIY	GDKSYTVINT	CAAIAQAFIT	GSLFYQAPSS	TLGAFSRSGV
(PRED)	sace_23_7_3860	CTIRGFLRIY	GDKSYTVINT	CAAIAQAFIT	GSLFYQAPSS	TLGAFSRSGV
(PRED)	sace_21_7_3790	CTIRGFLRIY	GDKSYTVINT	CAAIAQAFIT	GSLFYQAPSS	TLGAFSRSGV
(PRED)	sace_8_73_bu001	CTIRGFLRIY	GDKSYTVINT	CAAIAXAFIT	GSLFYQAPSS	TLGAFSRSGV
(PRED)	sapa_1_8_h03820	CTIRGFQRIY	GDRSYTVINT	CAAIAQAFIT	GSLFYQAPSS	TLGAFSRSGV
(PRED)	sapa_21_8_h0387	CTIRGFQRIY	GDRSYTVINT	CAAIAQAFIT	GSLFYQAPSS	TLGAFSRSGV
(PRED)	sapa_20_8_h0386	CTIRGFQRIY	GDRSYTVINT	CAAIAQAFIT	GSLFYQAPSS	TLGAFSRSGV
(PRED)	sapa_22_8_h0390	CTIRGFQRIY	GDRSYTVINT	CAAIAQAFIT	GSLFYQAPSS	TLGAFSRSGV
(PRED)	sapa_25_8_h0387	CTIRGFQRIY	GDRSYTVINT	CAAIAQAFIT	GSLFYQAPSS	TLGAFSRSGV
(PRED)	sapa_6_8_3750	CTIRGFQRIY	GDRSYTVINT	CAAIAQAFIT	GSLFYQAPSS	TLGAFSRSGV
(PRED)	sapa_9_8_3720	CTIRGFQRIY	GDRSYTVINT	CAAIAQAFIT	GSLFYQAPSS	TLGAFSRSGV
(PRED)	sapa_19_8_h0390	CTIRGFQRIY	GDRSYTVINT	CAAIAQAFIT	GSLFYQAPSS	TLGAFSRSGV
(PRED)	sapa_24_8_h0385	CTIRGFQRIY	GDRSYTVINT	CAAIAQAFIT	GSLFYQAPSS	TLGAFSRSGV
(PRED)	sapa_4_8_h03850	CTIRGFQRIY	GDRSYTVINT	CAAIAQAFIT	GSLFYQAPSS	TLGAFSRSGV
(PRED)	sapa_10_8_3760	CTIRGFQRIY	GDRSYTVINT	CAAIAQAFIT	GSLFYQAPSS	TLGAFSRSGV
(PRED)	sapa_13_8_h0382	CTIRGFQRIY	GDRSYTVINT	CAAIAQAFIT	GSLFYQAPSS	TLGAFSRSGV
(PRED)	sapa_8_8_3750	CTIRGFQRIY	GDRSYTVINT	CAAIAQAFIT	GSLFYQAPSS	TLGAFSRSGV
(PRED)	sapa_11_8_h0383	CTIRGFQRIY	GDRSYTVINT	CAAIAQAFIT	GSLFYQAPSS	TLGAFSRSGV
(PRED)	sapa_5_8_3700	CTIRGFQRIY	GDRSYTVINT	CAAIAQAFIT	GSLFYQAPSS	TLGAFSRSGV
(PRED)	sapa_16_8_h0389	CTVRGFQRIY	GDRSYTVINT	CAAIAQAFIT	GSLFYQAPSS	TLGAFSRSGV
(PRED)	sapa_17_8_3730	CTVRGFQRIY	GDRSYTVINT	CAAIAQAFIT	GSLFYQAPSS	TLGAFSRSGV
(PRED)	sapa_2_8_h03860	CTVRGFQRIY	GDRSYTVINT	CAAIAQAFIT	GSLFYQAPSS	TLGAFSRSGV
(PRED)	sapa_7_8_3740	CTVRGFQRIY	GDRSYTVINT	CAAIAQAFIT	GSLFYQAPSS	TLGAFSRSGV
(PRED)	sapa_23_8_h0385	CTVRGFQRIY	GDRSYTVINT	CAAIAQAFIT	GSLFYQAPSS	TLGAFSRSGV
(PRED)	sapa_3_8_h03890	CTVRGFQRIY	GDRSYTVINT	CAAIAQAFIT	GSLFYQAPSS	TLGAFSRSGV
(PRED)	sapa_18_8_3730	CTIRGFQRIY	GDRSYTVINT	CAAIAQAFVT	GSLFYQAPSS	TLGAFSRSGV
(PRED)	sami_1_14_399	CTIRGFQRIY	GDRSYTVINT	CAAIAQAFIT	GSLFYQSPPS	TLGAFSRSGV
(PRED)	sace_4_8_h03690	CTIRGFLRIY	GDKSYTVINT	CAAIAQAFIT	GSLFYQAPSS	TLGAFSRSGV
(PRED)	saku_1_14_404	CTIRGFQRIY	GDKSYTVINT	CAAIAQSFIT	GSLFYQTPSS	TLGAFSRSGV
(PRED)	sace_1_ynr070w	CTIRGFLRIY	GDKSYTVINT	CAAIAQAFIT	GSLFYQAPSS	TLGAFSRSGV
(PRED)	sace_49_8_h0383	CTIRGFLRIY	GDKSYTVINT	CAAIAQAFIT	GSLFYQAPSS	TLGAFSRSGV
(PRED)	saeu_1_2_b00130	CTVRGFQRIY	GNKSYTVINT	CAAIAQSFIT	GSLFYQSSPS	TLGAFSRSGV
(PRED)	sauv_1_7_3	CTARGFQRIY	GNKSYTVINT	CAAIVQSFIT	GSLFYQSSSS	TLGAFSRSGV
(PRED)	sami_1_17_26	CTIRGFQRIY	GNKSYTVINT	CAAIAQSFII	GSLFYQTSSS	TSGAFSRGGV
(PRED)	zyba_1_02055_AN	CTIRGFQRVY	GNRSYTVINV	AAAVVQAFIT	GSAFYNSPSS	TNGAFSRGGV
(PRED)	zyba_1_07912	CTIRGFQRVY	GNRSYTVINV	AAAVVQAFIT	GSAFYNSPSS	TNGAFSRGGV
(PRED)	zyba_2_2_b00600	CTIRGFQRVY	GNRSYTVINV	AAAVVQAFIT	GSAFYNSPSS	TNGAFSRGGV
(PRED)	zyba_3_3_c03460	CTIRGFQRVY	GNRSYTVINV	AAAVVQAFIT	GSAFYNSPSS	TNGAFSRGGV
(PRED)	zyba_1_04634	CTIRGLQRIY	GNKAYTIINI	VAAVVQAFIA	GSAYYNSPSS	TNGAFSRGGV
(PRED)	zyba_1_06675	CTIRGLQRIY	GNKAYTIINI	VAAVVQAFIA	GSAYYNSPSS	TNGAFSRGGV
(PRED)	zyba_3_2_b02230	CTIRGLQRIY	GNKAYTIINI	VAAVVQAFIA	GSAYYNSPSS	TNGAFSRGGV
(PRED)	zyba_2_1_a00860	CTIRGLQRIY	GNKAYTIINI	VAAVVQAFIA	GSAYYNSPSS	TNGAFSRGGI
(PRED)	zyro_1_a04114g	CTVRGFQNVY	GNKAYTVINI	AAAVIQAFIT	GSAFYNSPAS	TAGAFSRGGV
(PRED)	zyro_1_b14762g	CTVRGLQRIY	GNKAYTVINV	VAAVIQAFIT	GSAFYNSPSS	TAGAFSRGGV
(PRED)	zyba_2_14_n0149	CTIRGFQRVY	GNMAYTIINT	AAAVIQAFVT	GSAFYNSPSS	TDGAFSRGGV
(PRED)	zyba_2_33_ag001	CTIRGFQRVY	GNMAYTIINT	AAAVIQAFVT	GSAFYNSPSS	TDGAFSRGGV
(PRED)	lath_1_a01914g	CTKRGVQRIY	GDKAYTITNL	IASIIQSLVS	GSLYYNLPSG	VSGAFSRGGV
(PRED)	lawa_1_23_5161	CTIRGFQRIY	GDKAYTITNT	IAATIQGLVS	GSLYYNLPSG	VSGAFSRGGV
(PRED)	klae_1_14_n0012	CTIRGFQRIY	GDKAFTITNT	IASIVQALIQ	GSLFYNTSSG	TSGAFSRGGV
(PRED)	klla_1_d03432g	CTRRGFQRIY	GDKSFTITNT	VAAIIQALVT	GSLFYNTPSS	TQGAFSRGGV
(PRED)	klma_1_1_a01880	CTKRGFQRIY	GDKAFTITNT	VAAIIQAFIT	GSLYYNTPSG	TSGAFSRGGI
(PRED)	klwi_1_33_ag001	CTKRGFQRIY	GDKAFTITNV	IASIIQSLVS	GSLYWNTPSG	TDGAFSRGGI
(PRED)	teph_1_a04220	CTIRGFQRIY	GDMNYTVINI	VAAVIQAFII	GSLFYNTPVS	TTGAFSRGGI
(PRED)	vapo_1_1037_47	CTIRGFQRIY	GDKSFSIIST	VAAVFQSFII	GSLFYNTPSS	TSGAFSRGGT
(PRED)	pata_1_2_b05590	CTIRGFQKII	NDRSYTVTTV	AAAIIQSLIV	GSLYWKTPSS	TEGAFGRGGC
(PRED)	wian_1_3_c04380	LTKRGFQRIY	GDKAYTITQT	VAATIQALIC	GSLYYNTPSS	TKGSFSRSGT
(PRED)	wian_1_3_c04390	LTQRGFDRIY	GDKAYTITQL	IAAVIQALIA	GSLYYGTPAT	TRGSFSRSGS
(PRED)	wian_1_7_g01010	LTKRGFQRIY	GDKAYTVTQV	SAATIQALIT	GSLYWNTLPT	TQGAFSRSGT

```
(PRED) bain_1_1_a00100 LTIRGFQRNW  GDKAYTITQV  AAAIIQSLIT  GSLYWKTPEG  TSGAFSRGGV
(PRED) bain_1_17_q0038 LTVRGFQRNW  GDKAYTITQV  AAAIIQSLIT  GSLYWKTPEG  TSGAFSRSGV
(PRED) bain_1_8_h00410 LIVRGLQRAY  MDRAYTITQV  ASGVIQGLIT  GSLYWGTPNT  VFGAFSKGGA
(PRED) caal_1_19_5759  CFIRSYQRIL  GDSAYTITLM  FASVAQAFVA  GSLYYNTPDD  VSGAFSRGGV
(PRED) caal_11_25_y002 CFIRSYQRIL  GDSAYTITLM  FASVAQAFVA  GSLYYNTPDD  VSGAFSRGGV
(PRED) caal_4_4_d03320 CFIRSYQRIL  GDSAYTITLM  FASVAQAFVA  GSLYYNTPDD  VSGAFSRGGV
(PRED) caal_12_26_z005 CFIRSYQRIL  GDSAYTITLM  FASVAQAFVA  GSLYYNTPDD  VSGAFSRGGV
(PRED) caal_5_30_ad005 CFIRSYQRIL  GDSAYTITLM  FASVAQAFVA  GSLYYNTPDD  VSGAFSRGGV
(PRED) caal_8_3_c03320 CFIRSYQRIL  GDSAYTITLM  FASVAQAFVA  GSLYYNTPDD  VSGAFSRGGV
(PRED) caal_6_4_d03280 CFIRSYQRIL  GDSAYTITLM  FASVAQAFVA  GSLYYNTPDD  VSGAFSRGGV
(PRED) caal_10_3_c0334 CFIRSYQRIL  GDSAYTITLM  FASVAQAFVA  GSLYYNTPDD  VSGAFSRGGV
(PRED) caal_3_29_ac005 CFIRSYQRIL  GDSAYTITLM  FASVAQAFVA  GSLYYNTPDD  VSGAFSRGGV
(PRED) caal_2_04989    CFIRSYQRIL  GDSAYTITLM  FASVAQAFVA  GSLYYNTPDD  VSGAFSRGGV
(PRED) cadu_1_64350    CFIRSYQRIL  GDSAYTLTLM  FASVAQAFVA  GSLYYNTPDD  VSGAFSRGGV
(PRED) caor_1_h02090   CSIRSAQQIW  GDKAYTVTLI  GAGVSQAFVS  GSLYYNTPET  VLGAFSRGGV
(PRED) capa_1_600750   CSIRSSQQIW  GDKAYTVTLI  GAGVCQAFIN  GSLYYNTPES  VIGAFSRGGV
(PRED) loel_1_04930    CAKRSVQRIW  GDKAYTVTLM  GAGVSQAFVA  GSLYYNTPDD  VSGAFSRGGV
(PRED) spar_1_5_e03260 CTIRAFERTW  GDKAYTITLI  LATTVQAFIV  GSLYYNTPDD  VSGAFSRGGV
(PRED) sppa_1_7_g03160 CTMRTFDRTW  GDKAYTITLI  LAAVAQAFII  GSLYYNTPDD  VSGAFSRGGV
(PRED) catr_1_01205    CLIRSYQRIK  GDKAYTITLV  TAAIAQAFVA  GSLYYNTPED  VSGAFSRGGV
(PRED) catr_1_05498    CFIRSFYRIK  GDNAYTITLV  GAAVCQAFIA  GSLYYNTPND  VSGAFSRGGV
(PRED) catr_1_05971    CFIRSFQRIM  GDKAYTITLV  GAAVSQAFVA  GSLYYNTPEN  VAGAFSRGGV
(PRED) deha_1_a03696g  CTTRGFQRLW  GDKAYTITQL  VAAISQGLIA  GSLYYNTPDS  VSGAFSRGGV
(PRED) deha_2_5_e00720 CTTRGFQRLW  GDKAYTITQL  VAAISQGLIA  GSLYYNTPDS  VSGAFSRGGI
(PRED) scst_1_3_c02890 TTLRGFQRIW  GDKAYTVTLI  GAGVSQGLVA  GSLYYNTPET  VSGAFSRGGV
(PRED) mebi_1_8_h00300 NLKRSFQNIV  GDSAFTVIQV  AAAISQALII  GSLYYNTPET  VNGAFSRGGV
(PRED) lakl_1_h21010g  CTKRGFQRIS  GDSTSTVINI  AAATIQSLII  GSLYYNLNPS  TDSAFSRSGV
(PRED) caar_1_13_m0142 CCLRGVNNIV  NNKSYTVTLI  LAALIQSLIV  GSLYYNITEG  SIGSFSRGGV
(PRED) caar_1_14_n0143 CCTRGAQNIV  NNKAYTITLV  MASVFQSLIV  GSLYYNTTNE  TLGSFSRGGI
(PRED) hapo_1_1_a07220 CTMRGFHNIV  NNSAYTATLI  IAATIQALIV  GSLYYNTPSS  TIGSFPRGGV
(PRED) ogpa_1_1_a01680 CTMRGFHNIA  NNSAYTATLM  VAATIQALIV  GSLYYNTPSS  TIGSFPRGGV
(PRED) piku_1_96_cr001 CCQRRFQNIV  NNKAYTLTLM  FAATFQSLII  GSLFYNTPQT  TIGAFSRGGV
(PRED) pime_1_4_d03240 CCKRRVHNLI  NNKSYTITLV  AAAVIQSLII  GSLYYNTTRE  TIGSFSRGGI
(PRED) pime_1_1_a12110 CCKRRAQDSW  NNRAYAMTLF  FAASIQAFVI  GSMYYNITKE  TVGAFARGGV
(PRED) piku_1_227_hs00 CCIRRFQSTI  NDKAYTVTFI  SAAVIQSLII  GSLGYNSPNS  TLGAFTKGGV
(PRED) pime_1_5_e05800 CCQRRSQSIL  NNRAYTITFI  SASVVQSLII  GSLGYNTPTS  TLGAFSRSGI
(PRED) pime_1_1_a07690 CCNRRFHIAI  NNRAYPVTLI  FAAIMQSLVT  GSLMYNITSE  TVGAFSKGGV
(PRED) debr_2_5_e03380 CLWRAACTIA  NNTAYTVTNI  AVAVVQALIT  GSLYFNTPQT  TSGAFSRSGI
(PRED) kopa_1_2_b10040 CIVRGFQRIK  GDINYTIVMV  VSALIQGLVV  GSLYWNTPEN  SSGVFGRAGV
(PRED) kopa_2_7_g00500 CILRGFQRIK  GDIGYTIVMV  VSALVQGLVV  GSLYWNTPEN  SSGVFGRAGV
(PRED) asru_1_13_m0119 CTMRGFQRVI  GDSAYTITQV  VANVIQSLIV  GSLFYNLQNS  TISAYSKGGT
(PRED) asru_1_15_o0045 LTIRGFQRVL  GDPYYTVIQI  FSMTVESLVI  GSMFYNLPNN  TDSFFSRGGL
(PRED) wian_1_1_a02920 LVNRGYQRVI  NDTAFLIIQS  ALTVIIAFMI  GSLCYDTPIS  TAGAFSRGGF
(PRED) wian_1_1_a02930 LTKRGYQRII  GDKAFTTIQI  SMSIIISLVL  GSMFYDSPDS  TAGAFSRGGV

                       .........810 .........820 .........830 .........840 .........850
(PRED) asac_1_6_f03560 LYYSILYFSL  MGLANINFSN  --RPILQKHK  MYTLYHPSAE  ALASTISALA
(PRED) ergo_1_abr125c  LYYAILYFSL  MGLANINFSN  --RPILQKHK  MYTLYHPSAE  ALASTISACV
(PRED) ercy_1_3604     LYFAVLYYSL  MGLANINFSN  --RPILQKHK  LYTLYHPSAE  ALASTFSAFV
(PRED) cagl_1_i04862g  LYFCLLYYSL  MGLANLSFEH  --RPILQKHK  IYSLYHPAAE  ALGSTIANFP
(PRED) kaaf_1_c00830   IYFALLYYSL  MGLANITFDH  --RPILQKHK  GYSLYHPSAE  ALASTLSAFP
(PRED) kana_1_k01350   LYFCLLYYSL  MGLANISFDH  --RPILQKHK  GYSLYHPSAE  ALASTISGFP
(PRED) saar_1_2_b02590 LYFAILYFSL  MGLANISFEH  --RPILQKHK  GYSLYHPSAE  AIGSTLAAFP
(PRED) sace_1_ydr011w  LYFALLYYSL  MGLANISFEH  --RPILQKHK  GYSLYHPSAE  AIGSTLASFP
(PRED) sace_16_1_a0238 LYFALLYYSL  MGLANISFEH  --RPILQKHK  GYSLYHPSAE  AIGSTLASFP
(PRED) sace_45_1_a0242 LYFALLYYSL  MGLANISFEH  --RPILQKHK  GYSLYHPSAE  AIGSTLASFP
(PRED) sace_48_1_a0238 LYFALLYYSL  MGLANISFEH  --RPILQKHK  GYSLYHPSAE  AIGSTLASFP
(PRED) sace_60_4_d0244 LYFALLYYSL  MGLANISFEH  --RPILQKHK  GYSLYHPSAE  AIGSTLASFP
(PRED) sace_52_1_a0240 LYFALLYYSL  MGLANISFEH  --RPILQKHK  GYSLYHPSAE  AIGSTLASFP
(PRED) sace_46_1_a0240 LYFALLYYSL  MGLANISFEH  --RPILQKHK  GYSLYHPSAE  AIGSTLASFP
(PRED) sace_25_1_a0240 LYFALLYYSL  MGLANISFEH  --RPILQKHK  GYSLYHPSAE  AIGSTLASFP
(PRED) sace_24_1_2300  LYFALLYYSL  MGLANISFEH  --RPILQKHK  GYSLYHPSAE  AIGSTLASFP
(PRED) sace_47_1_a0240 LYFALLYYSL  MGLANISFEH  --RPILQKHK  GYSLYHPSAE  AIGSTLASFP
(PRED) sace_7_1_a02410 LYFALLYYSL  MGLANISFEH  --RPILQKHK  GYSLYHPSAE  AIGSTLASFP
(PRED) sace_59_110_df0 LYFALLYYSL  MGLANISFEH  --RPILQKHK  GYSLYHPSAE  AIGSTLASFP
(PRED) sace_56_1_a0202 LYFALLYYSL  MGLANISFEH  --RPILQKHK  GYSLYHPSAE  AIGSTLASFP
(PRED) sace_40_1_a0239 LYFALLYYSL  MGLANISFEH  --RPILQKHK  GYSLYHPSAE  AIGSTLASFP
(PRED) sace_15_1_a0242 LYFALLYYSL  MGLANISFEH  --RPILQKHK  GYSLYHPSAE  AIGSTLASFP
(PRED) sace_37_1_a0243 LYFALLYYSL  MGLANISFEH  --RPILQKHK  GYSLYHPSAE  AIGSTLASFP
(PRED) sace_9_1_a02440 LYFALLYYSL  MGLANISFEH  --RPILQKHK  GYSLYHPSAE  AIGSTLASFP
(PRED) sace_22_1_2300  LYFALLYYSL  MGLANISFEH  --RPILQKHK  GYSLYHPSAE  AIGSTLASFP
(PRED) sace_29_1_2290  LYFALLYYSL  MGLANISFEH  --RPILQKHK  GYSLYHPSAE  AIGSTLASFP
(PRED) sace_34_1_2320  LYFALLYYSL  MGLANISFEH  --RPILQKHK  GYSLYHPSAE  AIGSTLASFP
(PRED) sace_58_25_y007 LYFALLYYSL  MGLANISFEH  --RPILQKHK  GYSLYHPSAE  AIGSTLASFP
(PRED) sace_23_1_2290  LYFALLYYSL  MGLANISFEH  --RPILQKHK  GYSLYHPSAE  AIGSTLASFP
```

(PRED)	sace_6_120_dp00	LYFALLYYSL	MGLANISFEH	--RPILQKHK	GYSLYHPSAE	AIGSTLASFP
(PRED)	sace_57_1_a0241	LYFALLYYSL	MGLANISFEH	--RPILQKHK	GYSLYHPSAE	AIGSTLASFP
(PRED)	sace_17_1_a0241	LYFALLYYSL	MGLANISFEH	--RPILQKHK	GYSLYHPSAE	AIGSTLASFP
(PRED)	sace_21_1_2310	LYFALLYYSL	MGLANISFEH	--RPILQKHK	GYSLYHPSAE	AIGSTLASFP
(PRED)	sace_49_1_a0246	LYFALLYYSL	MGLANISFEH	--RPILQKHK	GYSLYHPSAE	AIGSTLASFP
(PRED)	sace_8_2_b02430	LYFALLYYSL	MGLANISFEH	--RPILQKHK	GYSLYHPSAE	AIGSTLASFP
(PRED)	sace_31_1_2300	LYFALLYYSL	MGLANISFEH	--RPILQKHK	GYSLYHPSAE	AIGSTLASFP
(PRED)	sace_50_1_a0241	LYFALLYYSL	MGLANISFEH	--RPILQKHK	GYSLYHPSAE	AIGSTLASFP
(PRED)	sace_4_1_a02360	LYXALLYYSL	MGLANISFEH	--RPILQKHK	GYSLYHPSAE	AIGSTLASFP
(PRED)	sace_2_1_a02390	LYFALLYYSL	MGLANISFEH	--RPILQKHK	GYSLYHPSAE	AIGSTLASFP
(PRED)	sace_5_33_ag005	LYXALLYYSL	MGLANISFEH	--RPILQKHK	GYSLYHPSAE	AIGSTLASFP
(PRED)	sapa_11_1_a0247	LYFALLYYSL	MGLANISFEH	--RPILQKHK	GYSLYHPSAE	AIGSTLASFP
(PRED)	sapa_25_1_a0246	LYFALLYYSL	MGLANISFEH	--RPILQKHK	GYSLYHPSAE	AIGSTLASFP
(PRED)	sapa_4_1_a02470	LYFALLYYSL	MGLANISFEH	--RPILQKHK	GYSLYHPSAE	AIGSTLASFP
(PRED)	sapa_5_1_2350	LYFALLYYSL	MGLANISFEH	--RPILQKHK	GYSLYHPSAE	AIGSTLASFP
(PRED)	sapa_9_1_2360	LYFALLYYSL	MGLANISFEH	--RPILQKHK	GYSLYHPSAE	AIGSTLASFP
(PRED)	sapa_14_1_a0244	LYFALLYYSL	MGLANISFEH	--RPILQKHK	GYSLYHPSAE	AIGSTLASFP
(PRED)	sapa_8_1_2350	LYFALLYYSL	MGLANISFEH	--RPILQKHK	GYSLYHPSAE	AIGSTLASFP
(PRED)	sapa_17_1_2380	LYFALLYYSL	MGLANISFEH	--RPILQKHK	GYSLYHPSAE	AIGSTLASFP
(PRED)	sapa_7_1_2370	LYFALLYYSL	MGLANISFEH	--RPILQKHK	GYSLYHPSAE	AIGSTLASFP
(PRED)	sapa_2_1_a02460	LYFALLYYSL	MGLANISFEH	--RPILQKHK	GYSLYHPSAE	AIGSTLASFP
(PRED)	sapa_23_1_a0248	LYFALLYYSL	MGLANISFEH	--RPILQKHK	GYSLYHPSAE	AIGSTLASFP
(PRED)	sapa_3_1_a02470	LYFALLYYSL	MGLANISFEH	--RPILQKHK	GYSLYHPSAE	AIGSTLASFP
(PRED)	sapa_18_1_2390	LYFALLYYSL	MGLANISFEH	--RPILQKHK	GYSLYHPSAE	AIGSTLASFP
(PRED)	sami_1_4_244	LYFALLYYSL	MGLANISFEH	--RPILQKHK	GYSLYHPSAE	AIGSTLASFP
(PRED)	saku_1_4_262	LYFALLYYSL	MGLANISFEH	--RPILQKHK	GYSLYHPSAE	AIGSTLAAFP
(PRED)	saba_1_58_bf002	LYFALLYYSL	MGLANISFEH	--RPILQKHK	GYSLYHPSAE	AIGSTLAAFP
(PRED)	saeu_1_4_d02400	LYFALLYYSL	MGLANISFDH	--RPILQKHK	GYSLYHPSAE	AIGSTLASFP
(PRED)	naca_1_e01640	LYFALLFYSL	MGLANISFDD	--RHILQKHK	GYSLYHPSAE	AIASTVSSFP
(PRED)	nada_1_g01850	LYFALLYYSL	MGLANISFEN	--RPILQKHK	GYSLYHPSAE	AIASTISEFP
(PRED)	naca_1_e01630	LYFTLLYYSL	MGLANMSFDH	--RPILQKHK	GYSLYHPGAE	AIASALAAFP
(PRED)	nada_1_g01840	LYFMLLYYSL	MGLANISLEE	--RPILQKHK	GYSLYHLSAE	PLGSMLAGFP
(PRED)	kaaf_1_c00820	LYFCLLYYSL	MGLANIRFDY	--RPILQKQK	LYSFYHPSAE	ALGSTFSAFP
(PRED)	teph_1_m00640	LYFALLYYSL	IGLANINLDS	--RPILQKHK	AYSLYHLSAE	AFASTIAPMP
(PRED)	vapo_1_1036_28	LYFALLYYSL	LGLANISFDH	--RPILQKHK	GYSLYHPSAE	ALASTISAMP
(PRED)	tebl_1_i01760	LYFALLYYSL	MGLANMSFEH	--RPILQKHK	MYSLYHPSAE	AIASTLSGFP
(PRED)	tode_1_d04040	LYFALLYYSL	MGLANISFEH	--RPILQKHK	YYSLYHPSAE	ALGSTISGFP
(PRED)	naca_1_e01650	LYFSLLFYSL	MGLANITFEH	--RPILQKHK	NYSLYHPSAE	ALASTFSAAP
(PRED)	tebl_1_g02820	LYFALLYYCL	MGLANISFEH	--RPILQKHK	GYSLYHPAAE	ALASTLSAFP
(PRED)	lakl_1_c11616g	LYFSVLYYSL	MGLANISFED	--RPILQKHK	TYSLYHPSAE	ALASTISAFP
(PRED)	saar_1_8_h03780	LFFSLLYYSL	MGLANISFEH	--RPILHKHK	VYSLYHPSAE	ALGSTISSFP
(PRED)	sace_14_7_g0015	LFFSLLYYSL	MGLANISFEH	--RPILQKHK	VYSLYHPSAE	ALASTISSFP
(PRED)	sace_15_7_g0387	LFFSLLYYSL	MGLANISFEH	--RPILQKHK	VYSLYHPSAE	ALASTISSFP
(PRED)	sace_24_8_3780	LFFSLLYYSL	MGLANISFEH	--RPILQKHK	VYSLYHPSAE	ALASTISSFP
(PRED)	sace_40_8_h0383	LFFSLLYYSL	MGLANISFEH	--RPILQKHK	VYSLYHPSAE	ALASTISSFP
(PRED)	sace_6_169_fm00	LFFSLLYYSL	MGLANISFEH	--RPILQKHK	VYSLYHPSAE	ALASTISSFP
(PRED)	sace_19_7_3840	LFFSLLYYSL	MGLANISFEH	--RPILQKHK	VYSLYHPSAE	ALASTISSFP
(PRED)	sace_32_7_3770	LFFSLLYYSL	MGLANISFEH	--RPILQKHK	VYSLYHPSAE	ALASTISSFP
(PRED)	sace_56_17_q011	LFFSLLYYSL	MGLANISFEH	--RPILQKHK	VYSLYHPSAE	ALASTISSFP
(PRED)	sace_5_78_bz001	LFFSLLYYSL	MGLANISFEH	--RPILQKHK	VYSLYHPSAE	ALXSTISSFP
(PRED)	sace_2_8_h03860	LFFSLLYYSL	MGLANISFEH	--RPILQKHK	VYSLYHPSAE	ALASTISSFP
(PRED)	sace_53_29_ac00	LFFSLLYYSL	MGLANISFEH	--RPILQKHK	VYSLYHPSAE	ALASTISSFP
(PRED)	sace_17_7_g0393	LFFSLLYYSL	MGLANITFEH	--RPILQKHK	VYSLYHPSAE	ALASTISSFP
(PRED)	sace_25_7_g0388	LFFSLLYYSL	MGLANISFEH	--RPILQKHK	VYSLYHPSAE	ALASTISSFP
(PRED)	sace_37_7_g0385	LFFSLLYYSL	MGLANISFEH	--RPILQKHK	VYSLYHPSAE	ALASTISSFP
(PRED)	sace_9_7_g00180	LFFSLLYYSL	IGLANISFEH	--RPILQKHK	VYSLYHPSAE	ALASTISSFP
(PRED)	sace_60_6_f0335	LFFSLLYYSL	IGLANISFEH	--RPILQKHK	VYSLYHPSAE	ALASTISSFP
(PRED)	sace_59_336_lx0	LFFSLLYYSL	MGLANISFEH	--RPILQKHK	VYSLYHPSAE	ALASTISSFP
(PRED)	sace_31_7_3780	LFFSLLYYSL	MGLANISFEH	--RPILQKHK	VYSLYHPSAE	ALASTISSFP
(PRED)	sace_34_8_3770	LFFSLLYYSL	MGLANISFEH	--RPILQKHK	VYSLYHPSAE	ALASTISSFP
(PRED)	sace_58_71_bs00	LFFSLLYYSL	MGLANISFEH	--RPILQKHK	VYSLYHPSAE	ALASTISSFP
(PRED)	sace_7_7_g03880	LFFSLLYYSL	MGLANISFEH	--RPILQKHK	VYSLYHPSAE	ALASTISSFP
(PRED)	sace_35_7_3840	LFFSLLYYSL	MGLANISFEH	--RPILQKHK	VYSLYHPSAE	ALASTISSFP
(PRED)	sace_43_7_g0387	LFFSLLYYSL	MGLANISFEH	--RPILQKHK	VYSLYHPSAE	ALASTISSFP
(PRED)	sace_57_8_h0390	LFFSLLYYSL	MGLANISFEH	--RPILQKHK	VYSLYHPSAE	ALASTISSFP
(PRED)	sace_45_7_g0389	LFFSLLYYSL	MGLANISFEH	--RPILQKHK	VYSLYHPSAE	ALASTISSFP
(PRED)	sace_46_8_h0391	LFFSLLYYSL	MGLANISFEH	--RPILQKHK	VYSLYHPSAE	ALASTISSFP
(PRED)	sace_23_7_3860	LFFSLLYYSL	MGLANISFEH	--RPILQKHK	VYSLYHPSAE	ALASTISSFP
(PRED)	sace_21_7_3790	LFFSLLYYSL	MGLANISFEH	--RPILQKHK	VYSLYHPSAE	ALASTISSFP
(PRED)	sace_8_73_bu001	LFFSLLYYSL	MGLANISFEH	--RPILQKHK	VYSLYHPSAE	ALASTISSFP
(PRED)	sapa_1_8_h03820	LFFSLLYYSL	MGLANISFEH	--RPILQKHK	VYSLYHPSAE	ALGSTISSFP
(PRED)	sapa_21_8_h0387	LFFSLLYYSL	MGLANISFEH	--RPILQKHK	VYSLYHPSAE	ALGSTISSFP
(PRED)	sapa_20_8_h0386	LFFSLLYYSL	MGLANISFEH	--RPILQKHK	VYSLYHPSAE	ALGSTISSFP
(PRED)	sapa_22_8_h0390	LFFSLLYYSL	MGLANISFEH	--RPILQKHK	VYSLYHPSAE	ALGSTISSFP
(PRED)	sapa_25_8_h0387	LFFSLLYYSL	MGLANISFEH	--RPILQKHK	VYSLYHPSAE	ALGSTISSFP

(PRED)	sapa_6_8_3750	LFFSLLYYSL	MGLANISFEH	--RPILQKHK	VYSLYHPSAE	ALGSTISSFP
(PRED)	sapa_9_8_3720	LFFSLLYYSL	MGLANISFEH	--RPILQKHK	VYSLYHPSAE	ALGSTISSFP
(PRED)	sapa_19_8_h0390	LFFSLLYYSL	MGLANISFEH	--RPILQKHK	VYSLYHPSAE	ALGSTISSFP
(PRED)	sapa_24_8_h0385	LFFSLLYYSL	MGLANISFEH	--RPILQKHK	VYSLYHPSAE	ALGSTISSFP
(PRED)	sapa_4_8_h03850	LFFSLLYYSL	MGLANISFEH	--RPILQKHK	VYSLYHPSAE	ALGSTISSFP
(PRED)	sapa_10_8_3760	LFFSLLYYSL	MGLANISFEH	--RPMLQKHK	VYSLYHPSAE	ALGSTISSFP
(PRED)	sapa_13_8_h0382	LFFSLLYYSL	MGLANISFEH	--RPILQKHK	VYSLYHPSAE	ALGSTISSFP
(PRED)	sapa_8_8_3750	LFFSLLYYSL	MGLANISFEH	--RPILQKHK	VYSLYHPSAE	ALGSTISSFP
(PRED)	sapa_11_8_h0383	LFFXLLYYSL	MGLANISFEH	--RPILQKHK	VYSLYHPSAE	ALGSTISSFP
(PRED)	sapa_5_8_3700	LFFSLLYYSL	MGLANISFEH	--RPILQKHK	VYSLYHPSAE	ALGSTISSFP
(PRED)	sapa_16_8_h0389	LFFSLLYYSL	MGLANISFEH	--RPILQKHK	VYSLYHPSAE	ALGSTISSFP
(PRED)	sapa_17_8_3730	LFFSLLYYSL	MGLANISFEH	--RPILQKHK	VYSLYHPSAE	ALGSTISSFP
(PRED)	sapa_2_8_h03860	LFFSLLYYSL	MGLANISFEH	--RPILQKHK	VYSLYHPSAE	ALGSTISSFP
(PRED)	sapa_7_8_3740	LFFSLLYYSL	MGLANISFEH	--RPILQKHK	VYSLYHPSAE	ALGSTISSFP
(PRED)	sapa_23_8_h0385	LFFSLLYYSL	MGLANISFEH	--RPILQKHK	VYSLYHPSAE	ALGSTISSFP
(PRED)	sapa_3_8_h03890	LFFSLLYYSL	MGLANISFEH	--RPILQKHK	VYSLYHPSAE	ALGSTISSFP
(PRED)	sapa_18_8_3730	LFFSLLYYSL	MGLANISFEH	--RPILQKHK	VYSLSHPSPE	ALGSTISSFP
(PRED)	sami_1_14_399	LFFSLLYYSL	MGLANISFEH	--RPILQKHK	VYSLYHPSAE	ALASTISSFP
(PRED)	sace_4_8_h03690	LFFSLLYYSL	MGLANISFEH	--RPILQKHK	VYSLYHPSAE	ALASTISSFP
(PRED)	saku_1_14_404	LFFSLLYYSL	MGLANISFEH	--RPILHKHK	VYSLYHPSAE	ALASTISSFP
(PRED)	sace_1_ynr070w	LFFSLLYYSL	MGLANISFEH	--RPILQKHK	VYSLYHPSAE	ALASTISSFP
(PRED)	sace_49_8_h0383	LFFSLLYYSL	MGLANISFEH	--RPILQKHK	VYSLYHPSAE	ALASTISSFP
(PRED)	saeu_1_2_b00130	LFFSLLYYSL	MGLASISFDH	--RLILKKHK	VYSLYHPSAE	ALASTISTFP
(PRED)	sauv_1_7_3	LFFSLLYYSL	MGLASISFDH	--RLILKKQK	VYSLYHPSAE	ALASTISTFP
(PRED)	sami_1_17_26	LFFSLLYYSL	MGLANINFEH	--RQILQKHR	VYSLYHPSAE	ALASTISSFP
(PRED)	zyba_1_02055_AN	LYFALLYYSL	MGLANITFDH	--RPILQKHK	TYSLYHPGAE	ALGSTLSGAP
(PRED)	zyba_1_07912	LYFALLYYSL	MGLANITFDH	--RPILQKHK	TYSLYHPGAE	ALGSTLSGAP
(PRED)	zyba_2_2_b00600	LYFALLYYSL	MGLANITFDH	--RPILQKHK	TYSLYHPGAE	ALGSTLSGAP
(PRED)	zyba_3_3_c03460	LYFALLYYSL	MGLANITFDH	--RPILQKHK	TYSLYHPGAE	ALGSTLSGAP
(PRED)	zyba_1_04634	LYFSLLYYSL	MGLANITFDH	--RPILQKHK	TYSLYHPGAE	ALGSTLSGVP
(PRED)	zyba_1_06675	LYFSLLYYSL	MGLANITFDH	--RPILQKHK	TYSLYHPGAE	ALGSTFSGMP
(PRED)	zyba_3_2_b02230	LYFSLLYYSL	MGLANITFDH	--RPILQKHK	TYSLYHPGAE	ALGSTFSGMP
(PRED)	zyba_2_1_a00860	LYFSLLYYSL	MGLANITFDH	--RPILQKHK	TYSLYHPGAE	ALGSTFSGMP
(PRED)	zyro_1_a04114g	LYFALLYYSL	MGLANISFDH	--RPILQKHK	TYSLYHPAAE	ALGSTFSGAP
(PRED)	zyro_1_b14762g	LYFSLLYYSL	MGLANISFDH	--RPILQKHK	TYSLYHPAAE	ALGSTFSGTP
(PRED)	zyba_2_14_n0149	LYFALLYYSL	MGMANISFEH	--RPILQKHK	IYSLYHPAAE	ALGSTMSGAP
(PRED)	zyba_2_33_ag001	LYFALLYYSL	MGMANISFEH	--RPILQKHK	IYSLYHPAAE	ALGSTMSGAP
(PRED)	lath_1_a01914g	FYFSILYVSL	MGLAKISLDG	--RPVVQKHK	SYSLYHPAAE	ALATSLSEFP
(PRED)	lawa_1_23_5161	FYFAILYASL	MGLAKINLDG	--RPVMLKHR	SYSLYHPAAD	ALAGSISEFP
(PRED)	klae_1_14_n0012	IYFSLLYFSL	MGLANINFEH	--RPILQKHK	AYSLYHPSAE	AFASTFSAAC
(PRED)	klla_1_d03432g	LYFAILYFSL	MGLANISLAN	--RPILQKHI	AYSLYHPSAE	ALASTISNAF
(PRED)	klma_1_1_a01880	LYFAILYFSL	MGLANISLAN	--RPILQKHI	AYSLYHPSAE	AFASTVSSAF
(PRED)	klwi_1_33_ag001	LYFAILYFSL	MGLANISLAN	--RPILQKHI	GYSFYHPSAE	SLASTISGAF
(PRED)	teph_1_a04220	LYFALLYYSL	MGLASVTFDQ	--KLIVHKHK	SYCLYHPSAE	ALASTIAAFP
(PRED)	vapo_1_1037_47	HFFMLLYYSL	ISLANISFAQ	--RPIINKHK	SYSFYHPAAE	ALSENLSFFP
(PRED)	pata_1_2_b05590	MFYSCLYFSL	MGLTEMPTVF	SDRPILLKQK	GYSFYHPSAE	ILSETVTLTP
(PRED)	wian_1_3_c04380	LYFLILYFSL	MGLAEISGQF	TERPILLKQK	SYSFYHPSAE	TIAATMTKFP
(PRED)	wian_1_3_c04390	LYFMILYFSL	MGLAEVSGQF	ADRPILLKQK	SYSFYHPSAE	TISATLTKFP
(PRED)	wian_1_7_g01010	IYFMVLYFSL	MGLAEVSGQF	ADRPILLKQK	SYSFYHPSAE	TLAGTITKFP
(PRED)	bain_1_1_a00100	LFFSCLYFCF	MGIAEMANCF	AKRPIVQKQK	SYSFYHPSAE	AFSSVLTDFP
(PRED)	bain_1_17_q0038	LFFACLYFAF	MGIAEMANCF	AKRPIIQKQK	SYSFYHPSAE	AFSSVLTDFP
(PRED)	bain_1_8_h00410	LFQGMFFFVF	MGVSEMATVF	GKRRIVEKQK	NYSFYHPSAE	SLATLISTLP
(PRED)	caal_1_19_5759	IFFAVLFMSL	MGLAEISASF	SSRPILMKQK	NYTMYHPSAD	SLSNFVMSIP
(PRED)	caal_11_25_y002	IFFAVLFMSL	MGLAEISASF	SSRPILMKQK	NYTMYHPSAD	SLSNFVMSIP
(PRED)	caal_4_4_d03320	IFFAVLFMSL	MGLAEISASF	SSRPILMKQK	NYTMYHPSAD	SLSNFVMSIP
(PRED)	caal_12_26_z005	IFFAVLFMSL	MGLAEISASF	SSRPILMKQK	NYTMYHPSAD	SLSNFVMSIP
(PRED)	caal_5_30_ad005	IFFAVLFMSL	MGLAEISASF	SSRPILMKQK	NYTMYHPSAD	SLSNFVMSIP
(PRED)	caal_8_3_c03320	IFFAVLFMSL	MGLAEISASF	SSRPILMKQK	NYTMYHPSAD	SLSNFVMSIP
(PRED)	caal_6_4_d03280	IFFAVLFMSL	MGLAEISASF	SSRPILMKQK	NYTMYHPSAD	SLSNFVMSIP
(PRED)	caal_10_3_c0334	IFFAVLFMSL	MGLAEISASF	SSRPILMKQK	NYTMYHPSAD	SLSNFVMSIP
(PRED)	caal_3_29_ac005	IFFAVLFMSL	MGLAEISASF	SSRPILMKQK	NYTMYHPSAD	SLSNFVMSIP
(PRED)	caal_2_04989	IFFAVLFMSL	MGLAEISASF	SSRPILMKQK	NYTMYHPSAD	SLSNFVMSIP
(PRED)	cadu_1_64350	IFFAVLFMSL	MGLAEISASF	SSRPILMKQK	NYTMYHPSAD	SLSNFVMSIP
(PRED)	caor_1_h02090	VFFAVLFMAL	MGLAEISASF	SSRPILMKQK	NYSMYHPSAD	ALSNFVTSIP
(PRED)	capa_1_600750	VFFAVLYMAL	MGLAEISASF	SSRMILMKQK	NYSMYHPSAD	ALANFVTSVP
(PRED)	loel_1_04930	IFFAVLFMSL	MGLAEISASF	ASRPILMKQK	NYSMYHPSAD	SLSNFVTSIP
(PRED)	spar_1_5_e03260	IFFAVLFMSL	MGLAQISASF	SSRPILMKHK	NYTLYHPSAD	ALGNFVISIP
(PRED)	sppa_1_7_g03160	IFFAVLYMSL	MGLAEISASF	GARPILMKHK	NYTLYHPSAD	ALGNFIISIP
(PRED)	catr_1_01205	IFFAVLYMSL	MGLAEISASF	SNRQILMKQK	NYSMYHPSAD	ALSQFIMSIP
(PRED)	catr_1_05498	IFFAVLFMSL	MGLAEISASF	RNRLILNKQK	NYSMYHPSAD	ALSQFVMAIP
(PRED)	catr_1_05971	IFFAVLFMSL	MGLAEISASF	SNRQILMKQK	NYSMYHPSAD	ALSQFVMSIP
(PRED)	deha_1_a03696g	IFFAALYVSL	MGLAEVSASF	NSRSILMKQK	NYSMYHPSAD	ALASVVTSIP
(PRED)	deha_2_5_e00720	IFFAALYVSL	MGLAEVSASF	SSRSILMKQK	NYSMYHPSAD	ALASVVTSIP
(PRED)	scst_1_3_c02890	VFFGVLYVSL	MGLAEVSASF	ANRPILMKHK	NYSMYHPAAD	AVGSFITSIP
(PRED)	mebi_1_8_h00300	IFFSVLYMSL	SGMAEISNAF	GAIPILMKQR	NYKLYHPAAF	ALADSVSFIP

(PRED) lakl_1_h21010g LYFSLLYYSL MGLANIGVSH --RLIVSKHK SYSLYHPSAE TLATYISSFP
(PRED) caar_1_13_m0142 IFFGLLYFCI MSLAEVSSIF ATKPILSKQR GYSLYHPSAE FLGSTLTQLP
(PRED) caar_1_14_n0143 IFFALLYFCI MGLAEVASVF ENKPILNKQR GYTLFHPSAE FLSSTITQIP
(PRED) hapo_1_1_a07220 IFFAFLYFCI MSLAEIAAFF ENKPITNKQR GYSFFHPSAD LVSSFLTQTP
(PRED) ogpa_1_1_a01680 IFFAFLYFCI MSLAEIAAFF ENKPITNKQR GYSFFHPSAD LVSSFLTQTP
(PRED) piku_1_96_cr001 LFFSCLYFCI MALAEIAALF MDKPILNKQI SYTMYRPSAE LLAKQMVSYP
(PRED) pime_1_4_d03240 LFFSCLYFCI MCLAETADLF MDKPILNKQY AYTMYQPSSE LLAKQLVAFP
(PRED) pime_1_1_a12110 IFFSLLYFSI MSLAETATLF EDKPILNKQY GYTLYHPSAE LLAKQIVSIP
(PRED) piku_1_227_hs00 IFFACLYFSI MSLAQTPVLF DDKPVLNKQY AYHFYHPSAE LIAKQIIQIP
(PRED) pime_1_5_e05800 IFFACLYFSI MTLAETPVLF QDKPILNKQY AYTMYHPSAE LISKQLVQFP
(PRED) pime_1_1_a07690 LFFSCLYFAL MTLAETPALF LDKPILNKQY GYKLYHPSAE LLAKHLVQFP
(PRED) debr_2_5_e03380 LFFAIFYFVV MGLAQIAALF QSRPILNKQR GYTLYHPSAQ LLSAKLIELP
(PRED) kopa_1_2_b10040 IFFAILFFVL MSLAEIANIF KDRPVLAKQI GYSLYHPSTE VIANALIQIP
(PRED) kopa_2_7_g00500 IFFAILFFVL MSLAEIANIF KDRPVLAKQI GYSLYHPSTE VIANALIQIP
(PRED) asru_1_13_m0119 LFFCVLHFTF LGLAEVSNSF DNRPIILKQK GYTLYHPAAE AIASVLTDIP
(PRED) asru_1_15_o0045 LFFATFLFTF TPMVSVINFF QIRFVLLKQK AYTFYHPGAE AIASTLADIP
(PRED) wian_1_1_a02920 IFFSVIFFVF NTFAEMIFLF QTCQIVNRHR SYSFYAPSAE VIADLVINLI
(PRED) wian_1_1_a02930 IFFSLIFFVF LASAEIIISF QNRPIIQRHR SYSFYSPSAE AIADLLTDFP

.........860 ....870 ....880 ....890 900

(PRED) asac_1_6_f03560 FRMIALTLFL IVLYFLSGLT VQAWRFFSIY LFLVLAAEGI NAMFEVITAL
(PRED) ergo_1_abr125c FRMIALTLFL IVLYFLSGLT VEAWRFFSIY LFLVLASEGI NAMFEVITAL
(PRED) ercy_1_3604 FRLIGLTAFF IVLYFLSGLT YEADRFFVVY LFLVMAAESI NALFEMITAL
(PRED) cagl_1_i04862g FRMIGMTCFL IIIYFLSGLN RTASSFFRVY LFLTMCSESI NALFELIAAG
(PRED) kaaf_1_c00830 FRMIGLTCFL IILYFLAGLH TNAGSFFTVY LFLTMCSESI TGLFEMVASA
(PRED) kana_1_k01350 FRMIGLTLFL IIIYFLAGLH TNAGSFFTVY LFLTMCSEAI TNLFDMISAG
(PRED) saar_1_2_b02590 FRMIGLTCFL IILFFLSGLH RTAGSFFTIY LFLTMCSEAI NGLFEMVSSV
(PRED) sace_1_ydr011w FRMIGLTCFF IILFFLSGLH RTAGSFFTIY LFLTMCSEAI NGLFEMVSSV
(PRED) sace_16_1_a0238 FRMIGLTCFF IILFFLSGLH RTAGSFFTIY LFLTMCSEAI NGLFEMVSSV
(PRED) sace_45_1_a0242 FRMIGLTCFF IILFFLSGLH RTAGSFFTIY LFLTMCSEAI NGLFEMVSSV
(PRED) sace_48_1_a0238 FRMIGLTCFF IILFFLSGLH RTAGSFFTIY LFLTMCSEAI NGLFEMVSSV
(PRED) sace_60_4_d0244 FRMIGLTCFF IILFFLSGLH RTAGSFFTIY LFLTMCSEAI NGLFEMVSSV
(PRED) sace_52_1_a0240 FRMIGLTCFF IMLFFLSGLH RTAGSFFTIY LFLTMCSEAI NGLFEMVSSV
(PRED) sace_46_1_a0240 FRMIGLTCFF IILFFLSGLH RTAGTFFTIY LFLTMCSEAI NGLFEMVSSV
(PRED) sace_25_1_a0240 FRMIGLTCFF IILFFLSGLH RTAGSFFTIY LFLTMCSEAI NGLFEMVSSV
(PRED) sace_24_1_2300 FRMIGLTCFF IILFFLSGLH RTAGTFFTIY LFLAMCSEAI NGLFEMVSSV
(PRED) sace_47_1_a0240 FRMIGLTCFF IILFFLSGLH RTAGTFFTIY LFLTMCSEAI NGLFEMVSSV
(PRED) sace_7_1_a02410 FRMIGLTCFF IILFFLSGLH RTAGTFFTIY LFLTMCSEAI NGLFEMVSSV
(PRED) sace_59_110_df0 FRMIGLTCFF IILFFLSGLH RTAGTFFTIY LFLTMCSEAI NGLFEMVSSV
(PRED) sace_56_1_a0202 FRMIGLTCFF IILFFLSGLH RTAGSFFTIY LFLTMCSEAI NGLFEMVSSV
(PRED) sace_40_1_a0239 FRMIGLTCFF IILFFLSGLH RTAGTFFTIY LFLTMCSEAI NGLFEMVSSV
(PRED) sace_15_1_a0242 FRMIGLTCFF IILFFLSGLH RTAGSFFTIY LFLTMCSEAI NGLFEMVSSV
(PRED) sace_37_1_a0243 FRMIGLTCFF IILFFLSGLH RTAGSFFTIY LFLTMCSEAI NGLFEMVSSV
(PRED) sace_9_1_a02440 FRMIGLTCFF IILFFLSGLH RTAGSFFTIY LFLTMCSEAI NGLFEMVSSV
(PRED) sace_22_1_2300 FRMIGLTCFF IILFFLSGLH RTAGSFFTIY LFLTMCSEAI NGLFEMVSSV
(PRED) sace_29_1_2290 FRMIGLTCFF IILFFLSGLH RTAGSFFTIY LFLTMCSEAI NGLFEMVSSV
(PRED) sace_34_1_2320 FRMIGLTCFF IILFFLSGLH RTAGSFFTIY LFLTMCSEAI NGLFEMVSSV
(PRED) sace_58_25_y007 FRMIGLTCFF IILFFLSGLH RTAGSFFTIY LFLTMCSEAI NGLFEMVSSV
(PRED) sace_23_1_2290 FRMIGLTCFF IILFFLSGLH RTAGSFFTIY LFLTMCSEAI NGLFEMVSSV
(PRED) sace_6_120_dp00 FRMIGLTCFF IILFFLSGLH RTAGSFFTIY LFLTMCSEAI NGLFEMVSSV
(PRED) sace_57_1_a0241 FRMIGLTCFF IILFFLSGLH RTAGSFFTIY LFLTMCSEAI NGLFEMVSSV
(PRED) sace_17_1_a0241 FRMIGLTCFF IILFFLSGLH RTAGSFFTIY LFLTMCSEAI NGLFEMVSSV
(PRED) sace_21_1_2310 FRMIGLTCFF IILFFLSGLH RTAGSFFTIY LFLTMCSEAI NGLFEMVSSV
(PRED) sace_49_1_a0246 FRMIGLTCFF IILFFLSGLH RTAGXFFTIY LFLTMCSEAI NGLFEMVSSV
(PRED) sace_8_2_b02430 FRMIGLTCFF IILFFLSGLH RTAGSFFTIY LFLTMCSEAI NGLFEMVSSV
(PRED) sace_31_1_2300 FRMIGLTCFF IILFFLSGLH RTAGSFFTIY LFLTMCSEAI NGLFEMVSSV
(PRED) sace_50_1_a0241 FRMIGLTCFF IILFFLSGLH RTAGTFFTIY LFLTMCSEAI NGLFEMVSSV
(PRED) sace_4_1_a02360 FRMIGLTCFF IILFFLSGLH RTAGTFFTIY LFLTMCSEAI NGLFEMVSSV
(PRED) sace_2_1_a02390 FRMIGLTCFF IILFFLSGLH RTAGSFFTIY LFLTMCSEAI NGLFEMVSSV
(PRED) sace_5_33_ag005 FRMIGLTCFF IILFFLSGLX RTAGXFFTIY LFLTMCSEAI NGLFEMVSSV
(PRED) sapa_11_1_a0247 FRMIGLTCFF IILFFLSGLH RTAGSFFTIY LFLTMCSEAI NGLFEMVSSV
(PRED) sapa_25_1_a0246 FRMIGLTCFF IILFFLSGLH RTAGSFFTIY LFLTMCSEAI NGLFEMVSSV
(PRED) sapa_4_1_a02470 FRMIGLTCFF IILFFLSGLH RTAGSFFTIY LFLTMCSEAI NGLFEMVSSV
(PRED) sapa_5_1_2350 FRMIGLTCFF IILFFLSGLH RTAGSFFTIY LFLTMCSEAI NGLFEMVSSV
(PRED) sapa_9_1_2360 FRMIGLTCFF IILFFLSGLH RTAGSFFTIY LFLTMCSEAI NGLFEMVSSV
(PRED) sapa_14_1_a0244 FRMIGLTCFF IILFFLSGLH RTAGSFFTIY LFLTMCSEAI NGLFEMVSSV
(PRED) sapa_8_1_2350 FRMIGLTCFF IILFFLSGLH RTAGSFFTIY LFLTMCSEAI NGLFEMVSSV
(PRED) sapa_17_1_2380 FRMIGLTCFF IILFFLSGLH RTAGSFFTIY LFLTMCSEAI NGLFEMVSSV
(PRED) sapa_7_1_2370 FRMIGLTCFF IILFFLSGLH RTAGSFFTIY LFLTMCSEAI NGLFEMVSSV
(PRED) sapa_2_1_a02460 FRMIGLTCFF IILFFLSGLH RTAGSFFTIY LFLTMCSEAI NGLFEMVSSV
(PRED) sapa_23_1_a0248 FRMIGLTCFF IILFFLSGLH RTAGSFFTIY LFLTMCSEAI NGLFEMVSSV
(PRED) sapa_3_1_a02470 FRMIGLTCFF IILFFLSGLH RTAGSFFTIY LFLTMCSEAI NGLFEMVSSV
(PRED) sapa_18_1_2390 FRMIGLTCFF ITLFFLSGLH RTAGSFFTIY LFLTMCSEAI NGLFEMVSSV
(PRED) sami_1_4_244 FRMIGLTCFF IILFFLSGLH RTAGSFFTIY LFLTMCSEAI NGLFEMVSSV
(PRED) saku_1_4_262 FRMIGLTCFF IILFFLSGLH RTAGSFFTIY LFLTMCSEAI NGLFEMVSSV

(PRED)	saba_1_58_bf002	FRMIGLTCFF	IILFFLSGLH	RTAGSFFTIY	LFLAMCSEAI	NGLFEMVSSV
(PRED)	saeu_1_4_d02400	FRMIGLTCFF	IILFFLSGLH	RTAGSFFTIY	LFLVMCSEAI	NGLFEMISSM
(PRED)	naca_1_e01640	FRMIGLTCFL	IILYFLAGLH	VNAGAFFMVY	LFLSMCSECI	TGLFQMIAAG
(PRED)	nada_1_g01850	FRMIGRTLFI	IILYFLAGLH	TSAGAFFMVY	LFLTLTTEGI	SLMFEMIASA
(PRED)	naca_1_e01630	FRMIGLTCFF	IIIFFLTNLH	RAPGPFFMMY	LFLTMCSEAI	NGLFEMIAAG
(PRED)	nada_1_g01840	FRMIGLTLFL	IIIFFLTNLH	RTPSAFFIIY	MFLTMCSEAI	NGLFQMVTSG
(PRED)	kaaf_1_c00820	FRMIGLTCFL	IILYFLSGLR	RSAGAFFIVY	LFLTLCSEAI	TGLFEMVAAA
(PRED)	teph_1_m00640	FRLIGLTCFI	IILYFLAGLH	TSAGAFFTVY	LFLFLCSETI	NGLFEMITSV
(PRED)	vapo_1_1036_28	FRLIGITCFL	IILFFLSGLH	RTASTFFIVY	LFLIMCAEAI	NGLFEMISSG
(PRED)	tebl_1_i01760	FRMIGLTCFL	IILYFLAYLH	QTAGSFFIIY	LFLVMCSEAI	NALFECVASA
(PRED)	tode_1_d04040	FRMIGLTCFI	IILFFLSGLH	RTASTFFIVY	LFLSMCSEAI	NGLFEMVAAA
(PRED)	naca_1_e01650	FRMISLTCFI	IILYFLAGLH	VDAGAFFIVY	LFLTMCSETI	NSLFEIDYRR
(PRED)	tebl_1_g02820	FRMISLTIFF	IVLFFLSGLH	RTASNFFICY	LFLSMCSEAI	NGLFEMISAG
(PRED)	lakl_1_c11616g	FRMVGLTCFL	IILYFLSGLT	REAGRFFIVY	LFLTICSESI	TALFEMVTAI
(PRED)	saar_1_8_h03780	FRMIGLTCFM	IILYFLAGLH	TNAGSFFIMY	LFLSMCSEAI	TGLFQMISSM
(PRED)	sace_14_7_g0015	FRMIGLTFFI	IILYFLAGLH	RSAGAFFTMY	LFLTMCSEAI	TSLFQMVSSL
(PRED)	sace_15_7_g0387	FRMIGLTFFI	IILYFLAGLH	RSAGAFFTMY	LLLTMCSEAI	TSLFQMVSSL
(PRED)	sace_24_8_3780	FRMIGLTFFI	IILYFLAGLH	RSAGAFFTMY	LLLTMCSEAI	TSLFQMVSSL
(PRED)	sace_40_8_h0383	FRMIGLTFFI	IILYFLAGLH	RSAGAFFTMY	LLLTMCSEAI	TSLFQMVSSL
(PRED)	sace_6_169_fm00	FRMIGLTFFI	IILYFLAGLH	RSAGAFFTMY	LLLTMCSEAI	TSLFQMVSSL
(PRED)	sace_19_7_3840	FRMIGLTFFI	IILYFLAGLH	RSAGAFFTMY	LLLTMCSEAI	TSLFQMVSSL
(PRED)	sace_32_7_3770	FRMIGLTFFI	IILYFLAGLH	RSAGAFFTMY	LLLTMCSEAI	TSLFQMVSSL
(PRED)	sace_56_17_q011	FRMIGLTFFI	IILYFLAGLH	RSAGAFFTMY	LFLTMCSEAI	TSLFQMVSSL
(PRED)	sace_5_78_bz001	FRMIGLTFFI	IILYFLAGLH	RSAGAFFTMY	LXLTMCSEAI	TSLFQMVSSL
(PRED)	sace_2_8_h03860	FRMIGLTFFI	IILYFLAGLH	RSAGAFFTMY	LLLTMCSEAI	TSLFQMVSSL
(PRED)	sace_53_29_ac00	FRMIGLTFFI	IILYFLAGLH	RSAGAFFTMY	LLLTMCSEAI	TSLFQMVSSL
(PRED)	sace_17_7_g0393	FRMIGLTFFI	IILYFLAGLH	RSAGAFFTMY	LFLTMCSEAI	TSLFQMVSSL
(PRED)	sace_25_7_g0388	FRMIGLTFFI	IILYFLAGLH	RSAGAFFTMY	LFLTMCSEAI	TSLFQMVSSL
(PRED)	sace_37_7_g0385	FRMIGLTFFI	IILYFLAGLH	RSAGAFFTMY	LFLTMCSEAI	TSLFQMVSSL
(PRED)	sace_9_7_g00180	FRMIGLTFFI	IILYFLAGLH	RSAGAFFTMY	LFLTMCSEAI	TSLFQMVSSL
(PRED)	sace_60_6_f0335	FRMIGLTFFI	IILYFLAGLH	RSAGAFFTMY	LFLTMCSEAI	TSLFQMVSSL
(PRED)	sace_59_336_lx0	FRMIGLTFFI	IILYFLAGLH	RSAGAFFTMY	LFLTMCSEAI	TSLFQMVSSL
(PRED)	sace_31_7_3780	FRMIGLTFFI	IILYFLAGLH	RSAGAFFTMY	LFLTMCSEAI	TSLFQMVSSL
(PRED)	sace_34_8_3770	FRMIGLTFFI	IILYFLAGLH	RSAGAFFTMY	LFLTMCSEAI	TSLFQMVSSL
(PRED)	sace_58_71_bs00	FRMIGLTFFI	IILYFLAGLH	RSAGAFFTMY	LFLTMCSEAI	TSLFQMVSSL
(PRED)	sace_7_7_g03880	FRMIGLTFFI	IILYFLAGLH	RSAGAFFTMY	LLLTMCSEAI	TSLFQMVSSL
(PRED)	sace_35_7_3840	FRMIGLTFFI	IILYFLAGLH	RSAGAFFTMY	LFLTMCSEAI	TSLFQMVSSL
(PRED)	sace_43_7_g0387	FRMIGLTFFI	IILYFLAGLH	RSAGAFFTMY	LFLTMCSEAI	TSLFQMVSSL
(PRED)	sace_57_8_h0390	FRMIGLTFFI	IILYFLAGLH	RSAGAFFTMY	LFLTMCSEAI	TSLFQMVSSL
(PRED)	sace_45_7_g0389	FRMIGLTFFI	IILYFLAGLH	RSAGAFFTMY	LFLTMCSEAI	TSLFQMVSSL
(PRED)	sace_46_8_h0391	FRMIGLTFFI	IILYFLAGLH	RSAGAFFTMY	LFLTMCSEAI	TSLFQMVSSL
(PRED)	sace_23_7_3860	FRMIGLTFFI	IILYFLAGLH	RSAGAFFTMY	LFLTMCSEAI	TSLFQMVSSL
(PRED)	sace_21_7_3790	FRMIGLTFFI	IILYFLAGLH	RSAGAFFTMY	LFLTMCSEAI	TSLFQMVSSL
(PRED)	sace_8_73_bu001	FRMIGLTFFI	IILYFLAGLH	RSAGAFFTMY	LFLTMCSEAI	TSLFQMVSSL
(PRED)	sapa_1_8_h03820	FRMIGLTFFI	IILYFLAGLH	RSAGAFFTMY	LFLTMCSEAI	TSLFQMVSSL
(PRED)	sapa_21_8_h0387	FRMIGLTFFI	IILYFLAGLH	RSAGAFFTMY	LFLTMCSEAI	TSLFQMVSSL
(PRED)	sapa_20_8_h0386	FRMIGLTFFI	IILYFLAGLH	RSAGAFFTMY	LFLTMCSEAI	TSLFQMVSSL
(PRED)	sapa_22_8_h0390	FRMIGLTFFI	IILYFLAGLH	RSAGAFFTMY	LFLTMCSEAI	TSLFQMVSSL
(PRED)	sapa_25_8_h0387	FRMIGLTFFI	IILYFLAGLH	RSAGAFFTMY	LFLTMCSEAI	TSLFQMVSSL
(PRED)	sapa_6_8_3750	FRMIGLTFFI	IILYFLAGLH	RSAGAFFTMY	LFLTMCSEAI	TSLFQMVSSL
(PRED)	sapa_9_8_3720	FRMIGLTFFI	IILYFLAGLH	RSAGAFFTMY	LFLTMCSEAI	TSLFQMVSSL
(PRED)	sapa_19_8_h0390	FRMIGLTFFI	IILYFLAGLH	RSAGAFFTMY	LFLTMCSEAI	TSLFQMVSSL
(PRED)	sapa_24_8_h0385	FRMIGLTFFI	IILYFLAGLH	RSAGAFFTMY	LFLTMCSEAI	TSLFQMVSSL
(PRED)	sapa_4_8_h03850	FRMIGLTFFI	IILYFLAGLH	RSAGAFFTMY	LFLTMCSEAI	TSLFQMVSSL
(PRED)	sapa_10_8_3760	FRMIGLTFFI	IILYFLAGLH	RSAGAFFTMY	LFLTMCSEAI	TSLFQMVSSL
(PRED)	sapa_13_8_h0382	FRMIGLTFFI	IILYFLAGLH	RSAGAFFTMY	LFLTMCSEAI	TSLFQMVSSL
(PRED)	sapa_8_8_3750	FRMIGLTFFI	IILYFLAGLH	RSAGAFFTMY	LFLTMCSEAI	TSLFQMVSSL
(PRED)	sapa_11_8_h0383	FRMIGLTFFI	IILYFLAGLH	RSAGAFFTMY	LFLTMCSEAI	TSLFQMVSSL
(PRED)	sapa_5_8_3700	FRMIGLTFFI	IILYFLAGLH	RSAGAFFTMY	LFLTMCSEAI	TSLFQMVSSL
(PRED)	sapa_16_8_h0389	FRMIGLTFFI	IILYFLAGLH	RSAGAFFTMY	LFLTMCSEAI	TSLFQMVSSL
(PRED)	sapa_17_8_3730	FRMIGLTFFI	IILYFLAGLH	RSAGAFFTMY	LFLTMCSEAI	TSLFQMVSSL
(PRED)	sapa_2_8_h03860	FRMIGLTFFI	IILYFLAGLH	RSAGAFFTMY	LFLTMCSEAI	TSLFQMVSSL
(PRED)	sapa_7_8_3740	FRMIGLTFFI	IILYFLAGLH	RSAGAFFTMY	LFLTMCSEAI	TSLFQMVSSL
(PRED)	sapa_23_8_h0385	FRMIGLTFFI	IILYFLAGLH	RSAGAFFTMY	LFLTMCSEAI	TSLFQMVSSL
(PRED)	sapa_3_8_h03890	FRMIGLTFFI	IILYFLAGLH	RSAGAFFTMY	LFLTMCSEAI	TSLFQMVSSL
(PRED)	sapa_18_8_3730	FRMIGLTFFI	IILYFLAGLH	RSAGAFFTMY	LFLTMCSEAI	TSLFQMVSSL
(PRED)	sami_1_14_399	FRMIGLTLFI	IILYFLAGLH	RSAGTFFTLY	LFLTMCSEAI	TSLFQMISSL
(PRED)	sace_4_8_h03690	FRMIGLTFFI	IILYFLAGLH	RSAGAFFTMY	LLLTMCSEAI	TSLFQMVSSL
(PRED)	saku_1_14_404	FRMIGLTFFI	IILYFLAGLH	TNAGVFFIMY	LFLTMCSEAI	TSLFQMISSL
(PRED)	sace_1_ynr070w	FRMIGLTFFI	IILYFLAGLH	RSAGAFFTMY	LLLTMCSEAI	TSLFQMVSSL
(PRED)	sace_49_8_h0383	FRMIGLTFFI	IILYFLAGLH	RSAGAFFTMY	LXLTMCSEAI	TSLFQMVSSL
(PRED)	saeu_1_2_b00130	FRMIGLTFFI	IILYFLAGLH	TSAGSFFMIY	LFLSMCSEAI	TGLFQMISSL
(PRED)	sauv_1_7_3	FRMIGLTFFI	IILYFLAGLH	RSAGSFFMIY	LFLTMCSEAI	TGLFQMISSL
(PRED)	sami_1_17_26	FRMIGLTFFL	IILYFLSGLH	VSAGSFFTVY	LFLSMCSETI	TGLFEMVSSL
(PRED)	zyba_1_02055_AN	FRMIGLTCFL	IILFFLSNLN	RTAGSFFRIY	FFLSLCSESI	YSLFEFIAAA

(PRED) zyba_1_07912 FRMIGLTCFL IILFFLSNLN RTAGSFFRIY FFLSLCSESI YSLFEFIAAA
(PRED) zyba_2_2_b00600 FRMIGLTCFL IILFFLSNLN RTAGSFFRIY FFLSLCSESI YSLFEFIAAA
(PRED) zyba_3_3_c03460 FRMIGLTCFL IILFFLSNLN RTAGSFFRIY FFLSLCSESI YSLFEFIAAA
(PRED) zyba_1_04634 FRMIGLTLFL IILFFLSNLN RTAGTFFRVY FFLTLSSESI YALFESIAAI
(PRED) zyba_1_06675 FRMIGLTLFL IILFFLSNLN RTAGTFFRVY FFLTLSSESI YALFESIAAI
(PRED) zyba_3_2_b02230 FRMIGLTLFL IILFFLSNLN RTAGTFFRVY FFLTLSSESI YALFESIAAI
(PRED) zyba_2_1_a00860 FRMIGLTLFL IILFFLSNLN RTAGTFFRVY FFLTLSSESI YALFESIAAI
(PRED) zyro_1_a04114g FRMIGLTLFI IILYFLAGLN RTAASFFRVY WFLAMCSEAI YALFDLIASA
(PRED) zyro_1_b14762g FRLIGLTLFI IILFFLSGLN RTAATFFRIY LFLAMCTECI YALFDFIAAA
(PRED) zyba_2_14_n0149 FRMIGLTVFL IILYFLSNLN RTAGSFFRVY FFLALCAESI YSLFEFLAAA
(PRED) zyba_2_33_ag001 FRMIGLTVFL IILYFLSNLN RTAGSFFRVY FFLALCAESI YSLFEFLAAA
(PRED) lath_1_a01914g FRFLSQTCFY IIIYFLSGLT REANRFFTSF LFLILCSESI NAFFELITAF
(PRED) lawa_1_23_5161 FRFISQTLFF IIIYFLAGLT REANRFFIAF LFLVMCSESV NALFDLITAF
(PRED) klae_1_14_n0012 FRMISVTVFL IILYFLSGLT RSAGRFFTIY LFLSLSSESI NSLFEMISAA
(PRED) klla_1_d03432g FRMISLTAFL IILYFLSGLT RNAGRFFMVY LFVALASESI NALFEFITAA
(PRED) klma_1_1_a01880 FRMISLTFFL IILYFLSGLT REANRFFMCY LFLALASEAI NALFEMITAA
(PRED) klwi_1_33_ag001 FRMISMTCFL IILYFLSNLT RAADRFFMVY LFLALSSEAI TGLFEMITAA
(PRED) teph_1_a04220 FRLIGLTLFL IILYFLSNLR RTPSAFFIIY LFLIIGAESI NCLFQMIGAA
(PRED) vapo_1_1037_47 FRMIGLTMFM VVIYFLAGLR RTASAFFITY LFLTMAAEII NCLFQMIAAA
(PRED) pata_1_2_b05590 IKFLSVVCFG LIVYFLADLK VDAGAFFTYI LFVNLNAQVV SAMFKMLAAF
(PRED) wian_1_3_c04380 FKFLSITILY LITYFLSNLT RDAGKFFLTF LFMVLSSETI TALFQMVAAL
(PRED) wian_1_3_c04390 FKLLSVTAFY LITYFLSNLT RDAGKFFLTY LFLVVSSETI TAMFQMVAAL
(PRED) wian_1_7_g01010 FKLLSVTVFY ILIYFLSNLT RDAGKFFLTY LFLILCSETI TALFQMIAAL
(PRED) bain_1_1_a00100 TKLITIIFFS IIYYFLTNLA VDAAKFFIFL LFIVVSALTM SSFFQMIAEF
(PRED) bain_1_17_q0038 TNLITIIFFA IIYYFLTNLA VDAAKFFIFL LFIVVSTLTM SSFFQMVAEF
(PRED) bain_1_8_h00410 TRVLTITLFT IIYYFLSNLT RSAGHFFIYY LFLLLSSLTM STLFVTIAEA
(PRED) caal_1_19_5759 ISIFINTFFV IILYFLSNLA RDAGKFFICY LFVIMLHLTM KSMFQAIAAI
(PRED) caal_11_25_y002 ISIFINTFFV IILYFLSNLA RDAGKFFICY LFVIMLHLTM KSMFQAIAAI
(PRED) caal_4_4_d03320 ISIFINTFFV IILYFLSNLA RDAGKFFICY LFVIMLHLTM KSMFQAIAAI
(PRED) caal_12_26_z005 ISIFINTFFV IILYFLSNLA RDAGKFFICY LFVIMLHLTM KSMFQAIAAI
(PRED) caal_5_30_ad005 ISIFINTFFV IILYFLSNLA RDAGKFFICY LFVIMLHLTM KSMFQAIAAI
(PRED) caal_8_3_c03320 ISIFINTFFV IILYFLSNLA RDAGKFFICY LFVIMLHLTM KSMFQAIAAI
(PRED) caal_6_4_d03280 ISIFINTFFV IILYFLSNLA RDAGKFFICY LFVIMLHLTM KSMFQAIAAI
(PRED) caal_10_3_c0334 ISIFINTFFV IILYFLSNLA RDAGKFFICY LFVIMLHLTM KSMFQAIAAI
(PRED) caal_3_29_ac005 ISIFINTFFV IILYFLSNLA RDAGKFFICY LFVIMLHLTM KSMFQAIAAI
(PRED) caal_2_04989 ISIFINTFFV IILYFLSNLA RDAGKFFICY LFVIMLHLTM KSMFQAIAAI
(PRED) cadu_1_64350 ISIFINTFFV IILYFLSNLA RDAGKFFICY LFVIMLHLTM KSMFQAIAAI
(PRED) caor_1_h02090 ISVLINIFFV IILYFLSNLA REPGKFFIAF LFVVLLHLTM GALFKAVASI
(PRED) capa_1_600750 ISIIVNVLFV IILYFLSNLA REAGKFFIAF LFIVLLHLTM GALFKAVAAI
(PRED) loel_1_04930 ISILINIFFV IILYFLSNLA REAGKFFICF LFVVLLHMTM GSLFQAVAAI
(PRED) spar_1_5_e03260 LSILINTLFV IVLYFLSNLA SDAGKFFTAY LFIIMLHLTM GCLFQAVASV
(PRED) sppa_1_7_g03160 LSILINTMFV IILYFLSNLA RDAGKFFIAY LFIIMLHLTM GSFFQAIASL
(PRED) catr_1_01205 ISLIVNVFFV IILYFLSNLA RDAGKFFICY LFVVLLHLTM GSMFQAIAAI
(PRED) catr_1_05498 ISLFVNALFV VILYFLSNLA VDAGKFFTCY LFVFMLHLTM GAMFQAVAAL
(PRED) catr_1_05971 ISLFINVFFV IILYFLSNLA RDAGKFFICY LFVVLLHLTM GSMFQAVAAI
(PRED) deha_1_a03696g VTLVVTFLFV LIIYFLSNLA ADAGKFFTCV LFVFLLSLTM SGLFEAVASL
(PRED) deha_2_5_e00720 VTLVVTFLFV LIIYFLSNLA ADAGKFFTCV LFVFLLSLTM AGLFEAVASL
(PRED) scst_1_3_c02890 VAFLVSFFFL IILYFLSNLA REAGKFFTAL LFVFLLQLTM SALFQAVASL
(PRED) mebi_1_8_h00300 VSVLTNIAFS LILYFLANLK REAGKFFTFV LFTNLVSLAM NYLFKATAAW
(PRED) lakl_1_h21010g FRLIGLTVFF IIIYFMSNMN RQPGKFFLNY LFLALGAETI NALFQMITAF
(PRED) caar_1_13_m0142 VRMFAILLFA LILYFLSDLK REAGAFFTFY LFINMTVICI NAYITLAASL
(PRED) caar_1_14_n0143 VRVFAILIFS LILYFLSNLK RQAGPFFAFV LFINVTVIAI NALFILIASF
(PRED) hapo_1_1_a07220 VRAVAIVVFS LILYFLSNMK REAGPFFAFI LFVNVAVLAV NCLFILIASL
(PRED) ogpa_1_1_a01680 VRAVAIVVFS LILYFLSNMK REAGPFFAFI LFINVAVLAV NCLFILIASL
(PRED) piku_1_96_cr001 VRLIAITSFS LILYFLTNMK RQPGAYFIYF LFINLVVPTI TSLFTLLASL
(PRED) pime_1_4_d03240 VRCTAIVCFS LIIYFLSNLK REAGAFFSYF LFVNLVVQAV NSLFTLLASF
(PRED) pime_1_1_a12110 IRSISIVVFG IVIYFLSGLK TDAGAFFTFL LFIQLCVQAI SSLFTLLASI
(PRED) piku_1_227_hs00 IRLIAIVLFS IIMYFLSNMK REPGPFFQFL LMINLVVLAV SSLFTLISSF
(PRED) pime_1_5_e05800 VRLFAIVIFT IIMYFLSNMK REPGPFFQFM LMTNLVVQAV SGLFTLFSSF
(PRED) pime_1_1_a07690 VRLIAITCFA IIIYFLTHLK TEAGAFFHYY LFINLVVQAV SGLFVMLASL
(PRED) debr_2_5_e03380 IRFFTILAFS LILYFLSNLK RQPGAFFFFL LFVNVAVEAV ASLFTLVASC
(PRED) kopa_1_2_b10040 VKFIASLFFS IVVYFLANMK RQPGPFFAFL LFVNLGSQTM AALFNLVAAV
(PRED) kopa_2_7_g00500 VKFIASSFFS IVVYFLANMK RQPGPFFAFL LFVNLGSQTM AALFNLVAAV
(PRED) asru_1_13_m0119 TKLLSLFSFS IVVYFMVNFD REAGKFVFV LFLFMTSSIM TCLFQSIAAF
(PRED) asru_1_15_o0045 LRLTLTVILS LIVYFLSNLE RKAGKFFIFF LFLFVTYCVL YSLFLAFGVY
(PRED) wian_1_1_a02920 VKVVMITIFM IISYFLAGMK YEAGAFFSFW LFFILTVLAF KVIFNFIATI
(PRED) wian_1_1_a02930 LKMVTIIVFT IISYFLAGLK SDAGAFFNFL LFFLLTVFIA KVMFNVVATI

.........910 ....920 ....930 ....940 950
(PRED) asac_1_6_f03560 SNSLSQANAV AGLVMFGISI YSTYMIQKPS MHPWFAWISY ILPIRYAFEN
(PRED) ergo_1_abr125c STSLSQANAV AGLVMFGISI YSTYMIQKPS MHPWFAWISY ILPIRYAFES
(PRED) ercy_1_3604 SGSIAQANAI SGIVMLAISM YSTYIIQPPS MHPWFKWLSY ILPIRYAFES
(PRED) cagl_1_i04862g CDNISQANSI SGIVMMSISL YSTYMIQLPS MRPWFKWISY ILPIRYAFES
(PRED) kaaf_1_c00830 CDNISQANSL AGILMMSISM YSTYMIQLPS MHPWFKWISY ILPIRYAFEA
(PRED) kana_1_k01350 CDNIAQANSI SGIIMLSISM YSTYMIQLPS MRPWFKWISY ILPIRYAFES

(PRED) saar_1_2_b02590 CDTLAQANSI SGILMLSISM YSTYMIQLPS MHPWFKWISY VLPIRYAFEA
(PRED) sace_1_ydr011w CDTLSQANSI SGILMMSISM YSTYMIQLPS MHPWFKWISY VLPIRYAFES
(PRED) sace_16_1_a0238 CDTLSQANSI SGILMMSISM YSTYMIQLPS MHPWFKWISY VLPIRYAFES
(PRED) sace_45_1_a0242 CDTLSQANSI SGILMMSISM YSTYMIQLPS MHPWFKWISY VLPIRYAFES
(PRED) sace_48_1_a0238 CDTLSQANSI SGILMMSISM YSTYMIQLPS MHPWFKWISY VLPIRYAFES
(PRED) sace_60_4_d0244 CDTLSQANSI SGILMMSISM YSTYMIQLPS MHPWFKWISY VLPIRYAFES
(PRED) sace_52_1_a0240 CDTLSQANSI SGILMMSISM YSTYMIQLPS MHPWFKWISY VLPIRYAFES
(PRED) sace_46_1_a0240 CDTLSQANSI SGILMMSISM YSTYMIQLPS MHPWFKWISY VLPIRYAFES
(PRED) sace_25_1_a0240 CDTLSQANSI SGILMMSISM YSTYMIQLPS MHPWFKWISY VLPIRYAFES
(PRED) sace_24_1_2300 CDTLSQANSI SGILMMSISM YSTYMIQLPS MHPWFKWISY VLPIRYAFES
(PRED) sace_47_1_a0240 CDTLSQANSI SGILMMSISM YSTYMIQLPS MHPWFKWISY VLPIRYAFES
(PRED) sace_7_1_a02410 CDTLSQANSI SGILMMSISM YSTYMIQLPS MHPWFKWISY VLPIRYAFES
(PRED) sace_59_110_df0 CDTLSQANSI SGILMMSISM YSTYMIQLPS MHPWFKWISY VLPIRYAFES
(PRED) sace_56_1_a0202 CDTLSQANSI SGILMMSISM YSTYMIQLPS MHPWFKWISY VLPIRYAFES
(PRED) sace_40_1_a0239 CDTLSQANSI SGILMMSISM YSTYMIQLPS MHPWFKWISY VLPIRYAFES
(PRED) sace_15_1_a0242 CDTLSQANSI SGILMMSISM YSTYMIQLPS MHPWFKWISY VLPIRYAFES
(PRED) sace_37_1_a0243 CDTLSQANSI SGILMMSISM YSTYMIQLPS MHPWFKWISY VLPIRYAFES
(PRED) sace_9_1_a02440 CDTLSQANSI SGILMMSISM YSTYMIQLPS MHPWFKWISY VLPIRYAFES
(PRED) sace_22_1_2300 CDTLSQANSI SGILMMSISM YSTYMIQLPS MHPWFKWISY VLPIRYAFES
(PRED) sace_29_1_2290 CDTLSQANSI SGILMMSISM YSTYMIQLPS MHPWFKWISY VLPIRYAFES
(PRED) sace_34_1_2320 CDTLSQANSI SGILMMSISM YSTYMIQLPS MHPWFKWISY VLPIRYAFES
(PRED) sace_58_25_y007 CDTLSQANSI SGILMMSISM YSTYMIQLPS MHPWFKWISY VLPIRYAFES
(PRED) sace_23_1_2290 CDTLSQANSI SGILMMSISM YSTYMIQLPS MHPWFKWISY VLPIRYAFES
(PRED) sace_6_120_dp00 CDTLSQANSI SGILMMSISM YSTYMIQLPS MHPWFKWISY VLPIRYAFES
(PRED) sace_57_1_a0241 CDTLSQANSI SGILMMSISM YSTYMIQLPS MHPWFKWISY VLPIRYAFES
(PRED) sace_17_1_a0241 CDTLSQANSI SGILMMSISM YSTYMIQLPS MHPWFKWISY VLPIRYAFES
(PRED) sace_21_1_2310 CDTLSQANSI SGILMMSISM YSTYMIQLPS MHPWFKWISY VLPIRYAFES
(PRED) sace_49_1_a0246 CDTLSQANSI SGILMMSISM YSTYMIQLPS MHPWXKWISY VLPIRYAFES
(PRED) sace_8_2_b02430 CDTLSQANSI SGILMMSISM YSTYMIQLPS MHPWXKWISY VLPIRYAFES
(PRED) sace_31_1_2300 CDTLSQANSI SGILMMSISM YSTYMIQLPS MHPWFKWISY VLPIRYAFES
(PRED) sace_50_1_a0241 CDTLSQANSI SGILMMSISM YSTYMIQLXS MHPWFKWISY VLPIRYAFES
(PRED) sace_4_1_a02360 CDTLSQANSI SGILMMSISM YSTYMIQLPS MHPWFKWISY VLPIRYAFES
(PRED) sace_2_1_a02390 CDTLSQANSI SGILMMSISM YSTYMIQLPS MHPWFKWISY VLPIRYAFES
(PRED) sace_5_33_ag005 CDTLSQANSI SGILMMSISM YSTYMIQLPS MHPWFKWISY VLPIRYAFES
(PRED) sapa_11_1_a0247 CDTLSQANSI SGILMMSISM YSTYMIQLPS MHPWFKWISY VLPIRYAFES
(PRED) sapa_25_1_a0246 CDTLSQANSI SGILMMSISM YSTYMIQLPS MHPWFKWISY VLPIRYAFES
(PRED) sapa_4_1_a02470 CDTLSQANSI SGILMMSISM YSTYMIQLPS MHPWFKWISY VLPIRYAFES
(PRED) sapa_5_1_2350 CDTLSQANSI SGILMMSISM YSTYMIQLPS MHPWFKWISY VLPIRYAFES
(PRED) sapa_9_1_2360 CDTLSQANSI SGILMMSISM YSTYMIQLPS MHPWFKWISY VLPIRYAFES
(PRED) sapa_14_1_a0244 CDTLSQANSI SGILMMSISM YSTYMIQLPS MHPWFKWISY VLPIRYAFES
(PRED) sapa_8_1_2350 CDTLSQANSI SGILMMSISM YSTYMIQLPS MHPWFKWISY VLPIRYAFES
(PRED) sapa_17_1_2380 CDTLSQANSI SGILMMSISM YSTYMIQLPS MHPWFKWISY VLPIRYAFES
(PRED) sapa_7_1_2370 CDTLSQANSI SGILMMSISM YSTYMIQLPS MHPWFKWISY VLPIRYAFES
(PRED) sapa_2_1_a02460 CDTLSQANSI SGILMMSISM YSTYMIQLPS MHPWFKWISY VLPIRYAFES
(PRED) sapa_23_1_a0248 CDTLSQANSI SGILMMSISM YSTYMIQLPS MHPWFKWISY VLPIRYAFES
(PRED) sapa_3_1_a02470 CDTLSQANSI SGILMMSISM YSTYMIQLPS MHPWFKWISY VLPIRYAFES
(PRED) sapa_18_1_2390 CDTLSQANSI SGILMMSISM YSTYMIQLPS MHPWFKWISY VLPIRYAFES
(PRED) sami_1_4_244 CDTLSQANSI SGILMMSISM YSTYMIQLPS MHPWFKWISY VLPIRYAFES
(PRED) saku_1_4_262 CDTLSQANSI SGILMMSISM YSTYMIQLPS MHPWFKWISY VLPIRYAFES
(PRED) saba_1_58_bf002 CDSLSQANSI SGILMMSISM YSTYMIQLPS MHPWFKWISY VLPIRYAFES
(PRED) saeu_1_4_d02400 CDTLSQANSI SGILMMSISM YSTYMIQLPS MHPWFKWISY VLPIRYAFES
(PRED) naca_1_e01640 CDTLAQANGI NGILMLSISM YSTYMIQLPS MHPWFKWISY ILPIRYAFEA
(PRED) nada_1_g01850 CDTLAQANAI NGVLMLAISL YSTYMIQLPS MHPWFQWISY VLPIRYGFES
(PRED) naca_1_e01630 CDTLAQANSI AGILMLCISL YSSYMIQLPS MHPWFKWISY IIPIRYAFES
(PRED) nada_1_g01840 CDTLAQANSI TGVFMLAISL YSTYMIQLPS MHPWFQWMSY VIPIRYAFES
(PRED) kaaf_1_c00820 CDTMAQANSI AGILMMSISM YSTYMIQLPS MHPWFKWISY VLPIRYAFES
(PRED) teph_1_m00640 CDNLAQANSI AGILMMAISM YSTYMIQLPA MHPWFKWISY VLPIRYAFES
(PRED) vapo_1_1036_28 CDNIAQANSI CGILMLAISM YSTYMIQLPS MHPWFKWISY ILPIRYSFES
(PRED) tebl_1_i01760 CDSLPQANSI AGIIMLAISL YSTYMIQAPK MHQWFKWISY ILPIRYAFES
(PRED) tode_1_d04040 TDTLAQANAI AGVLMMSISM YSTYMIQLPS IHPWFIWVAY ILPIRYSFES
(PRED) naca_1_e01650 VIVLAQANFI CGILTMAISL YSTYMIQLHS MRPWFKWISY ILPIRYCFES
(PRED) tebl_1_g02820 CDDIAQANSI AGILMMALSM YSTYMIQLPK MHPWFKWIAY ILPIQYSFES
(PRED) lakl_1_c11616g SDSISQANSI SGILMMAISL YSTYMIQLPS MHPWFKWIAY ILPIRYTFES
(PRED) saar_1_8_h03780 CDTLSQAHSI AGVVMLSIAM YSTYMIQLPS MHPWFKWISY ILPIRYAFES
(PRED) sace_14_7_g0015 CDTLSQANSI AGVVMLSIAM YSTYMIQLPS MHPWFKWISY ILPIRYAFES
(PRED) sace_15_7_g0387 CDTLSQANSI AGVVMLSIAM YSTYMIQLPS MHPWFKWISY ILPIRYAFES
(PRED) sace_24_8_3780 CDTLSQANSI AGVVMLSIAM YSTYMIQLPS MHPWFKWISY ILPIRYAFES
(PRED) sace_40_8_h0383 CDTLSQANSI AGVVMLSIAM YSTYMIQLPS MHPWFKWISY ILPIRYAFES
(PRED) sace_6_169_fm00 CDTLSQANSI AGVVMLSIAM YSTYMIQLPS MHPWFKWISY ILPIRYAFES
(PRED) sace_19_7_3840 CDTLSQANSI AGVVMLSIAM HSTYMIQLPS MHPWFKWISY ILPIRYAFES
(PRED) sace_32_7_3770 CDTLSQANSI AGVVMLSIAM YSTYMIQLPS MHPWFKWISY ILPIRYAFES
(PRED) sace_56_17_q011 CDTLSQANSI AGVVMLSIAM YSTYMIQLPS MHPWFKWISY ILPIRYAFES
(PRED) sace_5_78_bz001 CDTLSQANSI AGVVMLSIAM XSTYMIQLPS MHPWFXWISY ILPIRYAFES
(PRED) sace_2_8_h03860 CDTLSQANSI AGVVMLSIAM HSTYMIQLPS MHPWFKWISY ILPIRYAFES
(PRED) sace_53_29_ac00 CDTLSQANSI AGVVMLSIAM YSTYMIQLPS MHPWFKWISY ILPIRYAFES

```
(PRED) sace_17_7_g0393 CDTLSQANSI AGVVMLSIAM YSTYMIQLPS MHPWFKWISY ILPIRYAFES
(PRED) sace_25_7_g0388 CDTLSQANSI AGVVMLSIAM YSTYMIQLPS MHPWFKWISY ILPIRYAFES
(PRED) sace_37_7_g0385 CDTLSQANSI AGVVMLSIAM YSTYMIQLPS MHPWFKWISY ILPIRYAFES
(PRED) sace_9_7_g00180 CDTLSQANSI AGVVMLSIAM YSTYMIQLPS MHPWFKWISY ILPIRYAFES
(PRED) sace_60_6_f0335 CDTLSQANSI AGVVMLSIAM YSTYMIQLPS MHPWFKWISY ILPIRYAFES
(PRED) sace_59_336_1x0 CDTLSQANSI AGVVMLSIAM YSTYMIQLPS MHPWFKWISY ILPIRYAFES
(PRED) sace_31_7_3780  CDTLSQANSI AGVVMLSIAM YSTYMIQLPS MHPWFKWISY ILPIRYAFES
(PRED) sace_34_8_3770  CDTLSQANSI AGVVMLSIAM YSTYMIQLPS MHPWFKWISY ILPIRYAFES
(PRED) sace_58_71_bs00 CDTLSQANSI AGVVMLSIAM YSTYMIQLPS MHPWFKWISY ILPIRYAFES
(PRED) sace_7_7_g03880 CDTLSQANSI AGVVMLSIAM YSTYMIQLPS MHPWFKWISY ILPIRYAFES
(PRED) sace_35_7_3840  CDTLSQANSI AGVVMLSIAM YSTYMIQLPS MHPWFKWISY ILPIRYAFES
(PRED) sace_43_7_g0387 CDTLSQANSI AGVVMLSIAM YSTYMIQLPS MHPWFKWISY ILPIRYAFES
(PRED) sace_57_8_h0390 CDTLSQANSI AGVVMLSIAM YSTYMIQLPS MHPWFKWISY ILPIRYAFES
(PRED) sace_45_7_g0389 CDTLSQANSI AGVVMLSIAM YSTYMIQLPS MHPWFKWISY ILPIRYAFES
(PRED) sace_46_8_h0391 CDTLSQANSI AGVVMLSIAM YSTYMIQLPS MHPWFKWISY ILPIRYAFES
(PRED) sace_23_7_3860  CDTLSQANSI AGVVMLSIAM YSTYMIQLPS MHPWFKWISY ILPIRYAFES
(PRED) sace_21_7_3790  CDTLSQANSI AGVVMLSIAM YSTYMIQLPS MHPWFKWISY ILPIRYAFES
(PRED) sace_8_73_bu001 CDTLSQANSI AGVVMLSIAM YSTYMIQLPS MHPWFKWISY ILPIRYAFES
(PRED) sapa_1_8_h03820 CDTLSQANSI AGVVMLSIAM YSTYMIQLPS MHPWFKWISY ILPIRYAFES
(PRED) sapa_21_8_h0387 CDTLSQANSI AGVVMLSIAM YSTYMIQLPS MHPWFKWISY ILPIRYAFES
(PRED) sapa_20_8_h0386 CDTLSQANSI AGVVMLSIAM YSTYMIQLPS MHPWFKWISY ILPIRYAFES
(PRED) sapa_22_8_h0390 CDTLSQANSI AGVVMLSIAM YSTYMIQLPS MHPWFKWISY ILPIRYAFES
(PRED) sapa_25_8_h0387 CDTLSQANSI AGVVMLSIAM YSTYMIQLPS MHPWFKWISY ILPIRYAFES
(PRED) sapa_6_8_3750   CDTLSQANSI AGVVMLSIAM YSTYMIQLPS MHPWFKWISY ILPIRYAFES
(PRED) sapa_9_8_3720   CDTLSQANSI AGVVMLSIAM YSTYMIQLPS MHPWFKWISY ILPIRYAFES
(PRED) sapa_19_8_h0390 CDTLSQANSI AGVVMLSIAM YSTYMIQLPS MHPWFKWISY ILPIRYAFES
(PRED) sapa_24_8_h0385 CDTLSQANSI AGVVMLSIAM YSTYMIQLPS MHPWFKWISY ILPIRYAFES
(PRED) sapa_4_8_h03850 CDTLSQANSI AGVVMLSIAM YSTYMIQLPS MHPWFKWISY ILPIRYAFES
(PRED) sapa_10_8_3760  CDTLSQANSI AGVVMLSIAM YSTYMIQLPS MHPWFKWISY ILPIRYAFES
(PRED) sapa_13_8_h0382 CDTLSQANSI AGVVMLSIAM YSTYMIQLPS MHPWFKWISY ILPIRYAFES
(PRED) sapa_8_8_3750   CDTLSQANSI AGVVMLSIAM YSTYMIQLPS MHPWFKWISY ILPIRYAFES
(PRED) sapa_11_8_h0383 CDTLSQANSI AGVVMLSIAM YSTYMIQLPS MHPWFKWISY ILPIRYAFES
(PRED) sapa_5_8_3700   CDTLSQANSI AGVVMLSIAM YSTYMIQLPS MHPWFKWISY ILPIRYAFES
(PRED) sapa_16_8_h0389 CDTLSQANSI AGVVMLSIAM YSTYMIQLPS MHPWFKWISY ILPIRYAFES
(PRED) sapa_17_8_3730  CDTLSQANSI AGVVMLSIAM YSTYMIQLPS MHPWFKWISY ILPIRYAFES
(PRED) sapa_2_8_h03860 CDTLSQANSI AGVVMLSIAM YSTYMIQLPS MHPWFKWISY ILPIRYAFES
(PRED) sapa_7_8_3740   CDTLSQANSI AGVVMLSIAM YSTYMIQLPS MHPWFKWISY ILPIRYAFES
(PRED) sapa_23_8_h0385 CDTLSQANSI AGVVMLSIAM YSTYMIQLPS MHPWFKWISY ILPIRYAFES
(PRED) sapa_3_8_h03890 CDTLSQANSI AGVVMLSIAM YSTYMIQLPS MHPWFKWISY ILPIRYAFES
(PRED) sapa_18_8_3730  CDTLSQANSI AGVVMLSIAM YSTYMIQLPS MHPWFKWISY ILPIRYAFES
(PRED) sami_1_14_399   CDTLSQANSI AGVVMLSIAM YSTYMVQLPS MHPWFKWISY ILPIRYAFES
(PRED) sace_4_8_h03690 CDTLSQANSI AGVVMLSIAM YSTYMIQLPS MHPWFKWISY ILPIRYAFES
(PRED) saku_1_14_404   CDTLSQANSI AGVVMLSIAM YSTYMIQLPS MHPWFKWISY ILPIRYAFES
(PRED) sace_1_ynr070w  CDTLSQANSI AGVVMLSIAM YSTYMIQLPS MHPWFKWISY ILPIRYAFES
(PRED) sace_49_8_h0383 CDTLSQANSI AGVVMLSIAM YSTYMIQLPS MHPWFKWISY ILPIRYAFES
(PRED) saeu_1_2_b00130 CDSLAQANSI AGILMLSIAM YSTYMIQLPS MHPWFKWISY VLPIRYAFES
(PRED) sauv_1_7_3      CDSLAQANSI AGILMLSIAM YSTYMIQLPS MHPWFKWISY VLPIRYAFES
(PRED) sami_1_17_26    CDTLSQANSI AGVLMLAISM YSTYMIQLPS MHPWFKWISY ILPIRYAFES
(PRED) zyba_1_02055_AN CDNISQANSI AGIVMFSISM YSTYMIQYPN MHPWFQWISY VLPIRYAFEG
(PRED) zyba_1_07912    CDNISQANSI AGIVMFSISM YSTYMIQYAN MHPWFQWISY VLPIRYAFEG
(PRED) zyba_2_2_b00600 CDNISQANSI AGIVMFSISM YSTYMIQYPN MHPWFQWISY VLPIRYAFEG
(PRED) zyba_3_3_c03460 CDNISQANSI AGIVMFSISM YSTYMIQYPN MHPWFQWISY VLPVRYAFEG
(PRED) zyba_1_04634    CDNMSQATPI AGIVMFSISM YSTYVIQEPN MHPWFRWISF VLPIRYAFEG
(PRED) zyba_1_06675    CDNMSQATPI AGIVMFSISM YSTYVIQEPN MHPWFRWISY VLPVRYAFEG
(PRED) zyba_3_2_b02230 CDNMSQATPI AGIVMFSISM YSTYVIQEPN MHPWFRWISY VLPVRYAFEG
(PRED) zyba_2_1_a00860 CDNMSQATPI AGIVMFSISM YSTYVIQEPN MHPWFRWISY VLPVRYAFEG
(PRED) zyro_1_a04114g  CDDISQANSI AGLVMFAISM YSTYMIQLPN MHPWFKWISY VLPIRYAFEG
(PRED) zyro_1_b14762g  CDDIAQASSI SGLIMFSLSM YSTYMIQLPN MHPWFKWISY VLPIRYAFEA
(PRED) zyba_2_14_n0149 CDDMSQANSV AGIVMLAISM YSSYMIQLPN MHPWFKWISY VLPIRYAFEG
(PRED) zyba_2_33_ag001 CDDMSQANSV SGIVMLAISM YSSYMIQLPN MHPWFQWISY VLPIRYAFEG
(PRED) lath_1_a01914g  SSSVAQANSI AGTSLMALVL YSTYMIQLES MHPWFKWISY CIPIRYTFES
(PRED) lawa_1_23_5161  SSSVAQANSI AGTVLMALIL YSTYMIQLGS MHPWFKWISY VQPLRYSFES
(PRED) klae_1_14_n0012 CDSVSQANAI SGILMMALSL YSTYMIQPPS MHPWFKWISY VLPLRYAFES
(PRED) klla_1_d03432g  CDSISQANAI AGLVMMALSL YSTYMIQTPS MHPWFEWISY ILPLRYAFEN
(PRED) klma_1_1_a01880 CDSLSQANAI AGLVMMALSL YSTYIIQTPS MHPWFKWISY ILPLRFAFEN
(PRED) klwi_1_33_ag001 CDSLSQANAI AGLVMMALSL YSTYMLQAPS MHPWFKWISY ILPLRYSFES
(PRED) teph_1_a04220   CNTLAQANAI NGILMLSLSM YSTYMIQLPQ IKPWFIWIAY ILPLRYAFEA
(PRED) vapo_1_1037_47  CETINEANSI NGILMLSLSM YSTYMIQLPE MRVYFKWISF ALPLRYGFES
(PRED) pata_1_2_b05590 CSTISAANAV SGVMMLSTIL YSSYMIQRPS MVPWFKWFSY MNPVLFGFEA
(PRED) wian_1_3_c04380 SQNVAGANAV AGVLMLAISL YSCYVIQLKS MHPWFKWISY INPIRYGFEN
(PRED) wian_1_3_c04390 SQNVAGANAV AGILMLAISL YSCYMIQLKS MHPWFKWISY INPIRYGFEN
(PRED) wian_1_7_g01010 SQNVAGANAI AGILMLALSL YSCYMIQLNS MRPWFKWISY INPIRYGFEN
(PRED) bain_1_1_a00100 NQSFAGANAV AGCSILMTLM YASFMIQRPS MHPWFKWISY INPLFYAFEA
(PRED) bain_1_17_q0038 NQTFAGANAV AGCSILMTLM YTSFMIQKPS MHPWFKWITY INPLFYAFES
(PRED) bain_1_8_h00410 SPNIAVANSG AGITLLCVLM YSSFSIQRTY MHPWFKWISY INPIFYSFEA
```

Results colour-coded for transmembrane structure

```
(PRED) caal_1_19_5759  NKSIAGANAM GGILMLASLM YSSYMIQRPS MHPWFKWISY INPVLYAFEA
(PRED) caal_11_25_y002 NKSIAGANAM GGILMLASLM YSSYMIQRPS MHPWFKWISY INPVLYAFEA
(PRED) caal_4_4_d03320 NKSIAGANAM GGILMLASLM YSSYMIQRPS MHPWFKWISY INPVLYAFEA
(PRED) caal_12_26_z005 NKSIAGANAM GGILMLASLM YSSYMIQRPS MHPWFKWISY INPVLYAFEA
(PRED) caal_5_30_ad005 NKSIAGANAM GGILMLASLM YSSYMIQRPS MHPWFKWISY INPVLYAFEA
(PRED) caal_8_3_c03320 NKSIAGANAM GGILMLASLM YSSYMIQRPS MHPWFKWISY INPVLYAFEA
(PRED) caal_6_4_d03280 NKSIAGANAM GGILMLASLM YSSYMIQRPS MHPWFKWISY INPVLYAFEA
(PRED) caal_10_3_c0334 NKSIAGANAM GGILMLASLM YSSYMIQRPS MHPWFKWISY INPVLYAFEA
(PRED) caal_3_29_ac005 NKSIAGANAM GGILMLASLM YSSYMIQRPS MHPWFKWISY INPVLYAFEA
(PRED) caal_2_04989    NKSIAGANAM GGILMLASLM YSSYMIQRPS MHPWFKWISY INPVLYAFEA
(PRED) cadu_1_64350    NKSIAGANAM GGISVLASLM YSSYMIQRPS MHPWFKWISY INPVLYAFEA
(PRED) caor_1_h02090   NKTVAAANAL GGVLMMASLM YSSYMIQRPS MHPWFKWISY INPVLYAFEA
(PRED) capa_1_600750   NKTVAAANAL GGVLMMASLM YSSYLIQRPS MHPWFKWISY INPVLYAFEA
(PRED) loel_1_04930    NKSVAGANAL GGVLVLASLM YSSYMIQRPS MHPWFEWISY INPVLYAFEA
(PRED) spar_1_5_e03260 NKTISAANAF AGVLVLASLM YSSFMIQRPS MHPWFKWISY INPVLYAFEA
(PRED) sppa_1_7_g03160 NKTISAANAF AGVMVLASLM YSSFMIQRPS MHPWFKWISY INPVLYAFEA
(PRED) catr_1_01205    HKTIAGANAL GGIFMLASLM YSSYMIQRPS MHGYSRWISY INPVLYAFEA
(PRED) catr_1_05498    HKTIAGANAV GGILVLATLS YSSYMIQRPT MHGYSRWISY INPVLYAFEA
(PRED) catr_1_05971    HKTIAGANAI GGILVLASLM YSSYMIQRPS MHGYSRWISY INPVLYAFEA
(PRED) deha_1_a03696g  NKTISGANAI AGVLVLASLM YSSYMIQRPS MHPWFKWISY INPVLYAFEA
(PRED) deha_2_5_e00720 NKTISGANAI AGVLVLASLM YSSYMIQRPS MHPWFKWISY INPVLYAFEA
(PRED) scst_1_3_c02890 NKTISSANAF AGVLVLASLM YSSYMIQRPS MHPWFKWISY INPVLYAFEA
(PRED) mebi_1_8_h00300 NKSIAGANAI GGVFILASLM YSSYMIQRPS MHPWFKWISY INPVLYAFEA
(PRED) lakl_1_h21010g  TANLAQTNAL NGLFLLAMSM YSTYMIQLES MHPWFKWINY LSPLRWMLEN
(PRED) caar_1_13_m0142 CSSVNSANAV AGILYASS-- -SSFMIQRPS MHWWFKWYSY MNPILYGFEA
(PRED) caar_1_14_n0143 CATLSSANGV TGIYRY---V XSSFMIQRPS MYWWFKWFSY INPVLYGFES
(PRED) hapo_1_1_a07220 SPTLSAANGF VGIIMMSTIL YSSYMIQRPS MYWWFKWFSY INPILYGFEA
(PRED) ogpa_1_1_a01680 SPTLSAANGF VGIIMMSTIL YSSYMIQRPS MYWWFKWFSY MNPVLYGFEA
(PRED) piku_1_96_cr001 MPNVAAANGI SGLTMMSMVV YSSYMIQRPN MHWWFKWFSY CNPVLYAFEA
(PRED) pime_1_4_d03240 MPDLSAANGI NGICMMAMIV YSSYMIQRPS MYWWFKWFSY CNPVLFAFES
(PRED) pime_1_1_a12110 MPTLGAANGI NGVVMMACIL YSSYMIQSPS MYWWFEWFTY CNPIRFAFES
(PRED) piku_1_227_hs00 MPTLAAAMAI CGVVTLGLAL YSSYMIQLNS MYWWFKWFAY TNPILYAFEA
(PRED) pime_1_5_e05800 MPNLSSAMAI CGVILLGLGV YSSYMIQMNS MYWWFKWFAY TNPILYAFES
(PRED) pime_1_1_a07690 MPNASAAMGI CGISMISMVI YSSYMIQRPS MYWWFKWFSY CNPVLYAFES
(PRED) debr_2_5_e03380 CPTLTSANAL TGILMMLMIL YSSFMIQRPS MYWWFRWFSY ANPVLYGFES
(PRED) kopa_1_2_b10040 SPTLAVANAF DGLLVLSSVL YTSYMIQRPS MVPWFEWFSY MNPMLYAFES
(PRED) kopa_2_7_g00500 SPTLAVANAF DGLLVLSSVL YTSYMIQRPS MVPWFEWFSY MNPMLYAFES
(PRED) asru_1_13_m0119 SPTIAAANSF SGVGMLAVIV YTGFVIKMHD MHWALKWISY INPVTYGFES
(PRED) asru_1_15_o0045 CANISTAKSV TGLALVVILV YTGYVIQRPA MHWHFKWLSY INPVTYSFEA
(PRED) wian_1_1_a02920 SPTNALANTI GGIVLMAISI YSSFMIQFPS MHPWFSWIFY INPFTYGFES
(PRED) wian_1_1_a02930 SPTASLANSV GGVLLLAIAL YSSFMIQIPS MHPWFVWIAY INPAMYGFES

                       .........960 .........970 .........980 .........990 .........1000
(PRED) asac_1_6_f03560 MLNAEFHGRH MDCA-DTYVP AGPGYEGVAP -ENRVCAFIG SK-------P
(PRED) ergo_1_abr125c  MLNAEFHGRH MDCA-DTYVP TGPGYEGVTP -ENRVCAFIG SK-------P
(PRED) ercy_1_3604     MLNGEFHGRK MSCT-ESYVP SGPSYQGISD -QNKVCAFVG SK-------P
(PRED) cagl_1_i04862g  MLLAEFHGRH MGCG-GTLVP SGPGYENIAS -ENQVCAFVG SK-------P
(PRED) kaaf_1_c00830   MLEAEFHGRH MECT--TLVP TGPTYANVSS -SNRVCAFTG SQ-------F
(PRED) kana_1_k01350   MLNAEFHGRH MDCG-GTLVP TGPGYENVAE -ENRVCAFVG SK-------P
(PRED) saar_1_2_b02590 MLNAEFHGRH MDCA-NSLVP SGGNYDNLSD -DYKVCAFVG SK-------P
(PRED) sace_1_ydr011w  MLNAEFHGRH MDCA-NTLVP SGGDYDNLSD -DYKVCAFVG SK-------P
(PRED) sace_16_1_a0238 MLNAEFHGRH MDCA-NTLVP SGGDYDNLSD -DYKVCAFVG SK-------P
(PRED) sace_45_1_a0242 MLNAEFHGRH MDCA-NTLVP SGGDYDNLSD -DYKVCAFVG SK-------P
(PRED) sace_48_1_a0238 MLNAEFHGRH MDCA-NTLVP SGGDYDNLSD -DYKVCAFVG SK-------P
(PRED) sace_60_4_d0244 MLNAEFHGRH MDCA-NTLVP SGGDYDNLSD -DYKVCAFVG SK-------P
(PRED) sace_52_1_a0240 MLNAEFHGRH MDCA-NTLVP SGGDYDNLSD -DYKVCAFVG SK-------P
(PRED) sace_46_1_a0240 MLNAEFHGRH MDCA-NTLVP SGGDYDNLSD -DYKVCAFVG SK-------P
(PRED) sace_25_1_a0240 MLNAEFHGRH MDCA-NTLVP SGGDYDNLSD -DYKVCAFVG SK-------P
(PRED) sace_24_1_2300  MLNAEFHGRH MDCA-NTLVP SGGDYDNLSD -DYKVCAFVG SK-------P
(PRED) sace_47_1_a0240 MLNAEFHGRH MDCA-NTLVP SGGDYDNLSD -DYKVCAFVG SK-------P
(PRED) sace_7_1_a02410 MLNAEFHGRH MDCA-NTLVP SGGDYDNLSD -DYKVCAFVG SK-------P
(PRED) sace_59_110_df0 MLNAEFHGRH MDCA-NTLVP SGGDYDNLSD -DYKVCAFVG SK-------P
(PRED) sace_56_1_a0202 MLNAEFHGRH MDCA-NTLVP SGGDYDNLSD -DYKVCAFVG SK-------P
(PRED) sace_40_1_a0239 MLNAEFHGRH MDCA-NTLVP SGGDYDNLSD -DYKVCAFVG SK-------P
(PRED) sace_15_1_a0242 MLNAEFHGRH MDCA-NTLVP SGGDYDNLSD -DYKVCAFVG SK-------P
(PRED) sace_37_1_a0243 MLNAEFHGRH MDCA-NTLVP SGGDYDNLSD -DYKVCAFVG SK-------P
(PRED) sace_9_1_a02440 MLNAEFHGRH MDCA-NTLVP SGGDYDNLSD -DYKVCAFVG SK-------P
(PRED) sace_22_1_2300  MLNAEFHGRH MDCA-NTLVP SGGDYDNLSD -DYKVCAFVG SK-------P
(PRED) sace_29_1_2290  MLNAEFHGRH MDCA-NTLVP SGGDYDNLSD -DYKVCAFVG SK-------P
(PRED) sace_34_1_2320  MLNAEFHGRH MDCA-NTLVP SGGDYDNLSD -DYKVCAFVG SK-------P
(PRED) sace_58_25_y007 MLNAEFHGRH MDCA-NTLVP SGGDYDNLSD -DYKVCAFVG SK-------P
(PRED) sace_23_1_2290  MLNAEFHGRH MDCA-NTLVP SGGDYDNLSD -DYKVCAFVG SK-------P
(PRED) sace_6_120_dp00 MLNAEFHGRH MDCA-NTLVP SGGDYDNLSD -DYKVCAFVG SK-------P
(PRED) sace_57_1_a0241 MLNAEFHGRH MDCA-NTLVP SGGDYDNLSD -DYKVCAFVG SK-------P
(PRED) sace_17_1_a0241 MLNAEFHGRH MDCA-NTLVP SGGDYDNLSD -DYKVCAFVG SK-------P
```

(PRED)	sace_21_1_2310	MLNAEFHGRH	MDCA-NTLVP	SGGDYDNLSD	-DYKVCAFVG	SK-------P
(PRED)	sace_49_1_a0246	MLNAEFHGRH	MDCA-NTLVP	SGGDYDNLSD	-DYKVCAFVG	SK-------P
(PRED)	sace_8_2_b02430	MLNAEFHGRH	MDCA-NTLVP	SGGDYDNLSD	-DYKVCAFVG	SK-------P
(PRED)	sace_31_1_2300	MLNAEFHGRH	MDCA-NTLVP	SGGDYDNLSD	-DYKVCAFVG	SK-------P
(PRED)	sace_50_1_a0241	MLNAEFHGRH	MDCA-NTLVP	SGGDYDNLSD	-DYKVCAFVG	SK-------P
(PRED)	sace_4_1_a02360	MLNAEFHGRH	MDCA-NTLVP	SGGDYDNLSD	-DYKVCAFVG	SK-------P
(PRED)	sace_2_1_a02390	MLNAEFHGRH	MDCA-NTLVP	SGGDYDNLSD	-DYKVCAFVG	SK-------P
(PRED)	sace_5_33_ag005	MLNAEFHGRH	MDCA-NTLVP	SGGDYDNLSD	-DYKVCAFVG	SK-------P
(PRED)	sapa_11_1_a0247	MLNAEFHGRH	MNCA-NTLVP	SGGDYDNLSD	-DYKVCAFVG	SK-------P
(PRED)	sapa_25_1_a0246	MLNAEFHGRH	MNCA-NTLVP	SGGDYDNLSD	-DYKVCAFVG	SK-------P
(PRED)	sapa_4_1_a02470	MLNAEFHGRH	MNCA-NTLVP	SGGDYDNLSD	-DYKVCAFVG	SK-------P
(PRED)	sapa_5_1_2350	MLNAEFHGRH	MNCA-NTLVP	SGGDYDNLSD	-DYKVCAFVG	SK-------P
(PRED)	sapa_9_1_2360	MLNAEFHGRH	MNCA-NTLVP	SGGDYDNLSD	-DYKVCAFVG	SK-------P
(PRED)	sapa_14_1_a0244	MLNAEFHGRH	MDCA-NTLVP	SGGDYDSLSD	-DYKVCAFVG	SK-------P
(PRED)	sapa_8_1_2350	MLNAEFHGRH	MDCA-NTLVP	SGGDYDSLSD	-DYKVCAFVG	SK-------P
(PRED)	sapa_17_1_2380	MLNAEFHGRH	MDCA-NTLVP	SGGNYDNLSD	-DYKVCAFVG	SK-------P
(PRED)	sapa_7_1_2370	MLNAEFHGRH	MDCA-NTLVP	SGGNYDNLSD	-DYKVCAFVG	SK-------P
(PRED)	sapa_2_1_a02460	MLNAEFHGRH	MDCA-NTLVP	SGGNYDNLSD	-DYKVCAFVG	SK-------P
(PRED)	sapa_23_1_a0248	MLNAEFHGRH	MDCA-NTLVP	SGGNYDNLSD	-DYKVCAFVG	SK-------P
(PRED)	sapa_3_1_a02470	MLNAEFHGRH	MDCA-NTLVP	SGGNYDNLSD	-DYKVCAFVG	SK-------P
(PRED)	sapa_18_1_2390	MLNAEFHGRH	MDCA-NTLVP	SGGNYDNLSD	-DYKVCAFVG	SK-------P
(PRED)	sami_1_4_244	MLNAEFHGRH	MDCA-NTLVP	SGGNYDNLSD	-DYKVCAFVG	SK-------P
(PRED)	saku_1_4_262	MLNAEFHGRH	MDCA-DTLVP	SGGNYSSLSD	-DYKVCAFVG	SK-------P
(PRED)	saba_1_58_bf002	MLNAEFHGRH	MDCA-DTLVP	SGGSYDSLSD	-DYKVCAFVG	SK-------A
(PRED)	saeu_1_4_d02400	MLNAEFHGRH	MDCA-DTLVP	SGGNYNSLSD	-DYKVCAFVG	SK-------P
(PRED)	naca_1_e01640	MLNAEFHGRR	MDCG-GTLVP	TGPGYENVSS	-ENRVCAFVG	SE-------P
(PRED)	nada_1_g01850	MLNAEFHGRH	MDCG-GTLVP	TGPGYENVAD	-DNKVCAFVG	SE-------P
(PRED)	naca_1_e01630	MLNAEFHGRH	MDCG-GTLVP	TGPGYENVSS	-ENRVCAFVG	SE-------P
(PRED)	nada_1_g01840	MLNAEFHGRH	MDCG-GTLVP	TGPGYENVAD	-DNKVCAFVG	SE-------P
(PRED)	kaaf_1_c00820	MLNAEFHGRH	MDCG-GTLVP	SGQNYENVAA	-ENRVCAFVG	SE-------P
(PRED)	teph_1_m00640	MLNAEFHGRR	LDCG-GTLVP	SGAGYENIAS	-ENQVCAFTG	SV-------E
(PRED)	vapo_1_1036_28	MLNAEFHGRH	MECG-GTLVP	SGPGYENVAN	-ANQVCAFVG	ST-------P
(PRED)	tebl_1_i01760	MLEAEFHGRH	MNCN-KSYVP	RGPGYEDVSP	-DNRVCAFTG	SK-------P
(PRED)	tode_1_d04040	MLNAEFHGRR	MDCG-SGLVP	SGPGYENVAS	-SEQVCAFTG	SK-------P
(PRED)	naca_1_e01650	MLNAEFHGRH	MDCG-GTLVP	SGPGYENVSN	-DNRVCPFAG	SK-------P
(PRED)	tebl_1_g02820	MLNAEFHGRH	MDCG-GTLVP	AGPGYEDVAS	-NEKVCAFAG	SR-------P
(PRED)	lakl_1_c11616g	MLSAEFHNRK	MDCG-NTLVP	SGPGYQNVSS	-DNQVCAFIG	SK-------E
(PRED)	saar_1_8_h03780	MLNAEFHGRH	MDCG-GTLVP	SGPGFENVLS	-ENQVCAFVG	SR-------P
(PRED)	sace_14_7_g0015	MLNAEFHGRH	MDCG-GTLVP	SGPGFENILP	-ENQVCAFVG	SR-------P
(PRED)	sace_15_7_g0387	MLNAEFHGRH	MDCG-GTLVP	SGPGFENILP	-ENQVCAFVG	SR-------P
(PRED)	sace_24_8_3780	MLNAEFHGRH	MDCG-GTLVP	SGPGFENILP	-ENQVCAFVG	SR-------P
(PRED)	sace_40_8_h0383	MLNAEFHGRH	MDCG-GTLVP	SGPGFENILP	-ENQVCAFVG	SR-------P
(PRED)	sace_6_169_fm00	MLNAEFHGRH	MDCG-GTLVP	SGPGFENILP	-ENQVCAFVG	SR-------P
(PRED)	sace_19_7_3840	MLNAEFHGRH	MDCG-GTLVP	SGPGFENILP	-ENQVCAFVG	SR-------P
(PRED)	sace_32_7_3770	MLNAEFHGRH	MDCG-GTLVP	SGPGFENILP	-ENQVCAFVG	SR-------P
(PRED)	sace_56_17_q011	MLNAEFHGRH	MDCG-GTLVP	SGPGFENILP	-ENQVCAFVG	SR-------P
(PRED)	sace_5_78_bz001	MLNAEFHGRH	MDCG-GTLVP	SGPGFENILP	-ENQVCAFVG	SR-------P
(PRED)	sace_2_8_h03860	MLNAEFHGRH	MDCG-GTLVP	SGPGFENILP	-ENQVCAFVG	SR-------P
(PRED)	sace_53_29_ac00	MLNAEFHGRH	MDCG-GTLVP	SGPGFENILP	-ENQVCAFVG	SR-------P
(PRED)	sace_17_7_g0393	MLNAEFHGRH	MDCG-GTLVP	SGPGFENILP	-ENQVCAFVG	SR-------P
(PRED)	sace_25_7_g0388	MLNAEFHGRH	MDCG-GTLVP	SGPGFENILP	-ENQVCAFVG	SR-------P
(PRED)	sace_37_7_g0385	MLNAEFHGRH	MDCG-GTLVP	SGPGFENILP	-ENQVCAFVG	SR-------P
(PRED)	sace_9_7_g00180	MLNAEFHGRH	MDCG-GTLVP	SGPGFENILP	-ENQVCAFVG	SR-------P
(PRED)	sace_60_6_f0335	MLNAEFHGRH	MDCG-GTLVP	SGPGFENILP	-ENQVCAFVG	SR-------P
(PRED)	sace_59_336_lx0	MLNAEFHGRH	MDCG-GTLVP	SGPGFENILP	-ENQVCAFVG	SR-------P
(PRED)	sace_31_7_3780	MLNAEFHGRH	MDCG-GTLVP	SGPGFENILP	-ENQVCAFVG	SR-------P
(PRED)	sace_34_8_3770	MLNAEFHGRH	MDCG-GTLVP	SGPGFENILP	-ENQVCAFVG	SR-------P
(PRED)	sace_58_71_bs00	MLNAEFHGRH	MDCG-GTLVP	SGPGFENILP	-ENQVCAFVG	SR-------P
(PRED)	sace_7_7_g03880	MLNAEFHGRH	MDCG-GTLVP	SGPGFENILP	-ENQVCAFVG	SR-------P
(PRED)	sace_35_7_3840	MLNAEFHGRH	MDCG-GTLVP	SGPGFENILP	-ENQVCAFVG	SR-------P
(PRED)	sace_43_7_g0387	MLNAEFHGRH	MDCG-GTLVP	SGPGFENILP	-ENQVCAFVG	SR-------P
(PRED)	sace_57_8_h0390	MLNAEFHGRH	MDCG-GTLVP	SGPGFENILP	-ENQVCAFVG	SR-------P
(PRED)	sace_45_7_g0389	MLNAEFHGRH	MDCG-GTLVP	SGPGFENILP	-ENQVCAFVG	SR-------P
(PRED)	sace_46_8_h0391	MLNAEFHGRH	MDCG-GTLVP	SGPGFENILP	-ENQVCAFVG	SR-------P
(PRED)	sace_23_7_3860	MLNAEFHGRH	MDCG-GTLVP	SGPGFENILP	-ENQVCAFVG	SR-------P
(PRED)	sace_21_7_3790	MLNAEFHGRH	MDCG-GTLVP	SGPGFENILP	-ENQVCAFVG	SR-------P
(PRED)	sace_8_73_bu001	MLNAEFHGRH	MDCG-GTLVP	SGPGFENILP	-ENQVCAFVG	SR-------P
(PRED)	sapa_1_8_h03820	MLNAEFHGRH	MDCG-GTLVP	SGPGFENILP	-ENQVCAFAG	SR-------P
(PRED)	sapa_21_8_h0387	MLNAEFHGRH	MDCG-GTLVP	SGPGFENILP	-ENQVCAFAG	SR-------P
(PRED)	sapa_20_8_h0386	MLNAEFHGRH	MDCG-GTLVP	SGPGFENILP	-ENQVCAFAG	SR-------P
(PRED)	sapa_22_8_h0390	MLNAEFHGRH	MDCG-GTLVP	SGPGFENILP	-ENQVCAFAG	SR-------P
(PRED)	sapa_25_8_h0387	MLNAEFHGRH	MDCG-GTLVP	SGPGFENILP	-ENQVCAFAG	SR-------P
(PRED)	sapa_6_8_3750	MLNAEFHGRH	MDCG-GTLVP	SGPGFENILP	-ENQVCAFAG	SR-------P
(PRED)	sapa_9_8_3720	MLNAEFHGRH	MDCG-GTLVP	SGPGFENILP	-ENQVCAFAG	SR-------P
(PRED)	sapa_19_8_h0390	MLNAEFHGRH	MDCG-GTLVP	SGPGFENILP	-ENQVCAFAG	SR-------P

(PRED)	sapa_24_8_h0385	MLNAEFHGRH	MDCG-GTLVP	SGPGFENILP	-ENQVCAFAG	SR-------P
(PRED)	sapa_4_8_h03850	MLNAEFHGRH	MDCG-GTLVP	SGPGFENILP	-ENQVCAFAG	SR-------P
(PRED)	sapa_10_8_3760	MLNAEFHGRH	MDCG-GTLVP	SGPGFENILP	-ENQVCAFAG	SR-------P
(PRED)	sapa_13_8_h0382	MLNAEFHGRH	MDCG-GTLVP	SGPGFENILP	-ENQVCAFAG	SR-------P
(PRED)	sapa_8_8_3750	MLNAEFHGRH	MDCG-GTLVP	SGPGFENILP	-ENQVCAFAG	SR-------P
(PRED)	sapa_11_8_h0383	MLNAEFHGRH	MDCG-GTLVP	SGPGFENILP	-ENQVCAFAG	SR-------P
(PRED)	sapa_5_8_3700	MLNAEFHGRH	MDCG-GTLVP	SGPGFENILP	-ENQVCAFAG	SR-------P
(PRED)	sapa_16_8_h0389	MLNAEFHGRR	MDCG-GTLVP	SGPGFENILP	-ENQVCAFAG	SR-------P
(PRED)	sapa_17_8_3730	MLNAEFHGRR	MDCG-GTLVP	SGPGFENILP	-ENQVCAFAG	SR-------P
(PRED)	sapa_2_8_h03860	MLNAEFHGRR	MDCG-GTLVP	SGPGFENILP	-ENQVCAFAG	SR-------P
(PRED)	sapa_7_8_3740	MLNAEFHGRR	MDCG-GTLVP	SGPGFENILP	-ENQVCAFAG	SR-------P
(PRED)	sapa_23_8_h0385	MLNAEFHGRR	MDCG-GTLVP	SGPGFENILP	-ENQVCAFAG	SR-------P
(PRED)	sapa_3_8_h03890	MLNAEFHGRR	MDCG-GTLVP	SGPGFENILP	-ENQVCAFAG	SR-------P
(PRED)	sapa_18_8_3730	MLNAEFHGRH	MDCG-GTLVP	SGPGFENILP	-ENQVCAFAG	SR-------P
(PRED)	sami_1_14_399	MLNAEFHGRH	MDCG-GTLVP	SGPGFENVLP	-ENQVCAFIG	SR-------P
(PRED)	sace_4_8_h03690	MLNAEFHGRH	MDCG-GTLVP	SGPGFENILP	-ENQVCAFVG	SR-------P
(PRED)	saku_1_14_404	MLNAEFHGRH	MDCG-GTLVP	SGPGYENVLS	-ENQVCAFAG	SR-------P
(PRED)	sace_1_ynr070w	MLNAEFHGRH	MDCG-GTLVP	SGPGFENILP	-ENQVCAFVG	SR-------P
(PRED)	sace_49_8_h0383	MLNAEFHGRH	MDCG-GTLVP	SGPGFENILP	-ENQVCAFVG	SR-------P
(PRED)	saeu_1_2_b00130	MLNAEFHGRR	MDCG-GTLIP	SGPGFENVSP	-NNQVCAFVG	SR-------P
(PRED)	sauv_1_7_3	MLNAEFHGRH	MDCG-GTLVP	SGPGFENVSP	-NNQVCAFVG	SR-------P
(PRED)	sami_1_17_26	MLNAEFHGRR	MDCG-GTLVP	SGPGFEVVSS	-ENQVCAFVG	SR-------P
(PRED)	zyba_1_02055_AN	MLEAEFHGRH	MDCG-GTLVP	SGAGYENVAN	-ENRVCAFMG	SQ-------K
(PRED)	zyba_1_07912	MLEAEFHGRH	MDCG-GTLVP	SGAGYENVAN	-ENRVCAFMG	SQ-------K
(PRED)	zyba_2_2_b00600	MLEAEFHGRH	MDCG-GTLVP	SGAGYENVAN	-ENRVCAFMG	SQ-------K
(PRED)	zyba_3_3_c03460	MLEAEFHGRH	MDCG-GTLVP	SGAGYENVAN	-ENRVCAFMG	SQ-------K
(PRED)	zyba_1_04634	MLEAEFHGRH	MDCG-GSLVP	SGPGYENIGS	-ENRVCAFVG	SK-------K
(PRED)	zyba_1_06675	MLEAEFHGRH	MDCG-GSLVP	SGPGYEDILS	-DNRVCAFVG	SK-------K
(PRED)	zyba_3_2_b02230	MLEAEFHGRH	MDCG-GSLVP	SGPGYEDILS	-DNRVCAFVG	SK-------K
(PRED)	zyba_2_1_a00860	MLEAEFHGRH	MDCG-GSLVP	SGPGYEDILS	-DNRVCAFVG	SK-------K
(PRED)	zyro_1_a04114g	MLESEFHGRH	MACGKGSLVP	SGPGYENVPD	-SERVCAFIG	SK-------K
(PRED)	zyro_1_b14762g	MLEAEFHGRH	MSCGSGSLVP	SGPGYENVTD	-SHRVCAFIG	SK-------K
(PRED)	zyba_2_14_n0149	MLEAEFHGRH	MACG-GAVVP	SGAGYENVSN	-DNRVCAFIG	SK-------K
(PRED)	zyba_2_33_ag001	MLEAEFHGRH	MACG-GAVVP	SGSGYENVSN	-DNRVCAFIG	SK-------K
(PRED)	lath_1_a01914g	MLNSEFHARK	MDCT-GSIIP	AGPAYANVAT	-ENQVCAFVG	SK-------P
(PRED)	lawa_1_23_5161	MLNAEFHARE	MDCK-GNVIP	YGPSYANVST	-ENQVCAFVG	SR-------P
(PRED)	klae_1_14_n0012	MLEAEFNGRQ	MDCG-ENYIP	SGKAYANVSS	-DYKVCAFAG	SE-------I
(PRED)	klla_1_d03432g	MLNAEFHARR	MDCG-GTLVP	TGPVYENVSS	-EYKVCAFIG	SQ-------P
(PRED)	klma_1_1_a01880	MLEAEFYARK	MDCG-GTLVP	TGPAYANVSS	-DYKVCAFIG	SQ-------K
(PRED)	klwi_1_33_ag001	MLNAEFHGRH	MDCG-GTLIP	TGSGYEKVNK	-DNQVCAFIG	SK-------T
(PRED)	teph_1_a04220	MLLAEFHGRN	MDCG-GTLVP	SGSGYSDASS	-QHQVCAFIG	SE-------P
(PRED)	vapo_1_1037_47	MLNAEFHNRY	MSCG-GTLVP	SGPGYENVPP	-SNQVCAFPG	ST-------P
(PRED)	pata_1_2_b05590	IITTEFHGRH	MECQSDLLVP	SGTGYENVSS	-ANQVCAFTG	S--------D
(PRED)	wian_1_3_c04380	MLADEFHGRK	MDCGGT-LVP	SGDGYENVDS	-ANQVCAFTG	SE-------T
(PRED)	wian_1_3_c04390	MLADEFHGRK	MDCGGS-LVP	SGPGYENVDS	-ANQVCAFVG	SV-------P
(PRED)	wian_1_7_g01010	MLSDEFHGRR	LSCGGS-LVP	SGAGYEHVSS	-ANQVCAFAG	SV-------T
(PRED)	bain_1_1_a00100	IITNEFHGRE	MDCGSS-LIP	SGGQYTSISS	-VYKSCAFKG	SV-------V
(PRED)	bain_1_17_q0038	IITNEFHDRK	LDCGSS-LVP	SGGEYTNTSS	-AYKSCAFKG	SV-------A
(PRED)	bain_1_8_h00410	MMGNEFHNLQ	LNCTSM-MVP	QQSLYTAYGD	-EYKTCPFTA	SV-------P
(PRED)	caal_1_19_5759	VIASEFHGRK	MQCTSQYLTP	SGPGYENLGA	-GEQVCTFIG	SV-------P
(PRED)	caal_11_25_y002	VIASEFHGRK	MQCTSQYLTP	SGPGYENLGA	-GEQVCTFIG	SV-------P
(PRED)	caal_4_4_d03320	VIASEFHGRK	MQCTSQYLTP	SGPGYENLGA	-GEQVCTFIG	SV-------P
(PRED)	caal_12_26_z005	VIASEFHGRK	MQCTSQYLTP	SGPGYENLGA	-GEQVCTFIG	SV-------P
(PRED)	caal_5_30_ad005	VIASEFHGRK	MQCTSQYLTP	SGPGYENLGA	-GEQVCTFIG	SV-------P
(PRED)	caal_8_3_c03320	VIASEFHGRK	MQCTSQYLTP	SGPGYENLGA	-GEQVCTFIG	SV-------P
(PRED)	caal_6_4_d03280	VIASEFHGRK	MQCTSQYLTP	SGPGYENLGA	-GEQVCTFIG	SV-------P
(PRED)	caal_10_3_c0334	VIASEFHGRK	MQCTSQYLTP	SGPGYENLGA	-GEQVCTFIG	SV-------P
(PRED)	caal_3_29_ac005	VIASEFHGRK	MQCTSQYLTP	SGPGYENLGA	-GEQVCTFIG	SV-------P
(PRED)	caal_2_04989	VIASEFHGRK	MQCTSQYLTP	SGPGYENLGA	-GEQVCTFIG	SV-------P
(PRED)	cadu_1_64350	VIASEFHGRK	MQCTSQYLTP	SGPGYENLGA	-GEQVCTFIG	SV-------P
(PRED)	caor_1_h02090	IVASEFHGRH	MKCLGQYLTP	SGPGYENLGN	-GEQACAFLG	SK-------V
(PRED)	capa_1_600750	VVATEFHGRH	MKCLGSYLTP	SGPGYENLGN	-GEQACAFLG	SK-------P
(PRED)	loel_1_04930	IIASEFHGRR	MPCTGQYLTP	SGPGYENLSA	-GEQVCTFIG	SV-------A
(PRED)	spar_1_5_e03260	VIATEFHGRH	MECAGQYLTP	SGPGFENLGP	-GEQVCTFIG	SI-------P
(PRED)	sppa_1_7_g03160	IIASEFHGRH	MECAGQYLTP	SGPGFENLGP	-GEQVCSFIG	SV-------P
(PRED)	catr_1_01205	IIASEFHGRK	MECTSQYLTP	SGPGYENVGT	-GEQVCAFTG	SI-------P
(PRED)	catr_1_05498	IIASEFHHRK	MECTSEYLTP	SGPGYENVGE	-GEQVCAFTG	SI-------P
(PRED)	catr_1_05971	IIASEFHGRE	MECTYPYLTP	SGPGYENVGQ	-GEQVCAFTG	SV-------P
(PRED)	deha_1_a03696g	IIATEFHGRK	MECDGMYLTP	SGPGYENLSQ	-GSQVCAFKG	SV-------P
(PRED)	deha_2_5_e00720	IIATEFHGRK	MECDGMYLTP	SGPGYEDLGQ	-GNQVCAFKG	SV-------A
(PRED)	scst_1_3_c02890	VVATEFHGRH	MLCDGQYLVP	SGPGFENLSP	-GEQACSFKG	SV-------L
(PRED)	mebi_1_8_h00300	MLATEFHGRK	MPCDASRLTP	SGPGYSS--T	-NNTVCAFVG	SV-------K
(PRED)	lakl_1_h21010g	MLAVEFHGRR	MECT--NLVP	SGPGYETVTE	-ENQVCAFIG	SV-------P
(PRED)	caar_1_13_m0142	MITMEFRGRR	MPCSPTQLLP	RGVGYENISS	-ANQVCAFVG	GSISKEMY-G
(PRED)	caar_1_14_n0143	MITMEFRGRR	MSCSASELLP	RGTGYENVSI	-ANQVCTFVG	ASLSKEKY-G

```
(PRED) hapo_1_1_a07220 LITLEFRGRK  MPCAPSQIIP  RGPGYENISP  -DNRVCAFTG  ASASKALY-G
(PRED) ogpa_1_1_a01680 LITLEFRGRK  MPCTPSQIIP  RGPGYENISA  -DNRVCAFTG  ASASKALY-G
(PRED) piku_1_96_cr001 MILNEFHGRV  MPCAESQLIP  HGPSYSNINQ  LTNQVCGFVG  AAESKELY-G
(PRED) pime_1_4_d03240 MILMEFRGSK  MECSPIQLLP  RGDFYANIDP  LKNQVCAFTG  GALSKQEY-G
(PRED) pime_1_1_a12110 IILMEFRGSR  MPCYPSDLIP  RGEGYENIDT  MVNQVCGFIG  AALSKEKF-N
(PRED) piku_1_227_hs00 MITMQFHNRR  MECFPMSLIP  YGPGYENVNP  RLNQVCGFVG  ASLSKIKY-G
(PRED) pime_1_5_e05800 MITMQFHNLR  MPCSINNLIP  YGPFYTQIDT  KLNQVCGFVG  AANSKILY-N
(PRED) pime_1_1_a07690 MILMQFHNLR  MACSPFELIP  FGPSYANINS  RLNQVCGFVG  AAKSKPLY-D
(PRED) debr_2_5_e03380 LITSEFLGRL  MPCSAAQLVP  HGSHYS--SS  PNNTVCAFVG  AALTRQEFPS
(PRED) kopa_1_2_b10040 MLTNEFHGSI  IDCSDVDLIP  NGPGYEDY-P  DQYRSCAITG  A--------N
(PRED) kopa_2_7_g00500 MLTNEFHGSI  IDCSADDLIP  NGPGYEDY-P  DQYRSCAITG  A--------N
(PRED) asru_1_13_m0119 LISNEFHGLH  MNCA--NTVP  SGPGYENVSS  -ENQVCAAAG  AI-------A
(PRED) asru_1_15_o0045 VVANEFHGRR  MDCS--TLVP  SGPGYEGVSI  -INQACVAVG  SN-------I
(PRED) wian_1_1_a02920 VMLMEFHGRE  MECQPDSLVP  SGIGYETVDS  -VNQVCAFIG  SE-------A
(PRED) wian_1_1_a02930 MALGEFHGRK  MECSPEYLIP  SGPGYENVDS  -ANQVCAFVG  SE-------P

                       .........1010.........1020.........1030.........1040.........1050
(PRED) asac_1_6_f03560 SQSWVLGDDY  LSVNYEYDYA  NQWRNLGILI  AFWIGFLTLK  CLATEYKRPM
(PRED) ergo_1_abr125c   SQSWVLGDDY  LSVNYEYEYE  NQWRNLGILI  AFWIGFLTLK  CLATEYKRPM
(PRED) ercy_1_3604     GQSWVLGDDY  LGVQYEYSYS  HQWRNLGILC  CFFLGFLLIK  CLATEFKRPL
(PRED) cagl_1_i04862g  GQSWVLGDDY  LRLQFEYEYK  HEWRNFGIMW  CFLLGYIALK  ALITEIKRPV
(PRED) kaaf_1_c00830   GQSYVLGDDY  LQMQYQYTYG  HVWRNFGIMW  CFVIGYLVIK  AVITEYKRPV
(PRED) kana_1_k01350   GQSWVLGDDY  LKLQFQYEYK  HTWRNFGIMW  CFLLGYMVIK  CVVTEYKRPV
(PRED) saar_1_2_b02590 GQSYVLGDDY  LKSQFQYVYK  NTWRNFGIMW  CFLLGYVILK  IVFTEYKRPV
(PRED) sace_1_ydr011w  GQSYVLGDDY  LKNQFQYVYK  HTWRNFGILW  CFLLGYVVLK  VIFTEYKRPV
(PRED) sace_16_1_a0238 GQSYVLGDDY  LKNQFQYVYK  HTWRNFGILW  CFLLGYVVLK  VIFTEYKRPV
(PRED) sace_45_1_a0242 GQSYVLGDDY  LKNQFQYVYK  HTWRNFGILW  CFLLGYVVLK  VIFTEYKRPV
(PRED) sace_48_1_a0238 GQSYVLGDDY  LKNQFQYVYK  HTWRNFGILW  CFLLGYVVLK  VIFTEYKRPV
(PRED) sace_60_4_d0244 GQSYVLGDDY  LKNQFQYVYK  HTWRNFGILW  CFLLGYVVLK  VIFTEYKRPV
(PRED) sace_52_1_a0240 GQSYVLGDDY  LKNQFQYVYK  HTWRNFGILW  CFLLGYVVLK  VIFTEYKRPV
(PRED) sace_46_1_a0240 GQSYVLGDDY  LKNQFQYVYK  HTWRNFGILW  CFLLGYVVLK  VIFTEYKRPV
(PRED) sace_25_1_a0240 GQSYVLGDDY  LKNQFQYVYK  HTWRNFGILW  CFLLGYVVLK  VIFTEYKRPV
(PRED) sace_24_1_2300  GQSYVLGDDY  LKNQFQYVYK  HTWRNFGILW  CFLLGYVVLK  VIFTEYKRPV
(PRED) sace_47_1_a0240 GQSYVLGDDY  LKNQFQYVYK  HTWRNFGILW  CFLLGYVVLK  VIFTEYKRPV
(PRED) sace_7_1_a02410 GQSYVLGDDY  LKNQFQYVYK  HTWRNFGILW  CFLLGYVVLK  VIFTEYKRPV
(PRED) sace_59_110_df0 GQSYVLGDDY  LKNQFQYVYK  HTWRNFGILW  CFLLGYVVLK  VIFTEYKRPV
(PRED) sace_56_1_a0202 GQSYVLGDDY  LKNQFQYVYK  HTWRNFGILW  CFLLGYVVLK  VIFTEYKRPV
(PRED) sace_40_1_a0239 GQSYVLGDDY  LKNQFQYVYK  HTWRNFGILW  CFLLGYVVLK  VIFTEYKRPV
(PRED) sace_15_1_a0242 GQSYVLGDDY  LKNQFQYVYK  HTWRNFGILW  CFLLGYVVLK  VIFTEYKRPV
(PRED) sace_37_1_a0243 GQSYVLGDDY  LKNQFQYVYK  HTWRNFGILW  CFLLGYVVLK  VIFTEYKRPV
(PRED) sace_9_1_a02440 GQSYVLGDDY  LKNQFQYVYK  HTWRNFGILW  CFLLGYVVLK  VIFTEYKRPV
(PRED) sace_22_1_2300  GQSYVLGDDY  LKNQFQYVYK  HTWRNFGILW  CFLLGYVVLK  VIFTEYKRPV
(PRED) sace_29_1_2290  GQSYVLGDDY  LKNQFQYVYK  HTWRNFGILW  CFLLGYVVLK  VIFTEYKRPV
(PRED) sace_34_1_2320  GQSYVLGDDY  LKNQFQYVYK  HTWRNFGILW  CFLLGYVVLK  VIFTEYKRPV
(PRED) sace_58_25_y007 GQSYVLGDDY  LKNQFQYVYK  HTWRNFGILW  CFLLGYVVLK  VIFTEYKRPV
(PRED) sace_23_1_2290  GQSYVLGDDY  LKNQFQYVYK  HTWRNFGILW  CFLLGYVVLK  VIFTEYKRPV
(PRED) sace_6_120_dp00 GQSYVLGDDY  LKNQFQYVYK  HTWRNFGILW  CFLLGYVVLK  AIFTEYKRPV
(PRED) sace_57_1_a0241 GQSYVLGDDY  LKNQFQYVYK  HTWRNFGILW  CFLLGYVVLK  VIFTEYKRPV
(PRED) sace_17_1_a0241 GQSYVLGDDY  LKNQFQYVYK  HTWRNFGILW  CFLLGYVVLK  VIFTEYKRPV
(PRED) sace_21_1_2310  GQSYVLGDDY  LKNQFQYVYK  HTWRNFGILW  CFLLGYVVLK  AIFTEYKRPV
(PRED) sace_49_1_a0246 GQSYVLGDDY  LKNQFQYVYK  HTWRNFGILW  CFLLGYVVLK  VIFTEYKRPV
(PRED) sace_8_2_b02430 GQSYVLGDDY  LKNXFQYVYK  HTWRNFGILW  CFLLGYVVLK  VIFTEYKRPV
(PRED) sace_31_1_2300  GQSYVLGDDY  LKNQFQYVYK  HTWRNFGILW  CFLLGYVVLK  VIFTEYKRPV
(PRED) sace_50_1_a0241 GQSYVLGDDY  LKNQFQYVYK  HTWRNFGILW  CFLLGYVVLK  VIFTEYKRPV
(PRED) sace_4_1_a02360 GQSYVLGDDY  LKNQFQYVYK  HTWRNFGILW  CFLLGYVVLK  VIFTEYKRPV
(PRED) sace_2_1_a02390 GQSYVLGDDY  LKNQFQYVYK  HTWRNFGILW  CFLLGYVVLK  VIFTEYKRPV
(PRED) sace_5_33_ag007 GQSYVLGDDY  LKNQFQYVYK  HTWRNFGILW  CFLLGYVVLK  VIFTEYKRPV
(PRED) sapa_11_1_a0247 GQSYVLGDDY  LKNQFQYVYK  HTWRNFGIMW  CFLLGYIVLK  VIFTEYKRPV
(PRED) sapa_25_1_a0246 GQSYVLGDDY  LKNQFQYVYK  HTWRNFGIMW  CFLLGYIVLK  VIFTEYKRPV
(PRED) sapa_4_1_a02470 GQSYVLGDDY  LKNQFQYVYK  HTWRNFGIMW  CFLLGYIVLK  VIFTEYKRPV
(PRED) sapa_5_1_2350   GQSYVLGDDY  LKNQFQYVYK  HTWRNFGIMW  CFLLGYIVLK  VIFTEYKRPV
(PRED) sapa_9_1_2360   GQSYVLGDDY  LKNQFQYVYK  HTWRNFGIMW  CFLLGYIVLK  VIFTEYKRPV
(PRED) sapa_14_1_a0244 GQSYVLGDDY  LKNQFQYVYK  HTWRNFGIMW  CFLLGYIVLK  VIFTEYKRPV
(PRED) sapa_8_1_2350   GQSYVLGDDY  LKNQFQYVYK  HTWRNFGIMW  CFLLGYIVLK  VIFTEYKRPV
(PRED) sapa_17_1_2380  GQSYVLGDDY  LKNQFQYVYK  HTWRNFGIMW  CFLLGYIVLK  VIFTEYKRPV
(PRED) sapa_7_1_2370   GQSYVLGDDY  LKNQFQYVYK  HTWRNFGIMW  CFLLGYIVLK  VIFTEYKRPV
(PRED) sapa_2_1_a02460 GQSYVLGDDY  LKNQFQYVYK  HTWRNFGIMW  CFLLGYIVLK  VIFTEYKRPV
(PRED) sapa_23_1_a0248 GQSYVLGDDY  LKNQFQYVYK  HTWRNFGIMW  CFLLGYIVLK  VIFTEYKRPV
(PRED) sapa_3_1_a02470 GQSYVLGDDY  LKNQFQYVYK  HTWRNFGIMW  CFLLGYIVLK  VIFTEYKRPV
(PRED) sapa_18_1_2390  GQSYVLGDDY  LKNQFQYVYK  HTWRNFGIVW  CFLLGYIVLK  VIFTEYKRPV
(PRED) sami_1_4_244    GQSYVLGDDY  LKNQFQYVYK  HTWRNFGIMW  CFLLGYIVLK  VIFTEYKRPV
(PRED) saku_1_4_262    GQSYVLGDDY  LKNQFQYVYK  HTWRNFGILW  CFLIGYIVLK  VVFTEYKRPV
(PRED) saba_1_58_bf002 GQSYVLGDDY  LKNQFQYVYK  HTWRNFGIMC  CFLIGYIVLK  AVFTEFKRPV
(PRED) saeu_1_4_d02400 GQSYVLGDDY  LKNQFQYVYK  HTWRNFGIMC  CFLIGYVVLK  AVFTEYKRPV
(PRED) naca_1_e01640   GQSWVLGDDY  LKKQFTYEYK  HQWRNFGIMW  CFLIGYMVIK  AVVTEFKRPV
```

(PRED)	nada_1_g01850	GQSWVLGDDY	LKKQFQYEYK	HTWRNFGIIW	CFLLGYLCVK	AVITEFKRPV
(PRED)	naca_1_e01630	GQSWVLGDDY	LKKQFTYEYK	HQWRNFGIMW	CFLIGYMFIK	AFITEHKRPV
(PRED)	nada_1_g01840	GQSWVLGDNY	LRKQFQYEYK	HTWRNFGIMW	CFLIGFVVIR	AVITEHKRPM
(PRED)	kaaf_1_c00820	GQSWVLGDNY	LRKQFEYVYK	HVWRNFGIMW	CFVLGYITLK	AIITEYKTPV
(PRED)	teph_1_m00640	GQSYVLGDDY	LKAYFQYEYK	HTWRNFGILW	CFIIGYFSIK	AFLTEFKRPT
(PRED)	vapo_1_1036_28	GQSWVLGDNY	LRLQYTYEYK	HTWRNFGIMW	CFLIGYVGLK	ALITEFKRPV
(PRED)	tebl_1_i01760	GQNYVLGDDY	LRIQYTYVWA	HTWRNLAIMW	AFVLGYVGGK	IIFTEFKRPV
(PRED)	tode_1_d04040	GQSWVLGDDY	LKVQFQYEYK	HTWRNFGIMW	CFLIGYIVLK	SLITEFKRPI
(PRED)	naca_1_e01650	GQSWVLGDDY	LRLQYTYQYK	HTWRNFGIMW	CFLIGYLVIK	AVVTEFKRPV
(PRED)	tebl_1_g02820	GQSWVNGDDY	LKTQYRYQYK	HTWRNFGIMW	CFLLGYIGIK	AIVTEIKRPV
(PRED)	lakl_1_c11616g	GQSWVLGDDY	LKLQYEYKYS	HVWRNLGIMF	AFLCFYLTVK	CLVTEFKRPI
(PRED)	saar_1_8_h03780	GQSWVLGDDY	LRAQYEYEYK	NTWRNFGIMW	CFLIGYVVLR	ALFTEYKSPV
(PRED)	sace_14_7_g0015	GQSWVLGDDY	LRAQYQYEYK	NTWRNFGIMW	CFLIGYIVLR	AVFTEYKSPV
(PRED)	sace_15_7_g0387	GQSWVLGDDY	LRAQYQYEYK	NTWRNFGIMW	CFLIGYIVLR	AVFTEYKSPV
(PRED)	sace_24_8_3780	GQSWVLGDDY	LRAQYQYEYK	NTWRNFGIMW	CFLIGYIVLR	AVFTEYKSPV
(PRED)	sace_40_8_h0383	GQSWVLGDDY	LRAQYQYEYK	NTWRNFGIMW	CFLIGYIVLR	AVFTEYKSPV
(PRED)	sace_6_169_fm00	GQSWVLGDDY	LRAQYQYEYK	NTWRNFGIMW	CFLIGYIVLR	AVFTEYKSPV
(PRED)	sace_19_7_3840	GQSWVLGDDY	LRAQYQYEYK	NTWRNFGIMW	CFLIGYIVLR	ALFTEYKSPV
(PRED)	sace_32_7_3770	GQSWVLGDDY	LRAQYQYEYK	NTWRNFGIMW	CFLIGYIVLR	AVFTEYKSPV
(PRED)	sace_56_17_q011	GQSWVLGDDY	LRAQYQYEYK	NTWRNFGIMW	CFLIGYIVLR	AVFTEYKSPV
(PRED)	sace_5_78_bz001	GQSWVLGDDY	LRAQYQYEYK	NTWRNFGIMW	CFLIGYIVLR	AVFTEYKSPV
(PRED)	sace_2_8_h03860	GQSWVLGDDY	LRAQYQYEYK	NTWRNFGIMW	CFLIGYIVLR	ALFTEYKSPV
(PRED)	sace_53_29_ac00	GQSWVLGDDY	LRAQYQYEYK	NTWRNFGIMW	CFLIGYIVLR	AVFTEYKSPV
(PRED)	sace_17_7_g0393	GQSWVLGDDY	LRAQYQYEYK	NTWRNFGIMW	CFLIGYIVLR	AVFTEYKSPV
(PRED)	sace_25_7_g0388	GQSWVLGDDY	LRAQYQYEYK	NTWRNFGIMW	CFLIGYIVLR	AVFTEYKSPV
(PRED)	sace_37_7_g0385	GQSWVLGDDY	LRAQYQYEYK	NTWRNFGIMW	CFLIGYIVLR	AVFTEYKSPV
(PRED)	sace_9_7_g00180	GQSWVLGDDY	LRAQYQYEYK	NTWRNFGIMW	CFLIGYIVLR	AVFTEYKSPV
(PRED)	sace_60_6_f0335	GQSWVLGDDY	LRAQYQYEYK	NTWRNFGIMW	CFLIGYIVLR	AVFTEYKSPV
(PRED)	sace_59_336_lx0	GQSWVLGDDY	LRAQYQYEYK	NTWRNFGIMW	CFLIGYIVLR	AVFTEYKSPV
(PRED)	sace_31_7_3780	GQSWVLGDDY	LRAQYQYEYK	NTWRNFGIMW	CFLIGYIVLR	AVFTGYKSPV
(PRED)	sace_34_8_3770	GQSWVLGDDY	LRAQYQYEYK	NTWRNFGIMW	CFLIGYIVLR	AVFTEYKSPV
(PRED)	sace_58_71_bs00	GQSWVLGDDY	LRAQYQYEYK	NTWRNFGIMW	CFLIGYIVLR	AVFTEYKSPV
(PRED)	sace_7_7_g03880	GQSWVLGDDY	LRAQYQYEYK	NTWRNFGIMW	CFLIGYIVLR	AVFTEYKSPV
(PRED)	sace_35_7_3840	GQSWVLGDDY	LRAQYQYEYK	NTWRNFGIMW	CFLIGYIVLR	AVFTEYKSPV
(PRED)	sace_43_7_g0387	GQSWVLGDDY	LRAQYQYEYK	NTWRNFGIMW	CFLIGYIVLR	AVFTEYKSPV
(PRED)	sace_57_8_h0390	GQSWVLGDDY	LRAQYQYEYK	NTWRNFGIMW	CFLIGYIVLR	AVFTEYKSPV
(PRED)	sace_45_7_g0389	GQSWVLGDDY	LRAQYQYEYK	NTWRNFGIMW	CFLIGYIVLR	AVFTEYKSPV
(PRED)	sace_46_8_h0391	GQSWVLGDDY	LRAQYQYEYK	NTWRNFGIMW	CFLIGYIVLR	AVFTEYKSPV
(PRED)	sace_23_7_3860	GQSWVLGDDY	LRAQYQYEYK	NTWRNFGIMW	CFLIGYIVLR	AVFTEYKSPV
(PRED)	sace_21_7_3790	GQSWVLGDDY	LRAQYQYEYK	NTWRNFGIMW	CFLIGYIVLR	AVFTEYKSPV
(PRED)	sace_8_73_bu001	GQSWVLGDDY	LRAQYQYEYK	NTWRNFGIMW	CFLIGYIVLR	AVFTEYKSPV
(PRED)	sapa_1_8_h03820	GQSWVLGDDY	LRSQYEYEYK	NTWRNFGIMW	CFLIGYIVLR	ALFTEYKSPV
(PRED)	sapa_21_8_h0387	GQSWVLGDDY	LRSQYEYEYK	NTWRNFGIMW	CFLIGYIVLR	ALFTEYKSPV
(PRED)	sapa_20_8_h0386	GQSWVLGDDY	LRSQYEYEYK	NTWRNFGIMW	CFLIGYIVLR	ALFTEYKSPV
(PRED)	sapa_22_8_h0390	GQSWVLGDDY	LRSQYEYEYK	NTWRNFGIMW	CFLIGYIVLR	ALFTEYKSPV
(PRED)	sapa_25_8_h0387	GQSWVLGDDY	LRSQYEYEYK	NTWRNFGIMW	CFLIGYIVLR	ALFTEYKSPV
(PRED)	sapa_6_8_3750	GQSWVLGDDY	LRSQYEYEYK	NTWRNFGIMW	CFLIGYIVLR	ALFTEYKSPV
(PRED)	sapa_9_8_3720	GQSWVLGDDY	LRSQYEYEYK	NTWRNFGIMW	CFLIGYIVLR	ALFTEYKSPV
(PRED)	sapa_19_8_h0390	GQSWVLGDDY	LRSQYEYEYK	NTWRNFGIMW	CFLIGYIVLR	ALFTEYKSPV
(PRED)	sapa_24_8_h0385	GQSWVLGDDY	LRSQYEYEYK	NTWRNFGIMW	CFLIGYIVLR	ALFTEYKSPV
(PRED)	sapa_4_8_h03850	GQSWVLGDDY	LRSQYEYEYK	NTWRNFGIMW	CFLIGYIVLR	ALFTEYKSPV
(PRED)	sapa_10_8_3760	GQSWVLGDDY	LRSQYEYEYK	NTWRNFGIMW	CFLIGYIVLR	ALFTEYKSPV
(PRED)	sapa_13_8_h0382	GQSWVLGDDY	LRSQYEYEYK	NTWRNFGIMW	CFLIGYIVLR	ALFTEYKSPV
(PRED)	sapa_8_8_3750	GQSWVLGDDY	LRSQYEYEYK	NTWRNFGIMW	CFLIGYIVLR	ALFTEYKSPV
(PRED)	sapa_11_8_h0383	GQSWVLGDDY	LRSQYEYEYK	NTWRNFGIMW	CFLIGYIVLR	ALFTEYKSPV
(PRED)	sapa_5_8_3700	GQSWVLGDDY	LRSQYEYEYK	NTWRNFGIMW	CFLIGYIVLR	ALFTEYKSPV
(PRED)	sapa_16_8_h0389	GQSWVLGDDY	LRSQYQYEYK	NTWRNFGIMW	CFLIGYIVLR	ALFTEYKSPV
(PRED)	sapa_17_8_3730	GQSWVLGDDY	LRSQYQYEYK	NTWRNFGIMW	CFLIGYIVLR	ALFTEYKSPV
(PRED)	sapa_2_8_h03860	GQSWVLGDDY	LRSQYQYEYK	NTWRNFGIMW	CFLIGYIVLR	ALFTEYKSPV
(PRED)	sapa_7_8_3740	GQSWVLGDDY	LRSQYQYEYK	NTWRNFGIMW	CFLIGYIVLR	ALFTEYKSPV
(PRED)	sapa_23_8_h0385	GQSWVLGDDY	LRSQYQYEYK	NTWRNFGIMW	CFLIGYIVLR	ALFTEYKSPV
(PRED)	sapa_3_8_h03890	GQSWVLGDDY	LRSQYQYEYK	NTWRNFGIMW	CFLIGYIVLR	ALFTEYKSPV
(PRED)	sapa_18_8_3730	GQSWVLGDNY	LRSQYQYEYK	NTWRNFGIMW	CFLIGYIVLR	ALFTEYKSPV
(PRED)	sami_1_14_399	GQSWVLGDDY	LRAQYQYEYS	NTWRNFGIMW	CFLIGYIILR	ALFTEYKSPV
(PRED)	sace_4_8_h03690	GQSWVLGDDY	LRAQYQYEYK	NTWRNFGIMW	CFLIGYIVLR	AVFTEYKSPV
(PRED)	saku_1_14_404	GQAWVLGDDY	LRAQYQYEYK	NTWRNFGIMW	CFLIGYIVLR	ALFTEYKSPI
(PRED)	sace_1_ynr070w	GQSWVLGDDY	LRAQYQYEYK	NTWRNFGIMW	CFLIGYIVLR	AVFTEYKSPV
(PRED)	sace_49_8_h0383	GQSWVLGDDY	LRAQYQYEYK	NTWRNFGIMW	CFLIGYIVLR	AVFTEYKSPV
(PRED)	saeu_1_2_b00130	GQTWVLGDDY	LKDQYEYEYK	NTWRNFGIMW	CFLIGYIILK	ALFTEFKNPV
(PRED)	sauv_1_7_3	GQTWVLGDDY	LKAQYQYEYK	HTWRNFGIMW	CFLIGYIILK	ALFTEFKDPV
(PRED)	sami_1_17_26	GQSWVLGDDY	LKAQFQYDYK	HTWRNFGIMW	CFLIGYIVLK	AVFTEFKNPV
(PRED)	zyba_1_02055_AN	GQSWVLGDDY	LKSQYGYKYW	HMWRNWGIMF	IFLIGYIVVK	AVITEIKRPV
(PRED)	zyba_1_07912	GQSWVLGDDY	LKSQYGYKYW	HMWRNWGIMF	IFLVGYIVVK	AVITEIKRPV
(PRED)	zyba_2_2_b00600	GQSWVLGDDY	LKSQYGYKYW	HMWRNWGIMF	IFLVGYIVVK	AVITEIKRPV
(PRED)	zyba_3_3_c03460	GQSWVLGDDY	LKSQYGYKYW	HMWRNWGIMF	IFLVGYIVVK	AVITEIKRPV

```
(PRED) zyba_1_04634    GQSWVLGDDY LKTQFGYKYW HLWRNWGIMF IFLIGYIVAK TIFTEIKRPT
(PRED) zyba_1_06675    GQSWVLGDDY LKTQFGYKYW HMWRNWGIMF IFLVGYIVAK TLFTEIKRPT
(PRED) zyba_3_2_b02230 GQSWVLGDDY LKTQFGYKYW HMWRNWGIMF IFLVGYIVAK TLFTEIKRPT
(PRED) zyba_2_1_a00860 GQSWVLGDDY LKTQFGYKYW HMWRNWGIMF IFLVGYIVAK TLFTEIKRPT
(PRED) zyro_1_a04114g  GRSWVLGDDY LNVQYQYIYW HLWRNIGIMF AFLIGYNVFK AIITEIKRPV
(PRED) zyro_1_b14762g  GQSWVLGDDY LYAQYTYKYW HVWRNLGIMF AFLIGYVAVK AIITEIKRPV
(PRED) zyba_2_14_n0149 GQDWVLGDDY LKVQFQYKYW HMWRNWGIMF IFLVGYVALK AFFTEVKEPV
(PRED) zyba_2_33_ag001 GQDWVLGDDY LKVQFQYKYW HMWRNWGIMF IFLVGYVVLK AVFTEVKEPV
(PRED) lath_1_a01914g  GQDYVNGDDY IKANYGYKYD HTWRNLGFLF AFLAVYVALK CLITELNTTS
(PRED) lawa_1_23_5161  GQPFVTGDDY INTNYGYKYS HTWRNLGFMF AFFLFYLSLK CIITEFKHSS
(PRED) klae_1_14_n0012 GQSTVSGNAY LTARYGYTYS HVWRNLGIIF AFLVGYLVIK CVLTEFKTPV
(PRED) klla_1_d03432g  GESYVLGDNY LKLQYDYSYS HQWRNFGILI AFLVGFLVFK SVITEFKTPI
(PRED) klma_1_1_a01880 GQSYVLGNNY LKLQYGYTYS HVWRNFGILI AFLIGFFAIK CFITEFKTPI
(PRED) klwi_1_33_ag001 GESYVLGDNY LKLQYGYTYS HVWRNLGILF AFLVGYMVLK GFVSEFKTPV
(PRED) teph_1_a04220   GQNAVLGDNY LEVQYEYKYS HLWRNFGILW CFLLGYLVIR ALITEFKPTM
(PRED) vapo_1_1037_47  GSSEVLGDNY LRVQFTYQYK NTWRNFGIFW CFLLGYLGLK CLLAEFKPSF
(PRED) pata_1_2_b05590 GSTVVDGDTF VYVSYTYKFW HVWRNFGILC CFWIGFITIN CILTELLKQS
(PRED) wian_1_3_c04380 GQSWVSGDRY LEIQFNFKYS NLWRNFGIVI AFFVVFLAIN AISTEFKRPV
(PRED) wian_1_3_c04390 GQSWVSGDSY LKVQFNFKYT HIWRNFGILI GFFIFFLGVN AICTEFKRPV
(PRED) wian_1_7_g01010 GQTWVDGDRY LRVQYSFKYS HLWRNFGIII AFFVVFLAVN AVCTEFKRPM
(PRED) bain_1_1_a00100 GQSWVSGDAY MKVNFQYSYS HVWRNLGILI GFWIFFTSLK ILATEYKSPM
(PRED) bain_1_17_q0038 GQSWVSGDTY MEVNYQYSYS HVWRNLGILI GFWIFFTSLQ ILATEYKSSM
(PRED) bain_1_8_h00410 GQPWVDGDAY LRISFDYFHS HIWRNFGIMI GFWVFFIFLQ AMASEYRKPT
(PRED) caal_1_19_5759  GQSWVLGDDY LRIAYTYRFS HVWRNLGILF GFLAFFLAIA TLGTEYVKPI
(PRED) caal_11_25_y002 GQSWVLGDDY LRIAYTYRFS HVWRNLGILF GFLAFFLAIA TLGTEYVKPI
(PRED) caal_4_4_d03320 GQSWVLGDDY LRIAYTYRFS HVWRNLGILF GFLAFFLAIA TLGTEYVKPI
(PRED) caal_12_26_z005 GQSWVLGDDY LRIAYTYRFS HVWRNLGILF GFLAFFLAIA TLGTEYVKPI
(PRED) caal_5_30_ad005 GQSWVLGDDY LRIAYTYRFS HVWRNLGILF GFLAFFLAIA TLGTEYVKPI
(PRED) caal_8_3_c03320 GQSWVLGDDY LRIAYTYRFS HVWRNLGILF GFLAFFLAIA TLGTEYVKPI
(PRED) caal_6_4_d03280 GQSWVLGDDY LRIAYTYRFS HVWRNLGILF GFLAFFLAIA TLGTEYVKPI
(PRED) caal_10_3_c0334 GQSWVLGDDY LRIAYTYRFS HVWRNLGILF GFLAFFLAIA TLGTEYVKPI
(PRED) caal_3_29_ac005 GQSWVLGDDY LRIAYTYRFS HVWRNLGILF GFLAFFLAIA TLGTEYVKPI
(PRED) caal_2_04989    GQSWVLGDDY LRIAYTYRFS HVWRNLGILF GFLAFFLAIA TLGTEYVKPI
(PRED) cadu_1_64350    GQSWVLGDDY LRIAYTYRFS HVWRNLGILF GFLAFFLTIA TLGTEYVKPI
(PRED) caor_1_h02090   GQDWVLGDDY LKTAYTYSFN HVWRNFGIMI GFMAFFLAIN ALGTEYIKPI
(PRED) capa_1_600750   GQDWILGDDY LKTAYTYSFN HVWRNFGIMI GFLAFFLAIN ALGTEFIKPI
(PRED) loel_1_04930    GQNWVLGDDY LRIAYTYRFT HVWRNLGILI GFLAFFLAIT SLGTEYIKPI
(PRED) spar_1_5_e03260 GQSWVLGDDY LRIAFTYKFT HVWRNLGILF GFLFFFLAIN CLGTEYVKPI
(PRED) sppa_1_7_g03160 GQSWVLGDQY LRIAFTYEFS HVWRNLGILF GFLFFFLAIN ALGTEYVKPI
(PRED) catr_1_01205    GQNWVSGDKY LTVSYTYRFS HVWRNLGILI GFLAFFLTIN ALGTEYIKPI
(PRED) catr_1_05498    GTKWVSGEKY LSVSYTYKFI HVWRNFAILV GFLAFFLAVN ALGTEFIKPI
(PRED) catr_1_05971    GQDWVSGDRY LEVAYTYRFS HVWRNLGIII GFLAFFLAVN CLGTEFIKPI
(PRED) deha_1_a03696g  GQSWVSGDNY LKVAFTYSFS HVWRNFGIMI GFLVFFTCVK ALGVEFIRPI
(PRED) deha_2_5_e00720 GQSWVSGDSY LEVAFTYKFS HVWRNFGIMI AFLVLFTCVK ALGVEFIRPI
(PRED) scst_1_3_c02890 GQTWVLGDEY LKTAYTYSFS HVWRNFGIMI AFLIFFVTVT ALGTEFVRPI
(PRED) mebi_1_8_h00300 DQTWVDGDRY IKLSYEYSFS HVWRNFGILI GFIIFFIGVG ALGFEVVRPI
(PRED) lakl_1_h21010g  GQPWVSGDRY IRIQYDFRYS YIWRNFGICL GFLCFYVALN TFADEFRKKS
(PRED) caar_1_13_m0142 NND-VNGEVY LSLAFLYTFD HCWRNLGILF GIGLGVLAIN SLVVEFYNPI
(PRED) caar_1_14_n0143 NND-VSGEIY LSLSFLYTFD HCWRNFGIII GMVIGILAIN CFVVEFYNPL
(PRED) hapo_1_1_a07220 SGDYVSGDIY LSYSFQYTFS HCWRNFGILI AFVVGFLAIN MLIVEAYNPM
(PRED) ogpa_1_1_a01680 SGDYVSGDIY LSYSFQYTFS HCWRNFGILI GFVLGFLIIN MIIVEAYNPI
(PRED) piku_1_96_cr001 GKNDVDGMIY LKLAFQYVYP HLWRNLGIMF AFLCGYLTLN VILVEIYNPI
(PRED) pime_1_4_d03240 GQNYVDGMIY LKLAFQYTWN HMWRNFGIMF CFIVGYLVVN MILVEVYNPI
(PRED) pime_1_1_a12110 GANDVDGMIY LDLSYNYIWR HMWRNFGIMF CFIIGYLVIN CILVEIYNPI
(PRED) piku_1_227_hs00 GSNDVDGDIY IKLAYTYTFG HVWRNFGFMF IFIFGYLVIN AIAVELYNPI
(PRED) pime_1_5_e05800 GSNDVNGDIY LKLSFTYAFS HCWRNFGFMF IFIAGYLIIN SIAVELFNPI
(PRED) pime_1_1_a07690 GANDVKGDIY LELAFTYTFN HVWRNFGIMF AFIIGYIAIS CLIVENFNPI
(PRED) debr_2_5_e03380 AGNAVDGSIY LSLAFQYSYS HAWRNLGILF GFILGADILN VLLVEYYNPI
(PRED) kopa_1_2_b10040 GRTYVDGDTY LDLSFEYSYS HIWRNMGILF LFYVAFLVIH SVMSEIMNMS
(PRED) kopa_2_7_g00500 GRTYVDGDTY VELSFEYSFS HIWRNMGILF LFYIAFLVIH SVMSEIMNMS
(PRED) asru_1_13_m0119 GQAFVLGDDY IESSYDYSYS HLWRNFGILV GFWIFFILLS AIGLEILKPV
(PRED) asru_1_15_o0045 GENYVLGDDY ASLSYGYEYS HLWRNFGILI GFWVFFLVLT IHGMEILKLP
(PRED) wian_1_1_a02920 GQTWVSGDKY LEVVYDYTFG HIWRNLGILI GFIFFFLSLT VVCSDYVNYD
(PRED) wian_1_1_a02930 GQPWVSGDRY LDISFEYTYG HNWRNLGILI GFALFFTVCL SVFADLMKFG

                       .........1060.........1070.........1080.........1090.........1100
(PRED) asac_1_6_f03560 KGGGDSLVFK ---KGSLA-K RRI------- ---------- ----QHDSES
(PRED) ergo_1_abr125c  KGGGDSLVFK ---KGTLA-K RKV------- ---------- ----QDDSES
(PRED) ercy_1_3604     KGGGDSLVFK ---KGTLARK KKL------- ---------- ----QHDVES
(PRED) cagl_1_i04862g  KGGGDALIFK ---KGTRK-Y HM-------- ---------- ----KLDEED
(PRED) kaaf_1_c00830   KGGGDALLFK ---KGSKR-F EVT------- ---------- -----TDIES
(PRED) kana_1_k01350   KGGGDALIFK ---KGAKR-F -QL------- ---------- ----KHDEEA
(PRED) saar_1_2_b02590 KGGGDALIFK ---KGSKR-F -IA------- ---------- ----PADEES
(PRED) sace_1_ydr011w  KGGGDALIFK ---KGSKR-F -IA------- ---------- ----HADEES
(PRED) sace_16_1_a0238 KGGGDALIFK ---KGSKR-F -IA------- ---------- ----HADEES
```

(PRED)	sace_45_1_a0242	KGGGDALIFK	---KGSKR-F	-IA-------	----------	----HADEES
(PRED)	sace_48_1_a0238	KGGGDALIFK	---KGSKR-F	-IA-------	----------	----HADEES
(PRED)	sace_60_4_d0244	KGGGDALIFK	---KGSKR-F	-IA-------	----------	----HADEES
(PRED)	sace_52_1_a0240	KGGGDALIFK	---KGSKR-F	-IA-------	----------	----HADEES
(PRED)	sace_46_1_a0240	KGGGDALIFK	---KGSKR-F	-IA-------	----------	----HADEES
(PRED)	sace_25_1_a0240	KGGGDALIFK	---KGSKR-F	-IA-------	----------	----HADEES
(PRED)	sace_24_1_2300	KGGGDALIFK	---KGSKR-F	-IA-------	----------	----HADEES
(PRED)	sace_47_1_a0240	KGGGDALIFK	---KGSKR-F	-IA-------	----------	----HADEES
(PRED)	sace_7_1_a02410	KGGGDALIFK	---KGSKR-F	-IA-------	----------	----HADEES
(PRED)	sace_59_110_df0	KGGGDALIFK	---KGSKR-F	-IA-------	----------	----HADEES
(PRED)	sace_56_1_a0202	KGGGDALIFK	---KGSKR-F	-IA-------	----------	----HADEES
(PRED)	sace_40_1_a0239	KGGGDALIFK	---KGSKR-F	-IA-------	----------	----HADEES
(PRED)	sace_15_1_a0242	KGGGDALIFK	---KGSKR-F	-IA-------	----------	----HADEES
(PRED)	sace_37_1_a0243	KGGGDALIFK	---KGSKR-F	-IA-------	----------	----HADEES
(PRED)	sace_9_1_a02440	KGGGDALIFK	---KGSKR-F	-IA-------	----------	----HADEES
(PRED)	sace_22_1_2300	KGGGDALIFK	---KGSKR-F	-IA-------	----------	----HADEES
(PRED)	sace_29_1_2290	KGGGDALIFK	---KGSKR-F	-IA-------	----------	----HADEES
(PRED)	sace_34_1_2320	KGGGDALIFK	---KGSKR-F	-IA-------	----------	----HADEES
(PRED)	sace_58_25_y007	KGGGDALIFK	---KGSKR-F	-IA-------	----------	----HADEES
(PRED)	sace_23_1_2290	KGGGDALIFK	---KGSKR-F	-IA-------	----------	----HADEES
(PRED)	sace_6_120_dp00	KGGGDALIFK	---KGSKR-F	-IA-------	----------	----HADEES
(PRED)	sace_57_1_a0241	KGGGDALIFK	---KGSKR-F	-IA-------	----------	----HADEES
(PRED)	sace_17_1_a0241	KGGGDALIFK	---KGSKR-F	-IA-------	----------	----HADEES
(PRED)	sace_21_1_2310	KGGGDALIFK	---KGSKR-F	-IA-------	----------	----HADEES
(PRED)	sace_49_1_a0246	KGGGDALIFK	---KGSKR-F	-IA-------	----------	----HADEES
(PRED)	sace_8_2_b02430	KGGGDALIFK	---KGSKR-F	-IA-------	----------	----HADEES
(PRED)	sace_31_1_2300	KGGSDALIFK	---KGSKR-F	-IA-------	----------	----HADEES
(PRED)	sace_50_1_a0241	KGGGDALIFK	---KGSKR-F	-IA-------	----------	----HADEES
(PRED)	sace_4_1_a02360	KGGGDALIFK	---KGSKR-F	-IA-------	----------	----HADEES
(PRED)	sace_2_1_a02390	KGGGDALIFK	---KGSKR-F	-IA-------	----------	----HADEES
(PRED)	sace_5_33_ag005	KGGGDALIFK	---KGSKR-F	-IA-------	----------	----HADEES
(PRED)	sapa_11_1_a0247	KGGGDALIFK	---KGSKR-F	-IA-------	----------	----RADEES
(PRED)	sapa_25_1_a0246	KGGGDALIFK	---KGSKR-F	-IA-------	----------	----RADEES
(PRED)	sapa_4_1_a02470	KGGGDALIFK	---KGSKR-F	-IA-------	----------	----RADEES
(PRED)	sapa_5_1_2350	KGGGDALIFK	---KGSKR-F	-IA-------	----------	----RADEES
(PRED)	sapa_9_1_2360	KGGGDALIFK	---KGSKR-F	-IA-------	----------	----RADEES
(PRED)	sapa_14_1_a0244	KGGGDALIFK	---KGSKR-F	-IA-------	----------	----RADEES
(PRED)	sapa_8_1_2350	KGGGDALIFK	---KGSKR-F	-IA-------	----------	----RADEES
(PRED)	sapa_17_1_2380	KGGGDALIFK	---KGSKR-F	-IA-------	----------	----HADEES
(PRED)	sapa_7_1_2370	KGGGDALIFK	---KGSKR-F	-IA-------	----------	----HADEES
(PRED)	sapa_2_1_a02460	KGGGDALIFK	---KGSKR-F	-IA-------	----------	----HADEES
(PRED)	sapa_23_1_a0248	KGGGDALIFK	---KGSKR-F	-IA-------	----------	----HADEES
(PRED)	sapa_3_1_a02470	KGGGDALIFK	---KGSKR-F	-IA-------	----------	----HADEES
(PRED)	sapa_18_1_2390	KGGGDALIFK	---KGSKR-F	-IA-------	----------	----HADEES
(PRED)	sami_1_4_244	KGGGDALIFK	---KGSKR-F	-IT-------	----------	----PTDEES
(PRED)	saku_1_4_262	KGGGDALIFK	---KGSKR-F	-VA-------	----------	----PVDEES
(PRED)	saba_1_58_bf002	KGGGDALIFK	---KGSRK-F	-IA-------	----------	----PADEES
(PRED)	saeu_1_4_d02400	KGGGDALIFK	---KGSRK-F	-TA-------	----------	----PADEES
(PRED)	naca_1_e01640	KGGGDALLFK	---KGTLK-S	-KI-------	----------	----VDDEES
(PRED)	nada_1_g01850	KGGGDAMLYK	---KGTKI-M	-KV-------	----------	----ADDEES
(PRED)	naca_1_e01630	KGGGDALLFK	---KGSIR-Y	NDV-------	----------	-----NDLES
(PRED)	nada_1_g01840	QGGGDALVFK	---KGSES-F	RVI-------	----------	----AADEES
(PRED)	kaaf_1_c00820	KDTGDALIYK	---KGSKS-Y	KMK-------	----------	----N-DEES
(PRED)	teph_1_m00640	VGGGDALVFK	---KGYKH-P	NSN-------	----------	----TANDEE
(PRED)	vapo_1_1036_28	ISGGDALIFK	---KGYK---	RST-------	----------	----ISSDEE
(PRED)	tebl_1_i01760	KAGGDMLLYK	---KGTK--L	QFN-------	----------	----HNDEEN
(PRED)	tode_1_d04040	KGGGDALIFK	---KGAKSAI	KRV-------	----------	----KADDEE
(PRED)	naca_1_e01650	KGGGDALIFK	---KGAEQ-F	KHS-------	----------	-----SDPEF
(PRED)	tebl_1_g02820	KSGGDALIFK	---KGFKP--	--T-------	----------	----ETVEDI
(PRED)	lakl_1_c11616g	KGGGDALLFK	---KGTRN--	RPS-------	----------	----PTDEES
(PRED)	saar_1_8_h03780	KSGGDALVIK	---KGMKDGP	QK--------	----------	----SWNDKY
(PRED)	sace_14_7_g0015	KSGGDALVVK	---KGTKNAI	QR--------	----------	----SWSSKN
(PRED)	sace_15_7_g0387	KSGGDALVVK	---KGTKNAI	QR--------	----------	----SWSSKN
(PRED)	sace_24_8_3780	KSGGDALVVK	---KGTKNAI	QR--------	----------	----SWSSKN
(PRED)	sace_40_8_h0383	KSGGDALVVK	---KGTKNAI	QR--------	----------	----SWSSKN
(PRED)	sace_6_169_fm00	KSGGDALVVK	---KGTKNAI	QR--------	----------	----SWSSKN
(PRED)	sace_19_7_3840	KSGGDALVVK	---KGTKNAI	QR--------	----------	----SWSSKN
(PRED)	sace_32_7_3770	KSGGDALVVK	---KGTKNAI	QR--------	----------	----SWSSKN
(PRED)	sace_56_17_q011	KSGGDALVVK	---KGTKNAI	QR--------	----------	----SWSSKN
(PRED)	sace_5_78_bz001	KSGGDALVVK	---KGTKNAI	QR--------	----------	----SWSSKN
(PRED)	sace_2_8_h03860	KSGGDALVVK	---KGTKNAI	QR--------	----------	----SWSSKN
(PRED)	sace_53_29_ac00	KSGGDALVVK	---KGTKNAI	QR--------	----------	----SWSSKN
(PRED)	sace_17_7_g0393	KSGGDVLVVK	---KGTKNAI	QR--------	----------	----SWSSKN
(PRED)	sace_25_7_g0388	KSGGDVLVVK	---KGTKNAI	QR--------	----------	----SWSSKN
(PRED)	sace_37_7_g0385	KSGGDALVVK	---KGTKNAI	QR--------	----------	----SWSSKN

(PRED)	sace_9_7_g00180	KSGGDALVVK	---KGTKNAI	QR--------	----------	----SWSSKN
(PRED)	sace_60_6_f0335	KSGGDALVVK	---KGTKNAI	QR--------	----------	----SWSSKN
(PRED)	sace_59_336_lx0	KSGGDALVVK	---KGTKNAI	QR--------	----------	----SWSSKN
(PRED)	sace_31_7_3780	KSGGDALVVK	---KGTKNAI	QR--------	----------	----SWSSKN
(PRED)	sace_34_8_3770	KSGGDALVVK	---KGTKNAI	QR--------	----------	----SWSSKN
(PRED)	sace_58_71_bs00	KSGGDALVVK	---KGTKNAI	QR--------	----------	----SWSSKN
(PRED)	sace_7_7_g03880	KSGGDALVVK	---KGTKNAI	QR--------	----------	----SWSSKN
(PRED)	sace_35_7_3840	KSGGDALVVK	---KGTKNAI	QR--------	----------	----SWSSKN
(PRED)	sace_43_7_g0387	KSGGDALVVK	---KGTKNAI	QR--------	----------	----SWSSKN
(PRED)	sace_57_8_h0390	KSGGDVLVVK	---KGTKNAI	QR--------	----------	----SWSSKN
(PRED)	sace_45_7_g0389	KSGGDALVVK	---KGTKNAI	QR--------	----------	----SWSSKN
(PRED)	sace_46_8_h0391	KSGGDVLVVK	---KGTKNAI	QR--------	----------	----SWSSKN
(PRED)	sace_23_7_3860	KSGGDALVVK	---KGTKNAI	QR--------	----------	----SWSSKN
(PRED)	sace_21_7_3790	KSGGDALVVK	---KGTKNAI	QR--------	----------	----SWSSKN
(PRED)	sace_8_73_bu001	KSGGDALVVK	---KGTKNAI	QR--------	----------	----SWSSKN
(PRED)	sapa_1_8_h03820	KSGGDALVVK	---KGAKKAL	QR--------	----------	----SWSSKN
(PRED)	sapa_21_8_h0387	KSGGDALVVK	---KGAKKAL	QR--------	----------	----SWSSKN
(PRED)	sapa_20_8_h0386	KSGGDALVVK	---KGAKKAL	QR--------	----------	----SWSSKN
(PRED)	sapa_22_8_h0390	KSGGDALVVK	---KGAKKAL	QR--------	----------	----SWSSKN
(PRED)	sapa_25_8_h0387	KSGGDALVVK	---KGAKKAL	QR--------	----------	----SWSSKN
(PRED)	sapa_6_8_3750	KSGGDALVVK	---KGAKKAL	QR--------	----------	----SWSSKN
(PRED)	sapa_9_8_3720	KSGGDALVVK	---KGAKKAL	QR--------	----------	----SWSSKN
(PRED)	sapa_19_8_h0390	KSGGDALVVK	---KGAKKAL	QR--------	----------	----SWSSKN
(PRED)	sapa_24_8_h0385	KSGGDALVVK	---KGAKKAL	QR--------	----------	----SWSSKN
(PRED)	sapa_4_8_h03850	KSGGDALVVK	---KGAKKAL	QR--------	----------	----SWSSKN
(PRED)	sapa_10_8_3760	KSGGDALVVK	---KGAKKAL	QR--------	----------	----SWSSKN
(PRED)	sapa_13_8_h0382	KSGGDALVVK	---KGAKKAL	QR--------	----------	----SWSSKN
(PRED)	sapa_8_8_3750	KSGGDALVVK	---KGAKKAL	QR--------	----------	----SWSSKN
(PRED)	sapa_11_8_h0383	KSGGDALVVK	---KGAKKAL	QR--------	----------	----SWSSKN
(PRED)	sapa_5_8_3700	KSGGDALVVK	---KGAKKAL	QR--------	----------	----SWSSKN
(PRED)	sapa_16_8_h0389	KSGGDALVVK	---KGAKKAL	QR--------	----------	----SWSNKN
(PRED)	sapa_17_8_3730	KSGGDALVVK	---KGAKKAL	QR--------	----------	----SWSNKN
(PRED)	sapa_2_8_h03860	KSGGDALVVK	---KGAKKAL	QR--------	----------	----SWSNKN
(PRED)	sapa_7_8_3740	KSGGDALVVK	---KGAKKAL	QR--------	----------	----SWSNKN
(PRED)	sapa_23_8_h0385	KSGGDALVVK	---KGAKKAL	QR--------	----------	----SWSNKN
(PRED)	sapa_3_8_h03890	KSGGDALVVK	---KGAKKAL	QR--------	----------	----SWSNKN
(PRED)	sapa_18_8_3730	KSGGNALVVK	---KGAKKAL	QR--------	----------	----SWSNKN
(PRED)	sami_1_14_399	KSGGDALVFK	---KNAKNAP	QK--------	----------	----SWSDKN
(PRED)	sace_4_8_h03690	KSGGDALVVK	---KGTKNAI	QR--------	----------	----SWSSKN
(PRED)	saku_1_14_404	KSGGDALVIK	---KGAKNTL	QK--------	----------	----TWSNKN
(PRED)	sace_1_ynr070w	KSGGDALVVK	---KGTKNAI	QR--------	----------	----SWSSKN
(PRED)	sace_49_8_h0383	KSGGDALVVK	---KGTKNAI	QR--------	----------	----SWSSKN
(PRED)	saeu_1_2_b00130	KGGGDALVFK	---KGTNIGV	HEL-------	----------	----SRGGEK
(PRED)	sauv_1_7_3	KGGGDALVFK	---KGTKNGV	HEL-------	----------	----SSSDEK
(PRED)	sami_1_17_26	KGGGDALVFK	---KGTKNAL	QKM-------	----------	----QTTGRN
(PRED)	zyba_1_02055_AN	KGGGDALIFK	---KGTRRAA	AK--------	----------	----EPG-PG
(PRED)	zyba_1_07912	KGGGDALIFK	---KGTRRAA	AK--------	----------	----EPG-PG
(PRED)	zyba_2_2_b00600	KGGGDALIFK	---KGTRRAA	AK--------	----------	----EPG-PG
(PRED)	zyba_3_3_c03460	KGGGDALIFK	---KGTRRAA	AK--------	----------	----EPG-PG
(PRED)	zyba_1_04634	KGGGDTLVFK	---KGTRKAV	AR--------	----------	----EVG-PG
(PRED)	zyba_1_06675	KGGGDALVFK	---KGTRKAV	AR--------	----------	----EAG-PG
(PRED)	zyba_3_2_b02230	KGGGDALVFK	---KGTRKAV	AR--------	----------	----EAG-PG
(PRED)	zyba_2_1_a00860	KGGGDALVFK	---KGTRKAV	AR--------	----------	----EAG-PG
(PRED)	zyro_1_a04114g	KGGGDALIFK	---KGTRNAT	AK--------	----------	----KNNRPD
(PRED)	zyro_1_b14762g	TSGGEALIFK	---KGARRSL	DK--------	----------	----QKNTAQ
(PRED)	zyba_2_14_n0149	KGGGDSLIFK	---RGTKKAV	AK--------	----------	----QPP-AG
(PRED)	zyba_2_33_ag001	KGGGDSLIFK	---RGTKKAV	AK--------	----------	----HSP-AG
(PRED)	lath_1_a01914g	MGSGDTLVFK	---KGAKR--	----------	----------	-----VSPSS
(PRED)	lawa_1_23_5161	VGTGDTLVFK	---KGAKR--	----------	----------	-----VVSPS
(PRED)	klae_1_14_n0012	KSSGDALVFK	---KGTRL--	----------	----------	----RSVPKD
(PRED)	klla_1_d03432g	KSSGDALLFK	---KGTSL--	----------	----------	----NTIPKD
(PRED)	klma_1_1_a01880	KSNGDALIFK	---KGTGL--	----------	----------	----KKTLRD
(PRED)	klwi_1_33_ag001	KNSGDALVFK	---KGTRM--	----------	----------	----KKLKKD
(PRED)	teph_1_a04220	SSNANILILK	---KGIGK--	HRL-------	----------	----P-SDEE
(PRED)	vapo_1_1037_47	RKEGNILLYK	---KN-SK--	ILS-------	----------	----PFKDEE
(PRED)	pata_1_2_b05590	KGGGDHLSFI	---RGARIPD	TIL-------	----------	----NPADVA
(PRED)	wian_1_3_c04380	KGGGDHLYFK	---RGGKSSD	KIL-------	----------	----MQHG-S
(PRED)	wian_1_3_c04390	KGGGDHLYFK	---RGGKPSD	EVL-------	----------	----LSSD-A
(PRED)	wian_1_7_g01010	KGGGDHLYFK	---RGGKPSD	RIV-------	----------	----MSNE-S
(PRED)	bain_1_1_a00100	KGGGDRLMFK	---KGTSKDT	VMI-------	----------	----MSATKK
(PRED)	bain_1_17_q0038	KGGGDRLMFK	---KGTSKDT	VMI-------	----------	----MSATKK
(PRED)	bain_1_8_h00410	AANGDKLLFK	---RGGEATE	LLN-------	----------	----RLNTGA
(PRED)	caal_1_19_5759	TGGGDKLLFL	---KGKVPEH	ITL-------	----------	----PSEKKE
(PRED)	caal_11_25_y002	TGGGDKLLFL	---KGKVPEH	ITL-------	----------	----PSEKKE
(PRED)	caal_4_4_d03320	TGGGDKLLFL	---KGKVPEH	ITL-------	----------	----PSEKKE

```
(PRED) caal_12_26_z005 TGGGDKLLFL  ---KGKVPEH  ITL-------  ----------  ----PSEKKE
(PRED) caal_5_30_ad005 TGGGDKLLFL  ---KGKVPEH  ITL-------  ----------  ----PSEKKE
(PRED) caal_8_3_c03320 TGGGDKLLFL  ---KGKVPEH  ITL-------  ----------  ----PSEKKE
(PRED) caal_6_4_d03280 TGGGDKLLFL  ---KGKVPEH  ITL-------  ----------  ----PSEKKE
(PRED) caal_10_3_c0334 TGGGDKLLFL  ---KGKVPEH  ITL-------  ----------  ----PSEKKE
(PRED) caal_3_29_ac005 TGGGDKLLFL  ---KGKVPEH  ITL-------  ----------  ----PSEKKE
(PRED) caal_2_04989    TGGGDKLLFL  ---KGKVPEH  ITL-------  ----------  ----PSEKKE
(PRED) cadu_1_64350    TGGGDKLLFL  ---KGKVPEH  ITL-------  ----------  ----PSERKE
(PRED) caor_1_h02090   TGGGDKLLYL  ---RGKIPNK  IAL-------  ----------  ----PAEKQA
(PRED) capa_1_600750   TGGGDKLLYL  ---RGKIPHK  IAL-------  ----------  ----PAEKQA
(PRED) loel_1_04930    TGGGDKLLFL  ---RGKVPDK  IIL-------  ----------  ----AAKKGE
(PRED) spar_1_5_e03260 SGGGDKLLYL  ---KGKVPDH  LVN-------  ----------  ----AEEKQK
(PRED) sppa_1_7_g03160 SGGGDKLLYL  ---RGKVPDH  LAN-------  ----------  ----ASDKQQ
(PRED) catr_1_01205    TGGGDKLLFL  ---KGKVPDH  VTL-------  ----------  ----PSEKQD
(PRED) catr_1_05498    TGGGDKLLYL  ---RGKVPDH  VAL-------  ----------  ----PEEKQN
(PRED) catr_1_05971    VGGGDKLLFL  ---RGKVPDH  VTL-------  ----------  ----PSEKED
(PRED) deha_1_a03696g  SGGGDRLMFL  ---RGKVPDS  IVL-------  ----------  ----PQDKGQ
(PRED) deha_2_5_e00720 SGGGDRLMFL  ---RGKVPDS  IVL-------  ----------  ----PQDKGS
(PRED) scst_1_3_c02890 TGGGDRLLFL  ---KGKVPDH  IVL-------  ----------  ----PQDRSA
(PRED) mebi_1_8_h00300 NGGIDRLFFL  ---RGKKSTQ  IVM-------  ----------  ----SGEGAM
(PRED) lakl_1_h21010g  GSKGDSLIFK  ---RDAKAPV  VVA-------  ----------  ----AIDDE-
(PRED) caar_1_13_m0142 VPSADKMLFV  ---KGANIPS  EVI-------  ----------  -----SHIAA
(PRED) caar_1_14_n0143 VASSDKLLFI  ---KGAILPE  SLL-------  ----------  -----DLTGG
(PRED) hapo_1_1_a07220 VPSSDQLLFV  ---KGAKLPD  SLL-------  ----------  -----EATGQ
(PRED) ogpa_1_1_a01680 VPSSDQLLFV  ---KGAKLPD  SLL-------  ----------  -----EATGQ
(PRED) piku_1_96_cr001 SASADKLLFV  ---HGARIPS  SLL-------  ----------  -----DAIGF
(PRED) pime_1_4_d03240 VASSDKLLFI  ---HGASIPT  SLL-------  ----------  -----EAIGA
(PRED) pime_1_1_a12110 VASSDQLLFT  ---HGANIPI  SLF-------  ----------  -----EAIGF
(PRED) piku_1_227_hs00 PSSADKLLFI  ---RNARVSG  ALV-------  ----------  -----EHFKQ
(PRED) pime_1_5_e05800 PSSSDKLVMI  ---KNARVSK  ALI-------  ----------  -----DHFKQ
(PRED) pime_1_1_a07690 VASSDQLLFI  ---KNAKISK  RMF-------  ----------  -----EHLRQ
(PRED) debr_2_5_e03380 VVSADRMLFV  RRRRGVDLES  KLVGRNLDKP  ESTVIASTNA  QSTTPESTKS
(PRED) kopa_1_2_b10040 TSTADRLIFL  ---KANDLPV  EVA-------  ----------  -----AALNG
(PRED) kopa_2_7_g00500 TSTADRLIFL  ---KANDLPV  GVA-------  ----------  -----AALNG
(PRED) asru_1_13_m0119 NAGGDRLLFK  ---KGADVSL  VN--------  ----------  ------DEKK
(PRED) asru_1_15_o0045 KGGGERLLFK  ---KGTTESA  IA--------  ----------  ------SKIV
(PRED) wian_1_1_a02920 EVKGDKLLFK  ---KGKVPDD  VV--------  ----------  ------QPMP
(PRED) wian_1_1_a02930 EGSGDRLLFK  ---KGKLPDE  FV--------  ----------  ------RSSS

                       .........1110.........1120.........1130.........1140.........1150
(PRED) asac_1_6_f03560 RSAAGAA---  ---ESKMIAS  -----T----  ------VATE  DVFDD-----
(PRED) ergo_1_abr125c  RNVSGAA---  ---ESKMVAS  -----T----  ------VGTD  DVFDG-----
(PRED) ercy_1_3604     SSPGTTS---  ---EAKYMVS  DDSNLP----  ------VTGA  GIFEG-----
(PRED) cagl_1_i04862g  --GELHEI--  ---DTKEKFS  S-RSGSST--  ------TSED  EIFEE-----
(PRED) kaaf_1_c00830   --GETSPS--  ---DLKERYS  T-SS-SK---  -------GED  IQFED-----
(PRED) kana_1_k01350   A-DGASDL--  -----KDKYS  S-NT-TST--  ------DD-D  ETFAD-----
(PRED) saar_1_2_b02590 P-DNINDI--  ---DAKEQFS  S-ES-SGA--  -------N-D  EVFDD-----
(PRED) sace_1_ydr011w  P-DNVNDI--  ---DAKEQFS  S-ES-SGA--  -------N-D  EVFDD-----
(PRED) sace_16_1_a0238 P-DNVNDI--  ---DAKEQFS  S-ES-SGA--  -------N-D  EVFDD-----
(PRED) sace_45_1_a0242 P-DNVNDI--  ---DAKEQFS  S-ES-SGA--  -------N-D  EVFDD-----
(PRED) sace_48_1_a0238 P-DNVNDI--  ---DAKEQFS  S-ES-SGA--  -------N-D  EVFDD-----
(PRED) sace_60_4_d0244 P-DNVNDI--  ---DAKEQFS  S-ES-SGA--  -------N-D  EVFDD-----
(PRED) sace_52_1_a0240 P-DNVNDI--  ---DAKEQFS  S-ES-SGA--  -------N-D  EVFDD-----
(PRED) sace_46_1_a0240 P-DNVNDI--  ---DAKEQFS  S-ES-SGA--  -------N-D  EVFDD-----
(PRED) sace_25_1_a0240 P-DNVNDI--  ---DAEEQFS  S-ES-SGA--  -------N-D  EVFDD-----
(PRED) sace_24_1_2300  P-DNVNDI--  ---DAKEQFS  S-ES-SGA--  -------N-D  EVFDD-----
(PRED) sace_47_1_a0240 P-DNVNDI--  ---DAKEQFS  S-ES-SGA--  -------N-D  EVFDD-----
(PRED) sace_7_1_a02410 P-DNVNDI--  ---DAKEQFS  S-ES-SGA--  -------N-D  EVFDD-----
(PRED) sace_59_110_df0 P-DNVNDI--  ---DAKEQFS  S-ES-SGA--  -------N-D  EVFDD-----
(PRED) sace_56_1_a0202 P-DNVNDI--  ---DAKEQFS  S-ES-SGA--  -------N-D  EVFDD-----
(PRED) sace_40_1_a0239 P-DNVNDI--  ---DAKEQFS  S-ES-SGA--  -------N-D  EVFDD-----
(PRED) sace_15_1_a0242 P-DNVNDI--  ---DAKEQFS  S-ES-SGA--  -------N-D  EVFDD-----
(PRED) sace_37_1_a0243 P-DNVNDI--  ---DAKEQFS  S-ES-SGA--  -------N-D  EVFDD-----
(PRED) sace_9_1_a02440 P-DNVNDI--  ---DAKEQFS  S-ES-SGA--  -------N-D  EVFDD-----
(PRED) sace_22_1_2300  P-DNVNDI--  ---DAKEQFS  S-ES-SGA--  -------N-D  EVFDD-----
(PRED) sace_29_1_2290  P-DNVNDI--  ---DAKEQFS  S-ES-SGA--  -------N-D  EVFDD-----
(PRED) sace_34_1_2320  P-DNVNDI--  ---DAKEQFS  S-ES-SGA--  -------N-D  EVFDD-----
(PRED) sace_58_25_y007 P-DNVNDI--  ---DAKEQFS  S-ES-SGA--  -------N-D  EVFDD-----
(PRED) sace_23_1_2290  P-DNVNDI--  ---DAKEQFS  S-ES-SGA--  -------N-D  EVFDD-----
(PRED) sace_6_120_dp00 P-DNVNDI--  ---DAKEQFS  S-ES-SGA--  -------N-D  EVFDD-----
(PRED) sace_57_1_a0241 P-DNVNDI--  ---DAKEQFS  S-ES-SGA--  -------N-D  EVFDD-----
(PRED) sace_17_1_a0241 P-DNVNDI--  ---DAKEQFS  S-ES-SGA--  -------N-D  EVFDD-----
(PRED) sace_21_1_2310  P-DNVNDI--  ---DAKEQFS  S-KS-SGA--  -------N-D  EVFDD-----
(PRED) sace_49_1_a0246 P-DNVNDI--  ---DAKEQFS  S-ES-SGA--  -------N-D  EVFDD-----
(PRED) sace_8_2_b02430 P-DNVNDI--  ---DAKEQFS  S-ES-SGA--  -------N-D  EVFDD-----
```

(PRED)	sace_31_1_2300	P-DNVNDI--	---DAKEQFS	S-ES-SGA--	-------N-D	EVFDD-----
(PRED)	sace_50_1_a0241	P-DNVNDI--	---DAKEQFS	S-ES-SGA--	-------N-D	EVFDD-----
(PRED)	sace_4_1_a02360	P-DNVNDI--	---DAKEQFS	S-ES-SGA--	-------N-D	EVFDD-----
(PRED)	sace_2_1_a02390	P-DNVNDI--	---DAKEQFS	S-ES-SGA--	-------N-D	EVFDD-----
(PRED)	sace_5_33_ag005	P-DNVNDI--	---DAKEQFS	S-ES-SGA--	-------N-X	EVFDD-----
(PRED)	sapa_11_1_a0247	P-DNVNDI--	---DAKEQFS	S-ES-SGA--	-------N-D	EVFDE-----
(PRED)	sapa_25_1_a0246	P-DNVNDI--	---DAKEQFS	S-ES-SGA--	-------N-D	EVFDE-----
(PRED)	sapa_4_1_a02470	P-DNVNDI--	---DAKEQFS	S-ES-SGA--	-------N-D	EVFDE-----
(PRED)	sapa_5_1_2350	P-DNVNDI--	---DAKEQFS	S-ES-SGA--	-------N-D	EVFDE-----
(PRED)	sapa_9_1_2360	P-DNVNDI--	---DAKEQFS	S-ES-SGA--	-------N-D	EVFDE-----
(PRED)	sapa_14_1_a0244	P-DNVNDI--	---DAKEQFS	S-ES-SGA--	-------N-D	EVFDE-----
(PRED)	sapa_8_1_2350	P-DNVNDI--	---DAKEQFS	S-ES-SGA--	-------N-D	EVFDE-----
(PRED)	sapa_17_1_2380	P-DNVNDI--	---DAKEQFS	S-ES-SGA--	-------N-D	EVFDE-----
(PRED)	sapa_7_1_2370	P-DNVNDI--	---DAKEQFS	S-ES-SGA--	-------N-D	EVFDE-----
(PRED)	sapa_2_1_a02460	P-DNVNDI--	---DAKEQFS	S-ES-SGA--	-------N-D	EVFDE-----
(PRED)	sapa_23_1_a0248	P-DNVNDI--	---DAKEQFS	S-ES-SGA--	-------N-D	EVFDE-----
(PRED)	sapa_3_1_a02470	P-DNVNDI--	---DAKEQFS	S-ES-SGA--	-------N-D	EVFDE-----
(PRED)	sapa_18_1_2390	P-DNVNDI--	---DAKEQFS	S-ES-SGA--	-------N-D	EVFDE-----
(PRED)	sami_1_4_244	P-ENINDI--	---DAKEQFS	S-ES-SGA--	-------N-N	EVFDE-----
(PRED)	saku_1_4_262	P-DNINDI--	---DAKEQFS	S-ES-SGA--	-------N-D	EVFDE-----
(PRED)	saba_1_58_bf002	P-DNINDL--	---DAKEQFS	S-ES-SGA--	-------N-D	DVFEE-----
(PRED)	saeu_1_4_d02400	P-DNVNDI--	---DAKEQFS	S-ES-S-A--	-------N-D	DVFEE-----
(PRED)	naca_1_e01640	AED-SNSF--	---EMKERVS	S-GN-NSL--	------VE-E	DAFEE-----
(PRED)	nada_1_g01850	TSDTANSL--	---ELKERFS	S-SN-SSE--	------VDLA	GAFEN-----
(PRED)	naca_1_e01630	S-DNSNDT--	---EIKDKFT	S-TT-IST--	-------DEE	GAFEE-----
(PRED)	nada_1_g01840	S-ESSNNV--	---DLKEMYY	S-SN--SA--	-------EGE	AVFEG-----
(PRED)	kaaf_1_c00820	NHSSEN-L--	---EPQEKYS	S-GS-TAE--	------ESTD	AIFEE-----
(PRED)	teph_1_m00640	THDKLNLS--	---ESKEKYM	T-GS-S-S--	------SEDD	GTFEG-----
(PRED)	vapo_1_1036_28	SADSYNVA--	---DVKEKYS	T-GE-SFS--	------DEHD	NIFEG-----
(PRED)	tebl_1_i01760	DVDSQSKV-F	PVSSSTAVST	A-SSQEGA--	------VEPD	S-FKD-----
(PRED)	tode_1_d04040	TADNMNLA--	---DVKEKLS	SGESSNSN--	------FPEG	DDFED-----
(PRED)	naca_1_e01650	TASSSSD---	---------A	SNEDKVSD--	------PNAG	E-FEN-----
(PRED)	tebl_1_g02820	ESNAVINI--	--SENKEKFT	PAEEAD----	---------D	TNLEG-----
(PRED)	lakl_1_c11616g	S-DNELTI--	--ADAKKVSS	GGSSTETA--	----------	-VFEG-----
(PRED)	saar_1_8_h03780	DEETANLS-I	TAQDMKEIVL	KNGS-S----	------LHSE	--FEG-----
(PRED)	sace_14_7_g0015	DEENLNAS-I	ATQDMKEIAS	SNDD-S----	------TSAD	--FEG-----
(PRED)	sace_15_7_g0387	DEENLNAS-I	ATQDMKEIAS	SNDD-S----	------TSAD	--FEG-----
(PRED)	sace_24_8_3780	DEENLNAS-I	ATQDMKEIAS	SNDD-S----	------TSAD	--FEG-----
(PRED)	sace_40_8_h0383	DEENLNAS-I	ATQDMKEIAS	SNDD-S----	------TSAD	--FEG-----
(PRED)	sace_6_169_fm00	DEENLNAS-I	ATQDMKEIAS	SNDD-S----	------TSAD	--FEG-----
(PRED)	sace_19_7_3840	DEENLNAS-I	ATQDMKEIAS	SNDD-S----	------TSAD	--FEG-----
(PRED)	sace_32_7_3770	DEENLNAS-I	ATQDMKEIAS	SNDD-S----	------TSAD	--FEG-----
(PRED)	sace_56_17_q011	DEENLNAS-I	ATQDMKEIAS	SNDD-S----	------TSAD	--FEG-----
(PRED)	sace_5_78_bz001	DEENLNAS-I	ATQDMKEIAS	SNDD-S----	------TSAD	--FEG-----
(PRED)	sace_2_8_h03860	DEENLNAS-I	ATQDMKEIAS	SNDD-S----	------TSAD	--FEG-----
(PRED)	sace_53_29_ac00	DEENLNAS-I	ATQDMKEIAS	SNDD-S----	------TSAD	--FEG-----
(PRED)	sace_17_7_g0393	DEENLNAS-I	ATQDMKEIAS	SNDD-S----	------TSAD	--FEG-----
(PRED)	sace_25_7_g0388	DEENLNAS-I	ATQDMKEIAS	SNDD-S----	------TSAD	--FEG-----
(PRED)	sace_37_7_g0385	DEENLNAS-I	ATQDMKEIAS	SNDD-S----	------TSAD	--FEG-----
(PRED)	sace_9_7_g00180	DEENLNAS-I	ATQDMKEIAS	SNDD-S----	------TSAD	--FEG-----
(PRED)	sace_60_6_f0335	DEENLNAS-I	ATQDMKEIAS	SNDD-S----	------TSAD	--FEG-----
(PRED)	sace_59_336_lx0	DEENLNAS-I	ATQDMKEIAS	SNDD-S----	------TSAD	--FEG-----
(PRED)	sace_31_7_3780	DEENLNAS-I	ATQDMKEIAS	SNDD-S----	------TSAD	--FEG-----
(PRED)	sace_34_8_3770	DEENLNAS-I	ATQDMKEIAS	SNDD-S----	------TSAD	--FEG-----
(PRED)	sace_58_71_bs00	DEENLNAS-I	ATQDMKEIAS	SNDD-S----	------TSAD	--FEG-----
(PRED)	sace_7_7_g03880	DEENLNAS-I	ATQDMKEIAS	SNDD-S----	------TSAD	--FEG-----
(PRED)	sace_35_7_3840	DEENLNAS-I	ATQDMKEIAS	SNDD-S----	------TSAD	--FEG-----
(PRED)	sace_43_7_g0387	DEENLNAS-I	ATQDMKEIAS	SNDD-S----	------TSAD	--FEG-----
(PRED)	sace_57_8_h0390	DEENLNAS-I	ATQDMKEIAS	SNDD-S----	------TSAD	--FEG-----
(PRED)	sace_45_7_g0389	DEENLNAS-I	ATQDMKEIAS	SNDD-S----	------TSAD	--FEG-----
(PRED)	sace_46_8_h0391	DEENLNAS-I	ATQDMKEIAS	SNDD-S----	------TSAD	--FEG-----
(PRED)	sace_23_7_3860	DEENLNAS-I	ATQDTKEIAS	SNDD-S----	------TSAD	--FEG-----
(PRED)	sace_21_7_3790	DEENLKAS-I	ATQDMKEIAS	SNDD-S----	------TSAD	--FEG-----
(PRED)	sace_8_73_bu001	DEENLNAS-I	ATQDMKEIAS	SNDD-S----	------TSAD	--FEG-----
(PRED)	sapa_1_8_h03820	DEENLNVS-I	ATQDMKEIAS	SNGD-S----	------TQAD	--FEG-----
(PRED)	sapa_21_8_h0387	DEENLNVS-I	ATQDMKEIAS	SNGD-S----	------TQAD	--FEG-----
(PRED)	sapa_20_8_h0386	DEENLNVS-I	ATQDMKEIAS	SNGD-S----	------TQAD	--FEG-----
(PRED)	sapa_22_8_h0390	DEENLNVS-I	ATQDMKEIAS	SNGD-S----	------TQAD	--FEG-----
(PRED)	sapa_25_8_h0387	DEENLNVS-I	ATQDMKEIAS	SNGD-S----	------TQAD	--FEG-----
(PRED)	sapa_6_8_3750	DEENLNVS-I	ATQDMKEIAS	SNGD-S----	------TQAD	--FEG-----
(PRED)	sapa_9_8_3720	DEENLNVS-I	ATQDMKEIAS	SNGD-S----	------TQAD	--FEG-----
(PRED)	sapa_19_8_h0390	DEENLNVS-I	ATQDMKEIAS	SNGD-S----	------TQAD	--FEG-----
(PRED)	sapa_24_8_h0385	DEENLNVS-I	ATQDMKEIAS	SNGD-S----	------TQAD	--FEG-----
(PRED)	sapa_4_8_h03850	DEENLNVS-I	ATQDMKEIAS	SNGD-S----	------TQAD	--FEG-----
(PRED)	sapa_10_8_3760	DEENLNVS-I	ATQDMKEIAS	SNGD-S----	------TQAD	--FEG-----

(PRED)	sapa_13_8_h0382	DEENLNVS-I	ATQDMKEIAS	SNGD-S----	------TQAD	--FEG-----
(PRED)	sapa_8_8_3750	DEENLNVS-I	ATQDMKEIAS	SNGD-S----	------TQAD	--FEG-----
(PRED)	sapa_11_8_h0383	DEENLNVS-I	ATQDMKEIAS	SNGD-S----	------TQAD	--FEG-----
(PRED)	sapa_5_8_3700	DEENLNVS-I	ATQDMKEIAS	SNGD-S----	------TQAD	--FEG-----
(PRED)	sapa_16_8_h0389	DEENLNVS-I	ATQDMKEIVS	SNGD-S----	------THAD	--FEG-----
(PRED)	sapa_17_8_3730	DEENLNVS-I	ATQDMKEIVS	SNGD-S----	------THAD	--FEG-----
(PRED)	sapa_2_8_h03860	DEENLNVS-I	ATQDMKEIVS	SNGD-S----	------THAD	--FEG-----
(PRED)	sapa_7_8_3740	DEENLNVS-I	ATQDMKEIVS	SNGD-S----	------THAD	--FEG-----
(PRED)	sapa_23_8_h0385	DEENLNVS-I	ATQDMKEIVS	SNGD-S----	------THAD	--FEG-----
(PRED)	sapa_3_8_h03890	DEENLNVS-M	ATQDMKEIVS	SNGD-S----	------THAD	--FEG-----
(PRED)	sapa_18_8_3730	DEENLNVS-I	ATQDMKEIVS	SNGD-S----	------THAD	--FKG-----
(PRED)	sami_1_14_399	DEENLSTS-I	ATQDMKDVAS	SNGD-S----	------TYSE	--FDG-----
(PRED)	sace_4_8_h03690	DEENLNAS-I	ATQDMKEIAS	SNDD-S----	------TSAD	--FEG-----
(PRED)	saku_1_14_404	DEENINMS-I	TTQDNKDIAS	SNGD-S----	------THTE	--FEG-----
(PRED)	sace_1_ynr070w	DEENLNAS-I	ATQDMKEIAS	SNDD-S----	------TSAD	--FEG-----
(PRED)	sace_49_8_h0383	DEENLNAS-I	ATQDMKEIAS	SNDD-S----	------TSAD	--FEG-----
(PRED)	saeu_1_2_b00130	DEENLNLS-L	TAQDLKDMVS	SSGA-NKH--	------THAE	--FDG-----
(PRED)	sauv_1_7_3	DEENLNLS-L	TAQDLKDMVS	SSGA-NK---	-------HAE	--FDG-----
(PRED)	sami_1_17_26	DEEVFTSS-L	TAQDMKDMVS	SSDF-S----	------GKDD	--FEG-----
(PRED)	zyba_1_02055_AN	DLEALDQM-K	LQGDAREKFS	SGSDLTRGDA	TDVGAVDSSD	EVFEG-----
(PRED)	zyba_1_07912	DLEALDQM-K	LQGDAREKFS	SGSDLMRGDA	TDAGAVDSSD	EVFEG-----
(PRED)	zyba_2_2_b00600	DLEALDQM-K	LQGDAREKFS	SGSDLTRGDA	TDAGAVDSSD	EVFEG-----
(PRED)	zyba_3_3_c03460	DLEALDQM-K	LQGDAREKFS	SGSDLMRGDA	TDAGAVDSSD	EVFEG-----
(PRED)	zyba_1_04634	DIEALEDM-N	LQIDGRENFS	NDSDVTRKD-	-D----QLFD	KEFQS-----
(PRED)	zyba_1_06675	DIEALDDM-N	LHTDARENFS	NDSDVTRKD-	-D----QLFD	KEFQT-----
(PRED)	zyba_3_2_b02230	DIEALDDM-N	LHTDARENFS	NDSDVTRKD-	-D----QLFD	KEFQT-----
(PRED)	zyba_2_1_a00860	DIEALDDM-N	LHTDARENFS	NDSDVTRKD-	-D----QLFD	KEFQT-----
(PRED)	zyro_1_a04114g	DLESMDQM-N	VQ-DVKDKYS	SGSDITPNN-	-D----QEIE	QSFKD-----
(PRED)	zyro_1_b14762g	DLEAMDQM-N	MQ-EVREKIS	SNSSDVTRN-	-D----EQNS	GAIEG-----
(PRED)	zyba_2_14_n0149	DLESMDHL-T	MQADAREKFS	-GSEV-----	------EESN	ASFSG-----
(PRED)	zyba_2_33_ag001	DLESVDHV-T	MQADAREKFS	-GSEI-----	------QESD	ISFSG-----
(PRED)	lath_1_a01914g	DEESREKS-L	TMSEAKKNLD	SNASTSG---	-------SSA	EALQG-----
(PRED)	lawa_1_23_5161	DEESRDKS-I	TMSEAKRNAD	SSASCS----	-------SNS	DALQA-----
(PRED)	klae_1_14_n0012	VESNVNSS-E	SVSKTVDSSK	GSDEAA----	----------	-LFAD-----
(PRED)	klla_1_d03432g	EESNVNST-D	SITKTTDSSS	RSDDPA----	----------	-LFAD-----
(PRED)	klma_1_1_a01880	SESNIDPS-E	STNQTADSFA	KSDDPA----	----------	-LFAD-----
(PRED)	klwi_1_33_ag001	EESHVNSS-D	SITKTAGSSE	PEESAA----	----------	-IFAN-----
(PRED)	teph_1_a04220	AQP----L-E	YSEETNSIGS	NYNISK----	------KTTT	TLFRG-----
(PRED)	vapo_1_1037_47	STPDVGEV-S	LKHHTTATSS	NNETIV----	------HEKD	EVFEG-----
(PRED)	pata_1_2_b05590	PSSDVENGPL	SEKDTTENEV	DNAKKP----	-------VSK	AVAEDGE---
(PRED)	wian_1_3_c04380	KNSDIETG-V	TNS--QDNDL	KSSNSS----	-------DEL	DVFEG-----
(PRED)	wian_1_3_c04390	QAADIEAG-T	SDRPIQDHDL	KSGSSS----	-------TDN	DVFEG-----
(PRED)	wian_1_7_g01010	KQSDIETG-N	KDQSVQDNK-	SSLDDS----	-------VEN	EAFEG-----
(PRED)	bain_1_1_a00100	T-SDVENG--	N-NAMFDNAP	Q-----RIST	GA-----R--	KVFED-----
(PRED)	bain_1_17_q0038	T-SDVENG--	E-IAMFDNAP	Q-----RIST	GA-----R--	KVFED-----
(PRED)	bain_1_8_h00410	R-DDPEGR--	TADAIHTLLP	ESDVDSQASS	GV-----KNQ	KAFQS-----
(PRED)	caal_1_19_5759	--EDIES---	-GGNSDTTAT	SNGTLSQGKS	-EE----KAA	IADDG-----
(PRED)	caal_11_25_y002	--EDIES---	-GGNSDTTAT	SNGTLSQGKS	-EE----KAA	IVDDG-----
(PRED)	caal_4_4_d03320	--EDIES---	-GGNSDTTAT	SNGTLSQGKS	-EE----KAA	IVDDG-----
(PRED)	caal_12_26_z005	--EDIES---	-GGNSDTTAT	SNGTLSQGKS	-EE----KAA	IVDDG-----
(PRED)	caal_5_30_ad005	--EDIES---	-GGNSDTTAT	SNGTLSQGKS	-EE----KAA	IADDG-----
(PRED)	caal_8_3_c03320	--EDIES---	-GGNSDTTAT	SNGTLSQGKS	-EE----KAA	IADDG-----
(PRED)	caal_6_4_d03280	--EDIES---	-GGNSDTTAT	SNGTLSQGKS	-EE----KAA	IADDG-----
(PRED)	caal_10_3_c0334	--EDIES---	-GGNSDTTAT	SNGTLSQGKS	-EE----KAA	IADDG-----
(PRED)	caal_3_29_ac005	--EDIES---	-GGNSDTTAT	SNGTLSQGKS	-EE----KAA	IVDDG-----
(PRED)	caal_2_04989	--EDIES---	-GGNSDTTAT	SNGTLSQGKS	-EE----KAA	IADDG-----
(PRED)	cadu_1_64350	--EDIES---	-GG--DTTAT	SNGTLSQGKS	DDE----KGA	IVDEG-----
(PRED)	caor_1_h02090	--GDIEE---	-G------PA	MNDLDDR---	-EV----KVN	ANDQD-----
(PRED)	capa_1_600750	--GDIEE---	-G------PA	MNDLDDR---	-EV----KVG	TNDQD-----
(PRED)	loel_1_04930	--GDIEE---	-G------PA	MEGLDDR---	-EV----KVD	LGDDE-----
(PRED)	spar_1_5_e03260	--RDLEG---	-G-------P	ATNDIEKVAQ	NTN----NSD	L--EE-----
(PRED)	sppa_1_7_g03160	--RDLEG---	-G-------P	AVGDLEKVPG	QAN----DSD	L--DD-----
(PRED)	catr_1_01205	--GDIESS--	PGQTTSSSQL	EKSP-----S	KT--------	NKNTA-----
(PRED)	catr_1_05498	--GDIESA--	-GQRSGSTQL	EK-PFS---S	KEDTL-GQCE	KKDAT-----
(PRED)	catr_1_05971	--EDVESS--	-GQTSGSSEL	EKVPAANNQS	KVDALGGSTE	NKNVG-----
(PRED)	deha_1_a03696g	TPGDLE-T--	SSSSSNTLEK	T-NVNSEDKL	----------	KIFKN-----
(PRED)	deha_2_5_e00720	TSGDLE-T--	SSSSSNTLEK	TNNVNSEDKL	----------	KIFKN-----
(PRED)	scst_1_3_c02890	SPDDEEGL--	SGKYDNELGS	ETTAEKHAKN	----------	NVFED-----
(PRED)	mebi_1_8_h00300	--EDEES---	-GPATSANLE	KSNTSP----	--------AE	ILDD------
(PRED)	lakl_1_h21010g	--ESLNTN--	---NNPLIVT	TNSSTKD---	-------NND	DVFDG-----
(PRED)	caar_1_13_m0142	AAMIDQK---	--DLESIGG-	SVND------	-------F-N	EVKESHRATA
(PRED)	caar_1_14_n0143	GGV--SS---	--DLEATSGK	SSTD------	----------	DISESQRATA
(PRED)	hapo_1_1_a07220	ARP--KS---	--DEESAGG-	SHTD------	-------TKS	EIDEAQHSTA
(PRED)	ogpa_1_1_a01680	ARP--KS---	--DEESAAG-	SRTD------	-------TKS	EIDEAQHSTA
(PRED)	piku_1_96_cr001	SETNDND---	--LEAQTVLK	EKGDI-----	------QIET	DVVVSQSSHD

```
(PRED) pime_1_4_d03240 GE---ED---  --PEKTVGVD  AKSEG-----  ------KVTD  DIIESQHSAA
(PRED) pime_1_1_a12110 TD--------  ---EEIPGAE  NKED------  ------QISD  EVLESRRSLA
(PRED) piku_1_227_hs00 IAPADGE---  KIAEGDQEET  GGAN------  ---GTPLSFE  KDMESQPPDT
(PRED) pime_1_5_e05800 IAPADDEIHK  HVIESDTSFE  SNDDVLQKDL  EKMGADLADD  SNAINQTETA
(PRED) pime_1_1_a07690 LAAEDDN---  ---EVDLEKH  EDEDFL----  ------VTVD  SLTNSSHEGN
(PRED) debr_2_5_e03380 DSTNTQSTTP  RSANAGSAII  SSTNTQSVKP  ENTNAQSTMI  PSAHSQNTFT
(PRED) kopa_1_2_b10040 SASSN-----  ---DEETGQD  TSLN------  ----------  EKYELERDKS
(PRED) kopa_2_7_g00500 SASSN-----  ---DEETGQD  TSLN------  ----------  EKYEMERDKS
(PRED) asru_1_13_m0119 KASDIESGSS  GTVNK-LEED  EKNN------  ---------A  ENFEN-----
(PRED) asru_1_15_o0045 K-DDIESGER  LPSDKIIEKN  ESSD------  ---------D  ITFND-----
(PRED) wian_1_1_a02920 TH-DVESGNV  QEDSLV---P  IPHR------  ---------L  LKDEK-----
(PRED) wian_1_1_a02930 SSTDEEAGPV  -DDNLVRTRS  TKKN------  ---------F  LKDED-----

                       .........1160.........1170.........1180.........1190.........1200
(PRED) asac_1_6_f03560 ---------LK  NDDVFIWRDI  SFSITHNGDK  -KKLLDQITG  YCIPGTLTAL
(PRED) ergo_1_abr125c  ---------LK  NDDVFIWRDI  SFSITHNGDK  -KKLLDQITG  YCIPGTLTAL
(PRED) ercy_1_3604     ---------LK  NEDVFLWKNV  SYTIPYKGTK  -RKLLDEVSG  YCIPGTLTAL
(PRED) cagl_1_i04862g  ---------LE  SKGIFIWRNV  CYTIPYDGGM  -RQLLDNVSG  FCKPGTLTAL
(PRED) kaaf_1_c00830   ---------LK  SKGVFIWKDV  CYTIPYDGGQ  -RMLLDHVSG  FCKPGTLTAL
(PRED) kana_1_k01350   ---------MK  ATGVFIWRDV  CFTIPYDGGQ  -RRLLDNVSG  YCIPGTMTAL
(PRED) saar_1_2_b02590 ---------LE  AKGVFIWQNV  CFTIPYEGST  -RMLLDNVSG  YCIPGTMTAL
(PRED) sace_1_ydr011w  ---------LE  AKGVFIWKDV  CFTIPYEGGK  -RMLLDNVSG  YCIPGTMTAL
(PRED) sace_16_1_a0238 ---------LE  AKGVFIWKDV  CFTIPYEGGK  -RMLLDNVSG  YCIPGTMTAL
(PRED) sace_45_1_a0242 ---------LE  AKGVFIWKDV  CFTIPYEGGK  -RMLLDNVSG  YCIPGTMTAL
(PRED) sace_48_1_a0238 ---------LE  AKGVFIWKDV  CFTIPYEGGK  -RMLLDNVSG  YCIPGTMTAL
(PRED) sace_60_4_d0244 ---------LE  AKGVFIWKDV  CFTIPYEGGK  -RMLLDNVSG  YCIPGTMTAL
(PRED) sace_52_1_a0240 ---------LE  AKGVFIWKDV  CFTIPYEGGK  -RMLLDNVSG  YCIPGTMTAL
(PRED) sace_46_1_a0240 ---------LE  AKGVFIWKDV  CFTIPYEGGK  -RMLLDNVSG  YCIPGTMTAL
(PRED) sace_25_1_a0240 ---------LE  AKGVFIWKDV  CFTIPYEGGK  -RMLLDNVSG  YCIPGTMTAL
(PRED) sace_24_1_2300  ---------LE  AKGVFIWKDV  CFTIPYEGGK  -RMLLDNVSG  YCIPGTMTAL
(PRED) sace_47_1_a0240 ---------LE  AKGVFIWKDV  CFTIPYEGGK  -RMLLDNVSG  YCIPGTMTAL
(PRED) sace_7_1_a02410 ---------LE  AKGVFIWKDV  CFTIPYEGGK  -RMLLDNVSG  YCIPGTMTAL
(PRED) sace_59_110_df0 ---------LE  AKGVFIWKDV  CFTIPYEGGK  -RMLLDNVSG  YCIPGTMTAL
(PRED) sace_56_1_a0202 ---------LE  AKGVFIWKDV  CFTIPYEGGK  -RMLLDNVSG  YCIPGTMTAL
(PRED) sace_40_1_a0239 ---------LE  AKGVFIWKDV  CFTIPYEGGK  -RMLLDNVSG  YCIPGTMTAL
(PRED) sace_15_1_a0242 ---------LE  AKGVFIWKDV  CFTIPYEGGK  -RMLLDNVSG  YCIPGTMTAL
(PRED) sace_37_1_a0243 ---------LE  AKGVFIWKDV  CFTIPYEGGK  -RMLLDNVSG  YCIPGTMTAL
(PRED) sace_9_1_a02440 ---------LE  AKGVFIWKDV  CFTIPYEGGK  -RMLLDNVSG  YCIPGTMTAL
(PRED) sace_22_1_2300  ---------LE  AKGVFIWKDV  CFTIPYEGGK  -RMLLDNVSG  YCIPGTMTAL
(PRED) sace_29_1_2290  ---------LE  AKGVFIWKDV  CFTIPYEGGK  -RMLLDNVSG  YCIPGTMTAL
(PRED) sace_34_1_2320  ---------LE  AKGVFIWKDV  CFTIPYEGGK  -RMLLDNVSG  YCIPGTMTAL
(PRED) sace_58_25_y007 ---------LE  AKGVFIWKDV  CFTIPYEGGK  -RMLLDNVSG  YCIPGTMTAL
(PRED) sace_23_1_2290  ---------LE  AKGVFIWKDV  CFTIPYEGGK  -RMLLDNVSG  YCIPGTMTAL
(PRED) sace_6_120_dp00 ---------LE  AKGVFIWKDA  CFTIPYEGGK  -RMLLDNVSG  YCIPGTMTAL
(PRED) sace_57_1_a0241 ---------LE  AKGVFIWKDV  CFTIPYEGGK  -RMLLDNVSG  YCIPGTMTAL
(PRED) sace_17_1_a0241 ---------LE  AKGVFIWKDA  CFTIPYEGGK  -RMLLDNVSG  YCIPGTMTAL
(PRED) sace_21_1_2310  ---------LE  AKGVFIWKDV  CFTIPYEGGK  -RMLLDNVSG  YCIPGTMTAL
(PRED) sace_49_1_a0246 ---------LE  AKGVFIWKDV  CFTIPYEGGK  -RMLLDNVSG  YCIPGTMTAL
(PRED) sace_8_2_b02430 ---------LE  AKGVFIWKDV  CFTIPYEGGK  -RMLLDNVSG  YCIPGTMTAL
(PRED) sace_31_1_2300  ---------LE  AKGVFIWKDV  CFTIPYEGGK  -RMLLDNVSG  YCIPGTMTAL
(PRED) sace_50_1_a0241 ---------LE  AKGVFIWKDV  CFTIPYEGGK  -RMLLDNVSG  YCIPGTMTAL
(PRED) sace_4_1_a02360 ---------LE  AKGVFIWKDV  CFTIPYEGGK  -RMLLDNVSG  YCIPGTMTAL
(PRED) sace_2_1_a02390 ---------LE  AKGVFIWKDV  CFTIPYEGGK  -RMLLDNVSG  YCIPGTMTAL
(PRED) sace_5_33_ag005 ---------LE  AKGVFIWKDV  CFTIPYEGGX  -RMLLDNVSG  YCIPGTMTAL
(PRED) sapa_11_1_a0247 ---------LE  AKGVFIWKDV  CFTIPYEGGK  -RMLLDNVSG  YCIPGTMTAL
(PRED) sapa_25_1_a0246 ---------LE  AKGVFIWKDV  CFTIPYEGGK  -RMLLDNVSG  YCIPGTMTAL
(PRED) sapa_4_1_a02470 ---------LE  AKGVFIWKDV  CFTIPYEGGK  -RMLLDNVSG  YCIPGTMTAL
(PRED) sapa_5_1_2350   ---------LE  AKGVFIWKDV  CFTIPYEGGK  -RMLLDNVSG  YCIPGTMTAL
(PRED) sapa_9_1_2360   ---------LE  AKGVFIWKDV  CFTIPYEGGK  -RMLLDNVSG  YCIPGTMTAL
(PRED) sapa_14_1_a0244 ---------LE  AKGVFIWKDV  CFTIPYEGGK  -RMLLDNVSG  YCIPGTMTAL
(PRED) sapa_8_1_2350   ---------LE  AKGVFIWKDV  CFTIPYEGGK  -RMLLDNVSG  YCIPGTMTAL
(PRED) sapa_17_1_2380  ---------LE  AKGVFIWKDV  CFTIPYEGGK  -RMLLDNVSG  YCIPGTMTAL
(PRED) sapa_7_1_2370   ---------LE  AKGVFIWKDV  CFTIPYEGGK  -RMLLDNVSG  YCIPGTMTAL
(PRED) sapa_2_1_a02460 ---------LE  AKGVFIWKDV  CFTIPYEGGK  -RMLLDNVSG  YCIPGTMTAL
(PRED) sapa_23_1_a0248 ---------LE  AKGVFIWKDV  CFTIPYEGGK  -RMLLDNVSG  YCIPGTMTAL
(PRED) sapa_3_1_a02470 ---------LE  AKGVFIWKDV  CFTIPYEGGK  -RMLLDNVSG  YCIPGTMTAL
(PRED) sapa_18_1_2390  ---------LE  AKGVFIWKDV  CFTIPYEGGK  -RMLLDNVSG  YCIPGTMTAL
(PRED) sami_1_4_244    ---------LE  AKGVFIWKDV  CFTIPYEGGK  -RMLLDNVSG  YCIPGTMTAL
(PRED) saku_1_4_262    ---------LE  AKGVFIWKDV  CFTIPYEGST  -RMLLDNVSG  YCVPGTMTAL
(PRED) saba_1_58_bf002 ---------LE  AKGVFIWKDV  CFTIPYEGST  -RMLLDNVSG  YCIPGTMTAL
(PRED) saeu_1_4_d02400 ---------LE  AKGVFIWKDV  CFTIPYEGST  -RMLLDNVSG  YCIPGTMTAL
(PRED) naca_1_e01640   ---------LE  SKGVFIWRDV  CYTIPYDGGQ  -RMLLDHVSG  YCIPGTMTAL
(PRED) nada_1_g01850   ---------LK  STAVFAWKEV  CFTIPYDGGQ  -RMLLDHVSG  YCVPGTMTAL
(PRED) naca_1_e01630   ---------LE  SKGVFLWKEV  CYTIPYERGK  -RMLLDHVSG  YCVPGTLTAL
(PRED) nada_1_g01840   ---------LE  SRGVFIWKEI  LYTIPYEGTQ  -RKLLDNISG  YCIPGTMTAL
```

```
(PRED) kaaf_1_c00820   ---------LE  SKGVFIWKDV  CYTIPYDGGQ  -RMLLDHVSG  FCKPGTLTAL
(PRED) teph_1_m00640   ---------LE  STGTFVWNKV  CFTIPYDGGM  -RMLLDNVSG  FCRPGTMTAL
(PRED) vapo_1_1036_28  ---------LK  STGIFLWNKV  CFTIPYDGGQ  -RMLLDHVSG  YCIPGTLTAL
(PRED) tebl_1_i01760   ---------LE  STGIFIWRKV  CYTVPYNGSQ  -RQLLDKVTG  YCKPGTLTAL
(PRED) tode_1_d04040   ---------LK  SRGVFMWQKV  CYTIPYKGGP  -RRLLDNVSG  YCVPGTMTAL
(PRED) naca_1_e01650   ---------LE  ATGVFIWRDV  CYTIPYDGGQ  -RMLLDHVSG  YCIPGTLTAL
(PRED) tebl_1_g02820   ---------LE  STGIFIWRDV  CYTIPYDGGM  -RRLLDNVSG  YCRPGTMTAL
(PRED) lakl_1_c11616g  ---------LQ  SEGVFLWKDV  CYTIPFKGGQ  -RQLLDNISG  YCAPGTLTAL
(PRED) saar_1_8_h03780 ---------LE  STGVFIWRNV  SFSIQQNNEQ  -RKLLDNVSG  YCVPGTLTAL
(PRED) sace_14_7_g0015 ---------LE  STGVFIWKNV  SFTIPHSSGQ  -RKLLDSVSG  YCVPGTLTAL
(PRED) sace_15_7_g0387 ---------LE  STGVFIWKNV  SFTIPHSSGQ  -RKLLDSVSG  YCVPGTLTAL
(PRED) sace_24_8_3780  ---------LE  STGVFIWKNV  SFTIPHSSGQ  -RKLLDSVSG  YCVPGTLTAL
(PRED) sace_40_8_h0383 ---------LE  STGVFIWKNV  SFTIPHSSGQ  -RKLLDSVSG  YCVPGTLTAL
(PRED) sace_6_169_fm00 ---------LE  STGVFIWKNV  SFTIPHSSGQ  -RKLLDSVSG  YCVPGTLTAL
(PRED) sace_19_7_3840  ---------LE  STGVFIWKNV  SFTIPHSSGQ  -RKLLDSVSG  YCVPGTLTAL
(PRED) sace_32_7_3770  ---------LE  STGVFIWKNV  SFTIPHSSGQ  -RKLLDSVSG  YCVPGTLTAL
(PRED) sace_56_17_q011 ---------LE  STGVFIWKNV  SFTIPHSSGQ  -RKLLDSVSG  YCVPGTLTAL
(PRED) sace_5_78_bz001 ---------LE  STGVFIWKNV  SFTIPHSSGQ  -RKLLDSVSG  YCVPGTLTAL
(PRED) sace_2_8_h03860 ---------LE  STGVFIWKNV  SFTIPHSSGQ  -RKLLDSVSG  YCVPGTLTAL
(PRED) sace_53_29_ac00 ---------LE  STGVFIWKNV  SFTIPHSSGQ  -RKLLDSVSG  YCVPGTLTAL
(PRED) sace_17_7_g0393 ---------LE  STGVFIWKNV  SFTIPHSSGQ  -RKLLDSVSG  YCVPGTLTAL
(PRED) sace_25_7_g0388 ---------LE  STGVFIWKNV  SFTIPHSSGQ  -RKLLDSVSG  YCVPGTLTAL
(PRED) sace_37_7_g0385 ---------LE  STGVFIWKNV  SFTIPHSSGQ  -RKLLDSVSG  YCVPGTLTAL
(PRED) sace_9_7_g00180 ---------LE  STGVFIWKNV  SFTIPHSSGQ  -RKLLDSVSG  YCVPGTLTAL
(PRED) sace_60_6_f0335 ---------LE  STGVFIWKNV  SFTIPHSSGQ  -RKLLDSVSG  YCVPGTLTAL
(PRED) sace_59_336_lx0 ---------LE  STGVFIWKNV  SFTIPHSSGQ  -RKLLDSVSG  YCVPGTLTAL
(PRED) sace_31_7_3780  ---------LE  STGVFIWKNV  SFTIPHSSGQ  -RKLLDSVSG  YCVPGTLTAL
(PRED) sace_34_8_3770  ---------LE  STGVFIWKNV  SFTIPHSSGQ  -RKLLDSVSG  YCVPGTLTAL
(PRED) sace_58_71_bs00 ---------LE  STGVFIWKNV  SFTIPHSSGQ  -RKLLDSVSG  YCVPGTLTAL
(PRED) sace_7_7_g03880 ---------LE  STGVFIWKNV  SFTIPHSSGQ  -RKLLDSVSG  YCVPGTLTAL
(PRED) sace_35_7_3840  ---------LE  STGVFIWKNV  SFTIPHSSGQ  -RKLLDSVSG  YCVPGTLTAL
(PRED) sace_43_7_g0387 ---------LE  STGVFIWKNV  SFTIPHSSGQ  -RKLLDSVSG  YCVPGTLTAL
(PRED) sace_57_8_h0390 ---------LE  STGVFIWKNV  SFTIPHSSGQ  -RKLLDSVSG  YCVPGTLTAL
(PRED) sace_45_7_g0389 ---------LE  STGVFIWKNV  SFTIPHSSGQ  -RKLLDSVSG  YCVPGTLTAL
(PRED) sace_46_8_h0391 ---------LE  STGVFIWKNV  SFTIPHSSGQ  -RKLLDSVSG  YCVPGTLTAL
(PRED) sace_23_7_3860  ---------LE  STGVFIWKNV  SFTIPHSSGQ  -RKLLDSVSG  YCVPGTLTAL
(PRED) sace_21_7_3790  ---------LE  STGVFIWKNV  SFTIPHSSGQ  -RKLLDSVSG  YCVPGTLTAL
(PRED) sace_8_73_bu001 ---------LE  STGVFIWKNV  SFTIPHSSGQ  -RKLLDSVSG  YCVPGTLTAL
(PRED) sapa_1_8_h03820 ---------LE  STGVFIWRNV  SFTISHSSGQ  -RKLLDSVSG  YCVPGTLTAL
(PRED) sapa_21_8_h0387 ---------LE  STGVFIWRNV  SFTISHSSGQ  -RKLLDSVSG  YCVPGTLTAL
(PRED) sapa_20_8_h0386 ---------LE  STGVFIWRNV  SFTISHSSGQ  -RKLLDSVSG  YCVPGTLTAL
(PRED) sapa_22_8_h0390 ---------LE  STGVFIWRNV  SFTISHSSGQ  -RKLLDSVSG  YCVPGTLTAL
(PRED) sapa_25_8_h0387 ---------LE  STGVFIWRNV  SFTISHSSGQ  -RKLLDSVSG  YCVPGTLTAL
(PRED) sapa_6_8_3750   ---------LE  STGVFIWRNV  SFTISHSSGQ  -RKLLDSVSG  YCVPGTLTAL
(PRED) sapa_9_8_3720   ---------LE  STGVFIWRNV  SFTISHSSGQ  -RKLLDSVSG  YCVPGTLTAL
(PRED) sapa_19_8_h0390 ---------LE  STGVFIWRNV  SFTISHSSGQ  -RKLLDSVSG  YCVPGTLTAL
(PRED) sapa_24_8_h0385 ---------LE  STGVFIWRNV  SFTISHSSGQ  -RKLLDSVSG  YCVPGTLTAL
(PRED) sapa_4_8_h03850 ---------LE  STGVFIWRNV  SFTISHSSGQ  -RKLLDSVSG  YCVPGTLTAL
(PRED) sapa_10_8_3760  ---------LE  STGVFIWRNV  SFTISHSSGQ  -RKLLDSVSG  YCVPGTLTAL
(PRED) sapa_13_8_h0382 ---------LE  STGVFIWRNV  SFTISHSSGQ  -RKLLDSVSG  YCVPGTLTAL
(PRED) sapa_8_8_3750   ---------LE  STGVFIWRNV  SFTISHSSGQ  -RKLLDSVSG  YCVPGTLTAL
(PRED) sapa_11_8_h0383 ---------LE  STGVFIWRNV  SFTISHSSGQ  -RKLLDSVSG  YCVPGTLTAL
(PRED) sapa_5_8_3700   ---------LE  STGVFIWRNV  SFTISHSSGQ  -RKLLDSVSG  YCVPGTLTAL
(PRED) sapa_16_8_h0389 ---------LE  STGVFIWRNV  SFTIPHSSGQ  -RKLLDSVSG  YCVPGTLTAL
(PRED) sapa_17_8_3730  ---------LE  STGVFIWRNV  SFTIPHSSGQ  -RKLLDSVSG  YCVPGTLTAL
(PRED) sapa_2_8_h03860 ---------LE  STGVFIWRNV  SFTIPHSSGQ  -RKLLDSVSG  YCVPGTLTAL
(PRED) sapa_7_8_3740   ---------LE  STGVFIWRNV  SFTIPHSSGQ  -RKLLDSVSG  YCVPGTLTAL
(PRED) sapa_23_8_h0385 ---------LE  STGVFIWRNV  SFTIPHSSGQ  -RKLLDSVSG  YCVPGTLTAL
(PRED) sapa_3_8_h03890 ---------LE  STGVFIWRNV  SFTIPHSSGQ  -RKLLDSVSG  YCVPGTLTAL
(PRED) sapa_18_8_3730  ---------LE  STGVFIWRNV  SFTIPHSSGQ  -RKLLDSVSG  YCVPGTLTAL
(PRED) sami_1_14_399   ---------LE  STGVFIWRNV  TLTVQHSNGK  -RKLLDNVSG  YCVPGTLTAL
(PRED) sace_4_8_h03690 ---------LE  STGVFIWKNV  SFTIPHSSGQ  -RKLLDSVSG  YCVPGTLTAL
(PRED) saku_1_14_404   ---------LE  STGVFIWRNV  SFTIRHSNGQ  -RKLLDNVSG  YCVPGTLTAL
(PRED) sace_1_ynr070w  ---------LE  STGVFIWKNV  SFTIPHSSGQ  -RKLLDSVSG  YCVPGTLTAL
(PRED) sace_49_8_h0383 ---------LE  STGVFIWKNV  SFTIPHSSGQ  -RKLLDSVSG  YCVPGTLTAL
(PRED) saeu_1_2_b00130 ---------LA  ATGVFIWKDV  CLTIKYNNGE  -RMLLDNVSG  YCVPGTLTAL
(PRED) sauv_1_7_3      ---------LA  ATGVFIWKDV  CLTIKYNKGE  -RMLLDNVSG  YCVPGTLTAL
(PRED) sami_1_17_26    ---------LE  TTGVFVWKDV  CFTIPYNNGE  -RMLLDNVSG  YCVPGTLTAL
(PRED) zyba_1_02055_AN ---------FQ  SKGIFIWREV  CYTIPYMGSE  -RVLLDHVTG  YVAPGTMTAL
(PRED) zyba_1_07912    ---------FQ  SKGIFIWREV  CYTIPYMGSE  -RVLLDHVTG  YVAPGTMTAL
(PRED) zyba_2_2_b00600 ---------FQ  SKGIFIWREV  CYTIPYMGSE  -RVLLDHVTG  YVAPGTMTAL
(PRED) zyba_3_3_c03460 ---------FQ  SKGIFIWREV  CYTIPYMGSE  -RVLLDHVTG  YVAPGTMTAL
(PRED) zyba_1_04634    ---------FQ  SKGTFMWRDV  CFTIPYTGSE  -RVLLDHVSG  FVAPGTITAL
(PRED) zyba_1_06675    ---------FQ  SKGTFMWRDV  CYTIPYTGSE  -KILLDHVSG  FVAPGTITAL
(PRED) zyba_3_2_b02230 ---------FQ  SKGTFMWRDV  CYTIPYTGSE  -KILLDHVSG  FVAPGTITAL
```

(PRED)	zyba_2_1_a00860	--------FQ	SKGTFMWRDV	CYTIPYTGSE	-KILLDHVSG	FVAPGTITAL
(PRED)	zyro_1_a04114g	--------FA	SKGIFIWKNI	CYVIPYKGGE	-RTLLDNVTG	YVAPGTMTAL
(PRED)	zyro_1_b14762g	--------FG	SKGVFIWQDV	RYVIPYQGTE	-KILLDNVTG	YVAPGTMTAL
(PRED)	zyba_2_14_n0149	--------FE	SKGVFIWKDV	CYVIPYGGDE	-RVLLDHVTG	YVAPGTMTAL
(PRED)	zyba_2_33_ag001	--------FE	SKGVFIWKDV	CYVIPYGDSE	-RTLLNHVTG	YVAPGTMTAL
(PRED)	lath_1_a01914g	--------LK	STGIFIWRNV	CYTIPYRGST	-RRLLDNVSG	YCAPGTLTAL
(PRED)	lawa_1_23_5161	--------MK	STGVFVWKEV	NFTIPYKGST	-RKLLDNVSG	YCAPGSLTAL
(PRED)	klae_1_14_n0012	--------MR	SEGVFLWKDL	TYIIPYKGGE	-RTLLDNISG	YVKPGTMTAL
(PRED)	klla_1_d03432g	--------MR	SEGIFLWKDI	CYTIPYKGGE	-RLLLDNVSG	YVKPGTLTAL
(PRED)	klma_1_1_a01880	--------LR	SEGVFFWKEI	CYTIPYKGGE	-RLLLDHVSG	YVKPGTLTAL
(PRED)	klwi_1_33_ag001	--------MR	SEGIFLWNEL	CYTIPYKGGE	-RLLLDNVSG	YVKPGTMTAL
(PRED)	teph_1_a04220	--------LQ	STEILRWSQV	SYTIPFKTGD	-KQLLNNISG	YCVPGKLTAL
(PRED)	vapo_1_1037_47	--------LG	STGNFLWRDL	VYTIPLEGST	-RVLLDHVSG	YCIPGKLTAL
(PRED)	pata_1_2_b05590	------RLLG	SDEIFMWQHV	DYVIPYKGST	-RKLLDDIQG	YVKPGTLTAL
(PRED)	wian_1_3_c04380	--------LG	SKSIFAWKNV	DYVIPYKGST	-RKLLDNVQG	YVKPGTLTAL
(PRED)	wian_1_3_c04390	--------LG	SKSVFAWQNV	DYVIPYKGGS	-RKLLDNVQG	YVKPGTLTAL
(PRED)	wian_1_7_g01010	--------LG	SKSVFAWQNV	DYVIPYKGGS	-RKLLDNVQG	YVKPGTLTAL
(PRED)	bain_1_1_a00100	--------LG	GEDVFMWQNV	DYVIDIKGEK	-RKLLDNVQG	YVKPGTLTAL
(PRED)	bain_1_17_q0038	--------LG	GEDVFMWQNV	DYVIDIKGEK	-RKLLDSVQG	YVKPGTLTAL
(PRED)	bain_1_8_h00410	--------LS	GDDIFSWSHV	DYTIDIQGEK	-RKLLDDIQG	FVKPGTLTAL
(PRED)	caal_1_19_5759	--------LK	AKGVFVWKDV	DYVIPYEGKK	-RQLLQNVSG	YCVPGTLTAL
(PRED)	caal_11_25_y002	--------LK	AKGVFVWKDV	DYVIPYEGKK	-RQLLQNVSG	YCVPGTLTAL
(PRED)	caal_4_4_d03320	--------LK	AKGVFVWKDV	DYVIPYEGKK	-RQLLQNVSG	YCVPGTLTAL
(PRED)	caal_12_26_z005	--------LK	AKGVFVWKDV	DYVIPYEGKK	-RQLLQNVSG	YCVPGTLTAL
(PRED)	caal_5_30_ad005	--------LK	AKGVFVWKDV	DYVIPYEGKK	-RQLLQNVSG	YCVPGTLTAL
(PRED)	caal_8_3_c03320	--------LK	AKGVFVWKDV	DYVIPYEGKK	-RQLLQNVSG	YCVPGTLTAL
(PRED)	caal_6_4_d03280	--------LK	AKGVFVWKDV	DYVIPYEGKK	-RQLLQNVSG	YCVPGTLTAL
(PRED)	caal_10_3_c0334	--------LK	AKGVFVWKDV	DYVIPYEGKK	-RQLLQNVSG	YCVPGTLTAL
(PRED)	caal_3_29_ac005	--------LK	AKGVFVWKDV	DYVIPYEGKK	-RQLLQNVSG	YCVPGTLTAL
(PRED)	caal_2_04989	--------LK	AKGVFVWKDV	DYVIPYEGKK	-RQLLQNVSG	YCVPGTLTAL
(PRED)	cadu_1_64350	--------LK	AKGVFVWKDV	DYVIPYEGKK	-RQLLQNVSG	YCVPGTLTAL
(PRED)	caor_1_h02090	--------LR	VKDIFLWKNV	DYVIPYDGIE	-RKLLDDVNG	YCIPGTLTAL
(PRED)	capa_1_600750	--------LR	VKDIFLWKNV	NYVIPYDGKE	-RKLLDSVSG	YCIPGTLTAL
(PRED)	loel_1_04930	--------LK	VKDIFIWKDV	DYVIPYDGKQ	-RKLLDNVSG	YCIPGTLTAL
(PRED)	spar_1_5_e03260	--------LK	VEDIFVWKDV	NYVIPYDGAQ	-RKLLDQVSG	YCIPGTLTAL
(PRED)	sppa_1_7_g03160	--------LK	VDDIFVWKDV	DYVIPYDGAE	-RKLLDQVSG	FCVPGTLTAL
(PRED)	catr_1_01205	--------LA	TNDIYVWKKV	DYIIPYQGKQ	-RQLLNDVSG	FCIPGTLTAL
(PRED)	catr_1_05498	--------LA	TNDIYVWKDV	DYIIPYEGKQ	-RQLLNCVSG	FCIPGTMTAL
(PRED)	catr_1_05971	--------LG	VDDVYVWKDV	DYIIPYEGKQ	-RQLLDDVSG	YCIPGTLTAL
(PRED)	deha_1_a03696g	--------LK	SRDVFVWKDV	NYVVKYDGGD	-RKLLDSVSG	YCIPGTLTAL
(PRED)	deha_2_5_e00720	--------LK	SRDVFVWKDV	TYVVKYDGDD	-RTLLDSVSG	YCIPGTLTAL
(PRED)	scst_1_3_c02890	--------LK	SKDIFVWKNV	DYVIPYDGKE	-RKLLDDVSG	YCIPGTLTAL
(PRED)	mebi_1_8_h00300	--------LS	SKDIFVWKDV	NYVIPYEGSE	-RKLLDSVSG	FCLPGALTAL
(PRED)	lakl_1_h21010g	--------LK	SKGVFMWKDV	DYVIPYKGSE	-KKLLDKVSG	FVKPG-LTAL
(PRED)	caar_1_13_m0142	DAT--IKKLG	SDDIFMWQHV	NYTVPYQGEA	-RQLLQDVQG	VVLPGTLTAL
(PRED)	caar_1_14_n0143	DEN--VEQLG	SEDIFMWQDV	DYVVPYAGED	-RKLLDKVQG	VVYPGTLTAL
(PRED)	hapo_1_1_a07220	DTT--GEKLG	SSDIFMWRNV	NYVVPYDGKD	-RKLLEDVQG	YVLPGTLTAL
(PRED)	ogpa_1_1_a01680	DTT--GEKLG	SSDIFMWRNV	NYVVPYEGED	-RKLLEDVQG	YVLPGTLTAL
(PRED)	piku_1_96_cr001	GV---NERLG	SQDILMWKNV	DYVVPYEGEA	-RKLLENVQG	YVLPGTLTAL
(PRED)	pime_1_4_d03240	TIE--GQKLG	SDDIFMWQNV	DYVVPYDGAD	-RKLLDNVQG	YVLPGTLTAL
(PRED)	pime_1_1_a12110	NSE--GKTLG	SSDIFAWKNI	DYVVPYDGQE	-RKLLDNVQG	YVLPGTLTAL
(PRED)	piku_1_227_hs00	NDS--FEGLG	SSDIFCWRDV	NYTVPYANTE	-KKLLDNIQG	YVLPGTMTAL
(PRED)	pime_1_5_e05800	KSA--FNQLG	SSDIFCWKNV	DYVVPYDGTE	-KKLLDSIQG	YVLPGTMTAL
(PRED)	pime_1_1_a07690	SVN--SHKLG	SNNIFTWKNI	DYVVPYEGEE	-RKLLEDVQG	YVRPGSLTAL
(PRED)	debr_2_5_e03380	QSSPFGTHLG	SPDIFLWRHV	DYVVPYEGRD	-RKLLDDVQG	YVLPGTLTAL
(PRED)	kopa_1_2_b10040	EVKVSDKLLG	SDEVFTWKDV	NYVIPYQGSE	-RTLLDHVQG	YVKPGTLTAL
(PRED)	kopa_2_7_g00500	EVKVSDKLLG	SDEVFTWKDV	NYVIPYQGSD	-RTLLDHVQG	YVKPGTLTAL
(PRED)	asru_1_13_m0119	--------LR	GKDIFMWKNV	DYVIPTKDGN	TIKLLDNVQG	YIKPGTLTAL
(PRED)	asru_1_15_o0045	--------LK	GRDTFMWKNI	DYEITVKGDK	KVKLLDNIFG	YVKPGTLTAL
(PRED)	wian_1_1_a02920	--------LG	SGDVFSWKNL	DYTIKTKDGN	LRKLLDNVQG	YIKPGQLVAL
(PRED)	wian_1_1_a02930	--------LS	TADIFSWKNL	DYTIKTKDGN	LRKLLDNVQG	YIKPGQLVAL

		1210	1220	1230	1240	1250
(PRED)	asac_1_6_f03560	MGESGAGKTT	LLNTLAQRN-	VGII-TGDML	VNGLPIDASF	ERRTGYVQQQ
(PRED)	ergo_1_abr125c	MGESGAGKTT	LLNTLAQRN-	VGVI-TGDML	VNGLPIDASF	ERRTGYVQQQ
(PRED)	ercy_1_3604	MGESGAGKTT	LLNTLAQRN-	VGII-TGDML	VNGLPVDISF	ERRTGYVQQQ
(PRED)	cagl_1_i04862g	MGESGAGKTT	LLNTLAQRN-	VGII-TGDML	VNGKPIDISF	ERRTGYVQQQ
(PRED)	kaaf_1_c00830	MGESGAGKTT	LLNTLAQRN-	VGII-TGDML	VNGHHIDASF	ERRTGYVQQQ
(PRED)	kana_1_k01350	MGESGAGKTT	LLNTLAQRN-	VGVI-TGDML	VNGHPIDTSF	ERRTGYVQQQ
(PRED)	saar_1_2_b02590	MGESGAGKTT	LLNTLAQRN-	VGII-TGDML	VNGRPIDASF	ERRTGYVQQQ
(PRED)	sace_1_ydr011w	MGESGAGKTT	LLNTLAQRN-	VGII-TGDML	VNGRPIDASF	ERRTGYVQQQ
(PRED)	sace_16_1_a0238	MGESGAGKTT	LLNTLAQRN-	VGII-TGDML	VNGRPIDASF	ERRTGYVQQQ
(PRED)	sace_45_1_a0242	MGESGAGKTT	LLNTLAQRN-	VGII-TGDML	VNGRPIDASF	ERRTGYVQQQ
(PRED)	sace_48_1_a0238	MGESGAGKTT	LLNTLAQRN-	VGII-TGDML	VNGRPIDASF	ERRTGYVQQQ
(PRED)	sace_60_4_d0244	MGESGAGKTT	LLNTLAQRN-	VGII-TGDML	VNGRPIDASF	ERRTGYVQQQ

(PRED)	sace_52_1_a0240	MGESGAGKTT	LLNTLAQRN-	VGII-TGDML	VNGRPIDASF	ERRTGYVQQQ
(PRED)	sace_46_1_a0240	MGESGAGKTT	LLNTLAQRN-	VGII-TGDML	VNGRPIDASF	ERRTGYVQQQ
(PRED)	sace_25_1_a0240	MGESGAGKTT	LLNTLAQRN-	VGII-TGDML	VNGRPIDASF	ERRTGYVQQQ
(PRED)	sace_24_1_2300	MGESGAGKTT	LLNTLAQRN-	VGII-TGDML	VNGRPIDASF	ERRTGYVQQQ
(PRED)	sace_47_1_a0240	MGESGAGKTT	LLNTLAQRN-	VGII-TGDML	VNGRPIDASF	ERRTGYVQQQ
(PRED)	sace_7_1_a02410	MGESGAGKTT	LLNTLAQRN-	VGII-TGDML	VNGRPIDASF	ERRTGYVQQQ
(PRED)	sace_59_110_df0	MGESGAGKTT	LLNTLAQRN-	VGII-TGDML	VNGRPIDASF	ERRTGYVQQQ
(PRED)	sace_56_1_a0202	MGESGAGKTT	LLNTLAQRN-	VGII-TGDML	VNGRPIDASF	ERRTGYVQQQ
(PRED)	sace_40_1_a0239	MGESGAGKTT	LLNTLAQRN-	VGII-TGDML	VNGRPIDASF	ERRTGYVQQQ
(PRED)	sace_15_1_a0242	MGESGAGKTT	LLNTLAQRN-	VGII-TGDML	VNGRPIDASF	ERRTGYVQQQ
(PRED)	sace_37_1_a0243	MGESGAGKTT	LLNTLAQRN-	VGII-TGDML	VNGRPIDASF	ERRTGYVQQQ
(PRED)	sace_9_1_a02440	MGESGAGKTT	LLNTLAQRN-	VGII-TGDML	VNGRPIDASF	ERRTGYVQQQ
(PRED)	sace_22_1_2300	MGESGAGKTT	LLNTLAQRN-	VGII-TGDML	VNGRPIDASF	ERRTGYVQQQ
(PRED)	sace_29_1_2290	MGESGAGKTT	LLNTLAQRN-	VGII-TGDML	VNGRPIDASF	ERRTGYVQQQ
(PRED)	sace_34_1_2320	MGESGAGKTT	LLNTLAQRN-	VGII-TGDML	VNGRPIDASF	ERRTGYVQQQ
(PRED)	sace_58_25_y007	MGESGAGKTT	LLNTLAQRN-	VGII-TGDML	VNGRPIDASF	ERRTGYVQQQ
(PRED)	sace_23_1_2290	MGESGAGKTT	LLNTLAQRN-	VGII-TGDML	VNGRPIDASF	ERRTGYVQQQ
(PRED)	sace_6_120_dp00	MGESGAGKTT	LLNTLAQRN-	VGII-TGDML	VNGRPIDASF	ERRTGYVQQQ
(PRED)	sace_57_1_a0241	MGESGAGKTT	LLNTLAQRN-	VGII-TGDML	VNGRPIDASF	ERRTGYVQQQ
(PRED)	sace_17_1_a0241	MGESGAGKTT	LLNTLAQRN-	VGII-TGDML	VNGRPIDASF	ERRTGYVQQQ
(PRED)	sace_21_1_2310	MGESGAGKTT	LLNTLAQRN-	VGII-TGDML	VNGRPIDASF	ERRTGYVQQQ
(PRED)	sace_49_1_a0246	MGESGAGKTT	LLNTLAQRN-	VGII-TGDML	VNGRPIDASF	ERRTGYVQQQ
(PRED)	sace_8_2_b02430	MGESGAGKTT	LLNTLAQRN-	VGII-TGDML	VNGRPIDASF	ERRTGYVQQQ
(PRED)	sace_31_1_2300	MGESGAGKTT	LLNTLAQRN-	VGII-TGDML	VNGRPIDASF	ERRTGYVQQQ
(PRED)	sace_50_1_a0241	MGESGAGKTT	LLNTLAQRN-	VGII-TGDML	VNGRPIDASF	ERRTGYVQQQ
(PRED)	sace_4_1_a02360	MGESGAGKTT	LLNTLAQRN-	VGII-TGDML	VNGRPIDASF	ERRTGYVQQQ
(PRED)	sace_2_1_a02390	MGESGAGKTT	LLNTLAQRN-	VGII-TGDML	VNGRPIDASF	ERRTGYVQQQ
(PRED)	sace_5_33_ag005	MGESGAGKTT	LLNTLAQRN-	VGII-TGDML	VNGRPIDASF	ERRTGYVQQQ
(PRED)	sapa_11_1_a0247	MGESGAGKTT	LLNTLAQRN-	VGII-TGDML	VNGRPIDASF	ERRTGYVQQQ
(PRED)	sapa_25_1_a0246	MGESGAGKTT	LLNTLAQRN-	VGII-TGDML	VNGRPIDASF	ERRTGYVQQQ
(PRED)	sapa_4_1_a02470	MGESGAGKTT	LLNTLAQRN-	VGII-TGDML	VNGRPIDASF	ERRTGYVQQQ
(PRED)	sapa_5_1_2350	MGESGAGKTT	LLNTLAQRN-	VGII-TGDML	VNGRPIDASF	ERRTGYVQQQ
(PRED)	sapa_9_1_2360	MGESGAGKTT	LLNTLAQRN-	VGII-TGDML	VNGRPIDASF	ERRTGYVQQQ
(PRED)	sapa_14_1_a0244	MGESGAGKTT	LLNTLAQRN-	VGII-TGDML	VNGRPIDASF	ERRTGYVQQQ
(PRED)	sapa_8_1_2350	MGESGAGKTT	LLNTLAQRN-	VGII-TGDML	VNGRPIDASF	ERRTGYVQQQ
(PRED)	sapa_17_1_2380	MGESGAGKTT	LLNTLAQRN-	VGII-TGDML	VNGRPIDASF	ERRTGYVQQQ
(PRED)	sapa_7_1_2370	MGESGAGKTT	LLNTLAQRN-	VGII-TGDML	VNGRPIDASF	ERRTGYVQQQ
(PRED)	sapa_2_1_a02460	MGESGAGKTT	LLNTLAQRN-	VGII-TGDML	VNGRPIDASF	ERRTGYVQQQ
(PRED)	sapa_23_1_a0248	MGESGAGKTT	LLNTLAQRN-	VGII-TGDML	VNGRPIDASF	ERRTGYVQQQ
(PRED)	sapa_3_1_a02470	MGESGAGKTT	LLNTLAQRN-	VGII-TGDML	VNGRPIDASF	ERRTGYVQQQ
(PRED)	sapa_18_1_2390	MGESGAGKTT	LLNTLAQRN-	VGII-TGDML	VNGRPIDASF	ERRTGYVQQQ
(PRED)	sami_1_4_244	MGESGAGKTT	LLNTLAQRN-	VGII-TGDML	VNGRPIDASF	ERRTGYVQQQ
(PRED)	saku_1_4_262	MGESGAGKTT	LLNTLAQRN-	VGII-TGDML	VNGRPIDASF	ERRTGYVQQQ
(PRED)	saba_1_58_bf002	MGESGAGKTT	LLNTLAQRN-	VGII-TGDML	VNGRPIDASF	ERRTGYVQQQ
(PRED)	saeu_1_4_d02400	MGESGAGKTT	LLNTLAQRN-	VGII-TGDML	VNGRPIDASF	ERRTGYVQQQ
(PRED)	naca_1_e01640	MGESGAGKTT	LLNTLAQRN-	VGII-TGDML	INGRPIDASF	ERRTGYVQQQ
(PRED)	nada_1_g01850	MGESGAGKTT	LLNTLAQRN-	VGII-TGDML	INGRPIDSSF	ERRIGYVQQQ
(PRED)	naca_1_e01630	MGESGAGKTT	LLNTLAKRNE	IGVV-TGDML	VNGRPVDASF	ERRTGYVQQQ
(PRED)	nada_1_g01840	IGESGAGKTT	LLNTLAQRNE	IGVV-TGDIL	VNGRPIDSSF	ERRTGYVQQQ
(PRED)	kaaf_1_c00820	MGESGAGKTT	LLNTLAQRN-	VGII-TGDML	VNGHPIDTSF	ERRTGYVQQQ
(PRED)	teph_1_m00640	MGESGAGKTT	LLNTLAQRN-	VGII-TGDML	VNGKPIDASF	ERRTGYVQQQ
(PRED)	vapo_1_1036_28	MGESGAGKTT	LLNTLAQRN-	VGII-TGDML	VNGRPINASF	ERRTGYVQQQ
(PRED)	tebl_1_i01760	MGESGAGKTT	LLNTLAQRN-	VGII-TGDML	VNGHPVDITF	ERRTGYVQQQ
(PRED)	tode_1_d04040	MGESGAGKTT	LLNTLAQRN-	VGVI-TGDML	VNGRPIDASF	ERRTGYVQQQ
(PRED)	naca_1_e01650	MGESGAGKTT	LLNTLAQRN-	VGII-TGDML	INGHPIDASF	ERRTGYVQQQ
(PRED)	tebl_1_g02820	MGESGAGKTT	LLNTLAQRN-	VGII-TGDML	VNGQPIDASF	ERRAGYVQQQ
(PRED)	lakl_1_c11616g	MGESGAGKTT	LLNTLAQRN-	VGII-TGDML	VNGSPIDASF	ERRTGYVQQQ
(PRED)	saar_1_8_h03780	IGESGAGKTT	LLNTLAQRN-	VGVI-TGDML	VDGLPMDASF	ERRTGYVQQQ
(PRED)	sace_14_7_g0015	IGESGAGKTT	LLNTLAQRN-	VGTI-TGDML	VDGLPMDASF	KRRTGYVQQQ
(PRED)	sace_15_7_g0387	IGESGAGKTT	LLNTLAQRN-	VGTI-TGDML	VDGLPMDASF	KRRTGYVQQQ
(PRED)	sace_24_8_3780	IGESGAGKTT	LLNTLAQRN-	VGTI-TGDML	VDGLPMDASF	KRRTGYVQQQ
(PRED)	sace_40_8_h0383	IGESGAGKTT	LLNTLAQRN-	VGTI-TGDML	VDGLPMDASF	KRRTGYVQQQ
(PRED)	sace_6_169_fm00	IGESGAGKTT	LLNTLAQRN-	VGTI-TGDML	VDGLPMDASF	KRRTGYVQQQ
(PRED)	sace_19_7_3840	IGESGAGKTT	LLNTLAQRN-	VGTI-TGDML	VDGLPMDASF	KRRTGYVQQQ
(PRED)	sace_32_7_3770	IGESGAGKTT	LLNTLAQRN-	VGTI-TGDML	VDGLPMDASF	KRRTGYVQQQ
(PRED)	sace_56_17_q011	IGESGAGKTT	LLNTLAQRN-	VGTI-TGDML	VDGLPMDASF	KRRTGYVQQQ
(PRED)	sace_5_78_bz001	IGESGAGKTT	LLNTLAQRN-	VGTI-TGDML	VDGLPMDASF	KRRTGYVQQQ
(PRED)	sace_2_8_h03860	IGESGAGKTT	LLNTLAQRN-	VGTI-TGDML	VDGLPMDASF	KRRTGYVQQQ
(PRED)	sace_53_29_ac00	IGESGAGKTT	LLNTLAQRN-	VGTI-TGDML	VDGLPMDASF	KRRTGYVQQQ
(PRED)	sace_17_7_g0393	IGESGAGKTT	LLNTLAQRN-	VGTI-TGDML	VDGLPMDASF	KRRTGYVQQQ
(PRED)	sace_25_7_g0388	IGESGAGKTT	LLNTLAQRN-	VGTI-TGDML	VDGLPMDASF	KRRTGYVQQQ
(PRED)	sace_37_7_g0385	IGESGAGKTT	LLNTLAQRN-	VGTI-TGDML	VDGLPMDASF	KRRTGYVQQQ
(PRED)	sace_9_7_g00180	IGESGAGKTT	LLNTLAQRN-	VGTI-TGDML	VDGLPMDASF	KRRTGYVQQQ
(PRED)	sace_60_6_f0335	IGESGAGKTT	LLNTLAQRN-	VGTI-TGDML	VDGLPMDASF	KRRTGYVQQQ
(PRED)	sace_59_336_lx0	IGESGAGKTT	LLNTLAQRN-	VGTI-TGDML	VDGLPMDASF	KRRTGYVQQQ

(PRED)	sace_31_7_3780	IGESGAGKTT	LLNTLAQRN-	VGTI-TGDML	VDGLPMDASF	KRRTGYVQQQ
(PRED)	sace_34_8_3770	IGESGAGKTT	LLNTLAQRN-	VGTI-TGDML	VDGLPMDASF	KRRTGYVQQQ
(PRED)	sace_58_71_bs00	IGESGAGKTT	LLNTLAQRN-	VGTI-TGDML	VDGLPMDASF	KRRTGYVQQQ
(PRED)	sace_7_7_g03880	IGESGAGKTT	LLNTLAQRN-	VGTI-TGDML	VDGLPMDASF	KRRTGYVQQQ
(PRED)	sace_35_7_3840	IGESGAGKTT	LLNTLAQRN-	VGTI-TGDML	VDGLPMDASF	KRRTGYVQQQ
(PRED)	sace_43_7_g0387	IGESGAGKTT	LLNTLAQRN-	VGTI-TGDML	VDGLPMDASF	KRRTGYVQQQ
(PRED)	sace_57_8_h0390	IGESGAGKTT	LLNTLAQRN-	VGTI-TGDML	VDGLPMDASF	KRRTGYVQQQ
(PRED)	sace_45_7_g0389	IGESGAGKTT	LLNTLAQRN-	VGTI-TGDML	VDDLPMDASF	KRRTGYVQQQ
(PRED)	sace_46_8_h0391	IGESGAGKTT	LLNTLAQRN-	VGTI-TGDML	VDGLPMDASF	KRRTGYVQQQ
(PRED)	sace_23_7_3860	IGESGAGKTT	LLNTLAQRN-	VGTI-TGDML	VDGLPMDASF	KRRTGYVQQQ
(PRED)	sace_21_7_3790	IGESGAGKTT	LLNTLAQRN-	VGTI-TGDML	VDGLPMDASS	KRRTGYVQQQ
(PRED)	sace_8_73_bu001	IGESGAGKTT	LLNTLAQRN-	VGTI-TGDML	VDGLPMDASF	KRRTGYVQQQ
(PRED)	sapa_1_8_h03820	IGESGAGKTT	LLNTLAQRN-	VGMI-AGDML	VDGLPMDASF	KRRTGYVQQQ
(PRED)	sapa_21_8_h0387	IGESGAGKTT	LLNTLAQRN-	VGMI-AGDML	VDGLPMDASF	KRRTGYVQQQ
(PRED)	sapa_20_8_h0386	IGESGAGKTT	LLNTLAQRN-	VGMI-AGDML	VDGLPMDASF	KRRTGYVQQQ
(PRED)	sapa_22_8_h0390	IGESGAGKTT	LLNTLAQRN-	VGMI-AGDML	VDGLPMDASF	KRRTGYVQQQ
(PRED)	sapa_25_8_h0387	IGESGAGKTT	LLNTLAQRN-	VGMI-AGDML	VDGLPMDASF	KRRTGYVQQQ
(PRED)	sapa_6_8_3750	IGESGAGKTT	LLNTLAQRN-	VGMI-AGDML	VDGLPMDASF	KRRTGYVQQQ
(PRED)	sapa_9_8_3720	IGESGAGKTT	LLNTLAQRN-	VGMI-AGDML	VDGLPMDASF	KRRTGYVQQQ
(PRED)	sapa_19_8_h0390	IGESGAGKTT	LLNTLAQRN-	VGMI-AGDML	VDGLPMDASF	KRRTGYVQQQ
(PRED)	sapa_24_8_h0385	IGESGAGKTT	LLNTLAQRN-	VGMI-AGDML	VDGLPMDASF	KRRTGYVQQQ
(PRED)	sapa_4_8_h03850	IGESGAGKTT	LLNTLAQRN-	VGMI-AGDML	VDGLPMDASF	KRRTGYVQQQ
(PRED)	sapa_10_8_3760	IGESGAGKTT	LLNTLAQRN-	VGMI-TGDML	VDGLPMDASF	KRRTGYVQQQ
(PRED)	sapa_13_8_h0382	IGESGAGKTT	LLNTLAQRN-	VGMI-TGDML	VDGLPMDASF	KRRTGYVQQQ
(PRED)	sapa_8_8_3750	IGESGAGKTT	LLNTLAQRN-	VGMI-TGDML	VDGLPMDASF	KRRTGYVQQQ
(PRED)	sapa_11_8_h0383	IGESGAGKTT	LLNTLAQRN-	VGMI-AGDML	VDGLPMDASF	KRRTGYVQQQ
(PRED)	sapa_5_8_3700	IGESGAGKTT	LLNTLAQRN-	VGMI-AGDML	VDGLPMDASF	KRRTGYVQQQ
(PRED)	sapa_16_8_h0389	IGESGAGKTT	LLNTLAQRN-	VGMI-TGDML	VDGLPMDASF	KRRTGYVQQQ
(PRED)	sapa_17_8_3730	IGESGAGKTT	LLNTLAQRN-	VGMI-TGDML	VDGLPMDASF	KRRTGYVQQQ
(PRED)	sapa_2_8_h03860	IGESGAGKTT	LLNTLAQRN-	VGMI-TGDML	VDGLPMDASF	KRRTGYVQQQ
(PRED)	sapa_7_8_3740	IGESGAGKTT	LLNTLAQRN-	VGMI-TGDML	VDGLPMDASF	KRRTGYVQQQ
(PRED)	sapa_23_8_h0385	IGESGAGKTT	LLNTLAQRN-	VGMI-TGDML	VDGLPMDASF	KRRTGYVQQQ
(PRED)	sapa_3_8_h03890	IGESGAGKTT	LLNTLAQRN-	VGMI-TGDML	VDGLPMDASF	KRRTGYVQQQ
(PRED)	sapa_18_8_3730	IGESGAGKTT	LLNTLAQRN-	VGMI-TGDML	VNGLPMDASF	KRRTGYVQQQ
(PRED)	sami_1_14_399	IGESGAGKTT	LLNTLAQRN-	VGTI-TGDML	VNGLPIDASF	ARRTGYVQQQ
(PRED)	sace_4_8_h03690	IGESGAGKTT	LLNTLAQRN-	VGTI-TGDML	VDGLPMDASF	KRRTGYVQQQ
(PRED)	saku_1_14_404	IGESGAGKTT	LLNTLAQRN-	VGTV-TGDIL	VDGLPMDASF	ERRTGYVQQQ
(PRED)	sace_1_ynr070w	IGESGAGKTT	LLNTLAQRN-	VGTI-TGDML	VDGLPMDASF	KRRTGYVQQQ
(PRED)	sace_49_8_h0383	IGESGAGKTT	LLNTLAQRN-	VGTI-TGDML	VDGLPMDASF	KRRTGYVQQQ
(PRED)	saeu_1_2_b00130	IGESGAGKTT	LLNTLAQRN-	VGII-TGDIL	VNGLPINASF	ERRTGYVQQQ
(PRED)	sauv_1_7_3	IGESGAGKTT	LLNTLAQRN-	VGII-TGDIL	VNGLPINASF	ERRTGYVQQQ
(PRED)	sami_1_17_26	MGESGAGKTT	LLNTLAQRN-	VGTI-TGDML	VNGFPIDASF	ERRTGYVQQQ
(PRED)	zyba_1_02055_AN	MGESGAGKTT	LLNTLAQRND	IGVV-TGDML	VNGHPIDTSF	ERRTGYVQQQ
(PRED)	zyba_1_07912	MGESGAGKTT	LLNTLAQRND	IGVV-TGDML	VNGHPIDTSF	ERRTGYVQQQ
(PRED)	zyba_2_2_b00600	MGESGAGKTT	LLNTLAQRND	IGVV-TGDML	VNGHPIDTSF	ERRTGYVQQQ
(PRED)	zyba_3_3_c03460	MGESGAGKTT	LLNTLAQRND	IGVV-TGDML	VNGHPIDTSF	ERRTGYVQQQ
(PRED)	zyba_1_04634	MGESGAGKTT	LLNTLARRNN	FGVV-TGDML	VNGCPIDMSF	ERRTGYVQQQ
(PRED)	zyba_1_06675	MGESGAGKTT	LLNTLARRNN	FGVV-TGDML	VNGCPIDMSF	ERRTGYVQQQ
(PRED)	zyba_3_2_b02230	MGESGAGKTT	LLNTLARRNN	FGVV-TGDML	VNGCPIDMSF	ERRTGYVQQQ
(PRED)	zyba_2_1_a00860	MGESGAGKTT	LLNTLARRNN	FGVV-TGDML	VNGCPIDMSF	ERRTGYVQQQ
(PRED)	zyro_1_a04114g	MGESGAGKTT	LLNTLAKRVN	VGVV-TGDML	INGKPVDSSF	ERRTGYVQQQ
(PRED)	zyro_1_b14762g	MGESGAGKTT	LLNTLAKRAN	VGIV-TGDMS	INGKPVDSSF	ERRTGYVQQQ
(PRED)	zyba_2_14_n0149	MGESGAGKTT	LLNTLAQRNE	VGVV-TGDML	VNGFPIDTSF	ERRTGYVQQQ
(PRED)	zyba_2_33_ag001	MGESGAGKTT	LLNTLAQRNE	VGVV-TGDML	VNGFPIDASF	ERRTGYVQQQ
(PRED)	lath_1_a01914g	MGESGAGKTT	LLNTLAQRN-	VGII-TGDML	VNGNHIDASF	ERRTGYVQQQ
(PRED)	lawa_1_23_5161	MGESGAGKTT	LLNTLAQRN-	VGII-TGDML	VNGKPIDASF	ERRTGYVQQQ
(PRED)	klae_1_14_n0012	MGESGAGKTT	LLNTLAQRID	VGVI-HGDVL	VNGRPIDASF	ERRTGYVQQQ
(PRED)	klla_1_d03432g	MGESGAGKTT	LLNTLAQRTD	IGVV-TGDML	VNGKPIDASF	ERRTGYVQQQ
(PRED)	klma_1_1_a01880	MGESGAGKTT	LLNTLAQRTD	IGVV-TGDML	VNGKPIDASF	ERRTGYVQQQ
(PRED)	klwi_1_33_ag001	MGESGAGKTT	LLNTLAQRTD	IGVV-TGDML	VNGRPIDTTF	ERRTGYVQQQ
(PRED)	teph_1_a04220	MGESGAGKTT	LLNVLSKRNE	FGII-TGDIS	VGDTPIDSSF	ERRIGYVQQQ
(PRED)	vapo_1_1037_47	MGESGAGKTT	LLNTLAQRNE	IGVV-TGDIK	VDGAKIDISF	GRRTGYVQQQ
(PRED)	pata_1_2_b05590	MGESGAGKTT	LLNTLSQRNE	VGVI-TGDIL	VNGRSVDNSF	QRRTGYVQQQ
(PRED)	wian_1_3_c04380	MGESGAGKTT	LLNTLAQRND	IGTV-TGSIL	VDGKPLDTSF	KRSTGYVQQQ
(PRED)	wian_1_3_c04390	MGESGAGKTT	LLNTLAQRID	MGTI-TGSML	VNGKPLDTSF	KRSTGYVQQQ
(PRED)	wian_1_7_g01010	MGESGAGKTT	LLNTLAQRID	MGTV-TGSML	VNGKPLDTSF	KRSTGYVQQQ
(PRED)	bain_1_1_a00100	MGESGAGKTT	LLNVLSQRVD	MGVI-TGDML	VNGKPIDNSF	QRRTGYVQQQ
(PRED)	bain_1_17_q0038	MGESGAGKTT	LLNVLSQRVD	MGVI-TGDML	VNGKPIDNSF	QRRTGYVQQQ
(PRED)	bain_1_8_h00410	MGESGAGKTT	LLNVLSQRTD	IGVV-TGNML	VNGSQLTHSF	HRSTGYVQQQ
(PRED)	caal_1_19_5759	MGESGAGKTT	LLNVLAQRVD	FGVI-TGDML	VNGRPLDTSF	SRRTGYVQQQ
(PRED)	caal_11_25_y002	MGESGAGKTT	LLNVLAQRVD	FGVI-TGDML	VNGRPLDTSF	SRRTGYVQQQ
(PRED)	caal_4_4_d03320	MGESGAGKTT	LLNVLAQRVD	FGVI-TGDML	VNGRPLDTSF	SRRTGYVQQQ
(PRED)	caal_12_26_z005	MGESGAGKTT	LLNVLAQRVD	FGVI-TGDML	VNGRPLDTSF	SRRTGYVQQQ
(PRED)	caal_5_30_ad005	MGESGAGKTT	LLNVLAQRVD	FGVI-TGDML	VNGRPLDTSF	SRRTGYVQQQ
(PRED)	caal_8_3_c03320	MGESGAGKTT	LLNVLAQRVD	FGVI-TGDML	VNGRPLDTSF	SRRTGYVQQQ

(PRED)	caal_6_4_d03280	MGESGAGKTT	LLNVLAQRVD	FGVI-TGDML	VNGRPLDTSF	SRRTGYVQQQ
(PRED)	caal_10_3_c0334	MGESGAGKTT	LLNVLAQRVD	FGVI-TGDML	VNGRPLDTSF	SRRTGYVQQQ
(PRED)	caal_3_29_ac005	MGESGAGKTT	LLNVLAQRVD	FGVI-TGDML	VNGRPLDTSF	SRRTGYVQQQ
(PRED)	caal_2_04989	MGESGAGKTT	LLNVLAQRVD	FGVI-TGDML	VNGRPLDTSF	SRRTGYVQQQ
(PRED)	cadu_1_64350	MGESGAGKTT	LLNVLAQRID	FGVI-TGDML	VNGRPLDTSF	SRRTGYVQQQ
(PRED)	caor_1_h02090	MGESGAGKTT	LLNTLAQRID	FGTI-TGDML	VNGKPLDTSF	SRRTGYVQQQ
(PRED)	capa_1_600750	MGESGAGKTT	LLNTLAQRID	FGTI-TGDML	VNGKPLDTSF	SRRTGYVQQQ
(PRED)	loel_1_04930	MGESGAGKTT	LLNTLAQRID	FGVV-TGDML	VNGKPLDSSF	SRRTGYVQQQ
(PRED)	spar_1_5_e03260	MGESGAGKTT	LLNTLAQRID	FGVV-TGDML	VNGKPLDSSF	SRRTGYVQQQ
(PRED)	sppa_1_7_g03160	MGESGAGKTT	LLNTLAQRID	FGVV-TGDML	VNGKPLDSSF	SRRTGYVQQQ
(PRED)	catr_1_01205	MGESGAGKTT	LLNVLAQRID	FGTI-TGDML	VNGKPLDSSF	SRRTGYVQQQ
(PRED)	catr_1_05498	MGESGAGKTT	LLNVLAQRID	FGTI-TGDML	VNGRPLDSSF	SRRTGYVQQQ
(PRED)	catr_1_05971	MGESGAGKTT	LLNVLAQRVD	FGTI-TGDML	VNGRPLDSSF	SRRTGYVQQQ
(PRED)	deha_1_a03696g	MGESGAGKTT	LLNTLAQRID	VGVV-TGDML	VNGKPLDLSF	RRRTGYVQQQ
(PRED)	deha_2_5_e00720	MGESGAGKTT	LLNTLAQRID	VGVV-TGDML	VNGKPLDLSF	RRRTGYVQQQ
(PRED)	scst_1_3_c02890	MGESGAGKTT	LLNTLAQRID	MGVV-TGDML	VNGKPLDLSF	SRRTGYVQQQ
(PRED)	mebi_1_8_h00300	MGESGAGKTT	LLNTLAQRID	MGVI-TGDML	VNGKHLDSTF	VRRTGYVQQQ
(PRED)	lakl_1_h21010g	MGASGAGKTT	LLNVLAQRMD	FGIVSSGEIL	VDAKPVDRKF	KRSVGYVQQQ
(PRED)	caar_1_13_m0142	MGESGAGKTT	LLNVLSRRTE	VGVV-TGDML	INGKAIDNTF	ESKTGYVQQQ
(PRED)	caar_1_14_n0143	MGESGAGKTT	LLNVLSRRVE	TGVV-TGSMF	INGKDIDSSF	ERRTGYVQQQ
(PRED)	hapo_1_1_a07220	MGESGAGKTT	LLNVLSRRTD	VGVV-NGDML	INGKPIDNSF	ERRTGYVQQQ
(PRED)	ogpa_1_1_a01680	MGESGAGKTT	LLNVLSRRTD	VGVV-TGDML	INGKPIDNSF	ERRTGYVQQQ
(PRED)	piku_1_96_cr001	MGESGAGKTT	LLNVLSRRVD	VGVV-SGDML	INGKPIDSSF	ERRTGYVQQQ
(PRED)	pime_1_4_d03240	MGESGAGKTT	LLNVLSRRVD	VGVV-SGDML	INGKGIDSSF	ERRTGYVQQQ
(PRED)	pime_1_1_a12110	MGESGAGKTT	LLNVLSRRVD	VGVV-TGDML	INGQPLDSSF	ERRTGYVQQQ
(PRED)	piku_1_227_hs00	IGESGAGKTT	LLNVLSRRTE	VGVI-TGDMF	VNGAPVDSSF	ERRTGYVQQQ
(PRED)	pime_1_5_e05800	IGESGAGKTT	LLNVLSRRTE	VGVV-TGDML	VNGKPIDLSF	ERRTGYVQQQ
(PRED)	pime_1_1_a07690	MGESGAGKTT	LLNVLSRRTD	VGIV-TGDML	INGKPMGQSF	ERRTGYVQQQ
(PRED)	debr_2_5_e03380	MGESGAGKTT	LLNVLSQRTD	VGVV-TGDFL	VNGNRLDTSF	QKRTGYVQQQ
(PRED)	kopa_1_2_b10040	MGESGAGKTT	LLNVLSQRID	VGVV-TGDML	VNGNPVSASF	KKRTGYVQQQ
(PRED)	kopa_2_7_g00500	MGESGAGKTT	LLNVLSQRID	VGVV-TGDML	VNGNPVSASF	KKRTGYVQQQ
(PRED)	asru_1_13_m0119	MGESGAGKTT	LLNVLSQRVS	FGVI-TGDML	ANGRPLDSSF	QRSTGYVQQQ
(PRED)	asru_1_15_o0045	MGESGAGKTT	LLNVLSERVN	VGVV-SGDIL	VNGKDLDSSF	KRSTGYVQQQ
(PRED)	wian_1_1_a02920	MGESGAGKTT	LLNVLSQRID	MGVI-TGDML	VNGAPIDSTF	KKRTGYVQQQ
(PRED)	wian_1_1_a02930	MGESGAGKTT	LLNVLSQRID	MGVI-TGDML	VNGAPIDSTF	KKRTGYVQQQ

		1260	1270	1280	1290	1300
(PRED)	asac_1_6_f03560	DVHVKEMTVR	ESLQFSARLR	RPESVPDAEK	LNYVEKIIDI	LGMSDFADAL
(PRED)	ergo_1_abr125c	DVHVKEMTVR	ESLQFSARLR	RPESVSEAEK	MNYVEKIIEI	LGMSDFADAL
(PRED)	ercy_1_3604	DVHIKEMTVR	ESLQFSARLR	RPESVPDAEK	LDYVEKIIEV	LGMTDYADAV
(PRED)	cagl_1_i04862g	DIHISELTVR	ESLQFSARMR	RAQNVPEEEK	MEHVERIIKV	LDMEEYADAL
(PRED)	kaaf_1_c00830	DIHIAELTVR	ESLQFSARLR	RPQNISDKEK	MDYVEKIIDV	LDMEDYAEAL
(PRED)	kana_1_k01350	DIHIAEMTVR	ESLRFSARMR	RPQHIPDAEK	LDYVEKIIQV	LNMEEYAEAL
(PRED)	saar_1_2_b02590	DIHIAEMTVR	ESLQFSARMR	RPQHLPDSEK	MDYVEKIIRV	LGMEEYAEAL
(PRED)	sace_1_ydr011w	DIHIAELTVR	ESLQFSARMR	RPQHLPDSEK	MDYVEKIIRV	LGMEEYAEAL
(PRED)	sace_16_1_a0238	DIHIAELTVR	ESLQFSARMR	RPQHLPDSEK	MDYVEKIIRV	LGMEEYAEAL
(PRED)	sace_45_1_a0242	DIHIAELTVR	ESLQFSARMR	RPQHLPDSEK	MDYVEKIIRV	LGMEEYAEAL
(PRED)	sace_48_1_a0238	DIHIAELTVR	ESLQFSARMR	RPQHLPDSEK	MDYVEKIIRV	LGMEEYAEAL
(PRED)	sace_60_4_d0244	DIHIAELTVR	ESLQFSARMR	RPQHLPDSEK	MDYVEKIIRV	LGMEEYAEAL
(PRED)	sace_52_1_a0240	DIHIAELTVR	ESLQFSARMR	RPQHLPDSEK	MDYVEKIIRV	LGMEEYAEAL
(PRED)	sace_46_1_a0240	DIHIAELTVR	ESLQFSARMR	RPQHLPDSEK	MDYVEKIIRV	LGMEEYAEAL
(PRED)	sace_25_1_a0240	DIHIAELTVR	ESLQFSARMR	RPQHLPDSEK	MDYVEKIIRV	LGMEEYAEAL
(PRED)	sace_24_1_2300	DIHIAELTVR	ESLQFSARMR	RPQHLPDSEK	MDYVEKIIRV	LGMEEYAEAL
(PRED)	sace_47_1_a0240	DIHIAELTVR	ESLQFSARMR	RPQHLPDSEK	MDYVEKIIRV	LGMEEYAEAL
(PRED)	sace_7_1_a02410	DIHIAELTVR	ESLQFSARMR	RPQHLPDSEK	MDYVEKIIRV	LGMEEYAEAL
(PRED)	sace_59_110_df0	DIHIAELTVR	ESLQFSARMR	RPQHLPDSEK	MDYVEKIIRV	LGMEEYAEAL
(PRED)	sace_56_1_a0202	DIHIAELTVR	ESLQFSARMR	RPQHLPDSEK	MDYVEKIIRV	LGMEEYAEAL
(PRED)	sace_40_1_a0239	DIHIAELTVR	ESLQFSARMR	RPQHLPDSEK	MDYVEKIIRV	LGMEEYAEAL
(PRED)	sace_15_1_a0242	DIHIAELTVR	ESLQFSARMR	RPQHLPDSEK	MDYVEKIIRV	LGMEEYAEAL
(PRED)	sace_37_1_a0243	DIHIAELTVR	ESLQFSARMR	RPQHLPDSEK	MDYVEKIIRV	LGMEEYAEAL
(PRED)	sace_9_1_a02440	DIHIAELTVR	ESLQFSARMR	RPQHLPDSEK	MDYVEKIIRV	LGMEEYAEAL
(PRED)	sace_22_1_2300	DIHIAELTVR	ESLQFSARMR	RPQHLPDSEK	MDYVEKIIRV	LGMEEYAEAL
(PRED)	sace_29_1_2290	DIHIAELTVR	ESLQFSARMR	RPQHLPDSEK	MDYVEKIIRV	LGMEEYAEAL
(PRED)	sace_34_1_2320	DIHIAELTVR	ESLQFSARMR	RPQHLPDSEK	MDYVEKIIRV	LGMEEYAEAL
(PRED)	sace_58_25_y007	DIHIAELTVR	ESLQFSARMR	RPQHLPDSEK	MDYVEKIIRV	LGMEEYAEAL
(PRED)	sace_23_1_2290	DIHIAELTVR	ESLQFSARMR	RPQHLPDSEK	MDYVEKIIRV	LGMEDYAEAL
(PRED)	sace_6_120_dp00	DIHIAELTVR	ESLQFSARMR	RPQHLPDSEK	MDYVEKIIRV	LGMEEYAEAL
(PRED)	sace_57_1_a0241	DIHIAELTVR	ESLQFSARMR	RPQHLPDSEK	MDYVEKIIRV	LGMEEYAEAL
(PRED)	sace_17_1_a0241	DIHIAELTVR	ESLQFSARMR	RPQHLPDTEK	MDYVEKIIRV	LGMEEYAEAL
(PRED)	sace_21_1_2310	DIHIAELTVR	ESLQFSARMR	RPQHLPDSEK	MDYVEKIIRV	LGMEEYAEAL
(PRED)	sace_49_1_a0246	DIHIAELTVR	ESLQFSARMR	RPQHLPDSEK	MDYVEKIIRV	LGMEEYAEAL
(PRED)	sace_8_2_b02430	DIHIAELTVR	ESLQFSARMR	RPQHLPDSEK	MDYVEKIIRV	LGMEEYAEAL
(PRED)	sace_31_1_2300	DIHIAELTVR	ESLQFSARMR	RPQHLPDSEK	MDYVEKIIRV	LGMEEYAEAL
(PRED)	sace_50_1_a0241	DIHIAELTVR	ESLQFSARMR	RPQHLPDSEK	MDYVEKIIRV	LGMEEYAEAL
(PRED)	sace_4_1_a02360	DIHIAELTVR	ESLQFSARMR	RPQHLPDSEK	MDYVEKIIRV	LGMEEYAEAL

(PRED)	sace_2_1_a02390	DIHIAELTVR	ESLQFSARMR	RPQHLPDSEK	MDYVEKIIRV	LGMEEYAEAL
(PRED)	sace_5_33_ag005	DIHIAELTVR	ESLQFSARMR	RPQHLPDSEK	MDYXEKIIRV	LGMEEYAEAL
(PRED)	sapa_11_1_a0247	DIHIAELTVR	ESLQFSARMR	RPQRLPDSEK	MDYVEKIIRV	LGMEEYAEAL
(PRED)	sapa_25_1_a0246	DIHIAELTVR	ESLQFSARMR	RPQRLPDSEK	MDYVEKIIRV	LGMEEYAEAL
(PRED)	sapa_4_1_a02470	DIHIAELTVR	ESLQFSARMR	RPQRLPDSEK	MDYVEKIIRV	LGMEEYAEAL
(PRED)	sapa_5_1_2350	DIHIAELTVR	ESLQFSARMR	RPQRLPDSEK	MDYVEKIIRV	LGMEEYAEAL
(PRED)	sapa_9_1_2360	DIHIAELTVR	ESLQFSARMR	RPQRLPDSEK	MDYVEKIIRV	LGMEEYAEAL
(PRED)	sapa_14_1_a0244	DIHIAELTVR	ESLQFSARMR	RPQRLPDSEK	MDYVEKIIRV	LGMEEYAEAL
(PRED)	sapa_8_1_2350	DIHIAELTVR	ESLQFSARMR	RPQRLPDSEK	MDYVEKIIRV	LGMEEYAEAL
(PRED)	sapa_17_1_2380	DIHIAELTVR	ESLQFSARMR	RPQRLPDSEK	MDYVEKIIRV	LGMEEYAEAL
(PRED)	sapa_7_1_2370	DIHIAELTVR	ESLQFSARMR	RPQRLPDSEK	MDYVEKIIRV	LGMEEYAEAL
(PRED)	sapa_2_1_a02460	DIHIAELTVR	ESLQFSARMR	RPQRLPDSEK	MDYVEKIIRV	LGMEEYAEAL
(PRED)	sapa_23_1_a0248	DIHIAELTVR	ESLQFSARMR	RPQRLPDSEK	MDYVEKIIRV	LGMEEYAEAL
(PRED)	sapa_3_1_a02470	DIHIAELTVR	ESLQFSARMR	RPQRLPDSEK	MDYVEKIIRV	LGMEEYAEAL
(PRED)	sapa_18_1_2390	DIHIAELTVR	ESLQFSARMR	RPQRLPDSEK	MDYVEKIIRV	LGMEEYAEAL
(PRED)	sami_1_4_244	DIHIAELTVR	ESLQFSARMR	RPQSLPDCEK	MDYVEKIIRV	LGMEEYAEAL
(PRED)	saku_1_4_262	DIHIAELTVR	ESLQFSARMR	RPQRLPDSEK	MEYVEKIIRV	LGMEEYAEAL
(PRED)	saba_1_58_bf002	DIHIAELTVR	ESLQFSARMR	RPQHLPDSEK	MDYVEKIIRV	LSMEEYAEAL
(PRED)	saeu_1_4_d02400	DIHIAELTVR	ESLQFSARMR	RPQHLPDSEK	MDYVEKIIRV	LSMEEYAEAL
(PRED)	naca_1_e01640	DVHIAELTVR	ESLQFSARMR	RAQAIPDEEK	MAYVEKIIHV	LDMQEYAEAL
(PRED)	nada_1_g01850	DIHIAELSVR	ESLQFSARMR	RDQRIPDSEK	LAYVEKIIQV	LGMEPYAEAL
(PRED)	naca_1_e01630	DIHIAELTVR	ESLQFSARMR	RSEHVSDEEK	LAYVEKIIRV	LEMEEFADAL
(PRED)	nada_1_g01840	DIHIAELSVR	ESLQFSARMR	RPESVPDEEK	MAYVEKIIQV	LDMEEYAEAL
(PRED)	kaaf_1_c00820	DIHISELTVR	ESLQFSARMR	RPQSVTDKEK	MTYVEKIIHV	LDMEDYAEAL
(PRED)	teph_1_m00640	DVHIAELTVR	ESLQFSARMR	QPQHLPDSEK	MDYVERIIRV	LDMDEYAEAL
(PRED)	vapo_1_1036_28	DVHIAELTVR	ESLQFSARMR	RAQSIPDSEK	MAYVEKIIRI	LDMEEYAEAL
(PRED)	tebl_1_i01760	DMHIAQLSVR	ESLQFSARVR	RPASVSDEEK	MHYVERVIEV	LDMEQYAEAL
(PRED)	tode_1_d04040	DLHIAEMTVR	ESLIFSARMR	RKQSVPDAEK	IEFAEKIIDI	LDMGEYAEAL
(PRED)	naca_1_e01650	DIHIAELTVR	ESLQFSARMR	RAQAIPDEEK	MAYVEKIIQV	LDMEYYAEAL
(PRED)	tebl_1_g02820	DLHVAEMTVR	ESLQFSARMR	RNQDVPDEEK	MAYVEKIIEV	LGMEEYSEAL
(PRED)	lakl_1_c11616g	DIHIKEMTVR	ESLQFSARMR	RPESIPDSEK	MSYVEKLIEI	LGMQEYADAL
(PRED)	saar_1_8_h03780	DLHTSELTVK	ESLQFSARMR	RSQSIPDAEK	MDYVEKIIDI	LEMQGFSEAL
(PRED)	sace_14_7_g0015	DLHVAELTVK	ESLQFSARMR	RPQSIPDAEK	MEYVEKIISI	LEMQEFSEAL
(PRED)	sace_15_7_g0387	DLHVAELTVK	ESLQFSARMR	RPQSIPDAEK	MEYVEKIISI	LEMQEFSEAL
(PRED)	sace_24_8_3780	DLHVAELTVK	ESLQFSARMR	RPQSIPDAEK	MEYVEKIISI	LEMQEFSEAL
(PRED)	sace_40_8_h0383	DLHVAELTVK	ESLQFSARMR	RPQSIPDAEK	MEYVEKIISI	LEMQEFSEAL
(PRED)	sace_6_169_fm00	DLHVAELTVK	ESLQFSARMR	RPQSIPDAEK	MEYVEKIISI	LEMQEFSEAL
(PRED)	sace_19_7_3840	DLHVAELTVK	ESLQFSARMR	RPQSIPDAEK	MEYVEKIISI	LEMQEFSEAL
(PRED)	sace_32_7_3770	DLHVAELTVK	ESLQFSARMR	RPQSIPDAEK	MEYVEKIISI	LEMQEFSEAL
(PRED)	sace_56_17_q011	DLHVAELTVK	ESLQFSARMR	RPQSIPDAEK	MEYVEKIISI	LEMQEFSEAL
(PRED)	sace_5_78_bz001	DLHVAELTVK	ESLQFSARMR	RPQSIPDAEK	MEYVEKIISI	LEMQEFSEAL
(PRED)	sace_2_8_h03860	DLHVAELTVK	ESLQFSARMR	RPQSIPDAEK	MEYVEKIISI	LEMQEFSEAL
(PRED)	sace_53_29_ac00	DLHVAELTVK	ESLQFSARMR	RPQSIPDAEK	MEYVEKIISI	LEMQEFSEAL
(PRED)	sace_17_7_g0393	DLHVAELTVK	ESLQFSARMR	RPQSIPDAEK	MEYVEKIISI	LEMQEFSEAL
(PRED)	sace_25_7_g0388	DLHVAELTVK	ESLQFSARMR	RPQSIPDAEK	MEYVEKIISI	LEMQEFSEAL
(PRED)	sace_37_7_g0385	DIHVAELTVK	ESLQFSARMR	RPQSIPDAEK	MEYVEKIISI	LEMQEFSEAL
(PRED)	sace_9_7_g00180	DIHVAELTVK	ESLQFSARMR	RPQSIPDAEK	MEYVEKIISI	LEMQEFSEAL
(PRED)	sace_60_6_f0335	DIHVAELTVK	ESLQFSARMR	RPQSIPDAEK	MEYVEKIISI	LEMQEFSEAL
(PRED)	sace_59_336_lx0	DLHVAELTVK	ESLQFSARMR	RPQSIPDAEK	MEYVEKIISI	LEMQEFSEAL
(PRED)	sace_31_7_3780	DLHVAELTVK	ESLQFSARMR	RPQSIPDAEK	MEYVEKIISI	LEMQEFSEAL
(PRED)	sace_34_8_3770	DLHVAELTVK	ESLQFSARMR	RPQSIPDAEK	MEYVEKIISI	LEMQEFSEAL
(PRED)	sace_58_71_bs00	DLHVAELTVK	ESLQFSARMR	RPQSIPDAEK	MEYVEKIISI	LEMQEFSEAL
(PRED)	sace_7_7_g03880	DLHVAELTVK	ESLQFSARMR	RPQSIPDAEK	MEYVEKIISI	LEMQEFSEAL
(PRED)	sace_35_7_3840	DLHVAELTVK	ESLQFSARMR	RPQSIPDAEK	MEYVEKIISI	LEMQEFSEAL
(PRED)	sace_43_7_g0387	DLHVAELTVK	ESLQFSARMR	RPQSIPDAEK	MEYVEKIISI	LEMQEFSEAL
(PRED)	sace_57_8_h0390	DLHVAELTVK	ESLQFSARMR	RPQSIPDAEK	MEYVEKIISI	LEMQEFSEAL
(PRED)	sace_45_7_g0389	DLHVAELTVK	ESLQFSARMR	RPQSIPDAEK	MEYVEKIISI	LEMQEFSEAL
(PRED)	sace_46_8_h0391	DLHVAELTVK	ESLQFSARMR	RPQSIPDAEK	MEYVEKIISI	LEMQEFSEAL
(PRED)	sace_23_7_3860	DLHVAELTVK	ESLQFSARMR	RPQSIPDAEK	MEYVEKIISI	LEMQEFSEAL
(PRED)	sace_21_7_3790	DLHVAELTVK	ESLQFSARMR	RPQSIPDAEK	MEYVEKIISI	LEMQEFSEAL
(PRED)	sace_8_73_bu001	DLHVAELTVK	ESLQFSARMR	RPQSIPDAEK	MEYVEKIISI	LEMQEFSEAL
(PRED)	sapa_1_8_h03820	DLHIAELTVK	ESLEFSARMR	RPQSIPDAEK	MEYVEKIISI	LEMQEFSEAL
(PRED)	sapa_21_8_h0387	DLHIAELTVK	ESLEFSARMR	RPQSIPDAEK	MEYVEKIISI	LEMQEFSEAL
(PRED)	sapa_20_8_h0386	DLHIAELTVK	ESLEFSARMR	RPQSIPDAEK	MEYVEKIISI	LEMQEFSEAL
(PRED)	sapa_22_8_h0390	DLHIAELTVK	ESLEFSARMR	RPQSIPDAEK	MEYVEKIISI	LEMQEFSEAL
(PRED)	sapa_25_8_h0387	DLHIAELTVK	ESLEFSARMR	RPQSIPDAEK	MEYVEKIISI	LEMQEFSEAL
(PRED)	sapa_6_8_3750	DLHIAELTVK	ESLEFSARMR	RPQSIPDAEK	MEYVEKIISI	LEMQEFSEAL
(PRED)	sapa_9_8_3720	DLHIAELTVK	ESLEFSARMR	RPQSIPDAEK	MEYVEKIISI	LEMQEFSEAL
(PRED)	sapa_19_8_h0390	DLHIAELTVK	ESLEFSARMR	RPQSIPDAEK	MEYVEKIISI	LEMQEFSEAL
(PRED)	sapa_24_8_h0385	DLHIAELTVK	ESLEFSARMR	RPQSIPDAEK	MEYVEKIISI	LEMQEFSEAL
(PRED)	sapa_4_8_h03850	DLHIAELTVK	ESLEFSARMR	RPQSIPDAEK	MEYVEKIISI	LEMQEFSEAL
(PRED)	sapa_10_8_3760	DLHIAELTVK	ESLEFSARMR	RPQSIPDAEK	MEYVEKIISI	LEMQEFSEAL
(PRED)	sapa_13_8_h0382	DLHIAELTVK	ESLEFSARMR	RPQSIPDAEK	MEYVEKIISI	LEMQEFSEAL
(PRED)	sapa_8_8_3750	DLHIAELTVK	ESLEFSARMR	RPQSIPDAEK	MEYVEKIISI	LEMQEFSEAL
(PRED)	sapa_11_8_h0383	DLHIAELTVK	ESLEFSARMR	RPQSIPDAEK	MEYVEKIISI	LEMQEFSEAL

(PRED)	sapa_5_8_3700	DLHIAELTVK	ESLEFSARMR	RPQSIPDAEK	MEYVEKIISI	LEMQEFSEAL
(PRED)	sapa_16_8_h0389	DLHIAELTVK	ESLEFSARMR	RPQSIPDAEK	MEYVEKIISV	LEMQEFSEAL
(PRED)	sapa_17_8_3730	DLHIAELTVK	ESLEFSARMR	RPQSIPDAEK	MEYVEKIISV	LEMQEFSEAL
(PRED)	sapa_2_8_h03860	DLHIAELTVK	ESLEFSARMR	RPQSIPDAEK	MEYVEKIISV	LEMQEFSEAL
(PRED)	sapa_7_8_3740	DLHIAELTVK	ESLEFSARMR	RPQSIPDAEK	MEYVEKIISV	LEMQEFSEAL
(PRED)	sapa_23_8_h0385	DLHIAELTVK	ESLEFSARMR	RPQSIPDAEK	MEYVEKIISV	LEMQEFSEAL
(PRED)	sapa_3_8_h03890	DLHIAELTVK	ESLEFSARMR	RPQSIPDAEK	MEYVEKIISV	LEMQEFSEAL
(PRED)	sapa_18_8_3730	DLHIAELTVK	ESLEFNARMR	RPQSIPDAEK	MEYVEKIISI	LEMQEFSEAL
(PRED)	sami_1_14_399	DIHIAELTVR	ESLQFSARMR	RPQSIPDTEK	MEYVEKIISI	LEMQEFSEAL
(PRED)	sace_4_8_h03690	DLHVAELTVK	ESLQFSARMR	RPQSIPDAEK	MEYVEKIISI	LEMQEFSEAL
(PRED)	saku_1_14_404	DLHTAELTVR	ESLQFSARMR	RPQSIPDVEK	MEYVEKIIDI	LEMQEFSEAL
(PRED)	sace_1_ynr070w	DLHVAELTVK	ESLQFSARMR	RPQSIPDAEK	MEYVEKIISI	LEMQEFSEAL
(PRED)	sace_49_8_h0383	DLHVAELTVK	ESLQFSARMR	RPQSIPDAEK	MEYVEKIISI	LEMQEFSEAL
(PRED)	saeu_1_2_b00130	DLHIAELTVK	ESLQFSARMR	RSQSVTDTEK	MEYVEKIMDI	LEMQGFSEAL
(PRED)	sauv_1_7_3	DLHVAELTVK	ESLQFSARMR	RSQFVSDAEK	MEYVEKIMDI	LEMKGFSEAL
(PRED)	sami_1_17_26	DIHIAELTVR	ESLQFSARMR	RPQSVPDTEK	MKYVEKIMDI	LGMQEYSEAL
(PRED)	zyba_1_02055_AN	DIHIAEMTVR	ESLVFSARLR	RPQSVPDEEK	LHSVENVIRL	LDMEEYSEAL
(PRED)	zyba_1_07912	DIHIAEMTVR	ESLVFSARLR	RPQSVPDEEK	LHSVENVIRL	LDMEEYSEAL
(PRED)	zyba_2_2_b00600	DIHIAEMTVR	ESLVFSARLR	RPQSVPDEEK	LHSVENVIRL	LDMEEYSEAL
(PRED)	zyba_3_3_c03460	DIHIAEMTVR	ESLVFSARLR	RPQSVPDEEK	LHSVENVIRL	LDMEEYSEAL
(PRED)	zyba_1_04634	DVHISEMTVR	ESLIFSARLR	RPQSIPEEEK	LRSVNTVIKL	LDMEEYSEAL
(PRED)	zyba_1_06675	DVHISEMTVR	ESLIFSARLR	RPQSIPEEEK	LRSVNTVMKL	LDMEEYSEAL
(PRED)	zyba_3_2_b02230	DVHISEMTVR	ESLIFSARLR	RPQSIPEEEK	LRSVNTVMKL	LDMEEYSEAL
(PRED)	zyba_2_1_a00860	DVHISEMTVR	ESLIFSARLR	RPQSIPEEEK	LRSVNTVMKL	LDMEEYSEAL
(PRED)	zyro_1_a04114g	DIHIAEMTVR	ESLIFSARLR	RPQSIPDAEK	VADAETVMKL	LDMEEYADAL
(PRED)	zyro_1_b14762g	DIHIAEMTVR	ESLVFSARLR	RPQSIPDAEK	VADAETVMKL	LDMEEYGDAL
(PRED)	zyba_2_14_n0149	DIHISEMTVR	ESLIFSSRLR	RPQSVPDEEK	LRDVENVIGL	LDMEAFSEAL
(PRED)	zyba_2_33_ag001	DIHISEMTVR	ESLIFSSRLR	RPQSVPDEEK	LRDVETVIGL	LEMQDYSEAL
(PRED)	lath_1_a01914g	DVHVKEMTVR	ESLQFAARMR	RPQSVPEAEK	MAYVEDIIEI	LDMEEYADAL
(PRED)	lawa_1_23_5161	DVHVKEMTVR	ESFQFAARMR	RPQSIPESEK	LAYVEKIIEI	LDMEDYAEAL
(PRED)	klae_1_14_n0012	DVHIKEMTVR	ESLQFSARMR	RPQTVSDEEK	LEYVERVLEM	LDMTSYAEAL
(PRED)	klla_1_d03432g	DVHIKEMTVR	ESLQFSARMR	RPLTVPDEEK	LDYVEKVIEI	LDMSAYGEAL
(PRED)	klma_1_1_a01880	DVHIKEMTVR	ESLQFSARTR	RPLSVSDEEK	LDYVEKVIQI	LDMEPYSEAL
(PRED)	klwi_1_33_ag001	DVHIKEMTVK	ESLQFSARMR	RPMSVPDEEK	MEYVDRVIEM	LDMGEYTEAL
(PRED)	teph_1_a04220	DVHIAELTVR	ESLQFSARLR	RPESISDDEK	LEYVEDVLEI	LDMEEYADAL
(PRED)	vapo_1_1037_47	DVHLAELTVR	EALIFSAMMR	RPESVPYSEK	LDFVEKIIEV	LEMGDYADAL
(PRED)	pata_1_2_b05590	DLHIAELTVR	ESLRFAARLR	RPLSVPDAEK	MEYVEEIINI	LQMQDYADAV
(PRED)	wian_1_3_c04380	DLHIAEMTVR	ESLQFAARLR	RPKSVPDSEK	LEYVEKIIKI	LRMETYSEAI
(PRED)	wian_1_3_c04390	DLHIAEMTVR	ESLQFAARLR	RPKSVSDSEK	LDYVEKIIKI	LQMEAYSEAI
(PRED)	wian_1_7_g01010	DLHISELTVR	ESLQFAARLR	RPKSVPDSEK	LDYVEKIIKI	LQMEPYSEAI
(PRED)	bain_1_1_a00100	DLHVSESTVR	ESLRFSAKLR	RPTSISDEEK	FDYVERIIEI	LGMTNYADAI
(PRED)	bain_1_17_q0038	DLHVSESTVR	ESLRFSAKLR	RPTSVSDEEK	FDYVERIIEI	LGMTNYADAI
(PRED)	bain_1_8_h00410	DLHVAELTVR	ESLRFSARLR	RPASVPDSEK	LDYVETIIDI	LGMQAYAEAV
(PRED)	caal_1_19_5759	DIHFSEVTVR	ESLQFAARLR	RSNDVSDAEK	LEYVEKIIDV	LDMRGYADAV
(PRED)	caal_11_25_y002	DIHFSEVTVR	ESLQFAARLR	RSNDVSDAEK	LEYVEKIIDV	LDMRGYADAV
(PRED)	caal_4_4_d03320	DIHFSEVTVR	ESLQFAARLR	RSNDVSDAEK	LEYVEKIIDV	LDMRGYADAV
(PRED)	caal_12_26_z005	DIHFSEVTVR	ESLQFAARLR	RSNDVSDAEK	LEYVEKIIDV	LDMRGYADAV
(PRED)	caal_5_30_ad005	DIHFSEVTVR	ESLQFAARLR	RSNDVSDAEK	LEYVEKIIDV	LDMRGYADAV
(PRED)	caal_8_3_c03320	DIHFSEVTVR	ESLQFAARLR	RSNDVSDAEK	LEYVEKIIDV	LDMRGYADAV
(PRED)	caal_6_4_d03280	DIHFSEVTVR	ESLQFAARLR	RSNDVSDAEK	LEYVEKIIDV	LDMRGYADAV
(PRED)	caal_10_3_c0334	DIHFSEVTVR	ESLQFAARLR	RSNDVSDAEK	LEYVEKIIDV	LDMRGYADAV
(PRED)	caal_3_29_ac005	DIHFSEVTVR	ESLQFAARLR	RSNDVSDAEK	LEYVEKIIDV	LDMRGYADAV
(PRED)	caal_2_04989	DIHFSEVTVR	ESLQFAARLR	RSNDVSDAEK	LEYVEKIIDV	LDMRGYADAV
(PRED)	cadu_1_64350	DIHFSEVTVR	ESLQFAARLR	RSNDVSDAEK	LEYVEKIIDV	LDMRGYADAV
(PRED)	caor_1_h02090	DIHVSEVTVR	ESLQFAARLR	RSNDVSDAEK	LDYVEKIIDV	LDMGLYADAV
(PRED)	capa_1_600750	DIHVSEVTVR	ESLQFAARLR	RSNDVSDVEK	LDYVEKIIDV	LDMGLYADAI
(PRED)	loel_1_04930	DIHVSEVTVR	ESLQFAARLR	RPKGVSDKEK	LDYVEKIIDV	LDMSTYADAI
(PRED)	spar_1_5_e03260	DIHVTEVTVR	ESLQFAARLR	RSQDVSDEEK	LNYVEKIIDV	LDMNDYADSV
(PRED)	sppa_1_7_g03160	DIHVTEVTVR	ESLQFAARLR	RPQDVSDEEK	LNYVEKIIDV	LDMNDYADAV
(PRED)	catr_1_01205	DIHAEELTVR	ESLQFAARLR	RSNDVSDEEK	LDYVEKIIDV	LDMKGYADAI
(PRED)	catr_1_05498	DIHCEEVTVR	ESLQFAARLR	RSNDVSDEEK	LDYVEKIIDV	LDMKPYADAI
(PRED)	catr_1_05971	DIHCEEVTVR	ESLQFAARLR	RSNDVSDEEK	LDYVEKIIDV	LDMKGYADAI
(PRED)	deha_1_a03696g	DIHVESLTVR	ESLIFSARLR	RINDADDAEK	LDYVEKIIKA	LDMEDYADAL
(PRED)	deha_2_5_e00720	DIHVESLTVR	ESLIFSARLR	RINDSDDAEK	LDYVEKIIKA	LDMEDYADAL
(PRED)	scst_1_3_c02890	DIHVAEVTVR	ESLRFSARLR	RSNDISDAEK	LEYVEKIIHV	LNMEDYADAL
(PRED)	mebi_1_8_h00300	DIHMAESTVR	ESLQFAARMR	RPKSIPDAEK	LDYVEQIIKV	LDMEAYADAI
(PRED)	lakl_1_h21010g	DIHTQELTVR	ESLQFSARLR	RPDSVPDAEK	MEYVEKIIVV	LGMQDYSDAV
(PRED)	caar_1_13_m0142	DLHIAELTVR	ESLNFAARMR	RPPSVPDEEK	LAYVQTILEI	LNMEDYADSV
(PRED)	caar_1_14_n0143	DLHISELTVR	ESLIFAARLR	RPISVPDEEK	LAYVETIISI	LNMEEYADSI
(PRED)	hapo_1_1_a07220	DLHIAELTVR	ESLIFAARLR	RPADVPDEEK	IAYVDKILNI	LNMEEYADSI
(PRED)	ogpa_1_1_a01680	DLHIAELTVR	ESLIFAARLR	RPADVPDEEK	IAYVDKILHI	LNMEEYADSV
(PRED)	piku_1_96_cr001	DLHIAELTVR	ESLIFSARLR	RPHSVPDKEK	IDYVDEVIRI	LRMEEYSDSI
(PRED)	pime_1_4_d03240	DLHIAELTVR	ESLIFSARLR	RPSSVPDDEK	IGYVDEVIRI	LNMEEYVDSL
(PRED)	pime_1_1_a12110	DLHIAELTVR	ESLIFSARLR	RPASVSDEEK	IKYVDQVMEI	LNMNEYADSI
(PRED)	piku_1_227_hs00	DLHVAELTVK	ESLLFSARLR	RPRSVPDEEK	IEYVDKVMKI	LNMEDYADSI

```
(PRED) pime_1_5_e05800 DLHISELTVK  ESLLFSARLR  RPKSVPDAEK  LEYVETVMKI  LHMEDYADSI
(PRED) pime_1_1_a07690 DLHIAELTVK  ESLLFSARLR  RPMSVPDEEK  IDYVNMVMKF  LHMEDYADSI
(PRED) debr_2_5_e03380 DIHIAELTVR  ESLVFAARLR  RPYEVPDAEK  LEYVDRIMEI  LHMDEYADAV
(PRED) kopa_1_2_b10040 DLHISELTVR  ESLIFAAKLR  RPLSVPVAEK  IQYVDQVIEI  LQMTKYKDAV
(PRED) kopa_2_7_g00500 DLHISELTVR  ESLIFAAKLR  RPLSVPVEEK  IQYVDQVIEI  LQMTNYKDAV
(PRED) asru_1_13_m0119 DLHISELTVR  ESLQFAARLR  RPQSVPEEEK  LSYVETVIDL  LSMRSYADAV
(PRED) asru_1_15_o0045 DLHIAELTVR  ESLQFAARLR  RPTSVSEEEK  MDYVEKIIDV  LQMRSFANAV
(PRED) wian_1_1_a02920 DLHLDELTVR  ESLVFAARMR  RPANVPDAEK  LDYVEKIIKI  LDMELYADAI
(PRED) wian_1_1_a02930 DLHLDELTVR  ESLVFAARMR  RPANVPDAEK  LDYVEKIIKI  LDMELYADAI

                       .........1310.........1320.........1330.........1340.........1350
(PRED) asac_1_6_f03560 VGDAGYGLNV  EQRKKLSIGV  ELVAKPSLLL  FLDEPTSGLD  SQSSWAIVQL
(PRED) ergo_1_abr125c  VGDAGYGLNV  EQRKKLSIGV  ELVAKPSLLL  FLDEPTSGLD  SQSSWAIVQL
(PRED) ercy_1_3604     VGDVGYGLNV  GQRKKLSIGV  ELVAKPSLLL  FLDEPTSGLD  SQSSWAIVQL
(PRED) cagl_1_i04862g  VGDVGRGLNV  EQRKKLSIGV  ELVAKPDLLL  FLDEPTSGLD  SQSSWAIVQL
(PRED) kaaf_1_c00830   VGAVGNGLNV  EQRKKLSIGV  ELVAKPDLLL  FLDEPTSGLD  SQSSWAIIQL
(PRED) kana_1_k01350   VGALGSGLNV  EQRKKLSIGV  ELAAKPDLLL  FLDEPTSGLD  SQSSWAIIQL
(PRED) saar_1_2_b02590 VGEVGCGLNV  EQRKKLSIGV  ELVAKPDLLL  FLDEPTSGLD  SQSSWAIIQL
(PRED) sace_1_ydr011w  VGEVGCGLNV  EQRKKLSIGV  ELVAKPDLLL  FLDEPTSGLD  SQSSWAIIQL
(PRED) sace_16_1_a0238 VGEVGCGLNV  EQRKKLSIGV  ELVAKPDLLL  FLDEPTSGLD  SQSSWAIIQL
(PRED) sace_45_1_a0242 VGEVGCGLNV  EQRKKLSIGV  ELVAKPDLLL  FLDEPTSGLD  SQSSWAIIQL
(PRED) sace_48_1_a0238 VGEVGCGLNV  EQRKKLSIGV  ELVAKPDLLL  FLDEPTSGLD  SQSSWAIIQL
(PRED) sace_60_4_d0244 VGEVGCGLNV  EQRKKLSIGV  ELVAKPDLLL  FLDEPTSGLD  SQSSWAIIQL
(PRED) sace_52_1_a0240 VGEVGCGLNV  EQRKKLSIGV  ELVAKPDLLL  FLDEPTSGLD  SQSSWAIIQL
(PRED) sace_46_1_a0240 VGEVGCGLNV  EQRKKLSIGV  ELVAKPDLLL  FLDEPTSGLD  SQSSWAIIQL
(PRED) sace_25_1_a0240 VGEVGCGLNV  EQRKKLSIGV  ELVAKPDLLL  FLDEPTSGLD  SQSSWAIIQL
(PRED) sace_24_1_2300  VGEVGCGLNV  EQRKKLSIGV  ELVAKPDLLL  FLDEPTSGLD  SQSSWAIIQL
(PRED) sace_47_1_a0240 VGEVGCGLNV  EQRKKLSIGV  ELVAKPDLLL  FLDEPTSGLD  SQSSWAIIQL
(PRED) sace_7_1_a02410 VGEVGCGLNV  EQRKKLSIGV  ELVAKPDLLL  FLDEPTSGLD  SQSSWAIIQL
(PRED) sace_59_110_df0 VGEVGCGLNV  EQRKKLSIGV  ELVAKPDLLL  FLDEPTSGLD  SQSSWAIIQL
(PRED) sace_56_1_a0202 VGEVGCGLNV  EQRKKLSIGV  ELVAKPDLLL  FLDEPTSGLD  SQSSWAIIQL
(PRED) sace_40_1_a0239 VGEVGCGLNV  EQRKKLSIGV  ELVAKPDLLL  FLDEPTSGLD  SQSSWAIIQL
(PRED) sace_15_1_a0242 VGEVGCGLNV  EQRKKLSIGV  ELVAKPDLLL  FLDEPTSGLD  SQSSWAIIQL
(PRED) sace_37_1_a0243 VGEVGCGLNV  EQRKKLSIGV  ELVAKPDLLL  FLDEPTSGLD  SQSSWAIIQL
(PRED) sace_9_1_a02440 VGEVGCGLNV  EQRKKLSIGV  ELVAKPDLLL  FLDEPTSGLD  SQSSWAIIQL
(PRED) sace_22_1_2300  VGEVGCGLNV  EQRKKLSIGV  ELVAKPDLLL  FLDEPTSGLD  SQSSWAIIQL
(PRED) sace_29_1_2290  VGEVGCGLNV  EQRKKLSIGV  ELVAKPDLLL  FLDEPTSGLD  SQSSWAIIQL
(PRED) sace_34_1_2320  VGEVGCGLNV  EQRKKLSIGV  ELVAKPDLLL  FLDEPTSGLD  SQSSWAIIQL
(PRED) sace_58_25_y007 VGEVGCGLNV  EQRKKLSIGV  ELVAKPDLLL  FLDEPTSGLD  SQSSWAIIQL
(PRED) sace_23_1_2290  VGEVGCGLNV  EQRKKLSIGV  ELVAKPDLLL  FLDEPTSGLD  SQSSWAIIQL
(PRED) sace_6_120_dp00 VGEVGCGLNV  EQRKKLSIGV  ELVAKPDLLL  FLDEPTSGLD  SQSSWAIIQL
(PRED) sace_57_1_a0241 VGEVGCGLNV  EQRKKLSIGV  ELVAKPDLLL  FLDEPTSGLD  SQSSWAIIQL
(PRED) sace_17_1_a0241 VGEVGCGLNV  EQRKKLSIGV  ELVAKPDLLL  FLDEPTSGLD  SQSSWAIIQL
(PRED) sace_21_1_2310  VGEVGCGLNV  EQRKKLSIGV  ELVAKPDLLL  FLDEPTSGLD  SQSSWAIIQL
(PRED) sace_49_1_a0246 VGEVGCGLNV  EQRKKLSIGV  ELVAKPDLLL  FLDEPTSGLD  SQSSWAIIQL
(PRED) sace_8_2_b02430 VGEVGCGLNV  EQRKKLSIGV  ELVAKPDLLL  FLDEPTSGLD  SQSSWAIIQL
(PRED) sace_31_1_2300  VGEVGCGLNV  EQRKKLSIGV  ELVAKPDLLL  FLDEPTSGLD  SQSSWAIIQL
(PRED) sace_50_1_a0241 VGEVGCGLNV  EQRKKLSIGV  ELVAKPDLLL  FLDEPTSGLD  SQSSWAIIQL
(PRED) sace_4_1_a02360 VGEVGCGLNV  EQRKKLSIGV  ELVAKPDLLL  FLDEPTSGLD  SQSSWAIIQL
(PRED) sace_2_1_a02390 VGEVGCGLNV  EQRKKLSIGV  ELVAKPDLLL  FLDEPTSGLD  SQSSWAIIQL
(PRED) sace_5_33_ag005 VGEVGCGLNV  EQRKKLSIGV  ELVAKPDLLL  FLDEPTSGLD  SQSSWAIIQL
(PRED) sapa_11_1_a0247 VGEVGCGLNV  EQRKKLSIGV  ELVAKPDLLL  FLDEPTSGLD  SQSSWAIVQL
(PRED) sapa_25_1_a0246 VGEVGCGLNV  EQRKKLSIGV  ELVAKPDLLL  FLDEPTSGLD  SQSSWAIVQL
(PRED) sapa_4_1_a02470 VGEVGCGLNV  EQRKKLSIGV  ELVAKPDLLL  FLDEPTSGLD  SQSSWAIVQL
(PRED) sapa_5_1_2350   VGEVGCGLNV  EQRKKLSIGV  ELVAKPDLLL  FLDEPTSGLD  SQSSWAIVQL
(PRED) sapa_9_1_2360   VGEVGCGLNV  EQRKKLSIGV  ELVAKPDLLL  FLDEPTSGLD  SQSSWAIVQL
(PRED) sapa_14_1_a0244 VGEVGCGLNV  EQRKKLSIGV  ELVAKPDLLL  FLDEPTSGLD  SQSSWAIVQL
(PRED) sapa_8_1_2350   VGEVGCGLNV  EQRKKLSIGV  ELVAKPDLLL  FLDEPTSGLD  SQSSWAIVQL
(PRED) sapa_17_1_2380  VGEVGCGLNV  EQRKKLSVGV  ELVAKPDLLL  FLDEPTSGLD  SQSSWAIVQL
(PRED) sapa_7_1_2370   VGEVGCGLNV  EQRKKLSVGV  ELVAKPDLLL  FLDEPTSGLD  SQSSWAIVQL
(PRED) sapa_2_1_a02460 VGEVGCGLNV  EQRKKLSVGV  ELVAKPDLLL  FLDEPTSGLD  SQSSWAIVQL
(PRED) sapa_23_1_a0248 VGEVGCGLNV  EQRKKLSVGV  ELVAKPDLLL  FLDEPTSGLD  SQSSWAIVQL
(PRED) sapa_3_1_a02470 VGEVGCGLNV  EQRKKLSVGV  ELVAKPDLLL  FLDEPTSGLD  SQSSWAIVQL
(PRED) sapa_18_1_2390  VGEVGCGLNV  EQRKKLSIGV  ELVAKPDLLL  FLDEPTSGLD  SQSSWAIVQL
(PRED) sami_1_4_244    VGEVGCGLNV  EQRKKLSIGV  ELVAKPDLLL  FLDEPTSGLD  SQSSWAIIQL
(PRED) saku_1_4_262    VGEVGCGLNV  EQRKKLSIGV  ELVAKPDLLL  FLDEPTSGLD  SQSSWAIIQL
(PRED) saba_1_58_bf002 VGEVGCGLNV  EQRKKLSIGV  ELVAKPDLLL  FLDEPTSGLD  SQSSWSIIQL
(PRED) saeu_1_4_d02400 VGEVGCGLNV  EQRKKLSIGV  ELVAKPDLLL  FLDEPTSGLD  SQSSWAIIQL
(PRED) naca_1_e01640   VGEIGRGLNV  EQRKKLSIGV  ELVAKPDLLL  FLDEPTSGLD  SQSSWAIIQL
(PRED) nada_1_g01850   VGDVGRGLNV  EQRKKLSIGV  ELVAKPDLLL  FLDEPTSGLD  SQSSWAIIQL
(PRED) naca_1_e01630   VGAIGCGLNV  EQRKKLSIGV  ELVAKPDLLL  FLDEPTSGLD  SQSSWAIVQL
(PRED) nada_1_g01840   VGAIGYGLNV  EQRKKLSIGV  ELCAKPDLLL  FLDEPTSGLD  SQSSWAIIQL
(PRED) kaaf_1_c00820   VGTVGSGLNV  EQRKKLSIGV  ELVAKPDLLL  FLDEPTSGLD  SQSSWAIVQL
(PRED) teph_1_m00640   VGGIGRGLNV  EQRKKLSIGV  ELVAKPDLLL  FLDEPTSGLD  SQSSWSIIQL
(PRED) vapo_1_1036_28  VGAVGRGLNV  EQRKKLSIGV  ELVAKPDLLL  FLDEPTSGLD  SQSSWSIVQL
```

(PRED)	tebl_1_i01760	VGELGRGLNV	EQRKKLSIGV	ELVAKPDLLL	FLDEPTSGLD	SQSSWAIVQL
(PRED)	tode_1_d04040	VGEPGAGLSV	EQRKKLSIGV	ELVAKPDLLL	FLDEPTSGLD	SQSAWAIVQL
(PRED)	naca_1_e01650	VGEIGRGLNV	EQRKKLSIGV	ELVAKPDLLL	FLDEPTSGLD	SQSSWAIIQL
(PRED)	tebl_1_g02820	VGAIGCGLNV	EQRKKLSIGV	ELVAKPDLLL	FLDEPTSGLD	SQSSWAIVQL
(PRED)	lakl_1_c11616g	VGDVGYGLNV	EQRKKLSIGV	ELAAKPNLLL	FLDEPTSGLD	SQASWAIVQL
(PRED)	saar_1_8_h03780	VGDIGYGLNV	EQRKKLSIGV	ELVGKPDLLL	FLDEPTSGLD	SQSAWAIVKM
(PRED)	sace_14_7_g0015	VGEIGYGLNV	EQRKKLSIGV	ELVGKPDLLL	FLDEPTSGLD	SQSAWAVVKM
(PRED)	sace_15_7_g0387	VGEIGYGLNV	EQRKKLSIGV	ELVGKPDLLL	FLDEPTSGLD	SQSAWAVVKM
(PRED)	sace_24_8_3780	VGEIGYGLNV	EQRKKLSIGV	ELVGKPDLLL	FLDEPTSGLD	SQSAWAVVKM
(PRED)	sace_40_8_h0383	VGEIGYGLNV	EQRKKLSIGV	ELVGKPDLLL	FLDEPTSGLD	SQSAWAVVKM
(PRED)	sace_6_169_fm00	VGEIGYGLNV	EQRKKLSIGV	ELVGKPDLLL	FLDEPTSGLD	SQSAWAVVKM
(PRED)	sace_19_7_3840	VGEIGYGLNV	EQRKKLSIGV	ELVGKPDLLL	FLDEPTSGLD	SQSAWAVVKM
(PRED)	sace_32_7_3770	VGEIGYGLNV	EQRKKLSIGV	ELVGKPDLLL	FLDEPTSGLD	SQSAWAVVKM
(PRED)	sace_56_17_q011	VGEIGYGLNV	EQRKKLSIGV	ELVGKPDLLL	FLDEPTSGLD	SQSAWAVVKM
(PRED)	sace_5_78_bz001	VGEIGYGLNV	EQRKKLSIGV	ELVGKPDLLL	FLDEPTSGLD	SQSAWAVVKM
(PRED)	sace_2_8_h03860	VGEIGYGLNV	EQRKKLSIGV	ELVGKPDLLL	FLDEPTSGLD	SQSAWAVVKM
(PRED)	sace_53_29_ac00	VGEIGYGLNV	EQRKKLSIGV	ELVGKPDLLL	FLDEPTSGLD	SQSAWAVVKM
(PRED)	sace_17_7_g0393	VGEIGYGLNV	EQRKKLSIGV	ELVGKPDLLL	FLDEPTSGLD	SQSAWAVVKM
(PRED)	sace_25_7_g0388	VGEIGYGLNV	EQRKKLSIGV	ELVGKPDLLL	FLDEPTSGLD	SQSAWAVVKM
(PRED)	sace_37_7_g0385	VGEIGYGLNV	EQRKKLSIGV	ELVGKPDLLL	FLDEPTSGLD	SQSAWAVVKM
(PRED)	sace_9_7_g00180	VGEIGYGLNV	EQRKKLSIGV	ELVGKPDLLL	FLDEPTSGLD	SQSAWAVVKM
(PRED)	sace_60_6_f0335	VGEIGYGLNV	EQRKKLSIGV	ELVGKPDLLL	FLDEPTSGLD	SQSAWAVVKM
(PRED)	sace_59_336_lx0	VGEIGYGLNV	EQRKKLSIGV	ELVGKPDLLL	FLDEPTSGLD	SQSAWAVVKM
(PRED)	sace_31_7_3780	VGEIGYGLNV	EQRKKLSIGV	ELVGKPDLLL	FLDEPTSGLD	SQSAWAVVKM
(PRED)	sace_34_8_3770	VGEIGYGLNV	EQRKKLSIGV	ELVGKPDLLL	FLDEPTSGLD	SQSAWAVVKM
(PRED)	sace_58_71_bs00	VGEIGYGLNV	EQRKKLSIGV	ELVGKPDLLL	FLDEPTSGLD	SQSAWAVVKM
(PRED)	sace_7_7_g03880	VGEIGYGLNV	EQRKKLSIGV	ELVGKPDLLL	FLDEPTSGLD	SQSAWAVVKM
(PRED)	sace_35_7_3840	VGEIGYGLNV	EQRKKLSIGV	ELVGKPDLLL	FLDEPTSGLD	SQSAWAVVKM
(PRED)	sace_43_7_g0387	VGEIGYGLNV	EQRKKLSIGV	ELVGKPDLLL	FLDEPTSGLD	SQSAWAVVKM
(PRED)	sace_57_8_h0390	VGEIGYGLNV	EQRKKLSIGV	ELVGKPDLLL	FLDEPTSGLD	SQSAWAVVKM
(PRED)	sace_45_7_g0389	VGEIGYGLNV	EQRKKLSIGV	ELVGKPDLLL	FLDEPTSGLD	SQSAWAVVKM
(PRED)	sace_46_8_h0391	VGEIGYGLNV	EQRKKLSIGV	ELVGKPDLLL	FLDEPTSGLD	SQSAWAVVKM
(PRED)	sace_23_7_3860	VGEIGYGLNV	EQRKKLSIGV	ELVGKPDLLL	FLDEPTSGLD	SQSAWAVVKM
(PRED)	sace_21_7_3790	VGEIGYGLNV	EQRKKLSIGV	ELVGKPDLLL	FLDEPTSGLD	SQSAWAVVKM
(PRED)	sace_8_73_bu001	VGEIGYGLNV	EQRKKLSIGV	ELVGKPDLLL	FLDEPTSGLD	SQSAWAVVKM
(PRED)	sapa_1_8_h03820	VGEIGYGLNV	EQRKKLSIGV	ELVGKPDLLL	FLDEPTSGLD	SQSAWAIVKM
(PRED)	sapa_21_8_h0387	VGEIGYGLNV	EQRKKLSIGV	ELVGKPDLLL	FLDEPTSGLD	SQSAWAIVKM
(PRED)	sapa_20_8_h0386	VGEIGYGLNV	EQRKKLSIGV	ELVGKPDLLL	FLDEPTSGLD	SQSAWAIVKM
(PRED)	sapa_22_8_h0390	VGEIGYGLNV	EQRKKLSIGV	ELVGKPDLLL	FLDEPTSGLD	SQSAWAIVKM
(PRED)	sapa_25_8_h0387	VGEIGYGLNV	EQRKKLSIGV	ELVGKPDLLL	FLDEPTSGLD	SQSAWAIVKM
(PRED)	sapa_6_8_3750	VGEIGYGLNV	EQRKKLSIGV	ELVGKPDLLL	FLDEPTSGLD	SQSAWAIVKM
(PRED)	sapa_9_8_3720	VGEIGYGLNV	EQRKKLSIGV	ELVGKPDLLL	FLDEPTSGLD	SQSAWAIVKM
(PRED)	sapa_19_8_h0390	VGEIGYGLNV	EQRKKLSIGV	ELVGKPDLLL	FLDEPTSGLD	SQSAWAIVKM
(PRED)	sapa_24_8_h0385	VGEIGYGLNV	EQRKKLSIGV	ELVGKPDLLL	FLDEPTSGLD	SQSAWAIVKM
(PRED)	sapa_4_8_h03850	VGEIGYGLNV	EQRKKLSIGV	ELVGKPDLLL	FLDEPTSGLD	SQSAWAIVKM
(PRED)	sapa_10_8_3760	VGEIGYGLNV	EQRKKLSIGV	ELVGKPDLLL	FLDEPTSGLD	SQSAWAIVKM
(PRED)	sapa_13_8_h0382	VGEIGYGLNV	EQRKKLSIGV	ELVGKPDLLL	FLDEPTSGLD	SQSAWAIVKM
(PRED)	sapa_8_8_3750	VGEIGYGLNV	EQRKKLSIGV	ELVGKPDLLL	FLDEPTSGLD	SQSAWAIVKM
(PRED)	sapa_11_8_h0383	VGEIGYGLNV	EQRKKLSIGV	ELVGKPDLLL	FLDEPTSGLD	SQSAWAIVKM
(PRED)	sapa_5_8_3700	VGEIGYGLNV	EQRKKLSIGV	ELVGKPDLLL	FLDEPTSGLD	SQSAWAIVKM
(PRED)	sapa_16_8_h0389	VGEIGYGLNV	EQRKKLSIGV	ELVGKPDLLL	FLDEPTSGLD	SQSAWAIVKM
(PRED)	sapa_17_8_3730	VGEIGYGLNV	EQRKKLSIGV	ELVGKPDLLL	FLDEPTSGLD	SQSAWAIVKM
(PRED)	sapa_2_8_h03860	VGEIGYGLNV	EQRKKLSIGV	ELVGKPDLLL	FLDEPTSGLD	SQSAWAIVKM
(PRED)	sapa_7_8_3740	VGEIGYGLNV	EQRKKLSIGV	ELVGKPDLLL	FLDEPTSGLD	SQSAWAIVKM
(PRED)	sapa_23_8_h0385	VGEIGYGLNV	EQRKKLSIGV	ELVGKPDLLL	FLDEPTSGLD	SQSAWAIVKM
(PRED)	sapa_3_8_h03890	VGEIGYGLNV	EQRKKLSIGV	ELVGKPDLLL	FLDEPTSGLD	SQSAWAIVKM
(PRED)	sapa_18_8_3730	VGEIGYGLNV	EQRKKLSIGV	ELVGKPDLLL	FLDEPTSGLD	SQSAWAIVKM
(PRED)	sami_1_14_399	VGEIGHGLNV	EQRKKLSIGV	ELVGKPDLLL	FLDEPTSGLD	SQSAWAVIKV
(PRED)	sace_4_8_h03690	VGEIGYGLNV	EQRKKLSIGV	ELVGKPDLLL	FLDEPTSGLD	SQSAWAVVKM
(PRED)	saku_1_14_404	VGEIGYGLNV	EQRKKLSIGV	ELVGKPDLLL	FLDEPTSGLD	SQSAWSIVKM
(PRED)	sace_1_ynr070w	VGEIGYGLNV	EQRKKLSIGV	ELVGKPDLLL	FLDEPTSGLD	SQSAWAVVKM
(PRED)	sace_49_8_h0383	VGEIGYGLNV	EQRKKLSIGV	ELVGKPDLLL	FLDEPTSGLD	SQSAWAVVKM
(PRED)	saeu_1_2_b00130	VGETGYGLNV	EQRKKLSIGV	ELVGKPDLLL	FLDEPTSGLD	SQSAWAIVKM
(PRED)	sauv_1_7_3	VGEVGYGLNV	EQRKKLSIGV	ELVGKPDLLL	FLDEPTSGLD	SQSAWAIVKM
(PRED)	sami_1_17_26	VGEIGYGLNV	EQRKKLSIGV	ELVGKPDLLL	FLDEPTSGLD	SQAAWAIVTM
(PRED)	zyba_1_02055_AN	VGALGCGLNV	EQRKKLSIGV	ELVAKPDLLL	FLDEPTSGLD	SQSSWAIVQL
(PRED)	zyba_1_07912	VGALGCGLNV	EQRKKLSIGV	ELVAKPDLLL	FLDEPTSGLD	SQSSWAIVQL
(PRED)	zyba_2_2_b00600	VGALGCGLNV	EQRKKLSIGV	ELVAKPDLLL	FLDEPTSGLD	SQSSWAIVQL
(PRED)	zyba_3_3_c03460	VGALGCGLNV	EQRKKLSIGV	ELVAKPDLLL	FLDEPTSGLD	SQSSWAIVQL
(PRED)	zyba_1_04634	VGDLGAGLNV	EQRKKLSIGV	ELVAKPDLLL	FLDEPTSGLD	SQSSWAIVQL
(PRED)	zyba_1_06675	VGDLGAGLNV	EQRKKLSIGV	ELVAKPDLLL	FLDEPTSGLD	SQSSWAIVQL
(PRED)	zyba_3_2_b02230	VGDLGAGLNV	EQRKKLSIGV	ELVAKPDLLL	FLDEPTSGLD	SQSSWAIVQL
(PRED)	zyba_2_1_a00860	VGDLGAGLNV	EQRKKLSIGV	ELVAKPDLLL	FLDEPTSGLD	SQSSWAIVQL
(PRED)	zyro_1_a04114g	VGGLGAGLNV	EQRKKLSIGV	ELVSKPDLLL	FLDEPTSGLD	SQSSWAIVQL
(PRED)	zyro_1_b14762g	VGGLGAGLNV	EQRKKLSIGV	ELVAKPDLLL	FLDEPTSGLD	SQSSWAIVQL

(PRED)	zyba_2_14_n0149	VGALGAGLNV	EQRKKLSIGV	ELVAKPDLLL	FLDEPTSGLD	SQSSWAIVQL
(PRED)	zyba_2_33_ag001	VGALGAGLNV	EQRKKLSIGV	ELVAKPDLLL	FLDEPTSGLD	SQSSWAIVQL
(PRED)	lath_1_a01914g	VGDVGYGLNV	EQRKKVSIGV	ELAAKPDLLL	FLDEPTSGLD	SQSSWAIVQL
(PRED)	lawa_1_23_5161	IGDVGYGLNV	EQRKKVSIGV	ELAAKPDLLL	FLDEPTSGLD	SQSSWAIVQL
(PRED)	klae_1_14_n0012	VGDIGYGLNV	EQRKKLSIAV	ELVAKPSLLL	FLDEPTSGLD	SQSAWSIVQL
(PRED)	klla_1_d03432g	VGNIGYGLNV	EQRKKLSIAV	ELVAKPNLLL	FLDEPTSGLD	SQSAWAIVQL
(PRED)	klma_1_1_a01880	VGDIGYGLNV	EQRKKLSIAV	ELVAKPNLLL	FLDEPTSGLD	SQSAWSIVQL
(PRED)	klwi_1_33_ag001	VGDIGYGLNV	EQRKKLSIGV	ELVAKPSLLL	FLDEPTSGLD	SQSAWAIIQL
(PRED)	teph_1_a04220	VGEVGMGLNV	EQRKKLSIGV	ELAAKPDLLL	FLDEPTSGLD	SQSAWAIVRL
(PRED)	vapo_1_1037_47	VGLEGSGLNV	EQRKKLSIGV	ELVARPDILL	FVDEPTSGLD	SQEAWSIVQL
(PRED)	pata_1_2_b05590	AGDPGYGLNV	EQRKKLSIAT	ELVAKPDLLL	FLDEPTSGLD	SQSAWAIVQL
(PRED)	wian_1_3_c04380	IGTLGSGLNV	EQRKKLSIGV	ELVAKPSLLL	FLDEPTSGLD	SQSAWAIVHL
(PRED)	wian_1_3_c04390	VGTLGSGLNV	EQRKKLSIGV	ELVAKPSLLL	FLDEPTSGLD	SQSAWAIVHL
(PRED)	wian_1_7_g01010	VGVLGSGLNV	EQRKKLSIGV	ELVAKPSLLL	FLDEPTSGLD	SQSAWAIIHL
(PRED)	bain_1_1_a00100	VGDVGYGLNV	EQRKKLSIGV	ELVAKPSLLL	FLDEPTSGLD	SQSAWAIVKL
(PRED)	bain_1_17_q0038	VGDVGYGLNV	EQRKKLSIGV	ELVAKPSLLL	FLDEPTSGLD	SQSAWAIVKL
(PRED)	bain_1_8_h00410	VGVIGNGLNV	EQRKKLSVGV	ELVAKPSLLL	FLDEPTSGLD	SQSAWSIVKL
(PRED)	caal_1_19_5759	VGRLGNGLNV	EQRKKLSIGV	ELVAKPSLLL	FLDEPTSGLD	SQSAWAIVKL
(PRED)	caal_11_25_y002	VGRLGNGLNV	EQRKKLSIGV	ELVAKPSLLL	FLDEPTSGLD	SQSAWAIVKL
(PRED)	caal_4_4_d03320	VGRLGNGLNV	EQRKKLSIGV	ELVAKPSLLL	FLDEPTSGLD	SQSAWAIVKL
(PRED)	caal_12_26_z005	VGRLGNGLNV	EQRKKLSIGV	ELVAKPSLLL	FLDEPTSGLD	SQSAWAIVKL
(PRED)	caal_5_30_ad005	VGRLGNGLNV	EQRKKLSIGV	ELVAKPSLLL	FLDEPTSGLD	SQSAWAIVKL
(PRED)	caal_8_3_c03320	VGRLGNGLNV	EQRKKLSIGV	ELVAKPSLLL	FLDEPTSGLD	SQSAWAIVKL
(PRED)	caal_6_4_d03280	VGRLGNGLNV	EQRKKLSIGV	ELVAKPSLLL	FLDEPTSGLD	SQSAWAIVKL
(PRED)	caal_10_3_c0334	VGRLGNGLNV	EQRKKLSIGV	ELVAKPSLLL	FLDEPTSGLD	SQSAWAIVKL
(PRED)	caal_3_29_ac005	VGRLGNGLNV	EQRKKLSIGV	ELVAKPSLLL	FLDEPTSGLD	SQSAWAIVKL
(PRED)	caal_2_04989	VGRLGNGLNV	EQRKKLSIGV	ELVAKPSLLL	FLDEPTSGLD	SQSAWAIVKL
(PRED)	cadu_1_64350	VGRLGNGLNV	EQRKKLSIGV	ELVAKPSLLL	FLDEPTSGLD	SQSAWAIVKL
(PRED)	caor_1_h02090	VGRSGNGLNV	EQRKKLSIGV	ELVAKPSLLL	FLDEPTSGLD	SQSAWAIVKL
(PRED)	capa_1_600750	VGRSGNGLNV	EQRKKLSIGV	ELVAKPSLLL	FLDEPTSGLD	SQSAWAIVKL
(PRED)	loel_1_04930	VGRSGNGLNV	EQRKKLSIGV	ELVAKPSLLL	FLDEPTSGLD	SQSAWAIVKL
(PRED)	spar_1_5_e03260	VGRMGNGLNV	EQRKKLSIGV	ELVAKPTLLL	FLDEPTSGLD	SQSAWAIIKL
(PRED)	sppa_1_7_g03160	VGRPGNGLNV	EQRKKLSIGV	ELVAKPTLLL	FLDEPTSGLD	SQSAWAIVKL
(PRED)	catr_1_01205	VGKLGNGLNV	EQRKKLSIGV	ELVAKPSLLL	FLDEPTSGLD	SQSAWSIIKI
(PRED)	catr_1_05498	VGRLGNGLNV	EQRKKLSIGV	ELVAKPSLLL	FLDEPTSGLD	SQSAWAIVKL
(PRED)	catr_1_05971	VGRLGNGLNV	EQRKKLSIGV	ELVAKPSLLL	FLDEPTSGLD	SQSAWAIVKL
(PRED)	deha_1_a03696g	VGKTGDGLNV	EQKKKLSIGV	ELVAKPSLLL	FLDEPTSGLD	SQSAWAVVKL
(PRED)	deha_2_5_e00720	VGKTGDGLNV	EQKKKLSIGV	ELVAKPSLLL	FLDEPTSGLD	SQSAWAVVKL
(PRED)	scst_1_3_c02890	VGKSGSGLNV	EQRKKLSIGV	ELVAKPSLLL	FLDEPTSGLD	SQSAWAIIKL
(PRED)	mebi_1_8_h00300	IGSPGSGLNV	EQRKKLTIGV	ELVAKPSLLL	FLDEPTSGLD	SQSALSIVQL
(PRED)	lakl_1_h21010g	VGEAGFGLNV	EQRKKLSIGV	ELVAKPTLLL	FLDEPTSGLD	SQSSWAILQL
(PRED)	caar_1_13_m0142	AGCPGFGLNV	EQRKKLSIAT	ELVAKPSLLL	FLDEPTSGLD	SQSSWAIIQV
(PRED)	caar_1_14_n0143	AGKSGYGLNV	EQRKKLSIAT	ELVAKPSLLL	FLDEPTSGLD	SQSSWAIVQV
(PRED)	hapo_1_1_a07220	AGEVGYGLNV	EQRKKLSIAT	ELVAKPSLLL	FLDEPTSGLD	SQSSWAIVQV
(PRED)	ogpa_1_1_a01680	AGEIGYGLNV	EQRKKLSIAT	ELVAKPSLLL	FLDEPTSGLD	SQSSWAIVQV
(PRED)	piku_1_96_cr001	VGAAGYGLNV	EQRKKLSIAT	EMVAKPSLLL	FLDEPTSGLD	SQSSWAIVQV
(PRED)	pime_1_4_d03240	VGRTGYGLNV	EQRKKLSIAT	ELVAKPSLLL	FLDEPTSGLD	SQSSWAIVQV
(PRED)	pime_1_1_a12110	TGVTGYGLNV	EQRKKLSIAT	ELVAKPSLLL	FLDEPTSGLD	SQSSWAIIQV
(PRED)	piku_1_227_hs00	AGVPGYGLNV	EQRKKLSIAT	ELVAKPSLLL	FLDEPTSGLD	SQSSLAIIQV
(PRED)	pime_1_5_e05800	AGVPGYGLNV	EQRKKLSIAT	ELVAKPSLLL	FLDEPTSGLD	SQSSWAIVQV
(PRED)	pime_1_1_a07690	AGISGYGLNV	EQRKKLSIAT	ELVAKPSLLL	FLDEPTSGLD	SQSSWAIVQV
(PRED)	debr_2_5_e03380	AGKPGYGLNV	EQRKKLSIAT	ELVAKPSLLL	FLDEPTSGLD	SQSAWAIVQV
(PRED)	kopa_1_2_b10040	AGELGAGLNV	EQRKKLSIAT	ELVSKPDLLL	FLDEPTSGLD	SQSSWAIVKL
(PRED)	kopa_2_7_g00500	AGELGAGLNV	EQRKKLSIAT	ELVSKPDLLL	FLDEPTSGLD	SQSSWAIVKL
(PRED)	asru_1_13_m0119	VGDPGSGLNV	EQRKKLSIGV	ELVAKPSLLL	FLDEPTSGLD	SQSSWAIVKL
(PRED)	asru_1_15_o0045	VGKVGSGLNV	EQRKKLSIGV	ELVAKPSLLL	FLDEPTSGLD	FQSAWAIVKL
(PRED)	wian_1_1_a02920	VGQIGDGLNV	EQRKKLSIGV	ELVAKPSLLL	FLDEPSSGLD	SQSSWAVVQV
(PRED)	wian_1_1_a02930	VGQIGDGLNV	EQRKKLSIGV	ELVAKPSLLL	FLDEPSSGLD	SQSSWAVVQV

		1360	1370	1380	1390	1400
(PRED)	asac_1_6_f03560	LKRLAQSGQS	ILCTIHQPSA	TLFEQFDRLL	LLKKGGQTVY	FGDIGSESRI
(PRED)	ergo_1_abr125c	LKRLAQSGQA	ILCTIHQPSA	TLFEQFDRLL	LLQKGGQTVY	FGDIGEESRT
(PRED)	ercy_1_3604	LKKLAKAGQS	ILCTIHQPSA	TLFEQFDRLL	LLKKGGQTVY	FNDIGEHSKT
(PRED)	cagl_1_i04862g	LKKLAKAGQS	ILCTIHQPSA	TLFEEFDRLL	LLKKGGQTVY	FGDIGDNSKT
(PRED)	kaaf_1_c00830	LRKLAAAGQS	ILCTIHQPSA	TLFEQFDRLL	LLKKGGQTVY	FGDIGENSST
(PRED)	kana_1_k01350	LKKLAASGQS	ILCTIHQPSA	TLFEQFDRLL	LLKKGGQTVY	FGDIGENSNT
(PRED)	saar_1_2_b02590	LRKLSKAGQS	VLCTIHQPSA	TLFEEFDRLL	LLKKGGQTVY	FGDIGKNSAT
(PRED)	sace_1_ydr011w	LRKLSKAGQS	ILCTIHQPSA	TLFEEFDRLL	LLRKGGQTVY	FGDIGKNSAT
(PRED)	sace_16_1_a0238	LRKLSKAGQS	ILCTIHQPSA	TLFEEFDRLL	LLRKGGQTVY	FGDIGKNSAT
(PRED)	sace_45_1_a0242	LRKLSKAGQS	ILCTIHQPSA	TLFEEFDRLL	LLRKGGQTVY	FGDIGKNSAT
(PRED)	sace_48_1_a0238	LRKLSKAGQS	ILCTIHQPSA	TLFEEFDRLL	LLRKGGQTVY	FGDIGKNSAT
(PRED)	sace_60_4_d0244	LRKLSKAGQS	ILCTIHQPSA	TLFEEFDRLL	LLRKGGQTVY	FGDIGKNSAT
(PRED)	sace_52_1_a0240	LRKLSKAGQS	ILCTIHQPSA	TLFEEFDRLL	LLRKGGQTVY	FGDIGKNSAT
(PRED)	sace_46_1_a0240	LRKLSKAGQS	ILCTIHQPSA	TLFEEFDRLL	LLRKGGQTVY	FGDIGKNSAT
(PRED)	sace_25_1_a0240	LRKLSKAGQS	ILCTIHQPSA	TLFEEFDRLL	LLRKGGQTVY	FGDIGKNSAT

(PRED)	sace_24_1_2300	LRKLSKAGQS	ILCTIHQPSA	TLFEEFDRLL	LLRKGGQTVY	FGDIGKNSAT
(PRED)	sace_47_1_a0240	LRKLSKAGQS	ILCTIHQPSA	TLFEEFDRLL	LLRKGGQTVY	FGDIGKNSAT
(PRED)	sace_7_1_a02410	LRKLSKAGQS	ILCTIHQPSA	TLFEEFDRLL	LLRKGGQTVY	FGDIGKNSAT
(PRED)	sace_59_110_df0	LRKLSKAGQS	ILCTIHQPSA	TLFEEFDRLL	LLRKGGQTVY	FGDIGKNSAT
(PRED)	sace_56_1_a0202	LRKLSKAGQS	ILCTIHQPSA	TLFEEFDRLL	LLRKGGQTVY	FGDIGKNSAT
(PRED)	sace_40_1_a0239	LRKLSKAGQS	ILCTIHQPSA	TLFEEFDRLL	LLRKGGQTVY	FGDIGKNSAT
(PRED)	sace_15_1_a0242	LRKLSKAGQS	ILCTIHQPSA	TLFEEFDRLL	LLRKGGQTVY	FGDIGKNSAT
(PRED)	sace_37_1_a0243	LRKLSKAGQS	ILCTIHQPSA	TLFEEFDRLL	LLRKGGQTVY	FGDIGKNSAT
(PRED)	sace_9_1_a02440	LRKLSKAGQS	ILCTIHQPSA	TLFEEFDRLL	LLRKGGQTVY	FGDIGKNSAT
(PRED)	sace_22_1_2300	LRKLSKAGQS	ILCTIHQPSA	TLFEEFDRLL	LLRKGGQTVY	FGDIGKNSAT
(PRED)	sace_29_1_2290	LRKLSKAGQS	ILCTIHQPSA	TLFEEFDRLL	LLRKGGQTVY	FGDIGKNSAT
(PRED)	sace_34_1_2320	LRKLSKAGQS	ILCTIHQPSA	TLFEEFDRLL	LLRKGGQTVY	FGDIGKNSAT
(PRED)	sace_58_25_y007	LRKLSKAGQS	ILCTIHQPSA	TLFEEFDRLL	LLRKGGQTVY	FGDIGKNSAT
(PRED)	sace_23_1_2290	LRKLSKAGQS	ILCTIHQPSA	TLFEEFDRLL	LLRKGGQTVY	FGDIGKNSAT
(PRED)	sace_6_120_dp00	LRKLSKAGQS	ILCTIHQPSA	TLFEEFDRLL	LLRKGGQTVY	FGDIGKNSAT
(PRED)	sace_57_1_a0241	LRKLSKAGQS	ILCTIHQPSA	TLFEEFDRLL	LLRKGGQTVY	FGDIGKNSAT
(PRED)	sace_17_1_a0241	LRKLSKAGQS	ILCTIHQPSA	TLFEEFDRLL	LLRKGGQTVY	FGDIGKNSAT
(PRED)	sace_21_1_2310	LRKLSKAGQS	ILCTIHQPSA	TLFEEFDRLL	LLRKGGQTVY	FGDIGKNSAT
(PRED)	sace_49_1_a0246	LRKLSKAGQS	ILCTIHQPSA	TLFEEFDRLL	LLRKGGQTVY	FGDIGKNSAT
(PRED)	sace_8_2_b02430	LRKLSKAGQS	ILCTIHQPSA	TLFEEFDRLL	LLRKGGQTVY	FGDIGKNSAT
(PRED)	sace_31_1_2300	LRKLSKAGQS	ILCTIHQPSA	TLFEEFDRLL	LLRKGGQTVY	FGDIGKNSAT
(PRED)	sace_50_1_a0241	LRKLSKAGQS	ILCTIHQPSA	TLFEEFDRLL	LLRKGGQTVY	FGDIGKNSAT
(PRED)	sace_4_1_a02360	LRKLSKAGQS	ILCTIHQPSA	TLFEEFDRLL	LLRKGGQTVY	FGDIGKNSAT
(PRED)	sace_2_1_a02390	LRKLSKAGQS	ILCTIHQPSA	TLFEEFDRLL	LLRKGGQTVY	FGDIGKNSAT
(PRED)	sace_5_33_ag005	LRKLSKAGQS	ILCTIHQPSA	TLFEEFDRLL	LLRKGGQTVY	FGDIGKNSAT
(PRED)	sapa_11_1_a0247	LKRLSKAGQS	ILCTIHQPSA	TLFEEFDRLL	LLRKGGQTVY	FGDIGKNSAT
(PRED)	sapa_25_1_a0246	LKRLSKAGQS	ILCTIHQPSA	TLFEEFDRLL	LLRKGGQTVY	FGDIGKNSAT
(PRED)	sapa_4_1_a02470	LKRLSKAGQS	ILCTIHQPSA	TLFEEFDRLL	LLRKGGQTVY	FGDIGKNSAT
(PRED)	sapa_5_1_2350	LKRLSKAGQS	ILCTIHQPSA	TLFEEFDRLL	LLRKGGQTVY	FGDIGKNSAT
(PRED)	sapa_9_1_2360	LKRLSKAGQS	ILCTIHQPSA	TLFEEFDRLL	LLRKGGQTVY	FGDIGKNSAT
(PRED)	sapa_14_1_a0244	LKRLSKAGQS	ILCTIHQPSA	TLFEEFDRLL	LLRKGGQTVY	FGDIGKNSAT
(PRED)	sapa_8_1_2350	LKRLSKAGQS	ILCTIHQPSA	TLFEEFDRLL	LLRKGGQTVY	FGDIGKNSAT
(PRED)	sapa_17_1_2380	LKKLSKAGQS	ILCTIHQPSA	TLFEEFDRLL	LLRKGGQTVY	FGDIGKNSAT
(PRED)	sapa_7_1_2370	LKKLSKAGQS	ILCTIHQPSA	TLFEEFDRLL	LLRKGGQTVY	FGDIGKNSAT
(PRED)	sapa_2_1_a02460	LKKLSKAGQS	ILCTIHQPSA	TLFEEFDRLL	LLRKGGQTVY	FGDIGKNSAT
(PRED)	sapa_23_1_a0248	LKKLSKAGQS	ILCTIHQPSA	TLFEEFDRLL	LLRKGGQTVY	FGDIGKNSAT
(PRED)	sapa_3_1_a02470	LKKLSKAGQS	ILCTIHQPSA	TLFEEFDRLL	LLRKGGQTVY	FGDIGKNSAT
(PRED)	sapa_18_1_2390	LKKLSKAGQS	ILCTIHQPSA	TLFEEFDRLL	LLRKGGQTVY	FGDIGKSSAT
(PRED)	sami_1_4_244	LRKLSKAGQS	ILCTIHQPSA	TLFEEFDRLL	LLRKGGQTVY	FGDIGKNSAT
(PRED)	saku_1_4_262	LRKLSKAGQS	ILCTIHQPSA	TLFEEFDRLL	LLKKGGQTVY	FGDIGKNSAA
(PRED)	saba_1_58_bf002	LRKLSKAGQS	ILCTIHQPSA	TLFEEFDRLL	LLRKGGQTVY	FGDIGKNSAT
(PRED)	saeu_1_4_d02400	LRKLSKAGQS	ILCTIHQPSA	TLFEEFDRLL	LLKKGGQTVY	FGDIGKNSST
(PRED)	naca_1_e01640	LRKLAQAGQS	ILCTIHQPSA	TLFEQFDRLL	LLKKGGQTVY	FGDVGKNSRT
(PRED)	nada_1_g01850	LRRLADAGQS	ILCTIHQPSA	TLFEQFDRLL	LLKKGGQTVY	FGDIGHNSET
(PRED)	naca_1_e01630	LRKLAQAGQS	ILCTIHQPSA	TLFEQFDRLL	LLKKGGQTVY	FGDIGENSEI
(PRED)	nada_1_g01840	LKRLTHAGQS	ILCTVHQPSA	TLFEQFDRLL	LLRKGGQTVY	FGDIGENSEI
(PRED)	kaaf_1_c00820	LKKLAHSGQS	ILCTIHQPSA	TLFEQFDRLL	LLKKGGQTVY	FGNIGENSSL
(PRED)	teph_1_m00640	LKKLAASGQS	ILCTIHQPSA	TLFEQFDRLL	LLKKGGQTVY	FGDIGENSKD
(PRED)	vapo_1_1036_28	LRKLAEAGQS	ILCTIHQPSA	TLFEQFDRLL	LLKKGGQTVY	FGDIGQNSTT
(PRED)	tebl_1_i01760	LRRLANAGQA	ILCTIHQPSA	TLFEQFDRLL	LLKKGGQTVY	FGDIGKNSRT
(PRED)	tode_1_d04040	LRKLAKAGQS	ILCTIHQPSA	TLFEQFDRLL	LLKKGGQTVY	FGDVGKNSSI
(PRED)	naca_1_e01650	LRKLAQAGQS	ILCTIHQPSA	TLFEQFDRLL	LLRKGGQTVY	FGDIGKNSRT
(PRED)	tebl_1_g02820	LKKLANAGQS	ILCTIHQPSA	TLFEQFDRLL	LLKKGGQTVY	FGDIGENSDI
(PRED)	lakl_1_c11616g	LKKLAQAGQS	ILCTIHQPSA	TLFEQFDRLL	LLKKGGQTVY	FGPIGEHSRV
(PRED)	saar_1_8_h03780	LKRLTQAGQS	ILCTIHQPSA	TLFEQFDRLL	LLGKGGQTVY	FGEIGKDSTS
(PRED)	sace_14_7_g0015	LKRLALAGQS	ILCTIHQPSA	TLFEQFDRLL	LLGKGGQTIY	FGEIGKNSSS
(PRED)	sace_15_7_g0387	LKRLALAGQS	ILCTIHQPSA	TLFEQFDRLL	LLGKGGQTIY	FGEIGKNSSS
(PRED)	sace_24_8_3780	LKRLALAGQS	ILCTIHQPSA	TLFEQFDRLL	LLGKGGQTIY	FGEIGKNSSS
(PRED)	sace_40_8_h0383	LKRLALAGQS	ILCTIHQPSA	TLFEQFDRLL	LLGKGGQTIY	FGEIGKNSSS
(PRED)	sace_6_169_fm00	LKRLALAGQS	ILCTIHQPSA	TLFEQFDRLL	LLGKGGQTIY	FGEIGKNSSS
(PRED)	sace_19_7_3840	LKRLALAGQS	ILCTIHQPSA	TLFEQFDRLL	LLGKGGQTIY	FGEIGKNSSS
(PRED)	sace_32_7_3770	LKRLALAGQS	ILCTIHQPSA	TLFEQFDRLL	LLGKGGQTIY	FGEIGKNSSS
(PRED)	sace_56_17_q011	LKRLALAGQS	ILCTIHQPSA	TLFEQFDRLL	LLGKGGQTIY	FGEIGKNSSS
(PRED)	sace_5_78_bz001	LKRLALAGQS	ILCTIHQPSA	TLFEQFDRLL	LLGKGGQTIY	FGEIGKNSSS
(PRED)	sace_2_8_h03860	LKRLALAGQS	ILCTIHQPSA	TLFEQFDRLL	LLGKGGQTIY	FGEIGKNSSS
(PRED)	sace_53_29_ac00	LKRLALAGQS	ILCTIHQPSA	TLFEQFDRLL	LLGKGGQTIY	FGEIGKNSSS
(PRED)	sace_17_7_g0393	LKRLALAGQS	ILCTIHQPSA	TLFEQFDRLL	LLGKGGQTIY	FGEIGKNSSS
(PRED)	sace_25_7_g0388	LKRLALAGQS	ILCTIHQPSA	TLFEQFDRLL	LLGKGGQTIY	FGEIGKNSSS
(PRED)	sace_37_7_g0385	LKRLALAGQS	ILCTIHQPSA	TLFEQFDRLL	LLGKGGQTIY	FGEIGKNSSS
(PRED)	sace_9_7_g00180	LKRLALAGQS	ILCTIHQPSA	TLFEQFDRLL	LLGKGGQTIY	FGEIGKNSSS
(PRED)	sace_60_6_f0335	LKRLALAGQS	ILCTIHQPSA	TLFEQFDRLL	LLGKGGQTIY	FGEIGKNSSS
(PRED)	sace_59_336_lx0	LKRLALAGQS	ILCTIHQPSA	TLFEQFDRLL	LLGKGGQTIY	FGEIGKNSSS
(PRED)	sace_31_7_3780	LKRLALAGQS	ILCTIHQPSA	TLFEQFDRLL	LLGKGGQTIY	FGEIGKNSSS
(PRED)	sace_34_8_3770	LKRLALAGQS	ILCTIHQPSA	TLFEQFDRLL	LLGKGGQTIY	FGEIGKNSSS
(PRED)	sace_58_71_bs00	LKRLALAGQS	ILCTIHQPSA	TLFEQFDRLL	LLGKGGQTIY	FGEIGKNSSS

(PRED)	sace_7_7_g03880	LKRLALAGQS	ILCTIHQPSA	TLFEQFDRLL	LLGKGGQTIY	FGEIGKNSSS
(PRED)	sace_35_7_3840	LKRLALAGQS	ILCTIHQPSA	TLFEQFDRLL	LLGKGGQTIY	FGEIGKNSSS
(PRED)	sace_43_7_g0387	LKRLALAGQS	ILCTIHQPSA	TLFEQFDRLL	LLGKGGQTIY	FGEIGKNSSS
(PRED)	sace_57_8_h0390	LKRLALAGQS	ILCTIHQPSA	TLFEQFDRLL	LLGKGGQTIY	FGEIGKNSSS
(PRED)	sace_45_7_g0389	LKRLALAGQS	ILCTIHQPSA	TLFEQFDRLL	LLGKGGQTIY	FGEIGKNSSS
(PRED)	sace_46_8_h0391	LKRLALAGQS	ILCTIHQPSA	TLFEQFDRLL	LLGKGGQTIY	FGEIGKNSSS
(PRED)	sace_23_7_3860	LKRLALAGQS	ILCTIHQPSA	TLFEQFDRLL	LLGKGGQTIY	FGEIGKNSSS
(PRED)	sace_21_7_3790	LKRLALAGQS	ILCTIHQPSA	TLFEQFDRLL	LLGKGGQTIY	FGEIGKNSSS
(PRED)	sace_8_73_bu001	LKRLALAGQS	ILCTIHQPSA	TLFEQFDRLL	LLGKGGQTIY	FGEIGKNSSS
(PRED)	sapa_1_8_h03820	LKRLAQAGQS	ILCTIHQPSA	TLFEQFDRLL	LLGKGGQTVY	FGEIGKNSSS
(PRED)	sapa_21_8_h0387	LKRLAQAGQS	ILCTIHQPSA	TLFEQFDRLL	LLGKGGQTVY	FGEIGKNSSS
(PRED)	sapa_20_8_h0386	LKRLAQAGQS	ILCTIHQPSA	TLFEQFDRLL	LLGKGGQTVY	FGEIGKNSSS
(PRED)	sapa_22_8_h0390	LKRLAQAGQS	ILCTIHQPSA	TLFEQFDRLL	LLGKGGQTVY	FGEIGKNSSS
(PRED)	sapa_25_8_h0387	LKRLAQAGQS	ILCTIHQPSA	TLFEQFDRLL	LLGKGGQTVY	FGEIGKNSSS
(PRED)	sapa_6_8_3750	LKRLAQAGQS	ILCTIHQPSA	TLFEQFDRLL	LLGKGGQTVY	FGEIGKNSSS
(PRED)	sapa_9_8_3720	LKRLAQAGQS	ILCTIHQPSA	TLFEQFDRLL	LLGKGGQTVY	FGEIGKNSSS
(PRED)	sapa_19_8_h0390	LKRLAQAGQS	ILCTIHQPSA	TLFEQFDRLL	LLGKGGQTVY	FGEIGKNSSS
(PRED)	sapa_24_8_h0385	LKRLAQAGQS	ILCTIHQPSA	TLFEQFDRLL	LLGKGGQTVY	FGEIGKNSSS
(PRED)	sapa_4_8_h03850	LKRLAQAGQS	ILCTIHQPSA	TLFEQFDRLL	LLGKGGQTVY	FGEIGKNSSS
(PRED)	sapa_10_8_3760	LKRLAQAGQS	ILCTIHQPSA	TLFEQFDRLL	LLGKGGQTVY	FGEIGKNSSS
(PRED)	sapa_13_8_h0382	LKRLAQAGQS	ILCTIHQPSA	TLFEQFDRLL	LLGKGGQTVY	FGEIGKNSSS
(PRED)	sapa_8_8_3750	LKRLAQAGQS	ILCTIHQPSA	TLFEQFDRLL	LLGKGGQTVY	FGEIGKNSSS
(PRED)	sapa_11_8_h0383	LKRLAQAGQS	ILCTIHQPSA	TLFEQFDRLL	LLGKGGQTVY	FGEIGKNSSS
(PRED)	sapa_5_8_3700	LKRLAQAGQS	ILCTIHQPSA	TLFEQFDRLL	LLGKGGQTVY	FGEIGKNSSS
(PRED)	sapa_16_8_h0389	LKRLAQAGQS	ILCTIHQPSA	TLFEQFDRLL	LLGKGGQTVY	FGEIGKNSSS
(PRED)	sapa_17_8_3730	LKRLAQAGQS	ILCTIHQPSA	TLFEQFDRLL	LLGKGGQTVY	FGEIGKNSSS
(PRED)	sapa_2_8_h03860	LKRLAQAGQS	ILCTIHQPSA	TLFEQFDRLL	LLGKGGQTVY	FGEIGKNSSS
(PRED)	sapa_7_8_3740	LKRLAQAGQS	ILCTIHQPSA	TLFEQFDRLL	LLGKGGQTVY	FGEIGKNSSS
(PRED)	sapa_23_8_h0385	LKRLAQAGQS	ILCTIHQPSA	TLFEQFDRLL	LLGKGGQTVY	FGEIGKNSSS
(PRED)	sapa_3_8_h03890	LKRLAQAGQS	ILCTIHQPSA	TLFEQFDRLL	LLGKGGQTVY	FGEIGKNSSS
(PRED)	sapa_18_8_3730	LKRLAQAGQS	ILCTIHQPSA	TLFEQFDRLL	LLGKGGQTVY	CGEIGKNSSS
(PRED)	sami_1_14_399	LKRLARSGQS	ILCTIHQPSA	TLFEQFDWLL	LLGKGGRTIY	FGGIGENSSS
(PRED)	sace_4_8_h03690	LKRLALAGQS	ILCTIHQPSA	TLFEQFDRLL	LLGKGGQTIY	FGEIGKNSSS
(PRED)	saku_1_14_404	LKRLTQAGQS	ILCTIHQPSA	TLFEQFERLL	LLGKGGQTVY	FGEIGKNSSS
(PRED)	sace_1_ynr070w	LKRLALAGQS	ILCTIHQPSA	TLFEQFDRLL	LLGKGGQTIY	FGEIGKNSSS
(PRED)	sace_49_8_h0383	LKRLALAGQS	ILCTIHQPSA	TLFEQFDRLL	LLGKGGQTIY	FGEIGKNSSS
(PRED)	saeu_1_2_b00130	LKRLALAGQS	ILCTIHQPSA	TLFEQFDRLL	LLRKGGQTVY	FGDIGKNSNV
(PRED)	sauv_1_7_3	LKRLALAGQS	ILCTIHQPSA	TLFEQFDRLL	LLRKGGQTVY	FGDIGKNSNV
(PRED)	sami_1_17_26	LKKLAQAGQS	ILCTIHQPSA	TLFEQFDRLL	LLRKGGQTVY	FGDVGKNSSS
(PRED)	zyba_1_02055_AN	LRKLARSGQC	ILCTIHQPSA	TLFERFDRLL	LLRKGGQTVY	FGDIGDNSST
(PRED)	zyba_1_07912	LRKLARSGQC	ILCTIHQPSA	TLFERFDRLL	LLRKGGQTVY	FGDIGDNSST
(PRED)	zyba_2_2_b00600	LRKLARSGQC	ILCTIHQPSA	TLFERFDRLL	LLRKGGQTVY	FGDIGDNSST
(PRED)	zyba_3_3_c03460	LRKLARSGQC	ILCTIHQPSA	TLFERFDRLL	LLRKGGQTVY	FGDIGDNSST
(PRED)	zyba_1_04634	LRKLARSGQC	ILCTIHQPSA	TLFERFDRLL	LLRKGGQTVY	FGDIGENSST
(PRED)	zyba_1_06675	LRKLARAGQC	ILCTIHQPSA	TLFERFDRLL	LLRKGGQTVY	FGGIGENSST
(PRED)	zyba_3_2_b02230	LRKLARAGQC	ILCTIHQPSA	TLFERFDRLL	LLRKGGQTVY	FGDIGENSST
(PRED)	zyba_2_1_a00860	LRKLARAGQC	ILCTIHQPSA	TLFERFDRLL	LLRKGGQTVY	FGDIGENSST
(PRED)	zyro_1_a04114g	LKRISRAGQS	ILCTIHQPSA	TLFEQFDRLL	LLKKGGQTVY	FGDIGENSSI
(PRED)	zyro_1_b14762g	LRRIAKAGQC	ILCTIHQPSA	TLFEQFDRLL	LLKKGGQTVY	FGDIGENSSI
(PRED)	zyba_2_14_n0149	LKKLAHAGQS	ILCTIHQPSS	TLFESFDRLL	LLRRGGQTVY	FGDIGENSSC
(PRED)	zyba_2_33_ag001	LKKLAHAGQS	ILCTIHQPSS	TLFESFDRLL	LLRRGGQTVY	FGDIGDNSSR
(PRED)	lath_1_a01914g	MRRLAEAGQS	ILCTIHQPSA	TLFEQFDRLL	LLKKGGQTVY	FGPIGKNSRT
(PRED)	lawa_1_23_5161	MRRLAEAGQS	ILCTIHQPSA	TLFEEFDRLL	LLKKGGQTVY	FGPIGKNSRM
(PRED)	klae_1_14_n0012	LRKLAAAGQS	ILCTIHQPSA	TLFEQFDRLL	MLRKGGQTVY	FGDIGKHSST
(PRED)	klla_1_d03432g	LKKLAGAGQS	ILCTIHQPSA	TLFEEFDRLL	LLRKGGQTVY	FGDIGEHSST
(PRED)	klma_1_1_a01880	LKKLAMAGQS	ILCTIHQPSA	TLFEEFDRLL	LLRKGGQTVY	FGDIGEHSST
(PRED)	klwi_1_33_ag001	LKRLAQSGQS	ILCTIHQPSA	TLFEEFDRLL	LLRKGGQTVY	FGDIGSHSTT
(PRED)	teph_1_a04220	LKRLSQSGQS	ILCTIHQPSA	TLFEVFDRLL	LLQKGGETVY	FGDIGKNSNT
(PRED)	vapo_1_1037_47	LKRLSESGQS	ILCTIHQPSA	TLFEQFDRLL	LLKKGGKTVY	FGDIGNNSDT
(PRED)	pata_1_2_b05590	LRSLANAGQA	ILCTIHQPSA	TLFEQFDRLL	LLRKGGQTVY	FGDIGKNSET
(PRED)	wian_1_3_c04380	LRRLAQAGQS	ILCTIHQPSA	TLFEAFDRLL	LLEKGGKTVY	FGDIGHNSRV
(PRED)	wian_1_3_c04390	LRQLAQAGQS	ILCTIHQPSA	TLFEAFDRLL	LLKKGGQTVY	FGNIGENSRI
(PRED)	wian_1_7_g01010	LRKLAQAGQS	ILCTIHQPSA	TLFEAFDRLL	LLRKGGQTVY	FGNIGKNSST
(PRED)	bain_1_1_a00100	MRDLANAGQA	ILCTIHQPSA	TLFEEFDRLL	LLKKGGQTVY	FGDIGKHSQV
(PRED)	bain_1_17_q0038	MRDLAYAGQA	ILCTIHQPSA	TLFEEFDRLL	LLKKGGQTVY	FGDIGKHSHA
(PRED)	bain_1_8_h00410	IRNLAHAGQA	ILCTIHQPSA	TLFEEFDKLL	LLRKGGQTVY	FGDIGLHSET
(PRED)	caal_1_19_5759	LRDLANAGQS	ILCTIHQPSA	TLFEEFDRLL	LLKKGGIVTY	FGDIGPRSRT
(PRED)	caal_11_25_y002	LRDLANAGQS	ILCTIHQPSA	TLFEEFDRLL	LLKKGGIVTY	FGDIGPRSRT
(PRED)	caal_4_4_d03320	LRDLANAGQS	ILCTIHQPSA	TLFEEFDRLL	LLKKGGIVTY	FGDIGPRSRT
(PRED)	caal_12_26_z005	LRDLANAGQS	ILCTIHQPSA	TLFEEFDRLL	LLKKGGIVTY	FGDIGPRSRT
(PRED)	caal_5_30_ad005	LRDLANAGQS	ILCTIHQPSA	TLFEEFDRLL	LLKKGGIVTY	FGDIGPRSRT
(PRED)	caal_8_3_c03320	LRDLANAGQS	ILCTIHQPSA	TLFEEFDRLL	LLKKGGIVTY	FGDIGPRSRT
(PRED)	caal_6_4_d03280	LRDLANAGQS	ILCTIHQPSA	TLFEEFDRLL	LLKKGGIVTY	FGDIGPRSRT
(PRED)	caal_10_3_c0334	LRDLANAGQS	ILCTIHQPSA	TLFEEFDRLL	LLKKGGIVTY	FGDIGPRSRT
(PRED)	caal_3_29_ac005	LRDLANAGQS	ILCTIHQPSA	TLFEEFDRLL	LLKKGGIVTY	FGDIGPRSRT

(PRED)	caal_2_04989	LRDLANAGQS	ILCTIHQPSA	TLFEEFDRLL	LLKKGGIVTY	FGDIGPRSRT
(PRED)	cadu_1_64350	LRDLANAGQS	ILCTIHQPSA	TLFEEFDRLL	LLKKGGIVTY	FGDIGPRSRT
(PRED)	caor_1_h02090	LRDLANAGQS	ILCTIHQPSA	TLFEEFDRLL	LLRKGGQTVY	FGEIGDKSKT
(PRED)	capa_1_600750	LRDLANAGQS	ILCTIHQPSA	TLFEEFDRLL	LLRKGGQTVY	FGEIGDKSKT
(PRED)	loel_1_04930	LRELANAGQS	ILCTIHQPSA	TLFEEFDRLL	LLRKGGQTVY	FGDIGERSRT
(PRED)	spar_1_5_e03260	LRDLANAGQS	ILCTIHQPSA	TLFEEFDRLL	LLRKGGQTVY	FGDIGPRSRT
(PRED)	sppa_1_7_g03160	LRDLANAGQS	ILCTIHQPSA	TLFEEFDRLL	LLRKGGQTVY	FGDIGPRSRT
(PRED)	catr_1_01205	LRSLANSGQA	ILCTIHQPSA	TLFEEFDRLL	LLRKGGIVTY	FGDIGPRSSV
(PRED)	catr_1_05498	LRTLANSGQS	ILCTIHQPSA	TLFEEFDRLL	LLKKGGIVTY	FGDIGPRSHI
(PRED)	catr_1_05971	LRALANSGQS	ILCTIHQPSA	TLFEEFDRLL	LLKKGGIVTY	FGDIGDRSSV
(PRED)	deha_1_a03696g	LRELSNAGQS	ILCTIHQPSA	TLFEEFDRLL	LLKKGGQTVY	FGDIGDHSNA
(PRED)	deha_2_5_e00720	LRELSNAGQS	ILCTIHQPSA	TLFEEFDRLL	LLKKGGQTVY	FGDIGEHSNV
(PRED)	scst_1_3_c02890	LRDLANAGQS	ILCTIHQPSA	TLFEEFDRLL	LLRKGGQTVY	FGDIGDQSRV
(PRED)	mebi_1_8_h00300	LRTLANAGQA	ILCTIHQPSS	TLFEEFDRLL	LLRKGGQTVY	FGDIGEHSKD
(PRED)	lakl_1_h21010g	LRRLAEAGQA	ILCTIHQPSA	TLFESFDRLL	LLKDGGSVVY	WGNIGDHSKD
(PRED)	caar_1_13_m0142	LRSLAEAGQA	ILCTIHQPSA	TLFEQFDKLL	LLKTGGQTVY	FGDIGDNSRI
(PRED)	caar_1_14_n0143	LKSLAKAGQA	ILCTIHQPSA	TLFEEFDKLL	LLRRGGQTVY	FGDIGKNSAK
(PRED)	hapo_1_1_a07220	LRSLAAAGQA	ILCTIHQPSA	TLFEQFDRLL	LLKRGGQTVY	FGDIGPNSRT
(PRED)	ogpa_1_1_a01680	LRSLAAAGQA	ILCTIHQPSA	TLFEQFDRLL	LLKRGGQTVY	FGDIGPNSRI
(PRED)	piku_1_96_cr001	LKDLARAGQA	ILCTIHQPSA	TLFEEFDRLL	LLKTGGQTVY	FGEIGENSST
(PRED)	pime_1_4_d03240	LKELAKAGQA	ILCTIHQPSA	TLFEEFDKLL	LLRRGGQTVY	FGDIGENSST
(PRED)	pime_1_1_a12110	LRELAKAGQA	ILCTIHQPSA	TLIEQFDKLL	LLKRGGQTVY	FGDIGPNSET
(PRED)	piku_1_227_hs00	MRKLANAGQA	ILCTIHQPSA	VLFEQFDRLL	LLRKGGQTVY	FGDIGANSEV
(PRED)	pime_1_5_e05800	LKELANAGQA	ILCTIHQPSA	VLFEQFDRLL	LLKRGGQTVY	FGDIGQNSRV
(PRED)	pime_1_1_a07690	LKDLAKAGQA	ILCTIHQPSA	TLFEQFDRLL	LLKKGGQTVY	FGNIGPDSRT
(PRED)	debr_2_5_e03380	LRKLALAGQA	ILCTIHQPSA	TLFEQFDRLL	LLKKGGQTVY	FGPVGRNSHS
(PRED)	kopa_1_2_b10040	LRQLADAGQA	ILCTIHQPSA	TLFEQFDRLL	LLRKGGQTVY	FGDIGENSSV
(PRED)	kopa_2_7_g00500	LRQLADAGQA	ILCTIHQPSA	TLFEQFDRLL	LLRKGGQTVY	FGDIGENSSV
(PRED)	asru_1_13_m0119	MRQLAHAGQS	ILCTIHQPSA	TLFEQFDRLL	LLRKGGQTVY	FGDIGKSSRT
(PRED)	asru_1_15_o0045	LRRLADAGQC	IICTIHQPSA	TLFEQFDRLL	LLKKGGQTVY	FGDIGSSSNV
(PRED)	wian_1_1_a02920	LRHLAEAGQA	ILCTIHQPSA	TLFEQFDRLL	LLKKGGQTVY	FGDIGERSRT
(PRED)	wian_1_1_a02930	LRHLAEAGQA	ILCTIHQPSA	TLFEQFDRLL	LLKKGGQTVY	FGDIGERSRT

.........1410.........1420.........1430.........1440.........1450

(PRED)	asac_1_6_f03560	LLDYFERNGA	RPCAPSENPA	EYILEAIGAG	ATASTTEDWA	ALWRNSPECA
(PRED)	ergo_1_abr125c	LLDYFQRNGA	RPCTPSENPA	EYILESIGAG	ATATTTEDWA	ELWRNSPECA
(PRED)	ercy_1_3604	ILDYFERNGA	RKCSPSENPA	EYILEVIGAG	ATATTSEDWA	KIWRNSPEFA
(PRED)	cagl_1_i04862g	LLSYFERNGA	RKCSPSENPA	EYILEAIGAG	ATASVTEDWH	QIWKNSDEFI
(PRED)	kaaf_1_c00830	LLGYFERNGA	RKCSKAENPA	EYILEAIGAG	ATASTDADWH	EIWKTSSEFD
(PRED)	kana_1_k01350	VLSYFERNGA	RKCSAAENPA	EYILEAIGAG	ATASVKEDWY	DTWCSSTEFV
(PRED)	saar_1_2_b02590	ILNYFESNGG	RKCHSSENPA	EYILEAIGAG	ATASIKEDWH	EKWLNSSEFE
(PRED)	sace_1_ydr011w	ILNYFERNGA	RKCDSSENPA	EYILEAIGAG	ATASVKEDWH	EKWLNSVEFE
(PRED)	sace_16_1_a0238	ILNYFERNGA	RKCDSSENPA	EYILEAIGAG	ATASVKEDWH	EKWLNSVEFE
(PRED)	sace_45_1_a0242	ILNYFERNGA	RKCDSSENPA	EYILEAIGAG	ATASVKEDWH	EKWLNSVEFE
(PRED)	sace_48_1_a0238	ILNYFERNGA	RKCDSSENPA	EYILEAIGAG	ATASVKEDWH	EKWLNSVEFE
(PRED)	sace_60_4_d0244	ILNYFERNGA	RKCDSSENPA	EYILEAIGAG	ATASVKEDWH	EKWLNSVEFE
(PRED)	sace_52_1_a0240	ILNYFERNGA	RKCDSSENPA	EYILEAIGAG	ATASVKEDWH	EKWLNSVEFE
(PRED)	sace_46_1_a0240	ILNYFERNGA	RKCDSSENPA	EYILEAIGAG	ATASVKEDWH	EKWLNSVEFE
(PRED)	sace_25_1_a0240	ILNYFERNGA	RKCDSSENPA	EYILEAIGAG	ATASVKEDWH	EKWLNSVEFE
(PRED)	sace_24_1_2300	ILNYFERNGA	RKCDSSENPA	EYILEAIGAG	ATASVKEDWH	EKWLNSVEFE
(PRED)	sace_47_1_a0240	ILNYFERNGA	RKCDSSENPA	EYILEAIGAG	ATASVKEDWH	EKWLNSVEFE
(PRED)	sace_7_1_a02410	ILNYFERNGA	RKCDSSENPA	EYILEAIGAG	ATASVKEDWH	EKWLNSVEFE
(PRED)	sace_59_110_df0	ILNYFERNGA	RKCDSSENPA	EYILEAIGAG	ATASVKEDWH	EKWLNSVEFE
(PRED)	sace_56_1_a0202	ILNYFERNGA	RKCDSSENPA	EYILEAIGAG	ATASVKEDWH	EKWLNSVEFE
(PRED)	sace_40_1_a0239	ILNYFERNGA	RKCDSSENPA	EYILEAIGAG	ATASVKEDWH	EKWLNSVEFE
(PRED)	sace_15_1_a0242	ILNYFERNGA	RKCDSSENPA	EYILEAIGAG	ATASVKEDWH	EKWLNSVEFG
(PRED)	sace_37_1_a0243	ILNYFERNGA	RKCDSSENPA	EYILEAIGAG	ATASVKEDWH	EKWLNSVEFG
(PRED)	sace_9_1_a02440	ILNYFERNGA	RKCDSSENPA	EYILEAIGAG	ATASVKEDWH	EKWLNSVEFE
(PRED)	sace_22_1_2300	ILNYFERNGA	RKCDSSENPA	EYILEAIGAG	ATASVKEDWH	EKWLNSVEFG
(PRED)	sace_29_1_2290	ILNYFERNGA	RKCDSSENPA	EYILEAIGAG	ATASVKEDWH	EKWLNSVEFG
(PRED)	sace_34_1_2320	ILNYFERNGA	RKCDSSENPA	EYILEAIGAG	ATASVKEDWH	EKWLNSVEFG
(PRED)	sace_58_25_y007	ILNYFERNGA	RKCDSSENPA	EYILEAIGAG	ATASVKEDWH	EKWLNSVEFG
(PRED)	sace_23_1_2290	ILNYFERNGA	RKCDSSENPA	EYILEAIGAG	ATASVKEDWH	EKWLNSVEFG
(PRED)	sace_6_120_dp00	ILNYFERNGA	RKCDSSENPA	EYILEAIGAG	ATASVKEDWH	EKWLNSVEFG
(PRED)	sace_57_1_a0241	ILNYFERNGA	RKCDSSENPA	EYILEAIGAG	ATASVKEDWH	EKWLNSVEFE
(PRED)	sace_17_1_a0241	ILNYFERNGA	RKCDSSENPA	EYILEAIGAG	ATASVKEDWH	EKWLNSVEFG
(PRED)	sace_21_1_2310	ILNYFERNGA	RKCDSSENPA	EYILEAIGAG	ATASVKEDWH	EKWLNSVEFG
(PRED)	sace_49_1_a0246	ILNYFERNGA	RKCDSSENPA	EYILEAIGAG	ATASVKEDWH	EKWLNSVEFE
(PRED)	sace_8_2_b02430	ILNYFERNGA	RKCDSSENPA	EYILEAIGAG	ATASVKEDWH	EKWLNSVEFE
(PRED)	sace_31_1_2300	ILNYFERNGA	RKCDSSENPA	EYILEAIGAG	ATASVKEDWH	EKWLNSVEFG
(PRED)	sace_50_1_a0241	ILNYFERNGA	RKCDSSENPA	EYILEAIGAG	ATASVKEDWH	EKWLNSVEFE
(PRED)	sace_4_1_a02360	ILNYFERNGA	RKCDSSENPA	EYILEAIGAG	ATASVKEDWH	EKWLNSVEFE
(PRED)	sace_2_1_a02390	ILNYFERNGA	RKCDSSENPA	EYILEAIGAG	ATASVKEDWH	EKWLNSVEFE
(PRED)	sace_5_33_ag005	ILNYFEXNGA	RKCDSSENPA	EYILEAIGAG	ATASVKEDWH	EKWLNSVEFE
(PRED)	sapa_11_1_a0247	ILDYFERNGA	RKCDSSENPA	EYILEAIGAG	ATASVKEDWH	EKWLNSAEYN

(PRED)	sapa_25_1_a0246	ILDYFERNGA	RKCDSSENPA	EYILEAIGAG	ATASVKEDWH	EKWLNSAEYN
(PRED)	sapa_4_1_a02470	ILDYFERNGA	RKCDSSENPA	EYILEAIGAG	ATASVKEDWH	EKWLNSAEYN
(PRED)	sapa_5_1_2350	ILDYFERNGA	RKCDSSENPA	EYILEAIGAG	ATASVKEDWH	EKWLNSAEYN
(PRED)	sapa_9_1_2360	ILDYFERNGA	RKCDSSENPA	EYILEAIGAG	ATASVKEDWH	EKWLNSAEYN
(PRED)	sapa_14_1_a0244	ILDYFEKNGA	RKCDSSENPA	EYILEAIGAG	ATASVKEDWH	EKWLNSAEYN
(PRED)	sapa_8_1_2350	ILDYFEKNGA	RKCDSSENPA	EYILEAIGAG	ATASVKEDWH	EKWLNSAEYN
(PRED)	sapa_17_1_2380	ILDYFERNGA	RKCDSSENPA	EYILEAIGAG	ATASVKEDWH	EKWLNSSEYN
(PRED)	sapa_7_1_2370	ILDYFERNGA	RKCDSSENPA	EYILEAIGAG	ATASVKEDWH	EKWLNSSEYN
(PRED)	sapa_2_1_a02460	ILDYFERNGA	RKCDSSENPA	EYILEAIGAG	ATASVKEDWH	EKWLNSSEYN
(PRED)	sapa_23_1_a0248	ILDYFERNGA	RKCDSSENPA	EYILEAIGAG	ATASVKEDWH	EKWLNSSEYN
(PRED)	sapa_3_1_a02470	ILDYFERNGA	RKCDSSENPA	EYILEAIGAG	ATASVKEDWH	EKWLNSSEYN
(PRED)	sapa_18_1_2390	ILDYFERNGA	RKCDSSENPA	EYILEAIGAG	ATASVKEDWH	EKWLNSSEYN
(PRED)	sami_1_4_244	ILQYFERNGA	RKCDSSENPA	EYILEAIGAG	ATASVKENWH	EKWLNSSEFQ
(PRED)	saku_1_4_262	ILGYFEQNGA	RKCDHDENPA	EYILEAIGAG	ATASVKEDWH	EKWLNSPEYK
(PRED)	saba_1_58_bf002	LLEYFERNGA	RKCDSSENPA	EYILEAIGAG	ATASVKEDWH	EKWLNSPECI
(PRED)	saeu_1_4_d02400	LLEYFERNGA	RKCDSSENPA	EYILEAIGAG	ATASVKEDWH	EKWLNSSEYN
(PRED)	naca_1_e01640	LLDYFEGNGA	RKCDRKENPA	EYILEAIGAG	ATASVKEDWH	EIWKNSKEYM
(PRED)	nada_1_g01850	LLRYFERNGA	RKCTSKENPA	EYILEAIGAG	ATASVKENWH	DIWIRSPEYQ
(PRED)	naca_1_e01630	LLKYFEKSGA	RKCDRKENPA	EYILEAIGAG	ATASVKEDWH	QIWLDSAEHH
(PRED)	nada_1_g01840	LLSYFERNSA	RKCSPKENPA	EYILEIIGAG	ATASIKEDWH	SIWKNSSEYK
(PRED)	kaaf_1_c00820	LLDYFERNGA	RQCSSSENPA	EYILEAIGAG	ATATVKEDWN	EIWRNSPESR
(PRED)	teph_1_m00640	LLTYFEDHGA	RKCERKENPA	EYILEAIGAG	ATASVKDDWH	EIWKNSSTYK
(PRED)	vapo_1_1036_28	LLEYFERNGA	RKCDAKENPA	EYILEAIGAG	ATASVNEDWH	QIWKNSPEHT
(PRED)	tebl_1_i01760	LLDYFEGNGA	RICDFSENPA	EYILESIGAG	ATASVKEDWH	EIWLNSPEYT
(PRED)	tode_1_d04040	LLEYFERNGA	RKCEKSENPA	EYILEAIGAG	ATASVEEDWH	QIWTKSPEHK
(PRED)	naca_1_e01650	LLDYFEGNSA	RKCDQRENPA	EYILEAIGAG	ATASSESNWH	DLWINSPEYL
(PRED)	tebl_1_g02820	LLKYFERNGA	RKCTSSENPA	EYILEAIGAG	ATASVQEDWH	EVWKNSHEFA
(PRED)	lakl_1_c11616g	LLDYFERNGA	RQCEDSENPA	EYILESIGAG	ATASVKEDWH	LKWKASPEFQ
(PRED)	saar_1_8_h03780	VVGYFERNGA	RKCQHNENPA	EYILEVIGAG	ATASIHEDWY	DIWKESPEIV
(PRED)	sace_14_7_g0015	VIKYFEKNGA	RKCQQNENPA	EYILEAIGAG	ATASVQQNWP	DIWQKSHEYA
(PRED)	sace_15_7_g0387	VIKYFEKNGA	RKCQQNENPA	EYILEAIGAG	ATASVQQNWP	DIWQKSHEYA
(PRED)	sace_24_8_3780	VIKYFEKNGA	RKCQQNENPA	EYILEAIGAG	ATASVQQNWP	DIWQKSHEYA
(PRED)	sace_40_8_h0383	VIKYFEKNGA	RKCQQNENPA	EYILEAIGAG	ATASVQQNWP	DIWQKSHEYA
(PRED)	sace_6_169_fm00	VIKYFEKNGA	RKCQQNENPA	EYILEAIGAG	ATASVQQNWP	DIWQKSHEYA
(PRED)	sace_19_7_3840	VIKYFEKNGA	RKCQQNENPA	EYILEAIGAG	ATASVQQNWP	DIWQKSHEYA
(PRED)	sace_32_7_3770	VIKYFEKNGA	RKCQQNENPA	EYILEAIGAG	ATASVQQNWP	DIWQKSHEYA
(PRED)	sace_56_17_q011	VIKYFEKNGA	RKCQQNENPA	EYILEAIGAG	ATASVQQNWP	DIWQKSHEYA
(PRED)	sace_5_78_bz001	VIKYFEKNGA	RKCQQNENPA	EYILEAIGAG	ATASVQQNWP	DIWQKSHEYA
(PRED)	sace_2_8_h03860	VIKYFEKNGA	RKCQQNENPA	EYILEAIGAG	ATASVQQNWP	DIWQKSHEYA
(PRED)	sace_53_29_ac00	VIKYFEKNGA	RKCQQNENPA	EYILEAIGAG	ATASVQQNWP	DIWQKSHEYA
(PRED)	sace_17_7_g0393	VIKYFEKNGA	RKCQQNENPA	EYILEAIGAG	ATASVHQNWP	DIWQKSHEYA
(PRED)	sace_25_7_g0388	VIKYFEKNGA	RKCQQNENPA	EYILEAIGAG	ATASVQQNWP	DIWQKSHEYA
(PRED)	sace_37_7_g0385	VIKYFEKNGA	RKCQQNENPA	EYILEAIGAG	ATASVHQNWP	DIWQKSHEYA
(PRED)	sace_9_7_g00180	VIKYFEKNGA	RKCQQNENPA	EYILEAIGAG	ATASVHQNWP	DIWQKSHEYA
(PRED)	sace_60_6_f0335	VIKYFEKNGA	RKCQQNENPA	EYILEAIGAG	ATASVQQNWP	DIWQKSHEYA
(PRED)	sace_59_336_lx0	VIEYFEKNGA	RKCQQNENPA	EYILEAIGAG	ATASVQQNWP	DIWQKSHEYA
(PRED)	sace_31_7_3780	VIKYFEKNGA	RKCQQNENPA	EYILEAIGAG	ATASVQQNWP	DIWQKSHEYA
(PRED)	sace_34_8_3770	VIKYFEKNGA	RKCQQNENPA	EYILEAIGAG	ATASVQQNWP	DIWQKSHEYA
(PRED)	sace_58_71_bs00	VIKYFEKNGA	RKCQQNENPA	EYILEAIGAG	ATASVQQNWP	DIWQKSHEYA
(PRED)	sace_7_7_g03880	VIKYFEKNGA	RKCQQNENPA	EYILEAIGAG	ATASVQQNWP	DIWQKSHEYA
(PRED)	sace_35_7_3840	VIKYFEKNGA	RKCQQNENPA	EYILEAIGAG	ATASVHQNWP	DIWQKSHEYA
(PRED)	sace_43_7_g0387	VIKYFEKNGA	RKCQQNENPA	EYILEAIGAG	ATASVHQNWP	DIWQKSHEYA
(PRED)	sace_57_8_h0390	VIKYFEKNGA	RKCQQNENPA	EYILEAIGAG	ATASVQQNWP	DIWQKSHEYA
(PRED)	sace_45_7_g0389	VIKYFEKNGA	RKCQQNENPA	EYILEAIGAG	ATASVQQNWP	DIWQKSHEYA
(PRED)	sace_46_8_h0391	VIKYFEKNGA	RKCQQNENPA	EYILEAIGAG	ATASVHQNWP	DIWQKSHEYA
(PRED)	sace_23_7_3860	VIKYFEKNGA	RKCQQNENPA	EYILEAIGAG	ATASVHQNWP	DIWQKSHEYA
(PRED)	sace_21_7_3790	VIKYFEKNGA	RKCQQNENPA	EYILEAIGAG	ATASVQQNWP	DIWQKSHEYA
(PRED)	sace_8_73_bu001	VIKYFEKNGA	RKCQQNENPA	EYILEAIGAG	ATASVXQNWP	DIWQKSHEYA
(PRED)	sapa_1_8_h03820	VVGYFEKNGA	RKCQQNENPA	EYILEAIGAG	ATASVQQNWS	DIWQTSPENA
(PRED)	sapa_21_8_h0387	VVGYFEKNGA	RKCQQNENPA	EYILEAIGAG	ATASVQQNWS	DIWQTSPENA
(PRED)	sapa_20_8_h0386	VVGYFEKNGA	RKCQQNENPA	EYILEAIGAG	ATASVQQNWS	DIWQTSPENA
(PRED)	sapa_22_8_h0390	VVGYFEKNGA	RKCQQNENPA	EYILEAIGAG	ATASVQQNWS	DIWQTSPENA
(PRED)	sapa_25_8_h0387	VVGYFEKNGA	RKCQQNENPA	EYILEAIGAG	ATASVQQNWS	DIWQTSPENA
(PRED)	sapa_6_8_3750	VVGYFEKNGA	RKCQQNENPA	EYILEAIGAG	ATASVQQNWS	DIWQTSPENA
(PRED)	sapa_9_8_3720	VVGYFEKNGA	RKCQQNENPA	EYILEAIGAG	ATASVQQNWS	DIWQTSPENA
(PRED)	sapa_19_8_h0390	VVGYFEKNGA	RKCQQNENPA	EYILEAIGAG	ATASVQQNWS	DIWQTSPENA
(PRED)	sapa_24_8_h0385	VVGYFEKNGA	RKCQQNENPA	EYILEAIGAG	ATASVQQNWS	DIWQTSPENA
(PRED)	sapa_4_8_h03850	VVGYFEKNGA	RKCQQNENPA	EYILEAIGAG	ATASVQQNWS	DIWQTSPENA
(PRED)	sapa_10_8_3760	VVGYFEKNGA	RKCQQNENPA	EYILEAIGAG	ATASVQQNWS	DIWQTSPENA
(PRED)	sapa_13_8_h0382	VVGYFEKNGA	RKCQQNENPA	EYILEAIGAG	ATASVQQNWS	DIWQTSPENA
(PRED)	sapa_8_8_3750	VVGYFEKNGA	RKCQQNENPA	EYILEAIGAG	ATASVQQNWS	DIWQTSPENA
(PRED)	sapa_11_8_h0383	VVGYFEKNGA	RKCQQNENPA	EYILEAIGAG	ATASVQQNWS	DIWQTSPENA
(PRED)	sapa_5_8_3700	VVGYFEKNGA	RKCQQNENPA	EYILEAIGAG	ATASVQQNWS	DIWQTSPENA
(PRED)	sapa_16_8_h0389	VVGYFEKNGA	RKCEQNENPA	EYILEAIGAG	ATASVEQNWS	DIWQTSPENA
(PRED)	sapa_17_8_3730	VVGYFEKNGA	RKCEQNENPA	EYILEAIGAG	ATASVEQNWS	DIWQTSPENA

(PRED)	sapa_2_8_h03860	VVGYFEKNGA	RKCEQNENPA	EYILEAIGAG	ATASVEQNWS	DIWQTSPENA
(PRED)	sapa_7_8_3740	VVGYFEKNGA	RKCEQNENPA	EYILEAIGAG	ATASVEQNWS	DIWQTSPENA
(PRED)	sapa_23_8_h0385	VVGYFEKNGA	RKCEQNENPA	EYILEAIGAG	ATASVEQNWS	DIWQTSPENA
(PRED)	sapa_3_8_h03890	VVGYFEKNGA	RKCEQNENPA	EYILEAIGAG	ATASVEQNWS	DIWQTSPEKA
(PRED)	sapa_18_8_3730	VVGYFEKNGA	RKCEQNENPA	EYILEAIGAG	ATASVEQNWS	DIWQTSPENA
(PRED)	sami_1_14_399	VVEYFEKNGA	RKCKNNENPA	EYILEAIGAG	ASTTVQQDWS	TIWQASCGNA
(PRED)	sace_4_8_h03690	VIKYFEKNGA	RKCQQNENPA	EYILEAIGAG	ATASVQQNWP	DIWQKSHEYA
(PRED)	saku_1_14_404	VVKYFESNGA	RKCEQSENPA	EYILEAIGAG	ATASVEQNWY	DIWKASPEIA
(PRED)	sace_1_ynr070w	VIKYFEKNGA	RKCQQNENPA	EYILEAIGAG	ATASVQQNWP	DIWQKSHEYA
(PRED)	sace_49_8_h0383	VIKYFEKNGA	RKCQQNENPA	EYILEAIGAG	ATASVXQNWP	DIWQKSHEYA
(PRED)	saeu_1_2_b00130	VLEYFERNGA	RKCQKSENPA	EYILEAIGAG	ATASVQENWH	DIWKTSPEIA
(PRED)	sauv_1_7_3	VLEYFERNGA	RKCQQSENPA	EYILEAIGAG	ATASVEENWH	DIWKTSPEIV
(PRED)	sami_1_17_26	VLGYFERNGA	RKCQPNENPA	EYVLEAIGAG	ATASVLENWH	DIWKASPEMK
(PRED)	zyba_1_02055_AN	VLSYFEGNGA	RKCDQSENPA	EYILEVIGAG	ATATIEEDWH	EVWQNSPECR
(PRED)	zyba_1_07912	VLSYFEGNGA	RKCEQSENPA	EYILEVIGAG	ATATIEEDWH	EVWQNSSERR
(PRED)	zyba_2_2_b00600	VLSYFEGNGA	RKCEQSENPA	EYILEVIGAG	ATATIEEDWH	EVWQNSSERR
(PRED)	zyba_3_3_c03460	VLSYFEGNGA	RKCEQSENPA	EYILEVIGAG	ATATIEEDWH	EVWQNSSERR
(PRED)	zyba_1_04634	VLSYFENNGF	RPCSESENPA	EYILEAIGAG	ATASVEEDWH	AIWQNSEGSQ
(PRED)	zyba_1_06675	VLSYFESNGF	RPCSKSENPA	EYILEAIGAG	ATASVEEDWH	EIWRNSEESQ
(PRED)	zyba_3_2_b02230	VLSYFESNGF	RPCSKSENPA	EYILEAIGAG	ATASVEEDWH	EIWRNSEESQ
(PRED)	zyba_2_1_a00860	VLSYFESNGF	RPCSKSENPA	EYILEAIGAG	ATASVEEDWH	EIWRNSEESQ
(PRED)	zyro_1_a04114g	VLDYFERNGA	RHCEEKENPA	EYILEAIGAG	ATATVKDDWH	EIWQKSDEYG
(PRED)	zyro_1_b14762g	VLDYFERNGA	RHCEQRENPA	EYVLEAIGAG	ATASVKEDWH	EIWQRSEECR
(PRED)	zyba_2_14_n0149	VLSYFERNGA	RHCDQKENPA	EYILEAIGAG	ATAAVDLNWH	DIWENSEECV
(PRED)	zyba_2_33_ag001	VLSYFERNGA	RHCEQKENPA	EYILEAIGAG	ATAAVDRNWH	DIWKNSEECI
(PRED)	lath_1_a01914g	LLNYFEGNGA	RKCEHTENPA	EYILEAIGAG	ATASVKEDWH	EIWTNSPQFN
(PRED)	lawa_1_23_5161	LLDYFEENGA	RKCEKTENPA	EYILEAIGAG	ATASVKEDWH	DIWKKSSQFK
(PRED)	klae_1_14_n0012	LLNYFERNGA	RKCTDDENPA	EYILESIGAG	ATASVKEDWH	QKWLCSEEYV
(PRED)	klla_1_d03432g	LLSYFERNGA	RRCEEKENPA	EYILEAIGAG	ATASVKEDWH	EKWIKSSEFV
(PRED)	klma_1_1_a01880	LLGYFERNGA	RKCTEKENPA	EYILEAIGAG	ATASVKENWH	DKWVNSPEFT
(PRED)	klwi_1_33_ag001	MLSYFERNGA	RKCEDKENPA	EYILEAIGAG	ATASVKEDWH	TKWVNSPEFN
(PRED)	teph_1_a04220	LIRYFENHGA	RKCEVSENPA	EYILDVIGAG	ATTHNDVSMA	DVWVNSEECK
(PRED)	vapo_1_1037_47	VLDYFERNGG	RKCLESENPA	EYILEIIGAG	ASASVEEDWG	SVWNNSPEST
(PRED)	pata_1_2_b05590	ILSYFERNGA	RHCEPHENPA	EYILEAIGAG	ATAAVQENWY	EKWTQSKEFN
(PRED)	wian_1_3_c04380	LLDYFERNGA	RPCENNENPA	EYILESIGAG	ATAQVEEDWY	DKWCKSKEFE
(PRED)	wian_1_3_c04390	LLDYFERNGA	RKCEKHENPA	EYILESIGAG	ATASVHEDWY	EKWANSQEYL
(PRED)	wian_1_7_g01010	LLNYFERNGA	RHCEKSENPA	EYILEAIGAG	ATANINQNWH	DIWSNSQEFA
(PRED)	bain_1_1_a00100	LLDYFERNGA	RQCGRSENPA	EYILESIGAG	ATASVHEDWF	QKWVSSPEYA
(PRED)	bain_1_17_q0038	LLDYFERNGA	RKCGHSENPA	EYILESIGAG	ATASVHEDWF	DKWVSSPEYA
(PRED)	bain_1_8_h00410	ILGYFQRNGA	RRCEDYENPA	EYVLEIIGAG	ATATSTEDWF	TKWVNSTEYL
(PRED)	caal_1_19_5759	ILDYFERNGA	RHCDDKENPA	EYILEAIGAG	ATASTDFDWG	EIWAQSPEKV
(PRED)	caal_11_25_y002	ILDYFERNGA	RHCDDKENPA	EYILEAIGAG	ATASTDFDWG	EIWAQSPEKV
(PRED)	caal_4_4_d03320	ILDYFERNGA	RHCDDKENPA	EYILEAIGAG	ATASTDFDWG	EIWAQSPEKV
(PRED)	caal_12_26_z005	ILDYFERNGA	RHCDDKENPA	EYILEAIGAG	ATASTDFDWG	EIWAQSPEKV
(PRED)	caal_5_30_ad005	ILDYFERNGA	RHCDDKENPA	EYILEAIGAG	ATASTDFDWG	EIWAQSPEKV
(PRED)	caal_8_3_c03320	ILDYFERNGA	RHCDDKENPA	EYILEAIGAG	ATASTDFDWG	EIWAQSPEKV
(PRED)	caal_6_4_d03280	ILDYFERNGA	RHCDDKENPA	EYILEAIGAG	ATASTDFDWS	EIWAQSPEKV
(PRED)	caal_10_3_c0334	ILDYFERNGA	RHCDDKENPA	EYILEAIGAG	ATASTDFDWG	EIWAQSPEKV
(PRED)	caal_3_29_ac005	ILDYFERNGA	RHCDDKENPA	EYILEAIGAG	ATASTDFDWG	EIWAQSPEKV
(PRED)	caal_2_04989	ILDYFERNGA	RHCDDKENPA	EYILEAIGAG	ATASTDFDWG	EIWAQSPEKV
(PRED)	cadu_1_64350	ILDYFERNGA	RHCDDKENPA	EYILEAIGAG	ATASTEFDWG	DIWAQSPEKV
(PRED)	caor_1_h02090	ILDYFERNGA	RHCDEAENPA	EYILEAIGAG	ATASIEEDWF	EIWQNSSEKV
(PRED)	capa_1_600750	ILDYFERNGA	RHCDETENPA	EYILEAIGAG	ATAAIDEDWF	QIWQQSPEKV
(PRED)	loel_1_04930	ILDYFERNGA	RKCQDSENPA	EYILEAIGAG	ATAATTDDWF	DIWSNSPERL
(PRED)	spar_1_5_e03260	ILSYFERNGA	RTCDDHENPA	EYILEAIGAG	ATAVTEYDWF	KIWSESPEKK
(PRED)	sppa_1_7_g03160	ILSYFEKNGA	RTCDDHENPA	EYILEAIGAG	ATAVTEYDWF	KIWTQSPEKR
(PRED)	catr_1_01205	ILDYFERNGA	RHCEDNENPA	EYILEAIGAG	ATASSTFDWG	DIWANSPEKI
(PRED)	catr_1_05498	LLNYFESNGA	RHCGDDENPA	EYILEAIGAG	ATASSNFDWG	EIWAASPQKM
(PRED)	catr_1_05971	ILDYFERNGA	RHCEDHENPA	EYILEAIGAG	ATASTEFDWG	EVWANSSEKI
(PRED)	deha_1_a03696g	IVSYFEGNGA	RKCDDHENPA	EYILEAIGAG	ATASVTQDWF	ETWCNSPEKR
(PRED)	deha_2_5_e00720	IVDYFERNGA	RKCDDHENPA	EYILEAIGAG	ATATVTQDWF	ETWSNSSEKR
(PRED)	scst_1_3_c02890	ILDYFERNGA	RKCGSQENPA	EYILEAIGAG	ATASTEYNWF	DVWSGSAEKK
(PRED)	mebi_1_8_h00300	ITTYFESHGA	RKCSSAENPA	EYILEVIGAG	ATAASEKNWF	DVWQSSTERI
(PRED)	lakl_1_h21010g	VTNYFKKYGA	RECSDTENPA	EYMLEVIGAG	SSVA-KDDWG	EIWKQSTEYQ
(PRED)	caar_1_13_m0142	LLDYFESRGA	RTCQNGENPA	EYILEAIGAG	ATAVAQENWH	DKWIKSKEYT
(PRED)	caar_1_14_n0143	LLDYFEEQGA	RKCGHDENPA	EYILEAIGAG	ATASTEENWN	DKWLNSKLCA
(PRED)	hapo_1_1_a07220	LLDYFESNGA	RKCGPSENPA	EYILEVIGAG	ATAVIDEDWY	EIWKNSSLYE
(PRED)	ogpa_1_1_a01680	MLDYFESNGA	RKCSASENPA	EYILEVIGAG	ATAVIDEDWY	EIWKNSSLYE
(PRED)	piku_1_96_cr001	LLDYFEKQGA	RKCLPSENPA	EYILEAIGAG	SI-STTENWY	EKWCQSEEYI
(PRED)	pime_1_4_d03240	MLNYFQSQGG	RTCSETENPA	EYILEVIGAG	ATGDNIEDWN	QKWLASKQCS
(PRED)	pime_1_1_a12110	LISYFERQGA	KKCGEHENPA	EYILNVIGAG	ATATVEKDWY	KIWCDSEDFV
(PRED)	piku_1_227_hs00	LLSYFERHGT	RKCEPDENPA	EYILTIIGAG	ATSGVMTDWH	QLWLESEECA
(PRED)	pime_1_5_e05800	LLDYLEKRSG	LKCLESDNPA	EYMLEVIGAG	ATSAVSKDWH	QLWLESDECA
(PRED)	pime_1_1_a07690	VIDYFEKNGG	RVCSKEENPA	EYILDVIGAG	ATASTTNDWF	DVWQNSQEYQ
(PRED)	debr_2_5_e03380	VLSYFESKGA	RRCQPDENPA	EYVLEVIGAG	ATAVADRDWG	DVWRKSEENG

```
(PRED) kopa_1_2_b10040 ITGYFERNGA  RKCSPAENPA  EYILEVIGAG  ATASITENWF  DVWIKSPESQ
(PRED) kopa_2_7_g00500 ITGYFERNGA  RKCSPAENPA  EYILEVIGAG  ATASITENWF  DVWVKSPESQ
(PRED) asru_1_13_m0119 ILDYFEGNGA  RTCGIKENPA  EYVLEAIGAG  ATASVDEDWH  QKWMKSPECI
(PRED) asru_1_15_o0045 VLNYFEKNGA  RRCSDAENPA  EYILDVIGAG  ATSVVNEDWN  EKWINSTEFV
(PRED) wian_1_1_a02920 LLNYFERNGG  RPCGELENPA  EYILESIGAG  ATAVVNEDWY  EKWEKSPEYT
(PRED) wian_1_1_a02930 LLNYFERNGG  RPCGELENPA  EYILESIGAG  ATAVVNEDWH  EKWENSPEYI

                       .........1460.........1470.........1480.........1490.........1500
(PRED) asac_1_6_f03560 RENENVNKLV  NDLSAKYMST  ---TP-----  -------VPV  --SKYATSYV
(PRED) ergo_1_abr125c  RENEIVNKLV  NELSEKYLSN  ---AP-----  -------API  --SKYATSYF
(PRED) ercy_1_3604     KANAEIDKIL  DELKKTKTPH  ---NL-----  -------ENN  --DKYATSYF
(PRED) cagl_1_i04862g  STEKEVDHLI  DQLSNQKTES  ---EF-----  -----G-DAP  --TKYATSYA
(PRED) kaaf_1_c00830   SSSKEISELI  SELSQKHSDS  ---EG-----  -----K-ETA  --TKYATSYF
(PRED) kana_1_k01350   STKNKVDNLI  AELSSKENKS  ---EL-----  -----G-EHP  --SKYATSYL
(PRED) saar_1_2_b02590 QTQNKVQELI  KDLSKQETKS  ---EL-----  -----G-DEP  --SKYATSYA
(PRED) sace_1_ydr011w  QTKEKVQDLI  NDLSKQETKS  ---EV-----  -----G-DKP  --SKYATSYA
(PRED) sace_16_1_a0238 QTKEKVQDLI  NDLSKQETKS  ---EV-----  -----G-DKP  --SKYATSYA
(PRED) sace_45_1_a0242 QTKEKVQDLI  NDLSKQETKS  ---EV-----  -----G-DKP  --SKYATSYA
(PRED) sace_48_1_a0238 QTKEKVQDLI  NDLSKQETKS  ---EV-----  -----G-DKP  --SKYATSYA
(PRED) sace_60_4_d0244 QTKEKVQDLI  NDLSKQETKS  ---EV-----  -----G-DKP  --SKYATSYA
(PRED) sace_52_1_a0240 QTKEKVQDLI  NDLSKQETKS  ---EV-----  -----G-DKP  --SKYATSYA
(PRED) sace_46_1_a0240 QTKEKVQDLI  NDLSKQETKS  ---EV-----  -----G-DKP  --SKYATSYA
(PRED) sace_25_1_a0240 QTKEKVQDLI  NDLSKQETKS  ---EV-----  -----G-DKP  --SKYATSYA
(PRED) sace_24_1_2300  QTKEKVQDLI  NDLSKQETKS  ---EV-----  -----G-DKP  --SKYATSYA
(PRED) sace_47_1_a0240 QTKEKVQDLI  NDLSKQETKS  ---EV-----  -----G-DKP  --SKYATSYA
(PRED) sace_7_1_a02410 QTKEKVQDLI  NDLSKQETKS  ---EV-----  -----G-DKP  --SKYATSYA
(PRED) sace_59_110_df0 QTKEKVQDLI  NDLSKQETKS  ---EV-----  -----G-DKP  --SKYATSYA
(PRED) sace_56_1_a0202 QTKEKVQDLI  NDLSKQETKS  ---EV-----  -----G-DKP  --SKYATSYA
(PRED) sace_40_1_a0239 QTKEKVQDLI  NDLSKQETKS  ---EV-----  -----G-DKP  --SKYATSYA
(PRED) sace_15_1_a0242 QTKEKVQDLI  NDLSKQETKS  ---EV-----  -----G-DKP  --SKYATSYA
(PRED) sace_37_1_a0243 QTKEKVQDLI  NDLSKQETKS  ---EV-----  -----G-DKP  --SKYATSYA
(PRED) sace_9_1_a02440 QTKEKVQDLI  NDLSKQETKS  ---EV-----  -----G-DKP  --SKYATSYA
(PRED) sace_22_1_2300  QTKEKVQDLI  NDLSKQETKS  ---EV-----  -----G-DKP  --SKYATSYA
(PRED) sace_29_1_2290  QTKEKVQDLI  NDLSKQETKS  ---EV-----  -----G-DKP  --SKYATSYA
(PRED) sace_34_1_2320  QTKEKVQDLI  NDLSKQETKS  ---EV-----  -----G-DKP  --SKYATSYA
(PRED) sace_58_25_y007 QTKEKVQDLI  NDLSKQETKS  ---EV-----  -----G-DKP  --SKYATSYA
(PRED) sace_23_1_2290  QTKEKVQDLI  NDLSKQETKS  ---EV-----  -----G-DKP  --SKYATSYA
(PRED) sace_6_120_dp00 QTKEKVQDLI  NDLSKQETKS  ---EV-----  -----G-DKP  --SKYATSYA
(PRED) sace_57_1_a0241 QTKEKVQDLI  NDLSKQETKS  ---EV-----  -----G-DKP  --SKYATSYA
(PRED) sace_17_1_a0241 QTKEKVQDLI  NDLSKQETKS  ---EV-----  -----G-DKP  --SKYATSYA
(PRED) sace_21_1_2310  QTKEKVQDLI  NDLSKQETKS  ---EV-----  -----G-DKP  --SKYATSYA
(PRED) sace_49_1_a0246 QTKEKVQDLI  NDLSKQETKS  ---EV-----  -----G-DKP  --SKYATSYA
(PRED) sace_8_2_b02430 QTKEKVQDLI  NDLSKQETKS  ---EV-----  -----G-DKP  --SKYATSYA
(PRED) sace_31_1_2300  QTKEKVQDLI  NDLSKQETKS  ---EV-----  -----G-DKP  --SKYATSYA
(PRED) sace_50_1_a0241 QTKEKVQDLI  NDLSKQETKS  ---EV-----  -----G-DKP  --SKYATSYA
(PRED) sace_4_1_a02360 QTKEKVQDLI  NDLSKQETKS  ---EV-----  -----G-DKP  --SKYATSYA
(PRED) sace_2_1_a02390 QTKEKVQDLI  NDLSKQETKS  ---EV-----  -----G-DKP  --SKYATSYA
(PRED) sace_5_33_ag005 QTKEKVQDLI  NDLSKQETKS  ---EV-----  -----G-DKP  --SKYATSYA
(PRED) sapa_11_1_a0247 QTKEKVQELI  NDLSKEETKS  ---EL-----  -----G-DKP  --SKYATSYA
(PRED) sapa_25_1_a0246 QTKEKVQELI  NDLSKEETKS  ---EL-----  -----G-DKP  --SKYATSYA
(PRED) sapa_4_1_a02470 QTKEKVQELI  NDLSKEETKS  ---EL-----  -----G-DKP  --SKYATSYA
(PRED) sapa_5_1_2350   QTKEKVQELI  NDLSKEETKS  ---EL-----  -----G-DKP  --SKYATSYA
(PRED) sapa_9_1_2360   QTKEKVQELI  NDLSKEETKS  ---EL-----  -----G-DKP  --SKYATSYA
(PRED) sapa_14_1_a0244 QTKEKVQELI  NDLSKEETKS  ---EL-----  -----G-DEP  --SKYATSYA
(PRED) sapa_8_1_2350   QTKEKVQELI  NDLSKEETKS  ---EL-----  -----G-DEP  --SKYATSYA
(PRED) sapa_17_1_2380  QTKEKVQELI  NDLSKEETRS  ---EL-----  -----G-DKP  --SKYATSYA
(PRED) sapa_7_1_2370   QTKEKVQELI  NDLSKEETRS  ---EL-----  -----G-DKP  --SKYATSYA
(PRED) sapa_2_1_a02460 QTKEKVQELI  NDLSKEETRS  ---EL-----  -----G-DKP  --SKYATSYA
(PRED) sapa_23_1_a0248 QTKEKVQELI  NDLSKEETRS  ---EL-----  -----G-DKP  --SKYATSYA
(PRED) sapa_3_1_a02470 QTKEKVQELI  NDLSKEETRS  ---EL-----  -----G-DKP  --SKYATSYA
(PRED) sapa_18_1_2390  QTKEKVQELI  NDLSKEETKS  ---EL-----  -----G-DKP  --SKYATSYA
(PRED) sami_1_4_244    KTKYEVQNLI  NDLSKQETKS  ---DF-----  -----G-DKP  --SKYATSYA
(PRED) saku_1_4_262    QSQDKVQELI  NDLSKQETKS  ---EF-----  -----G-DQP  --SKYATSYI
(PRED) saba_1_58_bf002 QTKEKVQELI  DDLSKQENIS  ---DV-----  -----G-GEP  --SKYATSYF
(PRED) saeu_1_4_d02400 QTQDKVQELI  DDLSKQENKP  ---EV-----  -----G-DEP  --SKYATSYS
(PRED) naca_1_e01640   RTAEKVDELI  KELSSKPDDA  ---NG-----  -----D-SNS  --GKYATSYG
(PRED) nada_1_g01850   ASENKVSEII  NELSQKELTT  ---GD-----  -----D-VNT  --AKYATSYA
(PRED) naca_1_e01630   AAEEKVSQMI  SELSQKRDES  ---DI-----  -----G-DKA  --TKYATSYR
(PRED) nada_1_g01840   EVTNEVTSLI  EELREKPDEA  ---HI-----  -----G-KSP  --SKFATSYK
(PRED) kaaf_1_c00820   NSQNEITELI  ENLSKEVDPS  ---KN-----  -----G-AKP  --SKYATSYL
(PRED) teph_1_m00640   NSEIEINNMI  KGLSANANSD  ---GE-----  -----G-KAA  --QKYATSYF
(PRED) vapo_1_1036_28  ETEQRIEEMI  RDLTAK-GAD  ---SA-----  -----D-DDN  --SKYATSYF
(PRED) tebl_1_i01760   NLANEVDKLI  LELQAKEDPS  ---VF-----  -----S-RSK  --TKYANSYV
(PRED) tode_1_d04040   TNEEKIQKMI  SDLSSKPDDV  ---SE-----  -----G-KSA  --TKYATSYF
(PRED) naca_1_e01650   TVNEKVDELI  RDLSQQENKV  ---DP-----  -----A--AL  --STYATSYA
```

(PRED)	tebl_1_g02820	ENRIKIDHML	EELHNSPPET	---GT-----	-----V-SN-	--SKYATSYF
(PRED)	lakl_1_c11616g	ETNTQVEAYI	KELFGKRDPA	---DD-----	-----GKSSP	--SKYATSYF
(PRED)	saar_1_8_h03780	QVNEKVNTMI	KDLSSKSLQN	---SI-----	-------VNQ	--SKYATSYS
(PRED)	sace_14_7_g0015	NINEKINDMI	KDLSSTTLHK	---TA-----	-------TRA	--SKYATSYS
(PRED)	sace_15_7_g0387	NINEKINDMI	KDLSSTTLHK	---TA-----	-------TRA	--SKYATSYS
(PRED)	sace_24_8_3780	NINEKINDMI	KDLSSTTLHK	---TA-----	-------TRA	--SKYATSYS
(PRED)	sace_40_8_h0383	NINEKINDMI	KDLSSTTLHK	---TA-----	-------TRA	--SKYATSYS
(PRED)	sace_6_169_fm00	NINEKINDMI	KDLSSTTLHK	---TA-----	-------TRA	--SKYATSYS
(PRED)	sace_19_7_3840	NINEKINDMI	KDLSSTTLHK	---TA-----	-------TRA	--SKYATSYS
(PRED)	sace_32_7_3770	NINEKINDMI	KDLSSTTLHK	---TA-----	-------TRA	--SKYATSYS
(PRED)	sace_56_17_q011	NINEKINDMI	KDLSSTTLHK	---TA-----	-------TRA	--SKYATSYS
(PRED)	sace_5_78_bz001	NINEKINDMI	KDLSSTTLHK	---TA-----	-------TRA	--SKYATSYS
(PRED)	sace_2_8_h03860	NINEKINDMI	KDLSSTTLHK	---TA-----	-------TRA	--SKYATSYS
(PRED)	sace_53_29_ac00	NINEKINDMI	KDLSSTTLHK	---TA-----	-------TRA	--SKYATSYS
(PRED)	sace_17_7_g0393	NINEKINDMI	KDLSSTTLHK	---TA-----	-------TRA	--SKYATSYS
(PRED)	sace_25_7_g0388	NINEKINDMI	KDLSSTTLHK	---TA-----	-------TRA	--SKYATSYS
(PRED)	sace_37_7_g0385	NINEKINDMI	KDLSSTTLHK	---TA-----	-------TRA	--SKYATSYS
(PRED)	sace_9_7_g00180	NINEKINDMI	KDLSSTTLHK	---TA-----	-------TKA	--SKYATSYS
(PRED)	sace_60_6_f0335	NINEKINDMI	KDLSSTTLHK	---TA-----	-------TRA	--SKYATSYS
(PRED)	sace_59_336_lx0	NINEKINDMI	KDLSSATLHK	---TA-----	-------TKA	--SKYATSYS
(PRED)	sace_31_7_3780	NINEKINDMI	KDLSSTTLHK	---TA-----	-------TRA	--SKYATSYS
(PRED)	sace_34_8_3770	NINEKINDMI	KDLSSTTLHK	---TA-----	-------TRA	--SKYATSYS
(PRED)	sace_58_71_bs00	NINEKINDMI	KDLSSTTLHK	---TA-----	-------TRA	--SKYATSYS
(PRED)	sace_7_7_g03880	NINEKINDMI	KDLSSTTLHK	---TA-----	-------TRA	--SKYATSYS
(PRED)	sace_35_7_3840	NINEKINDMI	KDLSSTTLHK	---TA-----	-------TRA	--SKYATSYS
(PRED)	sace_43_7_g0387	NINEKINDMI	KDLSSTTLHK	---TA-----	-------TRA	--SKYATSYS
(PRED)	sace_57_8_h0390	NINEKINDMI	KDLSSTTLHK	---TA-----	-------TRA	--SKYATSYS
(PRED)	sace_45_7_g0389	NINEKINDMI	KDLSSTTLHK	---TA-----	-------TRA	--SKYATSYS
(PRED)	sace_46_8_h0391	NINEKINDMI	KDLSSTTLHK	---TA-----	-------TRA	--SKYATSYS
(PRED)	sace_23_7_3860	NINEKINDMI	KDLSSTTLHK	---TA-----	-------TRA	--SKYATSYS
(PRED)	sace_21_7_3790	NINEKINDMI	KDLSSTTLHK	---TA-----	-------TRA	--SKYATSYS
(PRED)	sace_8_73_bu001	NINEKINDMI	KDLSSTTLHK	---TA-----	-------TRA	--SKYATSYS
(PRED)	sapa_1_8_h03820	NVDEKVNAMI	KDLSSTTLQK	---AA-----	-------VKA	--SKYATSYF
(PRED)	sapa_21_8_h0387	NVDEKVNAMI	KDLSSTTLQK	---AA-----	-------VKA	--SKYATSYF
(PRED)	sapa_20_8_h0386	NVDEKVNAMI	KDLSSTTLQK	---AA-----	-------VKA	--SKYATSYF
(PRED)	sapa_22_8_h0390	NVDEKVNAMI	KDLSSTTLQK	---AA-----	-------VKA	--SKYATSYF
(PRED)	sapa_25_8_h0387	NVDEKVNAMI	KDLSSTTLQK	---AA-----	-------VKA	--SKYATSYF
(PRED)	sapa_6_8_3750	NVDEKVNAMI	KDLSSTTLQK	---AA-----	-------VKA	--SKYATSYF
(PRED)	sapa_9_8_3720	NVDEKVNAMI	KDLSSTTLQK	---AA-----	-------VKA	--SKYATSYF
(PRED)	sapa_19_8_h0390	NVDEKVNAMI	KDLSSTTLQK	---AA-----	-------VKA	--SKYATSYF
(PRED)	sapa_24_8_h0385	NVDEKVNAMI	KDLSSTTLQK	---AA-----	-------VKA	--SKYATSYF
(PRED)	sapa_4_8_h03850	NVDEKVNAMI	KDLSSTTLQK	---AA-----	-------VKA	--SKYATSYF
(PRED)	sapa_10_8_3760	NVDEKVNAMI	KDLSSTTLQK	---AA-----	-------VKA	--SKYATSYF
(PRED)	sapa_13_8_h0382	NVDEKVNAMI	KDLSSTTLQK	---AA-----	-------VKA	--SKYATSYF
(PRED)	sapa_8_8_3750	NVDEKVNAMI	KDLSSTTLQK	---AA-----	-------VKA	--SKYATSYF
(PRED)	sapa_11_8_h0383	NVDEKVNAMI	KDLSSTTLQK	---AA-----	-------VKA	--SKYATSYF
(PRED)	sapa_5_8_3700	NVDEKVNAMI	KDLSSTTLQK	---AA-----	-------VKA	--SKYATSYF
(PRED)	sapa_16_8_h0389	NIDEKVSAMI	KDLSSTTLQK	---TA-----	-------VKA	--SKYATSYF
(PRED)	sapa_17_8_3730	NIDEKVSAMI	KDLSSTTLQK	---TA-----	-------VKA	--SKYATSYF
(PRED)	sapa_2_8_h03860	NIDEKVSAMI	KDLSSTTLQK	---TA-----	-------VKA	--SKYATSYF
(PRED)	sapa_7_8_3740	NIDEKVSAMI	KDLSSTTLQK	---TA-----	-------VKA	--SKYATSYF
(PRED)	sapa_23_8_h0385	NIDEKVSAMI	KDLSSTTLQK	---TA-----	-------VKA	--SKYATSYF
(PRED)	sapa_3_8_h03890	NIDEKVSAMI	KDLSSTTLQK	---TA-----	-------VKA	--SKYATSYF
(PRED)	sapa_18_8_3730	NIDEKVSAMI	KDLSPTTLPK	---TA-----	-------VKA	--SKYATLYF
(PRED)	sami_1_14_399	NVCKKISTMI	KDLSSSKSKK	---VA-----	-------VKL	--SKYATSYS
(PRED)	sace_4_8_h03690	NINKKINDMI	KDLSSTTLHK	---TA-----	-------TRA	--SKYATSYS
(PRED)	saku_1_14_404	KVNEKVDTMI	KDLPSSSVRK	---ID-----	-------VKP	--SKYATSYF
(PRED)	sace_1_ynr070w	NINEKINDMI	KDLSSTTLHK	---TA-----	-------TRA	--SKYATSYS
(PRED)	sace_49_8_h0383	NINEKINDMI	KDLSSTTLHK	---TA-----	-------TRA	--SKYATSYS
(PRED)	saeu_1_2_b00130	KVDEKVSTMI	KDLSSTS-QD	---ID-----	-------KNP	--SKYATSYT
(PRED)	sauv_1_7_3	KVDEKVSTMI	SDLSSAS-QN	---TD-----	-------TNP	--SKYATSYS
(PRED)	sami_1_17_26	TANEEIDTMI	KDMSSSVSQN	---TG-----	-------IDS	--SKYAASYF
(PRED)	zyba_1_02055_AN	ETDERVTQWI	QELSSQAQEK	---SE-----	-----IGEKQ	--SKYATSYL
(PRED)	zyba_1_07912	ETDEHVTQWI	QELSSQAQEK	---SE-----	-----IGEKP	--SKYATSYL
(PRED)	zyba_2_2_b00600	ETDEHVTQWI	QELSSQAQEK	---SE-----	-----IGEKP	--SKYATSYL
(PRED)	zyba_3_3_c03460	ETDEHVTQWI	QELSSQAQEK	---SE-----	-----IGEKP	--SKYATSYL
(PRED)	zyba_1_04634	DVDNKIKTWI	EEPSSRAFND	---TQ-----	-----LTDKP	--SKYATDYF
(PRED)	zyba_1_06675	EVDDKIKKLI	EEPSSTAFND	---TQ-----	-----LVDKP	--SKYATDYF
(PRED)	zyba_3_2_b02230	EVDDKIKKLI	EEPSSTAFND	---TQ-----	-----LVDKP	--SKYATDYF
(PRED)	zyba_2_1_a00860	EVDDKIKKLI	EEPSSMAFND	---TQ-----	-----LVDKP	--SKYATDYF
(PRED)	zyro_1_a04114g	ATEQKASEWI	EKLANQECTN	---EE-----	-----IGSAP	--TKYATDYF
(PRED)	zyro_1_b14762g	ATDQKVAEWI	QELSTQDFDV	---GE-----	-----VGEKS	--SKYATNYF
(PRED)	zyba_2_14_n0149	QMNQKATDWI	NELTSQPRRD	---ED-----	-----A--NP	--KKYATSYM
(PRED)	zyba_2_33_ag001	QVNQKVTDWI	NELASRPRGD	---ED-----	-----A--NP	--RKYATSYM
(PRED)	lath_1_a01914g	EANEEIENYL	RDSKQNSPPA	---DK-----	-----N-EKI	--SKYATSYS

(PRED)	lawa_1_23_5161	IANEEIDNYV	RDSSGSS--G	---TE-----	-----G-DRV	--SKYATSYS
(PRED)	klae_1_14_n0012	KTNAEVEELI	KELSS--GQD	---TT-----	-----DSQPI	--SKYATSYA
(PRED)	klla_1_d03432g	SVNQEINDLI	EKLAHQPNDD	---S------	-----STELI	--TKYATPYW
(PRED)	klma_1_1_a01880	QVNKEIDELI	EKLSNQPVDE	---SA-----	-----SSALV	--SKYATPYR
(PRED)	klwi_1_33_ag001	NTNVEIAELI	EKLSSQPEDD	---S------	-----SSKLM	--SKFATPYK
(PRED)	teph_1_a04220	NSEEDLANLI	TE-GN-V-NN	---EH-----	-----GGNVS	--KKYATSYW
(PRED)	vapo_1_1037_47	TVQNDIEGLL	SEKGNRP-DG	---EH-----	-------SIN	--KTYATSYF
(PRED)	pata_1_2_b05590	DTTVEVTKLI	ESTQASNPEE	---SV-----	-----NKNAG	I---YATPYW
(PRED)	wian_1_3_c04380	KTTKEIQALM	QEGRD-LPEN	---DG-----	-----AEKSE	LHKTYALPYW
(PRED)	wian_1_3_c04390	DTTREIEILM	RETNEKLGDQ	---DQ-----	-----ASNKE	LQSTFALPYL
(PRED)	wian_1_7_g01010	DTTKEIEILM	NQANEKLGDD	---DQ-----	-----SSNKE	LQKTYALPYL
(PRED)	bain_1_1_a00100	DTTETISKLI	QETSQIVSQT	----------	------PTEQ	LNSTYAAPYL
(PRED)	bain_1_17_q0038	DTTETISKLI	QETSQIVSQT	----------	------PTEQ	LNSTYAAPYL
(PRED)	bain_1_8_h00410	EVTKEVNRL-	-EGVRSIPSL	----------	------DVHG	-DSTFAMPLW
(PRED)	caal_1_19_5759	QTDAKRDELI	NESAKNATDT	SATDS-----	-----PSEKN	LTSKYATPYW
(PRED)	caal_11_25_y002	QTDAKRDELI	NESAKNATDT	SATDS-----	-----PSEKN	LTSKYATPYW
(PRED)	caal_4_4_d03320	QTDAKRDELI	NESAKNATDT	SATDS-----	-----PSEKN	LTSKYATPYW
(PRED)	caal_12_26_z005	QTDAKRDELI	NESAKNATDT	SATDS-----	-----PSEKN	LTSKYATPYW
(PRED)	caal_5_30_ad005	QTDAKRDELI	NESAKNATDT	SATDS-----	-----PSEKN	LTSKYATPYW
(PRED)	caal_8_3_c03320	QTDAKRDELI	NESAKNATDT	SATDS-----	-----PSEKN	LTSKYATPYW
(PRED)	caal_6_4_d03280	QTDAKRDELI	NESAKNATDT	SATDS-----	-----PSEKN	LTSKYATPYW
(PRED)	caal_10_3_c0334	QTDAKRDELI	NESAKNATDT	SATDS-----	-----PSEKN	LTSKYATPYW
(PRED)	caal_3_29_ac005	QTDAKRDELI	NESAKNATDT	SATDS-----	-----PSEKN	LTSKYATPYW
(PRED)	caal_2_04989	QTDAKRDELI	NESAKNATDT	SATDS-----	-----PSEKN	LTSKYATPYW
(PRED)	cadu_1_64350	QTDAKRDELI	KESAQNAADT	--TTS-----	-----SSEKN	STSKYATPYW
(PRED)	caor_1_h02090	KEDEKLNNLI	QELEKKPSDL	SPEE------	-------EKQ	LHHKYATPYF
(PRED)	capa_1_600750	DEDQKLDNLI	RELESKPSEL	SHKE------	-------EKQ	LHHKYATPYW
(PRED)	loel_1_04930	AADKKRDELV	ESLKSKPSDL	TKEQ------	-------EIE	LSHRYAMPYW
(PRED)	spar_1_5_e03260	EADIKRDQLI	NELSK--VDM	ANNTG-----	-----SNTKK	LHRKYATGYF
(PRED)	sppa_1_7_g03160	EADAKRDQLI	--LAK--AES	SNHTS-----	-----SDSKD	LQRKYATGYF
(PRED)	catr_1_01205	QSDNKRDELI	QESSKK---S	NSGST-----	------EDKK	LQQKYATPYL
(PRED)	catr_1_05498	DTEKKRDELI	EESSKKPVGT	G-SEK-----	------EDKK	LHQKYATPYW
(PRED)	catr_1_05971	QTDKKRDQLI	NESSQKKLAT	DLSEK-----	------EVKK	LSSKYATPYF
(PRED)	deha_1_a03696g	ASDIERDRLI	EELSKQVEDV	--HDP-----	-----KEIKQ	LRSTYAVPYW
(PRED)	deha_2_5_e00720	ASDIERDRLI	EELSRKAEDN	--NDP-----	-----KELKQ	LRSTYAVPYW
(PRED)	scst_1_3_c02890	ETDKVRDQLI	SDLASKPNDE	SGYTA-----	-----RELNQ	MKNQYATPYW
(PRED)	mebi_1_8_h00300	KAEERIDELI	AAGSSRAPMT	K-DDP-----	-----LNSKE	LKSKYAASVS
(PRED)	lakl_1_h21010g	DFVGEYDDLY	TKLLNTNKGD	----------	-------DQD	LEKKFAVPYT
(PRED)	caar_1_13_m0142	AVTEQITKLI	QDTAH-LPSG	SSD-------	---------H	GTSSYAMPYM
(PRED)	caar_1_14_n0143	ETTKEISELI	ERTSK-IQQS	NHS-------	---------G	LTGTYASPYV
(PRED)	hapo_1_1_a07220	KTCADVEKLI	NDTKG-MQSS	DQS-------	---------H	LQSRFAVPYT
(PRED)	ogpa_1_1_a01680	KTCADVEKLI	NDTKG-MQSS	DQS-------	---------H	LQSRFAVPYR
(PRED)	piku_1_96_cr001	KVTKDIEQLI	EETSK-LPSV	DNS-------	---------E	LHTKFAAPYY
(PRED)	pime_1_4_d03240	ELTKEVGELI	ERTSK-LPAS	DNS-------	---------E	LTARYAASTY
(PRED)	pime_1_1_a12110	QRSEEIDKII	SDSSKNADSS	SHE-------	---------E	LKNKYATSYF
(PRED)	piku_1_227_hs00	NVTKKIDELV	ETGRHKEIKV	DP--------	---------E	LTKTYATPYS
(PRED)	pime_1_5_e05800	QVASKIDELI	ESGSHITAEV	DE--------	---------E	LKKTFATPYF
(PRED)	pime_1_1_a07690	SVSAEVDHLV	QQGLTIQDEE	SDN-------	--------AD	LDSTYATSYQ
(PRED)	debr_2_5_e03380	KVSQEVEQMT	RRAVKTTSAT	NETSATSATS	ANKATATASS	STSQFATPYH
(PRED)	kopa_1_2_b10040	EVSQEISTLV	TRAGNSTSSV	DDA-------	---------A	HLGTFATPWH
(PRED)	kopa_2_7_g00500	DVSNEISNLV	AKAGNGTSSF	DDT-------	---------T	QLGTFATPLF
(PRED)	asru_1_13_m0119	QTNKYIEFLI	QDGLSKPINE	SDP-------	-------E--	LRNKFATPYF
(PRED)	asru_1_15_o0045	EENNQTDLLI	RQGQSM-TNT	DDAEK-----	------IE--	LKKTFATPYS
(PRED)	wian_1_1_a02920	QLCYEVDTVV	K--------T	KAPSK-----	------TEEQ	LGSSYSSSWL
(PRED)	wian_1_1_a02930	QASTEIDEIN	KEGLRLTATT	TVTAS-----	------EDKY	LHSTYATSWF

		1510	1520	1530	1540	1550
(PRED)	asac_1_6_f03560	YQFRHVIART	CLIFYRDLDY	LLSKQFLYLS	AGLFIGF-TF	YDVGTSYTGL
(PRED)	ergo_1_abr125c	YQFRYVVSRT	MLIFYRDLDY	LLSKQFLFLS	AGLFIGF-TF	YDVGTSYTGL
(PRED)	ercy_1_3604	YQFWYVQQRT	CKMFFRDLDY	IMAKQMLFLV	GGLFIGF-TF	YDVGDSYTGL
(PRED)	cagl_1_i04862g	YQFKWVLIRT	SMSLWRNLDY	IMSKMMLMTV	GGLYIGF-TF	YDPGDSYTGL
(PRED)	kaaf_1_c00830	YQFRYVWLRT	ATMFWRNLDY	LMSKMMLMTV	GGLYIGF-TF	FNVGKSYIGL
(PRED)	kana_1_k01350	FQFRHVLIRT	NITFWRSLNY	LMSKMMLMTV	GGLYIGF-TF	YDPGNSYTGL
(PRED)	saar_1_2_b02590	YQFKYVLIRT	STSFWRSLNY	IMSKMMLMLV	GGLYIGF-TF	FNVGKSYVGL
(PRED)	sace_1_ydr011w	YQFRYVLIRT	STSFWRSLNY	IMSKMMLMLV	GGLYIGF-TF	FNVGKSYVGL
(PRED)	sace_16_1_a0238	YQFRYVLIRT	STSFWRSLNY	IMSKMMLMLV	GGLYIGF-TF	FNVGKSYVGL
(PRED)	sace_45_1_a0242	YQFRYVLIRT	STSFWRSLNY	IMSKMMLMLV	GGLYIGF-TF	FNVGKSYVGL
(PRED)	sace_48_1_a0238	YQFRYVLIRT	STSFWRSLNY	IMSKMMLMLV	GGLYIGF-TF	FNVGKSYVGL
(PRED)	sace_60_4_d0244	YQFRYVLIRT	STSFWRSLNY	IMSKMMLMLV	GGLYIGF-TF	FNVGKSYVGL
(PRED)	sace_52_1_a0240	YQFRYVLIRT	STSFWRSLNY	IMSKMMLMLV	GGLYIGF-TF	FNVGKSYVGL
(PRED)	sace_46_1_a0240	YQFRYVLIRT	STSFWRSLNY	IMSKMMLMLV	GGLYIGF-TF	FNVGKSYVGL
(PRED)	sace_25_1_a0240	YQFRYVLIRT	STSFWRSLNY	IMSKMMLMLV	GGLYIGF-TF	FNVGKSYVGL
(PRED)	sace_24_1_2300	YQFRYVLIRT	STSFWRSLNY	IMSKMMLMLV	GGLYIGF-TF	FNVGKSYVGL
(PRED)	sace_47_1_a0240	YQFRYVLIRT	STSFWRSLNY	IMSKMMLMLV	GGLYIGF-TF	FNVGKSYVGL
(PRED)	sace_7_1_a02410	YQFRYVLIRT	STSFWRSLNY	IMSKMMLMLV	GGLYIGF-TF	FNVGKSYVGL

```
(PRED) sace_59_110_df0 YQFRYVLIRT STSFWRSLNY IMSKMMLMLV GGLYIGF-TF FNVGKSYVGL
(PRED) sace_56_1_a0202 YQFRYVLIRT STSFWRSLNY IMSKMMLMLV GGLYIGF-TF FNVGKSYVGL
(PRED) sace_40_1_a0239 YQFRYVLIRT STSFWRSLNY IMSKMMLMLV GGLYIGF-TF FNVGKSYVGL
(PRED) sace_15_1_a0242 YQFRYVLIRT STSFWRSLNY IMSKMMLMLV GGLYIGF-TF FNVGKSYVGL
(PRED) sace_37_1_a0243 YQFRYVLIRT STSFWRSLNY IMSKMMLMLV GGLYIGF-TF FNVGKSYVGL
(PRED) sace_9_1_a02440 YQFRYVLIRT STSFWRSLNY IMSKMMLMLV GGLYIGF-TF FNVGKSYVGL
(PRED) sace_22_1_2300  YQFRYVLIRT STSFWRSLNY IMSKMMLMLV GGLYIGF-TF FNVGKSYVGL
(PRED) sace_29_1_2290  YQFRYVLIRT STSFWRSLNY IMSKMMLMLV GGLYIGF-TF FNVGKSYVGL
(PRED) sace_34_1_2320  YQFRYVLIRT STSFWRSLNY IMSKMMLMLV GGLYIGF-TF FNVGKSYVGL
(PRED) sace_58_25_y007 YQFRYVLIRT STSFWRSLNY IMSKMMLMLV GGLYIGF-TF FNVGKSYVGL
(PRED) sace_23_1_2290  YQFRYVLIRT STSFWRSLNY IMSKMMLMLV GGLYIGF-TF FNVGKSYVGL
(PRED) sace_6_120_dp00 YQFRYVLIRT STSFWRSLNY IMSKMMLMLV GGLYIGF-TF FNVGKSYVGL
(PRED) sace_57_1_a0241 YQFRYVLIRT STSFWRSLNY IMSKMMLMLV GGLYIGF-TF FNVGKSYVGL
(PRED) sace_17_1_a0241 YQFRYVLIRT STSFWRSLNY IMSKMMLMLV GGLYIGF-TF FNVGKSYVGL
(PRED) sace_21_1_2310  YQFRYVLIRT STSFWRSLNY IMSKMMLMLV GGLYIGF-TF FNVGKSYVGL
(PRED) sace_49_1_a0246 YQFRYVLIRT STSFWRSLNY IMSKMMLMLV GGLYIGF-TF FNVGKSYVGL
(PRED) sace_8_2_b02430 YQFRYVLIRT STSFWRSLNY IMSKMMLMLV GGLYIGF-TF FNVGKSYVGL
(PRED) sace_31_1_2300  YQFRYVLIRT STSFWRSLNY IMSKMMLMLV GGLYIGF-TF FNVGKSYVGL
(PRED) sace_50_1_a0241 YQFRYVLIRT STSFWRSLXY IMSKMMLMLV GGLYIGF-TF FNVGKSYVGL
(PRED) sace_4_1_a02360 YQFRYVLIRT STSFWRSLNY IMSKMMLMLV GGLYIGF-TF FNVGKSYVGL
(PRED) sace_2_1_a02390 YQFRYVLIRT STSFWRSLNY IMSKMMLMLV GGLYIGF-TF FNVGKSYVGL
(PRED) sace_5_33_ag005 YQFRYVLIRT STSFWRSLNY IMSKMMLMLV GGLYIGF-TF FNVGKSYVGL
(PRED) sapa_11_1_a0247 YQFKYVLIRT STSFWRSLNY IMSKMMLMLV GGLYIGF-TF FNVGKSYVGL
(PRED) sapa_25_1_a0246 YQFKYVLIRT STSFWRSLNY IMSKMMLMLV GGLYIGF-TF FNVGKSYVGL
(PRED) sapa_4_1_a02470 YQFKYVLIRT STSFWRSLNY IMSKMMLMLV GGLYIGF-TF FNVGKSYVGL
(PRED) sapa_5_1_2350   YQFKYVLIRT STSFWRSLNY IMSKMMLMLV GGLYIGF-TF FNVGKSYVGL
(PRED) sapa_9_1_2360   YQFKYVLIRT STSFWRSLNY IMSKMMLMLV GGLYIGF-TF FNVGKSYVGL
(PRED) sapa_14_1_a0244 YQFKYVLIRT STSFWRSLNY IMSKMMLMLV GGLYIGF-TF FNVGKSYVGL
(PRED) sapa_8_1_2350   YQFKYVLIRT STSFWRSLNY IMSKMMLMLV GGLYIGF-TF FNVGKSYVGL
(PRED) sapa_17_1_2380  YQFKYVLIRT STSFWRSLNY IMSKMMLMLV GGLYIGF-TF YNVGKSYVGL
(PRED) sapa_7_1_2370   YQFKYVLIRT STSFWRSLNY IMSKMMLMLV GGLYIGF-TF FNVGKSYVGL
(PRED) sapa_2_1_a02460 YQFKYVLIRT STSFWRSLNY IMSKMMLMLV GGLYIGF-TF FNVGKSYVGL
(PRED) sapa_23_1_a0248 YQFKYVLIRT STSFWRSLNY IMSKMMLMLV GGLYIGF-TF FNVGKSYVGL
(PRED) sapa_3_1_a02470 YQFKYVLIRT STSFWRSLNY IMSKMMLMLV GGLYIGF-TF FNVGKSYVGL
(PRED) sapa_18_1_2390  YQFKYVLIRT STSFWRSLNY IMSKMMLMLV GGLYIGF-TF FNVGKSYVGL
(PRED) sami_1_4_244    YQFKYVLIRT STSFWRSLNY IMSKLMLMLV GGLYIGF-TF FNVGKSYVGL
(PRED) saku_1_4_262    YQFRYVLIRT STSFWRSLNY IMSKMMLMMV GGLYIGF-TF FNVGKSYVGL
(PRED) saba_1_58_bf002 YQFKYVLIRT STSFWRSLNY IMSKLMLMLV GGLYIGF-TF YNVGESYVGL
(PRED) saeu_1_4_d02400 YQFRYVLIRT STSFWRSLNY IMSKLMLMLV GGLYIGF-TF YNVGNSYVGL
(PRED) naca_1_e01640   YQFSYVLRRT GITFWRNLNY IMSKMMLMTV GGLYIGF-TF YDVGKSYTGL
(PRED) nada_1_g01850   YQFNYVLRRT SLTFWRNLNY IMSKMMLLTT GGLFIGF-TF FGVGSSYTGL
(PRED) naca_1_e01630   YQFGFVLRRT GITFWRNLNY IMSKMMLMTV AGLFIGF-TF FGVGSSFTGL
(PRED) nada_1_g01840   YQFVYVLRRT AITFWRNLNY IMSKFMLFTV GGLYVGF-TF FDVGTSYTGL
(PRED) kaaf_1_c00820   YQFRYVWART NVTFWRSLNY IMSKFMLMAV GGLYIGF-TF FDVGNSYVGL
(PRED) teph_1_m00640   YQFRYVYFRT ATILWRDLNY IMSKLMLHIV GGLFIGF-TF YNVGTTYAGL
(PRED) vapo_1_1036_28  YQFRYVWIRT ATTLWRDLPY IMSKMMLNIT AGLFIGF-TF YDVGKTFTGL
(PRED) tebl_1_i01760   YQFYHVWLRT TLVFWRSTNY IFSKMMLFTF AGLFIGF-SF YNVGYSYAGL
(PRED) tode_1_d04040   YQFKYVYLRT FTTFWRDVNY LMSKLMLMVV GGLYVGF-TF YDVGESYTGL
(PRED) naca_1_e01650   YQFNYVLKRT AITFWRNVTY IGSKLLLLGL GGLFIGF-TF FNVGKSFIGL
(PRED) tebl_1_g02820   YQFRHVLWRN EVVFWRSMHY IFSKLMLHIV GGLFIGF-TF FNVGTSYRGL
(PRED) lakl_1_c11616g  YQFRYVWQRS SVMFWRDVNY IMSKMMLFLV GGLFIGF-TF YNVGSSFTGL
(PRED) saar_1_8_h03780 YQFRYVLIRS SLAFWRNLDY MMAKMMLLMI SGLFIGF-TF FDVGNTTIGL
(PRED) sace_14_7_g0015 YQFHHVLKRS SLTFWRNLNY IMAKMMLLMI SGLFIGF-TF FHVGVNAIGL
(PRED) sace_15_7_g0387 YQFHHVLKRS SLTFWRNLNY IMAKMMLLMI SGLFIGF-TF FHVGVNAIGL
(PRED) sace_24_8_3780  YQFHHVLKRS SLTFWRNLNY IMAKMMLLMI SGLFIGF-TF FHVGVNAIGL
(PRED) sace_40_8_h0383 YQFHHVLKRS SLTFWRNLNY IMAKMMLLMI SGLFIGF-TF FHVGVNAIGL
(PRED) sace_6_169_fm00 YQFHHVLKRS SLTFWRNLNY IMAKMMLLMI SGLFMGF-TF FHVGVNAIGL
(PRED) sace_19_7_3840  YQFHHVLKRS SLTFWRNLNY IMAKMMLLMI SGLFIGF-TF FHVGVNAIGL
(PRED) sace_32_7_3770  YQFHHVLKRS SLTFWRNLNY IMAKMMLLMI SGLFIGF-TF FHVGVNAIGL
(PRED) sace_56_17_q011 YQFHHVLKRS SLTFWRNLNY IMAKMMLLMI SGLFIGF-TF FHVGVNAIGL
(PRED) sace_5_78_bz001 YQFHHVLKRS SXTFWRNLNY IMAKMMLLMI SGLFIGF-TF FHVGVNAIGL
(PRED) sace_2_8_h03860 YQFHHVLKRS SLTFWRNLNY IMAKMMLLMI SGLFIGF-TF FHVGVNAIGL
(PRED) sace_53_29_ac00 YQFHHVLKRS SLTFWRNLNY IMAKMMLLMI SGLFIGF-TF FHVGVNAIGL
(PRED) sace_17_7_g0393 YQFHHVLKRS SLTFWRNLNY IMAKMMLLMI SGLFIGF-TF FHVGVNAIGL
(PRED) sace_25_7_g0388 YQFHHVLKRS SLTFWRNLNY IMAKMMLLMI SGLFIGF-TF FHVGVNAIGL
(PRED) sace_37_7_g0385 YQFHHVLKRS SLTFWRNLNY IMAKMMLLMI SGLFIGF-TF FHVGVNAIGL
(PRED) sace_9_7_g00180 YQFHHVLKRS SLTFWRNLNY IMAKMMLLMI SGLFIGF-TF FHVGVNAIGL
(PRED) sace_60_6_f0335 YQFHHVLKRS SLTFWRNLNY IMAKMMLLMI SGLFIGF-TF FHVGVNAIGL
(PRED) sace_59_336_lx0 YQFHHVLKRS SLTFWRNLNY IMAKMMLLMI SGLFIGF-TF FHVGVNAIGL
(PRED) sace_31_7_3780  YQFHHVLKRS SLTFWRNLNY IMAKMMLLMI SGLFIGF-TF FHVGVNAIGL
(PRED) sace_34_8_3770  YQFHHVLKRS SLTFWRNLNY IMAKMMLLMI SGLFIGF-TF FHVGVNAIGL
(PRED) sace_58_71_bs00 YQFHHVLKRS SLTFWRNLNY IMAKMMLLMI SGLFIGF-TF FHVGVNAIGL
(PRED) sace_7_7_g03880 YQFHHVLKRS SLTFWRNLNY IMAKMMLLMI SGLFIGF-TF FHVGVNAIGL
(PRED) sace_35_7_3840  YQFHHVLKRS SLTFWRNLNY IMAKMMLLMI SGLFIGF-TF FHVGVNAIGL
(PRED) sace_43_7_g0387 YQFHHVLKRS SLTFWRNLNY IMAKMMLLMI SGLFIGF-TF FHVGVNAIGL
```

```
(PRED) sace_57_8_h0390 YQFHHVLKRS SLTFWRNLNY IMAKMMLLMI SGLFIGF-TF FHVGVNAIGL
(PRED) sace_45_7_g0389 YQFHHVLKRS SLTFWRNLNY IMAKMMLLMI SGLFIGF-TF FHVGVNAIGL
(PRED) sace_46_8_h0391 YQFHHVLKRS SLTFWRNLNY IMAKMMLLMI SGLFIGF-TF FHVGVNAIGL
(PRED) sace_23_7_3860  YQFHHVLKRS SLTFWRNLNY IMAKMMLLMI SGLFIGF-TF FHVGVNAIGL
(PRED) sace_21_7_3790  YQFHHVLKRS SLTFWRNLNY IMAKMMLLMI SGLFIGF-TF FHVGVNAIGL
(PRED) sace_8_73_bu001 YQFHHVLKRS SLTFWRNLNY IMAKMMLLMI SGLFIGF-TF FHVGVNAIGL
(PRED) sapa_1_8_h03820 YQFWYVLRRS SLTFWRNLNY IMAKMMLLMI SGLFIGF-TF FHVGINAIGL
(PRED) sapa_21_8_h0387 YQFWYVLRRS SLTFWRNLNY IMAKMMLLMI SGLFIGF-TF FHVGINAIGL
(PRED) sapa_20_8_h0386 YQFWYVLRRS SLTFWRNLNY IMAKMMLLMI SGLFIGF-TF FHVGINAIGL
(PRED) sapa_22_8_h0390 YQFWYVLRRS SLTFWRNLNY IMAKMMLLMI SGLFIGF-TF FHVGINAIGL
(PRED) sapa_25_8_h0387 YQFWYVLRRS SLTFWRNLNY IMAKMMLLMI SGLFIGF-TF FHVGINAIGL
(PRED) sapa_6_8_3750   YQFWYVLRRS SLTFWRNLNY IMAKMMLLMI SGLFIGF-TF FHVGINAIGL
(PRED) sapa_9_8_3720   YQFWYVLRRS SLTFWRNLNY IMAKMMLLMI SGLFIGF-TF FHVGINAIGL
(PRED) sapa_19_8_h0390 YQFWYVLRRS SLTFWRNLNY IMAKMMLLMI SGLFIGF-TF FHVGINAIGL
(PRED) sapa_24_8_h0385 YQFWYVLRRS SLTFWRNLNY IMAKMMLLMI SGLFIGF-TF FHVGINAIGL
(PRED) sapa_4_8_h03850 YQFWYVLRRS SLTFWRNLNY IMAKMMLLMI SGLFIGF-TF FHVGINAIGL
(PRED) sapa_10_8_3760  YQFWYVLRRS SLTFWRNLNY IMAKMMLLMI SGLFIGF-TF FHVGINAIGL
(PRED) sapa_13_8_h0382 YQFWYVLRRS SLTFWRNLNY IMAKMMLLMI SGLFIGF-TF FHVGINAIGL
(PRED) sapa_8_8_3750   YQFWYVLRRS SLTFWRNLNY IMAKMMLLMI SGLFIGF-TF FHVGINAIGL
(PRED) sapa_11_8_h0383 YQFWYVLRRS SLTFWRNLNY IMAKMMLLMI SGLFIGF-TF FHVGINAIGL
(PRED) sapa_5_8_3700   YQFWYVLRRS SLTFWRNLNY IMAKMMLLMI SGLFIGF-TF FHVGINAIGL
(PRED) sapa_16_8_h0389 YQFWYVLRRS SLTFWRNLNY IMAKMMLLMI SGLFIGF-TF FHVGINAIGL
(PRED) sapa_17_8_3730  YQFWYVLRRS SLTFWRNLNY IMAKMMLLMI SGLFIGF-TF FHVGINAIGL
(PRED) sapa_2_8_h03860 YQFWYVLRRS SLTFWRNLNY IMAKMMLLMI SGLFIGF-TF FHVGINAIGL
(PRED) sapa_7_8_3740   YQFWYVLRRS SLTFWRNLNY IMAKMMLLMI SGLFIGF-TF FHVGINAIGL
(PRED) sapa_23_8_h0385 YQFWYVLRRS SLTFWRNLNY IMAKMMLLMI SGLFIGF-TF FHVGINAIGL
(PRED) sapa_3_8_h03890 YQFWYVLRRS SLTFWRNLNY IMAKMMLLMI SGLFIGF-TF FHVGINAIGL
(PRED) sapa_18_8_3730  YQFWYVLRRS SLTFWRNLNY IIAKMMLLMI SGLFIGF-TF FHVGINAIGL
(PRED) sami_1_14_399   YQFWYVLRRS SLTFWRNLNY VMAKMMLLIV SGLFIGF-TF FHVGANVIGL
(PRED) sace_4_8_h03690 YQFHHVLKRS SLTFWRNLNY IMAKMMLLMI SGLFIGF-TF FHVGVNAIGL
(PRED) saku_1_14_404   YQFRYVLFRS SLTFWRNLNY IMAKMMLLII SGLFIGF-TF YGVGIDAIGL
(PRED) sace_1_ynr070w  YQFHHVLKRS SLTFWRNLNY IMAKMMLLMI SGLFIGF-TF FHVGVNAIGL
(PRED) sace_49_8_h0383 YQFHHVLKRS SLTFWRNLNY IMAKMMLLMI SGLFIGF-TF FHVGVNAIGL
(PRED) saeu_1_2_b00130 YQFKRVLARS SLTFWRNLNY IMAKMMLLMV SGLFIGF-TF FHVGNSYIGL
(PRED) sauv_1_7_3      YQFKYVLVRS SLTFWRNLNY IMAKMMLLII SGLYIGF-TF FHVGTSYIGL
(PRED) sami_1_17_26    YQFRYVLSRT SLTFWRNLNY IMSKMMLLMV SGLFIGF-TF FHVDDSFIGL
(PRED) zyba_1_02055_AN YQFKYVWWRT TTMFWRDMPY LMSKMMLQVV SGLYIGW-TF FNPGKSFTGL
(PRED) zyba_1_07912    YQFKYVWWRT TTMFWRDMPY LMSKMMLQVV SGLYIGW-TF FNPGKSFTGL
(PRED) zyba_2_2_b00600 YQFKYVWWRT TTMFWRDMPY LMSKMMLQVV SGLYIGW-TF FNPGKSFTGL
(PRED) zyba_3_3_c03460 YQFKYVWWRT TTMFWRDMPY LMSKMMLQVV SGLYIGW-TF FNPGKSFTGL
(PRED) zyba_1_04634    FQFKHVWLRT NIMFWRDKQY LMSKMMLQVV SGLYIGW-TF FDPGESYTGL
(PRED) zyba_1_06675    FQFKHVWLRT NVMFWRDKQY LMSKMMLQVV SGLYIGW-TF FNPGESYTGL
(PRED) zyba_3_2_b02230 FQFKHVWLRT NVMFWRDKQY LMSKMMLQVV SGLYIGW-TF FNPGESYTGL
(PRED) zyba_2_1_a00860 FQFKHAWLRT NVMFWRDKQY LMSKMMLQVV SGLYIGW-TF FNPGESYTGL
(PRED) zyro_1_a04114g  YQFKYVWLRT NLMFWRDVPY IFSKCMLQVT AGLFIGW-TF FNPGKSYVGL
(PRED) zyro_1_b14762g  YQFKYVWMRT NLTLYRDLRY IFSKFMLQIM SGLYIGW-TF YNPGKSYVGL
(PRED) zyba_2_14_n0149 YQFKYVMNRT SIMFWRDWNY LLSKMLLQVI AGLFIGW-TF FDPGKSYSGL
(PRED) zyba_2_33_ag001 YQFRYVMDRT SIMFWRDWNY LLSKMLLQVI AGLFIGW-TF FNPGKSYTGL
(PRED) lath_1_a01914g  YQFRYVLQRT ATIFWRNSEY LIAKMMLYIS AGLFIGF-TF YNVGTSYRGL
(PRED) lawa_1_23_5161  YQFRYVLQRT ATIFWRDVNY LMAKMMLYIS TGLFIGF-TF YNVGTSYHGL
(PRED) klae_1_14_n0012 YQFKYVLQRT AAMFWRDLDY LLSKTFLFTI GGLFIGF-TF YNVGTSYIGL
(PRED) klla_1_d03432g  YQFVYVLRRT MVMFWRDVDY LMAKTMLYIS SGLFIGF-TF YNVGTSFVGL
(PRED) klma_1_1_a01880 YQFVQVLKRT MTMFWRDVDY LMAKSMLYIS SGLFIGF-TF YNVGTSYVGL
(PRED) klwi_1_33_ag001 YQFITVWKRT ATMFWRDTDY LMAKTMLYIS SGLFIGF-SY YNVGSSFIGL
(PRED) teph_1_a04220   YQFKYVQART FTIFWRDLNY LVSKLMLFMV GGLYIGF-TF FHVDNSFIGL
(PRED) vapo_1_1037_47  YQFKCVQMRA FTILWRDTNY LVSKFMLFLV TGLYIGF-TF FHVPQSYNGL
(PRED) pata_1_2_b05590 YQFRLVVTRT ATQFYRDLDY IMAKFMLFLT GALLIGF-SY WNLKHTRIGL
(PRED) wian_1_3_c04380 DQLYYVVKRN GTQFYRDPQY IGSKFFLLII GGLFVGF-TF WSLNDTIIGM
(PRED) wian_1_3_c04390 DQLYYVTRRT GIQFYRDPQY IGAKFSLMIT GGLFVGF-TF WSLKETIIGM
(PRED) wian_1_7_g01010 DQLFYVTRRT GTQFYRDPQY IGSKFALMIL GGLFVGF-TF WSLSDTVIGM
(PRED) bain_1_1_a00100 VQLKTVLGRT MIQFWRSPDY IMSKLVLMIF PGLFIGF-TF WALKNTLTGM
(PRED) bain_1_17_q0038 VQLRTVLGRT MTQFWRSPNY IMSKLVLMIF PGLFIGF-TF WALKNTLTGM
(PRED) bain_1_8_h00410 YQMKLVYHRT MLQFWRDPQY VMFKMVLLVF SGLFIGF-SF WDLPSTSTGM
(PRED) caal_1_19_5759  YQFRHVTHRT SLIFYRDPDY IAAKVFLMTI AGLFIGF-TF FGLKHTKTGA
(PRED) caal_11_25_y002 YQFRHVTHRT SLIFYRDPDY IAAKVFLMTI AGLFIGF-TF FGLKHTKTGA
(PRED) caal_4_4_d03320 YQFRHVTHRT SLIFYRDPDY IAAKVFLMTI AGLFIGF-TF FGLKHTKTGA
(PRED) caal_12_26_z005 YQFRHVTHRT SLIFYRDPDY IAAKVFLMTI AGLFIGF-TF FGLKHTKTGA
(PRED) caal_5_30_ad005 YQFRHVTHRT SLIFYRDPDY IAAKVFLMTI AGLFIGF-TF FGLKHTKTGA
(PRED) caal_8_3_c03320 YQFRHVTHRT SLIFYRDPDY IAAKVFLMTI AGLFIGF-TF FGLKHTKTGA
(PRED) caal_6_4_d03280 YQFRHVTHRT SLIFYRDPDY IAAKVFLMTI AGLFIGF-TF FGLKHTKTGA
(PRED) caal_10_3_c0334 YQFRHVTHRT SLIFYRDPDY IAAKIFLMTI AGLFIGF-TF FGLKHTKTGA
(PRED) caal_3_29_ac005 YQFRHVTHRT SLIFYRDPDY IAAKVFLMTI AGLFIGF-TF FGLKHTKTGA
(PRED) caal_2_04989    YQFRHVTHRT SLIFYRDPDY IAAKVFLMTI AGLFIGF-TF FGLKHTKTGA
(PRED) cadu_1_64350    YQFRHVTHRT SLIFYRDPDY IAAKIFLMTI AGLFIGF-TF FGLKHTKTGA
(PRED) caor_1_h02090   YQFRYVLHRN ALTFFRDPDY VMAKIFLMTI AGLFIGF-TF FGLKHTKTGA
```

```
(PRED) capa_1_600750   YQFRYVLHRN ALTFFRDPGY VMAKIFLMTV AGLFIGF-TF FGLKHTKTGA
(PRED) loel_1_04930    YQFRWVANRN ALTFYRDPDY IMAKIFLMTI SGLFIGF-TF FGLKHTKTGA
(PRED) spar_1_5_e03260 YQFKYVWQRN ALTFFRDPEY VAAKIFLMTI SGLFIGF-TF FGLKYTRTGA
(PRED) sppa_1_7_g03160 YQFRYVWHRN AMTFFRDPEY IAAKTFLMTI SGLFIGF-TF FGLKHTRAGA
(PRED) catr_1_01205    YQFRYTLQRS TTTLWRLPEY AMSKIGMMTS GGLFIGLVTF YNLKETYTGS
(PRED) catr_1_05498    YQFRITLQRS NTVLWRIPGY CVSKILVMTL SGLFIGLVTF FSLQQTYAGS
(PRED) catr_1_05971    YQFRYTLERS SKVLWRLPEY AMSKIMMMTF SGLFIGLVTF YNLKQTYTGS
(PRED) deha_1_a03696g  YQFIIVVRRN ALTFWRNPEY IMSKIMLMTM AGLFIGF-TF FGLKHSVTGM
(PRED) deha_2_5_e00720 YQFAIVVRRN ALTFWRNPEY IMSKLMLMTM AGLFIGF-TF FGLKHSVTGM
(PRED) scst_1_3_c02890 YQFWYVLERN ALTFWRDPEY IASKVFLMTM CGLFIGF-TF FGLKHTMTGA
(PRED) mebi_1_8_h00300 AEMKYVTWRT ALAFWRNPEY IMAKNMLMLI NGLFIGF-TF YDLKHTTTGM
(PRED) lakl_1_h21010g  TQFYYVYRRT VTQLWRDPGY IVSKMMLMIS AGLFIGF-TF YDVGTSFRGL
(PRED) caar_1_13_m0142 YQLRQVIIRT FTQFNRDLDY VMSKLMLMLL AGLLTGF-SF WNVKHTVIGM
(PRED) caar_1_14_n0143 YQLKHVLIRT ITQFYRDLDY IMAKFMLMLL AGLLAGF-SF WNVKHTVIGM
(PRED) hapo_1_1_a07220 TQFKNVLVRT WLQFYRDIDY VMSKFMLMLL AGLLVGF-SF WNVKHTSIGM
(PRED) ogpa_1_1_a01680 TQFKNVLVRT WLQFYRDIDY VMSKFMLMLL AGLLVGF-SF WNVKHTSIGM
(PRED) piku_1_96_cr001 VQFREVLKRT FLQFYRGLQY IMAKFILFLL AGLVVGF-TF WNSKHNASGM
(PRED) pime_1_4_d03240 IQFKEVYKRT FMQFYRSLDY VLAKFMLFVF GGLLAGF-SF WNVKHTIVGM
(PRED) pime_1_1_a12110 YQLSTVYKRT QLQVFRQVPY IMSKFMLMVI AGLLHGF-SF WNVKHSVIGL
(PRED) piku_1_227_hs00 YQFYQVNKRT YTQLYRSLPY VLPKLFLNVV GGLVTGF-SF WNAKYTIVGM
(PRED) pime_1_5_e05800 YQLYLVNKRT ALQLYRSLPY VAPKLFLYVI GSLFTGF-SF WNASHTIVGM
(PRED) pime_1_1_a07690 YQLYQVLNRT FIQLYRSLPY IMPKFMLTLL GGLIIGF-SF WNVKHTVIGM
(PRED) debr_2_5_e03380 RQLSLVLRRT WTQFYRDIDY LLSKLLLVVL GGLVHGF-SF WKVKHTTIGM
(PRED) kopa_1_2_b10040 YQYQLVLQRT AQQFFRDMEY FMAKFMLLLS GGLLIGF-SF WDVKHTIVGM
(PRED) kopa_2_7_g00500 YQYQLVLHRT AQQFFRDMEY FMAKFMLSLS GGLLIGF-SF WNVKHTIVGM
(PRED) asru_1_13_m0119 TQFKYVLRRT FTQFYRDPIY LKSKFFLLIL GGLFIGW-TF YNIRPNLSGL
(PRED) asru_1_15_o0045 TQLKHVLVRT FRQFYRDPTY VVAKFSLLIF GGLFFGW-TF FNLKTSFSGL
(PRED) wian_1_1_a02920 TQLKYVLLRT NIQFYRDPWY PFAKWVLFIF GGLFFGF-TF WDLKHTITDT
(PRED) wian_1_1_a02930 SQVKHVYFRT NLQFYRDPTY MFAKIGLYTL GGLFIGF-TF WGLKHSIIGN

                       .........1560.........1570.........1580.........1590.........1600
(PRED) asac_1_6_f03560 RNAMFAVFMS IVTSAPAMNQ IQARAIKMKD LYTVRESKSN LFHWSLLLIT
(PRED) ergo_1_abr125c  RNTMFAVFMS IITSAPAMNQ IQARAIKMKD LYIVRESKSN LFHWSLLLIT
(PRED) ercy_1_3604     QNTMFAVFMS VVMSAPAMNQ IQSRAVKSRD LYEVRESKSN MFHWSLVMIT
(PRED) cagl_1_i04862g  QNTLFAAFIS IILSAPAMNQ IQARAIAARE LFEVRESKSN MFHWSLLLIT
(PRED) kaaf_1_c00830   QNAMFAAFMS IVISAPAMNQ IQARAIASRA LFEVRESKSN MFHWSFLLIT
(PRED) kana_1_k01350   QNTLFAAFIS IILSAPSMNQ IQARAIAARE LYEVRESKSN MFHWSLLLIT
(PRED) saar_1_2_b02590 QNAMFAAFIS IILSAPAMNQ IQARAIASRE LFEVRESQSN MFHWSLVLIT
(PRED) sace_1_ydr011w  QNAMFAAFIS IILSAPAMNQ IQGRAIASRE LFEVRESQSN MFHWSLVLIT
(PRED) sace_16_1_a0238 QNAMFAAFIS IILSAPAMNQ IQGRAIASRE LFEVRESQSN MFHWSLVLIT
(PRED) sace_45_1_a0242 QNAMFAAFIS IILSAPAMNQ IQGRAIASRE LFEVRESQSN MFHWSLVLIT
(PRED) sace_48_1_a0238 QNAMFAAFIS IILSAPAMNQ IQGRAIASRE LFEVRESQSN MFHWSLVLIT
(PRED) sace_60_4_d0244 QNAMFAAFIS IILSAPAMNQ IQGRAIASRE LFEVRESQSN MFHWSLVLIT
(PRED) sace_52_1_a0240 QNAMFAAFIS IILSAPAMNQ IQGRAIASRE LFEVRESQSN MFHWSLVLIT
(PRED) sace_46_1_a0240 QNAMFAAFIS IILSAPAMNQ IQGRAIASRE LFEVRESQSN MFHWSLVLIT
(PRED) sace_25_1_a0240 QNAMFAAFIS IILSAPAMNQ IQGRAIASRE LFEVRESQSN MFHWSLVLIT
(PRED) sace_24_1_2300  QNAMFAAFIS IILSAPAMNQ IQGRAIASRE LFEVRESQSN MFHWSLVLIT
(PRED) sace_47_1_a0240 QNAMFAAFIS IILSAPAMNQ IQGRAIASRE LFEVRESQSN MFHWSLVLIT
(PRED) sace_7_1_a02410 QNAMFAAFIS IILSAPAMNQ IQGRAIASRE LFEVRESQSN MFHWSLVLIT
(PRED) sace_59_110_df0 QNAMFAAFIS IILSAPAMNQ IQGRAIASRE LFEVRESQSN MFHWSLVLIT
(PRED) sace_56_1_a0202 QNAMFAAFIS IILSAPAMNQ IQGRAIASRE LFEVRESQSN MFHWSLVLIT
(PRED) sace_40_1_a0239 QNAMFAAFIS IILSAPAMNQ IQGRAIASRE LFEVRESQSN MFHWSLVLIT
(PRED) sace_15_1_a0242 QNAMFAAFIS IILSAPAMNQ IQGRAIASRE LFEVRESQSN MFHWSLVLIT
(PRED) sace_37_1_a0243 QNAMFAAFIS IILSAPAMNQ IQGRAIASRE LFEVRESQSN MFHWSLVLIT
(PRED) sace_9_1_a02440 QNAMFAAFIS IILSAPAMNQ IQGRAIASRE LFEVRESQSN MFHWSLVLIT
(PRED) sace_22_1_2300  QNAMFAAFIS IILSAPAMNQ IQGRAIASRE LFEVRESQSN MFHWSLVLIT
(PRED) sace_29_1_2290  QNAMFAAFIS IILSAPAMNQ IQGRAIASRE LFEVRESQSN MFHWSLVLIT
(PRED) sace_34_1_2320  QNAMFAAFIS IILSAPAMNQ IQGRAIASRE LFEVRESQSN MFHWSLVLIT
(PRED) sace_58_25_y007 QNAMFAAFIS IILSAPAMNQ IQGRAIASRE LFEVRESQSN MFHWSLVLIT
(PRED) sace_23_1_2290  QNAMFAAFIS IILSAPAMNQ IQGRAIASRE LFEVRESQSN MFHWSLVLIT
(PRED) sace_6_120_dp00 QNAMFAAFIS IILSAPAMNQ IQGRAIASRE LFEVRESQSN MFHWSLVLIT
(PRED) sace_57_1_a0241 QNAMFAAFIS IILSAPAMNQ IQGRAIASRE LFEVRESQSN MFHWSLVLIT
(PRED) sace_17_1_a0241 QNAMFAAFIS IILSAPAMNQ IQGRAIASRE LFEVRESQSN MFHWSLVLIT
(PRED) sace_21_1_2310  QNAMFAAFIS IILSAPAMNQ IQGRAIASRE LFEVRESQSN MFHWSLVLIT
(PRED) sace_49_1_a0246 QNAMFAAFIS IILSAPAMNQ IQGRAIASRE LFEVRESQSN MFHWSLVLIT
(PRED) sace_8_2_b02430 QNAMFAAFIS IILSAPAMNQ IQGRAIASRE LFEVRESQSN MFHWSLVLIT
(PRED) sace_31_1_2300  QNAMFAAFIS IILSAPAMNQ IQGRAIASRE LFEVRESQSN MFHWSLVLIT
(PRED) sace_50_1_a0241 QNAMFAAFIS IILSAPAMNQ IQGRAIASRE LFEVRESQSN MFHWSLVLIT
(PRED) sace_4_1_a02360 QNAMFAAFIS IILSAPAMNQ IQXRAIASRE LFEVRESQSN MFHWSLVLIT
(PRED) sace_2_1_a02390 QNAMFAAFIS IILSAPAMNQ IQGRAIASRE LFEVRESQSN MFHWSLVLIT
(PRED) sace_5_33_ag005 QNAMFAAFIS IILSAPAMNQ IQGRAIASRE LFEVRESQSN MFHWSLVLIT
(PRED) sapa_11_1_a0247 QNAMFAAFIS IILSAPAMNQ IQARAIASRE LFEVRESQSN MFHWSLVLIT
(PRED) sapa_25_1_a0246 QNAMFAAFIS IILSAPAMNQ IQARAIASRE LFEVRESQSN MFHWSLVLIT
(PRED) sapa_4_1_a02470 QNAMFAAFIS IILSAPAMNQ IQARAIASRE LFEVRESQSN MFHWSLVLIT
(PRED) sapa_5_1_2350   QNAMFAAFIS IILSAPAMNQ IQARAIASRE LFEVRESQSN MFHWSLVLIT
```

(PRED)	sapa_9_1_2360	QNAMFAAFIS	IILSAPAMNQ	IQARAIASRE	LFEVRESQSN	MFHWSLVLIT
(PRED)	sapa_14_1_a0244	QNAMFAAFIS	IILSAPAMNQ	IQARAIASRE	LFEVRESQSN	MFHWSLVLIT
(PRED)	sapa_8_1_2350	QNAMFAAFIS	IILSAPAMNQ	IQARAIASRE	LFEVRESQSN	MFHWSLVLIT
(PRED)	sapa_17_1_2380	QNAMFAAFIS	IILSAPAMNQ	IQARAIASRE	LFEVRESQSN	MFHWSLVLIT
(PRED)	sapa_7_1_2370	QNAMFAAFIS	IILSAPAMNQ	IQARAIASRE	LFEVRESQSN	MFHWSLVLIT
(PRED)	sapa_2_1_a02460	QNAMFAAFIS	IILSAPAMNQ	IQARAIASRE	LFEVRESQSN	MFHWSLVLIT
(PRED)	sapa_23_1_a0248	QNAMFAAFIS	IILSAPAMNQ	IQARAIASRE	LFEVRESQSN	MFHWSLVLIT
(PRED)	sapa_3_1_a02470	QNAMFAAFIS	IILSAPAMNQ	IQARAIASRE	LFEVRESQSN	MFHWSLVLIT
(PRED)	sapa_18_1_2390	QNAMFTAFIS	IILSAPAMNQ	IQARAIASRE	LFEVRESKSN	MFHWSLVLIT
(PRED)	sami_1_4_244	QNAMFAAFIS	IILSAPAMNQ	IQGRAIASRE	LFEVRESQSN	MFHWSLVLIT
(PRED)	saku_1_4_262	QNAMFAAFIS	IILSAPAMNQ	IQARAIASRE	LFEVRESQSN	MFHWSLVLIT
(PRED)	saba_1_58_bf002	QNAMFAAFIS	IILSAPAMNQ	IQGRAIASRE	LFEVRESQSN	MFHWSLVLIT
(PRED)	saeu_1_4_d02400	QNAMFAAFIS	IILSAPAMNQ	IQGRAIASRE	LFEVRESQSN	MFHWSLVLIT
(PRED)	naca_1_e01640	QNAMFAAFIS	IVVSAPAMNQ	IQARAIAARD	LYEVRESKSN	MFHWSLLLVT
(PRED)	nada_1_g01850	QNAMFAAFIS	IIMAAPAMNQ	IQARAIAARD	LFEVRESKSN	MFHWSLLLIT
(PRED)	naca_1_e01630	QNAMFTAFIS	IIVSAPAMNQ	MQARAIAARD	LYEVRESKSN	MFHWSLLLIT
(PRED)	nada_1_g01840	QNAMFAAFIS	IIMSAPAMNQ	IQGRAIAARD	LYEVRESKSN	MFHWSLLLIT
(PRED)	kaaf_1_c00820	QNALFAAFIS	IVLSAPAMNQ	IQARAIASRE	LFEVRESKSN	MFHWSLLLIT
(PRED)	teph_1_m00640	QHALFAAFMA	IVISAPSMNQ	IQARAIASRE	LFEVRESKSN	MFHWSLVLIT
(PRED)	vapo_1_1036_28	QNALFATFMA	IVLSAPSMNQ	IQARAIASRE	LFEVRESKSN	MFHWSLVMVT
(PRED)	tebl_1_i01760	QNSLFSAFVS	IILSAPLMNQ	IQANAIQSRE	LFEVRESKSN	MFHWSFILVT
(PRED)	tode_1_d04040	QNALFAAFIS	IVLSAPAMNQ	IQARALASRE	LFEVRESKSN	MFHWSLLLIT
(PRED)	naca_1_e01650	QNAMFGAFMS	LVMAAPVIMQ	MMGFALASRE	LFETRESKSN	MFHWSLLLVT
(PRED)	tebl_1_g02820	QSSLFAAFMS	IVQSAPAMNQ	IQARAIAQRD	LFEVRESKSN	TFHWSLIIIT
(PRED)	lakl_1_c11616g	QNAMFAAFMA	IVLSAPAMNQ	IQARALASRE	LFEVRESKSN	MFHWSFLLLT
(PRED)	saar_1_8_h03780	QNTLFACFMA	IVISAPATNQ	IQEHATAAKE	LYEVRESKSN	MFHWSLLLLT
(PRED)	sace_14_7_g0015	QNSLFACFMA	IVISAPATNQ	IQERATVAKE	LYEVRESKSN	MFHWSLLLIT
(PRED)	sace_15_7_g0387	QNSLFACFMA	IVISAPATNQ	IQERATVAKE	LYEVRESKSN	MFHWSLLLIT
(PRED)	sace_24_8_3780	QNSLFACFMA	IVISAPATNQ	IQERATVAKE	LYEVRESKSN	MFHWSLLLIT
(PRED)	sace_40_8_h0383	QNSLFACFMA	IVISAPATNQ	IQERATVAKE	LYEVRESKSN	MFHWSLLLIT
(PRED)	sace_6_169_fm00	QNSLFACFMA	IVISAPATNQ	IQERATVAKE	LYEVRESKSN	MFHWSLLLIT
(PRED)	sace_19_7_3840	QNSLFACFMA	IVISAPATNQ	IQERATVAKE	LYEVRESKSN	MFHWSLLLIT
(PRED)	sace_32_7_3770	QNSLFACFMA	IVISAPATNQ	IQERATVAKE	LYEVRESKSN	MFHWSLLLIT
(PRED)	sace_56_17_q011	QNSLFACFMA	IVISAPATNQ	IQERATVAKE	LYEVRESKSN	MFHWSLLLIT
(PRED)	sace_5_78_bz001	QNSLFACFMA	IVISAPATNQ	IQERATVAKE	LYEVRESKSN	MFHWSLLLIT
(PRED)	sace_2_8_h03860	QNSLFACFMA	IVISAPATNQ	IQERATVAKE	LYEVRESKSN	MFHWSLLLIT
(PRED)	sace_53_29_ac00	QNSLFACFMA	IVISAPATNQ	IQERATVAKE	LYEVRESKSN	MFHWSLLLIT
(PRED)	sace_17_7_g0393	QNSLFACFMA	IVISAPATNQ	IQERATVAKE	LYEVRESKSN	MFHWSLLLIT
(PRED)	sace_25_7_g0388	QNSLFACFMA	IVISAPATNQ	IQERATVAKE	LYEVRESKSN	MFHWSLLLIT
(PRED)	sace_37_7_g0385	QNSLFACFMA	IVISAPATNQ	IQERATVAKE	LYEVRESKSN	MFHWSLLLIT
(PRED)	sace_9_7_g00180	QNSLFACFMA	IVISAPATNQ	IQERATVAKE	LYEVRESKSN	MFHWSLLLIT
(PRED)	sace_60_6_f0335	QNSLFACFMA	IVISAPATNQ	IQERATVAKE	LYEVRESKSN	MFHWSLLLIT
(PRED)	sace_59_336_lx0	QNSLFACFMA	IVISAPATNQ	IQERATVAKE	LYEVRESKSN	MFHWSLLLIT
(PRED)	sace_31_7_3780	QNSLFACFMA	IVISAPATNQ	IQERATVAKE	LYEVRESKSN	MFHWSLLLIT
(PRED)	sace_34_8_3770	QNSLFACFMA	IVISAPATNQ	IQERATVAKE	LYEVRESKSN	MFHWSLLLIT
(PRED)	sace_58_71_bs00	QNSLFACFMA	IVISAPATNQ	IQERATVAKE	LYEVRESKSN	MFHWSLLLIT
(PRED)	sace_7_7_g03880	QNSLFACFMA	IVISAPATNQ	IQERATVAKE	LYEVRESKSN	MFHWSLLLIT
(PRED)	sace_35_7_3840	QNSLFACFMA	IVISAPATNQ	IQERATVAKE	LYEVRESKSN	MFHWSLLLIT
(PRED)	sace_43_7_g0387	QNSLFACFMA	IVISAPATNQ	IQERATVAKE	LYEVRESKSN	MFHWSLLLIT
(PRED)	sace_57_8_h0390	QNSLFACFMA	IVISAPATNQ	IQERATVAKE	LYEVRESKSN	MFHWSLLLIT
(PRED)	sace_45_7_g0389	QNSLFACFMA	IVISAPATNQ	IQERATVAKE	LYEVRESKSN	MFHWSLLLIT
(PRED)	sace_46_8_h0391	QNSLFACFMA	IVISAPATNQ	IQERATVAKE	LYEVRESKSN	MFHWSLLLIT
(PRED)	sace_23_7_3860	QNNLFACFMA	IVISAPATNQ	IQERATVAKE	LYEVRESKSN	MFHWSLLLIT
(PRED)	sace_21_7_3790	QNSLFACFMA	IVISAPATNQ	IQERATVAKE	LYEVRESKSN	MFHWSLLLIT
(PRED)	sace_8_73_bu001	QNSLFACFMA	IVISAPATNQ	IQERATVAKE	LYEVRESKSN	MFHWSLLLIT
(PRED)	sapa_1_8_h03820	QNSLFACFMA	IVISAPATNQ	IQERATAAKE	LYEVRESKSN	MFHWSLLLIT
(PRED)	sapa_21_8_h0387	QNSLFACFMA	IVISAPATNQ	IQERATAAKE	LYEVRESKSN	MFHWSLLLIT
(PRED)	sapa_20_8_h0386	QNSLFACFMA	IVISAPATNQ	IQERATAAKE	LYEVRESKSN	MFHWSLLLIT
(PRED)	sapa_22_8_h0390	QNSLFACFMA	IVISAPATNQ	IQERATAAKE	LYEVRESKSN	MFHWSLLLIT
(PRED)	sapa_25_8_h0387	QNSLFACFMA	IVISAPATNQ	IQERATAAKE	LYEVRESKSN	MFHWSLLLIT
(PRED)	sapa_6_8_3750	QNSLFACFMA	IVISAPATNQ	IQERATAAKE	LYEVRESKSN	MFHWSLLLIT
(PRED)	sapa_9_8_3720	QNSLFACFMA	IVISAPATNQ	IQERATAAKE	LYEVRESKSN	MFHWSLLLIT
(PRED)	sapa_19_8_h0390	QNSLFACFMA	IVISAPATNQ	IQERATAAKE	LYEVRESKSN	MFHWSLLLIT
(PRED)	sapa_24_8_h0385	QNSLFACFMA	IVISAPATNQ	IQERATAAKE	LYEVRESKSN	MFHWSLLLIT
(PRED)	sapa_4_8_h03850	QNSLFACFMA	IVISAPATNQ	IQERATAAKE	LYEVRESKSN	MFHWSLLLIT
(PRED)	sapa_10_8_3760	QNSLFACFMA	IVISAPATNQ	IQERATAAKE	LYEVRESKSN	MFHWSLLLIT
(PRED)	sapa_13_8_h0382	QNSLFACFMA	IVISAPATNQ	IQERATAAKE	LYEVRESKSN	MFHWSLLLIT
(PRED)	sapa_8_8_3750	QNSLFACFMA	IVISAPATNQ	IQERATAAKE	LYEVRESKSN	MFHWSLLLIT
(PRED)	sapa_11_8_h0383	QNSLFACFMA	IVISAPATNQ	IQERATAAKE	LYEVRESKSN	MFHWSLLLIT
(PRED)	sapa_5_8_3700	QNSLFACFMA	IVISAPATNQ	IQERATAAKE	LYEVRESKSN	MFHWSLLLIT
(PRED)	sapa_16_8_h0389	QNSLFACFMA	IVISAPATNQ	IQERATAAKE	LYEVRESKSN	MFHWSLLLIT
(PRED)	sapa_17_8_3730	QNSLFACFMA	IVISAPATNQ	IQERATAAKE	LYEVRESKSN	MFHWSLLLIT
(PRED)	sapa_2_8_h03860	QNSLFACFMA	IVISAPATNQ	IQERATAAKE	LYEVRESKSN	MFHWSLLLIT
(PRED)	sapa_7_8_3740	QNSLFACFMA	IVISAPATNQ	IQERATAAKE	LYEVRESKSN	MFHWSLLLIT
(PRED)	sapa_23_8_h0385	QNSLFACFMA	IVISAPATNQ	IQERATAAKE	LYEVRESKSN	MFHWSLLLIT

(PRED)	sapa_3_8_h03890	QNSLFACFMA	IVISAPATNQ	IQERATAAKE	LYEVRESKSN	MFHWSLLLIT
(PRED)	sapa_18_8_3730	QNSLLACFMA	IVISAPATNQ	IQERATAAKE	LYEVRESKSN	MFHWSLLLIT
(PRED)	sami_1_14_399	QNTLFACFMA	IVISAPATNQ	IQERATAAKE	LYEVRESKSN	MFHWSLLLIT
(PRED)	sace_4_8_h03690	QNSLFACFMA	IVISAPATNQ	IQERATVAKE	LYEVRESKSN	MFHWSLLLIT
(PRED)	saku_1_14_404	QNSLFACFMA	IVISAPATNQ	IQERATAAKE	LYEVRESKSN	MFHWSLLLFT
(PRED)	sace_1_ynr070w	QNSLFACFMA	IVISAPATNQ	IQERATVAKE	LYEVRESKSN	MFHWSLLLIT
(PRED)	sace_49_8_h0383	QNSLFACFMA	IVISAPATNQ	IQERATVAKE	LYEVRESKSN	MFHWSLLLIT
(PRED)	saeu_1_2_b00130	QNTMFACFMA	IVISAPATNQ	IQDRATAAKE	LYEVRESKSN	MFHWSLLLIT
(PRED)	sauv_1_7_3	QNTMFACFMA	IVISAPATNQ	IQDRATSAKE	LYEIRESKSN	MFHWSLLLIT
(PRED)	sami_1_17_26	QNTMFACFMA	IVISAPATNQ	IQARAIAAKE	LYEVRESKSN	MFHWSLLLIT
(PRED)	zyba_1_02055_AN	QDTLFAAFMS	VIVSAPSMNQ	IQARALSSRE	SFEVRESKSN	MFHWSLLLIT
(PRED)	zyba_1_07912	QDTLFAAFMS	VIVSAPTMNQ	IQARALSSRE	SFEVRESKSN	MFHWSLLLIT
(PRED)	zyba_2_2_b00600	QDTLFAAFMS	VIVSAPSMNQ	IQARALSSRE	SFEVRESKSN	MFHWSLLLIT
(PRED)	zyba_3_3_c03460	QDTLFAAFMS	VIVSAPTMNQ	IQARALSSRE	SFEVRESKSN	MFHWSLLLIT
(PRED)	zyba_1_04634	QDTTFAAFMA	VIVSAPSMKQ	IQSRALASRE	LFEVRESKSN	MFHWSLLLFT
(PRED)	zyba_1_06675	QDTTFAAFMA	VIVSAPSMKQ	IQARALASRE	LFEVRESKSN	MFHWSLLLFT
(PRED)	zyba_3_2_b02230	QDTTFAAFMA	VIVSAPSMKQ	IQARALASRE	LFEVRESKSN	MFHWSLLLFT
(PRED)	zyba_2_1_a00860	QDTTFAAFMA	VIVSAPSMKQ	IQARALASRE	LFEVRESKSN	MFHWSLLLFT
(PRED)	zyro_1_a04114g	QDTMFAAFIS	VIVAAPSMNQ	IQARAISSRE	LFEVRESKSN	MFHWSLLLIT
(PRED)	zyro_1_b14762g	QDTLFAAFVS	VIVSAPSMKQ	IQARALASRS	LFEVRESKSN	MFHWSLLLLT
(PRED)	zyba_2_14_n0149	QDTLFAAFMA	VVLSAPTMNQ	IQARAMASRE	LFEVRESKSN	MFHWSLLLLT
(PRED)	zyba_2_33_ag001	QDTMFAAFMA	VILSAPTMNQ	IQARAMASRE	LFEVRESKSN	MFHWSLLLLT
(PRED)	lath_1_a01914g	QNTMFAAFMA	LIVSAPAMNQ	IQARAIASRE	LFEVRESKSN	TFHWIFLLLT
(PRED)	lawa_1_23_5161	QNAMFAAFMA	LIVSAPAMNQ	IQARAISSRE	LFEVRESKSN	TFHWAFLLVT
(PRED)	klae_1_14_n0012	QNAMFAAFMA	VIVSAPAMNQ	IQARALQSRE	LFEVRESKSN	MFHWSHLLTT
(PRED)	klla_1_d03432g	QNAMFAAFMA	CIVSAPAMNQ	IQARALQSRE	LYEVRESRSN	MFHWSCMLFS
(PRED)	klma_1_1_a01880	QNAMFAAFMA	CIVSAPAMNQ	IQSRALQSRE	LYEVRESRSN	MFHWSCLLIT
(PRED)	klwi_1_33_ag001	QNSMFAAFIA	VIVSAPAMNQ	IQSRALQSRE	LFEVRESKSN	MFHWSCLLLT
(PRED)	teph_1_a04220	QNSLFAAFIA	IILSAPSINQ	IQSRAIISRD	LFEVRESKSN	MFHWSLLVLT
(PRED)	vapo_1_1037_47	QGALFAAFLT	LITSAPVMNQ	IQARAIEARE	LYEVRESKSN	VFHWSLLLLC
(PRED)	pata_1_2_b05590	QNCMFAVFIS	MILSAPLGNQ	VQARAIASRE	LFEVRESKSN	TFHWSTLLIA
(PRED)	wian_1_3_c04380	QNGMFAVFLA	IIISAPATNQ	IQERAVASRE	LFEVRESKSN	TFHWSTLLIA
(PRED)	wian_1_3_c04390	QNGMFVVFMA	IIISAPAINQ	IQERAVASRE	LFEVRESKSN	TFHWSTLLIA
(PRED)	wian_1_7_g01010	QNGMFVVFNA	IIISAPAINQ	IQERAVASRE	LFEVRESKSN	TFHWSTLLIS
(PRED)	bain_1_1_a00100	QNAVFVAFLS	VVTSTPLINQ	IQDRAYASRE	LFEVRESKSN	TYHWSTLLLS
(PRED)	bain_1_17_q0038	QNAVFVAFLS	VVTSTPLINQ	IQGRAYASRE	LFEVRESKSN	TYHWSTLLLS
(PRED)	bain_1_8_h00410	QNCMFAVYLS	SMLCVPLINQ	IQERAFVNRE	LYEVRESKSH	TFHWATLLFS
(PRED)	caal_1_19_5759	QNGMFCAFLS	CVIAAPLINQ	MLEKA-GSRD	IYEVREKLSN	TYHWSLLILP
(PRED)	caal_11_25_y002	QNGMFCAFLS	CVIAAPLINQ	MLEKA-GSRD	IYEVREKLSN	TYHWSLLILP
(PRED)	caal_4_4_d03320	QNGMFCAFLS	CVIAAPLINQ	MLEKA-GSRD	IYEVREKLSN	TYHWSLLILP
(PRED)	caal_12_26_z005	QNGMFCAFLS	CVIAAPLINQ	MLEKA-GSRD	IYEVREKLSN	TYHWSLLILP
(PRED)	caal_5_30_ad005	QNGMFCAFLS	CVIAAPLINQ	MLEKA-GSRD	IYEVREKLSN	TYHWSLLILP
(PRED)	caal_8_3_c03320	QNGMFCAFLS	CVIAAPLINQ	MLEKA-GSRD	IYEVREKLSN	TYHWSLLILP
(PRED)	caal_6_4_d03280	QNGMFCAFLS	CVIAAPLINQ	MLEKA-GSRD	IYEVREKLSN	TYHWSLLILP
(PRED)	caal_10_3_c0334	QNGMFCAFLS	CVIAAPLINQ	MLEKA-GSRD	IYEVREKLSN	TYHWSLLILP
(PRED)	caal_3_29_ac005	QNGMFCAFLS	CVIAAPLINQ	MLEKA-VSRD	IYEVREKLSN	TYHWSLLILP
(PRED)	caal_2_04989	QNGMFCAFLS	CVIAAPLINQ	MLEKA-GSRD	IYEVREKLSN	TYHWSLLILP
(PRED)	cadu_1_64350	QNGMFCAFLS	CVIAAPLINQ	MLEKA-ASRD	IYEVREKLSN	TYHWSLLILP
(PRED)	caor_1_h02090	QNGMFCSFLT	VVVSAPVINQ	IQEKAINGRD	LYEVREKLSN	TYHWSLMILC
(PRED)	capa_1_600750	QNGMFCSFLT	VVISAPVINQ	IQEKAINGRD	LFEVREKLSN	TYHWSLMILC
(PRED)	loel_1_04930	QNGMFCAFLS	VVVSAPVINQ	IQEKAIGGRD	LFEVREKLSN	TYHWSLMIFC
(PRED)	spar_1_5_e03260	QNGMFCAFLS	VVVSAPVINQ	IQEKAYAGRE	LFEVREKLSN	TYHWSLLILN
(PRED)	sppa_1_7_g03160	QNGMFCAFLS	VVVSAPVINQ	IQEKAYAGRE	LFEVREKLSN	TYHWSLLIIT
(PRED)	catr_1_01205	QNGLFCGFLT	VVVAAPIANM	LMERFSYLEL	HLKQGTAV-Y	TYHWSLLIYV
(PRED)	catr_1_05498	RNGMFCGFLS	VVVVAPIANM	LMERYSYARA	IFEARESLSN	TYHWSLLVIS
(PRED)	catr_1_05971	RNGLFCAFLS	VVTAAPIANM	LMERYSYSRA	TFEARESLSN	TYHWSLLIVT
(PRED)	deha_1_a03696g	QNGMFAGFLA	VVVSAPVINQ	IQEHAIKGRD	LFEGREKLSN	TYHWSLMVIA
(PRED)	deha_2_5_e00720	QNGMFAGFLA	VVVSAPVINQ	IQEHAIKGRD	LFEGREKLSN	TYHWSLMIIA
(PRED)	scst_1_3_c02890	QNGMFCSFLA	VVVSAPVINQ	IQEKAIKGRD	LFEGREKLSN	TYHWSLIMIC
(PRED)	mebi_1_8_h00300	QNGLFCGFLS	MVVSAPLINQ	IQAQALDIRE	TYEGREKMSN	TYRWWVMVLS
(PRED)	lakl_1_h21010g	QNAMFAVFMS	IIVAAPLMNQ	IQSRAIQARE	LFEARESKSN	TFHWSCLLLS
(PRED)	caar_1_13_m0142	QNLLFAAFMA	LIVSAPIINQ	VQAKAIQSRE	LYEVRESKSN	TFHWSCLLIA
(PRED)	caar_1_14_n0143	QNLMFAAFMA	IIVCAPLTNQ	IQGRAIESRE	LFEVRESKSN	TFHWSCLLIS
(PRED)	hapo_1_1_a07220	QNLMFACFMA	IVVCAPLTNQ	IQERAIKSRE	LFEVRESKSN	TFHWSCLLLS
(PRED)	ogpa_1_1_a01680	QNLMFACFMA	LVVCAPLTNQ	IQERAIKSRE	LFEVRESKSN	TFHWSCLLLS
(PRED)	piku_1_96_cr001	QDVMFACLLA	VIIASPMMNQ	IQAKAIASRQ	LYEVRESKSN	TFHWSLLMIS
(PRED)	pime_1_4_d03240	QNVMFACFLA	VVVSSPLINQ	IQEKAIAARE	LYEVRESKSN	TFHWSLLMLS
(PRED)	pime_1_1_a12110	QNITFACFIA	IVLSNPLINQ	IQEQAIAGRE	LFEVRESKSN	TFHWSCMIIA
(PRED)	piku_1_227_hs00	QNVMFASFLT	LVVSAPIMNQ	IQTYAIASRE	LFEVRESKSN	TFHWSCLLIA
(PRED)	pime_1_5_e05800	QDVMFATFLT	LVVSAPIMNQ	IQTYAIASRE	LFEVRESKSN	TFHWSCLLIA
(PRED)	pime_1_1_a07690	QNVMFANFLT	LIISAPIMNQ	MQTKAISSRE	LYEVRESKSK	TFHWSCLLIS
(PRED)	debr_2_5_e03380	QNTMFANFMA	AVIAAPLINQ	IQARAIQSRE	LFEVRESKSS	TFHWSTLLLS
(PRED)	kopa_1_2_b10040	QNAMFAVFSA	MILSAPLSNQ	IQSKAIASRE	LYEARESKSN	TFHWSALLLS
(PRED)	kopa_2_7_g00500	QNAMFAVFSA	MILSAPLSNQ	IQSKAIASRE	LYEARESKSN	TFHWSALLLS
(PRED)	asru_1_13_m0119	QNCLFAVFMS	LTISAPLINQ	IQERAIASRE	LFEARESASN	TYHWSTLLFS

```
(PRED) asru_1_15_o0045 QNAVFCVFLS IGISFPLIGQ IQERAIDARE LFEARESSSN TYHWSTLLIS
(PRED) wian_1_1_a02920 QNSLFVVFLS LILHNPLVNQ MQAKSSKARA VYEARERMSK TFHWSALLLS
(PRED) wian_1_1_a02930 QNALFVAFLA VIISNPLLNQ IQERSMKSRV VYEARERMSN TFHWTALMVS

                       ......... 1610......... 1620......... 1630......... 1640......... 1650
(PRED) asac_1_6_f03560 QYLAELPY-Q FVFSTIYYVC MFFPMKLGLP ARE-NGVFFV NFCIVFQAYF
(PRED) ergo_1_abr125c  QYLAELPY-Q FLFSTIYFLC MFFPMKLGLS ARE-NGIFFL NFCIVFQAYF
(PRED) ercy_1_3604     EYLAELPY-Q FFFSTIFYCC MFFPLKSHAS SYR-AGVFFL NYVVIFQLYF
(PRED) cagl_1_i04862g  QYLSEIPY-H FLFSAIFFVS SYFPLRTHFQ ASA-SAVYYL NYSIMFQLYY
(PRED) kaaf_1_c00830   QYLCEIPY-H FLFSTIFFVS SYFPLRNHFG SSF-SGVYFL NYSIMFQLYY
(PRED) kana_1_k01350   QYLSEVPY-H LFFSAIFFVS SYFPLRNHFS AKY-SAVYYL NYSIMFQFYY
(PRED) saar_1_2_b02590 QYLSELPY-H LLFSTIFFVS SYFPLRIFFE ASR-SAVYFL NYCIMFQLYY
(PRED) sace_1_ydr011w  QYLSELPY-H LFFSTIFFVS SYFPLRIFFE ASR-SAVYFL NYCIMFQLYY
(PRED) sace_16_1_a0238 QYLSELPY-H LFFSTIFFVS SYFPLRIFFE ASR-SAVYFL NYCIMFQLYY
(PRED) sace_45_1_a0242 QYLSELPY-H LFFSTIFFVS SYFPLRIFFE ASR-SAVYFL NYCIMFQLYY
(PRED) sace_48_1_a0238 QYLSELPY-H LFFSTIFFVS SYFPLRIFFE ASR-SAVYFL NYCIMFQLYY
(PRED) sace_60_4_d0244 QYLSELPY-H LFFSTIFFVS SYFPLRIFFE ASR-SAVYFL NYCIMFQLYY
(PRED) sace_52_1_a0240 QYLSELPY-H LFFSTIFFVS SYFPLRIFFE ASR-SAVYFL NYCIMFQLYY
(PRED) sace_46_1_a0240 QYLSELPY-H LFFSTIFFVS SYFPLRIFFE ASR-SAVYFL NYCIMFQLYY
(PRED) sace_25_1_a0240 QYLSELPY-H LFFSTIFFVS SYFPLRIFFE ASR-SAVYFL NYCIMFQLYY
(PRED) sace_24_1_2300  QYLSELPY-H LFFSTIFFVS SYFPLRIFFE ASR-SAVYFL NYCIMFQLYY
(PRED) sace_47_1_a0240 QYLSELPY-H LFFSTIFFVS SYFPLRIFFE ASR-SAVYFL NYCIMFQLYY
(PRED) sace_7_1_a02410 QYLSELPY-H LFFSTIFFVS SYFPLRIFFE ASR-SAVYFL NYCIMFQLYY
(PRED) sace_59_110_df0 QYLSELPY-H LFFSTIFFVS SYFPLRIFFE ASR-SAVYFL NYCIMFQLYY
(PRED) sace_56_1_a0202 QYLSELPY-H LFFSTIFFVS SYFPLRIFFE ASR-SAVYFL NYCIMFQLYY
(PRED) sace_40_1_a0239 QYLSELPY-H LFFSTIFFVS SYFPLRIFFE ASR-SAVYFL NYCIMFQLYY
(PRED) sace_15_1_a0242 QYLSELPY-H LFFSTIFFVS SYFPLRIFFE ASR-SAVYFL NYCIMFQLYY
(PRED) sace_37_1_a0243 QYLSELPY-H LFFSTIFFVS SYFPLRIFFE ASR-SAVYFL NYCIMFQLYY
(PRED) sace_9_1_a02440 QYLSELPY-H LFFSTIFFVS SYFPLRIFFE ASR-SAVYFL NYCIMFQLYY
(PRED) sace_22_1_2300  QYLSELPY-H LFFSTIFFVS SYFPLRIFFE ASR-SAVYFL NYCIMFQLYY
(PRED) sace_29_1_2290  QYLSELPY-H LFFSTIFFVS SYFPLRIFFE ASR-SAVYFL NYCIMFQLYY
(PRED) sace_34_1_2320  QYLSELPY-H LFFSTIFFVS SYFPLRIFFE ASR-SAVYFL NYCIMFQLYY
(PRED) sace_58_25_y007 QYLSELPY-H LFFSTIFFVS SYFPLRIFFE ASR-SAVYFL NYCIMFQLYY
(PRED) sace_23_1_2290  QYLSELPY-H LFFSTIFFVS SYFPLRIFFE ASR-SAVYFL NYCIMFQLYY
(PRED) sace_6_120_dp00 QYLSELPY-H LFFSTIFFVS SYFPLRIFFE ASR-SAVYFL NYCIMFQLYY
(PRED) sace_57_1_a0241 QYLSELPY-H LFFSTIFFVS SYFPLRIFFE ASR-SAVYFL NYCIMFQLYY
(PRED) sace_17_1_a0241 QYLSELPY-H LFFSTIFFVS SYFPLRIFFE ASR-SAVYFL NYCIMFQLYY
(PRED) sace_21_1_2310  QYLSELPY-H LFFSTIFFVS SYFPLRIFFE ASR-SAVYFL NYCIMFQLYY
(PRED) sace_49_1_a0246 QYLSELPY-H LFFSTIFFVS SYFPLRIFFE ASR-SAVYFL NYCIMFQLYY
(PRED) sace_8_2_b02430 QYLSELPY-H LFFSTIFFVS SYFPLRIFFE ASR-SAVYFL NYCIMFQLYY
(PRED) sace_31_1_2300  QYLSELPY-H LFFSTIFFVS SYFPLRIFFE ASR-SAVYFL NYCIMFQLYY
(PRED) sace_50_1_a0241 QYLSELPY-H LFFSTIFFVS SYFPLRIFFE ASR-SAVYFL NYCIMFQLYY
(PRED) sace_4_1_a02360 QYLSELPY-H LFFSTIFFVS SYXPLRIFFE ASR-SAVYFL NYCIMFQLYY
(PRED) sace_2_1_a02390 QYLSELPYLF IFFRTIFFVS SYFPLRIFFE ASR-SAVYFL NYCIMFQLYY
(PRED) sace_5_33_ag005 QYLSELPY-H LFFSTIFFVS SXFPLRIFFE ASR-SAVYFL NYCIMFQLYY
(PRED) sapa_11_1_a0247 QYLSELPY-H LFFSTIFFVS SYFPLRIFFE ASR-SAVYFL NYCIMFQLYY
(PRED) sapa_25_1_a0246 QYLSELPY-H LFFSTIFFVS SYFPLRIFFE ASR-SAVYFL NYCIMFQLYY
(PRED) sapa_4_1_a02470 QYLSELPY-H LFFSTIFFVS SYFPLRIFFE ASR-SAVYFL NYCIMFQLYY
(PRED) sapa_5_1_2350   QYLSELPY-H LFFSTIFFVS SYFPLRIFFE ASR-SAVYFL NYCIMFQLYY
(PRED) sapa_9_1_2360   QYLSELPY-H LFFSTIFFVS SYFPLRIFFE ASR-SAVYFL NYCIMFQLYY
(PRED) sapa_14_1_a0244 QYLSELPY-H LFFSTIFFVS SYFPLRIFFE ASR-SAVYFL NYCIMFQLYY
(PRED) sapa_8_1_2350   QYLSELPY-H LFFSTIFFVS SYFPLRIFFE ASR-SAVYFL NYCIMFQLYY
(PRED) sapa_17_1_2380  QYLSELPY-H LFFSTIFFVS SYFPLRIFFE ASR-SAVYFL NYCIMFQLYY
(PRED) sapa_7_1_2370   QYLSELPY-H LFFSTIFFVS SYFPLRIFFE ASR-SAVYFL NYCIMFQLYY
(PRED) sapa_2_1_a02460 QYLSELPY-H LFFSTIFFVS SYFPLRIFFE ASR-SAVYFL NYCIMFQLYY
(PRED) sapa_23_1_a0248 QYLSELPY-H LFFSTIFFVS SYFPLRIFFE ASR-SAVYFL NYCIMFQLYY
(PRED) sapa_3_1_a02470 QYLSELPY-H LFFSTIFFVS SYFPLRIFFE ASR-SAVYFL NYCIMFQLYY
(PRED) sapa_18_1_2390  QYLSELPY-H LFFSTIFFVS SYFPLRIFFE ASR-SAVYFL NYCIMFQLYY
(PRED) sami_1_4_244    QYLSELPY-H LFFSTIFFVS SYFPLRIFFE ASR-SAVYFL NYCIMFQLYY
(PRED) saku_1_4_262    QYLSELPY-H LLFSTVFFVS SYFPLRIFFE ASR-SAVYFL NYCIMFQLYY
(PRED) saba_1_58_bf002 QYLSELPY-H LLFSTIFFVC SYFPLRIFFE ASR-SAVYFL NYCIMFQLYY
(PRED) saeu_1_4_d02400 QYLSELPY-H LLFSTIFFVS SYFPLRIFFE ASR-SAVYFL NYCIMFQLYY
(PRED) naca_1_e01640   QYLNELPY-H LFFSTIFFVS SYFPLRIFYE APR-SGVYFL NYCIMFQLYY
(PRED) nada_1_g01850   QFLNELPY-H LLFSTIFFVS SYFPLRNFFQ ASR-SGVYFL NYCIMYQLYY
(PRED) naca_1_e01630   QYLNELPY-H LFFSTIFFVS SYFPLRIFFE ASR-SGVYFL NYCIMFQLYY
(PRED) nada_1_g01840   QFLNELPY-Q LVFSTIFFVS SYFPLRIFFE ASR-SGVYFL NYCIVFPLYF
(PRED) kaaf_1_c00820   QYLSELPY-H LVFSIIFFVS SYFPLRNHFG TPF-SGVYFL NYCIMFQLYY
(PRED) teph_1_m00640   QYMSEIPY-H LVFSTIFFVS LYFPLRIFFE ASR-SAVFYL NYCILFQLFY
(PRED) vapo_1_1036_28  QYLSEIPY-H LFFSTLFFVS LYFPLRIFFE ASR-SAVFYL NYCILFQLFY
(PRED) tebl_1_i01760   QYLSELPY-H ILFSTFFFVS YYFPLRTFFE ASR-SAWFL  NYSFVFQLYF
(PRED) tode_1_d04040   QYLCEIPY-H FVFSTLYFVS FYFPLRIFFQ ASR-SAVFFL NYCIMFQLYY
(PRED) naca_1_e01650   QYLNELPY-H LFFSTIFFVS SYFPLRIFYE APR-SGVYFL NYCIMFQLYY
(PRED) tebl_1_g02820   QYICEIPY-H FVCSAIFFVS MYFPLRSFFE ASR-SAVYYL NYCIMFQLFY
(PRED) lakl_1_c11616g  QYLTELPY-H LFFSTVFFVS FYFPLRTDFA ASK-SGVFFL DYCIMFQLYF
(PRED) saar_1_8_h03780 QYLNELPY-H LLFSTIFFVS LYFPLGIFFE ASR-SGVFYL NYAILFQLYY
```

```
(PRED) sace_14_7_g0015 HYLNELPY-H LLFSTIFFVS LYFPLGVFTX ASR-SSXFYL XYXILXXLXX
(PRED) sace_15_7_g0387 HYLNELPY-H LLFSTIFFVS LYFPLGVFTE ASR-SSVFYL NYAILFQLYY
(PRED) sace_24_8_3780  HYLNELPY-H LLFSTIFFVS LYFPLGVFTE ASR-SSVFYL NYAILFQLYY
(PRED) sace_40_8_h0383 HYLNELPY-H LLFSTIFFVS LYFPLGVFTE ASR-SSVFYL NYAILFQLYY
(PRED) sace_6_169_fm00 HYLNELPY-H LLFSTIFFVS LYFPLGVFTE ASR-SSVFYL NYAILFQLYY
(PRED) sace_19_7_3840  HYLNELPY-H LLFSTIFFVS LYFPLGVFTE ASR-SSVFYL NYAILFQLYY
(PRED) sace_32_7_3770  HYLNELPY-H LLFSTIFFVS LYFPLGVFTE ASR-SGVFYL NYAILFQLYY
(PRED) sace_56_17_q011 HYLNELPY-H LLFSTIFFVS LYFPLGVFTE ASR-SGVFYL NYAILFQLYY
(PRED) sace_5_78_bz001 HYLNELPY-H LLFSTIFFVS LYFPLGVFTE ASR-SSVFYL NYAILFQLYY
(PRED) sace_2_8_h03860 HYLNELPY-H LLFSTIFFVS LYFPLGVFTE ASR-SSVFYL NYAILFQLYY
(PRED) sace_53_29_ac00 HYLNELPY-H LLFSTIFFVS LYFPLGVFTE ASR-SSVFYL NYAILFQLYY
(PRED) sace_17_7_g0393 HYLNELPY-H LLFSTIFFVS LYFPLGVFTE ASR-SGVFYL NYAILFQLYY
(PRED) sace_25_7_g0388 HYLNELPY-H LLFSTIFFVS LYFPLGVFTE ASR-SGVFYL NYAILFQLYY
(PRED) sace_37_7_g0385 HYLNELPY-H LLFSTIFFVS LYFPLGVFTE ASR-SGVFYL NYAILFQLYY
(PRED) sace_9_7_g00180 HYLNELPY-H LLFSTIFFVS LYFPLGVFTE ASR-SGVFYL NYAILFQLYY
(PRED) sace_60_6_f0335 HYLNELPY-H LLFSTIFFVS LYFPLGVFTE ASR-SGVFYL NYAILFQLYY
(PRED) sace_59_336_lx0 HYLNELPY-H LLFSTIFFVS LYFPLGVFTE ASR-SGVFYL NYAILFQLYY
(PRED) sace_31_7_3780  HYLNELPY-H LLFSTIFFVS LYFPLGVFTE ASR-SGVFYL NYAILFQLYY
(PRED) sace_34_8_3770  HYLNELPY-H LLFSTIFFVS LYFPLGVFTE ASR-SGVFYL NYAILFQLYY
(PRED) sace_58_71_bs00 HYLNELPY-H LLFSTIFFVS LYFPLGVFTE ASR-SGVFYL NYAILFQLYY
(PRED) sace_7_7_g03880 HYLNELPY-H LLFSTIFFVS LYFPLGVFTE ASR-SGVFYL NYAILFQLYY
(PRED) sace_35_7_3840  HYLNELPY-H LLFSTIFFVS LYFPLGVFTE ASR-SGVFYL NYAILFQLYY
(PRED) sace_43_7_g0387 HYLNELPY-H LLFSTIFFVS LYFPLGVFTE ASR-SGVFYL NYAILFQLYY
(PRED) sace_57_8_h0390 HYLNELPY-H LLFSTIFFVS LYFPLGVFTE ASR-SGVFYL NYAILFQLYY
(PRED) sace_45_7_g0389 HYLNELPY-H LLFSTIFFVS LYFPLGVFTE ASR-SGVFYL NYAILFQLYY
(PRED) sace_46_8_h0391 HYLNELPY-H LLFSTIFFVS LYFPLGVFTE ASR-SGVFYL NYAILFQLYY
(PRED) sace_23_7_3860  HYLNELPY-H LLFSTIFFVS LYFPLGVFTE ASR-SGVFYL NYAILFQLYY
(PRED) sace_21_7_3790  HYLNELPY-H LLFSTIFFVS LYFPLGVFTE ASR-SGVFYL NYAILFQLYY
(PRED) sace_8_73_bu001 HYLNELPY-H LLFSTIFFVS LYFPLGVFTE ASR-SGVFYL NYAILFQLYY
(PRED) sapa_1_8_h03820 HYLNELPY-H LLFSTIFFVS LYFPLGIFFE VSR-SGVFYL NYSILFQLYY
(PRED) sapa_21_8_h0387 HYLNELPY-H LLFSTIFFVS LYFPLGIFFE VSR-SGVFYL NYSILFQLYY
(PRED) sapa_20_8_h0386 HYLNELPY-H LLFSTIFFVS LYFPLGIFFE VSR-SGVFYL NYSILFQLYY
(PRED) sapa_22_8_h0390 HYLNELPY-H LLFSTIFFVS LYFPLGIFFE VSR-SGVFYL NYSILFQLYY
(PRED) sapa_25_8_h0387 HYLNELPY-H LLFSTIFFVS LYFPLGIFFE VSR-SGVFYL NYSILFQLYY
(PRED) sapa_6_8_3750   HYLNELPY-H LLFSTIFFVS LYFPLGIFFE VSR-SGVFYL NYSILFQLYY
(PRED) sapa_9_8_3720   HYLNELPY-H LLFSTIFFVS LYFPLGIFFE VSR-SGVFYL NYSILFQLYY
(PRED) sapa_19_8_h0390 HYLNELPY-H LLFSTIFFVS LYFPLGIFFE VSR-SGVFYL NYSILFQLYY
(PRED) sapa_24_8_h0385 HYLNELPY-H LLFSTIFFVS LYFPLGIFFE VSR-SGVFYL NYSILFQLYY
(PRED) sapa_4_8_h03850 HYLNELPY-H LLFSTIFFVS LYFPLGIFFE VSR-SGVFYL NYSILFQLYY
(PRED) sapa_10_8_3760  HYLNELPY-H LLFSTIFFVS LYFPLGIFFE VSR-SGVFYL NYSILFQLYY
(PRED) sapa_13_8_h0382 HYLNELPY-H LLFSTIFFVS LYFPLGIFFE VSR-SGVFYL NYSILFQLYY
(PRED) sapa_8_8_3750   HYLNELPY-H LLFSTIFFVS LYFPLGIFFE VSR-SGVFYL NYSILFQLYY
(PRED) sapa_11_8_h0383 HYLNELPY-H LLFSTIFFVS LYFPLGIFFE VSR-SGVFYL NYSILFQLYY
(PRED) sapa_5_8_3700   HYLNELPY-H LLFSTIFFVS LYFPLGIFFE VSR-SGVFYL NYSILFQLYY
(PRED) sapa_16_8_h0389 HYLNELPY-H LLFSTIFFVS LYFPLGIFFE VSR-SGVFYL NYSILFQLYY
(PRED) sapa_17_8_3730  HYLNELPY-H LLFSTIFFVS LYFPLGIFFE VSR-SGVFYL NYSILFQLYY
(PRED) sapa_2_8_h03860 HYLNELPY-H LLFSTIFFVS LYFPLGIFFE VSR-SGVFYL NYSILFQLYY
(PRED) sapa_7_8_3740   HYLNELPY-H LLFSTIFFVS LYFPLGIFFE VSR-SGVFYL NYSILFQLYY
(PRED) sapa_23_8_h0385 HYLNELPY-H LLFSTIFFVS LYFPLGIFFE VSR-SGVFYL NYSILFQLYY
(PRED) sapa_3_8_h03890 HYLNELPY-H LLFSTIFFVS LYFPLGIFFE VSR-SGVFYL NYSILFQLYY
(PRED) sapa_18_8_3730  HYLNELPY-H LLFSTIFFVS LYFPLGIFFE VSR-SGIFYL NYSILFQLYY
(PRED) sami_1_14_399   HYLNELPY-H LLFSTIFFVS LYFPLGIFFE VSR-SGVFYL NYAILFQLYY
(PRED) sace_4_8_h03690 HYLNELPY-H LLFSTIFFVS LYFPLGVFTE ASR-SSVFYL NYAILFQLYY
(PRED) saku_1_14_404   HYLNELPY-H LLFSTIFFVS LYFPLGIFFE ASR-SGVFYL NYAILFQLYY
(PRED) sace_1_ynr070w  HYLNELPY-H LLFSTIFFVS SYFPLGVFTE ASR-SSVFYL NYAILFQLYY
(PRED) sace_49_8_h0383 HYLNELPY-H LLFSTIFFVS XYFPLGVFTE ASR-SXVFYL NYAILFQLYY
(PRED) saeu_1_2_b00130 QYLNELPY-H LLFSTIFFVS FYFPLGIFFE VSR-SGIVYL NYAIIFQLYY
(PRED) sauv_1_7_3      QYLNELPY-H LLFSTIFFVS FYFPLGIFFE VSR-SGIVFL TYAIIFQLYY
(PRED) sami_1_17_26    QYLNELPY-H LLFSTIFFVS FYFPLGIFFE ASR-SGLFYL NYAIVFQFYY
(PRED) zyba_1_02055_AN QYLAEIPY-H LLFSTMFFVA FYFPLRVFFE ASR-SAVFYL NYCIMFQLYY
(PRED) zyba_1_07912    QYLAEIPY-H LLFSTMFFVA FYFPLRVFFE ASR-SAVFYL NYCIMFQLYY
(PRED) zyba_2_2_b00600 QYLAEIPY-H LLFSTMFFVA FYFPLRVFFE ASR-SAVFYL NYCIMFQLYY
(PRED) zyba_3_3_c03460 QYLAEIPY-Q LLFSTMFFVA FYFPLRVFFE ASR-SAVFYL NYCIMFQLYY
(PRED) zyba_1_04634    QYLAEIPY-Q FIFSTMFFCG MYFPLRVFFE ASR-SAVFFL NYSIMFQLYY
(PRED) zyba_1_06675    EYLAEIPY-Q FVFSAMFFCG MYFPLRVFFE ASR-SAVFFL NYSIMFQLYY
(PRED) zyba_3_2_b02230 EYLAEIPY-Q FVFSAMFFCG MYFPLRVFFE ASR-SAVFFL NYSIMFQLYY
(PRED) zyba_2_1_a00860 EYLAEIPY-Q FVFSTMFFCG MYFPLRVFFE ASR-SAVFFL NYSIMFQLYY
(PRED) zyro_1_a04114g  QYMAEIPY-H ILFSTFFFCA SYFPLRVWFE ASR-SAVFFL NYCIMFQLYY
(PRED) zyro_1_b14762g  QYLAEIPY-H ILFSTMFFCA FYFPLRVWFE ASR-SAVFFL NYCIMFQLFY
(PRED) zyba_2_14_n0149 QYLAELPY-Q ILFSTMFFVA FYFPLRVFFE ASR-SAVFFL NYCIMFQVYY
(PRED) zyba_2_33_ag001 QYLAELPY-Q ILFSTMFFVA FYFPLRVFFE ASR-SAVFFL NYCIMFQIYY
(PRED) lath_1_a01914g  QYLCEIPY-H LVFSTLFFVA FYFPLRVHFA ATY-SGVFFL NYCIMFQLYL
(PRED) lawa_1_23_5161  QYLCEIPY-H FVFSTMFFVA FYFPLRVHFE ASY-SAVFFL NYCIMFQLYI
(PRED) klae_1_14_n0012 QYLCELPY-N LFCTTIFFVS FFFALRADSN SLK-AGLFYL NYSIIFQLYF
(PRED) klla_1_d03432g  QYITELPY-Q LWCSTLFFVS FYFPLKAEYT SLK-AGLFYL NYCVIFQLYC
```

```
(PRED) klma_1_1_a01880 QYITELPY-Q  LWCSTLFFVS  FYFPLRAEYT  SVK-AGLFFL  NYCVMFQLYC
(PRED) klwi_1_33_ag001 QFLCELPY-Q  LWCSTLFFVS  FYFPLRAEST  SLK-AGLFFL  NYCVMFQLYS
(PRED) teph_1_a04220   EYIAELPF-H  LFVSTIFFVS  FYFPVGLFFE  ASR-SAVFFL  NYCIVFQLFF
(PRED) vapo_1_1037_47  QYFSEIPY-S  LVFSAIYFVS  FYFPVGIFYE  ASR-SAVFYL  NYGVMFQFFY
(PRED) pata_1_2_b05590 QFLNEIPY-S  LLFSTMYFVI  FYFTVQLDNN  VTR-SGVFWL  NYSFFFQFYY
(PRED) wian_1_3_c04380 QFVNEIPY-H  LVSGAMFFCC  LYFPLRIDNE  ASR-SAVWYL  NYSIIYQFYY
(PRED) wian_1_3_c04390 QFLNEIPY-H  LLSGAIFFCC  LYFPLKINNT  ASR-AAVWYL  NYSIIYQFYY
(PRED) wian_1_7_g01010 QFLNEMPY-H  LVSGAMFFCC  LYFPLRANDA  PDR-AAVFYL  NYAIIYQFYY
(PRED) bain_1_1_a00100 QFITELPY-C  IIGSTFFFCC  LYFPLRIDNS  ALF-AGNYFL  NYSILFQLYY
(PRED) bain_1_17_q0038 QFITELPY-C  IIGSTVFFCC  LYFPLRINNS  ALF-AGNYFL  NYSILFQLYY
(PRED) bain_1_8_h00410 QIFVELPY-T  VLGSTFFYLC  LYFPLKLAPS  NLV-AGTYYL  IYCICFQLYY
(PRED) caal_1_19_5759  QIIFEVIY-M  IIGGTIMFVC  LYFPTQVSTV  ASH-SGMFYF  SQAIFLQTFA
(PRED) caal_11_25_y002 QIIFEVIY-M  IIGGTIMFVC  LYFPTQVSTV  ASH-SGMFYF  SQAIFLQTFA
(PRED) caal_4_4_d03320 QIIFEVIY-M  IIGGTIMFVC  LYFPTQVSTV  ASH-SGMFYF  SQAIFLQTFA
(PRED) caal_12_26_z005 QIIFEVIY-M  IIGGTIMFVC  LYFPTQVSTV  ASH-SGMFYF  SQAIFLQTFA
(PRED) caal_5_30_ad005 QIIFEVIY-M  IIGGTIMFVC  LYFPTQVSTV  ASH-SGMFYF  SQAIFLQTFA
(PRED) caal_8_3_c03320 QIIFEVIY-M  IIGGTIMFVC  LYFPTQVSTV  ASH-SGMFYF  SQAIFLQTFA
(PRED) caal_6_4_d03280 QIIFEVIY-M  IIGGTIMFVC  LYFPTQVSTV  ASH-SGMFYF  SQAIFLQTFA
(PRED) caal_10_3_c0334 QIIFEVIY-M  IIGGTIMFVC  LYFPTQVSTV  ASH-SGMFYF  SQAIFLQTFA
(PRED) caal_3_29_ac005 QIIFEVIY-M  IIGGTIMFVC  LYFPTQVSTV  ASH-SGMFYF  SQAIFLQTFA
(PRED) caal_2_04989    HIIFEVIY-M  IIGGTIMFVC  LYFPTQVSTV  ASH-SGMFYV  SQAIFLQTFA
(PRED) cadu_1_64350    QVIFEVIY-M  IIGGTIMFVC  LYFPTQVNTV  ASH-SGIFYF  SQAIFLQTFA
(PRED) caor_1_h02090   QALNEMPY-L  LVGGAIMFVS  VYFPTQAATT  ASQ-SGMFYF  TQGVFVQAFA
(PRED) capa_1_600750   QALNEMPY-L  LVGGAIMFVS  VYFPTQAATT  ASQ-SGMFYF  TQGIFVQAFA
(PRED) loel_1_04930    QILNEMPY-L  MIGAAIMFVS  LYFPTQVDTL  GSTFRYVLLY  TWNFFARVCC
(PRED) spar_1_5_e03260 QFINELPY-L  FIGAAIMFVS  VYFPTQVDTS  PPH-SGMFYF  TQGIFLQGFA
(PRED) sppa_1_7_g03160 QFINELPY-L  FIGAAIMFVS  VYFPTQVDTS  PSH-SGMFYF  THGIFLQGFA
(PRED) catr_1_01205    TST-ENTY-L  FYRWSILFVC  VYFP--ATRE  AGSQAGMFYF  TQGIFLQLFT
(PRED) catr_1_05498    SMIPEIPY-L  IVGGTFFFIT  VYFP--ATRS  AGSQAGIFYF  TQGVFLQFFT
(PRED) catr_1_05971    SILPEIPY-L  IVGGTFFFVS  VYFP--ATRH  ASAQAGMFFF  TQGIFLQLFT
(PRED) deha_1_a03696g  QCINELPY-L  IFGSTIMFVS  LYFPTQADPS  PSH-SGMFYL  TQGIFLQGFV
(PRED) deha_2_5_e00720 QCINELPY-L  IFGSTIMFVS  LYFPTQADTS  PPH-SGIFYL  TQGIFLQGFV
(PRED) scst_1_3_c02890 QVIVEMPY-L  VFGATLMFVS  LYFPTQADTS  GPH-AGVFYL  AQGIFLQSFV
(PRED) mebi_1_8_h00300 EFLNEVPY-T  VFAATLLYVP  LFFATKADTD  ASH-AGVFFL  SYAIFLELFN
(PRED) lakl_1_h21010g  QYLSEVPF-Q  IFFSTIYFVA  FYFPLKVDDS  ASR-AGVFFL  HYCIMFQLYF
(PRED) caar_1_13_m0142 QFLVEIPY-S  IVCGTVYFVV  WYFPVQVDNE  PSR-AGLWWF  TQSFFFQLYY
(PRED) caar_1_14_n0143 QYVAEIPY-G  IAFGTIYFIC  WYFPIQLDNE  ASR-AGLWWF  TQSVLFQLYY
(PRED) hapo_1_1_a07220 QYLVELPY-S  IIFGTIYFIC  WYFPIQLDNE  ASR-AGLWWF  CQSIFFQLYY
(PRED) ogpa_1_1_a01680 QYLVELPY-S  ITFGTIYFIC  WYFPIQLDNE  ASR-AGLWWF  CQSVFFQLYY
(PRED) piku_1_96_cr001 QFLTEIPF-C  VCFSTIYFIC  WYFPIQLDNS  ASR-CGMWWF  TYCIFFQIYY
(PRED) pime_1_4_d03240 QFLSEIPY-G  IFFSTIYFIC  WYFPIQLDNQ  PSV-CGLWWF  TYCVFFQLYY
(PRED) pime_1_1_a12110 QALVEIPY-G  IIFSTIYFVC  WYFPIQLDNE  PSR-AGMWWF  TYCFFFQMFY
(PRED) piku_1_227_hs00 QYLNELPY-C  IIMSTIYFVT  WYFPIQLDNS  PSR-CGLWWF  VYCFFYQLYY
(PRED) pime_1_5_e05800 QYVNEVPY-C  IVFSTFYFIG  WYFPVQFNNS  PER-CGFWWF  TYCFFYSLYY
(PRED) pime_1_1_a07690 QYVNEIPY-C  IVFSTIFFVC  WYFPIQLDNE  PSR-AGFWWF  TYCFFYQLYF
(PRED) debr_2_5_e03380 QLLVEIPY-A  LVNATIYFIV  WYFPIQLPLS  AHI-AGFWWL  TYAIFFQIYI
(PRED) kopa_1_2_b10040 QFLVEIPY-S  VVFSTIFYIC  WYFPVQLDNA  PER-AGVWWL  HYCIFFQLYY
(PRED) kopa_2_7_g00500 QFLVEIPY-S  VVFSTIFYIC  WYFPVQLDNA  PER-AGVWWL  HYCIFFQLYY
(PRED) asru_1_13_m0119 QFITEIPY-C  IVAFTLFFCC  FYFPLKMDNT  APF-AGYYYF  ILCTVFQFYY
(PRED) asru_1_15_o0045 QLIAEIPY-S  LVGSAIFFCC  LYFTLKVNNS  PEF-AGYYYL  MVAVIFPLFY
(PRED) wian_1_1_a02920 QILTELPY-N  LLGCTIFFCS  CYFPLRAYQG  PSL-AGKYYF  TLAIMYELYI
(PRED) wian_1_1_a02930 QILTEIPY-N  FFGSTIYFLC  SYFPLRIYQN  STL-TGKFYF  MVSIIYQIYI

                       .........1660.........1670.........1680.........1690.........1700
(PRED) asac_1_6_f03560 VGLGLAVLYL  SPNLESANVI  LGVCLSFLIS  FCGVVQPYYL  MP-AFW-TFM
(PRED) ergo_1_abr125c  VGLGLAVLYL  APNLESANVI  LGVFLSFLIS  FCGVVQPYYL  MP-GFW-KFM
(PRED) ercy_1_3604     VGLGLAVLYA  SPNLESANVI  LGLCLSFLIS  FCGVVQPYRL  MP-GFW-KFM
(PRED) cagl_1_i04862g  IGFGLCVLYM  APNLQSANVI  LGLCLSFLIA  FCGVVQPVSL  MP-GFW-TFM
(PRED) kaaf_1_c00830   VGLGLMILYM  SPDLQSANVI  LGLILSFLIS  FCGVTQPKSL  MP-TFW-TFM
(PRED) kana_1_k01350   IGLGLLVLYM  SPNLQSANVI  LGLTLSFMIA  FCGVVQPKSL  LP-GFW-TFM
(PRED) saar_1_2_b02590 VGLGLLILYI  SPNLPSANVI  LGLCLSFMLS  FCGVTQPVSL  MP-GFW-TFM
(PRED) sace_1_ydr011w  VGLGLMILYM  SPNLPSANVI  LGLCLSFMLS  FCGVTQPVSL  MP-GFW-TFM
(PRED) sace_16_1_a0238 VGLGLMILYM  SPNLPSANVI  LGLCLSFMLS  FCGVTQPVSL  MP-GFW-TFM
(PRED) sace_45_1_a0242 VGLGLMILYM  SPNLPSANVI  LGLCLSFMLS  FCGVTQPVSL  MP-GFW-TFM
(PRED) sace_48_1_a0238 VGLGLMILYM  SPNLPSANVI  LGLCLSFMLS  FCGVTQPVSL  MP-GFW-TFM
(PRED) sace_60_4_d0244 VGLGLMILYM  SPNLPSANVI  LGLCLSFMLS  FCGVTQPVSL  MP-GFW-TFM
(PRED) sace_52_1_a0240 VGLGLMILYM  SPNLPSANVI  LGLCLSFMLS  FCGVTQPVSL  MP-GFW-TFM
(PRED) sace_46_1_a0240 VGLGLMILYM  SPNLPSANVI  LGLCLSFMLS  FCGVTQPVSL  MP-GFW-TFM
(PRED) sace_25_1_a0240 VGLGLMILYM  SPNLPSANVI  LGLCLSFMLS  FCGVTQPVSL  MP-GFW-TFM
(PRED) sace_24_1_2300  VGLGLMILYM  SPNLPSANVI  LGLCLSFMLS  FCGVTQPVSL  MP-GFW-TFM
(PRED) sace_47_1_a0240 VGLGLMILYM  SPNLPSANVI  LGLCLSFMLS  FCGVTQPVSL  MP-GFW-TFM
(PRED) sace_7_1_a02410 VGLGLMILYM  SPNLPSANVI  LGLCLSFMLS  FCGVTQPVSL  MP-GFW-TFM
(PRED) sace_59_110_df0 VGLGLMILYM  SPNLPSANVI  LGLCLSFMLS  FCGVTQPVSL  MP-GFW-TFM
(PRED) sace_56_1_a0202 VGLGLMILYM  SPNLPSANVI  LGLCLSFMLS  FCGVTQPVSL  MP-GFW-TFM
(PRED) sace_40_1_a0239 VGLGLMILYM  SPNLPSANVI  LGLCLSFMLS  FCGVTQPVSL  MP-GFW-TFM
```

```
(PRED) sace_15_1_a0242 VGLGLMILYM SPNLPSANVI LGLCLSFMLS FCGVTQPVSL MP-GFW-TFM
(PRED) sace_37_1_a0243 VGLGLMILYM SPNLPSANVI LGLCLSFMLS FCGVTQPVSL MP-GFW-TFM
(PRED) sace_9_1_a02440 VGLGLMILYM SPNLPSANVI LGLCLSFMLS FCGVTQPVSL MP-GFW-TFM
(PRED) sace_22_1_2300  VGLGLMILYM SPNLPSANVI LGLCLSFMLS FCGVTQPVSL MP-GFW-TFM
(PRED) sace_29_1_2290  VGLGLMILYM SPNLPSANVI LGLCLSFMLS FCGVTQPVSL MP-GFW-TFM
(PRED) sace_34_1_2320  VGLGLMILYM SPNLPSANVI LGLCLSFMLS FCGVTQPVSL MP-GFW-TFM
(PRED) sace_58_25_y007 VGLGLMILYM SPNLPSANVI LGLCLSFMLS FCGVTQPVSL MP-GFW-TFM
(PRED) sace_23_1_2290  VGLGLMILYM SPNLPSANVI LGLCLSFMLS FCGVTQPVSL MP-GFW-TFM
(PRED) sace_6_120_dp00 VGLGLMILYM SPNLPSANVI LGLCLSFMLS FCGVTQPVSL MP-GFW-TFM
(PRED) sace_57_1_a0241 VGLGLMILYM SPNLPSANVI LGLCLSFMLS FCGVTQPVSL MP-GFW-TFM
(PRED) sace_17_1_a0241 VGLGLMILYM SPNLPSANVI LGLCLSFMLS FCGVTQPVSL MP-GFW-TFM
(PRED) sace_21_1_2310  VGLGLMILYM SPNLPSANVI LGLCLSFMLS FCGVTQPVSL MP-GFW-TFM
(PRED) sace_49_1_a0246 VGLGLMILYM SPNLPSANVI LGLCLSFMLS FCGVTQPVSL MP-GFW-TFM
(PRED) sace_8_2_b02430 VGLGLMILYM SPNLPSANVI LGLCLSFMLS FCGVTQPVSL MP-GFW-TFM
(PRED) sace_31_1_2300  VGLGLMILYM SPNLPSANVI LGLCLSFMLS FCGVTQPVSL MP-GFW-TFM
(PRED) sace_50_1_a0241 VGLGLMILYM SPNLPSANVI LGLCLSFMLS FCGVTQPVSL MP-GFW-TFM
(PRED) sace_4_1_a02360 VGLGLMILYM SPNLPSANVI LGLCLSFMLS FCGVTQPVSL MP-GFW-TFM
(PRED) sace_2_1_a02390 VGLGLMILYM SPNLPSANVI LGLCLSFMLS FCGVTQPVSL MP-GFW-TFM
(PRED) sace_5_33_ag005 VGLGLMILYM SPNLPSANVI LGLCLSFMLS FCGVTQPVSL MP-GFW-TFM
(PRED) sapa_11_1_a0247 VGLGLMILYM SPNLPSANVI LGLCLSFMLS FCGVTQPVSL MP-GFW-TFM
(PRED) sapa_25_1_a0246 VGLGLMILYM SPNLPSANVI LGLCLSFMLS FCGVTQPVSL MP-GFW-TFM
(PRED) sapa_4_1_a02470 VGLGLMILYM SPNLPSANVI LGLCLSFMLS FCGVTQPVSL MP-GFW-TFM
(PRED) sapa_5_1_2350   VGLGLMILYM SPNLPSANVI LGLCLSFMLS FCGVTQPVSL MP-GFW-TFM
(PRED) sapa_9_1_2360   VGLGLMILYM SPNLPSANVI LGLCLSFMLS FCGVTQPVSL MP-GFW-TFM
(PRED) sapa_14_1_a0244 VGLGLMILYM SPNLPSANVI LGLCLSFMLS FCGVTQPVSL MP-GFW-TFM
(PRED) sapa_8_1_2350   VGLGLMILYM SPNLPSANVI LGLCLSFMLS FCGVTQPVSL MP-GFW-TFM
(PRED) sapa_17_1_2380  VGLGLMILYM SPNLPSANVI LGLCLSFMLS FCGVTQPVSL MP-GFW-TFM
(PRED) sapa_7_1_2370   VGLGLMILYM SPNLPSANVI LGLCLSFMLS FCGVTQPVSL MP-GFW-TFM
(PRED) sapa_2_1_a02460 VGLGLMILYM SPNLPSANVI LGLCLSFMLS FCGVTQPVSL MP-GFW-TFM
(PRED) sapa_23_1_a0248 VGLGLMILYM SPNLPSANVI LGLCLSFMLS FCGVTQPVSL MP-GFW-TFM
(PRED) sapa_3_1_a02470 VGLGLMILYM SPNLPSANVI LGLCLSFMLS FCGVTQPVSL MP-GFW-TFM
(PRED) sapa_18_1_2390  VGLGLMILYM SPNLPSANVI LGLCLSFMLS FCGVTQPVSL MP-GFW-TFM
(PRED) sami_1_4_244    VGLGLLILYM SPNLPSANVI LGLCLSFMLS FCGVTQPVSL MP-GFW-TFM
(PRED) saku_1_4_262    VGLGLLILYM SPNLPSANVI LGLCLSFMLS FCGVTQPVSL MP-GFW-TFM
(PRED) saba_1_58_bf002 VGLGLLVLYM SPNLPSANVI LGLCLSFMIS FCGVTQPVSL MP-GFW-TFM
(PRED) saeu_1_4_d02400 VGLGLLILYM SPNLPSANVI LGLCLSFMLS FCGVTQPVSL MP-GFW-TFM
(PRED) naca_1_e01640   VGFGLMVLYA SPNLPSANVI LGLCLSFLIS FCGVTQPKSL MP-GFW-TFM
(PRED) nada_1_g01850   VGFGLMVLYV SPNLPSANVI LGLCLSFLIS FCGVTQPPSL MP-GFW-TFM
(PRED) naca_1_e01630   VGLGLLILYM SPNLPSANVL LGLAMSFLIA FCGVTQPASL MP-GFW-TFM
(PRED) nada_1_g01840   VGFGLMVLYM APNLPSANVL LGLALSFLIA FCGVTQPPSL MP-GFWRRFM
(PRED) kaaf_1_c00820   VGFGLLILYM APNLPSANVI LGLALSFLIA FCGVVQPKSL MP-GFW-TFM
(PRED) teph_1_m00640   VGLGLMVLYM SPNLPSAAIL MGLTLSFLIS FCGVTQPERL MP-GFW-TFM
(PRED) vapo_1_1036_28  VALGLMVLYM SPDLPSANIL MGLTLSFLIS FCGVTQPESL MP-GFW-TFM
(PRED) tebl_1_i01760   VGLGLMVLYM SPNLQSANVI MGLTLSFLIG FCGVVQPKSL MP-GFW-TFM
(PRED) tode_1_d04040   VALGLSVLYM SPNLPSASVL MGLILAFLLS FCGVVQPPSL MP-GFW-TFM
(PRED) naca_1_e01650   VGFGLMVLYA SPNLPSANVI LGLCLSFLIS FCGVTQPKSL MP-GFW-TFM
(PRED) tebl_1_g02820   VSLGLMILYV APNLPSANVI MGLLLSFMLG FCGVVQPASL MP-TFW-TFM
(PRED) lakl_1_c11616g  VGLGLAVLYM SPNLPSANVI LGLFLSFLIS FCGIVQPSSL MP-GFW-TFM
(PRED) saar_1_8_h03780 VGLALMILYM SPNLQSANVI VGFILSFLLS FCGAVQPASL MP-GFW-TFM
(PRED) sace_14_7_g0015 XXXXXXXXYI XXXXXXXXXX XXXXLSFLLS FCGAVLPASL MP-GFW-TFM
(PRED) sace_15_7_g0387 IGLALMILYM SPNLQSANVI VGFILSFLLS FCGAVQPASL MP-GFW-TFM
(PRED) sace_24_8_3780  IGLALMILYM SPNLQSANVI VGFILSFLLS FCGAVQPASL MP-GFW-TFM
(PRED) sace_40_8_h0383 IGLALMILYM SPNLQSANVI VGFILSFLLS FCGAVQPASL MP-GFW-TFM
(PRED) sace_6_169_fm00 IGLALMILYM SPNLQSANVI VGFILSFLLS FCGAVQPASL MP-GFW-TFM
(PRED) sace_19_7_3840  IGLALMILYM SPNLQSANVI VGFILSFLLS FCGAVQPASL MP-GFW-TFM
(PRED) sace_32_7_3770  IGLALMILYM SPNLQSANVI VGFILSFLLS FCGAVQPASL MP-GFW-TFM
(PRED) sace_56_17_q011 IGLALMILYM SPNLQSANVI VGFILSFLLS FCGAVQPASL MP-GFW-TFM
(PRED) sace_5_78_bz001 IGLALMILYM SPNLQSANVI VGFILSFLLS FCGAVQPASL MP-GFW-TFM
(PRED) sace_2_8_h03860 IGLALMILYM SPNLQSANVI VGFILSFLLS FCGAVQPASL MP-GFW-TFM
(PRED) sace_53_29_ac00 IGLALMILYM SPNLQSANVI VGFILSFLLS FCGAVQPASL MP-GFW-TFM
(PRED) sace_17_7_g0393 IGLALMILYM SPNLQSANVI VGFILSFLLS FCGAVQPASL MP-GFW-TFM
(PRED) sace_25_7_g0388 IGLALMILYM SPNLQSANVI VGFILSFLLS FCGAVQPASL MP-GFW-TFM
(PRED) sace_37_7_g0385 IGLALMILYM SPNLQSANVI VGFILSFLLS FCGAVQPASL MP-GFW-TFM
(PRED) sace_9_7_g00180 IGLALMILYM SPNLQSANVI VGFILSFLLS FCGAVQPASL MP-GFW-TFM
(PRED) sace_60_6_f0335 IGLALMILYM SPNLQSANVI VGFILSFLLS FCGAVQPASL MP-GFW-TFM
(PRED) sace_59_336_lx0 IGLALMILYM SPNLQSANVI VGFILSFLLS FCGAVQPASL MP-GFW-TFM
(PRED) sace_31_7_3780  IGLALMILYM SPNLQSANVI VGFILSFLLS FCGAVQPASL MP-GFW-TFM
(PRED) sace_34_8_3770  IGLALMILYM SPNLQSANVI VGFILSFLLS FCGAVQPASL MP-GFW-TFM
(PRED) sace_58_71_bs00 IGLALMILYM SPNLQSANVI VGFILSFLLS FCGAVQPASL MP-GFW-TFM
(PRED) sace_7_7_g03880 IGLALMILYM SPNLQSANVI VGFILSFLLS FCGAVQPASL MP-GFW-TFM
(PRED) sace_35_7_3840  IGLALMILYM SPNLQSANVI VGFILSFLLS FCGAVQPASL MP-GFW-TFM
(PRED) sace_43_7_g0387 IGLALMILYM SPNLQSANVI VGFILSFLLS FCGAVQPASL MP-GFW-TFM
(PRED) sace_57_8_h0390 IGLALMILYM SPNLQSANVI VGFILSFLLS FCGAVQPASL MP-GFW-TFM
(PRED) sace_45_7_g0389 IGLALMILYM SPNLQSANVI VGFILSFLLS FCGAVQPASL MP-GFW-TFM
(PRED) sace_46_8_h0391 IGLALMILYM SPNLQSANVI VGFILSFLLS FCGAVQPASL MP-GFW-TFM
```

(PRED)	sace_23_7_3860	IGLALMILYM	SPNLQSANVI	VGFILSFLLS	FCGAVQPASL	MP-GFW-TFM
(PRED)	sace_21_7_3790	IGLALMILYM	SPNLQSANVI	VGFILSFLLS	FCGAVQPASL	MP-GFW-TFM
(PRED)	sace_8_73_bu001	IGLALMILYM	SPNLQSANVI	VGFILSFLLS	FCGAVQPASL	MP-GFW-TFM
(PRED)	sapa_1_8_h03820	IGLALMVLYM	SPNLQSANVI	VGFILSFLLS	FCGAVQPASL	MP-GFW-TFM
(PRED)	sapa_21_8_h0387	IGLALMVLYM	SPNLQSANVI	VGFILSFLLS	FCGAVQPASL	MP-GFW-TFM
(PRED)	sapa_20_8_h0386	IGLALMVLYM	SPNLQSANVI	VGFILSFLLS	FCGAVQPASL	MP-GFW-TFM
(PRED)	sapa_22_8_h0390	IGLALMVLYM	SPNLQSANVI	VGFILSFLLS	FCGAVQPASL	MP-GFW-TFM
(PRED)	sapa_25_8_h0387	IGLALMVLYM	SPNLQSANVI	VGFILSFLLS	FCGAVQPASL	MP-GFW-TFM
(PRED)	sapa_6_8_3750	IGLALMVLYM	SPNLQSANVI	VGFILSFLLS	FCGAVQPASL	MP-GFW-TFM
(PRED)	sapa_9_8_3720	IGLALMVLYM	SPNLQSANVI	VGFILSFLLS	FCGAVQPASL	MP-GFW-TFM
(PRED)	sapa_19_8_h0390	IGLALMVLYM	SPNLQSANVI	VGFILSFLLS	FCGAVQPASL	MP-GFW-TFM
(PRED)	sapa_24_8_h0385	IGLALMVLYM	SPNLQSANVI	VGFILSFLLS	FCGAVQPASL	MP-GFW-TFM
(PRED)	sapa_4_8_h03850	IGLALMVLYM	SPNLQSANVI	VGFILSFLLS	FCGAVQPASL	MP-GFW-TFM
(PRED)	sapa_10_8_3760	IGLALMVLYM	SPNLQSANVI	VGFILSFLLS	FCGAVQPASL	MP-GFW-TFM
(PRED)	sapa_13_8_h0382	IGLALMVLYM	SPNLQSANVI	VGFILSFLLS	FCGAVQPASL	MP-GFW-TFM
(PRED)	sapa_8_8_3750	IGLALMVLYM	SPNLQSANVI	VGFILSFLLS	FCGAVQPASL	MP-GFW-TFM
(PRED)	sapa_11_8_h0383	IGLALMVLYM	SPNLQSANVI	VGFILSFLLS	FCGAVQPASL	MP-GFW-TFM
(PRED)	sapa_5_8_3700	IGLALMVLYM	SPNLQSANVI	VGFILSFLLS	FCGAVQPASL	MP-GFW-TFM
(PRED)	sapa_16_8_h0389	IGLALMVLYM	SPNLQSANVI	VGFILSFLLS	FCGAVQPASL	MP-GFW-TFM
(PRED)	sapa_17_8_3730	IGLALMVLYM	SPNLQSANVI	VGFILSFLLS	FCGAVQPASL	MP-GFW-TFM
(PRED)	sapa_2_8_h03860	IGLALMVLYM	SPNLQSANVI	VGFILSFLLS	FCGAVQPASL	MP-GFW-TFM
(PRED)	sapa_7_8_3740	IGLALMVLYM	SPNLQSANVI	VGFILSFLLS	FCGAVQPASL	MP-GFW-TFM
(PRED)	sapa_23_8_h0385	IGLALMVLYM	SPNLQSANVI	VGFILSFLLS	FCGAVQPASL	MP-GFW-TFM
(PRED)	sapa_3_8_h03890	IGLALMVLYM	SPNLQSANVI	VGFILSFLLS	FCGAVQPASL	MP-GFW-TFM
(PRED)	sapa_18_8_3730	IGLALMVLYM	SPNLQSANVI	VGFILSFLLS	FCGAVQPASL	MP-GFW-TFM
(PRED)	sami_1_14_399	IGLALMVLYM	SPNLQSANVI	IGFVLSFLLS	FCGAVQPSFL	MP-GFW-TFM
(PRED)	sace_4_8_h03690	IGLALMILYM	SPNLQSANVI	VGFILSFLLS	FCGAVQPASL	MP-GFW-TFM
(PRED)	saku_1_14_404	IGLALVVLYM	SPNLQSANVI	VGFVLSFLLS	FCGAVQPASL	MP-GFW-TFM
(PRED)	sace_1_ynr070w	IGLALMILYM	SPNLQSANVI	VGFILSFLLS	FCGAVQPASL	MP-GFW-TFM
(PRED)	sace_49_8_h0383	IGLALMILYM	SPNLQSANVI	VGFILSFLLS	FCGAVQPASL	MP-GFW-TFM
(PRED)	saeu_1_2_b00130	VGLALMILYA	SPNLQSANVI	VGFLLSFLLS	FCGVVQPAFL	MP-GFW-TFM
(PRED)	sauv_1_7_3	VGLALMILYA	SPNLQSANVI	VGFLLSFLLS	FCGVVQPAFL	MP-GFW-TFM
(PRED)	sami_1_17_26	VGLALLVLYM	SPNLESANVI	MGFFLSMLIT	FCGVLQPSSL	MP-GFW-TFM
(PRED)	zyba_1_02055_AN	VALGLMILYM	SPNLPSANVL	MGLVLSMLIQ	FCGVTQPMSL	MP-GFW-TFM
(PRED)	zyba_1_07912	VALGLMILYM	SPNLPSANVL	MGLVLSMLIQ	FCGVTQPMSL	MP-GFW-TFM
(PRED)	zyba_2_2_b00600	VALGLMILYM	SPNLPSANVL	MGLVLSMLIQ	FCGVTQPMSL	MP-GFW-TFM
(PRED)	zyba_3_3_c03460	VALGLMILYM	SPNLPSANVL	MGLVLSMLIQ	FCGVTQPMSL	MP-GFW-TFM
(PRED)	zyba_1_04634	ISLGLMILYM	SPNLPAASVI	MGSVITMLIV	FSGVVQPVSL	MP-GFW-TFM
(PRED)	zyba_1_06675	ISLGLMILYM	SPNLPAASVI	MGVIVTILIV	FSGVVQPVSL	MP-GFW-TFM
(PRED)	zyba_3_2_b02230	ISLGLMILYM	SPNLPAASVI	MGVIVTILIV	FSGVVQPVSL	MP-GFW-TFM
(PRED)	zyba_2_1_a00860	ISLGLMILYM	SPNLPAASVI	MGVIVTILIV	FSGVVQPVSL	MP-GFW-TFM
(PRED)	zyro_1_a04114g	VGLGLTILYM	SPNLPSANVI	MGLILSMMIQ	FCGVTQPVSL	MP-GFW-TFM
(PRED)	zyro_1_b14762g	VGLGLMILYM	APNLPSANVI	MGLILSMMIQ	FCGVTQPMSL	MP-GFW-TFM
(PRED)	zyba_2_14_n0149	VALGLMVLYI	SPNLPSANVI	MGLVVSLLIQ	FCGVTQPMSL	MP-GFW-TFM
(PRED)	zyba_2_33_ag001	VALGLMVLYI	SPNLPSANVI	MGLVVSLLIQ	FCGVTQPMSL	MP-GFW-TFM
(PRED)	lath_1_a01914g	VGLGLMLLYA	APNLPSAGVL	LSLCLSFLIS	FCGVVQPASL	MP-GFW-TFM
(PRED)	lawa_1_23_5161	VGLGLMLIYA	APDLPSAGVL	LSLCLSFLIS	FCGVIQPASL	MP-GFW-TFM
(PRED)	klae_1_14_n0012	TGLGLAILYM	SPDLPSSNVI	MGFCLSLLIS	FCGVVQPVDL	MP-GFW-TFM
(PRED)	klla_1_d03432g	VGLGLAVLYM	SPDLPSSNVI	MGLLLSFMIT	FCGVVQPVNL	MP-GFW-TFM
(PRED)	klma_1_1_a01880	VGFGLAVLYM	SPDLPSSSVI	MGLLLSFMIS	FCGVVQPVNL	MP-GFW-TFM
(PRED)	klwi_1_33_ag001	VGFSLAVLYM	SPDLPSSNVI	MGLFLSMLIS	FCGVVQPVSL	MP-GFW-TFM
(PRED)	teph_1_a04220	IALGLLVLYL	SPDIQSANVL	MGLVISMLVA	FCGVVQPEYL	MP-GFW-TFM
(PRED)	vapo_1_1037_47	VALGLMVLYL	SPNLQSANVI	MGLTLSFLVA	FCGVVQPKTL	MP-GFW-TFM
(PRED)	pata_1_2_b05590	VSFSLWVVYM	SPDLPSANVL	FSLFFNFIVS	FCGVVQPEYL	MV-GFW-TFM
(PRED)	wian_1_3_c04380	ISLGLFIVYM	SPDLASSNVI	TGLFLAFMIS	FCGVTQPMSL	MP-GFW-HFM
(PRED)	wian_1_3_c04390	ISLGLFIVYM	SPDLASSTVI	TGLFLTMMIS	FCGVVQPMSL	MP-GFW-TFM
(PRED)	wian_1_7_g01010	VSLGLLIVYM	APDLASSTVI	TGLCFSFMIS	FCGVVQPVDL	MP-GFW-TFM
(PRED)	bain_1_1_a00100	VSFGLLVIYI	SPDLPSANII	SALCFTFMIS	FCGVVQPFKL	M--GWW-KWM
(PRED)	bain_1_17_q0038	VSFGLLVIYI	SPDLPSANVI	SALCFTFMIS	FCGVMQPFKL	M--GWW-KWM
(PRED)	bain_1_8_h00410	ISYGFLILTL	SPDVPSAAIL	TTLGFALILT	FCGVLQPAFL	MPVFWR-RFM
(PRED)	caal_1_19_5759	VSFGLMVSYV	SPDIESASVI	VSFLYTFIVS	FSGVVQPVNL	MP-GFW-TFM
(PRED)	caal_11_25_y002	VSFGLMVSYV	SPDIESASVI	VSFLYTFIVS	FSGVVQPVNL	MP-GFW-TFM
(PRED)	caal_4_4_d03320	VSFGLMVSYV	SPDIESASVI	VSFLYTFIVS	FSGVVQPVNL	MP-GFW-TFM
(PRED)	caal_12_26_z005	VSFGLMVSYV	SPDIESASVI	VSFLYTFIVS	FSGVVQPVNL	MP-GFW-TFM
(PRED)	caal_5_30_ad005	VSFGLMVSYV	SPDIESASVI	VSFLYTFIVS	FSGVVQPVNL	MP-GFW-TFM
(PRED)	caal_8_3_c03320	VSFGLMVSYV	SPDIESASVI	VSFLYTFIVS	FSGVVQPVNL	MP-GFW-TFM
(PRED)	caal_6_4_d03280	VSFGLMVSYV	SPDIESASVI	VSFLYTFIVS	FSGVVQPVNL	MP-GFW-TFM
(PRED)	caal_10_3_c0334	VSFGLMVSYV	SPDIESASVI	VSFLYTFIVS	FSGVVQPVNL	MP-GFW-TFM
(PRED)	caal_3_29_ac005	VSFGLMVSYV	SPDIESASVI	VSFLYTFIVS	FSGVVQPVNL	MP-GFW-TFM
(PRED)	caal_2_04989	VSFGLMVSYV	SPDIESASVI	VSFLYTFIVS	FSGVVQPVNL	MP-GFW-TFM
(PRED)	cadu_1_64350	VSFGLMVSYV	SPDVESASVI	VSFLYTFIVS	FSGVVQPVDL	MP-GFW-TFM
(PRED)	caor_1_h02090	VSFGLMVLYI	APNLQSAAVL	VSFLYTFIVA	FSGIVQPVNL	MP-GFW-TFM
(PRED)	capa_1_600750	VSFGLMVLYI	APDLQSAAVL	VSFLYSFIVA	FSGIVQPVNL	MP-GFW-TFM
(PRED)	loel_1_04930	FVWFXGVVHC	PRFGECSRPR	LLPLYVHC-C	XSGVVQPVQL	MP-GFW-TFM
(PRED)	spar_1_5_e03260	ASFGLMLLYI	APDLESAAVL	VSFFYTFIVS	FSGVVQPVSL	MP-GFW-TFM

(PRED)	sppa_1_7_g03160	ASFGLMLLYI	APDLESAAVL	VSFFYSFIVS	FSGVVQPVTL	MP-GFW-TFM
(PRED)	catr_1_01205	VSFSAMILYI	APDLESAAVI	FSFLYTFVVA	FSGVVQPVNL	MP-GFW-TFM
(PRED)	catr_1_05498	ITFAAMILFI	APDLESASVI	FSFLYTFIVA	FSGIVQPTNL	MP-GFW-TFM
(PRED)	catr_1_05971	VTFSAMILFV	APDLESASVI	FSFLYTFIVA	FSGVVQPVDV	MP-GFW-TFM
(PRED)	deha_1_a03696g	VTFGLLILYI	APDLESAAVL	TSFFYTFVVA	FSGVVQPVNL	MP-GFW-TFM
(PRED)	deha_2_5_e00720	VTFGLLILYI	APDLESAAVL	TSFFYTFVVA	FSGVVQPVNL	MP-GFW-TFM
(PRED)	scst_1_3_c02890	VTFACLVLYV	APDLETAAVL	VSFLYTFIVA	FSGVVQPVHL	MP-GFW-TFM
(PRED)	mebi_1_8_h00300	ISFGLMLAYF	APDVQSAAVL	VSFFYSFIVS	FSGVVQPVSL	MP-GFW-TFM
(PRED)	lakl_1_h21010g	IGFGLLVLYI	SPDLPSAAII	MGLSLSFMIG	FCGVVQPVRL	MP-GFW-TFM
(PRED)	caar_1_13_m0142	VSLALAVVYA	SPDLPSANVL	VGLLFNFIVS	FCGVVQPPSS	MP-HFW-KFM
(PRED)	caar_1_14_n0143	ISLSLAVVYA	SPDLPSANVL	VGLLFNFIIS	FCGVVQSPSL	MP-GFW-RFM
(PRED)	hapo_1_1_a07220	VSLALAVVYA	SPDLPSANVL	IGLVFNFIIS	FCGVVQNPNL	MP-GFW-HFM
(PRED)	ogpa_1_1_a01680	VSLGLAIVYA	APDLPSANVL	IGLVFNFIVS	FCGVVQNPSL	MP-GFW-HFM
(PRED)	piku_1_96_cr001	ISFSLTIVYA	APDLPSANVL	TGLGWSFVIA	FCGVLQNPHL	LP-GFW-KFM
(PRED)	pime_1_4_d03240	ISLGLAIVYV	SPDLPSANVL	TGLALNFIIS	FCGVVQSPKL	MP-GFW-KFM
(PRED)	pime_1_1_a12110	ITLALATVYV	SPDLPSANVL	MALIFSFIIA	FCGVVQRPNL	MP-GFW-KFM
(PRED)	piku_1_227_hs00	PSLALAILYP	SPDLPSANVI	MGLIFSFTMA	FCGVFQPPSL	MP-GFW-KFM
(PRED)	pime_1_5_e05800	PALALAILYP	SPDLPSANVI	MGLIFSFTMA	FCGVFQIPAL	MP-GFW-KFM
(PRED)	pime_1_1_a07690	PAFALMILYA	APDLPSANIL	LGLFFSFTIS	FCGVVQRPQL	MP-GFW-KFM
(PRED)	debr_2_5_e03380	ISFALAVIYF	SPDLPSANVL	CGMLLNFLIA	FCGVIQPPSL	LP-GFW-KFM
(PRED)	kopa_1_2_b10040	ISFALATVYF	APDLPTANVI	LSFLFNFIFA	FCGVVQPVDM	MP-GFW-TFM
(PRED)	kopa_2_7_g00500	ISFALATVYF	APDLPTANVI	LSFLFNFIFA	FCGVVQPVDM	MP-GFW-TFM
(PRED)	asru_1_13_m0119	ISFGLWVIYM	APDLPSASII	TSLLFSFMLN	FCGVLQQQSL	MP-GFW-TFM
(PRED)	asru_1_15_o0045	ISFGLWIVYF	LPDLASAEAI	YGLLFTFMFA	FAGIVQPYRY	IP-TFW-KFM
(PRED)	wian_1_1_a02920	TSFSVGVYYF	SPDLATANIL	GTILIVFMLT	FSGVVQPLSN	FP-KFW-TFM
(PRED)	wian_1_1_a02930	CTFAIGIYYV	SPDLPSAGLL	GSVVTVFMFA	FAGVLQPYSN	LP-GFW-RFM
		1710	1720	1730	1740	1750
(PRED)	asac_1_6_f03560	YRLSPYTYFT	QNFLGIMLHD	RPVRCSSKEF	SFLDPPSGST	CGEYMQPFVD
(PRED)	ergo_1_abr125c	YRLSPYTYFT	QNFLGIMLHD	RPVRCNSREF	SFLDPPNGVT	CGEYMQPFVN
(PRED)	ercy_1_3604	FRASPYTYFT	QNLLGLLLHD	KPVVCSPKEL	SYLNPPQGMT	CGDYMSDFLK
(PRED)	cagl_1_i04862g	WKTSPYTYFV	QNMVGILLHN	KPVICRKKEL	SIFDPPAGQT	CQEFTQAFLD
(PRED)	kaaf_1_c00830	WKASPYTYFV	QNIVGIMLHK	KEVVCSTKEL	NYFDPPSGQT	CGSYMEEYFK
(PRED)	kana_1_k01350	WKASPYTYFV	QNLVGVLLHD	KPVICKKKEL	NYFEPPSGQT	CGEYMGPFLQ
(PRED)	saar_1_2_b02590	WKASPYTYFV	QNLVGIMLHK	KPVVCKKKEL	NYFNPPNGST	CGEYMKSFLE
(PRED)	sace_1_ydr011w	WKASPYTYFV	QNLVGIMLHK	KPVVCKKKEL	NYFNPPNGST	CGEYMKPFLE
(PRED)	sace_16_1_a0238	WKASPYTYFV	QNLVGIMLHK	KPVVCKKKEL	NYFNPPNGST	CGEYMKPFLE
(PRED)	sace_45_1_a0242	WKASPYTYFV	QNLVGIMLHK	KPVVCKKKEL	NYFNPPNGST	CGEYMKPFLE
(PRED)	sace_48_1_a0238	WKASPYTYFV	QNLVGIMLHK	KPVVCKKKEL	NYFNPPNGST	CGEYMKPFLE
(PRED)	sace_60_4_d0244	WKASPYTYFV	QNLVGIMLHK	KPVVCKKKEL	NYFNPPNGST	CGEYMKPFLE
(PRED)	sace_52_1_a0240	WKASPYTYFV	QNLVGIMLHK	KPVVCKKKEL	NYFNPPNGST	CGEYMKPFLE
(PRED)	sace_46_1_a0240	WKASPYTYFV	QNLVGIMLHK	KPVVCKKKEL	NYFNPPNGST	CGEYMKPFLE
(PRED)	sace_25_1_a0240	WKASPYTYFV	QNLVGIMLHK	KPVVCKKKEL	NYFNPPNGST	CGEYMKPFLE
(PRED)	sace_24_1_2300	WKASPYTYFV	QNLVGIMLHK	KPVVCKKKEL	NYFNPPNGST	CGEYMKPFLE
(PRED)	sace_47_1_a0240	WKASPYTYFV	QNLVGIMLHK	KPVVCKKKEL	NYFNPPNGST	CGEYMKPFLE
(PRED)	sace_7_1_a02410	WKASPYTYFV	QNLVGIMLHK	KPVVCKKKEL	NYFNPPNGST	CGEYMKPFLE
(PRED)	sace_59_110_df0	WKASPYTYFV	QNLVGIMLHK	KPVVCKKKEL	NYFNPPNGST	CGEYMKPFLE
(PRED)	sace_56_1_a0202	WKASPYTYFV	QNLVGIMLHK	KPVVCKKKEL	NYFNPPNGST	CGEYMKPFLE
(PRED)	sace_40_1_a0239	WKASPYTYFV	QNLVGIMLHK	KPVVCKKKEL	NYFNPPNGST	CGEYMKPFLE
(PRED)	sace_15_1_a0242	WKASPYTYFV	QNLVGIMLHK	KPVVCKKKEL	NYFNPPNGST	CGEYMKPFLE
(PRED)	sace_37_1_a0243	WKASPYTYFV	QNLVGIMLHK	KPVVCKKKEL	NYFNPPNGST	CGEYMKPFLE
(PRED)	sace_9_1_a02440	WKASPYTYFV	QNLVGIMLHK	KPVVCKKKEL	NYFNPPNGST	CGEYMKPFLE
(PRED)	sace_22_1_2300	WKASPYTYFV	QNLVGIMLHK	KPVVCKKKEL	NYFNPPNGST	CGEYMKPFLE
(PRED)	sace_29_1_2290	WKASPYTYFV	QNLVGIMLHK	KPVVCKKKEL	NYFNPPNGST	CGEYMKPFLE
(PRED)	sace_34_1_2320	WKASPYTYFV	QNLVGIMLHK	KPVVCKKKEL	NYFNPPNGST	CGEYMKPFLE
(PRED)	sace_58_25_y007	WKASPYTYFV	QNLVGIMLHK	KPVVCKKKEL	NYFNPPNGST	CGEYMKPFLE
(PRED)	sace_23_1_2290	WKASPYTYFV	QNLVGIMLHK	KPVVCKKKEL	NYFNPPNGST	CGEYMKPFLE
(PRED)	sace_6_120_dp00	WKASPYTYFV	QNLVGIMLHK	KPVVCKKKEL	NYFNPPNGST	CGEYMKPFLE
(PRED)	sace_57_1_a0241	WKASPYTYFV	QNLVGIMLHK	KPVVCKKKEL	NYFNPPNGST	CGEYMKPFLE
(PRED)	sace_17_1_a0241	WKASPYTYFV	QNLVGIMLHK	KPVVCKKKEL	NYFNPPNGST	CGEYMKPFLE
(PRED)	sace_21_1_2310	WKASPYTYFV	QNLVGIMLHK	KPVVCKKKEL	NYFNPPNGST	CGEYMKPFLE
(PRED)	sace_49_1_a0246	WKASPYTYFV	QNLVGIMLHK	KPVVCKKKEL	NYFNPPNGST	CGEYMKPFLE
(PRED)	sace_8_2_b02430	WKASPYTYFV	QNLVGIMLHK	KPVVCKKKEL	NYFNPPNGST	CGEYMKPFLE
(PRED)	sace_31_1_2300	WKASPYTYFV	QNLVGIMLHK	KPVVCKKKEL	NYFNPPNGST	CGEYMKPFLE
(PRED)	sace_50_1_a0241	WKASPYTYFV	QNLVGIMLHK	KPVVCKKKEL	NYFNPPNGST	CGEYMKPFLE
(PRED)	sace_4_1_a02360	WKASPYTYFV	QNLVGIMLHK	KPVVCKKKEL	NYFNPPNGST	CGEYMKPFLE
(PRED)	sace_2_1_a02390	WKASPYTYFV	QNLVGIMLHK	KPVVCKKKEL	NYFNPPNGST	CGEYMKPFLE
(PRED)	sace_5_33_ag005	WKASPYTYFV	QNLVGIMLHK	KPVVCKKKEL	NYFNPPNGST	CGEYMKPFLE
(PRED)	sapa_11_1_a0247	WKASPYTYFV	QNLVGIMLHK	KPVVCKKKEL	NYFNPPNGST	CGEYMKPFLE
(PRED)	sapa_25_1_a0246	WKASPYTYFV	QNLVGIMLHK	KPVVCKKKEL	NYFNPPNGST	CGEYMKPFLE
(PRED)	sapa_4_1_a02470	WKASPYTYFV	QNLVGIMLHK	KPVVCKKKEL	NYFNPPNGST	CGEYMKPFLE
(PRED)	sapa_5_1_2350	WKASPYTYFV	QNLVGIMLHK	KPVVCKKKEL	NYFNPPNGST	CGEYMKPFLE
(PRED)	sapa_9_1_2360	WKASPYTYFV	QNLVGIMLHK	KPVVCKKKEL	NYFNPPNGST	CGEYMKPFLE
(PRED)	sapa_14_1_a0244	WKASPYTYFV	QNLVGIMLHK	KPVVCKKKEL	NYFNPPNGST	CGEYMKPFLE
(PRED)	sapa_8_1_2350	WKASPYTYFV	QNLVGIMLHK	KPVVCKKKEL	NYFNPPNGST	CGEYMKPFLE

(PRED)	sapa_17_1_2380	WKASPYTYFV	QNLVGIMLHK	KPVVCKKKEL	NYFNPPNGST	CGEYMKPFLE
(PRED)	sapa_7_1_2370	WKASPYTYFV	QNLVGIMLHK	KPVVCKKKEL	NYFNPPNGST	CGEYMKPFLE
(PRED)	sapa_2_1_a02460	WKASPYTYFV	QNLVGIMLHK	KPVVCKKKEL	NYFNPPNGST	CGEYMKPFLE
(PRED)	sapa_23_1_a0248	WKASPYTYFV	QNLVGIMLHK	KPVVCKKKEL	NYFNPPNGST	CGEYMKPFLE
(PRED)	sapa_3_1_a02470	WKASPYTYFV	QNLVGIMLHK	KPVVCKKKEL	NYFNPPNGST	CGEYMKPFLE
(PRED)	sapa_18_1_2390	WKASPYTYFV	QNLVGIMLHK	KPVVCKKKEL	NYFNPPNGST	CGEYMKPFLE
(PRED)	sami_1_4_244	WKASPYTYFV	QNLVGIMLHK	KPVVCKKKEL	NYFNPPNGST	CGEYMKPFLE
(PRED)	saku_1_4_262	WKASPYTYFV	QNLVGIMLHK	KPVVCKKKEL	NYFNPPNGST	CGEYMKQFLE
(PRED)	saba_1_58_bf002	WKASPYTYFV	QNLVGIMLHK	KPVVCKKKEL	NYFNPPNGST	CGEYMKSFLE
(PRED)	saeu_1_4_d02400	WKASPYTYFV	QNLVGIMLHK	KPVVCKKKEL	NYFNPPDGST	CGEYMKPFLE
(PRED)	naca_1_e01640	WKASPYTYFV	QNMVGIMLHK	KPVVCSKKEL	NYFNPPSGQT	CGQYMEKFLS
(PRED)	nada_1_g01850	WKASPLTYFV	QNMIGIMLHD	KKVVCSKKEL	NYFNPPAGQT	CGEYMDPFLS
(PRED)	naca_1_e01630	WKTSPYTYFV	QNLVGIMLHE	KPVICTKKEL	NFFDPPAGQT	CGQYMAPFLS
(PRED)	nada_1_g01840	WRTSPYTYYV	QNLVGIMLHK	KPVICSEKEL	NYFAPPSGQT	CGEYMEPFLS
(PRED)	kaaf_1_c00820	WKTSPFTYFV	QNLVGIMLHT	KAVVCTSNEL	NYFNPPSGQT	CGEYMQNYLQ
(PRED)	teph_1_m00640	WKASPYTYFV	QNLVGIMLHE	KPVICSKKEL	SYFNPPSGQT	CGQYLERFLE
(PRED)	vapo_1_1036_28	WKASPYTYFV	QNLVGIMLHE	KPVICSKKEL	NYFAPPSGMT	CGEYMEPFLN
(PRED)	tebl_1_i01760	WKTSPYTYFV	QNMVGILLHK	KPVRCKKKEF	NYFNPPEGQT	CGQYMEAFLK
(PRED)	tode_1_d04040	WKASPYTYFV	QNLLGIVLHE	KPVVCKKKEL	AFFDPPSGQT	CGDYMEEFLS
(PRED)	naca_1_e01650	WKASPYTYFV	QNMVGIMLHK	KPVVCSKKEL	NYFNPPSGQT	CGQYMERFLS
(PRED)	tebl_1_g02820	WKASPYTYFV	QNLMGIMLHK	KPVICTKSEL	SYFEPPSGQT	CGQYMEKFFQ
(PRED)	lakl_1_c11616g	WKVSPYTYFT	QNLIGIMLHD	KKVICKAKEY	SYLDPPTGKT	CGEYMEPFLK
(PRED)	saar_1_8_h03780	WKLSPYTYFL	QNFVGLTMHE	KPVRCSNKEL	SLFNPPAGQT	CAEFTEPFFR
(PRED)	sace_14_7_g0015	WKLSPYTYFL	QNLVGLLMHD	KPVRCSKKEL	SLFNPPVGQT	CGEFTKPFFE
(PRED)	sace_15_7_g0387	WKLSPYTYFL	QNLVGLLMHD	KPVRCSKKEL	SLFNPPVGQT	CGEFTKPFFE
(PRED)	sace_24_8_3780	WKLSPYTYFL	QNLVGLLMHD	KPVRCSKKEL	SLFNPPVGQT	CGEFTKPFFE
(PRED)	sace_40_8_h0383	WKLSPYTYFL	QNLVGLLMHD	KPVRCSKKEL	SLFNPPVGQT	CGEFTKPFFE
(PRED)	sace_6_169_fm00	WKLSPYTYFL	QNLVGLLMHD	KPVRCSKKEL	SLFNPPVGQT	CGEFTKPFFE
(PRED)	sace_19_7_3840	WKLSPYTYFL	QNLVGLLMHD	KPVRCSKKEL	SLFNPPVGQT	CGEFTKPFFE
(PRED)	sace_32_7_3770	WKLSPYTYFL	QNLVGLLMHD	KPVRCSKKEL	SLFNPPVGQT	CGEFTKPFFE
(PRED)	sace_56_17_q011	WKLSPYTYFL	QNLVGLLMHD	KPVRCSKKEL	SLFNPPVGQT	CGEFTKPFFE
(PRED)	sace_5_78_bz001	WKLSPYTYFL	QNLVGLLMHD	KPVRCSKKEL	SLFNPPVGQT	CGEFTKPFFE
(PRED)	sace_2_8_h03860	WKLSPYTYFL	QNLVGLLMHD	KPVRCSKKEL	SLFNPPVGQT	CGEFTKPFFE
(PRED)	sace_53_29_ac00	WKLSPYTYFL	QNLVGLLMHD	KPVRCSKKEL	SLFNPPVGQT	CGEFTKPFFE
(PRED)	sace_17_7_g0393	WKLSPYTYFL	QNLVGLLMHD	KPVRCSKKEL	SLFNPPVGQT	CGEFTKPFFE
(PRED)	sace_25_7_g0388	WKLSPYTYFL	QNLVGLLMHD	KPVRCSKKEL	SLFNPPVGQT	CGEFTKPFFE
(PRED)	sace_37_7_g0385	WKLSPYTYFL	QNLVGLLMHD	KPVRCSKKEL	SLFNPPVGQT	CGEFTKPFFE
(PRED)	sace_9_7_g00180	WKLSPYTYFL	QNLVGLLMHD	KPVRCSKKEL	SLFNPPVGQT	CGEFTKPFFE
(PRED)	sace_60_6_f0335	WKLSPYTYFL	QNLVGLLMHD	KPVRCSKKEL	SLFNPPVGQT	CGEFTKPFFE
(PRED)	sace_59_336_lx0	WKLSPYTYFL	QNLVGLLMHD	KPVRCSKKEL	SLFNPPVGQT	CGEFTKPFFE
(PRED)	sace_31_7_3780	WKLSPYTYFL	QNLVGLLMHD	KPVRCSKKEL	SLFNPPVGQT	CGEFTKPFFE
(PRED)	sace_34_8_3770	WKLSPYTYFL	QNLVGLLMHD	KPVRCSKKEL	SLFNPPVGQT	CGEFTKPFFE
(PRED)	sace_58_71_bs00	WKLSPYTYFL	QNLVGLLMHD	KPVRCSKKEL	SLFNPPVGQT	CGEFTKPFFE
(PRED)	sace_7_7_g03880	WKLSPYTYFL	QNLVGLLMHD	KPVRCSKKEL	SLFNPPVGQT	CGEFTKPFFE
(PRED)	sace_35_7_3840	WKLSPYTYFL	QNLVGLLMHD	KPVRCSKKEL	SLFNPPVGQT	CGEFTKPFFE
(PRED)	sace_43_7_g0387	WKLSPYTYFL	QNLVGLLMHD	KPVRCSKKEL	SLFNPPVGQT	CGEFTKPFFE
(PRED)	sace_57_8_h0390	WKLSPYTYFL	QNLVGLLMHD	KPVRCSKKEL	SLFNPPVGQT	CGEFTKPFFE
(PRED)	sace_45_7_g0389	WKLSPYTYFL	QNLVGLLMHD	KPVRCSKKEL	SLFNPPVGQT	CGEFTKPFFE
(PRED)	sace_46_8_h0391	WKLSPYTYFL	QNLVGLLMHD	KPVRCSKREL	SLFNPPAGQT	CGEFTKPFFE
(PRED)	sace_23_7_3860	WKLSPYTYFL	QNLVGLLMHD	KPVRCSKKEL	SLFNPPVGQT	CGEFTKPFFE
(PRED)	sace_21_7_3790	WKLSPYTYFL	QNLVGLLMHD	KPVRCSKKEL	SLFNPPVGQT	CGEFTKPFFE
(PRED)	sace_8_73_bu001	WKLSPYTYFL	QNLVGLLMHD	KPVXCSKKEL	SLFNPPVGQT	CGEFTKPFFE
(PRED)	sapa_1_8_h03820	WKLSPYTYFL	QNLVGLLMHD	KPVRCSEKEL	SVFNPPIGQT	CGEFTKPFFE
(PRED)	sapa_21_8_h0387	WKLSPYTYFL	QNLVGLLMHD	KPVRCSEKEL	SVFNPPIGQT	CGEFTKPFFE
(PRED)	sapa_20_8_h0386	WKLSPYTYFL	QNLVGLLMHD	KPVRCSEKEL	SVFNPPIGQT	CGEFTKPFFE
(PRED)	sapa_22_8_h0390	WKLSPYTYFL	QNLVGLLMHD	KPVRCSEKEL	SVFNPPIGQT	CGEFTKPFFE
(PRED)	sapa_25_8_h0387	WKLSPYTYFL	QNLVGLLMHD	KPVRCSEKEL	SVFNPPIGQT	CGEFTKPFFE
(PRED)	sapa_6_8_3750	WKLSPYTYFL	QNLVGLLMHD	KPVRCSEKEL	SVFNPPIGQT	CGEFTKPFFE
(PRED)	sapa_9_8_3720	WKLSPYTYFL	QNLVGLLMHD	KPVRCSEKEL	SVFNPPIGQT	CGEFTKPFFE
(PRED)	sapa_19_8_h0390	WKLSPYTYFL	QNLVGLLMHD	KPVRCSEKEL	SVFNPPIGQT	CGEFTKPFFE
(PRED)	sapa_24_8_h0385	WKLSPYTYFL	QNLVGLLMHD	KPVRCSEKEL	SVFNPPIGQT	CGEFTKPFFE
(PRED)	sapa_4_8_h03850	WKLSPYTYFL	QNLVGLLMHD	KPVRCSEKEL	SVFNPPIGQT	CGEFTKPFFE
(PRED)	sapa_10_8_3760	WKLSPYTYFL	QNLVGLLMHD	KPVRCSKKEL	SVFNPPIGQT	CGEFTKPFFE
(PRED)	sapa_13_8_h0382	WKLSPYTYFL	QNLVGLLMHD	KPVRCSKKEL	SVFNPPIGQT	CGEFTNPFFE
(PRED)	sapa_8_8_3750	WKLSPYTYFL	QNLVGLLMHD	KPVRCSKKEL	SVFNPPIGQT	CGEFTNPFFE
(PRED)	sapa_11_8_h0383	WKLSPYTYFL	QNLVGLLMHD	KPVRCSEKEL	SVFNPPIGQT	CGEFTKPFFE
(PRED)	sapa_5_8_3700	WKLSPYTYFL	QNLVGLLMHD	KPVRCSEKEL	SVFNPPIGQT	CGEFTKPFFE
(PRED)	sapa_16_8_h0389	WKLSPYTYFL	QNLIGLLMHD	KPVRCSKNEL	SVFNPPIGQT	CGEFTKPFFE
(PRED)	sapa_17_8_3730	WKLSPYTYFL	QNLIGLLMHD	KPVRCSKNEL	SVFNPPIGQT	CGEFTKPFFE
(PRED)	sapa_2_8_h03860	WKLSPYTYFL	QNLIGLLMHD	KPVRCSKNEL	SVFNPPIGQT	CGEFTKPFFE
(PRED)	sapa_7_8_3740	WKLSPYTYFL	QNLIGLLMHD	KPVRCSKNEL	SVFNPPIGQT	CGEFTKPFFE
(PRED)	sapa_23_8_h0385	WKLSPYTYFL	QNLIGLLMHD	KPVRCSKNEL	SVFNPPIGQT	CGEFTKPFFE
(PRED)	sapa_3_8_h03890	WKLSPYTYFL	QNLIGLLMHD	KPVRCSKNEL	SVFNPPIGQT	CGEFTKPFFE
(PRED)	sapa_18_8_3730	WKLSPYTYFL	QNLVGLLMHD	KPVRCSKNEL	SVFNPPIGQT	CGEFTKPFFE
(PRED)	sami_1_14_399	WKLSPYTYFL	QNFIGLLMHD	TPVICSEKEL	SVFNPPIGQT	CGEFTKPFFE

(PRED)	sace_4_8_h03690	WKLSPYTYFL	QNLVGLLMHD	KPVRCSKKEL	SLFNPPVGQT	CGEFTKPFFE
(PRED)	saku_1_14_404	WKLSPYTYFL	QNFVSLLMHK	KPVRCSKKEL	SFFNPPMGQT	CGEFTEPFFK
(PRED)	sace_1_ynr070w	WKLSPYTYFL	QNLVGLLMHD	KPVRCSKKEL	SLFNPPVGQT	CGEFTKPFFE
(PRED)	sace_49_8_h0383	WKLSPYTYFL	QNLVGLLMHD	KPVXCSKKEL	SLFNPPVGQT	CGEFTKPFFE
(PRED)	saeu_1_2_b00130	WKLSPYTYFL	QNFVGLMMHE	KPVKCSEKEL	SVFNPPTGQT	CGEFTKPFFK
(PRED)	sauv_1_7_3	WKLSPYTYFL	QNLVGLMMHE	KPVKCSKKEF	SVFNPPVGQT	CGEFTKPYFN
(PRED)	sami_1_17_26	WKLSPYTYFV	QNLIGLMMHK	KPVRCSKKEL	SIFNPPAGQT	CGEFTKPFFK
(PRED)	zyba_1_02055_AN	WKASPYTYFI	QNMVAIVLHK	KPVVCKKKEL	SYFDPPQGKT	CGQYMENFLK
(PRED)	zyba_1_07912	WKASPYTYFI	QNMVAILLHK	KPVVCKKKEL	SYFDPPQGKT	CGQYMENFLK
(PRED)	zyba_2_2_b00600	WKASPYTYFI	QNMVAIVLHK	KPVVCKKKEL	SYFDPPQGKT	CGQYMENFLK
(PRED)	zyba_3_3_c03460	WKASPYTYFI	QNMVAIVLHK	KPVVCKKKEL	SYFDPPQGKT	CGQYMENFLK
(PRED)	zyba_1_04634	WKVSPYTYFI	QNMVAIVLHK	KPVVCKTKEL	SFFDPPQGST	CGQYMESFLQ
(PRED)	zyba_1_06675	WKVSPYTYFI	QNMVAIVLHK	KPVVCKTKEL	SLFDPPQGET	CGQYMENFLQ
(PRED)	zyba_3_2_b02230	WKVSPYTYFI	QNMVAIVLHK	KPVVCKTKEL	SLFDPPQGET	CGQYMENFLQ
(PRED)	zyba_2_1_a00860	WKVSPYTYFI	QNMVAIVLHK	KPVVCKTKEL	SLFDPPQGET	CGQYMENFLQ
(PRED)	zyro_1_a04114g	WKVSPFTYFI	QNLVAIVLHK	KPVVCKKKEL	NYFDPPQGKT	CGEYMNGFMK
(PRED)	zyro_1_b14762g	WKASPYTYFI	QNLVAIVLHK	KPVVCKKKEL	NYFDPPQGKT	CGEYMHTFLK
(PRED)	zyba_2_14_n0149	WKASPYTYFI	QNLVAIVLHK	KPVKCKKKEL	SFFDPPAGKT	CGEYMERFLK
(PRED)	zyba_2_33_ag001	WKASPYTYFI	QNLVAIVLHK	KPVECKKKEL	SYFDPPAGET	CGEYMEKFLQ
(PRED)	lath_1_a01914g	WKASPYTYFV	QNAISLVLHK	KPVRCKKNEM	SYVNPPAGQT	CGEYMSAFLK
(PRED)	lawa_1_23_5161	WKASPYTYFV	QNAVSIVLNN	KPVRCRKKEM	SYLNPPEGQT	CGEYMADFLK
(PRED)	klae_1_14_n0012	WKVSPYTYVI	QNLVGLMLHN	KAVICTKSEL	SYFNPPSGET	CGSYMQPFFD
(PRED)	klla_1_d03432g	WKVSPYTYLI	QNFVSLMLHD	KTVICTDKEL	SYFNPPPGST	CGSYMTDYFT
(PRED)	klma_1_1_a01880	WKVSPYTYFI	QNLVSFMLHN	KEVKCTKKEL	SYFEPPSGET	CGSYMKSYLS
(PRED)	klwi_1_33_ag001	WKVSPFTYFV	QNLVSFMLHN	KKVVCSKKEL	SFLTPPSGET	CGSFMKSYLD
(PRED)	teph_1_a04220	FKASPYTYFV	QNLVGIMLHE	KPVICTSKEY	SYFNPPPGQT	CGEYMQVYLN
(PRED)	vapo_1_1037_47	WKASPYTYFV	QNLISIMLHD	KPVVCKEKEL	SFLDPPTGLT	CGEYMAPFLT
(PRED)	pata_1_2_b05590	YKISPFTYFV	QTMVSLVLHD	REVHCATDEL	SILQPPSGET	CGSYLEEYLS
(PRED)	wian_1_3_c04380	WKVSPLTYVI	QTLMALVLHD	KPVICAKDEY	SYFEPPSGMT	CQEFAGAYVE
(PRED)	wian_1_3_c04390	WKVSPLTYVI	QTLLSLILHE	RKIRCGKDEY	NYFEPPSGMT	CQDFAGPFVN
(PRED)	wian_1_7_g01010	WKVSPLTYVV	QTLVALVLHE	KKVECSHEEY	NFFEPPSGLT	CQEFAGTYVT
(PRED)	bain_1_1_a00100	YHVSPYTYFV	ANFVMLVLRD	RKVECSSVEM	AYFNPPNGQT	CQEYAGNYIT
(PRED)	bain_1_17_q0038	YHVSPYTYFV	ASFVMLVLRD	RKVNCSSVEM	AYFNPPDGQT	CQEYAGIYVT
(PRED)	bain_1_8_h00410	YHVSPYTYLV	SSLVSNVLHD	RPVVCSKVEM	SYLNPPAGLT	CSQYLAPFVQ
(PRED)	caal_1_19_5759	NKVSPYTYFI	QNLVSSFLHD	RTIRCNAKEL	SYFNPPSGQT	CKEFASAFIS
(PRED)	caal_11_25_y002	NKVSPYTYFI	QNLVSSFLHD	RTIRCNAKEL	SYFNPPSGQT	CKEFASAFIS
(PRED)	caal_4_4_d03320	NKVSPYTYFI	QNLVSSFLHD	RTIRCNAKEL	SYFNPPSGQT	CKEFASAFIS
(PRED)	caal_12_26_z005	NKVSPYTYFI	QNLVSSFLHD	RTIRCNAKEL	SYFNPPSGQT	CKEFASAFIS
(PRED)	caal_5_30_ad005	NKVSPYTYFI	QNLVSSFLHD	RTIRCNAKEL	SYFNPPSGQT	CKEFASAFIS
(PRED)	caal_8_3_c03320	NKVSPYTYFI	QNLVSSFLHD	RTIRCNAKEL	SYFNPPSGQT	CKEFASAFIS
(PRED)	caal_6_4_d03280	NKVSPYTYFI	QNLVSSFLHD	RTIRCNAKEL	SYFNPPSGQT	CKEFASAFIS
(PRED)	caal_10_3_c0334	NKVSPYTYFI	QNLVSSFLHD	RTIRCNAKEL	SYFNPPSGQT	CKEFASAFIS
(PRED)	caal_3_29_ac005	NKVSPYTYFI	QNLVSSFLHD	RTIRCNAKEL	SYFNPPSGQT	CKEFASAFIS
(PRED)	caal_2_04989	NKVSPYTYFI	QNLVSSFLHD	RTIRCNAKEL	SYFNPPSGQT	CKEFASAFIS
(PRED)	cadu_1_64350	NKVSPYTYFI	QNLVSSFLHD	RTIRCNAKEL	SYFNPPSGET	CKEFASAFIS
(PRED)	caor_1_h02090	YKLSPYTYFI	QNLVSSFLHR	REIHCSDKEM	AFFNPPAGKT	CGEFAEAFVN
(PRED)	capa_1_600750	YKLSPYTYFI	QNLVSSFLHR	REIHCSDKEM	AFFNPPSGQT	CGEFAKAYVQ
(PRED)	loel_1_04930	NKVSPYTYFI	QNLVSSFLHG	RTIRCSDKEL	AFFDPPSGQT	CAEFAGDFLK
(PRED)	spar_1_5_e03260	YKVSAYTYFI	QNLVTSFLHG	RPIHCSNQEL	SFFNPPADQT	CGEFVAPFLE
(PRED)	sppa_1_7_g03160	YKVSPYTYFI	QNLVTSFLHG	RPIHCSDQEL	SFFNPPAGET	CGQFAGPFVQ
(PRED)	catr_1_01205	NKLSPYTYFI	QNLVSSFLHG	RIIKCSESEL	SHFNPPLGQT	CQEYSKEFLS
(PRED)	catr_1_05498	YKASPYTYFI	SNLVSSFLHG	RKIRCTEEEL	AVFNPPAGQT	CQEYTAAFLS
(PRED)	catr_1_05971	NKASPYTYYI	QNLVSSFLHN	RKIVCSDDEL	SKFNPPSGET	CQQYLSEFLS
(PRED)	deha_1_a03696g	NKASPYTYFI	QNLITAFLHG	RKVHCSDVEL	AYFNPPSGQT	CQQFAGDFVK
(PRED)	deha_2_5_e00720	NKVSPYTYFI	QNLITAFLHG	RKVHCSDVEL	AFFNPPSGQT	CQQFAGDFVK
(PRED)	scst_1_3_c02890	HKVSPYTYFI	QNLVASFLHG	RPIHCSENEL	ALFNPPEGQT	CQEFAGSFVA
(PRED)	mebi_1_8_h00300	YKVSPYTYII	QNLVSSILHG	RVVSCSSTEF	SVFDPPSGQT	CGEYMKDYLE
(PRED)	lakl_1_h21010g	WKVSPFTYFI	QSFVSLLLHN	KKVECRQNET	SYFSPPSGMT	CGEYSQQFVE
(PRED)	caar_1_13_m0142	WRASPFTYII	QNLVGILLHD	RPVVCSDTEL	NYLEPPDGQT	CGNYLATFFE
(PRED)	caar_1_14_n0143	WRASPFTYFV	ENMVGILLHD	RPVTCSTTEF	NYLDPPDGES	CGTYLDKFFE
(PRED)	hapo_1_1_a07220	WRVSPFTYMV	ENFVGILLHD	RPVHCAEKEL	NYLDPPEGET	CGSYLAQYFE
(PRED)	ogpa_1_1_a01680	WRVSPFTYMV	ENLVGILLHD	RPVHCAEKEL	NYLDPPQGET	CGSYLAQYFE
(PRED)	piku_1_96_cr001	WRLSPLTYFV	DNFLSLVLHE	RKVTCSATEY	NYLQPPSGST	CGEYLQSYFL
(PRED)	pime_1_4_d03240	WRLSPFTYFM	DNFLSILLHH	RPVVCAEAEY	NYLDPPNGST	CGEYLENYFA
(PRED)	pime_1_1_a12110	WRLSPFSYFV	NNMISVLVHD	RPVTCKKEEF	NVLQPVTGQT	CGDYLSDFFA
(PRED)	piku_1_227_hs00	WRVSPLTYFV	SDLLSISLHN	RRVECAPEEM	NVLQPPSGMT	CGDYLEPYFK
(PRED)	pime_1_5_e05800	WRISPLTYFI	GNLFGTSLHG	RKVRCAKEEY	NYLEPPSGLT	CGEYLEPFFM
(PRED)	pime_1_1_a07690	WRLSPLTYFI	GNQVGTLLHD	RPVHCSEAEF	NYLDPPDGLT	CGSYLNEYFS
(PRED)	debr_2_5_e03380	WRTSSFTYFI	DNLVSTCLHD	RQVRCSSTEM	NYLDPPDGLT	CNQYLQPFFA
(PRED)	kopa_1_2_b10040	NKVSPYTYFV	QSFLGNVLHG	REVHCAANEM	TYIQPPSEQS	CGEYLTPFIE
(PRED)	kopa_2_7_g00500	NKVSPYTYFV	QSFLGNVLHG	REVHCAAKEM	TYIQPPDEQS	CQEYLGSFVE
(PRED)	asru_1_13_m0119	YKVSPYTYFV	QSLVSVLLHN	FEVKCNASDL	TYFNPPEGQT	CAEFSNDFIQ
(PRED)	asru_1_15_o0045	YRVSPLTYFV	QSLTGALLHG	VEVRCVPKDY	TIFNPPEGQT	CIEYSDTFVD
(PRED)	wian_1_1_a02920	YKASPVTYFV	QNMVSSLIHG	REVQCSDDEL	SYVNPPNGKT	CGDYLSSFIE
(PRED)	wian_1_1_a02930	YRVSPITYVV	ESLSSTLLHG	REIHCSKSEL	AYFDPPSGQT	CGEYVSDYIS

```
                            .........1760.........1770.........1780.........1790.........1800
(PRED) asac_1_6_f03560 A-VSGYVDNP  DATSHCAYCI  YRVGDEYL-A  SIGAKYSYLW  RNFGILWGYV
(PRED) ergo_1_abr125c  S-VSGYVDNP  DATSNCAYCI  YRVGDEYL-A  SIGAKYSYLW  RNFGILWGYV
(PRED) ercy_1_3604     S-ASGYIQNP  DATANCAYCT  YTVGDEYL-A  SISSKYSYIW  RNFGLFWLYI
(PRED) cagl_1_i04862g  K-RGGYIANP  NATTACEYCI  YEVGDDYL-K  HISASYSYLW  RNFGLYWAYI
(PRED) kaaf_1_c00830   T-GSGYVANP  DDTSNCAYCI  YSVGDEYL-T  HISSKYSYLW  RNFGIYWLYI
(PRED) kana_1_k01350   R-ATGYVANP  NATENCAYCI  YTVGDDYL-A  HISASYSYVW  RNFGFYFAYI
(PRED) saar_1_2_b02590 K-STGYVKNP  DATSDCAYCV  YEVGDNYL-T  HISSKYSYLW  RNFGIYWIYI
(PRED) sace_1_ydr011w  K-ATGYIENP  DATSDCAYCI  YEVGDNYL-T  HISSKYSYLW  RNFGIFWIYI
(PRED) sace_16_1_a0238 K-ATGYIENP  DATSDCAYCI  YEVGDNYL-T  HISSKYSYLW  RNFGIFWIYI
(PRED) sace_45_1_a0242 K-ATGYIENP  DATSDCAYCI  YEVGDNYL-T  HISSKYSYLW  RNFGIFWIYI
(PRED) sace_48_1_a0238 K-ATGYIENP  DATSDCAYCI  YEVGDNYL-T  HISSKYSYLW  RNFGIFWIYI
(PRED) sace_60_4_d0244 K-ATGYIENP  DATSDCAYCI  YEVGDNYL-T  HISSKYSYLW  RNFGIFWIYI
(PRED) sace_52_1_a0240 K-ATGYIENP  DATSDCAYCI  YEVGDNYL-T  HISSKYSYLW  RNFGIFWIYI
(PRED) sace_46_1_a0240 K-ATGYIENP  DATSDCAYCI  YEVGDNYL-T  HISSKYSYLW  RNFGIFWIYI
(PRED) sace_25_1_a0240 K-ATGYIENP  DATSDCAYCI  YEVGDNYL-T  HISSKYSYLW  RNFGIFWIYI
(PRED) sace_24_1_2300  K-ATGYIENP  DATSDCAYCI  YEVGDNYL-T  HISSKYSYLW  RNFGIFWIYI
(PRED) sace_47_1_a0240 K-ATGYIENP  DATSDCAYCI  YEVGDNYL-T  HISSKYSYLW  RNFGIFWIYI
(PRED) sace_7_1_a02410 K-ATGYIENP  DATSDCAYCI  YEVGDNYL-T  HISSKYSYLW  RNFGIFWIYI
(PRED) sace_59_110_df0 K-ATGYIENP  DATSDCAYCI  YEVGDNYL-T  HISSKYSYLW  RNFGIFWIYI
(PRED) sace_56_1_a0202 K-ATGYIENP  DATSDCAYCI  YEVGDNYL-T  HISSKYSYLW  RNFGIFWIYI
(PRED) sace_40_1_a0239 K-ATGYIENP  DATSDCAYCI  YEVGDNYL-T  HISSKYSYLW  RNFGIFWIYI
(PRED) sace_15_1_a0242 K-ATGYIENP  DATSDCAYCI  YEVGDNYL-T  HISSKYSYLW  RNFGIFWIYI
(PRED) sace_37_1_a0243 K-ATGYIENP  DATSDCAYCI  YEVGDNYL-T  HISSKYSYLW  RNFGIFWIYI
(PRED) sace_9_1_a02440 K-ATGYIENP  DATSDCAYCI  YEVGDNYL-T  HISSKYSYLW  RNFGIFWIYI
(PRED) sace_22_1_2300  K-ATGYIENP  DATSDCAYCI  YEVGDNYL-T  HISSKYSYLW  RNFGIFWIYI
(PRED) sace_29_1_2290  K-ATGYIENP  DATSDCAYCI  YEVGDNYL-T  HISSKYSYLW  RNFGIFWIYI
(PRED) sace_34_1_2320  K-ATGYIENP  DATSDCAYCI  YEVGDNYL-T  HISSKYSYLW  RNFGIFWIYI
(PRED) sace_58_25_y007 K-ATGYIENP  DATSDCAYCI  YEVGDNYL-T  HISSKYSYLW  RNFGIFWIYI
(PRED) sace_23_1_2290  K-ATGYIENP  DATSDCAYCI  YEVGDNYL-T  HISSKYSYLW  RNFGIFWIYI
(PRED) sace_6_120_dp00 K-ATGYIENP  DATSDCAYCI  YEVGDNYL-T  HISSKYSYLW  RNFGIFWIYI
(PRED) sace_57_1_a0241 K-ATGYIENP  DATSDCAYCI  YEVGDNYL-T  HISSKYSYLW  RNFGIFWIYI
(PRED) sace_17_1_a0241 K-ATGYIENP  DATSDCAYCI  YEVGDNYL-T  HISSKYSYLW  RNFGIFWIYI
(PRED) sace_21_1_2310  K-ATGYIENP  DATSDCAYCI  YEVGDNYL-T  HISSKYSYLW  RNFGIFWIYI
(PRED) sace_49_1_a0246 K-ATGYIENP  DATSDCAYCI  YEVGDNYL-T  HISSKYSYLW  RNFGIFWIYI
(PRED) sace_8_2_b02430 K-ATGYIENP  DATSDCAYCI  YEVGDNYL-T  HISSKYSYLW  RNFGIFWIYI
(PRED) sace_31_1_2300  K-ATGYIENP  DATSDCAYCI  YEVGDNYL-T  HISSKYSYLW  RNFGIFWIYI
(PRED) sace_50_1_a0241 K-ATGYIENP  DATSDCAYCI  YEVGDNYL-T  HISSKYSYLW  RNFGIFWIYI
(PRED) sace_4_1_a02360 K-ATGYIENP  DATSDCAYCI  YEVGDNYL-T  HISSKYSYLW  RNFGIFWIYI
(PRED) sace_2_1_a02390 K-ATGYIENP  DATSDCAYCI  YEVGDNYL-T  HISSKYSYLW  RNFGLFWIYI
(PRED) sace_5_33_ag005 K-ATGYIENP  DATSDCAYCI  YEVGDNYL-T  HISSKYSYLW  RNFGIFWIYI
(PRED) sapa_11_1_a0247 K-ATGYIENP  DATSDCAYCI  YEVGDNYL-T  HISSKYSYLW  RNFGIFWIYI
(PRED) sapa_25_1_a0246 K-ATGYIENP  DATSDCAYCI  YEVGDNYL-T  HISSKYSYLW  RNFGIFWIYI
(PRED) sapa_4_1_a02470 K-ATGYIENP  DATSDCAYCI  YEVGDNYL-T  HISSKYSYLW  RNFGIFWIYI
(PRED) sapa_5_1_2350   K-ATGYIENP  DATSDCAYCI  YEVGDNYL-T  HISSKYSYLW  RNFGIFWIYI
(PRED) sapa_9_1_2360   K-ATGYIENP  DATSDCAYCI  YEVGDNYL-T  HISSKYSYLW  RNFGIFWIYI
(PRED) sapa_14_1_a0244 K-ATGYIENP  DATSDCAYCI  YEVGDNYL-T  HISSKYSYLW  RNFGIFWIYI
(PRED) sapa_8_1_2350   K-ATGYIENP  DATSDCAYCI  YEVGDNYL-T  HISSKYSYLW  RNFGIFWIYI
(PRED) sapa_17_1_2380  K-ATGYIENP  DATSDCAYCI  YEVGDNYL-T  HISSKYSYLW  RNFGIFWIYI
(PRED) sapa_7_1_2370   K-ATGYIENP  DATSDCAYCI  YEVGDNYL-T  HISSKYSYLW  RNFGIFWIYI
(PRED) sapa_2_1_a02460 K-ATGYIENP  DATSDCAYCI  YEVGDNYL-T  HISSKYSYLW  RNFGIFWIYI
(PRED) sapa_23_1_a0248 K-ATGYIENP  DATSDCAYCI  YEVGDNYL-T  HISSKYSYLW  RNFGIFWIYI
(PRED) sapa_3_1_a02470 K-ATGYIENP  DATSDCAYCI  YEVGDNYL-T  HISSKYSYLW  RNFGIFWIYI
(PRED) sapa_18_1_2390  K-ATGYIENP  DATSDCAYCI  YEVGDNYL-T  HISSKYSYLW  RNFGIFWIYI
(PRED) sami_1_4_244    K-ATGYIENP  DATSDCAYCI  YEVGDNYL-S  HISSKYSYLW  RNFGIYWIYI
(PRED) saku_1_4_262    K-STGYIENP  DATSDCAYCI  YEVGDNYL-T  HISSKYSYLW  RNFGIYWIYI
(PRED) saba_1_58_bf002 K-STGYIKNP  DATSDCAYCI  YEVGDSYL-T  HISSKYSYLW  RNFGIYWIYI
(PRED) saeu_1_4_d02400 K-STGYIKNP  DATSDCAYCV  YEVGDNYL-T  HISSKYSYLW  RNFGIFWIYI
(PRED) naca_1_e01640   S-ASGYIKNP  DDTSNCAYCI  YSVGDEYL-T  HISSSYSYLW  RNFGLYWAYI
(PRED) nada_1_g01850   G-ATGYINNP  DATSDCAYCL  YEVGDNYL-A  SISAPYSNLW  RNFGIFWIYI
(PRED) naca_1_e01630   R-ATGYIADP  DATSNCAYCL  YSVGDEYL-T  RISASYSYLW  RNFGIYWAYI
(PRED) nada_1_g01840   R-ATGYITDP  ASTSNCSYCL  YKVGDEYL-T  RMESSYSYLW  RNFGIFWIYI
(PRED) kaaf_1_c00820   T-HSGYIANP  SATSNCGYCL  YSVGDQYL-T  HISSKYSYLW  RNFGFFWVYI
(PRED) teph_1_m00640   T-NTGYVSNP  NDTANCGYCI  YSVGDEYL-T  YIGSSYSYLW  RNFGFYWAYI
(PRED) vapo_1_1036_28  R-STGYIVDP  SSTTECAYCI  YNVGDDYL-T  HISSSYSYLW  RNFGFYWAYI
(PRED) tebl_1_i01760   T-GTGYIDNP  DATSDCAYCI  YSVGDEYL-T  YVSASYGNIW  RNFGFYWIYI
(PRED) tode_1_d04040   K-AQGYIQNP  EATENCAYCL  SSVGDEYL-R  RIGASYSYLW  RNFGIYWIFI
(PRED) naca_1_e01650   R-SPGYIQNP  DATSQCAYCV  YTQGDEYL-T  HLSSSYSYLW  RNFGFYWAYI
(PRED) tebl_1_g02820   T-NSGYIKNP  NDTSNCAYCV  YSVGDEYL-S  YVSSKYSYLW  RNFGFFWAYI
(PRED) lakl_1_c11616g  F-ASGYVKNP  DATEKCAYCT  YKVGDEYL-K  RIGSSYSYMW  RNFGFFWVYI
(PRED) saar_1_8_h03780 F-GTGYVANP  DATSDCAYCQ  YKVGDEYL-A  HISSSFSYLW  RNFGLLWAYI
(PRED) sace_14_7_g0015 F-GTGYIANP  DATADCAYCQ  YKVGDEYL-A  RINASFSYLW  RNFGFI----
(PRED) sace_15_7_g0387 F-GTGYIANP  DATADCAYCQ  YKVGDEYL-A  RINASFSYLW  RNFGFI----
(PRED) sace_24_8_3780  F-GTGYIANP  DATADCAYCQ  YKVGDEYL-A  RINASFSYLW  RNFGFI----
```

(PRED)	sace_40_8_h0383	F-GTGYIANP	DATADCAYCQ	YKVGDEYL-A	RINASFSYLW	RNFGFI----
(PRED)	sace_6_169_fm00	F-GTGYIANP	DATADCAYCQ	YKVGDEYL-A	RINASFSYLW	RNFGFI----
(PRED)	sace_19_7_3840	F-GTGYIANP	DATADCAYCQ	YKVGDEYL-A	RINASFSYLW	RNFGFI----
(PRED)	sace_32_7_3770	F-GTGYIANP	DATADCAYCQ	YKVGDEYL-A	RINASFSYLW	RNFGFI----
(PRED)	sace_56_17_q011	F-GTGYIANP	DATADCAYCQ	YKVGDEYL-A	RINASFSYLW	RNFGFI----
(PRED)	sace_5_78_bz001	F-GTGYIANP	DATADCAYCQ	YKVGDEYL-A	RINASFSYLW	RNFGFI----
(PRED)	sace_2_8_h03860	F-GTGYIANP	DATADCAYCQ	YKVGDEYL-A	RINASFSYLW	RNFGFI----
(PRED)	sace_53_29_ac00	F-GTGYIANP	DATADCAYCQ	YKVGDEYL-A	RINASFSYLW	RNVGFI----
(PRED)	sace_17_7_g0393	F-GTGYIANP	DATADCAYCQ	YKVGDEYL-A	RINASFSYLW	RNFGFIWAYI
(PRED)	sace_25_7_g0388	F-GTGYIANP	DATADCAYCQ	YKVGDEYL-A	RINASFSYLW	RNFGFIWAYI
(PRED)	sace_37_7_g0385	F-GTGYIANP	DATADCAYCQ	YKVGDEYL-A	RINASFSYLW	RNFGFIWAYI
(PRED)	sace_9_7_g00180	F-GTGYIANP	DATADCAYCQ	YKVGDEYL-A	RINASFSYLW	RNFGFIWAYI
(PRED)	sace_60_6_f0335	F-GTGYIANP	DATADCAYCQ	YKVGDEYL-A	RINASFSYLW	RNFGFIWAYI
(PRED)	sace_59_336_lx0	F-GTGYIANP	DATADCAYCQ	YKVGDEYL-A	RINASFSYLW	RNFGFIWAYI
(PRED)	sace_31_7_3780	F-GTGYIANP	DATADCAYCQ	YKVGDEYL-A	RINASFSYLW	RNFGFIWAYI
(PRED)	sace_34_8_3770	F-GTGYIANP	DATADCAYCQ	YKVGDEYL-A	RINASFSYLW	RNFGFIWAYI
(PRED)	sace_58_71_bs00	F-GTGYIANP	DATADCAYCQ	YKVGDEYL-A	RINASFSYLW	RNFGFIWAYI
(PRED)	sace_7_7_g03880	F-GTGYIANP	DATADCAYCQ	YKVGDEYL-A	RINASFSYLW	RNFGFIWAYI
(PRED)	sace_35_7_3840	F-GTGYIANP	DATADCAYCQ	YKVGDEYL-A	RINASFSYLW	RNFGFIWAYI
(PRED)	sace_43_7_g0387	I-GTGYIANP	DATADCAYCQ	YKVGDEYL-A	RINASFSYLW	RNFGFIWAYI
(PRED)	sace_57_8_h0390	F-GTGYIANP	DATADCAYCQ	YKVGDEYL-A	RINASFSYLW	RNFGFIWAYI
(PRED)	sace_45_7_g0389	F-GTGYIANP	DATADCAYCQ	YKVGDEYL-A	RINASFSYLW	RNFGFIWAYI
(PRED)	sace_46_8_h0391	F-GTGYIANP	DATADCAYCQ	YRVGDEYL-A	RINASFSYLW	RNFGFIWAYI
(PRED)	sace_23_7_3860	I-GTGYIANP	DATADCAYCQ	YKVGDEYL-A	RINASFSYLW	RNFGFIWAYI
(PRED)	sace_21_7_3790	F-GTGYIANP	DATADCAYCQ	YKVGDEYL-A	RINASFSYLW	RNFGFIWAYI
(PRED)	sace_8_73_bu001	F-GTGYIANP	DATADCAYCQ	YKVGDEYL-A	RINASFSYLW	RNFGFIXAYI
(PRED)	sapa_1_8_h03820	F-GTGYIANP	DATSGCAYCQ	YKVGDEYL-A	RINASFSYLW	RNFGLIWAYI
(PRED)	sapa_21_8_h0387	F-GTGYIANP	DATSGCAYCQ	YKVGDEYL-A	RINASFSYLW	RNFGLIWAYI
(PRED)	sapa_20_8_h0386	F-GTGYIANP	DATSGCAYCQ	YKVGDEYL-A	RINASFSYLW	RNFGLIWAYI
(PRED)	sapa_22_8_h0390	F-GTGYIANP	DATSGCAYCQ	YKVGDEYL-A	RINASFSYLW	RNFGLIWAYI
(PRED)	sapa_25_8_h0387	F-GTGYIANP	DATSGCAYCQ	YKVGDEYL-A	RINASFSYLW	RNFGLIWAYI
(PRED)	sapa_6_8_3750	F-GTGYIANP	DATSGCAYCQ	YKVGDEYL-A	RINASFSYLW	RNFGLIWAYI
(PRED)	sapa_9_8_3720	F-GTGYIANP	DATSGCAYCQ	YKVGDEYL-A	RINASFSYLW	RNFGLIWAYI
(PRED)	sapa_19_8_h0390	F-GTGYIANP	DATSGCAYCQ	YKVGDEYL-A	RINASFSYLW	RNFSLIWAYI
(PRED)	sapa_24_8_h0385	F-GTGYIANP	DATSGCAYCQ	YKVGDEYL-A	RINASFSYLW	RNFSLIWAYI
(PRED)	sapa_4_8_h03850	F-GTGYIANP	DATSGCAYCQ	YKVGDEYL-A	RINASFSYLW	RNFGLIWAYI
(PRED)	sapa_10_8_3760	F-GTGYIANP	DATSGCAYCQ	YKVGDEYL-A	RINASFSYLW	RNFGLIWAYI
(PRED)	sapa_13_8_h0382	F-GTGYIANP	DATSGCAYCQ	YKVGDEYL-A	RINASFSYLW	RNFGLIWAYI
(PRED)	sapa_8_8_3750	F-GTGYIANP	DATSGCAYCQ	YKVGDEYL-A	RINASFSYLW	RNFGLIWAYI
(PRED)	sapa_11_8_h0383	F-GTGYIANP	DATSGCAYCQ	YKVGDEYL-A	RINASFSYLW	RNFGLIWAYI
(PRED)	sapa_5_8_3700	F-GTGYIANP	DATSGCAYCQ	YKVGDEYL-A	RINASFSYLW	RNFGLIWAYI
(PRED)	sapa_16_8_h0389	F-GTGYITNP	DATSGCAYCQ	YKVGDEYL-A	RINASFSYLW	RNFGLIWAYI
(PRED)	sapa_17_8_3730	F-GTGYITNP	DATSGCAYCQ	YKVGDEYL-A	RINASFSYLW	RNFGLIWAYI
(PRED)	sapa_2_8_h03860	F-GTGYITNP	DATSGCAYCQ	YKVGDEYL-A	RINASFSYLW	RNFGLIWAYI
(PRED)	sapa_7_8_3740	F-GTGYITNP	DATSGCAYCQ	YKVGDEYL-A	RINASFSYLW	RNFGLIWAYI
(PRED)	sapa_23_8_h0385	F-GTGYITNP	DATSGCAYCQ	YKVGDEYL-A	RINASFSYLW	RNFGLIWAYI
(PRED)	sapa_3_8_h03890	F-GTGYITNP	DATSGCAYCQ	YKVGDEYL-A	RINASFSYLW	RNFGLIWAYI
(PRED)	sapa_18_8_3730	F-GTGYITNP	DATSGCAYCQ	YKVGDEYL-A	SINASFSYLW	RNFGLIWAYI
(PRED)	sami_1_14_399	L-GTGYIANP	DATSDCAYCQ	FKVGDEYL-A	RINASFSYLW	RNFGLIWAYI
(PRED)	sace_4_8_h03690	F-GTGYIANP	DATADCAYCQ	YKVGDEYL-A	RINASFSYLW	RNFGFI----
(PRED)	saku_1_14_404	F-GTGYIANP	DATSDCAYCQ	YKVGDEYL-A	RIDASFSYLW	RNLGFICAYI
(PRED)	sace_1_ynr070w	F-GTGYIANP	DATADCAYCQ	YKVGDEYL-A	RINASFSYLW	RNFGFI----
(PRED)	sace_49_8_h0383	F-GTGYIANP	DATADCAYCQ	YKVGDEYL-A	RINASFSYLW	RNFGFI----
(PRED)	saeu_1_2_b00130	F-GTGYIANP	DATSDCAYCV	YKVGDEYL-T	HLETTYNHLW	RNFGFIWAYI
(PRED)	sauv_1_7_3	F-GTGYIANP	QATSDCEYCL	YKVGDEYL-T	HLEASYSYLW	RNFGFLWAYI
(PRED)	sami_1_17_26	H-GSGYIANP	EATSKCAYCV	YKVGDEYL-A	HISSNFSYLW	RNFGIYWAYI
(PRED)	zyba_1_02055_AN	R-SPGYISNP	NATSNCAYCQ	VSVGDEYL-A	QRNVSYGNLW	RNFGFYWAYI
(PRED)	zyba_1_07912	R-SPGYISNP	NATSNCAYCQ	VSVGDEYL-A	QRNVSYGNLW	RNFGFYWAYI
(PRED)	zyba_2_2_b00600	R-SPGYISNP	NATSNCAYCQ	VSVGDEYL-A	QRNVSYGNLW	RNFGFYWAYI
(PRED)	zyba_3_3_c03460	R-SPGYISNP	NATSNCAYCQ	VSVGDEYL-A	QRNVSYGNLW	RNFGFYWAYI
(PRED)	zyba_1_04634	R-AEGYISNP	DATSNCAYCQ	FSVGDEYL-A	QRGVSYGNLW	RNFGFYWAYI
(PRED)	zyba_1_06675	R-AEGYISNP	DATSNCAYCQ	LSVGDEYL-A	QRGVSYGNLW	RNFGFYWAYI
(PRED)	zyba_3_2_b02230	R-AEGYISNP	DATSNCAYCQ	LSVGDEYL-A	QRGVSYGNLW	RNFGFYWAYI
(PRED)	zyba_2_1_a00860	R-AEGYISNP	DATSNCAYCQ	LSVGDEYL-A	QRGVSYGNLW	RNFGFYWAYI
(PRED)	zyro_1_a04114g	Q-AQGYIANP	DATSNCAYCQ	VSVGDDYM-A	QRGVYYGNLW	RNFGFYWVYI
(PRED)	zyro_1_b14762g	R-SQGYIANP	DATSNCAYCQ	VSVGDEYL-S	QRGVSYGNIW	RNFGFYWAYI
(PRED)	zyba_2_14_n0149	N-SRGYISNP	NATSSCGYCQ	ISVGDQYL-E	QRNVSYGNLW	RNFGFYWAYI
(PRED)	zyba_2_33_ag001	N-SPGYISNP	NATSNCGYCQ	VSVGDQYL-E	QRNVTYGNLW	RNFGFYWAYI
(PRED)	lath_1_a01914g	T-AAGYINNP	NDTENCGYCV	FEVGDDYL-S	QISVKYSYLW	RNFGFFWVYI
(PRED)	lawa_1_23_5161	S-APGYINNP	DATENCGYCV	FKVGDDYL-K	QIDTSFGNIW	RNFGFFWVYI
(PRED)	klae_1_14_n0012	AGGNGYISNP	EATSNCGYCS	YSVGDQFL-T	FISASYSYIW	RNFGFLWVYI
(PRED)	klla_1_d03432g	E-GFGYIKNP	EATSNCAYCR	YKIGDQYL-S	YISAKYADLW	RNFGFLWVYI
(PRED)	klma_1_1_a01880	S-GFGYVQNP	DANTNCAYCR	YKVGDEYL-S	FISASYNNIW	RNFGFLWVYI
(PRED)	klwi_1_33_ag001	A-GYGYVTDP	NATDSCGYCS	YSVGDEYL-S	HISASYSNIW	RNFGFFWVYI
(PRED)	teph_1_a04220	E-HTGYINNP	ESTTNCAYCT	YSVGDQYL-A	RIGAHYSNLW	RNFGFFWAFI

```
(PRED) vapo_1_1037_47 I-APGYINNP  DSTSNCGYCL  YSVGDSYL-E  RVSASYSNLW  RNFGFYWAYI
(PRED) pata_1_2_b05590 S-NYGYVSNP  DATSDCGYCA  YSVADQYL-D  IINIKYVYRW  RNVGFYCAFI
(PRED) wian_1_3_c04380 R-EHGYLLNG  NGTSNCAYCK  YSVGDDYL-K  SVSVSYSYVW  RNFGFMWVYI
(PRED) wian_1_3_c04390 H-VSGYLQNP  SATSNCAYCQ  YSVGDDYL-K  TVSVSFGNRW  RNFGFMWVYI
(PRED) wian_1_7_g01010 D-NSGYLLNP  NATSNCGYCE  YRVGDEYM-S  TVSVKYSYRW  RNFGFMWVYI
(PRED) bain_1_1_a00100 D-NSGYLSNP  DATSACGYCS  YKSGDEYL-K  AINTNFGQLW  RNFGLLWVYI
(PRED) bain_1_17_q0038 N-NSGYLSNP  DATSNCGYCS  YKSGDEYL-K  SINTNFGQLW  RNFGLLWVYI
(PRED) bain_1_8_h00410 A-SQGYISNP  NATESCGFCK  YKVGDEYL-S  FLGMKHSELW  RNVGLLWGYI
(PRED) caal_1_19_5759 R-NGGYLVDE  GATSNCGYCN  FSNADQYL-L  TIGAKFSYRW  RNIGFFCVYI
(PRED) caal_11_25_y002 R-NGGYLVDE  GATSNCGYCN  FSNADQYL-L  TIGAKFSYRW  RNIGFFCVYI
(PRED) caal_4_4_d03320 R-NGGYLVDE  GATSNCGYCN  FSNADQYL-L  TIGAKFSYRW  RNIGFFCVYI
(PRED) caal_12_26_z005 R-NGGYLVDE  GATSNCGYCN  FSNADQYL-L  TIGAKFSYRW  RNIGFFCVYI
(PRED) caal_5_30_ad005 R-NGGYLVDE  GATSNCGYCN  FSNADQYL-L  TIGAKFSYRW  RNIGFFCVYI
(PRED) caal_8_3_c03320 R-NGGYLVDE  GATSNCGYCN  FSNADQYL-L  TIGAKFSYRW  RNIGFFCVYI
(PRED) caal_6_4_d03280 R-NGGYLVDE  GATSNCGYCN  FSNADQYL-L  TIGAKFSYRW  RNIGFFCVYI
(PRED) caal_10_3_c0334 R-NGGYLVDE  GATSNCGYCN  FSNADQYL-L  TIGAKFSYRW  RNIGFFCVYI
(PRED) caal_3_29_ac005 R-NGGYLVDE  GATSNCGYCN  FSNADQYL-L  TIGAKFSYRW  RNIGFFCVYI
(PRED) caal_2_04989 R-NGGYLVDE  GATSNCGYCN  FSNADQYL-L  TIGAKFSYRW  RNIGFFCVYI
(PRED) cadu_1_64350 R-HGGYLIDD  GATSNCGYCS  FSNADEYL-L  TIGVKFAYRW  RNIGFFCAYI
(PRED) caor_1_h02090 R-AGGYIDNP  SDDSNCGYCS  FSNADEYL-L  TIGAKFSLRW  RNVGFFFAYI
(PRED) capa_1_600750 R-VGGYIDNP  SDTSNCGYCS  FSNADQYL-L  TIGAKFSLRW  RNVGFFFAYI
(PRED) loel_1_04930 R-AGGYLQDP  NATSNCGYCQ  FNNADQYL-S  TIGVKFSYRW  RNVGFFFTYI
(PRED) spar_1_5_e03260 V-RGGYLVDP  NATSQCGYCS  FRNADEYL-L  SIGAKFSYRW  RNIGFFVVYI
(PRED) sppa_1_7_g03160 A-HGGYLVDE  NATNQCGYCS  LSNADQYL-W  TIRAKYSYRW  RNVGFFVAYI
(PRED) catr_1_01205 R-TTGYLVNG  NDTSNCAYCP  YSNGDEFL-W  SVFVKYSYRW  RNIGFFVAYF
(PRED) catr_1_05498 R-KPGYLVDG  SATSNCSYCP  YSTADEYL-A  SIKAKYHYRW  RNIGFFCAYI
(PRED) catr_1_05971 R-NPGYLVNE  NATSNCEYCL  YSNADQYL-L  TVQASYSNRW  RNVGFL----
(PRED) deha_1_a03696g A-RGGYLSEP  QATKECGYCT  YSIADEYL-S  SVGAEFSNRW  RNIGFYCVYI
(PRED) deha_2_5_e00720 A-RGGYLSDP  QATKECGYCT  YSVADEYL-S  SIGAKFSNRW  RNVGFYCVYI
(PRED) scst_1_3_c02890 A-SGGYLDNP  NATTQCGFCS  FSNADQYL-L  TIGAKYSYVW  RNVGFFCVYF
(PRED) mebi_1_8_h00300 T-ATGYLRDE  NATSDCGYCP  YSNADQYL-A  TVGIKYSYVW  RNIGFFCAYT
(PRED) lakl_1_h21010g A-NFGYVMNP  EDRSNCAYCT  YKVGDQYM-N  TLSFKYSYLW  RNFGFYWVYI
(PRED) caar_1_13_m0142 S-HAGYVKNP  DDTSNCGVCQ  YKVGDEYL-K  TVGISYSYRW  RNIGFFCIYI
(PRED) caar_1_14_n0143 N-HSGYVDNP  DDYSNCAVCQ  YAYGDEYM-T  TVGMSYGHRW  RNIGLFFVYI
(PRED) hapo_1_1_a07220 N-YSGYVENP  DATSNCGVCM  YKVGDQWM-D  TVGMKYSHRW  RNIGFFCVYI
(PRED) ogpa_1_1_a01680 N-YSGYVENP  DATSNCGVCM  YKVGDQWM-D  TVGMKYSHRW  RNIGFFCVYI
(PRED) piku_1_96_cr001 D-NSGYVDNP  NATADCAVCQ  YASGDEYL-K  TVGMSYSHRW  RNVGFFCVYI
(PRED) pime_1_4_d03240 D-NSGYVRNP  NDTSSCAVCQ  YSVGDEYL-S  TIGMSFHHRW  RNIGFYCVYI
(PRED) pime_1_1_a12110 A-NDGYVNNP  NASSDCQVCQ  YSVGDEFL-K  DTGMSYSYIW  RNVGFFCAYI
(PRED) piku_1_227_hs00 V-ATGYVSNP  ESFSDCAVCR  YSIADQYL-A  SVGITFHQRW  RNIGFYCVYI
(PRED) pime_1_5_e05800 Y-SFGYVNNP  NDTSNCAVCK  YSVGDQYL-E  AIEIKYSQRW  RNIGFFCVYI
(PRED) pime_1_1_a07690 T-NAGYVENP  NDTSNCAVCQ  YSVGDDFL-H  SIGVSYNDRW  RNIGLFCAYI
(PRED) debr_2_5_e03380 T-NNGYVANP  SATSQCAVCQ  YSVGDQYL-A  TVGMSYSHRW  RNIGLFVVYI
(PRED) kopa_1_2_b10040 E-HTGYVANP  GAFEDCGFCK  FAVGDQYL-S  TVGIKYSYGW  RNVGFYWVYI
(PRED) kopa_2_7_g00500 E-HTGYIANP  GAFEDCGFCK  FAVGDQYL-G  TVGIKYSYGW  RNVGFYWVYI
(PRED) asru_1_13_m0119 S-AGGYISNP  DDTTQCGYCS  FLTGDAFM-A  TFNIKWSQRW  RNLGFFCIYI
(PRED) asru_1_15_o0045 N-FGGYIANP  NDTENCEYCV  YTTGDQFM-A  NFNIEWSDRW  RNFGLIWVFI
(PRED) wian_1_1_a02920 S-NGGYLVDS  SSTENCGYCA  YTGADEYIFK  TRNIDYGTYW  RNIGIFCVFI
(PRED) wian_1_1_a02930 A-RGGYLVDP  SATSDCGYCS  YSDADTYIGS  TRMMKYSHYW  RNFGLFWVYI

                .........1810.........1820.........1830.........1840.........1850
(PRED) asac_1_6_f03560 AFNLIAMLSL  YWLFHVKKLS  ----------  IKNPFKRD--  ----------
(PRED) ergo_1_abr125c AFNIIAMLSL  YWLFRVKKVT  ----------  IKNPFKRD--  ----------
(PRED) ercy_1_3604 GFNFIAMLGL  YVVFHSTFSF  ----------  KKHLFRK---  ----------
(PRED) cagl_1_i04862g GFNICAMVAI  YYIFHVRGVS  -F-KFDRVFK  LFSRITRR--  -GKKSN----
(PRED) kaaf_1_c00830 VFNIFAMVGV  YYIFHVRQTS  -LLNISKVKK  ILGKSKK---  ----------
(PRED) kana_1_k01350 FFNLFGMVVV  YYIFHVSGFT  -F--PNPIA-  LKNKLFRR--  -RGNKN----
(PRED) saar_1_2_b02590 FFNIVAMICG  YYLFHVRQSS  -F--LSPAA-  ILKKFKNI--  -RKKR-----
(PRED) sace_1_ydr011w FFNIIAMVCV  YYLFHVRQSS  -F--LSPVS-  ILNKIKNI--  -RKKKQ----
(PRED) sace_16_1_a0238 FFNIIAMVCV  YYLFHVRQSS  -F--LSPVS-  ILNKIKNI--  -RKKKQ----
(PRED) sace_45_1_a0242 FFNIIAMVCV  YYLFHVRQSS  -F--LSPVS-  ILNKIKNI--  -RKKKQ----
(PRED) sace_48_1_a0238 FFNIIAMVCV  YYLFHVRQSS  -F--LSPVS-  ILNKIKNI--  -RKKKQ----
(PRED) sace_60_4_d0244 FFNIIAMVCV  YYLFHVRQSS  -F--LSPVS-  ILNKIKNI--  -RKKKQ----
(PRED) sace_52_1_a0240 FFNIIAMVCV  YYLFHVRQSS  -F--LSPVS-  ILNKIKNI--  -RKKKQ----
(PRED) sace_46_1_a0240 FFNIIAMVCV  YYLFHVRQSS  -F--LSPVS-  ILNKIKNI--  -RKKKQ----
(PRED) sace_25_1_a0240 FFNIIAMVCV  YYLFHVRQSS  -F--LSPVS-  ILNKIKNI--  -RKKKQ----
(PRED) sace_24_1_2300 FFNIIAMVCV  YYLFHVRQSS  -F--LSPVS-  ILNKIKNI--  -RKKKQ----
(PRED) sace_47_1_a0240 FFNIIAMVCV  YYLFHVRQSS  -F--LSPVS-  ILNKIKNI--  -RKKKQ----
(PRED) sace_7_1_a02410 FFNIIAMVCV  YYLFHVRQSS  -F--LSPVS-  ILNKIKNI--  -RKKKQ----
(PRED) sace_59_110_df0 FFNIIAMVCV  YYLFHVRQSS  -F--LSPVS-  ILNKIKNI--  -RKKKQ----
(PRED) sace_56_1_a0202 FFNIIAMVCV  YYLFHVRQSS  -F--LSPVS-  ILNKIKNI--  -RKKKQ----
(PRED) sace_40_1_a0239 FFNIIAMVCV  YYLFHVRQSS  -F--LSPVS-  ILNKIKNI--  -RKKKQ----
(PRED) sace_15_1_a0242 FFNIIAMVCV  YYLFHVRQSS  -F--LSPVS-  ILNKIKNI--  -RKKKQ----
(PRED) sace_37_1_a0243 FFNIIAMVCV  YYLFHVRQSS  -F--LSPVS-  ILNKIKNI--  -RKKKQ----
(PRED) sace_9_1_a02440 FFNIIAMVCV  YYLFHVRQSS  -F--LSPVS-  ILNKIKNI--  -RKKKQ----
```

```
(PRED) sace_22_1_2300    FFNIIAMVCV  YYLFHVRQSS  -F--LSPVS-  ILNKIKNI--  -RKKKQ----
(PRED) sace_29_1_2290    FFNIIAMVCV  YYLFHVRQSS  -F--LSPVS-  ILNKIKNI--  -RKKKQ----
(PRED) sace_34_1_2320    FFNIIAMVCV  YYLFHVRQSS  -F--LSPVS-  ILNKIKNI--  -RKKKQ----
(PRED) sace_58_25_y007   FFNIIAMVCV  YYLFHVRQSS  -F--LSPVS-  ILNKIKNI--  -RKKKQ----
(PRED) sace_23_1_2290    FFNIIAMVCV  YYLFHVRQSS  -F--LSPVS-  ILNKIKNI--  -RKKKQ----
(PRED) sace_6_120_dp00   FFNIIAMVCV  YYLFHVRQSS  -F--LSPVS-  ILNKIKNI--  -RKKKQ----
(PRED) sace_57_1_a0241   FFNIIAMVCV  YYLFHVRQSS  -F--LSPVS-  ILNKIKNI--  -RKKKQ----
(PRED) sace_17_1_a0241   FFNIIAMVCV  YYLFHVRQSS  -F--LSPVS-  ILNKIKNI--  -RKKKQ----
(PRED) sace_21_1_2310    FFNIIAMVCV  YYLFHVRQSS  -F--LSPVS-  ILNKIKNI--  -RKKKQ----
(PRED) sace_49_1_a0246   FFNIIAMVCV  YYLFHVRQSS  -F--LSPVS-  ILNKIKNI--  -RKKKQ----
(PRED) sace_8_2_b02430   FFNIIAMVCV  YYLFHVRQSS  -F--LSPVS-  ILNKIKNI--  -RKKKQ----
(PRED) sace_31_1_2300    FFNIIAMVCV  YYLFHVRQSS  -F--LSPVS-  ILNKIKNI--  -RKKKQ----
(PRED) sace_50_1_a0241   FFNIIAMVCV  YYLFHVRQSS  -F--LSPVS-  ILNKIKNI--  -RKKKQ----
(PRED) sace_4_1_a02360   FFNIIAMVCV  YYLFHVRQSS  -F--XSPVS-  ILNKIKNI--  -RKKKQ----
(PRED) sace_2_1_a02390   FFNIIAMVCV  YYLFHVRQSS  -F--LSPVS-  ILNKIKNI--  -RKKKQ----
(PRED) sace_5_33_ag005   FFNIIAMVCV  YYLFHVRQSS  -F--LSPVS-  ILNKIKNI--  -RKKKQ----
(PRED) sapa_11_1_a0247   FFNIIAMVCL  YYIFHVRQSS  -L--LSPVS-  ILNKIKNI--  -RKKKQ----
(PRED) sapa_25_1_a0246   FFNIIAMVCL  YYIFHVRQSS  -L--LSPVS-  ILNKIKNI--  -RKKKQ----
(PRED) sapa_4_1_a02470   FFNIIAMVCL  YYIFHVRQSS  -L--LSPVS-  ILNKIKNI--  -RKKKQ----
(PRED) sapa_5_1_2350     FFNIIAMVCL  YYIFHVRQSS  -L--LSPVS-  ILNKIKNI--  -RKKKQ----
(PRED) sapa_9_1_2360     FFNIIAMVCL  YYIFHVRQSS  -L--LSPVS-  ILNKIKNI--  -RKKKQ----
(PRED) sapa_14_1_a0244   FFNIIAMVCL  YYIFHVRQSS  -L--LSPVS-  ILNKIKNI--  -RKKKQ----
(PRED) sapa_8_1_2350     FFNIIAMVCL  YYIFHVRQSS  -L--LSPVS-  ILNKIKNI--  -RKKKQ----
(PRED) sapa_17_1_2380    FFNIIAMICV  YYIFHVRQSS  -L--LSPVS-  ILNKIKNI--  -GKKKQ----
(PRED) sapa_7_1_2370     FFNIIAMICV  YYIFHVRQSS  -L--LSPVS-  ILNKIKNI--  -GKKKQ----
(PRED) sapa_2_1_a02460   FFNIIAMICV  YYIFHVRQSS  -L--LSPVS-  ILNKIKNI--  -GKKKQ----
(PRED) sapa_23_1_a0248   FFNIIAMICV  YYIFHVRQSS  -L--LSPVS-  ILNKIKNI--  -GKKKQ----
(PRED) sapa_3_1_a02470   FFNIIAMICV  YYIFHVRQSS  -L--LSPVS-  ILNKIKNI--  -GKKKQ----
(PRED) sapa_18_1_2390    FFNIIAMICV  YYIFHVRQSS  -L--LSPVS-  ILNKIKNI--  -GKKKQ----
(PRED) sami_1_4_244      FFNIIAMVCV  YYIFHVRQSS  -I--LSPAS-  ILKNIKNI--  -RKKKQ----
(PRED) saku_1_4_262      FFNIIAMICV  YYLFHVRQSS  -F--LSPAF-  IIKKIKNA--  -RKKKQ----
(PRED) saba_1_58_bf002   FFNIVGMVCV  YYLFHVRQSS  -F--LSPKF-  IIKKIKNI--  -RKSKK----
(PRED) saeu_1_4_d02400   FFNIVGMVCV  YYLFHVRQSS  -L--LSPKF-  MLKKIKNI--  -RKSKQ----
(PRED) naca_1_e01640     IFNIFGMISL  YYIFHVRQTS  -L--LNLSF-  VKKGLGKV--  -KGKK-----
(PRED) nada_1_g01850     VFNIIAMVSL  YYLLHVKNVS  -P--LTGVM-  KLATKVKS--  -KGKK-----
(PRED) naca_1_e01630     IFNLFAMVTL  YYIFHVKKLS  -IFKLKPVKR  LIRKIGGT--  -AQETMS---
(PRED) nada_1_g01840     IFNLFGMIAV  YYIFHVKKLS  -IFRLRLVKK  FLDKIRGI--  -APEEFD---
(PRED) kaaf_1_c00820     IFNIFAMVGV  YYIFHVRDAS  -IFNLKFVEK  LISHIRKS--  -KKKNVVD--
(PRED) teph_1_m00640     GFNICAMVGV  YYIVHVRGVS  -F--ISPEK-  ISKFLKRF--  -KK-------
(PRED) vapo_1_1036_28    GFNICAMIGV  YYIFHVRKIS  -M--IKPGN-  I---LKKF--  -KKN------
(PRED) tebl_1_i01760     VFNIFAMGTV  YYVFHVK--N  -LISISTITM  LIAQVKKRIV  PKKKDKSDEN
(PRED) tode_1_d04040     VFNFFAMVAV  YYIFHVRKVS  -LINVQAITN  FTQILKGKMP  FGKKKASSV-
(PRED) naca_1_e01650     IFNIFAMVIL  YYIFHVRQTQ  -LINLGS---  IMNRIKRV--  --KT------
(PRED) tebl_1_g02820     IFNFFAMVGL  YYIIHVRKIS  ----LGNPSA  ILKRFKKN--  ----------
(PRED) lakl_1_c11616g    IFNLAAMIIL  YYLFHVRKIS  -LKDSKLTRA  FMSKVKKE--  ----------
(PRED) saar_1_8_h03780   GFNIFGMIAV  YYVVWVKQFS  -LMKVGIVKQ  LIAKLK----  -RK-------
(PRED) sace_14_7_g0015   ----------  ----------  ----------  ----------  ----------
(PRED) sace_15_7_g0387   ----------  ----------  ----------  ----------  ----------
(PRED) sace_24_8_3780    ----------  ----------  ----------  ----------  ----------
(PRED) sace_40_8_h0383   ----------  ----------  ----------  ----------  ----------
(PRED) sace_6_169_fm00   ----------  ----------  ----------  ----------  ----------
(PRED) sace_19_7_3840    ----------  ----------  ----------  ----------  ----------
(PRED) sace_32_7_3770    ----------  ----------  ----------  ----------  ----------
(PRED) sace_56_17_q011   ----------  ----------  ----------  ----------  ----------
(PRED) sace_5_78_bz001   ----------  ----------  ----------  ----------  ----------
(PRED) sace_2_8_h03860   ----------  ----------  ----------  ----------  ----------
(PRED) sace_53_29_ac00   ----------  ----------  ----------  ----------  ----------
(PRED) sace_17_7_g0393   LFNIAGMIVV  YYVVQVKHFS  -PMKIGFVKR  ITSKFK----  -RK-------
(PRED) sace_25_7_g0388   LFNIAGMIVV  YYVVQVKHFS  -PMKIGFVKR  ITSKFK----  -RK-------
(PRED) sace_37_7_g0385   LFNIAGMIVV  YYVVQVKHFS  -PMKIGFVKR  ITSKFK----  -RK-------
(PRED) sace_9_7_g00180   LFNIAGMIVV  YYVVQVKHFS  -PMKIGFVKR  ITSKFK----  -RK-------
(PRED) sace_60_6_f0335   LFNIAGMIVV  YYVVQVKHFS  -PMKIGFVKR  ITSKFK----  -RK-------
(PRED) sace_59_336_lx0   LFNIAGMIVV  YYVVQVKHFS  -PMKIGFVKR  ITSKFK----  -RK-------
(PRED) sace_31_7_3780    LFNIAGMIVV  YYVVQVKHFS  -PMKIGFVKR  ITSKFK----  -RK-------
(PRED) sace_34_8_3770    LFNIAGMIVV  YYVVQVKHFS  -PMKIGFVKR  ITSKFK----  -RK-------
(PRED) sace_58_71_bs00   LFNIAGMIVV  YYVVQVKHFS  -PMKIGFVKR  ITSKFK----  -RK-------
(PRED) sace_7_7_g03880   LFNIAGMIVV  YYVVQVKHFS  -PMKIGFVKR  ITSKFK----  -RK-------
(PRED) sace_35_7_3840    LFNIAGMIVV  YYVVQVKHFS  -PMKIGFVKR  ITSKFK----  -RK-------
(PRED) sace_43_7_g0387   LFNIAGMIVV  YYVVQVKHFS  -PMKIGFVKR  ITSKFK----  -RK-------
(PRED) sace_57_8_h0390   LFDIAGMIVV  YYVVQVKHFS  -PMKIGFVKR  ITSKFK----  -RK-------
(PRED) sace_45_7_g0389   LFNIAGMIVV  YYVVQVKHFS  -PMKIGFVKR  ITSKFK----  -RK-------
(PRED) sace_46_8_h0391   LFNIAGMIVV  YYVVQVKHFS  -PMKIGFVKR  ITSKFK----  -RK-------
(PRED) sace_23_7_3860    LFNIACMIVV  YYVVQVKHFS  -PMKIGFVKR  ITSKFK----  -RK-------
(PRED) sace_21_7_3790    LFNIAGMIVV  YYVVQVKHFS  -PMKIGFVKR  ITSKFK----  -RK-------
(PRED) sace_8_73_bu001   LFNIAGMIVV  YYVVQVKHFS  -PMKIGFVKR  ITSKFK----  -RK-------
```

(PRED)	sapa_1_8_h03820	IFNIIAMIAV	YYVVQVKHFS	-PMKIGFMKR	ITISFK----	-RK-------
(PRED)	sapa_21_8_h0387	IFNIIAMIAV	YYVVQVKHFS	-PMKIGFMKR	ITISFK----	-RK-------
(PRED)	sapa_20_8_h0386	IFNIIAMIAV	YYVVQVKHFS	-PMKIGFVKR	ITISFK----	-RK-------
(PRED)	sapa_22_8_h0390	IFNIIAMIAV	YYVVQVKHFS	-PMKIGFVKR	ITISFK----	-RK-------
(PRED)	sapa_25_8_h0387	IFNIIAMIAV	YYVVQVKHFS	-PMKIGFVKR	ITISFK----	-RK-------
(PRED)	sapa_6_8_3750	IFNIIAMIAV	YYVVQVKHFS	-PMKIGFVKR	ITISFK----	-RK-------
(PRED)	sapa_9_8_3720	IFNIIAMIAV	YYVVQVKHFS	-PMKIGFVKR	ITISFK----	-RK-------
(PRED)	sapa_19_8_h0390	IFNIIAMIAV	YYVVQVKHFS	-PMKIGFVKR	ITISFK----	-RK-------
(PRED)	sapa_24_8_h0385	IFNIIAMIAV	YYVVQVKHFS	-PMKIGFVKR	ITISFK----	-RK-------
(PRED)	sapa_4_8_h03850	IFNIIAMIAV	YYVVQVKHFS	-PMKIGFVKR	ITISFK----	-RK-------
(PRED)	sapa_10_8_3760	IFNIIAMIAV	YYVVQVKHFS	-PMKIGFVKR	ITISFK----	-RK-------
(PRED)	sapa_13_8_h0382	IFNIIAMIAV	YYAVQVKHFS	-PMKIGFVKK	ITISFK----	-RK-------
(PRED)	sapa_8_8_3750	IFNIIAMIAV	YYAVQVKHFS	-PMKIGFVKK	ITISFK----	-RK-------
(PRED)	sapa_11_8_h0383	IFNIIAMIAV	YYVVQVKRFS	-PMKIGFVKR	ITISFK----	-RK-------
(PRED)	sapa_5_8_3700	IFNIIAMIAV	YYVVQVKHFS	-PMKIGFVKR	ITISFK----	-RK-------
(PRED)	sapa_16_8_h0389	IFNIIAMIAV	YYVVQVKHFS	-LMKIGFVKR	IKISFK----	-RK-------
(PRED)	sapa_17_8_3730	IFNIIAMIAV	YYVVQVKHFS	-LMKIGFVKR	IKISFK----	-RK-------
(PRED)	sapa_2_8_h03860	IFNIIAMIAV	YYVVQVKHFS	-LMKIGFVKR	IKISFK----	-RK-------
(PRED)	sapa_7_8_3740	IFNIIAMIAV	YYVVQVKHFS	-LMKIGFVKR	IKISFK----	-RK-------
(PRED)	sapa_23_8_h0385	IFNIIAMIAV	YYVVQVKHFS	-LMKIGFVKR	IKISFK----	-RK-------
(PRED)	sapa_3_8_h03890	IFNIIAMIAV	YYVVQVKHFS	-LMKIGFVKR	IKISFK----	-RK-------
(PRED)	sapa_18_8_3730	ISNIITMIAV	YYLVQVKHFS	-LMKIGFVKR	ITISFK----	-RK-------
(PRED)	sami_1_14_399	AFNIFGMIAV	YYVFQVRHFS	-LMKIGFVKR	IVFEFK----	-RK-------
(PRED)	sace_4_8_h03690	----------	----------	----------	----------	----------
(PRED)	saku_1_14_404	IFNIFAMVAV	YYVVQVKHFS	-PMNIGIVKK	LASKFK----	-RKRSST---
(PRED)	sace_1_ynr070w	----------	----------	----------	----------	----------
(PRED)	sace_49_8_h0383	----------	----------	----------	----------	----------
(PRED)	saeu_1_2_b00130	IFNIFGMIVV	YYVVQVKQFS	-PMKIGIVKN	LTERFK----	-RERENN---
(PRED)	sauv_1_7_3	IFNIFGMMAV	YYVVQVRQFS	-PMNIGIVKN	LIARFK----	-RESS-----
(PRED)	sami_1_17_26	AFNIFGMILM	YYLIQIKHIS	-PMNIGIVKR	LMARFK----	-RK-------
(PRED)	zyba_1_02055_AN	VFNIFAMVGI	YYIFSVRGVG	GFNPFAIVKR	L-IK------	-RK-------
(PRED)	zyba_1_07912	VFNIFAMVGI	YYIFSVRGVG	GFNPFAIVKR	L-IK------	-RK-------
(PRED)	zyba_2_2_b00600	VFNIFAMVGI	YYIFSVRGVG	GFNPFAIVKR	L-IK------	-RK-------
(PRED)	zyba_3_3_c03460	VFNIFAMVGI	YYIFSVRGVG	GFNPFAIVKR	L-IK------	-RK-------
(PRED)	zyba_1_04634	VFNFFAMVII	YYIFSVRGVG	RFHPIAIVKR	F-IK------	-RN-------
(PRED)	zyba_1_06675	VFNLFAMVAI	YYISSVRGVG	QFHPIAIFRR	F-FK------	-RN-------
(PRED)	zyba_3_2_b02230	VFNLFAMVAI	YYISSVRGVG	QFHPIAIFRR	F-FK------	-RN-------
(PRED)	zyba_2_1_a00860	VFNLFAMVAI	YYISSVRGVG	QFHPIAIFRR	F-FK------	-RN-------
(PRED)	zyro_1_a04114g	VFNMFAMVGI	YYIFSVRGVS	IFNPFAVIKR	I-IK------	-RK-------
(PRED)	zyro_1_b14762g	IFNLFAMVGV	YYIFSVRGLR	SFNPFLFIKR	I-IK------	-RQ-------
(PRED)	zyba_2_14_n0149	VFNMFAMVGV	YYIFSVRGLS	FFSPSAVFKR	LKLG------	-RKKS-----
(PRED)	zyba_2_33_ag001	VFNMFAMVGV	YYIFSVRGVS	FFSPSAVLKR	LKLG------	-RKKS-----
(PRED)	lath_1_a01914g	CFNIFAMAVL	YYIFHVRSFS	-LKQTKLGKM	IMGKM-----	-KKE------
(PRED)	lawa_1_23_5161	FFNVFAMLVL	YYVFHVSNFS	-LKETRIVKK	LMGKI-----	-RKE------
(PRED)	klae_1_14_n0012	CFNIFLMVAL	YYLFHVQKFS	-------ILP	LLKKLK----	--LSKD----
(PRED)	klla_1_d03432g	CFNLIAMVSL	YYIFHVKRFS	---PKSLVMG	VVGKFK----	--KEK-----
(PRED)	klma_1_1_a01880	CFNIFAMVAL	YYIFSVKRIS	---PRSLVER	TLARFK----	--KNKT----
(PRED)	klwi_1_33_ag001	VFNIGAMVVL	YYIFHVRKVS	-------FMP	LLKKFT----	--KSKD----
(PRED)	teph_1_a04220	LFNIGAMLAV	YYLFHVKNLG	-FQQKENIRY	FFNRIRQFL-	-RISKQKK--
(PRED)	vapo_1_1037_47	VFNLIAMVVS	YYIFHILKI-	---PSKIIHI	IKNALAAF--	-RIKQKNKNN
(PRED)	pata_1_2_b05590	LFNLSAMIIC	YYIFRVKKYS	---PVSLVKP	LISKVLKK--	-RE-------
(PRED)	wian_1_3_c04380	IFNICAMCVL	YYIFRVININ	---PVDSIKK	KIESQKLK--	-REKKKLKSS
(PRED)	wian_1_3_c04390	IFNIFTMCAV	YYIFRVANIS	---PLGFIKE	KLEASKRK--	-REEKKGQNV
(PRED)	wian_1_7_g01010	IFNIVAMCAL	YYIFRVANVS	---PLGYLKG	KLDAFKQK--	-RDQKKEANQ
(PRED)	bain_1_1_a00100	FFNIFAMVLT	YWLFRVKKFS	--FG------	-----K----	---KKA----
(PRED)	bain_1_17_q0038	FFNIFAMVLT	YWLFRVKKFS	--FG------	-----K----	---KA-----
(PRED)	bain_1_8_h00410	AFNFFACVFL	YWLLRVHKVS	--LP------	-----K----	-FARKA----
(PRED)	caal_1_19_5759	IFNISVCLVL	YYFLRYRKVS	--FNVTGLVN	---KFK----	-KSKK-----
(PRED)	caal_11_25_y002	IFNISVCLVL	YYFLRYRKVS	--FNVTGLVN	---KFK----	-KSKK-----
(PRED)	caal_4_4_d03320	IFNISVCLVL	YYFLRYRKVS	--FNVTGLVN	---KFK----	-KSKK-----
(PRED)	caal_12_26_z005	IFNISVCLVL	YYFLRYRKVS	--FNVTGLVN	---KFK----	-KSKK-----
(PRED)	caal_5_30_ad005	IFNISVCLVL	YYFLRYRKVS	--FNVTGLVN	---KFK----	-KSKK-----
(PRED)	caal_8_3_c03320	IFNISVCLVL	YYFLRYRKVS	--FNVTGLVN	---KFK----	-KSKK-----
(PRED)	caal_6_4_d03280	IFNISVCLVL	YYFLRYRKVS	--FNVTGLVN	---KFK----	-KSKK-----
(PRED)	caal_10_3_c0334	IFNISVCLVL	YYFLRYRKVS	--FNVTGLVN	---KFK----	-KSKK-----
(PRED)	caal_3_29_ac005	IFNISVCLVL	YYFLRYRKVS	--FNVTGLVN	---KFK----	-KSKK-----
(PRED)	caal_2_04989	IFNISVCLVL	YYFLRYRKVS	--FNVTGLVN	---KFK----	-KSKK-----
(PRED)	cadu_1_64350	IFNISICLIL	YYFIRYRKIS	--LNVTGLIS	---KFK----	-KSKI-----
(PRED)	caor_1_h02090	IFNIGFCMCL	YYLLRYSKLT	--NKATGFFA	---GFK----	-KNKK-----
(PRED)	capa_1_600750	LFNLGICMCL	YYFMRYSKLT	--NKLTGLFA	---RFK----	-KSKK-----
(PRED)	loel_1_04930	FFNIIICMAL	YYLFRFSKFT	--NKLKGLTT	---VLT----	-HEENM----
(PRED)	spar_1_5_e03260	IFNLGMCLVL	YYLLRYRKVF	--NNLDSPLK	---IFK----	-RKSKV----
(PRED)	sppa_1_7_g03160	IFNISACLSL	YYLMRYRKIF	--NNMGNPLN	---VFK----	-RKKD-----
(PRED)	catr_1_01205	IFNVGFCLFL	YYIIRYKKYL	--KG---MFD	LSQKLK----	-PSKK-----
(PRED)	catr_1_05498	LFNIGFCLFL	YYFIRYKRIF	--KGVPALFH	NILRMF----	-KKKKA----

```
(PRED) catr_1_05971   -----FCL--  -YFI------  ----------  ----------  ----------
(PRED) deha_1_a03696g AFNLCFVLAL  YWLFRFRK--  --GSIFSI--  ----FK----  -RNN------
(PRED) deha_2_5_e00720 VFNLCFVLVL YWFFRFRK--  --GSIFSV--  ----FK----  -RNG------
(PRED) scst_1_3_c02890 IFNIALVLFL YYSFRYKR--  --FNPFAI--  ----FK----  -KK-------
(PRED) mebi_1_8_h00300 IFNLCACLLL YKFVRLTKYN  --FSLS----  ----FL----  -KNKKK----
(PRED) lakl_1_h21010g LFNLVGMLVA  YYIFHIYNFS  ------PFTR  LKQQFM----  -RTKKTED--
(PRED) caar_1_13_m0142 IANVVFMISL YYTLRVRKFS  ---LTGL---  ----IS----  -RKKK-----
(PRED) caar_1_14_n0143 CFNLFFMVSL YYLLRVRRIS  ---LSLP---  ---KFG----  -KSKK-----
(PRED) hapo_1_1_a07220 GFNLFAMLSM YYVLRVRKWK  ---LPAF---  ---KFG----  -KK-------
(PRED) ogpa_1_1_a01680 GFNLFAMLSM YYVLRVRKWR  ---LPAF---  ---KFG----  -KK-------
(PRED) piku_1_96_cr001 CFNLCAMMSL HYFFRIRKYS  ---LKTPFVL  LAKKFQ----  -RK-------
(PRED) pime_1_4_d03240 GFNVWVMCFM YYVLRVKSWN  ---PMSPFLS  LQKRFK----  -KKN------
(PRED) pime_1_1_a12110 AFNFFCMVGL YYVLRVKSIN  ---PLSPIFK  LVEKIK----  -SKKK-----
(PRED) piku_1_227_hs00 IVNFFGMIVL YWIFRVKRFK  ---PWTKLPT  LKKH------  ----------
(PRED) pime_1_5_e05800 GFNCCAMVVL YYIFRVRRIN  ---PITPVLS  LLKSWK----  -KK-------
(PRED) pime_1_1_a07690 CFNLFLMNFL YFVFRVKRFS  ---PFN-LVR  FVKSFK----  -NKSKK----
(PRED) debr_2_5_e03380 LFNVFCMLSM YYIFRVRHFS  ---ISLPKLP  AFLRRK----  -KKVKDGEKP
(PRED) kopa_1_2_b10040 VFNLSAMLFL YYMFKVRKQS  ---IFAPIIG  L---FG----  -RKQKD----
(PRED) kopa_2_7_g00500 IFNLSAMLFL YYMFKVRKQS  ---IFAPIIG  L---FG----  -RKNKD----
(PRED) asru_1_13_m0119 FFNLFAMVFC YYFFRVKQFK  ------MPSL  FKKKAKN---  ----------
(PRED) asru_1_15_o0045 VFNVFAMLVF YRIFRVKK--  --------SL  FKRKHT----  ----------
(PRED) wian_1_1_a02920 AFNILGMMVL YWYFKDFKFK  ------RSKV  NSKKSND---  -QEK------
(PRED) wian_1_1_a02930 LFNVVSMMAL YWLAKDAKFS  ------TPKL  FQKKPKA---  -EKEPKVEQS

                       ...
(PRED) asac_1_6_f03560 ---
(PRED) ergo_1_abr125c ---
(PRED) ercy_1_3604    ---
(PRED) cagl_1_i04862g ---
(PRED) kaaf_1_c00830  ---
(PRED) kana_1_k01350  ---
(PRED) saar_1_2_b02590 ---
(PRED) sace_1_ydr011w ---
(PRED) sace_16_1_a0238 ---
(PRED) sace_45_1_a0242 ---
(PRED) sace_48_1_a0238 ---
(PRED) sace_60_4_d0244 ---
(PRED) sace_52_1_a0240 ---
(PRED) sace_46_1_a0240 ---
(PRED) sace_25_1_a0240 ---
(PRED) sace_24_1_2300 ---
(PRED) sace_47_1_a0240 ---
(PRED) sace_7_1_a02410 ---
(PRED) sace_59_110_df0 ---
(PRED) sace_56_1_a0202 ---
(PRED) sace_40_1_a0239 ---
(PRED) sace_15_1_a0242 ---
(PRED) sace_37_1_a0243 ---
(PRED) sace_9_1_a02440 ---
(PRED) sace_22_1_2300 ---
(PRED) sace_29_1_2290 ---
(PRED) sace_34_1_2320 ---
(PRED) sace_58_25_y007 ---
(PRED) sace_23_1_2290 ---
(PRED) sace_6_120_dp00 ---
(PRED) sace_57_1_a0241 ---
(PRED) sace_17_1_a0241 ---
(PRED) sace_21_1_2310 ---
(PRED) sace_49_1_a0246 ---
(PRED) sace_8_2_b02430 ---
(PRED) sace_31_1_2300 ---
(PRED) sace_50_1_a0241 ---
(PRED) sace_4_1_a02360 ---
(PRED) sace_2_1_a02390 ---
(PRED) sace_5_33_ag005 ---
(PRED) sapa_11_1_a0247 ---
(PRED) sapa_25_1_a0246 ---
(PRED) sapa_4_1_a02470 ---
(PRED) sapa_5_1_2350 ---
(PRED) sapa_9_1_2360 ---
(PRED) sapa_14_1_a0244 ---
(PRED) sapa_8_1_2350 ---
(PRED) sapa_17_1_2380 ---
(PRED) sapa_7_1_2370 ---
(PRED) sapa_2_1_a02460 ---
```

```
(PRED) sapa_23_1_a0248 ---
(PRED) sapa_3_1_a02470 ---
(PRED) sapa_18_1_2390  ---
(PRED) sami_1_4_244    ---
(PRED) saku_1_4_262    ---
(PRED) saba_1_58_bf002 ---
(PRED) saeu_1_4_d02400 ---
(PRED) naca_1_e01640   ---
(PRED) nada_1_g01850   ---
(PRED) naca_1_e01630   ---
(PRED) nada_1_g01840   ---
(PRED) kaaf_1_c00820   ---
(PRED) teph_1_m00640   ---
(PRED) vapo_1_1036_28  ---
(PRED) tebl_1_i01760   AD-
(PRED) tode_1_d04040   ---
(PRED) naca_1_e01650   ---
(PRED) tebl_1_g02820   ---
(PRED) lakl_1_c11616g  ---
(PRED) saar_1_8_h03780 ---
(PRED) sace_14_7_g0015 ---
(PRED) sace_15_7_g0387 ---
(PRED) sace_24_8_3780  ---
(PRED) sace_40_8_h0383 ---
(PRED) sace_6_169_fm00 ---
(PRED) sace_19_7_3840  ---
(PRED) sace_32_7_3770  ---
(PRED) sace_56_17_q011 ---
(PRED) sace_5_78_bz001 ---
(PRED) sace_2_8_h03860 ---
(PRED) sace_53_29_ac00 ---
(PRED) sace_17_7_g0393 ---
(PRED) sace_25_7_g0388 ---
(PRED) sace_37_7_g0385 ---
(PRED) sace_9_7_g00180 ---
(PRED) sace_60_6_f0335 ---
(PRED) sace_59_336_lx0 ---
(PRED) sace_31_7_3780  ---
(PRED) sace_34_8_3770  ---
(PRED) sace_58_71_bs00 ---
(PRED) sace_7_7_g03880 ---
(PRED) sace_35_7_3840  ---
(PRED) sace_43_7_g0387 ---
(PRED) sace_57_8_h0390 ---
(PRED) sace_45_7_g0389 ---
(PRED) sace_46_8_h0391 ---
(PRED) sace_23_7_3860  ---
(PRED) sace_21_7_3790  ---
(PRED) sace_8_73_bu001 ---
(PRED) sapa_1_8_h03820 ---
(PRED) sapa_21_8_h0387 ---
(PRED) sapa_20_8_h0386 ---
(PRED) sapa_22_8_h0390 ---
(PRED) sapa_25_8_h0387 ---
(PRED) sapa_6_8_3750   ---
(PRED) sapa_9_8_3720   ---
(PRED) sapa_19_8_h0390 ---
(PRED) sapa_24_8_h0385 ---
(PRED) sapa_4_8_h03850 ---
(PRED) sapa_10_8_3760  ---
(PRED) sapa_13_8_h0382 ---
(PRED) sapa_8_8_3750   ---
(PRED) sapa_11_8_h0383 ---
(PRED) sapa_5_8_3700   ---
(PRED) sapa_16_8_h0389 ---
(PRED) sapa_17_8_3730  ---
(PRED) sapa_2_8_h03860 ---
(PRED) sapa_7_8_3740   ---
(PRED) sapa_23_8_h0385 ---
(PRED) sapa_3_8_h03890 ---
(PRED) sapa_18_8_3730  ---
(PRED) sami_1_14_399   ---
(PRED) sace_4_8_h03690 ---
(PRED) saku_1_14_404   ---
(PRED) sace_1_ynr070w  ---
```

(PRED) sace_49_8_h0383 ---
(PRED) saeu_1_2_b00130 ---
(PRED) sauv_1_7_3 ---
(PRED) sami_1_17_26 ---
(PRED) zyba_1_02055_AN ---
(PRED) zyba_1_07912 ---
(PRED) zyba_2_2_b00600 ---
(PRED) zyba_3_3_c03460 ---
(PRED) zyba_1_04634 ---
(PRED) zyba_1_06675 ---
(PRED) zyba_3_2_b02230 ---
(PRED) zyba_2_1_a00860 ---
(PRED) zyro_1_a04114g ---
(PRED) zyro_1_b14762g ---
(PRED) zyba_2_14_n0149 ---
(PRED) zyba_2_33_ag001 ---
(PRED) lath_1_a01914g ---
(PRED) lawa_1_23_5161 ---
(PRED) klae_1_14_n0012 ---
(PRED) klla_1_d03432g ---
(PRED) klma_1_1_a01880 ---
(PRED) klwi_1_33_ag001 ---
(PRED) teph_1_a04220 ---
(PRED) vapo_1_1037_47 N--
(PRED) pata_1_2_b05590 ---
(PRED) wian_1_3_c04380 EE-
(PRED) wian_1_3_c04390 SEA
(PRED) wian_1_7_g01010 ---
(PRED) bain_1_1_a00100 ---
(PRED) bain_1_17_q0038 ---
(PRED) bain_1_8_h00410 ---
(PRED) caal_1_19_5759 ---
(PRED) caal_11_25_y002 ---
(PRED) caal_4_4_d03320 ---
(PRED) caal_12_26_z005 ---
(PRED) caal_5_30_ad005 ---
(PRED) caal_8_3_c03320 ---
(PRED) caal_6_4_d03280 ---
(PRED) caal_10_3_c0334 ---
(PRED) caal_3_29_ac005 ---
(PRED) caal_2_04989 ---
(PRED) cadu_1_64350 ---
(PRED) caor_1_h02090 ---
(PRED) capa_1_600750 ---
(PRED) loel_1_04930 ---
(PRED) spar_1_5_e03260 ---
(PRED) sppa_1_7_g03160 ---
(PRED) catr_1_01205 ---
(PRED) catr_1_05498 ---
(PRED) catr_1_05971 ---
(PRED) deha_1_a03696g ---
(PRED) deha_2_5_e00720 ---
(PRED) scst_1_3_c02890 ---
(PRED) mebi_1_8_h00300 ---
(PRED) lakl_1_h21010g ---
(PRED) caar_1_13_m0142 ---
(PRED) caar_1_14_n0143 ---
(PRED) hapo_1_1_a07220 ---
(PRED) ogpa_1_1_a01680 ---
(PRED) piku_1_96_cr001 ---
(PRED) pime_1_4_d03240 ---
(PRED) pime_1_1_a12110 ---
(PRED) piku_1_227_hs00 ---
(PRED) pime_1_5_e05800 ---
(PRED) pime_1_1_a07690 ---
(PRED) debr_2_5_e03380 QS-
(PRED) kopa_1_2_b10040 ---
(PRED) kopa_2_7_g00500 ---
(PRED) asru_1_13_m0119 ---
(PRED) asru_1_15_o0045 ---
(PRED) wian_1_1_a02920 ---
(PRED) wian_1_1_a02930 DLA
