# Supplementary material for: Molecular evolution of the members of the Snq2/Pdr18 subfamily of Pdr transporters in the Hemiascomycete yeasts
Source: FEMS Yeast Res. 2025 May 27;25:foaf026. doi: 10.1093/femsyr/foaf026 (PMC12202755; doi:10.1093/femsyr/foaf026)
Supplement: foaf026_Supplemental_Files [file foaf026_supplemental_files.zip › Figure A9_Supplementary Data.pdf]

						CaSNQ2					
Candida albicans SC5314	134	14916	13543	13836	225	caal_1_19.5759	20402	20403	16357	15534	20404
Candida dubliniensis CD36	134	14916	13543	13836	225	cadu_1_64350	20402	20403	16357	15534	20404
Candida tropicalis MYA-3404	4503	13994	19092	19055	19055	catr_1_01205	12356	16332	13404	13405	13404
	110045	41242	41243	41244	41243	catr_1_05498	225	2628	2681	4202	7618
	0	0	37728	3226	13543	catr_1_05971	16006	1313	15	8661	14020
Candida parapsilosis CDC317	13206	38695	1041	13836	225	capa_1_600750	13543	134	694	17016	20380
Candida orthopsilosis Co 90-125	38830	38634	38555	13836	225	caor_1_h02090	13543	12538	20380	17016	17554
Lodderomyces elongisporus NRLL YB-4239	1041	14044	13836	225	134	loel_1_04930	80003	80004	12538	17554	1601
Spathaspora arborariae UFMG-19.1A	20384	20401	14703	13836	225	spar_1_5_e03260	13543	12538	12290	12759	14176
Spathaspora passalidarum NRRL Y-27907	20384	20401	14703	13836	225	sppa_1_7_g03160	13543	12538	12290	17554	12759
Scheffersomyces stipitis CBS 6054	20380	20381	2671	13836	225	scst_1_3_c02890	13543	95894	12538	17554	12759
Debaryomyces hansenii CBS76	14032	13861	20253	2671	225	deha_1_a03696g	13543	46268	110830	16803	2908
Debaryomyces hansenii MTCC 234	14032	13861	20253	2671	225	deha_2_5_e00720	13543	0	0	0	0
Metschnikowia bicuspidata NRRL YB-4993	6088	81112	10587	13874	81113	mebi_1_8_h00300	18888	20386	4343	16107	7246
Babjeviella inositovora NRRLY-12698	0	0	0	0	0	bain_1_1_a00100	17993	17994	13292	13489	17995
	15155	12804	3184	9233	17920	bain_1_17_q00380	1774	17921	17922	17923	17924
	12657	19182	1801	106849	19183	bain_1_8_h00410	12617	13123	19184	14340	16780
