# Supplementary material for: Molecular evolution of the members of the Snq2/Pdr18 subfamily of Pdr transporters in the Hemiascomycete yeasts
Source: FEMS Yeast Res. 2025 May 27;25:foaf026. doi: 10.1093/femsyr/foaf026 (PMC12202755; doi:10.1093/femsyr/foaf026)
Supplement: foaf026_Supplemental_Files [file foaf026_supplemental_files.zip › Supplementary Data A13.pdf]

Motif 1 (LogOddsLogo)

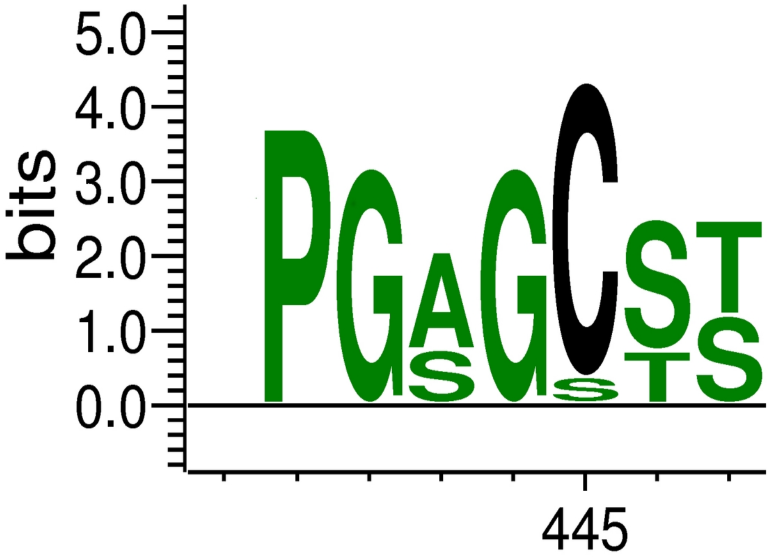

Motif 1 (frequency analysis)

Alanine	A	0.0	0.0	88.8	0.0	0.0	0.0	0.0
Arginine	R	0.0	0.0	0.0	0.0	0.0	0.0	0.0
Asparagine	N	0.0	0.0	0.0	0.0	0.0	0.0	0.0
Aspartate	D	0.0	0.0	0.0	0.0	0.0	0.0	0.0
Cysteine	C	0.0	0.0	0.0	0.0	99.5	0.0	0.0
Glutamine	Q	0.0	0.0	0.0	0.0	0.0	0.0	0.0
Glutamate	E	0.0	0.0	0.0	0.0	0.0	0.0	0.0
Glycine	G	0.0	100.0	0.0	100.0	0.0	0.0	0.0
Histidine	H	0.0	0.0	0.0	0.0	0.0	0.0	0.0
Isoleucine	I	0.0	0.0	0.0	0.0	0.0	0.0	0.0
Leucine	L	0.0	0.0	0.0	0.0	0.0	0.0	0.0
Lysine	K	0.0	0.0	0.0	0.0	0.0	0.0	0.0
Methionine	M	0.0	0.0	0.0	0.0	0.0	0.0	0.0
Phenylalanine	F	0.0	0.0	0.0	0.0	0.0	0.0	0.0
Proline	P	100.0	0.0	0.0	0.0	0.0	0.0	0.0
Serine	S	0.0	0.0	11.2	0.0	0.5	72.6	78.7
Threonine	T	0.0	0.0	0.0	0.0	0.0	27.4	21.3
Tryptophan	W	0.0	0.0	0.0	0.0	0.0	0.0	0.0
Tyrosine	Y	0.0	0.0	0.0	0.0	0.0	0.0	0.0
Valine	V	0.0	0.0	0.0	0.0	0.0	0.0	0.0
GAP	-	0.0	0.0	0.0	0.0	0.0	0.0	0.0
UNKNOWN AMINO ACID	X	0.0	0.0	0.0	0.0	0.0	0.0	0.0
		100.0	100.0	100.0	100.0	100.0	100.0	100.0
		P	G	A/S	G	C/S	S/T	S/T

Upstream sequence

Alanine	A	0.0	0.0	1.0	0.0	0.0	0.0
Arginine	R	0.0	0.0	0.0	0.0	0.0	99.0
Asparagine	N	0.0	0.0	0.0	0.0	0.0	0.0
Aspartate	D	0.0	0.0	0.0	0.0	0.0	0.0
Cysteine	C	4.6	0.0	0.0	0.0	0.0	0.0
Glutamine	Q	0.0	0.0	0.0	0.0	0.0	0.0
Glutamate	E	0.0	0.0	0.0	0.0	0.0	0.0
Glycine	G	0.0	0.0	0.0	0.0	100.0	0.0
Histidine	H	0.0	0.0	0.0	0.0	0.0	0.0
Isoleucine	I	28.4	0.0	0.0	0.0	0.0	0.0
Leucine	L	3.6	100.0	0.0	100.0	0.0	0.0
Lysine	K	0.0	0.0	0.0	0.0	0.0	1.0
Methionine	M	0.0	0.0	0.0	0.0	0.0	0.0
Phenylalanine	F	0.0	0.0	0.0	0.0	0.0	0.0
Proline	P	0.0	0.0	0.0	0.0	0.0	0.0
Serine	S	0.0	0.0	0.0	0.0	0.0	0.0
Threonine	T	5.6	0.0	0.0	0.0	0.0	0.0
Tryptophan	W	0.0	0.0	0.0	0.0	0.0	0.0
Tyrosine	Y	0.0	0.0	0.0	0.0	0.0	0.0
Valine	V	57.9	0.0	99.0	0.0	0.0	0.0
GAP	-	0.0	0.0	0.0	0.0	0.0	0.0
UNKNOWN AMINO ACID	X	0.0	0.0	0.0	0.0	0.0	0.0
		100.0	100.0	100.0	100.0	100.0	100.0
		X	L	V/A	L	G	R/K

Motif 2 (LogOddsLogo)

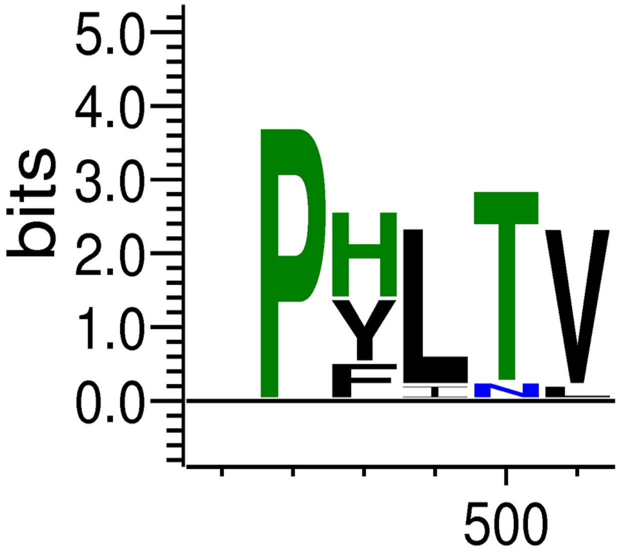

Motif 2 (frequency analysis)

Alanine	A	0.0	0.0	0.0	0.0	0.0
Arginine	R	0.0	0.0	0.0	0.0	0.0
Asparagine	N	0.0	0.0	0.0	0.5	0.0
Aspartate	D	0.0	0.0	0.0	0.0	0.0
Cysteine	C	0.0	0.0	0.0	0.0	0.0
Glutamine	Q	0.0	0.0	0.0	0.0	0.0
Glutamate	E	0.0	0.0	0.0	0.0	0.0
Glycine	G	0.0	0.0	0.0	0.0	0.0
Histidine	H	0.0	54.3	0.0	0.0	0.0
Isoleucine	I	0.0	0.0	0.5	0.0	0.0
Leucine	L	0.0	0.0	99.5	0.0	0.5
Lysine	K	0.0	0.0	0.0	0.0	0.0
Methionine	M	0.0	0.0	0.0	0.0	0.0
Phenylalanine	F	0.0	3.0	0.0	0.0	0.0
Proline	P	100.0	0.0	0.0	0.0	0.0
Serine	S	0.0	0.0	0.0	0.0	0.0
Threonine	T	0.0	0.0	0.0	99.5	0.0
Tryptophan	W	0.0	0.0	0.0	0.0	0.0
Tyrosine	Y	0.0	42.6	0.0	0.0	0.0
Valine	V	0.0	0.0	0.0	0.0	99.5
GAP	-	0.0	0.0	0.0	0.0	0.0
UNKNOWN AMINO ACID	X	0.0	0.0	0.0	0.0	0.0
		100.0	100.0	100.0	100.0	100.0
		P	H/Y/F	L/I	T/N	V/L

Upstream sequence

Alanine	A	0.0	0.0	0.0	0.5	0.0	0.0
Arginine	R	0.0	0.0	0.0	0.0	0.0	0.0
Asparagine	N	0.0	0.5	0.0	1.0	0.0	0.0
Aspartate	D	0.0	0.5	99.5	0.0	0.0	0.0
Cysteine	C	0.0	0.0	0.0	1.0	0.0	0.0
Glutamine	Q	0.0	29.9	0.0	0.0	0.0	0.0
Glutamate	E	100.0	0.5	0.5	1.5	0.0	0.0
Glycine	G	0.0	0.0	0.0	0.0	0.0	0.0
Histidine	H	0.0	1.0	0.0	0.0	100.0	0.0
Isoleucine	I	0.0	1.0	0.0	3.0	0.0	0.0
Leucine	L	0.0	58.9	0.0	1.0	0.0	1.0
Lysine	K	0.0	0.0	0.0	0.0	0.0	0.0
Methionine	M	0.0	5.6	0.0	0.0	0.0	0.0
Phenylalanine	F	0.0	0.0	0.0	0.0	0.0	97.0
Proline	P	0.0	0.0	0.0	0.0	0.0	0.0
Serine	S	0.0	0.0	0.0	0.0	0.0	0.0
Threonine	T	0.0	1.0	0.0	0.5	0.0	0.0
Tryptophan	W	0.0	0.0	0.0	0.0	0.0	0.0
Tyrosine	Y	0.0	0.5	0.0	0.0	0.0	2.0
Valine	V	0.0	0.5	0.0	91.4	0.0	0.0
GAP	-	0.0	0.0	0.0	0.0	0.0	0.0
UNKNOWN AMINO ACID	X	0.0	0.0	0.0	0.0	0.0	0.0
		100.0	100.0	100.0	100.0	100.0	100.0
		E	X	D/E	X	H	F/Y/L

Motif 3 (LogOddsLogo)

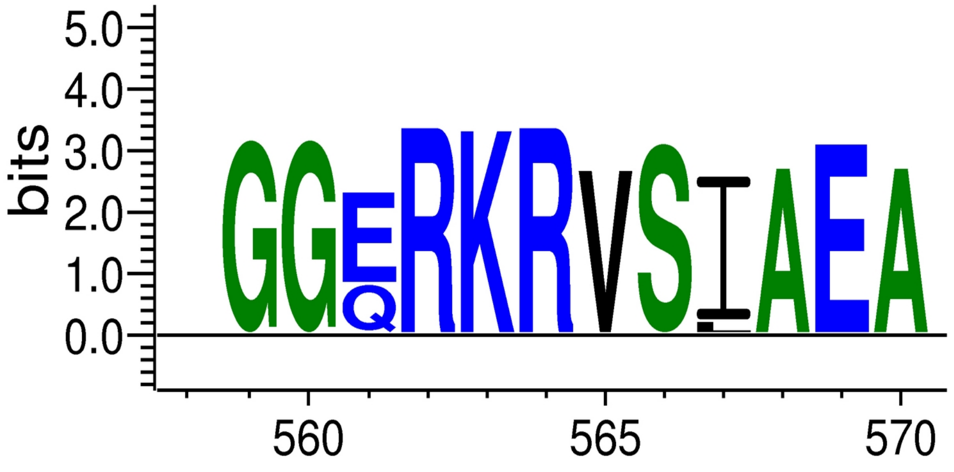

Motif 3 (frequency analysis)

Alanine	A	0.0	0.0	0.0	0.0	0.0	0.0	0.0	0.0	0.0	100.0	0.0	100.0
Arginine	R	0.0	0.0	0.0	100.0	0.0	100.0	0.0	0.0	0.0	0.0	0.0	0.0
Asparagine	N	0.0	0.0	0.0	0.0	0.0	0.0	0.0	0.0	0.0	0.0	0.0	0.0
Aspartate	D	0.0	0.0	0.0	0.0	0.0	0.0	0.0	0.0	0.0	0.0	0.0	0.0
Cysteine	C	0.0	0.0	0.0	0.0	0.0	0.0	0.0	0.0	0.0	0.0	0.0	0.0
Glutamine	Q	0.0	0.0	10.2	0.0	0.0	0.0	0.0	0.0	0.0	0.0	0.0	0.0
Glutamate	E	0.0	0.0	89.8	0.0	0.0	0.0	0.0	0.0	0.0	0.0	100.0	0.0
Glycine	G	100.0	100.0	0.0	0.0	0.0	0.0	0.0	0.0	0.0	0.0	0.0	0.0
Histidine	H	0.0	0.0	0.0	0.0	0.0	0.0	0.0	0.0	0.0	0.0	0.0	0.0
Isoleucine	I	0.0	0.0	0.0	0.0	0.0	0.0	0.0	0.0	99.5	0.0	0.0	0.0
Leucine	L	0.0	0.0	0.0	0.0	0.0	0.0	0.0	0.0	0.5	0.0	0.0	0.0
Lysine	K	0.0	0.0	0.0	0.0	100.0	0.0	0.0	0.0	0.0	0.0	0.0	0.0
Methionine	M	0.0	0.0	0.0	0.0	0.0	0.0	0.0	0.0	0.0	0.0	0.0	0.0
Phenylalanine	F	0.0	0.0	0.0	0.0	0.0	0.0	0.0	0.0	0.0	0.0	0.0	0.0
Proline	P	0.0	0.0	0.0	0.0	0.0	0.0	0.0	0.0	0.0	0.0	0.0	0.0
Serine	S	0.0	0.0	0.0	0.0	0.0	0.0	0.0	100.0	0.0	0.0	0.0	0.0
Threonine	T	0.0	0.0	0.0	0.0	0.0	0.0	0.0	0.0	0.0	0.0	0.0	0.0
Tryptophan	W	0.0	0.0	0.0	0.0	0.0	0.0	0.0	0.0	0.0	0.0	0.0	0.0
Tyrosine	Y	0.0	0.0	0.0	0.0	0.0	0.0	0.0	0.0	0.0	0.0	0.0	0.0
Valine	V	0.0	0.0	0.0	0.0	0.0	0.0	100.0	0.0	0.0	0.0	0.0	0.0
GAP	-	0.0	0.0	0.0	0.0	0.0	0.0	0.0	0.0	0.0	0.0	0.0	0.0
UNKNOWN AMINO ACID	X	0.0	0.0	0.0	0.0	0.0	0.0	0.0	0.0	0.0	0.0	0.0	0.0
		100.0	100.0	100.0	100.0	100.0	100.0	100.0	100.0	100.0	100.0	100.0	100.0
		G	G	E/Q	R	K	R	V	S	I/L	A	E	A

Upstream sequence

Alanine	A	0.0	0.0	0.0	0.0	0.0	0.0
Arginine	R	0.0	0.0	70.1	0.0	0.0	0.0
Asparagine	N	0.0	0.0	0.0	0.0	0.0	0.0
Aspartate	D	0.0	0.0	0.0	0.0	0.0	0.0
Cysteine	C	0.0	0.0	0.0	0.0	0.0	0.0
Glutamine	Q	0.0	0.0	0.0	0.0	0.0	0.0
Glutamate	E	0.0	0.0	0.0	0.0	0.0	0.0
Glycine	G	0.0	0.0	0.0	100.0	0.0	0.0
Histidine	H	0.0	0.0	0.0	0.0	0.0	0.0
Isoleucine	I	0.0	29.9	0.0	0.0	6.6	0.0
Leucine	L	1.5	0.0	0.0	0.0	0.0	0.0
Lysine	K	0.0	0.0	0.0	0.0	0.0	0.0
Methionine	M	0.5	0.0	0.0	0.0	0.0	0.0
Phenylalanine	F	92.4	1.0	0.0	0.0	0.0	0.0
Proline	P	0.0	0.0	0.0	0.0	0.0	0.0
Serine	S	0.0	0.0	29.9	0.0	0.0	100.0
Threonine	T	0.0	0.5	0.0	0.0	0.0	0.0
Tryptophan	W	0.0	0.0	0.0	0.0	0.0	0.0
Tyrosine	Y	4.6	0.0	0.0	0.0	0.0	0.0
Valine	V	1.0	68.5	0.0	0.0	93.4	0.0
GAP	-	0.0	0.0	0.0	0.0	0.0	0.0
UNKNOWN AMINO ACID	X	0.0	0.0	0.0	0.0	0.0	0.0
		100.0	100.0	100.0	100.0	100.0	100.0
		X	X	R/S	G	V/I	S

Motif 4 (LogOddsLogo)

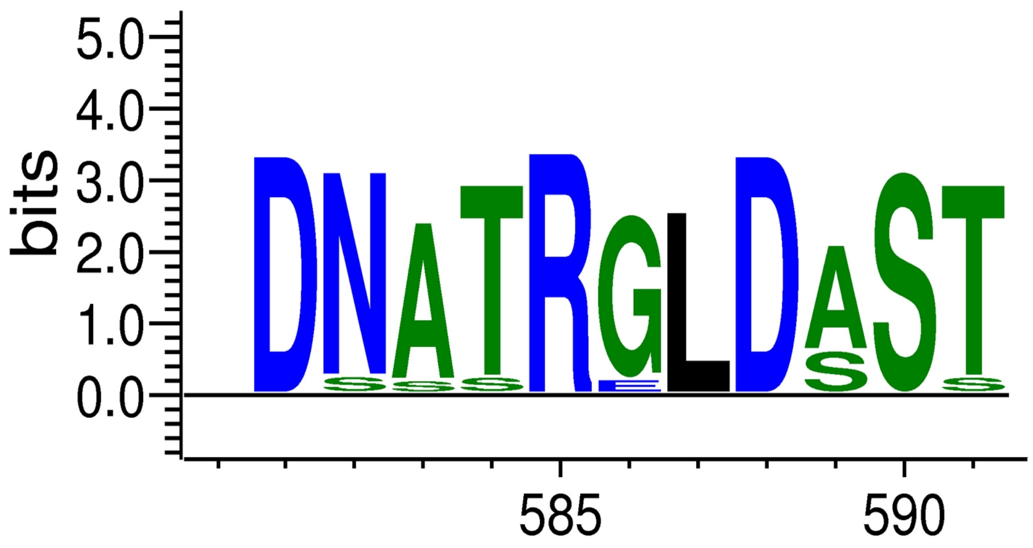

Motif 4 (frequency analysis)

Alanine	A	0.0	0.0	99.5	0.0	0.0	0.0	0.0	0.0	85.3	0.0	0.0
Arginine	R	0.0	0.0	0.0	0.0	100.0	0.0	0.0	0.0	0.0	0.0	0.0
Asparagine	N	0.0	99.5	0.0	0.0	0.0	0.0	0.0	0.0	0.0	0.0	0.0
Aspartate	D	100.0	0.0	0.0	0.0	0.0	0.0	0.0	100.0	0.0	0.0	0.0
Cysteine	C	0.0	0.0	0.0	0.0	0.0	0.0	0.0	0.0	0.0	0.0	0.0
Glutamine	Q	0.0	0.0	0.0	0.0	0.0	0.0	0.0	0.0	0.0	0.0	0.0
Glutamate	E	0.0	0.0	0.0	0.0	0.0	0.5	0.0	0.0	0.0	0.0	0.0
Glycine	G	0.0	0.0	0.0	0.0	0.0	99.5	0.0	0.0	0.0	0.0	0.0
Histidine	H	0.0	0.0	0.0	0.0	0.0	0.0	0.0	0.0	0.0	0.0	0.0
Isoleucine	I	0.0	0.0	0.0	0.0	0.0	0.0	0.0	0.0	0.0	0.0	0.0
Leucine	L	0.0	0.0	0.0	0.0	0.0	0.0	100.0	0.0	0.0	0.0	0.0
Lysine	K	0.0	0.0	0.0	0.0	0.0	0.0	0.0	0.0	0.0	0.0	0.0
Methionine	M	0.0	0.0	0.0	0.0	0.0	0.0	0.0	0.0	0.0	0.0	0.0
Phenylalanine	F	0.0	0.0	0.0	0.0	0.0	0.0	0.0	0.0	0.0	0.0	0.0
Proline	P	0.0	0.0	0.0	0.0	0.0	0.0	0.0	0.0	0.0	0.0	0.0
Serine	S	0.0	0.5	0.5	0.5	0.0	0.0	0.0	0.0	13.7	100.0	0.5
Threonine	T	0.0	0.0	0.0	99.5	0.0	0.0	0.0	0.0	0.0	0.0	99.5
Tryptophan	W	0.0	0.0	0.0	0.0	0.0	0.0	0.0	0.0	0.0	0.0	0.0
Tyrosine	Y	0.0	0.0	0.0	0.0	0.0	0.0	0.0	0.0	0.0	0.0	0.0
Valine	V	0.0	0.0	0.0	0.0	0.0	0.0	0.0	0.0	0.0	0.0	0.0
GAP	-	0.0	0.0	0.0	0.0	0.0	0.0	0.0	0.0	0.0	0.0	0.0
UNKNOWN AMINO ACID	X	0.0	0.0	0.0	0.0	0.0	0.0	0.0	0.0	1.0	0.0	0.0
		100.0	100.0	100.0	100.0	100.0	100.0	100.0	100.0	100.0	100.0	100.0
		D	N/S	A/S	T/S	R	G/E	L	D	A/S/X	S	T/S

Upstream sequence

Alanine	A	7.6	1.0	0.0	0.0	1.0	0.0
Arginine	R	0.0	0.0	0.0	0.0	0.0	0.0
Asparagine	N	0.0	0.5	0.0	0.0	0.0	0.0
Aspartate	D	0.0	0.0	0.0	0.0	0.0	0.0
Cysteine	C	0.0	1.0	0.0	0.0	97.5	0.0
Glutamine	Q	0.0	0.0	0.0	0.0	0.0	0.0
Glutamate	E	0.0	0.0	0.0	0.0	0.0	0.0
Glycine	G	92.4	0.0	0.0	0.0	0.0	0.0
Histidine	H	0.0	0.0	0.0	0.0	0.0	0.0
Isoleucine	I	0.0	0.0	77.7	0.0	0.0	0.0
Leucine	L	0.0	0.0	1.5	0.0	0.5	0.0
Lysine	K	0.0	1.0	0.0	0.0	0.0	0.0
Methionine	M	0.0	0.5	0.0	0.0	0.0	0.0
Phenylalanine	F	0.0	0.0	0.5	1.5	0.5	4.1
Proline	P	0.0	0.5	0.0	0.0	0.0	0.0
Serine	S	0.0	80.7	0.0	0.0	0.5	0.0
Threonine	T	0.0	14.7	0.0	0.5	0.0	0.0
Tryptophan	W	0.0	0.0	0.0	0.0	0.0	94.4
Tyrosine	Y	0.0	0.0	0.0	98.0	0.0	1.5
Valine	V	0.0	0.0	20.3	0.0	0.0	0.0
GAP	-	0.0	0.0	0.0	0.0	0.0	0.0
UNKNOWN AMINO ACID	X	0.0	0.0	0.0	0.0	0.0	0.0
		100.0	100.0	100.0	100.0	100.0	100.0
		G/A	X	X	Y/F/T	X	W/F/Y

Motif 5 (LogOddsLogo)

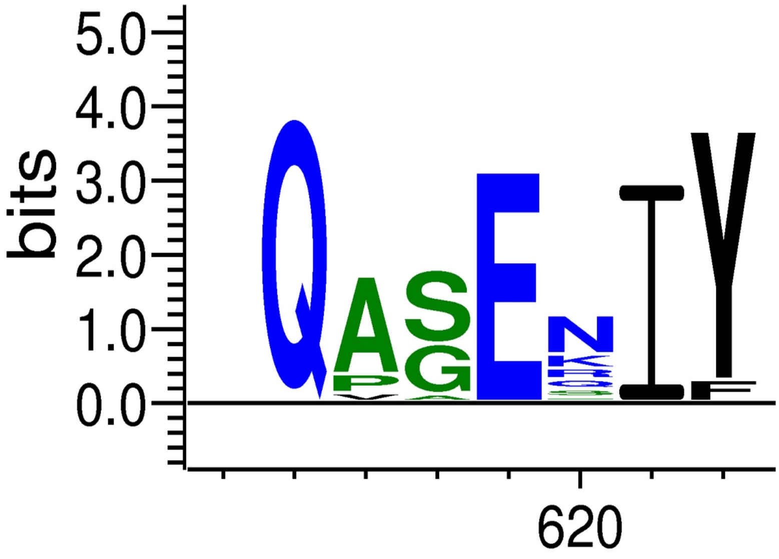

Motif 5 (frequency analysis)

Alanine	A	0.0	98.0	0.5	0.0	0.0	0.0	0.0
Arginine	R	0.0	0.0	0.0	0.0	2.0	0.0	0.0
Asparagine	N	0.0	0.0	0.0	0.0	87.3	0.0	0.0
Aspartate	D	0.0	0.0	0.0	0.0	0.0	0.0	0.0
Cysteine	C	0.0	0.0	0.0	0.0	0.0	0.0	0.0
Glutamine	Q	100.0	0.0	0.0	0.0	1.5	0.0	0.0
Glutamate	E	0.0	0.0	0.0	100.0	0.0	0.0	0.0
Glycine	G	0.0	0.0	15.2	0.0	5.6	0.0	0.0
Histidine	H	0.0	0.0	0.0	0.0	0.0	0.0	0.0
Isoleucine	I	0.0	0.0	0.0	0.0	0.0	100.0	0.0
Leucine	L	0.0	0.0	0.0	0.0	0.0	0.0	0.0
Lysine	K	0.0	0.0	0.0	0.0	2.5	0.0	0.0
Methionine	M	0.0	0.0	0.0	0.0	0.0	0.0	0.0
Phenylalanine	F	0.0	0.0	0.0	0.0	0.0	0.0	0.5
Proline	P	0.0	1.5	0.0	0.0	0.0	0.0	0.0
Serine	S	0.0	0.0	84.3	0.0	1.0	0.0	0.0
Threonine	T	0.0	0.0	0.0	0.0	0.0	0.0	0.0
Tryptophan	W	0.0	0.0	0.0	0.0	0.0	0.0	0.0
Tyrosine	Y	0.0	0.0	0.0	0.0	0.0	0.0	99.5
Valine	V	0.0	0.5	0.0	0.0	0.0	0.0	0.0
GAP	-	0.0	0.0	0.0	0.0	0.0	0.0	0.0
UNKNOWN AMINO ACID	X	0.0	0.0	0.0	0.0	0.0	0.0	0.0
		100.0	100.0	100.0	100.0	100.0	100.0	100.0
		Q	A/P/Y	S/G/A	E	X	I	Y/F

Upstream sequence

Alanine	A	85.3	0.0	0.0	1.5	1.5	0.0
Arginine	R	0.0	0.0	0.0	0.0	0.0	0.0
Asparagine	N	0.0	0.0	0.0	0.0	0.0	0.0
Aspartate	D	0.0	0.0	0.0	0.0	0.0	0.0
Cysteine	C	0.5	0.0	0.5	0.5	0.0	0.0
Glutamine	Q	0.0	0.0	0.0	0.0	0.0	0.0
Glutamate	E	0.0	0.0	0.0	0.0	0.0	0.0
Glycine	G	0.0	0.0	0.0	0.0	0.0	0.0
Histidine	H	0.0	0.0	0.0	0.0	0.0	0.0
Isoleucine	I	0.0	1.5	18.8	0.0	75.1	0.0
Leucine	L	0.0	38.6	0.0	0.0	6.1	0.0
Lysine	K	0.0	0.0	0.0	0.0	0.0	0.0
Methionine	M	0.0	0.0	0.0	0.0	0.0	0.0
Phenylalanine	F	0.0	57.4	0.0	0.0	0.0	0.0
Proline	P	0.0	0.0	0.0	0.0	0.0	0.0
Serine	S	10.2	0.0	0.0	2.5	0.0	0.0
Threonine	T	1.0	0.0	0.0	95.4	0.0	0.0
Tryptophan	W	0.0	0.0	0.0	0.0	0.0	0.0
Tyrosine	Y	0.0	0.0	0.0	0.0	0.0	100.0
Valine	V	3.0	2.5	80.7	0.0	17.3	0.0
GAP	-	0.0	0.0	0.0	0.0	0.0	0.0
UNKNOWN AMINO ACID	X	0.0	0.0	0.0	0.0	0.0	0.0
		100.0	100.0	100.0	100.0	100.0	100.0
		X	X	V/I/C	X	X	Y

Motif 6 (LogOddsLogo)

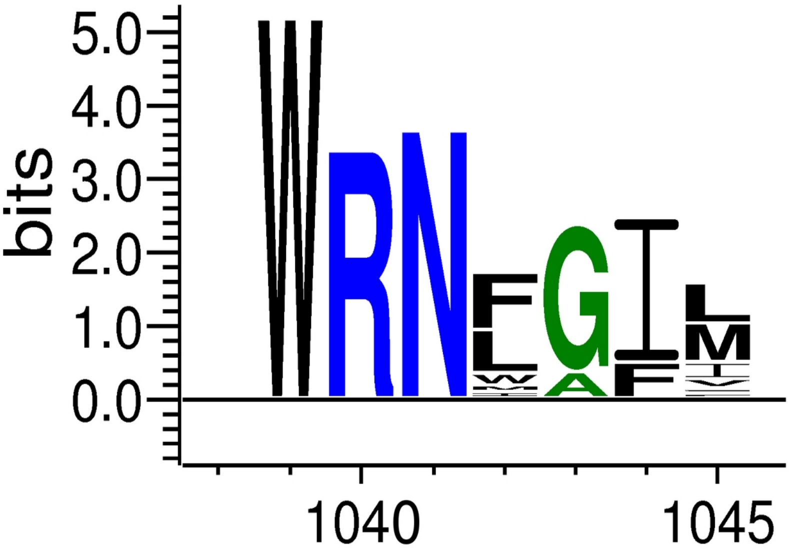

Motif 6 (frequency analysis)

Alanine	A	0.0	0.0	0.0	0.0	1.0	0.0	0.0
Arginine	R	0.0	100.0	0.0	0.0	0.0	0.0	0.0
Asparagine	N	0.0	0.0	100.0	0.0	0.0	0.0	0.0
Aspartate	D	0.0	0.0	0.0	0.0	0.0	0.0	0.0
Cysteine	C	0.0	0.0	0.0	0.0	0.0	0.0	0.5
Glutamine	Q	0.0	0.0	0.0	0.0	0.0	0.0	0.0
Glutamate	E	0.0	0.0	0.0	0.0	0.0	0.0	0.0
Glycine	G	0.0	0.0	0.0	0.0	99.0	0.0	0.0
Histidine	H	0.0	0.0	0.0	0.0	0.0	0.0	0.0
Isoleucine	I	0.0	0.0	0.0	0.5	0.0	98.0	2.5
Leucine	L	0.0	0.0	0.0	16.8	0.0	0.0	37.6
Lysine	K	0.0	0.0	0.0	0.0	0.0	0.0	0.0
Methionine	M	0.0	0.0	0.0	1.0	0.0	0.0	57.9
Phenylalanine	F	0.0	0.0	0.0	76.6	0.0	2.0	0.5
Proline	P	0.0	0.0	0.0	0.0	0.0	0.0	0.0
Serine	S	0.0	0.0	0.0	0.0	0.0	0.0	0.0
Threonine	T	0.0	0.0	0.0	0.0	0.0	0.0	0.0
Tryptophan	W	100.0	0.0	0.0	5.1	0.0	0.0	0.0
Tyrosine	Y	0.0	0.0	0.0	0.0	0.0	0.0	0.0
Valine	V	0.0	0.0	0.0	0.0	0.0	0.0	1.0
GAP	-	0.0	0.0	0.0	0.0	0.0	0.0	0.0
UNKNOWN AMINO ACID	X	0.0	0.0	0.0	0.0	0.0	0.0	0.0
		100.0	100.0	100.0	100.0	100.0	100.0	100.0
		W	R	N	X	G/A	I/F	X

Upstream sequence

Alanine	A	0.0	0.5	0.0	1.0	0.0	0.5
Arginine	R	0.0	7.6	0.0	0.5	0.0	0.0
Asparagine	N	0.0	0.0	0.0	2.0	32.0	0.5
Aspartate	D	0.0	1.0	0.0	1.5	0.0	0.0
Cysteine	C	0.0	0.0	0.0	0.0	0.0	2.5
Glutamine	Q	0.0	1.5	0.0	0.0	0.0	3.0
Glutamate	E	0.0	36.0	0.0	0.5	0.0	0.5
Glycine	G	0.0	0.0	0.0	2.0	0.0	0.0
Histidine	H	0.0	0.0	0.5	0.0	67.5	0.0
Isoleucine	I	0.0	1.0	0.0	0.5	0.0	3.0
Leucine	L	0.0	0.0	0.0	0.0	0.0	4.1
Lysine	K	0.0	11.2	0.0	62.4	0.0	0.0
Methionine	M	0.0	0.0	0.0	0.0	0.0	5.6
Phenylalanine	F	2.0	0.5	16.8	0.0	0.0	0.0
Proline	P	0.0	0.0	0.0	0.5	0.0	0.0
Serine	S	0.0	6.6	0.0	20.8	0.0	0.0
Threonine	T	0.0	6.6	0.0	1.5	0.0	62.4
Tryptophan	W	0.0	0.0	1.5	6.6	0.0	0.0
Tyrosine	Y	98.0	0.0	81.2	0.0	0.5	0.0
Valine	V	0.0	27.4	0.0	0.0	0.0	17.8
GAP	-	0.0	0.0	0.0	0.0	0.0	0.0
UNKNOWN AMINO ACID	X	0.0	0.0	0.0	0.0	0.0	0.0
		100.0	100.0	100.0	100.0	100.0	100.0
		Y/F	X	X	X	H/N/Y	X

Motif 7 (LogOddsLogo)

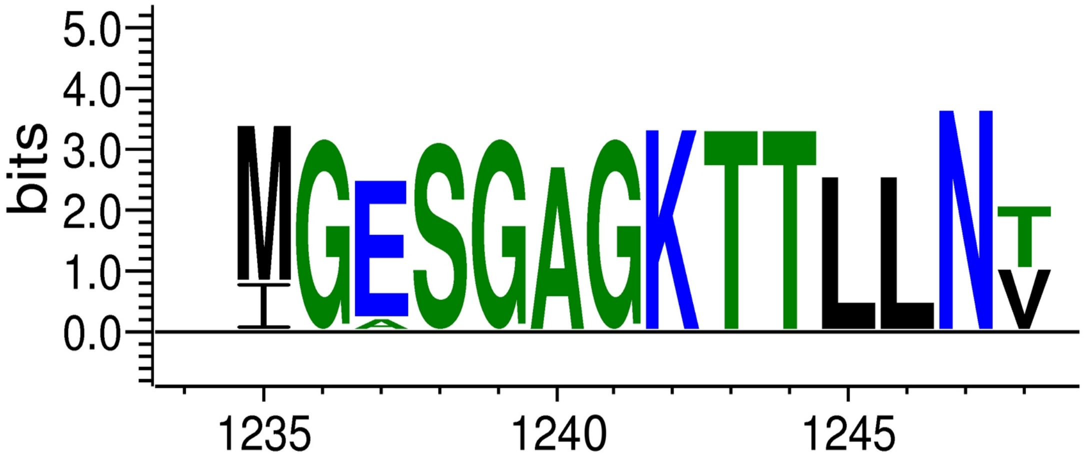

Motif 7 (frequency analysis)

Alanine	A	0.0	0.0	0.5	0.0	0.0	100.0	0.0	0.0	0.0	0.0	0.0	0.0	0.0	0.0
Arginine	R	0.0	0.0	0.0	0.0	0.0	0.0	0.0	0.0	0.0	0.0	0.0	0.0	0.0	0.0
Asparagine	N	0.0	0.0	0.0	0.0	0.0	0.0	0.0	0.0	0.0	0.0	0.0	0.0	100.0	0.0
Aspartate	D	0.0	0.0	0.0	0.0	0.0	0.0	0.0	0.0	0.0	0.0	0.0	0.0	0.0	0.0
Cysteine	C	0.0	0.0	0.0	0.0	0.0	0.0	0.0	0.0	0.0	0.0	0.0	0.0	0.0	0.0
Glutamine	Q	0.0	0.0	0.0	0.0	0.0	0.0	0.0	0.0	0.0	0.0	0.0	0.0	0.0	0.0
Glutamate	E	0.0	0.0	99.5	0.0	0.0	0.0	0.0	0.0	0.0	0.0	0.0	0.0	0.0	0.0
Glycine	G	0.0	100.0	0.0	0.0	100.0	0.0	100.0	0.0	0.0	0.0	0.0	0.0	0.0	0.0
Histidine	H	0.0	0.0	0.0	0.0	0.0	0.0	0.0	0.0	0.0	0.0	0.0	0.0	0.0	0.0
Isoleucine	I	31.5	0.0	0.0	0.0	0.0	0.0	0.0	0.0	0.0	0.0	0.0	0.0	0.0	0.0
Leucine	L	0.0	0.0	0.0	0.0	0.0	0.0	0.0	0.0	0.0	0.0	100.0	100.0	0.0	0.0
Lysine	K	0.0	0.0	0.0	0.0	0.0	0.0	0.0	100.0	0.0	0.0	0.0	0.0	0.0	0.0
Methionine	M	68.5	0.0	0.0	0.0	0.0	0.0	0.0	0.0	0.0	0.0	0.0	0.0	0.0	0.0
Phenylalanine	F	0.0	0.0	0.0	0.0	0.0	0.0	0.0	0.0	0.0	0.0	0.0	0.0	0.0	0.0
Proline	P	0.0	0.0	0.0	0.0	0.0	0.0	0.0	0.0	0.0	0.0	0.0	0.0	0.0	0.0
Serine	S	0.0	0.0	0.0	100.0	0.0	0.0	0.0	0.0	0.0	0.0	0.0	0.0	0.0	0.0
Threonine	T	0.0	0.0	0.0	0.0	0.0	0.0	0.0	0.0	100.0	100.0	0.0	0.0	0.0	81.7
Tryptophan	W	0.0	0.0	0.0	0.0	0.0	0.0	0.0	0.0	0.0	0.0	0.0	0.0	0.0	0.0
Tyrosine	Y	0.0	0.0	0.0	0.0	0.0	0.0	0.0	0.0	0.0	0.0	0.0	0.0	0.0	0.0
Valine	V	0.0	0.0	0.0	0.0	0.0	0.0	0.0	0.0	0.0	0.0	0.0	0.0	0.0	18.3
GAP	-	0.0	0.0	0.0	0.0	0.0	0.0	0.0	0.0	0.0	0.0	0.0	0.0	0.0	0.0
UNKNOWN AMINO ACID	X	0.0	0.0	0.0	0.0	0.0	0.0	0.0	0.0	0.0	0.0	0.0	0.0	0.0	0.0
		100.0	100.0	100.0	100.0	100.0	100.0	100.0	100.0	100.0	100.0	100.0	100.0	100.0	100.0
		M/I	G	E/A	S	G	A	G	K	T	T	L	L	N	T/V

Upstream sequence

Alanine	A	0.0	0.5	0.0	0.0	100.0	0.0
Arginine	R	0.0	0.0	0.0	0.0	0.0	0.0
Asparagine	N	0.0	0.0	0.0	0.0	0.0	0.0
Aspartate	D	0.0	0.0	0.0	0.0	0.0	0.0
Cysteine	C	0.0	0.0	0.0	0.0	0.0	0.0
Glutamine	Q	0.0	1.0	0.0	0.0	0.0	0.0
Glutamate	E	0.0	0.0	0.0	0.0	0.0	0.0
Glycine	G	100.0	0.0	0.0	0.0	0.0	0.0
Histidine	H	0.0	0.0	0.0	0.0	0.0	0.0
Isoleucine	I	0.0	0.0	2.0	0.0	0.0	0.0
Leucine	L	0.0	0.0	61.9	0.0	0.0	100.0
Lysine	K	0.0	1.0	0.0	0.0	0.0	0.0
Methionine	M	0.0	0.0	36.0	0.0	0.0	0.0
Phenylalanine	F	0.0	0.0	0.0	0.0	0.0	0.0
Proline	P	0.0	0.0	0.0	0.0	0.0	0.0
Serine	S	0.0	1.0	0.0	0.0	0.0	0.0
Threonine	T	0.0	95.9	0.0	99.0	0.0	0.0
Tryptophan	W	0.0	0.0	0.0	0.0	0.0	0.0
Tyrosine	Y	0.0	0.0	0.0	0.0	0.0	0.0
Valine	V	0.0	0.0	0.0	1.0	0.0	0.0
GAP	-	0.0	0.5	0.0	0.0	0.0	0.0
UNKNOWN AMINO ACID	X	0.0	0.0	0.0	0.0	0.0	0.0
		100.0	100.0	100.0	100.0	100.0	100.0
		G	X	L/M/I	T/V	A	L

Motif 8 (LogOddsLogo)

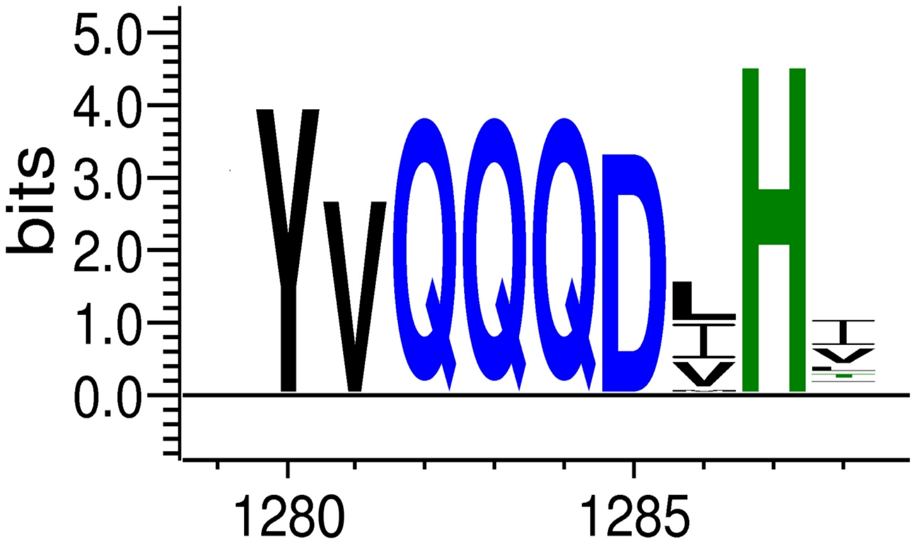

Motif 8 (frequency analysis)

Alanine	A	0.0	0.0	0.0	0.0	0.0	0.0	0.0	0.0	0.5
Arginine	R	0.0	0.0	0.0	0.0	0.0	0.0	0.0	0.0	0.0
Asparagine	N	0.0	0.0	0.0	0.0	0.0	0.0	0.0	0.0	0.0
Aspartate	D	0.0	0.0	0.0	0.0	0.0	100.0	0.0	0.0	0.0
Cysteine	C	0.0	0.0	0.0	0.0	0.0	0.0	0.0	0.0	1.0
Glutamine	Q	0.0	0.0	100.0	100.0	100.0	0.0	0.0	0.0	0.0
Glutamate	E	0.0	0.0	0.0	0.0	0.0	0.0	0.0	0.0	0.0
Glycine	G	0.0	0.0	0.0	0.0	0.0	0.0	0.0	0.0	0.0
Histidine	H	0.0	0.0	0.0	0.0	0.0	0.0	0.0	100.0	0.0
Isoleucine	I	0.0	0.0	0.0	0.0	0.0	0.0	49.7	0.0	64.0
Leucine	L	0.0	0.0	0.0	0.0	0.0	0.0	40.6	0.0	1.5
Lysine	K	0.0	0.0	0.0	0.0	0.0	0.0	0.0	0.0	0.0
Methionine	M	0.0	0.0	0.0	0.0	0.0	0.0	0.5	0.0	0.5
Phenylalanine	F	0.0	0.0	0.0	0.0	0.0	0.0	0.0	0.0	5.6
Proline	P	0.0	0.0	0.0	0.0	0.0	0.0	0.0	0.0	0.0
Serine	S	0.0	0.0	0.0	0.0	0.0	0.0	0.0	0.0	0.0
Threonine	T	0.0	0.0	0.0	0.0	0.0	0.0	0.0	0.0	1.5
Tryptophan	W	0.0	0.0	0.0	0.0	0.0	0.0	0.0	0.0	0.0
Tyrosine	Y	100.0	0.0	0.0	0.0	0.0	0.0	0.0	0.0	0.0
Valine	V	0.0	100.0	0.0	0.0	0.0	0.0	9.1	0.0	25.4
GAP	-	0.0	0.0	0.0	0.0	0.0	0.0	0.0	0.0	0.0
UNKNOWN AMINO ACID	X	0.0	0.0	0.0	0.0	0.0	0.0	0.0	0.0	0.0
		100.0	100.0	100.0	100.0	100.0	100.0	100.0	100.0	100.0
		Y	V	Q	Q	Q	D	X	H	X

Upstream sequence

Alanine	A	0.0	0.5	0.0	0.0	0.5	0.0
Arginine	R	0.0	1.0	98.0	95.9	0.0	0.0
Asparagine	N	0.0	0.0	0.0	0.0	0.0	0.0
Aspartate	D	0.0	0.0	0.0	0.0	0.0	0.0
Cysteine	C	0.0	0.0	0.0	0.0	0.0	0.0
Glutamine	Q	0.0	2.5	0.0	0.0	0.0	0.0
Glutamate	E	0.0	52.3	0.0	0.0	0.0	0.0
Glycine	G	0.0	0.5	0.0	0.0	0.0	100.0
Histidine	H	0.0	0.5	0.0	0.0	0.0	0.0
Isoleucine	I	0.0	0.0	0.0	0.0	1.0	0.0
Leucine	L	0.0	0.0	0.0	0.0	0.0	0.0
Lysine	K	0.0	32.0	1.5	0.5	0.0	0.0
Methionine	M	0.0	0.0	0.0	0.0	0.0	0.0
Phenylalanine	F	99.5	0.0	0.0	0.0	0.0	0.0
Proline	P	0.0	0.0	0.0	0.0	0.0	0.0
Serine	S	0.5	10.2	0.5	3.6	0.0	0.0
Threonine	T	0.0	0.0	0.0	0.0	98.0	0.0
Tryptophan	W	0.0	0.0	0.0	0.0	0.0	0.0
Tyrosine	Y	0.0	0.0	0.0	0.0	0.0	0.0
Valine	V	0.0	0.5	0.0	0.0	0.5	0.0
GAP	-	0.0	0.0	0.0	0.0	0.0	0.0
UNKNOWN AMINO ACID	X	0.0	0.0	0.0	0.0	0.0	0.0
		100.0	100.0	100.0	100.0	100.0	100.0
		F/S	X	R/K/S	R/S/K	T/V	G

Motif 9 (LogOddsLogo)

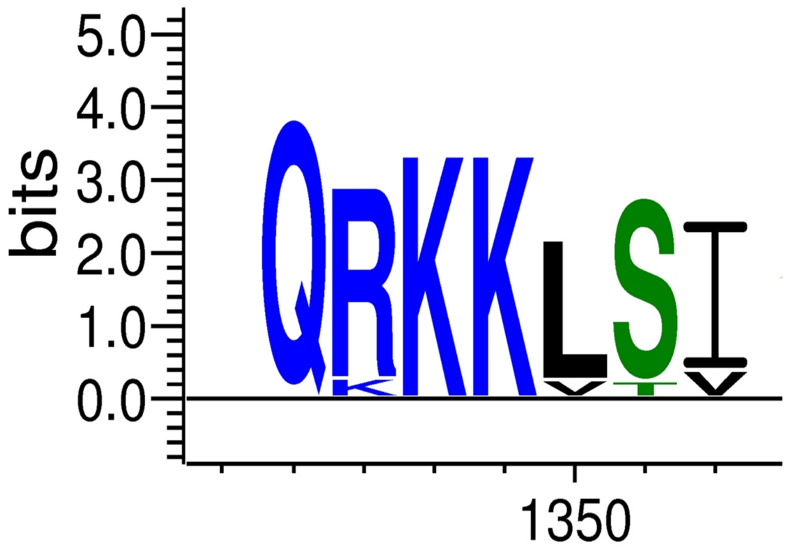

Motif 9 (frequency analysis)

Alanine	A	0.0	0.0	0.0	0.0	0.0	0.0	0.0
Arginine	R	0.0	99.0	0.0	0.0	0.0	0.0	0.0
Asparagine	N	0.0	0.0	0.0	0.0	0.0	0.0	0.0
Aspartate	D	0.0	0.0	0.0	0.0	0.0	0.0	0.0
Cysteine	C	0.0	0.0	0.0	0.0	0.0	0.0	0.0
Glutamine	Q	100.0	0.0	0.0	0.0	0.0	0.0	0.0
Glutamate	E	0.0	0.0	0.0	0.0	0.0	0.0	0.0
Glycine	G	0.0	0.0	0.0	0.0	0.0	0.0	0.0
Histidine	H	0.0	0.0	0.0	0.0	0.0	0.0	0.0
Isoleucine	I	0.0	0.0	0.0	0.0	0.0	0.0	97.0
Leucine	L	0.0	0.0	0.0	0.0	99.0	0.0	0.0
Lysine	K	0.0	1.0	100.0	100.0	0.0	0.0	0.0
Methionine	M	0.0	0.0	0.0	0.0	0.0	0.0	0.0
Phenylalanine	F	0.0	0.0	0.0	0.0	0.0	0.0	0.0
Proline	P	0.0	0.0	0.0	0.0	0.0	0.0	0.0
Serine	S	0.0	0.0	0.0	0.0	0.0	99.5	0.0
Threonine	T	0.0	0.0	0.0	0.0	0.0	0.5	0.0
Tryptophan	W	0.0	0.0	0.0	0.0	0.0	0.0	0.0
Tyrosine	Y	0.0	0.0	0.0	0.0	0.0	0.0	0.0
Valine	V	0.0	0.0	0.0	0.0	1.0	0.0	3.0
GAP	-	0.0	0.0	0.0	0.0	0.0	0.0	0.0
UNKNOWN AMINO ACID	X	0.0	0.0	0.0	0.0	0.0	0.0	0.0
		100.0	100.0	100.0	100.0	100.0	100.0	100.0
		Q	R/K	K	K	L/V	S/T	I/V

Upstream sequence

Alanine	A	5.6	0.0	0.0	0.0	0.0	0.0
Arginine	R	3.6	0.0	0.0	0.0	0.0	0.0
Asparagine	N	10.7	0.0	0.0	99.5	0.0	0.0
Aspartate	D	2.0	0.0	0.0	0.0	0.0	0.0
Cysteine	C	28.9	0.0	0.0	0.0	0.0	0.0
Glutamine	Q	0.0	0.0	0.0	0.0	0.0	0.0
Glutamate	E	0.0	0.0	0.0	0.0	0.0	99.5
Glycine	G	0.0	100.0	0.0	0.0	0.0	0.5
Histidine	H	0.5	0.0	0.0	0.0	0.0	0.0
Isoleucine	I	0.0	0.0	0.0	0.0	0.0	0.0
Leucine	L	0.0	0.0	100.0	0.0	0.0	0.0
Lysine	K	0.0	0.0	0.0	0.0	0.0	0.0
Methionine	M	0.5	0.0	0.0	0.0	0.0	0.0
Phenylalanine	F	1.0	0.0	0.0	0.0	0.0	0.0
Proline	P	0.0	0.0	0.0	0.0	0.0	0.0
Serine	S	5.1	0.0	0.0	0.5	0.0	0.0
Threonine	T	0.0	0.0	0.0	0.0	0.0	0.0
Tryptophan	W	0.0	0.0	0.0	0.0	0.0	0.0
Tyrosine	Y	42.1	0.0	0.0	0.0	0.0	0.0
Valine	V	0.0	0.0	0.0	0.0	100.0	0.0
GAP	-	0.0	0.0	0.0	0.0	0.0	0.0
UNKNOWN AMINO ACID	X	0.0	0.0	0.0	0.0	0.0	0.0
		100.0	100.0	100.0	100.0	100.0	100.0
		X	G	L	N/S	V	E/G

Motif 10 (LogOddsLogo)

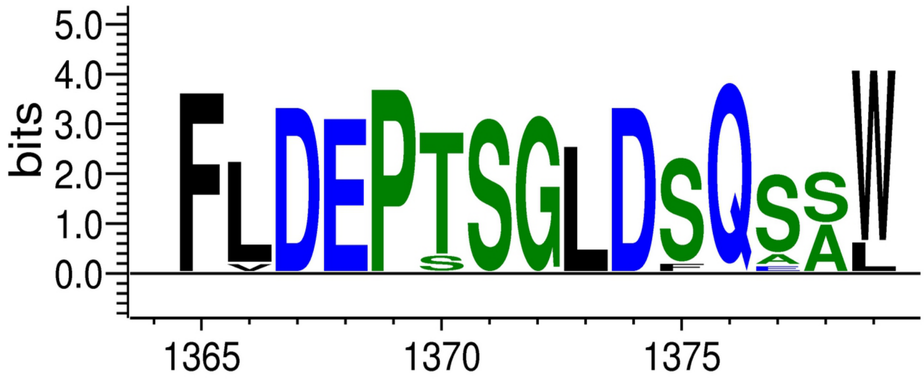

Motif 10 (frequency analysis)

Alanine	A	0.0	0.0	0.0	0.0	0.0	0.0	0.0	0.0	0.0	0.0	0.0	0.0	1.0	50.3	0.0
Arginine	R	0.0	0.0	0.0	0.0	0.0	0.0	0.0	0.0	0.0	0.0	0.0	0.0	0.0	0.0	0.0
Asparagine	N	0.0	0.0	0.0	0.0	0.0	0.0	0.0	0.0	0.0	0.0	0.0	0.0	0.0	0.0	0.0
Aspartate	D	0.0	0.0	100.0	0.0	0.0	0.0	0.0	0.0	0.0	100.0	0.0	0.0	0.0	0.0	0.0
Cysteine	C	0.0	0.0	0.0	0.0	0.0	0.0	0.0	0.0	0.0	0.0	0.0	0.0	0.0	0.0	0.0
Glutamine	Q	0.0	0.0	0.0	0.0	0.0	0.0	0.0	0.0	0.0	0.0	0.0	100.0	0.0	0.0	0.0
Glutamate	E	0.0	0.0	0.0	100.0	0.0	0.0	0.0	0.0	0.0	0.0	0.0	0.0	0.5	0.0	0.0
Glycine	G	0.0	0.0	0.0	0.0	0.0	0.0	0.0	100.0	0.0	0.0	0.0	0.0	0.0	0.0	0.0
Histidine	H	0.0	0.0	0.0	0.0	0.0	0.0	0.0	0.0	0.0	0.0	0.0	0.0	0.0	0.0	0.0
Isoleucine	I	0.0	0.0	0.0	0.0	0.0	0.0	0.0	0.0	0.0	0.0	0.0	0.0	0.0	0.0	0.0
Leucine	L	0.0	99.5	0.0	0.0	0.0	0.0	0.0	0.0	100.0	0.0	0.0	0.0	0.0	0.0	1.0
Lysine	K	0.0	0.0	0.0	0.0	0.0	0.0	0.0	0.0	0.0	0.0	0.0	0.0	0.0	0.0	0.0
Methionine	M	0.0	0.0	0.0	0.0	0.0	0.0	0.0	0.0	0.0	0.0	0.0	0.0	0.0	0.0	0.0
Phenylalanine	F	100.0	0.0	0.0	0.0	0.0	0.0	0.0	0.0	0.0	0.0	0.5	0.0	0.0	0.0	0.0
Proline	P	0.0	0.0	0.0	0.0	100.0	0.0	0.0	0.0	0.0	0.0	0.0	0.0	0.0	0.0	0.0
Serine	S	0.0	0.0	0.0	0.0	0.0	1.0	100.0	0.0	0.0	0.0	99.5	0.0	98.5	49.7	0.0
Threonine	T	0.0	0.0	0.0	0.0	0.0	99.0	0.0	0.0	0.0	0.0	0.0	0.0	0.0	0.0	0.0
Tryptophan	W	0.0	0.0	0.0	0.0	0.0	0.0	0.0	0.0	0.0	0.0	0.0	0.0	0.0	0.0	99.0
Tyrosine	Y	0.0	0.0	0.0	0.0	0.0	0.0	0.0	0.0	0.0	0.0	0.0	0.0	0.0	0.0	0.0
Valine	V	0.0	0.5	0.0	0.0	0.0	0.0	0.0	0.0	0.0	0.0	0.0	0.0	0.0	0.0	0.0
GAP	-	0.0	0.0	0.0	0.0	0.0	0.0	0.0	0.0	0.0	0.0	0.0	0.0	0.0	0.0	0.0
UNKNOWN AMINO ACID	X	0.0	0.0	0.0	0.0	0.0	0.0	0.0	0.0	0.0	0.0	0.0	0.0	0.0	0.0	0.0
		100.0	100.0	100.0	100.0	100.0	100.0	100.0	100.0	100.0	100.0	100.0	100.0	100.0	100.0	100.0
		F	L/V	D	E	P	T/S	S	G	L	D	S/F	Q	S/A/E	A/S	W/L

upstream sequence

Alanine	A	0	0	0	0	0	0
Arginine	R	0.5	0	0	0	0	0
Asparagine	N	0	0	1.5	0	0	0
Aspartate	D	0	0	73.1	0	0	0
Cysteine	C	0	0	0	0	0	0
Glutamine	Q	0	0	0	0	0	0
Glutamate	E	0	0	0	0	0	0
Glycine	G	0	0	0	0	0	0
Histidine	H	0	0	0	0	0	0
Isoleucine	I	0	0	0	0.5	0	0
Leucine	L	0	0	0	99.5	100	100
Lysine	K	99.5	0	0	0	0	0
Methionine	M	0	0	0	0	0	0
Phenylalanine	F	0	0	0	0	0	0
Proline	P	0	100	0	0	0	0
Serine	S	0	0	23.9	0	0	0
Threonine	T	0	0	1.5	0	0	0
Tryptophan	W	0	0	0	0	0	0
Tyrosine	Y	0	0	0	0	0	0
Valine	V	0	0	0	0	0	0
GAP	-	0	0	0	0	0	0
UNKNOWN AMINO ACID	X	0	0	0	0	0	0
		100	100	100	100	100	100
		K/R	P	X	L/I	L	L

Motif 11 (LogOddsLogo)

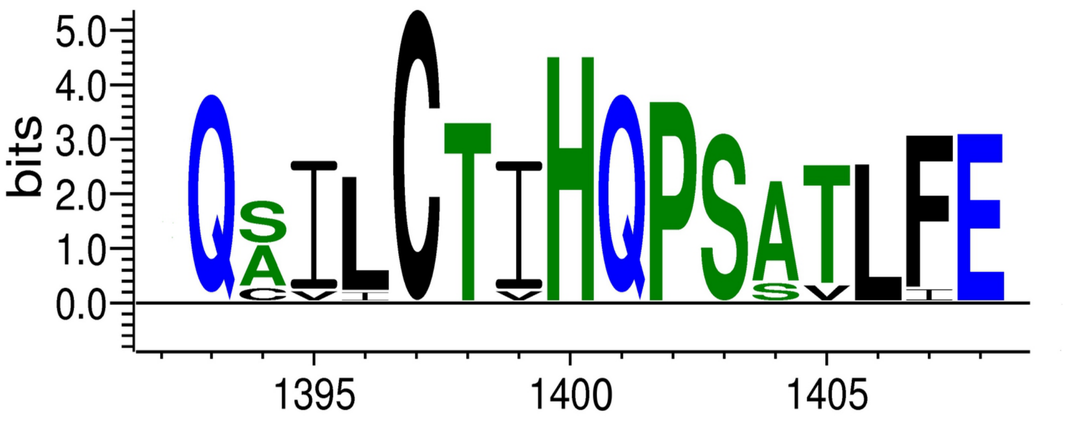

Motif 11 (frequency analysis)

Alanine	A	0.0	12.2	0.0	0.0	0.0	0.0	0.0	0.0	0.0	0.0	0.0	98.5	0.0	0.0	0.0	0.0
Arginine	R	0.0	0.0	0.0	0.0	0.0	0.0	0.0	0.0	0.0	0.0	0.0	0.0	0.0	0.0	0.0	0.0
Asparagine	N	0.0	0.0	0.0	0.0	0.0	0.0	0.0	0.0	0.0	0.0	0.0	0.0	0.0	0.0	0.0	0.0
Aspartate	D	0.0	0.0	0.0	0.0	0.0	0.0	0.0	0.0	0.0	0.0	0.0	0.0	0.0	0.0	0.0	0.0
Cysteine	C	0.0	5.1	0.0	0.0	100.0	0.0	0.0	0.0	0.0	0.0	0.0	0.0	0.0	0.0	0.0	0.0
Glutamine	Q	100.0	0.0	0.0	0.0	0.0	0.0	0.0	0.0	100.0	0.0	0.0	0.0	0.0	0.0	0.0	0.0
Glutamate	E	0.0	0.0	0.0	0.0	0.0	0.0	0.0	0.0	0.0	0.0	0.0	0.0	0.0	0.0	0.0	100.0
Glycine	G	0.0	0.0	0.0	0.0	0.0	0.0	0.0	0.0	0.0	0.0	0.0	0.0	0.0	0.0	0.0	0.0
Histidine	H	0.0	0.0	0.0	0.0	0.0	0.0	0.0	100.0	0.0	0.0	0.0	0.0	0.0	0.0	0.0	0.0
Isoleucine	I	0.0	0.0	99.5	0.5	0.0	0.0	99.5	0.0	0.0	0.0	0.0	0.0	0.0	0.0	0.5	0.0
Leucine	L	0.0	0.0	0.0	99.5	0.0	0.0	0.0	0.0	0.0	0.0	0.0	0.0	0.0	100.0	0.0	0.0
Lysine	K	0.0	0.0	0.0	0.0	0.0	0.0	0.0	0.0	0.0	0.0	0.0	0.0	0.0	0.0	0.0	0.0
Methionine	M	0.0	0.0	0.0	0.0	0.0	0.0	0.0	0.0	0.0	0.0	0.0	0.0	0.0	0.0	0.0	0.0
Phenylalanine	F	0.0	0.0	0.0	0.0	0.0	0.0	0.0	0.0	0.0	0.0	0.0	0.0	0.0	0.0	99.5	0.0
Proline	P	0.0	0.0	0.0	0.0	0.0	0.0	0.0	0.0	0.0	100.0	0.0	0.0	0.0	0.0	0.0	0.0
Serine	S	0.0	82.7	0.0	0.0	0.0	0.0	0.0	0.0	0.0	0.0	100.0	1.5	0.0	0.0	0.0	0.0
Threonine	T	0.0	0.0	0.0	0.0	0.0	100.0	0.0	0.0	0.0	0.0	0.0	0.0	99.0	0.0	0.0	0.0
Tryptophan	W	0.0	0.0	0.0	0.0	0.0	0.0	0.0	0.0	0.0	0.0	0.0	0.0	0.0	0.0	0.0	0.0
Tyrosine	Y	0.0	0.0	0.0	0.0	0.0	0.0	0.0	0.0	0.0	0.0	0.0	0.0	0.0	0.0	0.0	0.0
Valine	V	0.0	0.0	0.5	0.0	0.0	0.0	0.5	0.0	0.0	0.0	0.0	0.0	1.0	0.0	0.0	0.0
GAP	-	0.0	0.0	0.0	0.0	0.0	0.0	0.0	0.0	0.0	0.0	0.0	0.0	0.0	0.0	0.0	0.0
UNKNOWN AMINO ACID	X	0.0	0.0	0.0	0.0	0.0	0.0	0.0	0.0	0.0	0.0	0.0	0.0	0.0	0.0	0.0	0.0
		100.0	100.0	100.0	100.0	100.0	100.0	100.0	100.0	100.0	100.0	100.0	100.0	100.0	100.0	100.0	100.0
		Q	S/A/C	I/V	L/I	C	T	I/V	H	Q	P	S	A/S	T/V	L	F/I	E

upstream sequence

Alanine	A	0.5	0.0	70.1	3.0	91.4	0.0
Arginine	R	41.1	0.0	0.0	5.6	0.0	0.0
Asparagine	N	0.5	0.0	0.0	14.7	0.0	0.0
Aspartate	D	10.2	0.0	0.0	2.0	0.0	0.0
Cysteine	C	0.0	0.0	0.0	0.0	0.0	0.0
Glutamine	Q	2.0	0.0	0.0	18.3	0.0	0.0
Glutamate	E	3.0	0.0	0.0	4.1	0.0	0.0
Glycine	G	0.0	0.0	0.0	0.5	0.0	100.0
Histidine	H	1.0	0.0	0.0	3.0	0.0	0.0
Isoleucine	I	0.0	1.0	0.0	0.0	0.0	0.0
Leucine	L	0.0	99.0	0.0	17.8	0.0	0.0
Lysine	K	37.6	0.0	0.0	29.9	0.0	0.0
Methionine	M	0.0	0.0	0.0	0.5	0.0	0.0
Phenylalanine	F	0.0	0.0	0.0	0.0	0.0	0.0
Proline	P	0.0	0.0	0.0	0.0	0.0	0.0
Serine	S	3.0	0.0	28.4	0.0	8.6	0.0
Threonine	T	1.0	0.0	1.5	0.0	0.0	0.0
Tryptophan	W	0.0	0.0	0.0	0.0	0.0	0.0
Tyrosine	Y	0.0	0.0	0.0	0.5	0.0	0.0
Valine	V	0.0	0.0	0.0	0.0	0.0	0.0
GAP	-	0.0	0.0	0.0	0.0	0.0	0.0
UNKNOWN AMINO ACID	X	0.0	0.0	0.0	0.0	0.0	0.0
		100.0	100.0	100.0	100.0	100.0	100.0
		X	L/I	A/S/T	X	A/S	G

Motif 12 (LogOddsLogo)

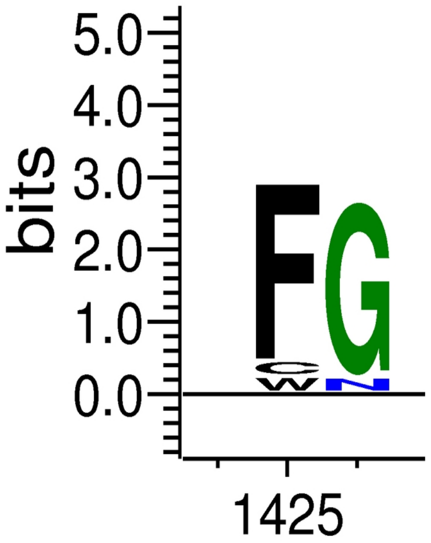

Motif 12 (frequency analysis)

Alanine	A	0.0	0.0
Arginine	R	0.0	0.0
Asparagine	N	0.0	0.5
Aspartate	D	0.0	0.0
Cysteine	C	0.5	0.0
Glutamine	Q	0.0	0.0
Glutamate	E	0.0	0.0
Glycine	G	0.0	99.5
Histidine	H	0.0	0.0
Isoleucine	I	0.0	0.0
Leucine	L	0.0	0.0
Lysine	K	0.0	0.0
Methionine	M	0.0	0.0
Phenylalanine	F	99.0	0.0
Proline	P	0.0	0.0
Serine	S	0.0	0.0
Threonine	T	0.0	0.0
Tryptophan	W	0.5	0.0
Tyrosine	Y	0.0	0.0
Valine	V	0.0	0.0
GAP	-	0.0	0.0
UNKNOWN AMINO ACID	X	0.0	0.0
		100.0	100.0
		F/C/W	G/N

upstream sequence

Alanine	A	0.0	0.0	0.0	0.0	0.0	0.0
Arginine	R	0.0	0.0	0.5	0.0	0.0	0.0
Asparagine	N	0.0	0.0	0.0	0.0	0.0	0.0
Aspartate	D	0.0	0.0	0.0	0.0	0.0	0.0
Cysteine	C	0.0	0.0	0.0	0.0	0.0	0.0
Glutamine	Q	0.0	0.0	90.4	0.0	0.0	0.0
Glutamate	E	0.0	0.0	0.5	0.0	0.0	0.0
Glycine	G	100.0	100.0	0.0	0.0	0.0	0.0
Histidine	H	0.0	0.0	0.0	0.0	0.0	0.0
Isoleucine	I	0.0	0.0	7.1	0.0	16.8	0.0
Leucine	L	0.0	0.0	0.0	0.0	0.0	0.0
Lysine	K	0.0	0.0	1.0	0.0	0.0	0.0
Methionine	M	0.0	0.0	0.0	0.0	0.0	0.0
Phenylalanine	F	0.0	0.0	0.0	0.0	0.0	0.0
Proline	P	0.0	0.0	0.0	0.0	0.0	0.0
Serine	S	0.0	0.0	0.5	0.0	0.0	0.0
Threonine	T	0.0	0.0	0.0	92.4	7.1	0.0
Tryptophan	W	0.0	0.0	0.0	0.0	0.0	0.0
Tyrosine	Y	0.0	0.0	0.0	0.0	0.0	100.0
Valine	V	0.0	0.0	0.0	7.6	76.1	0.0
GAP	-	0.0	0.0	0.0	0.0	0.0	0.0
UNKNOWN AMINO ACID	X	0.0	0.0	0.0	0.0	0.0	0.0
		100.0	100.0	100.0	100.0	100.0	100.0
		G	G	X	T/V	V/I/T	Y

Motif 13 (LogOddsLogo)

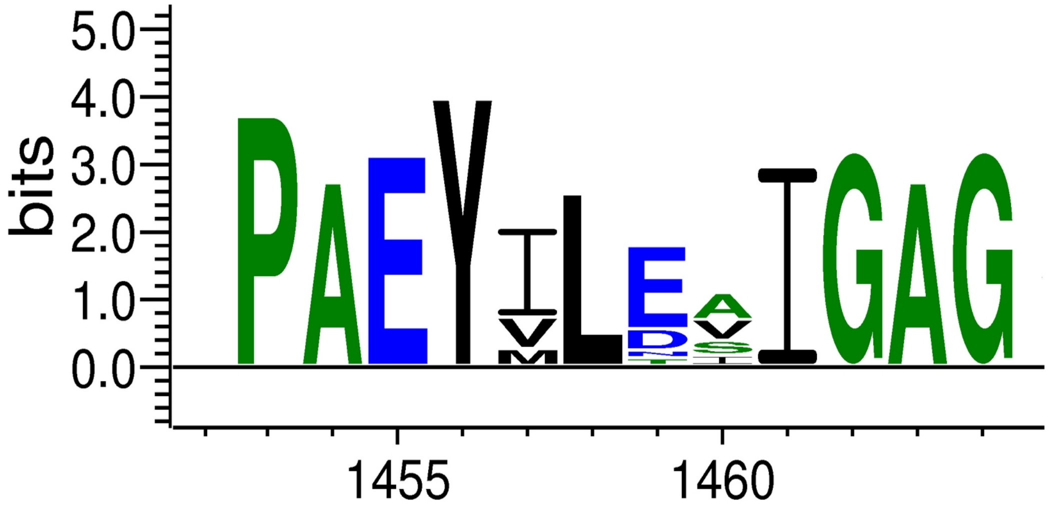

Motif 13 (frequency analysis)

Alanine	A	0.0	100.0	0.0	0.0	0.0	0.0	0.0	83.2	0.0	0.0	100.0	0.0
Arginine	R	0.0	0.0	0.0	0.0	0.0	0.0	0.0	0.0	0.0	0.0	0.0	0.0
Asparagine	N	0.0	0.0	0.0	0.0	0.0	0.0	0.5	0.0	0.0	0.0	0.0	0.0
Aspartate	D	0.0	0.0	0.0	0.0	0.0	0.0	1.5	0.0	0.0	0.0	0.0	0.0
Cysteine	C	0.0	0.0	0.0	0.0	0.0	0.0	0.0	0.0	0.0	0.0	0.0	0.0
Glutamine	Q	0.0	0.0	0.0	0.0	0.0	0.0	0.0	0.0	0.0	0.0	0.0	0.0
Glutamate	E	0.0	0.0	100.0	0.0	0.0	0.0	97.5	0.0	0.0	0.0	0.0	0.0
Glycine	G	0.0	0.0	0.0	0.0	0.0	0.0	0.0	0.0	0.0	100.0	0.0	100.0
Histidine	H	0.0	0.0	0.0	0.0	0.0	0.0	0.0	0.0	0.0	0.0	0.0	0.0
Isoleucine	I	0.0	0.0	0.0	0.0	96.4	0.0	0.0	2.0	100.0	0.0	0.0	0.0
Leucine	L	0.0	0.0	0.0	0.0	0.0	100.0	0.0	0.0	0.0	0.0	0.0	0.0
Lysine	K	0.0	0.0	0.0	0.0	0.0	0.0	0.0	0.0	0.0	0.0	0.0	0.0
Methionine	M	0.0	0.0	0.0	0.0	1.0	0.0	0.0	0.0	0.0	0.0	0.0	0.0
Phenylalanine	F	0.0	0.0	0.0	0.0	0.0	0.0	0.0	0.0	0.0	0.0	0.0	0.0
Proline	P	100.0	0.0	0.0	0.0	0.0	0.0	0.0	0.0	0.0	0.0	0.0	0.0
Serine	S	0.0	0.0	0.0	0.0	0.0	0.0	0.0	5.1	0.0	0.0	0.0	0.0
Threonine	T	0.0	0.0	0.0	0.0	0.0	0.0	0.5	0.0	0.0	0.0	0.0	0.0
Tryptophan	W	0.0	0.0	0.0	0.0	0.0	0.0	0.0	0.0	0.0	0.0	0.0	0.0
Tyrosine	Y	0.0	0.0	0.0	100.0	0.0	0.0	0.0	0.0	0.0	0.0	0.0	0.0
Valine	V	0.0	0.0	0.0	0.0	2.5	0.0	0.0	9.6	0.0	0.0	0.0	0.0
GAP	-	0.0	0.0	0.0	0.0	0.0	0.0	0.0	0.0	0.0	0.0	0.0	0.0
UNKNOWN AMINO ACID	X	0.0	0.0	0.0	0.0	0.0	0.0	0.0	0.0	0.0	0.0	0.0	0.0
		100.0	100.0	100.0	100.0	100.0	100.0	100.0	100.0	100.0	100.0	100.0	100.0
		P	A	E	Y	I/V/M	L	X	X	I	G	A	G

Upstream sequence

Alanine	A	0.0	0.5	1.5	3.6	0.0	0.0
Arginine	R	0.0	0.0	2.0	1.0	0.0	0.0
Asparagine	N	0.0	0.0	1.5	29.9	0.0	100.0
Aspartate	D	0.0	37.6	12.7	3.0	0.5	0.0
Cysteine	C	100.0	0.0	0.0	0.0	0.0	0.0
Glutamine	Q	0.0	27.4	32.5	0.5	0.0	0.0
Glutamate	E	0.0	15.2	6.1	0.5	99.5	0.0
Glycine	G	0.0	5.1	0.0	0.5	0.0	0.0
Histidine	H	0.0	0.5	2.5	4.1	0.0	0.0
Isoleucine	I	0.0	0.0	0.5	0.0	0.0	0.0
Leucine	L	0.0	1.5	0.0	1.0	0.0	0.0
Lysine	K	0.0	0.5	5.1	12.2	0.0	0.0
Methionine	M	0.0	0.0	0.0	0.0	0.0	0.0
Phenylalanine	F	0.0	0.0	0.5	0.0	0.0	0.0
Proline	P	0.0	0.0	6.6	0.0	0.0	0.0
Serine	S	0.0	9.1	27.9	40.6	0.0	0.0
Threonine	T	0.0	2.5	0.0	2.5	0.0	0.0
Tryptophan	W	0.0	0.0	0.0	0.0	0.0	0.0
Tyrosine	Y	0.0	0.0	0.0	0.5	0.0	0.0
Valine	V	0.0	0.0	0.5	0.0	0.0	0.0
GAP	-	0.0	0.0	0.0	0.0	0.0	0.0
UNKNOWN AMINO ACID	X	0.0	0.0	0.0	0.0	0.0	0.0
		100.0	100.0	100.0	100.0	100.0	100.0
		C	X	X	X	E/D	N

Motif 14 (LogOddsLogo)

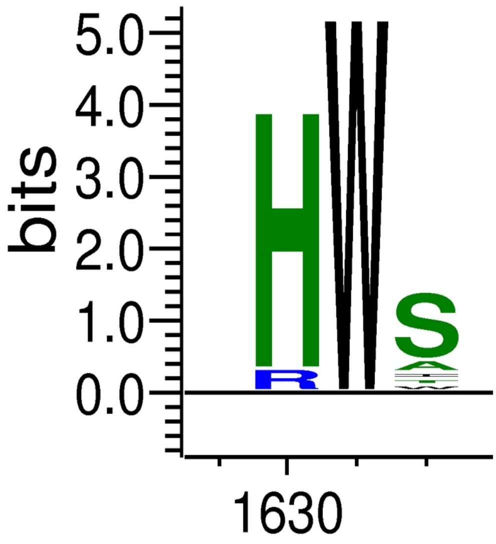

Motif 14 (frequency analysis)

Alanine	A	0.0	0.0	1.0
Arginine	R	0.5	0.0	0.0
Asparagine	N	0.0	0.0	0.0
Aspartate	D	0.0	0.0	0.0
Cysteine	C	0.0	0.0	0.0
Glutamine	Q	0.0	0.0	0.0
Glutamate	E	0.0	0.0	0.0
Glycine	G	0.0	0.0	0.0
Histidine	H	99.5	0.0	0.0
Isoleucine	I	0.0	0.0	0.5
Leucine	L	0.0	0.0	0.0
Lysine	K	0.0	0.0	0.0
Methionine	M	0.0	0.0	0.0
Phenylalanine	F	0.0	0.0	0.0
Proline	P	0.0	0.0	0.0
Serine	S	0.0	0.0	97.5
Threonine	T	0.0	0.0	0.5
Tryptophan	W	0.0	100.0	0.5
Tyrosine	Y	0.0	0.0	0.0
Valine	V	0.0	0.0	0.0
GAP	-	0.0	0.0	0.0
UNKNOWN AMINO ACID	X	0.0	0.0	0.0
		100.0	100.0	100.0
		H/R	W	X

Upstream sequence

Alanine	A	0.5	0.5	0.0	0.0	0.0	0.0
Arginine	R	1.0	1.0	0.0	0.0	0.0	0.0
Asparagine	N	0.0	0.0	0.0	97.5	0.0	0.0
Aspartate	D	0.0	0.0	0.0	0.0	0.0	0.0
Cysteine	C	0.0	0.0	0.0	0.0	0.0	0.0
Glutamine	Q	0.0	25.4	0.0	0.0	0.0	0.0
Glutamate	E	0.0	0.0	0.0	0.0	0.0	0.0
Glycine	G	0.0	0.0	0.0	0.0	0.0	0.0
Histidine	H	0.0	0.0	0.0	0.5	0.0	0.0
Isoleucine	I	0.0	0.0	0.0	0.0	0.0	0.0
Leucine	L	0.0	10.7	0.0	0.0	1.0	0.0
Lysine	K	10.2	59.9	0.0	1.0	0.0	0.0
Methionine	M	0.0	1.5	0.0	0.0	72.6	0.0
Phenylalanine	F	0.0	0.0	0.0	0.0	0.0	86.3
Proline	P	0.0	0.0	0.0	0.0	0.0	0.0
Serine	S	88.3	0.5	99.5	0.5	0.0	0.0
Threonine	T	0.0	0.0	0.0	0.0	25.9	0.0
Tryptophan	W	0.0	0.0	0.0	0.0	0.0	0.0
Tyrosine	Y	0.0	0.0	0.0	0.5	0.0	13.7
Valine	V	0.0	0.5	0.0	0.0	0.5	0.0
GAP	-	0.0	0.0	0.5	0.0	0.0	0.0
UNKNOWN AMINO ACID	X	0.0	0.0	0.0	0.0	0.0	0.0
		100.0	100.0	100.0	100.0	100.0	100.0
		X	X	S	X	X	F/Y

Motif 15 (LogOddsLogo)

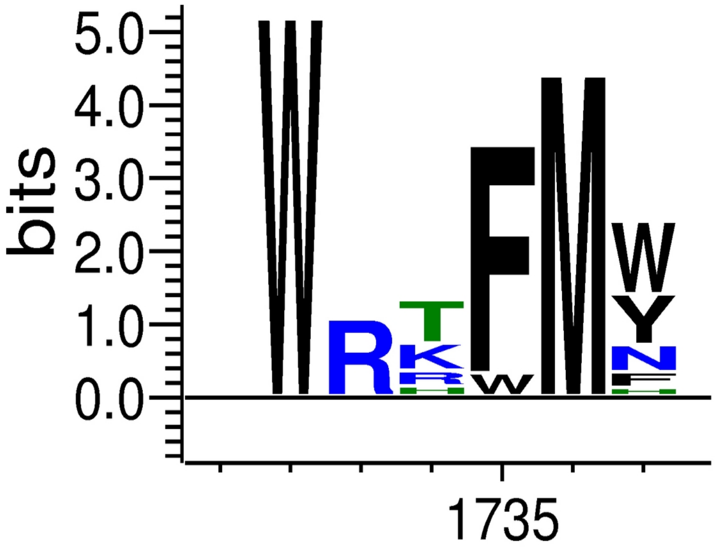

Motif 15 (frequency analysis)

Alanine	A	0.0	0.0	0.0	0.0	0.0	0.0
Arginine	R	0.0	1.0	2.0	0.0	0.0	0.0
Asparagine	N	0.0	0.0	0.0	0.0	0.0	9.1
Aspartate	D	0.0	0.0	0.0	0.0	0.0	0.0
Cysteine	C	0.0	0.0	0.0	0.0	0.0	0.0
Glutamine	Q	0.0	0.0	0.0	0.0	0.0	0.0
Glutamate	E	0.0	0.0	0.0	0.0	0.0	0.0
Glycine	G	0.0	0.0	0.0	0.0	0.0	0.0
Histidine	H	0.0	0.0	1.5	0.0	0.0	0.5
Isoleucine	I	0.0	0.0	0.0	0.0	0.0	0.0
Leucine	L	0.0	0.0	0.0	0.0	0.0	0.0
Lysine	K	0.0	0.0	6.6	0.0	0.0	0.0
Methionine	M	0.0	0.0	0.0	0.0	100.0	0.0
Phenylalanine	F	0.0	0.0	0.0	99.0	0.0	1.0
Proline	P	0.0	0.0	0.0	0.0	0.0	0.0
Serine	S	0.0	0.0	0.0	0.0	0.0	0.0
Threonine	T	0.0	0.0	89.8	0.0	0.0	0.0
Tryptophan	W	100.0	0.0	0.0	1.0	0.0	81.2
Tyrosine	Y	0.0	0.0	0.0	0.0	0.0	8.1
Valine	V	0.0	0.0	0.0	0.0	0.0	0.0
GAP	-	0.0	99.0	0.0	0.0	0.0	0.0
UNKNOWN AMINO ACID	X	0.0	0.0	0.0	0.0	0.0	0.0
		100.0	100.0	100.0	100.0	100.0	100.0
		W	-	T/K/H	F/W	M	X

Upstream sequence

Alanine	A	0.5	0.0	0.0	0.0	0.5	0.0
Arginine	R	2.0	0.0	0.0	0.0	0.0	0.0
Asparagine	N	10.2	1.0	0.0	0.0	0.0	0.0
Aspartate	D	3.0	0.0	0.0	0.0	0.0	0.0
Cysteine	C	0.0	0.0	0.0	0.0	0.0	0.0
Glutamine	Q	1.0	0.0	0.0	0.0	0.0	0.0
Glutamate	E	0.0	0.0	0.0	0.0	0.0	0.0
Glycine	G	0.0	0.0	0.0	0.0	96.4	0.0
Histidine	H	1.0	0.0	0.0	0.0	0.5	0.0
Isoleucine	I	0.0	0.0	0.5	0.0	0.0	0.0
Leucine	L	0.0	96.4	2.0	0.0	0.0	0.0
Lysine	K	1.5	0.0	0.0	0.0	0.5	0.0
Methionine	M	0.0	1.0	97.0	0.0	0.0	0.0
Phenylalanine	F	2.0	0.0	0.5	0.0	0.0	99.0
Proline	P	0.0	0.0	0.0	98.5	0.0	0.0
Serine	S	75.6	0.5	0.0	0.0	0.0	0.0
Threonine	T	1.0	0.0	0.0	0.0	1.5	0.0
Tryptophan	W	0.0	0.0	0.0	0.0	0.0	1.0
Tyrosine	Y	2.0	0.5	0.0	0.0	0.0	0.0
Valine	V	0.0	0.5	0.0	0.5	0.5	0.0
GAP	-	0.0	0.0	0.0	1.0	0.0	0.0
UNKNOWN AMINO ACID	X	0.0	0.0	0.0	0.0	0.0	0.0
		100.0	100.0	100.0	100.0	100.0	100.0
		X	X	X	P/V	X	F/W

Motif 16 (LogOddsLogo)

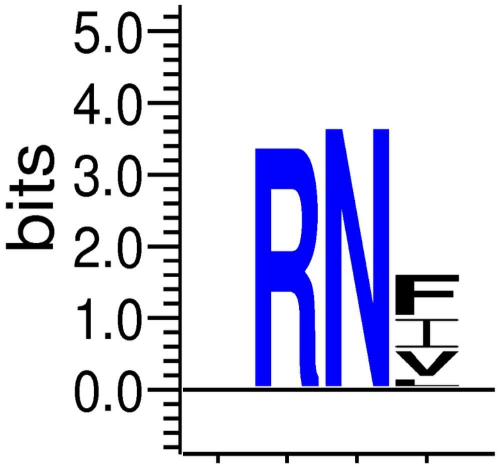

Motif 16 (frequency analysis)

Alanine	A	0.0	0.0	0.0
Arginine	R	100.0	0.0	0.0
Asparagine	N	0.0	100.0	0.0
Aspartate	D	0.0	0.0	0.0
Cysteine	C	0.0	0.0	0.0
Glutamine	Q	0.0	0.0	0.0
Glutamate	E	0.0	0.0	0.0
Glycine	G	0.0	0.0	0.0
Histidine	H	0.0	0.0	0.0
Isoleucine	I	0.0	0.0	13.2
Leucine	L	0.0	0.0	1.0
Lysine	K	0.0	0.0	0.0
Methionine	M	0.0	0.0	0.0
Phenylalanine	F	0.0	0.0	78.7
Proline	P	0.0	0.0	0.0
Serine	S	0.0	0.0	0.0
Threonine	T	0.0	0.0	0.0
Tryptophan	W	0.0	0.0	0.0
Tyrosine	Y	0.0	0.0	0.0
Valine	V	0.0	0.0	7.1
GAP	-	0.0	0.0	0.0
UNKNOWN AMINO ACID	X	0.0	0.0	0.0
		100.0	100.0	100.0
		R	N	X

Upstream sequence

Alanine	A	0.0	0.0	1.0	0.0	0.0	0.0
Arginine	R	0.0	0.0	0.0	0.0	18.3	0.0
Asparagine	N	1.5	0.0	1.5	11.7	0.0	0.0
Aspartate	D	0.5	0.0	0.0	1.5	0.0	0.0
Cysteine	C	0.0	0.0	0.0	0.0	0.0	0.0
Glutamine	Q	0.0	0.0	0.0	2.5	0.0	0.0
Glutamate	E	1.0	0.0	0.0	0.5	0.0	0.0
Glycine	G	0.0	0.0	9.6	0.0	1.0	0.0
Histidine	H	0.5	0.5	1.5	4.1	0.0	0.0
Isoleucine	I	0.0	0.0	0.0	0.0	4.1	0.0
Leucine	L	0.0	0.0	0.0	1.0	73.1	0.0
Lysine	K	46.2	0.0	0.0	0.0	0.0	0.0
Methionine	M	0.0	0.0	0.0	0.0	0.5	0.0
Phenylalanine	F	0.0	41.1	0.0	0.0	0.0	0.0
Proline	P	0.5	0.0	0.0	0.0	0.0	0.0
Serine	S	47.7	0.0	85.8	0.0	0.0	0.0
Threonine	T	1.5	0.0	0.0	0.5	0.0	0.0
Tryptophan	W	0.0	1.0	0.0	0.0	0.0	100.0
Tyrosine	Y	0.5	57.4	0.0	78.2	1.0	0.0
Valine	V	0.0	0.0	0.5	0.0	2.0	0.0
GAP	-	0.0	0.0	0.0	0.0	0.0	0.0
UNKNOWN AMINO ACID	X	0.0	0.0	0.0	0.0	0.0	0.0
		100.0	100.0	100.0	100.0	100.0	100.0
		X	X	X	X	X	W

Motif A (LogOddsLogo)

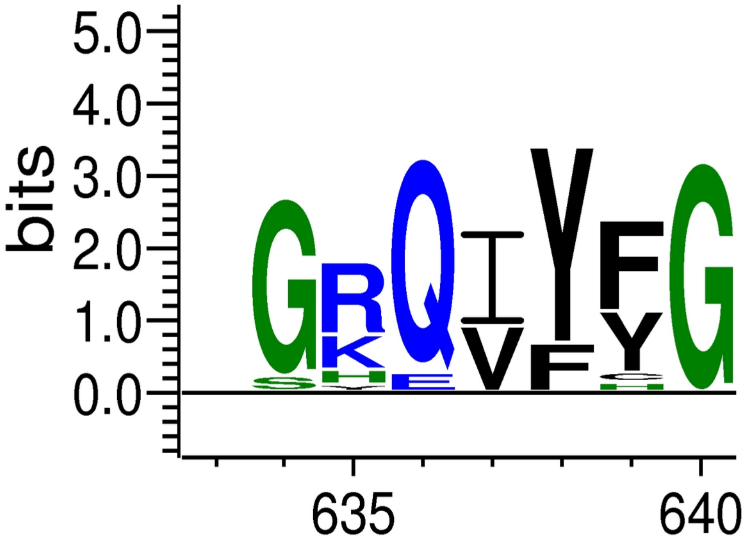

Motif A (frequency analysis)

Alanine	A	0.0	0.0	0.0	0.0	0.0	0.0	0.0
Arginine	R	0.0	61.9	0.0	0.0	0.0	0.0	0.0
Asparagine	N	0.0	0.0	0.0	0.0	0.0	0.0	0.0
Aspartate	D	0.0	0.0	0.0	0.0	0.0	0.0	0.0
Cysteine	C	0.0	0.0	0.0	0.0	0.0	16.2	0.0
Glutamine	Q	0.0	0.0	99.5	0.0	0.0	0.0	0.0
Glutamate	E	0.0	0.0	0.5	0.0	0.0	0.0	0.0
Glycine	G	99.5	0.0	0.0	0.0	0.0	0.0	100.0
Histidine	H	0.0	7.1	0.0	0.0	0.0	0.5	0.0
Isoleucine	I	0.0	0.0	0.0	84.8	0.0	0.0	0.0
Leucine	L	0.0	0.0	0.0	0.0	0.0	0.0	0.0
Lysine	K	0.0	30.5	0.0	0.0	0.0	0.0	0.0
Methionine	M	0.0	0.0	0.0	0.0	0.0	0.0	0.0
Phenylalanine	F	0.0	0.0	0.0	0.0	31.0	48.7	0.0
Proline	P	0.0	0.0	0.0	0.0	0.0	0.0	0.0
Serine	S	0.5	0.0	0.0	0.0	0.0	0.0	0.0
Threonine	T	0.0	0.0	0.0	0.0	0.0	0.0	0.0
Tryptophan	W	0.0	0.0	0.0	0.0	0.0	0.0	0.0
Tyrosine	Y	0.0	0.5	0.0	0.0	69.0	34.5	0.0
Valine	V	0.0	0.0	0.0	15.2	0.0	0.0	0.0
GAP	-	0.0	0.0	0.0	0.0	0.0	0.0	0.0
UNKNOWN AMINO ACID	X	0.0	0.0	0.0	0.0	0.0	0.0	0.0
		100.0	100.0	100.0	100.0	100.0	100.0	100.0

Motif B (LogOddsLogo)

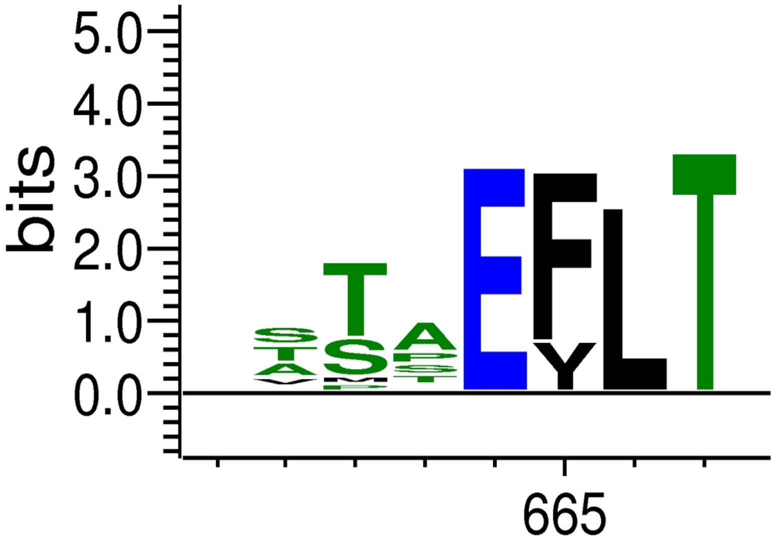

Motif B (frequency analysis)

Alanine	A	39.1	0.0	91.4	0.0	0.0	0.0	0.0
Arginine	R	0.0	0.0	0.0	0.0	0.0	0.0	0.0
Asparagine	N	0.0	0.0	0.0	0.0	0.0	0.0	0.0
Aspartate	D	0.5	0.0	0.0	0.0	0.0	0.0	0.0
Cysteine	C	0.0	0.0	0.5	0.0	0.0	0.0	0.0
Glutamine	Q	0.0	0.0	0.0	0.0	0.0	0.0	0.0
Glutamate	E	0.0	0.0	0.0	100.0	0.0	0.0	0.0
Glycine	G	0.0	0.0	1.0	0.0	0.0	0.0	0.0
Histidine	H	0.0	0.0	0.0	0.0	0.0	0.0	0.0
Isoleucine	I	0.0	0.0	0.0	0.0	0.0	0.0	0.0
Leucine	L	0.0	0.0	0.0	0.0	0.0	100.0	0.0
Lysine	K	0.0	0.0	0.0	0.0	0.0	0.0	0.0
Methionine	M	0.0	0.5	0.0	0.0	0.0	0.0	0.0
Phenylalanine	F	0.0	0.0	0.0	0.0	68.0	0.0	0.0
Proline	P	0.5	0.5	2.5	0.0	0.0	0.0	0.0
Serine	S	45.2	6.1	2.5	0.0	0.0	0.0	0.0
Threonine	T	10.2	92.9	2.0	0.0	0.0	0.0	100.0
Tryptophan	W	0.0	0.0	0.0	0.0	0.0	0.0	0.0
Tyrosine	Y	0.0	0.0	0.0	0.0	31.5	0.0	0.0
Valine	V	4.6	0.0	0.0	0.0	0.0	0.0	0.0
GAP	-	0.0	0.0	0.0	0.0	0.0	0.0	0.0
UNKNOWN AMINO ACID	X	0.0	0.0	0.0	0.0	0.5	0.0	0.0
		100.0	100.0	100.0	100.0	100.0	100.0	100.0

Motif C (LogOddsLogo)

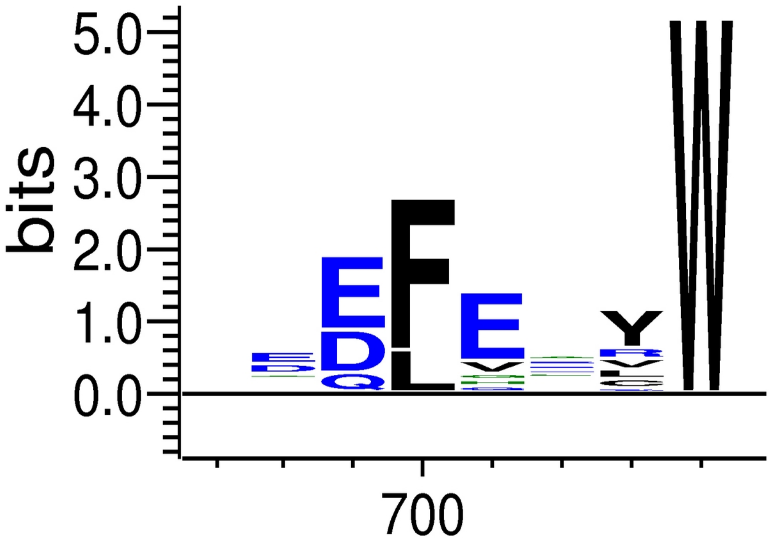

Motif C (frequency analysis)

Alanine	A	2.5	0.0	0.0	0.0	8.6	0.0	0.0
Arginine	R	0.0	0.0	0.0	0.0	5.6	3.0	0.0
Asparagine	N	0.0	0.0	0.0	0.0	2.5	0.0	0.0
Aspartate	D	41.6	14.2	0.0	0.0	3.6	0.0	0.0
Cysteine	C	0.0	0.0	0.0	0.0	0.0	2.0	0.0
Glutamine	Q	6.1	1.5	0.0	0.5	12.7	0.5	0.0
Glutamate	E	45.2	84.3	0.0	96.4	5.6	0.0	0.0
Glycine	G	1.0	0.0	0.0	0.5	0.0	0.0	0.0
Histidine	H	0.0	0.0	0.0	0.5	6.6	0.0	0.0
Isoleucine	I	0.5	0.0	0.0	0.0	0.0	0.0	0.0
Leucine	L	0.5	0.0	6.6	0.0	0.0	1.5	0.0
Lysine	K	2.0	0.0	0.0	0.0	23.9	0.0	0.0
Methionine	M	0.0	0.0	0.0	0.0	0.0	0.0	0.0
Phenylalanine	F	0.0	0.0	93.4	0.0	0.0	0.0	0.0
Proline	P	0.0	0.0	0.0	0.0	0.0	0.0	0.0
Serine	S	0.0	0.0	0.0	0.0	3.6	0.0	0.0
Threonine	T	0.0	0.0	0.0	0.0	27.4	0.0	0.0
Tryptophan	W	0.0	0.0	0.0	0.0	0.0	0.0	100.0
Tyrosine	Y	0.0	0.0	0.0	0.0	0.0	90.9	0.0
Valine	V	0.5	0.0	0.0	2.0	0.0	2.0	0.0
GAP	-	0.0	0.0	0.0	0.0	0.0	0.0	0.0
UNKNOWN AMINO ACID	X	0.0	0.0	0.0	0.0	0.0	0.0	0.0
		100.0	100.0	100.0	100.0	100.0	100.0	100.0

Motif D (LogOddsLogo)

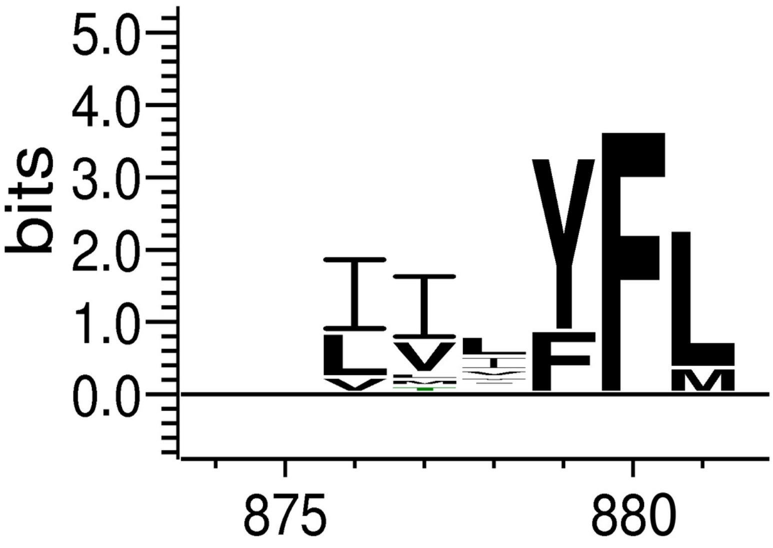

Motif D (frequency analysis)

Alanine	A	1.5	0.0	0.0	0.0	0.0	0.0	0.0
Arginine	R	0.0	0.0	0.0	0.0	0.0	0.0	0.0
Asparagine	N	0.0	0.0	0.0	0.0	0.0	0.0	0.0
Aspartate	D	0.0	0.0	0.0	0.0	0.0	0.0	0.0
Cysteine	C	0.0	0.0	0.0	0.0	0.0	0.0	0.0
Glutamine	Q	0.0	0.0	0.0	0.0	0.0	0.0	0.0
Glutamate	E	0.0	0.0	0.0	0.0	0.0	0.0	0.0
Glycine	G	1.0	0.0	0.0	0.0	0.0	0.0	0.0
Histidine	H	0.0	0.0	0.0	0.0	0.0	0.0	0.0
Isoleucine	I	32.5	91.9	93.4	7.1	0.0	0.0	0.0
Leucine	L	14.7	7.1	0.5	85.8	0.0	0.0	99.0
Lysine	K	0.0	0.0	0.0	0.0	0.0	0.0	0.0
Methionine	M	1.5	0.0	0.5	1.0	0.0	0.0	1.0
Phenylalanine	F	27.9	0.0	0.0	0.0	33.0	100.0	0.0
Proline	P	0.0	0.0	0.0	0.0	0.0	0.0	0.0
Serine	S	6.6	0.0	0.0	1.0	0.0	0.0	0.0
Threonine	T	1.5	0.0	0.5	1.0	0.0	0.0	0.0
Tryptophan	W	0.0	0.0	0.0	0.0	0.0	0.0	0.0
Tyrosine	Y	2.0	0.0	0.0	1.5	67.0	0.0	0.0
Valine	V	10.7	1.0	5.1	2.5	0.0	0.0	0.0
GAP	-	0.0	0.0	0.0	0.0	0.0	0.0	0.0
UNKNOWN AMINO ACID	X	0.0	0.0	0.0	0.0	0.0	0.0	0.0
		100.0	100.0	100.0	100.0	100.0	100.0	100.0

Motif E (LogOddsLogo)

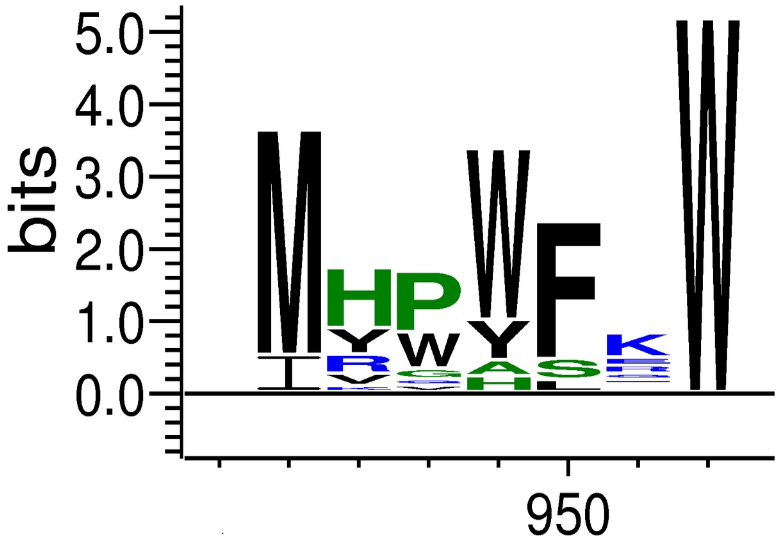

Motif E (frequency analysis)

Alanine	A	0.0	0.0	0.0	0.5	0.0	1.0	0.0
Arginine	R	0.0	2.5	0.0	0.0	0.0	4.1	0.0
Asparagine	N	0.0	0.0	0.0	0.0	0.0	0.0	0.0
Aspartate	D	0.0	0.0	0.0	0.0	0.0	0.0	0.0
Cysteine	C	0.0	0.0	0.0	0.0	0.0	0.0	0.0
Glutamine	Q	0.0	0.0	0.5	0.0	0.0	3.6	0.0
Glutamate	E	0.0	0.0	0.0	0.0	0.0	2.5	0.0
Glycine	G	0.0	0.0	1.5	0.0	0.0	0.0	0.0
Histidine	H	0.0	90.9	0.0	0.5	0.0	0.0	0.0
Isoleucine	I	1.0	0.0	0.0	0.0	0.0	1.0	0.0
Leucine	L	0.0	0.0	0.0	0.0	0.5	0.0	0.0
Lysine	K	0.0	0.5	0.0	0.0	0.0	86.3	0.0
Methionine	M	99.0	0.0	0.0	0.0	0.0	0.0	0.0
Phenylalanine	F	0.0	0.0	0.0	0.0	97.0	0.0	0.0
Proline	P	0.0	0.0	90.9	0.0	0.0	0.0	0.0
Serine	S	0.0	0.0	0.0	0.0	1.5	0.5	0.0
Threonine	T	0.0	0.0	0.0	0.0	0.0	0.0	0.0
Tryptophan	W	0.0	0.0	6.6	97.0	0.0	0.0	100.0
Tyrosine	Y	0.0	4.6	0.0	2.0	0.0	0.0	0.0
Valine	V	0.0	1.5	0.5	0.0	0.0	0.5	0.0
GAP	-	0.0	0.0	0.0	0.0	0.0	0.0	0.0
UNKNOWN AMINO ACID	X	0.0	0.0	0.0	0.0	1.0	0.5	0.0
		100.0	100.0	100.0	100.0	100.0	100.0	100.0

Motif F (LogOddsLogo)

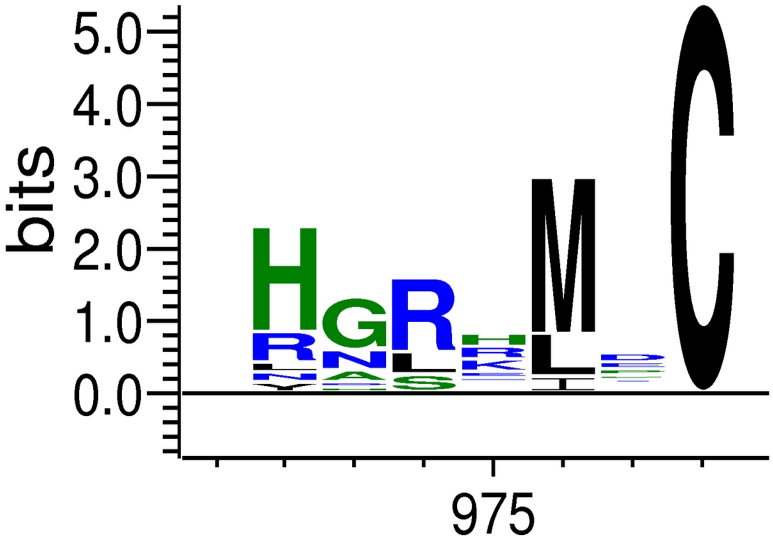

Motif F (frequency analysis)

Alanine	A	0.0	2.0	0.0	0.0	0.0	2.0	0.0
Arginine	R	3.0	0.0	95.9	11.2	0.0	0.0	0.0
Asparagine	N	0.5	3.0	0.0	0.5	0.0	4.1	0.0
Aspartate	D	0.0	0.5	0.0	0.0	0.0	71.6	0.0
Cysteine	C	0.0	0.0	0.0	0.0	0.0	0.0	100.0
Glutamine	Q	0.0	0.0	0.0	1.0	0.0	5.6	0.0
Glutamate	E	0.0	0.0	0.0	2.0	0.0	7.6	0.0
Glycine	G	0.0	93.9	0.0	0.0	0.0	0.5	0.0
Histidine	H	95.4	0.5	0.0	69.0	0.0	0.0	0.0
Isoleucine	I	0.0	0.0	0.0	1.0	1.0	0.0	0.0
Leucine	L	0.5	0.0	2.0	0.5	2.0	0.5	0.0
Lysine	K	0.0	0.0	0.0	13.7	0.0	1.0	0.0
Methionine	M	0.0	0.0	0.0	0.0	97.0	0.0	0.0
Phenylalanine	F	0.0	0.0	0.0	0.0	0.0	0.0	0.0
Proline	P	0.0	0.0	0.0	0.0	0.0	4.6	0.0
Serine	S	0.0	0.0	2.0	0.0	0.0	2.5	0.0
Threonine	T	0.0	0.0	0.0	0.0	0.0	0.0	0.0
Tryptophan	W	0.0	0.0	0.0	0.0	0.0	0.0	0.0
Tyrosine	Y	0.5	0.0	0.0	0.5	0.0	0.0	0.0
Valine	V	0.0	0.0	0.0	0.5	0.0	0.0	0.0
GAP	-	0.0	0.0	0.0	0.0	0.0	0.0	0.0
UNKNOWN AMINO ACID	X	0.0	0.0	0.0	0.0	0.0	0.0	0.0
		100.0	100.0	100.0	100.0	100.0	100.0	100.0

Motif G (LogOddsLogo)

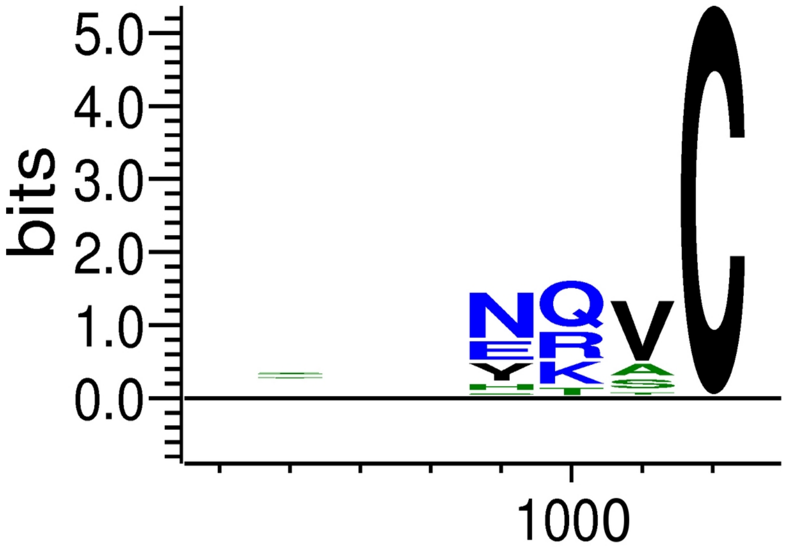

Motif G (frequency analysis)

Alanine	A	7.1	0.0	4.6	0.0	0.0	2.0	0.0
Arginine	R	0.0	1.0	0.0	0.0	12.7	0.0	0.0
Asparagine	N	5.6	0.0	2.0	56.9	0.0	0.0	0.0
Aspartate	D	29.9	0.0	33.5	0.0	0.0	0.0	0.0
Cysteine	C	0.0	0.0	0.0	0.0	0.0	0.0	100.0
Glutamine	Q	2.0	0.0	2.0	0.0	55.3	0.0	0.0
Glutamate	E	1.5	0.0	39.1	11.7	0.0	0.0	0.0
Glycine	G	0.0	0.0	11.2	0.0	0.0	0.0	0.0
Histidine	H	0.0	0.0	0.0	1.0	0.0	0.0	0.0
Isoleucine	I	1.0	0.0	0.5	0.0	0.0	0.0	0.0
Leucine	L	0.0	1.0	1.5	0.0	0.0	0.0	0.0
Lysine	K	0.5	0.5	0.5	0.0	31.0	0.0	0.0
Methionine	M	0.0	0.5	0.0	0.0	0.0	0.0	0.0
Phenylalanine	F	0.0	0.0	0.0	0.0	0.0	0.0	0.0
Proline	P	34.0	0.0	0.0	0.0	0.0	0.0	0.0
Serine	S	15.2	0.0	2.5	0.5	0.0	2.0	0.0
Threonine	T	3.0	0.0	0.5	0.0	1.0	0.5	0.0
Tryptophan	W	0.0	0.0	0.0	0.0	0.0	0.0	0.0
Tyrosine	Y	0.0	0.0	0.0	29.9	0.0	0.0	0.0
Valine	V	0.0	0.0	1.5	0.0	0.0	95.4	0.0
GAP	-	0.0	97.0	0.5	0.0	0.0	0.0	0.0
UNKNOWN AMINO ACID	X	0.0	0.0	0.0	0.0	0.0	0.0	0.0
		100.0	100.0	100.0	100.0	100.0	100.0	100.0

Motif H (LogOddsLogo)

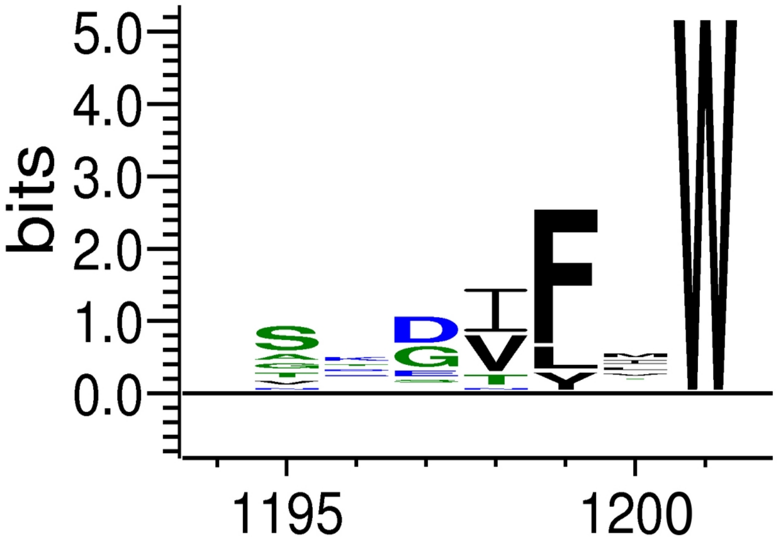

Motif H (frequency analysis)

Alanine	A	33.5	0.5	0.5	0.0	0.0	2.5	0.0
Arginine	R	0.0	2.5	0.0	0.0	0.0	0.5	0.0
Asparagine	N	1.5	1.5	0.5	0.5	0.0	0.0	0.0
Aspartate	D	0.0	5.1	16.2	0.0	0.0	0.0	0.0
Cysteine	C	0.0	0.0	0.0	0.0	0.0	1.0	0.0
Glutamine	Q	0.0	0.5	0.0	0.0	0.0	0.0	0.0
Glutamate	E	0.0	5.1	2.0	0.0	0.0	0.0	0.0
Glycine	G	2.5	0.5	79.2	0.0	0.0	0.0	0.0
Histidine	H	0.0	0.0	0.0	0.0	0.0	0.0	0.0
Isoleucine	I	0.0	0.0	0.0	19.3	0.0	66.5	0.0
Leucine	L	0.0	0.0	0.0	0.0	1.0	5.6	0.0
Lysine	K	0.0	45.2	0.0	0.0	0.0	0.0	0.0
Methionine	M	0.0	0.0	0.0	0.0	0.0	8.6	0.0
Phenylalanine	F	0.0	0.0	0.0	0.0	97.5	0.5	0.0
Proline	P	0.0	0.5	0.0	0.0	0.0	0.0	0.0
Serine	S	57.4	2.5	1.5	0.0	0.0	1.5	0.0
Threonine	T	2.0	36.0	0.0	3.0	0.0	1.5	0.0
Tryptophan	W	0.0	0.0	0.0	0.0	0.0	0.0	100.0
Tyrosine	Y	0.0	0.0	0.0	0.0	1.5	0.0	0.0
Valine	V	3.0	0.0	0.0	77.2	0.0	11.7	0.0
GAP	-	0.0	0.0	0.0	0.0	0.0	0.0	0.0
UNKNOWN AMINO ACID	X	0.0	0.0	0.0	0.0	0.0	0.0	0.0
		100.0	100.0	100.0	100.0	100.0	100.0	100.0

Motif I (LogOddsLogo)

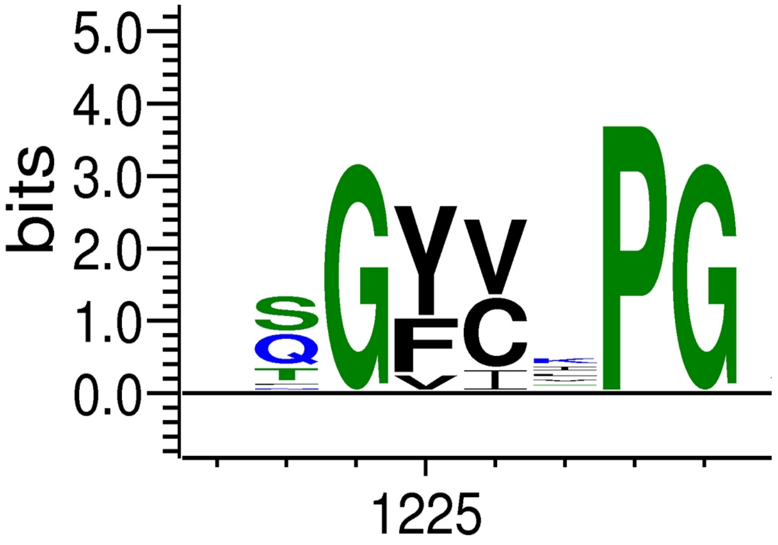

Motif I (frequency analysis)

Alanine	A	0.0	0.0	0.0	0.0	7.6	0.0	0.0
Arginine	R	0.0	0.0	0.0	0.0	1.5	0.0	0.0
Asparagine	N	0.5	0.0	0.0	0.0	0.0	0.0	0.0
Aspartate	D	0.0	0.0	0.0	0.0	0.0	0.0	0.0
Cysteine	C	0.0	0.0	0.0	79.2	0.0	0.0	0.0
Glutamine	Q	11.7	0.0	0.0	0.0	0.0	0.0	0.0
Glutamate	E	0.0	0.0	0.0	0.0	0.0	0.0	0.0
Glycine	G	0.0	100.0	0.0	0.0	0.0	0.0	100.0
Histidine	H	0.0	0.0	0.0	0.0	0.0	0.0	0.0
Isoleucine	I	0.0	0.0	0.0	1.5	35.0	0.0	0.0
Leucine	L	0.0	0.0	0.0	0.0	5.1	0.0	0.0
Lysine	K	0.0	0.0	0.0	0.0	11.2	0.0	0.0
Methionine	M	0.0	0.0	0.0	0.0	0.0	0.0	0.0
Phenylalanine	F	0.5	0.0	7.1	0.0	0.0	0.0	0.0
Proline	P	0.0	0.0	0.0	0.0	0.0	100.0	0.0
Serine	S	81.7	0.0	0.0	0.0	0.0	0.0	0.0
Threonine	T	5.6	0.0	0.0	0.0	0.0	0.0	0.0
Tryptophan	W	0.0	0.0	0.0	0.0	0.0	0.0	0.0
Tyrosine	Y	0.0	0.0	91.9	0.0	0.5	0.0	0.0
Valine	V	0.0	0.0	1.0	19.3	39.1	0.0	0.0
GAP	-	0.0	0.0	0.0	0.0	0.0	0.0	0.0
UNKNOWN AMINO ACID	X	0.0	0.0	0.0	0.0	0.0	0.0	0.0
		100.0	100.0	100.0	100.0	100.0	100.0	100.0

Motif J (LogOddsLogo)

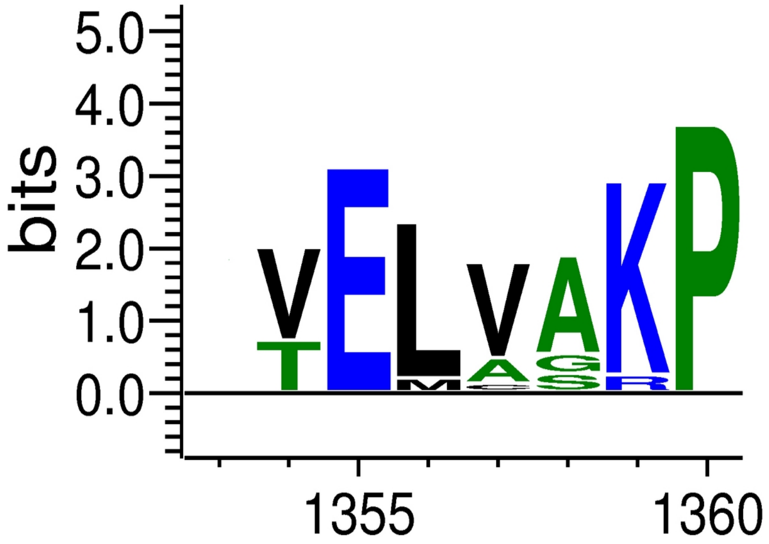

Motif J (frequency analysis)

Alanine	A	0.0	0.0	0.0	2.5	68.0	0.0	0.0
Arginine	R	0.0	0.0	0.0	0.0	0.0	0.5	0.0
Asparagine	N	0.0	0.0	0.0	0.0	0.0	0.0	0.0
Aspartate	D	0.0	0.0	0.0	0.0	0.0	0.0	0.0
Cysteine	C	0.0	0.0	0.0	0.5	0.0	0.0	0.0
Glutamine	Q	0.0	0.0	0.0	0.0	0.0	0.0	0.0
Glutamate	E	0.0	100.0	0.0	0.0	0.0	0.0	0.0
Glycine	G	0.0	0.0	0.0	0.0	30.5	0.0	0.0
Histidine	H	0.0	0.0	0.0	0.0	0.0	0.0	0.0
Isoleucine	I	0.0	0.0	0.0	0.0	0.0	0.0	0.0
Leucine	L	0.0	0.0	99.5	0.0	0.0	0.0	0.0
Lysine	K	0.0	0.0	0.0	0.0	0.0	99.5	0.0
Methionine	M	0.0	0.0	0.5	0.0	0.0	0.0	0.0
Phenylalanine	F	0.0	0.0	0.0	0.0	0.0	0.0	0.0
Proline	P	0.0	0.0	0.0	0.0	0.0	0.0	100.0
Serine	S	0.0	0.0	0.0	0.0	1.5	0.0	0.0
Threonine	T	7.1	0.0	0.0	0.0	0.0	0.0	0.0
Tryptophan	W	0.0	0.0	0.0	0.0	0.0	0.0	0.0
Tyrosine	Y	0.0	0.0	0.0	0.0	0.0	0.0	0.0
Valine	V	92.9	0.0	0.0	97.0	0.0	0.0	0.0
GAP	-	0.0	0.0	0.0	0.0	0.0	0.0	0.0
UNKNOWN AMINO ACID	X	0.0	0.0	0.0	0.0	0.0	0.0	0.0
		100.0	100.0	100.0	100.0	100.0	100.0	100.0

Motif K (LogOddsLogo)

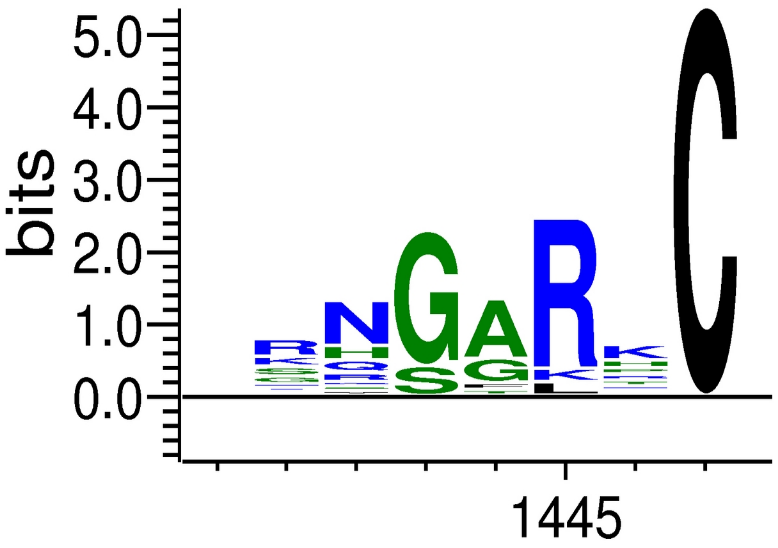

Motif K (frequency analysis)

Alanine	A	0.0	0.0	0.0	93.9	0.0	0.0	0.0
Arginine	R	52.8	1.0	0.0	0.0	99.0	2.0	0.0
Asparagine	N	1.0	93.4	0.0	0.0	0.0	0.0	0.0
Aspartate	D	0.5	0.0	0.0	0.0	0.0	0.0	0.0
Cysteine	C	0.0	0.0	0.0	0.0	0.0	0.0	100.0
Glutamine	Q	0.5	2.0	0.0	0.0	0.0	1.5	0.0
Glutamate	E	1.0	0.0	0.0	0.0	0.0	0.5	0.0
Glycine	G	5.1	0.0	98.5	3.6	0.0	0.0	0.0
Histidine	H	0.0	2.0	0.0	0.0	0.0	11.2	0.0
Isoleucine	I	0.0	0.0	0.0	0.0	0.0	0.5	0.0
Leucine	L	0.0	0.0	0.0	0.0	0.5	0.0	0.0
Lysine	K	32.5	0.5	0.0	0.0	0.5	76.6	0.0
Methionine	M	0.0	0.0	0.0	0.0	0.0	0.0	0.0
Phenylalanine	F	0.0	0.0	0.0	2.0	0.0	0.0	0.0
Proline	P	0.0	0.0	0.0	0.0	0.0	4.6	0.0
Serine	S	6.1	0.5	1.5	0.0	0.0	0.0	0.0
Threonine	T	0.0	0.0	0.0	0.5	0.0	2.5	0.0
Tryptophan	W	0.0	0.0	0.0	0.0	0.0	0.0	0.0
Tyrosine	Y	0.0	0.5	0.0	0.0	0.0	0.0	0.0
Valine	V	0.0	0.0	0.0	0.0	0.0	0.5	0.0
GAP	-	0.0	0.0	0.0	0.0	0.0	0.0	0.0
UNKNOWN AMINO ACID	X	0.5	0.0	0.0	0.0	0.0	0.0	0.0
		100.0	100.0	100.0	100.0	100.0	100.0	100.0

Motif L (LogOddsLogo)

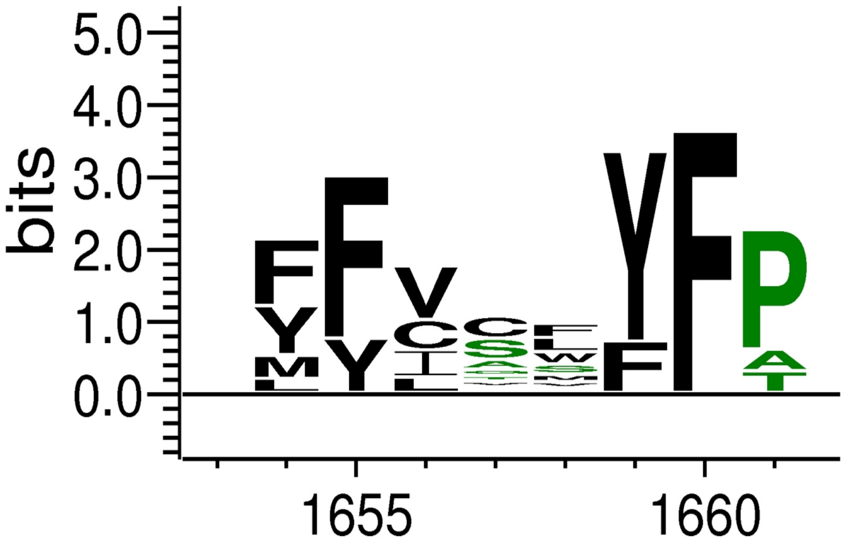

Motif L (frequency analysis)

Alanine	A	0.0	0.0	0.0	5.6	0.0	0.0	0.0	1.0
Arginine	R	0.0	0.0	0.0	0.0	0.0	0.0	0.0	0.0
Asparagine	N	0.0	0.0	0.0	0.0	0.0	0.0	0.0	0.0
Aspartate	D	0.0	0.0	0.0	0.0	0.0	0.0	0.0	0.0
Cysteine	C	0.0	0.0	7.6	17.3	0.5	0.0	0.0	0.0
Glutamine	Q	0.0	0.0	0.0	0.0	0.0	0.0	0.0	0.0
Glutamate	E	0.0	0.0	0.0	0.0	0.0	0.0	0.0	0.0
Glycine	G	0.0	0.0	0.0	2.5	0.0	0.0	0.0	0.0
Histidine	H	0.0	0.0	0.0	0.0	0.0	0.0	0.0	0.0
Isoleucine	I	0.0	0.0	5.1	0.5	0.0	0.0	0.0	0.0
Leucine	L	1.0	0.0	1.5	0.0	40.6	0.0	0.0	0.0
Lysine	K	0.0	0.0	0.0	0.0	0.0	0.0	0.0	0.0
Methionine	M	9.6	0.0	0.0	0.0	4.1	0.0	0.0	0.0
Phenylalanine	F	80.7	97.0	0.0	0.0	11.7	2.5	99.5	0.0
Proline	P	0.0	0.0	0.0	0.5	0.0	0.0	0.0	98.0
Serine	S	0.0	0.0	0.0	71.6	32.0	0.0	0.0	0.0
Threonine	T	0.0	0.0	0.0	1.0	0.0	0.0	0.0	1.0
Tryptophan	W	0.0	0.0	0.0	0.0	6.6	0.0	0.0	0.0
Tyrosine	Y	8.6	3.0	0.0	0.0	0.5	97.0	0.0	0.0
Valine	V	0.0	0.0	85.8	1.0	3.6	0.0	0.0	0.0
GAP	-	0.0	0.0	0.0	0.0	0.0	0.0	0.0	0.0
UNKNOWN AMINO ACID	X	0.0	0.0	0.0	0.0	0.5	0.5	0.5	0.0
		100.0	100.0	100.0	100.0	100.0	100.0	100.0	100.0

Motif M (LogOddsLogo)

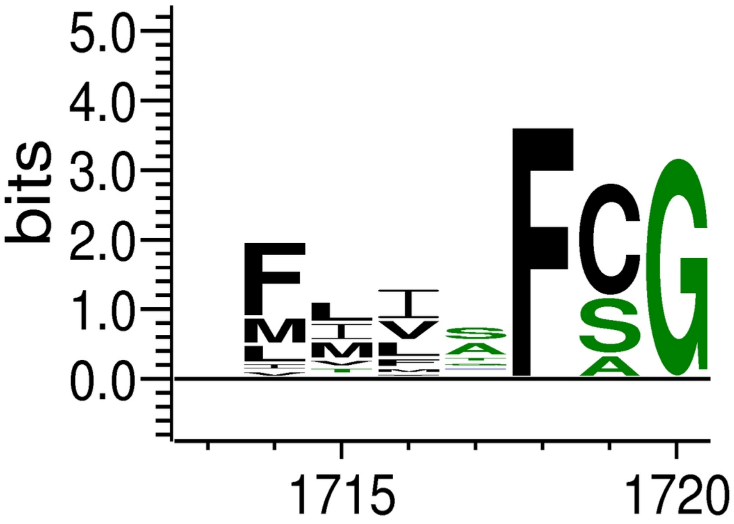

Motif M (frequency analysis)

Alanine	A	0.0	0.0	0.0	12.2	0.0	1.0	0.0
Arginine	R	0.0	0.0	0.0	0.0	0.0	0.0	0.0
Asparagine	N	0.0	0.0	0.0	0.5	0.0	0.0	0.0
Aspartate	D	0.0	0.0	0.0	0.0	0.0	0.0	0.0
Cysteine	C	0.0	0.0	0.5	0.5	0.0	84.8	0.0
Glutamine	Q	0.0	0.0	0.0	4.1	0.0	0.0	0.0
Glutamate	E	0.0	0.0	0.0	0.0	0.0	0.0	0.0
Glycine	G	0.0	0.0	0.0	1.5	0.0	0.0	100.0
Histidine	H	0.0	0.5	0.0	0.0	0.0	0.0	0.0
Isoleucine	I	1.5	14.7	24.9	0.0	0.0	0.0	0.0
Leucine	L	2.0	47.2	57.9	0.0	0.0	0.0	0.0
Lysine	K	0.0	0.0	0.0	0.0	0.0	0.0	0.0
Methionine	M	5.6	34.0	1.0	0.0	0.0	0.0	0.0
Phenylalanine	F	90.4	0.0	2.0	0.0	99.5	0.0	0.0
Proline	P	0.0	0.0	0.0	0.0	0.0	0.0	0.0
Serine	S	0.0	0.0	0.0	77.2	0.0	14.2	0.0
Threonine	T	0.0	1.5	0.0	2.0	0.0	0.0	0.0
Tryptophan	W	0.0	0.0	0.0	0.0	0.0	0.0	0.0
Tyrosine	Y	0.0	0.0	0.0	0.0	0.0	0.0	0.0
Valine	V	0.5	2.0	13.7	2.0	0.0	0.0	0.0
GAP	-	0.0	0.0	0.0	0.0	0.0	0.0	0.0
UNKNOWN AMINO ACID	X	0.0	0.0	0.0	0.0	0.5	0.0	0.0
		100.0	100.0	100.0	100.0	100.0	100.0	100.0

Motif N (LogOddsLogo)

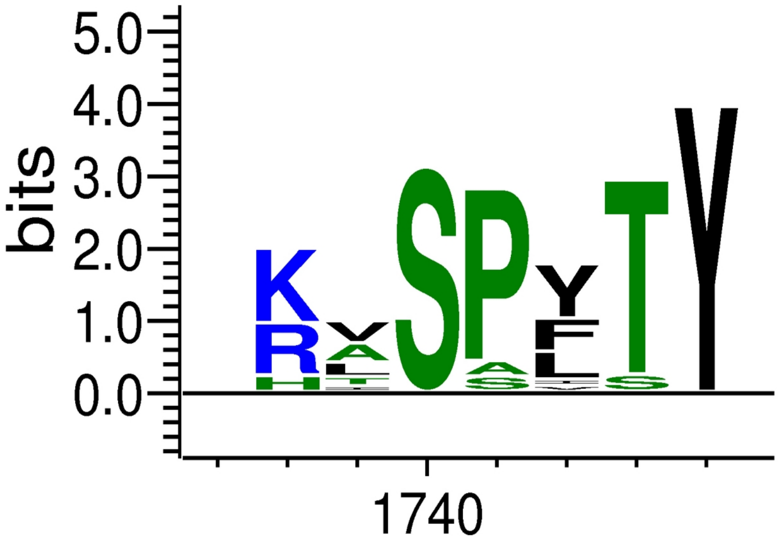

Motif N (frequency table)

Alanine	A	0.0	39.6	0.0	0.5	0.0	0.0	0.0
Arginine	R	8.6	0.0	0.0	0.0	0.0	0.0	0.0
Asparagine	N	0.0	0.0	0.0	0.0	0.0	0.0	0.0
Aspartate	D	0.0	0.0	0.0	0.0	0.0	0.0	0.0
Cysteine	C	0.0	0.0	0.0	0.0	0.0	0.0	0.0
Glutamine	Q	0.0	0.0	0.0	0.0	0.0	0.0	0.0
Glutamate	E	0.0	0.0	0.0	0.0	0.0	0.0	0.0
Glycine	G	0.0	0.0	0.0	0.0	0.0	0.0	0.0
Histidine	H	1.5	0.0	0.0	0.0	0.0	0.0	0.0
Isoleucine	I	0.0	1.0	0.0	0.0	0.5	0.0	0.0
Leucine	L	0.0	35.0	0.0	0.0	4.6	0.0	0.0
Lysine	K	89.8	0.0	0.0	0.0	0.0	0.0	0.0
Methionine	M	0.0	0.0	0.0	0.0	0.0	0.0	0.0
Phenylalanine	F	0.0	0.0	0.0	0.0	6.1	0.0	0.0
Proline	P	0.0	0.0	0.0	99.0	0.0	0.0	0.0
Serine	S	0.0	0.0	100.0	0.5	0.0	0.5	0.0
Threonine	T	0.0	3.0	0.0	0.0	0.0	99.5	0.0
Tryptophan	W	0.0	0.0	0.0	0.0	0.0	0.0	0.0
Tyrosine	Y	0.0	0.0	0.0	0.0	88.3	0.0	100.0
Valine	V	0.0	21.3	0.0	0.0	0.5	0.0	0.0
GAP	-	0.0	0.0	0.0	0.0	0.0	0.0	0.0
UNKNOWN AMINO ACID	X	0.0	0.0	0.0	0.0	0.0	0.0	0.0
		100.0	100.0	100.0	100.0	100.0	100.0	100.0

Motif O (LogOddsLogo)

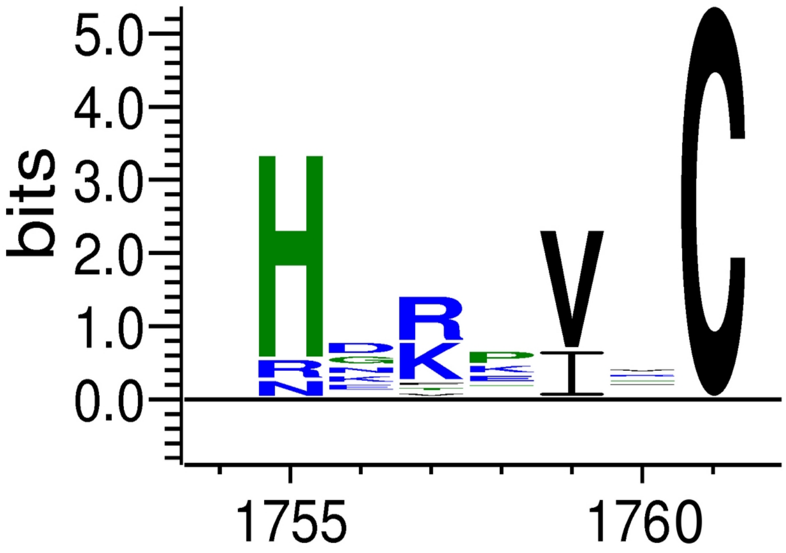

Motif O (frequency analysis)

Alanine	A	0.0	0.0	0.0	1.0	0.0	0.0	0.0
Arginine	R	1.0	1.0	22.8	0.5	0.0	39.1	0.0
Asparagine	N	0.5	4.6	0.0	0.0	0.0	0.5	0.0
Aspartate	D	0.0	43.7	0.0	0.0	0.0	0.0	0.0
Cysteine	C	0.0	0.0	0.0	0.0	0.0	0.0	100.0
Glutamine	Q	0.0	0.0	0.0	0.5	0.0	0.5	0.0
Glutamate	E	0.0	5.6	0.0	5.6	0.0	2.5	0.0
Glycine	G	0.0	7.6	0.0	0.0	0.0	0.0	0.0
Histidine	H	98.5	0.5	0.0	0.0	0.0	7.1	0.0
Isoleucine	I	0.0	0.0	0.0	0.5	11.2	6.6	0.0
Leucine	L	0.0	0.0	0.0	0.0	0.0	0.0	0.0
Lysine	K	0.0	36.5	75.6	7.1	0.0	3.0	0.0
Methionine	M	0.0	0.0	0.0	0.0	0.0	0.0	0.0
Phenylalanine	F	0.0	0.0	0.5	0.0	0.0	0.0	0.0
Proline	P	0.0	0.0	0.0	77.7	0.0	0.0	0.0
Serine	S	0.0	0.0	0.0	0.0	0.0	0.5	0.0
Threonine	T	0.0	0.5	0.5	6.6	0.0	1.5	0.0
Tryptophan	W	0.0	0.0	0.0	0.0	0.0	0.0	0.0
Tyrosine	Y	0.0	0.0	0.0	0.0	0.0	0.0	0.0
Valine	V	0.0	0.0	0.5	0.5	88.8	37.6	0.0
GAP	-	0.0	0.0	0.0	0.0	0.0	0.0	0.0
UNKNOWN AMINO ACID	X	0.0	0.0	0.0	0.0	0.0	1.0	0.0
		100.0	100.0	100.0	100.0	100.0	100.0	100.0

Motif P (LogOddsLogo)

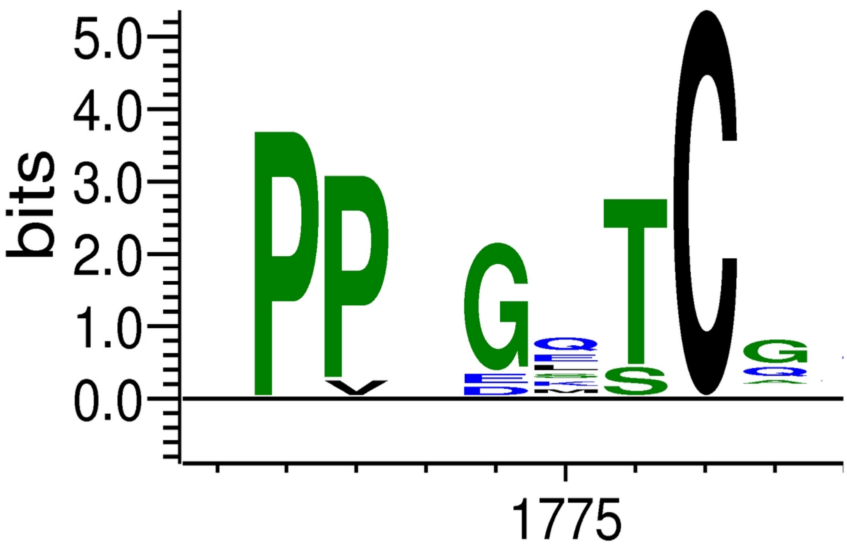

Motif P (frequency analysis)

Alanine	A	0.0	0.0	7.1	0.0	0.0	0.0	0.0	1.5
Arginine	R	0.0	0.0	0.0	0.0	0.0	0.0	0.0	0.0
Asparagine	N	0.0	0.0	27.4	0.0	0.0	0.0	0.0	0.5
Aspartate	D	0.0	0.0	3.6	0.5	0.0	0.0	0.0	0.0
Cysteine	C	0.0	0.0	0.0	0.0	0.0	0.0	100.0	0.0
Glutamine	Q	0.0	0.0	6.1	0.0	52.8	0.0	0.0	6.6
Glutamate	E	0.0	0.0	3.0	1.0	7.1	0.0	0.0	0.0
Glycine	G	0.0	0.0	0.0	98.5	0.0	0.0	0.0	84.8
Histidine	H	0.0	0.0	0.0	0.0	0.0	0.0	0.0	0.0
Isoleucine	I	0.0	0.0	11.7	0.0	0.0	0.0	0.0	0.5
Leucine	L	0.0	0.0	0.5	0.0	3.0	0.0	0.0	0.0
Lysine	K	0.0	0.0	0.0	0.0	5.1	0.0	0.0	5.6
Methionine	M	0.0	0.0	0.5	0.0	3.0	0.0	0.0	0.0
Phenylalanine	F	0.0	0.0	0.0	0.0	0.0	0.0	0.0	0.0
Proline	P	100.0	99.5	1.0	0.0	0.0	0.0	0.0	0.0
Serine	S	0.0	0.0	20.8	0.0	28.4	1.5	0.0	0.5
Threonine	T	0.0	0.0	2.0	0.0	0.0	98.5	0.0	0.0
Tryptophan	W	0.0	0.0	0.0	0.0	0.0	0.0	0.0	0.0
Tyrosine	Y	0.0	0.0	0.0	0.0	0.0	0.0	0.0	0.0
Valine	V	0.0	0.5	16.2	0.0	0.5	0.0	0.0	0.0
GAP	-	0.0	0.0	0.0	0.0	0.0	0.0	0.0	0.0
UNKNOWN AMINO ACID	X	0.0	0.0	0.0	0.0	0.0	0.0	0.0	0.0
		100.0	100.0	100.0	100.0	100.0	100.0	100.0	100.0

Motif Q (LogOddsLogo)

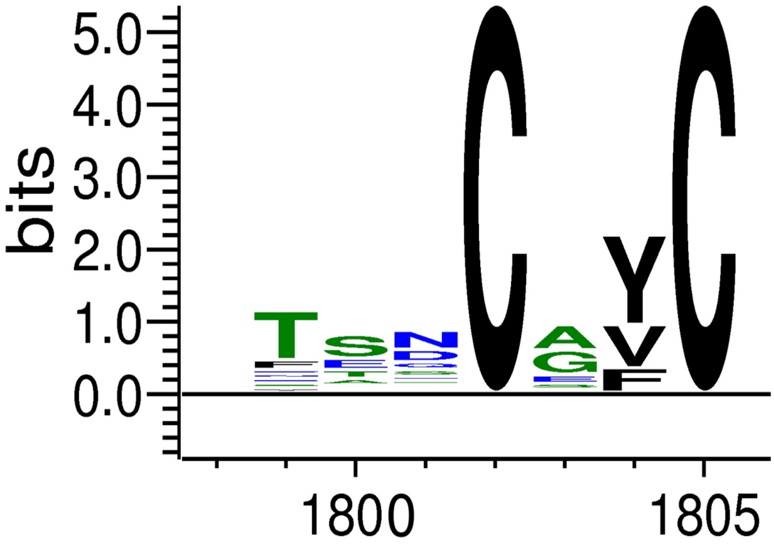

Motif Q (frequency analysis)

Alanine	A	0.0	17.8	1.0	0.0	75.1	0.0	0.0
Arginine	R	0.5	0.0	0.0	0.0	0.0	0.0	0.0
Asparagine	N	0.5	0.5	29.9	0.0	0.0	0.0	0.0
Aspartate	D	0.5	0.5	49.7	0.0	0.0	0.0	0.0
Cysteine	C	0.0	0.0	0.0	100.0	0.0	0.0	100.0
Glutamine	Q	0.0	0.0	3.0	0.0	0.5	0.0	0.0
Glutamate	E	0.0	5.1	1.5	0.0	2.0	0.0	0.0
Glycine	G	0.0	0.0	11.2	0.0	21.3	0.0	0.0
Histidine	H	0.0	0.0	0.5	0.0	0.0	0.0	0.0
Isoleucine	I	0.0	0.0	0.0	0.0	0.0	0.0	0.0
Leucine	L	0.0	0.0	0.0	0.0	0.0	0.0	0.0
Lysine	K	0.0	1.0	1.0	0.0	0.0	0.0	0.0
Methionine	M	0.0	0.0	0.0	0.0	0.0	0.0	0.0
Phenylalanine	F	1.5	0.0	0.0	0.0	0.0	2.0	0.0
Proline	P	0.0	0.0	0.0	0.0	0.0	0.0	0.0
Serine	S	0.5	72.1	2.0	0.0	1.0	0.0	0.0
Threonine	T	95.9	3.0	0.0	0.0	0.0	0.0	0.0
Tryptophan	W	0.0	0.0	0.0	0.0	0.0	0.0	0.0
Tyrosine	Y	0.5	0.0	0.0	0.0	0.0	92.4	0.0
Valine	V	0.0	0.0	0.0	0.0	0.0	5.6	0.0
GAP	-	0.0	0.0	0.0	0.0	0.0	0.0	0.0
UNKNOWN AMINO ACID	X	0.0	0.0	0.0	0.0	0.0	0.0	0.0
		100.0	100.0	100.0	100.0	100.0	100.0	100.0
