# Supplementary material for: Molecular evolution of the members of the Snq2/Pdr18 subfamily of Pdr transporters in the Hemiascomycete yeasts
Source: FEMS Yeast Res. 2025 May 27;25:foaf026. doi: 10.1093/femsyr/foaf026 (PMC12202755; doi:10.1093/femsyr/foaf026)
Supplement: foaf026_Supplemental_Files [file foaf026_supplemental_files.zip › Supplementary Data A8.pdf]

Sheet1

12848	12849	12850	12851	12853	12854	37489	119529	12857	12858	12860	1786	92725	6478	92726	sace_1_ydr011w	6614	15129	65805	91580	119530	91581	15126	14166	880	12604	1208	91582	881	92443	7797
12846	83568	12848	12849	12850	12851	12853	12854	37489	12857	12858	12860	1786	6478	134	sace_2_1_a02390	6614	15129	65805	93381	91580	91581	15126	14166	880	12604	1208	91582	881	92443	7797
174	12846	83568	12848	12849	12850	12851	12853	12854	37489	12857	12858	12860	1786	6478	sace_4_1_a02360	6614	15129	65805	91580	91581	15126	14166	880	93881	1208	91582	881	92443	7797	14641
174	12846	83568	12848	12849	12850	12851	12853	12854	37489	12857	12858	12860	1786	6478	sace_5_33_ag00510	6614	15129	65805	94385	91581	15126	14166	880	12604	1208	91582	94386	7797	14641	14640
174	12846	83568	12848	12849	12850	12851	12853	12854	37489	12857	12858	12860	1786	6478	sace_6_120_dp00100	0	0	0	0	0	0	0	0	0	0	0	0	0	0	0
174	12846	83568	12848	12849	12850	12851	12853	12854	37489	93723	12858	12860	1786	6478	sace_8_2_b02430	6614	15129	65805	91580	91581	15126	14166	880	12604	1208	91582	881	7797	14641	14640
174	12846	83568	12848	12849	12850	12851	12853	12854	37489	12857	12858	12860	1786	6478	sace_9_1_a02440	6614	15129	65805	91580	91581	15126	14166	880	12604	1208	91582	881	92443	7797	14641
174	12846	83568	12848	12849	12850	12851	12853	12854	37489	12857	12858	12860	118414	6478	sace_15_1_a02420	6614	15129	65805	91580	91581	15126	14166	880	12604	1208	91582	881	92443	7797	14641
174	12846	83568	12848	12849	12850	12851	12853	12854	37489	12857	12858	12860	118608	6478	sace_16_1_a02380	6614	15129	65805	91580	91581	15126	14166	880	12604	1208	91582	881	92443	7797	14641
174	12846	83568	12848	12849	12850	12851	12853	12854	37489	12857	12858	12860	118824	6478	sace_17_1_a02410	6614	15129	65805	91580	91581	15126	14166	880	12604	1208	91582	881	92443	7797	14641
12854	37489	12853	12857	12854	12858	37489	12857	12860	12858	119099	12860	6478	119080	6478	sace_18_1_a02410	134	6614	15129	65805	6614	15129	91580	65805	91581	15126	91580	91581	14166	15126	880
174	12846	83568	12848	12849	12850	12851	12853	12854	37489	12857	12858	12860	120034	6478	sace_21_1_2310	6614	15129	65805	91580	91581	15126	14166	880	12604	1208	91582	881	92443	7797	14641
174	12846	83568	12848	12849	12850	12851	12853	12854	37489	12857	12858	12860	120228	6478	sace_22_1_2300	6614	15129	65805	91580	91581	15126	14166	880	12604	1208	91582	881	92443	7797	14641
174	12846	83568	12848	12849	12850	12851	12853	12854	37489	12857	12858	12860	1786	6478	sace_23_1_2290	6614	15129	65805	91580	91581	15126	14166	880	12604	1208	91582	881	7797	14641	14640
174	12846	83568	12848	12849	12850	12851	12853	12854	37489	12857	12858	12860	120615	6478	sace_24_1_2300	6614	15129	65805	91580	91581	15126	14166	880	12604	1208	91582	881	92443	7797	14641
174	12846	83568	12848	12849	12850	12851	12853	12854	37489	12857	12858	12860	120791	6478	sace_25_1_a02400	6614	15129	65805	91580	91581	15126	14166	880	12604	1208	91582	881	92443	7797	14641
174	12846	83568	12848	12849	12850	12851	12853	12854	37489	12857	12858	12860	1786	6478	sace_29_1_2290	6614	15129	65805	91580	91581	15126	14166	880	12604	1208	91582	881	92443	7797	14641
174	12846	83568	12848	12849	12850	12851	12853	12854	37489	12857	93545	12860	122054	6478	sace_31_1_2300	6614	15129	65805	91580	91581	15126	14166	880	12604	1208	91582	881	92443	7797	14641
174	12846	83568	12848	12849	12850	12851	12853	12854	37489	12857	12858	12860	122865	6478	sace_34_1_2320	6614	15129	65805	65805	91580	91581	15126	14166	880	12604	1208	91582	881	92443	7797
174	12846	83568	12848	12849	12850	12851	12853	12854	37489	12857	12858	12860	123443	6478	sace_37_1_a02430	6614	15129	65805	91580	91581	15126	14166	880	12604	1208	91582	881	92443	7797	14641
92444	12846	83568	12848	12849	12850	12851	12853	12854	37489	12857	12858	12860	124165	6478	sace_40_1_a02390	6614	15129	65805	91580	91581	15126	14166	880	12604	1208	91582	881	92443	7797	14641
174	12846	83568	12848	12849	12850	12851	12853	12854	37489	12857	12858	12860	125084	6478	sace_45_1_a02420	6614	15129	65805	91580	91581	15126	14166	880	12604	1208	91582	881	92443	7797	14641
174	12846	83568	12848	12849	12850	12851	12853	12854	37489	12857	12858	12860	125276	6478	sace_46_1_a02400	6614	15129	65805	91580	91581	15126	14166	880	12604	1208	91582	881	92443	7797	14641
174	12846	83568	12848	12849	12850	12851	12853	12854	37489	12857	12858	12860	125468	6478	sace_47_1_a02400	6614	15129	65805	91580	91581	15126	14166	880	12604	1208	91582	881	92443	7797	14641
174	12846	83568	12848	12849	12850	12851	12853	12854	37489	12857	12858	12860	1786	6478	sace_48_1_a02380	6614	15129	65805	91580	91581	15126	14166	880	12604	1208	91582	881	92443	7797	14641
174	93721	83568	93722	12849	12850	12851	12853	12854	37489	93723	12858	12860	1786	6478	sace_49_1_a02460	6614	15129	65805	93381	93724	91581	15126	14166	880	12604	1208	91582	881	7797	14641
174	12846	83568	12848	12849	12850	12851	12853	12854	37489	12857	12858	12860	1786	6478	sace_50_1_a02410	134	6614	15129	65805	93381	91580	91581	15126	14166	880	12604	1208	91582	881	92443
174	12846	83568	12848	12849	12850	12851	12853	12854	37489	12857	12858	12860	1786	6478	sace_52_1_a02400	134	6614	15129	65805	91580	91581	15126	14166	880	12604	1208	91582	881	92443	7797
174	12846	83568	12848	12849	12850	12851	12853	12854	37489	12857	12858	12860	1786	6478	sace_56_1_a02020	6614	15129	65805	91580	91581	15126	14166	880	12604	1208	91582	881	92443	7797	14641
174	12846	83568	12848	12849	12850	12851	12853	12854	37489	12857	12858	12860	1786	6478	sace_57_1_a02410	6614	15129	65805	91580	91581	15126	14166	880	12604	1208	91582	881	92443	94208	14641

174	12846	83568	12848	12849	12850	12851	12853	12854	37489	12857	12858	12860	1786	6478	sace_58_25_y00780	6614	0	0	0	0	0	0	0	0	0	0	0	0	0	0
12846	83568	126904	12848	12849	12850	12851	12853	12854	37489	12857	93545	12860	1786	6478	sace_59_110_df00100	0	0	0	0	0	0	0	0	0	0	0	0	0	0	0
174	12846	83568	12848	12849	12850	12851	12853	12854	37489	12857	12858	12860	1786	6478	sace_60_4_d02440	6614	15129	65805	91580	91581	15126	14166	880	12604	1208	91582	881	92443	94208	14641
174	12846	83568	12848	12849	12850	12851	12853	12854	37489	12857	12858	12860	1786	6478	sapa_2_1_a02460	6614	15129	65805	91580	91581	15126	14166	880	12604	1208	91582	132947	881	7797	14641
174	12846	83568	12848	12849	12850	12851	12853	12854	37489	12857	12858	12860	1786	6478	sapa_3_1_a02470	6614	15129	65805	91580	91581	15126	14166	880	12604	1208	91582	133180	881	7797	14641
174	12846	83568	12848	12849	12850	12851	12853	12854	37489	12857	12858	12860	1786	6478	sapa_4_1_a02470	6614	15129	65805	91580	91581	15126	14166	880	12604	1208	91582	881	7797	14641	14640
174	12846	83568	12848	12849	12850	12851	12853	12854	37489	12857	12858	12860	1786	6478	sapa_5_1_2350	6614	15129	65805	91580	91581	15126	14166	880	12604	1208	91582	881	7797	14641	14640
174	12846	83568	12848	12849	12850	12851	12853	12854	37489	12857	12858	12860	1786	6478	sapa_7_1_2370	6614	15129	65805	91580	91581	15126	14166	880	12604	1208	91582	133889	881	7797	14641
174	12846	83568	12848	12849	12850	12851	12853	12854	37489	12857	12858	12860	1786	6478	sapa_8_1_2350	6614	15129	65805	91580	91581	15126	14166	880	12604	1208	91582	134122	134123	881	7797
174	12846	83568	12848	12849	12850	12851	12853	12854	37489	12857	12858	12860	1786	6478	sapa_9_1_2360	6614	15129	65805	134320	91580	91581	15126	14166	880	12604	1208	91582	134321	881	7797
174	12846	83568	12848	12849	12850	12851	12853	12854	37489	12857	12858	12860	1786	6478	sapa_11_1_a02470	6614	15129	65805	91580	91581	15126	14166	880	12604	1208	91582	129443	881	7797	14641
174	12846	83568	12848	12849	12850	12851	12853	12854	37489	12857	12858	12860	1786	6478	sapa_14_1_a02440	6614	15129	65805	91580	91581	15126	14166	880	12604	1208	91582	881	7797	14641	14640
174	12846	83568	12848	12849	12850	12851	12853	12854	37489	12857	12858	12860	1786	6478	sapa_17_1_2380	6614	15129	65805	91580	91581	15126	14166	880	12604	1208	91582	130639	881	7797	14641
130889	174	12846	83568	12848	12849	12850	12851	12853	12854	37489	12857	12858	12860	1786	sapa_18_1_2390	6614	15129	65805	91580	91581	15126	14166	880	12604	1208	91582	130890	881	7797	14641
174	12846	83568	12848	12849	12850	12851	12853	12854	37489	12857	12858	12860	1786	6478	sapa_23_1_a02480	6614	15129	65805	91580	91581	15126	14166	880	12604	1208	91582	132166	881	7797	14641
174	12846	83568	12848	12849	12850	12851	12853	12854	37489	12857	12858	12860	1786	6478	sapa_25_1_a02460	6614	15129	65805	132619	91580	91581	15126	14166	880	12604	1208	91582	132620	881	7797
174	12846	83568	12848	12849	12850	12851	12853	12854	37489	12857	12858	12860	1786	6478	sami_1_4.244	6614	15129	65805	15620	91580	91581	15126	14166	880	12604	1208	91582	881	7797	14641
174	12846	83568	12848	12849	12850	12851	12853	12854	37489	12857	12858	12860	1786	6478	saku_1_4.262	6614	15129	65805	15620	91580	91581	15126	14166	880	12604	1208	91582	881	7797	14641
12846	83568	12848	12849	12850	12851	12853	12854	37489	12857	12858	12860	1786	91579	6478	saar_1_2_b02590	117417	15129	65805	91580	91581	15126	14166	880	12604	1208	91582	881	7797	14641	14640
92175	12846	83568	12848	12849	12850	12851	12853	12854	37489	12858	12860	1786	6478	134	saba_1_58_bf00280	12333	348	11474	6584	12731	5312	14168	0	0	0	0	0	0	0	0
348	1786	677	557	12859	1720	65613	12855	12854	12853	570	655	12848	18251	6478	kaaf_1_c00820	134	12732	6584	37045	12763	65614	9355	4478	12997	2913	285	976	1664	65615	13001
1786	677	557	12859	1720	65613	12855	12854	12853	570	655	12848	18251	6478	134	kaaf_1_c00830	12732	6584	37045	12763	65614	9355	4478	12997	2913	285	976	1664	65615	13001	9312
174	12846	67036	12852	12851	12850	9574	12849	12848	655	570	2692	949	6478	66431	kana_1_k01350	12732	6584	37045	12763	12763	67035	4478	12997	2913	285	976	1664	67034	13001	9312
14299	12773	153	14295	83954	83953	83952	174	14358	7275	18251	949	6478	134	134	naca_1_e01630	6584	37045	12862	12763	4478	12997	2913	134	13688	4503	54785	6008	13695	12853	12854
83955	14299	12773	153	14295	83954	83953	83952	174	14358	7275	18251	949	6478	134	naca_1_e01640	134	6584	37045	12862	12763	4478	12997	2913	134	13688	4503	54785	6008	13695	12853
83956	83955	14299	12773	153	14295	83954	83953	83952	174	14358	7275	18251	949	6478	naca_1_e01650	134	134	6584	37045	12862	12763	4478	12997	2913	134	13688	4503	54785	6008	13695
84890	84889	14299	12773	153	14295	84888	84887	83952	174	14358	7275	18251	6478	134	nada_1_g01840	6584	37045	84737	12862	12763	4478	12997	2913	134	13688	1340	4503	54785	6008	13695
4411	84890	84889	14299	12773	153	14295	84888	84887	83952	174	14358	7275	18251	6478	nada_1_g01850	134	6584	37045	84737	12862	12763	4478	12997	2913	134	13688	1340	4503	54785	6008
37050	9785	6632	13675	37049	13677	37048	4927	5143	6307	37047	13682	37046	1579	174	cagl_1_i04862g	12732	12731	6584	37045	3184	348	11474	5312	14168	14167	14166	12603	37044	37043	12763
485	8151	98097	16722	11173	13495	11429	13493	98098	13485	135147	98099	18251	949	6478	tebl_1_i01760	36605	6584	37045	15	98100	15126	14168	14167	14166	1208	98101	98102	14032	2150	135148

10261	12763	12994	9355	8078	12995	4478	12997	98258	83568	12846	174	11810	18251	6478	teph_1_a04220	36605	12732	6584	37045	15	3184	348	98257	15126	14168	14166	12603	881	98256	15842
11810	99782	55	1828	14469	135404	1798	14470	135335	14471	3683	14473	14474	18251	6478	vapo_1_1037.47	36605	12732	6584	37045	15	3184	348	99783	15126	14168	14166	12603	881	99784	0
2292	3241	97965	97966	13861	12382	97967	97968	14334	2911	14331	159	265	2989	6478	tebl_1_g02820	10010	12731	6584	2080	37045	7669	3184	12664	6614	348	15129	97969	11474	15620	5312
12864	135219	12867	12868	1208	963	12871	12872	98916	7161	7161	98915	65620	949	6478	teph_1_m00640	10010	12732	12731	2080	7669	12664	6614	11474	12991	12990	12989	12988	12987	65617	12983
12867	12868	1208	963	12871	12872	99767	7161	7161	99768	65620	949	6478	55	55	vapo_1_1036.28	10010	12732	12731	2080	7669	9574	12852	12853	4508	1260	99769	12857	12859	12860	557
12345	4008	19789	3978	12154	14411	92456	67505	67447	40328	15	15	12254	14615	14615	sace_1_ynr070w	949	134	91469	44102	10469	119752	1213	93154	0	0	0	0	0	0	0
2819	93517	12345	4008	19789	3978	12154	14411	92456	67505	67447	40328	15	12254	14615	sace_2_8_h03860	949	134	91469	12973	23124	12266	12266	68144	121782	10469	0	0	0	0	0
5143	12716	91549	12244	12243	12241	1208	12239	12236	9256	5600	91968	12254	14615	14615	sace_4_8_h03690	949	91968	12228	13041	15	1214	91969	9670	14781	1188	2819	2819	14782	12345	4008
0	0	0	0	0	0	0	0	0	0	0	0	0	12254	14615	sace_5_78_bz00120	134	949	0	0	0	0	0	0	0	0	0	0	0	0	0
0	0	0	0	0	0	0	0	0	0	0	15	12254	14615	14615	sace_6_169_fm00160	949	0	0	0	0	0	0	0	0	0	0	0	0	0	0
2819	14782	12345	4008	19789	3978	12154	14411	92456	67505	67447	40328	15	12254	14615	sace_9_7_g00180	949	134	91469	12973	23124	12266	68144	10469	0	0	0	0	0	0	0
12345	12345	4008	19789	3978	12154	14411	92456	67505	67447	40328	15	12254	14615	14615	sace_14_7_g00150	949	134	91469	44102	10469	0	0	0	0	0	0	0	0	0	0
12345	12345	4008	19789	3978	12154	14411	92456	67505	67447	40328	15	12254	14615	14615	sace_15_7_g03870	949	134	91469	44102	10469	118509	0	0	0	0	0	0	0	0	0
14782	12345	12345	4008	19789	3978	12154	14411	92456	67505	67447	40328	15	12254	14615	sace_17_7_g03930	949	134	134	91469	44102	10469	118922	0	0	0	0	0	0	0	0
12349	67505	12348	12347	67447	9925	40328	55	15	1610	12254	3226	14615	17089	91379	sace_18_7_g03880	134	18147	949	134	1213	10469	91469	44102	10469	0	0	0	0	0	0
12345	12345	4008	19789	3978	12154	14411	92456	67505	67447	40328	15	12254	14615	14615	sace_19_7_3840	949	134	91469	44102	119449	0	0	0	0	0	0	0	0	0	0
14782	12345	4008	19789	3978	12154	14411	92456	67505	67447	40328	15	15	12254	14615	sace_21_7_3790	949	134	91469	44102	10469	120141	0	0	0	0	0	0	0	0	0
2819	14782	12345	4008	19789	3978	12154	14411	92456	67505	67447	40328	15	12254	14615	sace_23_7_3860	949	134	134	91469	44102	10469	120533	120534	0	0	0	0	0	0	0
14782	12345	4008	19789	3978	12154	14411	92456	67505	67447	40328	15	12254	14615	14615	sace_24_8_3780	949	134	91469	44102	10469	1213	92588	0	0	0	0	0	0	0	0
2819	14782	12345	4008	19789	3978	12154	14411	92456	67505	67447	40328	15	12254	14615	sace_25_7_g03880	949	134	91469	44102	10469	120889	0	0	0	0	0	0	0	0	0
14782	12345	4008	4008	19789	3978	12154	14411	92456	67505	67447	40328	15	12254	14615	sace_31_7_3780	949	134	91469	44102	10469	122154	1213	122155	0	0	0	0	0	0	0
2819	14782	12345	4008	19789	3978	12154	14411	92456	67505	67447	40328	15	12254	14615	sace_34_8_3770	949	134	91469	44102	10469	93582	0	0	0	0	0	0	0	0	0
2819	14782	12345	4008	19789	3978	12154	14411	92456	67505	67447	40328	15	12254	14615	sace_35_7_3840	949	134	91469	44102	10469	123170	123171	123172	0	0	0	0	0	0	0
2819	14782	12345	4008	19789	3978	12154	14411	92456	67505	67447	40328	15	12254	14615	sace_37_7_g03850	949	134	91469	44102	10469	123536	0	0	0	0	0	0	0	0	0
12345	12345	4008	19789	3978	12154	14411	92456	67505	67447	40328	15	12254	14615	14615	sace_40_8_h03830	949	134	91469	44102	124261	0	0	0	0	0	0	0	0	0	0
14782	12345	4008	4008	19789	3978	12154	14411	92456	67505	67447	40328	15	12254	14615	sace_43_7_g03870	949	134	91469	44102	10469	1213	124785	0	0	0	0	0	0	0	0
2819	14782	12345	4008	19789	3978	12154	14411	92456	67505	67447	40328	15	12254	14615	sace_45_7_g03890	949	134	91469	44102	10469	125184	0	0	0	0	0	0	0	0	0
2819	14782	12345	4008	19789	3978	12154	14411	92456	67505	67447	40328	15	12254	14615	sace_46_8_h03910	949	134	134	91469	44102	125377	0	0	0	0	0	0	0	0	0
12345	4008	19789	3978	12154	14411	92456	67505	67447	40328	15	15	12254	14615	14615	sace_49_8_h03830	949	134	91469	44102	10469	10469	0	0	0	0	0	0	0	0	0
12345	4008	19789	3978	12154	14411	92456	67505	67447	40328	15	15	12254	14615	14615	sace_53_29_ac00130	949	134	91469	0	0	0	0	0	0	0	0	0	0	0	0

Sheet1

4008	19789	3978	12154	14411	92456	67505	67447	40328	15	15	12254	14615	14615	14615	sace_56_17_q01110	949	134	91469	9785	91971	91972	0	0	0	0	0	0	0	0	0
2819	14782	12345	4008	19789	3978	12154	14411	92456	67505	67447	40328	15	12254	14615	sace_57_8_h03900	949	134	134	91469	12973	23124	12266	68144	10469	10469	0	0	0	0	0
2819	14782	12345	4008	19789	3978	12154	14411	92456	67505	67447	40328	15	12254	14615	sace_58_71_bs00120	134	949	0	0	0	0	0	0	0	0	0	0	0	0	0
0	0	0	0	0	0	0	0	0	0	0	0	0	0	0	sace_59_336_lx00100	949	0	0	0	0	0	0	0	0	0	0	0	0	0	0
2819	14782	12345	4008	19789	3978	12154	14411	92456	67505	67447	40328	15	12254	14615	sace_60_6_f03350	949	134	134	91469	0	0	0	0	0	0	0	0	0	0	0
2819	14782	12345	4008	19789	3978	12154	14411	92456	67505	67447	40328	15	12254	14615	sapa_1_8_h03820	949	134	91469	12973	23124	12266	1213	1213	55	0	0	0	0	0	0
12345	4008	19789	3978	12154	14411	92456	67505	67447	40328	1213	133073	15	12254	14615	sapa_2_8_h03860	949	134	91469	12973	23124	12266	1213	10469	0	0	0	0	0	0	0
12345	4008	19789	3978	12154	14411	92456	67505	67447	40328	1213	133312	15	12254	14615	sapa_3_8_h03890	949	134	91469	12973	23124	12266	1213	10469	133313	0	0	0	0	0	0
14782	133461	12345	4008	19789	3978	12154	14411	92456	67505	67447	40328	15	12254	14615	sapa_4_8_h03850	949	134	91469	12973	23124	12266	1213	68144	133462	0	0	0	0	0	0
14782	133601	12345	4008	19789	3978	12154	14411	92456	67505	67447	40328	15	12254	14615	sapa_5_8_3700	949	134	91469	12973	23124	12266	1213	68144	0	0	0	0	0	0	0
2819	14782	12345	4008	19789	3978	12154	14411	92456	67505	67447	40328	15	12254	14615	sapa_6_8_3750	949	134	91469	12973	23124	12266	1213	133781	0	0	0	0	0	0	0
12345	4008	19789	3978	12154	14411	92456	67505	67447	40328	1213	134023	15	12254	14615	sapa_7_8_3740	949	134	91469	12973	23124	12266	1213	10469	134024	0	0	0	0	0	0
14782	134240	12345	4008	19789	3978	12154	14411	92456	67505	67447	40328	15	12254	14615	sapa_8_8_3750	949	134	91469	12973	23124	12266	1213	134241	0	0	0	0	0	0	0
14782	12345	4008	19789	3978	12154	14411	92456	67505	67447	40328	134430	15	12254	14615	sapa_9_8_3720	949	134	91469	12973	23124	12266	1213	134431	0	0	0	0	0	0	0
14782	12345	4008	19789	3978	12154	14411	92456	67505	67447	40328	129368	15	12254	14615	sapa_10_8_3760	949	134	91469	12973	23124	12266	1213	129369	0	0	0	0	0	0	0
2819	14782	12345	4008	19789	3978	12154	14411	92456	67505	67447	40328	15	12254	14615	sapa_11_8_h03830	949	134	91469	12973	23124	12266	1213	68144	0	0	0	0	0	0	0
14782	129880	12345	4008	19789	3978	12154	14411	92456	67505	67447	40328	15	12254	14615	sapa_13_8_h03820	949	134	91469	12973	23124	12266	1213	129881	0	0	0	0	0	0	0
12345	4008	19789	3978	12154	14411	92456	67505	67447	40328	130521	15	12254	14615	130522	sapa_16_8_h03890	949	134	91469	12973	23124	12266	1213	1213	130523	0	0	0	0	0	0
12345	4008	19789	3978	12154	14411	92456	67505	67447	40328	1213	130768	15	12254	14615	sapa_17_8_3730	949	134	91469	12973	23124	12266	1213	10469	130769	0	0	0	0	0	0
2819	14782	12345	4008	19789	3978	12154	14411	92456	67505	67447	40328	15	12254	14615	sapa_18_8_3730	949	134	91469	12973	23124	92340	1213	131036	0	0	0	0	0	0	0
131295	131296	12345	4008	19789	3978	12154	14411	92456	67505	67447	40328	15	12254	14615	sapa_19_8_h03900	949	134	91469	12973	23124	12266	1213	1213	131297	0	0	0	0	0	0
131592	131593	12345	4008	19789	3978	12154	14411	92456	67505	67447	40328	15	12254	14615	sapa_20_8_h03860	949	134	91469	12973	23124	12266	1213	131594	0	0	0	0	0	0	0
14782	131788	12345	4008	19789	3978	12154	14411	92456	67505	67447	40328	15	12254	14615	sapa_21_8_h03870	949	134	91469	12973	23124	12266	1213	131789	0	0	0	0	0	0	0
14782	132050	12345	4008	19789	3978	12154	14411	92456	67505	67447	40328	15	12254	14615	sapa_22_8_h03900	949	134	91469	12973	23124	12266	1213	1213	132051	0	0	0	0	0	0
14782	132295	12345	4008	19789	3978	12154	14411	92456	67505	67447	40328	15	12254	14615	sapa_23_8_h03850	949	134	91469	12973	23124	12266	1213	10469	132296	0	0	0	0	0	0
2819	14782	12345	4008	19789	3978	12154	14411	92456	67505	67447	40328	15	12254	14615	sapa_24_8_h03850	949	134	91469	12973	23124	12266	1213	1213	132544	0	0	0	0	0	0
14782	132734	12345	4008	19789	3978	12154	14411	92456	67505	67447	40328	15	12254	14615	sapa_25_8_h03870	949	134	91469	12973	23124	12266	1213	20797	0	0	0	0	0	0	0
1188	2819	14782	128590	128591	128592	12345	4008	19789	3978	12154	14411	15	12254	14615	sami_1_14.399	949	134	91469	128593	128594	128595	12973	23124	128596	128597	94902	1213	36521	0	0
55	128716	94929	128717	128718	94930	128719	94931	128720	128721	128722	5571	14186	8115	44102	sami_1_17.26	12234	0	0	0	0	0	0	0	0	0	0	0	0	0	0
127925	127926	12345	4008	19789	3978	12154	14411	92456	67505	67447	40328	15	12254	14615	saku_1_14.404	949	134	0	0	0	0	0	0	0	0	0	0	0	0	0

1188	2819	14782	91970	12345	4008	19789	3978	12154	14411	67505	67447	40328	12254	14615	saar_1_8_h03780	949	134	91469	44102	9785	91971	117499	91972	14893	71547	1213	0	0	0	0
14091	14090	1798	12786	91625	14087	55053	14098	1528	14100	2524	66156	12301	13962	13962	sauv_1_7.3	44102	949	0	0	0	0	0	0	0	0	0	0	0	0	0
4014	14674	174	55	14675	3199	104137	7261	10054	15	12871	225	13884	3841	13883	zyba_1_02055	134	10010	36605	12732	11260	12731	6584	2080	37045	7669	15	3184	12664	6614	348
0	0	0	635	13838	13837	13836	13835	13834	5643	13833	104391	15510	13829	20246	zyba_1_04634	46332	13828	104392	1464	7843	13826	104393	6558	13824	13823	4116	104394	13821	13819	13818
10392	13840	20797	635	13838	13837	13836	13835	13834	5643	13833	104391	15510	13829	20246	zyba_1_06675	46332	13828	104392	1464	7843	13826	104393	6558	13824	13823	4116	104394	13821	13819	13818
4014	14674	174	55	14675	3199	104137	7261	10054	15	12871	225	13884	3841	13883	zyba_1_07912	10010	36605	12732	11260	12731	6584	2080	37045	7669	15	3184	12664	6614	348	15130
13840	20797	135761	635	13838	13837	13836	13835	13834	5643	13833	104391	15510	13829	20246	zyba_2_1_a00860	46332	13828	104392	1464	7843	13826	104393	6558	13824	13823	4116	104394	13821	13819	13818
0	0	0	0	0	0	0	0	0	0	0	0	0	0	12234	zyba_2_14_n01490	104590	12155	104589	104588	135751	104587	7184	13181	37146	134	14235	12155	55	7184	12155
4014	14674	174	55	14675	3199	104137	7261	10054	15	12871	225	13884	3841	13883	zyba_2_2_b00600	10010	36605	12732	11260	12731	6584	2080	37045	7669	15	3184	12664	6614	348	15130
0	0	0	0	0	0	0	0	22208	12155	735	12155	104642	104641	12155	zyba_2_33_ag00120	104640	1213	0	0	0	0	0	0	0	0	0	0	0	0	0
135863	20797	135862	635	13838	13837	13836	13835	13834	5643	13833	104391	15510	13829	20246	zyba_3_2_b02230	46332	13828	104392	1464	7843	13826	104393	6558	13824	13823	4116	104394	13821	13819	13818
4014	14674	174	55	14675	3199	104137	7261	10054	15	12871	225	13884	3841	13883	zyba_3_3_c03460	10010	36605	12732	11260	12731	6584	2080	37045	7669	15	3184	12664	6614	348	15130
12852	570	655	12851	12850	9574	12849	12848	104745	83568	12846	174	18251	949	6478	zyro_1_a04114g	10010	36605	12732	11260	12731	6584	2080	37045	7669	15	3184	12664	6614	348	15129
14273	104796	4008	14271	18958	14270	12954	12953	135678	12950	12142	21974	8053	4430	12949	zyro_1_b14762g	12948	12947	3817	12946	1366	12606	12945	7457	97400	12942	12940	104256	2564	7459	15893
4508	12854	12853	12852	570	655	12851	12850	9574	12849	12848	83568	12846	174	99300	tode_1_d04040	10010	12732	11260	12731	6584	2080	37045	7669	15	3184	12664	6614	348	15129	65805
70687	7100	9725	65466	55	55	2080	6584	12731	11260	12732	134	36605	10802	70686	lakl_1_c11616g	10010	6478	949	18251	174	12846	12847	12848	12849	9574	12850	12851	655	570	12852
13371	1759	15	71172	13709	1072	71171	13373	13374	13375	13376	71170	13378	16550	54775	lakl_1_h21010g	71169	71168	13384	8144	13385	14827	13396	14190	67495	12357	12356	71167	71166	12355	12354
12384	375	1208	2278	9414	12385	12386	12389	71216	10785	9209	9511	18251	949	6478	lath_1_a01914g	36605	12732	11260	12731	6584	2080	65466	9725	7100	71215	71214	12730	16803	1575	12729
1208	2278	9414	12385	12385	12386	114064	12389	72647	10785	72648	9209	9511	72649	72650	lawa_1_23.5161	36605	12732	72651	114065	11260	114066	12731	72652	114067	114068	6584	72653	2080	72654	65466
0	0	0	0	0	0	0	0	0	0	0	0	0	6478	67288	klae_1_14_n00120	10010	134	12732	11260	12731	2080	6584	225	12983	8661	67289	67290	12986	12987	12988
12908	485	18191	265	15	13857	13858	13859	67444	13861	113096	9785	67341	13863	3986	klla_1_d03432g	10010	134	12732	11260	12731	2080	6584	225	12983	8661	67289	68137	67290	12986	12987
68416	12908	485	18191	265	15	13857	13858	13859	67444	13861	9785	67341	13863	3986	klma_1_a01880	10010	134	12732	11260	12731	2080	6584	225	12983	8661	67289	68415	67290	12986	12987
0	0	0	0	0	0	0	0	11521	3969	18251	949	6478	67288	68963	klwi_1_33_ag00170	68964	10010	134	12732	11260	12731	2080	6584	225	12983	8661	67289	68965	67290	12986
174	14793	7000	11212	14792	6284	14791	14790	14789	14788	14787	14786	14785	14784	134	asac_1_6_f03560	10010	14783	5571	14782	2819	1188	14781	14780	9670	14779	3093	12155	12155	12155	12155
4493	54920	7019	400	1786	12490	13396	13397	5174	140	13398	2080	6584	12732	134	ercy_1_3604	10010	13447	13448	13449	2681	13450	13451	6929	13452	54919	13454	13456	13457	15	13458
7332	174	14793	7000	11212	14792	6284	14791	14790	14789	14788	14787	14786	14784	134	ergo_1_abr125c	10010	14783	5571	14782	2819	1188	14781	14780	9670	14779	3093	12155	105	14778	9579
15	15	15	2671	14703	20400	20401	1601	14176	12759	134	14916	13543	13836	225	caal_1_19.5759	20402	20403	16357	15534	20404	20405	13906	13905	13903	20406	9312	19553	3905	14010	14661
15	15	15	2671	14703	20400	20401	1601	14176	12759	134	14916	13543	13836	225	caal_10_3_c03340	20402	20403	16357	15534	20404	20405	13906	13905	13903	20406	9312	19553	106927	0	0
0	0	15	2671	14703	20400	20401	1601	14176	12759	134	14916	13543	13836	225	caal_11_25_y00230	20402	20403	16357	15534	20404	20405	13906	13905	13903	20406	9312	107002	3905	14010	14661
15	20852	20853	2671	14703	20400	20401	1601	14176	12759	134	14916	13543	13836	225	caal_12_26_z00530	20402	20403	16357	15534	20404	20405	13906	13905	13903	20406	9312	107111	0	0	0

1214	15	15	2671	14703	20400	20401	1601	14176	12759	134	14916	13543	13836	225	caal_2_04989	20402	20403	16357	15534	20404	20405	13906	13905	13903	20406	9312	19553	3905	14010	14661
15	15	15	2671	14703	20400	20401	1601	14176	12759	134	14916	13543	13836	225	caal_3_29_ac00530	20402	20403	16357	15534	20404	20405	13906	13905	13903	20406	9312	0	0	0	0
15	15	15	2671	14703	20400	20401	1601	14176	12759	134	14916	13543	13836	225	caal_4_4_d03320	20402	20403	16357	15534	20404	20405	13906	13905	13903	20406	9312	19553	0	0	0
1214	15	15	2671	14703	20400	20401	1601	14176	12759	134	14916	13543	13836	225	caal_5_30_ad00540	20402	20403	16357	15534	20404	20405	13906	13905	20406	9312	0	0	0	0	0
1214	20852	20853	2671	14703	20400	20401	1601	14176	12759	134	14916	13543	13836	225	caal_6_4_d03280	20402	20403	16357	15534	20404	20405	13906	13905	13903	20406	9312	0	0	0	0
15	15	15	2671	14703	20400	20401	1601	14176	12759	134	14916	13543	13836	225	caal_8_3_c03320	20402	20403	16357	15534	20404	20405	13906	13905	13903	20406	9312	107827	3905	14010	14661
1214	15	15	2671	14703	20400	20401	1601	14176	12759	134	14916	13543	13836	225	cadu_1_64350	20402	20403	16357	15534	20404	20405	13906	13905	13903	20406	9312	19553	3905	14010	14661
12591	12796	40781	20630	20631	14123	20632	40782	40783	14865	4503	13994	19092	19055	19055	catr_1_01205	12356	16332	13404	13405	13404	16006	20634	9081	13054	12763	13191	19998	203	14707	12389
3071	5391	15052	19709	1253	19683	11037	21143	13491	3703	110045	41242	41243	41244	41243	catr_1_05498	225	2628	2681	4202	7618	7651	14760	3804	14812	19482	14719	19473	19472	19470	7184
0	0	0	0	0	0	0	0	0	0	0	0	37728	3226	13543	catr_1_05971	16006	1313	15	8661	14020	19913	3093	19225	19909	3226	5949	7619	13590	4987	19908
15338	13993	18920	59	38697	20395	14641	17071	14104	38696	13206	38695	1041	13836	225	capa_1_600750	13543	134	694	17016	20380	12538	17554	498	38973	8524	14719	12878	38831	38832	20391
20262	15000	12457	12731	109753	38829	20561	4550	17233	14044	38830	38634	38555	13836	225	caor_1_h02090	13543	12538	20380	17016	17554	498	8524	14719	12878	38831	38832	20391	3841	1790	38634
17071	79999	14104	14104	80000	79564	11810	80001	13206	80002	1041	14044	13836	225	134	loel_1_04930	80003	80004	12538	17554	1601	80005	79567	14176	12759	12457	977	80006	17016	16223	80007
13553	6621	96727	14641	20396	20386	18888	1041	96728	13206	20384	20401	14703	13836	225	spar_1_5_e03260	13543	12538	12290	12759	14176	1601	18213	134	96729	0	0	0	0	0	0
13553	6621	96727	20396	14641	20386	18888	1041	97055	13206	20384	20401	14703	13836	225	sppa_1_7_g03160	13543	12538	12290	17554	12759	14176	1601	18213	14916	13404	96361	13661	96533	20391	3841
55	21695	8524	17071	14104	14719	12878	20391	3841	498	20380	20381	2671	13836	225	scst_1_3_c02890	13543	95894	12538	17554	12759	14176	1601	18213	134	14916	13404	95895	15160	2819	1188
19379	13882	46265	46266	1208	3655	46267	14904	2564	37937	14032	13861	20253	2671	225	deha_1_a03696g	13543	46268	110830	16803	2908	12751	4811	19647	4008	46269	20260	9407	5680	2147	865
9725	111164	19379	13882	1208	3655	46267	14904	2564	37937	14032	13861	20253	2671	225	deha_2_5_e00720	13543	0	0	0	0	0	0	0	0	0	0	0	0	0	0
669	16136	17901	3045	41975	1693	20670	2150	4618	11487	6088	81112	10587	13874	81113	mebi_1_8_h00300	18888	20386	4343	16107	7246	42489	81114	42491	19386	138	115961	13193	81115	10220	81116
0	0	0	0	0	0	0	0	0	0	0	0	0	0	0	bain_1_1_a00100	17993	17994	13292	13489	17995	106756	5983	17996	17997	13614	17998	3694	485	2533	14019
6020	17915	17916	12155	17917	12709	914	17918	1313	17919	15155	12804	3184	9233	17920	bain_1_17_q00380	1774	17921	17922	17923	17924	13376	13375	13389	7184	17925	8650	17926	12932	57	14661
93	19178	12878	12531	19179	15534	19180	19181	8524	12888	12657	19182	1801	106849	19183	bain_1_8_h00410	12617	13123	19184	14340	16780	106850	13951	15267	12577	12386	19185	12582	13275	17254	19186
4430	116877	9437	116876	12159	12159	140	2741	17488	15120	87378	87377	12895	13396	87376	pata_1_2_b05590	14827	8144	87375	13291	5470	5470	17254	13426	525	12092	14340	285	7184	13791	13792
0	0	21776	14091	14092	14322	21775	511	21774	16581	13343	8461	21773	13344	134	caar_1_13_m01420	12612	12613	2390	21772	21771	263	14020	21770	12999	21769	21768	17365	14019	21767	13928
14103	14103	2588	21812	6568	6507	21813	15	11038	14101	12855	13164	3674	13404	134	caar_1_14_n01430	21814	0	0	0	0	0	0	0	0	0	0	0	0	0	0
44702	21707	17330	114	13848	153	22330	44766	13581	13580	44768	3674	13404	13404	134	debr_2_5_e03380	44721	3084	518	12887	12793	46166	15514	3719	13245	14537	46167	46168	13240	12190	18861
17014	134	14103	14103	2588	55410	6568	6507	55409	15	11038	14101	12855	13164	13404	hapo_1_1_a07220	1628	174	7267	3525	13263	22946	55408	7759	3953	22944	55407	22942	1316	12795	8737
17014	134	14103	14103	2588	55410	6568	6507	55409	15	11038	14101	12855	13164	13404	ogpa_1_1_a01680	1628	174	7267	3525	13263	22946	55408	7759	3953	22944	55407	22942	1316	12795	8737
12514	12159	12345	12299	44032	577	12300	13828	20750	14469	100365	12298	7184	14551	11038	wian_1_1_a02920	134	17246	100366	100367	1464	7843	13826	100368	6558	13824	13823	15025	13374	694	18950
12159	12345	12299	44032	577	12300	13828	20750	14469	100365	12298	7184	14551	11038	134	wian_1_1_a02930	17246	100366	100367	1464	7843	13826	100368	6558	13824	13823	15025	13374	694	18950	22517

43877	100902	15562	16833	14240	14240	14240	135491	12531	13280	100903	100904	15275	43191	43191	wian_1_3_c04380	134	13973	7241	285	43678	13975	13426	3108	153	14877	100905	14878	43685	14880	5640
100902	15562	16833	14240	14240	14240	135491	12531	13280	100903	100904	15275	43191	43191	134	wian_1_3_c04390	13973	7241	285	43678	13975	13426	3108	153	14877	100905	14878	43685	14880	5640	16836
525	17464	10111	1786	10261	101413	101414	15105	153	12155	7184	12877	1581	134	134	wian_1_7_g01010	9735	16803	4116	135558	13821	17899	15466	13818	1061	10621	14233	101415	174	101416	101197
14059	12372	15476	15477	15478	13517	343	6657	15479	15480	15481	15	11530	6222	15482	asru_1_13_m01190	153	285	1615	13404	15478	10054	14934	2766	285	5603	12799	9081	2592	12550	15483
14286	3349	3243	15577	3114	15578	12202	14303	15579	14302	14299	13927	15580	15581	14297	asru_1_15_o00450	14295	14296	153	12773	15582	15583	4839	13128	14902	12217	15584	10591	174	12988	7738
0	0	0	0	0	0	0	0	0	0	0	0	0	0	0	piku_1_227_hs00100	12155	21794	13983	0	0	0	0	0	0	0	0	0	0	0	0
0	0	0	0	0	0	0	13068	55827	11429	485	89481	89482	11966	89483	piku_1_96_cr00180	11681	14186	14410	12155	89484	43165	15520	0	0	0	0	0	0	0	0
89313	89712	2481	5778	1647	21608	89711	88887	356	13086	3978	15094	8204	10962	9462	pime_1_1_a12110	14955	285	12610	13807	13808	13809	4503	13367	13033	89292	13035	13036	89291	5385	2766
18766	13254	13603	10808	14133	408	15507	15507	19241	18878	12858	12857	3093	3093	88683	pime_1_4_d03240	14230	14231	13673	12773	153	12784	90092	14719	88645	15509	90093	88642	111	1168	17184
13821	14233	7618	1571	5354	14998	14512	14084	14135	13981	90253	13654	90252	12712	21590	pime_1_5_e05800	12155	21794	23024	14074	763	90251	14905	9586	9157	21593	17764	15	3099	1078	15029
8758	89644	89643	89642	14546	14545	88829	14774	89641	12867	6856	89640	14498	88826	22423	pime_1_1_a07690	7790	14619	89076	13795	89639	89078	12999	19245	89638	11810	89637	15079	89636	10316	2496
69744	69745	69746	69747	12518	2493	69748	20068	12166	14833	14830	14083	69749	6706	69750	kopa_1_2_b10040	8151	15268	20072	69751	69752	15038	11068	69753	4469	6200	15921	15045	15044	1149	15042
69744	69745	69746	69747	12518	2493	69748	20068	12166	14833	14830	14083	69749	6706	69750	kopa_2_7_g00500	8151	15268	20072	69751	69752	15038	11068	69753	4469	6200	15921	15045	15044	1149	15042
69745	69746	69747	12518	2493	69748	20068	12166	14833	14833	14830	14083	69749	6706	69750	kopa_3_2_b02790	8151	15268	20072	69751	69752	15038	11068	69753	4469	6200	15921	15045	15044	1149	15042
