# Supplementary figures and images for: Molecular evolution of the members of the Snq2/Pdr18 subfamily of Pdr transporters in the Hemiascomycete yeasts
Source: FEMS Yeast Res. 2025 May 27;25:foaf026. doi: 10.1093/femsyr/foaf026 (PMC12202755; doi:10.1093/femsyr/foaf026)

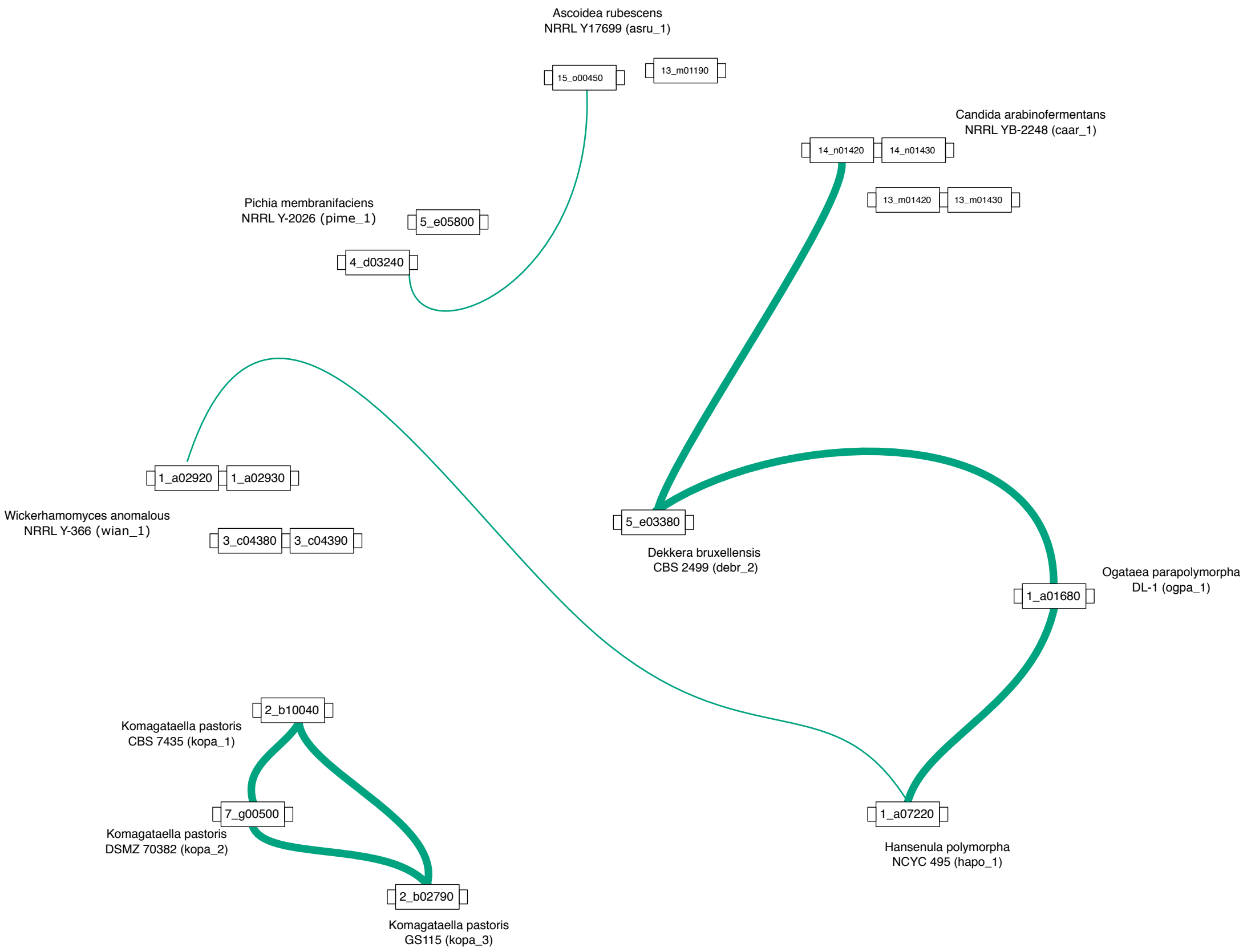

Supplement: foaf026_Supplemental_Files [file foaf026_supplemental_files.zip › Figure A11_Supplementary Data.pdf]

A)

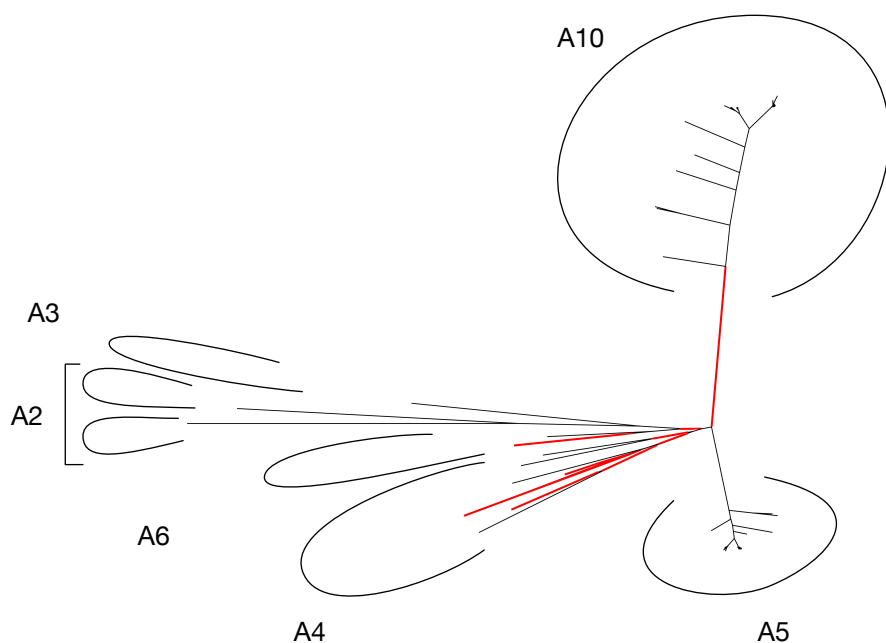

B)

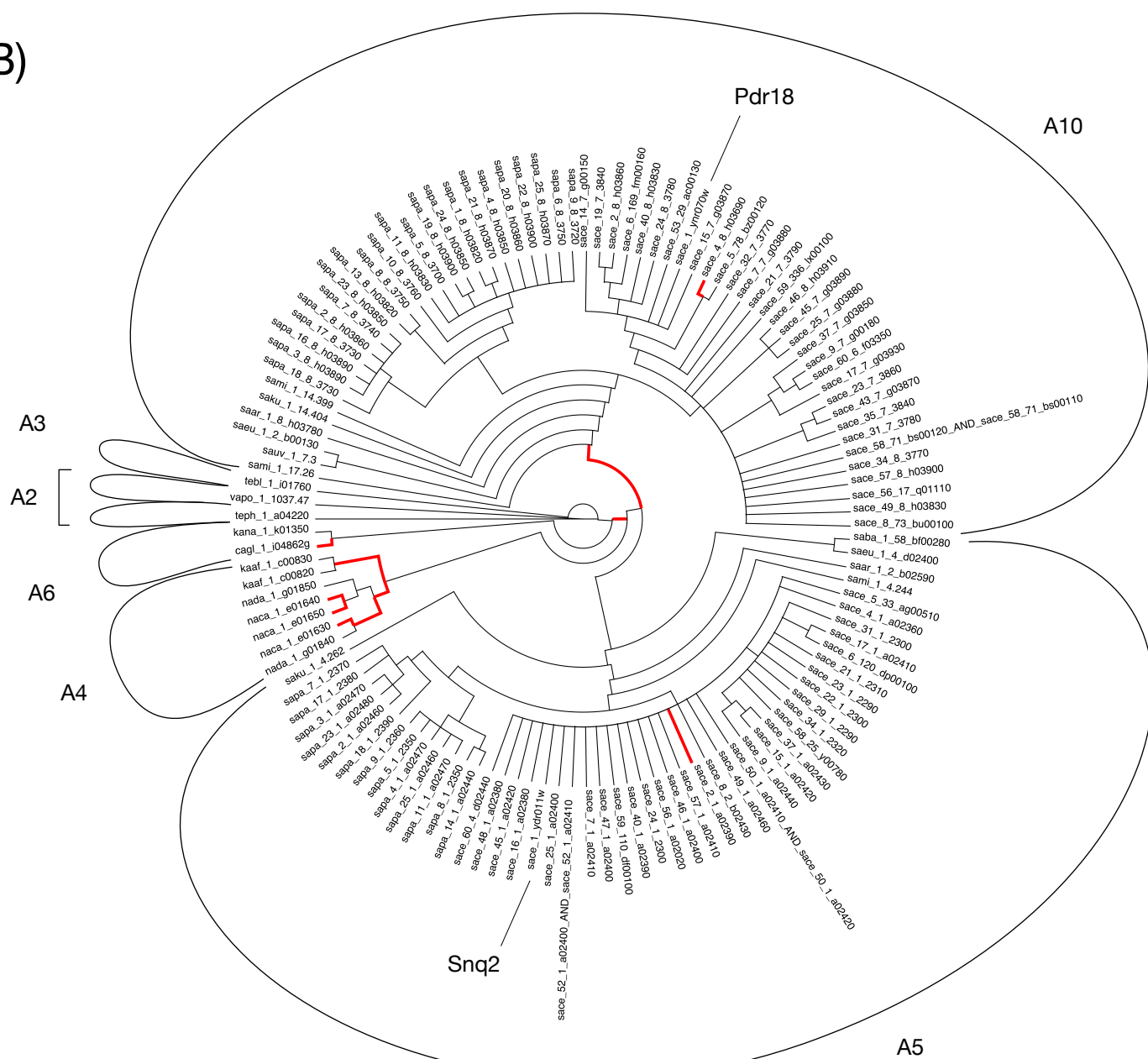

Supplement: foaf026_Supplemental_Files [file foaf026_supplemental_files.zip › Figure A19_Supplementary Data.pdf]

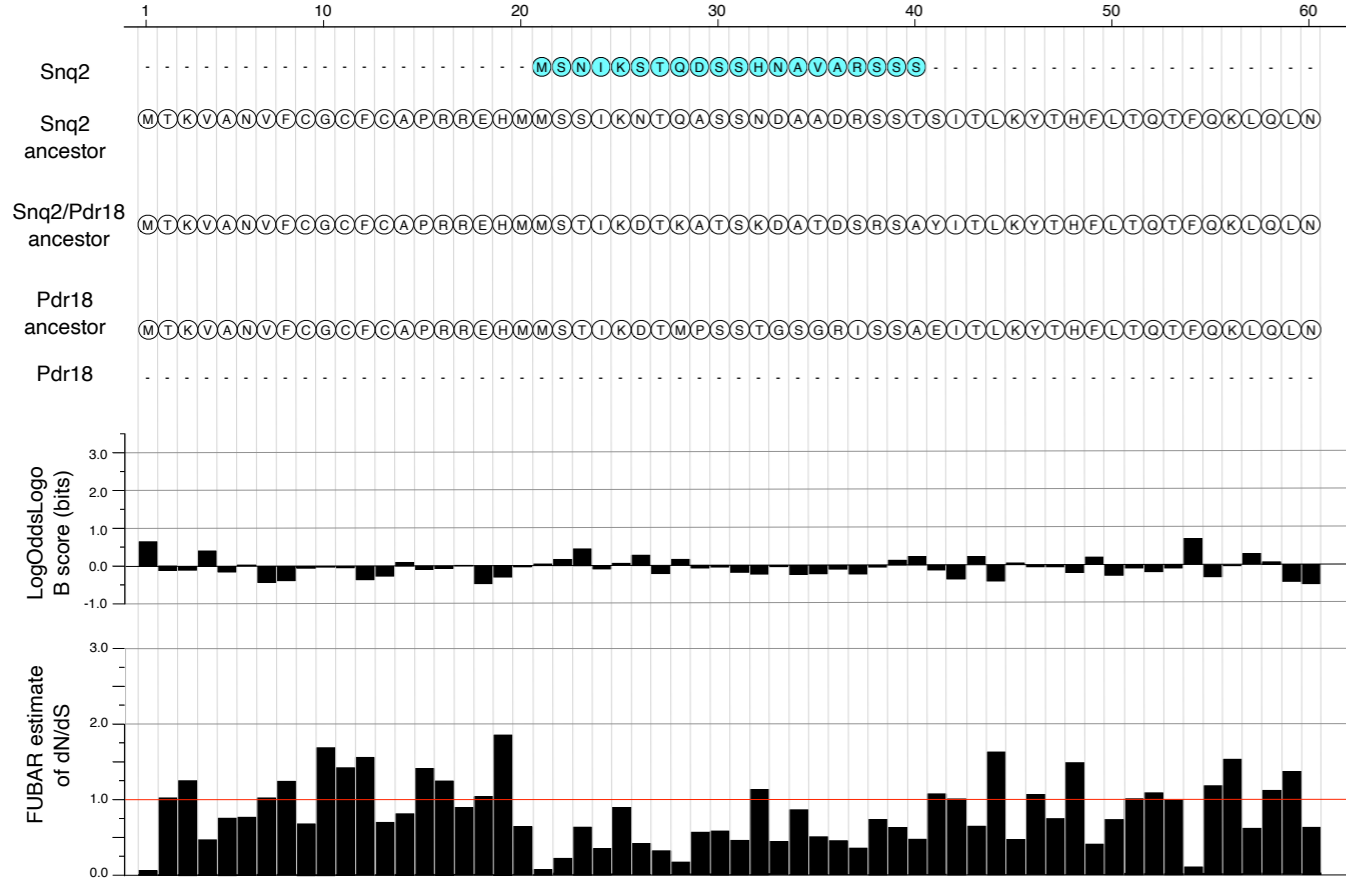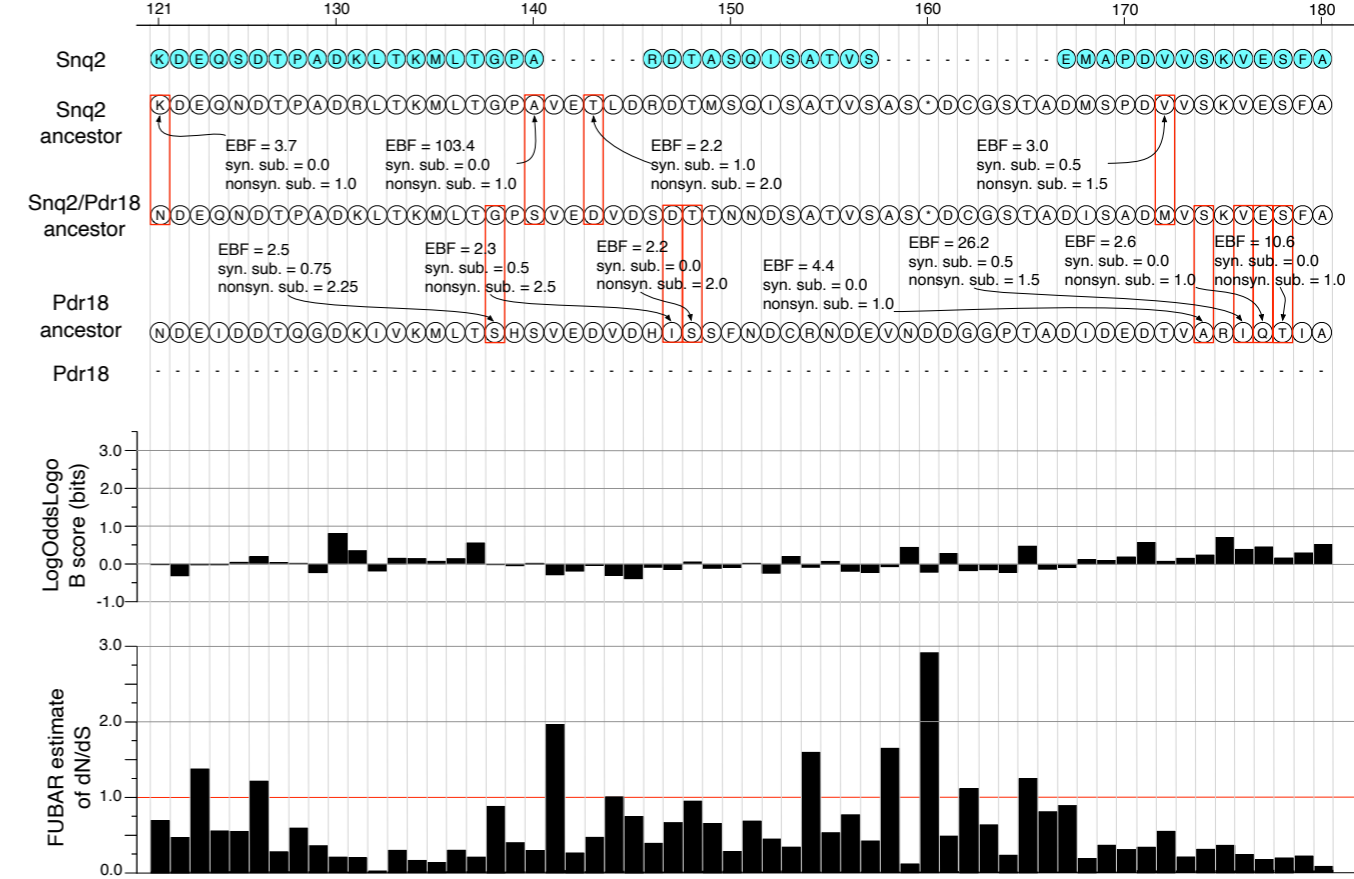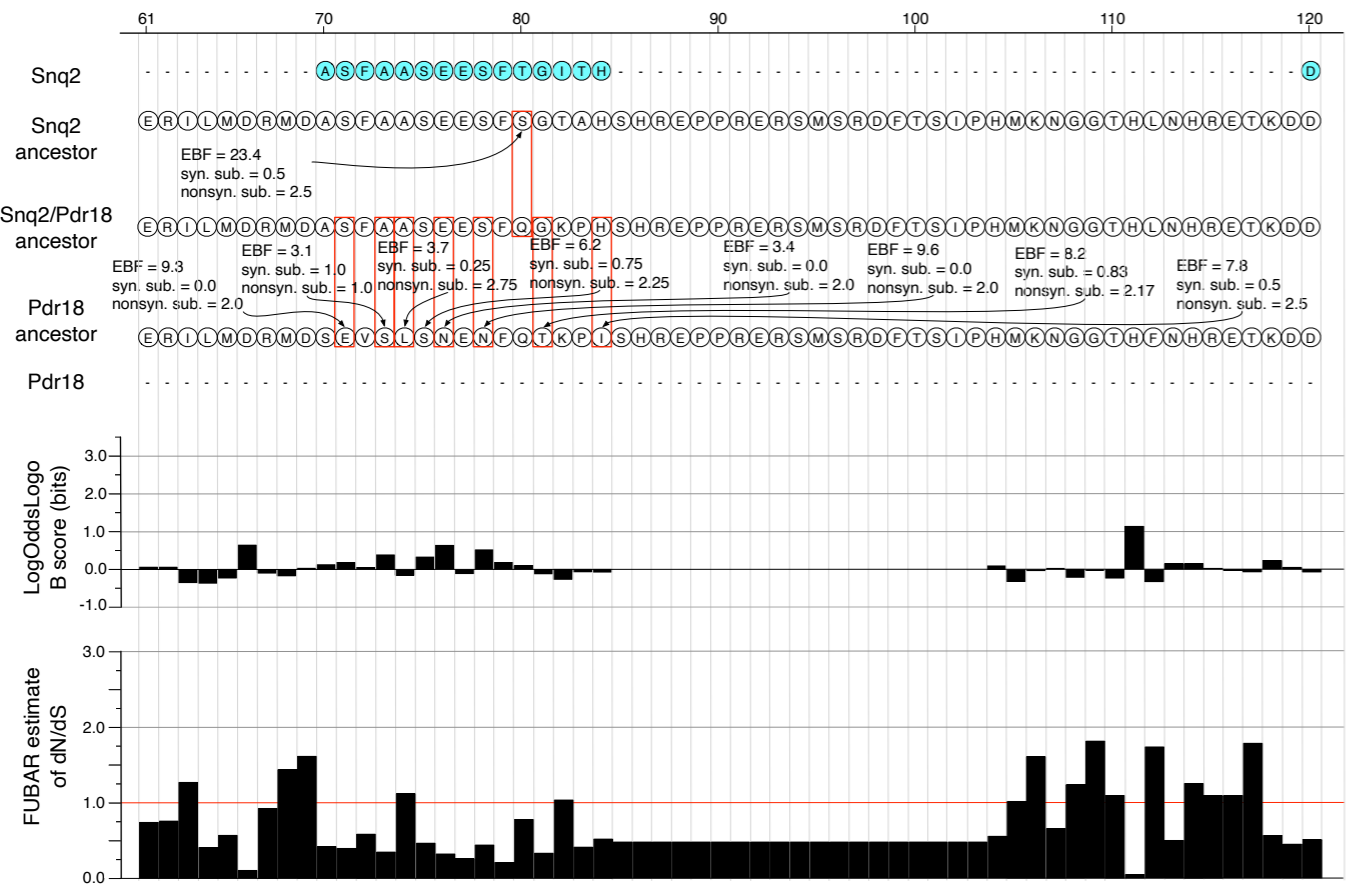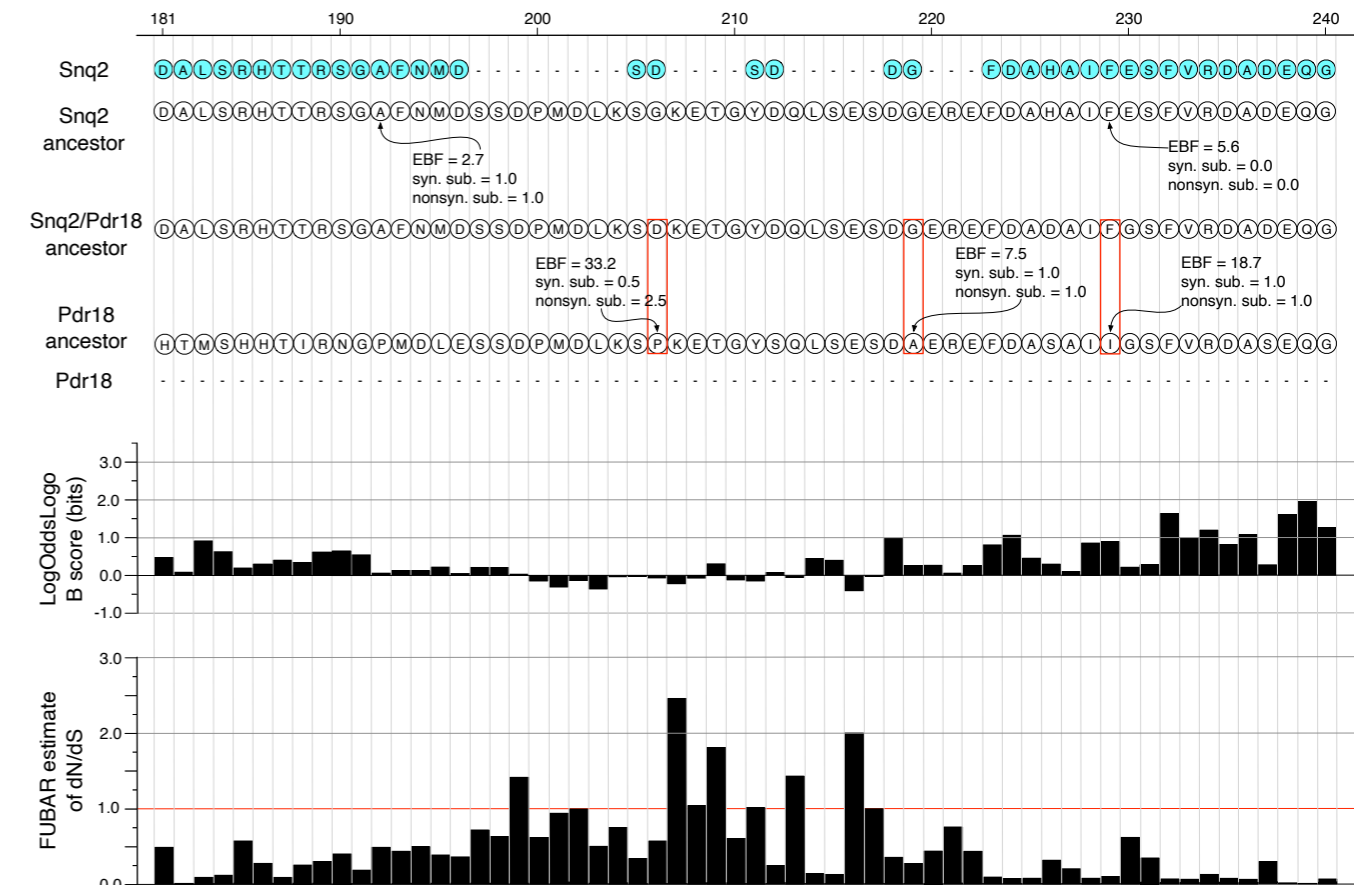

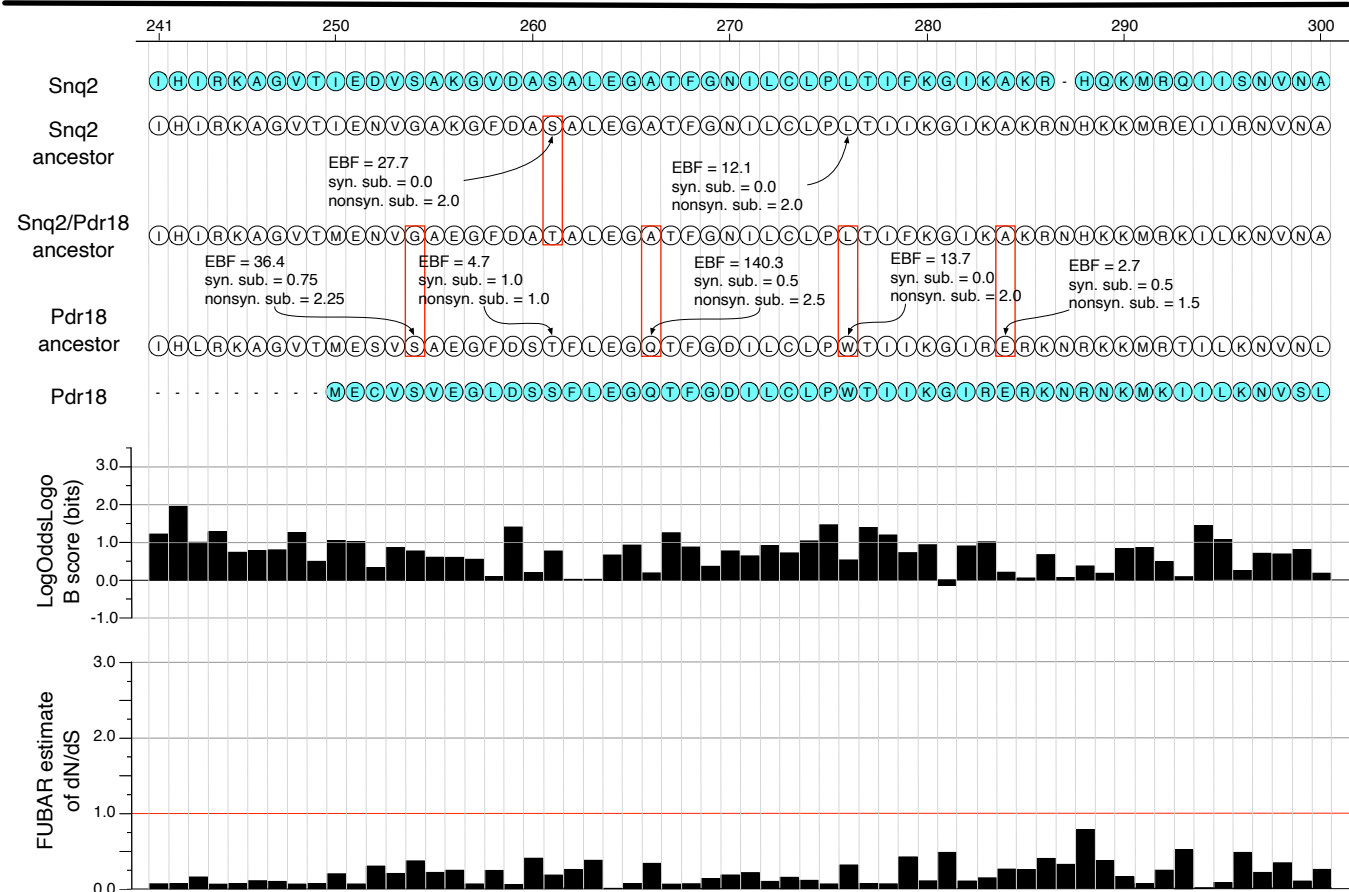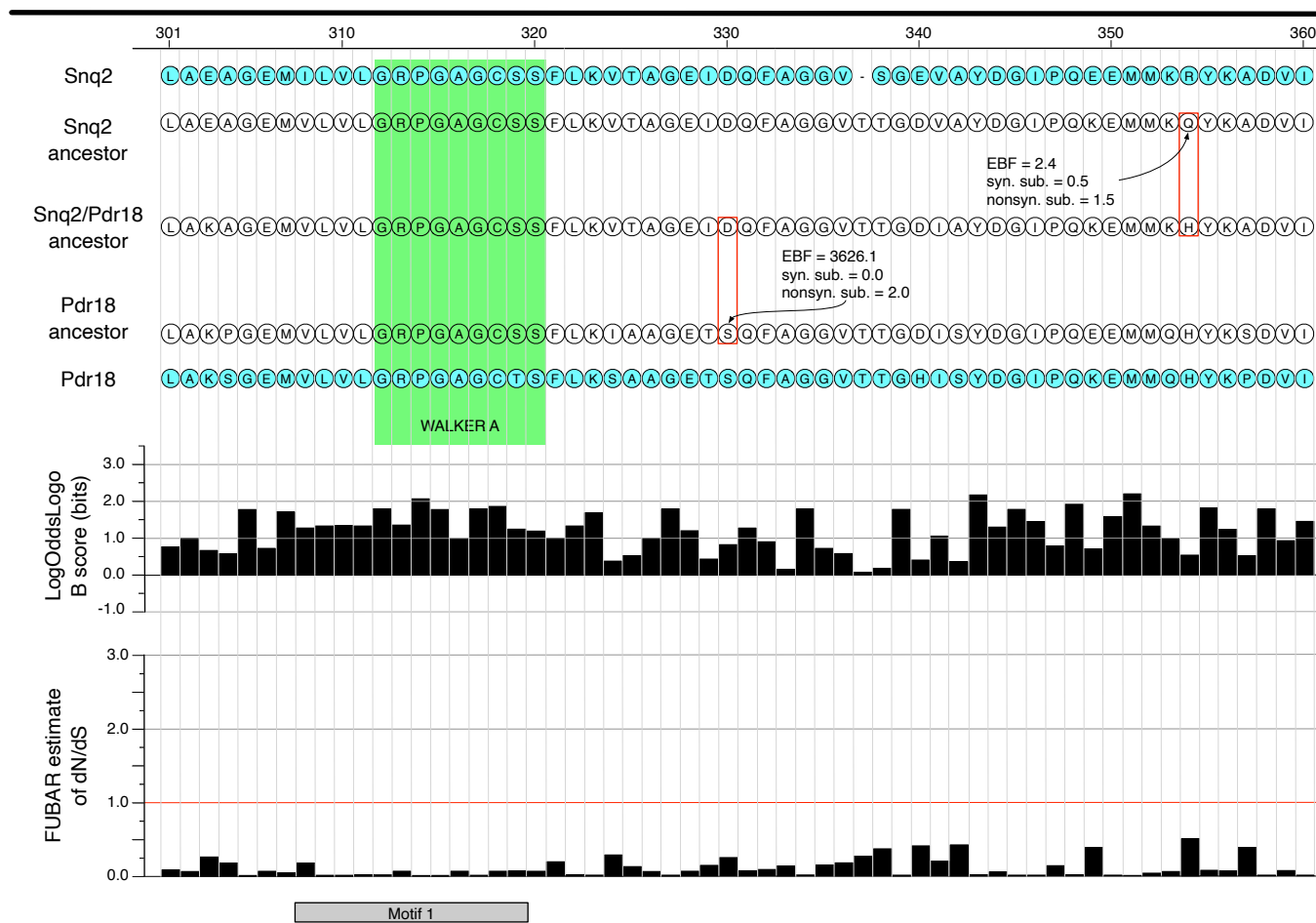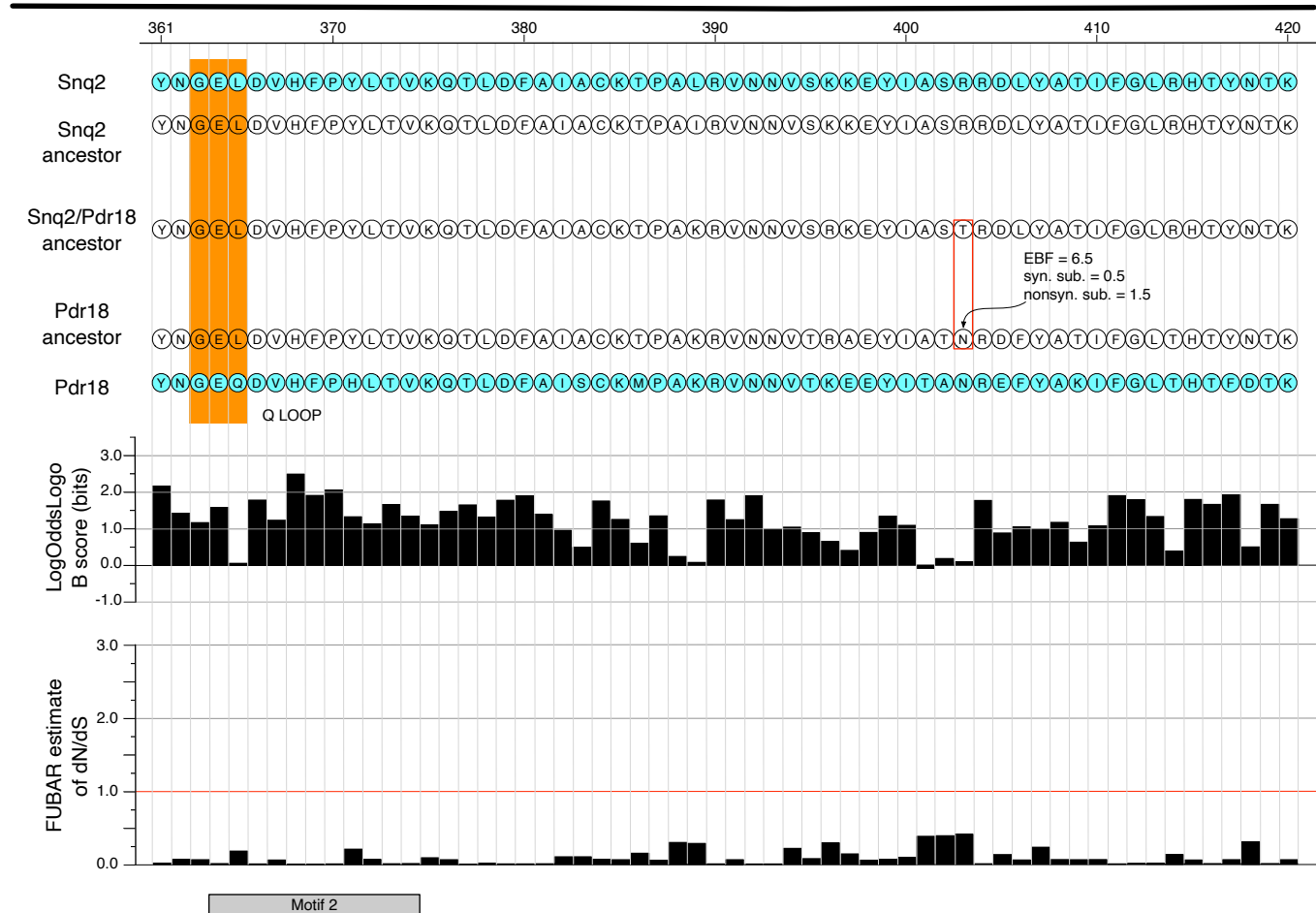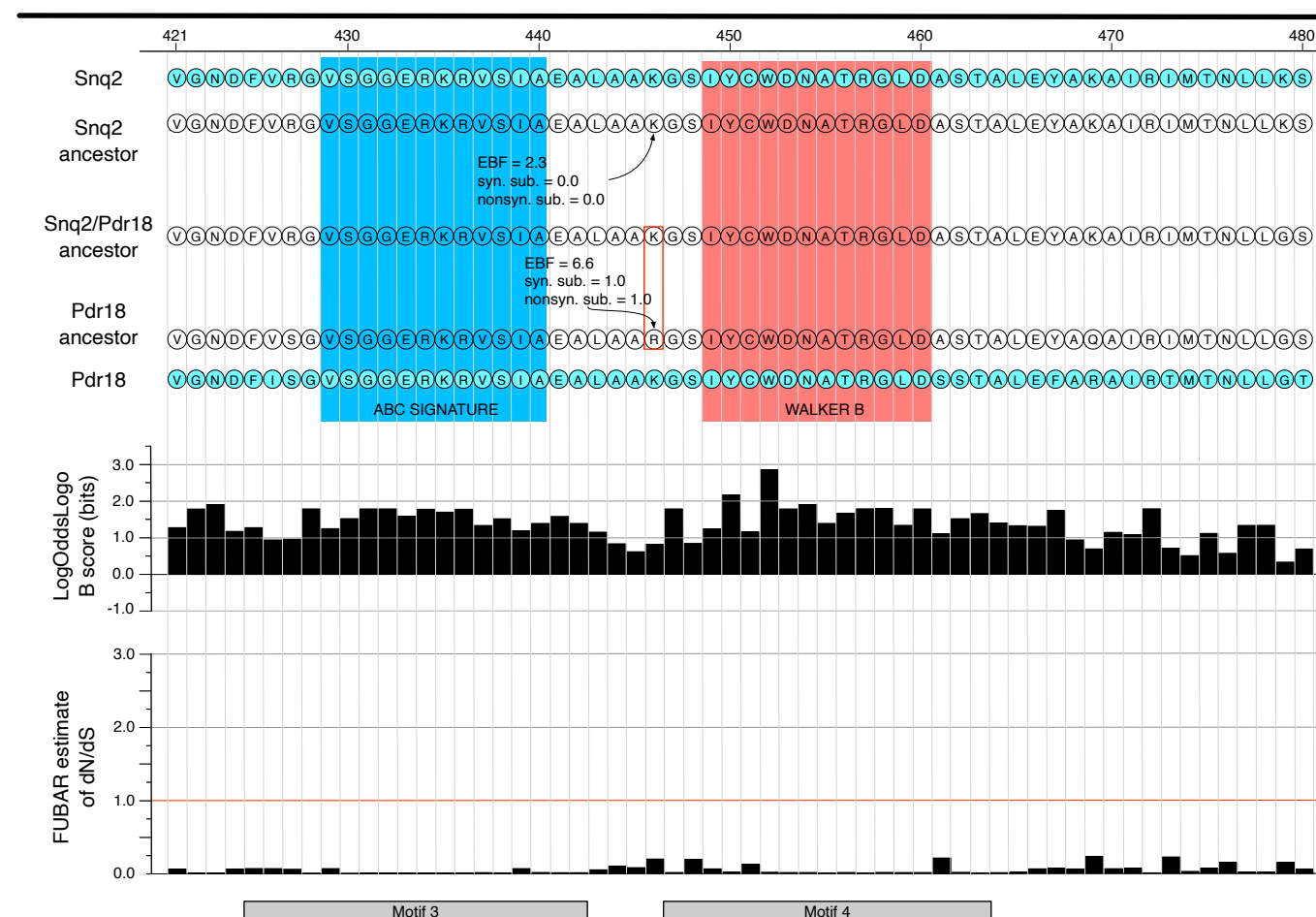

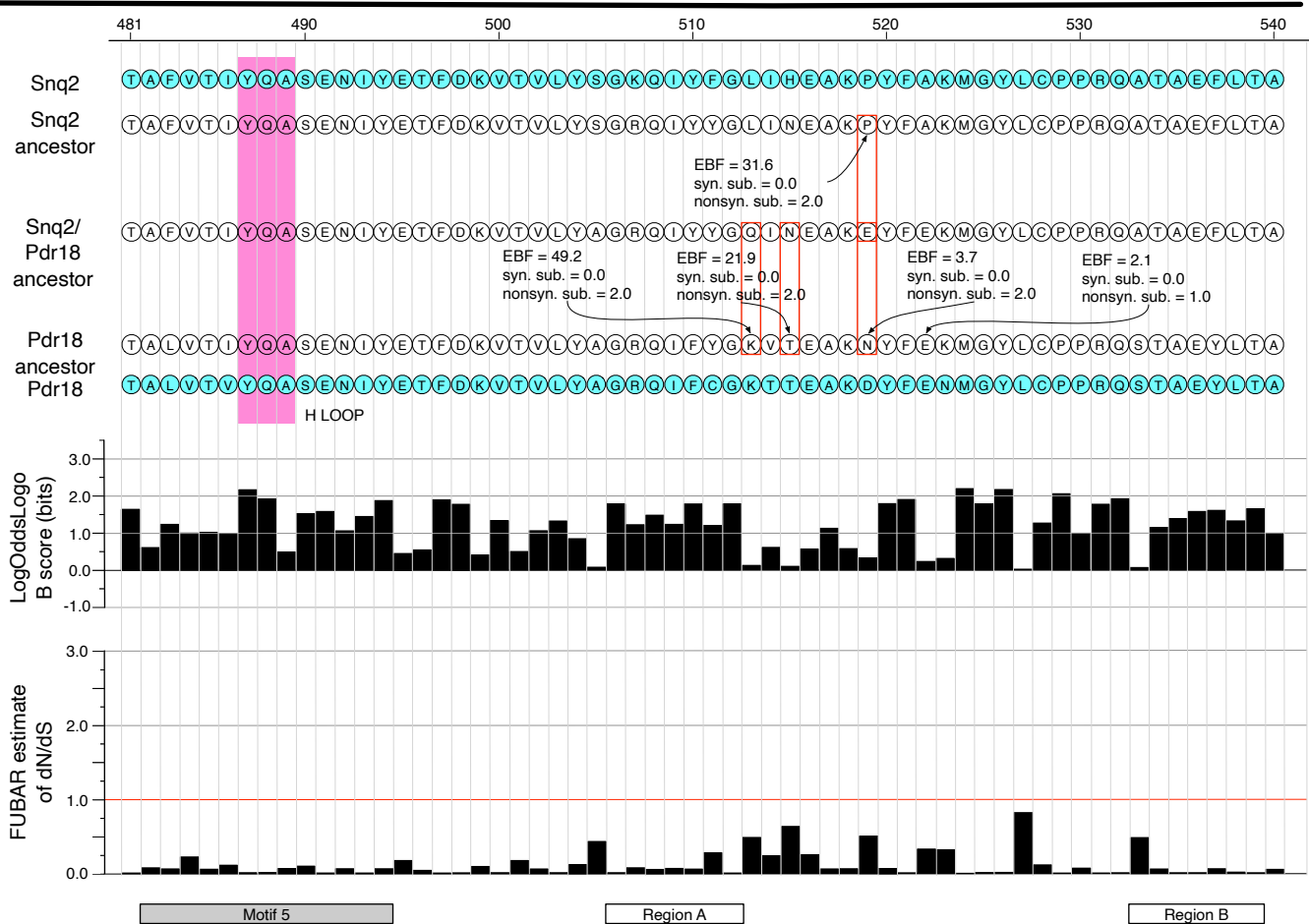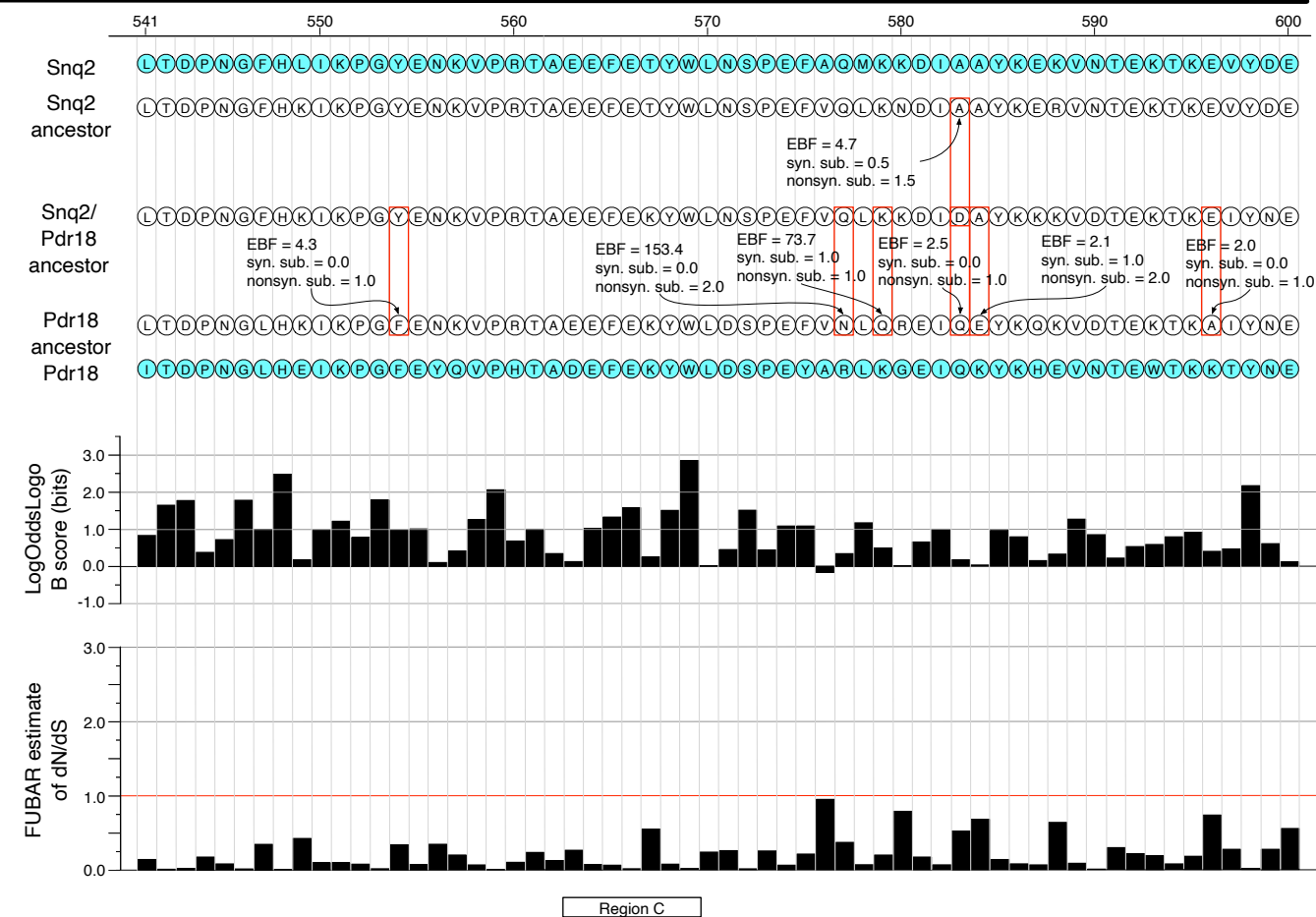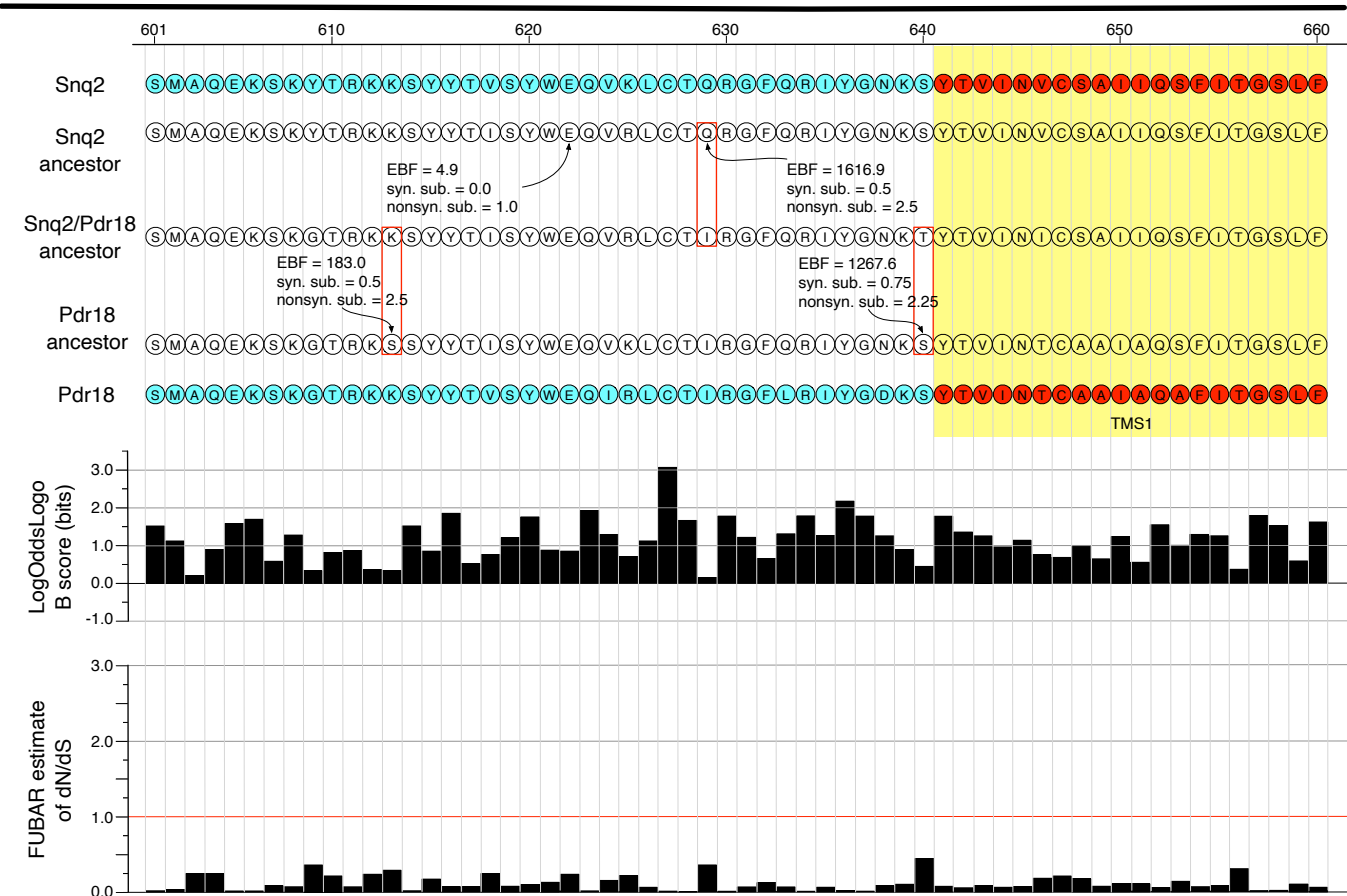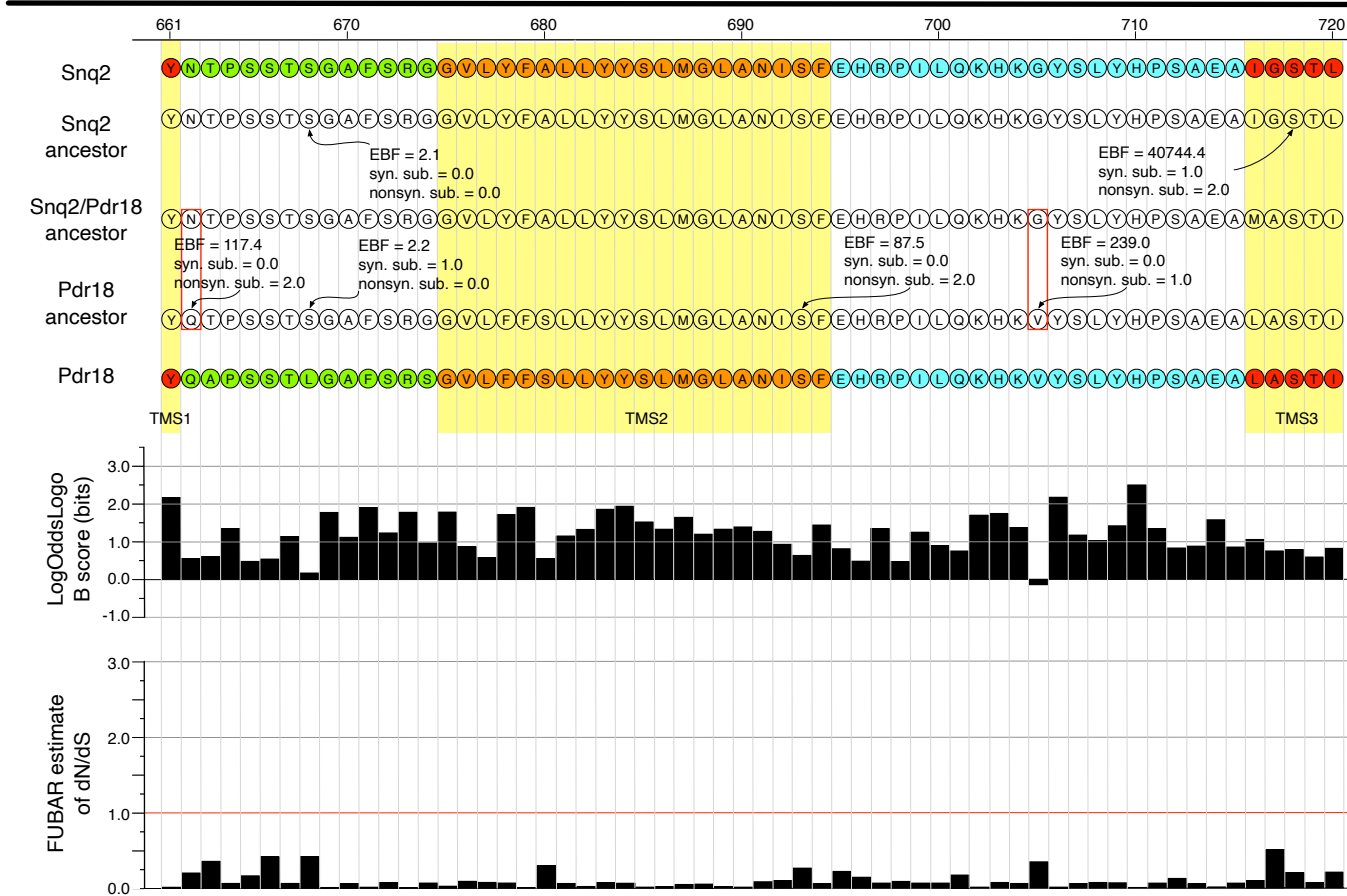

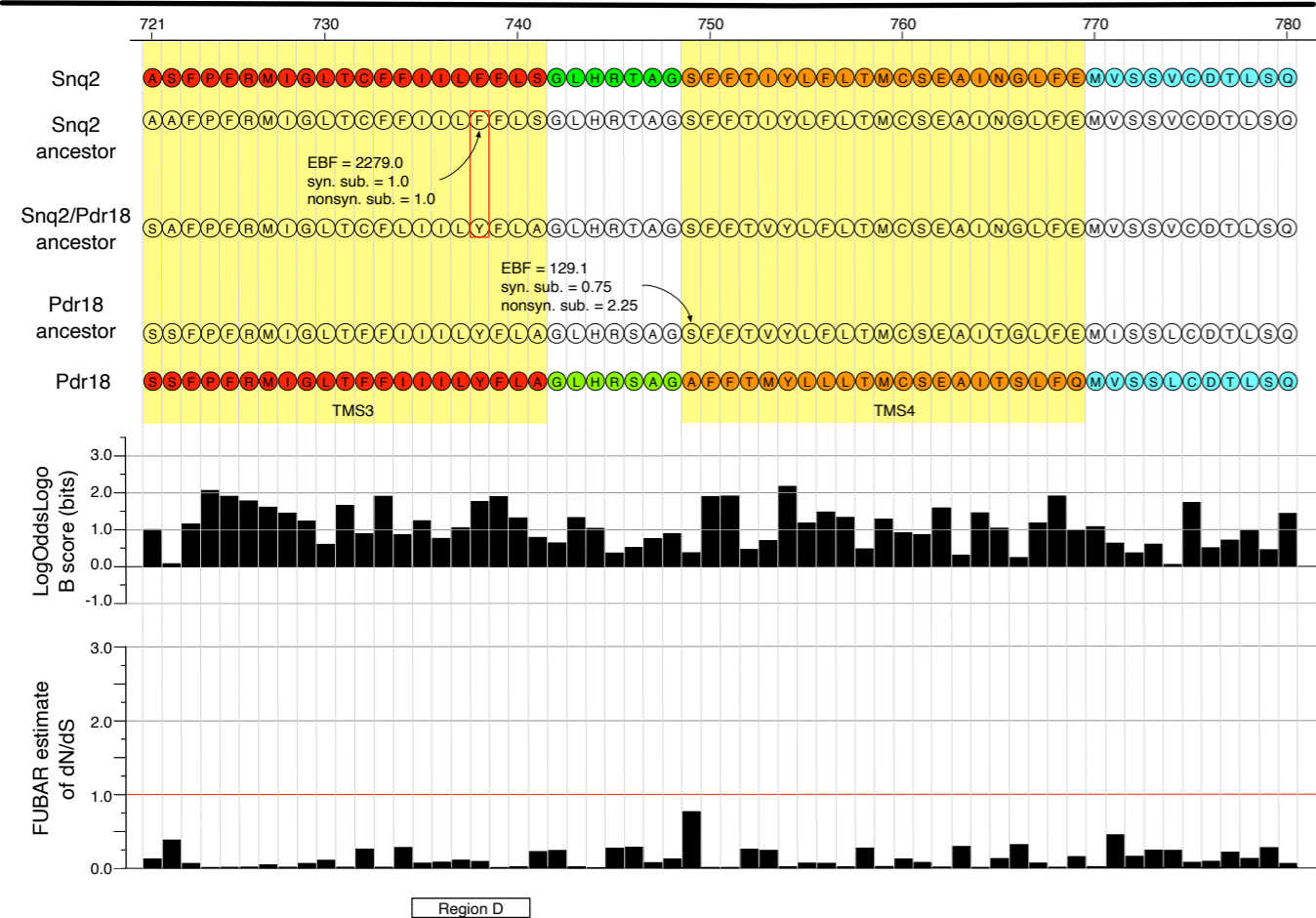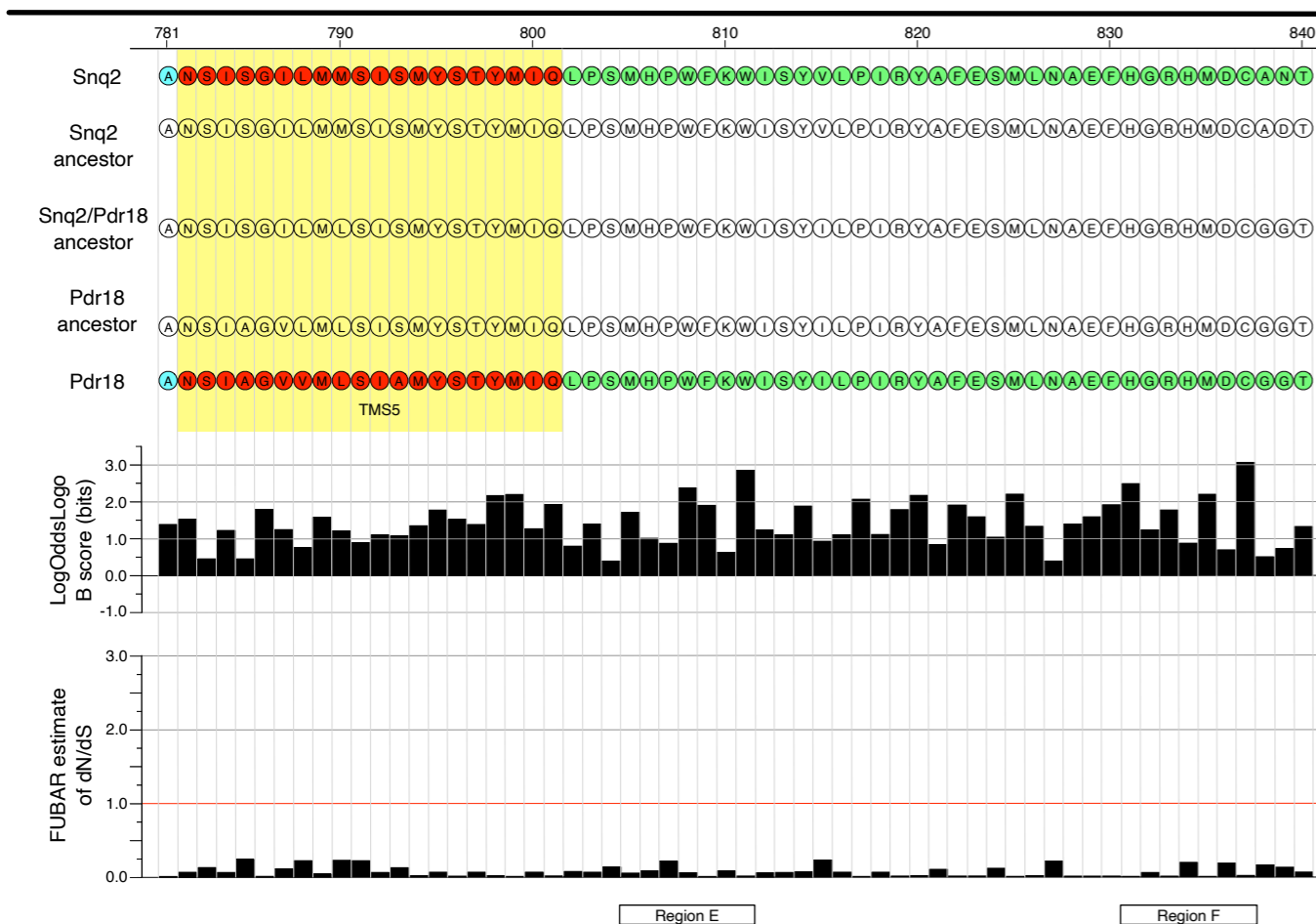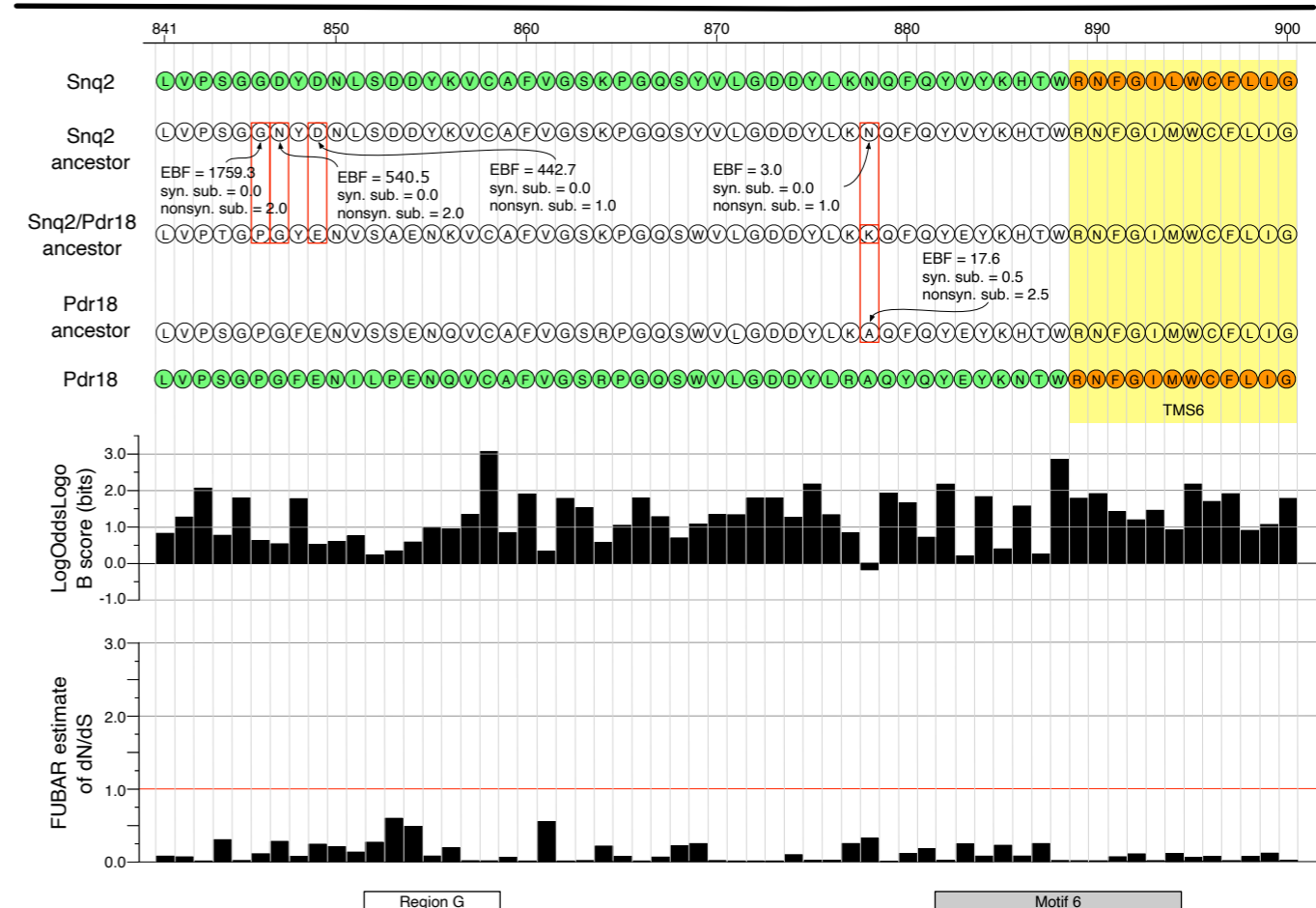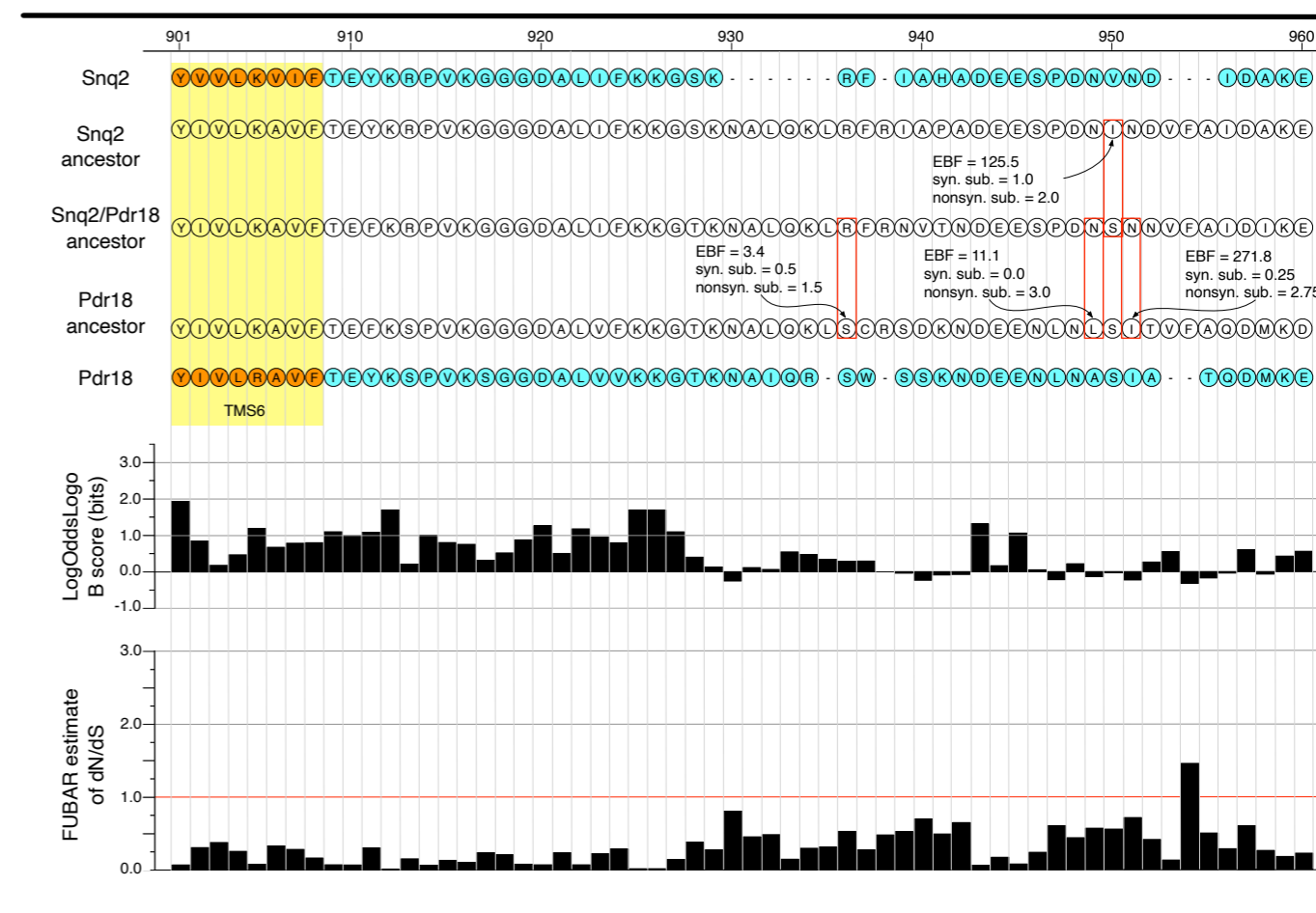

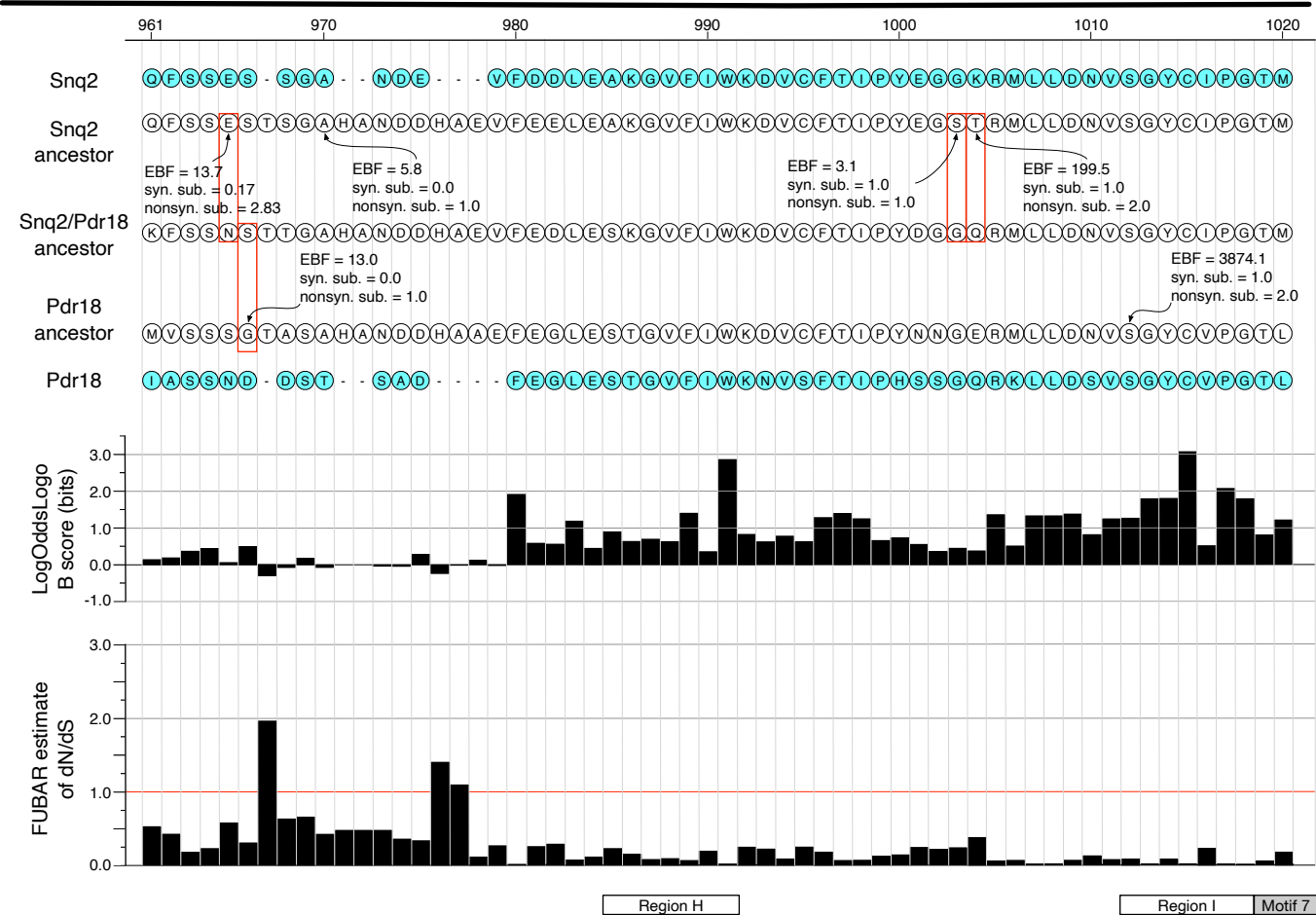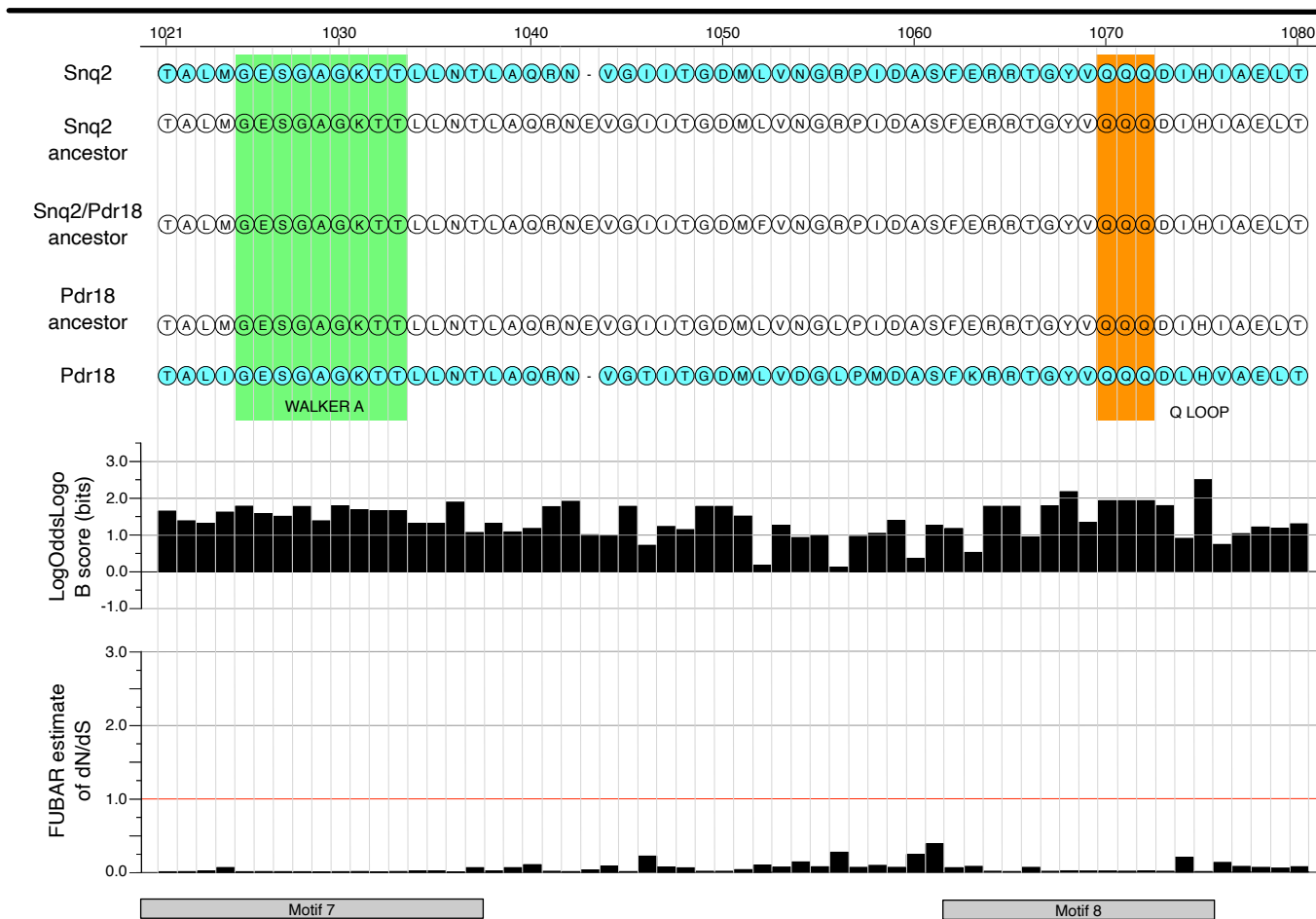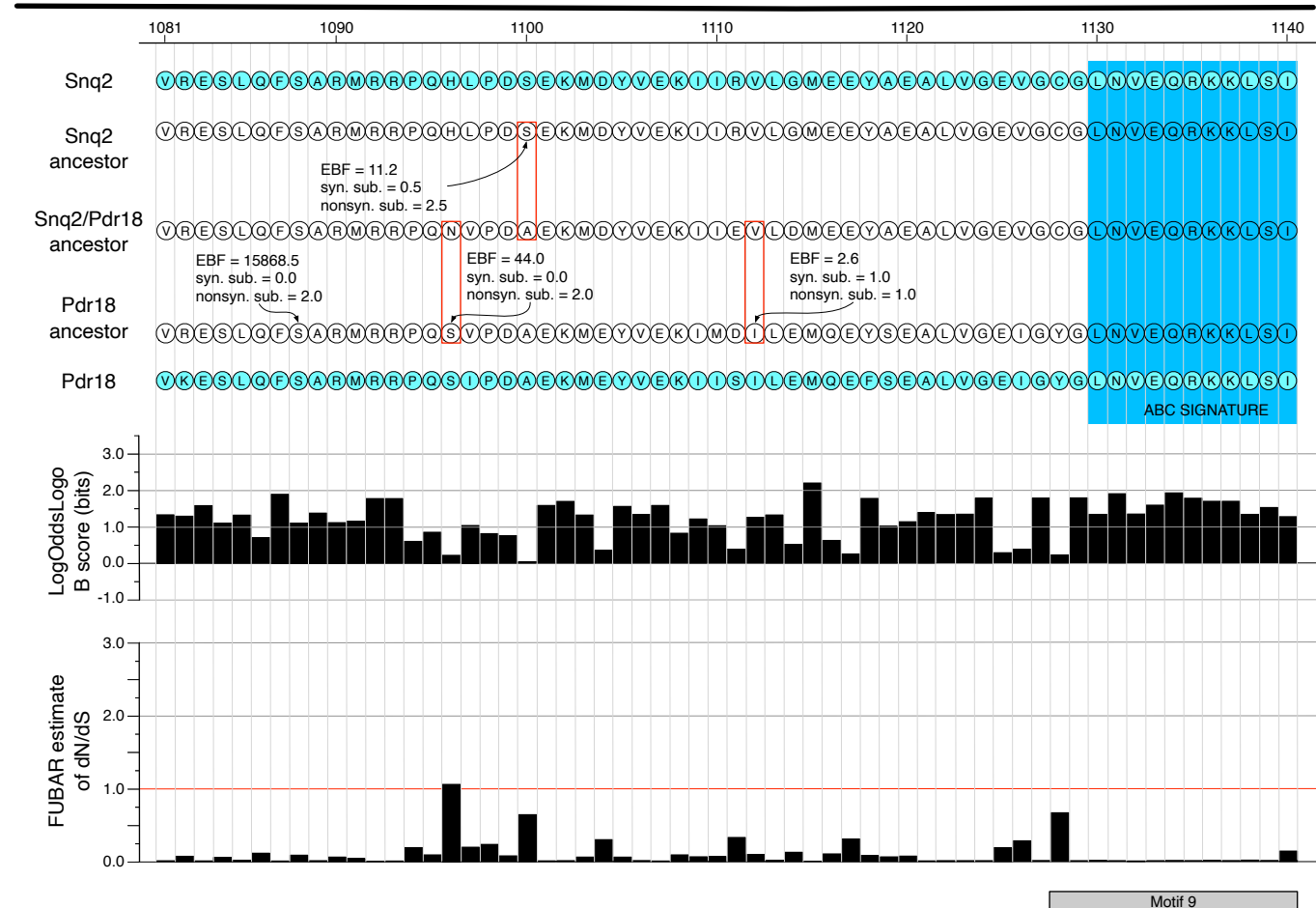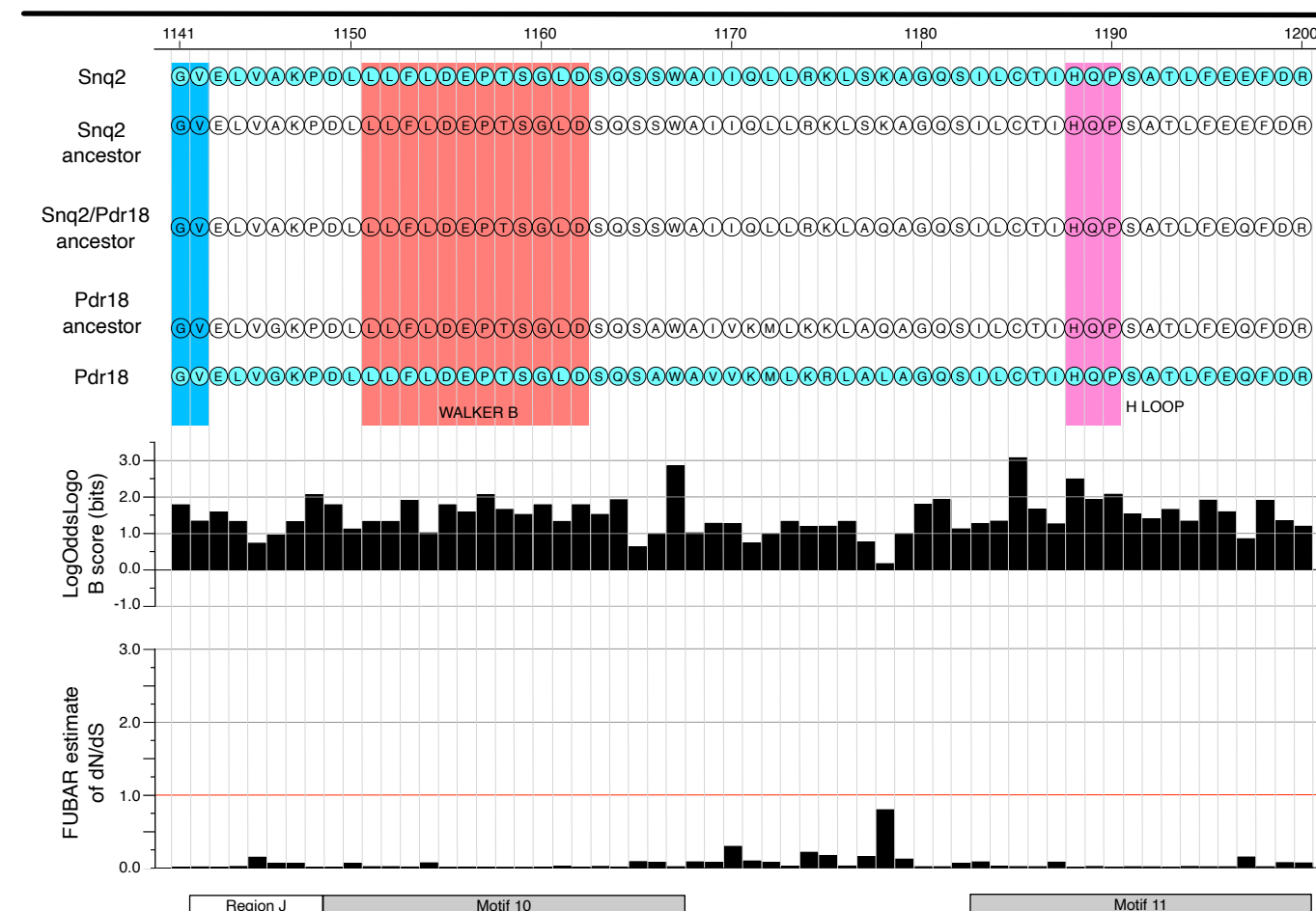

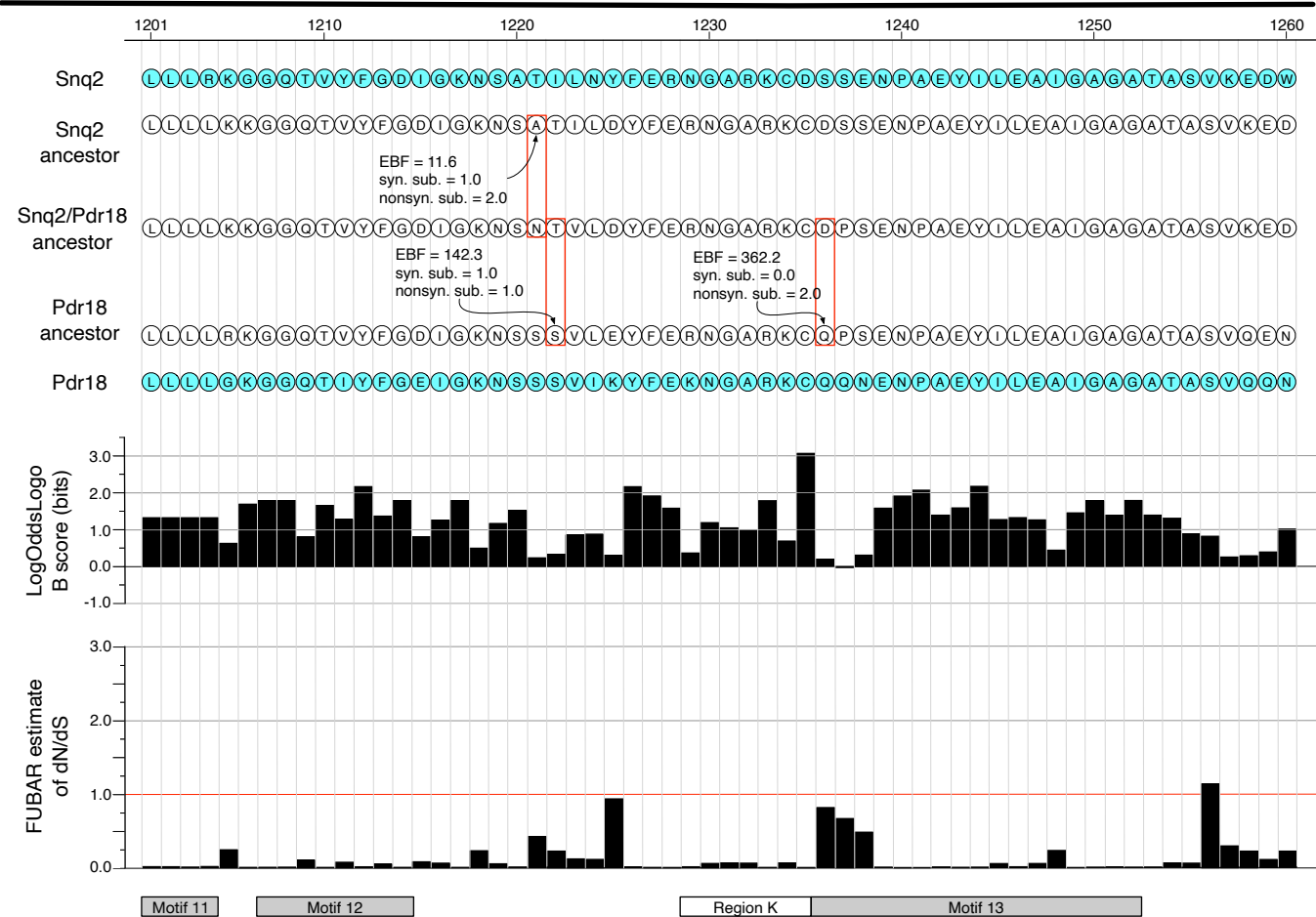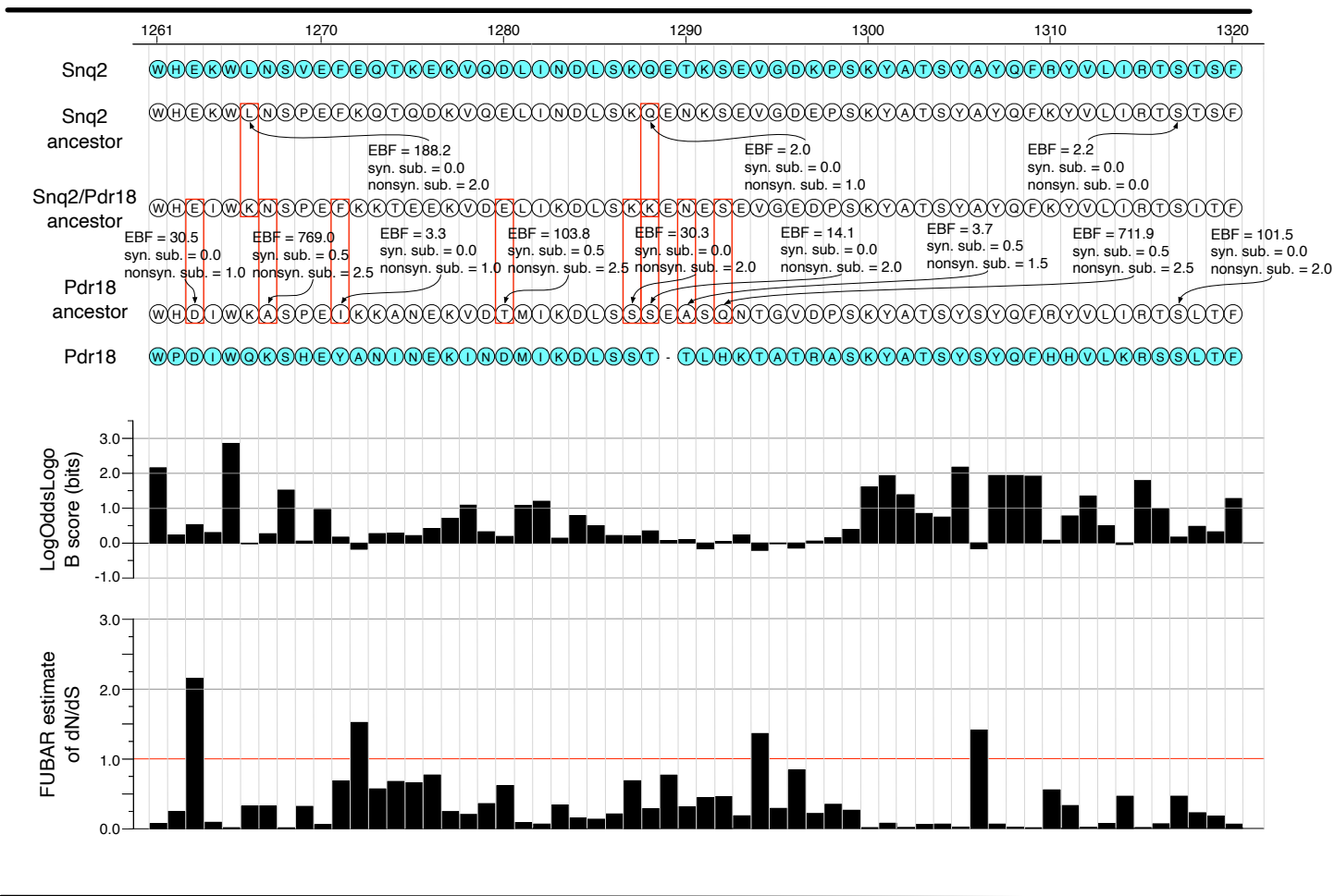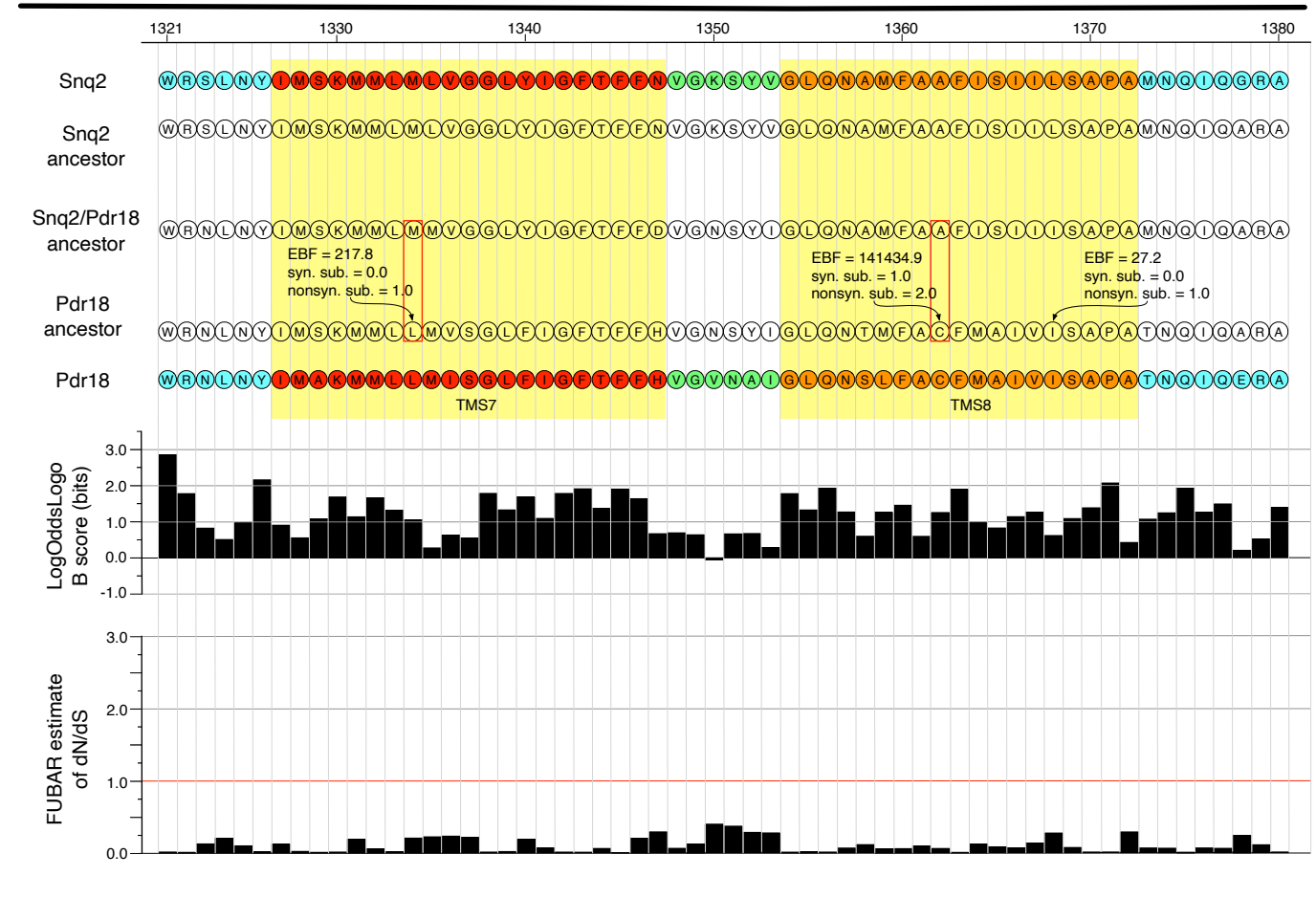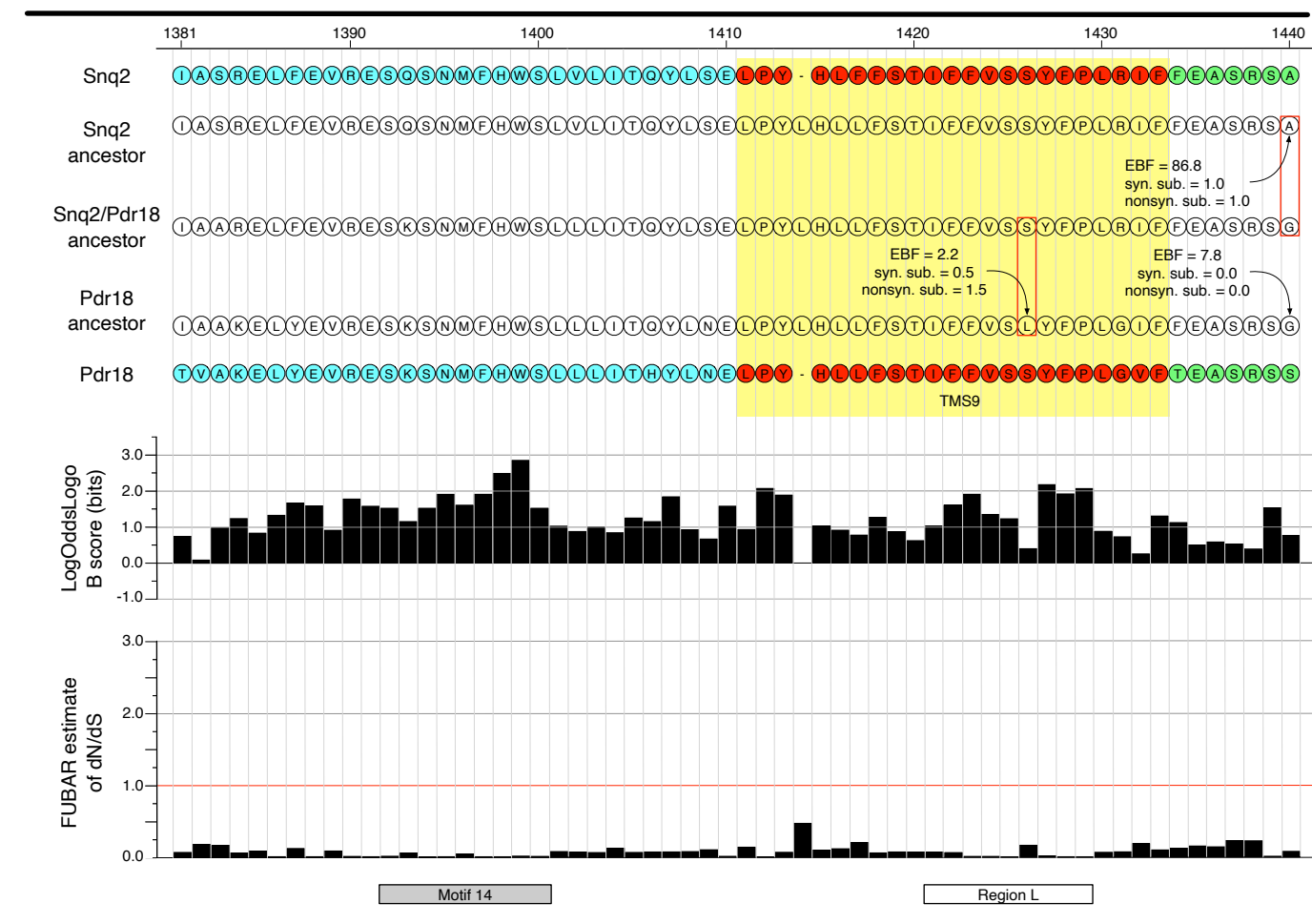

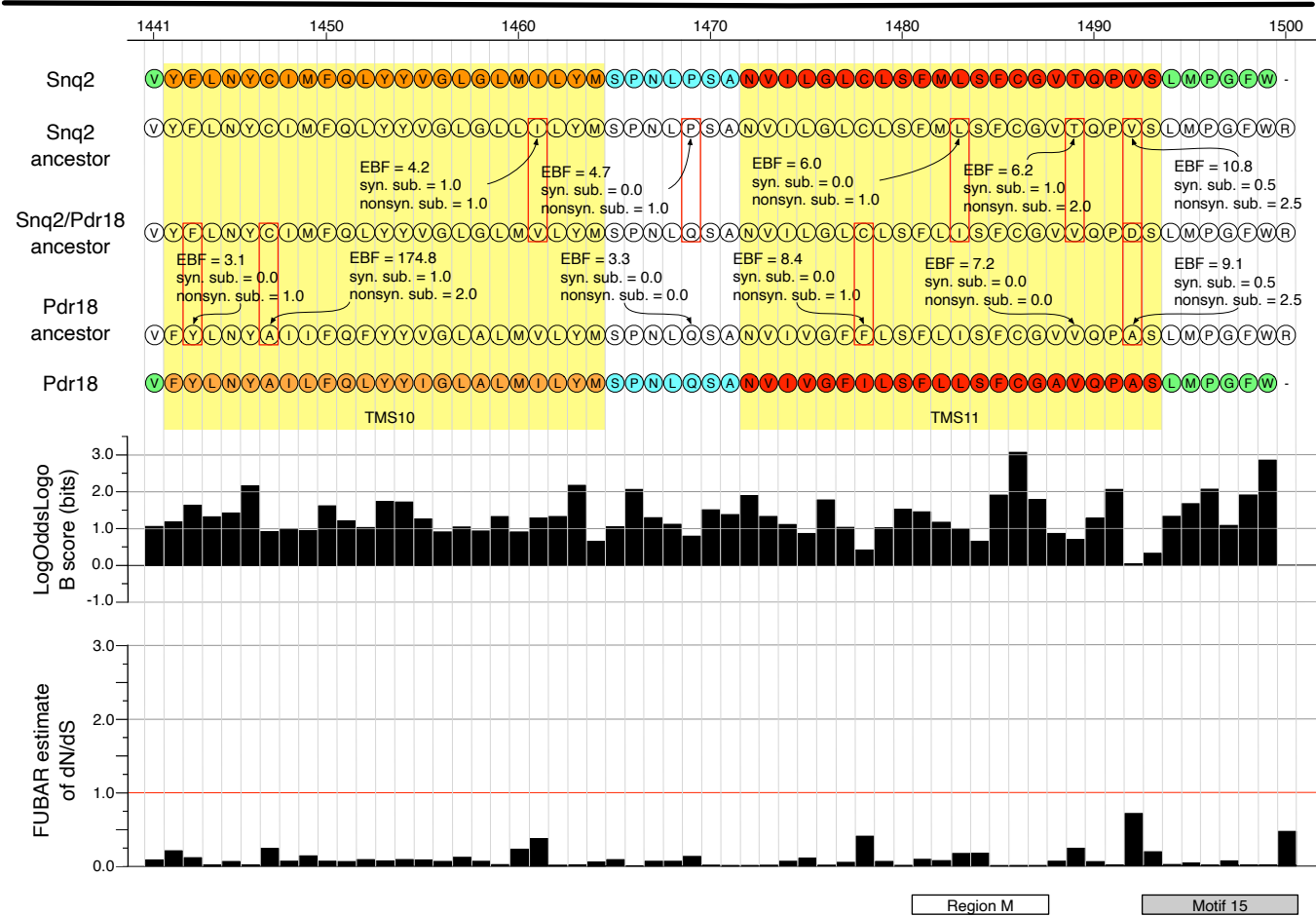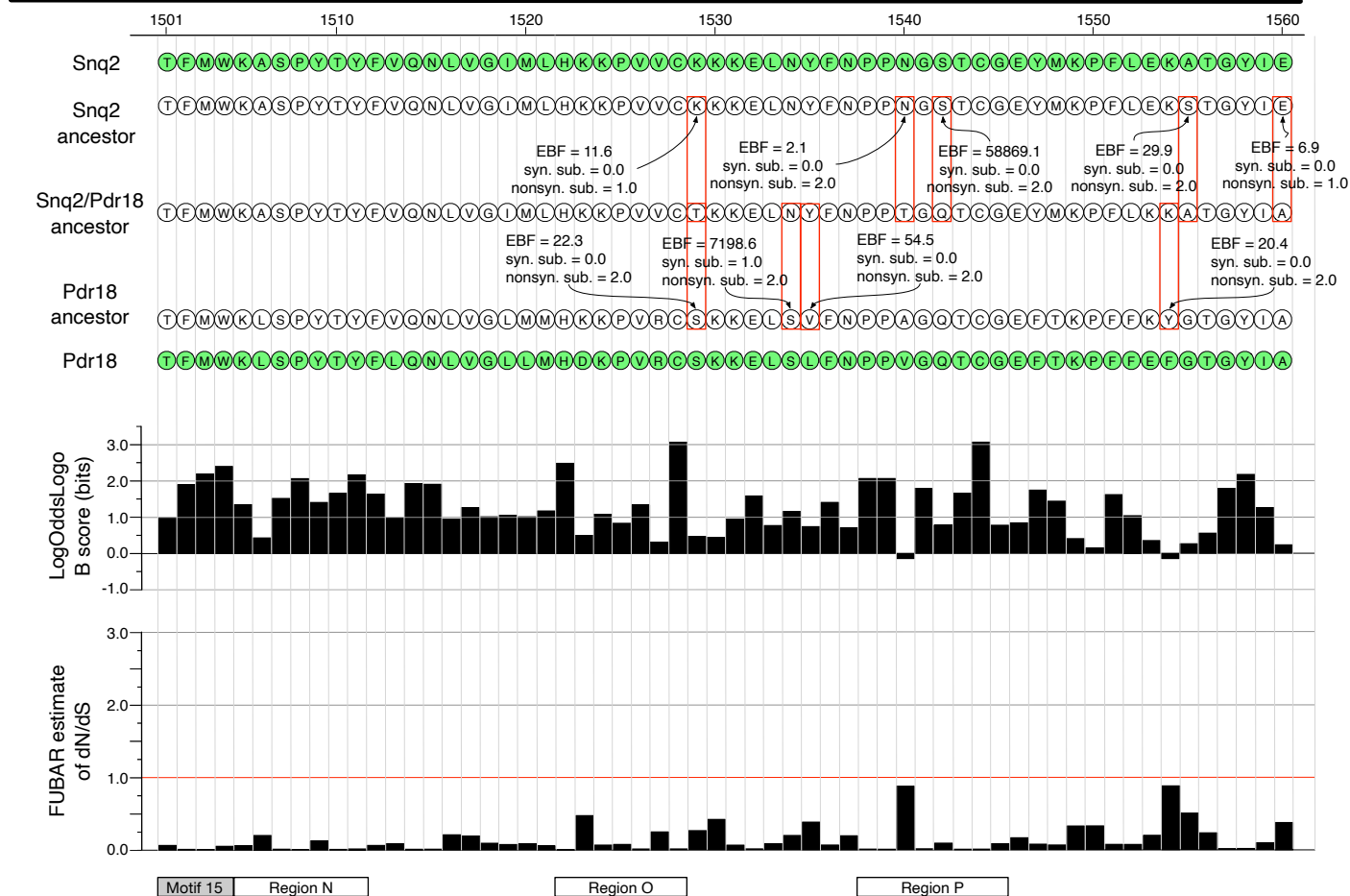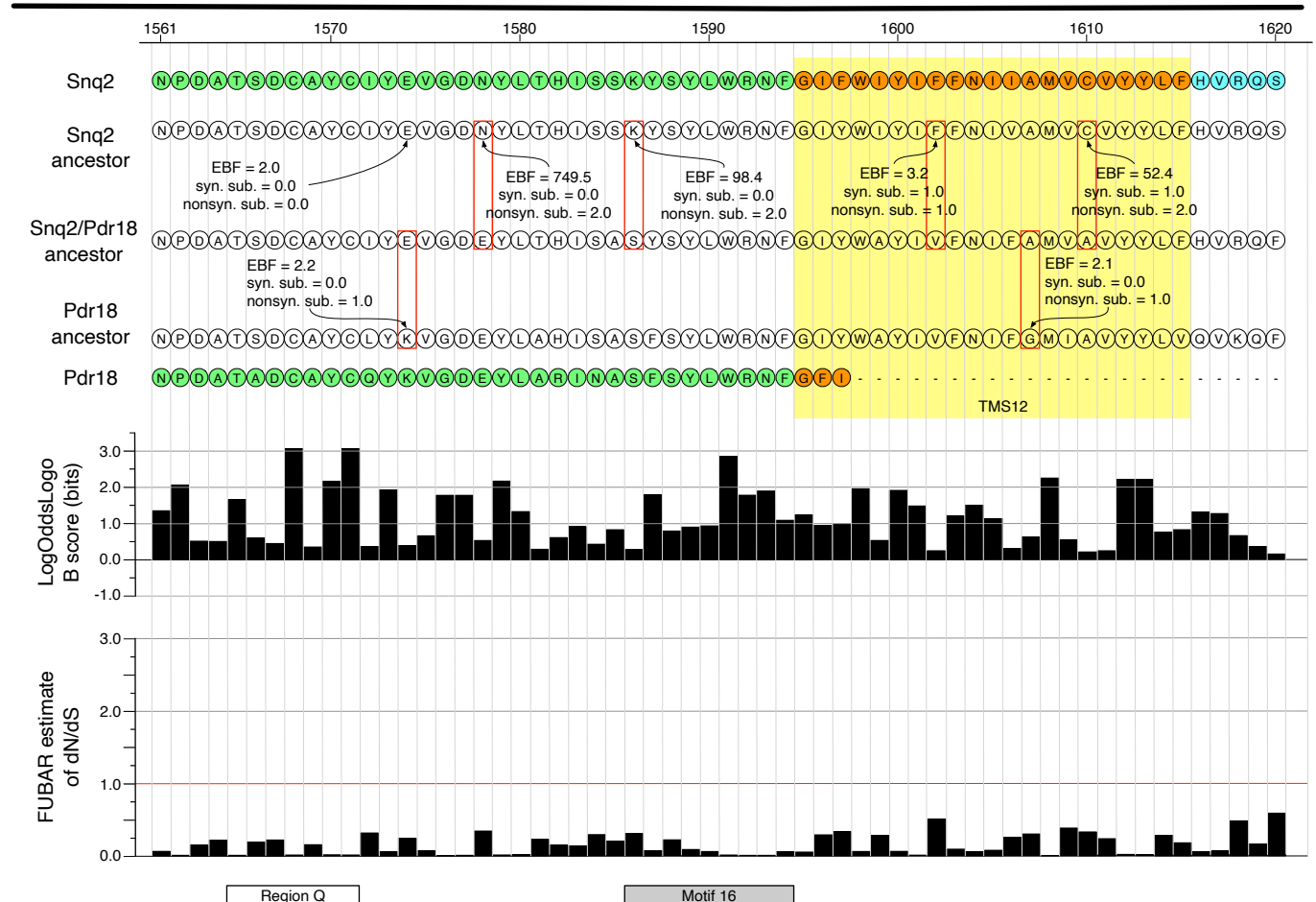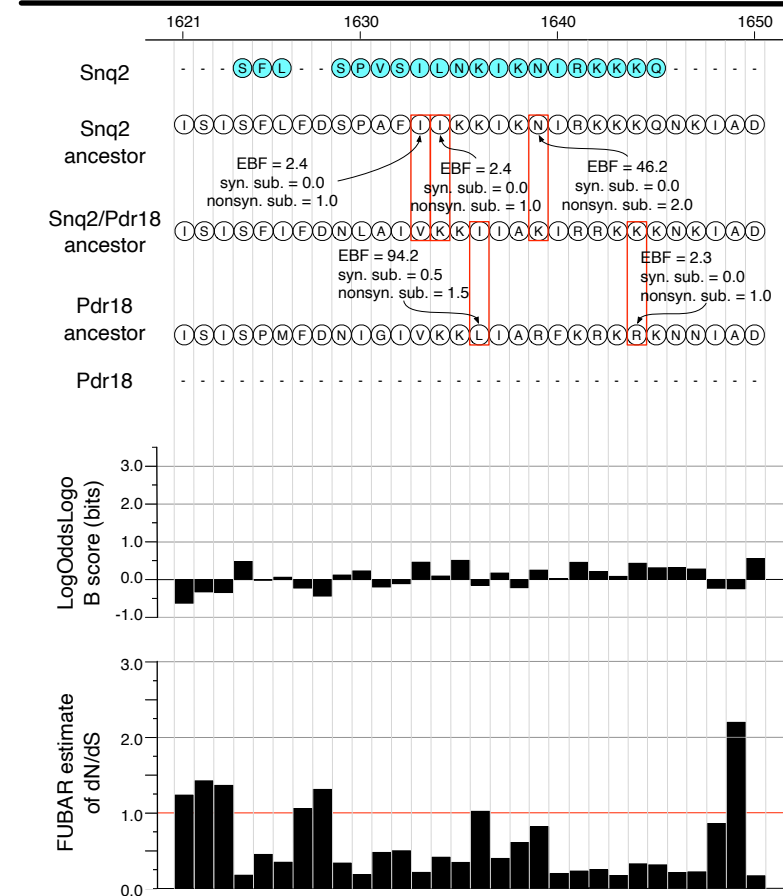

Supplement: foaf026_Supplemental_Files [file foaf026_supplemental_files.zip › Figure A30_Supplementary_Data.pdf]

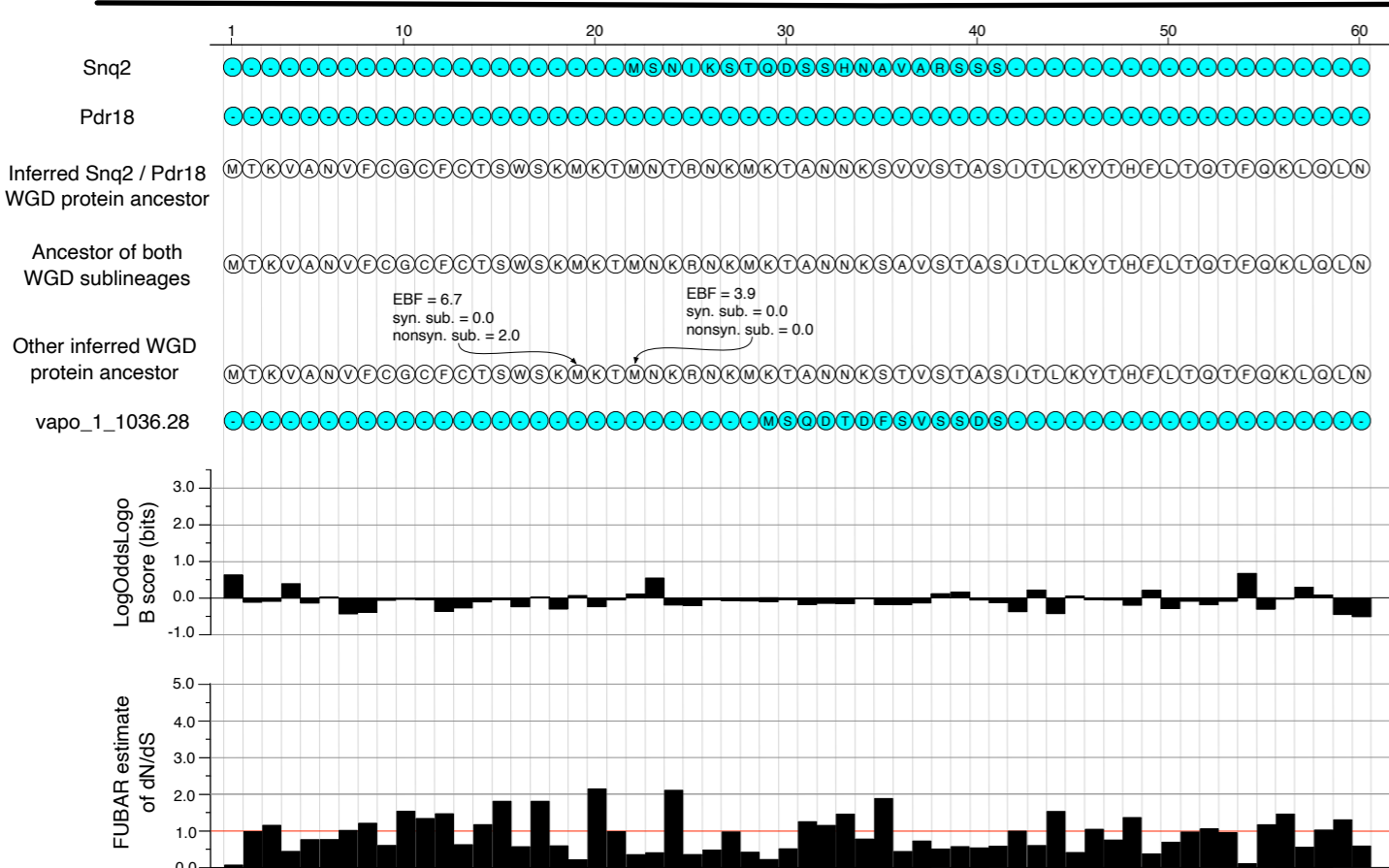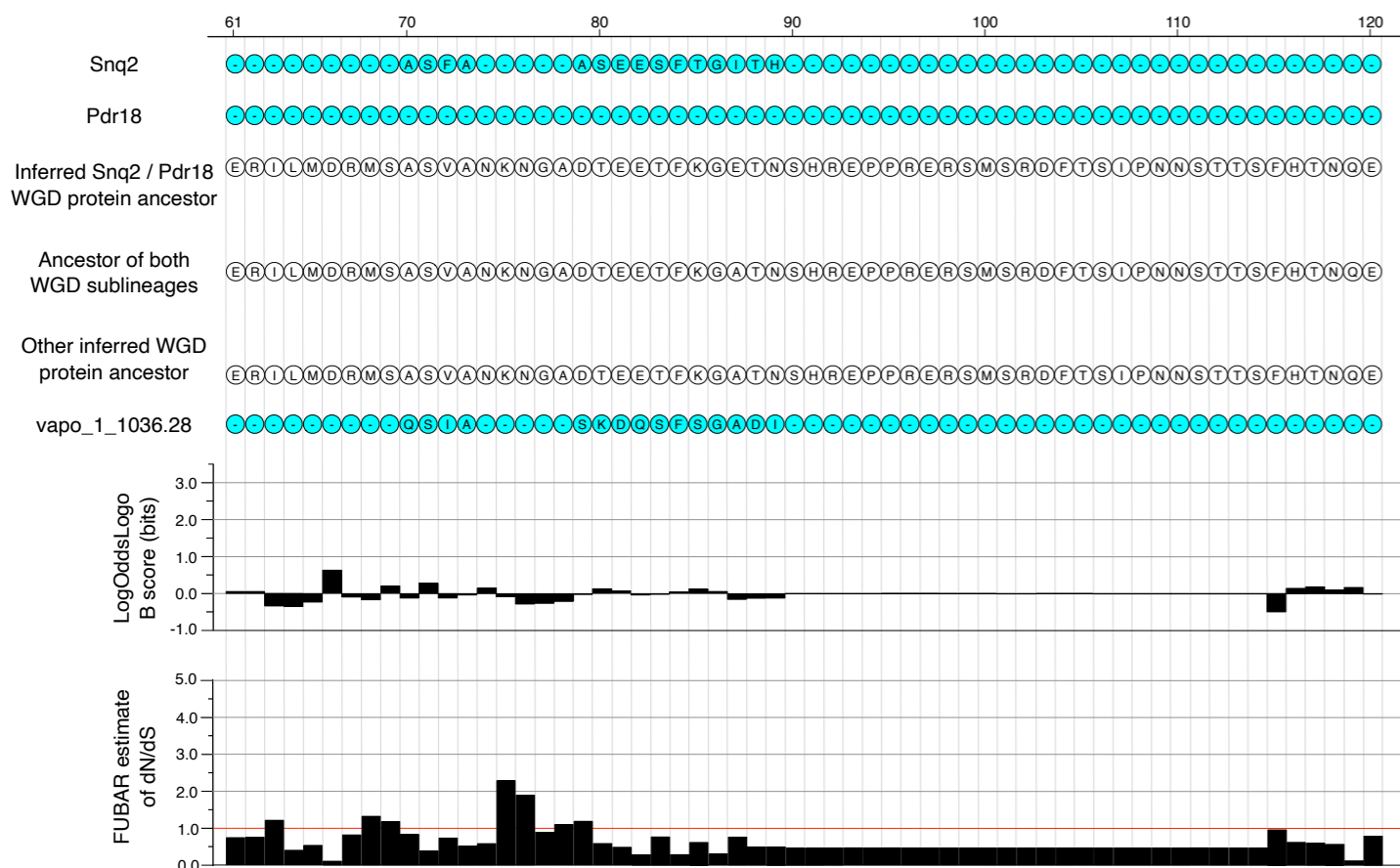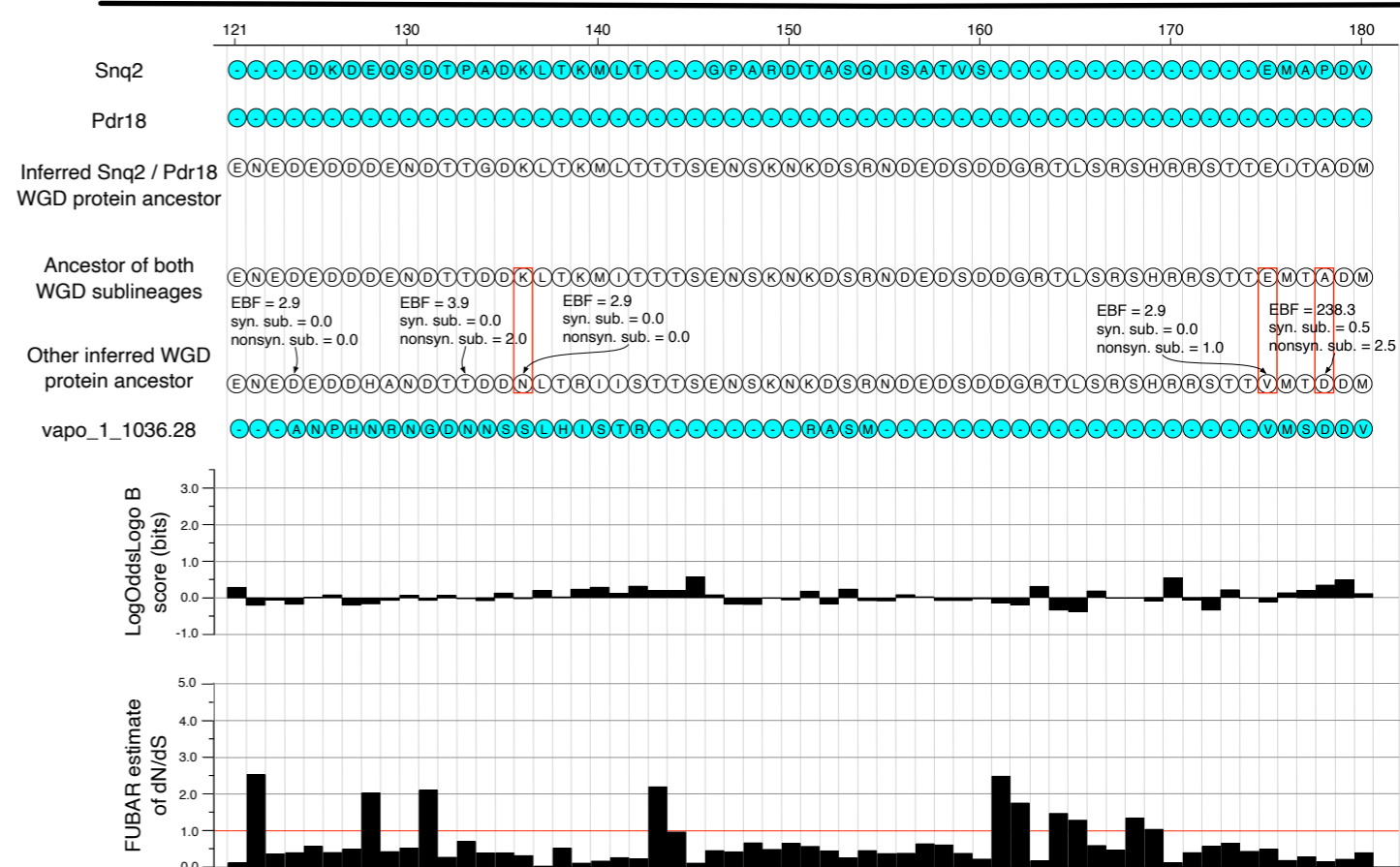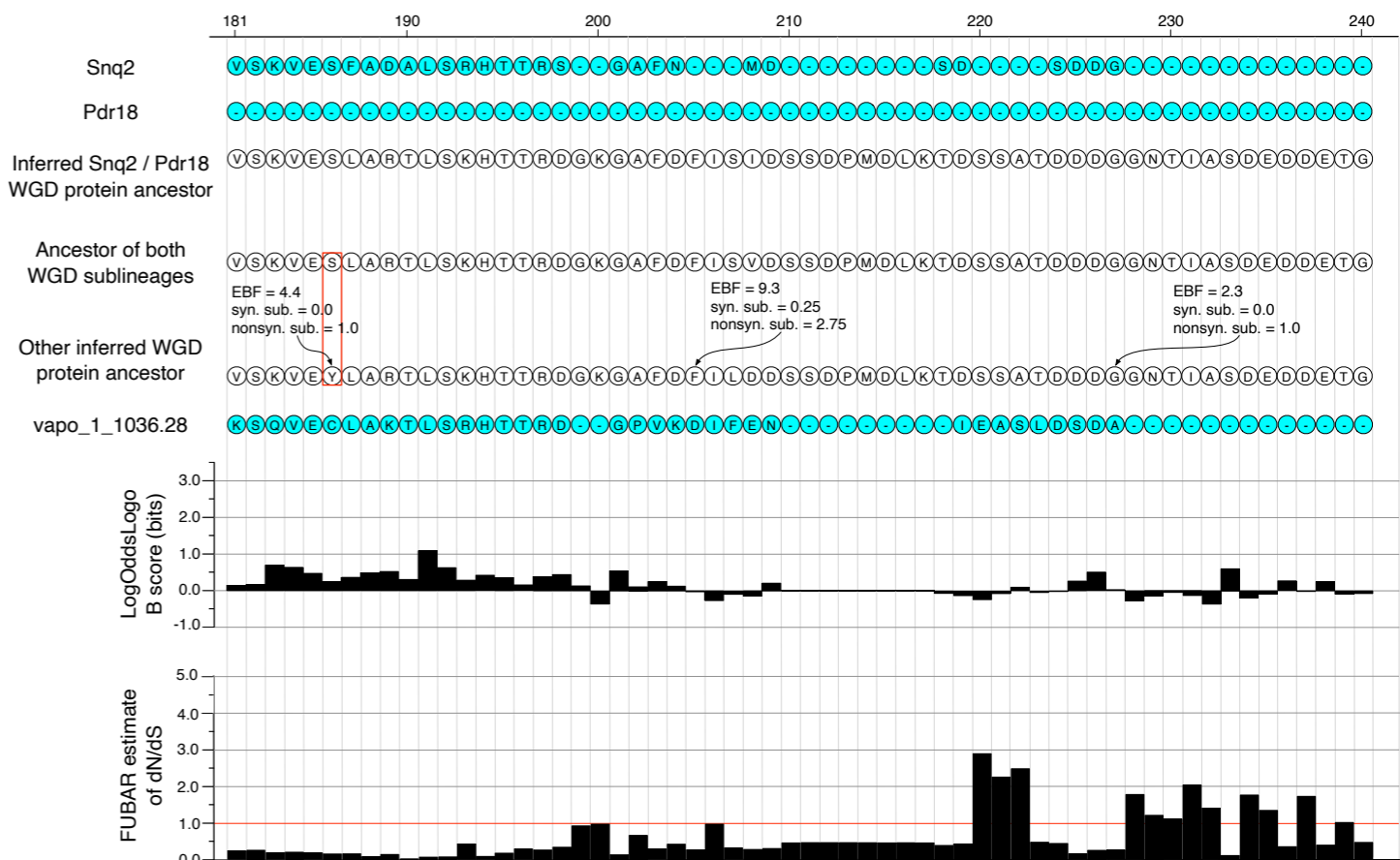



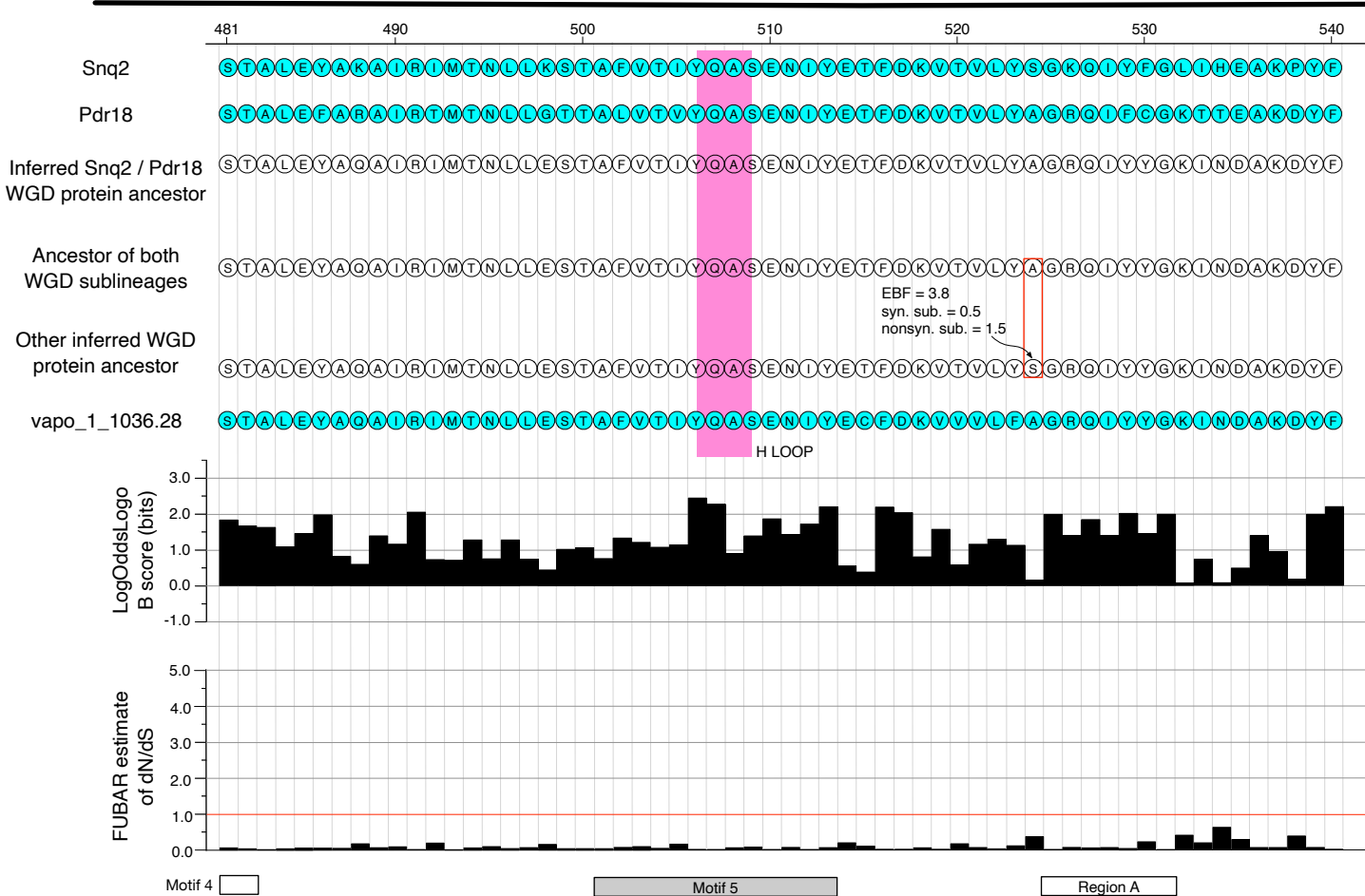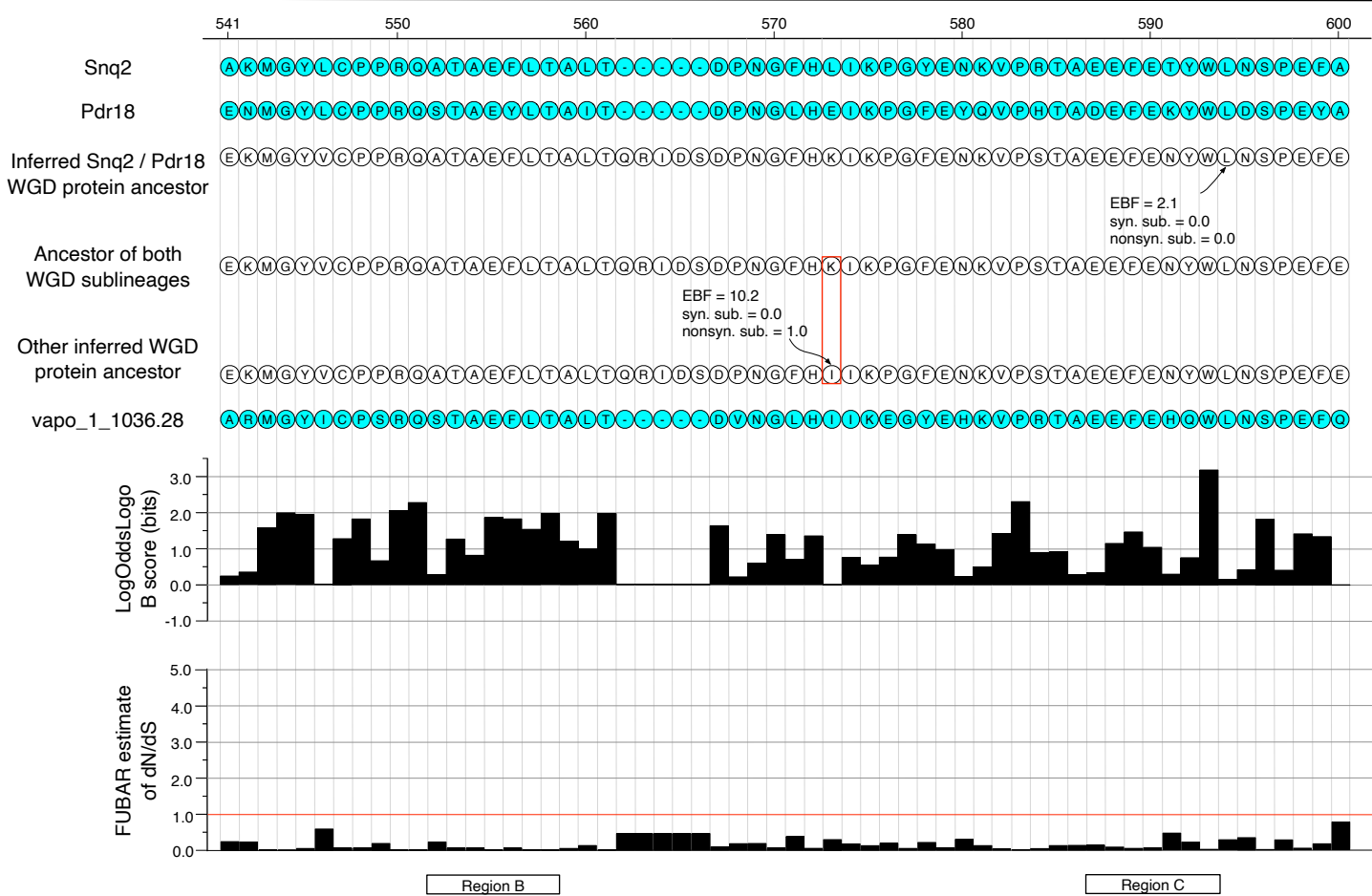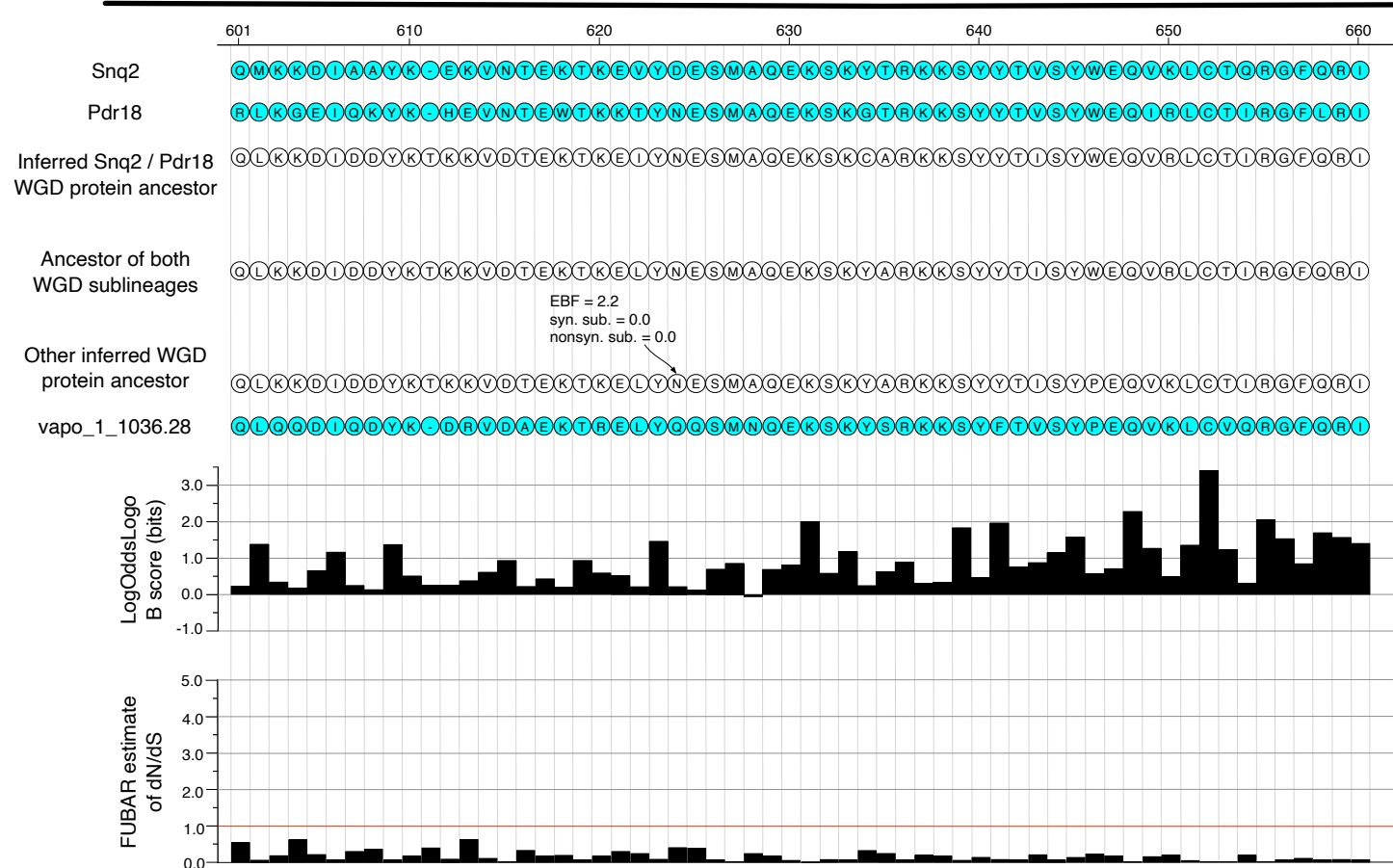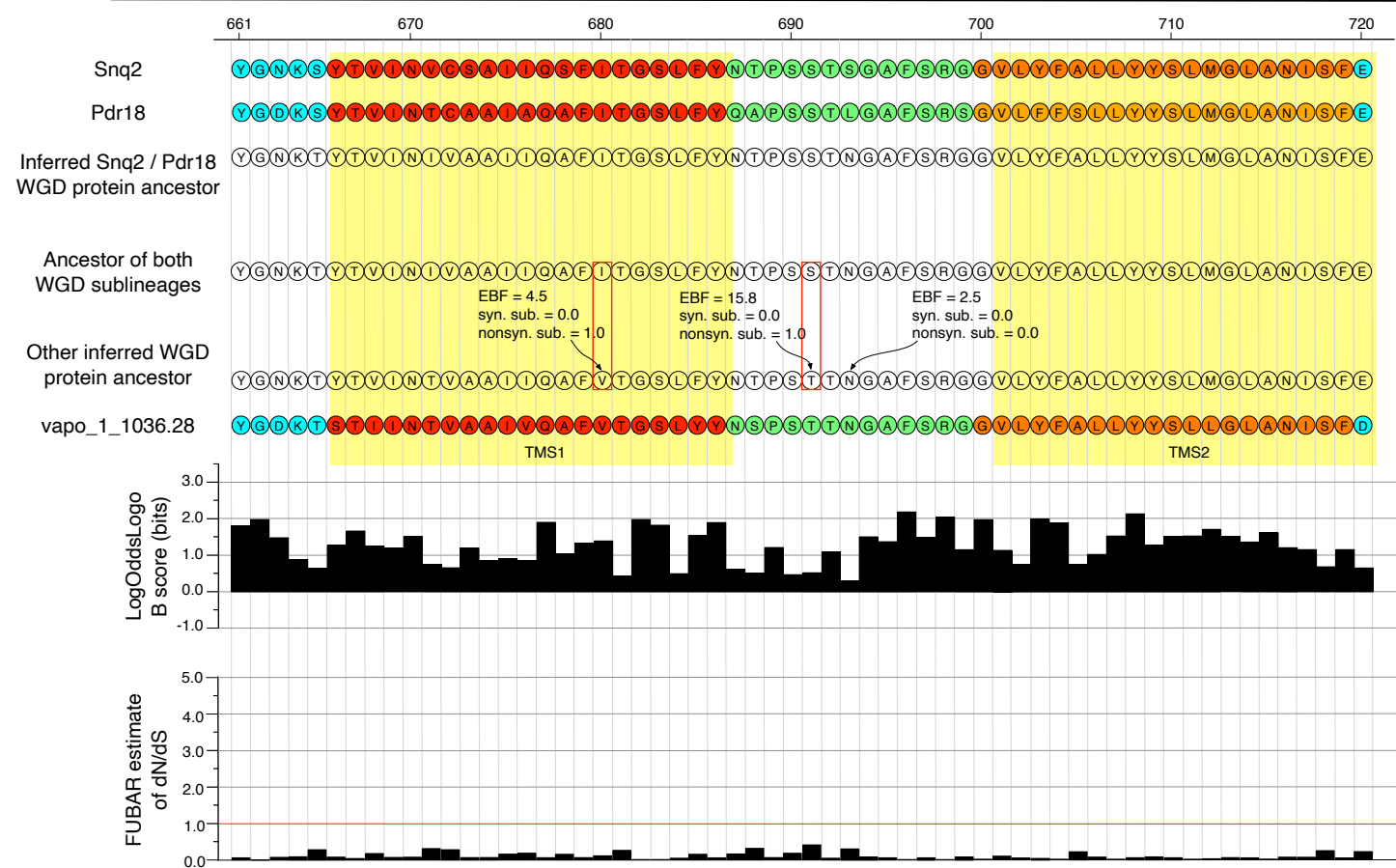

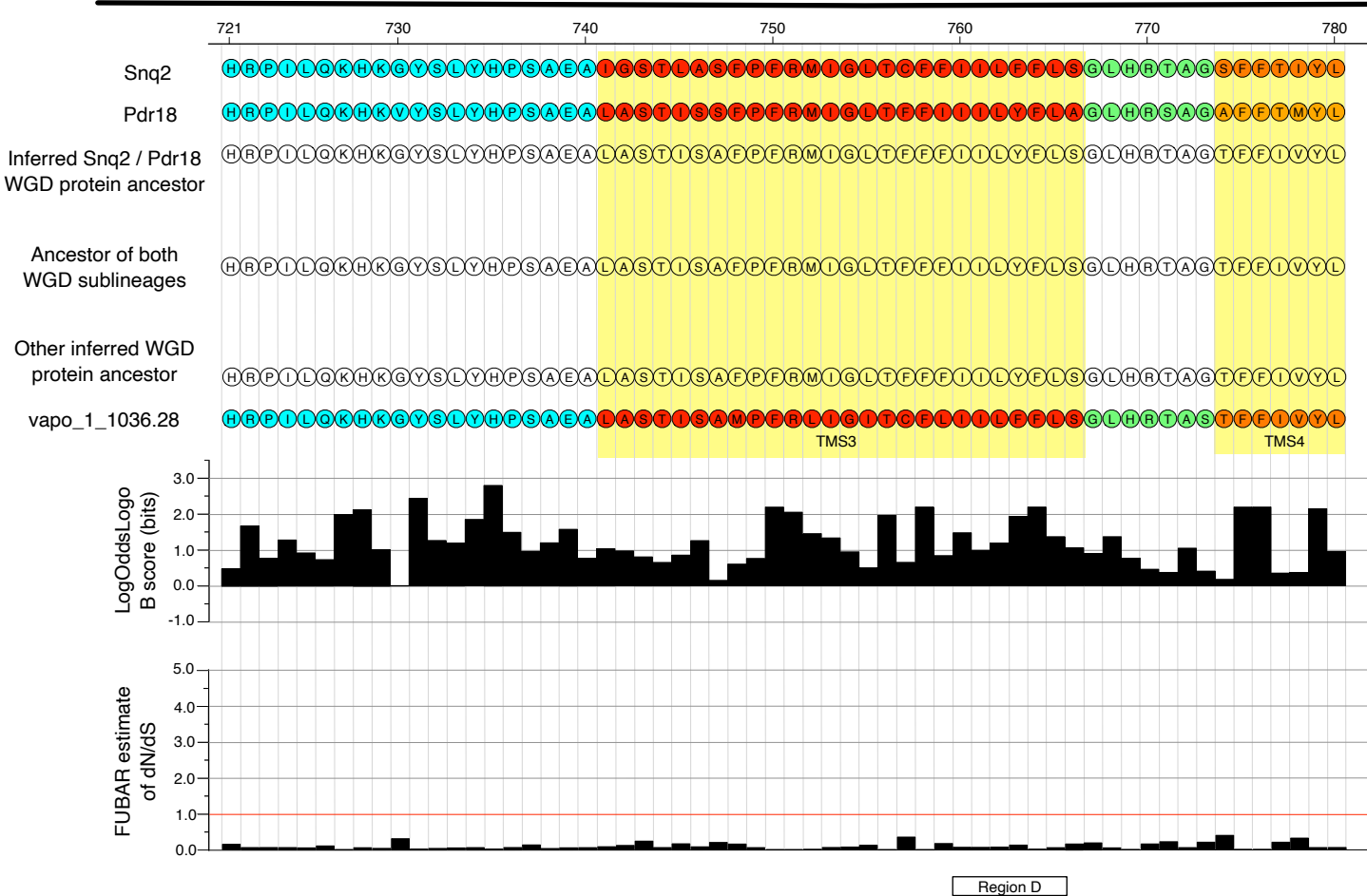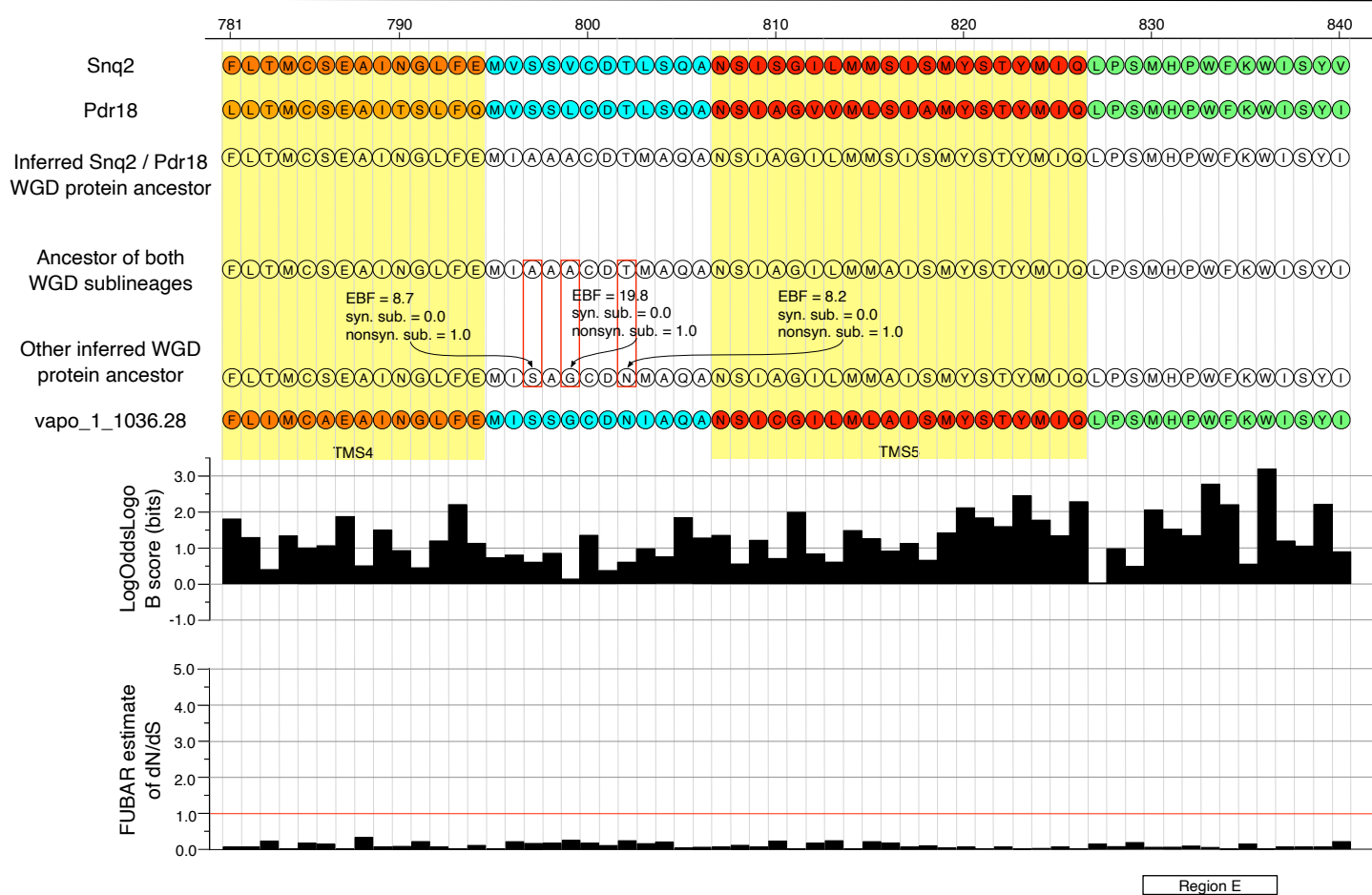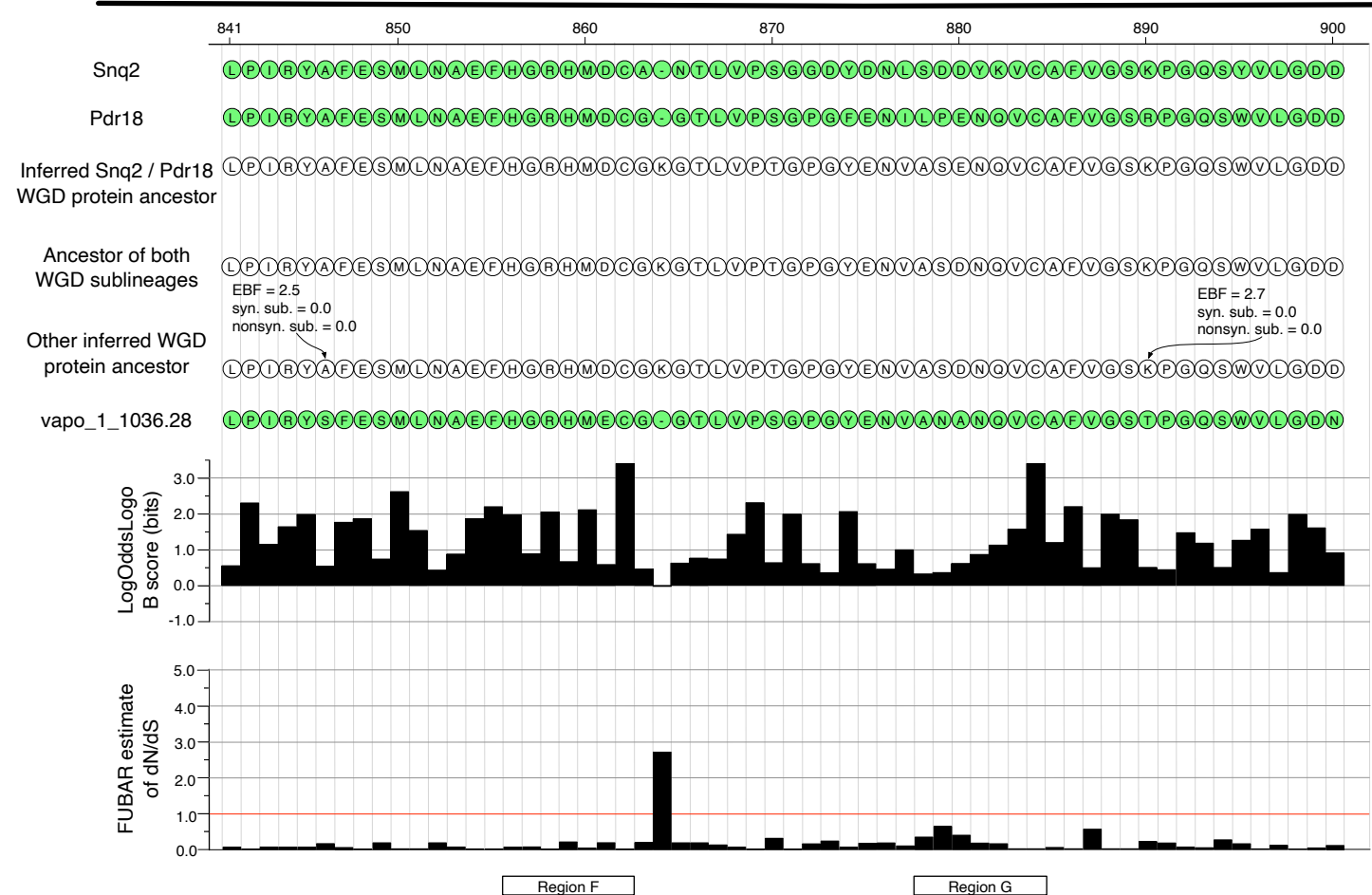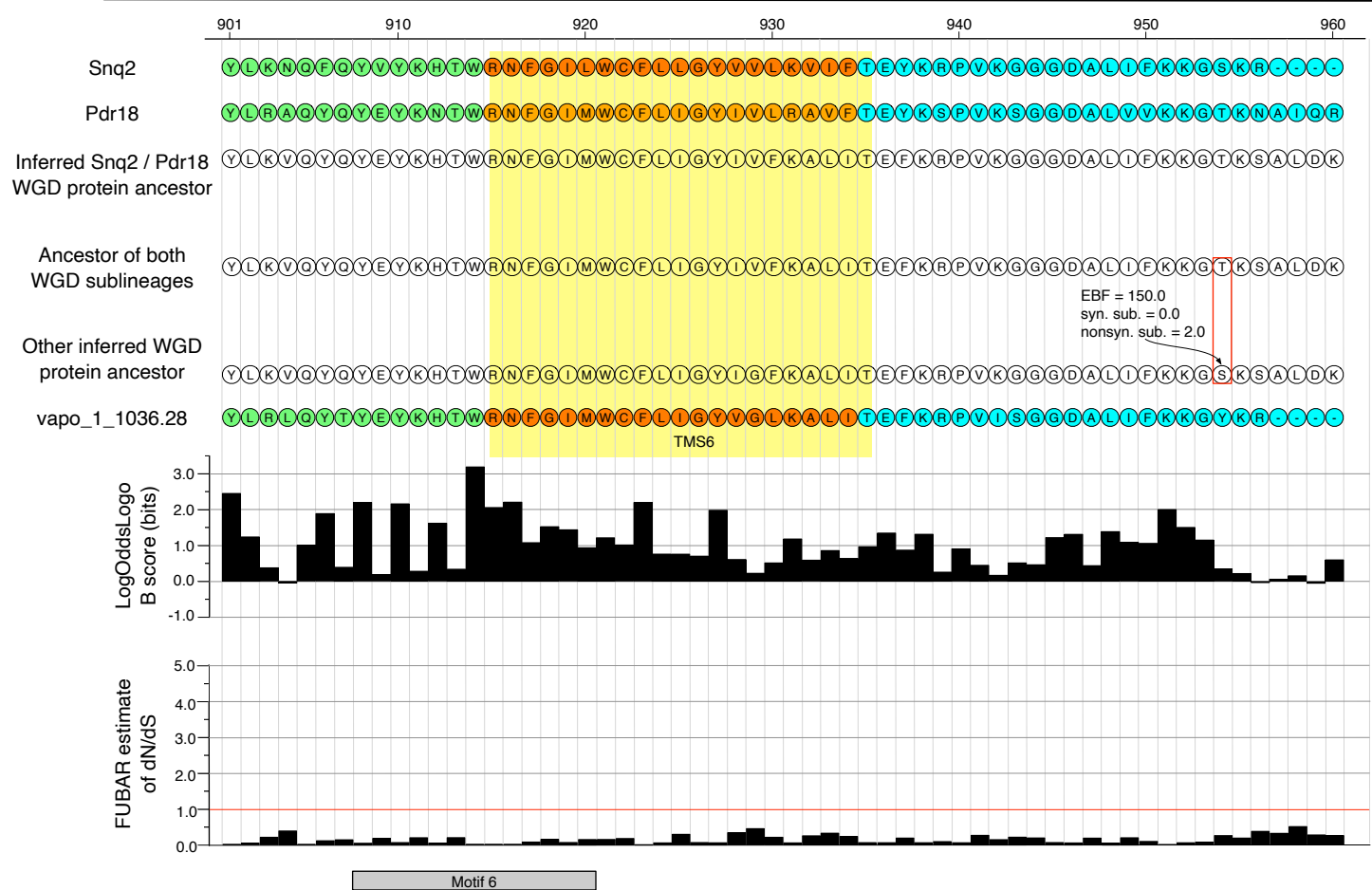

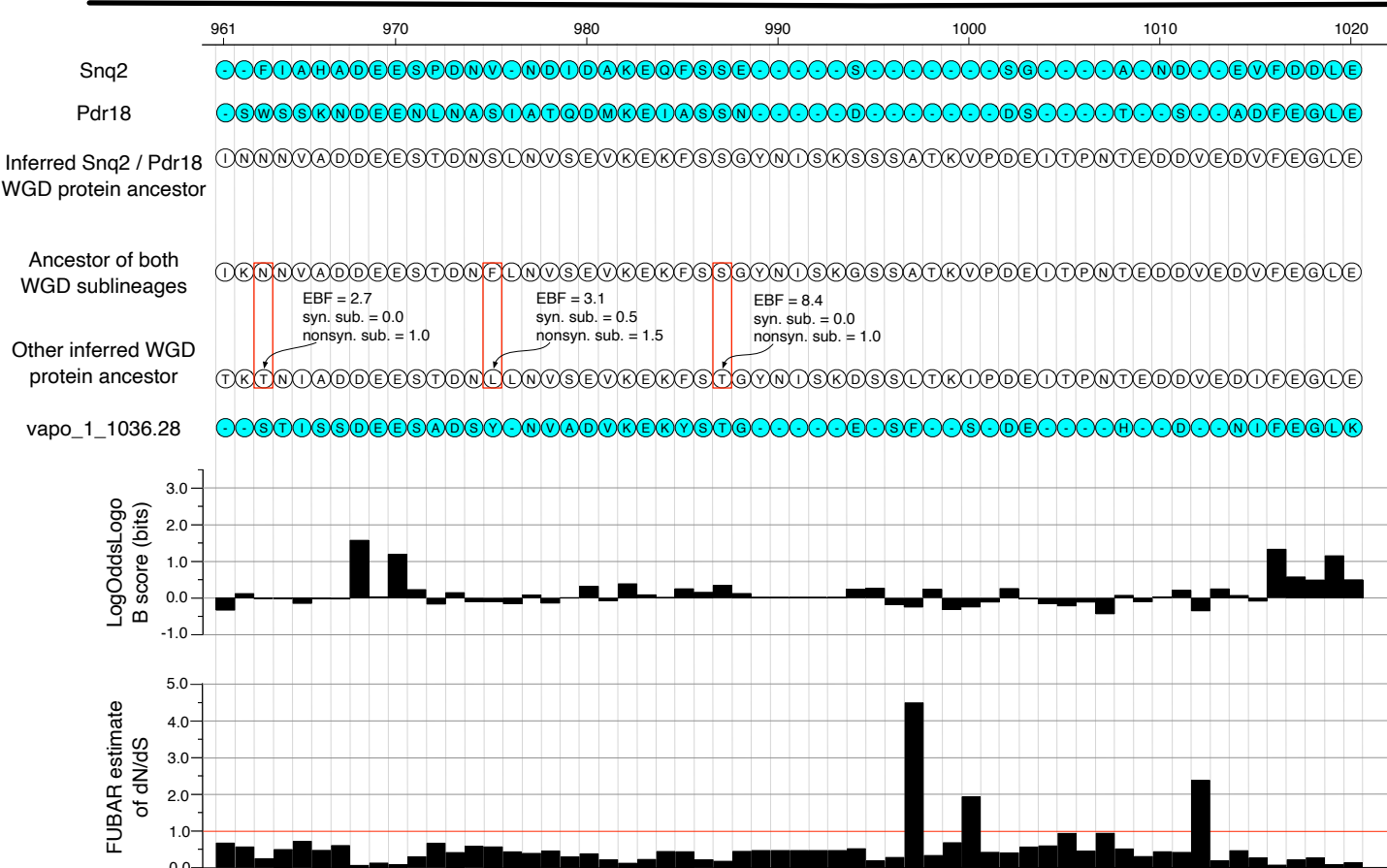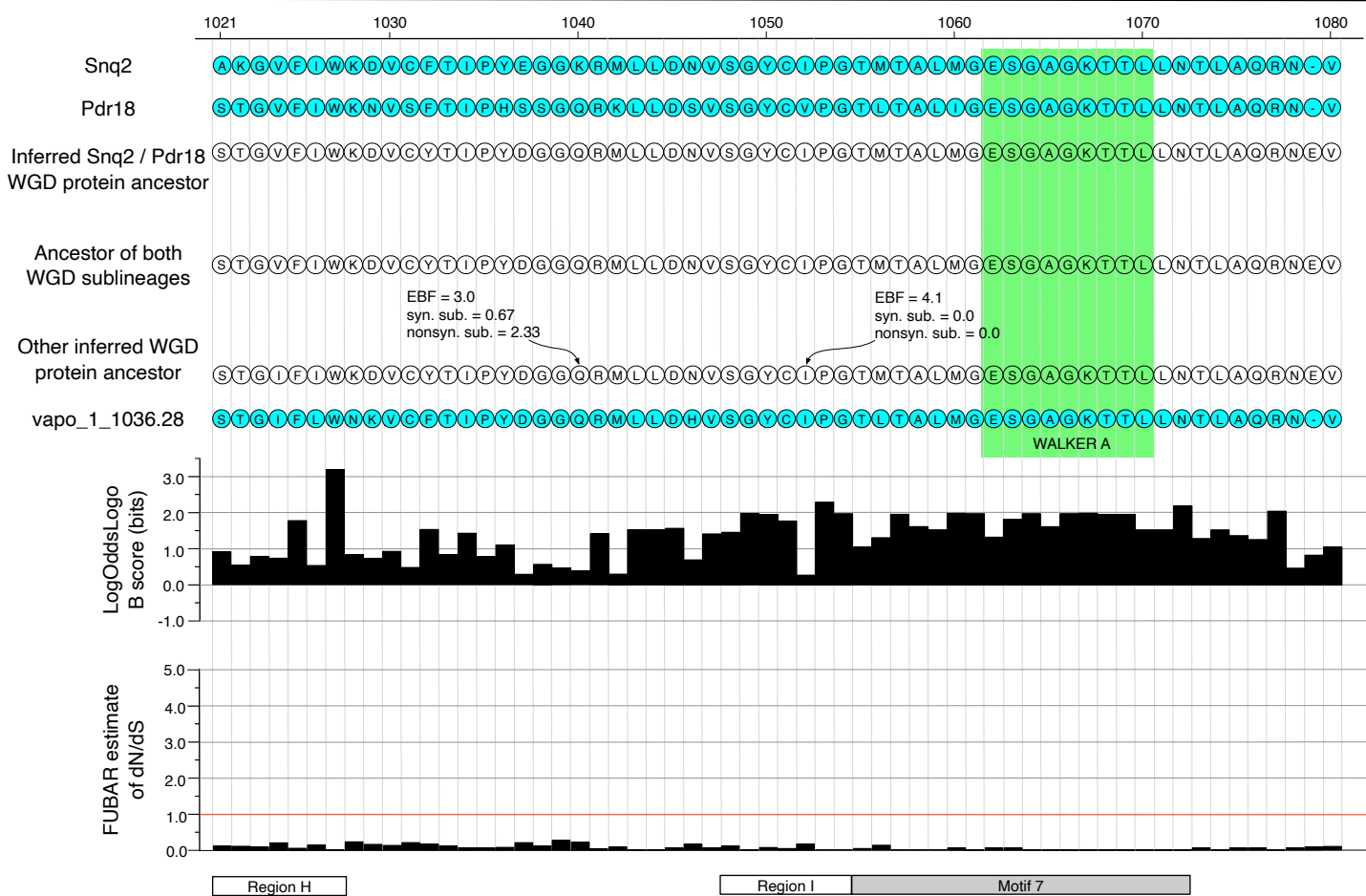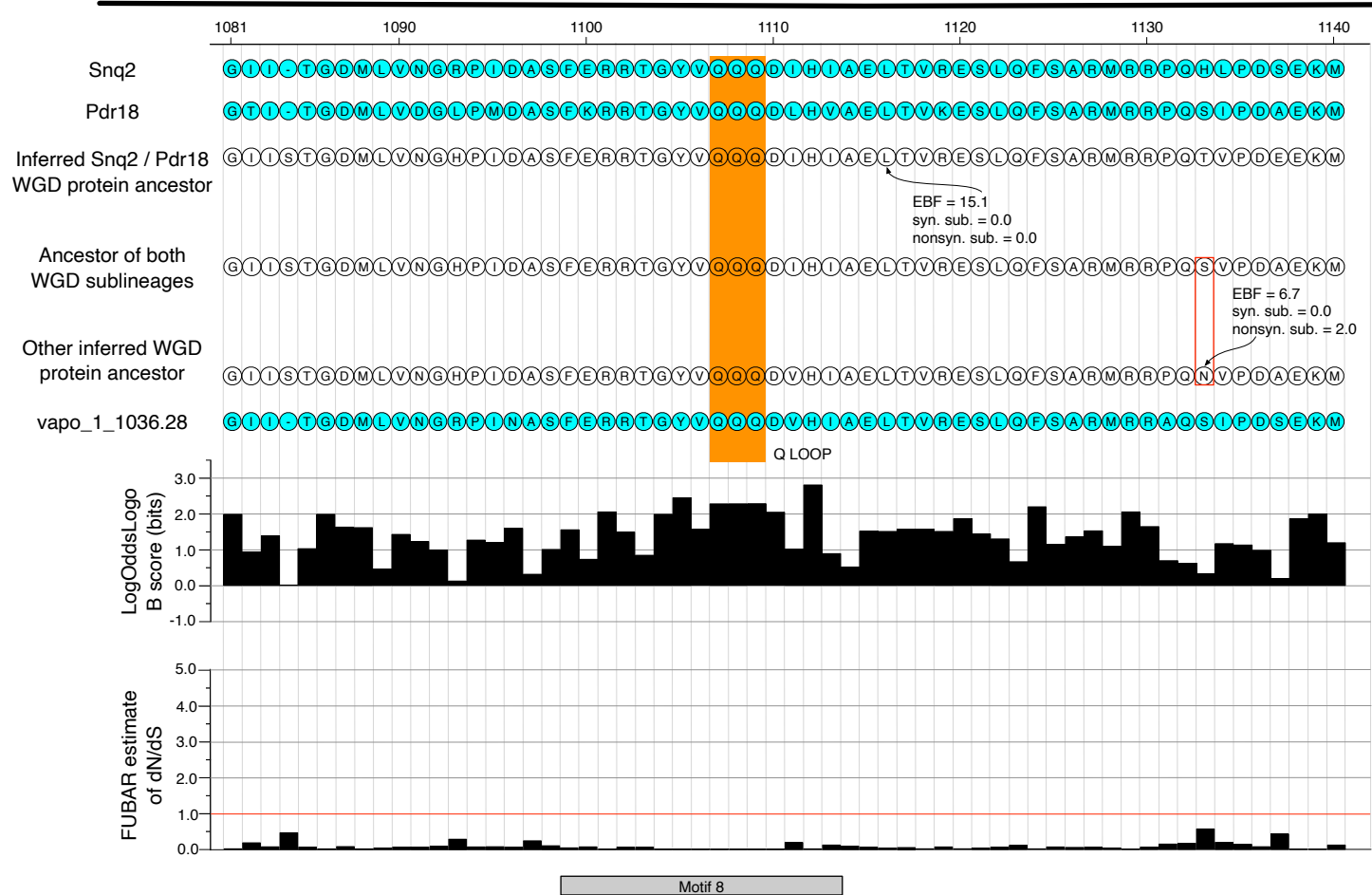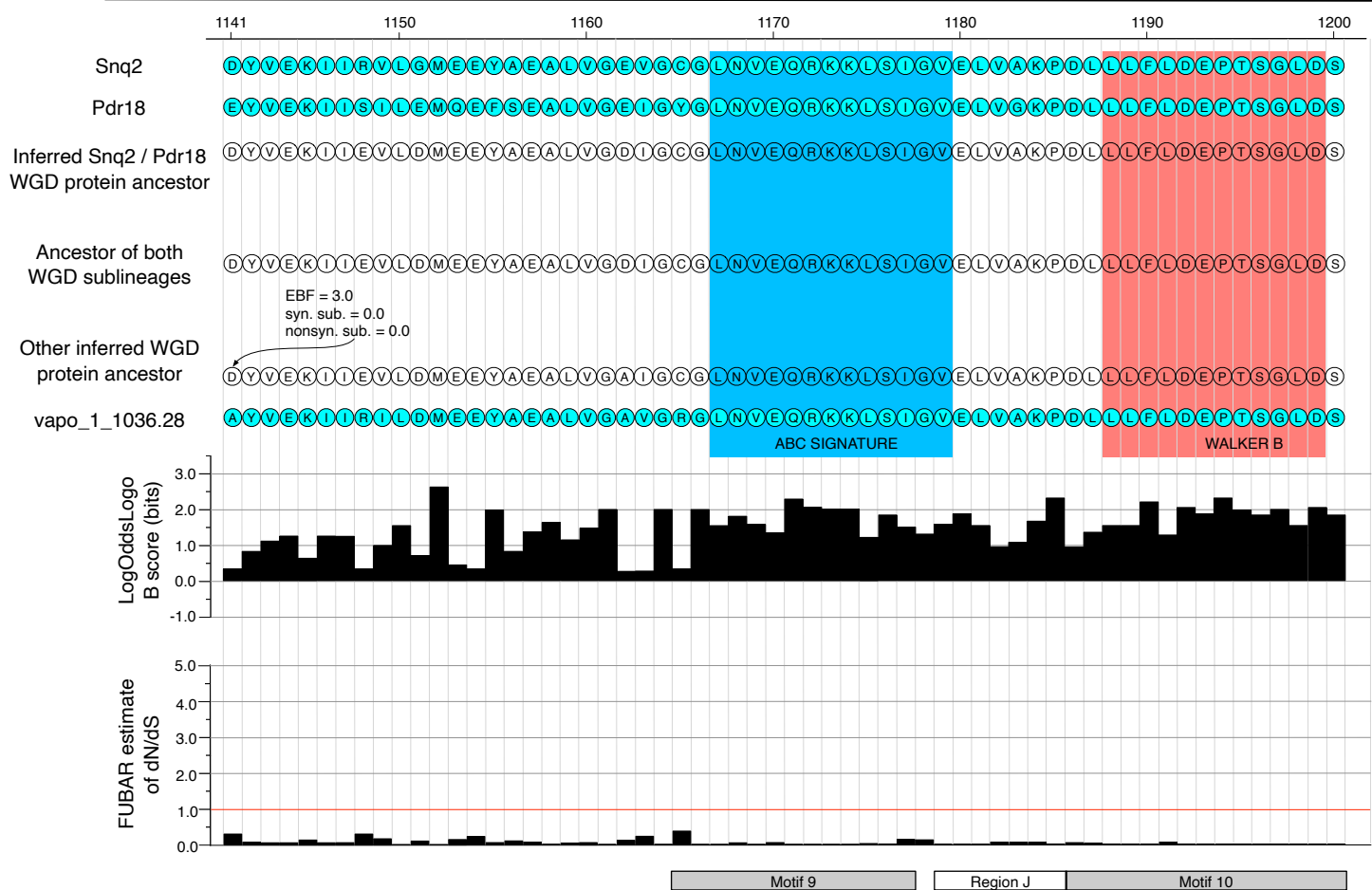

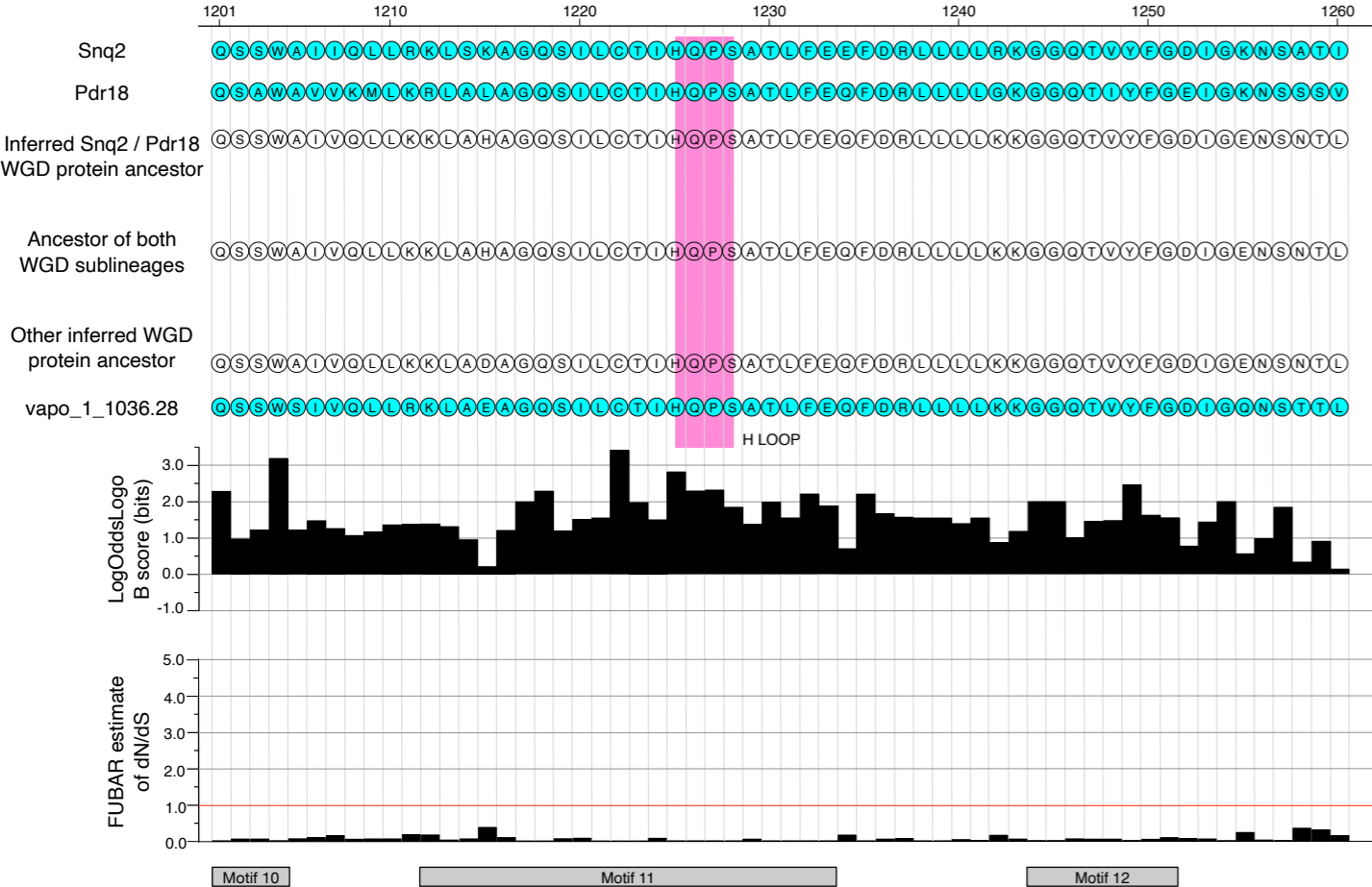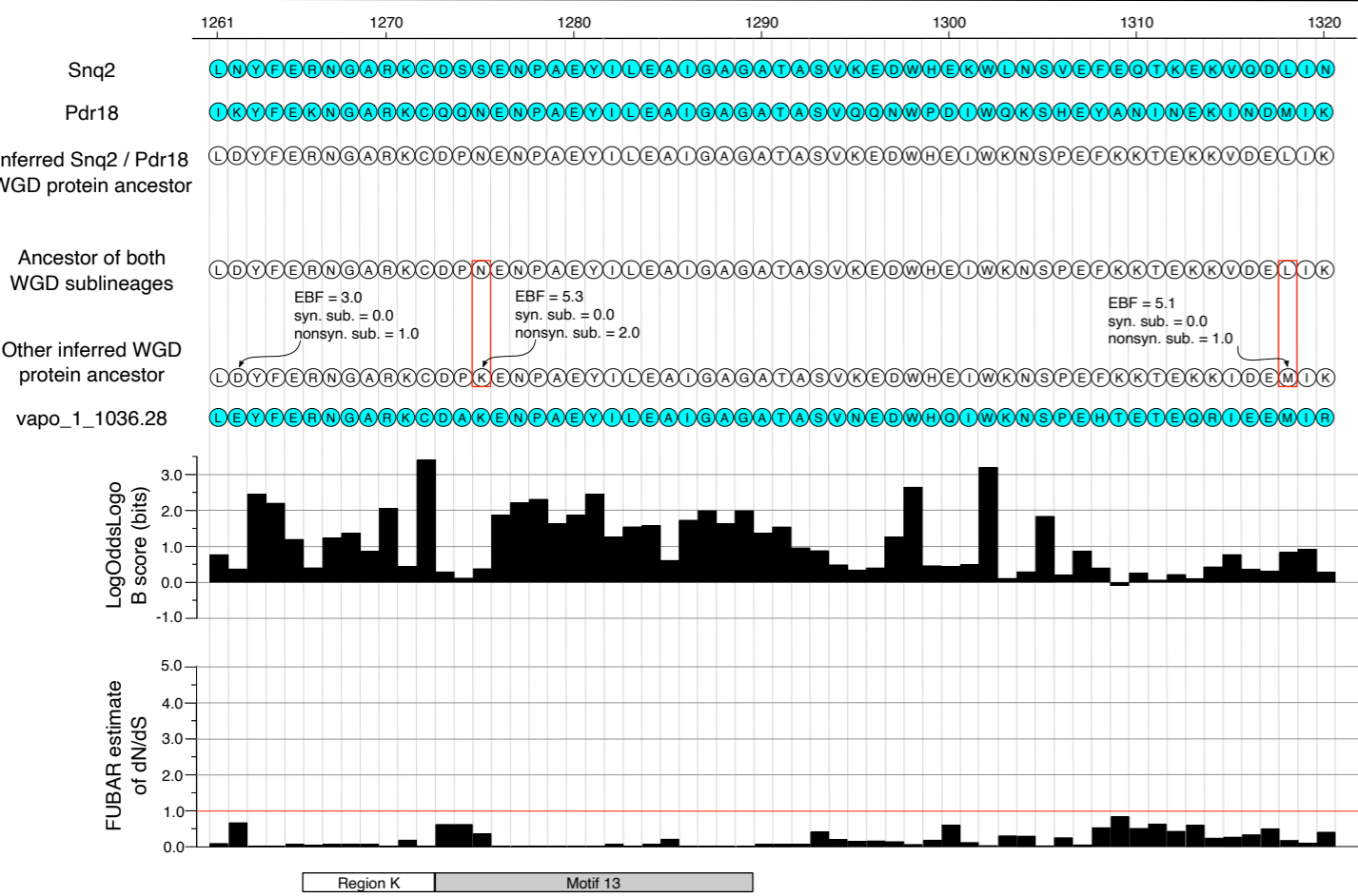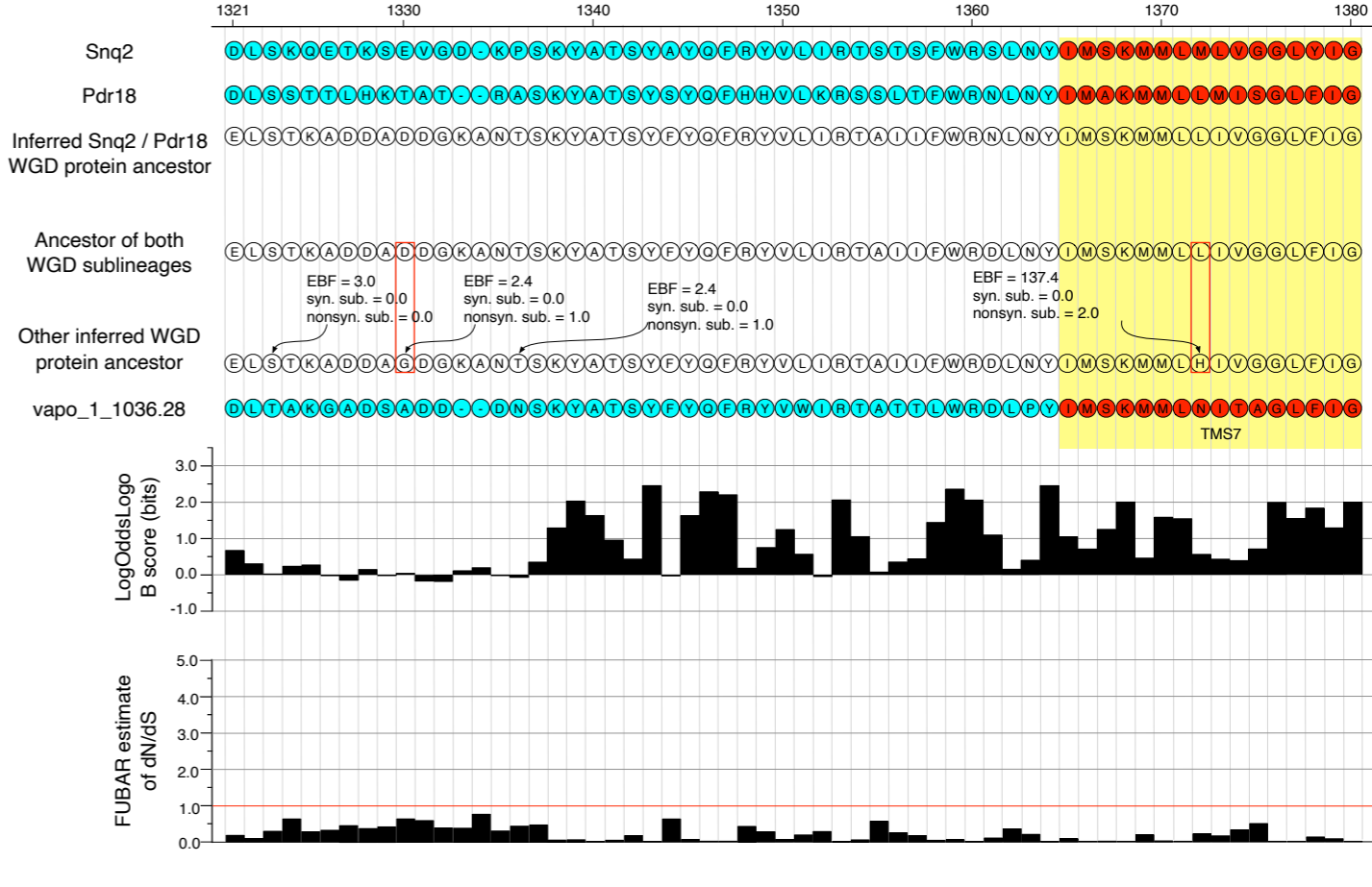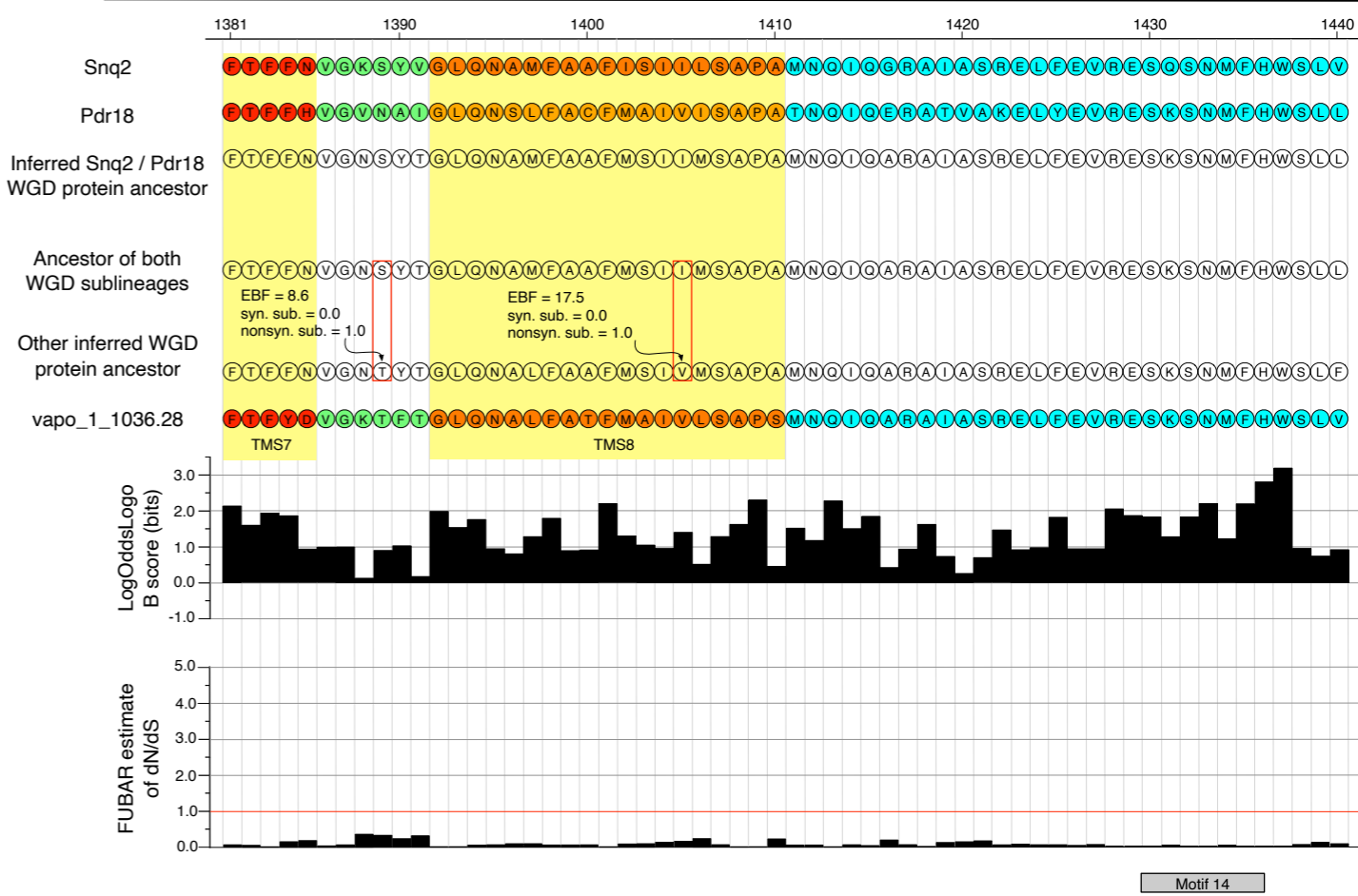

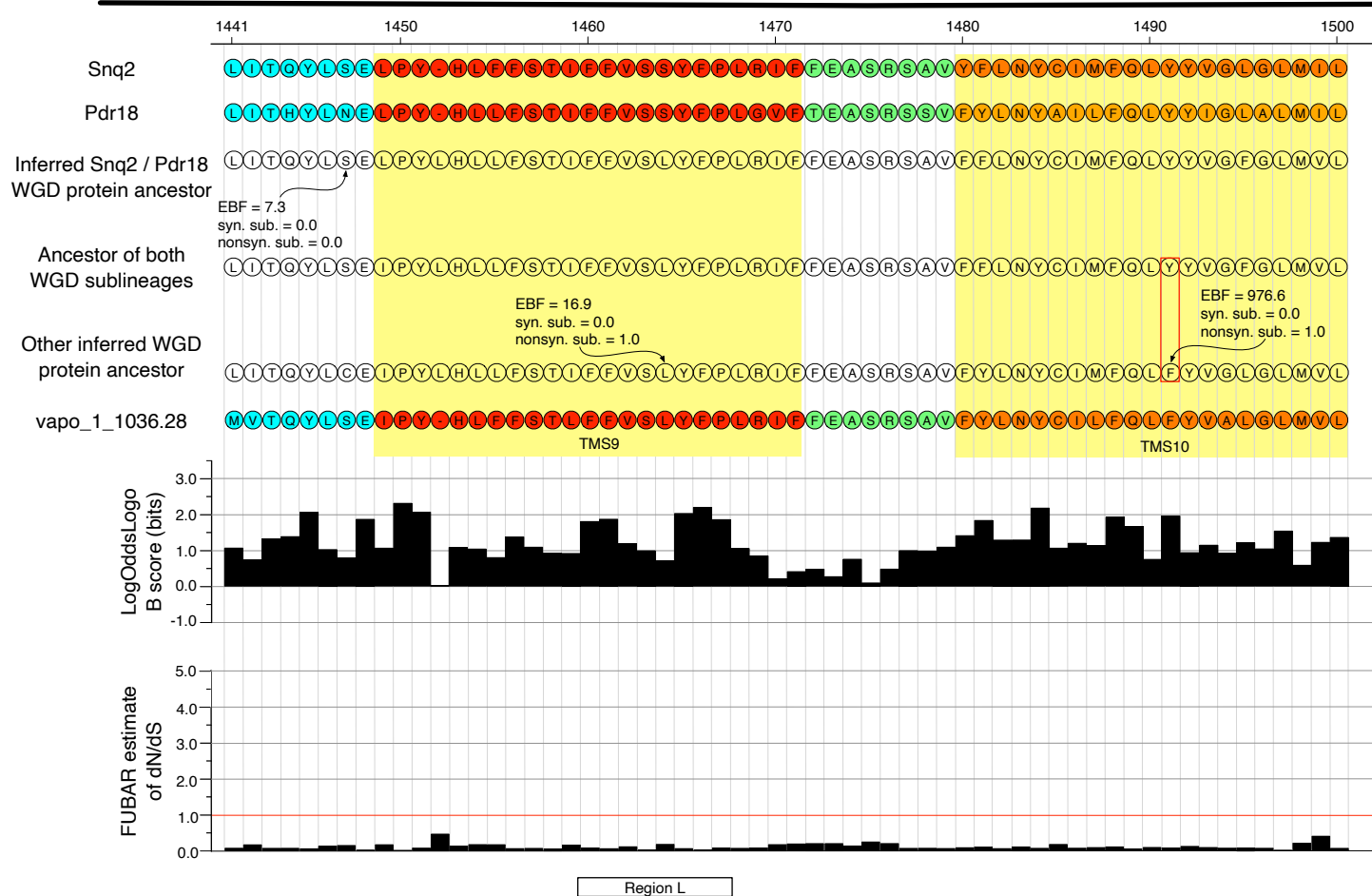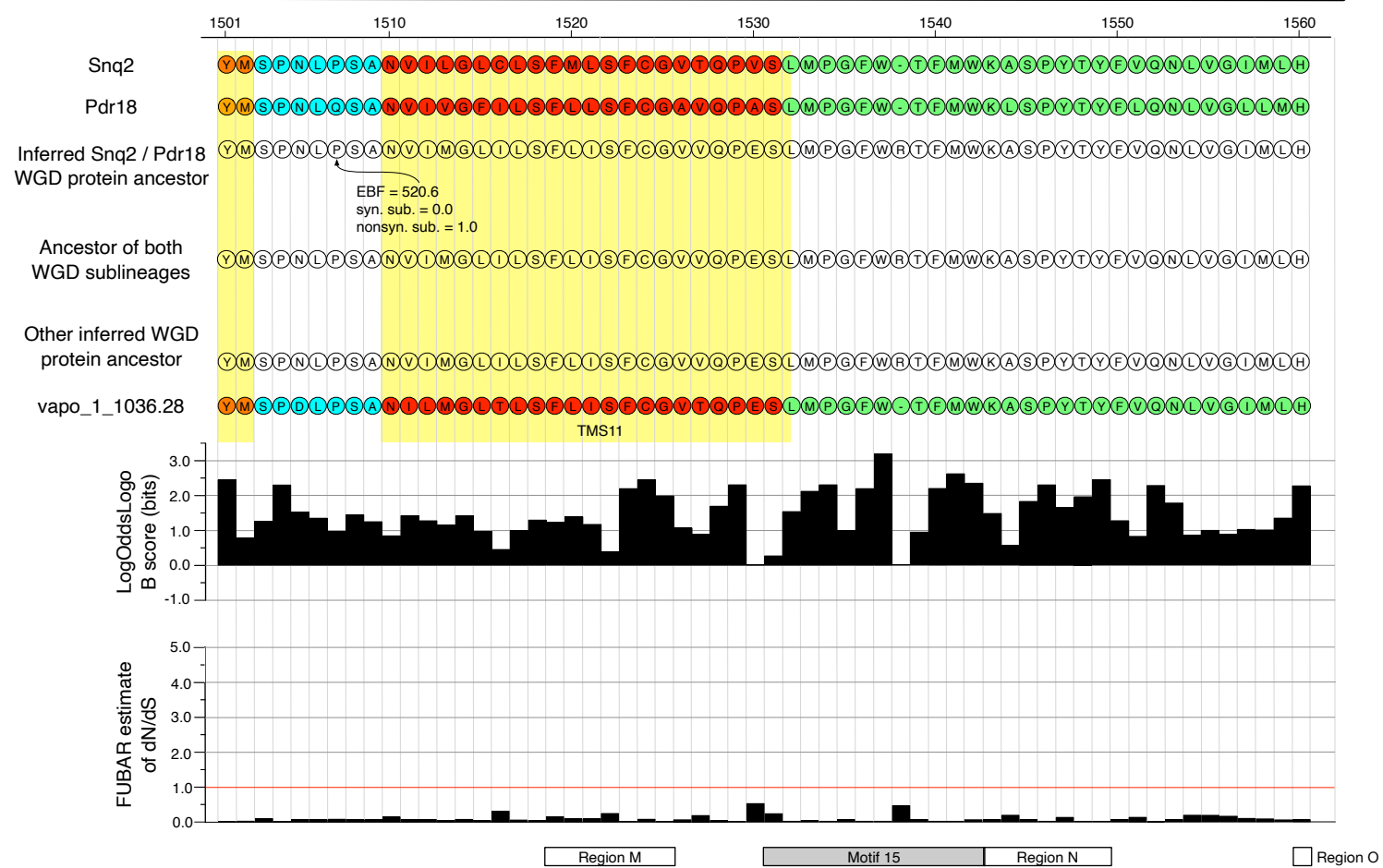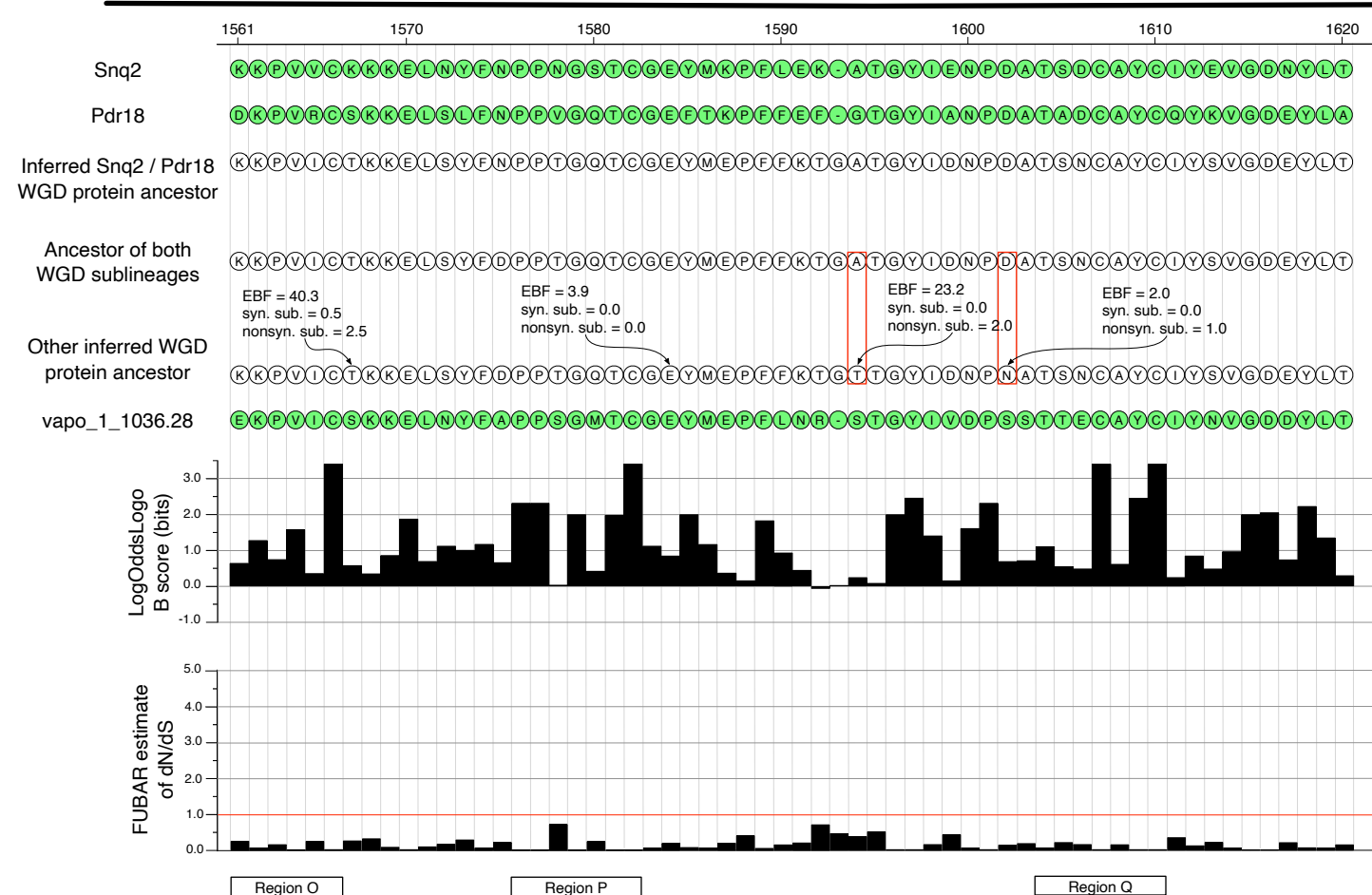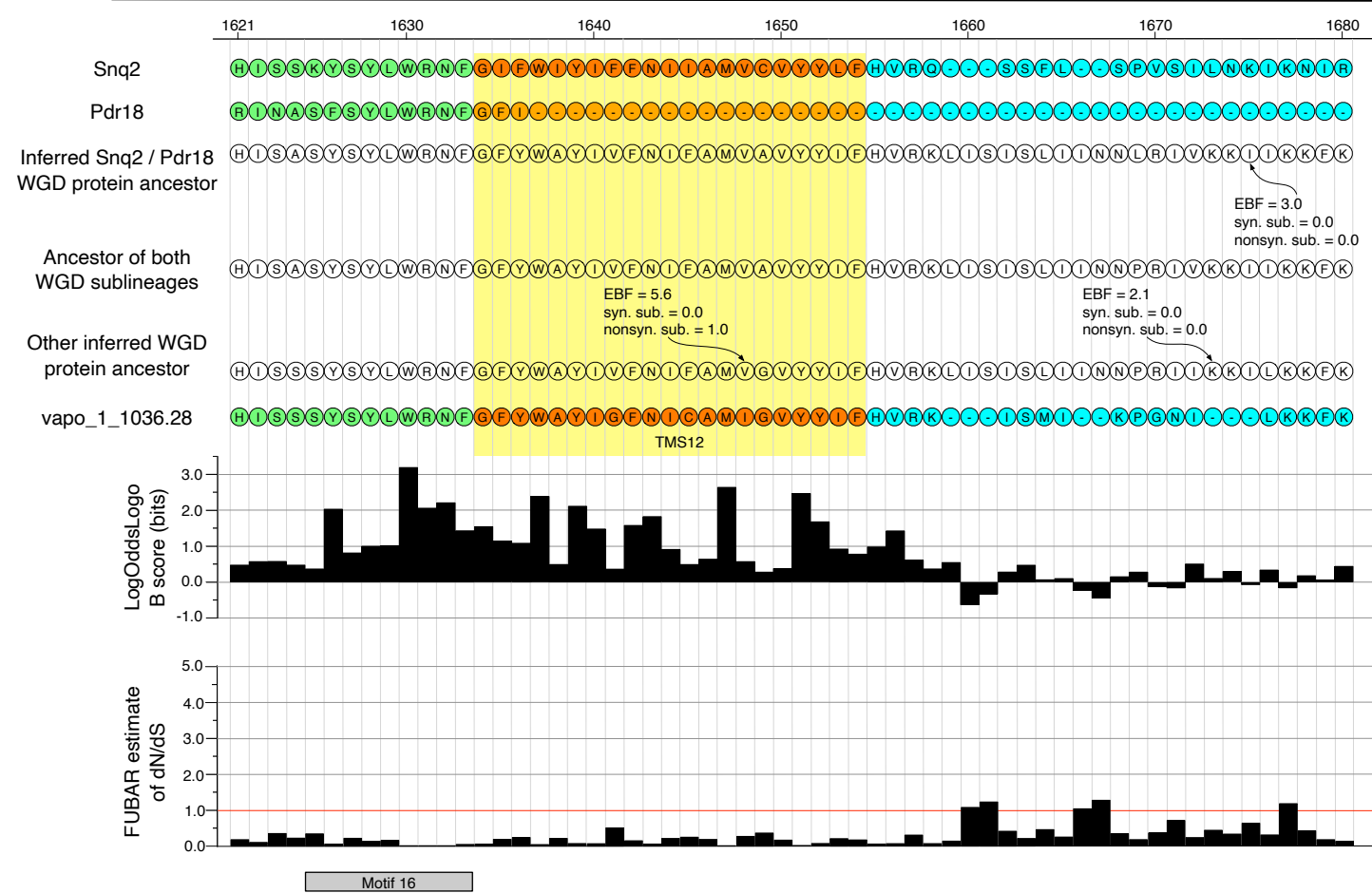

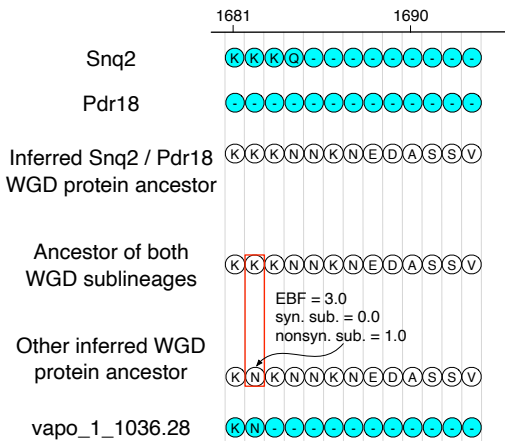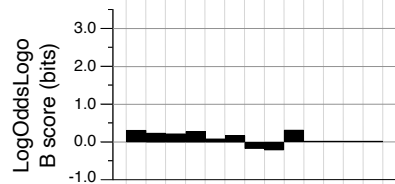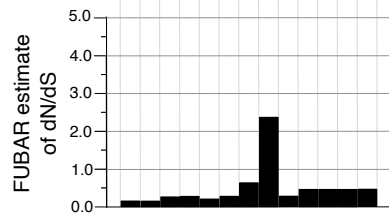

Supplement: foaf026_Supplemental_Files [file foaf026_supplemental_files.zip › Figure A31_Supplementary_Data.pdf]

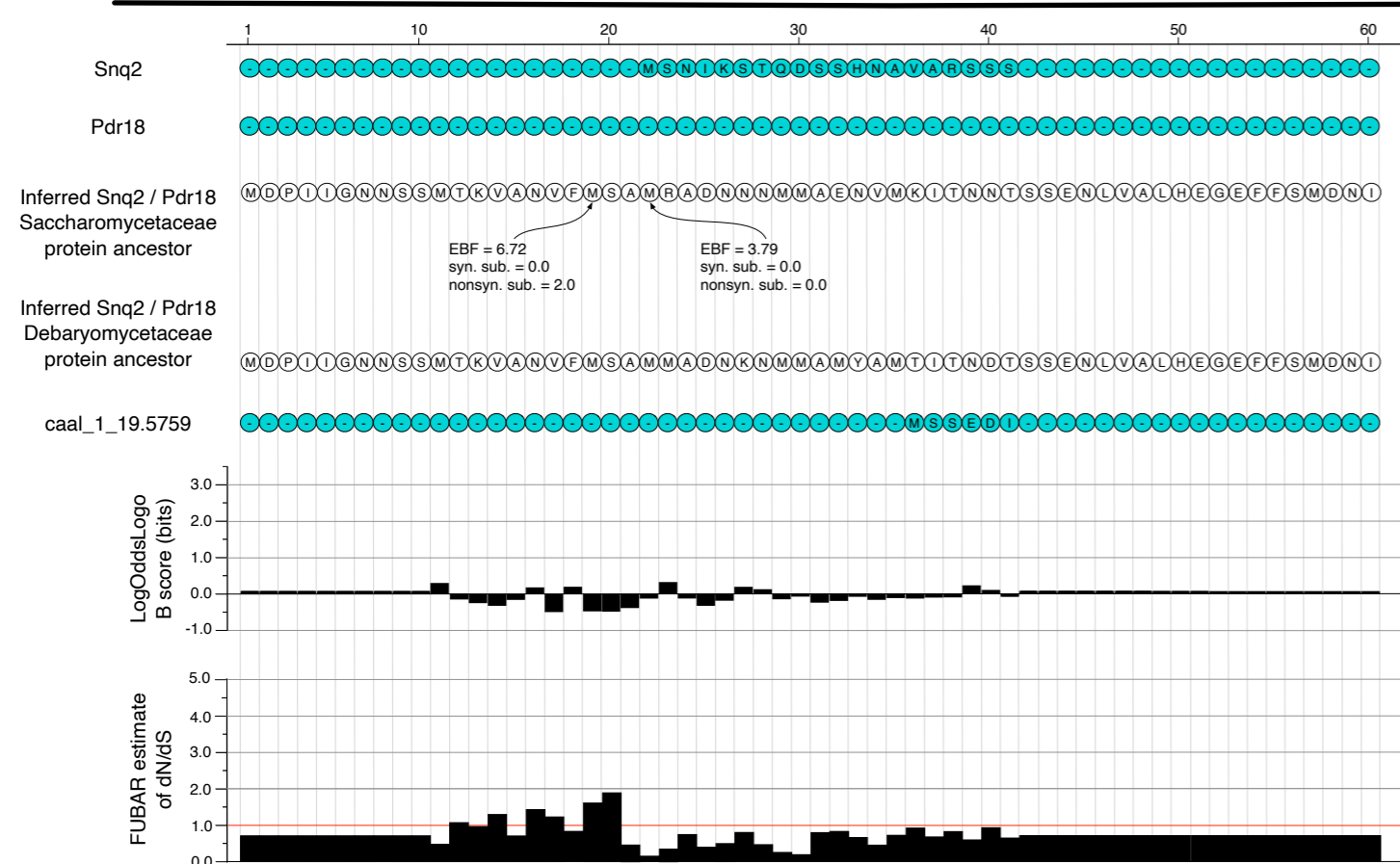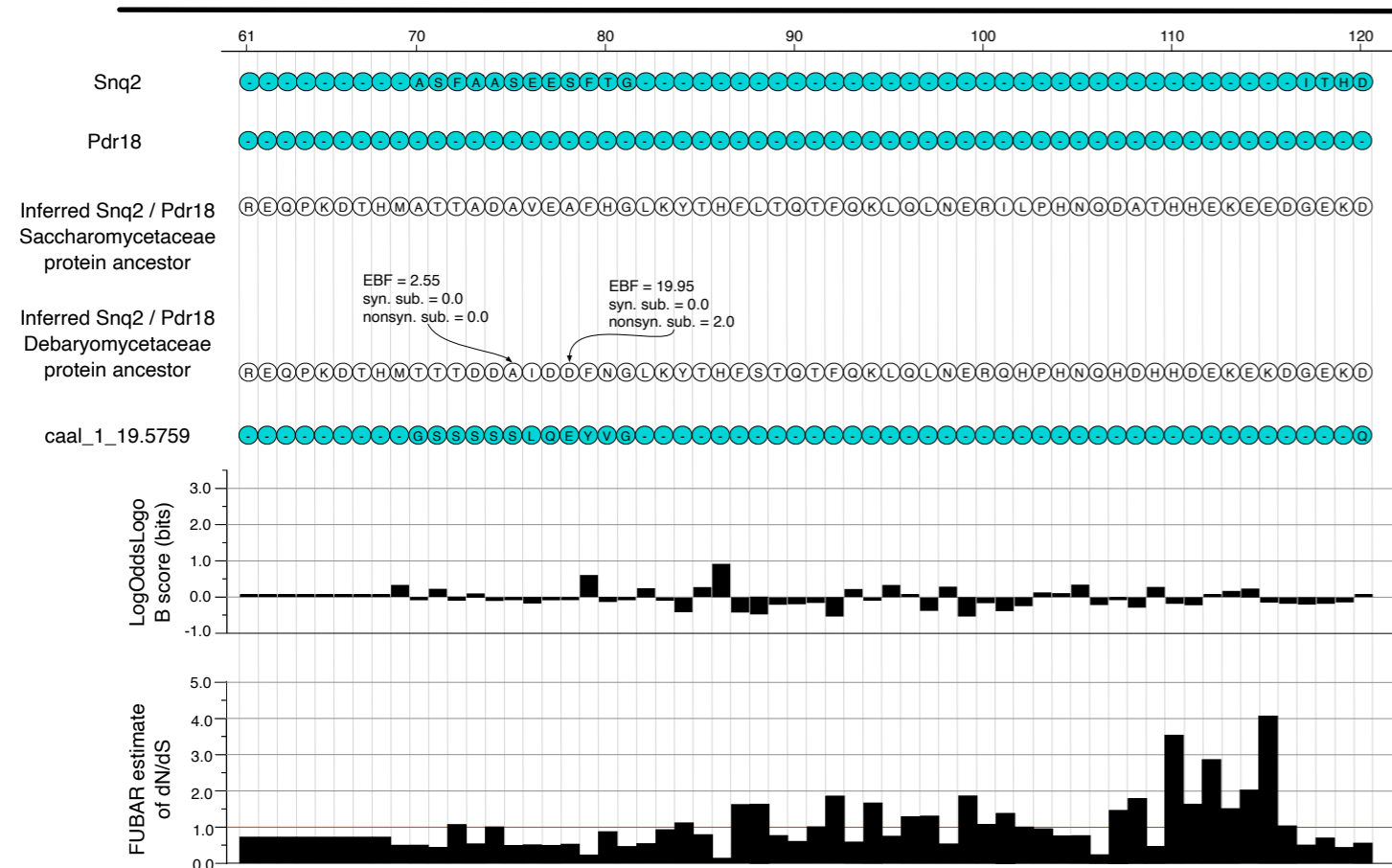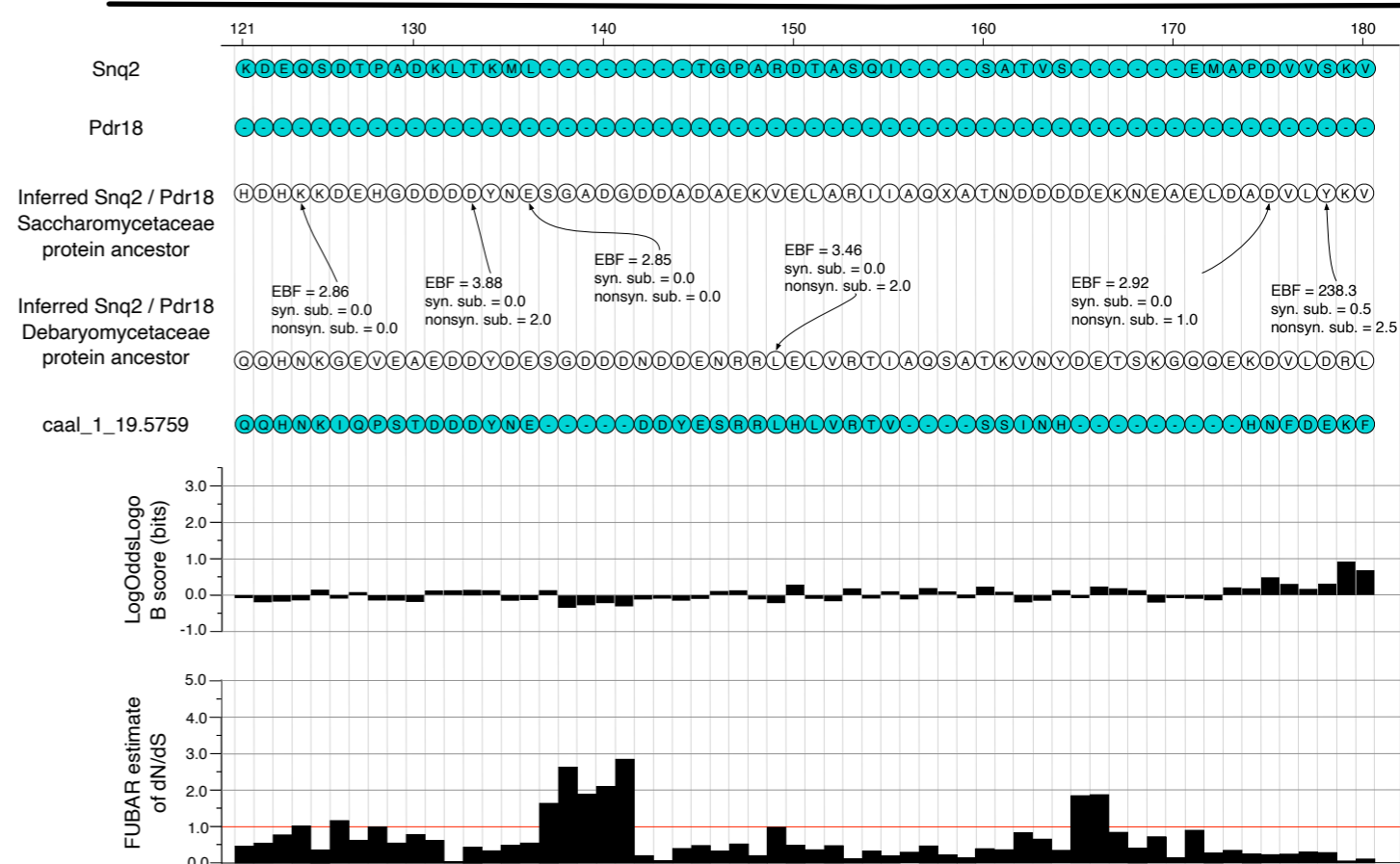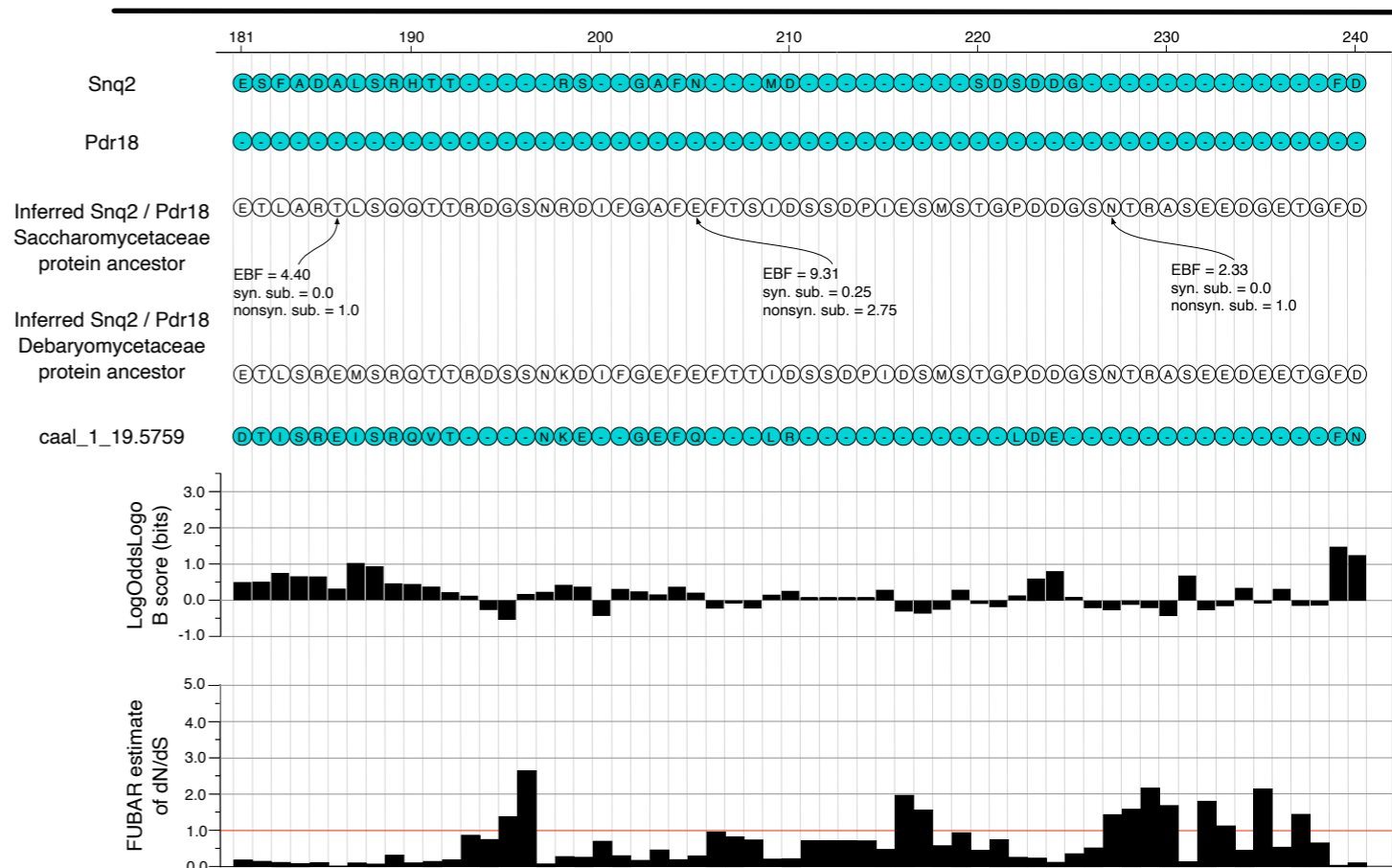

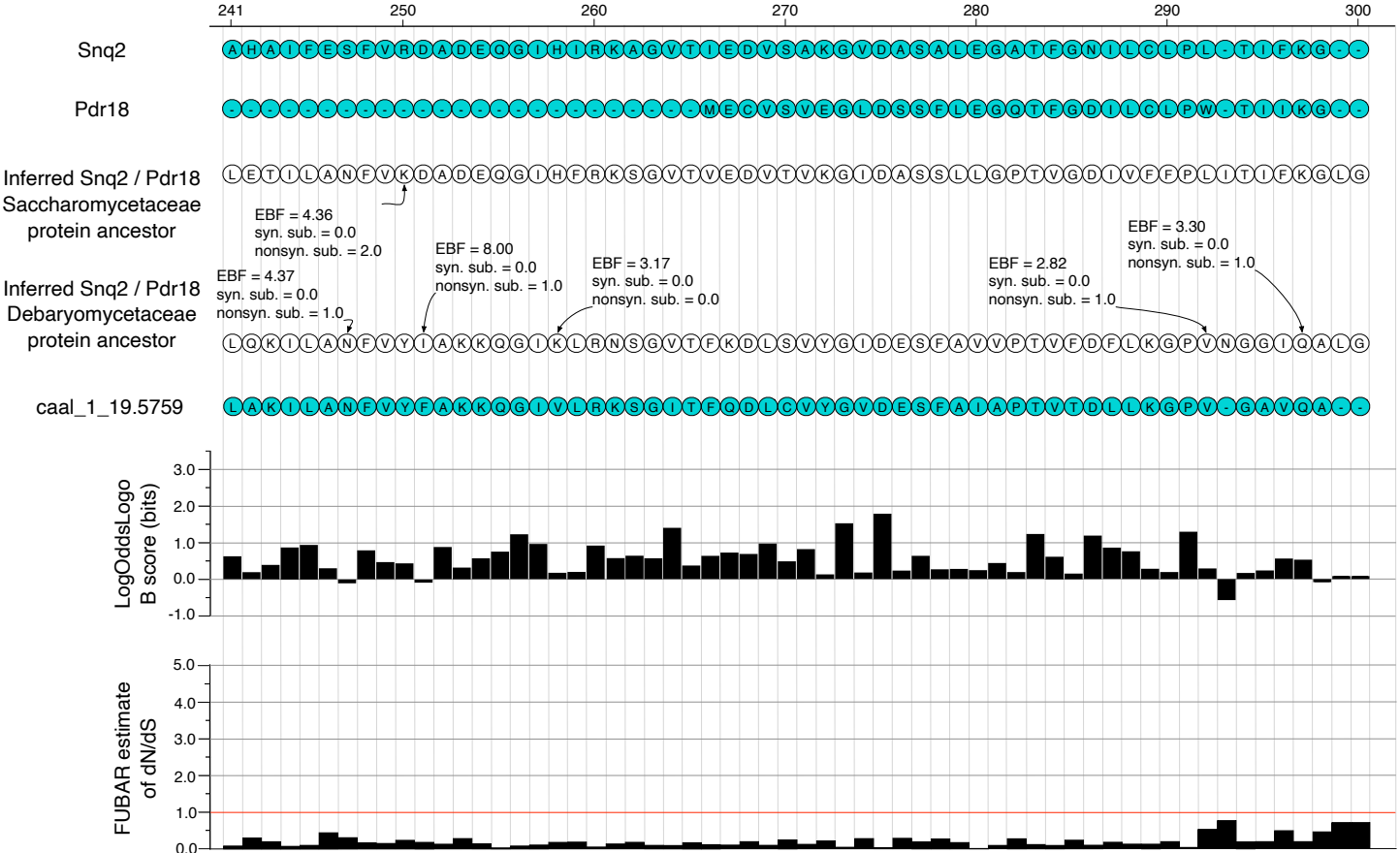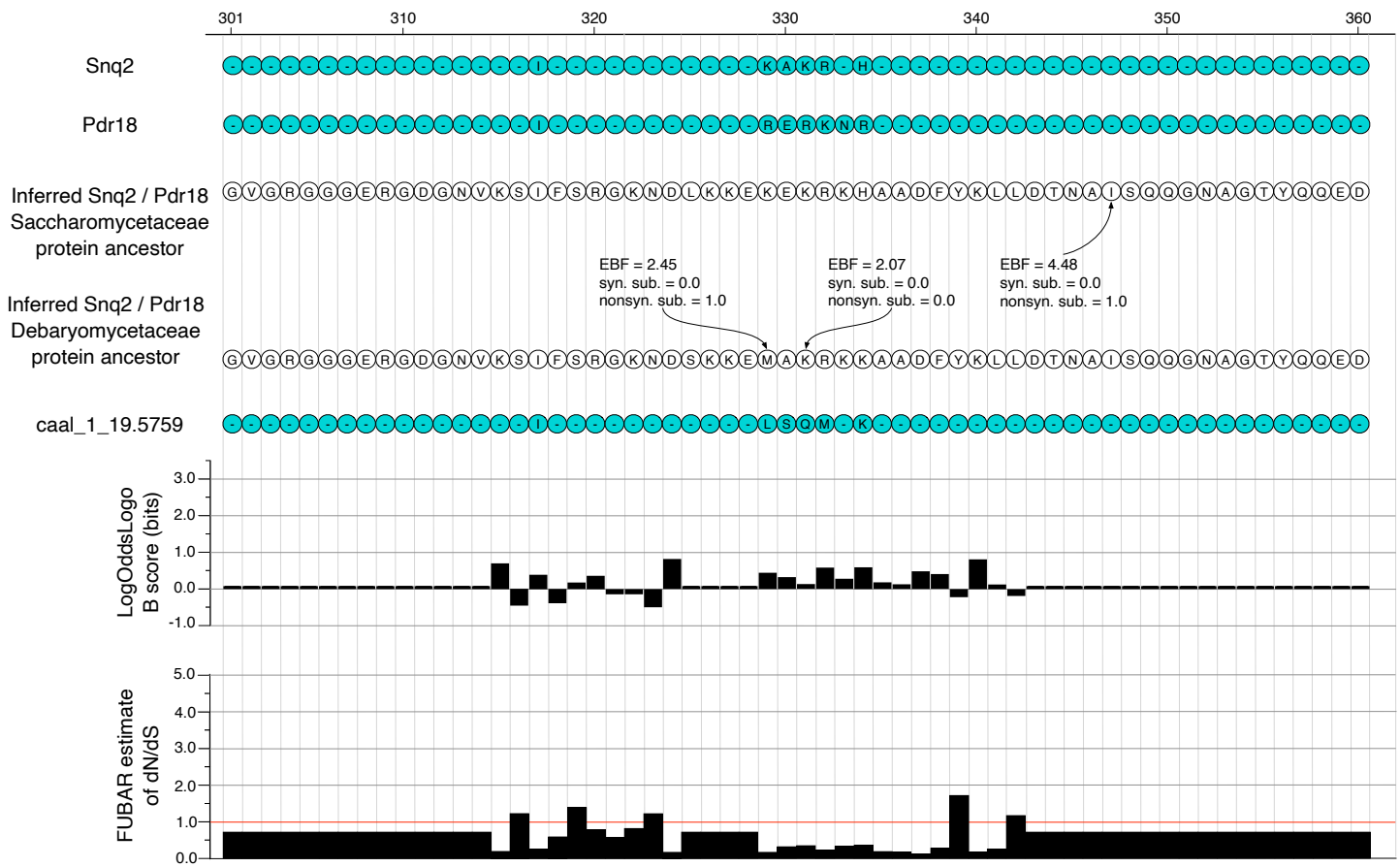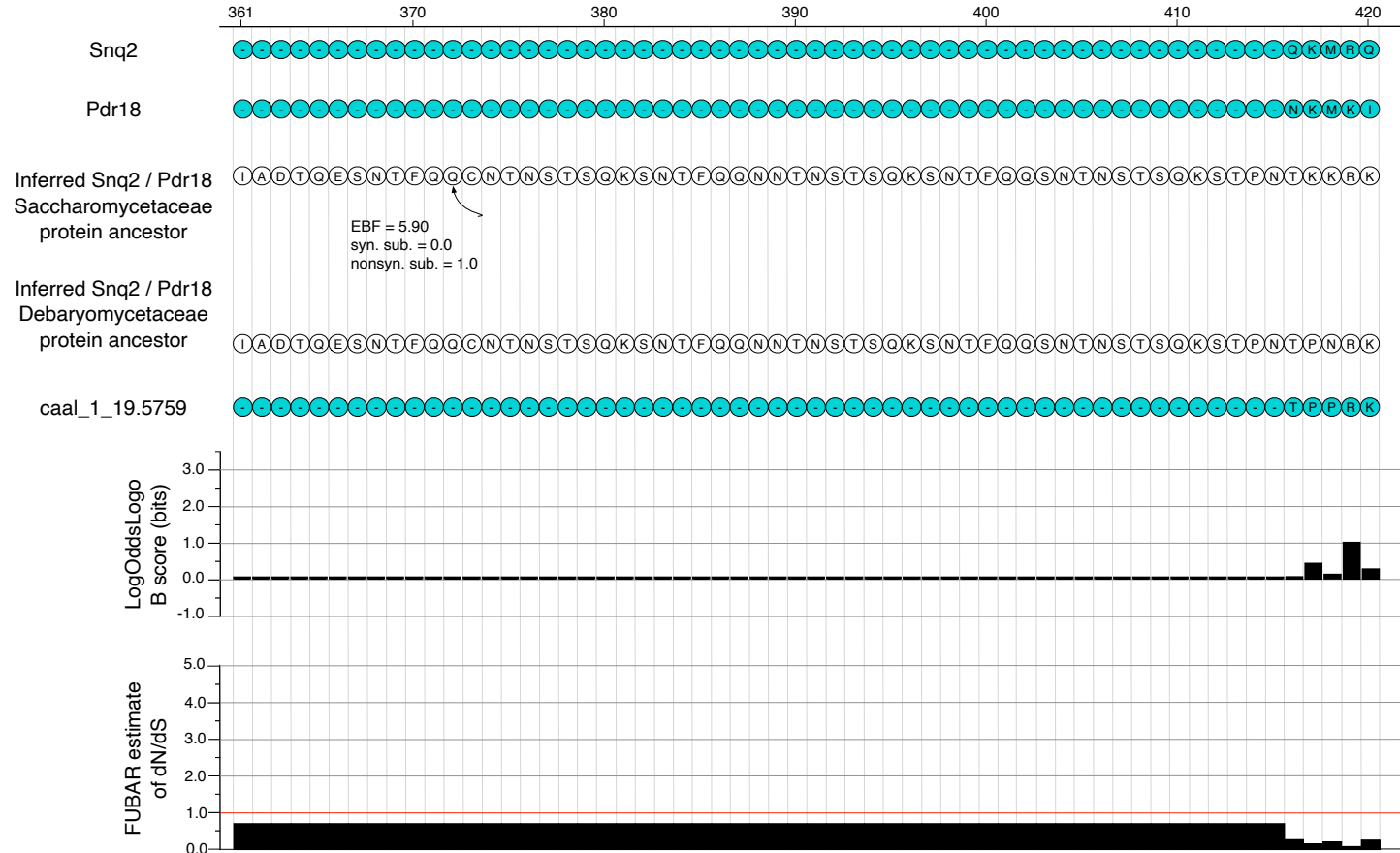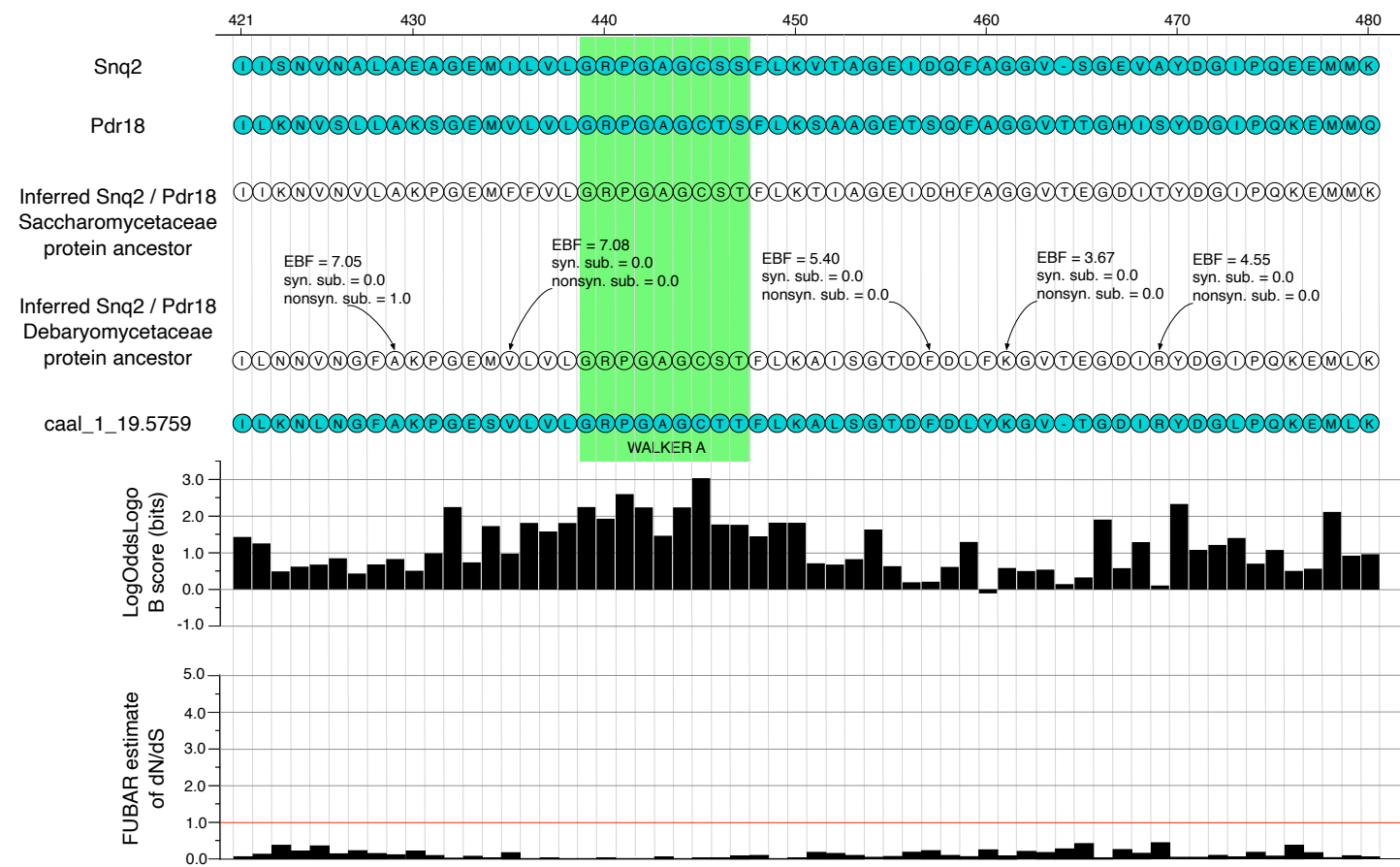

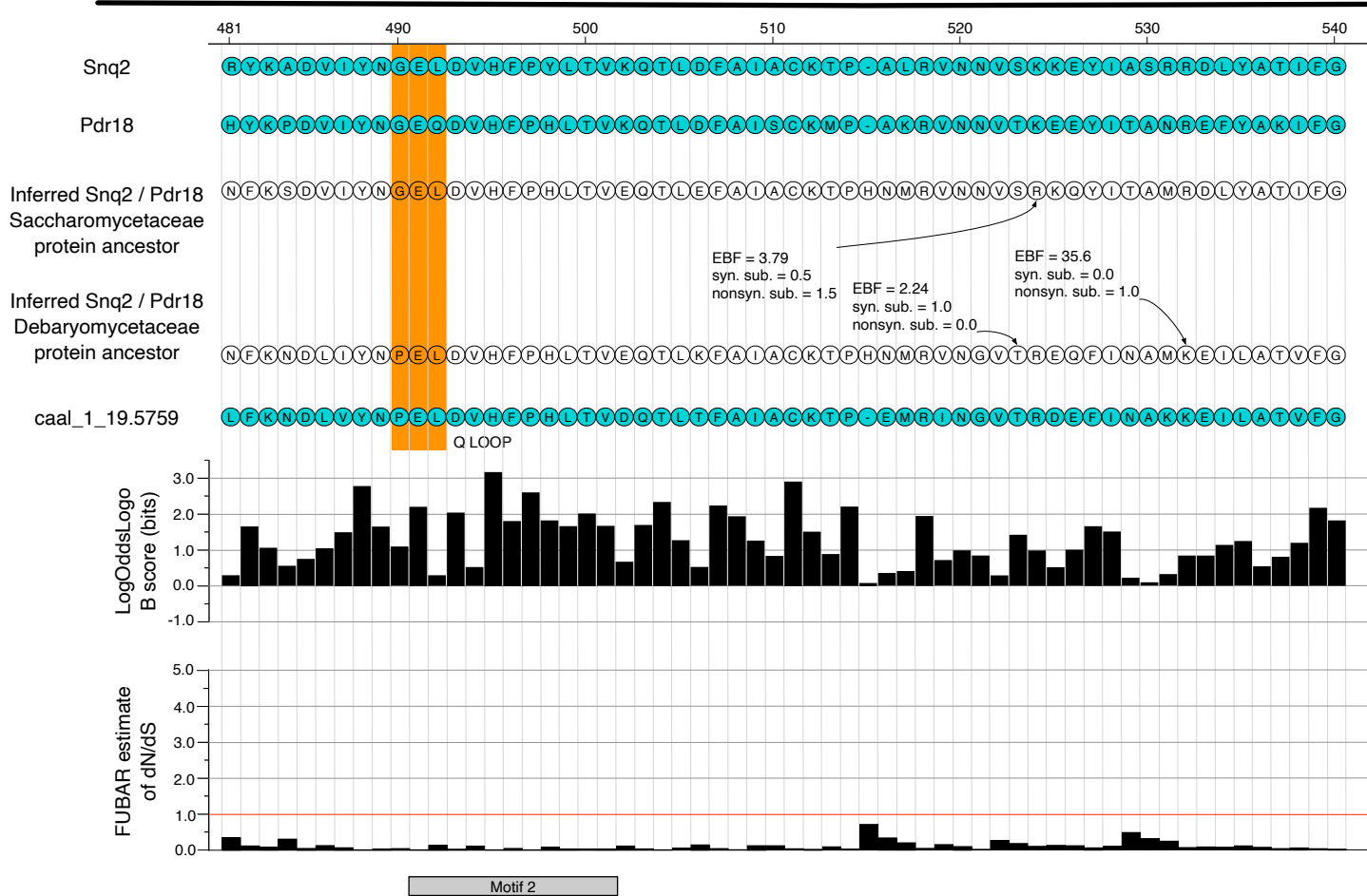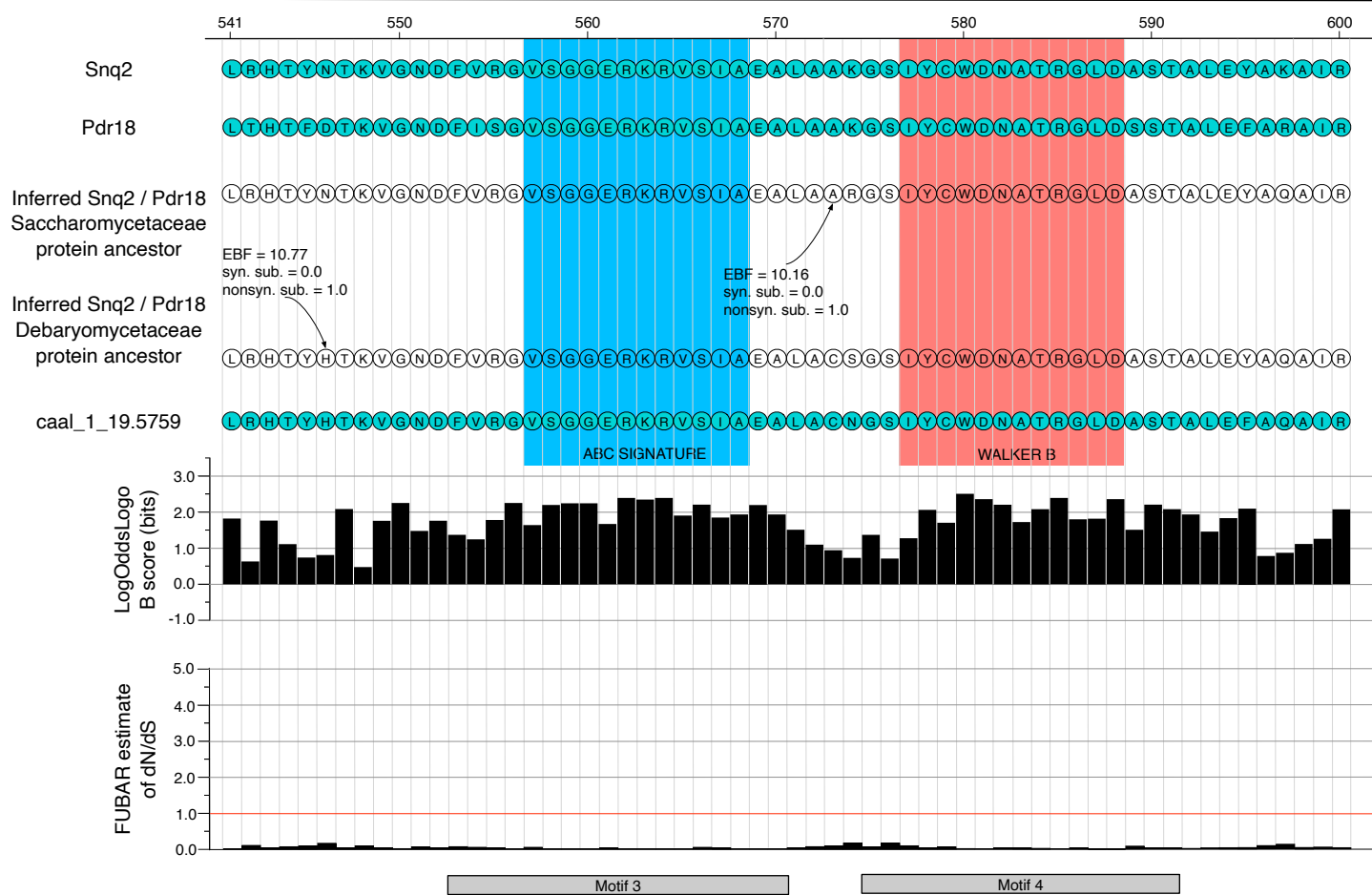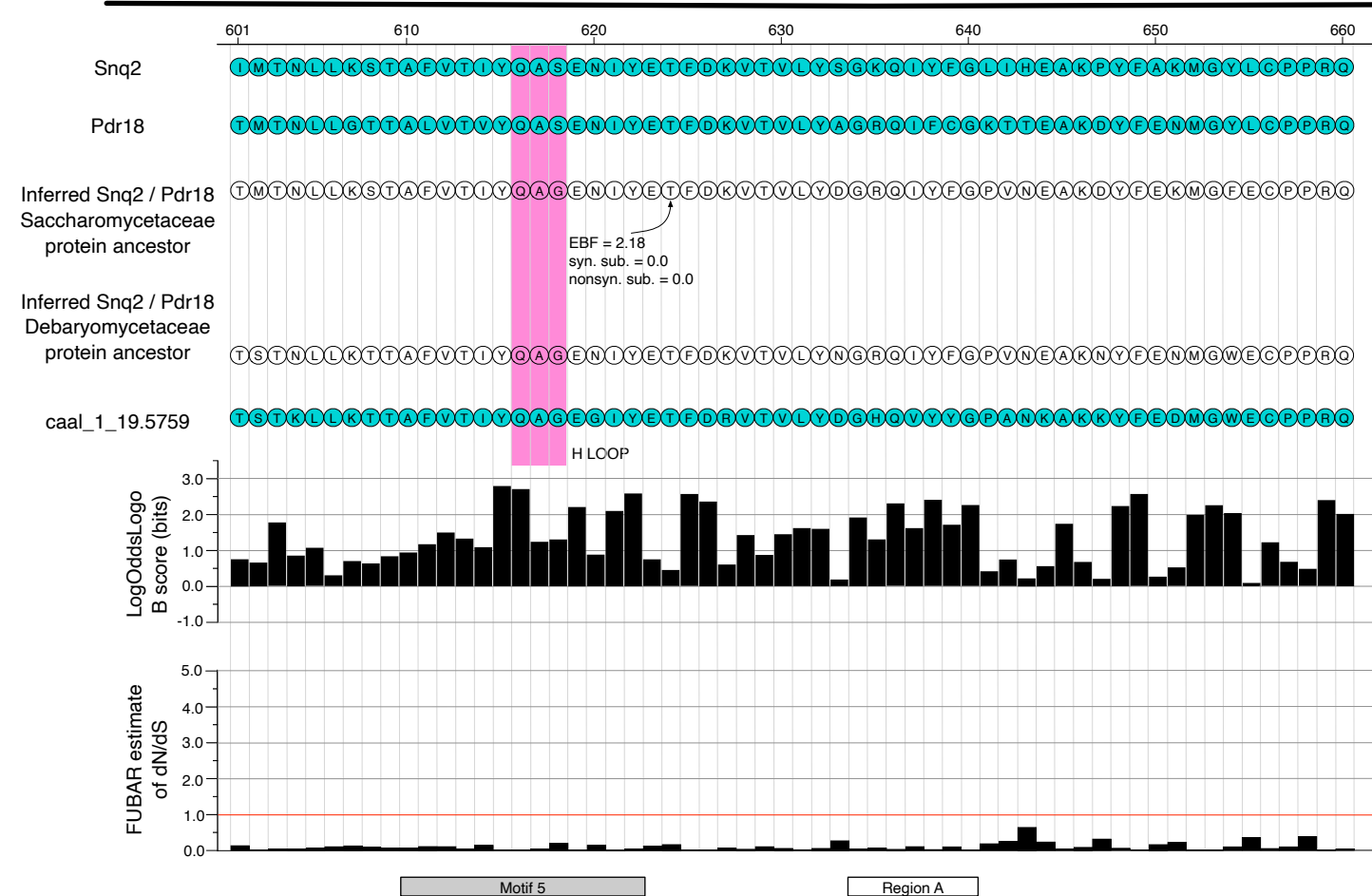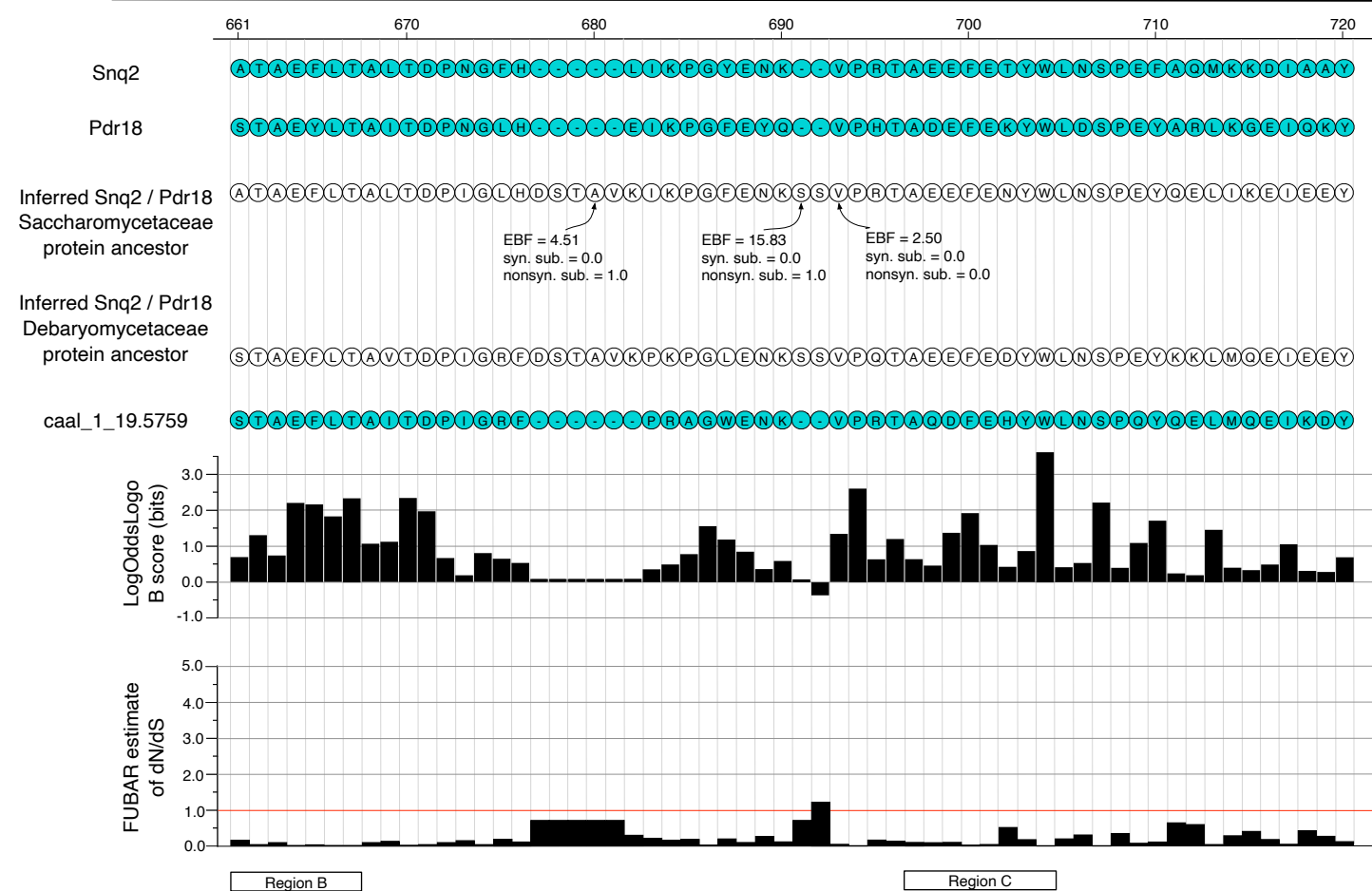

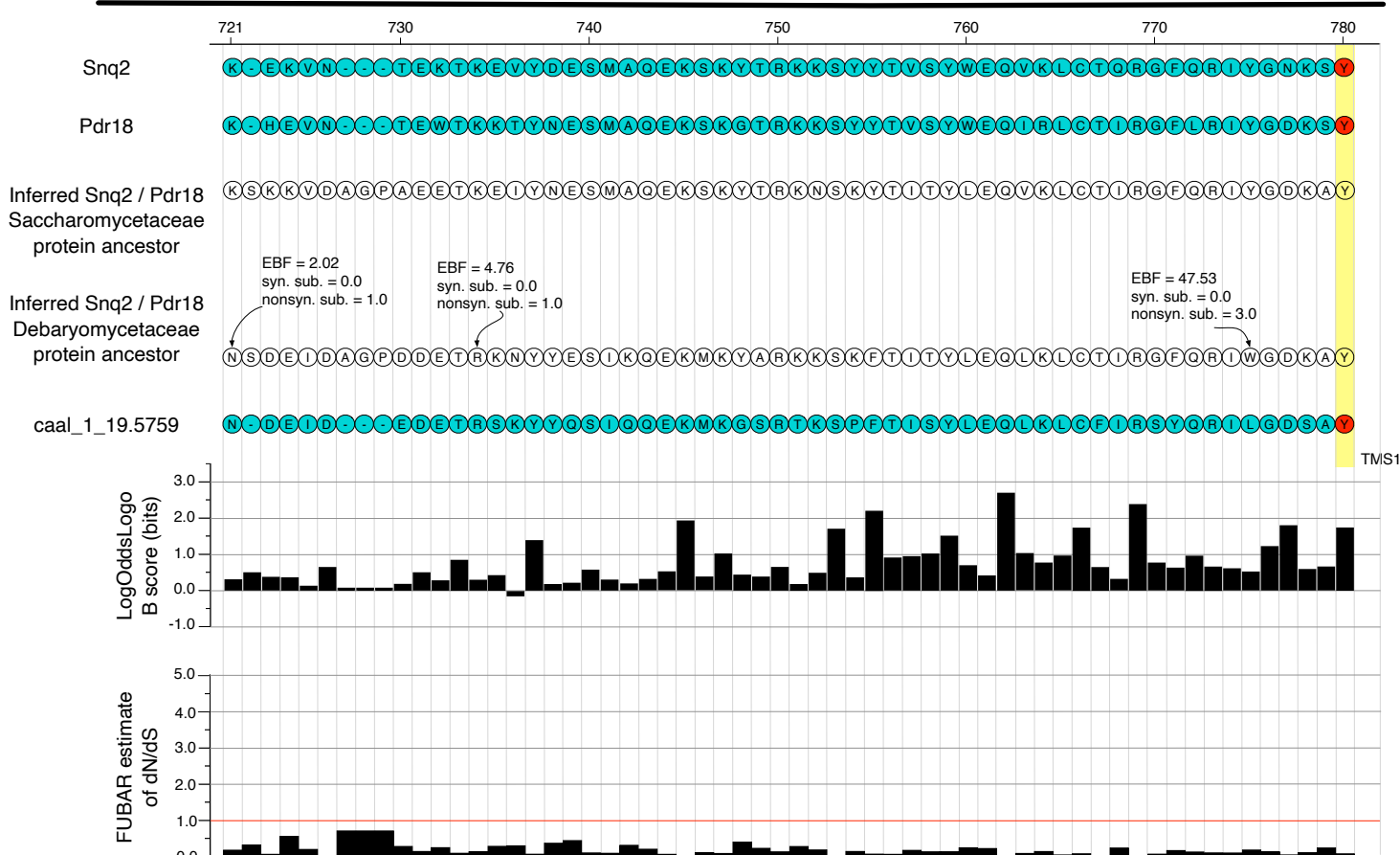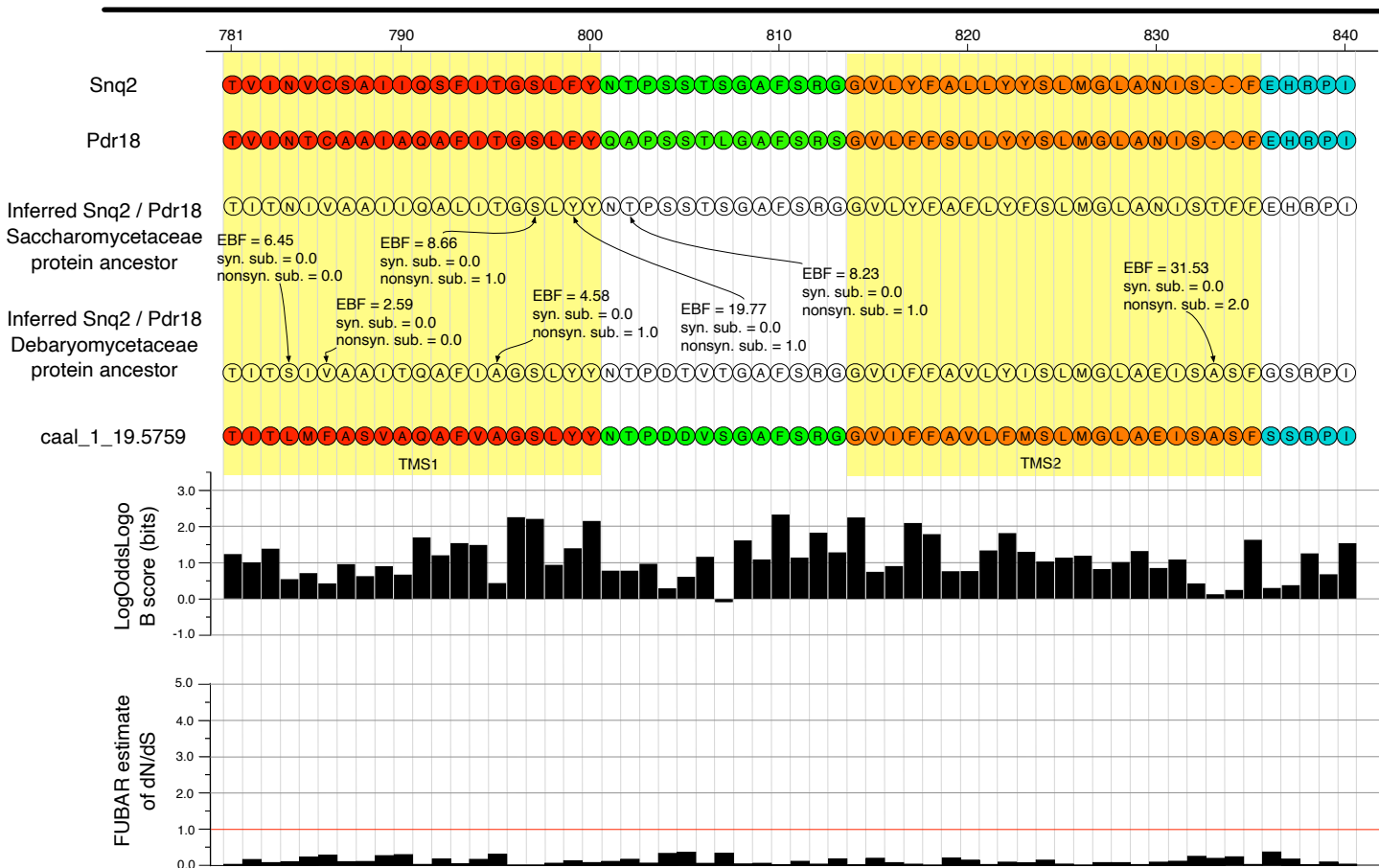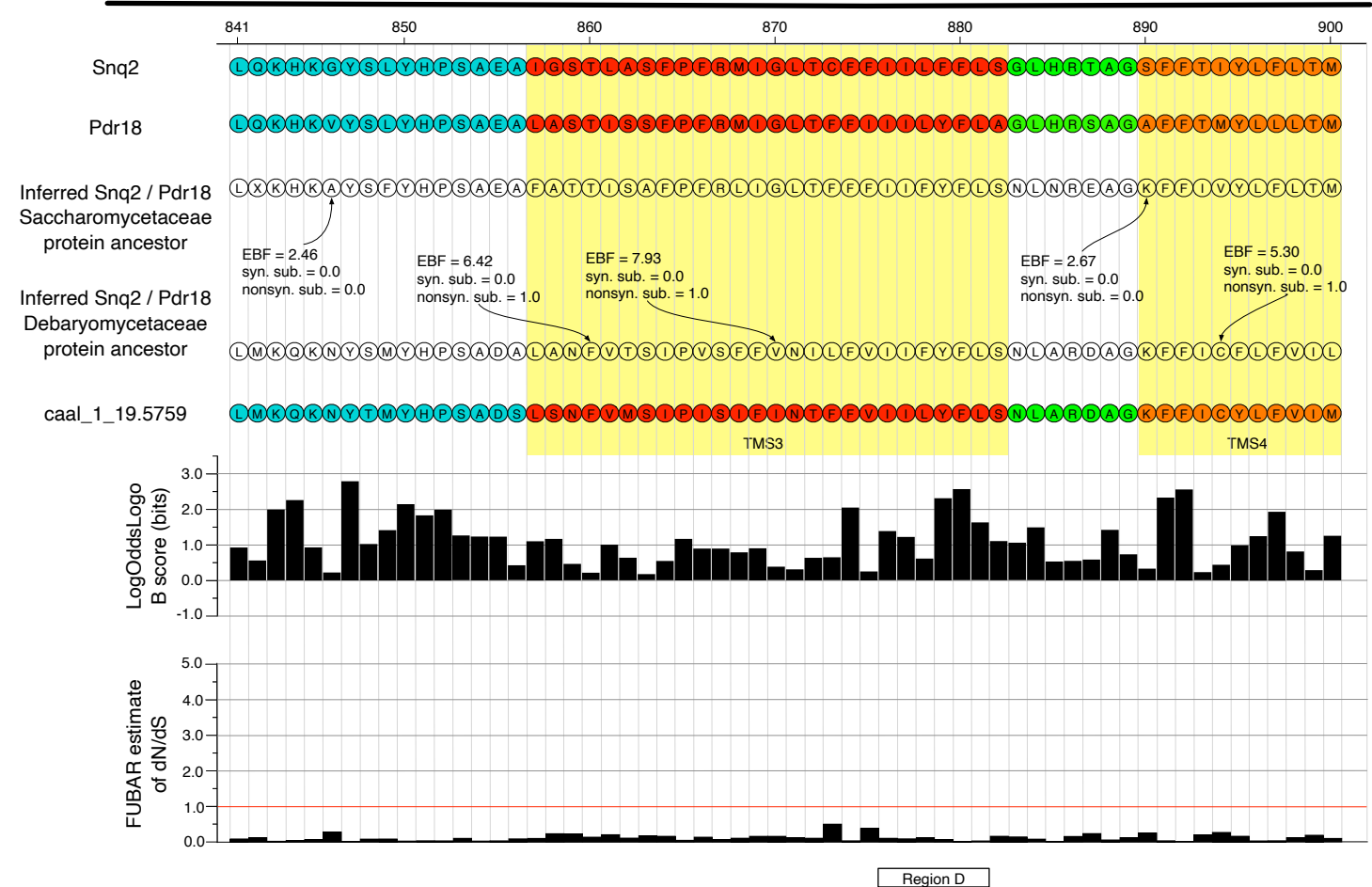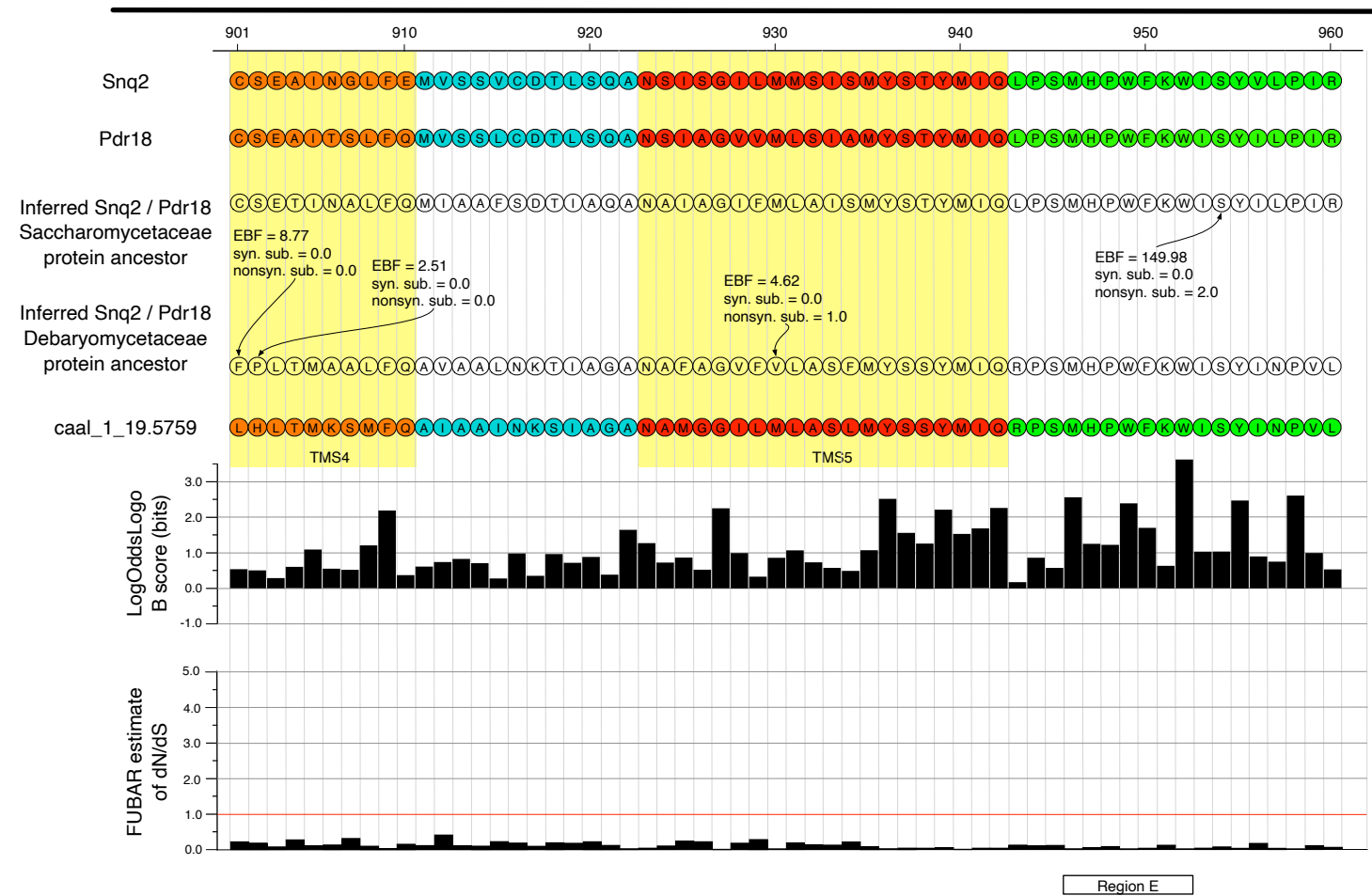

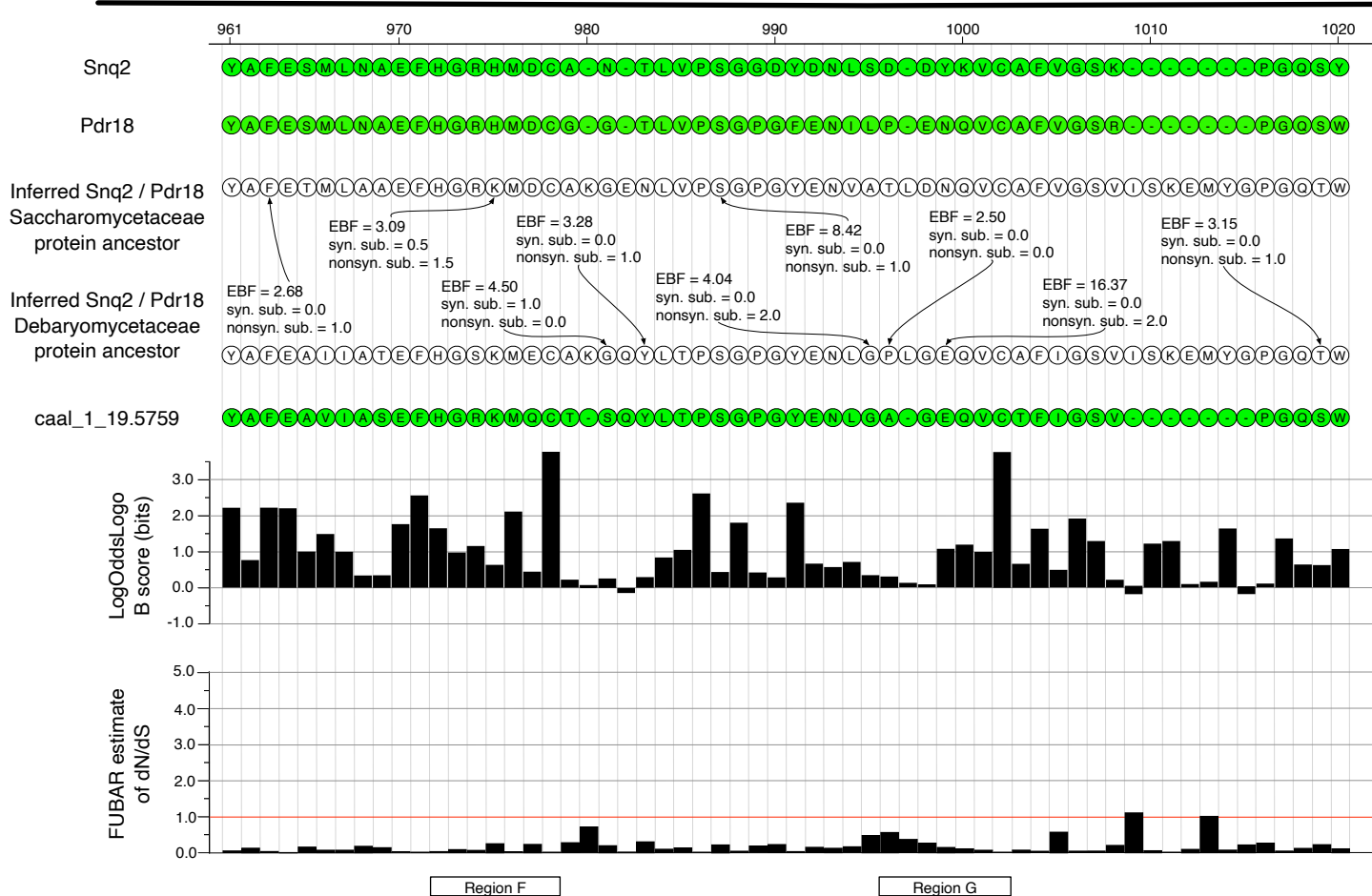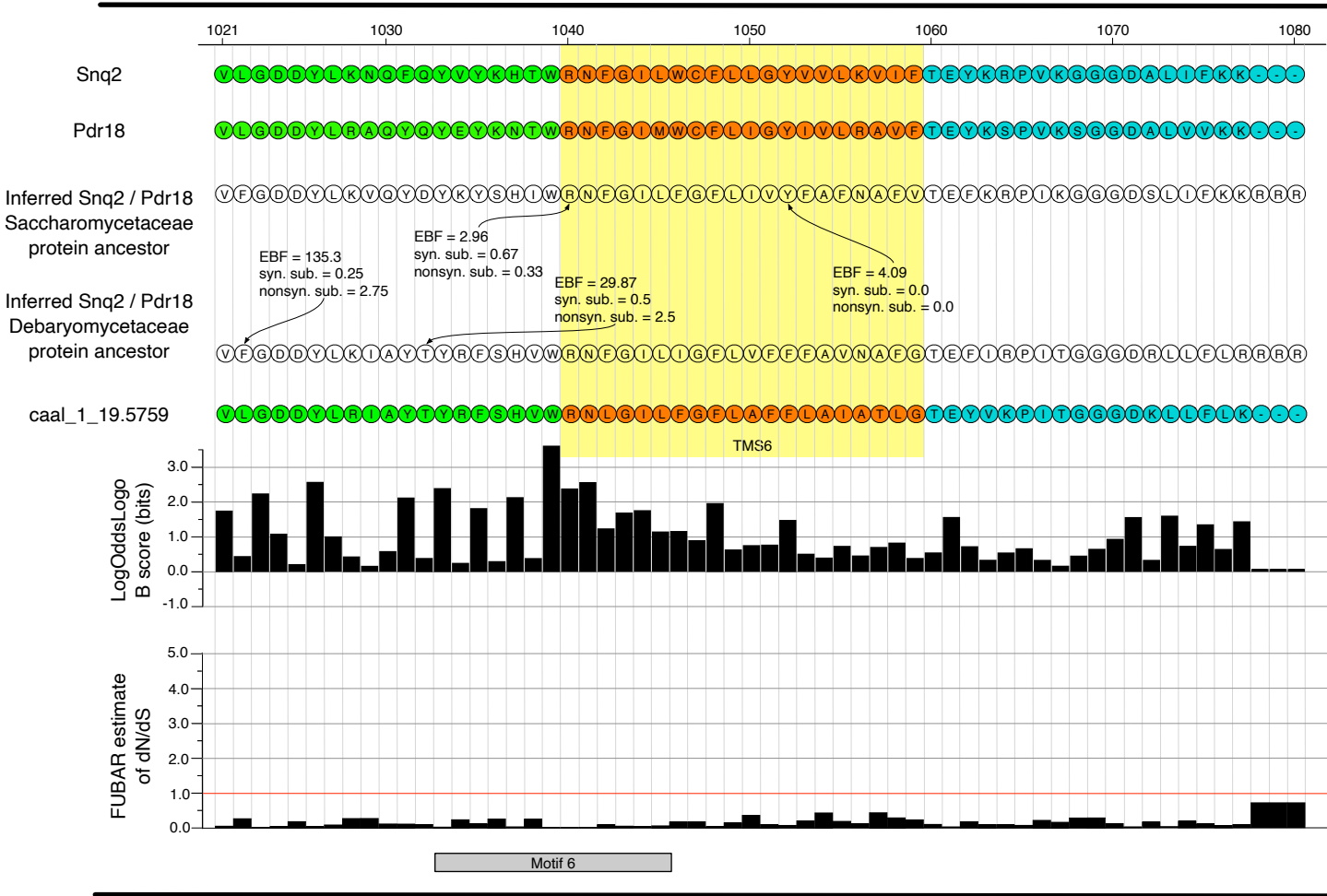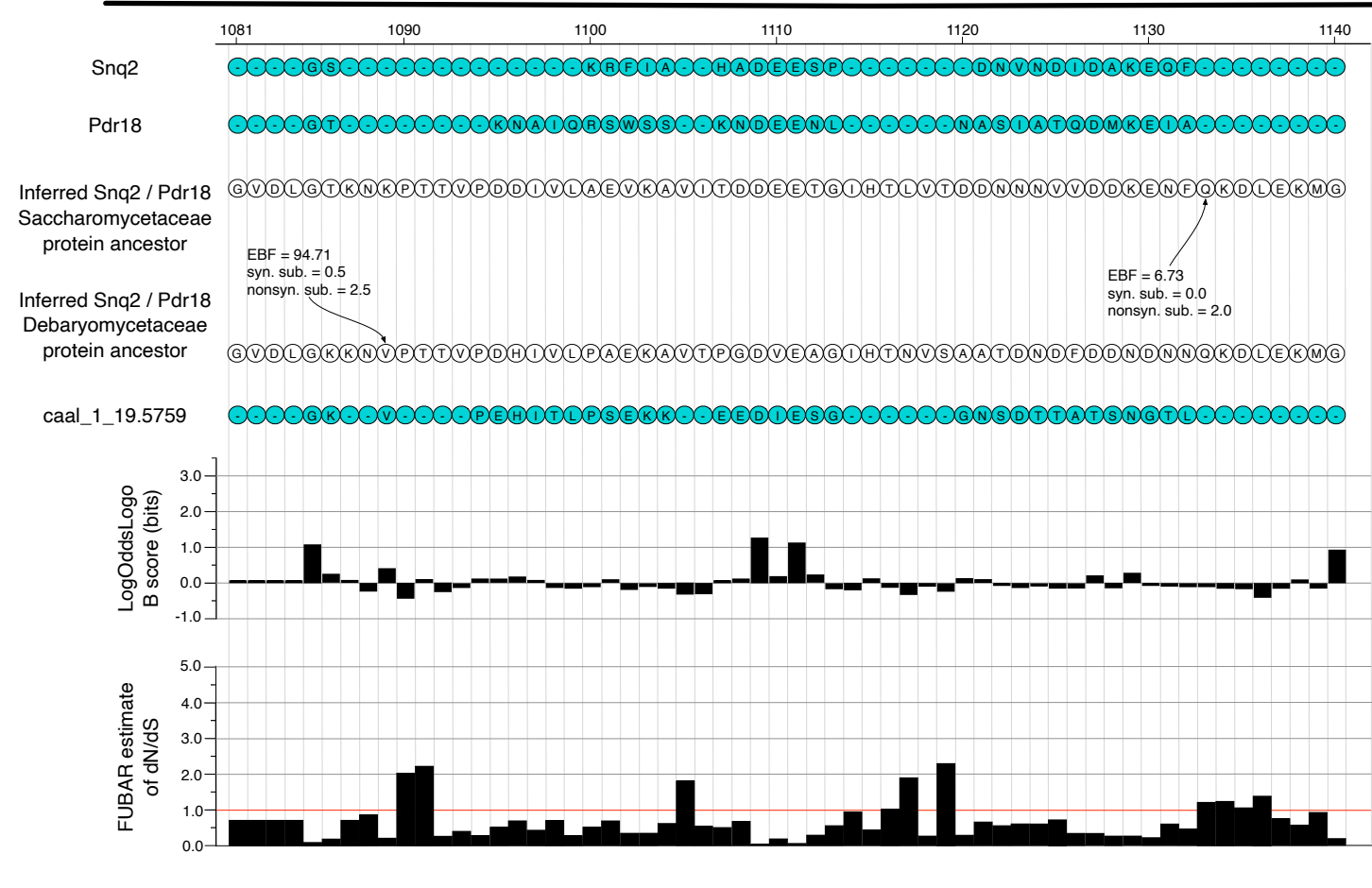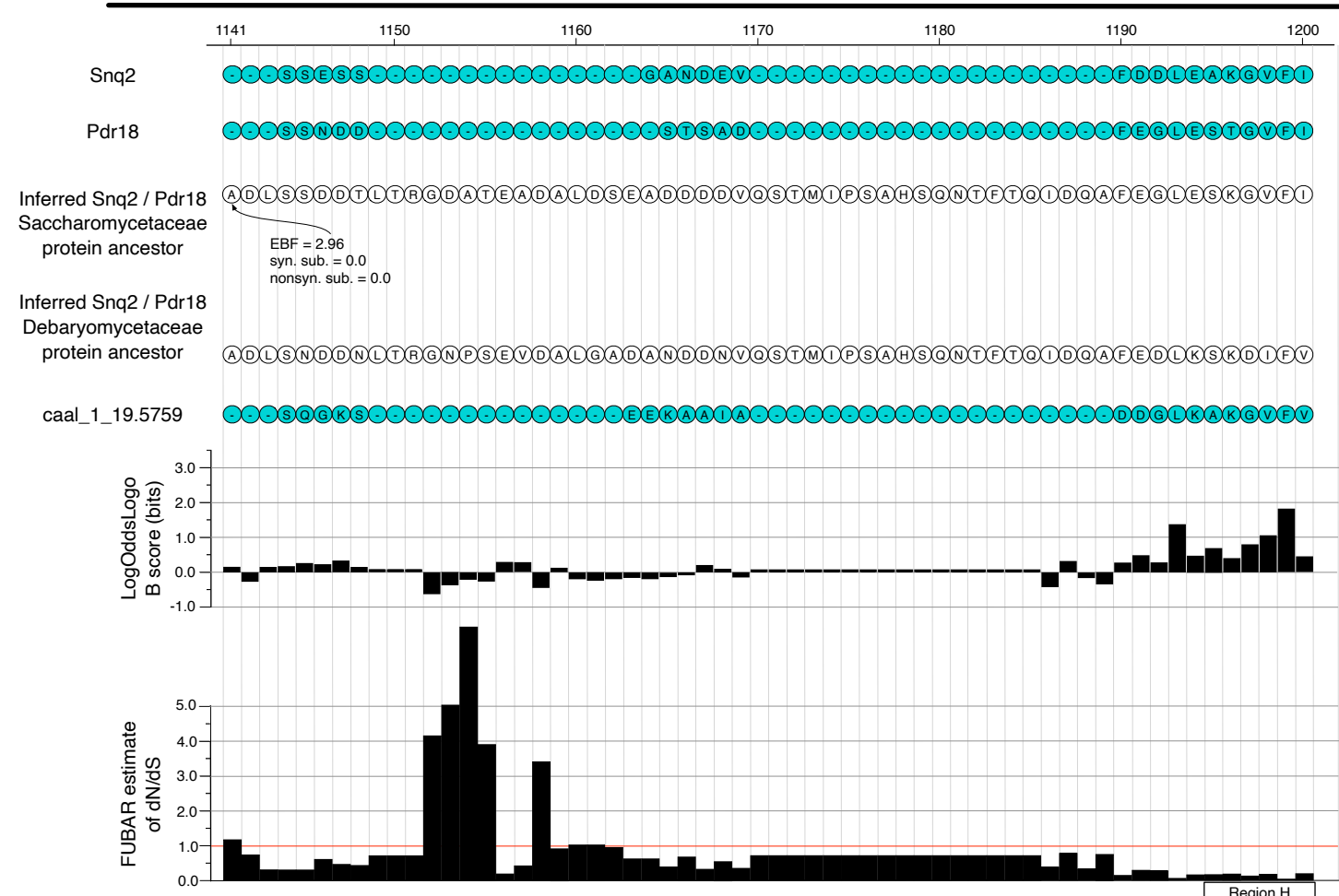

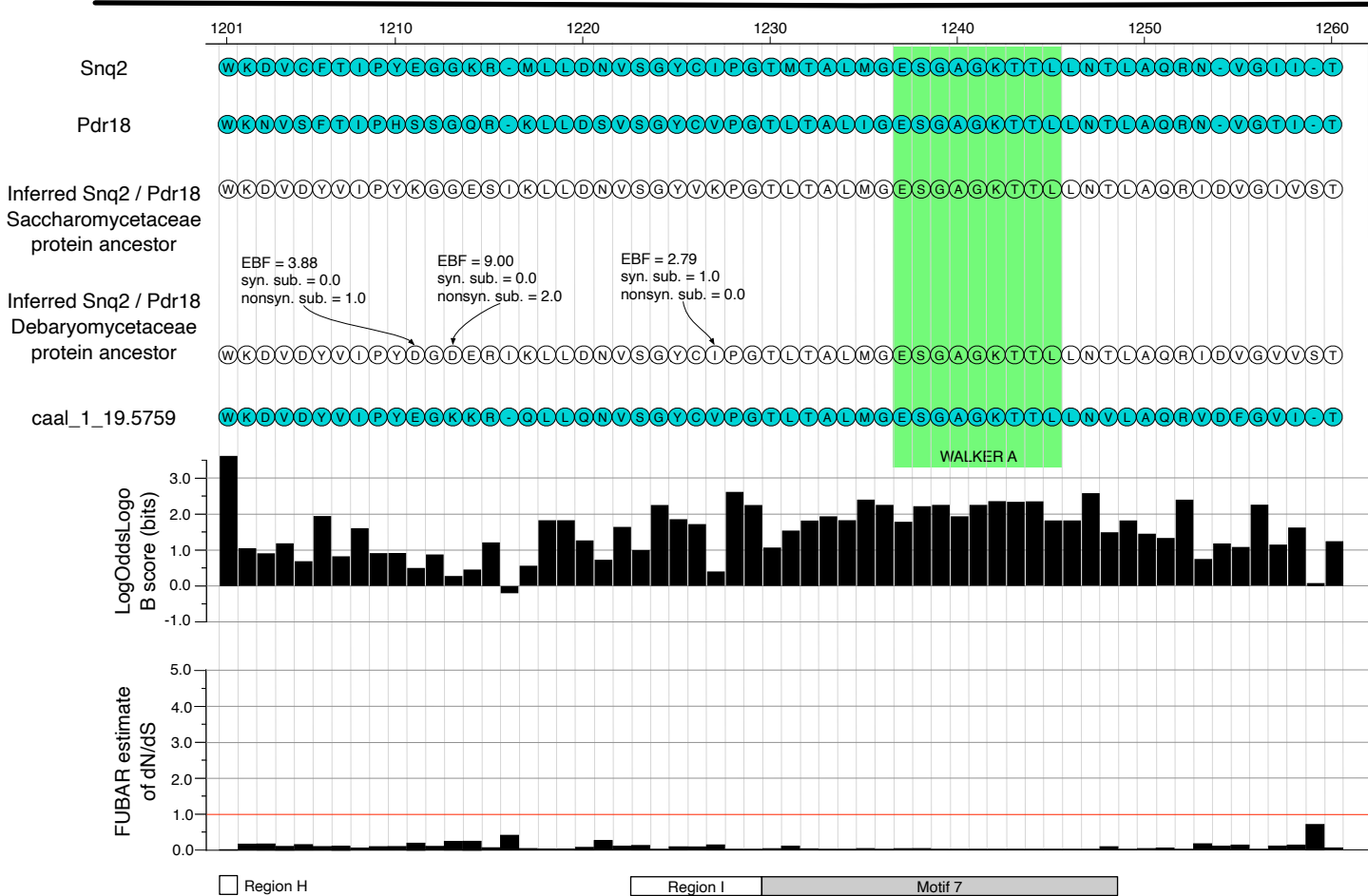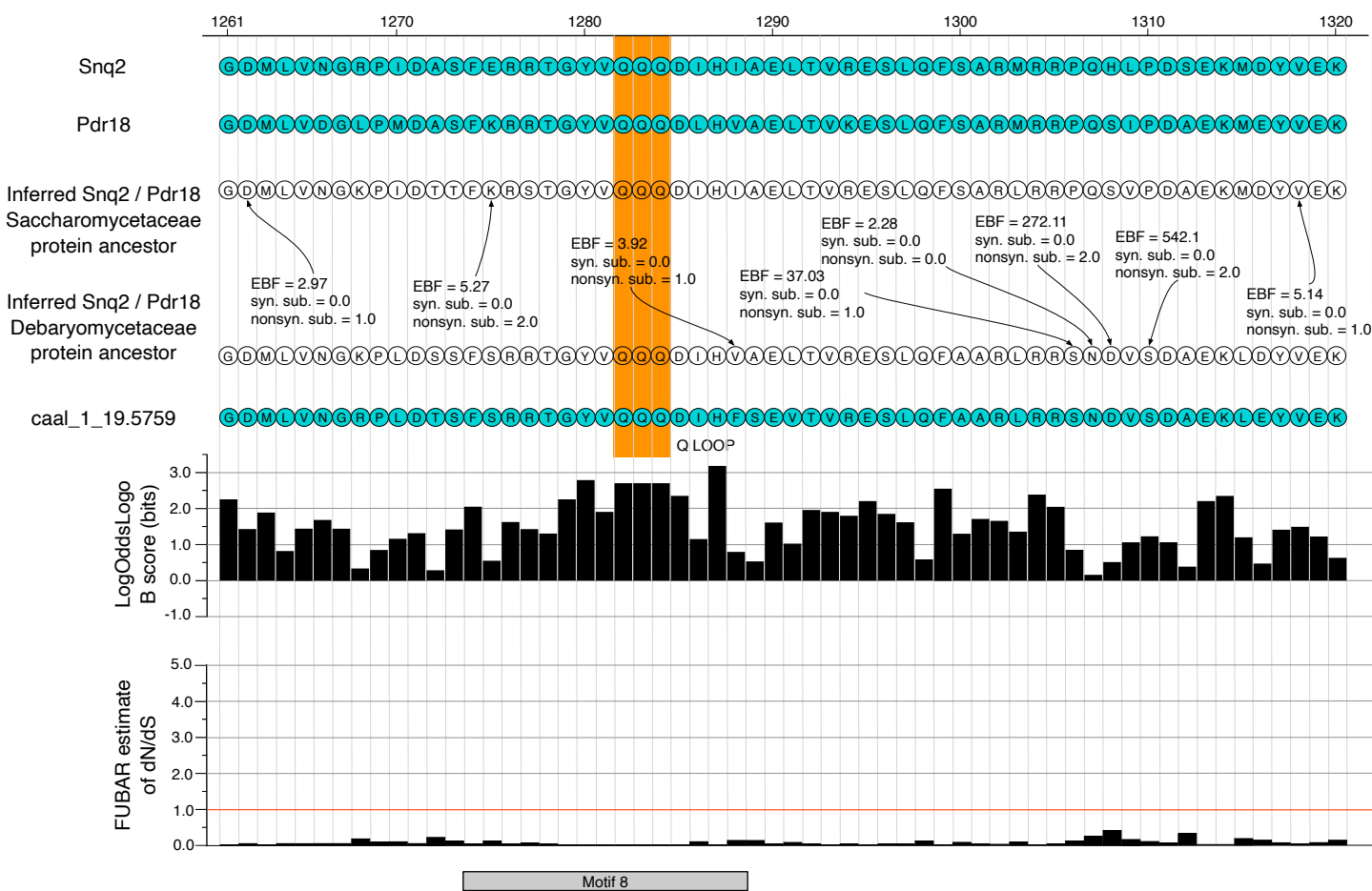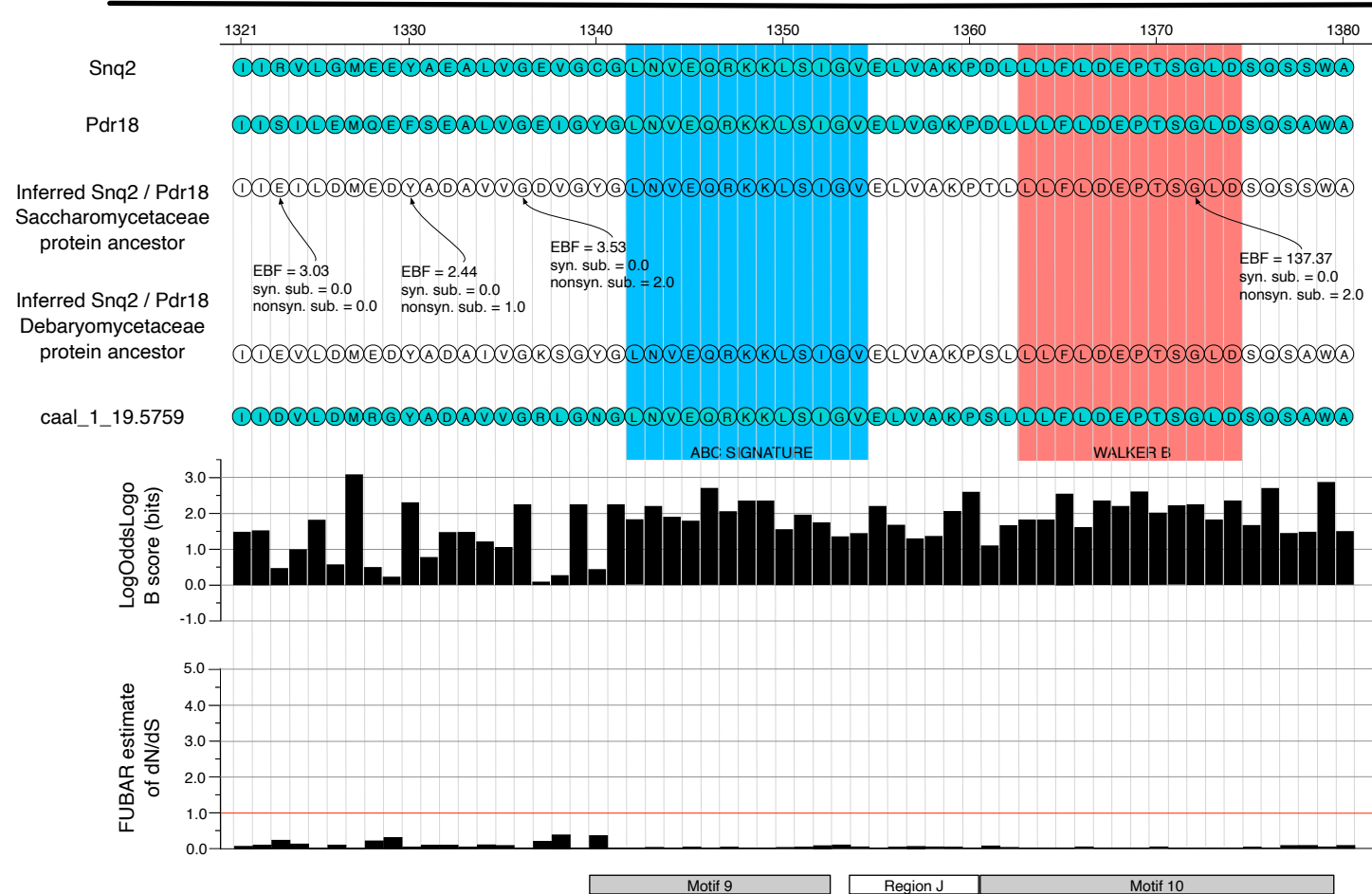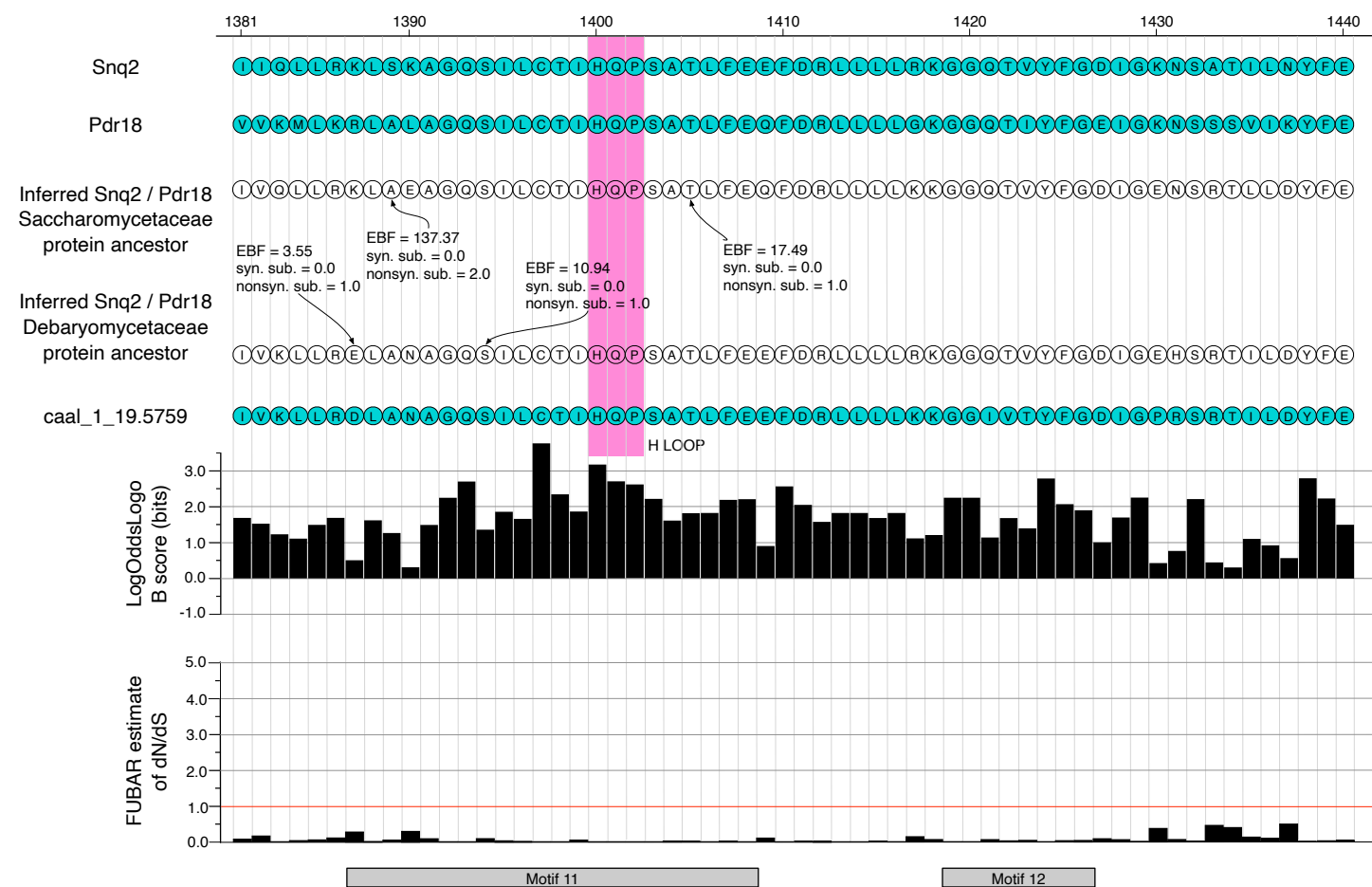

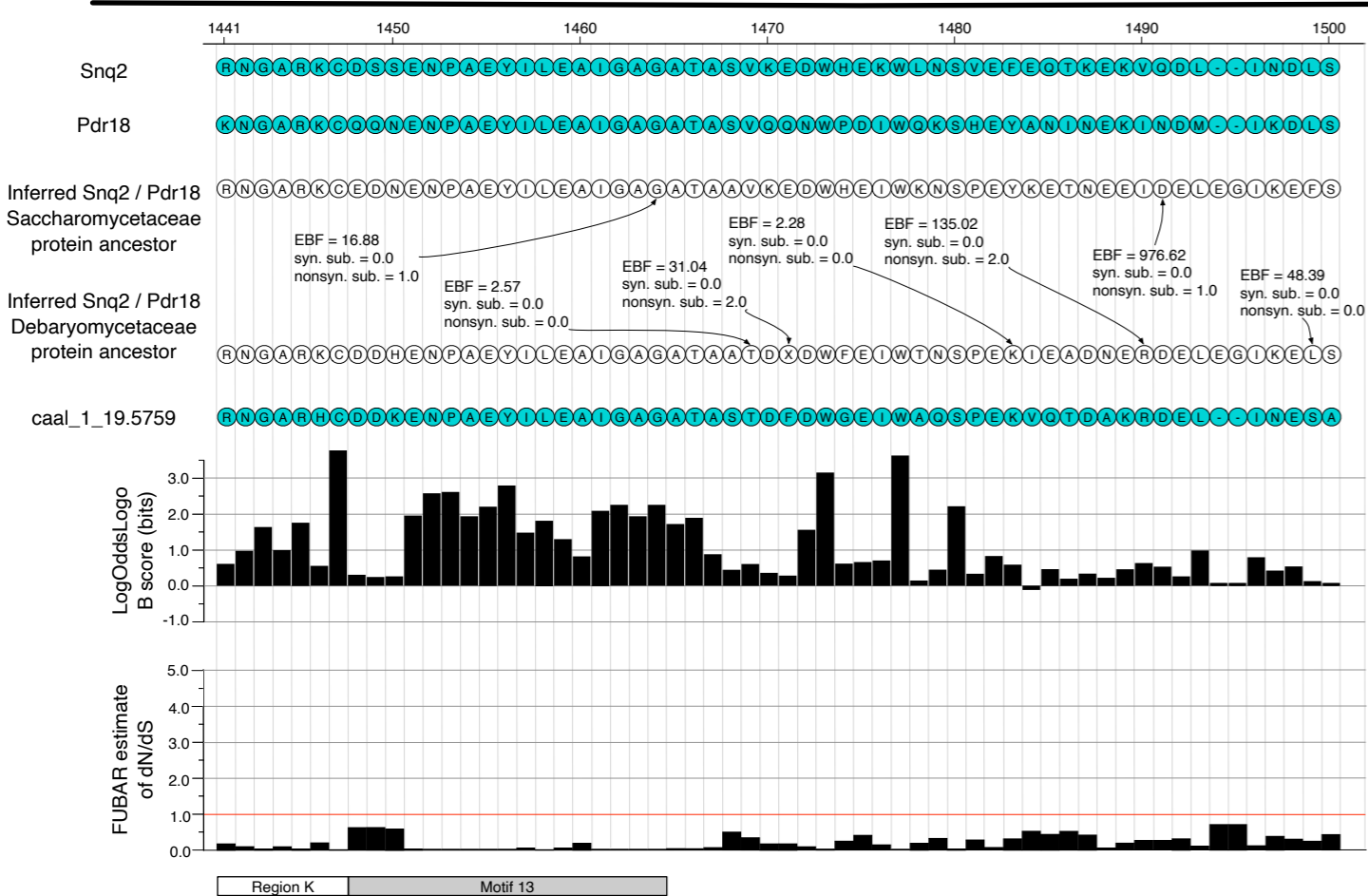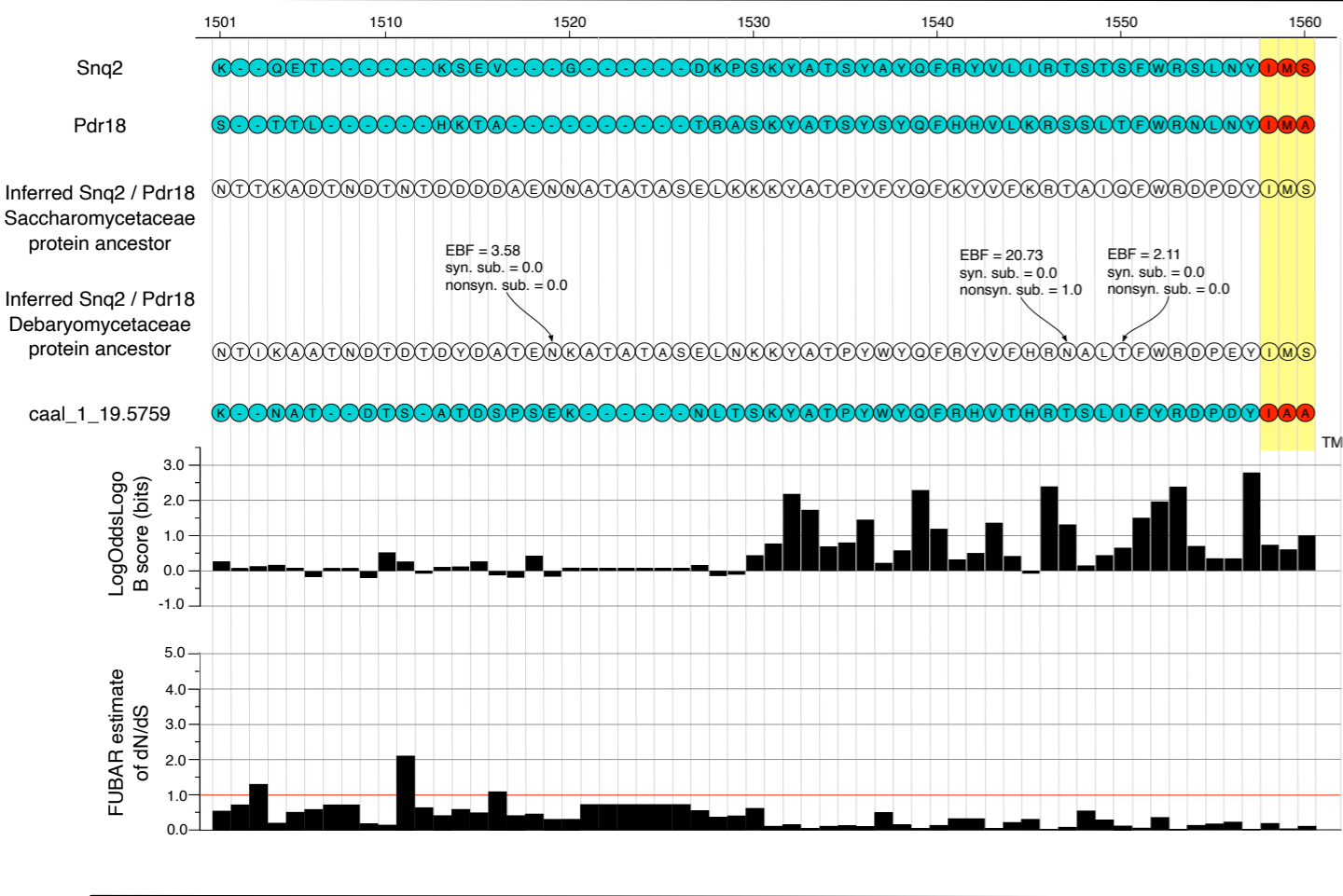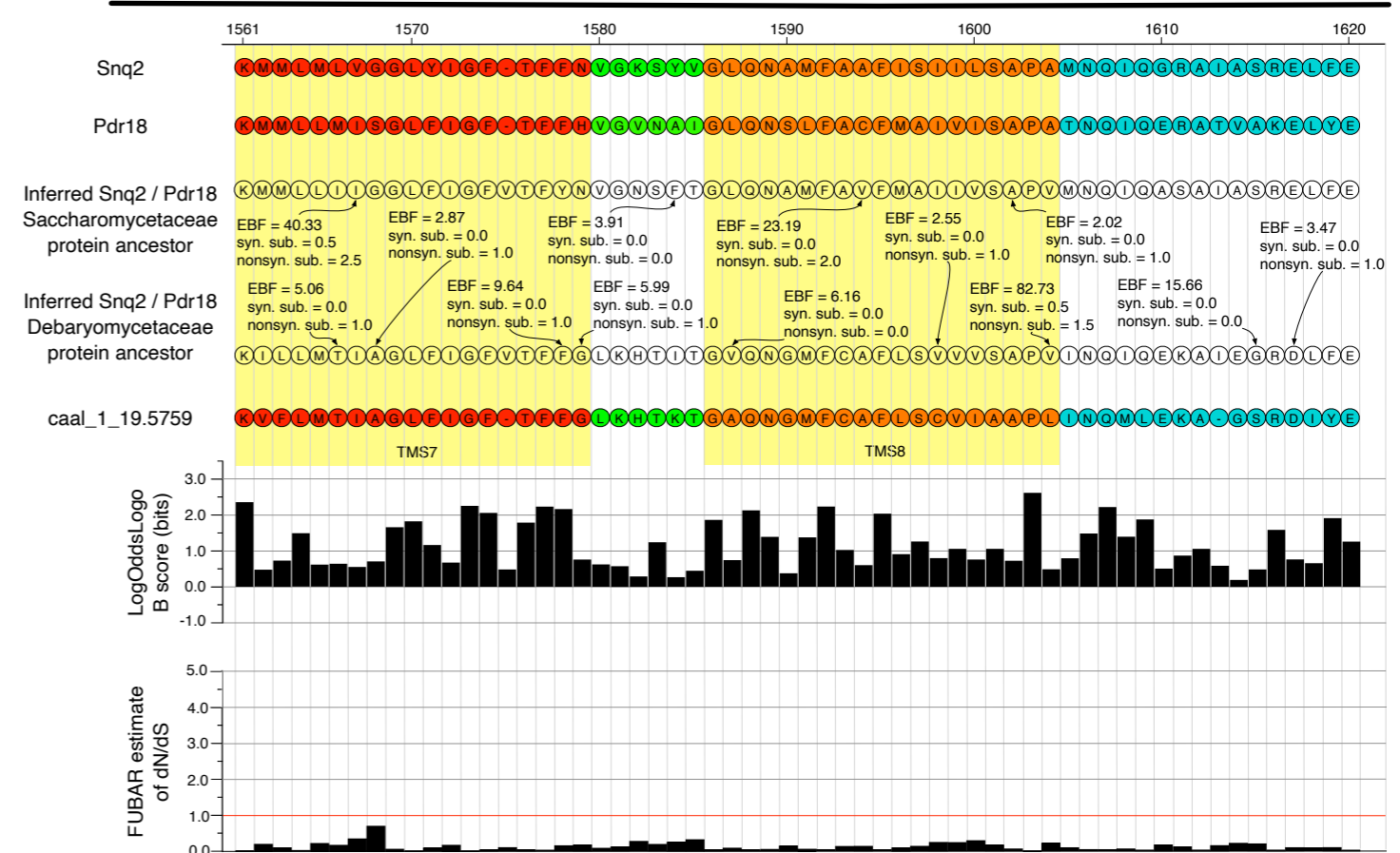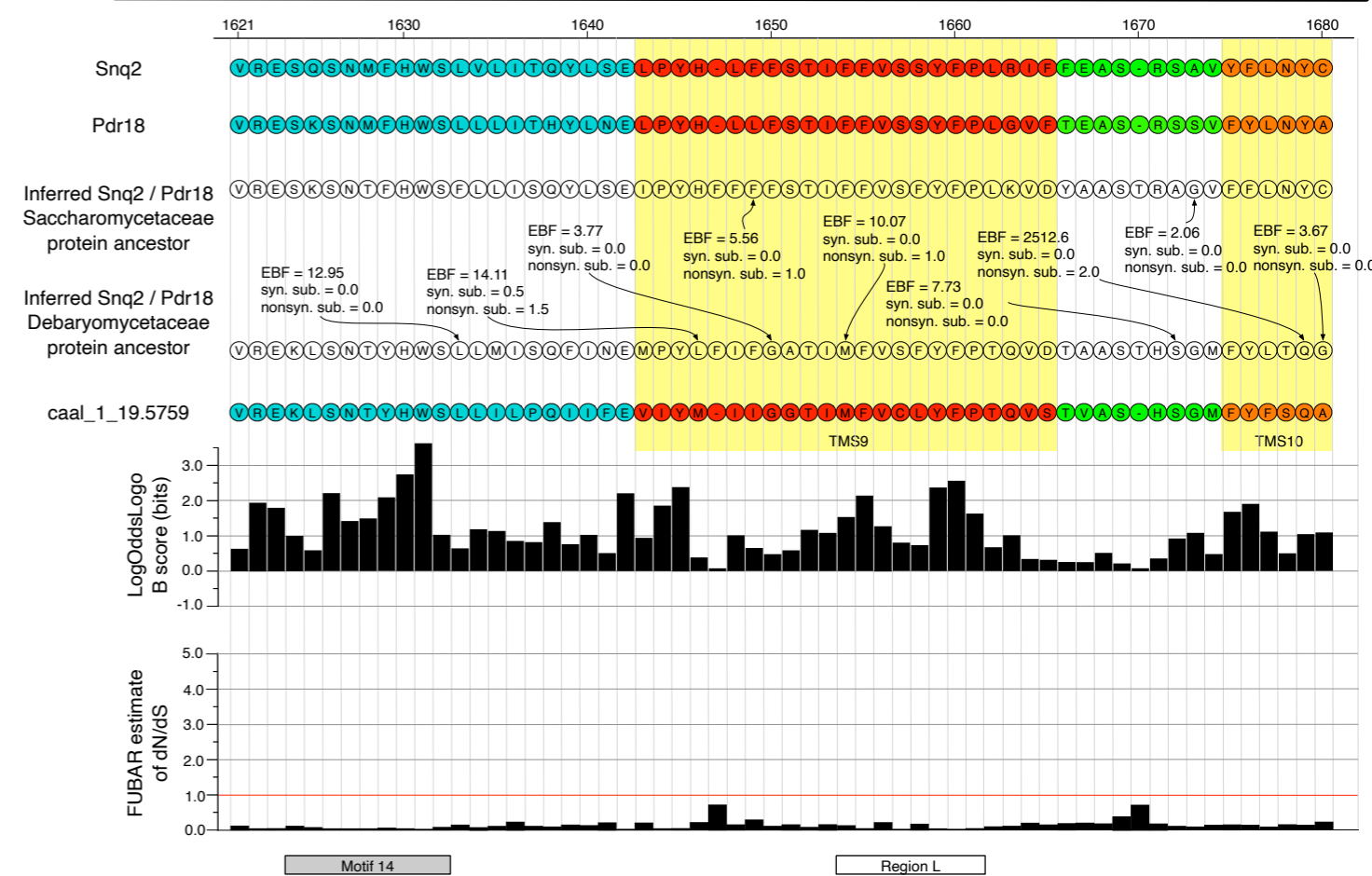

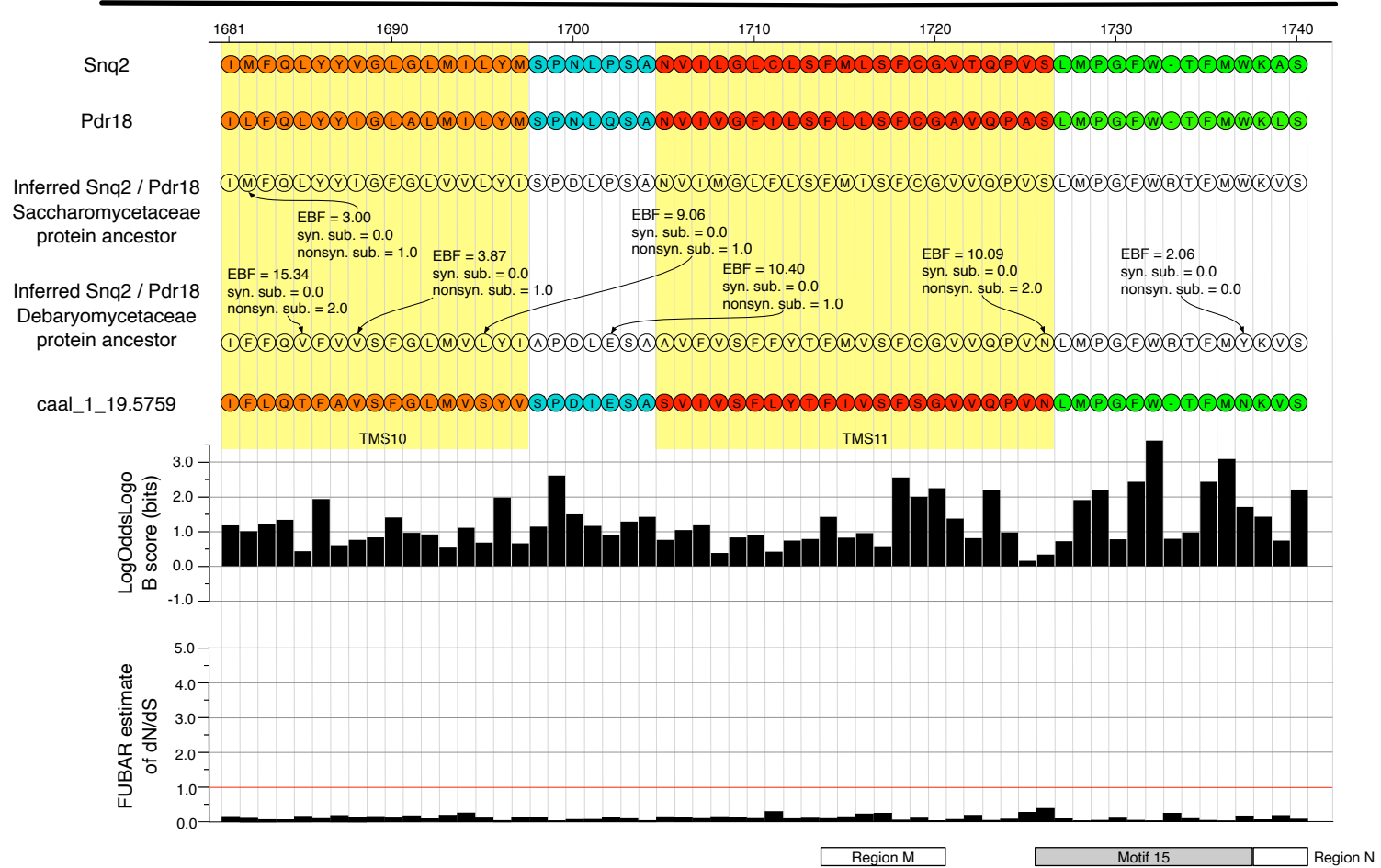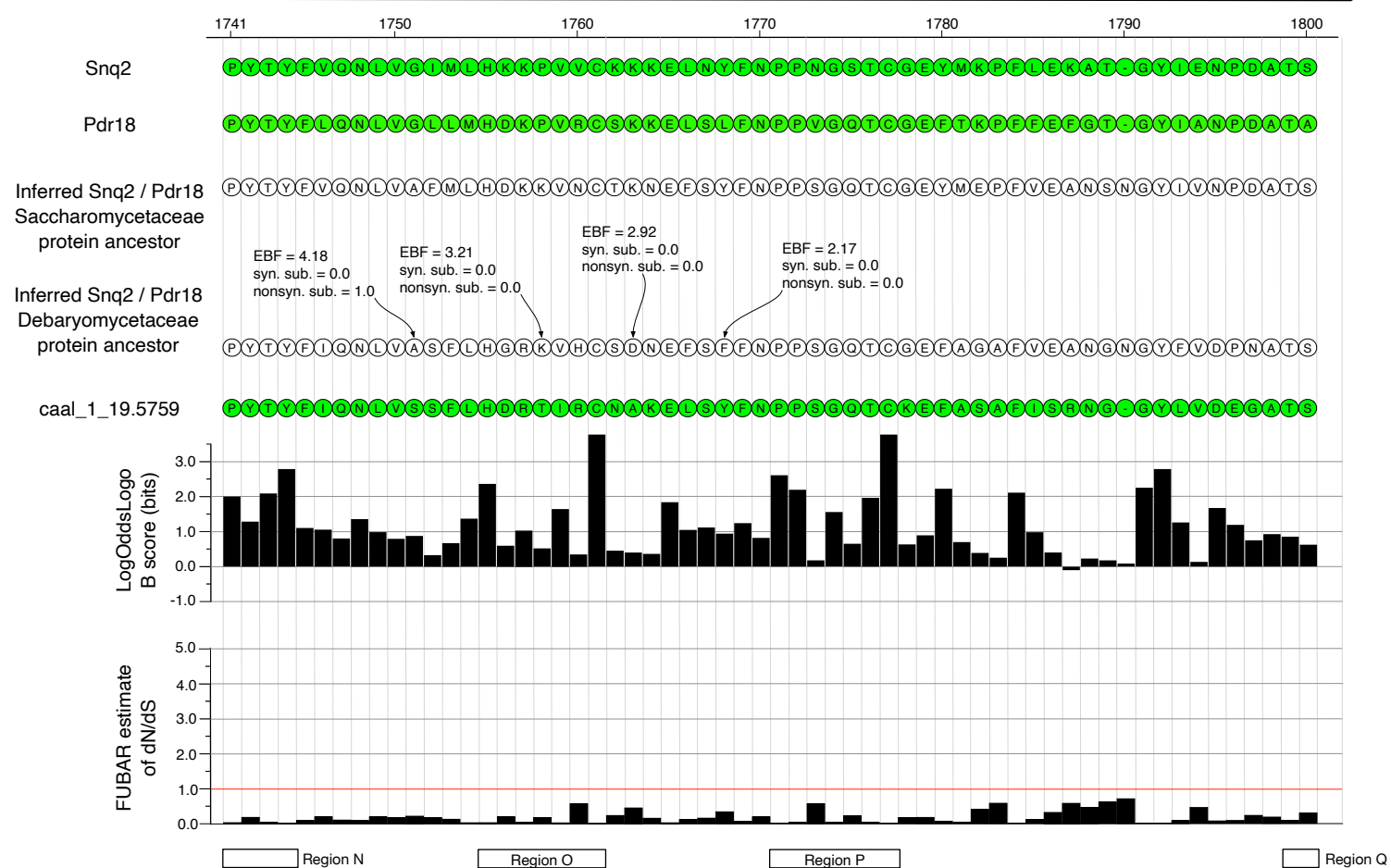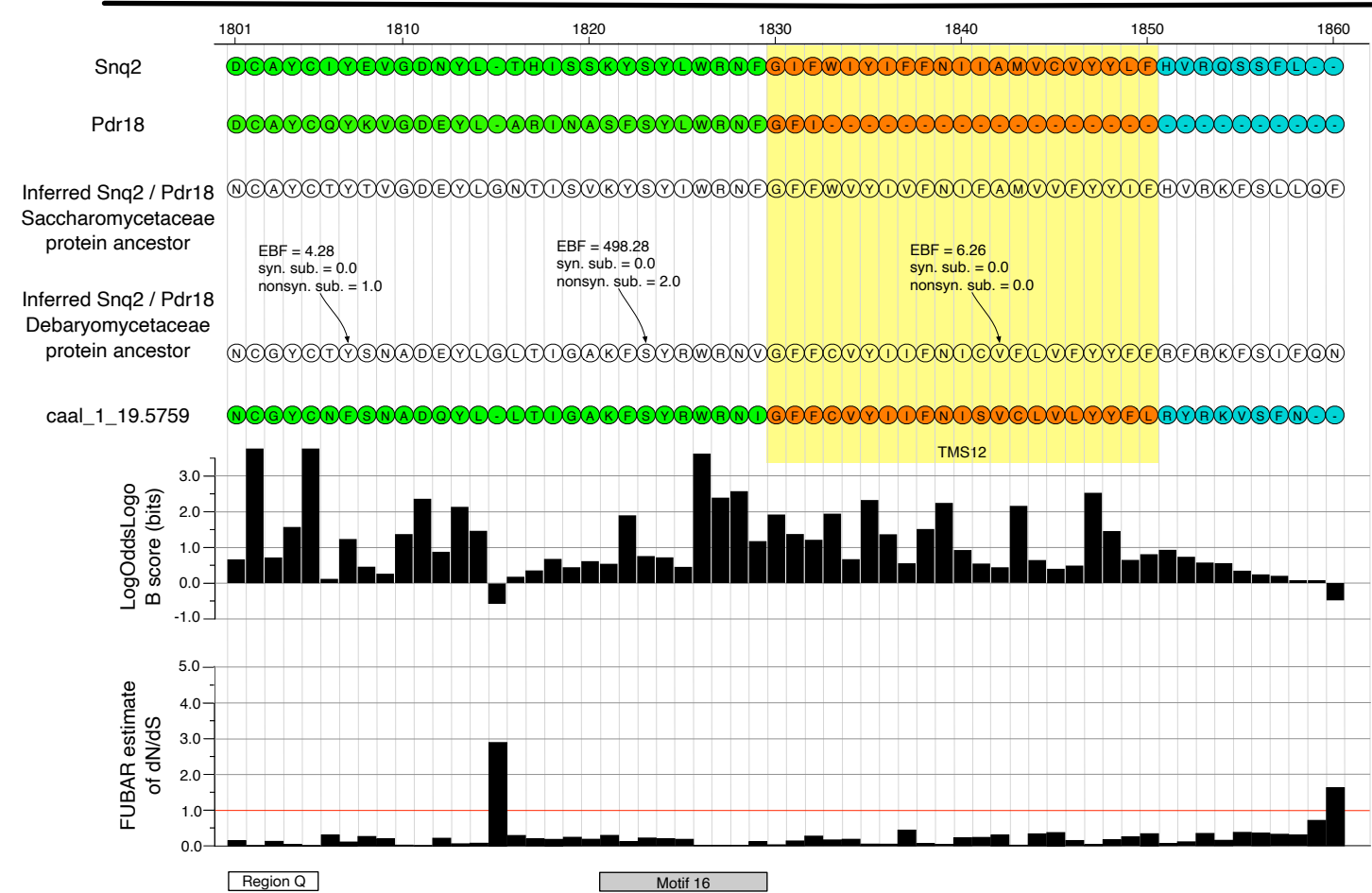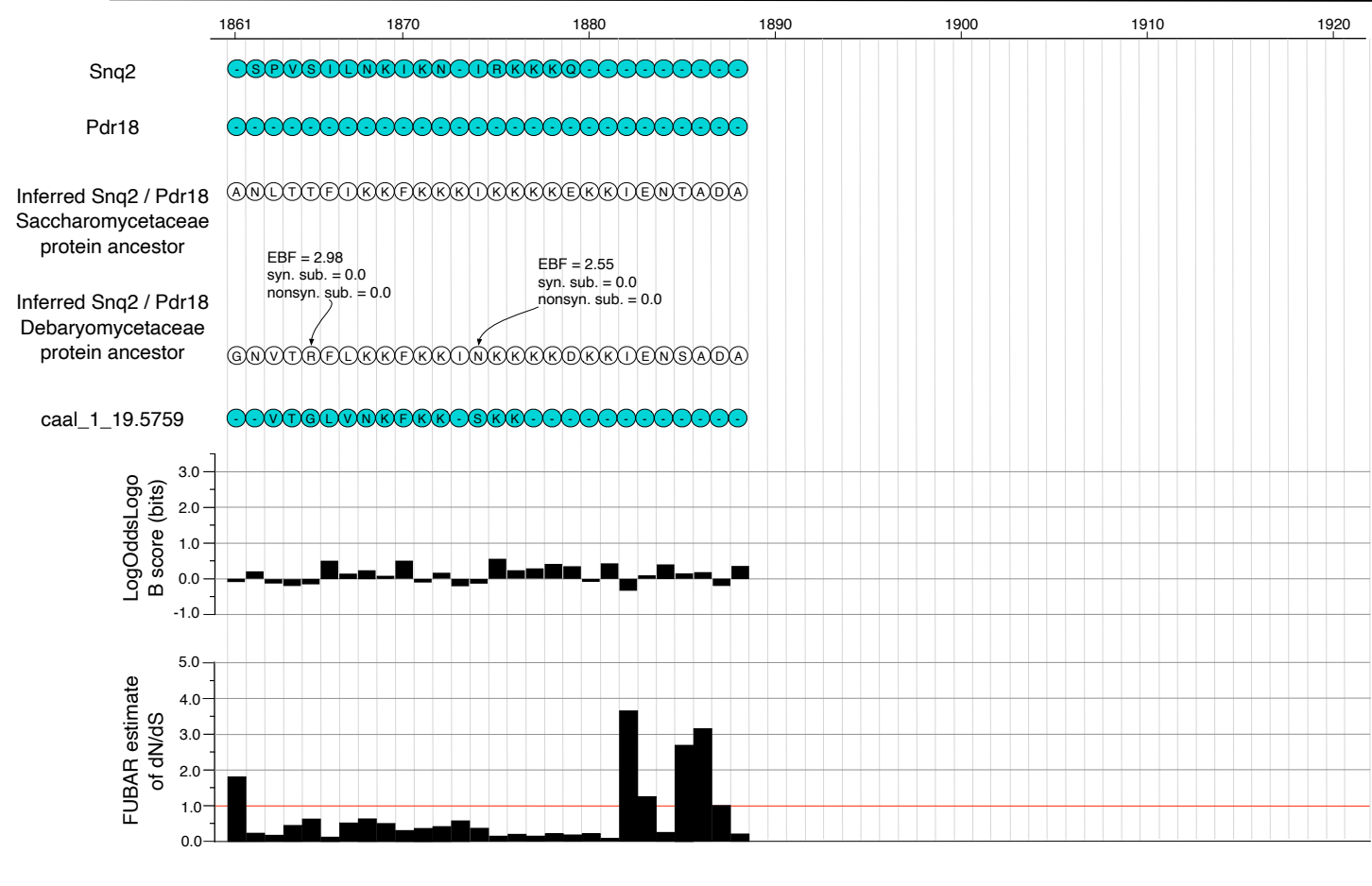

Supplement: foaf026_Supplemental_Files [file foaf026_supplemental_files.zip › Figure A32_Supplementary_Data.pdf]

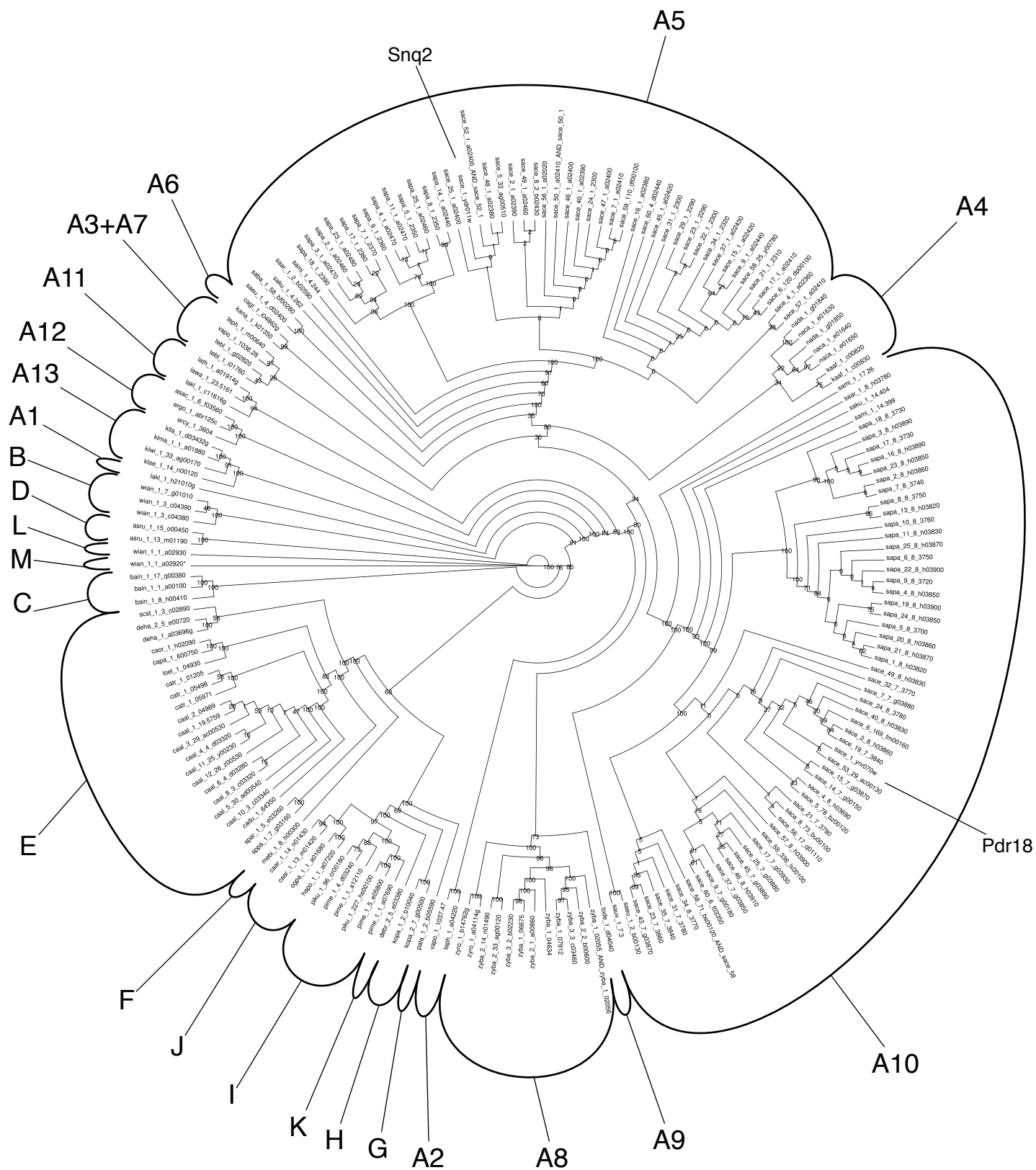

Figure Supplementary X

Supplement: foaf026_Supplemental_Files [file foaf026_supplemental_files.zip › Figure A5_Supplementary Data.pdf]
