# Supplementary material for: Molecular evolution of the members of the Snq2/Pdr18 subfamily of Pdr transporters in the Hemiascomycete yeasts
Source: FEMS Yeast Res. 2025 May 27;25:foaf026. doi: 10.1093/femsyr/foaf026 (PMC12202755; doi:10.1093/femsyr/foaf026)
Supplement: foaf026_Supplemental_Files [file Figure A7_Supplementary Data.pdf › Figure A7_Supplementary Data.pdf]

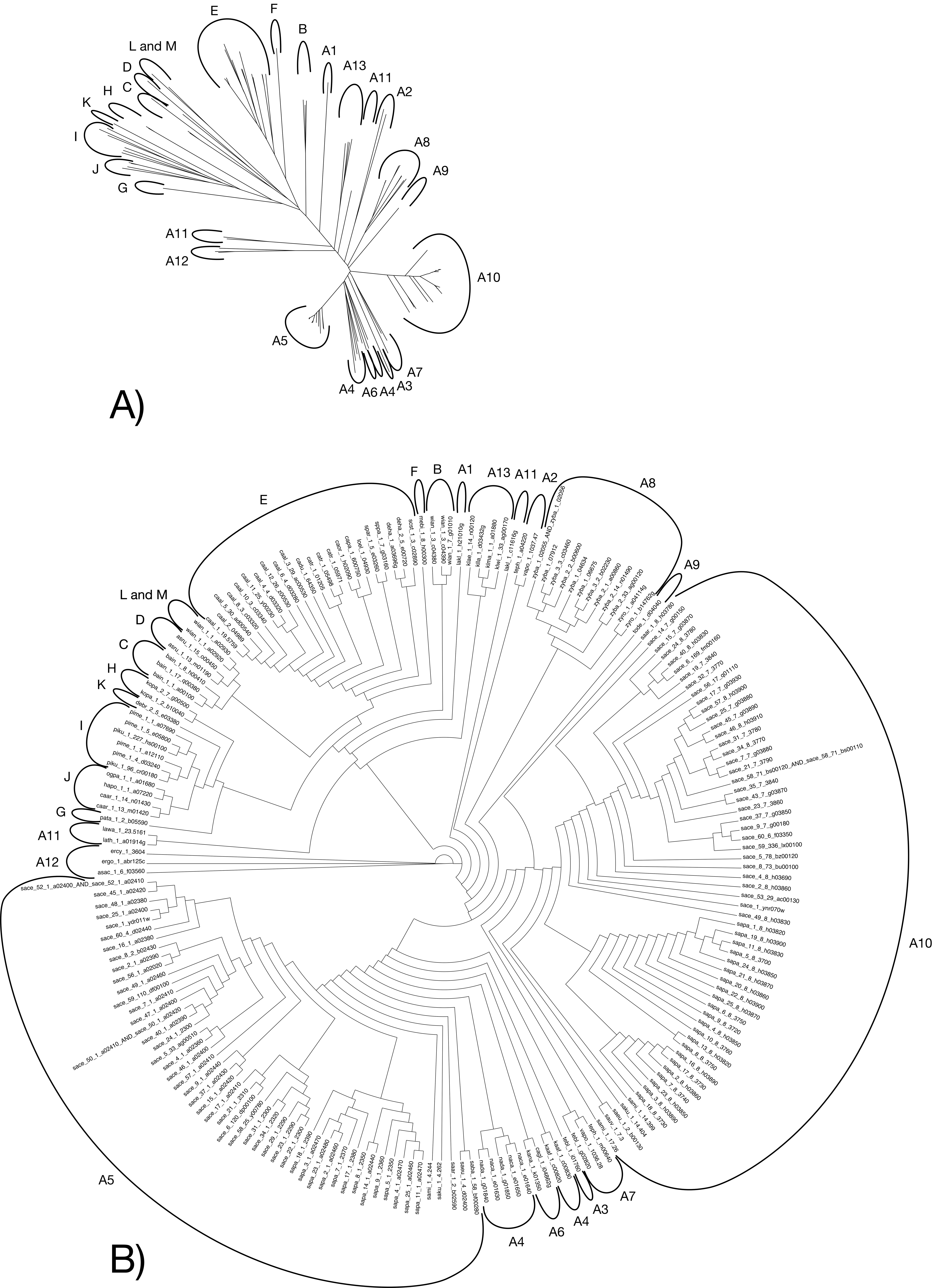

E
F
B
A1
L and M
A13
A11
A2
D
C
H
K
I
J
G
A8
A9
A11
A12
A10
A5
A4
A6
A4
A3
A7
A)
B)
